# Supplementary material for: Estimating progress towards meeting women’s contraceptive needs in 185 countries: A Bayesian hierarchical modelling study
Source: PLoS Med. 2020 Feb 18;17(2):e1003026. doi: 10.1371/journal.pmed.1003026 (PMC7028249; doi:10.1371/journal.pmed.1003026)
Supplement: S1 Appendix — (PDF) [file pmed.1003026.s001.pdf]

Estimating Progress Towards Meeting Women's Contraceptive Needs in 185 Countries: A Bayesian Hierarchical Modelling Study

S1 APPENDIX

Vladimíra Kantorová* Mark C. Wheldon Philipp Ueffing
Aisha N. Z. Dasgupta
United Nations Population Division, New York, NY†

23rd January 2020

*Corresponding author. Vladimíra Kantorová, Population Division, Department of Economic and Social Affairs, United Nations, kantorova@un.org

†The views and opinions expressed in this paper are those of the authors and do not necessarily represent those of the United Nations. This paper has not been formally edited and cleared by the United Nations.

CONTENTS

1 OVERVIEW **1**

2 DATA **2**
2.1 Definitions of regions, sub-regions and income groups . . . 2
2.2 Definitions of family planning indicators . . . 2
2.3 Data on contraceptive prevalence and unmet need for family planning . . . 4
2.3.1 Data set compilation . . . 4
2.3.2 Data availability . . . 6
2.4 Classification of countries based on sexual activity among UWRA . . . 8

3 METHODS **11**
3.1 Models for contraceptive prevalence . . . 11
3.2 Target of inference . . . 11
3.3 Time trends in contraceptive prevalence and unmet need . . . 12
3.3.1 Modeling components of the compositional vector . . . 12
3.3.2 Systematic trends in contraceptive use . . . 13
3.3.3 Systematic trends in unmet need . . . 15
3.4 Bayesian hierarchical model . . . 15
3.4.1 Hierarchical modelling and estimation by pooling . . . 15
3.4.2 Hierarchical model with sexual activity for unmarried women . . . 16
3.4.3 Parameter definitions and hierarchical structure . . . 17
3.5 Data Model . . . 20
3.5.1 Total prevalence greater than one percent . . . 20
3.5.2 Total prevalence less than one percent . . . 20
3.5.3 Unmet need . . . 21
3.5.4 Sampling and non-sampling errors . . . 21
3.5.5 Reference periods . . . 21
3.5.6 Data categorization based on source types . . . 21
3.5.7 Data categorization based on population characteristics . . . 21
3.5.8 Misclassification biases . . . 23
3.5.9 Bias and perturbation parameters: The examples of Austria and Belgium . . . 24
3.6 Full model specification and prior distributions . . . 25
3.6.1 List of main symbols . . . 25
3.6.2 List of indices . . . 26
3.6.3 Model specification . . . 27
3.6.4 Prior distributions . . . 30
3.7 Computation and Inference . . . 30
3.7.1 Countries and parameters without data . . . 31
3.7.2 Estimates and projections of numbers of users . . . 31
3.7.3 Estimates and projections for country aggregates . . . 32
3.7.4 Estimates and projections for all women . . . 32
3.7.5 Inference on changes . . . 32
3.7.6 Aggregate median adjustments . . . 32
3.8 Model validation . . . 33

4 RESULTS **34**
4.1 Error variance parameters 34
4.2 Misclassification biases and perturbation multipliers 34
4.3 Model validation 34

5 SUPPLEMENTARY TABLES **39**
5.1 Classification of countries by geographical area 40
5.2 Unmarried women 47
5.2.1 Modern contraceptive prevalence in 185 countries or areas 47
5.2.2 Demand for family planning satisfied by modern methods in SA group 1 countries 56
5.3 Married women 63
5.3.1 Modern contraceptive prevalence in 185 countries or areas 63
5.3.2 Demand for family planning satisfied by modern methods 72
5.4 All women 81
5.4.1 Modern contraceptive prevalence in 185 countries or areas 81
5.4.2 Demand for family planning satisfied by modern methods 90

6 GLOSSARY **99**

7 REFERENCES **100**

LIST OF TABLES

A Frequency table of countries by sexual activity classification and survey or method used to classify them. Survey type DHS are Demographic and Health Survey; survey type MICS are the Multiple Indicator Cluster Survey. See text for explanation of the method labelled "Main religion". . . . 10
B Comparison of the purely geographic (original) and sexual activity inclusive (new) classifications of countries for estimating contraceptive prevalence among unmarried and not in a union women of reproductive age (UWRA). . . . 17
C Number of observations by data model source 22
D Categorisation of non-base population samples, number of observations in each category and comparison of the expected prevalence levels in the non-base category compared to the base category of unmarried and not in a union women of reproductive age (UWRA). . . . 22
E Example: Input data for Austria and Belgium. . . . 24
F Summary of model validtion results. . . . 38
G Classification of countries by geographical area, income and sexual activity groups, and data sources 40
H Modern contraceptive prevalence, UWRA, 2000, 2019, and 2000–2019 change 47
I Demand for family planning met by modern methods, UWRA, 2000, 2019, and 2000–2019 change, SA group 1 countries 56
J Modern contraceptive prevalence, MWRA, 2000, 2019, and 2000–2019 change 63
K Demand for family planning met by modern methods, MWRA, 2000, 2019, and 2000–2019 change, SA group 1 countries 72
L Modern contraceptive prevalence, WRA, 2000, 2019, and 2000–2019 change 81
M Demand for family planning met by modern methods, WRA, 2000, 2019, and 2000–2019 change 90

LIST OF FIGURES

A Diagram illustrating project workflow. 1
B Scheme of unmet need for family planning indicator 4
C Overview of the observations on total contraceptive prevalence. 7
D Classification of countries around the world based on the information about the level of, acceptence of, or justification for sexual activity among unmarried women. 9
E Illustration of composition vector of contraceptive prevalence. 11
F Theoretical model of contraceptive prevalence (total, modern and traditional methods) over time. 14
G Available data on total contraceptive prevalence. Ga Bangladesh; Gb Ecuador. . . . 15
H Nested structure of the sexual activity inclusive hierarchy to model contraceptive prevalance among unmarried and not in a union women of reproductive age 18
I Posterior estimates of standard deviation parameters of observed contraceptive prevalence log-ratios by source. 35
J Posterior estimates of misclassification bias parameters for unmarried and not in a union women of reproductive age. 36
K Posterior estimates of the perturbation multiplier parameters for unmarried and not in a union women of reproductive age (UWRA). 37

1 OVERVIEW

The major components of the project, their interconnections, and sequencing are shown in Figure A.

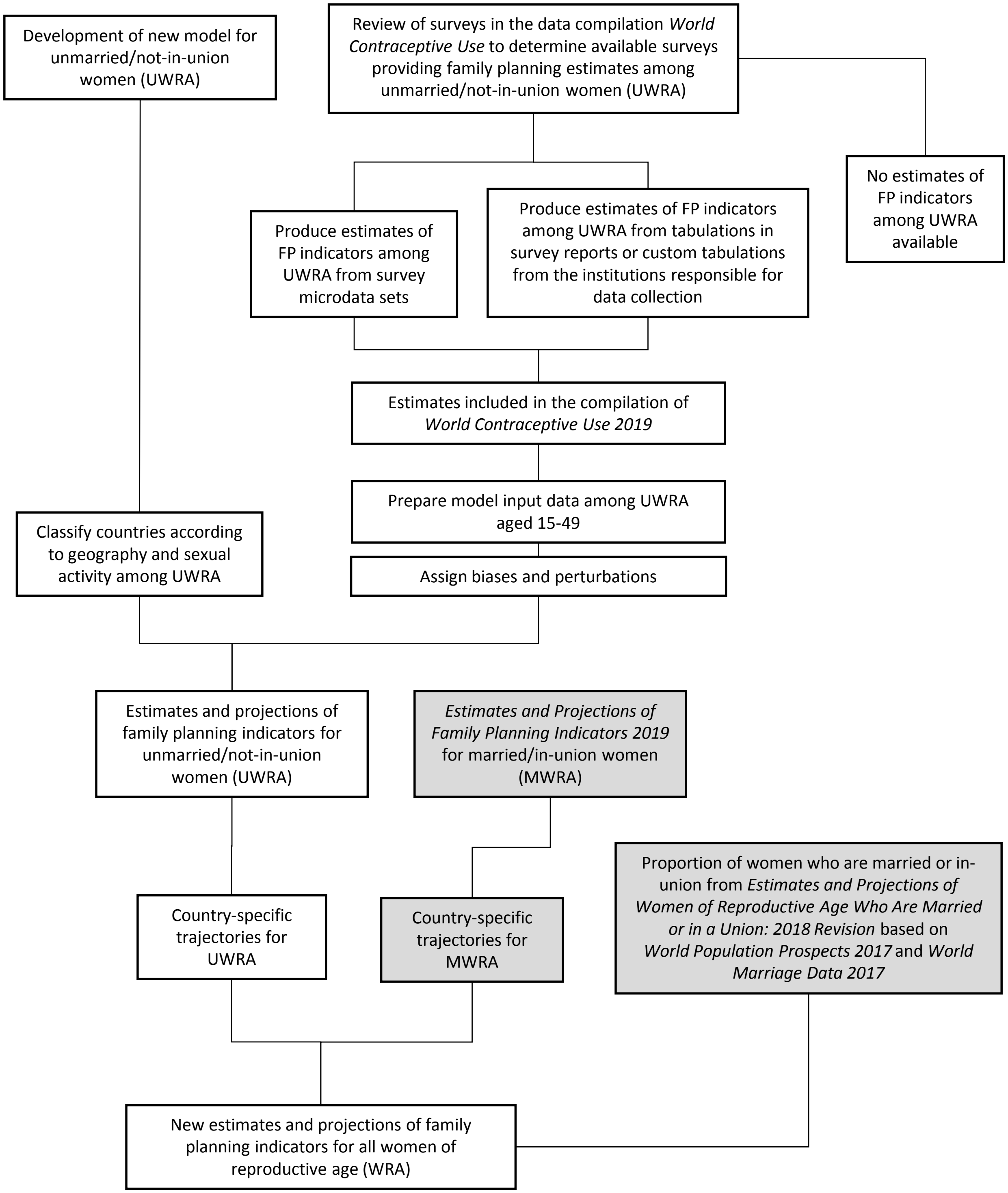

Figure A. Diagram illustrating project workflow. Components were worked on in order, from top to bottom. Boxes in light grey indicate inputs from external projects.

2 DATA

2.1 Definitions of regions, sub-regions and income groups

The definition of regions and sub-regions follows that implemented in the most recent publications of the United Nations (2019; see supplementary Table G). The classification of countries into income groups follows the World Bank (2019). Results for the total population of Melanesia, Micronesia and Polynesia combined are referred to as Mela-Micro-Polynesia.

2.2 Definitions of family planning indicators

While family planning indicators among married or in a union women of reproductive age (MWRA) are commonly used and readily interpreted, more caution and consideration needs to be given to the calculation and interpretation of the family planning indicators among unmarried and not in a union women of reproductive age (UWRA) and women of reproductive age irrespective of marital status (WRA).

The major differences in the approaches used for estimating family planning indicators among UWRA were related to the distinction of whether the universe of analyses was all UWRA or only UWRA who are deemed to be sexually active (as defined by sexual activity in past 28 days; Kantorová et al., 2017). In this paper, the population of UWRA refers to all unmarried and not in a union women of reproductive age, irrespective of sexual activity (United Nations, Department of Economic and Social Affairs, Population Division [UN Population Division], 2019).

Marital/Union status WRA pertains to all women of reproductive age. MWRA pertains to women who are married (defined in relation to the marriage laws or customs of a country) and to women in a union, which refers to women living with their partner in the same household (also referred to as cohabiting unions, consensual unions, unmarried unions, or "living together"). UWRA pertains to women who are not married and not in a union and is a complement to MWRA (UN Population Division, 2017, 2018a).

Contraceptive prevalence Contraceptive prevalence is the proportion of women who are currently using, or whose sexual partner is currently using, at least one method of contraception, regardless of the method being used (UN Population Division, 2019). It is reported as a percentage with reference to women of the respective marital status. For analytical purposes, contraceptive methods are classified as either modern or traditional. Modern methods of contraception include female and male sterilization, the intra-uterine device (IUD), the implant, injectables, oral contraceptive pills, male and female condoms, vaginal barrier methods (including the diaphragm, cervical cap and spermicidal foam, jelly, cream and sponge), the lactational amenorrhoea method (LAM), emergency contraception and other modern methods not reported separately (e.g., the contraceptive patch or vaginal ring). Traditional methods of contraception include rhythm (e.g., fertility awareness-based methods, periodic abstinence), withdrawal, and other traditional methods not reported separately.

Unmet need for family planning The unmet need for family planning illustrates the gap between women's reproductive intentions and their contraceptive behaviour. It is defined as the proportion of women who want to stop or delay childbearing but are not using any method of contraception. In this paper, unmet need for family planning is reported as a percentage with reference to women of the respective marital status (UN Population Division, 2019).

The standard definition of unmet need for family planning includes in the numerator women who are fecund and sexually active and report not wanting any (more) children, or who report wanting to delay the birth of their next child for at least two years, or are undecided about the timing of the next birth but who are not using any method of contraception. The numerator also includes i) pregnant women whose pregnancies were unwanted or mistimed at the time of conception, and ii) postpartum amenorrheic women who are not using family planning and whose last birth was unwanted or mistimed. Infecund women are excluded from the numerator. Women are assumed to be infecund if:

1. They were first married more than five years ago, have not had a birth in the past five years, are not currently pregnant, and have never used any kind of contraceptive method; or
2. They report being infecund or menopausal, having had a hysterectomy, never having menstruated, or being postpartum amenorrheic for five years or longer; or
3. They are not pregnant or in postpartum amenorrhea and they report that their last menstrual period occurred six months or more prior to the survey.

Postpartum amenorrheic women are women who have not had a menstrual period since the birth of their last child where the birth occurred in the period 0–23 months prior to the survey. If their period has not returned 24 months or more after the previous birth, women are considered fecund, unless they fall into one of the infecund categories above.

MWRA are assumed to be sexually active. For UWRA, it is necessary to determine the timing of their most recent sexual activity. UWRA who are not pregnant or postpartum amenorrheic are considered currently at risk of pregnancy (and thus could potentially be included in the numerator as having unmet need) if they have had intercourse in the four weeks prior to the survey interview. The unmet need for UWRA who are pregnant or postpartum amenorrheic is determined in the same way as for MWRA and regardless of their most recent sexual activity. Pregnant UWRA whose pregnancies were unwanted or mistimed at the time of conception, and postpartum amenorrheic UWRA who are not using family planning and whose last birth was unwanted or mistimed, are assumed to have an unmet need.

Figure B indicates the procedure set out by the Demographic and Health Survey (DHS) program for computing the number of women of reproductive age who have an unmet need for family planning (referred to as the 2012 DHS definition). These data are available in DHSs from Round 2, and in Multiple Indicator Cluster Surveys (MICSs) from Round 4 for MWRA and from Round 5 for UWRA. Further information on the operational definition of the unmet need for family planning, as well as survey questions and statistical programs needed to derive the indicator, can be found in Bradley et al. (2012) and DHS Program (2019).

Demand satisfied for family planning The demand for family planning that is satisfied by using modern methods of contraception (as defined in UN Population Division, 2019) describes the number of women who are currently using, or whose sexual partner is currently using, at least one modern contraceptive method as a proportion of the number of women of reproductive age who have a demand for family planning.

The indicator is calculated using measures of contraceptive prevalence and the unmet need for family planning, the numerator being the prevalence of contraceptive use for any modern method, and the denominator being the total demand for family planning, which equals the sum of contraceptive prevalence for any method and the unmet need for family planning.

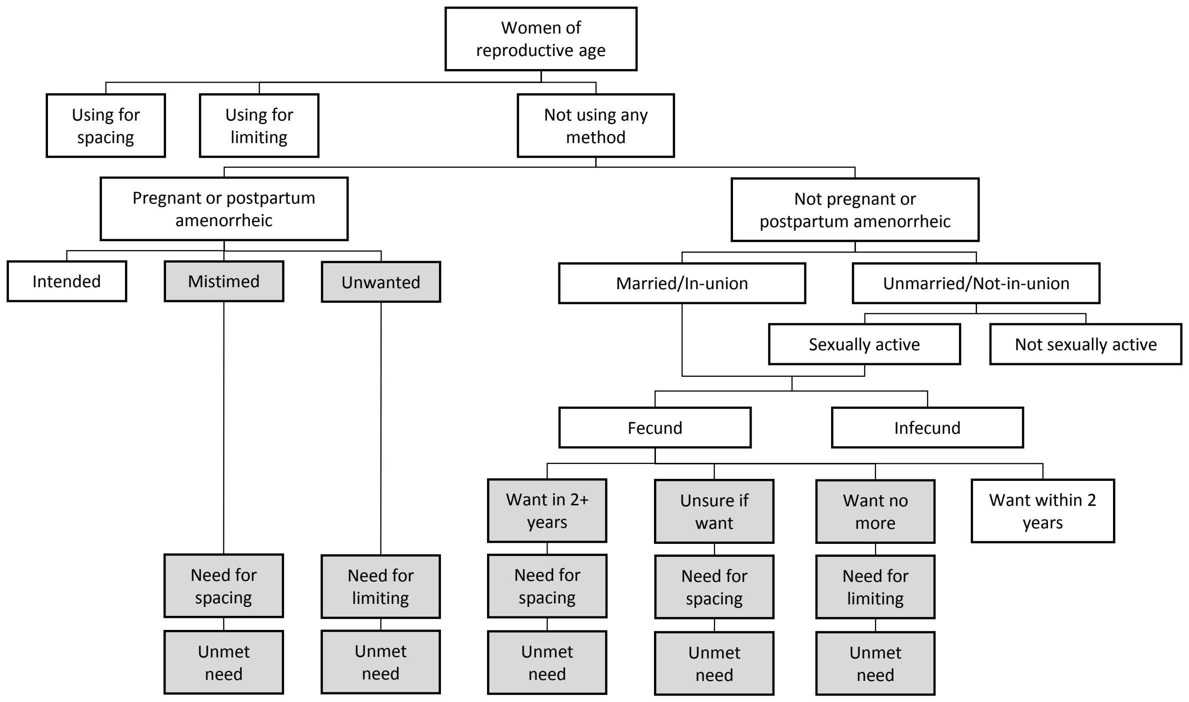

Figure B. DHS 2012 revised definition of the unmet need for family planning indicator Based on (Bradley et al., 2012)

2.3 Data on contraceptive prevalence and unmet need for family planning

2.3.1 Data set compilation

To obtain the data compilation needed to estimate and project family planning indicators by marital status, we assessed and compiled data from nationally representative household surveys. The starting point was the data sources listed in UN Population Division (2019).

Demographic and Health Surveys We used data from 306 DHSs. Family planning indicators were derived from microdata for 274 of these surveys. To facilitate the calculation of survey estimates, original DHS family planning variables were harmonized across surveys. The harmonization process followed the approach used in the Integrated Demographic and Health Survey (iDHS), part of the Integrated Public Use Microdata Series (IPUMS; Boyle et al., 2017). The iDHS project harmonizes DHS data sets over time and across countries. Variables are consistently coded and documentation is organized in a cross-survey, variable specific way. For each variable, a 'translation-table' is created that shows the original and the harmonized variable name and variable codes across all available DHSs in a single spreadsheet. These translation tables were available for 116 DHSs from the iDHS project and were extended by the UN Population Division to cover an additional 158 surveys. Minor differences exist between the iDHS project and the work of the UN Population Division in the classification of contraceptive methods into modern and traditional methods related to the distinction of breastfeeding and LAM.

In the case of 25 surveys from Bangladesh, Egypt, Jordan, Nepal, Maldives, Thailand, Turkey

and Sri Lanka, where the DHS women questionnaire was limited to ever-married women, survey estimates were adjusted to produce family planning indicators for all women and for UWRA. The assumption was made that contraceptive use among never married women of reproductive age was zero. The available survey estimates for formerly married women were then weighted by the proportion of formerly married women among UWRA to produce estimates of contraceptive prevalence among UWRA. Perturbation multipliers were used in the model to account for the expected downward bias (see Section 3.5.7).

For 32 DHSs where microdata sets were not available the data were obtained from calculations based on the data presented in the survey reports.

Multiple Indicator Cluster Surveys The MICS final reports present family planning indicators only for MWRA. We used the MICS microdata sets to estimate family planning indicators for UWRA. Microdata sets were available for 135 of the 160 MICSs included in the data set. For the remaining 25 surveys, data were obtained from calculations based on the data presented in the survey reports.

There is a high level of diversity among MICSs in terms of availability of questions, variable names, categories within the variables, and labels. Whereas DHS produces one cleaned variable for current use of contraception, MICS produces a multitude of variables, one for each method. Additionally, codebooks were not widely available for MICSs. These inconsistencies have meant that the impressive availability of MICS microdata sets have previously been underused because the variables are not in a format that can be easily used.

In response to this challenge, we developed a methodology to standardise the variables, following the approach of iDHS. For each variable requiring standardization, cross-survey variable-specific 'translation-tables' were organized in spreadsheet format. Variable names, categories, and labels for each survey were listed out and 'translated' into standardised variable names, categories and labels. The variables of interest included age, marital status, current use of contraception, the 22 variables which captured current method of contraception, and the 31 variables needed to calculate unmet need for family planning.

Performance, Monitoring and Accountability 2020 surveys Family planning estimates were derived from publicly available microdata sets from the Performance Monitoring and Accountability 2020 surveys (PMAs).

Gender and Generation Survey Harmonised microdata from the first two rounds of the Generations and Gender Survey (GGS) are publicly available and were used to produce family planning estimates. A review of the survey questions underlying harmonised family planning variables revealed differences that did not allow the inclusion of all surveys into the data set.

Other multi-country survey programmes and national surveys Additional data were from the published reports and tabulations of other multi-country survey programmes that routinely collect the necessary data, including Contraceptive Prevalence Surveys (CPSs), Fertility and Family Surveyss (FFSs), Reproductive Health Surveys (RHSs), World Fertility Surveys (WFSs) and national surveys. If observations of contraceptive prevalence among WRA were tabulated in the survey reports, generally there is no discrepancy between them and the estimates in the data compilation (UN Population Division, 2019), except for occasional re-categorization of methods according to modern/traditional.

In some cases, when contraceptive prevalence among all UWRA was not tabulated, we calculated the estimates based on the published tabulations of the contraceptive prevalence among WRA and MWRA, weighted by the number of women by marital status and age. We also summarised metadata, such as the survey population eligible for the woman's questionnaire, availability of resources (including final report and questionnaire), whether formerly and never married women are asked about current use, and how the results were tabulated. Where relevant, biases concerning age, marital status, contraceptive method, weighting and questionnaire were noted. As part of a separate investigation, we also recorded whether questions on knowledge/awareness of family planning methods preceded the question on current use.

2.3.2 Data availability

For MWRA, the data compilation (UN Population Division, 2019) contains family planning indicators for 1243 observations of contraceptive prevalence for 195 countries or areas for the period from 1950 to 2019 and 540 observations of unmet need for family planning for 143 countries. For UWRA, the data compilation includes 551 observations of contraceptive prevalence for 136 countries and areas for the period from 1976 to 2019. The earliest observation is from the New Zealand 1976 National Survey of Contraceptive Practice, and the most recent is from the Kazakhstan 2018 Gender and Generations Survey. Out of the 136 countries or areas with any data on contraceptive prevalence among UWRA, 72 countries or areas had data on unmet need for family planning (250 observations in total). Reasons for fewer data points for UWRA include:

1. UWRA were not eligible for the women's questionnaire;
2. UWRA were not asked about current contraceptive use;
3. The results for contraceptive prevalence among UWRA were not tabulated and presented in reports; or
4. Only a subset of UWRA (usually formerly married women) were included.

Data availability for all countries Figure C gives an overview on data availability by development status, region, and period, for observations on total prevalence among UWRA compared to MWRA. More than half of the 195 countries and areas with data on contraceptive prevalence for MWRA had five or more observations. Data series for UWRA are shorter. Only 35 percent of the 136 countries and areas with data on contraceptive prevalence for UWRA had five or more observations. Only 26 percent of countries had any data before 1990 and 51 percent of countries had data available for the period 1990 to 1999. Therefore, we interpret the model-based estimates only for the period after 2000.

In Africa, 45 developing countries and areas had any data on contraceptive prevalence among UWRA compared to 54 among MWRA. Developing countries with data in Africa also had the most observations on contraceptive prevalence over the full time period (56 percent had five or more observations for UWRA and another 8.9 percent had four observations). Developing countries in Latin America and the Caribbean also had relatively good data coverage overall; of 39 countries in the MWRA data compilation, 26 countries had data for UWRA (of which 46 percent had five or more observations). Only about half of the developing countries in Asia had any data on contraceptive use among UWRA (25 countries compared to 45 countries in the MWRA women data set). Of these, 28 percent had only one data point. Developing countries in Oceania tended to have the most limited data available overall and across the different time periods in both marital groups.

	0	1	2	3	4	5+
All (136)	0	22	20	12	11	35
Before 1990	74	21	4	1	0	0
1990−1999	50	26	18	4	1	1
2000−2009	24	35	29	8	4	1
2010 − 2019	21	43	24	6	0	6
Developed (31)	0	32	32	10	16	10
Before 1990	77	16	6	0	0	0
1990−1999	68	23	6	3	0	0
2000−2009	35	39	16	6	0	3
2010 − 2019	32	52	16	0	0	0
Africa (45)	0	7	9	20	9	56
Before 1990	76	22	2	0	0	0
1990−1999	29	40	24	4	2	0
2000−2009	11	36	33	16	4	0
2010 − 2019	9	29	36	11	0	16
Asia (25)	0	28	20	4	16	32
Before 1990	92	8	0	0	0	0
1990−1999	64	16	12	4	0	4
2000−2009	20	44	16	8	8	4
2010 − 2019	24	36	28	12	0	0
LAC (26)	0	23	12	12	8	46
Before 1990	42	46	8	4	0	0
1990−1999	42	15	35	8	0	0
2000−2009	35	8	54	0	4	0
2010 − 2019	15	62	19	0	0	4
Oceania (9)	0	44	56	0	0	0
Before 1990	100	0	0	0	0	0
1990−1999	78	22	0	0	0	0
2000−2009	22	67	11	0	0	0
2010 − 2019	56	44	0	0	0	0

Number of observations

	0	1	2	3	4	5+
All (195)	0	9	10	9	13	58
Before 1990	37	32	10	8	6	7
1990−1999	26	34	26	8	2	4
2000−2009	18	24	31	15	6	6
2010 − 2019	28	35	22	8	2	5
Developed (43)	0	7	21	7	16	49
Before 1990	30	42	9	9	0	9
1990−1999	16	53	21	2	2	5
2000−2009	30	23	23	14	7	2
2010 − 2019	44	37	16	2	0	0
Africa (54)	0	0	6	11	15	69
Before 1990	44	39	9	6	2	0
1990−1999	19	41	24	11	4	2
2000−2009	2	30	43	19	6	2
2010 − 2019	7	30	33	13	4	13
Asia (45)	0	2	2	4	13	78
Before 1990	42	18	9	9	7	16
1990−1999	13	29	36	13	0	9
2000−2009	11	9	31	22	7	20
2010 − 2019	18	38	27	11	2	4
LAC (39)	0	18	10	10	10	51
Before 1990	15	31	15	13	21	5
1990−1999	46	15	31	8	0	0
2000−2009	33	18	36	5	8	0
2010 − 2019	36	41	15	5	0	3
Oceania (14)	0	50	21	21	7	0
Before 1990	71	21	7	0	0	0
1990−1999	71	21	7	0	0	0
2000−2009	29	64	0	7	0	0
2010 − 2019	71	29	0	0	0	0

Number of observations

Figure C. Overview of the percentage of countries with 0, 1, 2, 3, 4, or 5+ observations on total contraceptive prevalence among UWRA (left) and MWRA (right), summarized for all countries, for all developed countries combined, and by region for the developing countries. The first row (green) for each group of countries refers to the entire observation period; each cell contains the percentage of countries with 0, 1, 2, 3, 4, or 5+ observations. Similarly, subsequent rows (blue) show the percentage of countries by number of observations in the period before 1990, from 1990 to 1999, from 2000 to 2009 and from 2010 to 2019. The coloured shading visualizes the percentage of countries in each cell. The number with the subgroup refers to the total number of countries within that subgroup. Country totals for geographic areas exclude developed countries. “LAC” refers to Latin America and the Caribbean.

Of the 551 observations on contraceptive prevalence for UWRA across all countries and years, 265 observations were from DHSs. Twenty-seven of these were classified separately for modeling purposes because the values for UWRAs were derived from the number ever married. Of the remaining, 94 were from MICSs, 14 from GGSs, 8 from CPS, 36 from RHSs, and 34 from PMAs. Other international survey programs, and national surveys that were not conducted as part of an internationally coordinated program, accounted for 100 observations.

The concentration towards one international survey programme was more pronounced for the unmet need for family planning indicator where 184 observations are from DHSs. A further 21 observations came from MICSs, 34 from PMAs, 7 from RHSs, and 4 from national surveys.

The largest gap in the data compilation was China, which represented 14 percent of the global population of UWRA. In China, only ever married women were asked about contraceptive use, even in light of growing evidence from studies (though not nationally-representative) that sexual activity and contraceptive use among unmarried women is increasingly common (Li et al., 2013; Li and Newcomer, 1996).

2.4 Classification of countries based on data and information on sexual activity among UWRA

Models of reproductive behaviour among MWRA commonly assume that all MWRA are sexually active. This assumption could not be applied to UWRA. There were large differences in the prevalence of sex among UWRA (Ueffing et al., 2017) that needed to be accounted for in the hierarchical structure of our model of reproductive behaviour (further explained in Section 3). Two groups of countries were defined: i) countries with very low levels of sexual activity (Group 0), and ii) all other countries (Group 1; Table G).

Countries were classified as having very low prevalence of sex among UWRA when the proportion of UWRA reporting recent sexual activity (sexual intercourse in past four weeks) was less than 2 percent. These estimates were sourced from 81 DHSs and MICSs surveys.

For countries where no data on sexual activity were available from DHS or MICS, information on the acceptance of sex between unmarried adults was used as a proxy for sexual activity among UWRA. The Pew Research Center's 2013 Global Attitudes survey asked 40,117 respondents in 40 countries if they "personally believe that sex between unmarried adults is morally acceptable, morally unacceptable, or is it not a moral issue?" (Pew Research Center, 2014). The World Values Survey (WVS) Wave 6, covering 2010 and 2014, asked 86,274 respondents in 51 countries how much they would agree with the statement that sex before marriage is justifiable on a scale from 1 "Never just fiable" to 10 "Always justifiable" (Inglehart et al., 2013). Both surveys found that countries with predominantly Muslim populations in Asia and Northern Africa were least accepting of sex between unmarried adults. More than 80 percent of respondents in the Pew surveys in Egypt, Jordan, Lebanon, Malaysia, Pakistan, State of Palestine, Tunisia, and Turkey answered that sex between unmarried adults is morally unacceptable. In the WVS, respondents in Jordan, Qatar, Pakistan, Libya, Azerbaijan, Turkey, Morocco and Uzbekistan least agreed with the statement that sex before marriage is justifiable (average score of less than 2.0). The justifiability scores from WVS correlated highly with the proportion of the population regarding unmarried sex as acceptable from Pew (R-Square = 0.94, n = 22). Ten countries were assigned to the low sexual activity group (Group 0) based on these two surveys, and 33 to Group 1.

When neither data on sexual activity nor on the acceptance/justification of sex among unmarried adults were available, information on religious affiliation published in the 2012 Study on the Global Religious Landscape (Pew Research Center, 2012) was used. Eighteen countries in Asia and Northern Africa with 70 percent or more of the population Muslim were assigned to the low sexual

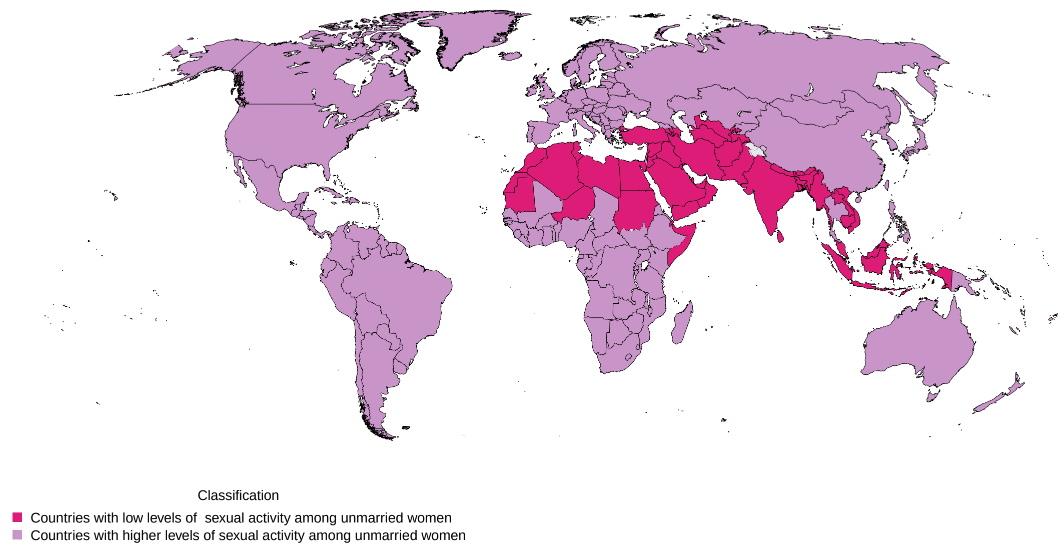

Figure D. Classification of countries around the world based on the information about the level of, acceptence of, or justification for sexual activity among unmarried women.
The boundaries and names shown and the designations used on this map do not imply official endorsement or acceptance by the United Nations. The dotted lines represent approximates. The Line of Control in Jammu and Kashmir agreed upon by India and Pakistan. The final status of Jammu and Kashmir has not yet been agreed upon by the parties. The final boundary between the Republic of Sudan and the Republic of South Sudan has not yet been determined.

activity group. The 70 percent break was derived from the set of countries that were classified as low sexual activity countries based on one of the previous direct or indirect measures of sexual activity.

An additional two countries (Myanmar and Sri Lanka) that lacked data on the sexual activity among UWRA were classified as low sexual activity countries on the basis of cultural and geographical proximity. All other countries in South and South-Eastern Asia were classified as low sexual activity countries, with the exception of Thailand, Philippines, and Singapore.

In total, 45 countries (23 percent) were classified as having low sexual activity among unmarried women. All of these countries were in either Africa or Asia, predominantly in the following subregions: Northern Africa, Western Asia, South-Central Asia and South-Eastern Asia (Figure D).

Table A. Frequency table of countries by sexual activity classification and survey or method used to classify them. Survey type DHS are Demographic and Health Survey; survey type MICS are the Multiple Indicator Cluster Survey. See text for explanation of the method labelled "Main religion".

	Sexual Activity Group		
Survey/Method	Group 0	Group 1	Total
DHS and MICS	17	64	81
Pew 2013 Global Attitudes Survey	8	21	29
World Values Survey Wave 6	2	12	14
Main religion	18	53	71
Total	45	150	195

3 METHODS

3.1 Models for contraceptive prevalence among married and unmarried women

Alkema et al. (2013) developed a model for national-level contraceptive prevalence and unmet need among MWRA. This was extended to UWRA by Wheldon et al. (2019). Further methodological improvements to Alkema et al.'s (2013) MWRA model were made by Cahill et al. (2017) and we used this model to generate our estimates for MWRA. For UWRA, we applied Cahill et al.'s (2017) improvements to Wheldon et al.'s (2019) model. To avoid too much repetition of explanations that remain unchanged, we give brief descriptions here and refer the reader to the aforementioned studies for further details. Unless otherwise specified, discussion of specific parameters or data characteristics throughout this Methods section refers to the new model for UWRA only.

3.2 Target of inference

The goal of the study was the categorization of UWRA (the base population) as users of traditional contraceptive methods, users of modern contraceptive methods, having unmet need for contraceptive methods, and not having need for any method. Thus the outcome of interest was the same compositional vector modelled by Cahill et al. (2017) and Alkema et al. (2013): $\boldsymbol{p}_{c,t} = (p_{c,t,1}, p_{c,t,2}, p_{c,t,3}, p_{c,t,4})$, where $p_{c,t,m}$ denotes the proportion of women in country c, in year t, who use traditional methods ($m = 1$), modern methods ($m = 2$), have unmet need for contraceptive methods ($m = 3$), or do not use and do not need contraceptive methods ($m = 4$), see Figure E.

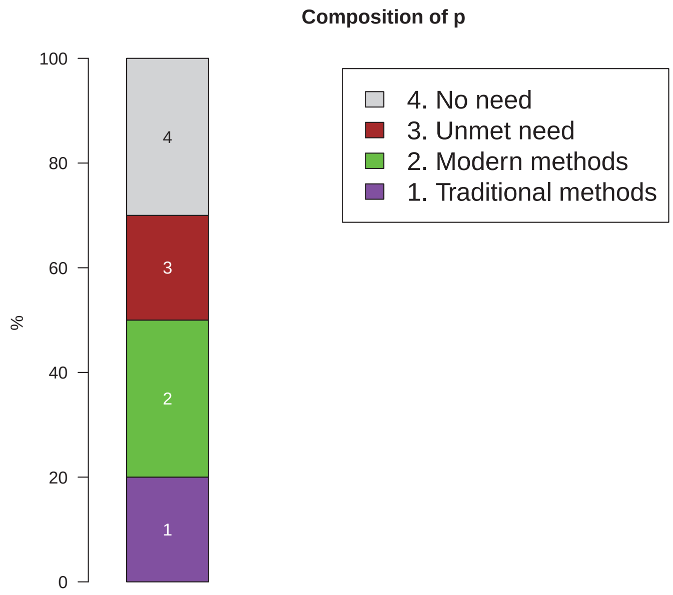

Figure E. Illustration of composition $\boldsymbol{p}_{c,t} = p_{c,t}$. Categorization of women who use traditional contraceptive methods, women who use modern contraceptive methods, women who have unmet need for contraceptive methods, and women who do not need any method (who are not avoiding a pregnancy).

The vector $\boldsymbol{p}_{c,t}$ was not observed. Instead, we observed $\boldsymbol{y}_i = y_{i,1:4}$, where $y_{i,m}$ denotes the proportion of women in category m (traditional, modern, unmet need, no need, respectively) for observation $i = 1, \ldots, I$ for country $c[i]$ and year $t[i]$. The data model for an observation $\boldsymbol{y}_i$ given $\boldsymbol{p}_{c[i],m[i]}$ is described in Section 3.5.

3.3 Time trends in contraceptive prevalence and unmet need

3.3.1 Modeling components of the compositional vector

To ensure that the components of $\boldsymbol{p}_{c,t}$ sum to unity, as required, we modelled the following quantities:

$$P_{c,t} = p_{c,t,1} + p_{c,t,2} \tag{3.1}$$
$$R_{c,t} = p_{c,t,2}/(p_{c,t,1} + p_{c,t,2}) \tag{3.2}$$
$$Z_{c,t} = p_{c,t,3}/(p_{c,t,3} + p_{c,t,4}) \tag{3.3}$$

where $0 \leq P_{c,t}, R_{c,t}, Z_{c,t} \leq 1$. $P_{c,t}$ is the total contraceptive prevalence, $R_{c,t}$ is the ratio of modern to total prevalence, and $Z_{c,t}$ is the ratio of unmet need to no contraceptive use, all in country c, year t. An expanded explanation is given in Alkema et al. (2013, Supplementary Appendix Section 2.1). Briefly, these three equations completely specify all four elements of the compositional vector since

$$p_{c,t,1} = (1 - R_{c,t}) \cdot P_{c,t} \tag{3.4}$$
$$p_{c,t,2} = R_{c,t} \cdot P_{c,t} \tag{3.5}$$
$$p_{c,t,3} = (1 - P_{c,t}) \cdot Z_{c,t} \tag{3.6}$$
$$p_{c,t,4} = (1 - P_{c,t}) \cdot (1 - Z_{c,t}) \tag{3.7}$$

Moreover, by substituting (3.4)–(3.7) for $p_{c,t,m}$ we have $\sum_{m=1}^{4} p_{c,t,m} = 1$.

We applied the logit transform to $\{P_{c,t}, R_{c,t}, Z_{c,t}\}$ to restrict the outcomes to be between 0 and 1. $R_{c,t}$ and $Z_{c,t}$ were modelled by systematic (latent) trends with autocorrelated distortions added:

$$\text{logit}(R_{c,t}) = \text{logit}(R^*_{c,t}) + \eta_{c,t} \tag{3.8}$$
$$\text{logit}(Z_{c,t}) = \text{logit}(Z^*_{c,t}) + \theta_{c,t} \tag{3.9}$$

In the above, $\{R^*_{c,t}, Z^*_{c,t}\}$ are country-specific systematic trends and $\{\eta_{c,t}, \theta_{c,t}\}$ are their respective autocorrelated distortions. Distortions were added to first differences of $\text{logit}(P_{c,t})$ (Cahill et al., 2017, Supplementary Appendix Section 2.2, explained further below):

$$\text{logit}(P_{c,t}) - \text{logit}(P_{c,t-1}) = \delta_{c,t} + \varepsilon_{c,t}, \; t \neq t^* = 1990 \tag{3.10}$$

All distortions were modelled by autoregressive processes of order 1:

$$\varepsilon_{c,t} \sim N(\rho_\varepsilon \cdot \varepsilon_{c,t-1}, \tau^2_\varepsilon) \tag{3.11}$$
$$\eta_{c,t} \sim N(\rho_\eta \cdot \eta_{c,t-1}, \tau^2_\eta) \tag{3.12}$$
$$\theta_{c,t} \sim N(\rho_\theta \cdot \theta_{c,t-1}, \tau^2_\theta) \tag{3.13}$$

with autoregressive parameter $0 < \rho_{\cdot} < 1$ and variance $\tau^2_{\cdot}$. The distributions for the distortions in

the first observation year $t_{c,1}$ in country c were:

$$\varepsilon_{c,t_{c,1}} \sim N\left(0, \frac{\sigma_\varepsilon^2}{1-\rho_\varepsilon^2}\right) \tag{3.14}$$

$$\eta_{c,t_{c,1}} \sim N\left(0, \frac{\sigma_\eta^2}{1-\rho_\eta^2}\right) \tag{3.15}$$

$$\theta_{c,t_{c,1}} \sim N\left(0, \frac{\sigma_\theta^2}{1-\rho_\theta^2}\right) \tag{3.16}$$

3.3.2 Systematic trends in contraceptive use

Alkema et al. (2013) modelled the systematic trends in total contraceptive prevalence, $P^*_{c,t}$, and the ratio of modern to total use, $R^*_{c,t}$, as logistic curves parameterized by, respectively, asymptotes, $\{\tilde{P}_c,\ \tilde{R}_c\}$, pace parameters, $\{\omega_c,\ \psi_c\}$, and locations $\{\Omega_c,\ \Psi_c\}$:

$$P^*_{c,t} = \frac{\tilde{P}_c}{1+\exp(-\omega_c(t-\Omega_c))} \tag{3.17}$$

$$R^*_{c,t} = \frac{\tilde{R}_c}{1+\exp(-\psi_c(t-\Psi_c))} \tag{3.18}$$

Cahill et al. (2017) found that defining $P_{c,t}$ in terms of successive differences and the level at a specific year, P_{c,t^*}, yielded a better fit:

$$\text{logit}(P_{c,t}) = \text{logit}(P_{c,t-1}) + \delta_{c,t} + \varepsilon_{c,t},\ t \neq t^* = 1990 \tag{3.19}$$

where $\delta_{c,t}$ is a function of the asymptote ($\tilde{P}_c$), pace (ω_c), and prevalence in the previous year ($P_{c,t-1}$). The systematic trend in total prevalence is still a logistic curve, but in this parameterization the location parameter Ω_c is replaced with prevalence at a specific year, P_{c,t^*}; we call this the “set-level” parameter. t^* was fixed at 1990 for all countries because this was close to the centre of the range of available data.

Diffusion Process Among UWRA It is reasonable to expect that contraceptive prevalence among UWRA is driven by a similar diffusion of ideas as in MWRA, with an important exception. Among UWRA, we hypothesize a prerequisite stage in which sexual activity increases before contraceptive prevalence can become more prevalent. Following sufficient increase in sexual activity, contraceptive prevalence among UWRA follows a similar pattern as among MWRA, with different parameter values.

Examples of systematic trends The systematic trends in total prevalence, its break-down into modern and traditional method use, and example trajectories after adding the autocorrelated distortion terms, are illustrated in Figure F (Panel (a)). Note that the trend in traditional method use (the inverted U-shape in the illustration) is not modelled explicitly, it follows from the logistic curves for total prevalence and for the ratio of modern to total prevalence. The actual trend in a country of interest depends on the timing, pace and asymptotes for total prevalence, and the uptake of modern methods as a ratio of any method. The asymptotes of total contraceptive use and the ratio of modern to total prevalence in a country may vary for a number of reasons, in part due to restrictions on the availability of modern methods or the extent to which induced abortion is practised. We do not take into account these other factors.

Examples of different segments of "contraceptive prevalence transitions" are given in Panels (b) and (c) for Colombia and Uganda, respectively.

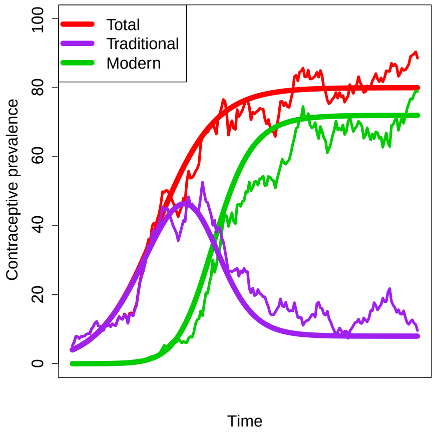

(a) Model representation.

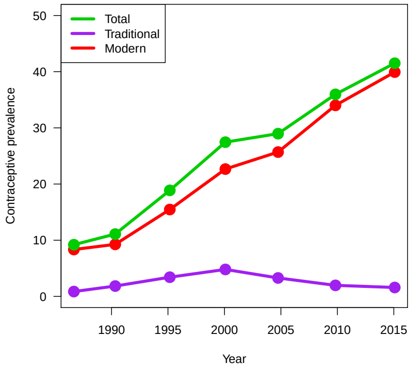

(b) Illustration: Colombia.

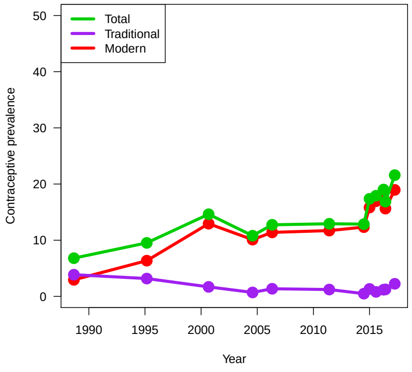

(c) Illustration: Uganda.

Figure F. Theoretical model of contraceptive prevalence (total, modern and traditional methods) over time. (a) Model representation: stylized examples of systematic trends (smooth lines, modelled by parametric functions on contraceptive prevalence and the ratio of modern use to any method use), and simulated trajectories (non-smooth lines, modelled by the systematic trends with autocorrelated distortions) of total, modern and traditional prevalence. (b) and (c): Trajectories of contraceptive prevalence for Colombia and Uganda among unmarried and not in a union women of reproductive age.

Systematic trends among UWRA We used the same functional forms for the systematic trends in prevalence for UWRA as Cahill et al. (2017) used for MWRA. However, we modelled the two marital groups separately because, even within the same country, the timing and pace of the uptake can be very different among the two marital groups. As illustrated in Figure G, prevalence has already begun to increase among UWRA in some countries (e.g., Ecuador), while in others it has not (e.g,. Bangladesh). Even in Ecuador, the timing of the increase is much later for UWRA.

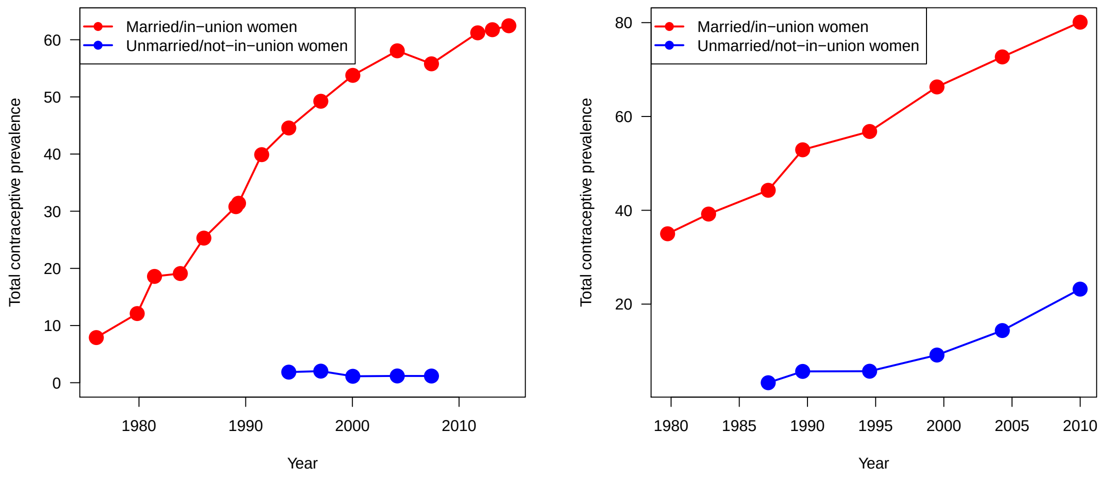

(a) Illustration: Bangladesh. **(b)** Illustration: Ecuador.

Figure G. Available data on total contraceptive prevalence. Ga Bangladesh; Gb Ecuador.

3.3.3 Systematic trends in unmet need

The country-specific systematic trend in the ratio of unmet need to no contraceptive use, $Z^*_{c,t}$, was modelled as a function of total prevalence $P_{c,t}$ using the same functional form as Cahill et al. (2017) and Alkema et al. (2013). We did this because we expected the systematic trend in unmet need as a function of total prevalence for UWRA would have the same characteristics as the trend for MWRA. The model for the ratio is given by:

$$Z^*_{c,t} = \frac{1}{1 + \exp(-z_c - \beta_1(P_{c,t} - 0.4) - \beta_2 \cdot (P_{c,t} - 0.4)^2)}, \tag{3.20}$$

with country-specific intercept z_c and world-level parameters $\{\beta_1, \beta_2\}$. (Note that 0.4 was subtracted from $P_{c,t}$ to reduce correlation between the z_c's and the β's; it does not affect the shape of the curve). This model was motivated by observed trends on the world and country level.

3.4 Bayesian hierarchical model

Estimating the country-specific parameters of the systematic trends presented a challenge because of the limited number of observations for each country. We used a Bayesian hierarchical model (Gelman et al., 2013; Lindley and Smith, 1972) to estimate the parameters in each country, such that the estimates were based on the observations in the country of interest, as well as the experiences of other countries. As described by Wheldon et al. (2019), we used the classification of countries based on estimated sexual activity (see Section 2.4) and United Nations (sub-)regional classifications (United Nations, 2019).

3.4.1 Hierarchical modelling and estimation by pooling

Cahill et al. (2017) and Alkema et al. (2013) used a four-level hierarchy based on United Nations (2019) geographical aggregates to improve estimation among MWRA for countries with few data points. The levels of the hierarchy were: i) country (e.g., Kenya), ii) subregion (e.g., Eastern

Africa), iii) region (e.g., Africa), and iv) world. Each country belonged to one of 22 subregions and each subregion belonged to one of six regions. The world consisted of all regions. The imposition of such a hierarchy had the effect of clustering countries together in subregions and clustering subregions into regions.

Clustering countries into subregions meant that country-specific parameters were estimated by pooling data within subregion; similarly, subregional parameters were estimated by pooling subregions within regions. This implied that results for countries in the same subregion were *a priori* expected to be more strongly correlated with one another than with countries in different subregions (Bijak and Bryant, 2016; Gelman et al., 2013). Under the assumption that countries within a subregion really are more similar to each other than to other countries in general, point estimates for countries with few observations from a hierarchical model are more accurate (less biased) and uncertainty intervals are narrower (more precise) than under a model with no hierarchical structure. In contrast, grouping dissimilar countries and subregions together can lead to biased parameter estimates and mis-estimation of precision.

3.4.2 Hierarchical model with sexual activity for unmarried women

Per country, data for contraceptive use among MWRA were scarce or not recent but there was at least one data point for each of the countries. Data for UWRA were more scarce and, in some cases, non-existent (see Section 2). In a hierarchical model, the impact of pooling on the results is greatest for countries with relatively few observations. Hence, the structure of the hierarchy is particularly important for UWRA.

Exploratory investigations identified countries that, based on prior subject matter knowledge, and data available, differed markedly from those in countries in the same geographic subregion. For example, in Eastern Africa, contraceptive prevalence among UWRA in Kenya was estimated to be over 10 percent in 2003 (DHS), while in Eritrea it was estimated to be 1.1 percent in 2010 (DHS). The Eastern Africa subregion also contains countries such as Somalia, where premarital sex is viewed as unacceptable. Therefore demand for family planning and, consequently, contraceptive prevalence among UWRA was expected to be low.

Variation in contraceptive prevalence among UWRA in many cases is likely due to variation in sexual activity. Sexual activity was not included in either of Cahill et al.'s (2017) or Alkema et al.'s (2013) models because being married was taken as a reasonable proxy for being sexually active in all countries. Something different was needed for UWRA. One approach to accounting for inter-country variation in sexual activity would be to enter it into the statistical model explicitly as a parameter to be estimated. This would have required the specification of its functional relationship with prevalence. However, sufficient data to estimate and check these were not available. We took a different approach and, instead, modified the hierarchical structure to include information about sexual activity by using the sexual activity classification described in Section 2.4. We retained a four-level hierarchy:

1. country,
2. region / subregion / India,
3. sexual activity group,
4. world

For countries in sexual activity group 0 (countries with very low levels of sexual activity), region was used at Level 2 for all countries except India which was treated as its own cluster. For countries in group 1 (all other countries), subregion was used at Level 2. Sexual activity group 0 had far fewer countries than group 1, making the use of subregions at Level 2 infeasible. The choice to model

India separately was based on exploratory data analysis and expert knowledge. Careful attention to India is warranted because the country's large population means that small changes in prevalence estimates translate to large changes in absolute numbers and India is of particular interest to the family planning research community (e.g., FP2020 2016). The structure is illustrated in Figure H and compared with the geographic structure in Table B.

Table B. Comparison of the purely geographic (original) and sexual activity inclusive (new) classifications of countries for estimating contraceptive prevalence among unmarried and not in a union women of reproductive age (UWRA).

	Classification Scheme	
	Geographic	Sexual Activity
Level 1	Country	Country
Level 2	Subregion	Region / Subregion / India
Level 3	Major Region	Sexual activity among UWRA
Level 4	World	World

3.4.3 Parameter definitions and hierarchical structure

Different levels of hierarchy were used for different sets of country parameters to best incorporate expected differences and similarities across countries, geographical areas, and sexual activity groups. Country-specific asymptotes for total contraceptive prevalence (denoted $\tilde{P}_c$) and the ratio of modern to total contraceptive use (denoted $\tilde{R}_c$) were estimated with a hierarchical model with two levels (world and country):

$$\log\left(\frac{\tilde{P}_c - 0.1}{1 - \tilde{P}_c}\right) \sim N(\tilde{P}_w, \kappa_P^{(c)}), \quad (3.21)$$

$$\log\left(\frac{\tilde{R}_c - 0.1}{1 - \tilde{R}_c}\right) \sim N(\tilde{R}_w, \kappa_R^{(c)}), \quad (3.22)$$

where both asymptotes were restricted to be between 10 percent and 100 percent, and $\tilde{P}_w$ is the world mean and $\kappa_P^{(c)}$ the variance of the $\tilde{P}_c$'s, and $\tilde{R}_w$ is the world mean and $\kappa_R^{(c)}$ the variance of the $\tilde{R}_c$'s. Alkema et al. (2013) and Cahill et al. (2017) restricted asymptotes to be above 50 percent for MWRA but this was considered too high for UWRA given the very low levels of contraceptive prevalence expected in some countries.

For pace parameters ω_c and ψ_c, four-level hierarchical models were used because these parameters were expected to vary across countries, (sub-)regions, and sexual activity groups. For pace parameters ω_c, the uptake of any method, the transformation

$$\omega_c^* = \log\left(\frac{\omega_c - 0.01}{0.5 - \omega_c}\right)$$

was used, such that ω_c was restricted to be between 0.01 and 0.5. This range was chosen to be weakly informative; it corresponds to assuming the duration of the transition from 10 percent to 90 percent of $\tilde{P}_c$ is between 10 and 400 years.

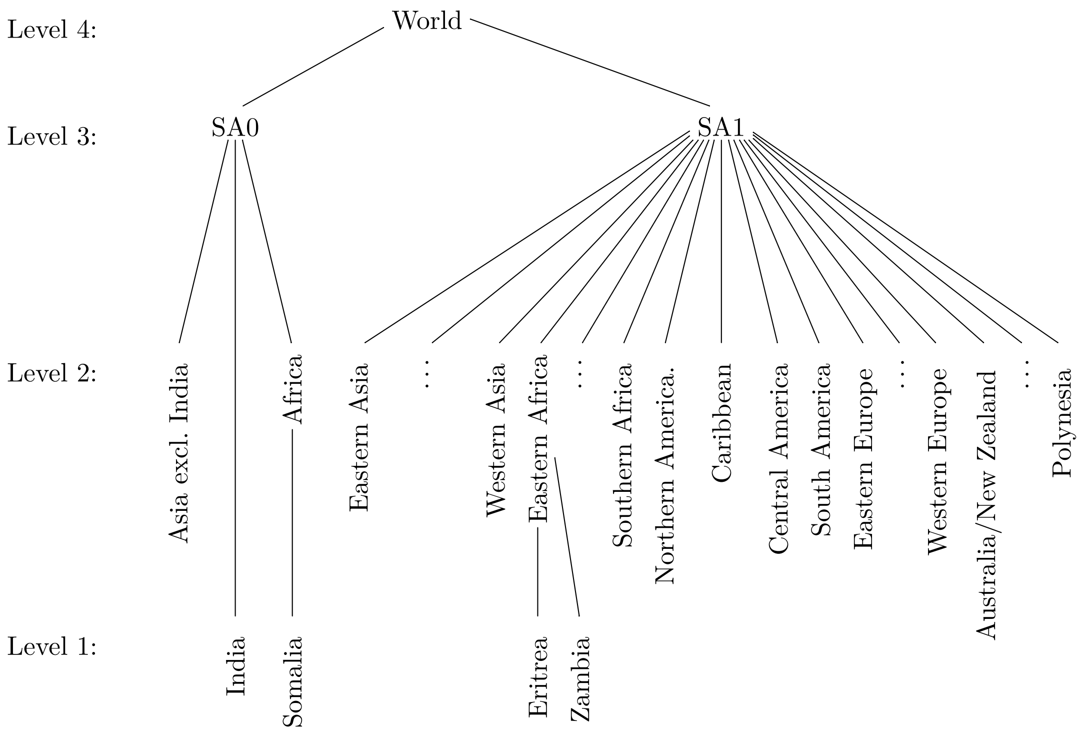

Figure H. Nested structure of the sexual activity inclusive hierarchy used to model contraceptive prevalance among unmarried and not in a union women of reproductive age (UWRA). Level 1 consists of individual countries which, save the examples, are omitted due to lack of space. "SA0" and "SA1" are sexual activity groups 0 and 1, respectively.

The hierarchical distributions for countries in sexual activity group 0 (denoted $c \in \text{SA0}$) were:

$$\text{Level 1:} \quad \omega_c^* \sim N(\omega_{r[c]}^*, \kappa_\omega^{(c)}),\ c \in \text{SA0} \tag{3.23}$$

$$\text{Level 2:} \quad \omega_r^* \sim N(\omega_{\text{SA0}}^*, \kappa_\omega^{(r)}), \tag{3.24}$$

$$\text{Level 3:} \quad \omega_{\text{SA0}}^* \sim N(\omega_w^*, \kappa_\omega^{(\text{SA})}). \tag{3.25}$$

(3.26)

ω_r^* (the Level 2 parameter) is the logistic trend for pace for region r, where India was considered a separate region. $r[c]$ is the region of country c. For countries in sexual activity group 1 (denoted $c \in \text{SA1}$):

$$\text{Level 1:} \quad \omega_c^* \sim N(\omega_{s[c]}^*, \kappa_\omega^{(c)}),\ c \in \text{SA1} \tag{3.27}$$

$$\text{Level 2:} \quad \omega_s^* \sim N(\omega_{\text{SA1}}^*, \kappa_\omega^{(s)}), \tag{3.28}$$

$$\text{Level 3:} \quad \omega_{\text{SA1}}^* \sim N(\omega_w^*, \kappa_\omega^{(\text{SA})}). \tag{3.29}$$

ω_s^* (the Level 2 parameter) is the logistic trend for pace for subregion s. $s[c]$ is the subregion of country c. This structure meant that the (logit-transformed) ω_c's were distributed around (sub-)regional means; $\omega_{r[s]}^*$ for countries in sexual activity group 0 and $\omega_{s[c]}^*$ for countries in group 1. The variances on the country, subregional and regional level were $\kappa_\omega^{(c)}$, $\kappa_\omega^{(s)}$ and $\kappa_\omega^{(r)}$ respectively.

Similarly, for pace parameter ψ_c, the uptake of modern methods as a proportion of any method,

$$\psi_c^* = \log\left(\frac{\psi_c - 0.01}{0.5 - \psi_c}\right)$$

$$\text{Level 1:} \quad \psi_c^* \sim N(\psi_{r[c]}^*, \kappa_\psi^{(c)}),\ c \in \text{SA0} \tag{3.30}$$

$$\text{Level 2:} \quad \psi_r^* \sim N(\psi_{\text{SA0}}^*, \kappa_\psi^{(r)}), \tag{3.31}$$

$$\text{Level 3:} \quad \psi_{\text{SA0}}^* \sim N(\psi_w^*, \kappa_\psi^{(\text{SA})}), \tag{3.32}$$

and

$$\text{Level 1:} \quad \psi_c^* \sim N(\psi_{s[c]}^*, \kappa_\psi^{(c)}),\ c \in \text{SA1} \tag{3.33}$$

$$\text{Level 2:} \quad \psi_s^* \sim N(\psi_{\text{SA1}}^*, \kappa_\psi^{(s)}), \tag{3.34}$$

$$\text{Level 3:} \quad \psi_{\text{SA1}}^* \sim N(\psi_w^*, \kappa_\psi^{(\text{SA})}), \tag{3.35}$$

The same structure was used for the timing of the uptake of modern methods as a proportion of any method, Ψ_c:

$$\text{Level 1:} \quad \Psi_c \sim N_T(\Psi_{r[c]}, \kappa_\Psi^{(c)}),\ c \in \text{SA0} \tag{3.36}$$

$$\text{Level 2:} \quad \Psi_r \sim N(\Psi_{\text{SA0}}, \kappa_\Psi^{(r)}), \tag{3.37}$$

$$\text{Level 3:} \quad \Psi_{\text{SA0}} \sim N(\Psi_w, \kappa_\Psi^{(\text{SA})}), \tag{3.38}$$

and

$$\text{Level 1:} \quad \Psi_c \sim N_T(\Psi_{s[c]}, \kappa_\Psi^{(c)}),\ c \in \text{SA1} \tag{3.39}$$

$$\text{Level 2:} \quad \Psi_s \sim N(\Psi_{\text{SA1}}, \kappa_\Psi^{(s)}), \tag{3.40}$$

$$\text{Level 3:} \quad \Psi_{\text{SA1}} \sim N(\Psi_w, \kappa_\Psi^{(\text{SA})}), \tag{3.41}$$

where the country-specific timings were restricted to be later than 1800 (a non-informative lower bound).

For countries in sexual activity group 0, the set-levels, $S_{c,t^*} := \text{logit}(P_{c,t^*})$, were modelled as distributed around a single mean:

$$S_{c,t^*} \sim N(S_{SA0,t^*}, \kappa_S^{(\text{SA0})}),\ c \in \text{SA0}. \tag{3.42}$$

For countries in sexual activity group 1, the following hierarchical structure was used:

$$\text{Level 1:} \quad S_{c,t^*} \sim N(S_{s[c],t^*}, \kappa_S^{(c)}),\ c \in \text{SA1} \tag{3.43}$$

$$\text{Level 2:} \quad S_{s,t^*} \sim N(S_{\text{SA1},t^*}, \kappa_S^{(s)}), \tag{3.44}$$

$$\text{Level 3:} \quad S_{\text{SA1},t^*} \sim N(S_{w,t^*}, \kappa_S^{(\text{SA1})}), \tag{3.45}$$

where $t^* = 1990$. There was no pooling between the sexual activity group parameters S_{SA0,t^*} and S_{SA1,t^*}. This is similar to what was done by Cahill et al. (2017) for MWRA, except they classified countries as "developed" and "developing" instead of according to sexual activity group. We did this because we wanted to ensure that the model was flexible enough to capture the difference in prevalence between the two sexual activity groups. Modeling the set-level parameters at the sexual activity group level as if they were from a common world distribution would have undermined this.

All other parameters were modelled as in Cahill et al. (2017).

3.5 Data Model

The data model was the same as Cahill et al.'s (2017), with one exception. Those authors rounded all direct estimates of prevalence less than one percent up to one percent to avoid computational difficulties due to numerical over/under-flow. This approach was not followed for UWRA because too many data points would have been affected, introducing bias. For these surveys, the approach described in Section 3.5.2 was used.

3.5.1 Total prevalence greater than one percent

The data model for observations with total prevalence greater than or equal to 1 percent was identical to that used by Cahill et al. (2017) and Alkema et al. (2013, Online Supplement, Section 2.3). Briefly, observations which provided an estimate of prevalence broken down by modern/traditional status were modelled using a bivariate normal distribution on the logit scale

$$\begin{bmatrix} \log\left(\frac{y_{i,1}}{y_{i,3+4}}\right) \\ \log\left(\frac{y_{i,2}}{y_{i,3+4}}\right) \end{bmatrix} \sim N\left(\begin{bmatrix} \log\left(\frac{q_{i,1}}{q_{i,3+4}}\right) \\ \log\left(\frac{q_{i,2}}{q_{i,3+4}}\right) \end{bmatrix}, \boldsymbol{\Sigma}_{S[i]}\right), \tag{3.46}$$

where $y_{i,3+4} = y_{i,3}+y_{i,4}$, the $q_{i,m}$ are the bias-adjusted and perturbed proportions (see Sections 3.5.7 and 3.5.8), and

$$\boldsymbol{\Sigma}_i = \begin{bmatrix} \sigma^2_{i,1} & \phi_i\sigma_{i,1}\sigma_{i,2} \\ \phi_i\sigma_{i,1}\sigma_{i,2} & \sigma^2_{i,2} \end{bmatrix} \tag{3.47}$$

In the above, $\sigma^2_{i,k}$ is the error variance of observation i coming from source S for the log-ratios $k = 1$ (traditional) and $k = 2$ (modern), and ϕ_i is the correlation of the log-ratios. Error variances were decomposed further into sampling and non-sampling components (see Section 3.5.4).

Observations providing only an estimate of total prevalence were modelled similarly but with a univariate normal:

$$\log\left(\frac{y_{i,1+2}}{1-y_{i,1+2}}\right) \sim N\left(\log\left(\frac{q_{i,1+2}}{1-q_{i,1+2}}\right), \sigma^2_T\right), \tag{3.48}$$

where σ^2_T is the error variance for total prevalence on the logit-transformed scale. A common error variance was assumed for all sources due to the small number of observations falling in this category.

3.5.2 Total prevalence less than one percent

For 41 observations (7.4 percent), estimated total prevalence was less than 1 percent ($y_1+y_2 < 0.01$). These observations were found to have a large influence on posterior estimates of source variances ($\sigma^2_{S,k}$). This appeared to be a side effect of the transformation used. On the logistic scale a few small proportions become extreme outliers after transformation and the resulting set of transformed observations are not well-modelled by a single (source-specific) logistic-normal distribution. To account for this, results from all surveys reporting a total prevalence estimate of less than or equal to one percent were assigned to the new source type. This was done irrespective of the original source type (DHS, MICS, etc.).

3.5.3 Unmet need

The data model for the break-down of women who do not use any method (categories 3 and 4) into the category unmet/no need was the same as that used by Cahill et al. (2017):

$$\text{logit}\left(\frac{y_{i,3}}{y_{i,3+4}}\right) = \log\left(\frac{y_{i,3}}{y_{i,4}}\right) \sim N\left(\log\left(\frac{q_{i,3}}{q_{i,4}}\right), \sigma^2_{S[i],3}\right), \tag{3.49}$$

where $\sigma^2_{S[i],3}$ is the error variance of observation i coming from source S for the log-ratios of unmet need to no need. This model was used irrespective of the estimate for total prevalence. Error variances were decomposed further into sampling and non-sampling components (see Section 3.5.4).

3.5.4 Sampling and non-sampling errors

The variance components of the data models for contraceptive prevalence and unmet need for observations providing a breakdown between modern and traditional methods (Eqns. (3.47) and (3.49)) were decomposed into sampling error, $\nu_{i,k}$, and non-sampling error, $\zeta^2_{S[i],k}$. One correlation parameter, $r_{S[i]}$, was used for all observations with a common source.

$$\sigma^2_{i,k} = \nu^2_{i,k} + \zeta^2_{S[i],k} \tag{3.50}$$

$$\phi_i = r_{S[i]} \tag{3.51}$$

Estimates of sampling error were derived from microdata, thereby using information about the survey-design when available. The $\nu_{i,k}$ were fixed at these estimated values. Sampling errors for breakdown observations were imputed when insufficient information was available to calculate them. Details are given in Cahill et al. (2017, Supplementary Appendix Section 2.5). The variance components for observations not providing a breakdown into modern and traditional prevalence were not decomposed in this way as none had sufficient survey design information.

3.5.5 Reference periods

The data model means (Eqns. (3.46), (3.48), (3.49)) applied to the entire reference period of the survey. See Cahill et al. (2017, Supplementary Appendix Section 2.6) for further details.

3.5.6 Data categorization based on source types

The number of observations by data source category, estimate of total prevalence (less than, or greater than or equal to, 1 percent), and availability of modern-traditional breakdown are shown in Table C. Separate variance-covariance matrices (Σ_S) were estimated for observations with a modern-traditional breakdown. The data model for unmet need grouped all non-DHS observations together in one category.

3.5.7 Data categorization based on characteristics of the population sampled and perturbation multipliers

As in Cahill et al. (2017) and Alkema et al. (2013), perturbation multipliers were included to account for differences between the characteristics of sampled populations and the base population. Table D shows the seven categories of different characteristics that were summarized.

The first two categories describe differences specific to sampled populations of UWRA. Category one comprises observations from surveys where questions on contraceptive use were only asked

Table C. Number of observations for contraceptive prevalence and unmet need by data model source and availability of breakdown of contraceptive use into use of modern methods and use of traditional methods.

Indicator	Trad./Mod. Breakdown	DHS	MICS	PMA	National survey	Other	CP < 1%	Total
Contraceptive use	Avail.	176	92	34	79	106	27	511
	Unavail.	—	—	—	16	—	14	40
	Total	176	92	34	95	53	41	551
Unmet	—	—	—	—	—	10	—	282

Table D. Categorisation of non-base population samples, number of observations in each category and comparison of the expected prevalence levels in the non-base category compared to the base category of unmarried and not in a union women of reproductive age (UWRA).

No.	Label	Characteristics of sample population	# obs.	Contraceptive use compared to base population of UWRA
1	With partner only	Contraceptive use questions were asked only among women with a partner	10	Modern and traditional use expected to be lower
2	Sterilization only	Unmarried/Not-in-union data pertain to female sterilization only	18	Modern and traditional use expected to be lower
3	Geographical region	Specific geographical region or population group	14	Potentially different
4	Higher risk of pregnancy	Data pertain to women exposed to an elevated risk of pregnancy, e.g., recently sexually active or incl. women in cohabiting unions.	5	Modern and traditional use expected to be higher
5	Age group with - bias	Age group starts at ages 13-17 but ends after 51	1	Modern and traditional use expected to be lower
6	Age group with + bias	Age groups starts at ages 18-25 and ends before 51	37	Modern and traditional use expected to be higher
7	Age group different	Other age group (not described by groups 6 and 7)	40	Potentially different

among UWRA who had a non-cohabiting partner. Women without a partner, while included in the samples, were not asked about contraceptive use and were therefore not counted in the numerator when calculating the family planning indicators. As a result, observations of contraceptive use were expected to be too low. This concerns the majority of observations (10) from the first and second rounds of the Gender and Generation Program. Category two consists of observations (18) from DHS data that pertain to female sterilisation only (asked of formerly married women only). Other contraceptive methods were not reported for UWRA so that these samples under-estimate contraceptive use.

Categories three to seven describe differences between characteristics of sampled populations and the base population. Category three refers to samples covering specific geographic regions or population groups with potentially different levels of contraceptive prevalence compared to the base population (14 observations). Category four includes observations that covered women living in a cohabiting union in the group of UWRA (5) because this was likely to have elevated the risk of pregnancy. This was the case for data from the German 1985 Survey on Family Planning Behaviour and from the Japan 2014 Biodemography Project Survey. Contraceptive prevalence among UWRA is expected to be higher for these two observations than for the base population due to the inclusion of women in cohabiting unions who tend to have higher levels of contraceptive use.

Categories five, six and seven apply to observations from surveys, which sampled UWRA populations in age groups other than 13–51 years, which was set as the “baseline” population, allowing for some flexibility outside the nominal 15–49 years age range. Age groups starting at ages between 13 and 17 (inclusive) and ending at ages 52 or above (1 observation) were assumed to have lower contraceptive use than the baseline. Age groups starting at ages between 18 and 25 (inclusive) and ending at ages 51 and below (37 observations) were expected to have higher contraceptive use relative to baseline. Sample populations with other age ranges (40 observations) were deemed to be potentially different, but with unknown direction.

Perturbation multipliers to model these expected differences in prevalence between non-baseline groups and UWRA were applied in the same way as in Cahill et al. (2017) and Alkema et al. (2013). The perturbed compositional vector for observation i is denoted $\tilde{\boldsymbol{q}}_i = (\tilde{q}_{i,1}, \tilde{q}_{i,2}, \tilde{q}_{i,3}, \tilde{q}_{i,4})$.

3.5.8 Misclassification biases

Bias parameters were included in the model to account for survey misclassification errors; that is, women who were classified as belonging to one contraceptive use component when they should have been classified as belonging to another. The same parameters as used by Cahill et al. (2017) and Alkema et al. (2013, see Supplementary Appendix Section 2.3.3) for MWRA were used here for UWRA. These were:

1. Exclusion of sterilization from modern method use, expected to have lead to under-reporting of total and modern method use (9 observations).
2. Inclusion of sterilization for non-contraceptive reasons in modern method use, expected to have led to over-reporting of total and modern method use (24 observations).
3. Inclusion of folk methods in traditional method use, expected to have lead to over-reporting of total and traditional method use (26 observations).
4. Absence of probing questions about knowledge of contraceptive methods, expected to have led to under-reporting of traditional method use (94 observations).

The corrected (perturbed and bias adjusted) compositional vector for observation i is denoted $\boldsymbol{q}_i = (q_{i,1}, q_{i,2}, q_{i,3}, q_{i,4})$.

3.5.9 Bias and perturbation parameters: The examples of Austria and Belgium

Misclassification biases were included to account for survey misclassification errors, while perturbation multipliers were intended to account for errors due to sampling from a population different from the target (i.e., UWRA). We explain by way of the examples of Austria and Belgium. The model inputs for these two countries are in Table E. The age groups targeted by the surveys available for Austria and Belgium were all different from the nominal range 15–49, hence a perturbation multiplier for "Age group different" (group 7, Table D) will be applied. The observation for Belgium from the 2009 GGS pertains not to UWRA but to partnered women (PW) only so a multiplier for "With partner only" (group 1, Table D) will also be applied. Finally, since the GGS for these two countries counted sterilization for non-contraceptive reasons as modern method use, a portion of those women classified as modern method users need to be re-classified as non-users. This is done through the misclassification bias parameter $\gamma_{2,4}$, where subscripts correspond to the components of the composition $\boldsymbol{p}_{c,t} = p_{c,t}$ (see Section 3.2).

Table E. Example: Input data for Austria and Belgium, including indicators used to estimate misclassification biases and perturbation multipliers. Data source 'GGS' is the Generations and Gender Survey (GGS) and 'NS' is national surveys; population type 'PW' is partnered women (PW).
Source: UN Population Division (2018b) and surveys as indicated.

Country	Year	Age	Data source	Source In model	Modern use	Trad. use	Pop. type	Age bias	Modern method bias
Austria	2013.1	18-49	GGS	Other	57.37	1.64	UWRA	+	+
Belgium	2009.2	18-49	GGS	Other	35.00	0.20	PW	+	+
Belgium	2013.5	15-54	NS	NS	55.32	0.71	UWRA	-	none

3.6 Full model specification and prior distributions

3.6.1 List of main symbols

$p_{c,t,m}$	Unobserved proportion of UWRA in country c, year t in category m (referring to traditional and modern use, unmet need and no need respectively)
$n_{c,t,m}$	Unobserved number of UWRA in country c, year t in category m (referring to traditional and modern use, unmet need and no need respectively)
$P_{c,t}$	Total contraceptive prevalence in country c, year t
$R_{c,t}$	Ratio of modern to total prevalence in country c, year t
$Z_{c,t}$	Ratio of unmet need to no method in country c, year t
$S_{c,t}$	logit($P_{c,t}$)
$P^*_{c,t}$	Systematic trend in $P_{c,t}$
$R^*_{c,t}$	Systematic trend in $R_{c,t}$
$Z^*_{c,t}$	Systematic trend in $Z_{c,t}$
$\tilde{P}_c$	Asymptote of $P^*_{c,t}$
$\tilde{R}_c$	Asymptote of $R^*_{c,t}$
ψ_c	Pace parameter for increase in $R^*_{c,t}$
ω_c	Pace parameter for increase in $P^*_{c,t}$
Ψ_c	Midpoint for increase in $R^*_{c,t}$
P_{c,t^*}	Set-level of prevalence, $P_{c,t}$, $t^* = 1990$.
z_c	"Intercept" parametric model for $Z^*_{c,t}$
$\{\beta_1, \beta_2\}$	Coefficients of parametric model for $Z^*_{c,t}$
$\kappa_{\cdot}^{(\cdot)}$	Variance parameter in hierarchical distributions on country, subregional and regional level ($\kappa_{\cdot}^{(c)}$, $\kappa_{\cdot}^{(s)}$ and $\kappa_{\cdot}^{(r)}$ respectively)
$\varepsilon_{c,t}$	AR(1) distortion for $P_{c,t}$
$\eta_{c,t}$	AR(1) distortion for $R_{c,t}$
$\theta_{c,t}$	AR(1) distortion for $Z_{c,t}$
$\{\rho_\varepsilon, \rho_\eta, \rho_\theta\}$	Autoregressive coefficients for the AR(1) distortions
$\{\tau_\varepsilon, \tau_\eta, \tau_\theta\}$	Variance parameters of the AR(1) distortions

$q_{i,m}$	Perturbed and bias-adjusted proportion of women for observation i
$y_{i,m}$	Observed proportion of women in observation i
$\gamma_{n,m}$	Misclassification bias parameter (from category n to m)
$\{\xi_m^2, \lambda_m^2\}$	Variance parameters for perturbation multipliers for $m = 1, 2$
μ_p	Mean of (transformed) perturbation multipliers (that were expected to be different from 1)
σ_T^2	Error variance for all observations providing only an estimate of total prevalence
$\sigma_{i,k}^2$	Error variance for observation i, for traditional/total use, modern/total use and unmet need/no use ($k = 1, 2, 3$)<br>$\sigma_{i,k}^2 = \nu_{i,k}^2 + \zeta_{S[i],k}^2$, where observation i comes from source S
$\nu_{i,k}^2$	Sampling error variance for observation i, for traditional/total use, modern/total use and unmet need/no use ($k = 1, 2, 3$)
$\zeta_{S[i],k}^2$	Non-sampling error variance for observation i coming from source S, for traditional/total use, modern/total use and unmet need/no use ($k = 1, 2, 3$)
ϕ_i	Correlation parameter for observation i coming from source S.<br>$\phi_i = r_{S[i]}$
$r_{S[i]}$	Correlation parameter for all observations i coming from source S.
$V_{j,m}^{(g)}$	j-th Multiplier for perturbation category g, contraceptive use category m

3.6.2 List of indices

These symbols index the following quantities when used as indices of the main symbols.

c	country
g	perturbation category
i	observation (i.e,. one survey data point)
j	perturbation multiplier for a given perturbation category, g
k	data model (traditional/total use, modern/total use and unmet need/no use ($k = 1, 2, 3$)
m	contraceptive prevalence category (referring to traditional and modern use, unmet need and no need respectively)
r, $r[s]$, $r[c]$	r indicates region (e.g., Africa), $r[s]$ or $r[c]$ indicates the region the subregion or country belongs to
s, $s[c]$	s indicates subregion (e.g., Eastern Africa), $s[c]$ indicates subregion country c belongs to
S	observation source (e.g., DHS, MICS, etc.)
$S[i]$	source (e.g., DHS, MICS, etc.) from which observation i comes
SA, $SA[c]$,	SA indicates sexual activity group (0 or 1), $SA[c]$ indicates the sexual activity group country c belongs to
t	time (in years)
w	world

3.6.3 Model specification

$$p_{c,t,1} = (1 - R_{c,t}) \cdot P_{c,t} \tag{3.52}$$
$$p_{c,t,2} = R_{c,t} \cdot P_{c,t} \tag{3.53}$$
$$p_{c,t,3} = (1 - P_{c,t}) \cdot Z_{c,t} \tag{3.54}$$
$$p_{c,t,4} = (1 - P_{c,t}) \cdot (1 - Z_{c,t}) \tag{3.55}$$
$$R_{c,t} = \text{logit}^{-1}\left(\text{logit}(R^*_{c,t}) + \eta_{c,t}\right) \tag{3.56}$$
$$Z_{c,t} = \text{logit}^{-1}\left(\text{logit}(Z^*_{c,t}) + \theta_{c,t}\right) \tag{3.57}$$
$$\eta_{c,t} \sim N(\rho_\eta \cdot \eta_{c,t-1}, \tau^2_\eta) \tag{3.58}$$
$$\theta_{c,t} \sim N(\rho_\theta \cdot \theta_{c,t-1}, \tau^2_\theta) \tag{3.59}$$
$$\eta_{c,t_{c,1}} \sim N\left(0, \frac{\tau^2_\eta}{1 - \rho^2_\eta}\right) \tag{3.60}$$
$$\theta_{c,t_{c,1}} \sim N\left(0, \frac{\tau^2_\theta}{1 - \rho^2_\theta}\right) \tag{3.61}$$
$$R^*_{c,t} = \frac{\tilde{R}_c}{1 + \exp(-\psi_c(t - \Psi_c))} \tag{3.62}$$
$$Z^*_{c,t} = \frac{1}{1 + \exp(z_c + \beta_1(P_{c,t} - 0.4) + \beta_2 \cdot (P_{c,t} - 0.4)^2)} \tag{3.63}$$

$$S_{c,t} \coloneqq \text{logit}(P_{c,t}) \tag{3.64}$$
$$t > t^*\colon S_{c,t} = \begin{cases} \text{logit}\left(\tilde{P}_c \, \text{logit}^{-1}\left(\text{logit}\left(\frac{P_{c,t-1}}{\tilde{P}_c}\right) + \omega_c\right)\right) + \varepsilon_{c,t}, & \text{when } P_{c,t-1} < \tilde{P}_c, \\ S_{c,t-1} + \varepsilon_{c,t}, & \text{otherwise.} \end{cases} \tag{3.65}$$
$$t < t^*\colon S_{c,t} = \begin{cases} \text{logit}\left(\tilde{P}_c \, \text{logit}^{-1}\left(\text{logit}\left(\frac{\text{logit}^{-1}(S_{c,t+1} - \varepsilon_t)}{\tilde{P}_c}\right) - \omega_c\right)\right), & \text{when } \text{logit}^{-1}(S_{c,t+1} - \varepsilon_{c,t}) < \tilde{P}_c, \\ S_{c,t+1} - \varepsilon_{c,t}, & \text{otherwise.} \end{cases} \tag{3.66}$$
$$\varepsilon_{c,t} \sim N(\rho_\varepsilon \cdot \varepsilon_{c,t-1}, \tau^2_\varepsilon) \tag{3.67}$$
$$\varepsilon_{c,t_{c,1}} \sim N\left(0, \frac{\tau^2_\varepsilon}{1 - \rho^2_\varepsilon}\right) \tag{3.68}$$

$$\log\left(\frac{\tilde{P}_c - 0.1}{1 - \tilde{P}_c}\right) \sim N(\tilde{P}_w, \kappa_P^{(c)}) \tag{3.69}$$

$$\log\left(\frac{\tilde{R}_c - 0.1}{1 - \tilde{R}_c}\right) \sim N(\tilde{R}_w, \kappa_R^{(c)}) \tag{3.70}$$

$$\omega_c^* = \log\left(\frac{\omega_c - 0.01}{0.5 - \omega_c}\right) \tag{3.71}$$

$$\omega_c^* \sim \begin{cases} N(\omega_{r[c]}, \kappa_\Omega^{(c)}), & c \in SA0 \\ N(\omega_{s[c]}, \kappa_\Omega^{(c)}), & c \in SA1 \end{cases} \tag{3.72}$$

$$\omega_s^* \sim N(\omega_{SA1}^*, \kappa_\omega^{(s)}) \tag{3.73}$$

$$\omega_r^* \sim N(\omega_{SA0}^*, \kappa_\omega^{(r)}) \tag{3.74}$$

$$\omega_{SA0}^* \sim N(\omega_w^*, \kappa_\omega^{(SA)}) \tag{3.75}$$

$$\omega_{SA1}^* \sim N(\omega_w^*, \kappa_\omega^{(SA)}) \tag{3.76}$$

$$\psi_c^* = \log\left(\frac{\psi_c - 0.01}{0.5 - \psi_c}\right) \tag{3.77}$$

$$\psi_c^* \sim \begin{cases} N(\psi_{r[c]}, \kappa_\psi^{(c)}), & c \in SA0 \\ N(\psi_{s[c]}, \kappa_\psi^{(c)}), & c \in SA1 \end{cases} \tag{3.78}$$

$$\psi_s^* \sim N(\psi_{SA1}^*, \kappa_\psi^{(s)}) \tag{3.79}$$

$$\psi_r^* \sim N(\psi_{SA0}^*, \kappa_\psi^{(r)}) \tag{3.80}$$

$$\psi_{SA0}^* \sim N(\psi_w^*, \kappa_\psi^{(SA)}) \tag{3.81}$$

$$\psi_{SA1}^* \sim N(\psi_w^*, \kappa_\psi^{(SA)}) \tag{3.82}$$

$$\Psi_c \sim \begin{cases} N_T(\Psi_{r[c]}, \kappa_\Psi^{(c)}), & c \in SA0 \\ N_T(\Psi_{s[c]}, \kappa_\Psi^{(c)}), & c \in SA1 \end{cases} \tag{3.83}$$

$$\Psi_s \sim N(\Psi_{SA1}, \kappa_\Psi^{(s)}) \tag{3.84}$$

$$\Psi_r \sim N(\Psi_{SA0}, \kappa_\Psi^{(r)}) \tag{3.85}$$

$$\Psi_{SA0} \sim N(\Psi_w, \kappa_\Psi^{(SA)} \tag{3.86}$$

$$\Psi_{SA1} \sim N(\Psi_w, \kappa_\Psi^{(SA)} \tag{3.87}$$

$$S_{c,t^*} \sim \begin{cases} N(S_{SA0,t^*}, \kappa_S^{(\mathrm{SA0})}), & c \in SA0 \\ N(S_{s[c],t^*}, \kappa_S^{(c)}), & c \in SA1 \end{cases} \tag{3.88}$$

$$S_{s,t^*} \sim N(S_{\mathrm{SA1},t^*}, \kappa_S^{(s)}) \tag{3.89}$$

$$S_{\mathrm{SA1},t^*} \sim N(S_{w,t^*}, \kappa_S^{(\mathrm{SA1})}) \tag{3.90}$$

$$z_c \sim N(z_{s[c]}, \kappa_z^{(c)}) \tag{3.91}$$

$$z_s \sim N(z_w, \kappa_z^{(r)}) \tag{3.92}$$

Data Model

$$\left[\begin{array}{c}\log\left(\frac{y_{i,1}}{y_{i,3+4}}\right)\\ \log\left(\frac{y_{i,2}}{y_{i,3+4}}\right)\end{array}\right] \sim N\left(\left[\begin{array}{c}\log\left(\frac{q_{i,1}}{q_{i,3+4}}\right)\\ \log\left(\frac{q_{i,2}}{q_{i,3+4}}\right)\end{array}\right], \boldsymbol{\Sigma}_i\right),\ \boldsymbol{y}_i = (y_{i,1}, y_{i,2}, y_{i,3}, y_{i,4}) \tag{3.93}$$

$$\boldsymbol{\Sigma}_i = \left[\begin{array}{cc}\sigma^2_{i,1} & \phi_i\sigma_{i,1}\sigma_{i,2}\\ \phi_i\sigma_{i,1}\sigma_{i,2} & \sigma^2_{i,2}\end{array}\right] \tag{3.94}$$

$$\log\left(\frac{y_{i,1+2}}{1-y_{i,1+2}}\right) \sim N\left(\log\left(\frac{q_{i,1+2}}{1-q_{i,1+2}}\right), \sigma^2_T\right),\ \boldsymbol{y}_i = (y_{i,1+2}, y_{i,3}, y_{i,4}) \tag{3.95}$$

$$\log\left(\frac{y_{i,3}}{y_{i,4}}\right) \sim N\left(\log\left(\frac{q_{i,3}}{q_{i,4}}\right), \sigma^2_{i,3}\right) \tag{3.96}$$

$$\sigma^2_{i,k} = \nu^2_{i,k} + \zeta^2_{S[i],k} \tag{3.97}$$

$$\phi_i = r_{S[i]} \tag{3.98}$$

Perturbation multipliers

$$\tilde{q}_{i,m} = \frac{p_{c[i],t[i],m} \cdot v_{i,m}}{\sum_{n=1}^{4} p_{i,n} \cdot v_{i,n}} \tag{3.99}$$

$$v_{i,m} = \prod_{g=1}^{G} V^{(g)}_{i,m} \tag{3.100}$$

$$\tilde{V}^{(g)}_{i,m} = \begin{cases} 1 & \text{if } m = 3,4 \text{ or if } i \notin S^{(g)},\\ V^{(g)}_{j[i,g],m} & \text{if } m = 1,2 \text{ and if } i \in S^{(g)}, \end{cases} \tag{3.101}$$

$$V^{(g)}_{j,m} \begin{cases} \sim \text{logN}(0, \xi^2_m), & \text{for } g = 1,2,3,6,\ m = 1,2 \text{ and for } g = 4, m = 1\\ = 1/(1 + W^{(g)}_{j,m}), & \text{for } g = 8, m = 1,2\\ = 1 + W^{(g)}_{j,m}, & \text{otherwise,} \end{cases} \tag{3.102}$$

$$\log(W^{(g)}_{j,m}) \begin{cases} = \mu_1, & \text{for } m = 1\\ \sim N(\mu_2, \lambda^2_2), & \text{for } m = 2, \end{cases} \tag{3.103}$$

Misclassification biases

$$q_{i,1} = \tilde{q}_{i,1}(1-\gamma_{1,3}) + \tilde{q}_{i,3}\gamma_{3,1} \tag{3.104}$$

$$q_{i,2} = \tilde{q}_{i,2}(1-\gamma_{2,4}) + \tilde{q}_{i,4}\gamma_{4,2} \tag{3.105}$$

$$q_{i,3} = \tilde{q}_{i,3}(1-\gamma_{3,1}) + \tilde{q}_{i,1}\gamma_{1,3} \tag{3.106}$$

$$q_{i,4} = \tilde{q}_{i,4}(1-\gamma_{4,2}) + \tilde{q}_{i,2}\gamma_{2,4} \tag{3.107}$$

3.6.4 Prior distributions

Spread-out prior distributions were used for the world-level mean parameters of the logistic curves and parametric function for unmet need:

$$\tilde{P}_w \sim N(0, 10^2) \quad (3.108)$$
$$\tilde{R}_w \sim N(0, 10^2) \quad (3.109)$$
$$\omega^*_w \sim N(-1, 10^2) \quad (3.110)$$
$$S_{\text{SA0},t^*} \sim N(-1, 10^2) \quad (3.111)$$
$$S_{\text{SA1},t^*} \sim N(-1, 10^2) \quad (3.112)$$
$$\psi^*_w \sim N(-1, 10^2) \quad (3.113)$$
$$\Psi_w \sim N(1980, 50^2) \quad (3.114)$$
$$z_w \sim N(-2, 1) \quad (3.115)$$
$$\beta_1 \sim N(-6, 5^2) \quad (3.116)$$
$$\beta_2 \sim U(-35, 0) \quad (3.117)$$

The priors on the correlations and non-sampling error variance parameters in the data model (see Sections 3.5.1–3.5.4 and Eqns (3.93)–(3.51)) were defined marginally in *JAGS* as:

$$\sigma_T \sim \text{IGamma}(0.5, 0.5 \cdot 0.15^2) \quad (3.118)$$
$$\zeta_{S,k} \sim \text{U}(0.01, 2) \quad (3.119)$$
$$r_S \sim \text{U}(-1, 1) \quad (3.120)$$

The covariance matrices for observations with modern/traditional breakdown, $\boldsymbol{\Sigma}_{S[i]}$ were constrained to be positive definite. The prior on σ_T was somewhat informative due to the small number of observations with no modern/traditional breakdown. It was set using estimates from observations that did provide a modern/traditional breakdown. The priors for the $\zeta_{S,k}$s and r_S were intended to be proper but non-informative. Examination of traceplots and prior/posterior plots for these parameters did not suggest the posteriors were restricted by the priors.

Unless specified below, the remaining prior distributions were the same as those used by Cahill et al. (2017, Supplementary Appendix, Section 2):

$$\sqrt{\kappa_{\cdot}^{(c)}} \sim \text{U}(0, 5) \quad (3.121)$$
$$\sqrt{\kappa_{\cdot}^{(s,r,SA0,SA1)}} \sim \text{U}\big(0, K_{\kappa,\cdot}^{(s,r,SA0,SA1)}\big) \quad (3.122)$$

$K_{\kappa,\cdot}^{(s,r,SA0,SA1)}$ was set large enough to ensure the prior did not restrict the posterior.

3.7 Computation and Inference

Samples from the joint posterior distribution of the model parameters (e.g., $\tilde{P}_{c,t}$, $\omega_{c,t}$, $\varepsilon_{c,t}$, etc.) were obtained via Markov chain Monte Carlo (MCMC) sampling implemented in the statistical software packages *R 3.5.2* (R Core Team, 2018) and *JAGS 4.2.0* (Plummer, 2003, 2015), and *R*-packages *R2jags 0.5-7* (Su and Yajima, 2015) and *rjags 4-6* (Plummer et al., 2016). We ran 20 chains, discarded the first 20 000 as burn-in, and kept every 30th iteration thereafter. The total number of saved iterations before thinning, across all chains, was 500 000. Convergence of the MCMC algorithm and the sufficiency of the number of samples obtained was checked through

visual inspection of trace plots and convergence diagnostics of Raftery and Lewis (Raftery and Lewis, 1992a,b, 1996), and Gelman and Rubin (1992), both implemented in the *coda* package (Plummer et al., 2006).

The trajectories of contraceptive prevalence and unmet need for each country were obtained from the MCMC sample by transforming the vector of country-specific model parameters into the indicators in the same way as done by Alkema et al. (2013, Supplementary Appendix, Section 2.5). We summarized the joint posterior distribution with 2.5, 50 (median) and 97.5 percentiles of each parameter for each country, for each year from 1970 to 2030. Our preferred point estimates (the "best" estimates) are the median outcomes in each year.

3.7.1 Countries and parameters without data

Fifty-nine countries had no data on contraceptive prevalence among UWRA. Sixty-four countries had data on prevalence, but not on unmet need (all countries with data on unmet need also had data on prevalence). Estimates of prevalence and unmet need in these cases were based on samples from the respective hierarchical distributions as described below.

Estimates of unmet need for countries without data on unmet need were derived using the method described by Alkema et al., 2013, Supplementary Appendix, Section 2.5. For countries without data on contraceptive prevalence, the same idea was applied. For example, the jth sample $R^{*(j)}_{c,t}$ for a country c with no prevalence data was defined as

$$R^{*(j)}_{c,t} = \frac{\tilde{R}^{(j)}_c}{1+\exp(-\psi^{(j)}_c(t-\Psi^{(j)}_c))} \tag{3.123}$$

where the component parameters were sampled from their hierarchical distributions. For example,

$$\log\left(\frac{\tilde{R}^{(j)}_c - 0.1}{1-\tilde{R}^{(j)}_c}\right) \sim N(\tilde{R}^{(j)}_w, \kappa^{(c),(j)}_P) \tag{3.124}$$

$$\psi^{*(j)}_c = \log\left(\frac{\psi^{(j)}_c - 0.01}{0.5-\psi^{(j)}_c}\right) \tag{3.125}$$

$$\psi^{*(j)}_c \sim \begin{cases} N(\psi^{(j)}_{r[c]}, \kappa^{(r),(j)}_\psi), & c \in SA0, \\ N(\psi^{(j)}_{s[c]}, \kappa^{(c),(j)}_\psi), & c \in SA1, \end{cases} \tag{3.126}$$

$$\psi^{*(j)}_s \sim N(\psi^{*(j)}_{SA1}, \kappa^{(s),(j)}_\psi) \tag{3.127}$$

$$\psi^{*(j)}_r \sim N(\psi^{*(j)}_{SA0}, \kappa^{(r),(j)}_\psi) \tag{3.128}$$

$$\psi^{*(j)}_{SA0} \sim N(\psi^{*(j)}_w, \kappa^{(SA),(j)}_\psi) \tag{3.129}$$

$$\psi^{*(j)}_{SA1} \sim N(\psi^{*(j)}_w, \kappa^{(SA),(j)}_\psi) \tag{3.130}$$

3.7.2 Estimates and projections of numbers of users

The model produces estimates and projections on the proportion scale, where the proportion expresses the share of each country's population in each of the four categories of interest: prevalences of modern method use ($p_{c,t,1}$), traditional method use ($p_{c,t,2}$), unmet need ($p_{c,t,3}$), and no need ($p_{c,t,4}$; see Section 3.2). However, estimates on the count scale were also of interest. For example, at the 2012 London Summit the Family Planning 2020 (FP2020) initiative set a goal of 120 million additional users of modern contraceptives by 2020 (FP2020, 2016; Stover and Sonneveldt, 2017).

Estimates and projections of numbers of users, and number experiencing unmet need, were produced by using the estimated number of UWRA in each country to transform the proportion vector ($\boldsymbol{p}_{c,t}$) to a count vector. Denoting the number of UWRA in country c in year y as $W_{c,t}$, the number of women in each category, $\boldsymbol{n}_{c,t} = (n_{c,t,1}, n_{c,t,2}, n_{c,t,3}, n_{c,t,4})$, was calculated as

$$\boldsymbol{n}_{c,t} = W_{c,t} \cdot \boldsymbol{p}_{c,t}.$$

3.7.3 Estimates and projections for country aggregates

Estimates and projections for country aggregates (e.g., for sexual activity groups or (sub)-regions) were constructed following Alkema et al. (2013, Supplementary Appendix, Section 2.5.1), that is by summing numbers of users over countries in the respective aggregate on the count scale. These were summarized by sample quantiles, as was done for the $\boldsymbol{p}_{c,t}$. This approach assumes that future/past distortions of the time trends are independent.

3.7.4 Estimates and projections for women of reproductive age irrespective of marital status

Estimates and projections for WRA were derived as follows. A posterior distribution of counts was constructed by summing MCMC sample trajectories of numbers of users and numbers experiencing unmet need among UWRA and MWRA, within country, within year. Sample trajectories of counts for MWRA were obtained from the latest model-based estimates and projections of family planning indicators (UN Population Division, 2019). For country c, year t, denote the jth count trajectory for WRA, MWRA, and UWRA as $\boldsymbol{n}_{c,t}^{[\text{WRA}],(j)}$, $\boldsymbol{n}_{c,t}^{[\text{MWRA}],(j)}$, $\boldsymbol{n}_{c,t}^{[\text{UWRA}],(j)}$, respectively. Then,

$$\boldsymbol{n}_{c,t}^{[\text{WRA}],(j)} = \boldsymbol{n}_{c,t}^{[\text{MWRA}],(j)} + \boldsymbol{n}_{c,t}^{[\text{UWRA}],(j)}, \; j = 1, \ldots, J \tag{3.131}$$

where J is the number of trajectories in the smaller of the two sets ([UWRA] and [MWRA]). Note that, since the MCMC samples for UWRA and MWRA are random samples, hence in a random order, we obtain a random sample for WRA regardless of which UWRA trajectory is paired with, and added to, which MWRA (as long as each trajectory is used only once). For convenience, we add them in the order they appear in the dataset. The $\boldsymbol{n}_{c,t}^{[\text{WRA}],(j)}$ can be summarized by sample quantiles in the usual way. They can also be converted to proportions for WRA using the method in Section 3.7.3 but using the numbers of WRA instead. This approach assumes that the [UWRA] and [MWRA] trajectories are conditionally independent, given the data.

3.7.5 Inference on changes

Inference about changes in the indicators over set time periods of interest were derived in exactly the same was as done by Alkema et al. (2013, Supplementary Appendix, Section 2.5.2).

3.7.6 Aggregate median adjustments

The estimates and projections include adjusted median values derived from the posterior distributions of the Bayesian hierarchical model. To perform the adjustments, the medians of the Bayesian posterior distributions for total contraceptive prevalence, $p_{c,t,1} + p_{c,t,2} = P_{c,t}$, modern contraceptive prevalence, $p_{c,t,2} = P_{c,t}R_{c,t}$, and unmet need for any method of contraception, $p_{c,t,3} = (1 - P_{c,t})Z_{c,t}$, were retained as estimated by the model. Posterior medians of these values were used to compute adjusted posterior medians for the other indicators, such as traditional contraceptive prevalence,

$p_{c,t,1}$, total demand for family planning, $D_{c,t} := p_{c,t,1} + p_{c,t,2} + p_{c,t,3}$, and the ratio of modern contraceptive prevalence to total demand for family planning $M_{c,t} := p_{c,t,2}/D_{c,t}$. The last of these measures serves as sustainable development goal (SDG) indicator 3.7.1.

The mathematical operations performed to obtain the adjusted indicators were as follows:

$$p^*_{c,t,1} = \tilde{P}_{c,t} - \tilde{p}_{c,t,2} \quad (3.132)$$

$$D^*_{c,t} = \tilde{P}_{c,t} + \tilde{p}_{c,t,3} \quad (3.133)$$

$$M^*_{c,t} = \frac{\tilde{p}_{c,t,2}}{D^*_{c,t}} \quad (3.134)$$

$$(3.135)$$

where the notation x^* signifies the adjusted value of variable x and $\tilde{y}$ signifies the posterior median of the variable y. These adjustments ensure that the reported values conform to the identities required by their definitions, namely: $\tilde{P}_{c,t} = p^*_{c,t,1} + \tilde{p}_{c,t,2}$; $D^*_{c,t} = \tilde{P}_{c,t} + \tilde{p}_{c,t,3}$; and $M^*_{c,t} = \tilde{p}_{c,t,2}/D^*_{c,t}$.

The adjustments described here were used to derive adjusted median values only. A similar adjustment was not applied to other percentiles of the posterior distributions, and therefore the identities mentioned above do not hold, in general, for the endpoints of the uncertainty ranges.

3.8 Model validation

Model performance was assessed using a set of cross-validation exercises like those employed by Alkema et al. (2013, Supplementary Appendix, Section 2.6):

Exercise 1 Leave out 20 percent of the observations within each country at random.
Exercise 2 Leave out all data with observation years 2014 or later (20 percent).
Exercise 3 Leave out all unmet need observations for a randomly chosen 20 percent of countries with at least one. Due to a small number of countries remaining in the test set, this exercise was repeated five times and the results averaged.

Exercise 1 assesses general out-of-sample performance, Exercise 2 assesses forecast performance, and Exercise 3 assesses the fit to the unmet need data.

The following measures were used to summarize the results:

1. Median prediction error and median absolute prediction error.
   For example, the error in predicting total prevalence for left out observation i was computed as
   $$e_{i,1+2} = y_{i,1+2} - \hat{y}_{i,1+2},$$
   where $\hat{y}_{i,1+2}$ is the predictive posterior median of $y_{i,1+2}$ (taking into account perturbations and biases).
2. Proportion of the left out observations less than their posterior predictive median. If the model is well calibrated, we expect this to be around 50 percent.
3. Coverage of 95 percent prediction intervals with respect to the left out observations.
   This was defined as the proportion of the left out observations that fell inside the respective posterior predictive intervals. If the model is well calibrated, and if the left out observations are independent from one another, we expect this to be the nominal level (e.g,. for 95 percent intervals, this should be close to 0.95).

Only one left out observation per country was used to calculate the above measures so as to reduce bias due to dependence among observations within country.

4 RESULTS

In this section we present estimates of model parameters other than the main contraceptive prevalence parameter components, such as source variances, bias parameters, and perturbation multipliers. We also summarize the results of the model assessment exercises. Supplementary tables and figures summarizing results for the contraceptive prevalence components are in Sections 5 and Supplementary Appendix II.

4.1 Error variance parameters

The error variance parameters of the data model represent uncertainty due to sampling and non-sampling error in the data. Posterior estimates for the log-ratios of modern to no-use, traditional to no-use, and unmet need, by source type, for the UWRA and MWRA models are in Figure I. Overall, errors for MWRA are lower than those for UWRA, primarily reflecting the greater data availability for the former group.

Within source type, errors are smaller for the modern to no-use log-ratios than they are for the traditional to no-use log-ratios. Among sources, errors for DHS sourced observations are the generally the lowest, particularly for traditional to no-use log-ratios.

4.2 Misclassification biases and perturbation multipliers

Posterior estimates of the misclassification bias parameters for UWRA are in Figure J. Proportions potentially misclassified due to exclusion of sterilization or an absence of probing questions were at 32 percent and 34 percent, respectively (95 percent UIs: (9, 53) and (20, 46)). The estimated proportion misclassified due to inclusion of sterilization was 0.4 percent (95 percent UI: (0.02, 1)) and due to inclusion of folk methods was 3 percent (95 percent UI: (0.2, 9)).

Estimates of the perturbation multipliers are in Figure K. Uncertainty about these parameters was generally high for those about which a direction was not assumed. Most of these were due to surveys sampling age groups different from the nominal 15–49 year old group. Multipliers for the directional age group multipliers, and those for PW, sterilization only (SO), and other positive biases, were estimated with lower variance.

4.3 Model validation

Results of the out-of-sample validations are in Table F. For Exercises 1 and 2 the proportions of left out observations falling inside the 80% and 95% prediction intervals are close to the nominal amounts for all components of contraceptive prevalence. Proportions of left out observations falling below the posterior predictive median are also close to the nominal amounts for Exercise 1. For Exercise 2 they are a little lower than the nominal value but the median errors (MEs) and median absolute errors (MAEs) are small (less than 3.2 percent).

Exercise 3 was repeated five times, and the results averaged, to compensate for the small size of the test set. The estimated coverages of the posterior predictive intervals under Exercise 3 are close to the nominal values and MEs and MAE are small.

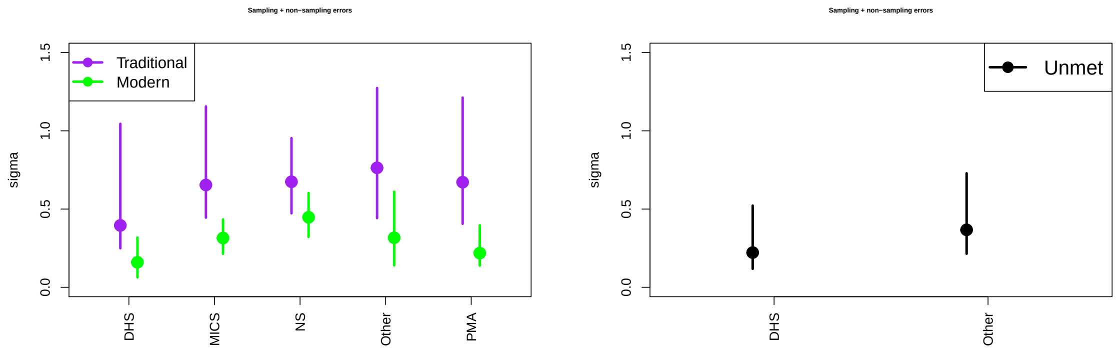

(a) Unmarried and not in a union women of reproductive age (UWRA)

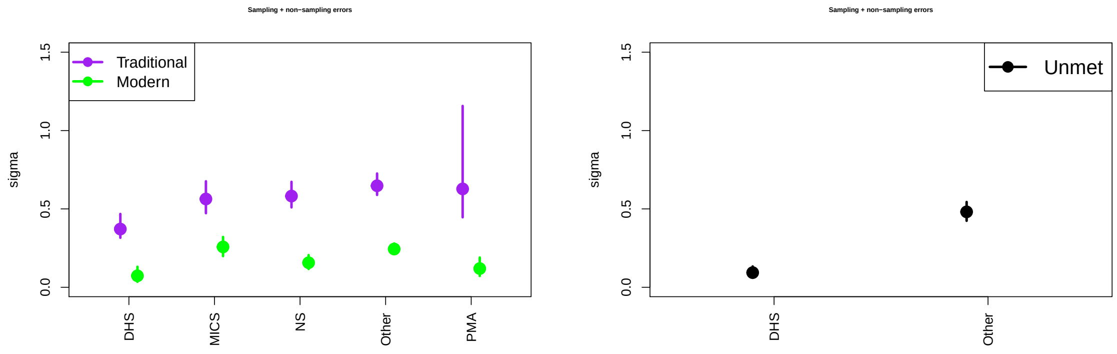

(b) Married or in a union women of reproductive age (MWRA)

Figure I. Posterior estimates of standard deviation parameters of observed contraceptive prevalence log-ratios by source for (a) unmarried and not in a union women of reproductive age (UWRA) and (b) married or in a union women of reproductive age (MWRA). Dots are posterior medians, vertical lines indicate posterior 95% uncertainty intervals. The source types are: Demographic and Health Survey (DHS), Multiple Indicator Cluster Survey (MICS), national surveys (NS), other international survey (Other) and Performance Monitoring and Accountability 2020 survey (PMA).

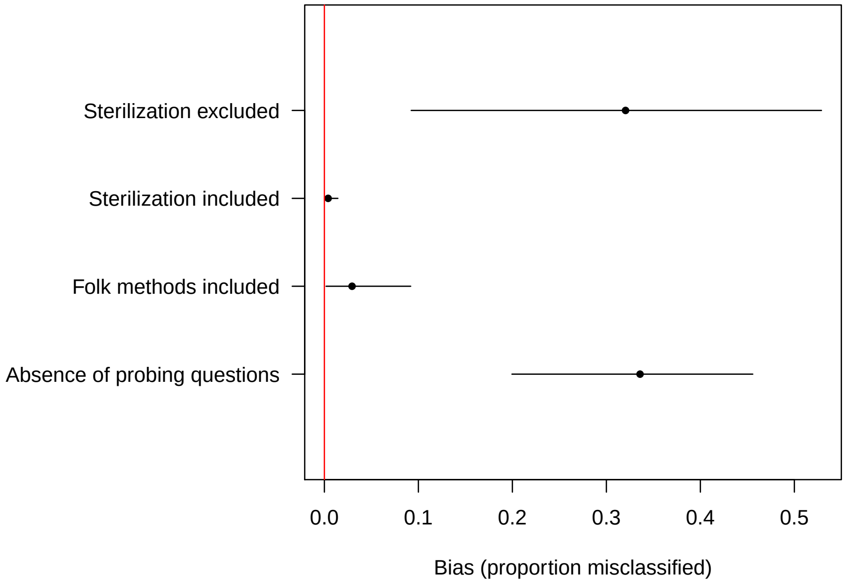

Figure J. Posterior estimates of misclassification bias parameters for unmarried and not in a union women of reproductive age (UWRA). The parameters estimate bias due to exclusion of sterilization from modern method use regardless of the reason of the sterilization, inclusion of sterilization for non-contraceptive reason in modern method use, inclusion of folk methods in traditional method use, and the absence of probing questions about knowledge of specific methods.

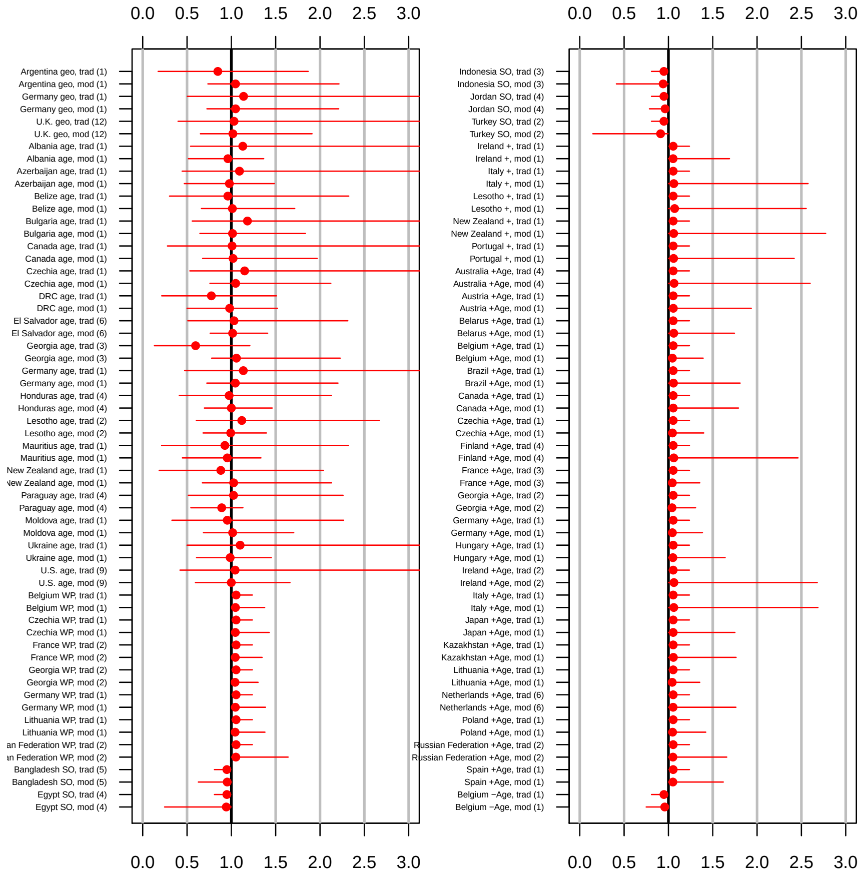

Figure K. Posterior estimates of the perturbation multiplier parameters for unmarried and not in a union women of reproductive age (UWRA).

Table F. Summary of model validation results based on out-of-sample validation experiments. For each exercise and component, the values are the proportion of left out observations that fall outside, or inside, the respective 95% prediction intervals, and below their posterior predictive median estimate, and their median error (ME) and median absolute error (MAE). The '# Obs' column gives the number of observations in the test set in each replication of each exercise. Exercise 3 was repeated five times with different randomly chosen test sets of size '# Obs'.

Component	# Obs	95% prediction interval %Below	%Within	%Above	Median % Below	Errors (%) ME	MAE
			Exercise 1 (leave out 20% of obs.)				
Total	73	2.7	93.2	4.1	45.2	0.4	2.1
Modern	73	2.7	94.5	2.7	47.9	0.1	1.7
Traditional	73	1.4	94.5	4.1	43.8	0.1	0.4
Unmet	34	2.9	91.2	5.9	41.2	0.7	1.4
			Exercise 2 (end)				
Total	64	2.0	97.0	2.0	40.6	0.8	3.1
Modern	64	3.0	97.0	0.0	42.2	1.0	3.0
Traditional	64	2.0	95.0	3.0	42.2	0.1	0.3
Unmet	45	0.0	100.0	0.0	37.8	0.5	1.0
			Exercise 3 (unmet)*				
Unmet	14	2.8	94.4	2.8	45.7	0.6	2.1
			Values Expected				
		2.5	95.0	2.5	50.0		

* Repeated five times; results are averages over all replicates.

5 SUPPLEMENTARY TABLES

These tables contain results for selected years from a systematic and comprehensive set of annual, model-based estimates and projections of key indicators of the practice of family planning in a population. They include the prevalence of the use of modern contraceptive methods and the demand for family planning that is being met by use of modern contraceptive methods alone. The results for the latter for UWRA are shown only for the countries not classified as belonging to the low sexually activity (see Section 2.4).

Estimates based on medians, as well as 95 percent uncertainty intervals, are provided for 185 countries or areas, sub-regions, regions, and the world. The results are based on data available as of February 2019.

Note: The designations employed and the material presented in this publication do not imply the expression of any opinion whatsoever on the part of the Secretariat of the United Nations concerning the legal status of any country, territory or area or of its authorities, or concerning the delimitation of its frontiers or boundaries. The term “country” as used in this publication also refers, as appropriate, to territories or areas. Countries or aggregates listed individually are only those with 90,000 inhabitants or more in 2017; the rest are included in the aggregates but are not listed separately.

5.1 Classification of countries by geographical area

Table G. Classification of countries by geographical area, income and sexual activity groups, and data sources used to determine sexual activity group.
Key. Regions: 'LAC' = Latin America and the Caribbean; 'N. America' = Northern America. Sexual activity group: '0' = Low sexual activity among unmarried and not in a union women of reproductive age; '1' = All other countries. Source data for sexual activity group: 'DHS/MICS' = Demographic and Health Survey or Multiple Indicator Cluster Survey; 'PEW GRL' = Pew Global Religious Landscape Survey (Pew Research Center, 2012); 'PEW GAS' = Pew Global Attitudes Survey Pew Research Center, 2014.

Country or aggregate	Region	Sub-region	World Bank income group[a]	Sexual activity group	Source data for sexual activity group
Afghanistan	Asia	Southern Asia	Low	0	PEW GRL
Albania	Europe	Southern Europe	Upper middle	1	DHS/MICS
Algeria	Africa	Northern Africa	Upper middle	0	PEW GRL
Angola	Africa	Middle Africa	Lower middle	1	PEW GRL
Anguilla[b]	LAC	Caribbean		1	PEW GRL
Antigua and Barbuda	LAC	Caribbean	High	1	PEW GRL
Argentina	LAC	South America	Upper middle	1	PEW GAS
Armenia	Asia	Western Asia	Upper middle	0	DHS/MICS
Australia	Oceania	Australia and New Zealand	High	1	PEW GAS
Austria	Europe	Western Europe	High	1	PEW GRL
Azerbaijan	Asia	Western Asia	Upper middle	0	DHS/MICS
Bahamas	LAC	Caribbean	High	1	PEW GRL
Bahrain	Asia	Western Asia	High	0	PEW GRL
Bangladesh	Asia	Southern Asia	Lower middle	0	PEW GRL
Barbados	LAC	Caribbean	High	1	DHS/MICS
Belarus	Europe	Eastern Europe	Upper middle	1	DHS/MICS
Belgium	Europe	Western Europe	High	1	PEW GRL
Belize	LAC	Central America	Upper middle	1	DHS/MICS
Benin	Africa	Western Africa	Low	1	DHS/MICS
Bhutan	Asia	Southern Asia	Lower middle	0	DHS/MICS
Bolivia, Plurinational State of	LAC	South America	Lower middle	1	DHS/MICS
Bosnia and Herzegovina	Europe	Southern Europe	Upper middle	1	DHS/MICS
Botswana	Africa	Southern Africa	Upper middle	1	DHS/MICS
Brazil	LAC	South America	Upper middle	1	DHS/MICS

continued

Table G. Classification of countries by World Bank income group and sexual activity group, and data sources for sexual activity group (cont'd).

Country or aggregate	Region	Sub-region	World Bank income group[a]	Sexual activity group	Source data for sexual activity group
Bulgaria	Europe	Eastern Europe	Upper middle	1	PEW GRL
Burkina Faso	Africa	Western Africa	Low	1	DHS/MICS
Burundi	Africa	Eastern Africa	Low	1	DHS/MICS
Cabo Verde	Africa	Western Africa	Lower middle	1	PEW GRL
Cambodia	Asia	South-eastern Asia	Lower middle	0	DHS/MICS
Cameroon	Africa	Middle Africa	Lower middle	1	DHS/MICS
Canada	N. America	Northern America	High	1	PEW GAS
Central African Republic	Africa	Middle Africa	Low	1	DHS/MICS
Chad	Africa	Middle Africa	Low	1	DHS/MICS
Chile	LAC	South America	High	1	PEW GAS
China	Asia	Eastern Asia	Upper middle	1	PEW GAS
China, Hong Kong SAR	Asia	Eastern Asia	High	1	World Values Survey
Colombia	LAC	South America	Upper middle	1	DHS/MICS
Comoros	Africa	Eastern Africa	Lower middle	1	DHS/MICS
Congo	Africa	Middle Africa	Lower middle	1	DHS/MICS
Cook Islands[b]	Oceania	Polynesia		1	PEW GRL
Costa Rica	LAC	Central America	Upper middle	1	PEW GRL
Côte d'Ivoire	Africa	Western Africa	Lower middle	1	DHS/MICS
Croatia	Europe	Southern Europe	High	1	PEW GRL
Cuba	LAC	Caribbean	Upper middle	1	PEW GRL
Czechia	Europe	Eastern Europe	High	1	PEW GAS
Democratic People's Rep. of Korea	Asia	Eastern Asia	Low	1	PEW GRL
Democratic Rep. of the Congo	Africa	Middle Africa	Low	1	DHS/MICS
Denmark	Europe	Northern Europe	High	1	PEW GRL
Djibouti	Africa	Eastern Africa	Lower middle	0	PEW GRL
Dominica	LAC	Caribbean	Upper middle	1	PEW GRL
Dominican Republic	LAC	Caribbean	Upper middle	1	DHS/MICS
Ecuador	LAC	South America	Upper middle	1	World Values Survey
Egypt	Africa	Northern Africa	Lower middle	0	PEW GAS
El Salvador	LAC	Central America	Lower middle	1	PEW GAS
Equatorial Guinea	Africa	Middle Africa	Upper middle	1	PEW GRL
Eritrea	Africa	Eastern Africa	Low	1	DHS/MICS

continued

Table G. Classification of countries by World Bank income group and sexual activity group, and data sources for sexual activity group (cont'd).

Country or aggregate	Region	Sub-region	World Bank income group[a]	Sexual activity group	Source data for sexual activity group
Estonia	Europe	Northern Europe	High	1	World Values Survey
Eswatini	Africa	Southern Africa	Lower middle	1	DHS/MICS
Ethiopia	Africa	Eastern Africa	Low	1	DHS/MICS
Fiji	Oceania	Melanesia	Upper middle	1	PEW GRL
Finland	Europe	Northern Europe	High	1	PEW GRL
France	Europe	Western Europe	High	1	PEW GAS
Gabon	Africa	Middle Africa	Upper middle	1	DHS/MICS
Gambia	Africa	Western Africa	Low	1	DHS/MICS
Georgia	Asia	Western Asia	Upper middle	1	World Values Survey
Germany	Europe	Western Europe	High	1	PEW GAS
Ghana	Africa	Western Africa	Lower middle	1	DHS/MICS
Greece	Europe	Southern Europe	High	1	PEW GAS
Grenada	LAC	Caribbean	Upper middle	1	PEW GRL
Guadeloupe[b]	LAC	Caribbean		1	PEW GRL
Guam	Oceania	Micronesia	High	1	PEW GRL
Guatemala	LAC	Central America	Upper middle	1	DHS/MICS
Guinea	Africa	Western Africa	Low	1	DHS/MICS
Guinea-Bissau	Africa	Western Africa	Low	1	DHS/MICS
Guyana	LAC	South America	Upper middle	1	DHS/MICS
Haiti	LAC	Caribbean	Low	1	DHS/MICS
Honduras	LAC	Central America	Lower middle	1	DHS/MICS
Hungary	Europe	Eastern Europe	High	1	PEW GRL
India	Asia	Southern Asia	Lower middle	0	DHS/MICS
Indonesia	Asia	South-eastern Asia	Lower middle	0	DHS/MICS
Iran, Islamic Republic of	Asia	Southern Asia	Upper middle	0	PEW GRL
Iraq	Asia	Western Asia	Upper middle	0	PEW GRL
Ireland	Europe	Northern Europe	High	1	PEW GRL
Israel	Asia	Western Asia	High	1	PEW GAS
Italy	Europe	Southern Europe	High	1	PEW GAS
Jamaica	LAC	Caribbean	Upper middle	1	PEW GRL
Japan	Asia	Eastern Asia	High	1	PEW GAS
Jordan	Asia	Western Asia	Upper middle	0	PEW GAS

continued

Table G. Classification of countries by World Bank income group and sexual activity group, and data sources for sexual activity group (cont'd).

Country or aggregate	Region	Sub-region	World Bank income group[a]	Sexual activity group	Source data for sexual activity group
Kazakhstan	Asia	Central Asia	Upper middle	1	DHS/MICS
Kenya	Africa	Eastern Africa	Lower middle	1	DHS/MICS
Kiribati	Oceania	Micronesia	Lower middle	1	PEW GRL
Kuwait	Asia	Western Asia	High	0	PEW GRL
Kyrgyzstan	Asia	Central Asia	Lower middle	1	DHS/MICS
Lao People's Dem. Republic	Asia	South-eastern Asia	Lower middle	0	DHS/MICS
Latvia	Europe	Northern Europe	High	1	PEW GRL
Lebanon	Asia	Western Asia	Upper middle	0	PEW GAS
Lesotho	Africa	Southern Africa	Lower middle	1	DHS/MICS
Liberia	Africa	Western Africa	Low	1	DHS/MICS
Libya	Africa	Northern Africa	Upper middle	0	World Values Survey
Lithuania	Europe	Northern Europe	High	1	PEW GRL
Madagascar	Africa	Eastern Africa	Low	1	DHS/MICS
Malawi	Africa	Eastern Africa	Low	1	DHS/MICS
Malaysia	Asia	South-eastern Asia	Upper middle	0	PEW GAS
Maldives	Asia	Southern Asia	Upper middle	0	PEW GRL
Mali	Africa	Western Africa	Low	1	DHS/MICS
Malta	Europe	Southern Europe	High	1	PEW GRL
Marshall Islands	Oceania	Micronesia	Upper middle	1	PEW GRL
Martinique[b]	LAC	Caribbean		1	PEW GRL
Mauritania	Africa	Western Africa	Lower middle	0	DHS/MICS
Mauritius	Africa	Eastern Africa	Upper middle	1	PEW GRL
Mexico	LAC	Central America	Upper middle	1	PEW GAS
Mongolia	Asia	Eastern Asia	Lower middle	1	PEW GRL
Montenegro	Europe	Southern Europe	Upper middle	1	DHS/MICS
Montserrat[b]	LAC	Caribbean		1	PEW GRL
Morocco	Africa	Northern Africa	Lower middle	0	DHS/MICS
Mozambique	Africa	Eastern Africa	Low	1	DHS/MICS
Myanmar	Asia	South-eastern Asia	Lower middle	0	PEW GRL
Namibia	Africa	Southern Africa	Upper middle	1	DHS/MICS
Nauru	Oceania	Micronesia	Upper middle	1	PEW GRL
Nepal	Asia	Southern Asia	Low	0	DHS/MICS

continued

Table G. Classification of countries by World Bank income group and sexual activity group, and data sources for sexual activity group (cont'd).

Country or aggregate	Region	Sub-region	World Bank income group[a]	Sexual activity group	Source data for sexual activity group
Netherlands	Europe	Western Europe	High	1	World Values Survey
New Zealand	Oceania	Australia and New Zealand	High	1	World Values Survey
Nicaragua	LAC	Central America	Lower middle	1	PEW GRL
Niger	Africa	Western Africa	Low	0	DHS/MICS
Nigeria	Africa	Western Africa	Lower middle	1	DHS/MICS
Northern Mariana Islands	Oceania	Micronesia	High	1	PEW GRL
Norway	Europe	Northern Europe	High	1	PEW GRL
Oman	Asia	Western Asia	High	0	PEW GRL
Pakistan	Asia	Southern Asia	Lower middle	0	PEW GAS
Palau	Oceania	Micronesia	High	1	PEW GRL
Panama	LAC	Central America	High	1	PEW GRL
Papua New Guinea	Oceania	Melanesia	Lower middle	1	PEW GRL
Paraguay	LAC	South America	Upper middle	1	DHS/MICS
Peru	LAC	South America	Upper middle	1	DHS/MICS
Philippines	Asia	South-eastern Asia	Lower middle	1	DHS/MICS
Poland	Europe	Eastern Europe	High	1	PEW GAS
Portugal	Europe	Southern Europe	High	1	PEW GRL
Puerto Rico	LAC	Caribbean	High	1	PEW GRL
Qatar	Asia	Western Asia	High	0	World Values Survey
Republic of Korea	Asia	Eastern Asia	High	1	PEW GAS
Republic of Moldova	Europe	Eastern Europe	Lower middle	1	DHS/MICS
Réunion[b]	Africa	Eastern Africa		1	PEW GRL
Romania	Europe	Eastern Europe	Upper middle	1	World Values Survey
Russian Federation	Europe	Eastern Europe	Upper middle	1	PEW GAS
Rwanda	Africa	Eastern Africa	Low	1	DHS/MICS
Saint Kitts and Nevis	LAC	Caribbean	High	1	PEW GRL
St. Lucia	LAC	Caribbean	Upper middle	1	DHS/MICS
St. Vincent and the Grenadines	LAC	Caribbean	Upper middle	1	PEW GRL
Samoa	Oceania	Polynesia	Upper middle	1	PEW GRL
Sao Tome and Principe	Africa	Middle Africa	Lower middle	1	DHS/MICS
Saudi Arabia	Asia	Western Asia	High	0	PEW GRL
Senegal	Africa	Western Africa	Lower middle	1	DHS/MICS

continued

Table G. Classification of countries by World Bank income group and sexual activity group, and data sources for sexual activity group (cont'd).

Country or aggregate	Region	Sub-region	World Bank income group[a]	Sexual activity group	Source data for sexual activity group
Serbia	Europe	Southern Europe	Upper middle	1	DHS/MICS
Sierra Leone	Africa	Western Africa	Low	1	DHS/MICS
Singapore	Asia	South-eastern Asia	High	1	World Values Survey
Slovakia	Europe	Eastern Europe	High	1	PEW GRL
Slovenia	Europe	Southern Europe	High	1	World Values Survey
Solomon Islands	Oceania	Melanesia	Lower middle	1	PEW GRL
Somalia	Africa	Eastern Africa	Low	0	PEW GRL
South Africa	Africa	Southern Africa	Upper middle	1	DHS/MICS
South Sudan	Africa	Eastern Africa	Low	0	DHS/MICS
Spain	Europe	Southern Europe	High	1	PEW GAS
Sri Lanka	Asia	Southern Asia	Upper middle	0	PEW GRL
State of Palestine	Asia	Western Asia	Lower middle	0	PEW GAS
Sudan	Africa	Northern Africa	Lower middle	0	PEW GRL
Suriname	LAC	South America	Upper middle	1	DHS/MICS
Sweden	Europe	Northern Europe	High	1	World Values Survey
Switzerland	Europe	Western Europe	High	1	PEW GRL
Syrian Arab Republic	Asia	Western Asia	Low	0	PEW GRL
Tajikistan	Asia	Central Asia	Low	0	DHS/MICS
Thailand	Asia	South-eastern Asia	Upper middle	1	World Values Survey
TFYR Macedonia	Europe	Southern Europe	Upper middle	1	DHS/MICS
Democratic Rep. of Timor-Leste	Asia	South-eastern Asia	Lower middle	0	DHS/MICS
Togo	Africa	Western Africa	Low	1	DHS/MICS
Tonga	Oceania	Polynesia	Upper middle	1	PEW GRL
Trinidad and Tobago	LAC	Caribbean	High	1	DHS/MICS
Tunisia	Africa	Northern Africa	Lower middle	0	PEW GAS
Turkey	Asia	Western Asia	Upper middle	0	PEW GAS
Turkmenistan	Asia	Central Asia	Upper middle	0	DHS/MICS
Tuvalu	Oceania	Polynesia	Upper middle	1	PEW GRL
Uganda	Africa	Eastern Africa	Low	1	DHS/MICS
Ukraine	Europe	Eastern Europe	Lower middle	1	DHS/MICS
United Arab Emirates	Asia	Western Asia	High	0	PEW GRL
United Kingdom	Europe	Northern Europe	High	1	PEW GAS

continued

Table G. Classification of countries by World Bank income group and sexual activity group, and data sources for sexual activity group (cont'd).

Country or aggregate	Region	Sub-region	World Bank income group[a]	Sexual activity group	Source data for sexual activity group
United Rep. of Tanzania	Africa	Eastern Africa	Low	1	DHS/MICS
United States of America	N. America	Northern America	High	1	PEW GAS
United States Virgin Islands	LAC	Caribbean	High	1	PEW GRL
Uruguay	LAC	South America	High	1	World Values Survey
Uzbekistan	Asia	Central Asia	Lower middle	0	DHS/MICS
Vanuatu	Oceania	Melanesia	Lower middle	1	PEW GRL
Venezuela, Bolivarian Republic of	LAC	South America	Upper middle	1	PEW GAS
Viet Nam	Asia	South-eastern Asia	Lower middle	0	DHS/MICS
Yemen	Asia	Western Asia	Low	0	PEW GRL
Zambia	Africa	Eastern Africa	Lower middle	1	DHS/MICS
Zimbabwe	Africa	Eastern Africa	Lower middle	1	DHS/MICS

[a] World Bank (2019). *World Bank Country and Lending Groups (2020 fiscal year)*. URL: https://datahelpdesk.worldbank.org/knowledgebase/articles/906519-world-bank-country-and-lending-groups (visited on 08/11/2019)

[b] World Bank income groups are not available for Anguilla, Cook Islands, Guadeloupe, Martinique, Montserrat, Réunion.

5.2 Unmarried and not in a union women of reproductive age

5.2.1 Modern contraceptive prevalence in 185 countries or areas

Table H. Modern contraceptive prevalence, unmarried and not in a union women of reproductive age (UWRA), 2000, 2019, and 2000–2019 change, in 185 countries or areas.
Key. PPI = posterior probability of an increase; • = observations available; ○ no observations available.

Country or aggregate	Modern contraceptive prevalence 2000	2019	Change 2000–2019	PPI 2000–2019	Prevalence Obs.	Unmet Obs.
World	15.4 (12–23.1)	20.1 (16.3–26.3)	4.5 (0.2–8.7)	98		
More developed regions[a,b]	31.4 (25.8–37.4)	39.3 (32–46.9)	7.8 (1–15.1)	99		
Less developed regions[a,c]	10.4 (6.7–20.1)	15.3 (11.2–22.7)	4.6 (-0.7–9.3)	96		
Least developed countries[a,d]	4.5 (4–5.2)	9.3 (8–10.9)	4.8 (3.5–6.3)	100		
Less dev. regions excl. China[a,c]	8.6 (7.4–10.1)	13.3 (11.6–15.3)	4.7 (2.8–6.8)	100		
Less dev. excl. least dev. [a,e]	11.2 (7–22.2)	16.5 (11.6–25.3)	4.9 (-1–10.5)	96		
High-income countries	33.3 (27.0–40.3)	39.7 (32–47.6)	6.4 (-0.8–13.8)	96		
Upper-middle-income countries	15.8 (9.3–33.5)	25.1 (16.3–41.6)	8.6 (-0.5–18.9)	97		
Lower-middle-income countries	4.4 (3.3–6.2)	7.1 (5.9–9.1)	2.7 (0.7–4.9)	99		
Low-income countries	6.3 (5.1–9)	11.7 (9.8–14.1)	5.3 (3.1–7.4)	100		
Africa	10.2 (9.2–11.3)	15.2 (13.7–16.9)	5 (3.3–6.8)	100		
Eastern Africa	7.7 (6.9–8.5)	14.4 (12.4–16.7)	6.7 (4.6–9.1)	100		
Burundi	1.9 (1.2–3)	3.5 (2.1–5.8)	1.6 (-0.2–4)	96	•	•
Comoros	5.2 (3.3–7.8)	4.8 (2.1–10.5)	-0.3 (-4.1–5.3)	44	•	•
Djibouti	0.9 (0.1–9)	1.3 (0.1–15)	0.3 (-1.4–8.1)	72	○	○
Eritrea	1.4 (0.9–1.9)	1.8 (0.6–4.7)	0.4 (-0.8–3.2)	70	•	•
Ethiopia	1.9 (1.4–2.7)	6.6 (4.4–9.7)	4.7 (2.4–7.9)	100	•	•
Kenya	10.8 (7.9–14.3)	22 (15.7–29.8)	11.1 (4–19.5)	100	•	•
Madagascar	4.6 (2.5–7.5)	17.3 (9.4–28.9)	12.7 (4.4–24.4)	100	•	•
Malawi	9.4 (7.5–11.1)	21.3 (14–30.8)	12 (4.5–21.5)	100	•	•
Mauritius	2 (1.1–3.8)	2.9 (1.3–6.6)	0.9 (-1.3–4.4)	78	•	○
Mozambique	13.5 (9.7–18.1)	20 (11.4–32.4)	6.6 (-3.3–19.5)	89	•	•
Réunion	29.6 (12.3–54)	34.3 (11–67.9)	4.5 (-15.4–29.4)	66	•	○

continued

Table H. Modern contraceptive prevalence, unmarried and not in a union women of reproductive age (UWRA), 2000, 2019, and 2000–2019 change (cont'd).

Country or aggregate	Modern contraceptive prevalence 2000	2019	Change 2000–2019	PPI 2000–2019	Prevalence Obs.	Unmet Obs.
Rwanda	1.4 (1–2)	7.5 (4.4–12.4)	6.1 (2.9–11)	100	•	•
Somalia	0.9 (0.1–8.9)	1.3 (0.1–15.1)	0.3 (-1.5–8.1)	71	◦	◦
South Sudan	1 (0.1–9)	1.4 (0.1–15.1)	0.3 (-1.4–8)	72	◦	◦
Uganda	11.6 (9–14.2)	17.9 (12.6–24.7)	6.4 (0.3–13.4)	98	•	•
United Rep. of Tanzania	12.4 (9.4–16)	19.6 (12.2–29.5)	7.2 (-1.1–17.6)	96	•	•
Zambia	8 (6.1–10.4)	15.7 (9.2–25.3)	7.6 (0.7–17.5)	99	•	•
Zimbabwe	12.6 (9.9–15.9)	19.6 (12.6–29.1)	7 (-0.9–17)	96	•	•
Middle Africa	6.9 (5.1–9.2)	15.8 (11.4–21.3)	8.9 (4–14.7)	100		
Angola	6.2 (3.4–10.8)	14.2 (9–21.7)	7.9 (1.2–16.1)	99	•	•
Cameroon	12.2 (7.2–18.5)	27.9 (15.6–43.4)	15.6 (1.9–31.7)	99	•	•
Central African Republic	5.2 (2.2–10.6)	10.4 (3.8–23.9)	5.1 (-2.6–18.1)	89	•	•
Chad	1.6 (0.9–2.6)	5 (2.8–8.7)	3.4 (0.9–7.2)	100	•	•
Congo	10.7 (3.4–23.7)	28.5 (15.4–44.3)	17.3 (-1–35.8)	97	•	•
Democratic Rep. of the Congo	5.1 (2.6–9.1)	12.4 (5.7–22.4)	7.1 (-0.4–17.5)	97	•	•
Equatorial Guinea	7.9 (2.3–19)	15.7 (7.5–28.8)	7.5 (-5.4–22.3)	88	•	◦
Gabon	17.7 (14.2–21.4)	31.9 (18.1–48.5)	14.1 (-0.1–31)	97	•	•
Sao Tome and Principe	11.9 (5.4–22.7)	22.3 (12.6–36.4)	10.1 (-4–25.7)	92	•	•
Northern Africa	1 (0.3–3.9)	1.5 (0.4–6.6)	0.5 (-0.3–3.6)	90		
Algeria	0.9 (0.1–8.6)	1.4 (0.1–14.5)	0.3 (-1.4–7.9)	72	◦	◦
Egypt	0.2 (0.1–0.5)	0.2 (0.1–0.6)	0 (-0.2–0.3)	55	•	◦
Libya	1 (0.1–9.3)	1.4 (0.1–15.5)	0.3 (-1.4–8.2)	72	◦	◦
Morocco	1 (0.1–9.3)	1.4 (0.1–15.3)	0.3 (-1.5–8.2)	72	◦	◦
Sudan	0.9 (0.1–8.8)	1.4 (0.1–14.5)	0.3 (-1.5–8)	71	◦	◦
Tunisia	0.9 (0.1–8.9)	1.4 (0.1–14.1)	0.3 (-1.5–7.5)	71	◦	◦
Southern Africa	42.7 (36.2–49.4)	45.1 (35.3–55.1)	2.4 (-9.2–14.1)	66		
Botswana	34.1 (17.2–56.9)	40.9 (15.7–73.1)	6.4 (-14.1–31.1)	72	•	◦
Eswatini	21 (12.6–32.9)	41.9 (26.6–59.5)	20.6 (1.7–40.9)	98	•	•
Lesotho	19 (14.1–25)	35.9 (23.9–50.5)	16.9 (3–32.3)	99	•	•

continued

Table H. Modern contraceptive prevalence, unmarried and not in a union women of reproductive age (UWRA), 2000, 2019, and 2000–2019 change (cont'd).

Country or aggregate	Modern contraceptive prevalence 2000	2019	Change 2000–2019	PPI 2000–2019	Prevalence Obs.	Unmet Obs.
Namibia	33.6 (28.6–38.5)	48.2 (33.3–63.7)	14.6 (-0.9–30.9)	97	●	●
South Africa	44.4 (37.1–51.9)	45.4 (34.6–56.6)	1 (-12.1–14.1)	56	●	●
Western Africa	8.1 (6.9–9.5)	15.1 (12.7–17.8)	7 (4.2–10)	100		
Benin	5.6 (3.5–8.6)	14.6 (7.5–25.1)	8.9 (1.3–19.5)	99	●	●
Burkina Faso	10.8 (8.1–13.8)	14.6 (10.3–20.4)	3.9 (-1.4–10.1)	92	●	●
Cabo Verde	23.2 (16.4–32.1)	32.6 (15.5–56.5)	9.3 (-9.2–33.5)	82	●	○
Côte d'Ivoire	14.8 (9.7–20.7)	23.3 (15.6–32.6)	8.5 (-1.2–18.9)	96	●	●
Gambia	4.8 (2.3–9.5)	4.4 (2.2–8.4)	-0.3 (-5.5–4.3)	44	●	●
Ghana	7.3 (5.1–10.1)	16 (10–24)	8.6 (2–16.9)	100	●	●
Guinea	8.2 (5.7–11.3)	13.4 (7.7–21.9)	5.2 (-1.4–14)	93	●	●
Guinea-Bissau	10.3 (6.6–16.6)	35.9 (21.5–53.8)	25.4 (9.9–43.6)	100	●	●
Liberia	13.6 (7.7–21.9)	23.8 (13.9–37.5)	10.1 (-2.6–25.1)	94	●	●
Mali	6.4 (4.5–8.8)	10.5 (5.9–18.2)	4.1 (-1.2–11.9)	93	●	●
Mauritania	0.9 (0.1–8.8)	1.3 (0.1–15.4)	0.3 (-1.4–8.1)	71	○	○
Niger	3.2 (2.2–4.6)	2.5 (1.4–4.3)	-0.7 (-2.4–1.3)	21	●	●
Nigeria	7.2 (5.2–9.8)	14 (10.1–19)	6.8 (2.1–12.2)	100	●	●
Senegal	4 (2.9–5.4)	4.7 (3.1–7.1)	0.8 (-1.4–3.3)	75	●	●
Sierra Leone	11.3 (7.4–16.9)	30.3 (18.6–45.8)	18.8 (5.8–35.1)	100	●	●
Togo	12.8 (7.6–19.4)	17.6 (10.2–28.4)	4.8 (-5–16.6)	82	●	●
Asia	9 (3.5–23.1)	11.6 (5.4–23)	2 (-5.2–9.2)	76		
Central Asia	6.3 (5.1–7.7)	8.5 (5.8–12.3)	2.2 (-0.8–6.1)	92		
Kazakhstan	13.8 (10.4–17.5)	21.5 (13.6–32)	7.8 (-1.1–18.6)	96	●	●
Kyrgyzstan	4.4 (2.9–6.7)	3.8 (1.9–7.6)	-0.6 (-3.6–3.4)	37	●	●
Tajikistan	0.8 (0.4–1.9)	1 (0.3–2.7)	0.1 (-0.9–1.7)	60	●	○
Turkmenistan	2.6 (1.8–3.6)	3.4 (1–10.8)	0.8 (-1.6–8)	69	●	○
Uzbekistan	3.2 (2–5)	3.5 (1.2–9.5)	0.3 (-2.6–6.2)	56	●	○
Eastern Asia	17.1 (4.2–51)	26.2 (7.8–61)	7.2 (-8.3–29)	85		
China	16.1 (1.9–55.6)	24.7 (3.4–67)	6.5 (-12–32.6)	78	○	○

continued

Table H. Modern contraceptive prevalence, unmarried and not in a union women of reproductive age (UWRA), 2000, 2019, and 2000–2019 change (cont'd).

Country or aggregate	Modern contraceptive prevalence 2000	2019	Change 2000–2019	PPI 2000–2019	Prevalence Obs.	Unmet Obs.
China, Hong Kong SAR	15.9 (1.9–56.2)	24.4 (3.3–67.1)	6.5 (-11.6–32.8)	79	○	○
Democratic People's Rep. of Korea	15.8 (1.9–55.6)	24.6 (3.4–67.7)	6.5 (-10.9–32.9)	79	○	○
Japan	22 (6–52.8)	34.5 (15.2–62.3)	11.3 (-11.1–35.5)	85	●	○
Mongolia	12.6 (8.9–17.6)	17.8 (9.1–31.1)	5.1 (-4.8–18.9)	82	●	●
Republic of Korea	15.7 (1.9–56.1)	24.3 (3.3–66.7)	6.5 (-11.4–33)	79	○	○
South-eastern Asia	1.2 (0.8–1.8)	2.4 (1.7–3.7)	1.2 (0.5–2.3)	100		
Cambodia	0.5 (0.2–1)	1 (0.5–2)	0.5 (-0.2–1.5)	94	●	●
Indonesia	0.6 (0.3–1.1)	1.3 (0.8–2.3)	0.8 (0–1.7)	98	●	●
Lao People's Dem. Republic	1.3 (0.3–7.7)	1.9 (0.3–12.4)	0.5 (-1.8–6.7)	73	●	●
Malaysia	1 (0.1–9.5)	1.4 (0.1–15.8)	0.3 (-1.5–8.3)	70	○	○
Myanmar	0.6 (0.1–2.6)	0.8 (0.2–3.1)	0.1 (-1–1.6)	63	●	●
Philippines	1.2 (0.9–1.7)	2.4 (1.4–3.9)	1.2 (0.1–2.8)	98	●	●
Singapore	2.8 (0.3–19)	5.8 (0.5–35.6)	2.4 (-1.9–23.2)	87	○	○
Thailand	2.5 (1.3–4.6)	6.7 (3.5–12.3)	4.1 (0.4–9.9)	99	●	○
Democratic Rep. of Timor-Leste	0.5 (0.1–1.8)	0.6 (0.2–2.3)	0.1 (-0.7–1.3)	64	●	●
Viet Nam	1.2 (0.5–3)	1.5 (0.7–3.2)	0.2 (-1.5–1.9)	64	●	●
Southern Asia	4.1 (2.3–7.5)	5.3 (3.3–8.8)	1.1 (-2.4–4.9)	75		
Afghanistan	1 (0.1–9.2)	1.4 (0.1–14.9)	0.3 (-1.5–7.3)	70	○	○
Bangladesh	1.9 (0.9–3.5)	2.2 (0.7–6.5)	0.3 (-1.4–4.2)	62	●	○
Bhutan	2.4 (0.9–6)	3.6 (1.4–9.3)	1.2 (-2.2–6.3)	77	●	○
India	5.1 (2.6–9.4)	6.5 (3.8–10.8)	1.3 (-3.6–6.5)	72	●	○
Iran, Islamic Republic of	1 (0.1–9.8)	1.4 (0.1–15.6)	0.3 (-1.6–8)	70	○	○
Maldives	1.4 (0.6–3.5)	2 (0.7–5.5)	0.5 (-1.4–3.7)	72	●	○
Nepal	2.1 (1.1–4)	1.6 (1–2.8)	-0.4 (-2.5–1.1)	29	●	●
Pakistan	1 (0.1–9.4)	1.4 (0.1–14.5)	0.3 (-1.6–7.5)	69	○	○
Sri Lanka	0.9 (0.2–4.3)	1.2 (0.2–8.2)	0.3 (-0.9–4.9)	69	●	○
Western Asia	1.2 (0.6–3.3)	2.2 (1–5.6)	0.9 (0.1–3)	98		
Armenia	0.7 (0.3–1.9)	0.9 (0.3–2.8)	0.2 (-0.6–1.8)	68	●	●

continued

Table H. Modern contraceptive prevalence, unmarried and not in a union women of reproductive age (UWRA), 2000, 2019, and 2000–2019 change (cont'd).

Country or aggregate	Modern contraceptive prevalence 2000	2019	Change 2000–2019	PPI 2000–2019	Prevalence Obs.	Unmet Obs.
Azerbaijan	0.5 (0.1–2.2)	0.7 (0.1–4.4)	0.2 (-0.5–2.8)	71	●	○
Bahrain	1 (0.1–9.1)	1.4 (0.1–14.5)	0.3 (-1.5–7.6)	70	○	○
Georgia	0.7 (0.3–1.7)	1.2 (0.4–3.5)	0.5 (-0.6–2.7)	81	●	○
Iraq	1 (0.1–9.1)	1.4 (0.1–14.8)	0.3 (-1.5–7.6)	70	○	○
Israel	2.3 (0.1–21.2)	4.4 (0.3–33.8)	1.5 (-2.5–20.3)	83	○	○
Jordan	0.2 (0.1–0.4)	0.2 (0.1–0.4)	0 (-0.2–0.2)	39	●	○
Kuwait	1.1 (0.1–9.4)	1.4 (0.1–15)	0.3 (-1.5–7.6)	70	○	○
Lebanon	1 (0.1–9.8)	1.4 (0.1–15.7)	0.3 (-1.5–8)	70	○	○
Oman	1 (0.1–8.9)	1.4 (0.1–14.8)	0.3 (-1.5–7.5)	70	○	○
Qatar	1 (0.1–9.5)	1.4 (0.1–15.5)	0.3 (-1.5–7.6)	70	○	○
Saudi Arabia	1 (0.1–9)	1.4 (0.1–14.6)	0.2 (-1.6–7.4)	69	○	○
State of Palestine	1 (0.1–9)	1.4 (0.1–14.9)	0.3 (-1.5–7.6)	69	○	○
Syrian Arab Republic	1 (0.1–10)	1.4 (0.1–15.4)	0.3 (-1.6–7.7)	69	○	○
Turkey	0.5 (0.2–1.6)	1.4 (0.5–3.9)	0.8 (0–3.1)	97	●	○
United Arab Emirates	1 (0.1–9)	1.4 (0.1–14.8)	0.3 (-1.5–7.8)	70	○	○
Yemen	1 (0.1–9.4)	1.4 (0.1–15.3)	0.2 (-1.6–7.9)	69	○	○
Europe	26.8 (22.1–31.9)	35.7 (29–43.1)	8.8 (2.5–16.1)	100		
Eastern Europe	15.1 (10.3–21.9)	25.1 (16.4–37.8)	9.8 (0.5–22.8)	98		
Belarus	22.9 (11.8–38.3)	43.7 (29.1–56.8)	20.2 (0.8–38.4)	98	●	○
Bulgaria	30.9 (13.6–54)	38.6 (14–70.6)	7.4 (-14.8–33.9)	74	●	○
Czechia	22.2 (12.7–34.3)	25.7 (10.5–49.1)	3.5 (-13.3–26.2)	64	●	○
Hungary	14.9 (5.9–33)	21.7 (8.5–44.5)	6.3 (-10.3–27.3)	78	●	○
Poland	14.2 (5.6–30.8)	21.3 (11–36.8)	6.6 (-10.2–23.3)	80	●	○
Republic of Moldova	10.8 (6.5–16.6)	18.7 (8.9–33.9)	7.9 (-3.4–24)	91	●	●
Romania	17.8 (3.1–52.3)	26.3 (5.1–65.3)	6.9 (-10.8–32.1)	78	○	○
Russian Federation	11 (4.4–21.5)	22.7 (8.8–44.7)	11.4 (-3.8–33.6)	92	●	○
Slovakia	17.9 (3.2–53)	26.4 (5–66.2)	7.1 (-11.3–32.4)	79	○	○
Ukraine	19.1 (12.5–28.1)	28.1 (15.1–45.5)	8.8 (-6.9–27.4)	86	●	●

continued

Table H. Modern contraceptive prevalence, unmarried and not in a union women of reproductive age (UWRA), 2000, 2019, and 2000–2019 change (cont'd).

Country or aggregate	Modern contraceptive prevalence 2000	2019	Change 2000–2019	PPI 2000–2019	Prevalence Obs.	Unmet Obs.
Northern Europe	51.3 (38.8–61.9)	53.9 (36–69.3)	2.7 (-13–16.9)	63		
Denmark	37.8 (8.9–79)	41.6 (9.7–83.8)	3.1 (-19.9–28.2)	61	○	○
Estonia	38.1 (8.7–79.4)	41.8 (9.3–84)	3.3 (-19.3–27.7)	62	○	○
Finland	65.3 (43.6–76.8)	68.4 (44.3–82.8)	3.1 (-15.8–20.9)	64	•	○
Ireland	52.7 (28.5–71.2)	57.3 (29.1–79.3)	4.6 (-20.6–28.7)	64	•	○
Latvia	38.3 (9.2–79)	42.2 (9.6–83.4)	3.2 (-19.8–27.7)	62	○	○
Lithuania	12.5 (5.5–26.1)	14.8 (5.3–34.9)	2.1 (-10.9–20)	63	•	○
Norway	37.8 (8.6–79)	41.5 (9.2–83.6)	3.1 (-19.7–27.3)	61	○	○
Sweden	38 (8.7–78.9)	41.6 (9.5–83.9)	3.1 (-19.8–27.9)	61	○	○
United Kingdom	57.1 (40.3–69.6)	58.2 (32.6–79.4)	1.3 (-22.1–22)	55	•	○
Southern Europe	22 (12.9–34.2)	34.9 (21.7–48.9)	12.4 (-0.3–26.4)	97		
Albania	1.2 (0.5–2.8)	1.7 (0.8–3.5)	0.5 (-1.4–2.5)	71	•	•
Bosnia and Herzegovina	5.5 (1.8–12.9)	14.4 (6.2–28.9)	8.6 (-2.1–23.6)	94	•	○
Croatia	11.3 (1.4–41.3)	21.7 (3.7–58.6)	8.6 (-6.9–34.6)	87	○	○
Greece	11.3 (1.4–41.5)	21.8 (3.7–58.7)	8.8 (-7.5–34.5)	86	○	○
Italy	22 (5.6–48.4)	40.4 (17.1–64.5)	16.9 (-7.1–42.4)	92	•	○
Malta	11.4 (1.3–41.5)	22.1 (3.8–59.1)	8.8 (-7.4–34.9)	87	○	○
Montenegro	9 (3.5–19)	21.4 (10.8–37.2)	12 (-1.7–29.2)	96	•	•
Portugal	18.2 (3.6–43.3)	34.6 (15.1–56.3)	15.2 (-7.1–37.8)	91	•	○
Serbia	23.1 (11.1–39)	34.6 (20.3–51.6)	11.4 (-9.4–32.9)	86	•	○
Slovenia	11.3 (1.4–42.6)	21.8 (3.7–58.3)	8.7 (-7.6–34.2)	87	○	○
Spain	27.6 (15–43.5)	35.1 (13.2–65.8)	7.6 (-12.4–33.3)	75	•	○
TFYR Macedonia	10.3 (2.9–25.3)	20.5 (9–38.7)	9.5 (-6.8–28.8)	88	•	○
Western Europe	38.9 (29.5–49.9)	42.1 (30–55.8)	3.1 (-9.5–16.6)	69		
Austria	48.8 (25.5–70.9)	54 (31.9–72.6)	4.7 (-20.1–30.3)	64	•	○
Belgium	37.8 (20–59.4)	45.2 (26.3–65.6)	7.1 (-17.5–32.1)	71	•	○
France	38.7 (24.3–55.4)	44.4 (24.4–66.9)	5.5 (-17–29.5)	68	•	○
Germany	36.2 (21.4–53.8)	34.4 (14.7–60.3)	-1.8 (-23.3–22.7)	44	•	○

continued

Table H. Modern contraceptive prevalence, unmarried and not in a union women of reproductive age (UWRA), 2000, 2019, and 2000–2019 change (cont'd).

Country or aggregate	Modern contraceptive prevalence 2000	2019	Change 2000–2019	PPI 2000–2019	Prevalence Obs.	Unmet Obs.
Netherlands	39.6 (25.6–52.5)	47.2 (27.4–66.8)	7.6 (-13–28.5)	76	•	○
Switzerland	59.1 (32.4–79.8)	65.8 (40.9–83.7)	6.3 (-16.9–30.9)	70	•	○
Latin America and the Caribbean	20 (16.5–24.2)	35.3 (27.8–43.5)	15.2 (7.5–23.7)	100		
Caribbean	21.4 (15.5–28.1)	33.5 (27.2–40.2)	11.9 (3.9–20.2)	100		
Antigua and Barbuda	16.3 (3–47.2)	28.1 (6.4–63.5)	10.1 (-7.6–35.2)	88	○	○
Bahamas	16.4 (3.1–46.6)	28 (6.4–63.1)	10.1 (-7.5–34.5)	87	○	○
Barbados	20.5 (10–36.5)	29.1 (14.8–48.2)	8.3 (-9.2–27.4)	83	•	○
Cuba	41.5 (20.7–65)	60.7 (43.2–75.7)	18.5 (-8.2–44.4)	91	•	•
Dominican Republic	21 (17.3–25.3)	36.7 (24–51)	15.6 (2.2–30.5)	99	•	•
Grenada	16.2 (3.1–47.2)	27.9 (6.2–63.4)	10.1 (-8–35.3)	87	○	○
Guadeloupe	16.3 (3.1–47.2)	28.1 (6.1–63.3)	10 (-7.7–35)	87	○	○
Haiti	5.6 (4.4–7)	13.8 (9.2–20.2)	8.2 (3.4–14.7)	100	•	•
Jamaica	18.7 (13.1–26.3)	26.2 (12.5–46.6)	7.2 (-7.2–27.9)	82	•	○
Martinique	16.3 (3.1–47.2)	28.1 (6.1–63)	10 (-8.2–34.3)	87	○	○
Puerto Rico	16.4 (3.1–47.1)	28 (6.1–63.4)	9.9 (-7.8–34.3)	87	○	○
St. Lucia	18.6 (7.4–37.9)	31.4 (16.5–51.3)	12.1 (-8.7–34)	88	•	○
St. Vincent and the Grenadines	16.2 (3.1–46.7)	27.9 (6–63.1)	9.8 (-8–34.7)	87	○	○
Trinidad and Tobago	8.8 (5.8–13.4)	23.4 (9.9–44.6)	14.4 (1–35.5)	98	•	○
United States Virgin Islands	16.4 (3–47.3)	28 (6–64)	10.1 (-7.5–35.3)	88	○	○
Central America	11.8 (7.7–18.6)	24.7 (16.9–34.9)	12.7 (3–23.4)	99		
Belize	14.9 (7.8–26.7)	24.2 (11.9–42.7)	8.9 (-5.8–27.3)	88	•	○
Costa Rica	23.3 (10.1–44.3)	36.9 (19.8–58.2)	13 (-9.6–36.3)	87	•	○
El Salvador	14.7 (9.8–21.7)	24.4 (13.9–39.3)	9.6 (-2.3–24.9)	94	•	•
Guatemala	4.4 (3.2–6.1)	10.7 (6.4–17.2)	6.2 (1.6–12.8)	100	•	•
Honduras	10.9 (7.3–15.7)	19 (10.4–31.9)	8 (-2–21.3)	94	•	•
Mexico	11.5 (6.1–20.7)	26.9 (16.2–40.9)	15.1 (1.9–29.7)	99	•	•
Nicaragua	13.5 (11–16.5)	9.8 (4.9–18.3)	-3.7 (-9.6–5.1)	18	•	•
Panama	17.9 (7.2–37)	30 (16.6–47.8)	11.5 (-9.1–31.2)	88	•	•

continued

Table H. Modern contraceptive prevalence, unmarried and not in a union women of reproductive age (UWRA), 2000, 2019, and 2000–2019 change (cont'd).

Country or aggregate	Modern contraceptive prevalence 2000	2019	Change 2000–2019	PPI 2000–2019	Prevalence Obs.	Unmet Obs.
South America	22.7 (17.9–28.3)	39.7 (29.3–51.2)	16.8 (6.3–28.7)	100		
Argentina	19.6 (6.4–42.7)	39 (19.2–61.9)	18.3 (-4.4–42.2)	94	●	○
Bolivia, Plurinational State of	4.8 (3.1–6.9)	15.7 (8.9–25.8)	10.8 (3.8–21.1)	100	●	●
Brazil	28.1 (19.8–38)	45.4 (26.8–66.8)	17 (-2.1–39.1)	96	●	●
Chile	20.9 (6.7–44.6)	44.6 (25.5–65)	22.4 (-0.8–45.2)	97	●	○
Colombia	22.2 (18.7–25.3)	41 (29.6–53.5)	18.9 (7.1–31.6)	100	●	●
Ecuador	8.6 (6–12.5)	26.7 (12.9–47)	18 (3.9–38.1)	100	●	●
Guyana	10 (4.6–19.7)	16.2 (8.2–28.8)	6 (-6.1–20)	84	●	●
Paraguay	15.9 (10.2–24.3)	36.5 (24.1–50.8)	20.4 (6.6–35.5)	100	●	●
Peru	8.3 (6.9–9.9)	20.9 (14.7–28.1)	12.5 (6.2–19.9)	100	●	●
Suriname	11.3 (7.4–17.4)	30.1 (15.5–50.1)	18.5 (3–39.3)	99	●	○
Uruguay	13.4 (2.4–39.6)	29.1 (7.7–61.7)	14.2 (-3.6–39.4)	94	○	○
Venezuela, Bolivarian Republic of	15.5 (7.5–28.6)	31.2 (12–59.2)	15 (-2.9–40.5)	94	●	○
Northern America	44.5 (30.2–59.4)	46.8 (30.3–64.2)	2.4 (-14.9–19.5)	61		
Canada	59.3 (39.3–75.2)	59.9 (29.4–84.7)	0.9 (-24.6–22.3)	53	●	○
United States of America	42.9 (27.5–59.4)	45.4 (27.3–64.6)	2.6 (-16.4–21.4)	60	●	○
Oceania	32.1 (17.4–46.5)	36.3 (21.5–49.4)	4.1 (-11.8–20.1)	69		
Australia and New Zealand	39.6 (20.9–57.8)	45.3 (25.9–62.5)	5.6 (-15–26)	70		
Australia	39.7 (18.8–60.2)	45.9 (24.3–65)	5.9 (-18–29.8)	69	●	○
New Zealand	37.9 (13.4–69.9)	40.8 (12.3–77.8)	2.8 (-19.5–27)	60	●	○
Melanesia	3.5 (1.8–7.6)	6.8 (2.8–16.9)	3 (-1–12.7)	92		
Fiji	5.4 (0.6–29.8)	10.2 (1.1–44.8)	3.7 (-4.6–25.6)	83	○	○
Papua New Guinea	2.6 (1.2–5.5)	5.6 (1.7–17.4)	2.9 (-1.3–14.1)	89	●	○
Solomon Islands	4.4 (1.5–11)	7.2 (3.1–15.9)	2.6 (-4.1–11.4)	80	●	○
Vanuatu	7.4 (3.1–15.9)	12.3 (5.7–24.3)	4.6 (-5.1–17.8)	83	●	○
Micronesia	14.8 (6.1–38.1)	18.5 (6.4–43.5)	2.9 (-7–16.9)	72		
Guam	14.1 (1.9–53.8)	20.1 (2.4–63.2)	4.2 (-11.4–28.1)	74	○	○
Kiribati	14.1 (6.9–23.1)	11 (3.7–27.4)	-2.7 (-13.1–11.9)	32	●	○

continued

Table H. Modern contraceptive prevalence, unmarried and not in a union women of reproductive age (UWRA), 2000, 2019, and 2000–2019 change (cont'd).

Country or aggregate	Modern contraceptive prevalence 2000	2019	Change 2000–2019	PPI 2000–2019	Prevalence Obs.	Unmet Obs.
Polynesia	4.2 (2.4–6.7)	5 (2.8–8)	0.8 (-1.9–3.8)	72		
Samoa	1.8 (0.7–4.2)	2.1 (1–4.7)	0.3 (-2.3–3.1)	60	•	•
Tonga	2.4 (0.7–7)	2.9 (1–8.2)	0.5 (-3.2–4.9)	64	•	○

[a] The designation "more developed" and "less developed" regions are intended for statistical purposes and do not express a judgment about the stage reached by a particular country or area in the development process.

[b] More developed regions comprise Europe, Northern America, Australia/New Zealand and Japan.

[c] Less developed regions comprise all regions of Africa, Asia (except Japan), Latin America and the Caribbean plus Melanesia, Micronesia and Polynesia.

[d] The group of least developed countries includes 47 countries: 32 in Sub-Saharan Africa, 2 in Northern Africa and Western Asia, 4 in Central and Southern Asia, 4 in Eastern and South- Eastern Asia, 1 in Latin America and the Caribbean, 4 in Oceania. Further information is available at http://unohrlls.org/about-ldcs/.

[e] Other less developed countries comprise the less developed regions excluding the least developed countries.

5.2.2 Demand for family planning satisfied by modern methods for countries in sexual activity group 1

Table I. Demand for family planning met by modern methods, unmarried and not in a union women of reproductive age (UWRA), 2000, 2019, and 2000–2019 change, in countries in sexual activity group 1 and country aggregates with at least one country in sexual activity group 1. Country aggregates with at least one country in sexual activity group 1 are based on all countries in the aggregate.
Key. PPI = posterior probability of an increase; • = observations available; ○ no observations available.

Country or aggregate	Met demand (modern) 2000	2019	Change 2000–2019	PPI 2000–2019	Prevalence Obs.	Unmet Obs.
World	68.6 (56.2–78.6)	75.4 (66.1–82.6)	6.1 (-0.4–16.2)	97		
More developed regions[a,b]	76.1 (65.3–83.5)	81.9 (71.5–88.5)	5.6 (-1.4–13.4)	95		
Less developed regions[a,c]	63.2 (45.5–78.9)	72 (59.8–82)	7.8 (-2.1–22.8)	94		
Least developed countries[a,d]	39.2 (34.9–43.9)	55.9 (50.9–60.7)	16.6 (10.4–22.6)	100		
Less dev. regions excl. China[a,c]	64.8 (54.3–69.7)	72.3 (62.8–77)	7.6 (2.6–13)	100		
Less dev. excl. least dev. [a,e]	65.3 (46–81.4)	74.5 (60.4–85.1)	7.8 (-2.2–24.2)	94		
High-income countries	76.5 (65–84.6)	81.9 (70.9–88.8)	5.1 (-2–13.3)	93		
Upper-middle-income countries	67.9 (45.5–84.4)	78 (62.7–89.3)	8.7 (-2.1–27.4)	94		
Lower-middle-income countries	57.4 (36–67.9)	65.5 (44.9–73.8)	8 (-1.1–17.4)	96		
Low-income countries	40.6 (34.2–49.8)	56.8 (50.9–62.6)	16.1 (7.7–23.5)	100		
Africa	52.8 (48.7–56.6)	62.7 (58.8–66.4)	10 (4.9–15)	100		
Eastern Africa	46.8 (43.1–50.6)	63 (58.4–67.5)	16.2 (10.1–22.1)	100		
Burundi	31.7 (19.4–46.3)	48.3 (33.4–62.8)	16.4 (-2.6–34.4)	95	•	•
Comoros	51 (37.9–62.9)	53.8 (32.5–71.4)	3.3 (-20.4–23.5)	61	•	•
Eritrea	49.3 (36.6–61.4)	59.6 (33.5–79.9)	10.6 (-18.5–34.6)	77	•	•
Ethiopia	35.8 (26.9–46.2)	67.7 (56.4–77)	31.5 (17.5–44.9)	100	•	•
Kenya	49 (38–59)	69.3 (59.6–77.8)	20.2 (6.5–33.9)	100	•	•
Madagascar	22.7 (13–35.1)	53.3 (37.5–68.4)	30.4 (10.8–48.6)	100	•	•
Malawi	48.4 (40.8–55.6)	66.8 (55.8–76.8)	18.4 (5.5–31)	100	•	•
Mauritius	37.2 (16.1–62.9)	41.5 (19.1–66.2)	4.3 (-19.1–26.4)	64	•	○
Mozambique	48.3 (38.4–58.1)	58.5 (45–71.6)	10 (-6.5–26.7)	89	•	•
Réunion	72.3 (48.1–89.6)	76.9 (50–95.3)	3.6 (-14.8–24)	66	•	○
Rwanda	24.6 (18.4–32.1)	54.7 (41–67.4)	30 (14.9–44.1)	100	•	•

continued

Table I. Demand for family planning met by modern methods, unmarried and not in a union women of reproductive age (UWRA), 2000, 2019, and 2000–2019 change, in countries in sexual activity group 1 (cont'd).

Country or aggregate	Met demand (modern) 2000	2019	Change 2000–2019	PPI 2000–2019	Prevalence Obs.	Unmet Data
Uganda	52.4 (44.4–59.8)	61.7 (51.6–70.9)	9.4 (-2.8–21.5)	94	●	●
United Rep. of Tanzania	47.8 (38.7–56.7)	60.7 (46.8–72.5)	12.8 (-3.2–27.6)	94	●	●
Zambia	42.3 (33.4–51.4)	57.4 (44.8–69.8)	15 (0.3–30.3)	98	●	●
Zimbabwe	71.5 (63.9–78)	77.7 (67.9–85.3)	6.1 (-5.1–16.7)	87	●	●
Middle Africa	24.8 (18–32.8)	48.1 (37.5–57.9)	23.4 (10.5–35.2)	100		
Angola	29.2 (15.8–46.1)	47.5 (35.5–60.6)	18 (-0.5–35.4)	97	●	●
Cameroon	33.6 (20.3–49.2)	67.2 (45.1–82.4)	33.6 (7.2–54.2)	99	●	●
Central African Republic	25.4 (11.3–43.6)	44.7 (23.2–66)	19.2 (-6.7–43.2)	93	●	●
Chad	24 (14–36.2)	46.6 (31.3–61.2)	22.4 (2.3–40.8)	98	●	●
Congo	23.4 (7.5–48.6)	58 (35.1–76.7)	33.8 (-0.5–62)	97	●	●
Democratic Rep. of the Congo	18.5 (9.5–32.2)	38.5 (20.3–57)	20 (-2.3–40.9)	96	●	●
Equatorial Guinea	37.7 (12.1–65.1)	57.6 (35.1–77.5)	18.4 (-9.9–50.1)	90	●	○
Gabon	35.3 (27.9–43.4)	62.4 (41.8–79.3)	27 (5.9–45.9)	99	●	●
Sao Tome and Principe	51.1 (29.5–68.5)	62.4 (47.3–75.9)	10.4 (-10.9–35.7)	83	●	●
Southern Africa	82.1 (74.7–88)	83 (73.6–90.6)	0.8 (-10.2–11.3)	56		
Botswana	79.4 (57.1–93.3)	83.8 (58.2–97.6)	3.3 (-12.4–21.3)	67	●	○
Eswatini	68.9 (55.6–79.9)	83.3 (69.5–93.8)	13.8 (-2.4–31.5)	95	●	●
Lesotho	70.2 (59–79.3)	82.2 (71.5–91)	11.6 (-1.2–25.9)	96	●	●
Namibia	75.8 (69.6–81.1)	85.8 (72.8–95)	9.7 (-3.5–21.1)	93	●	●
South Africa	82.8 (74.7–89.1)	82.8 (72.2–91.3)	0 (-12.1–11.5)	50	●	●
Western Africa	40.9 (35.3–46.5)	58.1 (52–63.6)	17.1 (9–25)	100		
Benin	23.7 (15–34.8)	48.4 (29.9–64.5)	25 (3.1–43.3)	99	●	●
Burkina Faso	59 (48.8–67.4)	62.8 (52–72.6)	3.8 (-9.9–17.3)	71	●	●
Cabo Verde	64.9 (45.9–80.9)	73.6 (50.8–90.9)	7.2 (-11.2–28.5)	79	●	○
Côte d'Ivoire	37.1 (24.7–49.6)	56.3 (43.3–68.1)	19.2 (1.8–36)	98	●	●
Gambia	53.6 (32.7–71.3)	60.5 (40.9–75.6)	6.6 (-16.9–30.3)	71	●	●
Ghana	36.7 (26.2–47.5)	53.2 (40–65)	16.5 (-0.3–32.6)	97	●	●
Guinea	38 (27.3–48.6)	51.8 (36.7–65.6)	13.9 (-4.1–30.9)	94	●	●

continued

Table I. Demand for family planning met by modern methods, unmarried and not in a union women of reproductive age (UWRA), 2000, 2019, and 2000–2019 change, in countries in sexual activity group 1 (cont'd).

Country or aggregate	Met demand (modern) 2000	2019	Change 2000–2019	PPI 2000–2019	Prevalence Obs.	Unmet Data
Guinea-Bissau	40.4 (25.1–57.8)	69.5 (51.2–85.4)	28.1 (6.4–50.2)	99	•	•
Liberia	38.9 (26–53)	50.8 (37.3–65.4)	11.5 (-6.4–30.7)	90	•	•
Mali	32.6 (23.6–42.2)	50.9 (37–64.7)	18.1 (2.2–34.4)	99	•	•
Nigeria	44 (32.3–55.3)	60.7 (48.9–70.7)	16.6 (0.6–32.4)	98	•	•
Senegal	52.3 (41.4–62.6)	64.4 (52.7–74.3)	12.1 (-3.3–26.3)	94	•	•
Sierra Leone	46.4 (33.2–60.1)	65.7 (51.2–79.7)	18.6 (0.6–38.1)	98	•	•
Togo	35.2 (21.5–50.3)	60 (45.5–72.8)	24.7 (4.8–43.2)	99	•	•
Asia	62.2 (33.5–84.8)	71 (47.7–88.4)	6.9 (-7.5–30)	82		
Central Asia	73.5 (65.7–79.5)	78.6 (69.1–85.5)	4.9 (-4.7–14)	85		
Kazakhstan	73.4 (63.9–80.8)	79.7 (68.5–87.6)	6 (-6.1–17.6)	84	•	•
Kyrgyzstan	69.5 (51.5–82.4)	67.5 (47.6–81.7)	-1.7 (-22.3–17.6)	43	•	•
Eastern Asia	62.3 (26.7–87.5)	72.9 (43.3–92.1)	7.7 (-8.8–36.2)	82		
China	62.1 (18.6–89.9)	72.7 (33.6–94.3)	6.9 (-14.1–40.5)	75	○	○
China, Hong Kong SAR	62.5 (19.2–90)	72.5 (33–94.6)	7.2 (-13.9–39.8)	76	○	○
Democratic People's Rep. of Korea	61.7 (19.3–90.2)	72.7 (33.5–94.6)	7.2 (-13.6–39.9)	76	○	○
Japan	64 (28.4–88.6)	75.8 (47.2–93)	9.1 (-12.3–42.2)	80	•	○
Mongolia	57.1 (39.1–72.8)	63.1 (43.3–79.2)	5.9 (-11.9–22.5)	75	•	•
Republic of Korea	62 (18.8–90.2)	72.7 (33.8–94.5)	6.9 (-14–41.1)	76	○	○
South-eastern Asia	63 (47.5–73.8)	68.5 (56.4–77.7)	5.6 (-5.7–17.4)	84		
Philippines	53.8 (41.8–64.4)	48.7 (32.6–63.3)	-4.8 (-24.2–13.9)	31	•	•
Singapore	61 (16.5–87.9)	71 (23–91.2)	7.5 (-14.7–34.8)	77	○	○
Thailand	64.3 (31.9–86.4)	77.3 (49.5–91.6)	11.8 (-5.7–33.8)	91	•	○
Western Asia	60.5 (38.2–78.7)	71.6 (52.6–84.7)	10.7 (-1–24.2)	96		
Georgia	27 (5–65.5)	37.9 (7.8–78.1)	9.5 (-14–38)	80	•	○
Israel	43.2 (4.1–85.3)	57.1 (8.4–89.5)	8.4 (-14.4–41.2)	78	○	○
Europe	75.1 (65.2–82.2)	81.9 (72.8–88.1)	6.6 (0.2–13.9)	98		
Eastern Europe	69.4 (53–80.9)	80.6 (66.7–89.1)	10.5 (-2.1–26.6)	95		
Belarus	72.9 (48.3–88.4)	86.4 (71.1–93.9)	11.8 (-5.1–37.5)	91	•	○

continued

Table I. Demand for family planning met by modern methods, unmarried and not in a union women of reproductive age (UWRA), 2000, 2019, and 2000–2019 change, in countries in sexual activity group 1 (cont'd).

Country or aggregate	Met demand (modern) 2000	2019	Change 2000–2019	PPI 2000–2019	Prevalence Obs.	Unmet Data
Bulgaria	69.5 (39.4–89.9)	81.1 (51.7–96.1)	10.2 (-14–38.2)	82	•	○
Czechia	75.7 (54.2–88.8)	82 (59.7–93.5)	5.9 (-12.5–25.6)	76	•	○
Hungary	74.8 (47.6–90.1)	82 (60–93.2)	6.1 (-12.5–31.3)	76	•	○
Poland	76.4 (50.3–91.2)	83.2 (63.1–93.3)	5.2 (-10.2–28.1)	76	•	○
Republic of Moldova	63 (44–77.3)	73.5 (53.3–86.6)	10.4 (-12.4–33.1)	83	•	•
Romania	70.7 (30.9–92.8)	81.2 (49.5–95.9)	6.8 (-12.4–42)	78	○	○
Russian Federation	63.4 (33.6–83.6)	79.1 (54.3–92.3)	13.5 (-10.2–45)	88	•	○
Slovakia	70.7 (30.7–92.9)	81.2 (50.3–96)	6.7 (-11.8–41.5)	78	○	○
Ukraine	74.3 (56.5–86.5)	83.2 (65.2–92.8)	8.3 (-11.6–28.3)	82	•	•
Northern Europe	87.6 (70.8–94.2)	88.5 (69.2–95.7)	1.1 (-11–10.5)	60		
Denmark	82.3 (44.2–97.9)	85.3 (47.6–98.5)	1.7 (-16.1–23.2)	61	○	○
Estonia	82.8 (44.3–97.8)	85.7 (47.5–98.5)	1.6 (-16–23)	61	○	○
Finland	91.6 (74–96.7)	92.2 (73.9–97.7)	1 (-11.7–12.6)	58	•	○
Ireland	91.6 (65.1–98.4)	93.5 (66.9–98.9)	1.2 (-13.3–17.7)	62	•	○
Latvia	82.8 (43.8–97.8)	85.8 (47.1–98.4)	1.7 (-16–24)	61	○	○
Lithuania	66.2 (32.8–87.8)	69.1 (34.8–89.5)	2.4 (-18.1–24)	60	•	○
Norway	82.6 (43.7–97.9)	85.5 (47.9–98.3)	1.7 (-16.1–23.7)	60	○	○
Sweden	82.9 (43.8–97.9)	85.4 (47.6–98.4)	1.7 (-16.1–22.9)	61	○	○
United Kingdom	89.1 (70–96.2)	89.6 (64.9–97.7)	0.7 (-17.1–13.5)	55	•	○
Southern Europe	62.5 (43.3–78.5)	76.4 (58.8–87.8)	12.6 (-2.2–31.3)	95		
Albania	22.7 (9.1–43.6)	20.6 (9.8–36.8)	-2.1 (-25.9–19.4)	42	•	•
Bosnia and Herzegovina	41 (15.1–68.2)	60.9 (35.1–81.8)	19 (-13.1–51.8)	89	•	○
Croatia	50.6 (12.2–84.1)	68 (32.2–91.5)	13.4 (-11.8–50.3)	85	○	○
Greece	50.6 (11.5–83.5)	68.1 (32–91.5)	13.5 (-12.7–50.5)	85	○	○
Italy	58.8 (22.2–85.1)	78.7 (49.7–93.8)	16.9 (-9.3–53.5)	90	•	○
Malta	50.6 (11.9–83.9)	68.6 (32.4–91.6)	13.5 (-12.1–50.5)	85	○	○
Montenegro	44.2 (19.6–67.9)	64.9 (44.1–81.1)	18.9 (-8.1–49.9)	92	•	•
Portugal	53.7 (13.5–85.2)	72.5 (37.9–92.7)	15.5 (-12–53.1)	88	•	○

continued

Table I. Demand for family planning met by modern methods, unmarried and not in a union women of reproductive age (UWRA), 2000, 2019, and 2000–2019 change, in countries in sexual activity group 1 (cont'd).

Country or aggregate	Met demand (modern) 2000	Met demand (modern) 2019	Change 2000–2019	PPI 2000–2019	Prevalence Obs.	Unmet Data
Serbia	60.2 (33.3–80.5)	72.5 (48.8–88.2)	11.6 (-16.7–41)	80	●	○
Slovenia	49.7 (12.3–83.5)	67.8 (31.9–91.3)	13.6 (-12.6–49.4)	85	○	○
Spain	73.7 (51.6–88.3)	79.8 (52.3–95.2)	5.4 (-13.9–23.8)	74	●	○
TFYR Macedonia	47.4 (17.2–75.6)	65.3 (38–84.4)	15.6 (-14.8–51.4)	84	●	○
Western Europe	82.7 (61.1–93.4)	85.1 (64.1–94.6)	2.1 (-6.6–12.6)	70		
Austria	87.4 (59–97.1)	90.7 (66.3–97.6)	2.6 (-12.7–23.8)	65	●	○
Belgium	84.6 (54.9–96.5)	88.2 (60.6–97.7)	3 (-12.2–22.6)	68	●	○
France	83.7 (56.2–95.3)	86.5 (58.2–96.8)	2 (-13.9–20.8)	62	●	○
Germany	82.3 (54.4–94.9)	82.7 (51.5–95.9)	-0.1 (-18–17.9)	50	●	○
Netherlands	81.4 (55.1–94.3)	86.1 (58.3–96.9)	4 (-12.7–22.9)	72	●	○
Switzerland	90.2 (64.9–98)	93.6 (73.9–98.6)	2.5 (-10.3–22.7)	68	●	○
Latin America and the Caribbean	74.6 (68.7–79.6)	82.8 (76.1–88.3)	8 (1.6–15)	99		
Caribbean	71.2 (59.7–80.7)	77.5 (70.1–83.4)	6.5 (-3.9–17.7)	89		
Antigua and Barbuda	66.2 (34.3–87.3)	74.4 (43.2–93.8)	6.7 (-12.4–29.4)	77	○	○
Bahamas	66.3 (33.8–87.4)	74.1 (42.8–93.6)	6.7 (-12.7–29.1)	77	○	○
Barbados	66 (42.7–83.6)	69.5 (46.2–86.3)	2.9 (-16.6–23.7)	62	●	○
Cuba	77.4 (54.5–93.2)	90 (75.5–96.9)	12.3 (-7.1–35.8)	89	●	●
Dominican Republic	79.9 (74–84.7)	82.8 (71.6–90.9)	2.7 (-8.9–12.8)	69	●	●
Grenada	66.1 (34.1–87.6)	74.2 (43–93.4)	6.7 (-12.5–29.5)	76	○	○
Guadeloupe	65.9 (34.1–87.8)	74.2 (42.6–93.9)	6.8 (-12.3–29.6)	77	○	○
Haiti	43.6 (35.5–51.7)	53.5 (42.2–64.5)	10 (-3.5–22.9)	93	●	●
Jamaica	69.3 (46.5–85.6)	72.5 (49.3–88.5)	2.4 (-14.2–21.4)	62	●	○
Martinique	66.6 (33.9–87.7)	74.4 (42.8–93.2)	6.6 (-12.2–28.7)	76	○	○
Puerto Rico	66.2 (34.4–87.6)	74.2 (43.4–93.4)	6.6 (-12.4–28.9)	77	○	○
St. Lucia	66.5 (41.8–85)	74 (51.5–89.5)	6.8 (-12.4–28.9)	76	●	○
St. Vincent and the Grenadines	66 (33.3–87.5)	74.1 (42.4–93.4)	6.6 (-12.4–29.2)	76	○	○
Trinidad and Tobago	50.4 (30.6–68.6)	63.4 (37.8–82.9)	12.7 (-10.8–33.7)	87	●	○
United States Virgin Islands	66.2 (33.6–87.5)	74.2 (42.6–93.8)	6.7 (-12.6–29.5)	77	○	○

continued

Table I. Demand for family planning met by modern methods, unmarried and not in a union women of reproductive age (UWRA), 2000, 2019, and 2000–2019 change, in countries in sexual activity group 1 (cont'd).

Country or aggregate	Met demand (modern) 2000	Met demand (modern) 2019	Change 2000–2019	PPI 2000–2019	Prevalence Obs.	Unmet Data
Central America	69.4 (55.3–80.1)	75.5 (63.7–84.7)	5.6 (-5.8–19)	84		
Belize	71.1 (48.5–85.9)	74.8 (52.9–88.4)	3.3 (-13.7–22.3)	66	●	○
Costa Rica	78.2 (56.7–91.3)	84.2 (64.2–95.1)	5.2 (-9.4–23.5)	77	●	○
El Salvador	73 (56.7–84.8)	77 (61.5–87.5)	3.6 (-10.1–18.1)	71	●	●
Guatemala	63.6 (52–73.2)	71.1 (58.3–80.7)	7.4 (-7.5–21.6)	85	●	●
Honduras	69.6 (58.4–78.7)	74.3 (60.8–84.2)	4.6 (-10.2–18.7)	75	●	●
Mexico	68.7 (49.9–82.7)	75.5 (60.4–86.7)	6.3 (-8.7–23.8)	80	●	●
Nicaragua	76.6 (69.8–82.2)	75.1 (58.6–85.8)	-1.4 (-18.5–10.4)	41	●	●
Panama	69.2 (48.6–83.9)	74.7 (56.9–87.5)	4.9 (-13.2–24.8)	71	●	●
South America	76.2 (69.4–81.9)	85.5 (77.3–91.7)	9 (1.2–17.2)	99		
Argentina	76.7 (52.4–90.6)	86.6 (66.4–96.4)	8.5 (-5.9–30)	88	●	○
Bolivia, Plurinational State of	40.3 (27.7–52.7)	60.8 (43.2–75)	20.6 (-0.6–39.4)	97	●	●
Brazil	80.4 (70.4–87.7)	89 (74.8–97.1)	8.2 (-4.6–19.9)	90	●	●
Chile	71 (42.2–88.7)	85.9 (67.2–95.6)	12.9 (-4.5–40.9)	92	●	○
Colombia	73.4 (67.2–78.6)	87 (78.2–93)	13.7 (3.7–22.2)	99	●	●
Ecuador	63.2 (47.5–76.3)	75.1 (55.9–88.4)	11.7 (-7.9–29.4)	89	●	●
Guyana	59.8 (38.4–76.1)	65.2 (45.9–79.4)	5.4 (-20.1–30.6)	68	●	●
Paraguay	67.1 (52–78.8)	80.5 (67.4–89.9)	12.5 (-3.2–30)	94	●	●
Peru	56.8 (49.2–63.8)	71.3 (57.1–81.5)	14.7 (-1.1–27.3)	97	●	●
Suriname	75.4 (53.8–88.4)	83 (64–93.6)	6.8 (-6.2–24)	85	●	○
Uruguay	65.8 (29–87.5)	78.5 (48.7–94.4)	10.4 (-9.1–40.1)	86	○	○
Venezuela, Bolivarian Republic of	71.2 (49.8–85.6)	80.2 (56–94)	8.2 (-10.1–27.1)	84	●	○
Northern America	81.9 (58–94)	85 (61.5–95.5)	2.5 (-11.7–18.8)	66		
Canada	91.9 (70.6–98.3)	92.3 (65.8–98.9)	0.2 (-16.8–13.2)	52	●	○
United States of America	80.8 (55.1–94.1)	84.2 (58.9–95.6)	2.8 (-13.4–20.9)	66	●	○
Oceania	83.2 (56.7–94.3)	85.5 (61.1–95.2)	2.1 (-10.4–17.4)	65		
Australia and New Zealand	86 (57.8–96.8)	88.7 (62.2–97.6)	2.2 (-10.3–18.1)	68		
Australia	86.3 (55.9–97.4)	89.1 (60.6–98.2)	2.3 (-12.2–20.5)	66	●	○

continued

Table I. Demand for family planning met by modern methods, unmarried and not in a union women of reproductive age (UWRA), 2000, 2019, and 2000–2019 change, in countries in sexual activity group 1 (cont'd).

Country or aggregate	Met demand (modern) 2000	2019	Change 2000–2019	PPI 2000–2019	Prevalence Obs.	Unmet Data
New Zealand	85.4 (52.4–97.7)	87.4 (52.5–98.5)	1 (-14.2–17.9)	58	•	○
Melanesia	40.7 (14.9–67.9)	54.9 (22.4–80.6)	12.7 (-7.5–35.1)	90		
Fiji	48.5 (9–84.9)	62.7 (17.9–90)	9.8 (-13–42.3)	81	○	○
Papua New Guinea	36.3 (10.9–64.9)	52.3 (17.4–81.6)	14.6 (-11.9–41.3)	87	•	○
Solomon Islands	44.2 (13.2–75.2)	56.6 (22–81.9)	10.6 (-15.5–42.5)	79	•	○
Vanuatu	58.3 (22.4–85.4)	65.8 (29.7–88.5)	6.3 (-14.4–31.1)	73	•	○
Micronesia	70.6 (39.6–90.2)	74.9 (44–92.5)	3 (-8.9–19.9)	71		
Guam	70.1 (26.2–93)	76.3 (33.5–95.2)	3.8 (-14.7–30.1)	68	○	○
Kiribati	73 (38.4–92)	72.1 (35.1–92.1)	-0.8 (-19.1–16.4)	45	•	○
Polynesia	54.5 (32.2–72.6)	60.3 (39–77.3)	5.2 (-10.6–23.8)	74		
Samoa	39.4 (17–64.1)	44 (22.1–66.8)	4.7 (-23.4–32.9)	63	•	•
Tonga	48.5 (17.7–77.8)	53.5 (22.1–80.7)	4.4 (-21.8–32)	63	•	○

[a] The designation "more developed" and "less developed" regions are intended for statistical purposes and do not express a judgment about the stage reached by a particular country or area in the development process.

[b] More developed regions comprise Europe, Northern America, Australia/New Zealand and Japan.

[c] Less developed regions comprise all regions of Africa, Asia (except Japan), Latin America and the Caribbean plus Melanesia, Micronesia and Polynesia.

[d] The group of least developed countries includes 47 countries: 32 in Sub-Saharan Africa, 2 in Northern Africa and Western Asia, 4 in Central and Southern Asia, 4 in Eastern and South- Eastern Asia, 1 in Latin America and the Caribbean, 4 in Oceania. Further information is available at http://unohrlls.org/about-ldcs/.

[e] Other less developed countries comprise the less developed regions excluding the least developed countries.

5.3 Married or in a union women of reproductive age

5.3.1 Modern contraceptive prevalence in 185 countries or areas

Table J. Modern contraceptive prevalence, married or in a union women of reproductive age (MWRA), 2000, 2019, and 2000–2019 change, in 185 countries or areas.
Key. PPI = posterior probability of an increase; • = observations available; ○ no observations available.

Country or aggregate	Modern contraceptive prevalence 2000	2019	Change 2000–2019	PPI 2000–2019	Prevalence Obs.	Unmet Obs.
World	55 (53.7–56.3)	57.1 (54.6–59.5)	2.1 (-0.7–4.8)	93		
More developed regions[a,b]	56.5 (52.9–59.7)	62 (57.5–66.1)	5.5 (0.9–10)	99		
Less developed regions[a,c]	54.7 (53.3–56.1)	56.5 (53.7–59.1)	1.7 (-1.3–4.7)	87		
Least developed countries[a,d]	20.7 (19.8–21.6)	36 (33.2–38.6)	15.2 (12.2–18.1)	100		
Less dev. regions excl. China[a,c]	41.8 (40.5–43.2)	48.4 (45.4–51.6)	6.6 (3.2–10)	100		
Less dev. excl. least dev. [a,e]	59.6 (58–61.1)	60.4 (57.1–63.5)	0.8 (-2.8–4.3)	67		
High-income countries	59.9 (56.1–63.2)	62.4 (58.1–66.3)	2.6 (-1.6–6.7)	89		
Upper-middle-income countries	73.5 (71.2–75.5)	73.9 (69.8–77.1)	0.4 (-3.9–4.3)	57		
Lower-middle-income countries	40.5 (38.5–42.5)	47.9 (43.4–52.5)	7.4 (2.3–12.4)	100		
Low-income countries	15.1 (14.4–15.9)	30.5 (28.7–32.5)	15.4 (13.4–17.5)	100		
Africa	20.5 (19.8–21.2)	33 (31.3–34.8)	12.5 (10.6–14.5)	100		
Eastern Africa	16.5 (15.7–17.4)	40.2 (37.6–43)	23.7 (20.9–26.7)	100		
Burundi	8.2 (6–10.9)	26.2 (19.4–34.2)	17.9 (10.7–26.3)	100	•	•
Comoros	15.5 (11.6–20.1)	20.1 (11.5–32.3)	4.6 (-5.4–17.2)	80	•	•
Djibouti	6.2 (3.9–9.9)	25.4 (13.8–41.8)	19.1 (6.8–35.8)	100	•	○
Eritrea	6.3 (4.9–8.1)	12 (5.7–23.6)	5.7 (-0.9–17.3)	95	•	•
Ethiopia	6.3 (5.5–7.4)	37.6 (31.9–43.8)	31.2 (25.5–37.6)	100	•	•
Kenya	32 (27.3–36.9)	60.8 (53.3–68)	28.8 (19.8–37.4)	100	•	•
Madagascar	13.2 (10.5–16.3)	41.9 (32.5–51.5)	28.6 (18.8–38.8)	100	•	•
Malawi	25.4 (23.1–27.3)	61.3 (51.4–70.3)	36 (25.8–45.4)	100	•	•
Mauritius	47.4 (37–56.8)	41 (27–55.4)	-6.2 (-22.4–10.4)	23	•	•
Mozambique	11.9 (9.1–15.3)	24.8 (15.8–36.4)	12.8 (3.2–24.8)	100	•	•
Réunion	66.5 (54.6–76.9)	70.4 (47.5–87.7)	4 (-16.1–20.3)	66	•	○

continued

Table J. Modern contraceptive prevalence, married or in a union women of reproductive age (MWRA), 2000, 2019, and 2000–2019 change (cont'd).

Country or aggregate	Modern contraceptive prevalence 2000	2019	Change 2000–2019	PPI 2000–2019	Prevalence Obs.	Unmet Obs.
Rwanda	6.1 (5.2–7.3)	51 (40.1–61.6)	44.8 (34–55.4)	100	●	●
Somalia	0.4 (0–1.2)	8.7 (1.5–26.5)	8.2 (1.2–26.1)	100	●	○
South Sudan	3.3 (1.6–6.5)	5.9 (2.9–11.3)	2.5 (-1.7–8.1)	88	●	●
Uganda	16.8 (14.5–19.2)	37.4 (32.1–42.9)	20.6 (14.9–26.5)	100	●	●
United Rep. of Tanzania	18.7 (15.6–22.2)	36.6 (26.5–47.4)	17.9 (7.3–29.2)	100	●	●
Zambia	21.3 (17.8–25.1)	50.4 (38–62.5)	29.1 (16.1–41.8)	100	●	●
Zimbabwe	51.8 (47.3–56.3)	67.5 (57.1–76.3)	15.6 (4.6–25.5)	100	●	●
Middle Africa	6.6 (5.4–8.2)	13.7 (10.5–17.9)	7.1 (3.5–11.5)	100		
Angola	5.1 (3.5–7.5)	14.3 (9.6–20.7)	9.1 (4–15.8)	100	●	●
Cameroon	9.3 (7–12.1)	23 (13.7–35.4)	13.7 (3.9–26.2)	100	●	●
Central African Republic	8.7 (6.2–11.9)	18.2 (8.6–33.6)	9.4 (-0.7–24.8)	96	●	●
Chad	2.4 (1.7–3.4)	6.2 (3.9–10)	3.8 (1.2–7.7)	100	●	●
Congo	9.7 (4.8–16.9)	24.7 (14.7–37.5)	14.7 (2.4–29.2)	99	●	●
Democratic Rep. of the Congo	6.3 (4.1–9.4)	10.4 (5.6–18)	4.1 (-1.6–11.9)	91	●	●
Equatorial Guinea	5.6 (3.7–8.4)	13.5 (7.1–24.1)	7.8 (0.7–18.8)	99	●	●
Gabon	13.5 (11.6–15.6)	24.9 (14.7–38.2)	11.4 (0.9–25)	98	●	●
Sao Tome and Principe	24.6 (17.7–32.7)	41.8 (27.7–57.2)	17.1 (0.9–34.3)	98	●	●
Northern Africa	44.3 (42.2–46.4)	49.9 (43.3–56.1)	5.7 (-1.2–12.1)	95		
Algeria	50.2 (41.8–58.5)	55.9 (40.1–70.6)	5.7 (-11.6–22.2)	74	●	●
Egypt	53.7 (51.5–55.9)	58.6 (46.5–69.7)	5 (-7.3–16.2)	79	●	●
Libya	24.1 (17.1–32.2)	20.5 (12.1–31.4)	-3.6 (-15.2–9.3)	28	●	●
Morocco	50.6 (43.8–57.4)	60.1 (52–67.7)	9.4 (-0.9–19.8)	96	●	●
Sudan	6.2 (4.1–9.1)	13.8 (7.5–23.9)	7.6 (0.8–17.8)	99	●	●
Tunisia	53.6 (46.9–60.2)	57.6 (41.2–71.9)	4 (-13.2–19.3)	68	●	●
Southern Africa	54.7 (49.6–59.6)	56.9 (48.8–64.5)	2.2 (-7.3–11.3)	68		
Botswana	44.3 (35.4–53.6)	59.8 (39.4–77.2)	15.3 (-5–33.5)	93	●	●
Eswatini	31.3 (24.8–38.9)	64.8 (49.9–77)	33.3 (16.7–47.4)	100	●	●
Lesotho	29.4 (24.7–34.8)	63.4 (51.2–73.9)	33.8 (20.8–45.8)	100	●	●

continued

Table J. Modern contraceptive prevalence, married or in a union women of reproductive age (MWRA), 2000, 2019, and 2000–2019 change (cont'd).

Country or aggregate	Modern contraceptive prevalence 2000	2019	Change 2000–2019	PPI 2000–2019	Prevalence Obs.	Unmet Obs.
Namibia	42.5 (38.9–46.1)	59.1 (45.6–71.4)	16.6 (2.8–29.5)	99	●	●
South Africa	57.5 (51.6–63)	56.1 (46.6–64.9)	-1.4 (-12.3–9.3)	40	●	●
Western Africa	8.3 (7.5–9.2)	20 (17.8–22.5)	11.7 (9.3–14.4)	100		
Benin	6.2 (4.8–7.8)	13.3 (10.1–17.3)	7.2 (3.5–11.3)	100	●	●
Burkina Faso	6 (4.8–7.6)	29.5 (23.3–36.4)	23.4 (17.2–30.5)	100	●	●
Cabo Verde	47.9 (39.7–56.2)	63.3 (43.3–79.9)	15.3 (-6.1–34)	92	●	●
Côte d'Ivoire	7.8 (5.7–10.4)	19.9 (15.8–24.6)	12.1 (7.2–17.2)	100	●	●
Gambia	11.5 (9–14.6)	10 (5.7–17)	-1.4 (-6.8–6.1)	33	●	●
Ghana	14.9 (12.4–17.7)	28.7 (22.6–35.4)	13.8 (7.1–20.9)	100	●	●
Guinea	4.3 (3.5–5.4)	7.8 (4.6–12.8)	3.4 (0.1–8.5)	98	●	●
Guinea-Bissau	5.3 (3.5–8)	17.2 (9.5–28.7)	11.7 (3.7–23.5)	100	●	●
Liberia	8.7 (5.3–13.9)	30.5 (20.9–42.1)	21.6 (10.8–33.8)	100	●	●
Mali	6.5 (5.4–7.8)	14.8 (9–23.6)	8.3 (2.3–17.2)	100	●	●
Mauritania	5 (4–6.4)	14 (8–23.2)	8.9 (2.8–18.2)	100	●	●
Niger	6.7 (5.2–8.6)	16.1 (11.9–21.4)	9.4 (4.7–14.9)	100	●	●
Nigeria	8.4 (6.9–10.1)	19.3 (15.5–23.6)	10.8 (6.6–15.5)	100	●	●
Senegal	8.7 (7–10.8)	26.9 (20.8–34.2)	18.2 (11.8–25.6)	100	●	●
Sierra Leone	4.4 (3.1–6.2)	21.6 (14.7–30.7)	17.2 (9.9–26.4)	100	●	●
Togo	9.7 (7.5–12.4)	21.1 (13.3–31.2)	11.3 (3.1–21.7)	100	●	●
Asia	59.8 (58–61.4)	60.4 (56.8–63.9)	0.6 (-3.4–4.6)	62		
Central Asia	53.9 (51–56.7)	54.1 (43.9–62.4)	0.3 (-10.2–9)	52		
Kazakhstan	55.4 (50.2–60.3)	51.9 (43.3–59.8)	-3.4 (-13.1–5.8)	23	●	●
Kyrgyzstan	47.7 (40.2–55.5)	38.9 (26.1–52.9)	-8.7 (-23.8–7)	14	●	●
Tajikistan	29.8 (22.4–38.3)	28.5 (22.5–35.3)	-1.3 (-11.7–8.9)	40	●	●
Turkmenistan	54 (50.4–57.2)	51.1 (36.8–64.7)	-2.8 (-17.3–11.3)	35	●	●
Uzbekistan	60 (54.9–64.8)	65.1 (45–81.1)	5.1 (-15.3–21.7)	70	●	●
Eastern Asia	82.2 (79–84.9)	80.8 (74.8–85.4)	-1.4 (-7.9–4.2)	31		
China	85.1 (81.5–88)	83 (76.6–88.1)	-2 (-9.1–4.1)	26	●	●

continued

Table J. Modern contraceptive prevalence, married or in a union women of reproductive age (MWRA), 2000, 2019, and 2000–2019 change (cont'd).

Country or aggregate	Modern contraceptive prevalence 2000	2019	Change 2000–2019	PPI 2000–2019	Prevalence Obs.	Unmet Obs.
China, Hong Kong SAR	74.9 (55–83.8)	71.3 (51.3–84.3)	-2.8 (-18.6–11.9)	35	●	○
Democratic People's Rep. of Korea	57 (49.3–64.1)	72.8 (61.3–81.8)	15.7 (2.7–27.4)	99	●	●
Japan	48.8 (42.6–54.9)	44.1 (31.7–56.3)	-4.7 (-18.6–8.9)	25	●	○
Mongolia	52.4 (46.1–58.4)	53.9 (39.8–67.2)	1.5 (-13.7–16)	58	●	●
Republic of Korea	69.2 (55.7–77.9)	71.2 (54.1–82.2)	2 (-12.6–15)	62	●	○
South-eastern Asia	51.1 (48.7–53.4)	57.6 (53.2–61.8)	6.5 (1.6–11.3)	100		
Cambodia	18.5 (16.8–20.3)	44.1 (32.8–55.7)	25.7 (14.3–37.3)	100	●	●
Indonesia	55.6 (51.1–59.9)	59.7 (51.1–67.5)	4 (-5.4–13.3)	80	●	●
Lao People's Dem. Republic	27.8 (23.4–32.5)	51 (39.8–62.2)	23.1 (11–34.9)	100	●	●
Malaysia	32.7 (21.6–44.8)	39.2 (27–52)	6.5 (-9.5–22.6)	78	●	○
Myanmar	29.4 (24.6–34.8)	54.3 (44.9–63.2)	24.8 (14.2–34.9)	100	●	●
Philippines	31.9 (28.2–35.6)	41.2 (33.9–48.4)	9.3 (1.1–17.4)	99	●	●
Singapore	55.2 (42.3–66.7)	60.8 (39.7–79.8)	5.8 (-13.8–23.7)	72	●	○
Thailand	73.8 (67.9–78.9)	77.7 (67.9–85.4)	3.9 (-7.1–13.7)	77	●	●
Democratic Rep. of Timor-Leste	14.4 (11.3–17.8)	26.8 (19.7–35.1)	12.4 (4.6–21.4)	100	●	●
Viet Nam	59.2 (54.2–63.8)	65.7 (54.6–75.1)	6.5 (-5.3–17)	86	●	●
Southern Asia	41.7 (38.9–44.6)	48.4 (41.6–55.2)	6.7 (-0.6–14)	96		
Afghanistan	6.8 (4.3–10.6)	23.7 (16.7–32.3)	16.8 (8.9–25.9)	100	●	●
Bangladesh	44.2 (41–47.3)	57.1 (45.6–67.3)	12.8 (1–23.5)	98	●	●
Bhutan	31.8 (23.7–41.4)	63.3 (45.4–78.7)	31.3 (12–48.4)	100	●	●
India	43.5 (39.8–47.4)	49.7 (40.4–59)	6.2 (-3.9–16.2)	89	●	●
Iran, Islamic Republic of	56.1 (49–62.2)	62.1 (45.4–76.2)	6.2 (-11.1–21)	77	●	●
Maldives	31.8 (26.3–37.5)	36.6 (21.9–52.7)	4.8 (-11–21.9)	72	●	●
Nepal	34 (30.1–38.1)	46.9 (38.6–55.3)	12.9 (3.6–22.1)	100	●	●
Pakistan	19.3 (16.2–22.7)	26.6 (21.5–32.4)	7.3 (1.1–13.9)	99	●	●
Sri Lanka	50.2 (42.6–57.2)	54.3 (42.7–64.7)	4.1 (-9–16.3)	74	●	●
Western Asia	31.5 (28.2–34.7)	39.3 (33.7–44.7)	7.9 (1.7–13.8)	99		
Armenia	24.2 (20.5–27.9)	30.1 (21.2–39.8)	5.9 (-3.5–16.2)	88	●	●

continued

Table J. Modern contraceptive prevalence, married or in a union women of reproductive age (MWRA), 2000, 2019, and 2000–2019 change (cont'd).

Country or aggregate	Modern contraceptive prevalence 2000	2019	Change 2000–2019	PPI 2000–2019	Prevalence Obs.	Unmet Obs.
Azerbaijan	16 (11.5–21.4)	20.8 (10.7–34.8)	4.8 (-6.3–19.2)	79	●	●
Bahrain	36.1 (22–52.3)	45.1 (22.5–68.6)	8.8 (-11.6–29.6)	79	●	○
Georgia	19.8 (14.1–26.8)	35.5 (20.8–51.7)	15.6 (0.3–32.2)	98	●	●
Iraq	26.4 (19.9–33.7)	38.5 (28.3–49.2)	12.1 (-0.1–24.3)	97	●	●
Israel	51.7 (32.2–69.9)	55.8 (31–78.3)	4.1 (-16.7–23.2)	66	●	○
Jordan	39.8 (34.1–45.3)	37.5 (31.1–44)	-2.2 (-10.6–6.3)	30	●	●
Kuwait	40.6 (29.7–50.6)	49.7 (27.5–70.8)	9.2 (-11.4–29)	81	●	○
Lebanon	39.7 (32.1–47.5)	47 (30.7–63.7)	7.2 (-10.5–25.3)	78	●	○
Oman	21.8 (15.4–29.5)	23 (13.4–35.5)	1.3 (-10.7–15)	58	●	●
Qatar	33.2 (23.4–44.4)	39.7 (23.4–56.7)	6.2 (-11.2–24.6)	75	●	●
Saudi Arabia	21 (13.4–31.4)	23 (13.9–35.2)	2 (-7.9–12.8)	66	●	○
State of Palestine	35 (27.3–42.6)	46.5 (32.6–60.4)	11.5 (-4.2–27.2)	92	●	●
Syrian Arab Republic	34.7 (27.5–42.5)	44.8 (28.5–61.7)	9.9 (-7.7–28)	86	●	●
Turkey	39.8 (32.4–46.7)	49.2 (34.8–62.6)	9.4 (-6.2–24.2)	88	●	●
United Arab Emirates	27.4 (15.2–43.2)	40.6 (17.2–64.8)	12.6 (-7.2–33.6)	89	●	○
Yemen	12.1 (8.9–16.1)	30 (19.3–42.1)	17.8 (6.7–30.4)	100	●	●
Europe	53.8 (50.6–56.8)	62.1 (57.2–66.6)	8.4 (3–13.5)	100		
Eastern Europe	46.2 (40.7–51.1)	56.7 (47.7–64.8)	10.5 (0.6–19.9)	98		
Belarus	48.5 (37.1–59.3)	58.5 (47.1–68.7)	9.9 (-4.8–24.5)	91	●	●
Bulgaria	38 (28.2–47.9)	53.7 (33.4–71.8)	15.7 (-6.2–35.6)	92	●	●
Czechia	58.7 (47.8–68.3)	74.4 (56.3–86.4)	15.5 (-3.4–30.9)	95	●	●
Hungary	61.9 (47.2–73.2)	60.9 (39.7–78)	-0.8 (-21.7–17.9)	47	●	●
Poland	38.8 (24.8–53.4)	50.6 (33.6–65.8)	11.4 (-7.7–30)	88	●	○
Republic of Moldova	44.3 (35.3–52.7)	50.1 (33.5–66)	5.8 (-12.3–23.8)	73	●	●
Romania	31.7 (22–42.8)	57.3 (35–76)	25.3 (2.6–45.6)	99	●	●
Russian Federation	49 (39.5–57.3)	57.7 (41.3–72.2)	8.8 (-9.6–25.8)	83	●	●
Slovakia	56.4 (36.9–72.2)	65.8 (38.4–85.8)	9.4 (-13.2–28.5)	80	●	○
Ukraine	44.5 (33.4–54.6)	55.2 (38.5–70.1)	10.8 (-8.6–29.6)	86	●	●

continued

Table J. Modern contraceptive prevalence, married or in a union women of reproductive age (MWRA), 2000, 2019, and 2000–2019 change (cont'd).

Country or aggregate	Modern contraceptive prevalence 2000	2019	Change 2000–2019	PPI 2000–2019	Prevalence Obs.	Unmet Obs.
Northern Europe	68.1 (61.4–73.3)	71.5 (59.8–80.3)	3.6 (-7.1–11.8)	76		
Denmark	70.4 (52.1–83.8)	72.1 (44.6–89.6)	1.9 (-19.9–17.6)	58	●	○
Estonia	50.8 (38.8–61.9)	58 (36.1–76.4)	7.1 (-15.2–26.9)	74	●	○
Finland	69.4 (52.5–77.1)	76.8 (60–85.7)	7.8 (-2.9–18)	92	●	○
Ireland	64.1 (52.9–71.1)	66.5 (47.6–80.7)	2.7 (-15.7–18.1)	62	●	○
Latvia	55.5 (39.9–69.4)	60.6 (34.2–81.7)	5.1 (-17.1–24.2)	68	●	●
Lithuania	41.1 (29.4–52.6)	52.5 (31.9–71.7)	11.4 (-10.1–32)	85	●	●
Norway	76 (60.4–83.1)	79.3 (58.5–90.3)	3.8 (-12.9–16)	70	●	○
Sweden	62.7 (45.8–75.1)	65.5 (38.6–84.5)	3 (-19.4–20.3)	61	●	○
United Kingdom	71 (61.1–78.4)	73.6 (55.9–86.3)	2.8 (-13.4–15.1)	64	●	○
Southern Europe	45.9 (39.8–51.7)	54.2 (46.2–61.4)	8.3 (-0.3–16.5)	97		
Albania	13.9 (9.3–19.9)	4.5 (3–6.8)	-9.3 (-15.5—4.4)	0	●	●
Bosnia and Herzegovina	13.9 (9.3–20)	20.7 (10.1–36.1)	6.6 (-5.2–22.4)	85	●	●
Croatia	36.8 (12.5–67.9)	52.8 (20.7–82)	14.4 (-9.2–37.7)	89	●	○
Greece	35.2 (24.1–45.9)	50.5 (26.8–72.8)	15.3 (-7.4–37.3)	90	●	○
Italy	39.9 (25.8–53.3)	51.2 (34.2–66.2)	11.3 (-7.4–29.5)	88	●	●
Malta	57 (38.4–73.4)	61.7 (31.3–84.5)	4.6 (-20–25)	65	●	○
Montenegro	26.5 (19.9–34.2)	21.4 (11.5–35.5)	-5 (-17.6–10)	24	●	●
Portugal	65.6 (44.3–76.9)	68.6 (48.3–82.2)	3.5 (-13.6–20.2)	66	●	○
Serbia	25.8 (18.9–33.9)	29.4 (17.3–44.1)	3.5 (-10.6–19.4)	68	●	●
Slovenia	60.8 (42.1–75.6)	68.6 (42–87.4)	7.8 (-14.6–26.4)	77	●	●
Spain	65.1 (58.6–71)	68.8 (55.3–79.7)	3.7 (-10.9–16.3)	70	●	●
TFYR Macedonia	12.6 (5.2–26.4)	21.3 (10.2–37.8)	8.4 (-6.5–25)	87	●	●
Western Europe	66.8 (61–71.9)	73.3 (64.8–79.8)	6.4 (-3.1–14.8)	92		
Austria	54.7 (42.9–65.7)	62.2 (45.1–76.1)	7.4 (-11.4–25)	78	●	○
Belgium	60.4 (52–68.9)	68.3 (53.4–80.5)	7.8 (-8.1–21.9)	84	●	●
France	72.3 (65.3–78.1)	75 (59.7–85.6)	2.7 (-13.5–15.5)	64	●	●
Germany	63.7 (51.7–73.9)	75.7 (59.4–86.9)	11.9 (-6.1–27.7)	91	●	○

continued

Table J. Modern contraceptive prevalence, married or in a union women of reproductive age (MWRA), 2000, 2019, and 2000–2019 change (cont'd).

Country or aggregate	Modern contraceptive prevalence 2000	2019	Change 2000–2019	PPI 2000–2019	Prevalence Obs.	Unmet Obs.
Netherlands	71.1 (59.8–78.4)	70 (52.7–82.5)	-0.9 (-17.3–13.2)	46	●	○
Switzerland	71.2 (59.1–80.3)	69.8 (55.3–81.4)	-1.1 (-17.8–14.7)	45	●	○
Latin America and the Caribbean	63.8 (60.9–66.4)	69.3 (64.5–73.4)	5.6 (0.1–10.4)	98		
Caribbean	56.8 (53.3–59.8)	58.3 (53–63)	1.5 (-4.5–7.2)	69		
Antigua and Barbuda	53.6 (30–73.9)	60.8 (31.9–83.5)	7 (-15.2–27.5)	74	●	○
Bahamas	60.3 (36.7–79.2)	65.3 (35.6–86.8)	5.3 (-17.8–24.8)	69	●	○
Barbados	55.1 (39–70)	59.9 (42.5–75.1)	4.7 (-15.7–24.6)	68	●	●
Cuba	70 (60.9–77.3)	73.5 (59.5–83.8)	3.5 (-11.4–16.9)	69	●	●
Dominican Republic	64.4 (60.7–67.9)	69.4 (57.1–79.4)	5 (-7.5–15.6)	79	●	●
Grenada	49.3 (29.1–69.1)	58.1 (29.6–80.7)	8.4 (-13.5–28.2)	77	●	○
Guadeloupe	42.6 (19.2–66.8)	52.9 (21.8–78.5)	9.5 (-12–30.4)	81	●	○
Haiti	22.4 (20–24.7)	33.4 (26.5–40.8)	11 (3.7–18.9)	100	●	●
Jamaica	62.3 (54.2–69.6)	62.6 (44.3–78)	0.3 (-19.1–17.6)	51	●	●
Martinique	46.4 (22.8–69.8)	55.4 (24.7–80.5)	8.3 (-13.1–29.3)	77	●	○
Puerto Rico	69.7 (60–77.1)	72.5 (51.1–86.7)	2.8 (-17.3–17.4)	62	●	●
St. Lucia	51.1 (34.4–66.9)	56.5 (38.5–72.7)	5.4 (-15.6–26.3)	70	●	●
St. Vincent and the Grenadines	57.5 (33.9–76.6)	63.1 (33.8–84.9)	5.7 (-16.1–25.9)	70	●	○
Trinidad and Tobago	37.4 (29.5–46.3)	43.6 (26.5–60.6)	6.1 (-12.1–24.5)	74	●	●
United States Virgin Islands	65.3 (45.7–78.5)	69 (45–86)	4 (-17–21.8)	65	●	○
Central America	60.8 (55.7–65.5)	66.4 (57.9–73.5)	5.5 (-4–14.4)	88		
Belize	45.1 (34.3–55.8)	52.4 (39.3–65)	7.3 (-9.4–23.5)	81	●	●
Costa Rica	71.3 (63.8–77.3)	74.8 (63–83.5)	3.6 (-9.1–14.4)	72	●	●
El Salvador	59.1 (49.2–68)	68.3 (54.9–79.3)	9.2 (-5.9–23.3)	89	●	●
Guatemala	32.6 (27.6–38)	52.2 (41.3–62.6)	19.5 (7.6–31.2)	100	●	●
Honduras	49.9 (41.1–58.7)	66.3 (53.3–77.3)	16.3 (0.9–30.5)	98	●	●
Mexico	64.2 (57.5–70.3)	67.7 (56.4–77.1)	3.3 (-9.2–15)	70	●	●
Nicaragua	62.9 (59.3–66.2)	78.8 (67.6–86.9)	15.9 (4.3–24.8)	100	●	●
Panama	53 (38.2–66.9)	58.8 (44–72.5)	5.8 (-13.5–25.1)	72	●	●

continued

Table J. Modern contraceptive prevalence, married or in a union women of reproductive age (MWRA), 2000, 2019, and 2000–2019 change (cont'd).

Country or aggregate	Modern contraceptive prevalence 2000	2019	Change 2000–2019	PPI 2000–2019	Prevalence Obs.	Unmet Obs.
South America	65.8 (61.8–69.3)	71.9 (65.3–77)	6 (-1.2–12.4)	95		
Argentina	64.3 (51.9–71.9)	66.4 (51.7–78.6)	2.4 (-14–18.3)	62	●	○
Bolivia, Plurinational State of	29.2 (24.3–34.2)	45.9 (34.3–57.9)	16.8 (4.3–29.6)	100	●	●
Brazil	72.7 (65.5–78.7)	76.6 (64.7–85.4)	3.9 (-9.4–15.1)	73	●	●
Chile	56.1 (42.7–68.4)	70.9 (60.4–79.6)	14.6 (-0.9–30)	97	●	○
Colombia	65.2 (61.3–68.4)	76 (68.3–82.3)	10.8 (2.5–18.2)	99	●	●
Ecuador	55 (47.5–62.3)	71.7 (56.3–83.2)	16.7 (0.1–30.1)	98	●	●
Guyana	34.9 (28–42.7)	42.4 (28.3–57.4)	7.3 (-8.3–24.1)	81	●	●
Paraguay	51.6 (42.1–60.4)	65.7 (53.5–76.1)	14 (-0.1–27.3)	97	●	●
Peru	48.3 (43.5–52.4)	55.2 (46.5–62.9)	7 (-2.6–15.8)	92	●	●
Suriname	43.2 (34.3–52.5)	54.3 (35.7–71.2)	10.9 (-9–29.9)	86	●	●
Uruguay	74.7 (63.9–83.2)	76.7 (63.4–86.2)	2 (-12.3–15.2)	62	●	○
Venezuela, Bolivarian Republic of	63 (54.4–70.6)	70.1 (52.6–83.3)	7 (-10.1–21.2)	80	●	●
Northern America	65 (54.3–74.1)	67.2 (55.6–76.8)	2.2 (-8.6–12.5)	66		
Canada	74.1 (67–79.9)	77.1 (58.9–88.8)	3 (-15.2–15.6)	64	●	○
United States of America	63.9 (52–74.1)	66.2 (53.5–76.7)	2.2 (-9.7–13.6)	65	●	●
Oceania	54.9 (48.5–60.4)	53.9 (45.2–62.4)	-0.8 (-10.6–9.1)	43		
Australia and New Zealand	65.5 (57.1–72.7)	64.4 (53.1–74)	-1.1 (-13.6–10.8)	43		
Australia	65 (55.1–73.1)	63.6 (50.8–74)	-1.3 (-15.7–12.4)	43	●	○
New Zealand	68.8 (52.6–80.2)	69.8 (42.2–87.5)	1.1 (-21.3–17.3)	55	●	○
Melanesia	23.8 (18.3–30.2)	31.3 (18.2–48.2)	7.5 (-6.4–24.4)	83		
Fiji	39.8 (17.1–64.9)	46 (15.7–75.8)	5.6 (-15.8–27.4)	69	●	○
Papua New Guinea	21.4 (15.6–28.5)	29.9 (14.6–49.8)	8.5 (-8.1–28.7)	83	●	●
Solomon Islands	24.6 (14.7–38)	26.4 (17.4–37.4)	1.7 (-14.6–16.8)	58	●	●
Vanuatu	28.1 (19.3–38.6)	41.7 (26.4–57.9)	13.4 (-4.1–31.3)	93	●	●
Micronesia	38.4 (28.2–45.6)	38.9 (27.7–49.6)	0.8 (-10.5–12.2)	55		
Guam	49.9 (29–63.2)	54.7 (29.3–75.7)	5.5 (-16.9–25.9)	67	●	○
Kiribati	25.8 (16.8–36)	21.8 (10.8–37.9)	-3.8 (-18.7–14.1)	33	●	●

continued

Table J. Modern contraceptive prevalence, married or in a union women of reproductive age (MWRA), 2000, 2019, and 2000–2019 change (cont'd).

Country or aggregate	Modern contraceptive prevalence 2000	2019	Change 2000–2019	PPI 2000–2019	Prevalence Obs.	Unmet Obs.
Polynesia	25.7 (19.7–33.2)	29.9 (22.1–39.2)	4.1 (-6.7–15.5)	77		
Samoa	23.7 (16.7–32.2)	27 (16.8–39.8)	3.3 (-10.3–18)	68	•	•
Tonga	24.8 (12.1–43.5)	31.5 (18.8–47.1)	6.4 (-15–26.3)	73	•	•

[a] The designation "more developed" and "less developed" regions are intended for statistical purposes and do not express a judgment about the stage reached by a particular country or area in the development process.

[b] More developed regions comprise Europe, Northern America, Australia/New Zealand and Japan.

[c] Less developed regions comprise all regions of Africa, Asia (except Japan), Latin America and the Caribbean plus Melanesia, Micronesia and Polynesia.

[d] The group of least developed countries includes 47 countries: 32 in Sub-Saharan Africa, 2 in Northern Africa and Western Asia, 4 in Central and Southern Asia, 4 in Eastern and South- Eastern Asia, 1 in Latin America and the Caribbean, 4 in Oceania. Further information is available at `http://unohrlls.org/about-ldcs/`.

[e] Other less developed countries comprise the less developed regions excluding the least developed countries.

5.3.2 Demand for family planning satisfied by modern methods in 185 countries or areas

Table K. Demand for family planning met by modern methods, married or in a union women of reproductive age (MWRA), 2000, 2019, and 2000–2019 change.
Key. PPI = posterior probability of an increase; • = observations available; ○ no observations available.

Country or aggregate	Met demand (modern) 2000	Met demand (modern) 2019	Change 2000–2019	PPI 2000–2019	Prevalence Obs.	Unmet Obs.
World	74.3 (73–75.4)	75.8 (73.4–78)	1.5 (-1–3.9)	88		
More developed regions[a,b]	71.7 (68–74.9)	78 (73.7–81.5)	6.3 (2–10.5)	100		
Less developed regions[a,c]	74.8 (73.5–76.1)	75.5 (72.9–77.9)	0.7 (-2.2–3.4)	68		
Least developed countries[a,d]	39.1 (37.3–40.8)	57.9 (54.4–61.1)	18.7 (14.8–22.3)	100		
Less dev. regions excl. China[a,c]	62.9 (61.2–64.6)	68.4 (65.1–71.5)	5.5 (1.7–9)	100		
Less dev. excl. least dev. [a,e]	78.4 (76.9–79.7)	78.2 (75.2–80.9)	-0.2 (-3.5–2.8)	45		
High-income countries	75.8 (71.8–79)	78.8 (74.7–82.2)	3 (-0.9–7)	94		
Upper-middle-income countries	86.3 (84.7–87.6)	87.2 (84.5–89.4)	0.9 (-2–3.3)	75		
Lower-middle-income countries	62.5 (60–64.9)	68.1 (63.1–72.8)	5.6 (0.1–10.9)	98		
Low-income countries	30.9 (29.3–32.4)	51.8 (49.3–54.3)	21 (18–23.9)	100		
Africa	41.1 (39.8–42.4)	56.5 (54.1–58.9)	15.5 (12.7–18.1)	100		
Eastern Africa	32.7 (31.1–34.4)	62.5 (59.3–65.6)	29.8 (26.3–33.3)	100		
Burundi	18.4 (13.4–24.5)	42.8 (33.7–52.4)	24.3 (13.3–35.2)	100	•	•
Comoros	27.2 (20.7–34.2)	36 (23–51)	8.8 (-6.1–24.8)	87	•	•
Djibouti	16.8 (9.6–28.3)	46.9 (28.9–66.6)	29.3 (10.4–49.4)	100	•	○
Eritrea	17.2 (13.3–21.7)	28.8 (15.4–47)	11.6 (-3.6–30.9)	92	•	•
Ethiopia	14.6 (12.6–17.1)	62 (54.6–69.4)	47.3 (39.7–55.1)	100	•	•
Kenya	47.9 (41.9–53.9)	81.7 (74.7–87.4)	33.7 (24.6–42.1)	100	•	•
Madagascar	26.7 (21.4–32.5)	63.4 (51.8–73.9)	36.7 (23.8–48.6)	100	•	•
Malawi	41.6 (38.2–44.7)	77.9 (68.4–85.3)	36.2 (26.3–44.6)	100	•	•
Mauritius	63.5 (48.6–75.3)	56.1 (37.5–72.6)	-7.1 (-27–12.3)	24	•	•
Mozambique	32.8 (26.3–39.7)	50.1 (36.2–64)	17 (0.9–33.4)	98	•	•
Réunion	83.8 (72.2–91.6)	87.1 (67.3–96.4)	3.1 (-13.5–14.2)	67	•	○
Rwanda	12.2 (10.3–14.6)	69 (57.6–78.8)	56.8 (45.1–66.7)	100	•	•
Somalia	0.9 (0–3.4)	16.6 (2.9–42.8)	15.4 (2.4–41.9)	100	•	○

continued

Table K. Demand for family planning met by modern methods, married or in a union women of reproductive age (MWRA), 2000, 2019, and 2000–2019 change, in countries in sexual activity group 1 (cont'd).

Country or aggregate	Met demand (modern) 2000	2019	Change 2000–2019	PPI 2000–2019	Prevalence Obs.	Unmet Data
South Sudan	10.2 (4.7–20.3)	16.7 (8.5–30.4)	6.4 (-5.4–20.5)	86	•	•
Uganda	29.8 (26–33.7)	53.6 (47–60.3)	23.8 (16.1–31.4)	100	•	•
United Rep. of Tanzania	38.4 (32.9–44)	57 (45–68.4)	18.6 (5.3–31.3)	100	•	•
Zambia	36.9 (31.2–42.7)	69.3 (56.3–80.2)	32.3 (18.4–44.8)	100	•	•
Zimbabwe	72.7 (67.9–77.1)	86.4 (78.1–92)	13.6 (4.6–21.2)	100	•	•
Middle Africa	14.8 (11.9–18.3)	27.3 (21.9–33.6)	12.4 (6.2–19.4)	100		
Angola	12.2 (7.9–18.7)	27.5 (19.5–36.9)	15.1 (5–25.9)	100	•	•
Cameroon	21.5 (16.1–27.5)	41.2 (27–56.6)	19.7 (4.3–35.9)	100	•	•
Central African Republic	20.7 (14.7–27.8)	38.2 (20.9–57.9)	17.1 (-0.5–37.5)	97	•	•
Chad	10.1 (7.2–13.9)	20.5 (13.1–30.2)	10.3 (1.9–20.4)	99	•	•
Congo	17.1 (8.5–28.6)	40.1 (25.1–56.5)	22.7 (2.9–43.1)	99	•	•
Democratic Rep. of the Congo	12.9 (8.3–19.1)	20.5 (11.9–31.8)	7.5 (-2.7–19.8)	92	•	•
Equatorial Guinea	13.6 (8.7–20.5)	27.8 (16.2–42.6)	14 (0.8–29.9)	98	•	•
Gabon	23 (18.7–27.3)	40.8 (26.4–56.7)	17.8 (3.1–34.5)	99	•	•
Sao Tome and Principe	38.5 (28.9–49.1)	57.1 (41.1–72.8)	18.3 (-0.5–36.9)	97	•	•
Northern Africa	68 (64.9–70.9)	72.8 (66.2–78.2)	4.7 (-2.2–10.7)	91		
Algeria	71.7 (61.8–80.1)	76.5 (60.8–87.7)	4.6 (-11.4–18.6)	73	•	•
Egypt	76.6 (74.3–78.8)	80.7 (70.1–88.4)	4 (-6.8–12.3)	79	•	•
Libya	36 (25.6–47.6)	32.6 (20.3–47.2)	-3.4 (-19.5–13.8)	34	•	•
Morocco	69.8 (62.3–76.5)	75.4 (65.9–83)	5.6 (-5.5–15.9)	85	•	•
Sudan	17.6 (11.2–27)	32.2 (19.5–48.6)	14.4 (0–30.8)	98	•	•
Tunisia	72 (63.7–79.2)	75.7 (59.2–87.1)	3.9 (-12.7–16.7)	69	•	•
Southern Africa	75.7 (70.8–80.1)	79.1 (71.6–85.2)	3.4 (-5.2–11.2)	79		
Botswana	65.4 (54.1–76.1)	79.1 (59.2–91.5)	13.2 (-6.1–28.5)	92	•	•
Eswatini	47.9 (39.1–57.6)	81 (67.3–90.2)	32.8 (16.4–46)	100	•	•
Lesotho	46 (39–53.2)	79.9 (68.6–88.3)	33.8 (20.5–44.9)	100	•	•
Namibia	63.1 (58.9–67.2)	78.5 (65.3–88.2)	15.3 (1.4–25.9)	99	•	•
South Africa	78.5 (72.9–83.4)	79.2 (70–86.3)	0.7 (-9.5–9.6)	56	•	•

continued

Table K. Demand for family planning met by modern methods, married or in a union women of reproductive age (MWRA), 2000, 2019, and 2000–2019 change, in countries in sexual activity group 1 (cont'd).

Country or aggregate	Met demand (modern) 2000	2019	Change 2000–2019	PPI 2000–2019	Prevalence Obs.	Unmet Data
Western Africa	22.2 (20–24.4)	42.3 (38.2–46.6)	20.1 (15.3–24.9)	100		
Benin	13.4 (10.4–16.9)	27.5 (21.5–34.2)	14 (7.2–21.4)	100	•	•
Burkina Faso	14.3 (11.3–17.8)	52.4 (43.2–61.9)	38 (28.3–48.1)	100	•	•
Cabo Verde	65.9 (56.7–74.1)	80.6 (61.8–92)	14.5 (-6–30.1)	92	•	•
Côte d'Ivoire	17.9 (13.4–23.3)	39.1 (31.8–46.9)	21.1 (12.2–30.2)	100	•	•
Gambia	29.2 (21.6–39.1)	27.7 (17.2–41.1)	-1.6 (-15.4–13.5)	42	•	•
Ghana	26 (21.8–30.4)	46.6 (38.2–55.1)	20.6 (11.2–30)	100	•	•
Guinea	14.1 (11.2–17.4)	23 (14.3–34.6)	8.9 (-0.3–20.8)	97	•	•
Guinea-Bissau	19.3 (11.7–31)	44.4 (28.1–62)	24.5 (6.8–42.6)	100	•	•
Liberia	20.3 (12.9–30.3)	51.3 (38.8–64.5)	30.7 (15.1–46.2)	100	•	•
Mali	17.7 (14.5–21.3)	35.2 (23.5–48.8)	17.4 (5.2–31.5)	100	•	•
Mauritania	12.8 (10.1–16.1)	30.5 (18.9–44.9)	17.7 (5.7–32.3)	100	•	•
Niger	24.6 (19.4–30.5)	45.3 (35.4–55.7)	20.6 (9–32.3)	100	•	•
Nigeria	25.4 (21.1–30)	42.1 (34.7–49.9)	16.7 (7.7–25.6)	100	•	•
Senegal	19.6 (15.7–24.1)	50.4 (41.7–59.1)	30.7 (21.1–40.3)	100	•	•
Sierra Leone	13.5 (8.9–20.2)	45.1 (33.5–57.6)	31.4 (18.3–44.8)	100	•	•
Togo	16.8 (13–21.3)	38 (26.5–50.6)	21 (8.8–34.5)	100	•	•
Asia	78.4 (76.8–79.8)	77.8 (74.5–80.9)	-0.5 (-4.1–2.8)	39		
Central Asia	74.2 (71–77.2)	75.9 (66.5–82.2)	1.6 (-8–8.6)	65		
Kazakhstan	73.2 (67.4–78.3)	76.1 (67.2–83.4)	2.9 (-6.9–12.1)	72	•	•
Kyrgyzstan	70.4 (62.1–77.7)	65.5 (50.7–78)	-5 (-22.1–10.9)	27	•	•
Tajikistan	51.7 (40.7–63.2)	47.7 (39.7–55.9)	-4.1 (-18.2–9.7)	28	•	•
Turkmenistan	73.2 (68.4–77.3)	73.7 (58.6–85)	0.4 (-14.9–12.1)	52	•	•
Uzbekistan	80 (74.6–84.7)	83.5 (66.4–93)	3.4 (-13.7–14)	68	•	•
Eastern Asia	93.6 (91.6–95)	92.9 (88.9–95.6)	-0.6 (-4.5–2.2)	34		
China	95.9 (93.8–97.3)	94.6 (90.3–97.4)	-1.2 (-5.3–1.7)	23	•	•
China, Hong Kong SAR	85.5 (63.2–94.2)	84.6 (62.8–94.5)	-0.3 (-13.9–13.3)	48	•	○
Democratic People's Rep. of Korea	70.5 (61.7–78.1)	85.3 (75.4–91.7)	14.6 (3.3–25.1)	99	•	•

continued

Table K. Demand for family planning met by modern methods, married or in a union women of reproductive age (MWRA), 2000, 2019, and 2000–2019 change, in countries in sexual activity group 1 (cont'd).

Country or aggregate	Met demand (modern) 2000	Met demand (modern) 2019	Change 2000–2019	PPI 2000–2019	Prevalence Obs.	Unmet Data
Japan	63.8 (54.9–71.9)	61.2 (45.5–74.7)	-2.5 (-18.6–12.3)	38	●	○
Mongolia	70.2 (62–77.3)	72.8 (58–83.9)	2.5 (-13.1–15.9)	63	●	●
Republic of Korea	81.6 (66.4–90.2)	84 (65.3–93.2)	2.4 (-11.6–14)	67	●	○
South-eastern Asia	70.3 (67.7–72.7)	75 (70.8–78.9)	4.8 (0–9.3)	98		
Cambodia	32.2 (29–35.5)	61.2 (46.9–73.3)	29.1 (14.5–41.4)	100	●	●
Indonesia	77.9 (73.4–81.8)	79.5 (71.3–86.1)	1.6 (-7.4–9.7)	65	●	●
Lao People's Dem. Republic	45 (37.3–53.2)	70 (57.8–80.3)	24.9 (11–37.3)	100	●	●
Malaysia	47.1 (32.1–62.3)	54.5 (37.7–69.7)	7.4 (-11.9–26.2)	78	●	○
Myanmar	54.3 (45.8–62.8)	77 (68.1–84.4)	22.6 (10.8–33.8)	100	●	●
Philippines	44.3 (39.1–49.4)	54.5 (45.5–63.2)	10.3 (-0.1–20.2)	97	●	●
Singapore	72.1 (57.8–83.1)	77.2 (56.1–91)	5.2 (-13.3–19.8)	72	●	○
Thailand	89.9 (85.2–93.1)	91.4 (84.7–95.3)	1.4 (-5.6–7.3)	67	●	●
Democratic Rep. of Timor-Leste	38.4 (31.4–45.7)	42.8 (33.4–52.9)	4.5 (-7.4–16.7)	77	●	●
Viet Nam	71.3 (65.1–76.6)	79 (68.1–87)	7.8 (-4.2–17.6)	90	●	●
Southern Asia	63.4 (59.8–66.8)	67.7 (60.4–74.4)	4.3 (-3.7–11.9)	86		
Afghanistan	19.9 (12.3–30.8)	47.3 (36.8–58.2)	27 (12.3–40.3)	100	●	●
Bangladesh	61.5 (57–65.6)	75.1 (64–83.6)	13.5 (1.7–23.1)	99	●	●
Bhutan	54.8 (42.2–68.2)	83.4 (66.5–93.3)	27.9 (9.3–43.8)	100	●	●
India	66.2 (61.5–70.8)	68.2 (58.4–77.1)	2 (-8.9–12)	64	●	●
Iran, Islamic Republic of	69.1 (59.8–76.7)	74.2 (56.1–86.8)	5.6 (-12.9–19.2)	74	●	●
Maldives	48.3 (39.9–56.8)	53.9 (36.5–70.4)	5.4 (-13.6–23.9)	71	●	●
Nepal	52 (47.1–57)	61.9 (52.5–70.5)	9.9 (-0.6–20)	97	●	●
Pakistan	36 (30.3–42.3)	49.8 (41.6–57.7)	13.7 (3.5–23.4)	100	●	●
Sri Lanka	64.7 (54.7–73.3)	71.6 (57.4–82)	7 (-8.3–20.1)	83	●	●
Western Asia	45.6 (40.8–50.2)	54.9 (47.6–61.5)	9.4 (1.1–16.9)	99		
Armenia	32.1 (26.2–38.2)	40.2 (28.4–52.7)	8.1 (-4.6–21.4)	89	●	●
Azerbaijan	24 (16.5–32.7)	29.8 (15.7–48.2)	6 (-9.6–24.4)	76	●	●
Bahrain	49.1 (31–67.3)	58.7 (32.1–80.6)	9.3 (-13.7–30.3)	78	●	○

continued

Table K. Demand for family planning met by modern methods, married or in a union women of reproductive age (MWRA), 2000, 2019, and 2000–2019 change, in countries in sexual activity group 1 (cont'd).

Country or aggregate	Met demand (modern) 2000	2019	Change 2000–2019	PPI 2000–2019	Prevalence Obs.	Unmet Data
Georgia	34.4 (24.8–45.2)	52.5 (34.5–69.7)	17.9 (-1.1–36.2)	97	●	●
Iraq	44.5 (33.6–56.1)	56.1 (42–68.8)	11.6 (-5.1–27.3)	91	●	●
Israel	65.2 (42.4–82.5)	69.2 (42.2–87.9)	4 (-17.2–22.7)	66	●	○
Jordan	55.5 (47.8–62.5)	55.9 (46–64.4)	0.4 (-11.6–11.4)	53	●	●
Kuwait	60 (46–72.3)	67.9 (43.8–85.4)	7.8 (-13.9–25.6)	77	●	○
Lebanon	53.3 (42.6–63.9)	62.4 (43.5–78.5)	9 (-10.8–26.8)	81	●	○
Oman	35.9 (25.7–47.8)	38 (24.2–53.4)	1.9 (-14.5–18.9)	59	●	●
Qatar	55.5 (41.5–68.8)	62.9 (43.8–78.7)	7.2 (-12.6–25.9)	76	●	●
Saudi Arabia	40 (26.5–56.2)	42.4 (27.3–59.7)	2.2 (-12.8–17.3)	61	●	○
State of Palestine	52.6 (41–63.5)	63.9 (47.6–77.7)	11.3 (-6.9–28.5)	89	●	●
Syrian Arab Republic	51.5 (40.9–62.3)	60.7 (41.7–76.9)	9.1 (-11–27.2)	82	●	●
Turkey	50.4 (40.7–59.4)	60.9 (43.6–75.6)	10.7 (-8.2–27.4)	87	●	●
United Arab Emirates	46.2 (28.8–64.9)	59.2 (32.2–81.3)	12.3 (-10.1–33.2)	86	●	○
Yemen	21 (15.8–27)	43.5 (30.3–57.1)	22.4 (8.2–36.8)	100	●	●
Europe	68.5 (64.9–71.8)	78.1 (73.7–82)	9.6 (4.5–14.3)	100		
Eastern Europe	59.6 (53–65.4)	72.9 (63.7–80.1)	13.3 (3.1–22.5)	99		
Belarus	64.1 (49.7–76.2)	75.5 (63.3–84.7)	11.2 (-3.9–27.4)	92	●	●
Bulgaria	44.9 (32.9–56.6)	65.3 (42–82.7)	20.4 (-3.7–40.2)	95	●	●
Czechia	72.3 (60.5–81.4)	86.6 (71.4–94.4)	14 (-1.9–27.2)	96	●	●
Hungary	76.8 (62–86.3)	77.9 (57.4–90.3)	1.2 (-17.9–16.6)	55	●	●
Poland	50.9 (33–67.8)	66.8 (46.2–82.2)	15.4 (-5.6–35.2)	93	●	○
Republic of Moldova	56.4 (44.5–66.9)	65.5 (46.6–80.5)	9.3 (-11.3–27.6)	81	●	●
Romania	41 (28–55.5)	71.8 (46.9–88.1)	30.4 (5.6–49.6)	99	●	●
Russian Federation	64.4 (52.8–73.8)	74.7 (58.1–86.6)	10.5 (-8.3–26.7)	87	●	●
Slovakia	68.2 (45.9–84.2)	79.2 (51.4–94.2)	10.6 (-10.9–28.7)	86	●	○
Ukraine	56.3 (42–68.5)	71.4 (53.2–84.8)	15 (-5.9–34.8)	92	●	●
Northern Europe	82 (75.4–86.6)	84.8 (74.8–90.7)	2.8 (-5.7–9.1)	77		
Denmark	85 (68.2–94.2)	86.5 (62.5–96.5)	1.5 (-16.1–13.2)	60	●	○

continued

Table K. Demand for family planning met by modern methods, married or in a union women of reproductive age (MWRA), 2000, 2019, and 2000–2019 change, in countries in sexual activity group 1 (cont'd).

Country or aggregate	Met demand (modern) 2000	2019	Change 2000–2019	PPI 2000–2019	Prevalence Obs.	Unmet Data
Estonia	67.4 (53.2–78.9)	75.2 (52.8–89.2)	7.6 (-14.6–25.6)	76	●	○
Finland	84.3 (68.8–90.9)	88.8 (75.5–94.7)	4.8 (-3.6–14.1)	88	●	○
Ireland	79.4 (67.7–86.5)	83.1 (65.5–92.6)	3.8 (-12.6–15.9)	70	●	○
Latvia	69.7 (52.3–82.8)	75.7 (49.8–91.5)	5.8 (-15.7–22.5)	72	●	●
Lithuania	55.7 (40.5–69.3)	68.5 (46–85)	12.6 (-10.3–32.9)	86	●	●
Norway	87 (73.6–92.7)	89.3 (72.6–96.1)	2.6 (-9.8–11.8)	71	●	○
Sweden	78.1 (60.5–88.6)	81 (56.2–93.7)	2.9 (-17–16.7)	64	●	○
United Kingdom	84.3 (75.1–90.5)	86.1 (71.2–94.2)	1.9 (-11–10.9)	64	●	○
Southern Europe	58.8 (51.2–65.6)	70.2 (61.3–77.4)	11.3 (2.4–20.2)	99		
Albania	17.4 (11.3–25.6)	6.9 (4.3–10.5)	-10.5 (-18.9—3.7)	0	●	●
Bosnia and Herzegovina	20.6 (13–30.8)	32.7 (16.6–53.2)	12 (-5.3–32.6)	91	●	●
Croatia	46.9 (16.5–82.2)	66.6 (28.6–92.4)	17.3 (-7.2–42.2)	92	●	○
Greece	45.3 (30.5–59.8)	63.8 (36.6–84.7)	18.6 (-6.6–39.8)	93	●	○
Italy	52.9 (34.6–68.6)	67.8 (47.6–82.5)	14.6 (-6.2–34.7)	92	●	●
Malta	78.6 (59.5–91.1)	82.2 (52.6–95.7)	3.3 (-19.3–19.6)	64	●	○
Montenegro	38.7 (28.2–50.6)	38.4 (22.3–56.6)	-0.6 (-18.4–18.6)	48	●	●
Portugal	78.2 (55.3–89.1)	83.4 (61–93.7)	5.4 (-9.6–20.9)	79	●	○
Serbia	35.4 (24.9–47.7)	41.7 (25–60.2)	6.2 (-13–25.9)	74	●	●
Slovenia	72.6 (51.4–86.6)	82.1 (56.2–94.8)	9 (-11–26.2)	84	●	●
Spain	79.1 (71.4–85.3)	85.2 (72.5–92.9)	6.1 (-6.8–16.1)	84	●	●
TFYR Macedonia	18 (7.3–37.4)	32.1 (15.7–54)	13.7 (-6.3–35)	91	●	●
Western Europe	85.4 (79.9–89.5)	89.7 (83.5–93.6)	4.3 (-1.8–10)	92		
Austria	75.4 (62–85.7)	82.2 (65.9–91.9)	6.5 (-10.8–22.3)	79	●	○
Belgium	83.9 (74.7–90.9)	88.5 (76.1–95.3)	4.4 (-7.7–14.2)	79	●	●
France	89.6 (84.4–93.1)	91.1 (81.1–96.1)	1.5 (-8.4–8.2)	64	●	●
Germany	83 (71.1–90.8)	91.5 (79.3–96.8)	8.1 (-3.8–19.9)	92	●	○
Netherlands	87.8 (77.6–93.6)	87.5 (72.1–95)	-0.3 (-13.1–9)	47	●	○
Switzerland	86.8 (76.6–93)	86.5 (74–93.8)	-0.3 (-12.6–10.5)	48	●	○

continued

Table K. Demand for family planning met by modern methods, married or in a union women of reproductive age (MWRA), 2000, 2019, and 2000–2019 change, in countries in sexual activity group 1 (cont'd).

Country or aggregate	Met demand (modern) 2000	2019	Change 2000–2019	PPI 2000–2019	Prevalence Obs.	Unmet Data
Latin America and the Caribbean	76.6 (73.8–79.1)	82.5 (78.5–85.7)	5.9 (1.4–9.9)	99		
Caribbean	73.2 (69.7–75.9)	73 (68.2–77.1)	-0.1 (-5.4–4.9)	48		
Antigua and Barbuda	73.2 (48.6–89.3)	79.1 (50.6–94.1)	5.5 (-14.9–24)	73	•	○
Bahamas	79.2 (55.9–92.4)	83.2 (55.1–95.8)	3.7 (-16.4–20.4)	68	•	○
Barbados	73.4 (56.4–86.1)	77.2 (60–89.3)	3.7 (-14.8–22.3)	66	•	•
Cuba	86.6 (78.1–92.2)	89 (78.1–94.9)	2.3 (-8.8–11.8)	68	•	•
Dominican Republic	79.2 (75.6–82.5)	84.2 (73.8–91.1)	5 (-5.7–12.8)	84	•	•
Grenada	67.8 (45.4–85.1)	75.5 (46.6–92.2)	7.2 (-14.1–25.3)	77	•	○
Guadeloupe	60.6 (33.1–83)	70.5 (36.9–90.8)	8.9 (-12.8–28.8)	80	•	○
Haiti	33.2 (29.8–36.6)	44.2 (36.3–52.5)	11 (2.4–20)	99	•	•
Jamaica	79.9 (72.3–85.9)	80.6 (63.6–90.9)	0.5 (-16.9–13.4)	53	•	•
Martinique	64.7 (37.6–85.5)	73 (40.8–91.6)	7.6 (-13.5–27.2)	77	•	○
Puerto Rico	82.6 (72.7–89.3)	84.7 (66.6–94.1)	2.1 (-14.3–13)	63	•	•
St. Lucia	70.4 (52–84.7)	75.5 (56.8–88.5)	5 (-14.4–24.6)	70	•	•
St. Vincent and the Grenadines	76.6 (52.4–90.9)	81.2 (53–94.9)	4.2 (-15.6–22.4)	70	•	○
Trinidad and Tobago	57.7 (47.3–68.1)	63.8 (44.6–79.5)	5.9 (-14.3–23.8)	72	•	•
United States Virgin Islands	80.6 (61.7–90.8)	83.7 (61.4–94.4)	3 (-14.1–17.8)	66	•	○
Central America	75.4 (70–79.9)	81.4 (73.6–86.9)	5.9 (-2.8–13.5)	92		
Belize	63.3 (51–74.4)	71 (56.9–82.5)	7.5 (-9.1–23.8)	81	•	•
Costa Rica	83.9 (76.6–89.1)	87.3 (77.9–93)	3.4 (-6.7–11.9)	77	•	•
El Salvador	76.3 (66.8–83.8)	82.6 (70.5–90.5)	6.2 (-6.9–18)	84	•	•
Guatemala	49.6 (43.2–56.1)	68.9 (56.9–78.8)	19.3 (6.1–31.1)	100	•	•
Honduras	65.5 (55.8–74.2)	78.8 (66.5–87.7)	13.2 (-1.4–26.2)	96	•	•
Mexico	77.7 (70.9–83.5)	82.6 (72.2–89.6)	4.7 (-6.7–14.4)	80	•	•
Nicaragua	77.7 (74.1–80.9)	92.3 (85.7–96)	14.4 (7.4–19.7)	100	•	•
Panama	70.9 (55.3–83.1)	76 (61–87.2)	5.1 (-12.7–22.9)	72	•	•
South America	77.6 (73.8–80.6)	84.1 (78.6–87.9)	6.5 (0.6–11.6)	98		
Argentina	78 (65.4–86)	82.3 (68.3–91.2)	4.2 (-10.2–18)	73	•	○

continued

Table K. Demand for family planning met by modern methods, married or in a union women of reproductive age (MWRA), 2000, 2019, and 2000–2019 change, in countries in sexual activity group 1 (cont'd).

Country or aggregate	Met demand (modern) 2000	Met demand (modern) 2019	Change 2000–2019	PPI 2000–2019	Prevalence Obs.	Unmet Data
Bolivia, Plurinational State of	37.4 (31–43.9)	55.7 (42–68.6)	18.3 (3.6–32.6)	99	●	●
Brazil	83.4 (76.9–88.3)	88.3 (78.4–94)	4.8 (-5.7–13.1)	84	●	●
Chile	73.7 (58.8–85.4)	84.8 (74.4–91.7)	10.9 (-2.5–25.9)	95	●	○
Colombia	75.7 (70.7–79.4)	86.4 (79.6–91.2)	10.7 (3–17.5)	100	●	●
Ecuador	69.7 (60.9–77.3)	82.8 (68.8–91.6)	13.2 (-1.7–24.8)	96	●	●
Guyana	52.7 (43.2–62.8)	60.9 (44.6–75.7)	7.8 (-10.2–25.7)	80	●	●
Paraguay	68.2 (57.7–76.8)	82.3 (71.3–89.9)	13.9 (1.3–25.9)	98	●	●
Peru	57.6 (51.2–63.3)	66.9 (56.4–75.7)	9.4 (-2.5–20)	94	●	●
Suriname	64.9 (52.5–76.5)	75.4 (55.9–88.9)	10 (-10.5–27.5)	85	●	●
Uruguay	87.8 (78.5–93.5)	90.1 (79.6–95.6)	2.2 (-8–11.5)	69	●	○
Venezuela, Bolivarian Republic of	75.8 (66.4–83.6)	82.9 (66.4–92.7)	7 (-8.5–18.3)	84	●	●
Northern America	81.2 (70.6–88.4)	82.3 (71.4–89.4)	1 (-8.8–10.5)	59		
Canada	88.1 (81.2–92.9)	89.5 (75.7–95.9)	1.4 (-11.4–9.1)	62	●	○
United States of America	80.3 (68.5–88.5)	81.5 (69.4–89.3)	1.1 (-9.7–11.6)	58	●	●
Oceania	74.3 (67.1–80)	73.6 (64.5–81)	-0.7 (-9.8–8)	44		
Australia and New Zealand	82.8 (74–88.9)	82.3 (71.3–89.9)	-0.3 (-10.9–9.2)	48		
Australia	82.4 (72.3–89.4)	82.1 (69.1–90.4)	-0.4 (-12.7–10.7)	47	●	○
New Zealand	84.8 (70.4–92.5)	85.6 (61.5–95.5)	0.7 (-18–12.2)	54	●	○
Melanesia	41.5 (32–51.7)	50.4 (33.2–68)	8.6 (-9.7–27.2)	82		
Fiji	59.5 (32.2–82.2)	65.6 (31.6–88.8)	5.3 (-17.4–26.3)	69	●	○
Papua New Guinea	38.1 (27.7–50)	48.6 (28–69.1)	10 (-12.4–32.3)	81	●	●
Solomon Islands	45.3 (29.9–61.9)	46.4 (33.1–60.4)	1.1 (-19.7–21.2)	54	●	●
Vanuatu	46.3 (33.2–60.4)	60.1 (42.2–76.1)	13.5 (-6.7–32.6)	91	●	●
Micronesia	58.8 (46–67.8)	60.4 (46.9–71.6)	1.8 (-10.8–13.9)	62		
Guam	67.9 (44.7–81.2)	72.5 (46.1–88.6)	5 (-16.7–23.3)	68	●	○
Kiribati	44.4 (29.5–59.6)	41.3 (23.5–60.8)	-3.3 (-24.1–18.6)	38	●	●
Polynesia	38.9 (30.9–48.1)	44.3 (34.5–54.8)	5.3 (-8.1–19)	78		
Samoa	34.3 (25.1–44.9)	38.8 (26–53.3)	4.5 (-12.9–22.4)	69	●	●

continued

Table K. Demand for family planning met by modern methods, married or in a union women of reproductive age (MWRA), 2000, 2019, and 2000–2019 change, in countries in sexual activity group 1 (cont'd).

Country or aggregate	Met demand (modern) 2000	Met demand (modern) 2019	Change 2000–2019	PPI 2000–2019	Prevalence Obs.	Unmet Data
Tonga	42.5 (24.2–63.3)	50.3 (33.2–67.4)	7.6 (-17.1–31.5)	73	•	•

[a] The designation "more developed" and "less developed" regions are intended for statistical purposes and do not express a judgment about the stage reached by a particular country or area in the development process.

[b] More developed regions comprise Europe, Northern America, Australia/New Zealand and Japan.

[c] Less developed regions comprise all regions of Africa, Asia (except Japan), Latin America and the Caribbean plus Melanesia, Micronesia and Polynesia.

[d] The group of least developed countries includes 47 countries: 32 in Sub-Saharan Africa, 2 in Northern Africa and Western Asia, 4 in Central and Southern Asia, 4 in Eastern and South- Eastern Asia, 1 in Latin America and the Caribbean, 4 in Oceania. Further information is available at http://unohrlls.org/about-ldcs/.

[e] Other less developed countries comprise the less developed regions excluding the least developed countries.

5.4 Women of reproductive age irrespective of marital status

5.4.1 Modern contraceptive prevalence in 185 countries or areas

Table L. Modern contraceptive prevalence, women of reproductive age irrespective of marital status (WRA), 2000, 2019, and 2000–2019 change, in 185 countries or areas.
Key. PPI = posterior probability of an increase.

Country or aggregate	Modern contraceptive prevalence 2000	2019	Change 2000–2019	PPI 2000–2019
World	42 (40.5–44.6)	44.3 (42.1–47)	2.2 (-0.1–4.6)	97
More developed regions[a,b]	46.3 (43.2–49.4)	51.3 (46.9–55.8)	5 (0.8–9.4)	99
Less developed regions[a,c]	40.9 (39.4–44.1)	43.1 (40.7–46.1)	2 (-0.6–4.7)	94
Least developed countries[a,d]	15.9 (15.2–16.6)	27.1 (25.1–29)	11.2 (9.1–13.2)	100
Less dev. regions excl. China[a,c]	30.9 (29.9–31.9)	36.4 (34.2–38.7)	5.5 (3.1–8)	100
Less dev. excl. least dev. [a,e]	44.5 (42.7–48)	46.2 (43.3–49.7)	1.6 (-1.4–4.6)	85
High-income countries	48.7 (45.3–52.3)	51.6 (47.2–56.1)	2.9 (-1.3–7.2)	91
Upper-middle-income countries	54.5 (51.7–60.4)	57.1 (52.9–63.1)	2.4 (-1.7–6.8)	88
Lower-middle-income countries	30 (28.6–31.5)	35.5 (32.2–38.9)	5.4 (1.8–9.1)	100
Low-income countries	12.4 (11.7–13.3)	23.9 (22.4–25.5)	11.5 (9.9–13.1)	100
Africa	16.8 (16.3–17.4)	26.1 (24.8–27.4)	9.3 (7.9–10.7)	100
Eastern Africa	13.5 (12.8–14.1)	30.2 (28.3–32.2)	16.7 (14.7–18.9)	100
Burundi	5.5 (4.3–7.2)	17 (12.8–22)	11.4 (7–16.6)	100
Comoros	11.7 (9.1–14.9)	14.4 (8.3–23.2)	2.7 (-4.4–11.7)	76
Djibouti	3.2 (2–8.1)	11 (6.2–21.8)	8 (2.7–15.9)	100
Eritrea	4.5 (3.6–5.6)	8.1 (4–15.8)	3.7 (-0.7–11.3)	94
Ethiopia	5 (4.4–5.7)	26 (22.3–30.1)	21 (17.3–25.1)	100
Kenya	23.5 (20.5–26.7)	43.8 (38.7–49.2)	20.4 (14.3–26.5)	100
Madagascar	10.5 (8.6–12.8)	34.1 (27–41.7)	23.7 (16.1–31.5)	100
Malawi	20.4 (18.7–21.8)	46.9 (39.1–54.7)	26.6 (18.5–34.4)	100
Mauritius	30.4 (23.9–36.3)	26.4 (17.5–35.7)	-3.8 (-14.1–6.8)	24
Mozambique	12.4 (10.1–15.2)	23.1 (15.8–32.5)	10.8 (2.9–20.4)	100
Réunion	46.4 (35.1–60.8)	47.5 (26.9–73.4)	0.9 (-16.9–21.8)	54

continued

Table L. Modern contraceptive prevalence, women of reproductive age irrespective of marital status (WRA), 2000, 2019, and 2000–2019 change, in all countries (cont'd).

Country or aggregate	Modern contraceptive prevalence 2000	2019	Change 2000–2019	PPI 2000–2019
Rwanda	3.9 (3.4–4.6)	29.3 (22.9–36.1)	25.4 (19–32.2)	100
Somalia	0.6 (0.1–3.5)	5.6 (1.3–18.3)	5.2 (0.8–16.3)	100
South Sudan	2.6 (1.4–5.8)	4.4 (2.3–10.3)	1.9 (-1.2–6.5)	88
Uganda	14.9 (13.2–16.7)	29.6 (25.7–33.9)	14.7 (10.3–19.2)	100
United Rep. of Tanzania	16.5 (14.1–19.2)	29.9 (22.3–38.5)	13.5 (5.3–22.3)	100
Zambia	16.3 (13.9–18.9)	35.6 (26.5–45.4)	19.3 (9.8–29.5)	100
Zimbabwe	37.2 (34.3–40.3)	49.7 (41.9–57.2)	12.4 (4–20.4)	100
Middle Africa	6.7 (5.7–8)	14.5 (11.3–18.7)	7.8 (4.4–12)	100
Angola	5.5 (4–7.8)	14.3 (10.2–19.9)	8.7 (4–14.5)	100
Cameroon	10.3 (8–13.2)	24.9 (15.9–35.9)	14.6 (5.1–25.7)	100
Central African Republic	7.9 (5.8–10.6)	16.2 (8–29.4)	8.5 (-0.3–21.5)	97
Chad	2.2 (1.7–3)	5.9 (3.8–9.2)	3.7 (1.4–7)	100
Congo	10.1 (4.9–18.4)	26.3 (16.5–37.9)	16 (3–29.3)	99
Democratic Rep. of the Congo	5.9 (4.1–8.4)	11.2 (6.1–18.6)	5.2 (-0.4–13)	96
Equatorial Guinea	6.5 (3.9–11.2)	14.4 (8–24.7)	7.8 (-0.1–18.4)	97
Gabon	15.2 (13.4–17.1)	28.3 (17.6–41.5)	13.3 (2.3–26.5)	99
Sao Tome and Principe	19.7 (14.8–26)	35 (24.2–47.1)	15.2 (2.7–28.7)	99
Northern Africa	26.8 (25.6–28.5)	30.6 (26.7–34.9)	3.9 (-0.3–7.9)	97
Algeria	25.2 (21.1–30.6)	27 (19.5–37.2)	2.1 (-6.3–10.8)	68
Egypt	35.4 (33.9–36.8)	41.7 (33.1–49.6)	6.4 (-2.3–14.4)	93
Libya	11.2 (8.1–16.9)	8.6 (5.3–18.5)	-2.4 (-7.4–4.4)	21
Morocco	29.4 (25.5–34.5)	31.8 (27.7–39.2)	2.6 (-3.1–9)	82
Sudan	4.4 (3–7.6)	9 (5.1–17.1)	4.8 (0.3–11.6)	98
Tunisia	28.6 (25.2–33.5)	29.5 (21.3–39.4)	1.1 (-7.7–9.7)	60
Southern Africa	47.8 (43.4–52.1)	49.4 (42–56.7)	1.6 (-6.8–10)	65
Botswana	38.3 (27.6–52.2)	49.1 (30.1–70.8)	10.3 (-4.9–27.8)	90
Eswatini	25.6 (20.1–33)	52.3 (40.1–64.7)	26.4 (12.2–40.4)	100
Lesotho	25.2 (21.7–29.1)	50.7 (40.4–61.2)	25.3 (14.3–36.7)	100

continued

Table L. Modern contraceptive prevalence, women of reproductive age irrespective of marital status (WRA), 2000, 2019, and 2000–2019 change, in all countries (cont'd).

Country or aggregate	Modern contraceptive prevalence 2000	2019	Change 2000–2019	PPI 2000–2019
Namibia	37.3 (34.1–40.6)	51.7 (38.8–64.8)	14.5 (1.1–27.8)	98
South Africa	49.9 (44.9–54.7)	49.1 (40.9–57.5)	-0.7 (-10.3–8.9)	44
Western Africa	8.2 (7.5–9)	18.4 (16.6–20.3)	10.2 (8.2–12.2)	100
Benin	6 (4.9–7.4)	13.8 (10.4–18.3)	7.9 (4–12.5)	100
Burkina Faso	7.1 (5.8–8.5)	25.7 (21–31)	18.6 (13.7–24.1)	100
Cabo Verde	34 (28.7–40.1)	46.4 (29.1–65.5)	12.3 (-5.6–31.9)	91
Côte d'Ivoire	10.2 (7.9–12.8)	21.4 (17.2–26.3)	11.2 (6.3–16.5)	100
Gambia	9.6 (7.7–12.2)	8 (4.8–13.4)	-1.6 (-5.7–4)	26
Ghana	11.9 (10.1–14)	22.6 (18.3–27.8)	10.7 (5.9–16.2)	100
Guinea	5.1 (4.2–6.3)	9.6 (6.5–14.1)	4.5 (1.2–9)	100
Guinea-Bissau	7 (5.3–9.8)	25.9 (16.9–37.4)	18.9 (9.3–30.5)	100
Liberia	10.5 (7.4–15)	27.6 (20.1–36.8)	17 (8.3–26.7)	100
Mali	6.5 (5.4–7.7)	14 (9–21.6)	7.5 (2.3–15.1)	100
Mauritania	3.4 (2.6–6.7)	8.8 (5.2–16.6)	5.5 (1.7–11.7)	100
Niger	6.2 (4.9–7.8)	13.9 (10.3–18.3)	7.6 (3.7–12.3)	100
Nigeria	8.1 (6.9–9.5)	17.6 (14.5–21)	9.5 (6.1–13.2)	100
Senegal	7.2 (6–8.7)	19.6 (15.5–24.5)	12.4 (8–17.4)	100
Sierra Leone	6.3 (4.9–8.4)	24.9 (18.6–33)	18.6 (11.7–26.8)	100
Togo	10.7 (8.4–13.5)	19.7 (12.9–29)	9 (1.5–18.4)	99
Asia	45.1 (43–49.3)	46 (42.7–50.2)	0.8 (-2.7–4.3)	67
Central Asia	37 (35.1–38.9)	38.9 (31.7–45)	1.9 (-5.5–8.3)	70
Kazakhstan	39.4 (35.9–42.9)	40 (33.9–46.3)	0.6 (-6.4–7.7)	57
Kyrgyzstan	32.8 (27.9–38)	27.7 (18.7–37.7)	-5.1 (-15.5–6)	18
Tajikistan	20.3 (15.4–26)	20.1 (15.9–24.8)	-0.2 (-7.4–6.7)	47
Turkmenistan	34.9 (32.6–36.9)	33.4 (24.3–42.8)	-1.2 (-10.6–8.1)	40
Uzbekistan	40.5 (37.2–43.7)	46 (31.6–58.2)	5.6 (-9.1–18)	78
Eastern Asia	63.6 (59–73.4)	65.2 (58.3–75.6)	1.3 (-5.1–8.4)	66
China	66.9 (62.1–77.5)	67.9 (60.4–79.3)	0.6 (-6.5–8.5)	57

continued

Table L. Modern contraceptive prevalence, women of reproductive age irrespective of marital status (WRA), 2000, 2019, and 2000–2019 change, in all countries (cont'd).

Country or aggregate	Modern contraceptive prevalence 2000	2019	Change 2000–2019	PPI 2000–2019
China, Hong Kong SAR	48.5 (35.7–66.8)	46.5 (30.9–70.3)	-2.3 (-14–13.8)	38
Democratic People's Rep. of Korea	43.3 (36.1–57.2)	56 (44.7–72.1)	12.1 (1.7–23.7)	99
Japan	36 (27.6–50.9)	39.2 (26.5–55.4)	2.6 (-11.1–17.4)	65
Mongolia	36.5 (32.5–40.6)	39.8 (29.2–50.8)	3.3 (-8–14.9)	71
Republic of Korea	45.5 (35.5–63.7)	49.2 (35–70.1)	3 (-8.1–16.6)	70
South-eastern Asia	33.6 (32.1–35.1)	39 (36.1–41.9)	5.5 (2.2–8.7)	100
Cambodia	11.8 (10.8–13)	30 (22.3–37.9)	18.2 (10.5–26.1)	100
Indonesia	39 (35.9–42)	43.3 (37.1–49)	4.3 (-2.5–10.9)	89
Lao People's Dem. Republic	18.3 (15.5–22.1)	34.1 (26.9–42.1)	15.8 (7.7–23.7)	100
Malaysia	20.1 (13.6–28.5)	23.3 (16.4–33.1)	3.4 (-6.3–13.3)	75
Myanmar	17.7 (14.9–21.1)	31.5 (26.1–36.7)	13.7 (7.5–19.7)	100
Philippines	19.9 (17.7–22.2)	25.8 (21.3–30.3)	5.9 (0.9–10.9)	99
Singapore	34 (26.4–43.3)	34.7 (22.2–54)	1.1 (-11–16.7)	57
Thailand	45.3 (41.8–48.5)	46.6 (40.6–52)	1.4 (-5.5–7.9)	66
Democratic Rep. of Timor-Leste	9.9 (7.8–12.3)	12.9 (9.5–16.9)	3 (-1–7.5)	92
Viet Nam	38.7 (35.5–41.8)	48.4 (40.3–55.4)	9.6 (1.1–17.2)	99
Southern Asia	32.2 (30.1–34.5)	36.7 (31.6–42.1)	4.5 (-1.1–10.1)	94
Afghanistan	5.3 (3.4–8.8)	16.7 (11.9–24.1)	11.6 (5.9–18.1)	100
Bangladesh	36.3 (33.7–38.9)	46.5 (37.2–55)	10.2 (0.5–19)	98
Bhutan	20.9 (15.8–27.1)	42.1 (30.1–53)	21.1 (8.1–32.8)	100
India	34.1 (31.2–37.1)	38.1 (31–45.4)	4 (-3.7–11.8)	85
Iran, Islamic Republic of	37.6 (33.1–42.6)	43.1 (31.7–54.1)	5.6 (-6.3–16.1)	83
Maldives	20.8 (17.3–24.6)	25.9 (15.4–37.5)	5.1 (-6.1–17.2)	80
Nepal	26.8 (23.8–30)	35.7 (29.4–42)	8.9 (1.8–15.9)	99
Pakistan	13.4 (11.3–16.8)	17.8 (14.6–23.6)	4.6 (0.4–9.4)	98
Sri Lanka	33 (28.1–37.7)	37.4 (29.5–44.9)	4.5 (-4.4–12.8)	85
Western Asia	20.3 (18.2–22.4)	24.6 (21.2–28.2)	4.4 (0.6–8.1)	99
Armenia	15.6 (13.3–18)	20.1 (14.2–26.6)	4.5 (-1.7–11.4)	92

continued

Table L. Modern contraceptive prevalence, women of reproductive age irrespective of marital status (WRA), 2000, 2019, and 2000–2019 change, in all countries (cont'd).

Country or aggregate	Modern contraceptive prevalence 2000	2019	Change 2000–2019	PPI 2000–2019
Azerbaijan	10.2 (7.4–13.6)	13.4 (7.1–22.7)	3.3 (-3.8–12.6)	80
Bahrain	20.7 (12.9–30.6)	22.2 (11.3–36.2)	1.6 (-9–13.5)	61
Georgia	12.8 (9.1–17.2)	23.1 (13.6–33.8)	10.3 (0.4–21.2)	98
Iraq	17.2 (13.2–22.9)	24 (18–32.1)	7 (-0.6–15)	96
Israel	30.3 (19.2–43.2)	30.1 (16.8–50.6)	0.2 (-12–15.4)	51
Jordan	24.4 (20.9–27.7)	22.1 (18.3–25.9)	-2.2 (-7.2–2.8)	19
Kuwait	25.5 (19–32.5)	30 (16.7–44.9)	4.7 (-7.9–17.9)	76
Lebanon	20.3 (16.6–26.1)	21.4 (14.1–33)	1.3 (-6.9–10.6)	62
Oman	13.6 (9.8–19.3)	13.5 (8.2–22.8)	0.2 (-6.9–8.6)	52
Qatar	21.4 (15.3–29.4)	25.1 (15.1–37.3)	3.7 (-7.3–15.8)	74
Saudi Arabia	13.2 (8.6–20.4)	15.6 (9.7–25)	2.5 (-3.9–9.7)	77
State of Palestine	23.3 (18.4–29.1)	28.3 (20.1–38.5)	5.3 (-4.4–15.3)	86
Syrian Arab Republic	20.5 (16.4–26.2)	23.7 (15.2–35.5)	3.3 (-6.2–13.9)	75
Turkey	26.1 (21.4–30.7)	30.9 (21.9–39.5)	4.8 (-5.1–14.2)	84
United Arab Emirates	18.1 (10.3–29.2)	26.8 (11.5–44.7)	8.3 (-4.9–23.3)	88
Yemen	8.3 (6.2–12.1)	18.8 (12.4–28.3)	10.7 (3.7–19)	100
Europe	43.2 (40.5–45.9)	50.3 (45.7–55)	7.1 (2.5–11.9)	100
Eastern Europe	34.2 (30.4–38.1)	42.9 (35.5–51.1)	8.7 (0.7–17.5)	98
Belarus	38.5 (30.2–47.5)	52.2 (43.1–60.4)	13.4 (1.6–25.3)	98
Bulgaria	35.5 (26.7–45.5)	47.5 (29.8–66.9)	12 (-5–29.5)	91
Czechia	42.9 (35.1–50.7)	49.8 (35.5–65.4)	6.9 (-8.6–22.8)	81
Hungary	42.7 (33.4–52.6)	41.3 (26.4–58.4)	-1.4 (-16.9–15.4)	43
Poland	28.7 (19.6–39.5)	38.6 (26.7–50.8)	9.5 (-4.2–23.4)	91
Republic of Moldova	33.1 (27–39)	41.5 (28.2–55.2)	8.5 (-6–23.4)	87
Romania	26.4 (17.8–41)	44 (26.5–65.9)	17 (1.5–33.9)	98
Russian Federation	34.3 (27.9–40.8)	41.9 (28.9–56.8)	7.7 (-6.6–23.2)	85
Slovakia	39.2 (26–57.4)	45.6 (25.1–71.4)	5.8 (-10.9–25.2)	74
Ukraine	35.7 (28.2–42.9)	44.6 (31.4–58.2)	9 (-6.4–24.5)	88

continued

Table L. Modern contraceptive prevalence, women of reproductive age irrespective of marital status (WRA), 2000, 2019, and 2000–2019 change, in all countries (cont'd).

Country or aggregate	Modern contraceptive prevalence 2000	2019	Change 2000–2019	PPI 2000–2019
Northern Europe	61.5 (55.2–66.8)	63.3 (50.6–73.6)	1.8 (-9.5–11.5)	63
Denmark	58.8 (41.9–77)	58.5 (32.8–83.3)	-0.4 (-20–17.9)	48
Estonia	45.4 (30.6–64.2)	49.8 (27.1–75.6)	4.3 (-12.5–21.4)	69
Finland	67.8 (54.8–74.6)	73.2 (58.9–82.1)	5.7 (-5–15.9)	86
Ireland	58.5 (45.4–68.4)	62.3 (43.2–77.6)	3.9 (-14.2–20.5)	67
Latvia	47.9 (31.3–68.5)	51.5 (26.4–78.9)	3.2 (-16.2–22.5)	62
Lithuania	29.6 (22.2–38.3)	34.9 (20.9–51.8)	5.2 (-9.9–22.1)	75
Norway	61.3 (46.4–77.8)	61.4 (40.8–83.7)	0.1 (-13.6–14.2)	51
Sweden	52.9 (36.7–71.5)	54.7 (29.6–80.4)	1.8 (-17.1–19.7)	58
United Kingdom	65.7 (57–72.3)	66.3 (47.5–81)	0.7 (-16.5–15)	53
Southern Europe	35.9 (30.7–42.1)	45.6 (37.7–53.5)	9.5 (1.4–17.7)	99
Albania	9.7 (6.6–13.7)	3.5 (2.3–5.2)	-6.1 (-10.3—2.7)	0
Bosnia and Herzegovina	11 (7.7–15.8)	18.4 (9.7–31.3)	7.4 (-2.4–20.3)	92
Croatia	27.1 (11.2–50)	39 (16.8–65.2)	10.8 (-7–30.2)	88
Greece	25 (17–39.3)	37.9 (19.9–60.9)	12.5 (-3.7–30.4)	93
Italy	32.3 (21.4–46)	46.2 (30.7–61.8)	13.3 (-3.1–29.7)	94
Malta	37.1 (24.7–54.1)	45.9 (22.8–70.3)	8.1 (-11–27.6)	80
Montenegro	19.9 (15.3–25.9)	21.4 (12.7–33.4)	1.6 (-9–14.1)	60
Portugal	47.3 (33.3–59.1)	53 (37.9–66.4)	5.6 (-8.9–20.6)	77
Serbia	24.7 (18.2–32.6)	31.9 (20.8–45)	7.1 (-6.3–21.8)	85
Slovenia	38.3 (26.4–55.3)	43.9 (24.8–68.3)	5.1 (-11.5–24.2)	72
Spain	48.8 (42.2–56.4)	54.5 (41.9–69.1)	5.8 (-6.8–19.1)	82
TFYR Macedonia	11.9 (6.1–22.4)	21 (11.3–35.4)	8.8 (-3.6–23.2)	92
Western Europe	56.1 (51–61.4)	59.2 (50.7–67.4)	3.1 (-5.7–11.9)	76
Austria	52.4 (40.7–63.5)	58.5 (42.8–72.1)	5.9 (-11–22.5)	75
Belgium	52 (43.6–61.4)	57.6 (43.7–70.8)	5.3 (-9.9–20.3)	76
France	59.4 (52.5–66.8)	61.1 (45.9–75.3)	1.6 (-14.4–16.7)	58
Germany	52.9 (43.5–62.2)	57 (42.3–72.4)	4.1 (-11.8–20.1)	69

continued

Table L. Modern contraceptive prevalence, women of reproductive age irrespective of marital status (WRA), 2000, 2019, and 2000–2019 change, in all countries (cont'd).

Country or aggregate	Modern contraceptive prevalence 2000	2019	Change 2000–2019	PPI 2000–2019
Netherlands	59.9 (51.5–66.5)	59.7 (45.2–72.6)	-0.1 (-14.5–13.4)	49
Switzerland	66.6 (54.1–76.4)	68.1 (52.5–80.1)	1.6 (-13.9–16.5)	58
Latin America and the Caribbean	44.7 (42.5–47.1)	54.4 (49.7–59.2)	9.6 (4.6–14.7)	100
Caribbean	41.3 (38.1–44.7)	47 (42.5–51.5)	5.7 (0.4–10.9)	98
Antigua and Barbuda	31.5 (18.1–52.3)	41.2 (20.3–67.7)	8.7 (-7.8–29)	84
Bahamas	35.3 (21.7–54.9)	42.1 (21.2–68.4)	6 (-10.8–26.1)	75
Barbados	41 (30.5–52.1)	46.8 (33.4–60.9)	5.8 (-10–21.6)	76
Cuba	60.3 (51.1–69.6)	68.5 (56.5–78.4)	7.9 (-6.2–21.4)	87
Dominican Republic	45.6 (42.9–48.3)	54.3 (43.9–64.2)	8.7 (-1.8–19)	94
Grenada	25.8 (13.7–49)	38.5 (18–66.1)	11.4 (-5.4–31.5)	91
Guadeloupe	26.4 (13–48.6)	37.1 (15.7–64.7)	9.3 (-6.7–29.9)	86
Haiti	14.6 (13.3–16.1)	25.4 (20.6–30.9)	10.8 (5.7–16.5)	100
Jamaica	36.4 (31.7–41.6)	37.8 (24–55.1)	1.3 (-13–18.8)	56
Martinique	26.8 (13.6–49.2)	37.2 (16.2–64.9)	9.2 (-7–29.4)	86
Puerto Rico	44.1 (35.8–59.2)	50.7 (33.4–71.2)	5.7 (-8.1–21)	79
St. Lucia	36.1 (25.7–48.3)	44.8 (30.7–59.6)	8.4 (-8.2–25.3)	84
St. Vincent and the Grenadines	38.8 (23.7–57)	46.4 (24.6–70.2)	6.9 (-10.2–26.3)	78
Trinidad and Tobago	23.4 (19.1–28.4)	34.5 (21.5–49.7)	11.2 (-2.5–26.5)	94
United States Virgin Islands	33.3 (22–53.8)	40.9 (22.4–67.6)	6.8 (-7.2–25.7)	81
Central America	40.7 (37.2–44.6)	49 (42.3–55.5)	8.2 (0.8–15.4)	98
Belize	33.1 (26–41)	40.2 (30.2–51.9)	7 (-5.2–20.1)	87
Costa Rica	47.8 (40.2–58.4)	54.7 (43.4–67.4)	6.5 (-7–20.4)	83
El Salvador	38.7 (32.9–44.5)	47 (37.2–57.4)	8.4 (-2.6–19.6)	93
Guatemala	21.3 (18.3–24.6)	34.9 (27.4–42.7)	13.6 (5.5–21.9)	100
Honduras	33.3 (27.9–38.7)	45.9 (35.7–56.4)	12.6 (1.1–24.5)	98
Mexico	43 (38.4–48.2)	51 (42.1–59.7)	7.9 (-2–17.7)	94
Nicaragua	41.3 (39–43.5)	49.3 (41.2–56.7)	8.1 (-0.3–15.8)	97
Panama	38.5 (28.4–49.7)	46.4 (34.4–59.1)	7.7 (-7.8–23.1)	84

continued

Table L. Modern contraceptive prevalence, women of reproductive age irrespective of marital status (WRA), 2000, 2019, and 2000–2019 change, in all countries (cont'd).

Country or aggregate	Modern contraceptive prevalence 2000	2019	Change 2000–2019	PPI 2000–2019
South America	46.6 (43.5–49.8)	57.5 (50.8–64.2)	10.8 (3.9–17.8)	100
Argentina	45.1 (36.1–55.9)	54.7 (41.2–68.1)	9.2 (-5.7–24.3)	89
Bolivia, Plurinational State of	17.9 (15.1–20.8)	31.1 (23.5–39.7)	13.3 (5.1–22.1)	100
Brazil	53.1 (47.4–58.6)	63.1 (50.8–75.1)	9.9 (-2.8–22.7)	94
Chile	38 (27.9–51.5)	56.2 (43.7–69)	17.6 (2.4–32.4)	99
Colombia	45.4 (42.8–47.6)	59.9 (52.2–67.5)	14.6 (6.6–22.5)	100
Ecuador	35.7 (31.2–40.3)	52.3 (39.9–64.9)	16.6 (3.5–29.8)	99
Guyana	24.9 (20.1–31)	31.4 (21.6–42.7)	6.4 (-4.8–18.6)	86
Paraguay	36.1 (30.2–42.2)	54 (44.7–62.8)	17.8 (7.3–27.9)	100
Peru	31 (28.3–33.4)	40.2 (33.9–46.2)	9.3 (2.5–15.7)	100
Suriname	29.3 (24–35.1)	43 (28.5–58.9)	13.7 (-1.8–30.2)	96
Uruguay	48 (40–60.2)	54.4 (41.8–70.6)	5.8 (-5.4–19.5)	84
Venezuela, Bolivarian Republic of	41.1 (35.1–48.4)	51.8 (37.4–68)	10.4 (-3.5–25.6)	93
Northern America	56.5 (48–64.6)	56.9 (46.5–67)	0.4 (-9.8–10.5)	53
Canada	68.2 (59.2–75.4)	68.5 (47.4–84.6)	0.3 (-19–15.4)	52
United States of America	55.2 (45.9–64.2)	55.7 (44.3–66.8)	0.5 (-10.7–11.6)	54
Oceania	46.1 (39.1–52.7)	46.5 (37.9–54.8)	0.4 (-8.8–9.7)	53
Australia and New Zealand	55.2 (46–63.8)	55.8 (44.4–66.4)	0.6 (-11.4–12.5)	54
Australia	54.9 (44.7–64.6)	55.6 (42.9–67)	0.5 (-12.9–14.1)	53
New Zealand	56.4 (41.6–72.1)	57.5 (32.9–80.6)	1.2 (-19.6–19.7)	54
Melanesia	17.1 (13.3–21.6)	22.7 (13.3–35.5)	5.7 (-4.3–18.3)	85
Fiji	26 (12.1–44.6)	31.3 (11.6–57.3)	4.9 (-10.4–23.4)	73
Papua New Guinea	15.4 (11.4–20.5)	21.6 (10.6–36.8)	6.3 (-5.7–21.6)	83
Solomon Islands	17.3 (10.8–26.3)	19.4 (13.1–27.5)	2.1 (-9–12.6)	65
Vanuatu	20.6 (14.7–27.9)	31.8 (20.7–44.4)	11.1 (-1.8–24.4)	95
Micronesia	28.6 (21.6–39)	30 (20.6–43.8)	1.4 (-7.2–11.2)	62
Guam	33.6 (20.9–52.7)	36.2 (18.5–63.9)	2.4 (-11.9–20.8)	62
Kiribati	21.6 (15.2–29)	18.1 (9.3–32.1)	-3.3 (-14.6–11.2)	32

continued

Table L. Modern contraceptive prevalence, women of reproductive age irrespective of marital status (WRA), 2000, 2019, and 2000–2019 change, in all countries (cont'd).

Country or aggregate	Modern contraceptive prevalence 2000	2019	Change 2000–2019	PPI 2000–2019
Polynesia	16.4 (12.9–20.6)	18.1 (13.6–23.5)	1.8 (-4.5–8.3)	71
Samoa	14.9 (10.7–20.1)	15.7 (10–23.2)	0.9 (-7.1–9.4)	58
Tonga	13.8 (7.3–23.5)	17.1 (10.3–25.9)	3.2 (-8–13.9)	72

[a] The designation "more developed" and "less developed" regions are intended for statistical purposes and do not express a judgment about the stage reached by a particular country or area in the development process.
[b] More developed regions comprise Europe, Northern America, Australia/New Zealand and Japan.
[c] Less developed regions comprise all regions of Africa, Asia (except Japan), Latin America and the Caribbean plus Melanesia, Micronesia and Polynesia.
[d] The group of least developed countries includes 47 countries: 32 in Sub-Saharan Africa, 2 in Northern Africa and Western Asia, 4 in Central and Southern Asia, 4 in Eastern and South- Eastern Asia, 1 in Latin America and the Caribbean, 4 in Oceania. Further information is available at http://unohrlls.org/about-ldcs/.
[e] Other less developed countries comprise the less developed regions excluding the least developed countries.

5.4.2 Demand for family planning satisfied by modern methods in 185 countries or areas

Table M. Demand for family planning met by modern methods, women of reproductive age irrespective of marital status (WRA), 2000, 2019, and 2000–2019 change, in 185 countries or areas.
Key. PPI = posterior probability of an increase.

Country or aggregate	Met demand (modern) 2000	2019	Change 2000–2019	PPI 2000–2019
World	73.5 (71.3–75.3)	75.7 (73.2–78)	2.2 (-0.3–4.7)	96
More developed regions[a,b]	72.9 (68.9–76)	79.4 (74.6–82.9)	6.5 (2.4–10.3)	100
Less developed regions[a,c]	73.8 (71.2–75.7)	75.1 (72.3–77.6)	1.3 (-1.6–4.2)	81
Least developed countries[a,d]	39.1 (37.5–40.8)	57.6 (54.4–60.6)	18.5 (14.9–21.8)	100
Less dev. regions excl. China[a,c]	63.1 (61.2–64.6)	68.8 (65.6–71.7)	5.8 (2.3–9.1)	100
Less dev. excl. least dev. [a,e]	77.2 (74.3–79.2)	77.8 (74.5–80.5)	0.5 (-2.6–3.8)	63
High-income countries	76 (71.7–79.3)	79.9 (75–83.5)	3.9 (0–6.4)	98
Upper-middle-income countries	84.1 (79.9–86.4)	85.7 (81.8–88.4)	1.5 (-1.6–3.6)	84
Lower-middle-income countries	62.2 (59.4–64.5)	67.9 (62.8–72.4)	5.7 (0.3–9)	98
Low-income countries	32.1 (30.5–34)	52.6 (50.1–55.2)	20.5 (17.6–22.4)	100
Africa	43.1 (41.8–44.4)	57.8 (55.6–60)	14.7 (12.2–17.1)	100
Eastern Africa	34.8 (33.2–36.4)	62.6 (59.7–65.4)	27.8 (24.5–31)	100
Burundi	19.6 (14.9–25.3)	43.2 (34.5–52.4)	23.5 (13.1–34)	100
Comoros	29.4 (23.4–35.9)	37.6 (24.5–52.4)	8.2 (-6.5–23.9)	86
Djibouti	18.3 (10.9–36.1)	46.4 (29.1–66.3)	26.7 (9.4–45.3)	100
Eritrea	18.5 (14.6–23.1)	30.1 (16.5–48.3)	11.7 (-3.9–30.8)	92
Ethiopia	15.7 (13.7–18.2)	62.5 (55.5–69.4)	46.7 (39.5–54)	100
Kenya	48.1 (42.8–53.3)	78.7 (72.2–83.9)	30.4 (22.3–38.1)	100
Madagascar	26.1 (21.4–31.3)	61.5 (50.9–71.3)	35.4 (23.7–46.2)	100
Malawi	42.5 (39.3–45.3)	75.8 (67.3–82.7)	33.3 (24.4–41.1)	100
Mauritius	62.4 (47.9–73.6)	55.2 (37.3–71.1)	-6.7 (-26–11.9)	24
Mozambique	36.6 (30.6–43.2)	52.4 (40.7–64.3)	15.7 (1.5–30)	98
Réunion	79.4 (67.3–88.1)	82.1 (63.5–94.5)	1.9 (-12.5–15.3)	61
Rwanda	13.3 (11.4–15.6)	66.8 (56.1–75.9)	53.3 (42.5–62.7)	100
Somalia	2.1 (0.5–11.7)	17.7 (4.6–44.8)	15.5 (2.7–40.4)	100

continued

Table M. Demand for family planning met by modern methods, women of reproductive age irrespective of marital status (WRA), 2000, 2019, and 2000–2019 change, in all countries (cont'd).

Country or aggregate	Met demand (modern) 2000	Met demand (modern) 2019	Change 2000–2019	PPI 2000–2019
South Sudan	11 (5.6–23.2)	17.8 (9.5–35.4)	7.1 (-4.6–21.1)	88
Uganda	33.9 (30.4–37.4)	55.4 (49.5–61.3)	21.5 (14.5–28.3)	100
United Rep. of Tanzania	40.6 (35.2–45.9)	57.9 (47.2–67.8)	17.4 (5.4–28.7)	100
Zambia	37.8 (32.6–43.1)	66.7 (55.2–76.5)	28.7 (16.4–39.9)	100
Zimbabwe	72.6 (68.3–76.4)	85 (77.5–90.3)	12.4 (4.2–19.3)	100
Middle Africa	17.2 (14.4–20.5)	33.3 (27.4–39.9)	16.1 (9.5–23.3)	100
Angola	16.3 (11.4–22.9)	34.2 (26.3–43.2)	17.8 (7.5–28.1)	100
Cameroon	25.2 (19.5–31.6)	49.3 (34.6–63)	24.2 (8.3–38.9)	100
Central African Republic	21.4 (15.8–28)	39.1 (22.7–57.4)	17.5 (0.5–36.5)	98
Chad	11.2 (8.3–14.9)	23.6 (15.9–33.5)	12.5 (3.6–22.7)	100
Congo	19.5 (9.5–34.4)	46.8 (31.2–61.9)	27 (4.7–46.9)	99
Democratic Rep. of the Congo	14.2 (9.9–19.9)	25.6 (15.1–38)	11.3 (-0.4–24.4)	97
Equatorial Guinea	19.5 (12–29.8)	35.8 (22.9–50.3)	15.8 (-0.1–32.6)	97
Gabon	27.4 (23.5–31.3)	50.4 (34.8–65.5)	23.1 (7.3–38.9)	100
Sao Tome and Principe	40.9 (32.2–50.4)	58.2 (44.3–71.8)	17 (0.5–33.3)	98
Northern Africa	67 (63.5–70)	71.7 (65.2–77.2)	4.8 (-2–10.6)	92
Algeria	70.2 (60.2–78.4)	74.8 (59.3–85.8)	4.5 (-10.7–17.8)	73
Egypt	75.8 (72.8–78)	80.1 (69.2–87.8)	4.2 (-6.5–12.5)	80
Libya	35.9 (26–48)	33.3 (21.6–51.5)	-1.8 (-17.3–14.6)	42
Morocco	68.8 (60.9–75.4)	74.1 (64.5–81.7)	5.4 (-5.4–15.4)	85
Sudan	18.3 (11.9–29.7)	32.6 (20.2–50)	14.3 (0.4–30.1)	98
Tunisia	70.7 (62.1–77.8)	74.2 (58.2–85.5)	3.7 (-12.2–16.1)	70
Southern Africa	78.9 (74.4–82.6)	81.3 (74.3–87.2)	2.4 (-5.6–9.7)	73
Botswana	72.1 (59.9–82)	81.3 (64–92.3)	8.7 (-4.4–20.4)	91
Eswatini	55.6 (47.9–63.5)	82 (70.8–90.2)	26.1 (12.8–37.7)	100
Lesotho	51.4 (45.3–57.6)	80.6 (70.9–88.2)	29 (17.9–39)	100
Namibia	69.2 (65.4–72.6)	83 (71.3–91.5)	13.6 (1.8–23.1)	99
South Africa	80.7 (75.7–84.7)	81.3 (73.3–88)	0.7 (-8.5–8.9)	56

continued

Table M. Demand for family planning met by modern methods, women of reproductive age irrespective of marital status (WRA), 2000, 2019, and 2000–2019 change, in all countries (cont'd).

Country or aggregate	Met demand (modern) 2000	Met demand (modern) 2019	Change 2000–2019	PPI 2000–2019
Western Africa	25.4 (23.4–27.5)	45.7 (41.9–49.5)	20.3 (15.9–24.7)	100
Benin	15.2 (12.2–18.7)	33.1 (26–40.4)	17.9 (10–25.9)	100
Burkina Faso	19.2 (15.6–22.9)	53.7 (45.5–62.2)	34.5 (25.4–43.8)	100
Cabo Verde	65.5 (55.4–73.3)	77.7 (59.9–90.3)	11.7 (-5.8–27)	91
Côte d'Ivoire	24.1 (19–29.5)	45.7 (38.4–53.2)	21.5 (12.4–30.5)	100
Gambia	31.1 (23.7–40.7)	31 (20.3–44.3)	-0.3 (-14.1–14.7)	49
Ghana	27.9 (24–32)	48.6 (40.8–56.4)	20.7 (11.9–29.2)	100
Guinea	17.7 (14.3–21.6)	30.6 (22–41.2)	13 (3.4–23.9)	100
Guinea-Bissau	26.1 (18.3–36.1)	57.9 (43.4–72)	31.5 (15.4–46.8)	100
Liberia	26.4 (19.1–35.5)	51.2 (41–61.7)	24.5 (11.1–37.7)	100
Mali	19.4 (16.2–23.2)	36.9 (25.9–49.7)	17.4 (5.9–30.5)	100
Mauritania	13.7 (10.6–22.9)	31 (19.9–47.2)	17.3 (5.6–31.1)	100
Niger	25.6 (20.5–31.4)	45.6 (35.9–55.7)	19.9 (8.5–31.3)	100
Nigeria	28.3 (24.2–32.6)	45.7 (38.4–53)	17.4 (8.8–25.7)	100
Senegal	22 (18.2–26.4)	51.3 (43–59.6)	29.1 (20–38.4)	100
Sierra Leone	21.1 (15.6–28.1)	52.7 (43–62.7)	31.4 (19.8–43)	100
Togo	21.3 (16.7–26.2)	43.4 (32.2–55.1)	22 (9.9–34.7)	100
Asia	77.2 (73.9–79.5)	77.3 (73.4–80.6)	0.1 (-3.6–3.9)	51
Central Asia	74.2 (71–77.1)	76.1 (67.2–82.1)	1.9 (-7.3–8.5)	67
Kazakhstan	73.2 (67.6–78)	76.9 (69.2–83)	3.6 (-5.1–11.8)	79
Kyrgyzstan	70.3 (62.2–77.4)	65.6 (51.1–77.8)	-4.8 (-21.6–10.8)	28
Tajikistan	51.8 (40.9–63.1)	47.9 (39.9–56.1)	-4 (-17.9–9.7)	29
Turkmenistan	73.5 (68.6–77.4)	74 (59.4–85)	0.6 (-14.2–11.9)	53
Uzbekistan	79.9 (74.6–84.5)	83.4 (66.4–92.7)	3.3 (-13.6–13.8)	68
Eastern Asia	90.1 (83.3–93.3)	90.1 (83.8–93.8)	-0.2 (-4.6–5)	47
China	92.7 (85.5–95.9)	92 (85.3–95.8)	-0.8 (-5.6–4.7)	35
China, Hong Kong SAR	81.1 (60.8–91)	80.8 (62–91.8)	0 (-12.6–13.8)	50
Democratic People's Rep. of Korea	69.3 (59.2–77.8)	83.1 (72.1–90.1)	13.2 (2.9–23.3)	99

continued

Table M. Demand for family planning met by modern methods, women of reproductive age irrespective of marital status (WRA), 2000, 2019, and 2000–2019 change, in all countries (cont'd).

Country or aggregate	Met demand (modern) 2000	Met demand (modern) 2019	Change 2000–2019	PPI 2000–2019
Japan	63.8 (51.2–74.6)	67.1 (50.7–80)	2.6 (-12.4–17.9)	64
Mongolia	68 (60.1–74.6)	70.9 (57.2–81.4)	2.8 (-11.3–15)	66
Republic of Korea	77.9 (62.1–87.6)	81 (64.2–90.9)	2.8 (-9.2–14.9)	70
South-eastern Asia	70.2 (67.6–72.5)	74.9 (70.7–78.7)	4.7 (0–9.2)	98
Cambodia	32.4 (29.2–35.7)	61.2 (46.9–73.3)	29 (14.4–41.2)	100
Indonesia	77.8 (73.4–81.7)	79.5 (71.3–86)	1.7 (-7.2–9.7)	65
Lao People's Dem. Republic	45.1 (37.6–53.6)	69.7 (57.8–80)	24.4 (10.8–36.5)	100
Malaysia	47.4 (32.7–62.8)	54.8 (38.7–70.2)	7.6 (-11.5–25.9)	79
Myanmar	54.4 (46.1–62.9)	76.9 (68–84.3)	22.3 (10.6–33.5)	100
Philippines	44.5 (39.3–49.5)	54.3 (45.4–62.9)	9.9 (-0.4–19.6)	97
Singapore	71.7 (57.9–82.4)	76.6 (56.3–89.7)	5 (-12.9–19.3)	73
Thailand	89.1 (84–92.3)	90.3 (83–94.3)	1.1 (-5.9–7)	63
Democratic Rep. of Timor-Leste	38.4 (31.7–45.7)	42.9 (33.6–53)	4.6 (-7.2–16.6)	78
Viet Nam	71.3 (65.2–76.5)	78.9 (68–87)	7.7 (-4.2–17.5)	90
Southern Asia	63.5 (59.3–66.8)	67.7 (60.1–74.3)	4.3 (-3.7–11.8)	86
Afghanistan	20.6 (13.1–32.9)	47.7 (37.6–59.5)	26.6 (12.1–39.8)	100
Bangladesh	61.6 (57.2–65.7)	75.1 (64.1–83.6)	13.4 (1.7–23)	99
Bhutan	55.5 (43.3–68.5)	83.4 (66.7–93)	27.1 (9–42.8)	100
India	66.2 (60.7–70.6)	68.3 (58–76.8)	2 (-8.7–11.8)	65
Iran, Islamic Republic of	69 (60–76.7)	74.2 (56.4–86.6)	5.6 (-12.5–19)	75
Maldives	48.7 (40.5–57.1)	54.3 (36.9–70.8)	5.3 (-13.7–23.8)	71
Nepal	52.4 (47.5–57.3)	62.1 (52.7–70.7)	9.7 (-0.7–19.7)	97
Pakistan	36.4 (30.9–43.7)	50.2 (42.4–59.3)	13.8 (3.7–23.4)	100
Sri Lanka	64.7 (54.8–73.3)	71.6 (57.7–82)	7 (-8.1–19.9)	83
Western Asia	45.8 (41.1–50.5)	55.3 (48.2–61.9)	9.6 (1.6–17)	99
Armenia	32.3 (26.5–38.4)	40.4 (28.7–53)	8.2 (-4.5–21.3)	89
Azerbaijan	24.2 (16.9–33)	30.1 (16.2–48.5)	6.1 (-9.3–24.4)	77
Bahrain	49.3 (31.8–67.7)	59 (33.3–80.8)	9.5 (-13.3–30.2)	79

continued

Table M. Demand for family planning met by modern methods, women of reproductive age irrespective of marital status (WRA), 2000, 2019, and 2000–2019 change, in all countries (cont'd).

Country	Met demand (modern)		Change	PPI
or aggregate	2000	2019	2000–2019	2000–2019
Georgia	34.2 (24.5–44.5)	52.1 (33.9–68.9)	17.6 (-1–35.7)	97
Iraq	44.8 (34.2–56.7)	56.4 (42.8–69.4)	11.6 (-4.6–26.8)	92
Israel	64.1 (42.3–80.8)	68.2 (42.4–85.8)	3.8 (-16–21.9)	67
Jordan	55.4 (47.7–62.3)	55.7 (45.9–64.2)	0.4 (-11.6–11.3)	52
Kuwait	60.1 (46.3–72.4)	68 (44.2–85.5)	7.8 (-13.7–25.2)	78
Lebanon	53.5 (43.2–64.6)	62.6 (44.3–79)	9.1 (-9.9–26.2)	83
Oman	36.4 (26.6–48.9)	38.8 (25.5–55.6)	2.7 (-13.5–19)	62
Qatar	55.7 (42.1–68.8)	63.1 (44.4–78.9)	7.3 (-12–25.2)	77
Saudi Arabia	40.4 (27.5–57)	42.9 (28.3–60.4)	2.3 (-12.3–17.1)	62
State of Palestine	52.8 (41.4–63.8)	64 (48.3–77.8)	11.3 (-6.4–28.1)	90
Syrian Arab Republic	51.7 (41.6–62.7)	60.9 (42.7–77.3)	9.3 (-10.5–26.6)	83
Turkey	50.4 (40.7–59.4)	61.1 (44–75.6)	10.9 (-7.8–27.4)	88
United Arab Emirates	46.5 (29.3–65.3)	59.4 (32.7–81.5)	12.2 (-10.2–32.9)	86
Yemen	21.6 (16.6–29.4)	44 (31.2–58.6)	22.3 (8.3–36.2)	100
Europe	70 (66.4–73.1)	79.3 (74.8–82.9)	9.3 (4.8–13.4)	100
Eastern Europe	61.1 (55–66.4)	74.7 (66.3–81.2)	13.6 (4.4–22)	100
Belarus	65.9 (53.3–76.4)	79.1 (68.6–86.4)	12.8 (-0.1–26.6)	97
Bulgaria	50.3 (38.8–61.1)	69.8 (49.7–84.6)	19.4 (0–35.2)	98
Czechia	73 (61.7–81.4)	85.3 (71.4–92.8)	11.9 (-2.6–24.6)	95
Hungary	76.5 (63.2–85.1)	78.9 (60.7–89.7)	2.3 (-14.7–16.3)	62
Poland	54.6 (38.7–69.5)	70 (52.1–82.9)	14.6 (-3.9–32.3)	94
Republic of Moldova	57 (46.2–66.5)	66.4 (48.3–80.4)	9.6 (-10–26.7)	84
Romania	45.9 (32.8–61)	74 (52.4–88.2)	27 (7.1–43.8)	100
Russian Federation	64.3 (53.4–73.1)	75.7 (60.3–86.3)	11.4 (-5.7–26.4)	91
Slovakia	68.7 (48.7–83.6)	79.8 (55.3–92.9)	10.1 (-8.7–27.7)	87
Ukraine	59 (46.6–69.6)	74 (57.4–85.8)	15.1 (-4.1–32.4)	94
Northern Europe	83.7 (76.8–87.6)	86.2 (75.3–91.8)	2.6 (-6.1–8.5)	75
Denmark	84.3 (68–93.4)	86.1 (63.6–96)	1.2 (-14.5–12.9)	58

continued

Table M. Demand for family planning met by modern methods, women of reproductive age irrespective of marital status (WRA), 2000, 2019, and 2000–2019 change, in all countries (cont'd).

Country or aggregate	Met demand (modern) 2000	Met demand (modern) 2019	Change 2000–2019	PPI 2000–2019
Estonia	72.2 (56.9–83.1)	79.4 (56.7–91.5)	6.5 (-10.6–20.5)	80
Finland	86.9 (75.3–91.6)	90.1 (79.2–95)	3.6 (-4.3–11.3)	84
Ireland	84.4 (71.5–90)	87.2 (70.8–94.3)	2.8 (-10.8–13.8)	69
Latvia	73.8 (55.6–85.9)	79.4 (53.7–92.8)	5 (-13.8–20.1)	73
Lithuania	57.2 (43–69.1)	68.6 (46.8–83.6)	10.8 (-10.1–29.4)	85
Norway	85.9 (72.2–92.3)	88 (71–95.6)	2 (-9–10.8)	67
Sweden	79.4 (62.2–89.2)	82.5 (58.6–94)	2.7 (-14.9–15.6)	65
United Kingdom	85.9 (77–90.8)	87.6 (72.1–94.7)	1.8 (-11.4–10.1)	63
Southern Europe	59.7 (52.2–66.4)	72.2 (63.4–79)	12.2 (3.7–20.7)	100
Albania	17.6 (11.7–25.6)	7.8 (5–11.7)	-9.8 (-18—2.9)	0
Bosnia and Herzegovina	22.5 (15–32.5)	37.7 (21.5–56.9)	15.1 (-2.5–34.5)	95
Croatia	47.4 (20.5–78.8)	66.9 (35.2–88.4)	17.1 (-4–37.8)	95
Greece	46.2 (32.3–61.3)	64.8 (40.5–83.4)	18.1 (-3.5–36.8)	95
Italy	54.5 (38–69)	71.8 (53.3–84.6)	16.5 (-2.7–35.2)	96
Malta	73.1 (54.5–86.4)	79.2 (53.6–92.4)	5.2 (-15.2–22.1)	72
Montenegro	39.6 (29.8–50.2)	46.5 (30.9–62.2)	6.7 (-10.7–24.2)	78
Portugal	73.2 (53.7–85.1)	79.8 (60.1–90.8)	6.5 (-8.6–22.6)	81
Serbia	41.9 (31.1–53.1)	53.5 (36.8–68.9)	11.5 (-7.8–29.4)	88
Slovenia	68.4 (48.5–82.9)	77.8 (54.5–91.2)	8.4 (-10–26.6)	83
Spain	77.7 (69.2–83.9)	83.6 (71.5–91.4)	5.9 (-5–15)	87
TFYR Macedonia	21.7 (11.3–39.3)	38.7 (22–58.3)	16.1 (-3.2–36.2)	95
Western Europe	84.7 (76.6–89.1)	88.2 (78.9–92.8)	3.5 (-2.6–9)	88
Austria	79.3 (66.3–87.4)	85.5 (70.2–93.1)	5.8 (-8.6–19.2)	80
Belgium	84.1 (73–90.6)	88.4 (73.9–95.1)	4 (-7–13.5)	79
France	88.1 (78.5–92.2)	89.5 (75.8–95.4)	1.3 (-9–9.2)	62
Germany	82.8 (70.3–89.9)	88.9 (74.1–95.3)	5.7 (-5.7–16.6)	85
Netherlands	86.2 (75.4–91.8)	87 (71.7–94.1)	0.6 (-10.9–9.8)	55
Switzerland	87.9 (77.4–93.2)	89.3 (77.2–94.9)	1.2 (-9.3–11)	61

continued

Table M. Demand for family planning met by modern methods, women of reproductive age irrespective of marital status (WRA), 2000, 2019, and 2000–2019 change, in all countries (cont'd).

Country or aggregate	Met demand (modern) 2000	Met demand (modern) 2019	Change 2000–2019	PPI 2000–2019
Latin America and the Caribbean	76.2 (73.5–78.6)	82.6 (78.8–85.7)	6.3 (2.1–10.2)	100
Caribbean	72.7 (68.9–75.8)	74.4 (70–78.2)	1.8 (-3.3–6.7)	76
Antigua and Barbuda	70.9 (50.7–85.2)	77.1 (53.7–91.7)	5.3 (-11.5–21.8)	75
Bahamas	75.4 (56.2–87.6)	79.1 (56.6–92.5)	3.2 (-13.2–18.9)	66
Barbados	71.8 (56.8–83.1)	75 (59.5–86)	3.1 (-13.5–19.5)	64
Cuba	84.2 (75.8–90.2)	89.3 (79.6–94.7)	4.9 (-5.4–14.6)	84
Dominican Republic	79.4 (76.2–82.2)	83.8 (74.8–90.1)	4.3 (-5–11.5)	84
Grenada	67 (47.4–82.6)	74.9 (50.9–90.3)	6.7 (-10–23.4)	80
Guadeloupe	62.5 (40.7–80.8)	72.2 (46.4–89.3)	8.3 (-8.1–24.8)	84
Haiti	34.7 (31.6–37.7)	46 (38.7–53.5)	11.4 (3.4–19.5)	100
Jamaica	76.3 (65–83.3)	76.5 (59.4–87.8)	-0.1 (-14.9–12.9)	50
Martinique	65.4 (43.9–82.1)	73.7 (48.4–89.7)	7 (-8.9–22.8)	82
Puerto Rico	79.1 (68.4–86.6)	81.6 (65–91.5)	2.2 (-11.9–13.1)	64
St. Lucia	69.4 (54.2–81.3)	75 (58.6–86.6)	5.4 (-11.1–21.9)	75
St. Vincent and the Grenadines	74.3 (53.6–87.5)	79 (55–92)	4 (-12.8–20.4)	70
Trinidad and Tobago	56.2 (46.2–65.4)	63.7 (46.6–77.7)	7.4 (-9.9–22.9)	80
United States Virgin Islands	75.3 (58.7–86.3)	79 (59.1–91.8)	3.1 (-11.9–17.3)	67
Central America	74.6 (69.5–78.8)	80 (73–85.2)	5.4 (-2.4–12.3)	92
Belize	64.6 (53.5–74.2)	71.9 (59.3–81.8)	7.2 (-7.3–21.2)	84
Costa Rica	82.5 (74.4–88)	86.2 (76.2–92.3)	3.5 (-5.5–11.9)	79
El Salvador	75.7 (67.3–82.2)	81.1 (70.5–88.2)	5.3 (-5.8–15.4)	84
Guatemala	50.5 (44.6–56.7)	69.2 (57.7–78.5)	18.6 (6.1–29.7)	100
Honduras	66.1 (57.4–73.7)	77.9 (66.4–86.2)	11.7 (-1.7–23.5)	96
Mexico	76.6 (70–82)	80.9 (71.6–87.4)	4.2 (-5.9–13.1)	80
Nicaragua	77.6 (74.3–80.4)	90.5 (84–94.2)	12.7 (5.8–17.9)	100
Panama	70.5 (57.4–81.1)	75.6 (62.4–85.6)	4.9 (-10.6–20.4)	74
South America	77.3 (73.6–80.2)	84.5 (79.4–88.3)	7.2 (1.7–12.1)	99
Argentina	77.7 (66.6–85)	83.5 (71.1–91.4)	5.5 (-7.1–17.8)	82

continued

Table M. Demand for family planning met by modern methods, women of reproductive age irrespective of marital status (WRA), 2000, 2019, and 2000–2019 change, in all countries (cont'd).

Country or aggregate	Met demand (modern) 2000	2019	Change 2000–2019	PPI 2000–2019
Bolivia, Plurinational State of	37.7 (31.8–43.6)	56.9 (44–68.5)	19.2 (5.4–32.2)	100
Brazil	82.7 (76.7–87.3)	88.5 (79.5–94.2)	5.7 (-3.9–13.6)	89
Chile	72.9 (59.4–83.4)	85.3 (74.4–92)	11.8 (-0.4–25.7)	97
Colombia	75.1 (71.1–78.3)	86.6 (80.2–91.1)	11.5 (4.4–17.5)	100
Ecuador	69 (60.8–75.9)	81 (67.9–89.5)	12.1 (-1.7–23)	96
Guyana	53.7 (44.9–62.9)	61.8 (47.5–74.6)	7.8 (-8.4–23.5)	83
Paraguay	68 (59.2–75.4)	81.8 (72.5–88.4)	13.5 (2.7–23.9)	99
Peru	57.5 (51.8–62.6)	67.9 (57.5–76.2)	10.5 (-1.1–20.3)	96
Suriname	66.5 (55.3–76.4)	77.7 (61–88.8)	10.7 (-6.2–25.9)	90
Uruguay	84.4 (73.8–90.9)	86.9 (75.7–93.3)	2.3 (-7.7–12.1)	70
Venezuela, Bolivarian Republic of	74.9 (65.9–82)	82.1 (67.6–91)	7 (-5.8–17.3)	87
Northern America	81.4 (70.8–88.1)	83.4 (71.3–90.1)	1.8 (-7.2–10.4)	67
Canada	89.4 (80.9–93.5)	90.7 (75.6–96.4)	1.2 (-10.8–8.4)	60
United States of America	80.5 (68.8–87.9)	82.6 (69.4–89.9)	1.9 (-8–11.5)	66
Oceania	76.5 (67.7–82)	77.1 (66.9–83.6)	0.6 (-7.6–8.6)	56
Australia and New Zealand	83.7 (73.1–89.3)	84.5 (72.2–91.2)	0.8 (-8.2–9.3)	58
Australia	83.5 (71.9–89.8)	84.6 (70.7–91.7)	0.9 (-9.6–10.6)	57
New Zealand	84.9 (70.6–92.3)	86.1 (63.5–95.4)	0.8 (-15.3–11.5)	55
Melanesia	41.4 (31.7–51.2)	50.8 (33.7–67.1)	9 (-8.7–26.8)	84
Fiji	58.4 (33.1–79.2)	65.2 (33.8–86.2)	5.8 (-15–24.5)	73
Papua New Guinea	38 (27.5–49.3)	48.9 (28.5–68.4)	10.4 (-11.5–32)	82
Solomon Islands	45.2 (29.7–60.8)	47.5 (33.8–60.7)	2.2 (-17.6–21.5)	59
Vanuatu	47.6 (34.6–60.3)	60.8 (43.1–75.2)	12.8 (-5.9–30.4)	91
Micronesia	61 (48.8–70.3)	63.7 (49.9–75.2)	2.7 (-8.6–13.5)	68
Guam	68.4 (47.9–81.1)	73.6 (49.5–88.3)	4.9 (-13.7–20.9)	71
Kiribati	48.9 (35–61.8)	45.3 (27.4–63.7)	-3.5 (-22.8–16.6)	37
Polynesia	40.2 (32.6–48.7)	45.9 (36.3–55.8)	5.6 (-7.1–18.2)	81
Samoa	34.5 (25.7–44.7)	39.1 (26.7–53.2)	4.5 (-12.5–21.9)	70

continued

Table M. Demand for family planning met by modern methods, women of reproductive age irrespective of marital status (WRA), 2000, 2019, and 2000–2019 change, in all countries (cont'd).

Country	Met demand (modern)		Change	PPI
or aggregate	2000	2019	2000–2019	2000–2019
Tonga	43 (25.7–62.5)	50.5 (33.7–67)	7.2 (-16.4–30)	73

[a] The designation "more developed" and "less developed" regions are intended for statistical purposes and do not express a judgment about the stage reached by a particular country or area in the development process.

[b] More developed regions comprise Europe, Northern America, Australia/New Zealand and Japan.

[c] Less developed regions comprise all regions of Africa, Asia (except Japan), Latin America and the Caribbean plus Melanesia, Micronesia and Polynesia.

[d] The group of least developed countries includes 47 countries: 32 in Sub-Saharan Africa, 2 in Northern Africa and Western Asia, 4 in Central and Southern Asia, 4 in Eastern and South- Eastern Asia, 1 in Latin America and the Caribbean, 4 in Oceania. Further information is available at http://unohrlls.org/about-ldcs/.

[e] Other less developed countries comprise the less developed regions excluding the least developed countries.

6 GLOSSARY

CPS Contraceptive Prevalence Survey.

DHS Demographic and Health Survey.

FFS Fertility and Family Surveys.
FP2020 Family Planning 2020.

GGS Generations and Gender Survey.

iDHS Integrated Demographic and Health Survey.
IPUMS Integrated Public Use Microdata Series.
IUD intra-uterine device.

LAM lactational amenorrhoea method.

MAE median absolute error.
MCMC Markov chain Monte Carlo.
ME median error.
MICS Multiple Indicator Cluster Survey.
MWRA married or in a union women of reproductive age.

PMA Performance Monitoring and Accountability 2020 survey.
PPI posterior probability of an increase.
PW partnered women.

RHS Reproductive Health Survey.

SDG sustainable development goal.
SO sterilization only.

UN Population Division United Nations, Department of Economic and Social Affairs, Population Division.
UWRA unmarried and not in a union women of reproductive age.

WFS World Fertility Survey.
WRA women of reproductive age irrespective of marital status.
WVS World Values Survey.

7 REFERENCES

Alkema, L., Kantorova, V., Menozzi, C. and Biddlecom, A. (2013). National, Regional, and Global Rates and Trends in Contraceptive Prevalence and Unmet Need for Family Planning Between 1990 and 2015: A Systematic and Comprehensive Analysis. *The Lancet* 381 (9878), pp. 1642–1652.

Bijak, J. and Bryant, J. (2016). Bayesian demography 250 years after Bayes. *Population Studies* 70 (1), pp. 1–19.

Boyle, E., King, M. and Sobek, M. (2017). *IPUMS-Demographic and Health Surveys: Version 4.1.* URL: https://www.idhsdata.org/idhs/citation.shtml (visited on 24/07/2017).

Bradley, S., Croft, T., Fishel, J. and Westoff, C. (2012). *Revising Unmet Need for Family Planning.* DHS Analytical Studies 25. Calverton, Maryland, USA: ICF International.

Cahill, N., Sonneveldt, E., Stover, J., Weinberger, M., Williamson, J., Wei, C., Brown, W. and Alkema, L. (2017). Modern Contraceptive Use, Unmet Need, and Demand Satisfied Among Women of Reproductive Age Who Are Married or In A Union in the Focus Countries of the Family Planning 2020 Initiative: A Systematic Analysis Using the Family Planning Estimation Tool. *The Lancet* 391 (10123), pp. 870–882. URL: http://www.thelancet.com/journals/lancet/article/PIIS0140-6736(17)33104-5/abstract (visited on 08/12/2017).

DHS Program, Demographic and Health Surveys (2019). *Unmet Need for Family Planning.* URL: https://www.dhsprogram.com/topics/Unmet-Need.cfm (visited on 17/04/2019).

FP2020 (2016). *Family Planning 2020.* URL: http://www.familyplanning2020.org/ (visited on 23/09/2016).

Gelman, A. and Rubin, D. (1992). Inference From Iterative Simulation Using Multiple Sequences. *Statistical Science* (7), pp. 457–511.

Gelman, A., Carlin, J. B., Stern, H. S., Dunson, D. B., Vehtari, A. and Rubin, D. B. (2013). *Bayesian Data Analysis, Third Edition.* CRC Press.

Inglehart, R., Haerpfer, C., Moreno, A., Welze, C., Kizilova, K., Diez-Medrano, J., Lagos, M., Norris, P., Ponarin, E. and Puranen, B. (2013). *World Values Survey: Round 6.* URL: http://www.worldvaluessurvey.org/WVSDocumentationWV6.jsp (visited on 14/07/2017).

Kantorová, V., Dasgupta, A. N. Z. and Ueffing, P. (2017). 'Understanding Family Planning Indicators Amongst Unmarried Women: Theory and Measurement'. In: XXVIII International Population Conference. Cape Town, South Africa: International Union for the Scientific Study of Population. URL: https://iussp.confex.com/iussp/ipc2017/meetingapp.cgi/Paper/7363 (visited on 17/05/2018).

Li, J., Temmerman, M., Chen, Q., Xu, J., Hu, L. and Zhang, W. (2013). A Review of Contraceptive Practices Among Married and Unmarried Women in China From 1982 to 2010. *The European Journal of Contraception and Reproductive Health Care* 18 (3), pp. 148–158.

Li, R. M. and Newcomer, S. F. (1996). The Exclusion of Never-Married Women From Chinese Fertility Surveys. *Studies in Family Planning* 27 (3), pp. 148–154.

Lindley, D. V. and Smith, A. F. M. (1972). Bayes Estimates for the Linear Model. *Journal of the Royal Statistical Society, Series B* (34), pp. 1–41.

Pew Research Center (2012). *The Global Religious Landscape: A Report on the Size and Distribution of the World's Major Religious Groups.* URL: http://www.pewforum.org/2012/12/18/global-religious-landscape-exec/ (visited on 16/08/2017).

Pew Research Center (2014). *Global Attitudes Survey Spring 2013*. URL: http://www.pewglobal.org/2014/04/15/global-morality/table/premarital-sex/ (visited on 14/07/2017).

Plummer, M. (2003). 'JAGS: A Program for Analysis of Bayesian Graphical Models Using Gibbs Sampling'. In: *Proceedings of the 3rd International Workshop on Distributed Statistical Computing (DSC 2003)* (Vienna, Austria, 20th–22nd Mar. 2003).

Plummer, M. (2015). *JAGS: Just Another Gibbs Sampler v. 4.2.0*. URL: http://mcmc-jags.sourceforge.net/.

Plummer, M., Best, N., Cowles, K. and Vines, K. (2006). CODA: Convergence Diagnosis and Output Analysis for MCMC. *R News* 6 (1), pp. 7–11. URL: https://journal.r-project.org/archive/.

Plummer, M., Stukalov, A. and Denwood, M. (2016). *rjags v. 4-6*. URL: http://mcmc-jags.sourceforge.net.

R Core Team (2018). *R: A Language and Environment for Statistical Computing*. R Foundation for Statistical Computing. Vienna, Austria. URL: https://www.R-project.org/.

Raftery, A. E. and Lewis, S. M. (1992a). 'How Many Iterations in the Gibbs Sampler?' In: *Bayesian Statistics 4. Proceedings of the Fourth Valencia International Meeting*. Ed. by Bernardo, J. M., Berger, J. O., Dawid, A. P. and Smith, A. F. M. Oxford, England: Oxford University Press.

Raftery, A. E. and Lewis, S. M. (1992b). One Long Run With Diagnostics: Implementation Strategies for Markov Chain Monte Carlo. *Statistical Science* (7), pp. 493–497.

Raftery, A. E. and Lewis, S. M. (1996). 'Implementing MCMC'. In: *Markov Chain Monte Carlo in Practice*. Ed. by Gilks, W. R., Spiegelhalter, D. J. and Richardson, S. London: Chapman and Hall, pp. 115–130.

Stover, J. and Sonneveldt, E. (2017). Progress Toward the Goals of FP2020. *Studies in Family Planning* 48 (1), pp. 83–88. (Visited on 25/07/2017).

Su, Y.-S. and Yajima, M. (2015). *R2jags v. 0.5-7*. URL: https://CRAN.R-project.org/package=R2jags.

Ueffing, P., Dasgupta, A. N. Z., Kantorová, V. and Wheldon, M. C. (2017). 'Patterns and Trends in Sexual Activity by Marital Status and Age: A comparative Perspective'. In: XXVIII International Population Conference. Cape Town, South Africa: International Union for the Scientific Study of Population. URL: https://iussp.confex.com/iussp/ipc2017/meetingapp.cgi/Paper/7233 (visited on 17/05/2018).

United Nations (2019). *Standard Country or Area Codes for Statistical Use (M49)*. New York: United Nations. URL: https://unstats.un.org/unsd/methodology/m49/ (visited on 25/03/2019).

United Nations, Department of Economic and Social Affairs, Population Division (2017). *World Marriage Data 2017*. New York: United Nations. URL: http://www.un.org/en/development/desa/population/theme/marriage-unions/WMD2017.shtml (visited on 20/04/2018).

United Nations, Department of Economic and Social Affairs, Population Division (2018a). *Estimates and Projections of Women of Reproductive Age Who Are Married or in a Union: 2018 Revision*. New York: United Nations. URL: http://www.un.org/en/development/desa/population/theme/marriage-unions/marriage_estimates.shtml (visited on 20/04/2018).

United Nations, Department of Economic and Social Affairs, Population Division (2018b). *World Contraceptive Use 2018*. New York: United Nations. URL: http://www.un.org/en/development/desa/population/publications/dataset/contraception/wcu2018/UNPD_WCU2018_Country_Data_Survey-Based.xlsx (visited on 20/04/2018).

United Nations, Department of Economic and Social Affairs, Population Division (2019). *World Contraceptive Use 2019*. New York: United Nations. URL: https://www.un.org/en/development/desa/population/publications/dataset/contraception/wcu2019.asp (visited on 17/04/2019).

Wheldon, M. C., Kantorová, V., Ueffing, P. and Dasgupta, A. N. Z. (2019). *Methods for Estimating and Projecting Key Family Planning Indicators Among All Women of Reproductive Age*. Technical Paper 2018/2. New York: United Nations, Department of Economic and Social Affairs, Population Division. URL: https://www.un.org/en/development/desa/population/publications/pdf/technical/TP2018-2.pdf (visited on 08/01/2019).

World Bank (2019). *World Bank Country and Lending Groups (2020 fiscal year)*. URL: https://datahelpdesk.worldbank.org/knowledgebase/articles/906519-world-bank-country-and-lending-groups (visited on 08/11/2019).
