# Supplementary material for: Estimating progress towards meeting women’s contraceptive needs in 185 countries: A Bayesian hierarchical modelling study
Source: PLoS Med. 2020 Feb 18;17(2):e1003026. doi: 10.1371/journal.pmed.1003026 (PMC7028249; doi:10.1371/journal.pmed.1003026)
Supplement: S1 Checklist — (DOCX) [file pmed.1003026.s013.docx]

**S1 GATHER Checklist**

PMEDICINE-D-19-03179

| **Item number** | **Checklist item** |
| --- | --- |
| **Objectives and funding** | |
| 1 | Define the indicator(s), populations (including age, sex, and geographic entities), and time period(s) for which estimates were made.  The indicators, population, and time period for which estimates were made are defined in Subsections ‘Definitions’ and ‘Indicators used’ of Section ‘Methods’ in the main manuscript. Further detail is in Subsection 2.2 of S1 Appendix. |
| 2 | List the funding sources for the work.  Listed with the article online. |
| **Data inputs** | |
| *For all data inputs from multiple sources that are synthesised as part of the study:* | |
| 3 | Describe how the data were identified and how the data were accessed.  Described in Subsection ‘Data compilation’ of Section ‘Methods’ in the main article and Subsection 2.3 of S1 Appendix. |
| 4 | Specify the inclusion and exclusion criteria. Identify all ad-hoc exclusions.  Described in Subsection ‘Data compilation’ of Section ‘Methods’ in the main article and Subsection 2.3 of S1 Appendix. |
| 5 | Provide information about all included data sources and their main characteristics. For each data source used, report reference information or contact name/institution, population represented, data collection method, year(s) of data collection, sex and age range, diagnostic criteria or measurement method, and sample size, as relevant.  A narrative description of the data sources is given in Subsection ‘Data compilation’ of Section ‘Methods’ in the main article and Subsection 2.3 of S1 Appendix.  The input data themselves are included in S1 Data and S2 Data. Meta-data are contained in the same table and in the online open-access publication: United Nations, Department of Economic and Social Affairs, Population Division. World Contraceptive Use 2019. New York: 2019. https://www.un.org/en/development/desa/population/publications/dataset/contraception/wcu2019.asp |
| 6 | Identify and describe any categories of input data that have potentially important biases (eg, based on characteristics listed in item 5).  Biases and misclassifications in the input data are described in the ‘Statistical model’ Subsection of Section ‘Methods’ in the main manuscript, and in more detail in S1 Appendix Subsections 3.5.7–3.5.9. |
| *For data inputs that contribute to the analysis but were not synthesised as part of the study:* | |
| 7 | Describe and give sources for any other data inputs.  These are data on the national population sizes of women of reproductive age and the proportion of women married/in-union. These are cited in the main article. |
| *For all data inputs:* | |
| 8 | Provide all data inputs in a file format from which data can be efficiently extracted (eg, a spreadsheet rather than a PDF), including all relevant meta-data listed in item 5. For any data inputs that cannot be shared because of ethical or legal reasons, such as third-party ownership, provide a contact name or the name of the institution that retains the right to the data.  Data inputs are included as comma separated variable (csv) files in S1 Data and S2 Data. |
| **Data analysis** | |
| 9 | Provide a conceptual overview of the data analysis method. A diagram may be helpful.  An overview diagram is provided in S1 Appendix, Section 1. |
| 10 | Provide a detailed description of all steps of the analysis, including mathematical formulae. This description should cover, as relevant, data cleaning, data pre-processing, data adjustments and weighting of data sources, and mathematical or statistical model(s).  A full, mathematical description of the statistical model, including bias adjustments and weighting (e.g., to produce aggregates) is provided in S1 Appendix, Section ‘Methods’. |
| 11 | Describe how candidate models were evaluated and how the final model(s) were selected.  Provided in S1 Appendix Section 3.8. |
| 12 | Provide the results of an evaluation of model performance, if done, as well as the results of any relevant sensitivity analysis.  Provided in S1 Appendix Section 4.3. |
| 13 | Describe methods of calculating uncertainty of the estimates. State which sources of uncertainty were, and were not, accounted for in the uncertainty analysis.  A full description of the statistical model and the uncertainties accounted for is given in S1 Appendix, Section 3. |
| 14 | State how analytical or statistical source code used to generate estimates can be accessed.  Noted in Subsection ‘Statistical model’ of Section ‘Methods’ in the main article. |
| **Results and discussion** | |
| 15 | Provide published estimates in a file format from which data can be efficiently extracted.  The published estimates are provided in comma separated variable (csv) files S1 Results–S7 Results. |
| 16 | Report a quantitative measure of the uncertainty of the estimates (eg, uncertainty intervals).  We report 95 percent (Bayesian) uncertainty intervals throughout the article and include them in the published results S2 Table Estimates and Projections. |
| 17 | Interpret results in light of existing evidence. If updating a previous set of estimates, describe the reasons for changes in estimates.  Provided in the ‘Discussion’ section of the main article. |
| 18 | Discuss limitations of the estimates. Include a discussion of any modelling assumptions or data limitations that affect interpretation of the estimates.  Provided in the ‘Discussion’ section of the main article. |
