# Supplementary material for: Estimating progress towards meeting women’s contraceptive needs in 185 countries: A Bayesian hierarchical modelling study
Source: PLoS Med. 2020 Feb 18;17(2):e1003026. doi: 10.1371/journal.pmed.1003026 (PMC7028249; doi:10.1371/journal.pmed.1003026)

Estimating Progress Towards Meeting Women's Contraceptive Needs in 185 Countries: A Bayesian Hierarchical Modelling Study

S2 APPENDIX
(Supplementary Figures)

Vladimíra Kantorová* Mark C. Wheldon Philipp Ueffing Aisha N. Z. Dasgupta

United Nations Population Division, New York, NY†

23rd January 2020

*Corresponding author. Vladimíra Kantorová, Population Division, Department of Economic and Social Affairs, United Nations, kantorova@un.org

†The views and opinions expressed in this paper are those of the authors and do not necessarily represent those of the United Nations. This paper has not been formally edited and cleared by the United Nations.

CONTENTS

1 Unmarried women **2**
1.1 Countries and areas . . . 2
1.2 Country aggregates . . . 188

2 Married women **221**
2.1 Countries and areas . . . 221
2.2 Country aggregates . . . 407

3 All women **440**
3.1 Countries and areas . . . 440
3.2 Country aggregates . . . 626

These figures contain a systematic and comprehensive set of annual, model-based estimates and projections, and underlying survey-based observations, for a collection of family planning indicators, including contraceptive prevalence, the unmet need for family planning, and the demand for family planning satisfied by use of contraception (any method or modern methods alone).

The results pertain to unmarried and not in a union women of reproductive age (UWRA), married or in a union women of reproductive age (MWRA) and women of reproductive age irrespective of marital status (WRA) and cover the period from 1970 to 2030. Estimates based on medians, as well as 80 per cent uncertainty intervals (represented by lines) and 95 per cent uncertainty intervals (represented by shaded areas), are provided for 185 countries or areas. The results are based on data available as of February 2019.

Note: The designations employed and the material presented in this publication do not imply the expression of any opinion whatsoever on the part of the Secretariat of the United Nations concerning the legal status of any country, territory or area or of its authorities, or concerning the delimitation of its frontiers or boundaries. The term “country” as used in this publication also refers, as appropriate, to territories or areas. Countries or areas listed individually are only those with 90,000 inhabitants or more in 2017; the rest are included in the aggregates but are not listed separately.

1 Unmarried and not in a union women of reproductive age

1.1 Countries and areas

Afghanistan (Southern Asia, SA Group 0) −−− Unmarried / Not In−Union

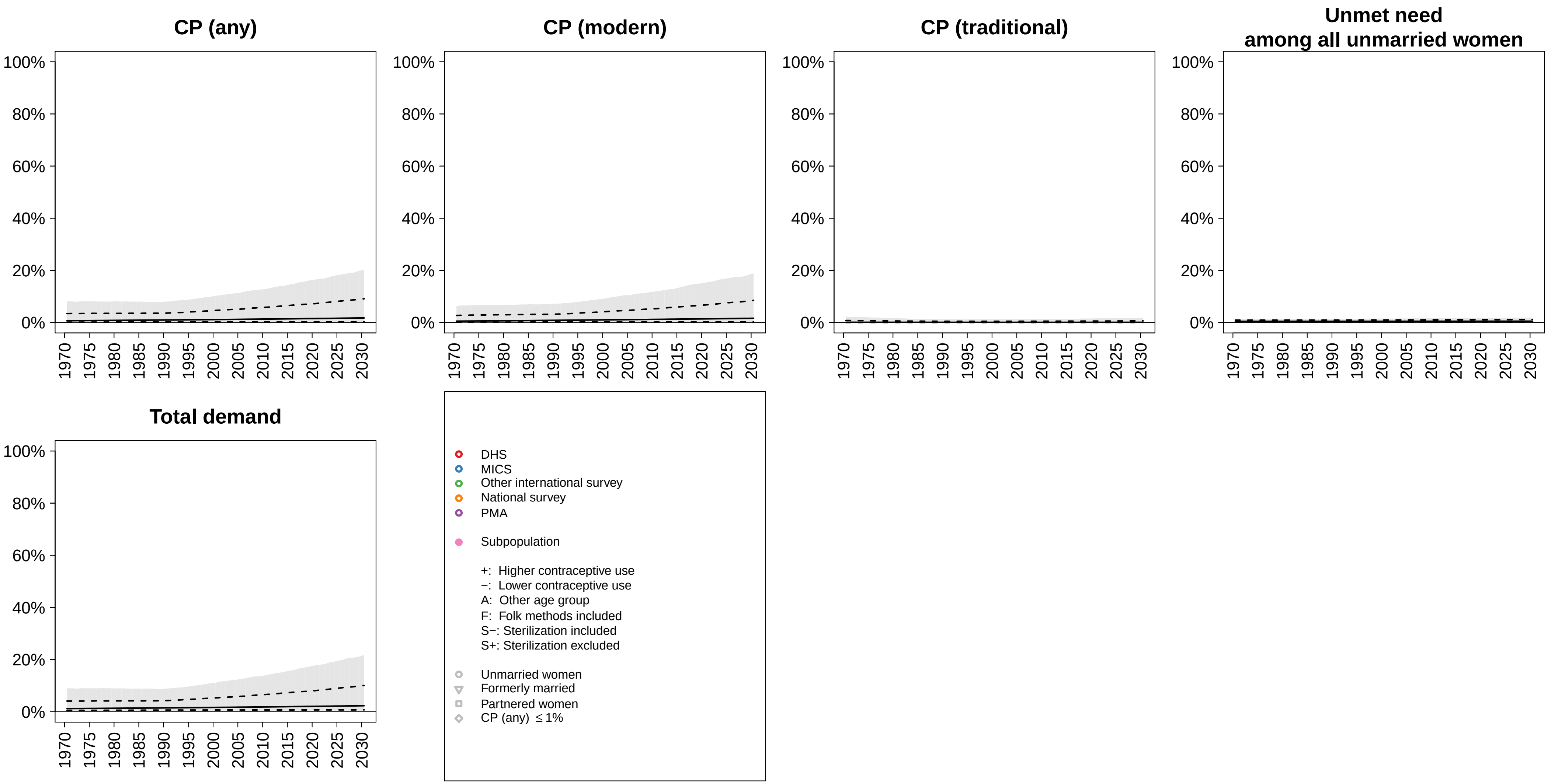

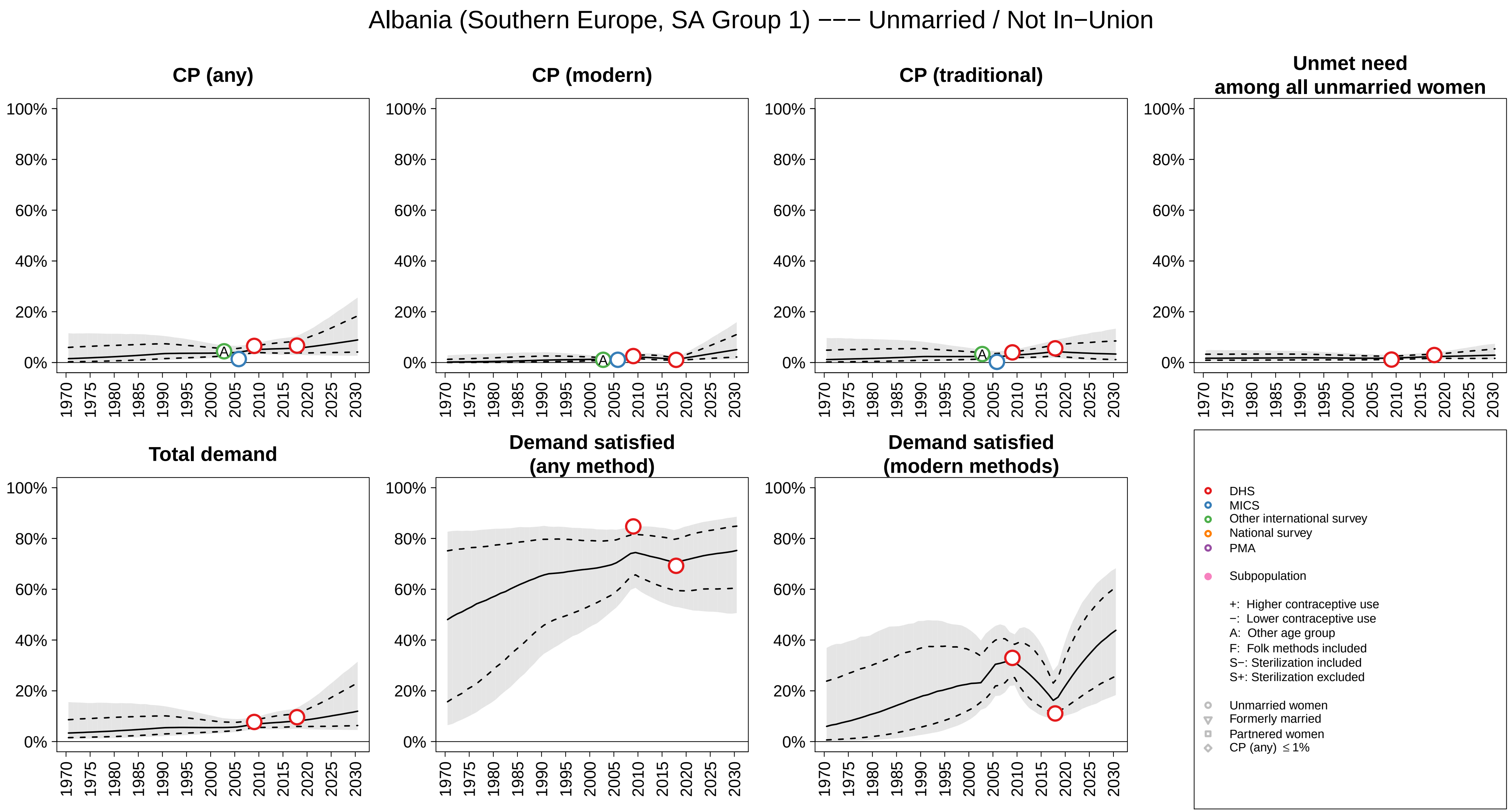
Albania (Southern Europe, SA Group 1) −−− Unmarried / Not In−Union
CP (any)
CP (modern)
CP (traditional)
Unmet need among all unmarried women
Total demand
Demand satisfied (any method)
Demand satisfied (modern methods)
DHS
MICS
Other international survey
National survey
PMA
Subpopulation
+: Higher contraceptive use
−: Lower contraceptive use
A: Other age group
F: Folk methods included
S−: Sterilization included
S+: Sterilization excluded
Unmarried women
Formerly married
Partnered women
CP (any) ≤ 1%

Algeria (Northern Africa, SA Group 0) −−− Unmarried / Not In−Union

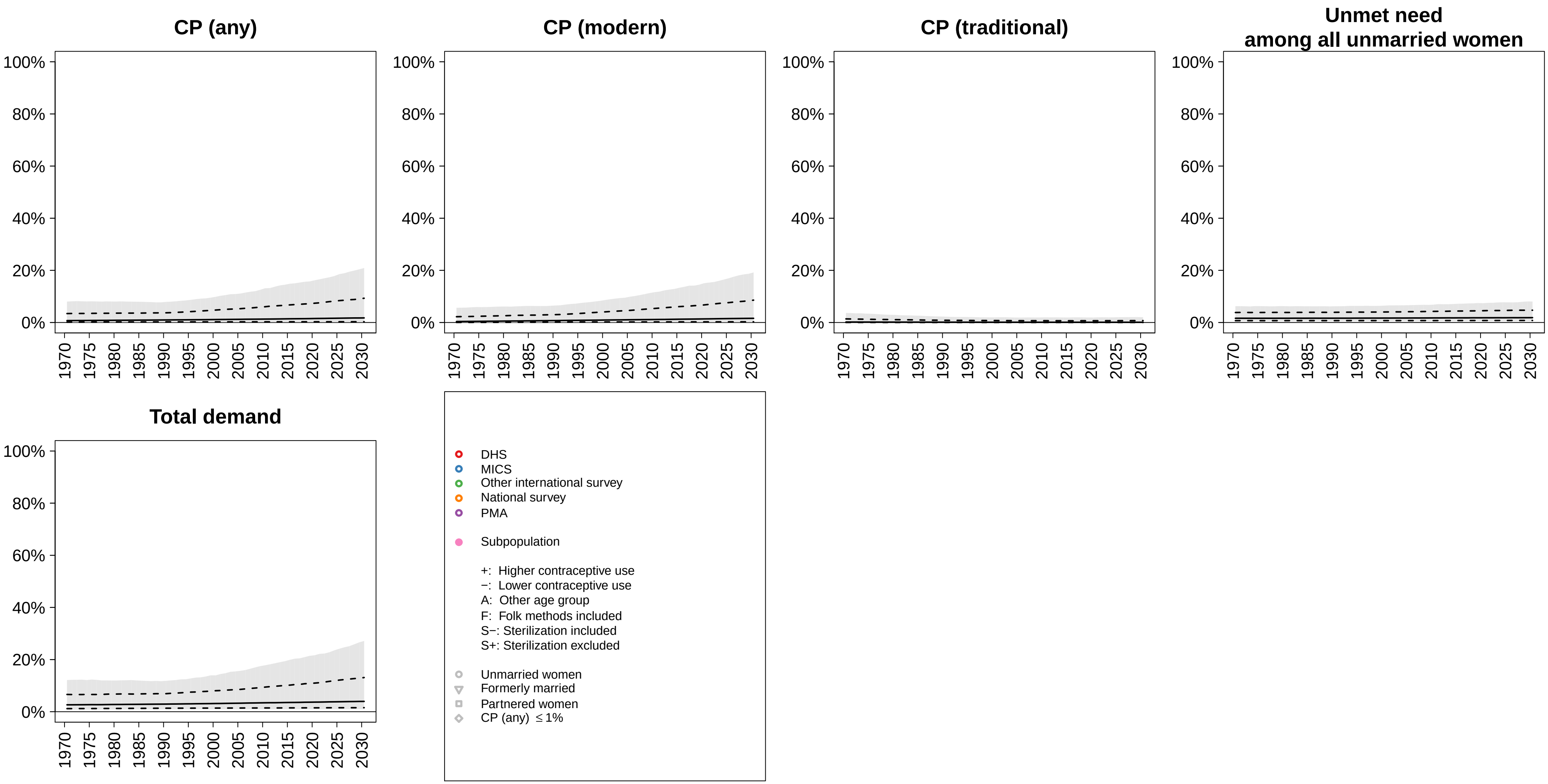

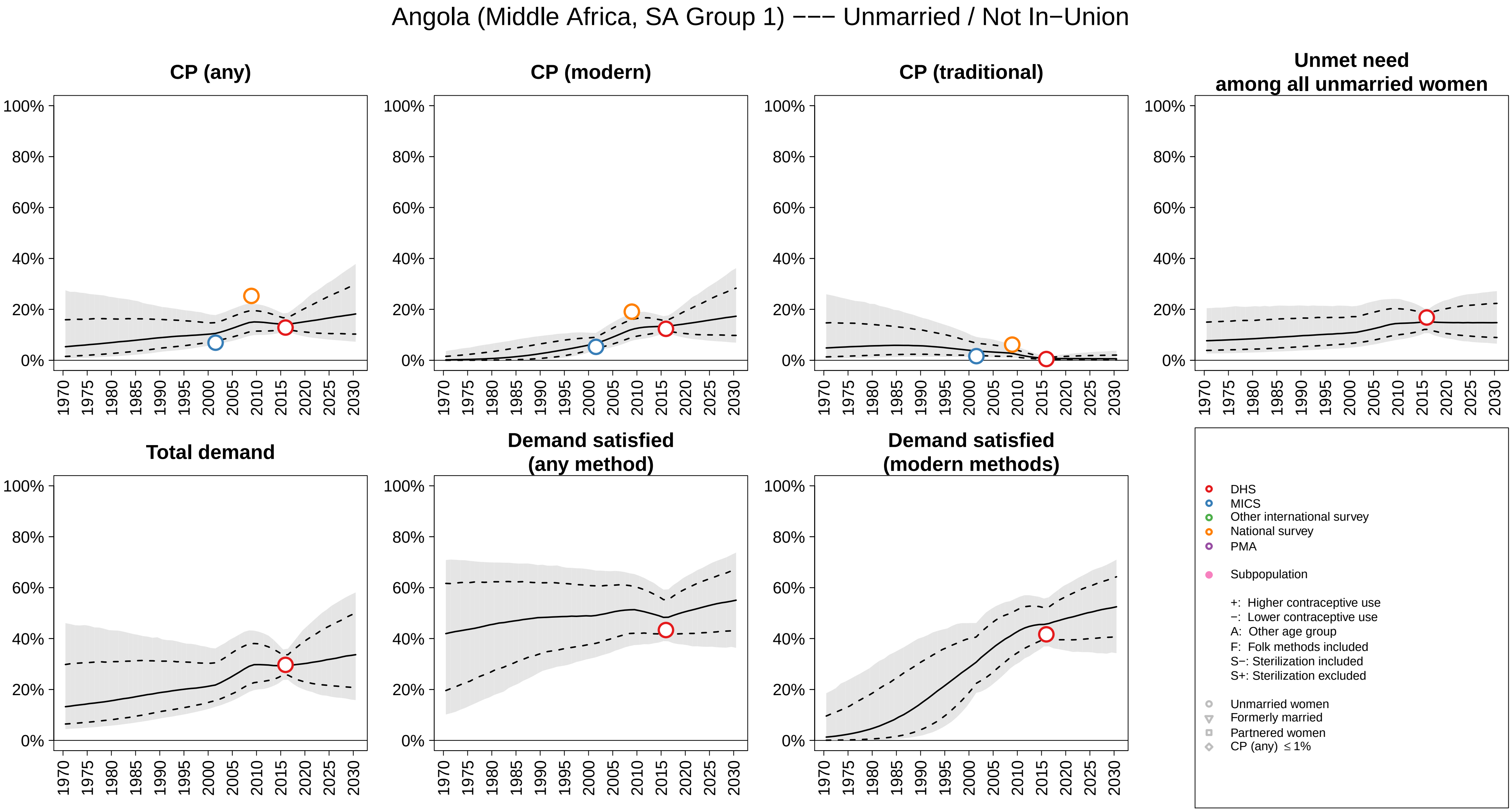
Angola (Middle Africa, SA Group 1) −−− Unmarried / Not In−Union
CP (any)
CP (modern)
CP (traditional)
Unmet need among all unmarried women
Total demand
Demand satisfied (any method)
Demand satisfied (modern methods)
0%
20%
40%
60%
80%
100%
1970
1975
1980
1985
1990
1995
2000
2005
2010
2015
2020
2025
2030
DHS
MICS
Other international survey
National survey
PMA
Subpopulation
+: Higher contraceptive use
−: Lower contraceptive use
A: Other age group
F: Folk methods included
S−: Sterilization included
S+: Sterilization excluded
Unmarried women
Formerly married
Partnered women
CP (any) ≤ 1%

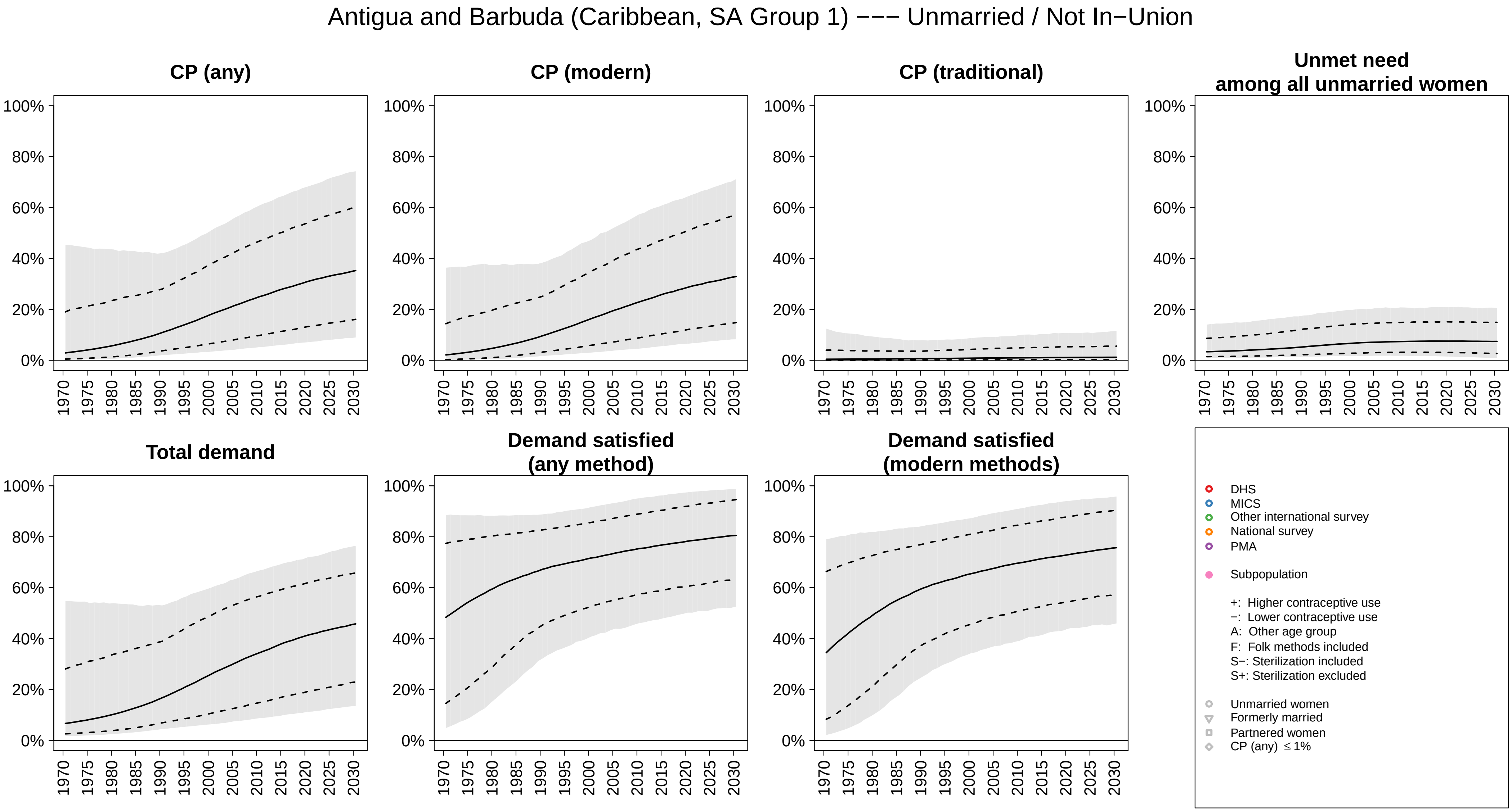

Antigua and Barbuda (Caribbean, SA Group 1) −−− Unmarried / Not In−Union
CP (any)
CP (modern)
CP (traditional)
Unmet need among all unmarried women
Total demand
Demand satisfied (any method)
Demand satisfied (modern methods)
0%
20%
40%
60%
80%
100%
1970
1975
1980
1985
1990
1995
2000
2005
2010
2015
2020
2025
2030
DHS
MICS
Other international survey
National survey
PMA
Subpopulation
+: Higher contraceptive use
−: Lower contraceptive use
A: Other age group
F: Folk methods included
S−: Sterilization included
S+: Sterilization excluded
Unmarried women
Formerly married
Partnered women
CP (any) ≤ 1%

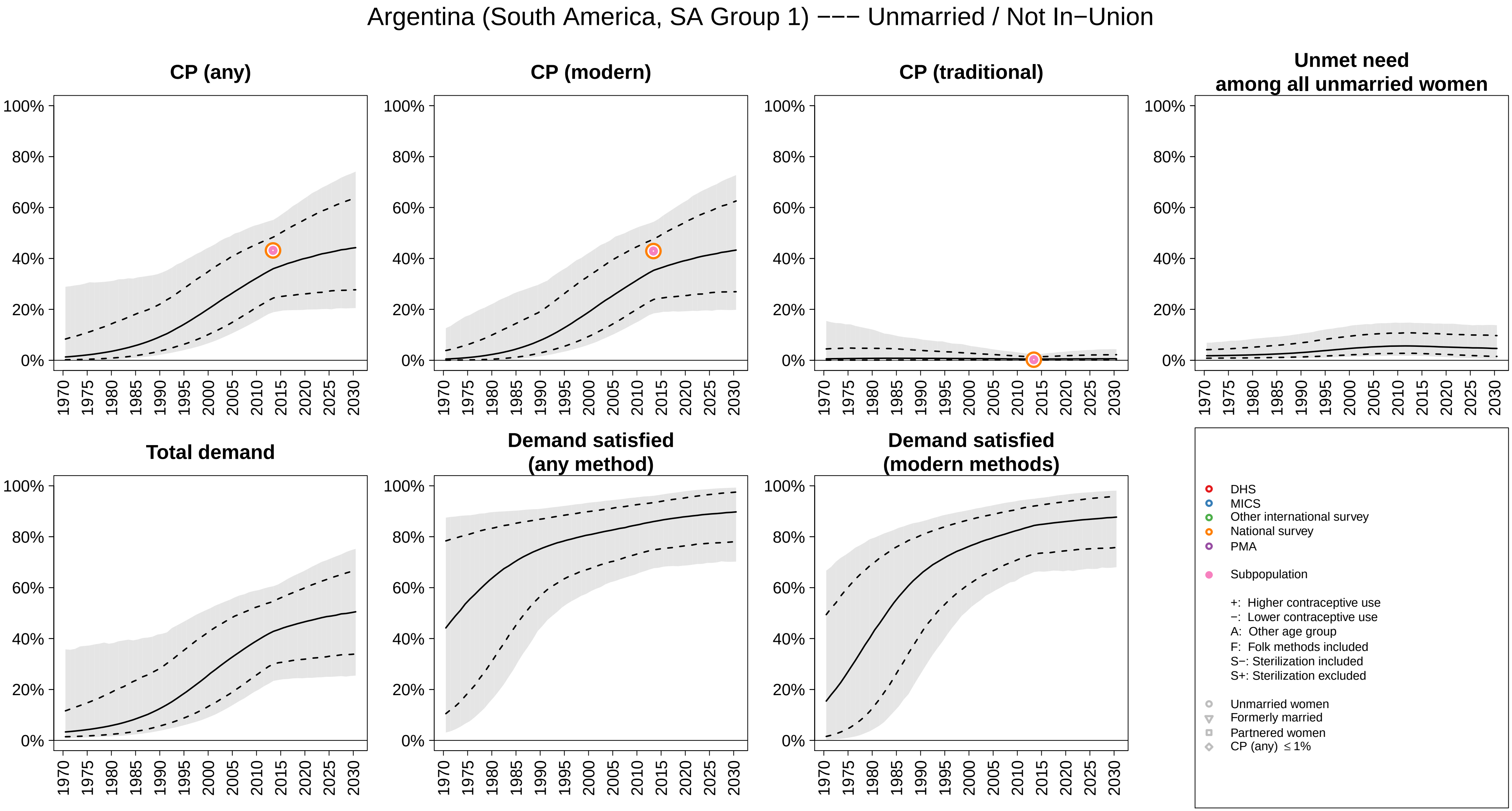
Argentina (South America, SA Group 1) −−− Unmarried / Not In−Union
CP (any)
CP (modern)
CP (traditional)
Unmet need among all unmarried women
Total demand
Demand satisfied (any method)
Demand satisfied (modern methods)
0%
20%
40%
60%
80%
100%
1970
1975
1980
1985
1990
1995
2000
2005
2010
2015
2020
2025
2030
DHS
MICS
Other international survey
National survey
PMA
Subpopulation
+: Higher contraceptive use
−: Lower contraceptive use
A: Other age group
F: Folk methods included
S−: Sterilization included
S+: Sterilization excluded
Unmarried women
Formerly married
Partnered women
CP (any) ≤ 1%

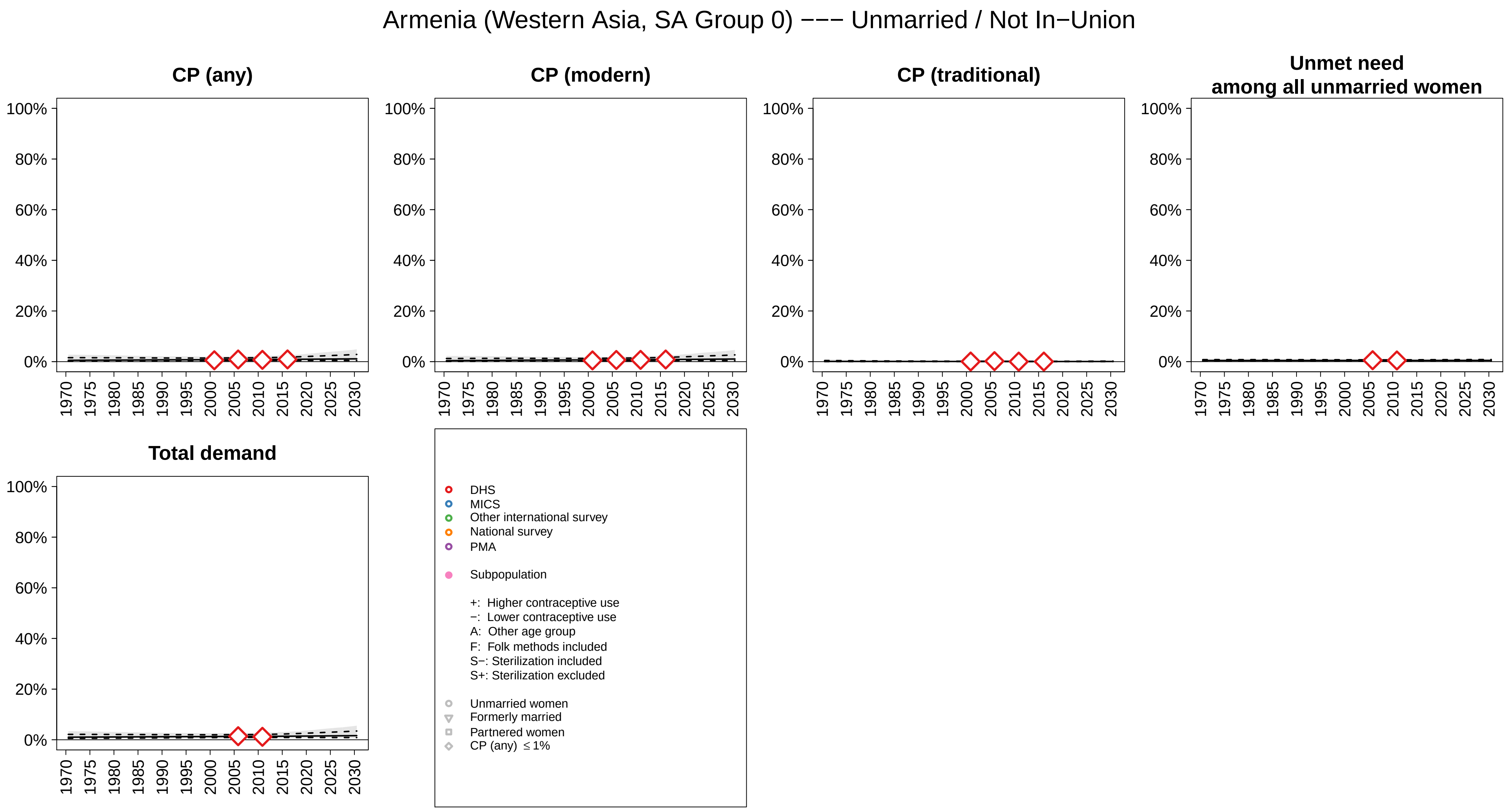
Armenia (Western Asia, SA Group 0) −−− Unmarried / Not In−Union
CP (any)
CP (modern)
CP (traditional)
Unmet need among all unmarried women
Total demand
100%
80%
60%
40%
20%
0%
1970
1975
1980
1985
1990
1995
2000
2005
2010
2015
2020
2025
2030
DHS
MICS
Other international survey
National survey
PMA
Subpopulation
+: Higher contraceptive use
−: Lower contraceptive use
A: Other age group
F: Folk methods included
S−: Sterilization included
S+: Sterilization excluded
Unmarried women
Formerly married
Partnered women
CP (any) ≤ 1%

Australia (Australia and New Zealand, SA Group 1) --- Unmarried / Not In−Union

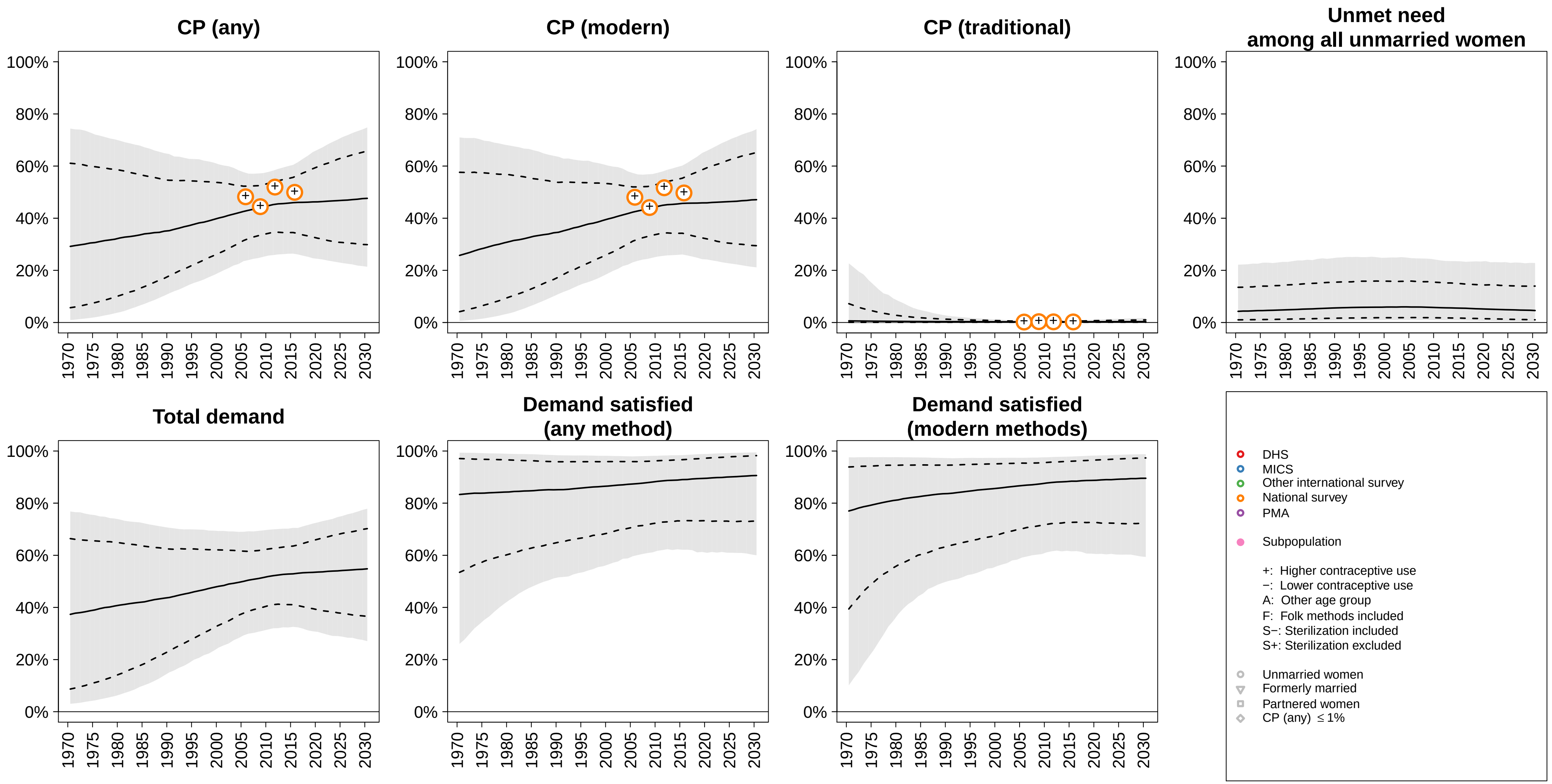

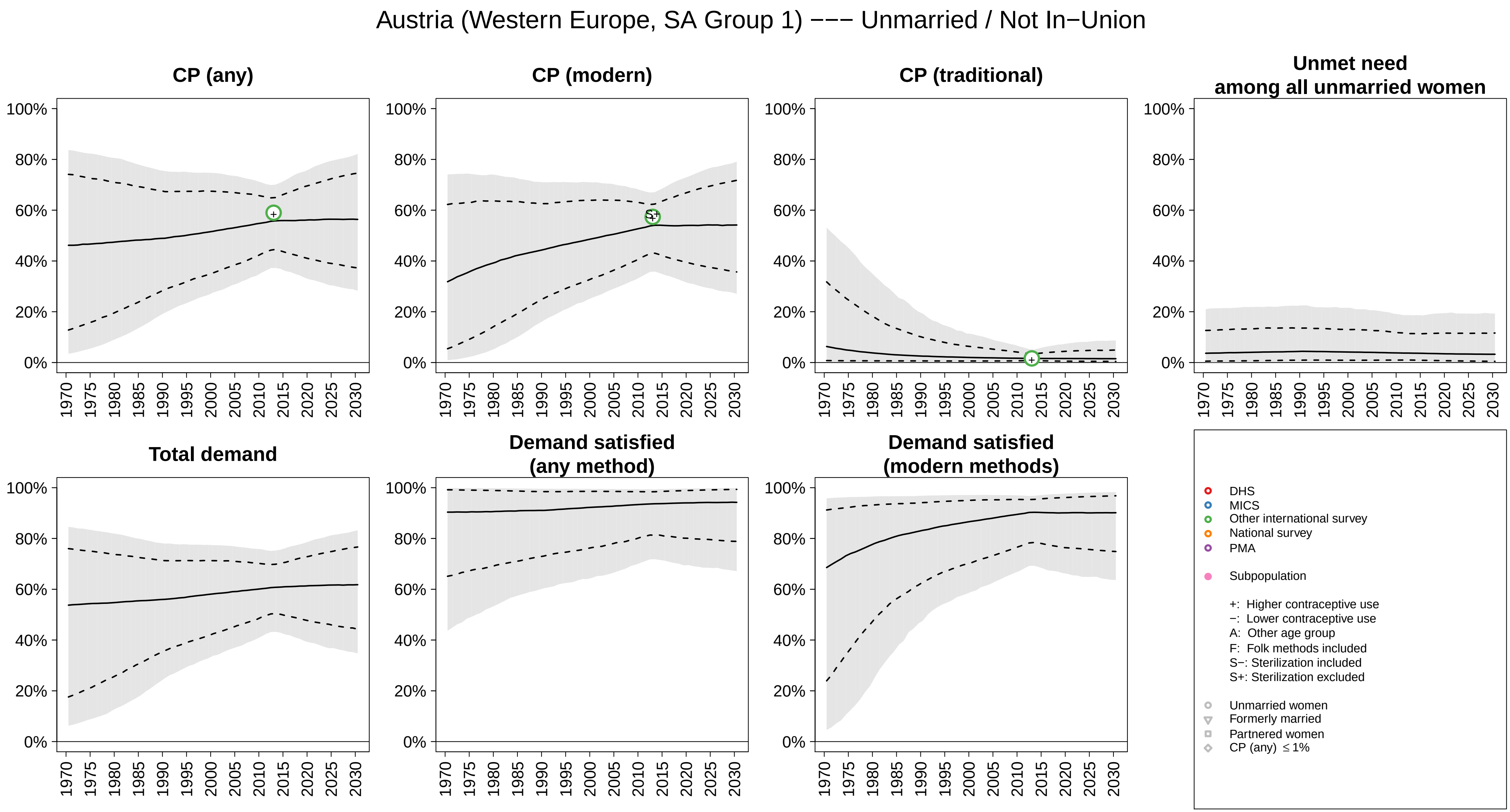
Austria (Western Europe, SA Group 1) −−− Unmarried / Not In−Union
CP (any)
CP (modern)
CP (traditional)
Unmet need among all unmarried women
Total demand
Demand satisfied (any method)
Demand satisfied (modern methods)
100%
80%
60%
40%
20%
0%
1970 1975 1980 1985 1990 1995 2000 2005 2010 2015 2020 2025 2030
DHS
MICS
Other international survey
National survey
PMA
Subpopulation
+: Higher contraceptive use
−: Lower contraceptive use
A: Other age group
F: Folk methods included
S−: Sterilization included
S+: Sterilization excluded
Unmarried women
Formerly married
Partnered women
CP (any) ≤ 1%

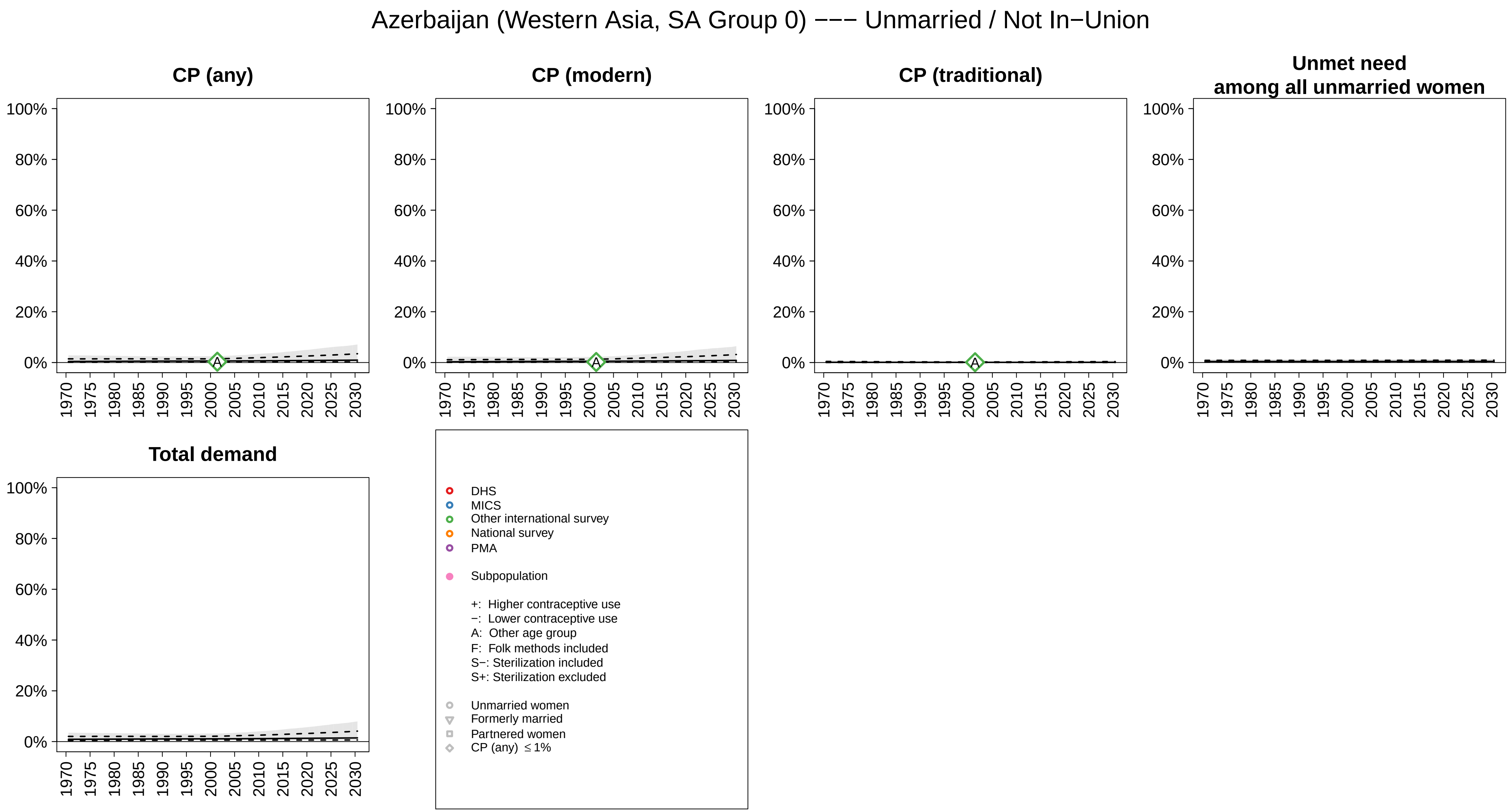
Azerbaijan (Western Asia, SA Group 0) −−− Unmarried / Not In−Union
CP (any)
CP (modern)
CP (traditional)
Unmet need among all unmarried women
Total demand
100%
80%
60%
40%
20%
0%
1970
1975
1980
1985
1990
1995
2000
2005
2010
2015
2020
2025
2030
A
DHS
MICS
Other international survey
National survey
PMA
Subpopulation
+: Higher contraceptive use
−: Lower contraceptive use
A: Other age group
F: Folk methods included
S−: Sterilization included
S+: Sterilization excluded
Unmarried women
Formerly married
Partnered women
CP (any) ≤ 1%

Bahamas (Caribbean, SA Group 1) −−− Unmarried / Not In−Union

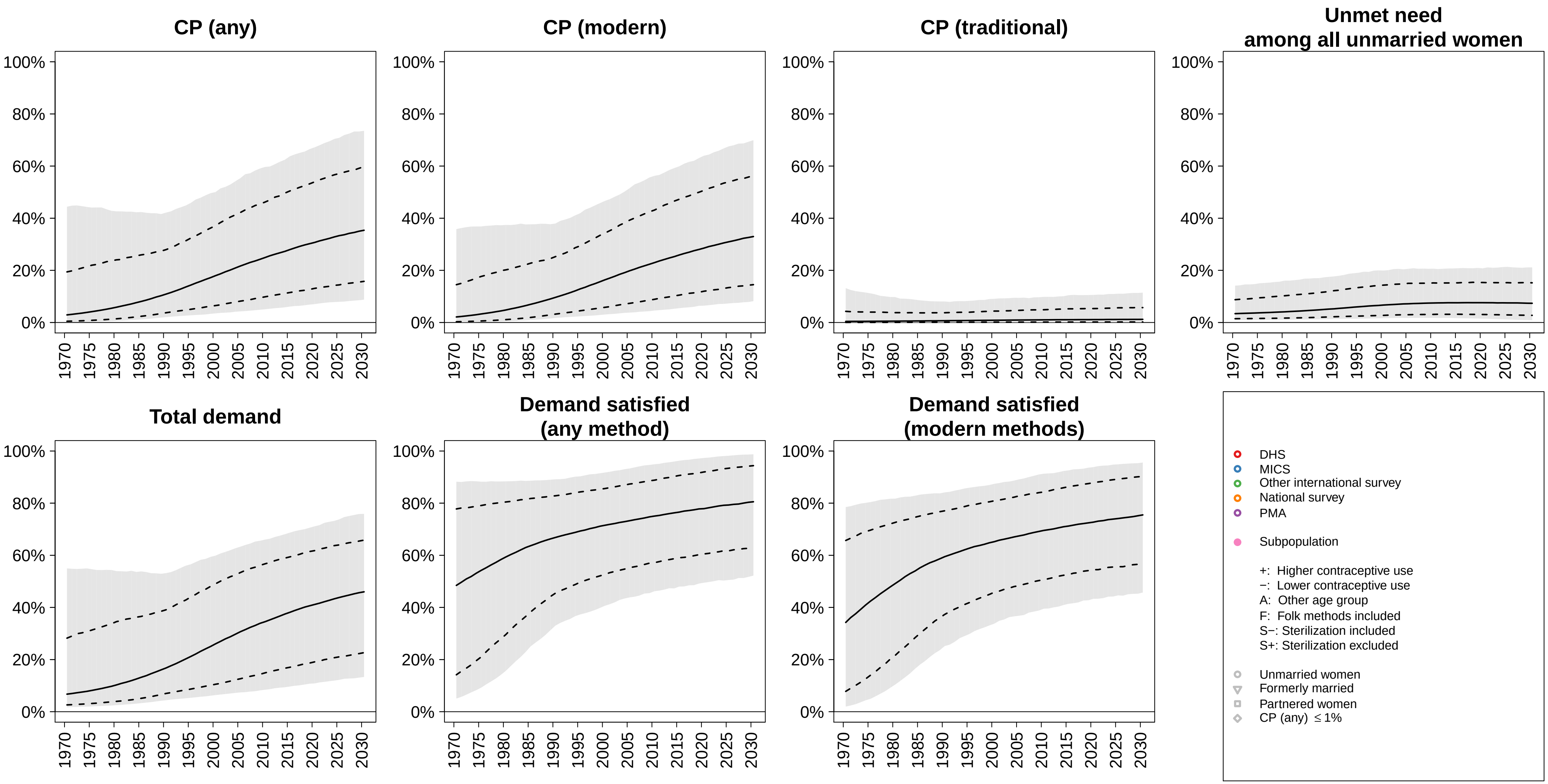

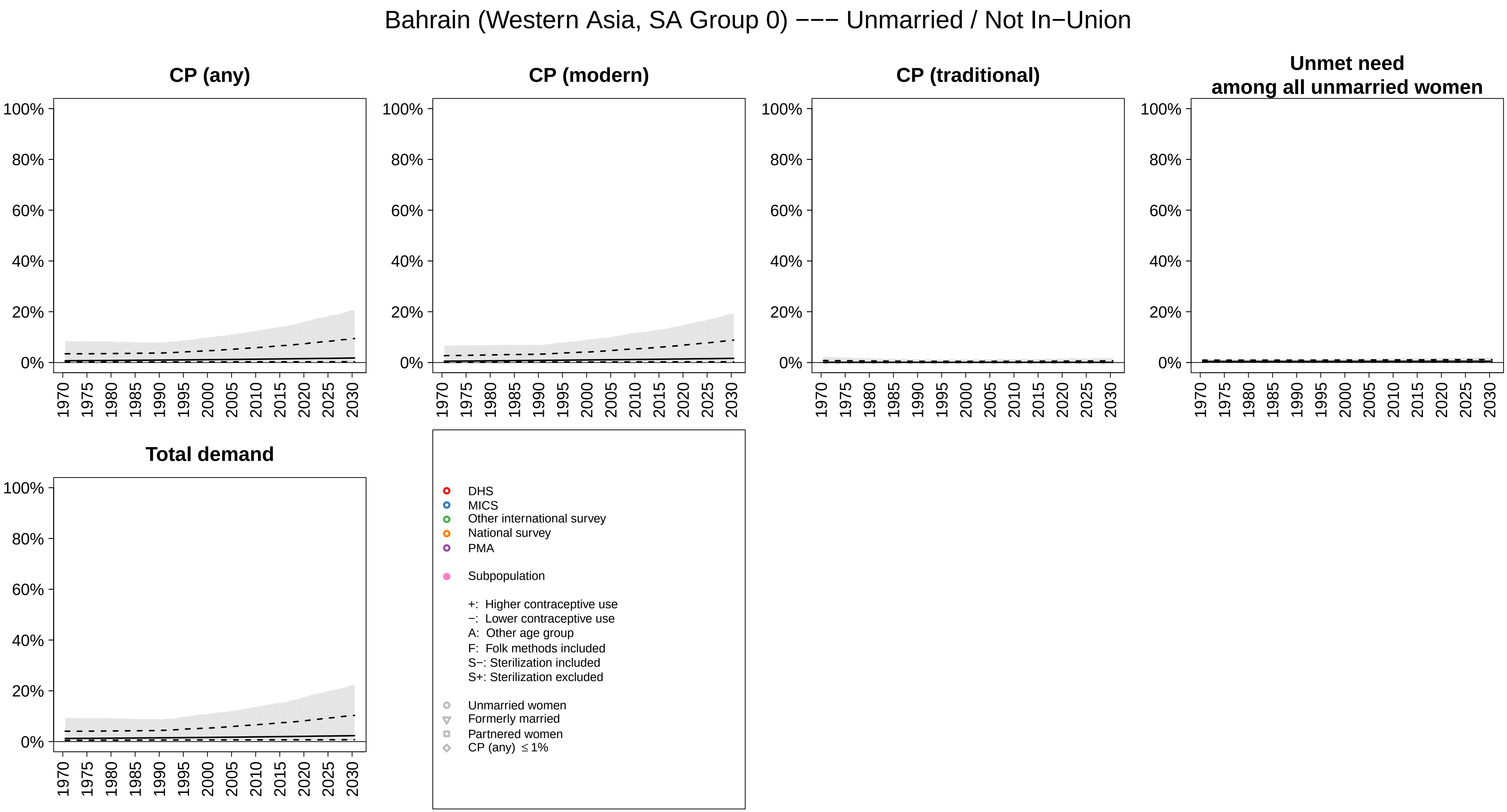
Bahrain (Western Asia, SA Group 0) −−− Unmarried / Not In−Union
CP (any)
CP (modern)
CP (traditional)
Unmet need
among all unmarried women
Total demand
100%
80%
60%
40%
20%
0%
1970
1975
1980
1985
1990
1995
2000
2005
2010
2015
2020
2025
2030
DHS
MICS
Other international survey
National survey
PMA
Subpopulation
+: Higher contraceptive use
−: Lower contraceptive use
A: Other age group
F: Folk methods included
S−: Sterilization included
S+: Sterilization excluded
Unmarried women
Formerly married
Partnered women
CP (any) ≤ 1%

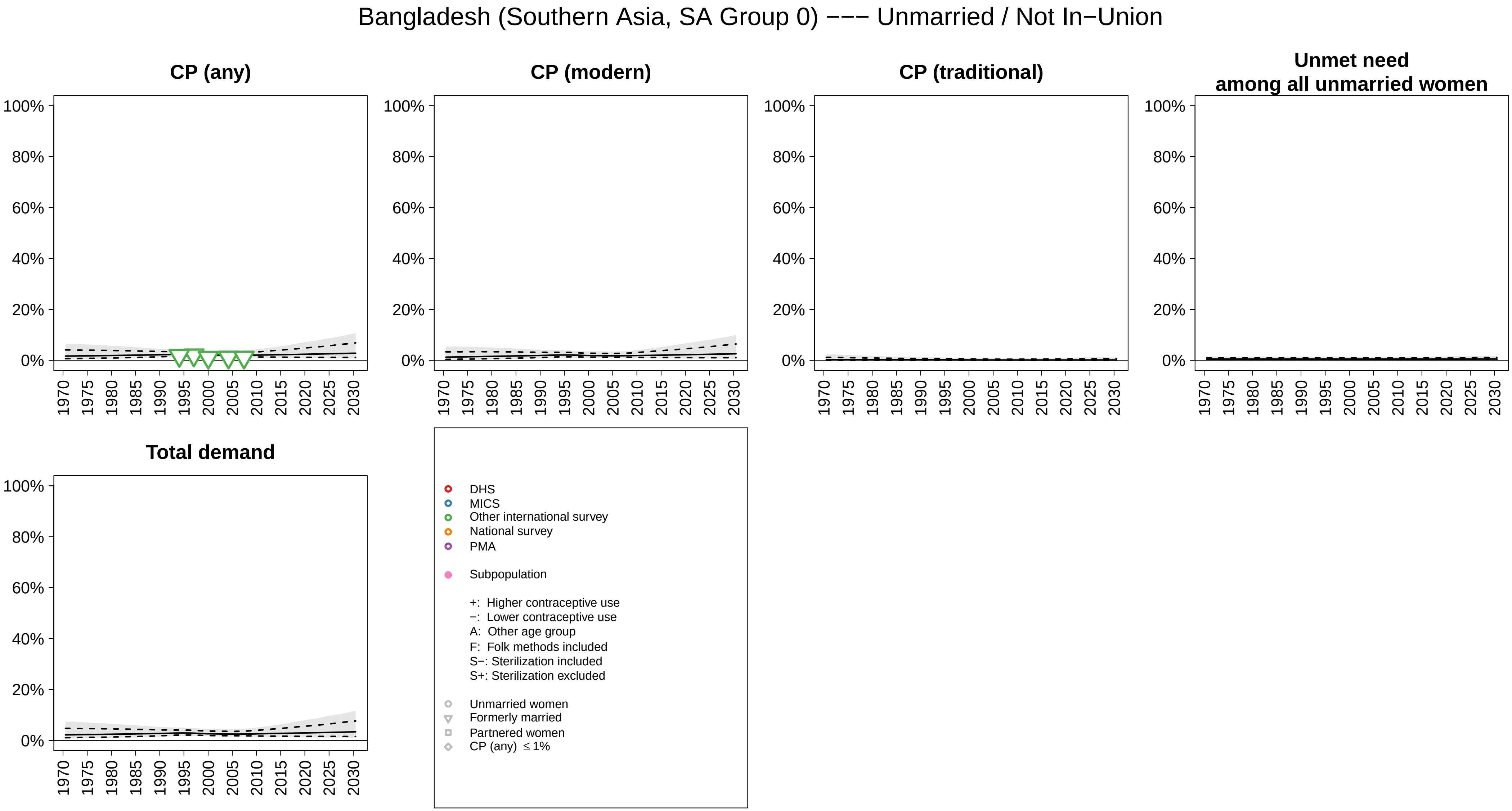
Bangladesh (Southern Asia, SA Group 0) −−− Unmarried / Not In−Union
CP (any)
CP (modern)
CP (traditional)
Unmet need among all unmarried women
Total demand
100%
80%
60%
40%
20%
0%
1970 1975 1980 1985 1990 1995 2000 2005 2010 2015 2020 2025 2030
DHS
MICS
Other international survey
National survey
PMA
Subpopulation
+: Higher contraceptive use
−: Lower contraceptive use
A: Other age group
F: Folk methods included
S−: Sterilization included
S+: Sterilization excluded
Unmarried women
Formerly married
Partnered women
CP (any) ≤ 1%

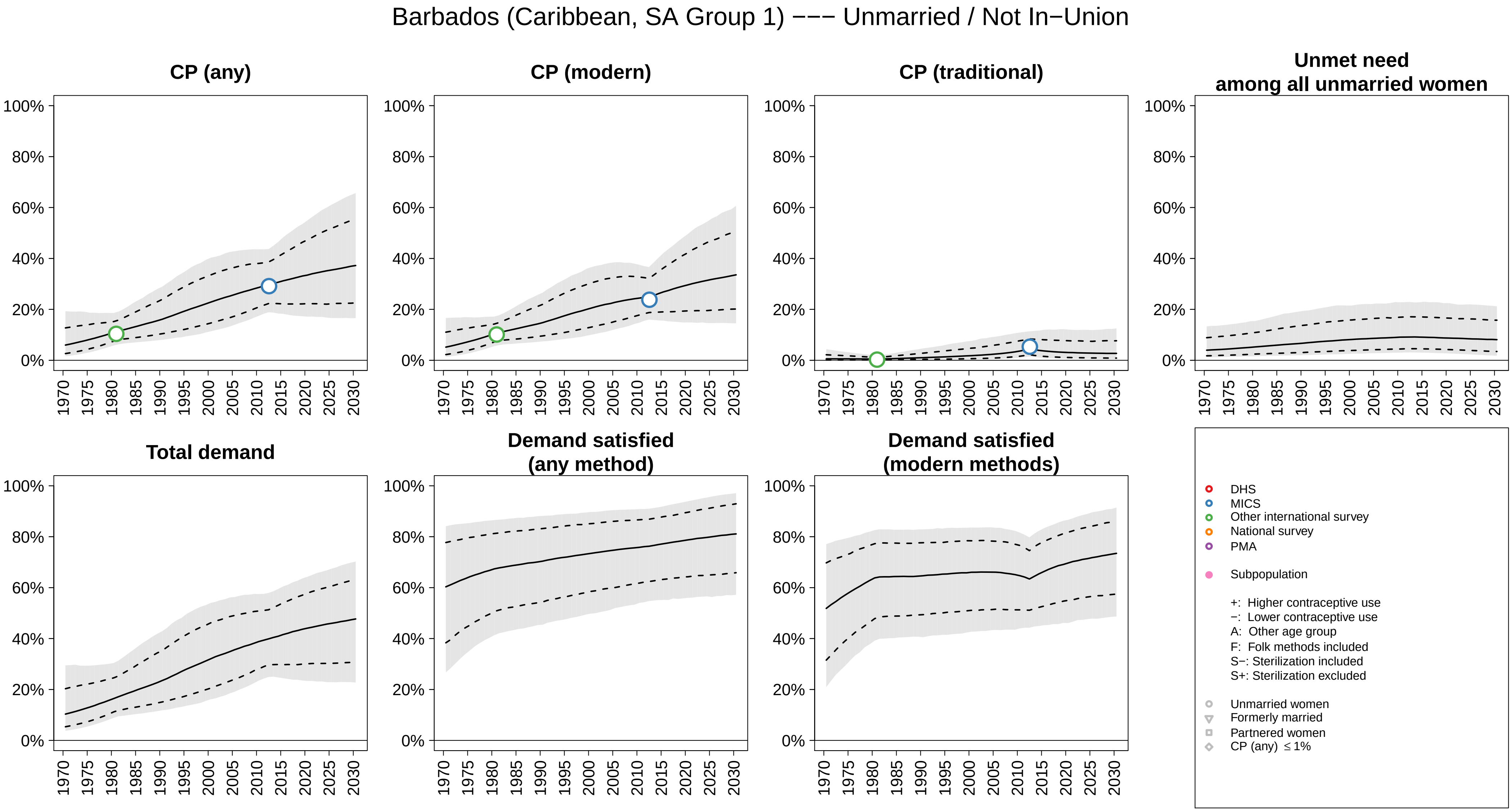
Barbados (Caribbean, SA Group 1) −−− Unmarried / Not In−Union
CP (any)
CP (modern)
CP (traditional)
Unmet need among all unmarried women
Total demand
Demand satisfied (any method)
Demand satisfied (modern methods)
0%
20%
40%
60%
80%
100%
1970
1975
1980
1985
1990
1995
2000
2005
2010
2015
2020
2025
2030
DHS
MICS
Other international survey
National survey
PMA
Subpopulation
+: Higher contraceptive use
−: Lower contraceptive use
A: Other age group
F: Folk methods included
S−: Sterilization included
S+: Sterilization excluded
Unmarried women
Formerly married
Partnered women
CP (any) ≤ 1%

Belarus (Eastern Europe, SA Group 1) −−− Unmarried / Not In−Union

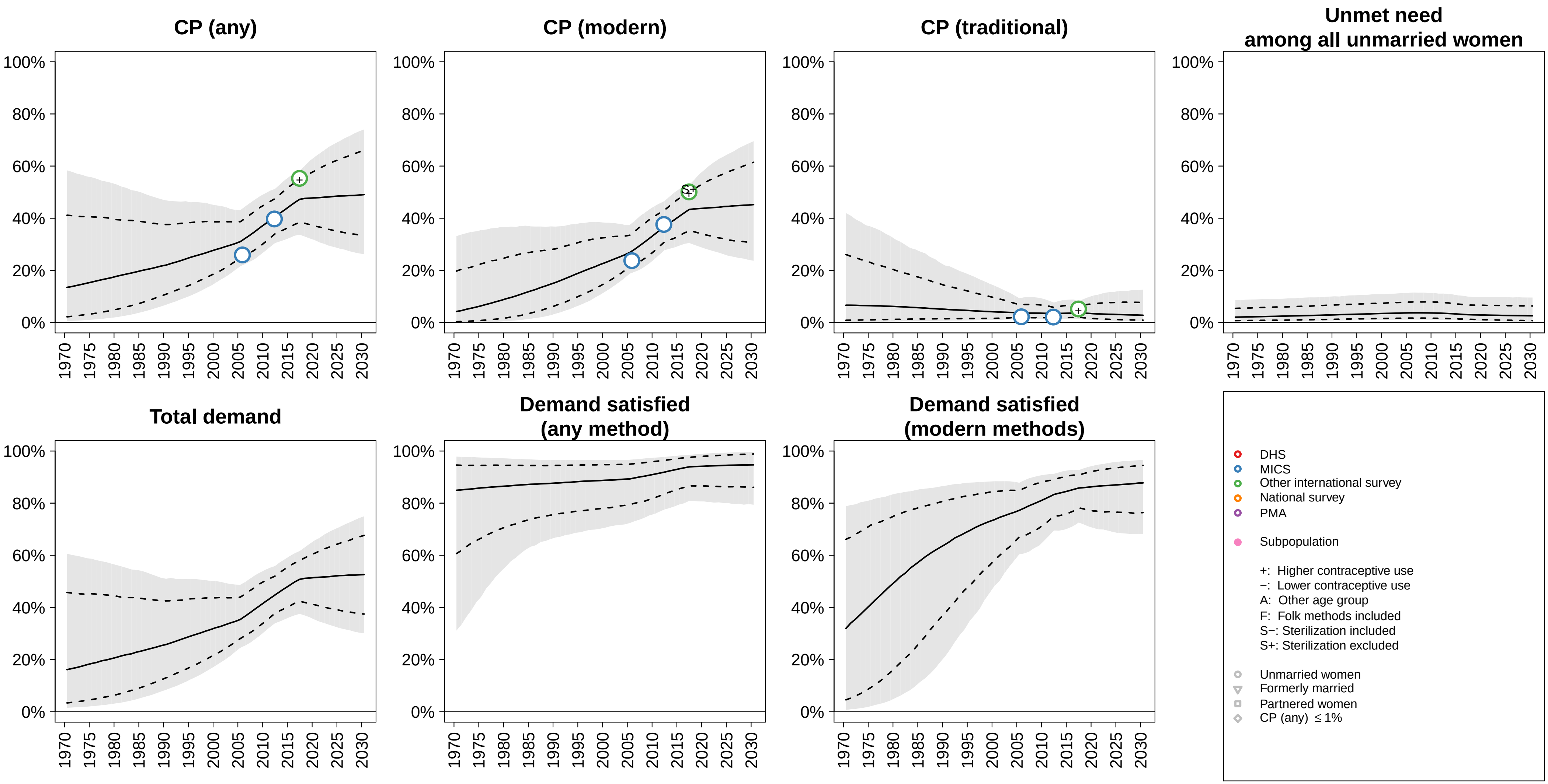

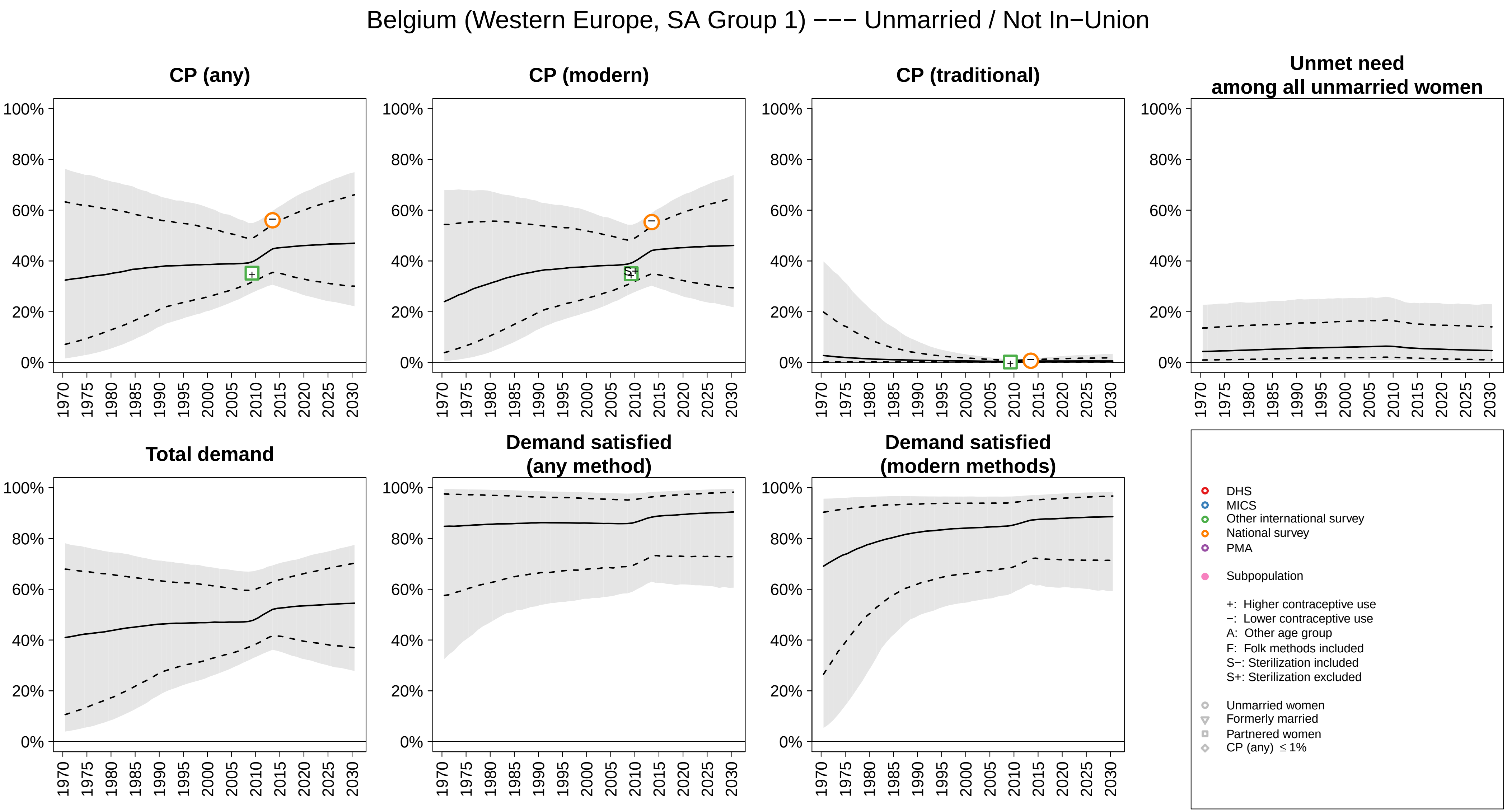
Belgium (Western Europe, SA Group 1) −−− Unmarried / Not In−Union
CP (any)
CP (modern)
CP (traditional)
Unmet need among all unmarried women
Total demand
Demand satisfied (any method)
Demand satisfied (modern methods)
DHS
MICS
Other international survey
National survey
PMA
Subpopulation
+: Higher contraceptive use
−: Lower contraceptive use
A: Other age group
F: Folk methods included
S−: Sterilization included
S+: Sterilization excluded
Unmarried women
Formerly married
Partnered women
CP (any) ≤ 1%

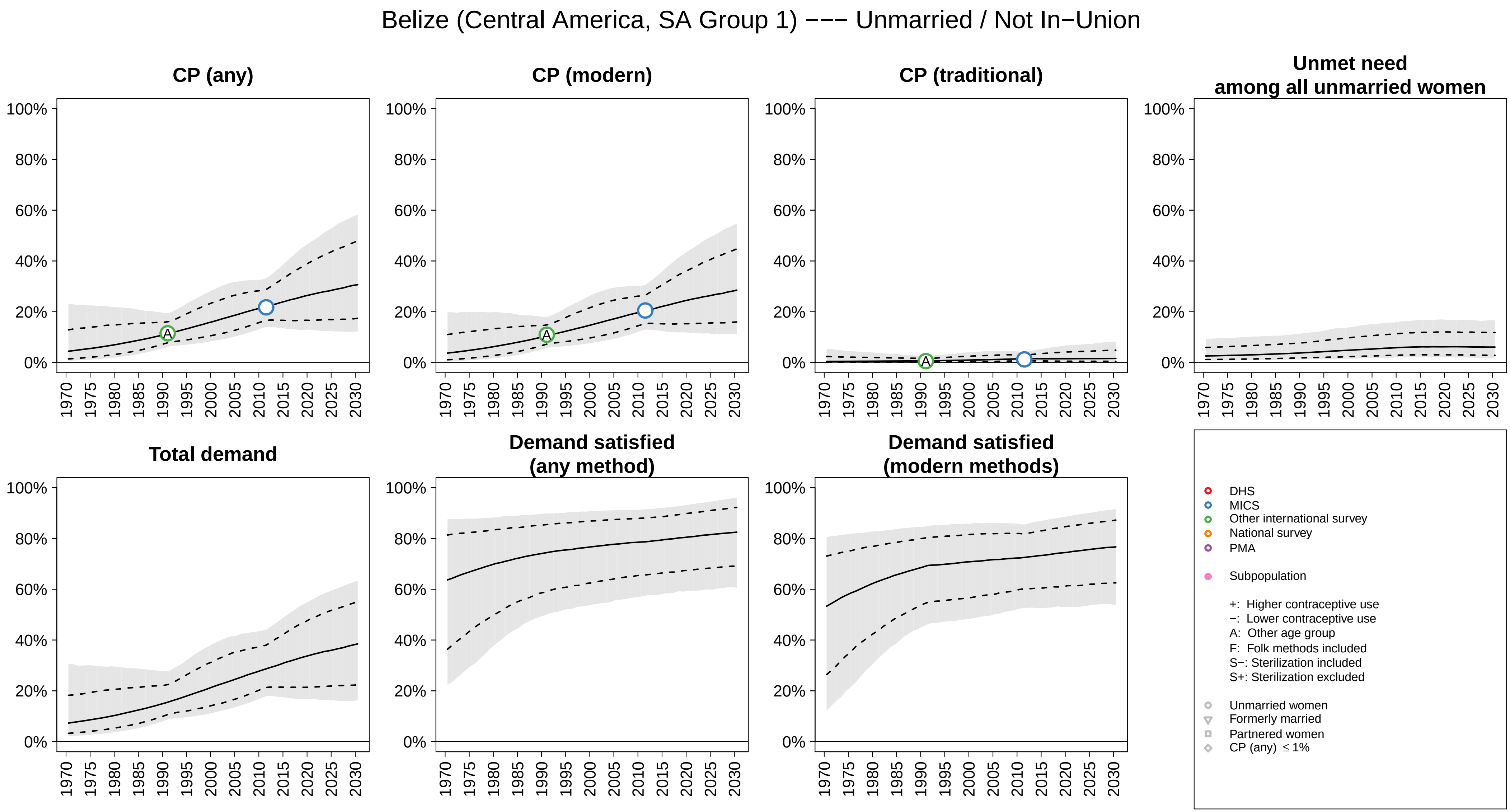
Belize (Central America, SA Group 1) −−− Unmarried / Not In−Union
CP (any)
CP (modern)
CP (traditional)
Unmet need among all unmarried women
Total demand
Demand satisfied (any method)
Demand satisfied (modern methods)
100%
80%
60%
40%
20%
0%
1970
1975
1980
1985
1990
1995
2000
2005
2010
2015
2020
2025
2030
A
DHS
MICS
Other international survey
National survey
PMA
Subpopulation
+: Higher contraceptive use
−: Lower contraceptive use
A: Other age group
F: Folk methods included
S−: Sterilization included
S+: Sterilization excluded
Unmarried women
Formerly married
Partnered women
CP (any) ≤ 1%

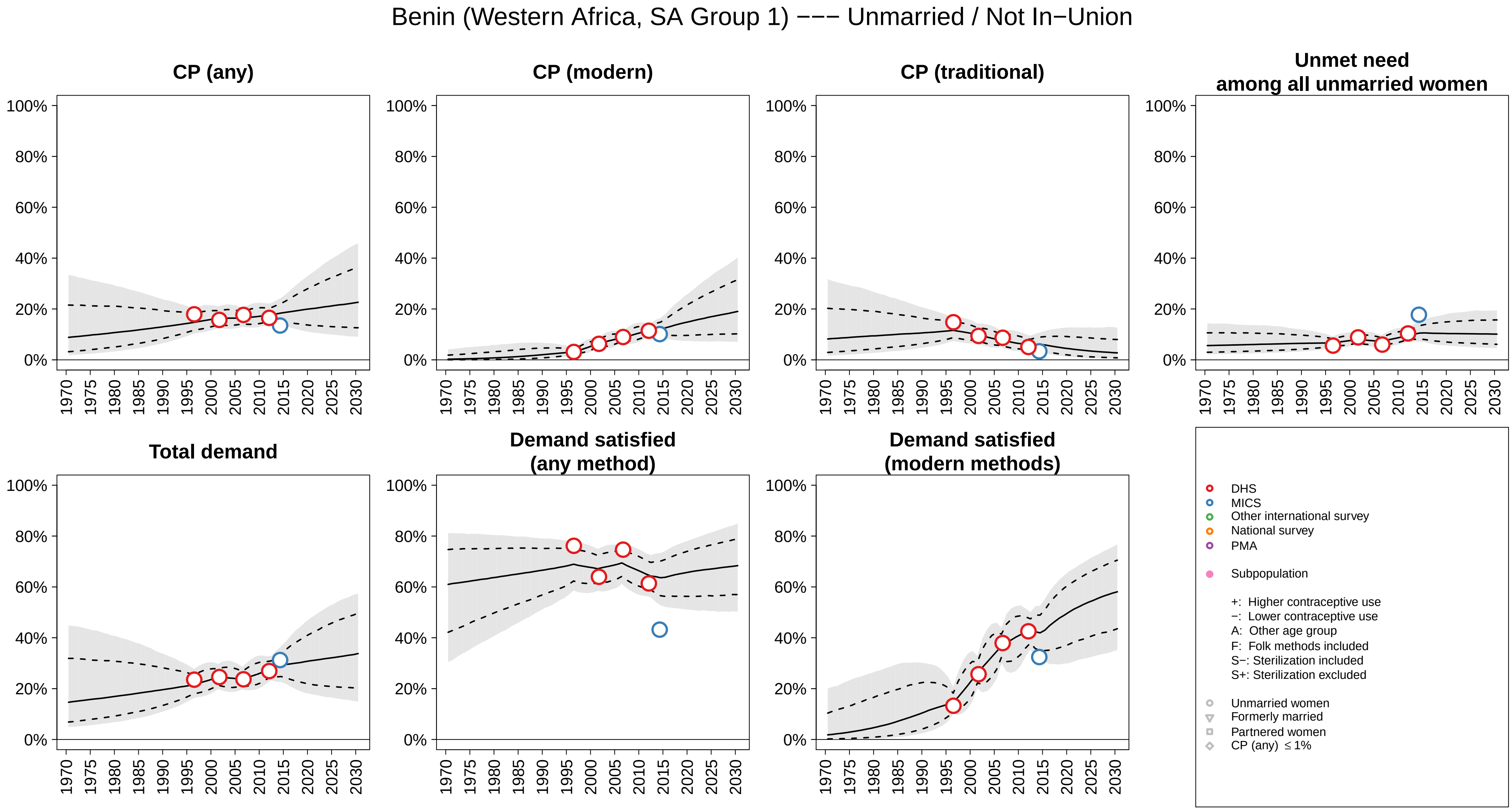
Benin (Western Africa, SA Group 1) −−− Unmarried / Not In−Union
CP (any)
CP (modern)
CP (traditional)
Unmet need among all unmarried women
Total demand
Demand satisfied (any method)
Demand satisfied (modern methods)
DHS
MICS
Other international survey
National survey
PMA
Subpopulation
+: Higher contraceptive use
−: Lower contraceptive use
A: Other age group
F: Folk methods included
S−: Sterilization included
S+: Sterilization excluded
Unmarried women
Formerly married
Partnered women
CP (any) ≤ 1%

Bhutan (Southern Asia, SA Group 0) −−− Unmarried / Not In−Union

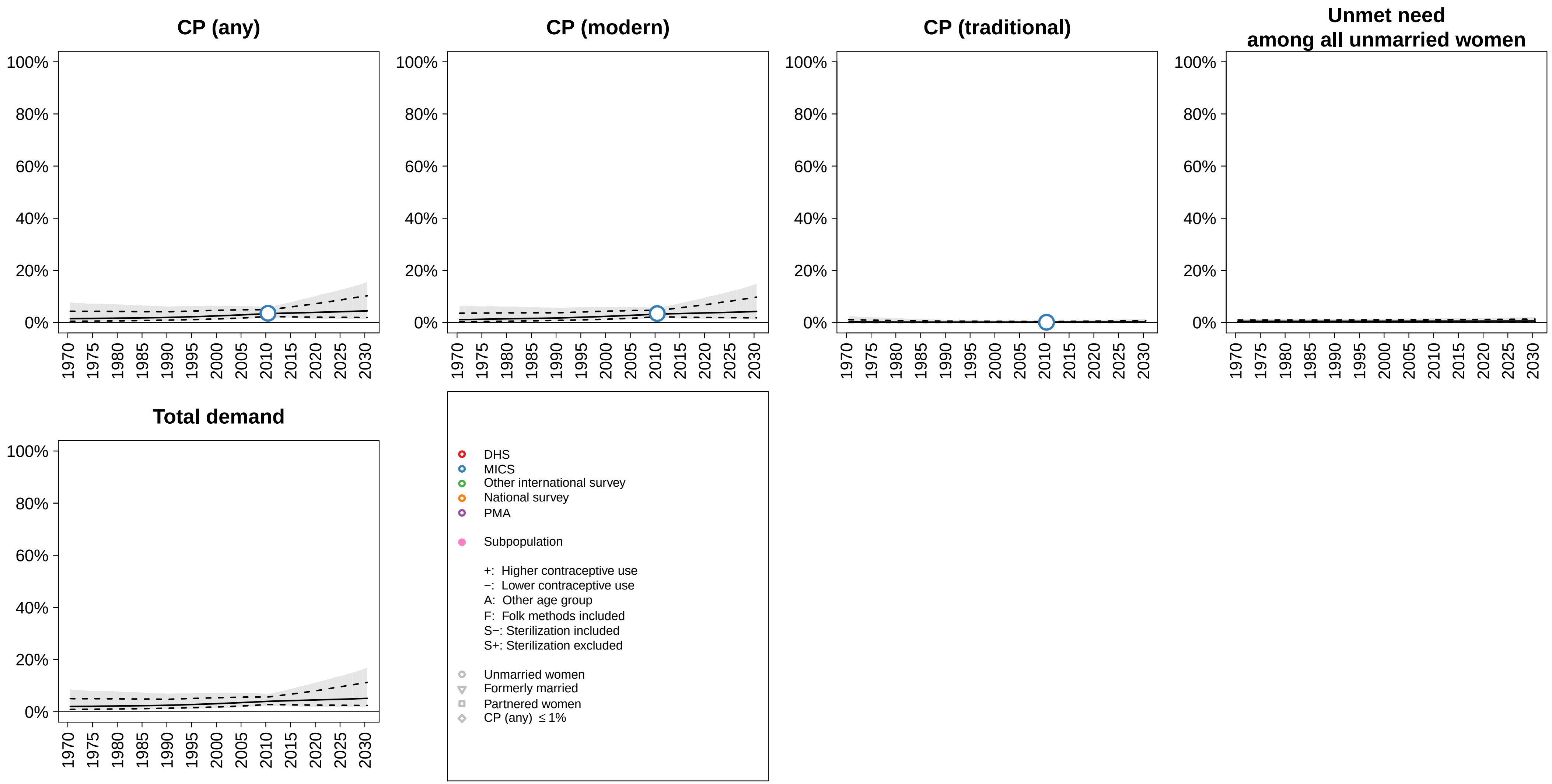

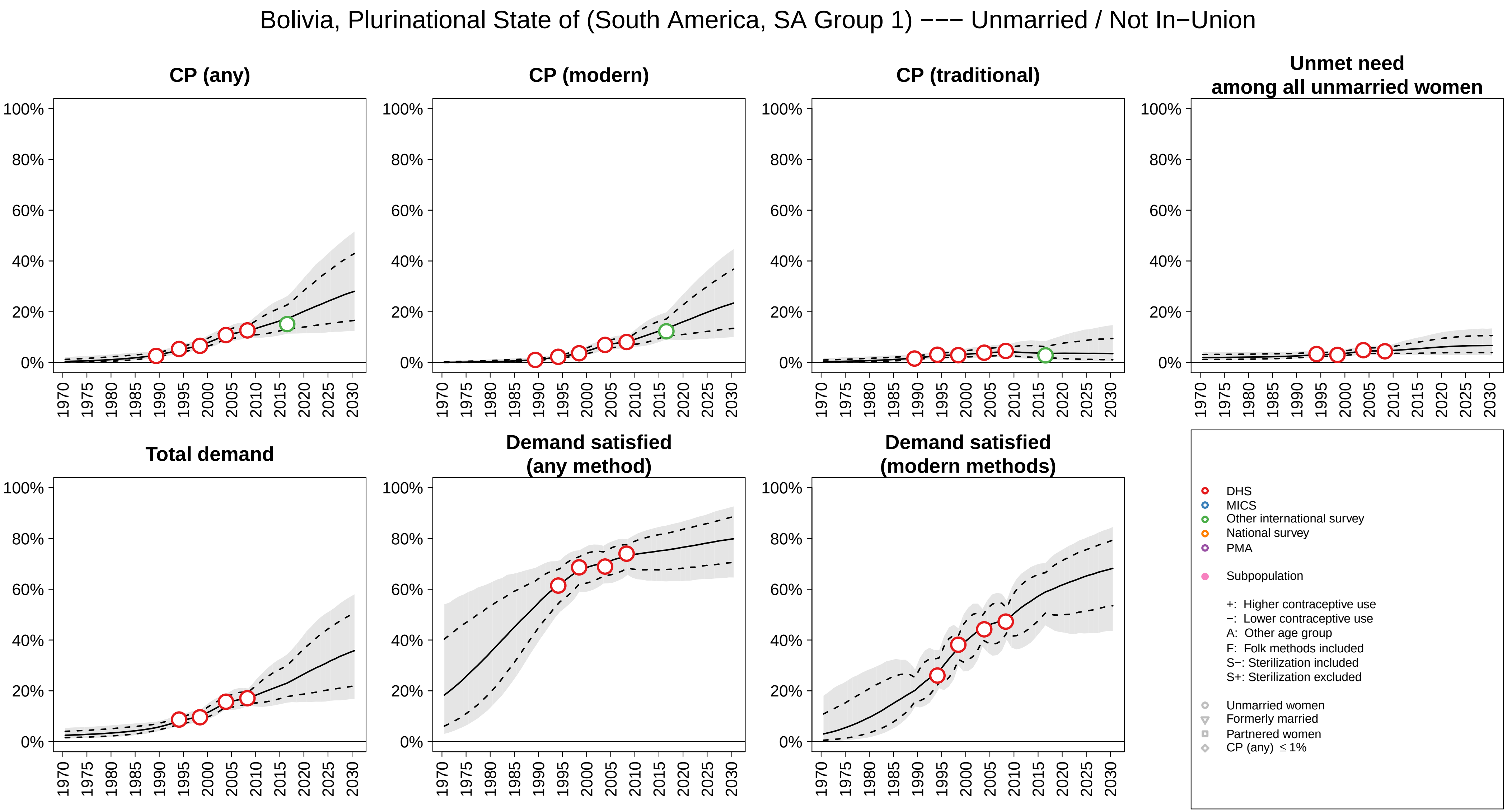
Bolivia, Plurinational State of (South America, SA Group 1) −−− Unmarried / Not In−Union
CP (any)
CP (modern)
CP (traditional)
Unmet need among all unmarried women
Total demand
Demand satisfied (any method)
Demand satisfied (modern methods)
0%
20%
40%
60%
80%
100%
1970
1975
1980
1985
1990
1995
2000
2005
2010
2015
2020
2025
2030
DHS
MICS
Other international survey
National survey
PMA
Subpopulation
+: Higher contraceptive use
−: Lower contraceptive use
A: Other age group
F: Folk methods included
S−: Sterilization included
S+: Sterilization excluded
Unmarried women
Formerly married
Partnered women
CP (any) ≤ 1%

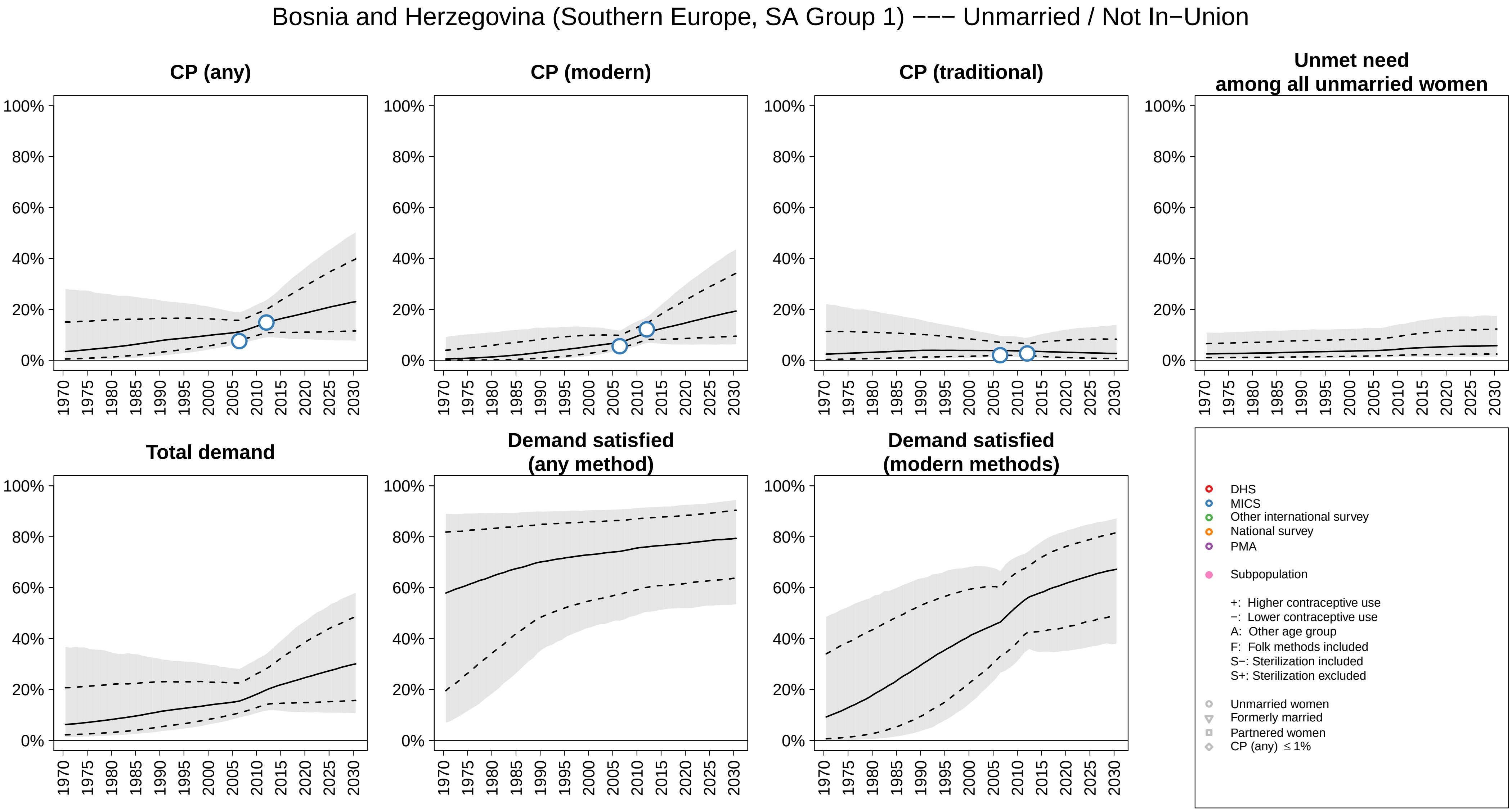
Bosnia and Herzegovina (Southern Europe, SA Group 1) −−− Unmarried / Not In−Union
CP (any)
CP (modern)
CP (traditional)
Unmet need among all unmarried women
Total demand
Demand satisfied (any method)
Demand satisfied (modern methods)
100%
80%
60%
40%
20%
0%
1970
1975
1980
1985
1990
1995
2000
2005
2010
2015
2020
2025
2030
DHS
MICS
Other international survey
National survey
PMA
Subpopulation
+: Higher contraceptive use
−: Lower contraceptive use
A: Other age group
F: Folk methods included
S−: Sterilization included
S+: Sterilization excluded
Unmarried women
Formerly married
Partnered women
CP (any) ≤ 1%

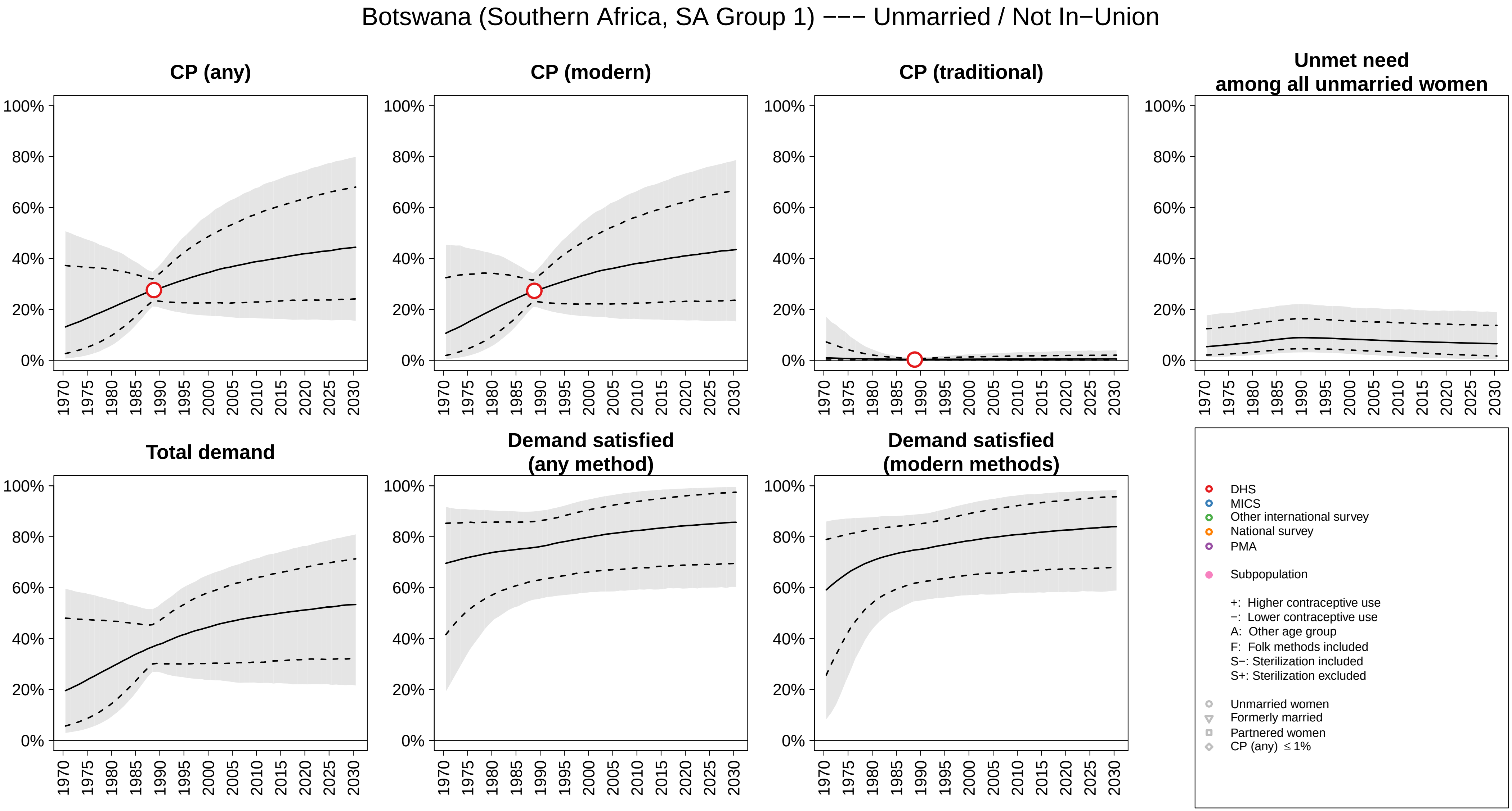
Botswana (Southern Africa, SA Group 1) −−− Unmarried / Not In−Union
CP (any)
CP (modern)
CP (traditional)
Unmet need among all unmarried women
Total demand
Demand satisfied (any method)
Demand satisfied (modern methods)
0%
20%
40%
60%
80%
100%
1970
1975
1980
1985
1990
1995
2000
2005
2010
2015
2020
2025
2030
DHS
MICS
Other international survey
National survey
PMA
Subpopulation
+: Higher contraceptive use
−: Lower contraceptive use
A: Other age group
F: Folk methods included
S−: Sterilization included
S+: Sterilization excluded
Unmarried women
Formerly married
Partnered women
CP (any) ≤ 1%

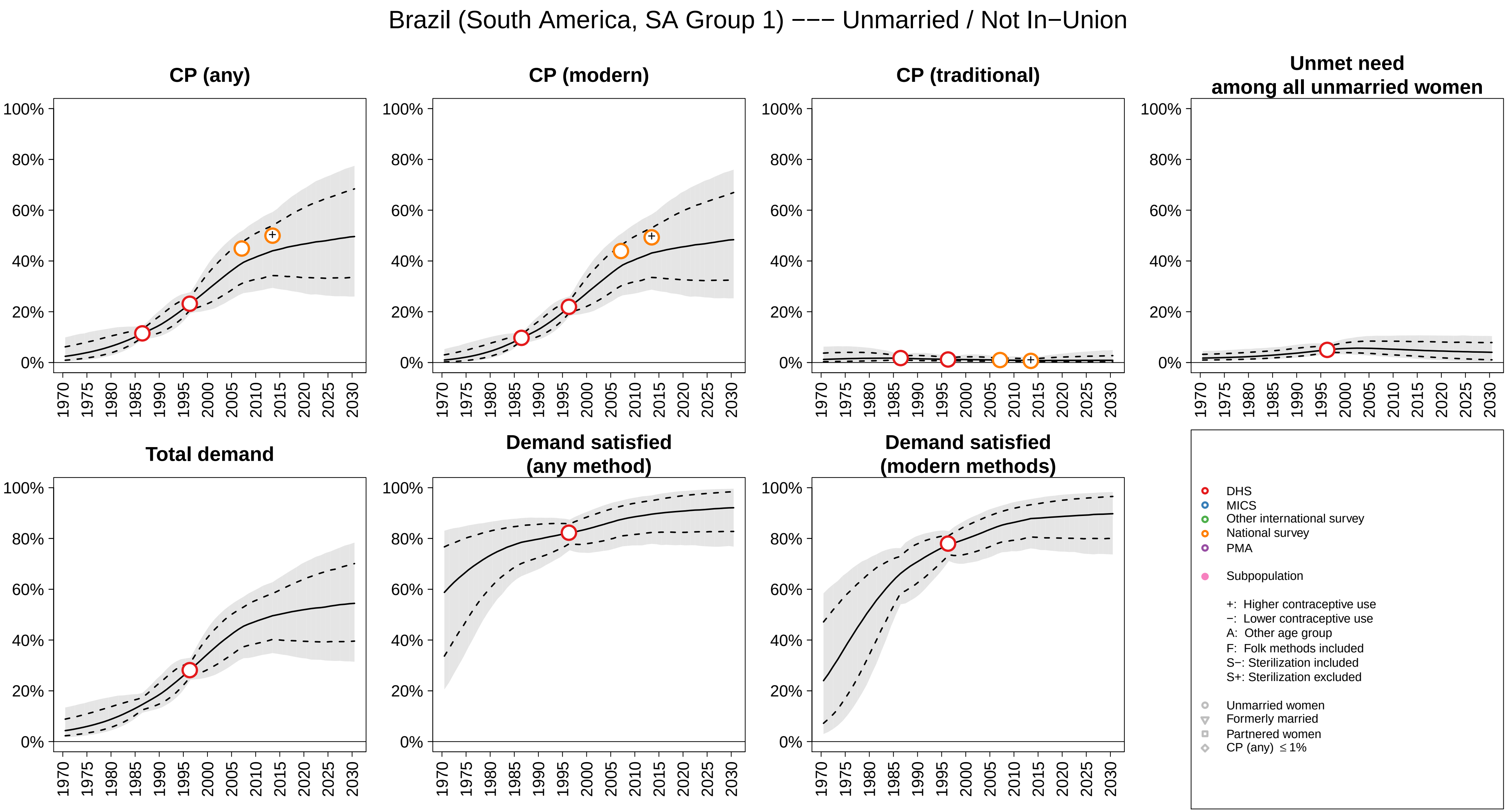
Brazil (South America, SA Group 1) −−− Unmarried / Not In−Union
CP (any)
CP (modern)
CP (traditional)
Unmet need among all unmarried women
Total demand
Demand satisfied (any method)
Demand satisfied (modern methods)
100%
80%
60%
40%
20%
0%
1970
1975
1980
1985
1990
1995
2000
2005
2010
2015
2020
2025
2030
DHS
MICS
Other international survey
National survey
PMA
Subpopulation
+: Higher contraceptive use
−: Lower contraceptive use
A: Other age group
F: Folk methods included
S−: Sterilization included
S+: Sterilization excluded
Unmarried women
Formerly married
Partnered women
CP (any) ≤ 1%

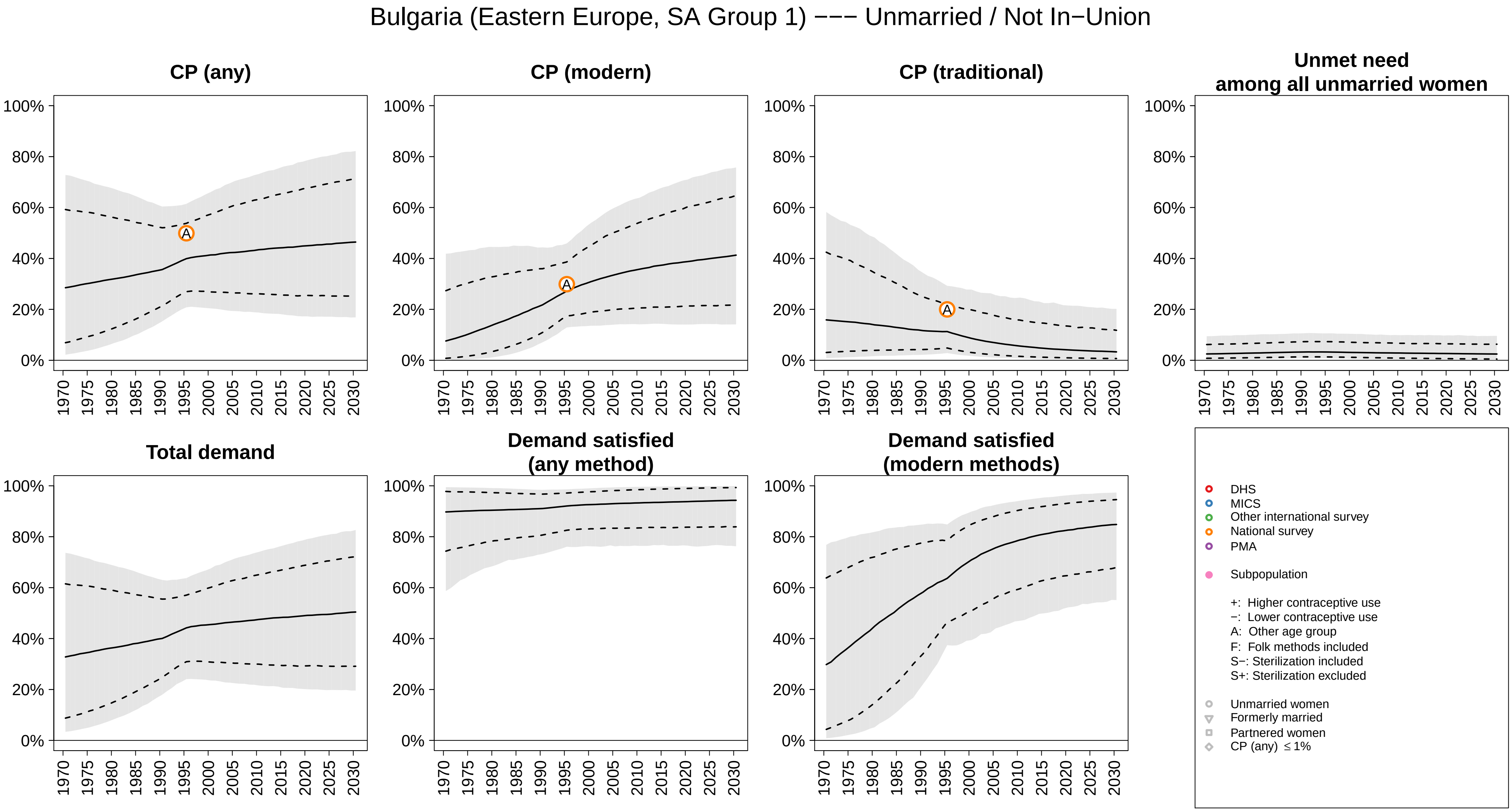
Bulgaria (Eastern Europe, SA Group 1) −−− Unmarried / Not In−Union
CP (any)
CP (modern)
CP (traditional)
Unmet need among all unmarried women
Total demand
Demand satisfied (any method)
Demand satisfied (modern methods)
100%
80%
60%
40%
20%
0%
1970 1975 1980 1985 1990 1995 2000 2005 2010 2015 2020 2025 2030
A
DHS
MICS
Other international survey
National survey
PMA
Subpopulation
+: Higher contraceptive use
−: Lower contraceptive use
A: Other age group
F: Folk methods included
S−: Sterilization included
S+: Sterilization excluded
Unmarried women
Formerly married
Partnered women
CP (any) ≤ 1%

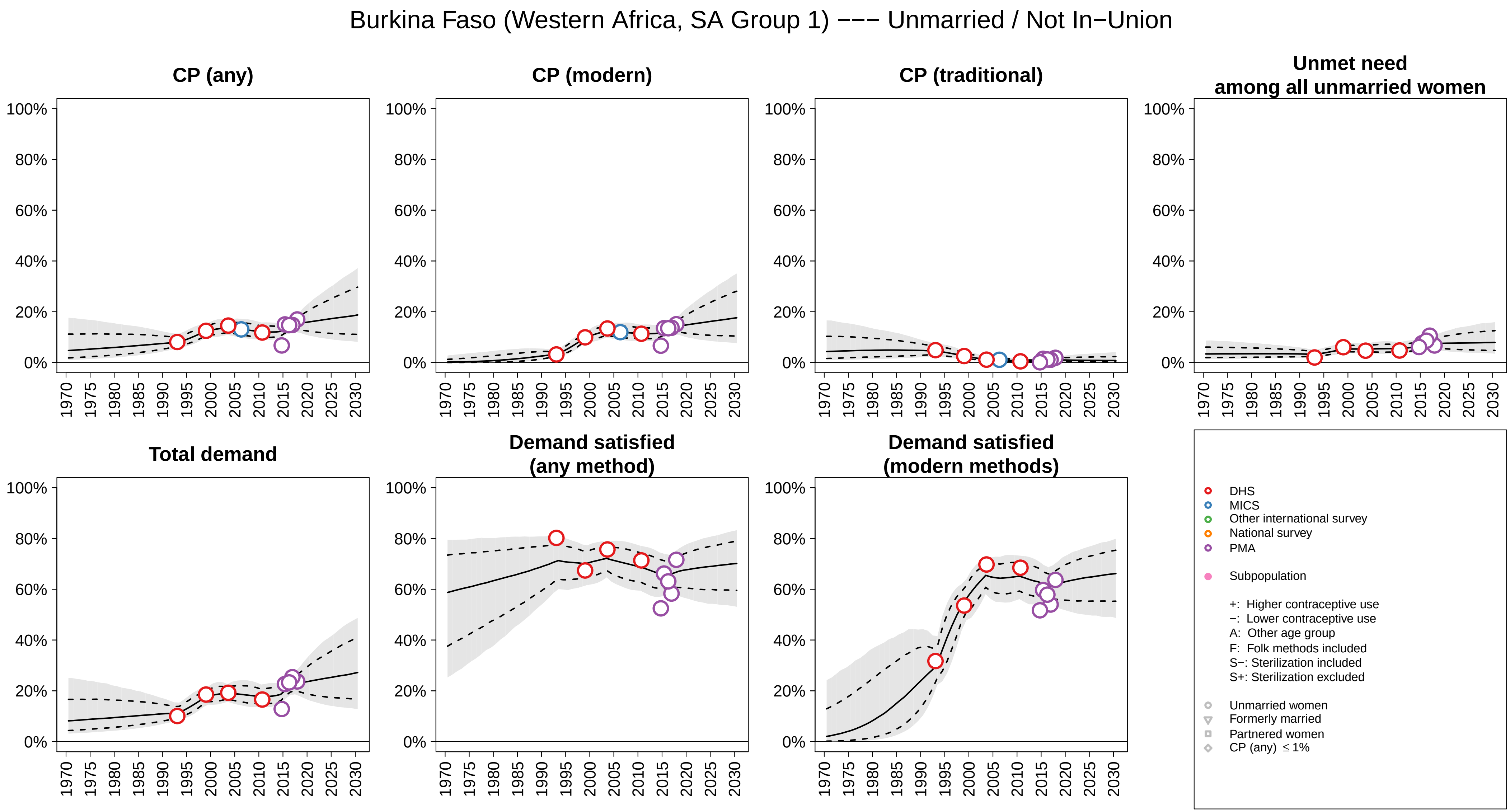
Burkina Faso (Western Africa, SA Group 1) ––– Unmarried / Not In−Union
CP (any)
CP (modern)
CP (traditional)
Unmet need among all unmarried women
Total demand
Demand satisfied (any method)
Demand satisfied (modern methods)
100%
80%
60%
40%
20%
0%
1970
1975
1980
1985
1990
1995
2000
2005
2010
2015
2020
2025
2030
DHS
MICS
Other international survey
National survey
PMA
Subpopulation
+: Higher contraceptive use
−: Lower contraceptive use
A: Other age group
F: Folk methods included
S−: Sterilization included
S+: Sterilization excluded
Unmarried women
Formerly married
Partnered women
CP (any) ≤ 1%

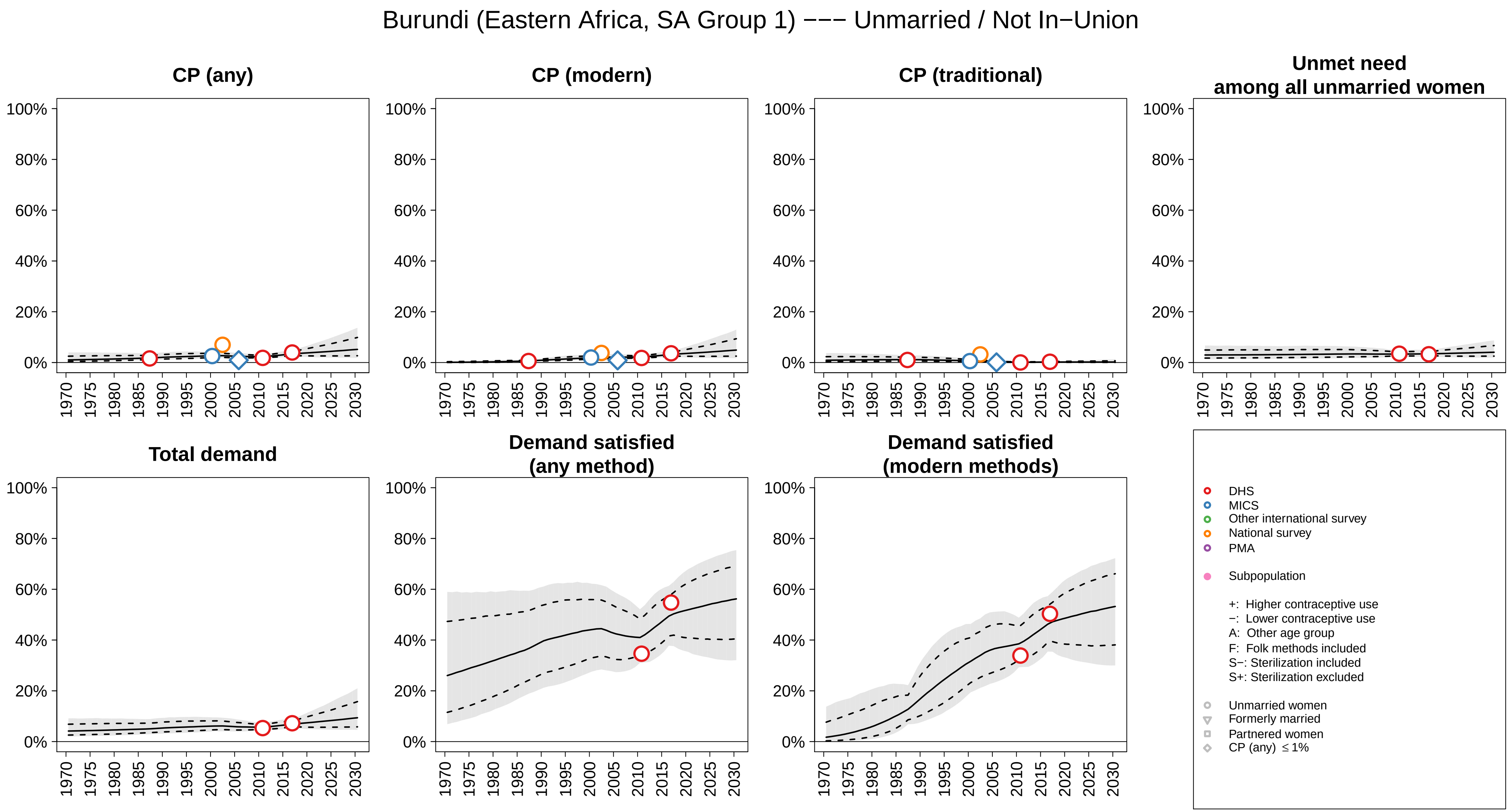

Burundi (Eastern Africa, SA Group 1) −−− Unmarried / Not In−Union
CP (any)
CP (modern)
CP (traditional)
Unmet need among all unmarried women
Total demand
Demand satisfied (any method)
Demand satisfied (modern methods)
100%
80%
60%
40%
20%
0%
1970 1975 1980 1985 1990 1995 2000 2005 2010 2015 2020 2025 2030
DHS
MICS
Other international survey
National survey
PMA
Subpopulation
+: Higher contraceptive use
−: Lower contraceptive use
A: Other age group
F: Folk methods included
S−: Sterilization included
S+: Sterilization excluded
Unmarried women
Formerly married
Partnered women
CP (any) ≤ 1%

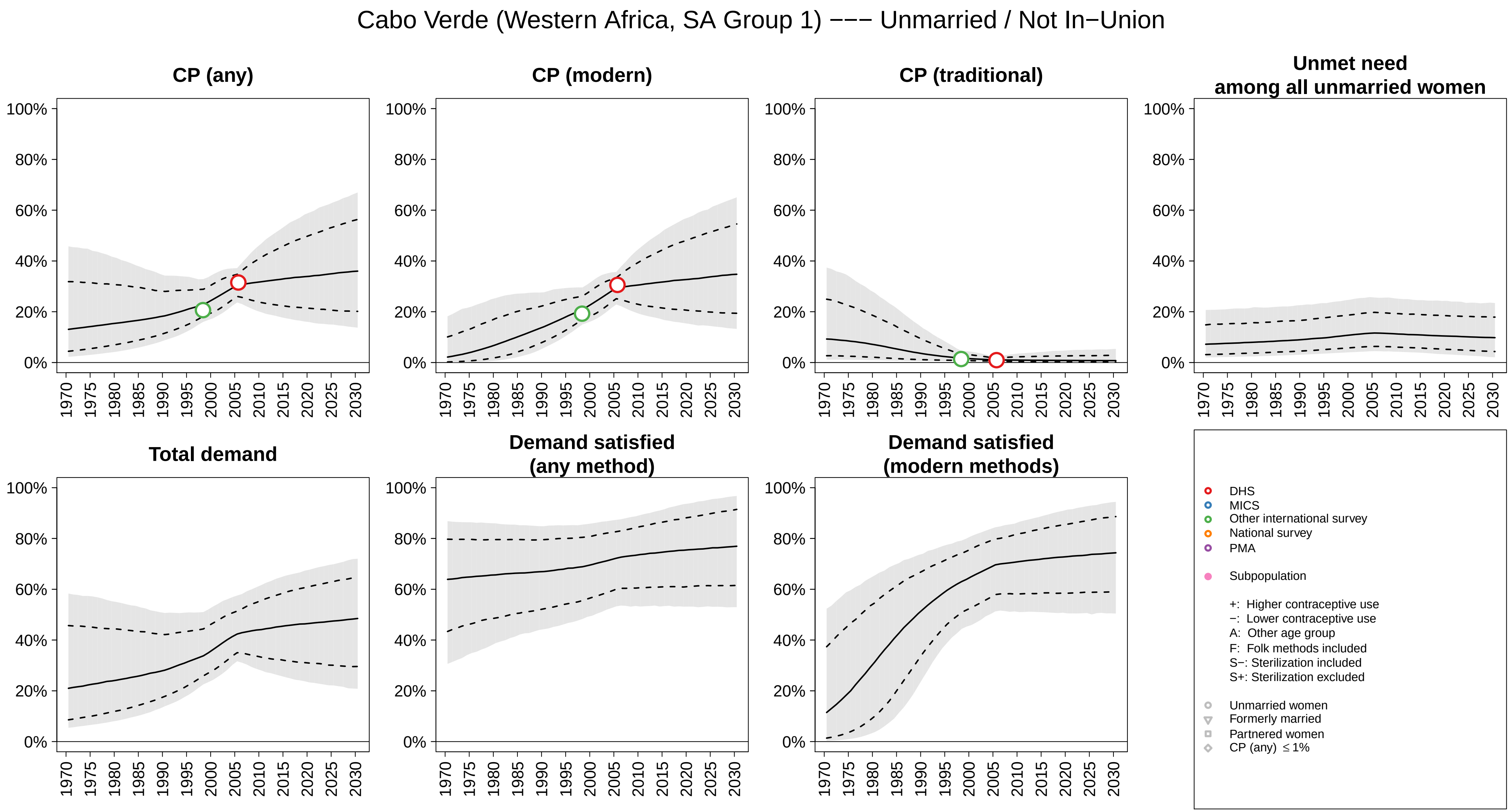
Cabo Verde (Western Africa, SA Group 1) --- Unmarried / Not In−Union
CP (any)
CP (modern)
CP (traditional)
Unmet need among all unmarried women
Total demand
Demand satisfied (any method)
Demand satisfied (modern methods)
100%
80%
60%
40%
20%
0%
1970 1975 1980 1985 1990 1995 2000 2005 2010 2015 2020 2025 2030
DHS
MICS
Other international survey
National survey
PMA
Subpopulation
+: Higher contraceptive use
−: Lower contraceptive use
A: Other age group
F: Folk methods included
S−: Sterilization included
S+: Sterilization excluded
Unmarried women
Formerly married
Partnered women
CP (any) ≤ 1%

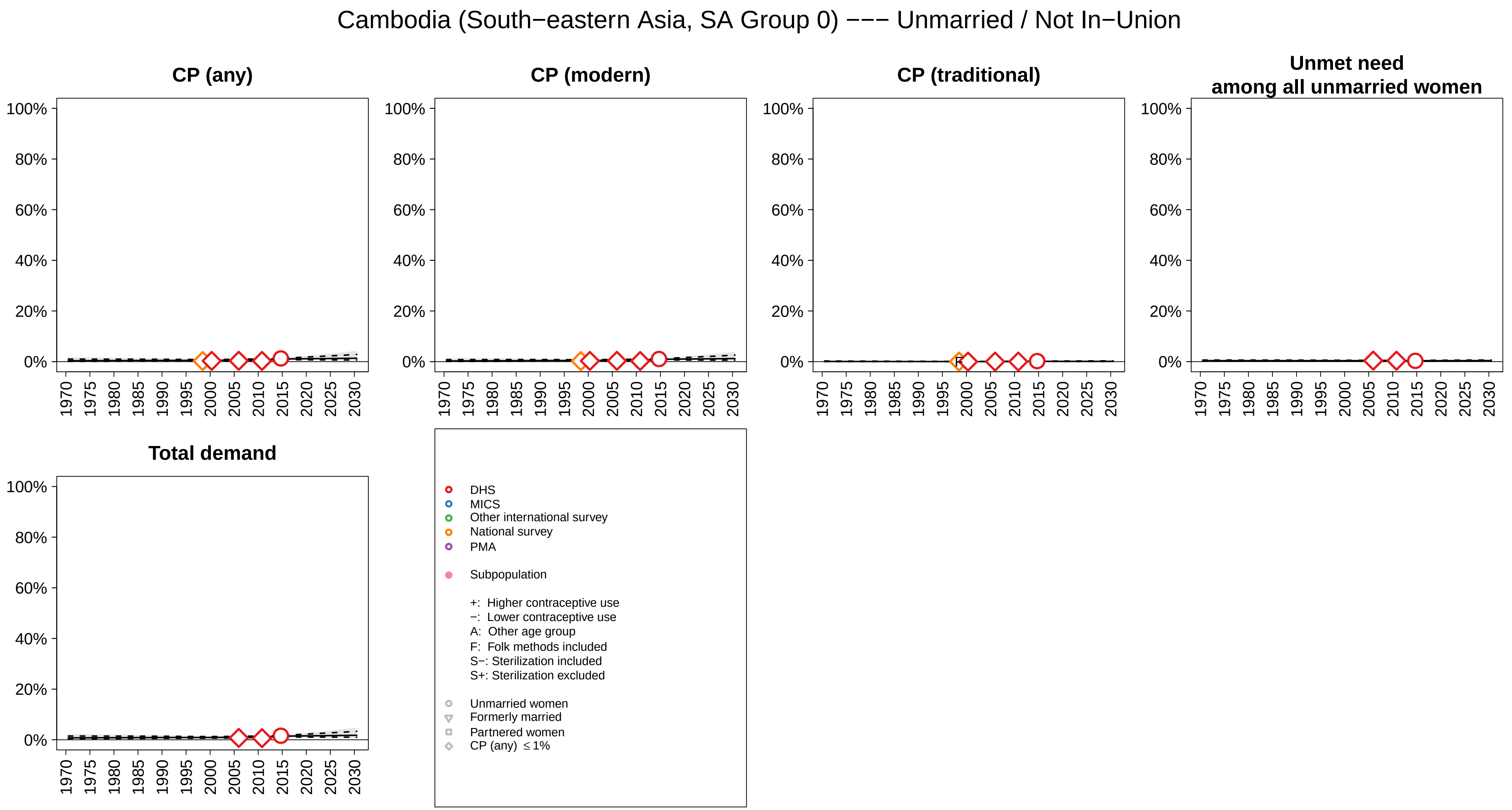
Cambodia (South−eastern Asia, SA Group 0) −−− Unmarried / Not In−Union
CP (any)
CP (modern)
CP (traditional)
Unmet need among all unmarried women
Total demand
100%
80%
60%
40%
20%
0%
1970 1975 1980 1985 1990 1995 2000 2005 2010 2015 2020 2025 2030
F
DHS
MICS
Other international survey
National survey
PMA
Subpopulation
+: Higher contraceptive use
−: Lower contraceptive use
A: Other age group
F: Folk methods included
S−: Sterilization included
S+: Sterilization excluded
Unmarried women
Formerly married
Partnered women
CP (any) ≤ 1%

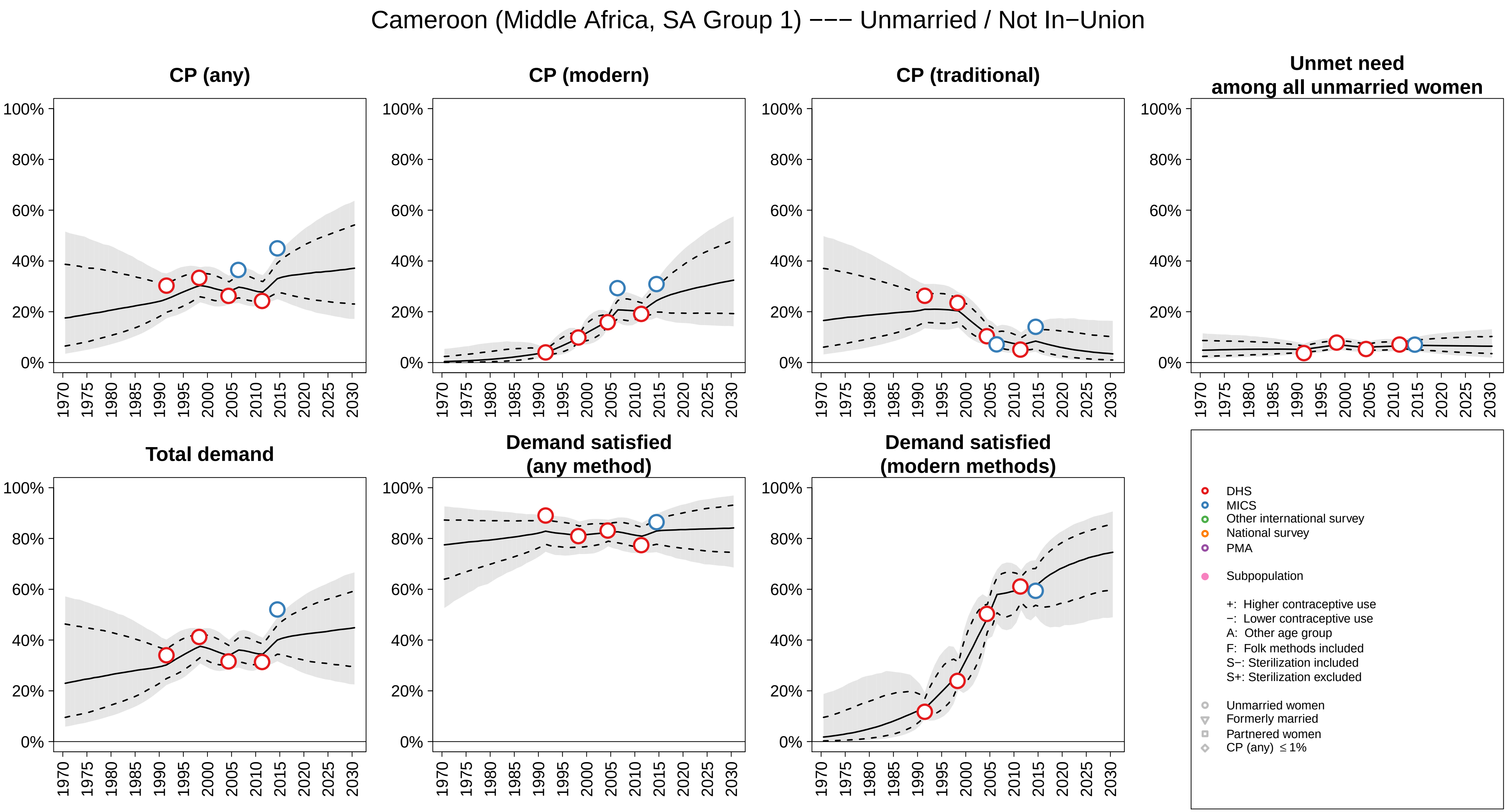

Cameroon (Middle Africa, SA Group 1) −−− Unmarried / Not In−Union
CP (any)
CP (modern)
CP (traditional)
Unmet need among all unmarried women
Total demand
Demand satisfied (any method)
Demand satisfied (modern methods)
0%
20%
40%
60%
80%
100%
1970
1975
1980
1985
1990
1995
2000
2005
2010
2015
2020
2025
2030
DHS
MICS
Other international survey
National survey
PMA
Subpopulation
+: Higher contraceptive use
−: Lower contraceptive use
A: Other age group
F: Folk methods included
S−: Sterilization included
S+: Sterilization excluded
Unmarried women
Formerly married
Partnered women
CP (any) ≤ 1%

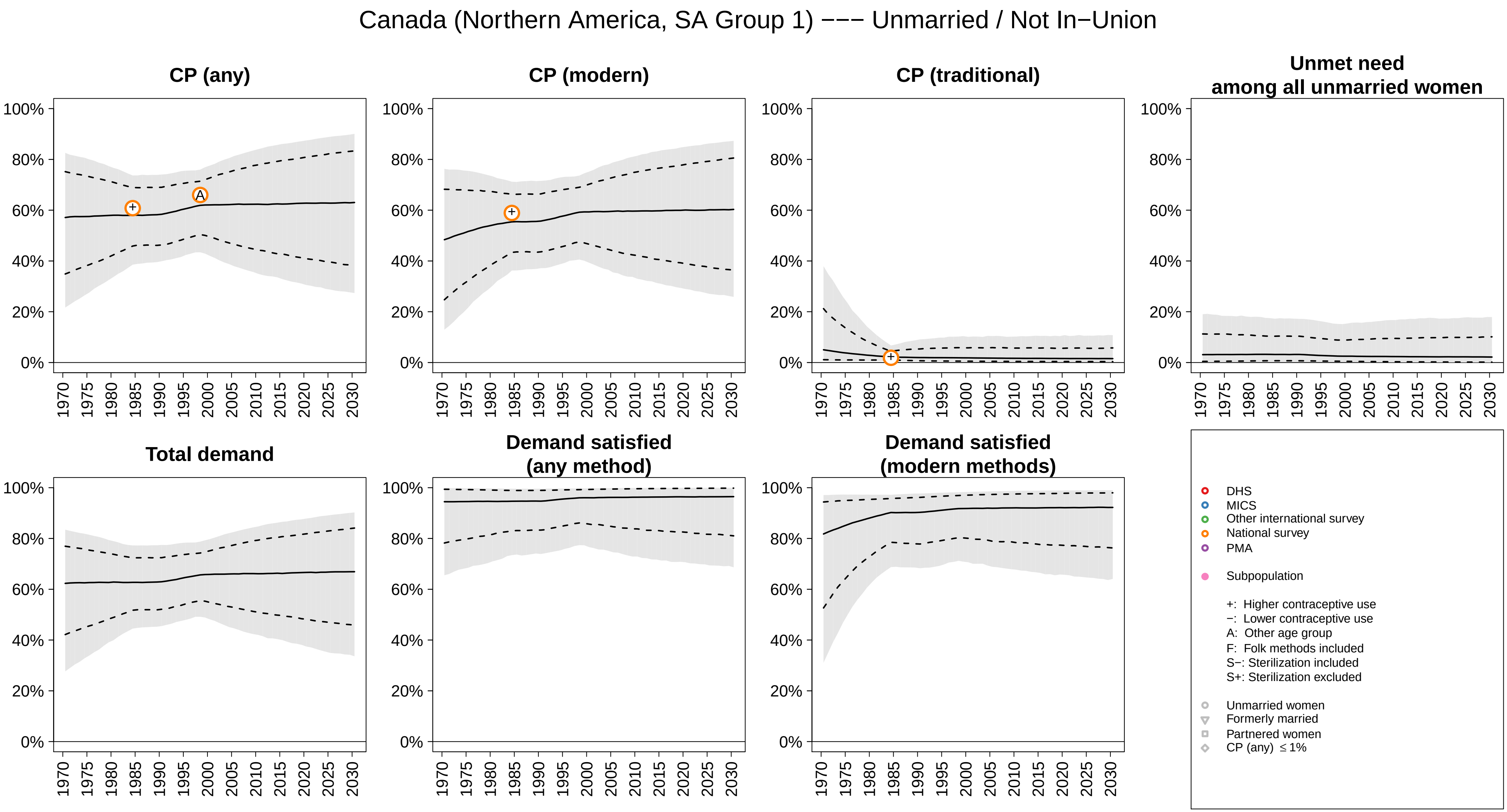
Canada (Northern America, SA Group 1) −−− Unmarried / Not In−Union
CP (any)
CP (modern)
CP (traditional)
Unmet need among all unmarried women
Total demand
Demand satisfied (any method)
Demand satisfied (modern methods)
0%
20%
40%
60%
80%
100%
1970
1975
1980
1985
1990
1995
2000
2005
2010
2015
2020
2025
2030
DHS
MICS
Other international survey
National survey
PMA
Subpopulation
+: Higher contraceptive use
−: Lower contraceptive use
A: Other age group
F: Folk methods included
S−: Sterilization included
S+: Sterilization excluded
Unmarried women
Formerly married
Partnered women
CP (any) ≤ 1%

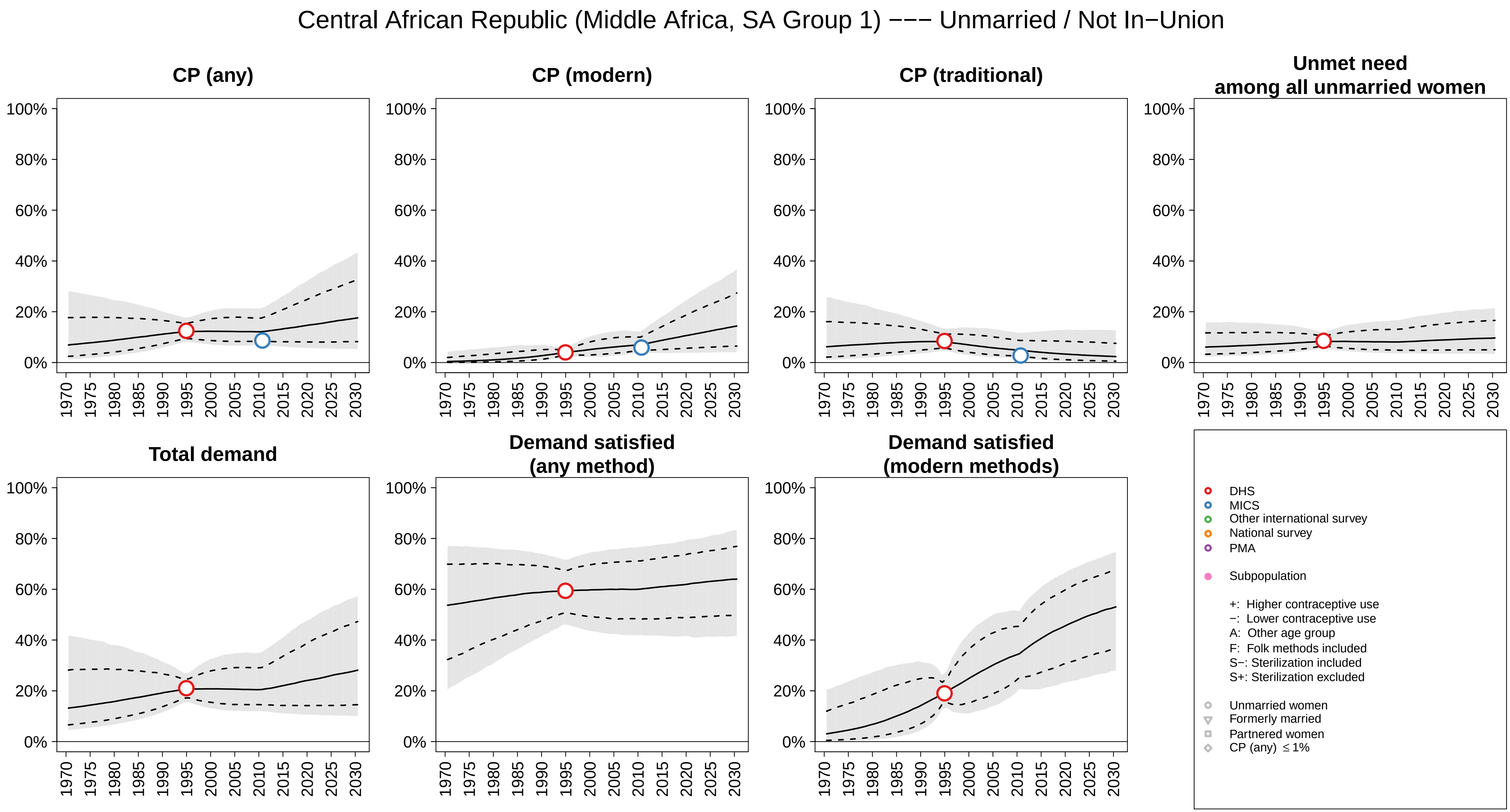
Central African Republic (Middle Africa, SA Group 1) −−− Unmarried / Not In−Union
CP (any)
CP (modern)
CP (traditional)
Unmet need among all unmarried women
Total demand
Demand satisfied (any method)
Demand satisfied (modern methods)
100%
80%
60%
40%
20%
0%
1970 1975 1980 1985 1990 1995 2000 2005 2010 2015 2020 2025 2030
DHS
MICS
Other international survey
National survey
PMA
Subpopulation
+: Higher contraceptive use
−: Lower contraceptive use
A: Other age group
F: Folk methods included
S−: Sterilization included
S+: Sterilization excluded
Unmarried women
Formerly married
Partnered women
CP (any) ≤ 1%

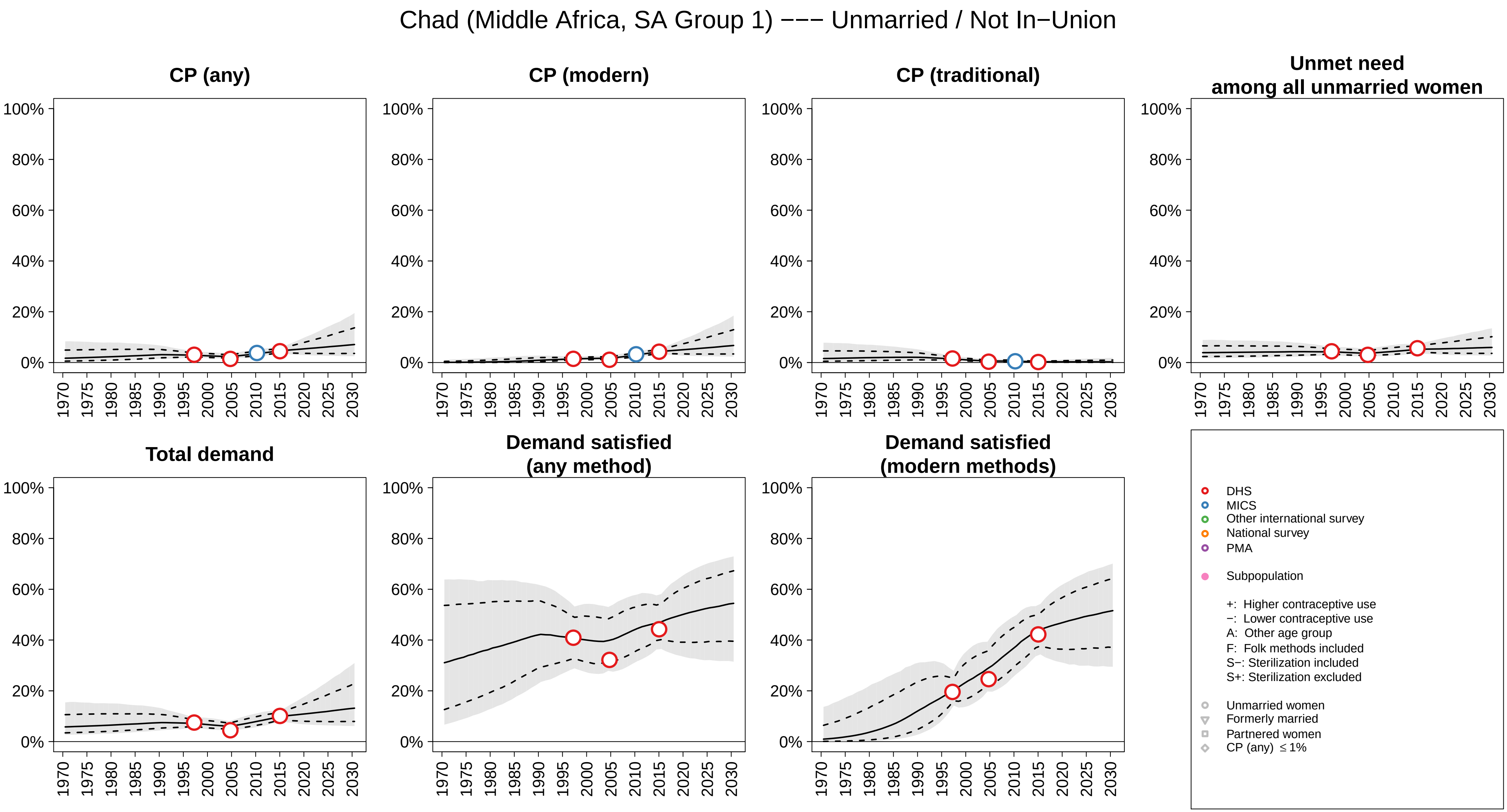
Chad (Middle Africa, SA Group 1) −−− Unmarried / Not In−Union
CP (any)
CP (modern)
CP (traditional)
Unmet need among all unmarried women
Total demand
Demand satisfied (any method)
Demand satisfied (modern methods)
100%
80%
60%
40%
20%
0%
1970
1975
1980
1985
1990
1995
2000
2005
2010
2015
2020
2025
2030
DHS
MICS
Other international survey
National survey
PMA
Subpopulation
+: Higher contraceptive use
−: Lower contraceptive use
A: Other age group
F: Folk methods included
S−: Sterilization included
S+: Sterilization excluded
Unmarried women
Formerly married
Partnered women
CP (any) ≤ 1%

Chile (South America, SA Group 1) −−− Unmarried / Not In−Union

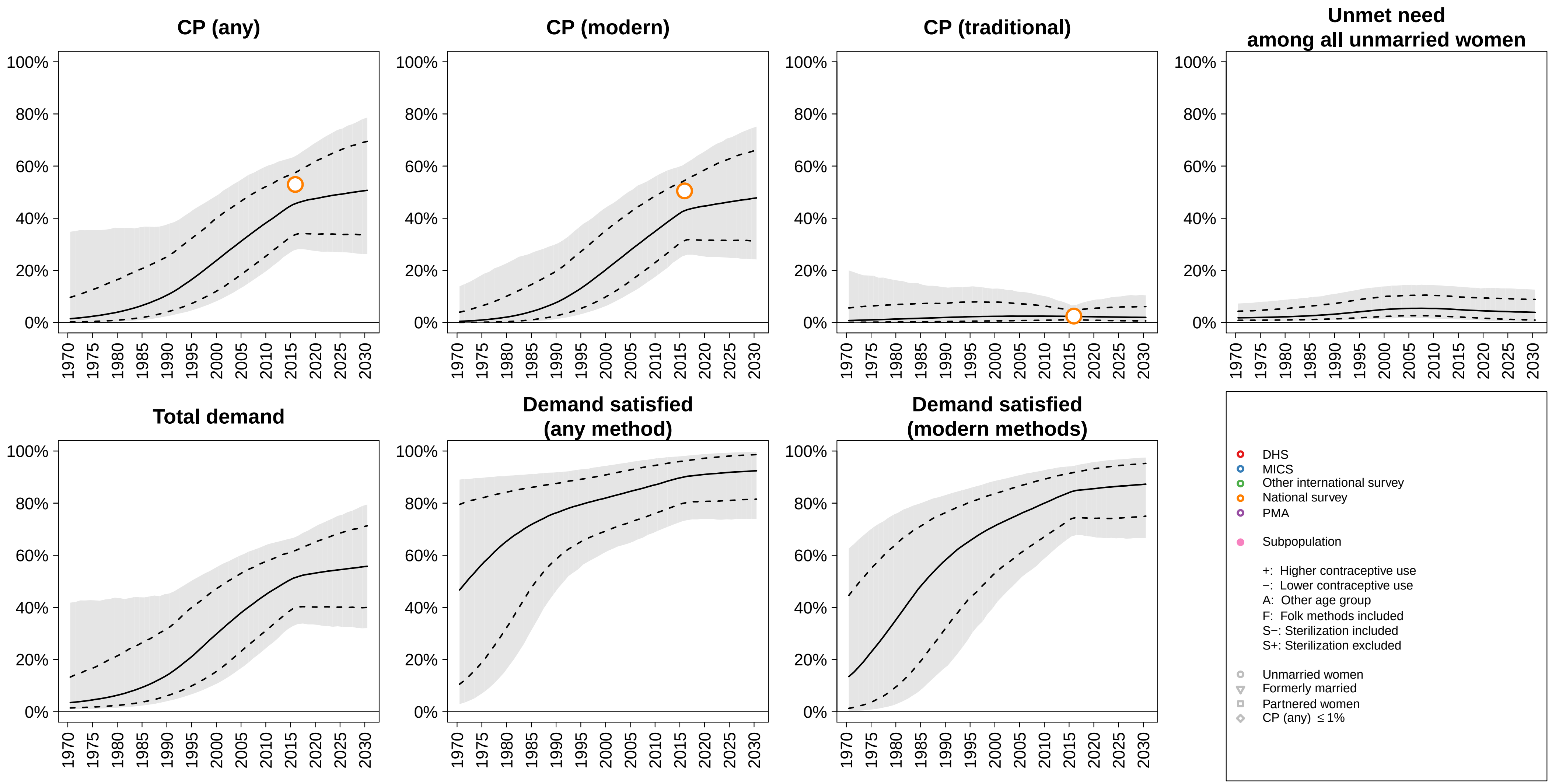

China (Eastern Asia, SA Group 1) −−− Unmarried / Not In−Union

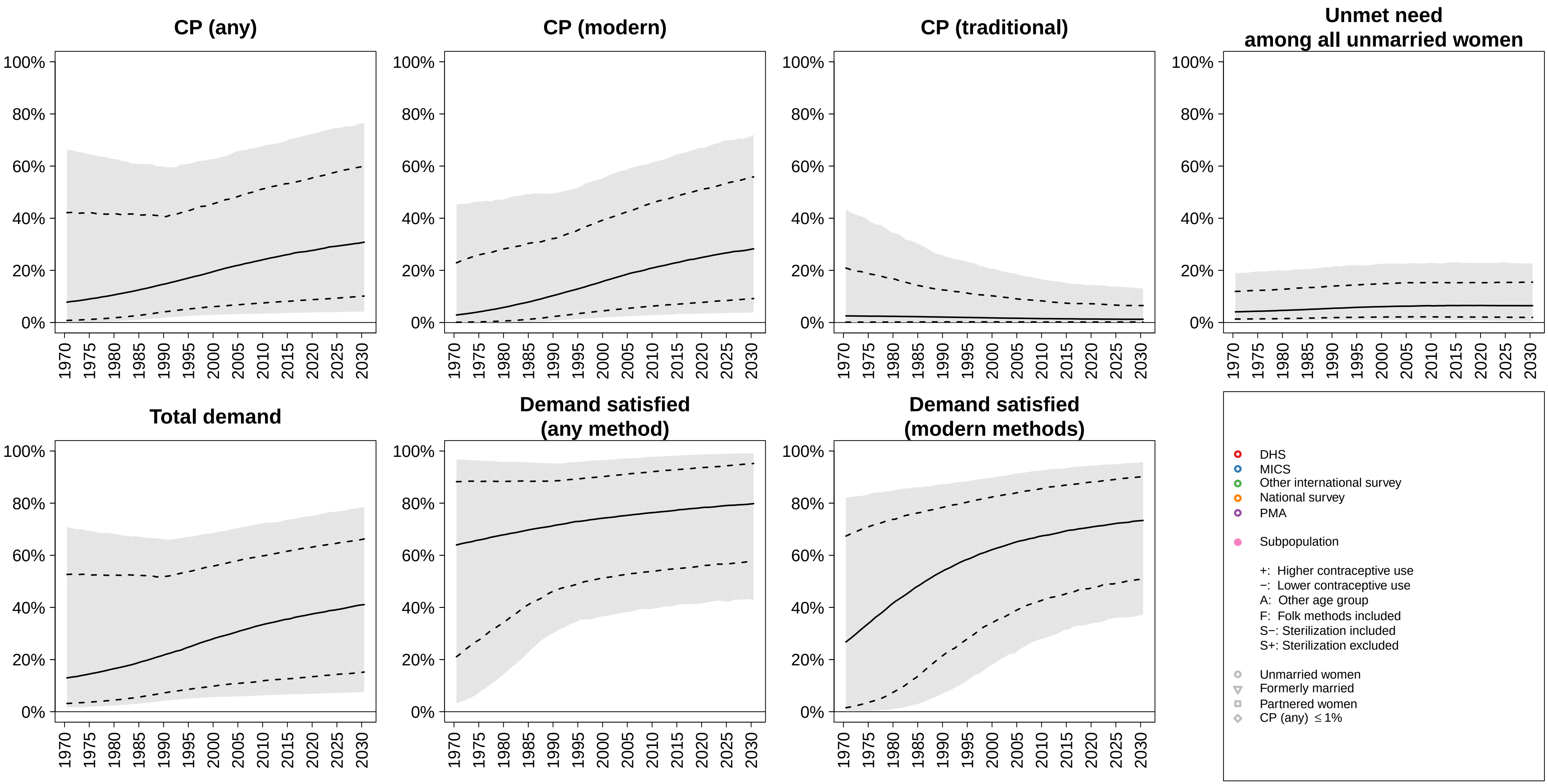

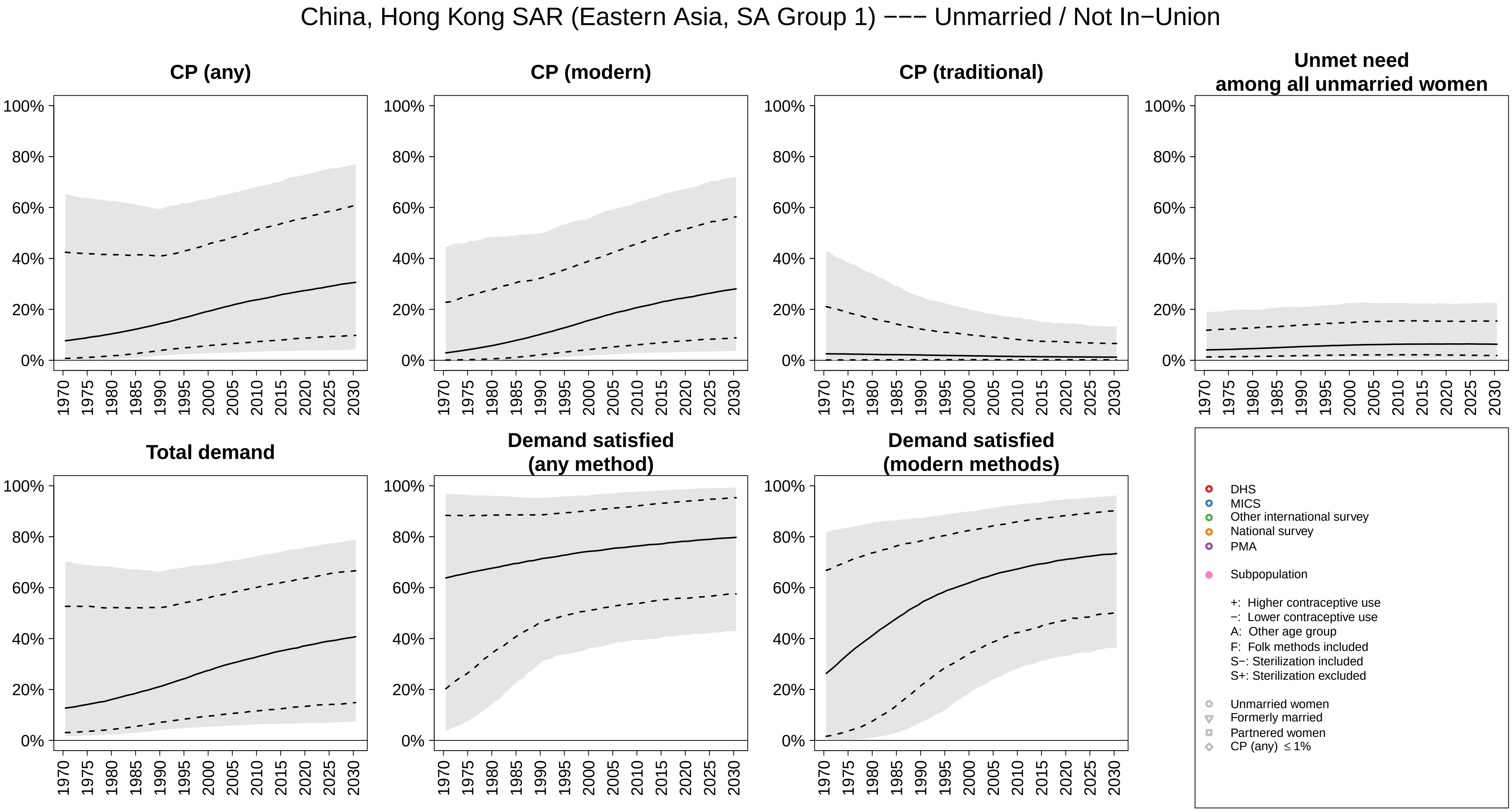
China, Hong Kong SAR (Eastern Asia, SA Group 1) −−− Unmarried / Not In−Union
CP (any)
CP (modern)
CP (traditional)
Unmet need among all unmarried women
Total demand
Demand satisfied (any method)
Demand satisfied (modern methods)
0%
20%
40%
60%
80%
100%
1970
1975
1980
1985
1990
1995
2000
2005
2010
2015
2020
2025
2030
DHS
MICS
Other international survey
National survey
PMA
Subpopulation
+: Higher contraceptive use
−: Lower contraceptive use
A: Other age group
F: Folk methods included
S−: Sterilization included
S+: Sterilization excluded
Unmarried women
Formerly married
Partnered women
CP (any) ≤ 1%

Colombia (South America, SA Group 1) −−− Unmarried / Not In−Union

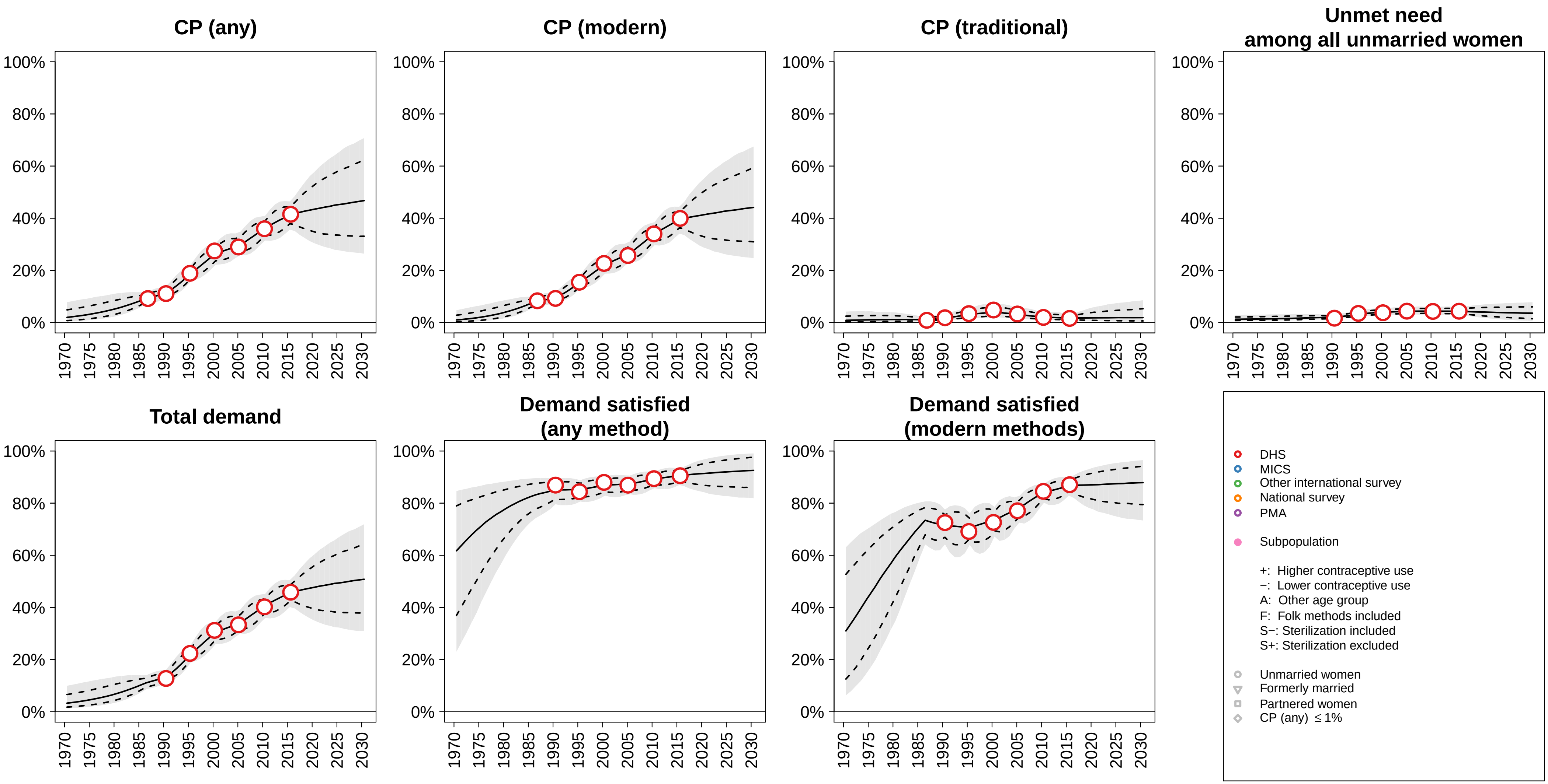

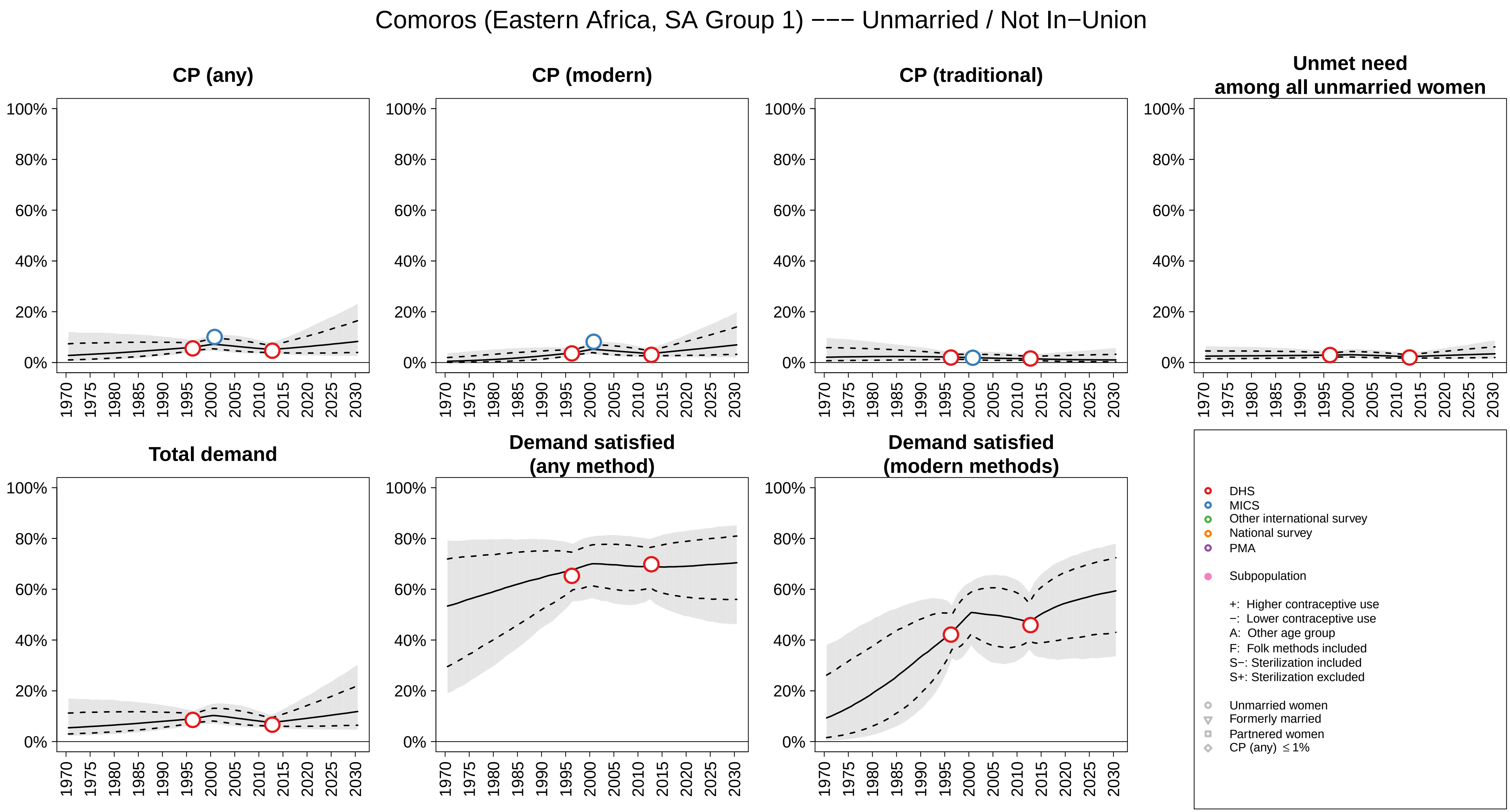
Comoros (Eastern Africa, SA Group 1) −−− Unmarried / Not In−Union
CP (any)
CP (modern)
CP (traditional)
Unmet need among all unmarried women
Total demand
Demand satisfied (any method)
Demand satisfied (modern methods)
100%
80%
60%
40%
20%
0%
1970
1975
1980
1985
1990
1995
2000
2005
2010
2015
2020
2025
2030
DHS
MICS
Other international survey
National survey
PMA
Subpopulation
+: Higher contraceptive use
−: Lower contraceptive use
A: Other age group
F: Folk methods included
S−: Sterilization included
S+: Sterilization excluded
Unmarried women
Formerly married
Partnered women
CP (any) ≤ 1%

Congo (Middle Africa, SA Group 1) --- Unmarried / Not In−Union

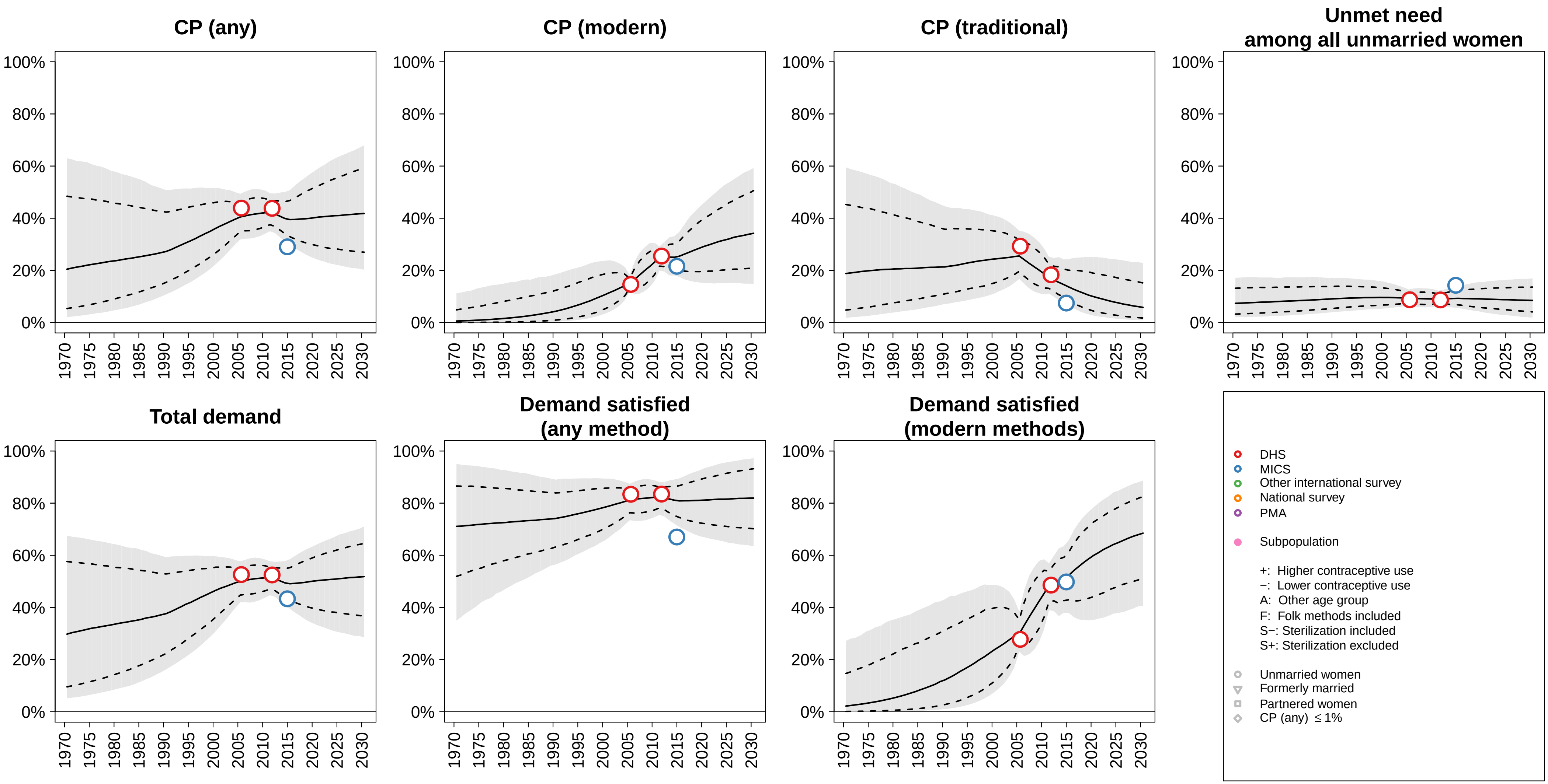

Costa Rica (Central America, SA Group 1) −−− Unmarried / Not In−Union

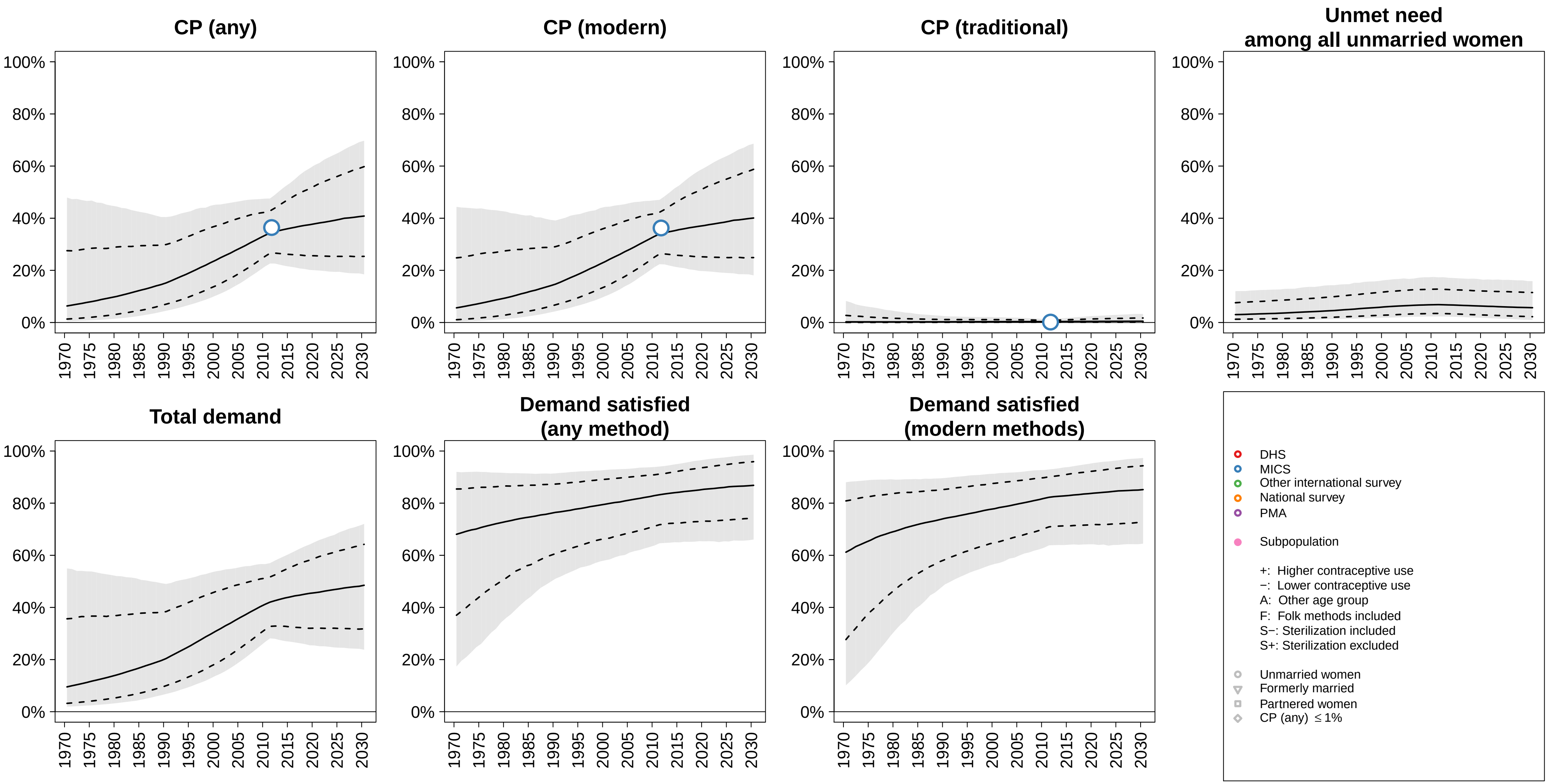

Côte d’Ivoire (Western Africa, SA Group 1) −−− Unmarried / Not In−Union

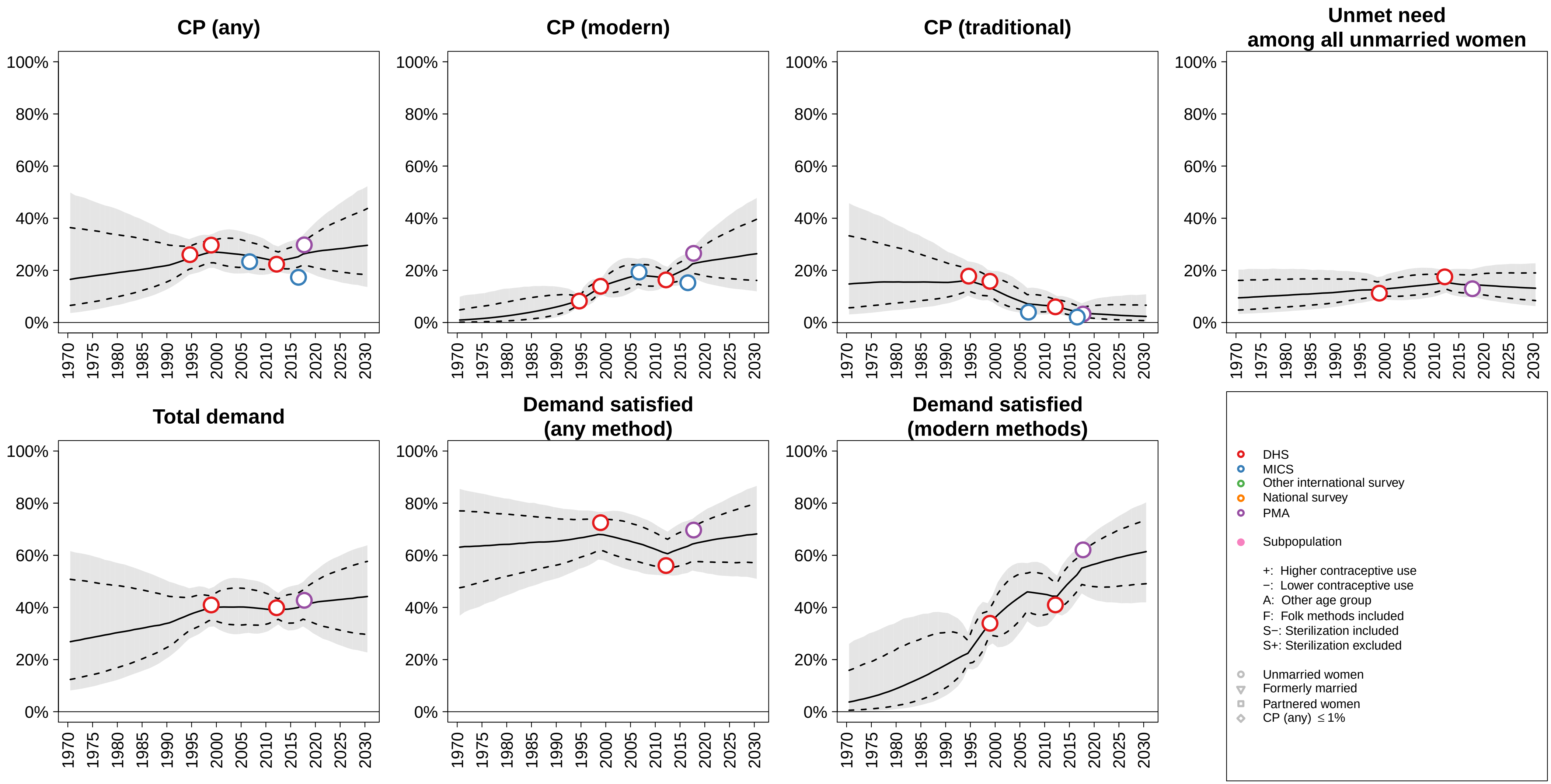

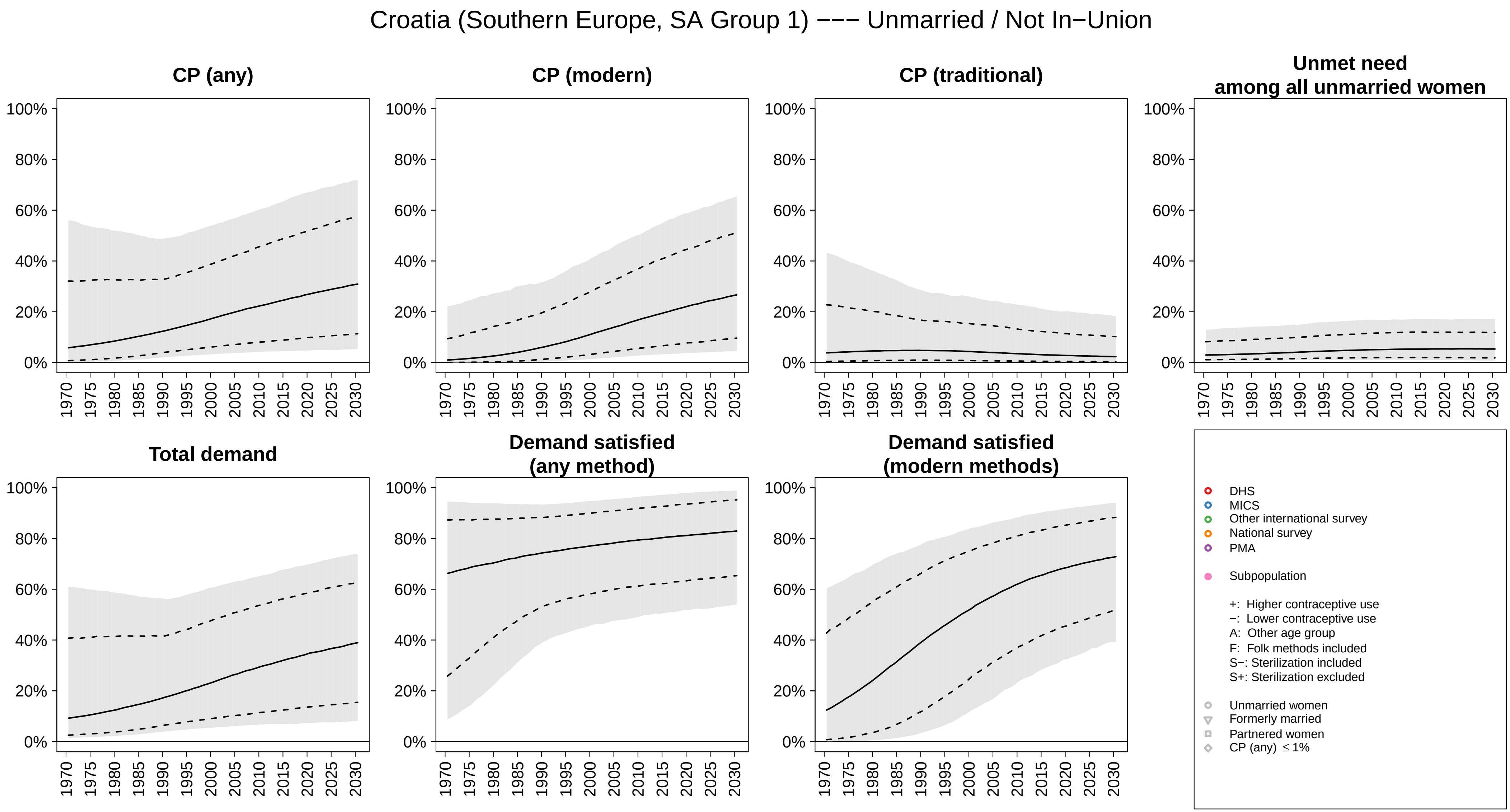
Croatia (Southern Europe, SA Group 1) −−− Unmarried / Not In−Union
CP (any)
CP (modern)
CP (traditional)
Unmet need among all unmarried women
Total demand
Demand satisfied (any method)
Demand satisfied (modern methods)
100%
80%
60%
40%
20%
0%
1970
1975
1980
1985
1990
1995
2000
2005
2010
2015
2020
2025
2030
DHS
MICS
Other international survey
National survey
PMA
Subpopulation
+: Higher contraceptive use
−: Lower contraceptive use
A: Other age group
F: Folk methods included
S−: Sterilization included
S+: Sterilization excluded
Unmarried women
Formerly married
Partnered women
CP (any) ≤ 1%

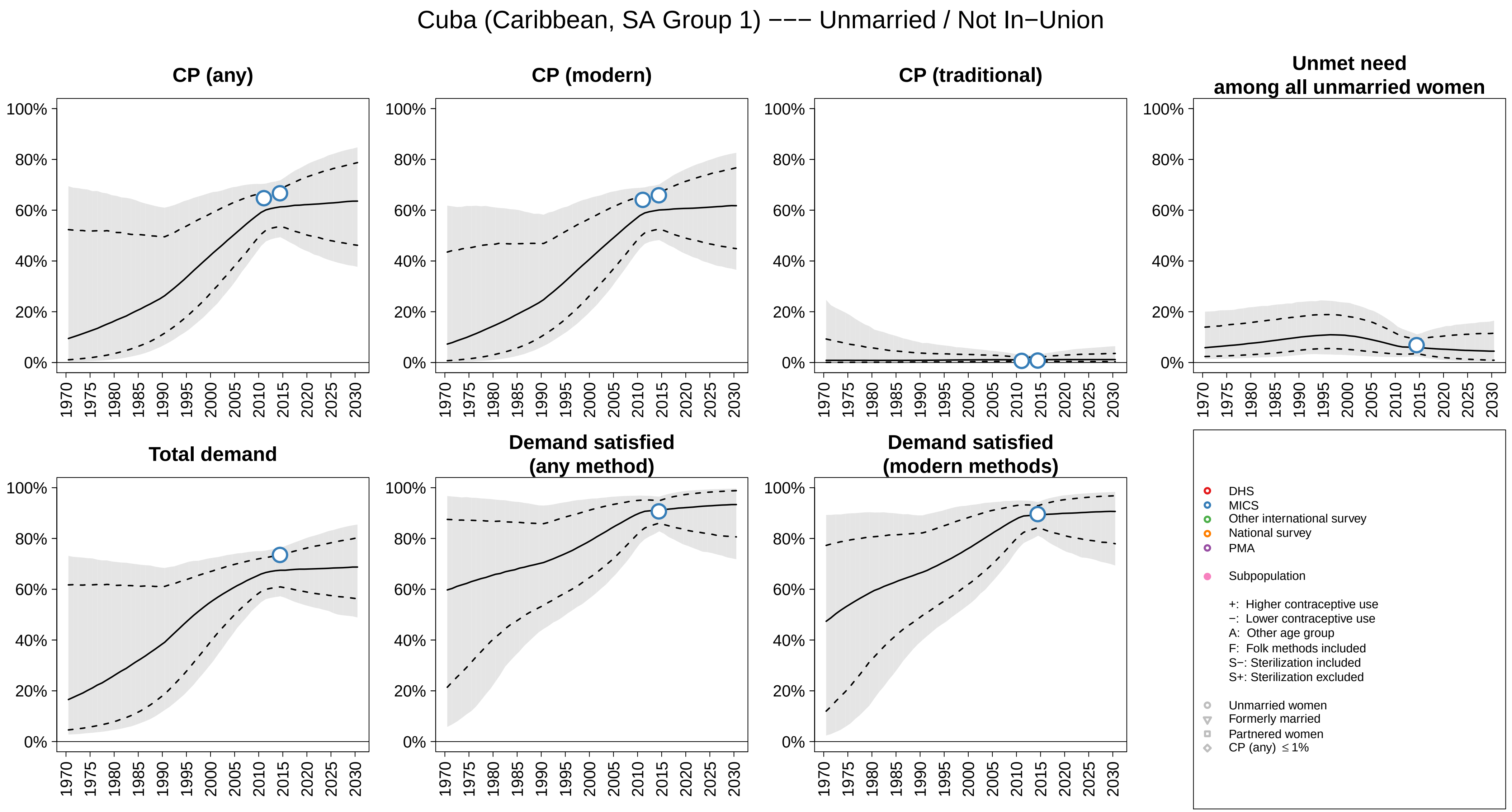
Cuba (Caribbean, SA Group 1) −−− Unmarried / Not In−Union
CP (any)
CP (modern)
CP (traditional)
Unmet need among all unmarried women
Total demand
Demand satisfied (any method)
Demand satisfied (modern methods)
0%
20%
40%
60%
80%
100%
1970
1975
1980
1985
1990
1995
2000
2005
2010
2015
2020
2025
2030
DHS
MICS
Other international survey
National survey
PMA
Subpopulation
+: Higher contraceptive use
−: Lower contraceptive use
A: Other age group
F: Folk methods included
S−: Sterilization included
S+: Sterilization excluded
Unmarried women
Formerly married
Partnered women
CP (any) ≤ 1%

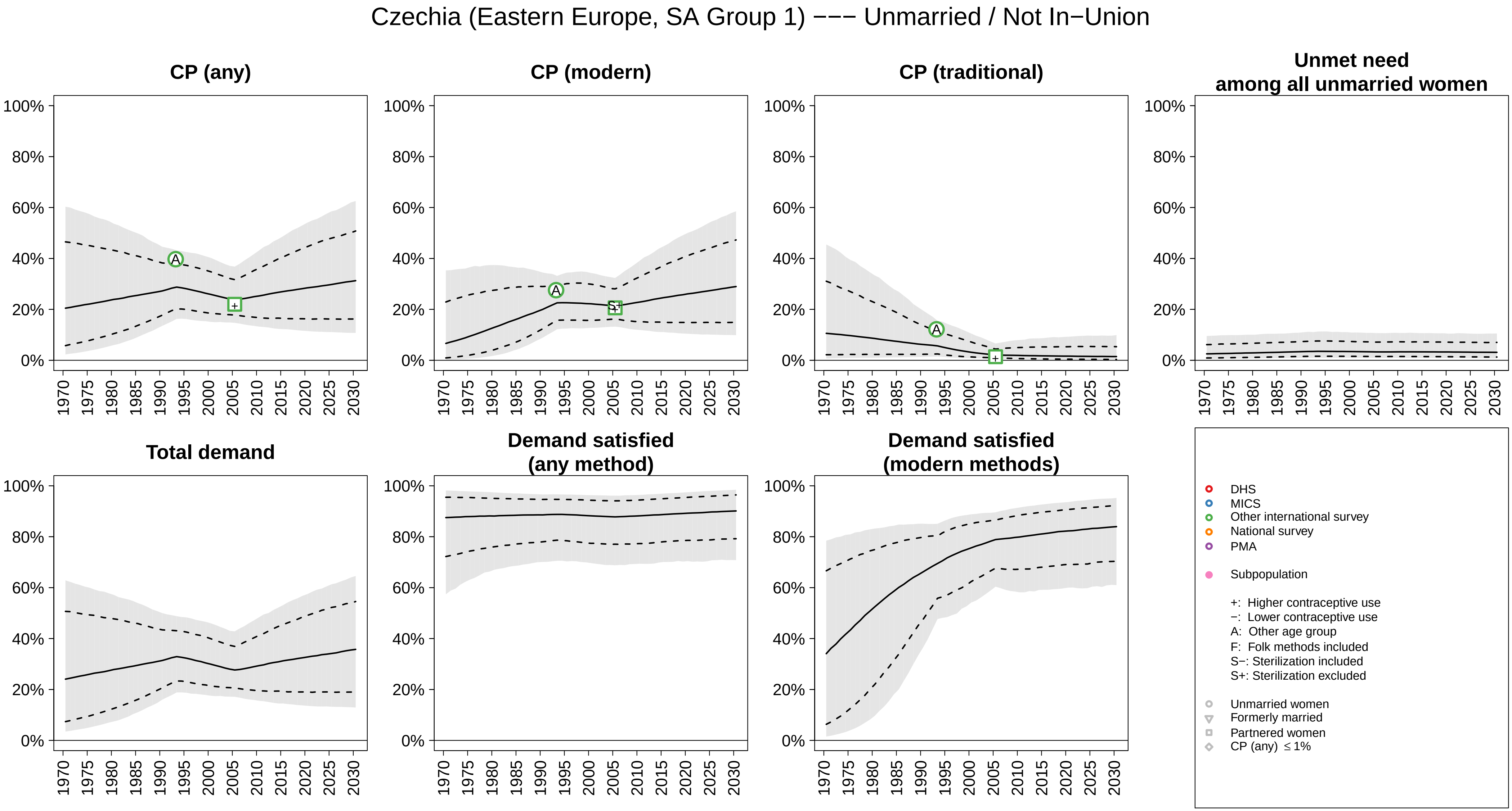
Czechia (Eastern Europe, SA Group 1) −−− Unmarried / Not In−Union
CP (any)
CP (modern)
CP (traditional)
Unmet need among all unmarried women
Total demand
Demand satisfied (any method)
Demand satisfied (modern methods)
0%
20%
40%
60%
80%
100%
1970
1975
1980
1985
1990
1995
2000
2005
2010
2015
2020
2025
2030
DHS
MICS
Other international survey
National survey
PMA
Subpopulation
+: Higher contraceptive use
−: Lower contraceptive use
A: Other age group
F: Folk methods included
S−: Sterilization included
S+: Sterilization excluded
Unmarried women
Formerly married
Partnered women
CP (any) ≤ 1%

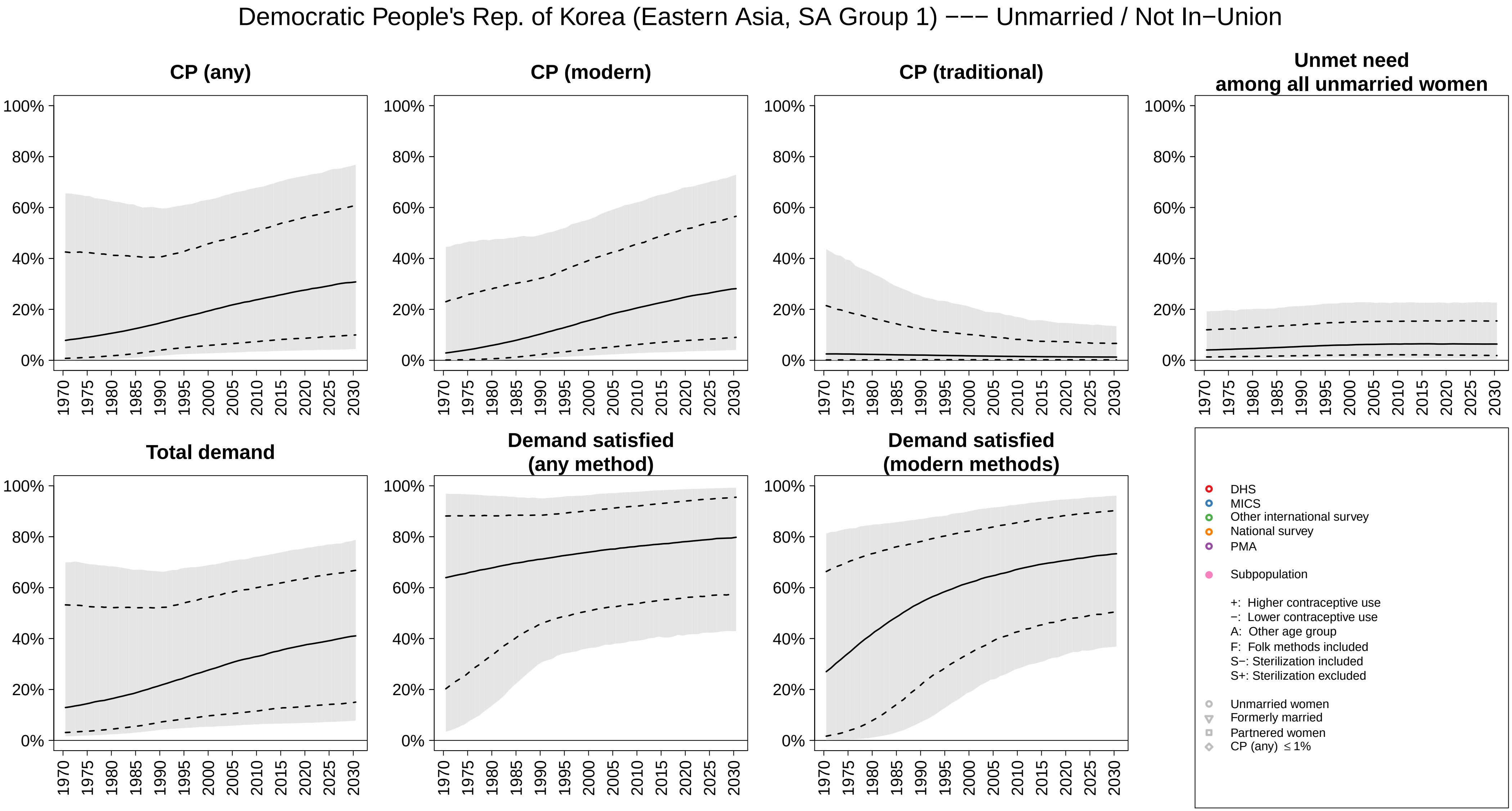
Democratic People's Rep. of Korea (Eastern Asia, SA Group 1) −−− Unmarried / Not In−Union
CP (any)
CP (modern)
CP (traditional)
Unmet need among all unmarried women
Total demand
Demand satisfied (any method)
Demand satisfied (modern methods)
0%
20%
40%
60%
80%
100%
1970
1975
1980
1985
1990
1995
2000
2005
2010
2015
2020
2025
2030
DHS
MICS
Other international survey
National survey
PMA
Subpopulation
+: Higher contraceptive use
−: Lower contraceptive use
A: Other age group
F: Folk methods included
S−: Sterilization included
S+: Sterilization excluded
Unmarried women
Formerly married
Partnered women
CP (any) ≤ 1%

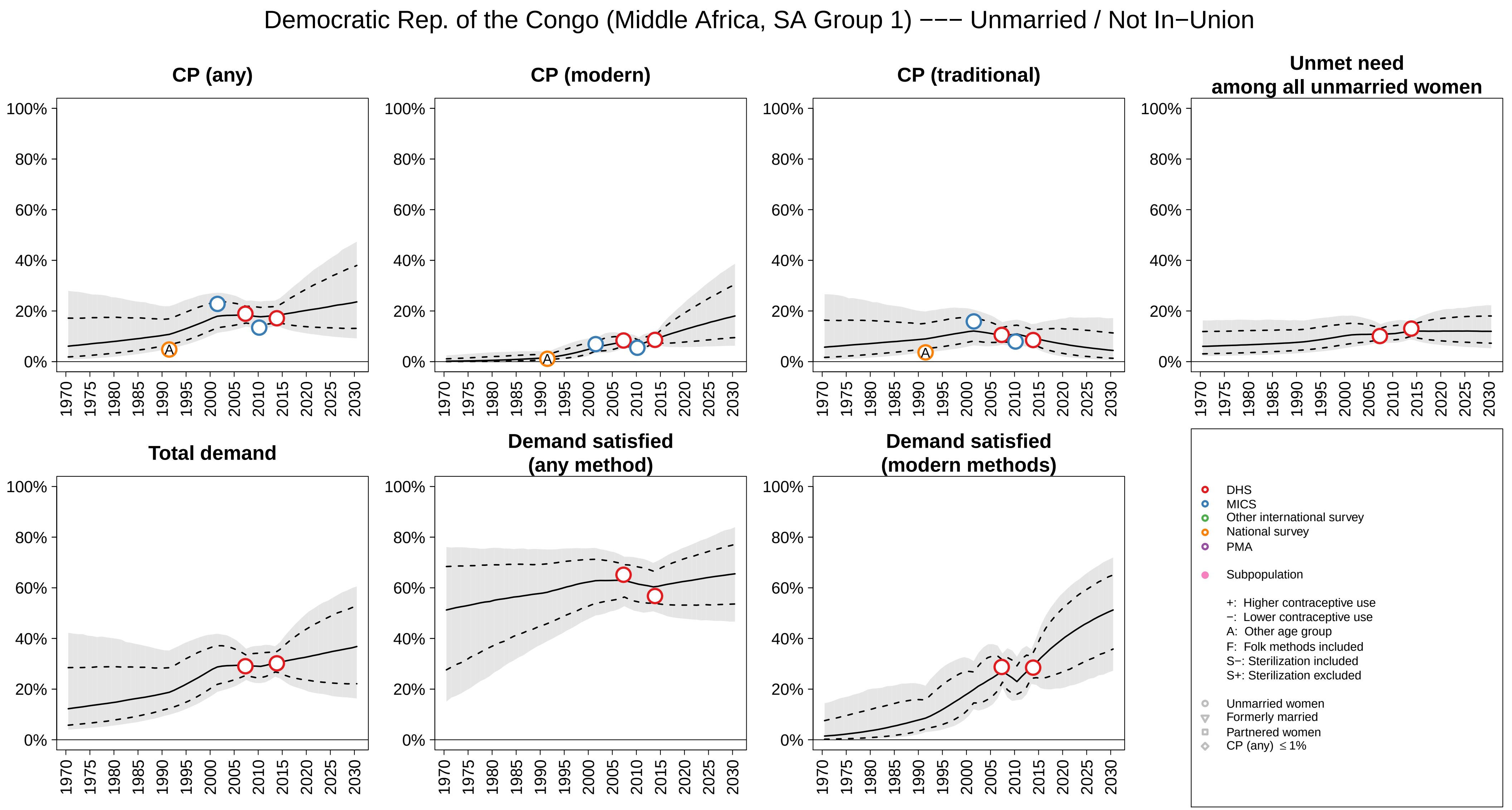
Democratic Rep. of the Congo (Middle Africa, SA Group 1) ––– Unmarried / Not In−Union
CP (any)
CP (modern)
CP (traditional)
Unmet need among all unmarried women
Total demand
Demand satisfied (any method)
Demand satisfied (modern methods)
100%
80%
60%
40%
20%
0%
1970
1975
1980
1985
1990
1995
2000
2005
2010
2015
2020
2025
2030
A
DHS
MICS
Other international survey
National survey
PMA
Subpopulation
+: Higher contraceptive use
−: Lower contraceptive use
A: Other age group
F: Folk methods included
S−: Sterilization included
S+: Sterilization excluded
Unmarried women
Formerly married
Partnered women
CP (any) ≤ 1%

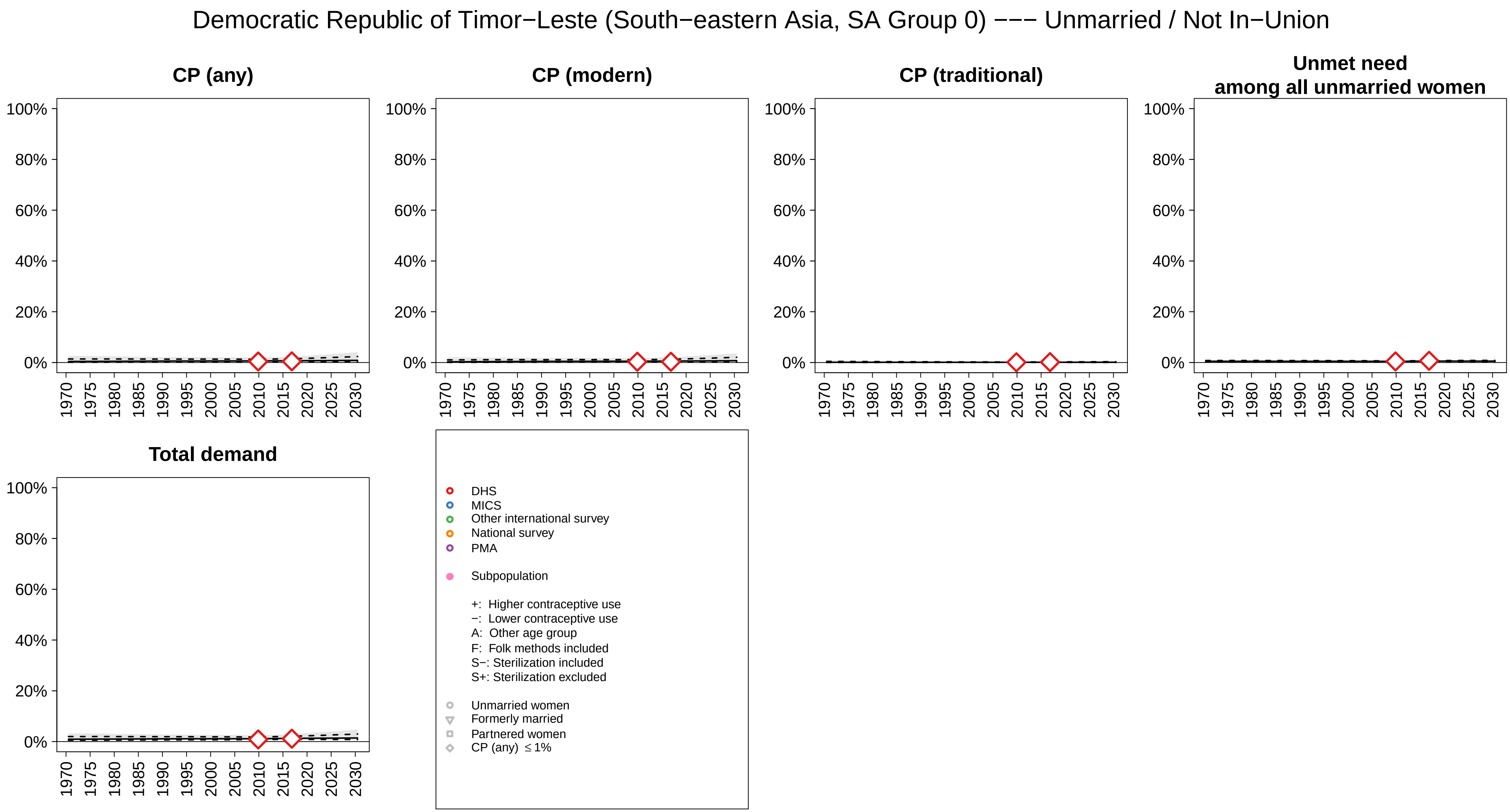
Democratic Republic of Timor−Leste (South−eastern Asia, SA Group 0) −−− Unmarried / Not In−Union
CP (any)
CP (modern)
CP (traditional)
Unmet need among all unmarried women
Total demand
100%
80%
60%
40%
20%
0%
1970
1975
1980
1985
1990
1995
2000
2005
2010
2015
2020
2025
2030
DHS
MICS
Other international survey
National survey
PMA
Subpopulation
+: Higher contraceptive use
−: Lower contraceptive use
A: Other age group
F: Folk methods included
S−: Sterilization included
S+: Sterilization excluded
Unmarried women
Formerly married
Partnered women
CP (any) ≤ 1%

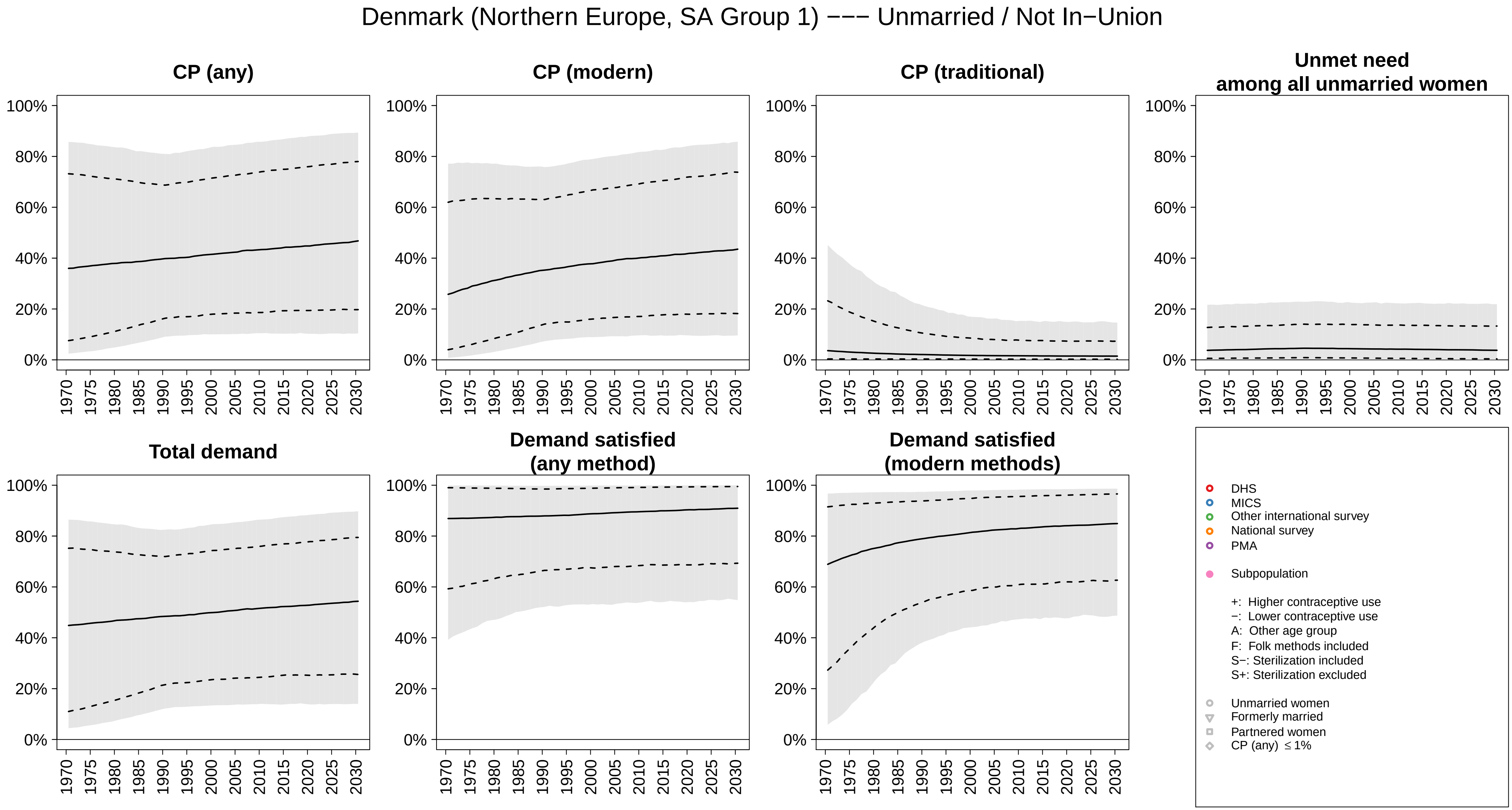
Denmark (Northern Europe, SA Group 1) −−− Unmarried / Not In−Union
CP (any)
CP (modern)
CP (traditional)
Unmet need among all unmarried women
Total demand
Demand satisfied (any method)
Demand satisfied (modern methods)
DHS
MICS
Other international survey
National survey
PMA
Subpopulation
+: Higher contraceptive use
−: Lower contraceptive use
A: Other age group
F: Folk methods included
S−: Sterilization included
S+: Sterilization excluded
Unmarried women
Formerly married
Partnered women
CP (any) ≤ 1%

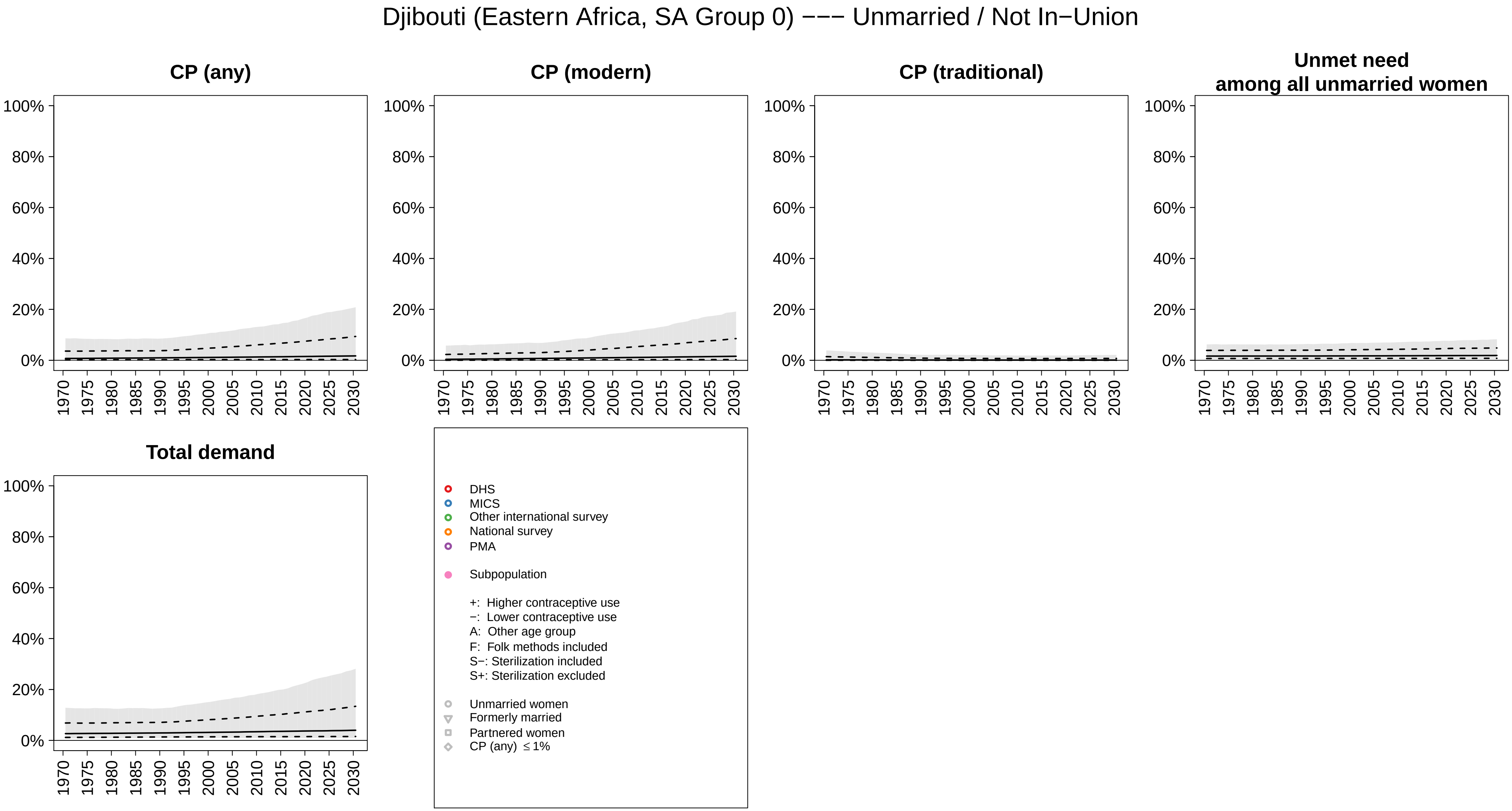
Djibouti (Eastern Africa, SA Group 0) −−− Unmarried / Not In−Union
CP (any)
CP (modern)
CP (traditional)
Unmet need among all unmarried women
Total demand
100%
80%
60%
40%
20%
0%
1970 1975 1980 1985 1990 1995 2000 2005 2010 2015 2020 2025 2030
DHS
MICS
Other international survey
National survey
PMA
Subpopulation
+: Higher contraceptive use
−: Lower contraceptive use
A: Other age group
F: Folk methods included
S−: Sterilization included
S+: Sterilization excluded
Unmarried women
Formerly married
Partnered women
CP (any) ≤ 1%

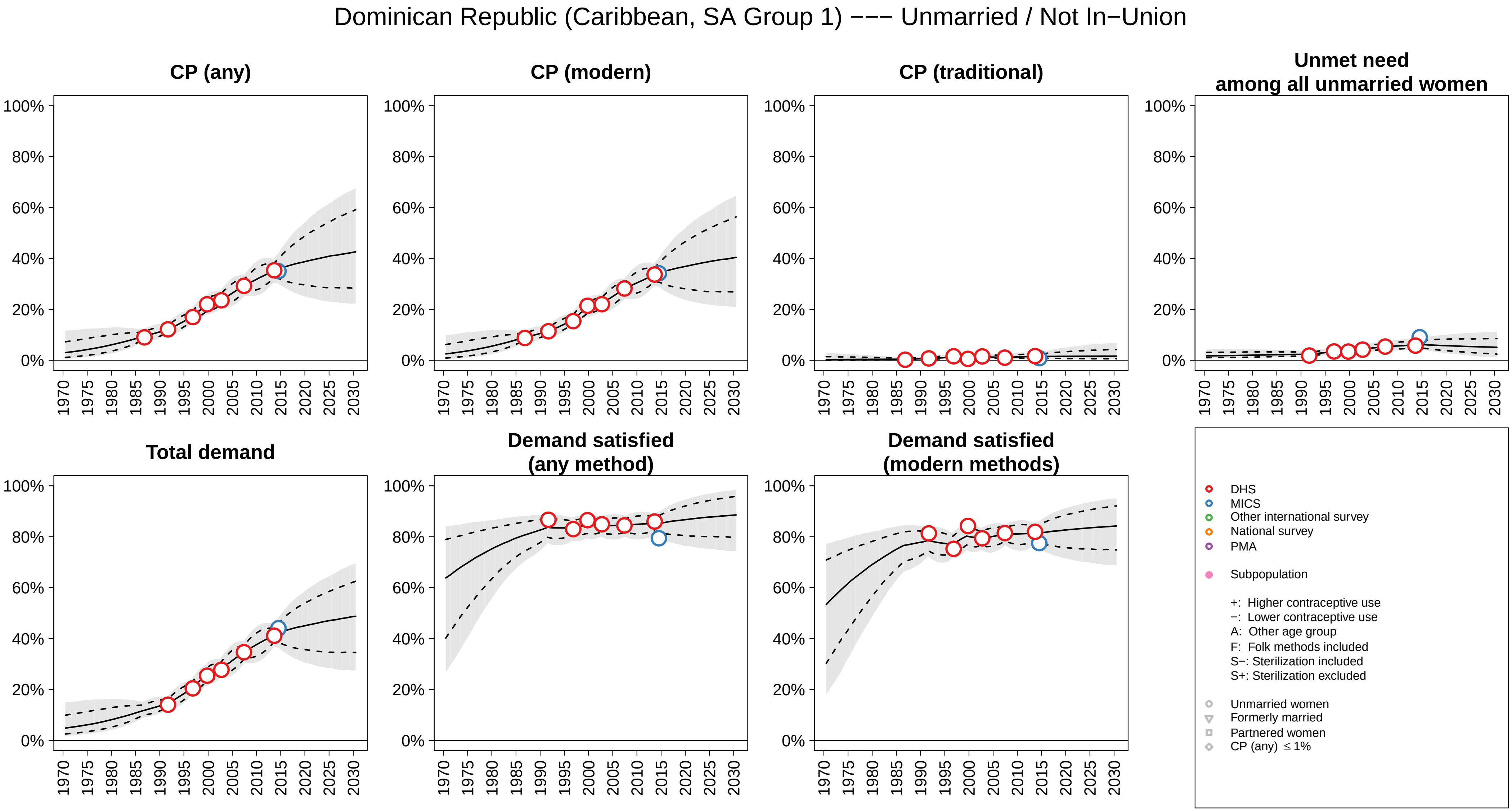
Dominican Republic (Caribbean, SA Group 1) --- Unmarried / Not In-Union
CP (any)
CP (modern)
CP (traditional)
Unmet need among all unmarried women
Total demand
Demand satisfied (any method)
Demand satisfied (modern methods)
100%
80%
60%
40%
20%
0%
1970 1975 1980 1985 1990 1995 2000 2005 2010 2015 2020 2025 2030
DHS
MICS
Other international survey
National survey
PMA
Subpopulation
+: Higher contraceptive use
−: Lower contraceptive use
A: Other age group
F: Folk methods included
S−: Sterilization included
S+: Sterilization excluded
Unmarried women
Formerly married
Partnered women
CP (any) ≤ 1%

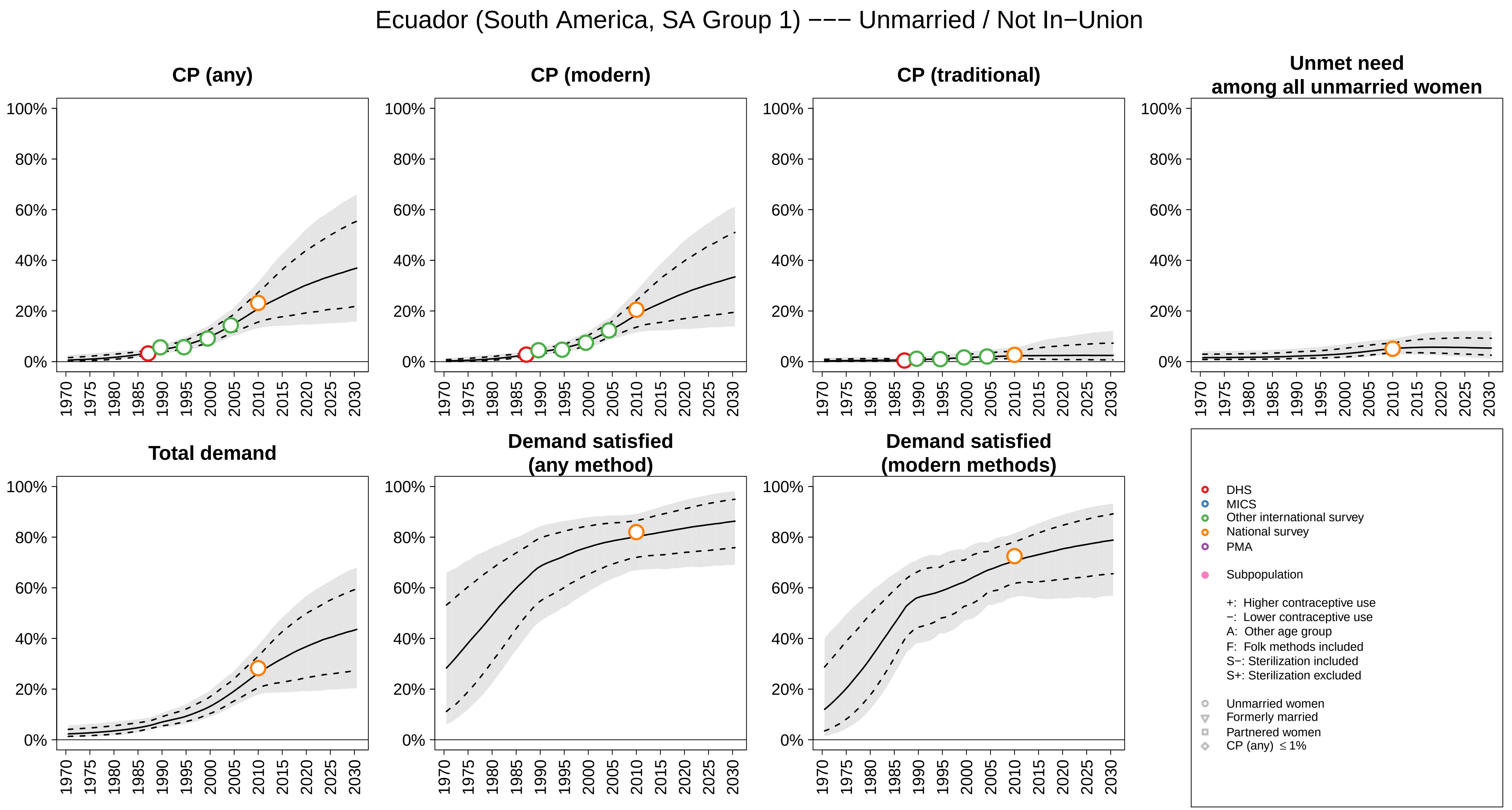
Ecuador (South America, SA Group 1) −−− Unmarried / Not In−Union
CP (any)
CP (modern)
CP (traditional)
Unmet need among all unmarried women
Total demand
Demand satisfied (any method)
Demand satisfied (modern methods)
100%
80%
60%
40%
20%
0%
1970 1975 1980 1985 1990 1995 2000 2005 2010 2015 2020 2025 2030
DHS
MICS
Other international survey
National survey
PMA
Subpopulation
+: Higher contraceptive use
−: Lower contraceptive use
A: Other age group
F: Folk methods included
S−: Sterilization included
S+: Sterilization excluded
Unmarried women
Formerly married
Partnered women
CP (any) ≤ 1%

Egypt (Northern Africa, SA Group 0) −−− Unmarried / Not In−Union

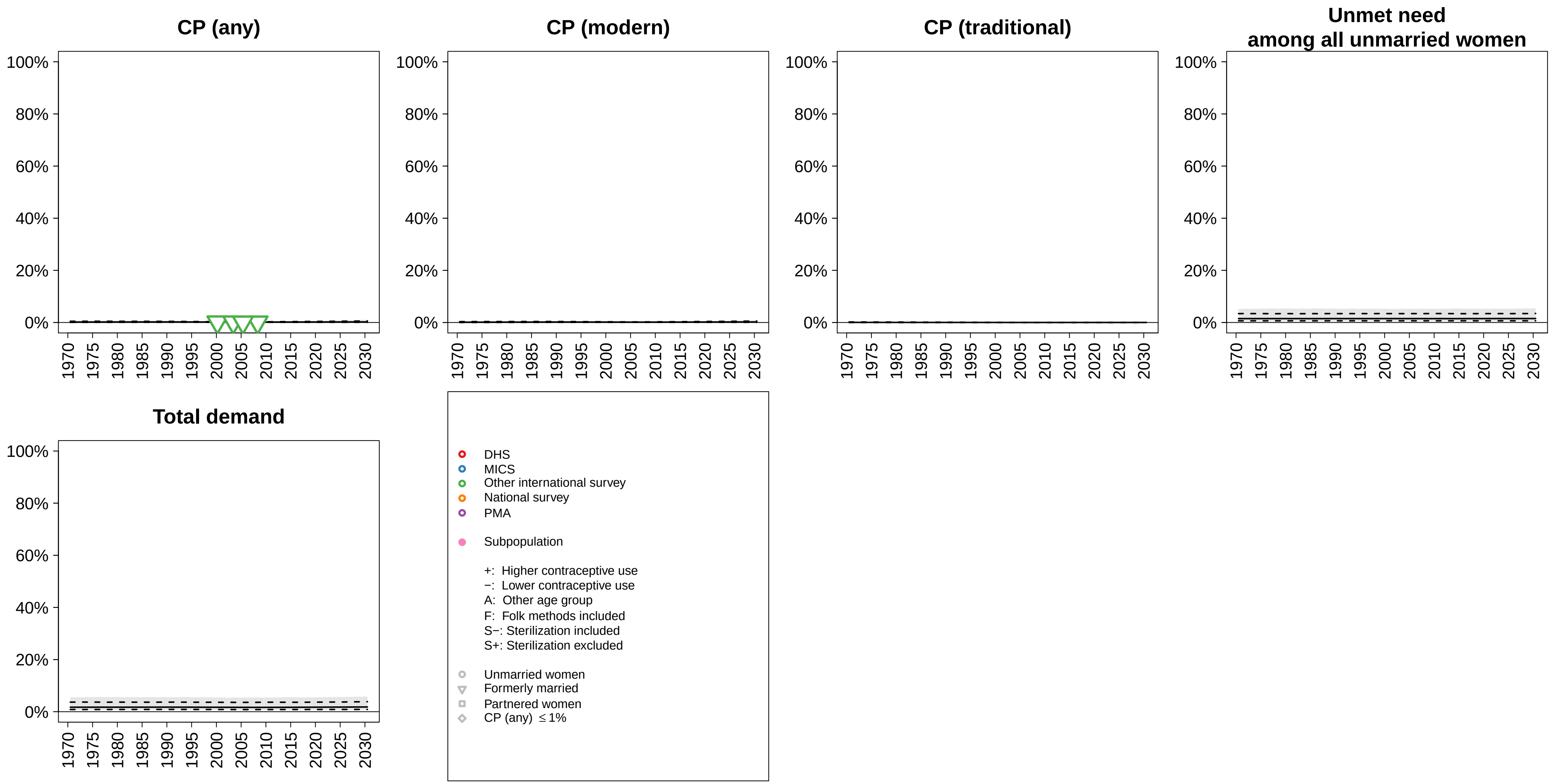

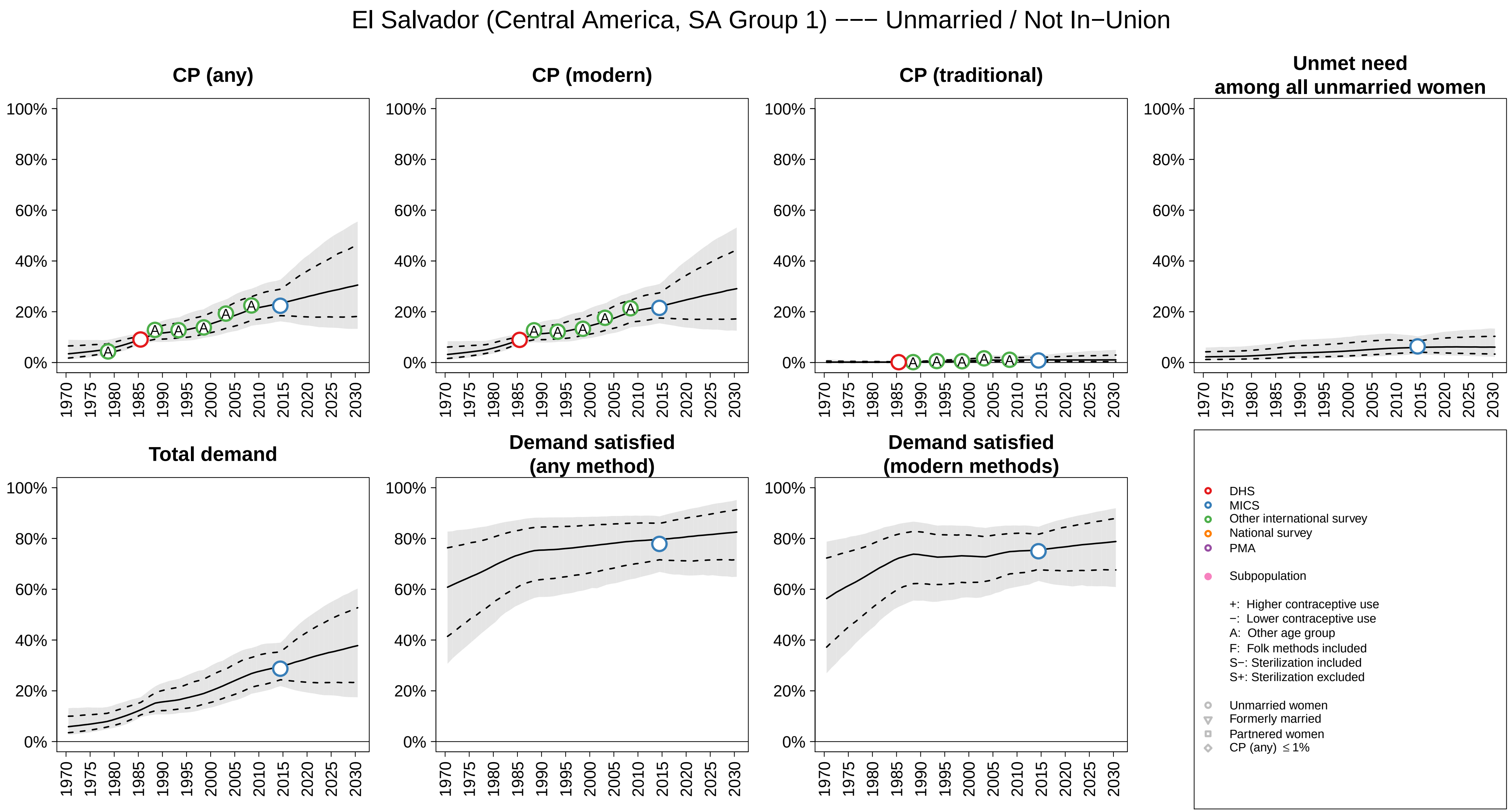
El Salvador (Central America, SA Group 1) −−− Unmarried / Not In−Union
CP (any)
CP (modern)
CP (traditional)
Unmet need among all unmarried women
Total demand
Demand satisfied (any method)
Demand satisfied (modern methods)
0%
20%
40%
60%
80%
100%
1970
1975
1980
1985
1990
1995
2000
2005
2010
2015
2020
2025
2030
A
DHS
MICS
Other international survey
National survey
PMA
Subpopulation
+: Higher contraceptive use
−: Lower contraceptive use
A: Other age group
F: Folk methods included
S−: Sterilization included
S+: Sterilization excluded
Unmarried women
Formerly married
Partnered women
CP (any) ≤ 1%

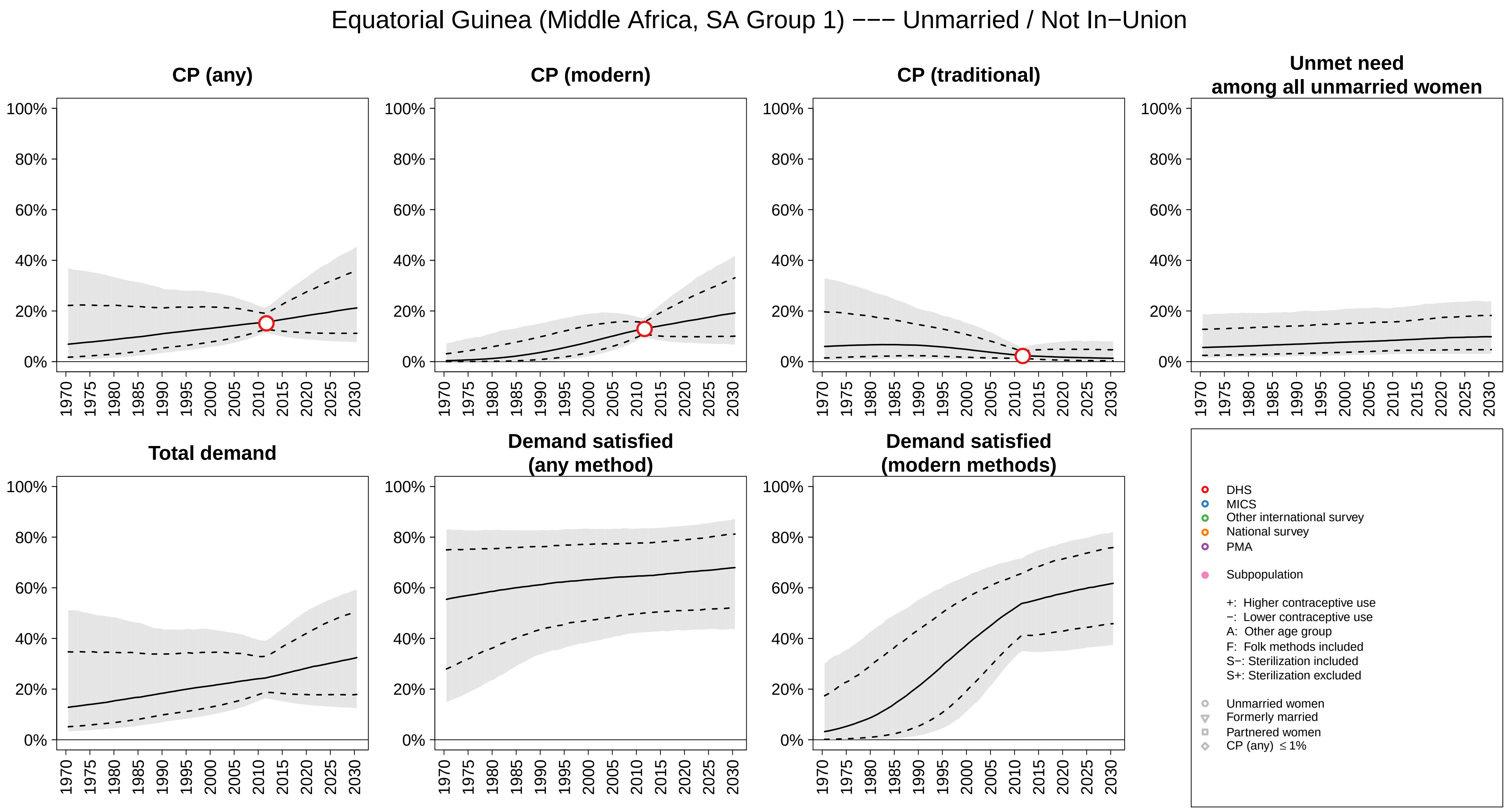
Equatorial Guinea (Middle Africa, SA Group 1) --- Unmarried / Not In−Union
CP (any)
CP (modern)
CP (traditional)
Unmet need among all unmarried women
Total demand
Demand satisfied (any method)
Demand satisfied (modern methods)
0%
20%
40%
60%
80%
100%
1970
1975
1980
1985
1990
1995
2000
2005
2010
2015
2020
2025
2030
DHS
MICS
Other international survey
National survey
PMA
Subpopulation
+: Higher contraceptive use
−: Lower contraceptive use
A: Other age group
F: Folk methods included
S−: Sterilization included
S+: Sterilization excluded
Unmarried women
Formerly married
Partnered women
CP (any) ≤ 1%

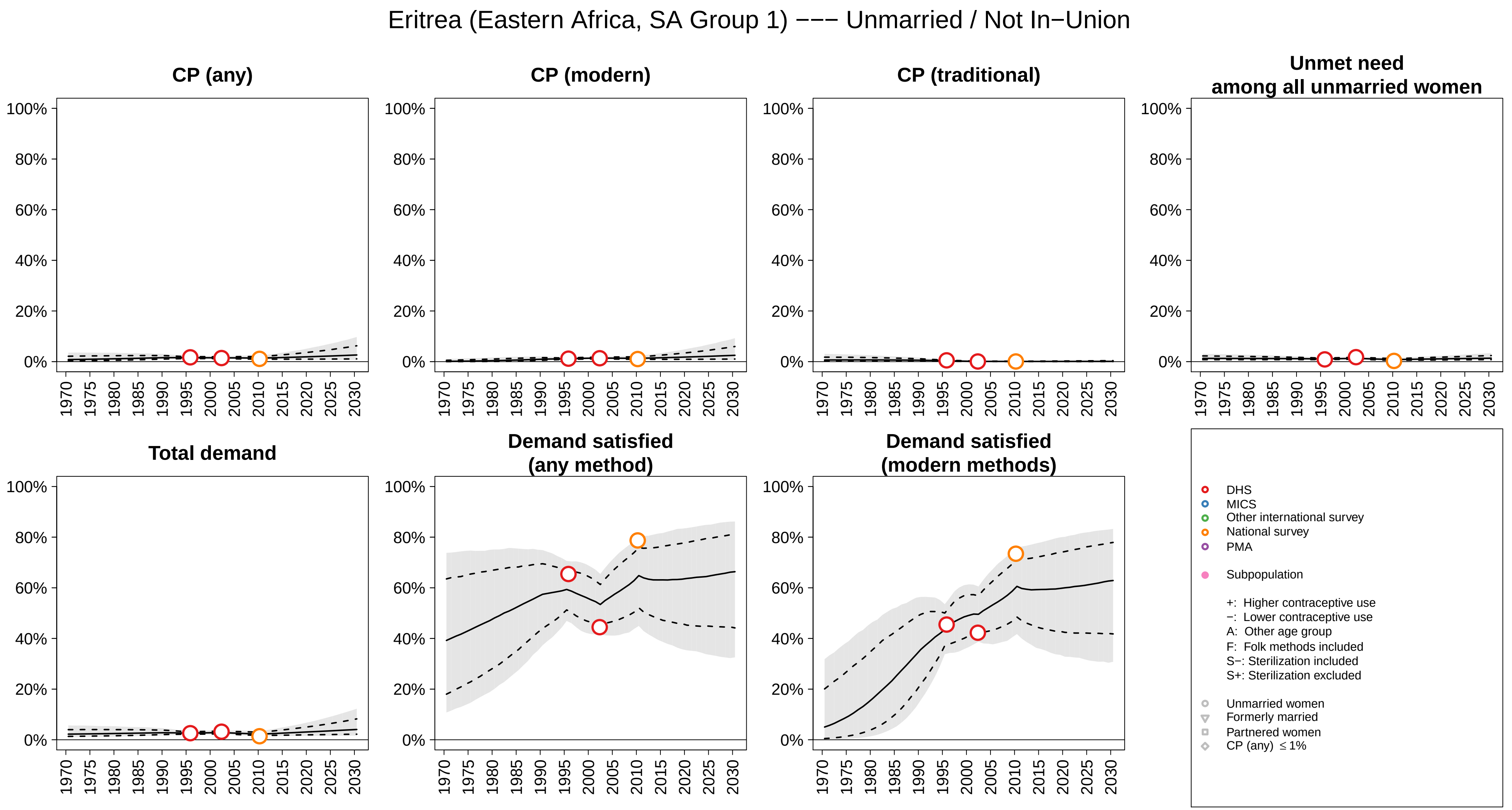
Eritrea (Eastern Africa, SA Group 1) −−− Unmarried / Not In−Union
CP (any)
CP (modern)
CP (traditional)
Unmet need among all unmarried women
Total demand
Demand satisfied (any method)
Demand satisfied (modern methods)
100%
80%
60%
40%
20%
0%
1970 1975 1980 1985 1990 1995 2000 2005 2010 2015 2020 2025 2030
DHS
MICS
Other international survey
National survey
PMA
Subpopulation
+: Higher contraceptive use
−: Lower contraceptive use
A: Other age group
F: Folk methods included
S−: Sterilization included
S+: Sterilization excluded
Unmarried women
Formerly married
Partnered women
CP (any) ≤ 1%

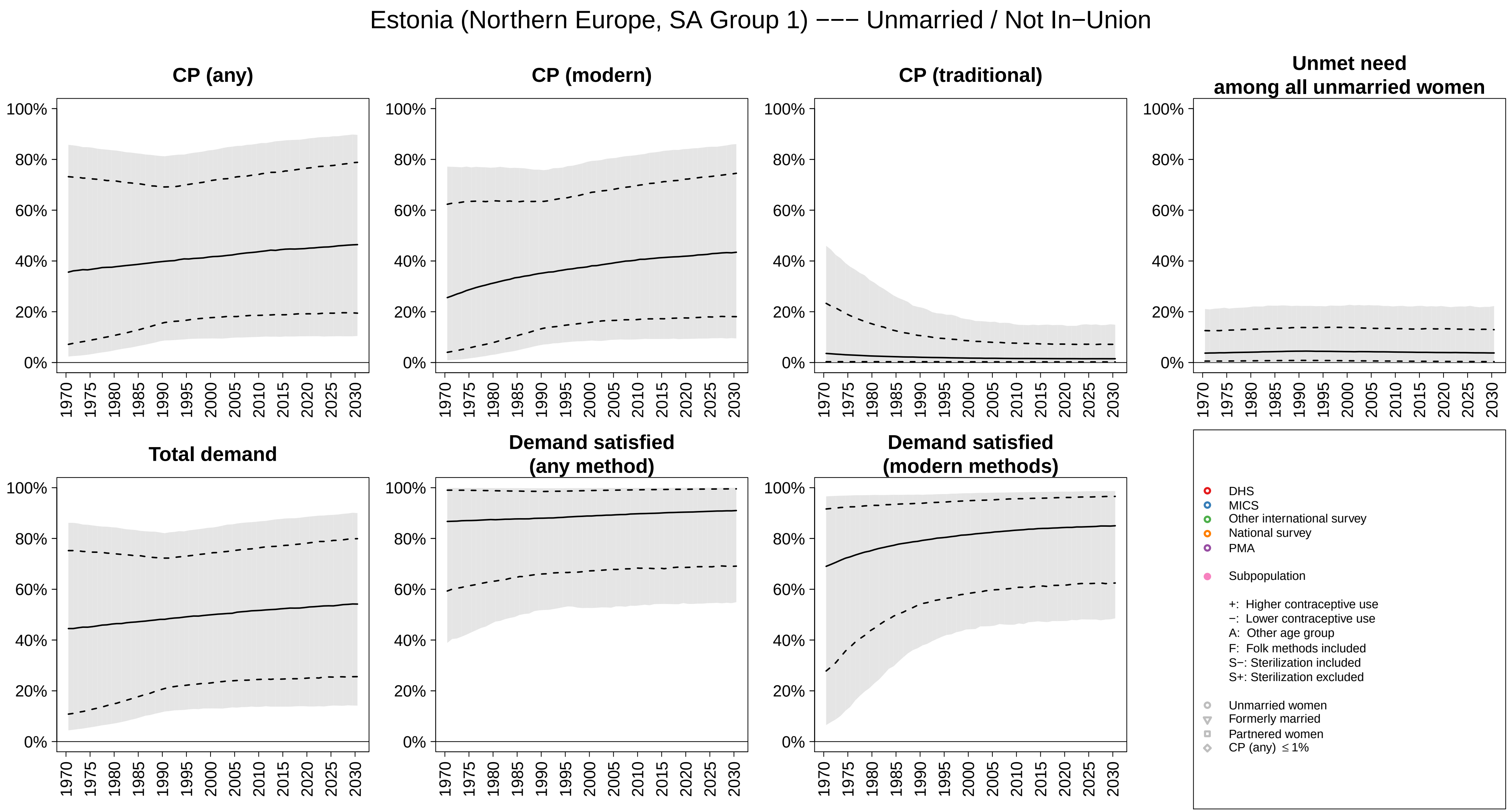
Estonia (Northern Europe, SA Group 1) --- Unmarried / Not In−Union
CP (any)
CP (modern)
CP (traditional)
Unmet need among all unmarried women
Total demand
Demand satisfied (any method)
Demand satisfied (modern methods)
0%
20%
40%
60%
80%
100%
1970
1975
1980
1985
1990
1995
2000
2005
2010
2015
2020
2025
2030
DHS
MICS
Other international survey
National survey
PMA
Subpopulation
+: Higher contraceptive use
−: Lower contraceptive use
A: Other age group
F: Folk methods included
S−: Sterilization included
S+: Sterilization excluded
Unmarried women
Formerly married
Partnered women
CP (any) ≤ 1%

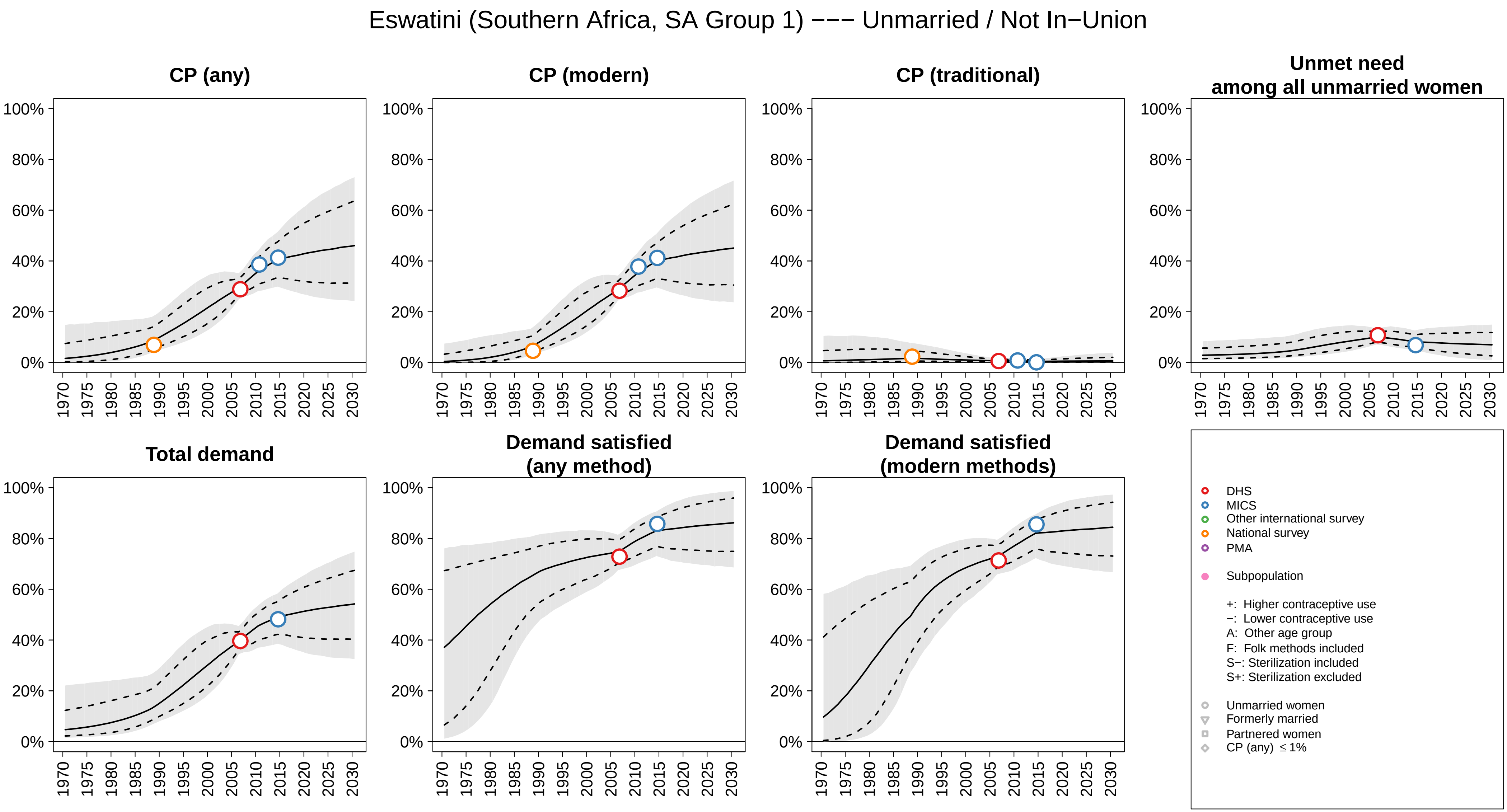
Eswatini (Southern Africa, SA Group 1) --- Unmarried / Not In−Union
CP (any)
CP (modern)
CP (traditional)
Unmet need among all unmarried women
Total demand
Demand satisfied (any method)
Demand satisfied (modern methods)
0%
20%
40%
60%
80%
100%
1970
1975
1980
1985
1990
1995
2000
2005
2010
2015
2020
2025
2030
DHS
MICS
Other international survey
National survey
PMA
Subpopulation
+: Higher contraceptive use
−: Lower contraceptive use
A: Other age group
F: Folk methods included
S−: Sterilization included
S+: Sterilization excluded
Unmarried women
Formerly married
Partnered women
CP (any) ≤ 1%

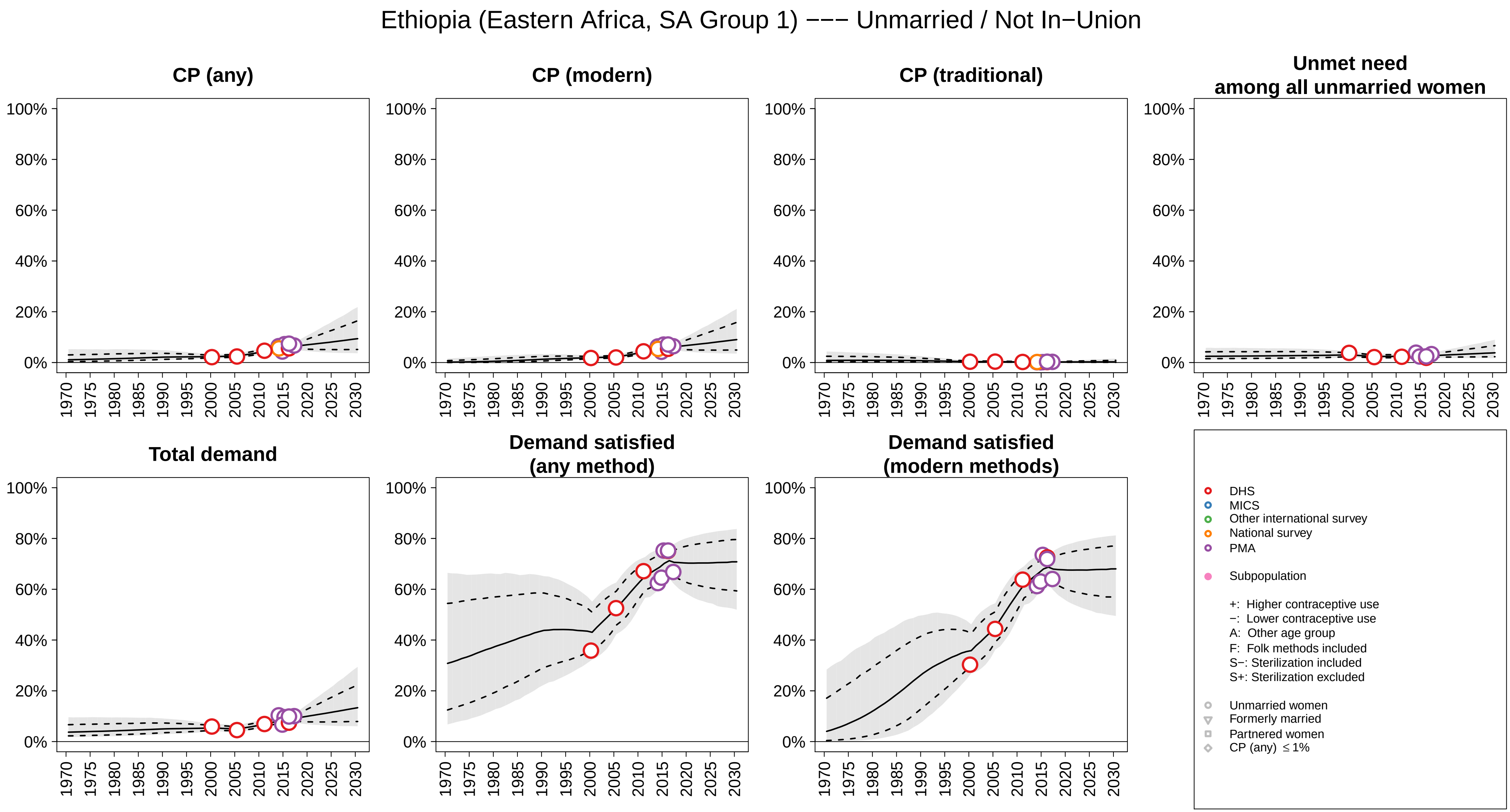

Ethiopia (Eastern Africa, SA Group 1) −−− Unmarried / Not In−Union
CP (any)
CP (modern)
CP (traditional)
Unmet need among all unmarried women
Total demand
Demand satisfied (any method)
Demand satisfied (modern methods)
100%
80%
60%
40%
20%
0%
1970 1975 1980 1985 1990 1995 2000 2005 2010 2015 2020 2025 2030
DHS
MICS
Other international survey
National survey
PMA
Subpopulation
+: Higher contraceptive use
−: Lower contraceptive use
A: Other age group
F: Folk methods included
S−: Sterilization included
S+: Sterilization excluded
Unmarried women
Formerly married
Partnered women
CP (any) ≤ 1%

Fiji (Melanesia, SA Group 1) −−− Unmarried / Not In−Union

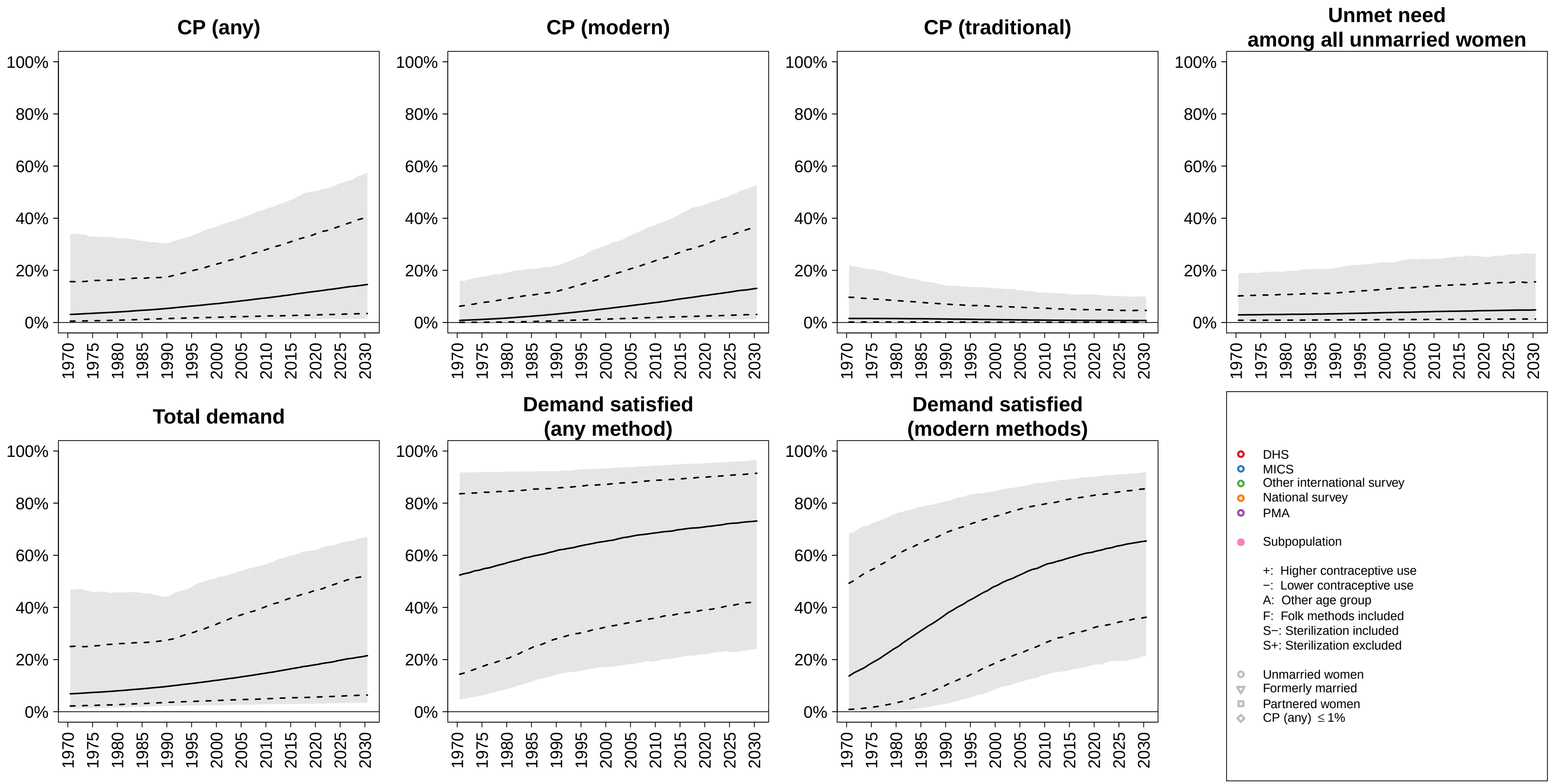

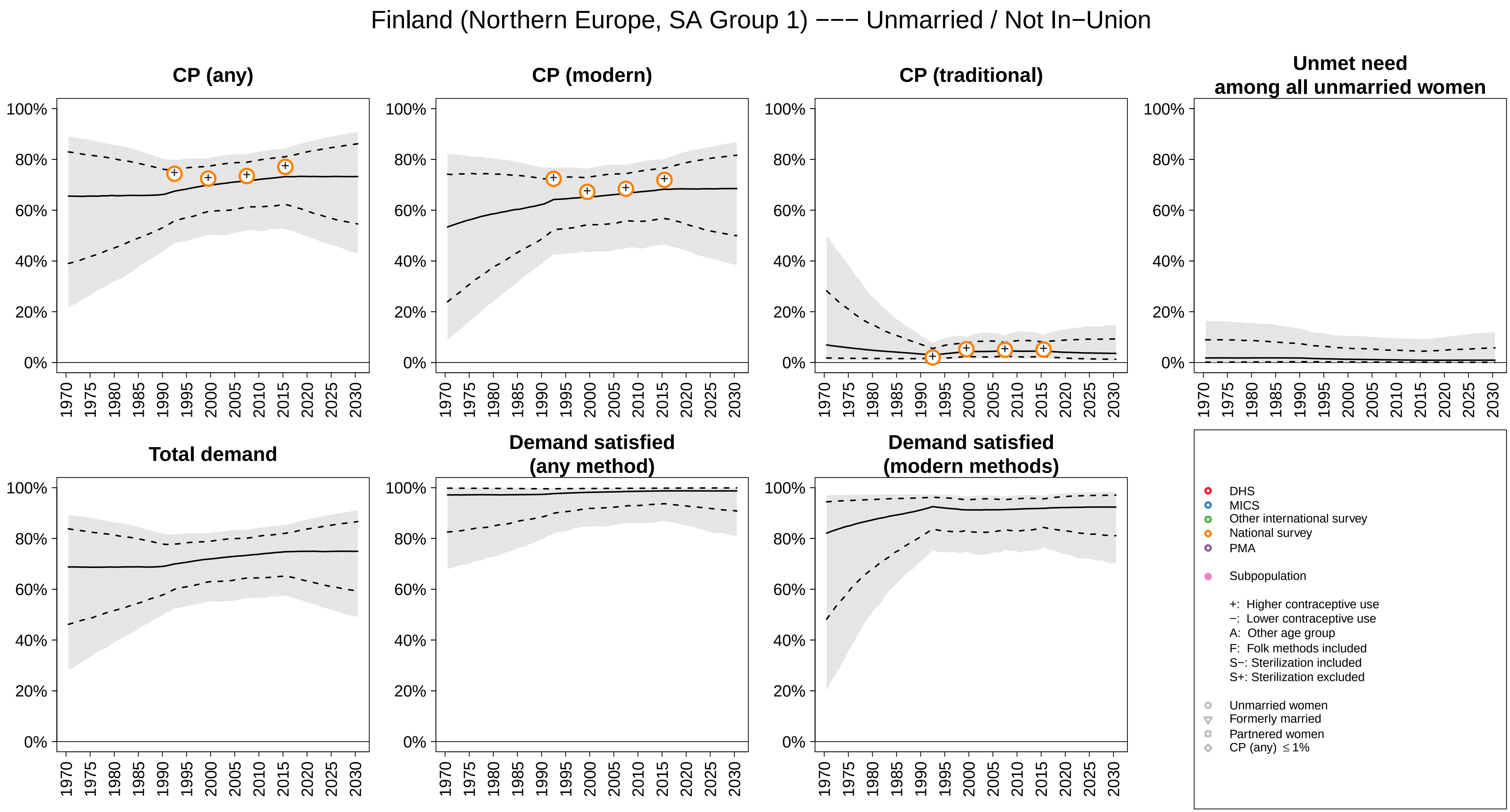

Finland (Northern Europe, SA Group 1) --- Unmarried / Not In−Union
CP (any)
CP (modern)
CP (traditional)
Unmet need among all unmarried women
Total demand
Demand satisfied (any method)
Demand satisfied (modern methods)
0%
20%
40%
60%
80%
100%
1970
1975
1980
1985
1990
1995
2000
2005
2010
2015
2020
2025
2030
DHS
MICS
Other international survey
National survey
PMA
Subpopulation
+: Higher contraceptive use
−: Lower contraceptive use
A: Other age group
F: Folk methods included
S−: Sterilization included
S+: Sterilization excluded
Unmarried women
Formerly married
Partnered women
CP (any) ≤ 1%

France (Western Europe, SA Group 1) −−− Unmarried / Not In−Union

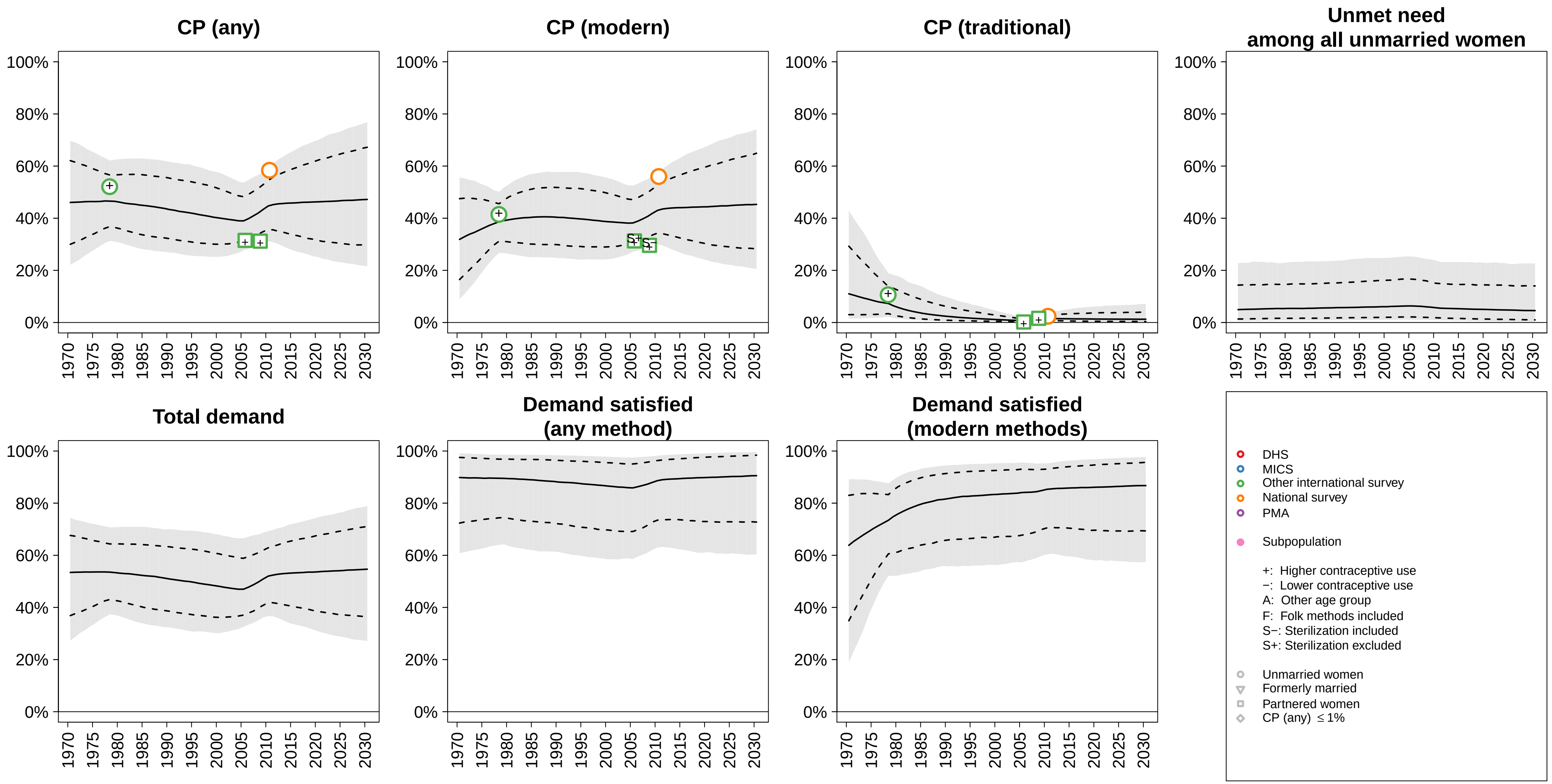

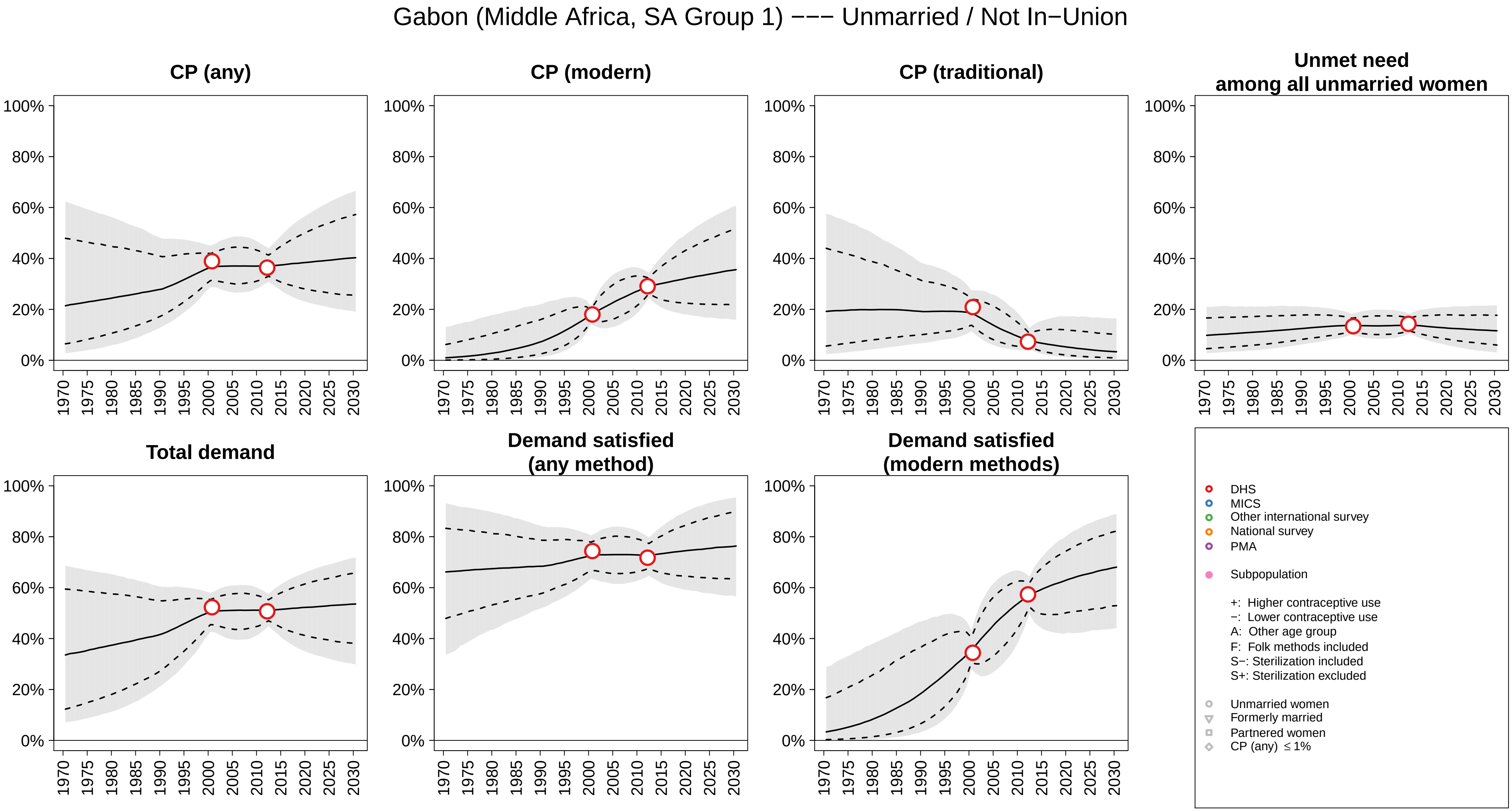
Gabon (Middle Africa, SA Group 1) −−− Unmarried / Not In−Union
CP (any)
CP (modern)
CP (traditional)
Unmet need
among all unmarried women
Total demand
Demand satisfied
(any method)
Demand satisfied
(modern methods)
0%
20%
40%
60%
80%
100%
1970
1975
1980
1985
1990
1995
2000
2005
2010
2015
2020
2025
2030
DHS
MICS
Other international survey
National survey
PMA
Subpopulation
+: Higher contraceptive use
−: Lower contraceptive use
A: Other age group
F: Folk methods included
S−: Sterilization included
S+: Sterilization excluded
Unmarried women
Formerly married
Partnered women
CP (any) ≤ 1%

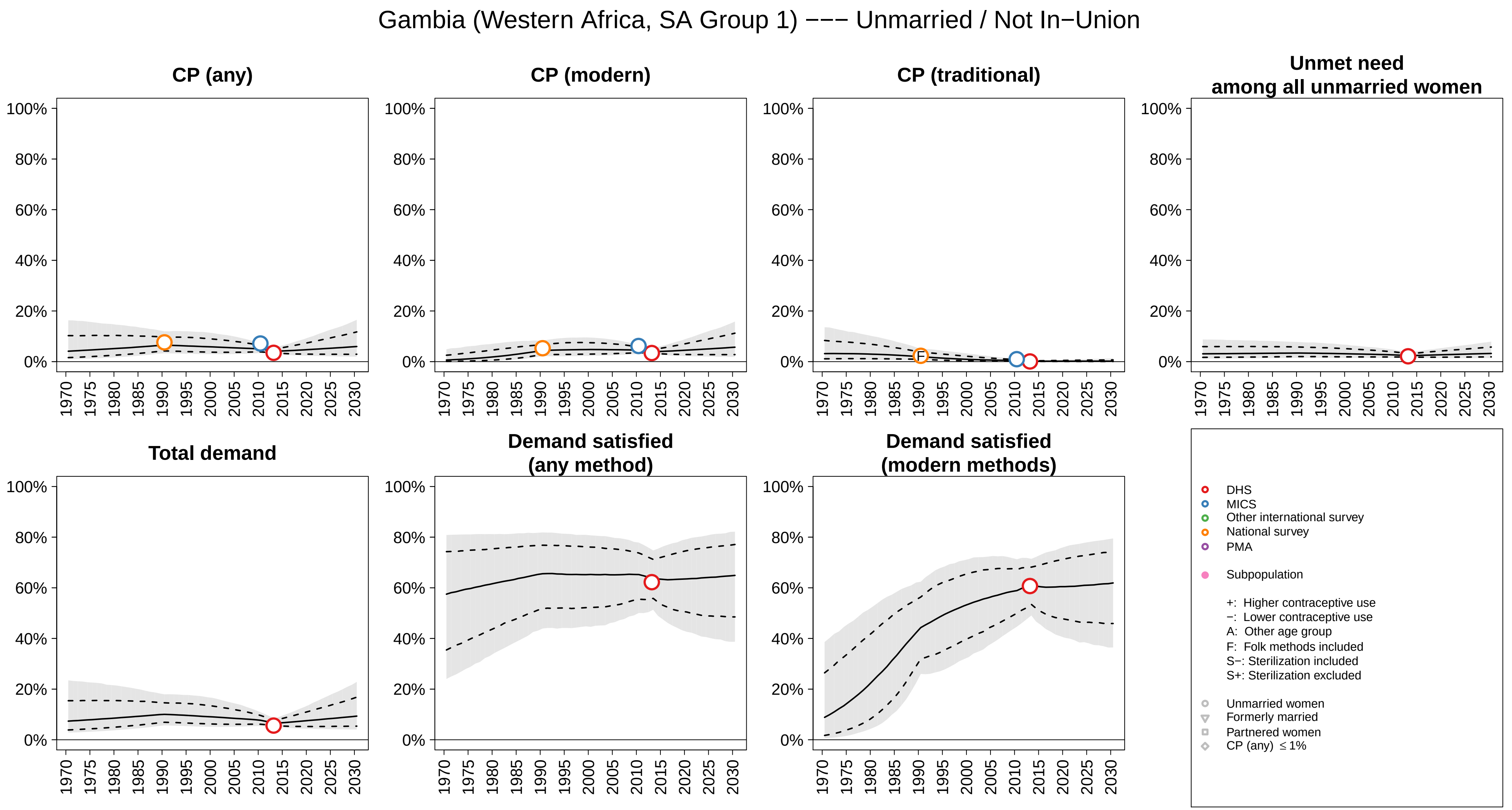

Gambia (Western Africa, SA Group 1) --- Unmarried / Not In−Union
CP (any)
CP (modern)
CP (traditional)
Unmet need among all unmarried women
Total demand
Demand satisfied (any method)
Demand satisfied (modern methods)
100%
80%
60%
40%
20%
0%
1970 1975 1980 1985 1990 1995 2000 2005 2010 2015 2020 2025 2030
DHS
MICS
Other international survey
National survey
PMA
Subpopulation
+: Higher contraceptive use
−: Lower contraceptive use
A: Other age group
F: Folk methods included
S−: Sterilization included
S+: Sterilization excluded
Unmarried women
Formerly married
Partnered women
CP (any) ≤ 1%

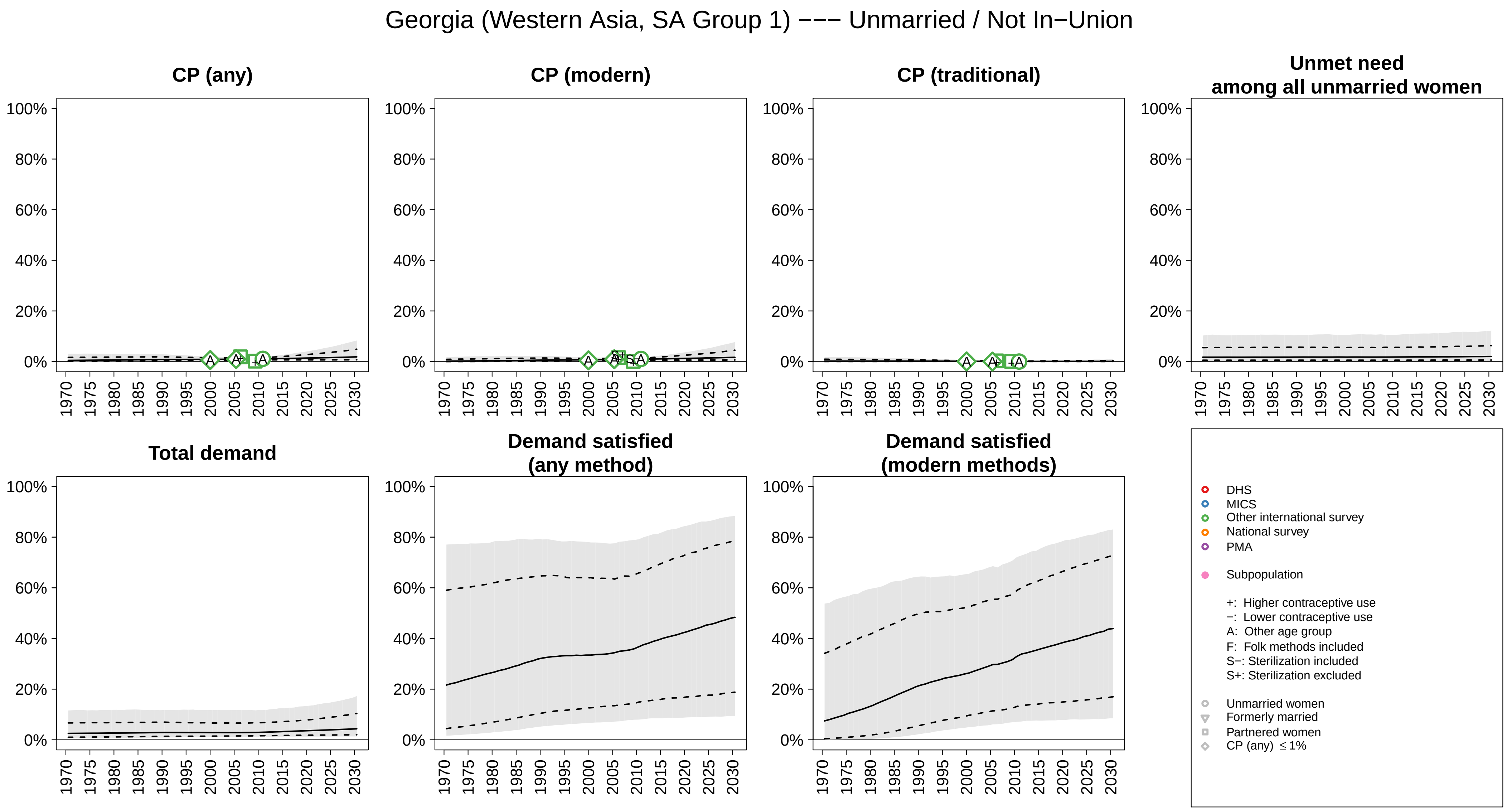
Georgia (Western Asia, SA Group 1) −−− Unmarried / Not In−Union
CP (any)
CP (modern)
CP (traditional)
Unmet need among all unmarried women
Total demand
Demand satisfied (any method)
Demand satisfied (modern methods)
100%
80%
60%
40%
20%
0%
1970 1975 1980 1985 1990 1995 2000 2005 2010 2015 2020 2025 2030
DHS
MICS
Other international survey
National survey
PMA
Subpopulation
+: Higher contraceptive use
−: Lower contraceptive use
A: Other age group
F: Folk methods included
S−: Sterilization included
S+: Sterilization excluded
Unmarried women
Formerly married
Partnered women
CP (any) ≤ 1%

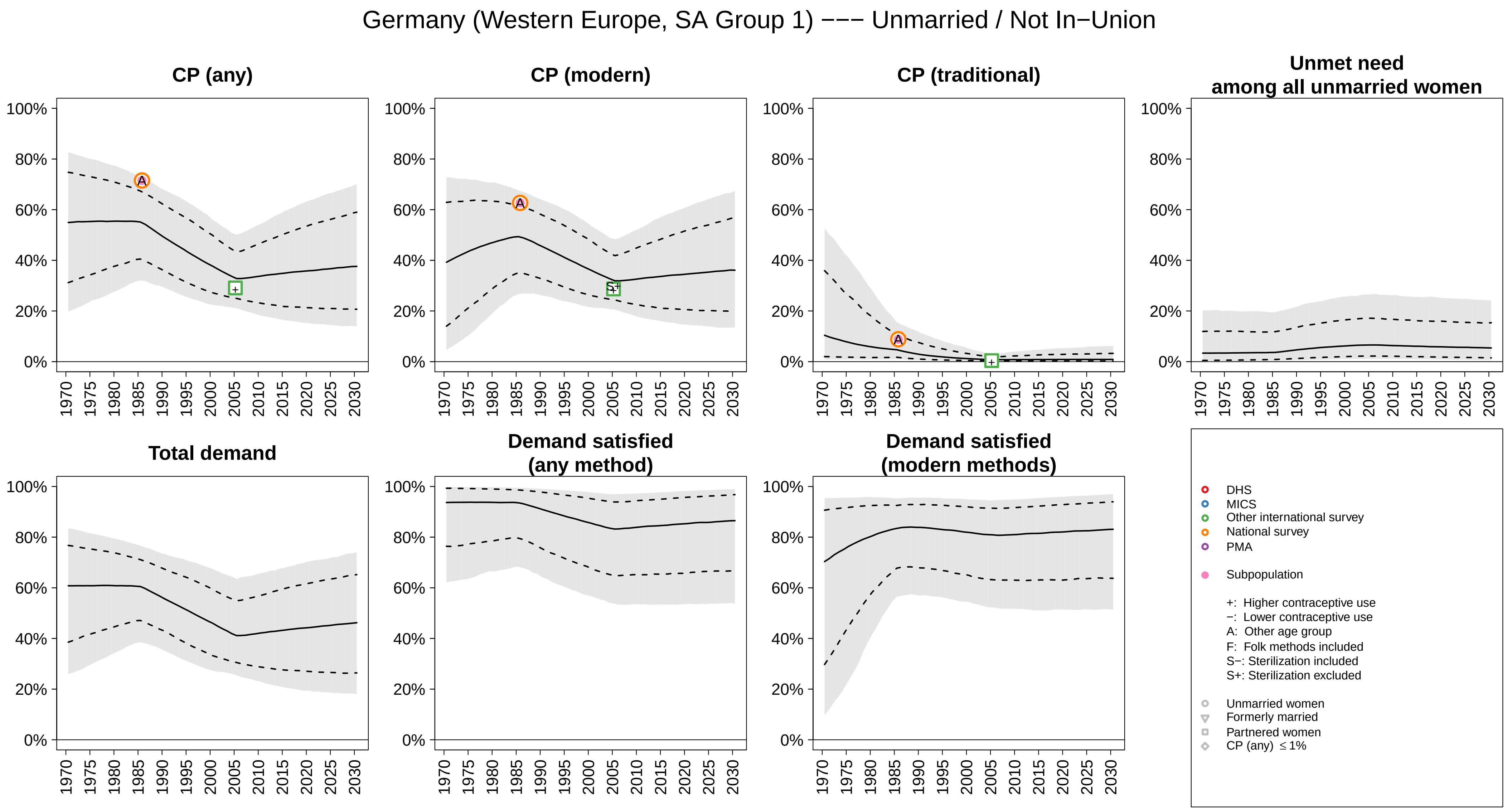
Germany (Western Europe, SA Group 1) --- Unmarried / Not In−Union
CP (any)
CP (modern)
CP (traditional)
Unmet need among all unmarried women
Total demand
Demand satisfied (any method)
Demand satisfied (modern methods)
100%
80%
60%
40%
20%
0%
1970 1975 1980 1985 1990 1995 2000 2005 2010 2015 2020 2025 2030
DHS
MICS
Other international survey
National survey
PMA
Subpopulation
+: Higher contraceptive use
−: Lower contraceptive use
A: Other age group
F: Folk methods included
S−: Sterilization included
S+: Sterilization excluded
Unmarried women
Formerly married
Partnered women
CP (any) ≤ 1%

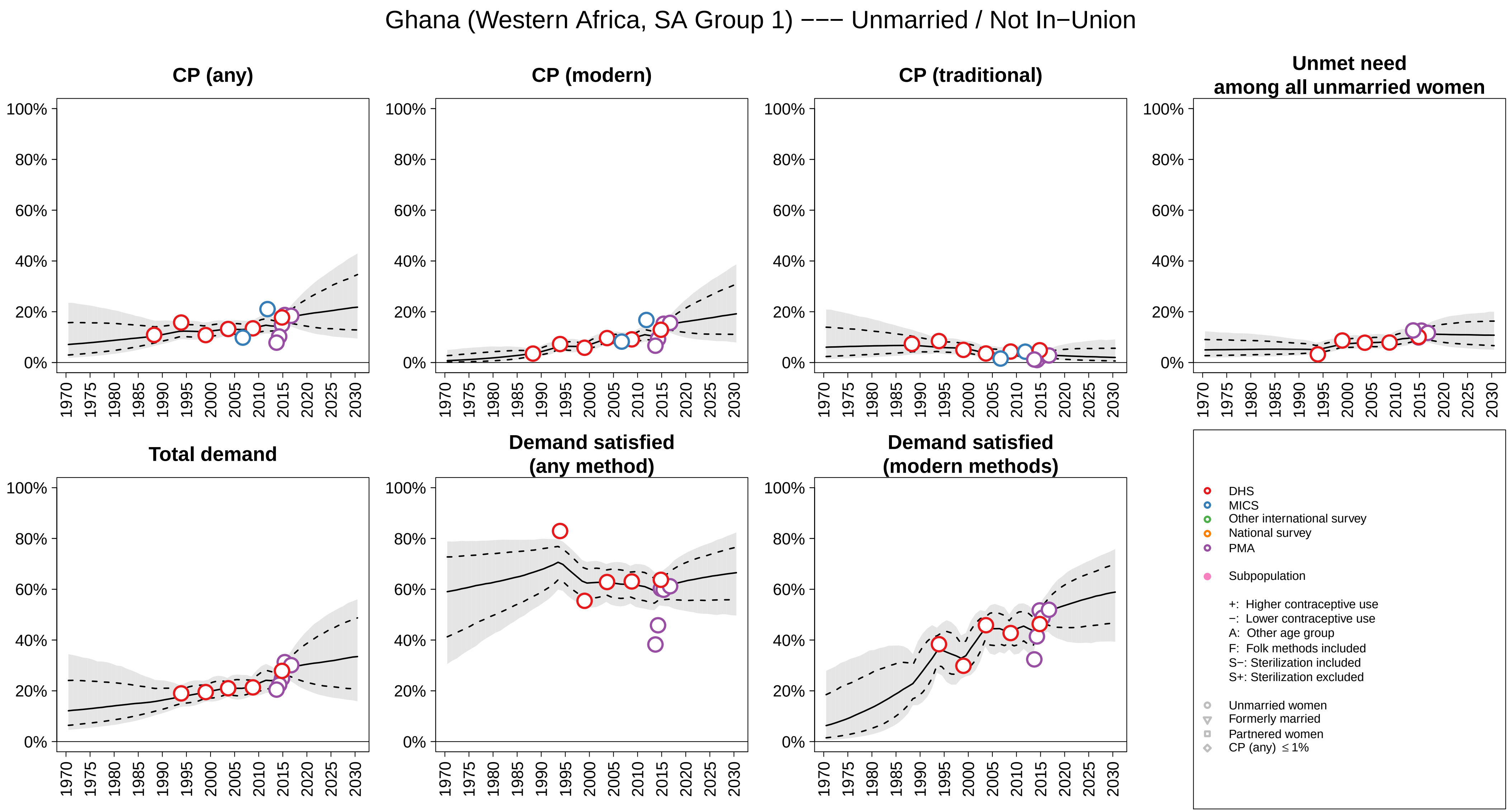
Ghana (Western Africa, SA Group 1) −−− Unmarried / Not In−Union
CP (any)
CP (modern)
CP (traditional)
Unmet need among all unmarried women
Total demand
Demand satisfied (any method)
Demand satisfied (modern methods)
0%
20%
40%
60%
80%
100%
1970
1975
1980
1985
1990
1995
2000
2005
2010
2015
2020
2025
2030
DHS
MICS
Other international survey
National survey
PMA
Subpopulation
+: Higher contraceptive use
−: Lower contraceptive use
A: Other age group
F: Folk methods included
S−: Sterilization included
S+: Sterilization excluded
Unmarried women
Formerly married
Partnered women
CP (any) ≤ 1%

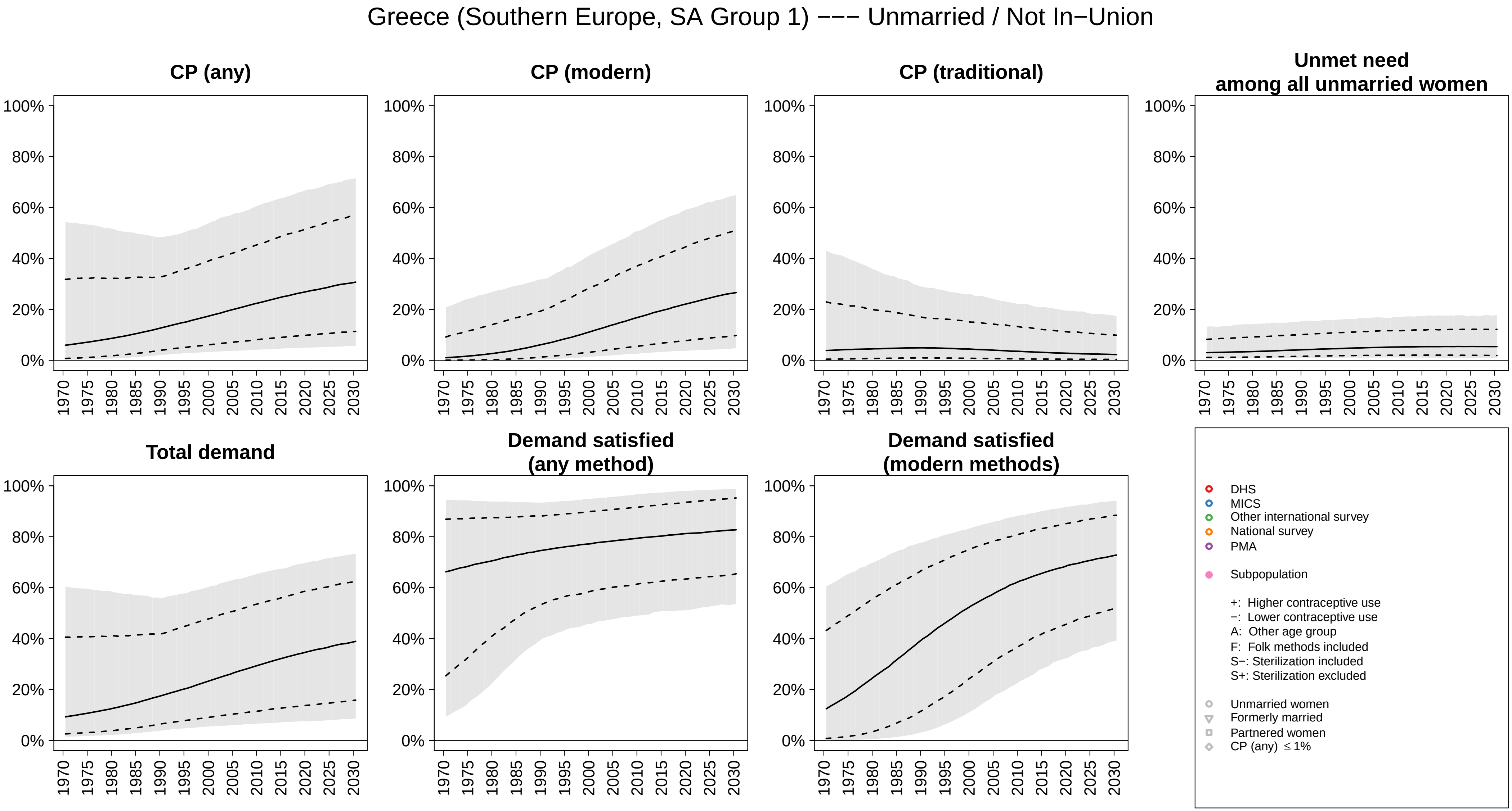
Greece (Southern Europe, SA Group 1) −−− Unmarried / Not In−Union
CP (any)
CP (modern)
CP (traditional)
Unmet need
among all unmarried women
Total demand
Demand satisfied
(any method)
Demand satisfied
(modern methods)
100%
80%
60%
40%
20%
0%
1970
1975
1980
1985
1990
1995
2000
2005
2010
2015
2020
2025
2030
DHS
MICS
Other international survey
National survey
PMA
Subpopulation
+: Higher contraceptive use
−: Lower contraceptive use
A: Other age group
F: Folk methods included
S−: Sterilization included
S+: Sterilization excluded
Unmarried women
Formerly married
Partnered women
CP (any) ≤ 1%

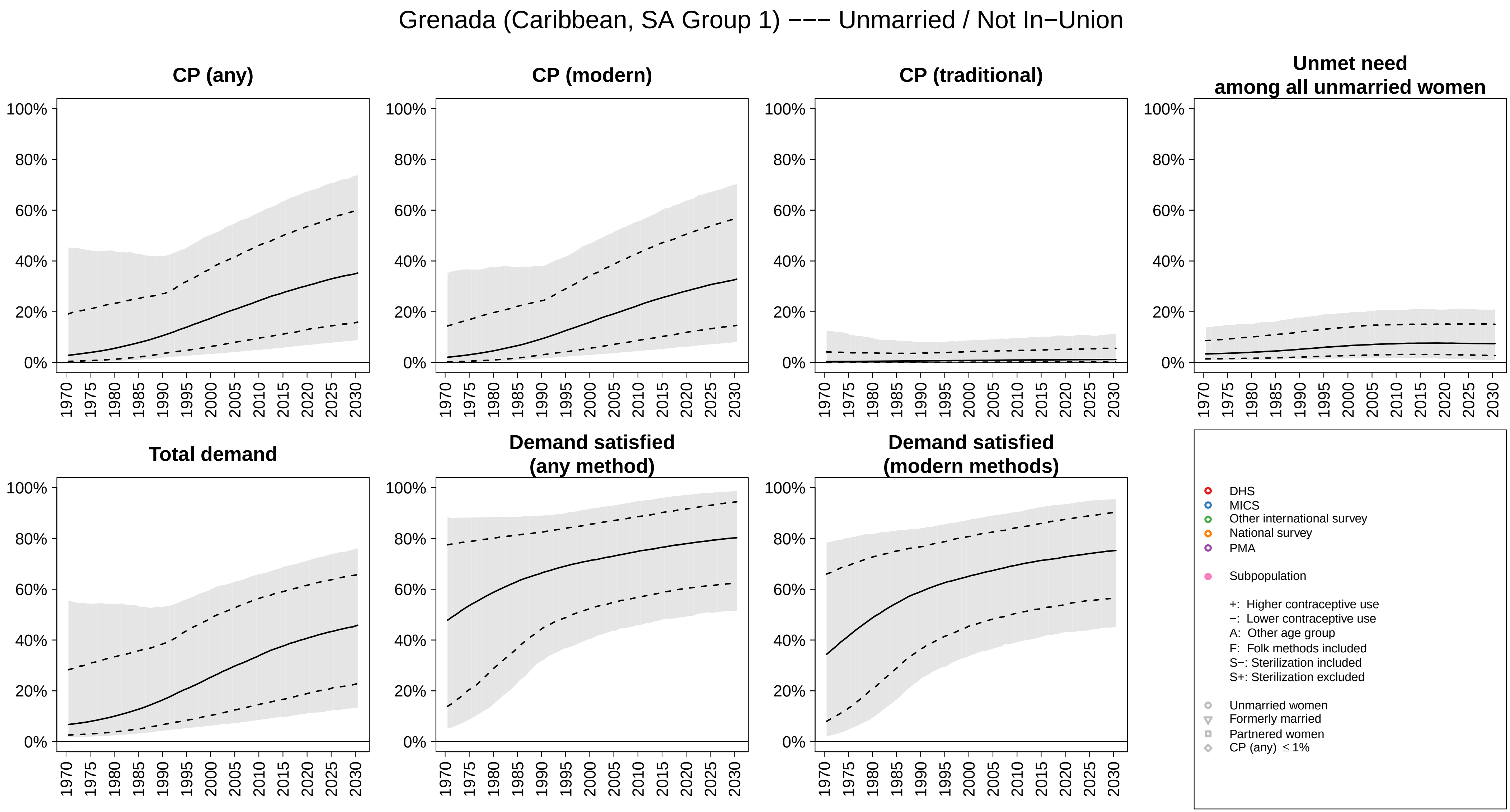

Grenada (Caribbean, SA Group 1) --- Unmarried / Not In-Union
CP (any)
CP (modern)
CP (traditional)
Unmet need among all unmarried women
Total demand
Demand satisfied (any method)
Demand satisfied (modern methods)
0%
20%
40%
60%
80%
100%
1970
1975
1980
1985
1990
1995
2000
2005
2010
2015
2020
2025
2030
DHS
MICS
Other international survey
National survey
PMA
Subpopulation
+: Higher contraceptive use
−: Lower contraceptive use
A: Other age group
F: Folk methods included
S−: Sterilization included
S+: Sterilization excluded
Unmarried women
Formerly married
Partnered women
CP (any) ≤ 1%

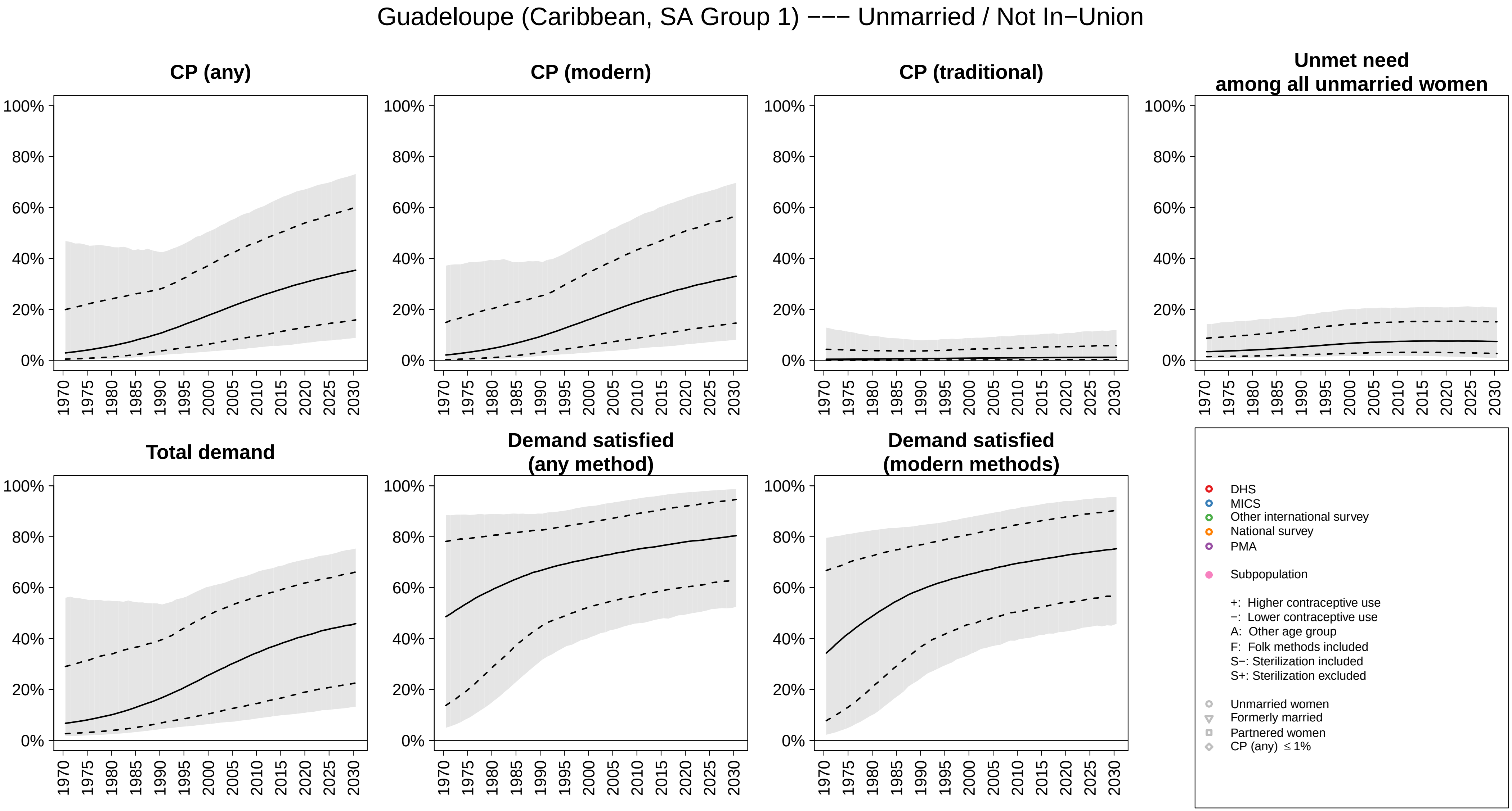
Guadeloupe (Caribbean, SA Group 1) −−− Unmarried / Not In−Union
CP (any)
CP (modern)
CP (traditional)
Unmet need among all unmarried women
Total demand
Demand satisfied (any method)
Demand satisfied (modern methods)
0%
20%
40%
60%
80%
100%
1970
1975
1980
1985
1990
1995
2000
2005
2010
2015
2020
2025
2030
DHS
MICS
Other international survey
National survey
PMA
Subpopulation
+: Higher contraceptive use
−: Lower contraceptive use
A: Other age group
F: Folk methods included
S−: Sterilization included
S+: Sterilization excluded
Unmarried women
Formerly married
Partnered women
CP (any) ≤ 1%

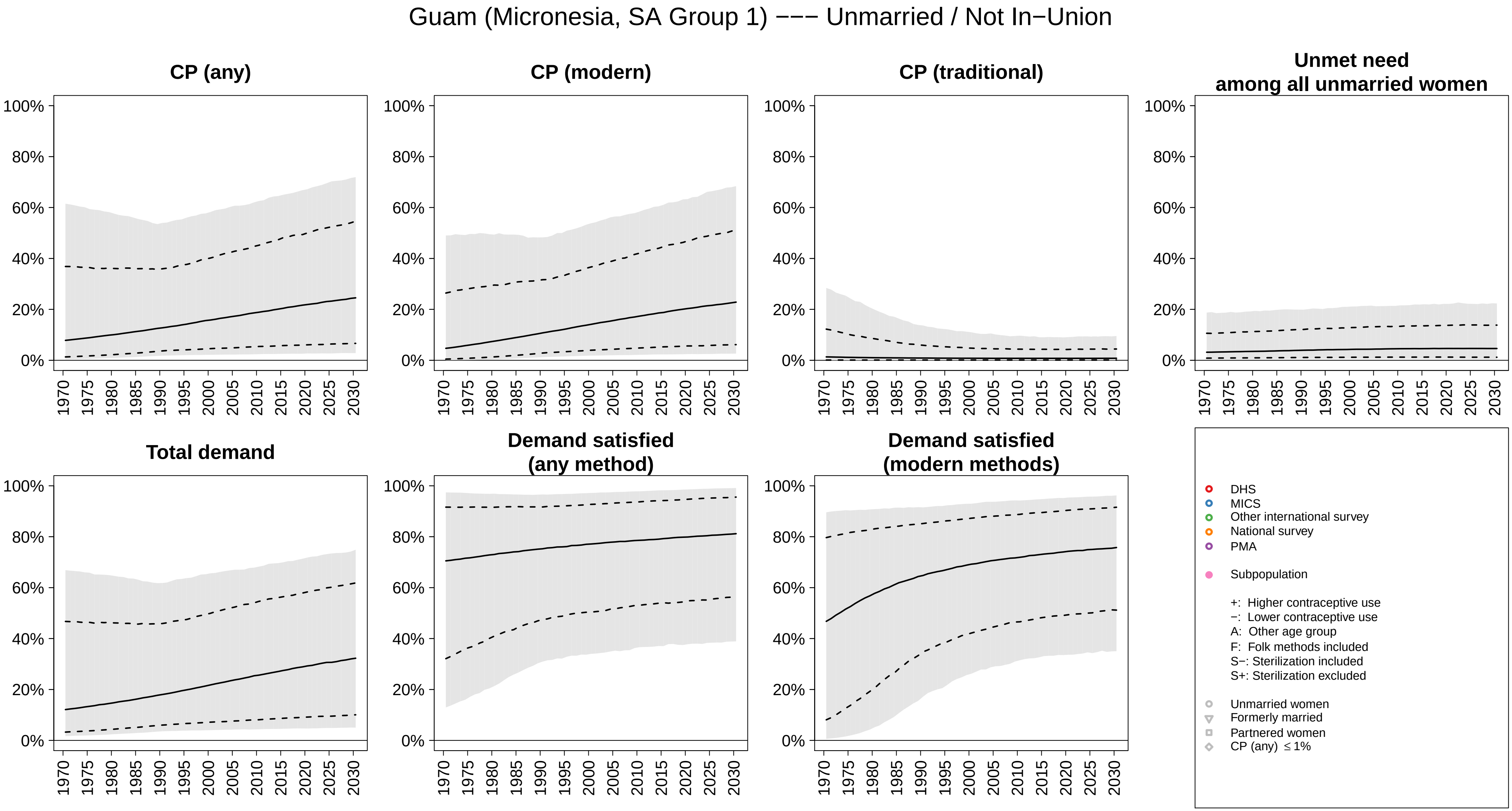
Guam (Micronesia, SA Group 1) −−− Unmarried / Not In−Union
CP (any)
CP (modern)
CP (traditional)
Unmet need
among all unmarried women
Total demand
Demand satisfied
(any method)
Demand satisfied
(modern methods)
100%
80%
60%
40%
20%
0%
1970
1975
1980
1985
1990
1995
2000
2005
2010
2015
2020
2025
2030
DHS
MICS
Other international survey
National survey
PMA
Subpopulation
+: Higher contraceptive use
−: Lower contraceptive use
A: Other age group
F: Folk methods included
S−: Sterilization included
S+: Sterilization excluded
Unmarried women
Formerly married
Partnered women
CP (any) ≤ 1%

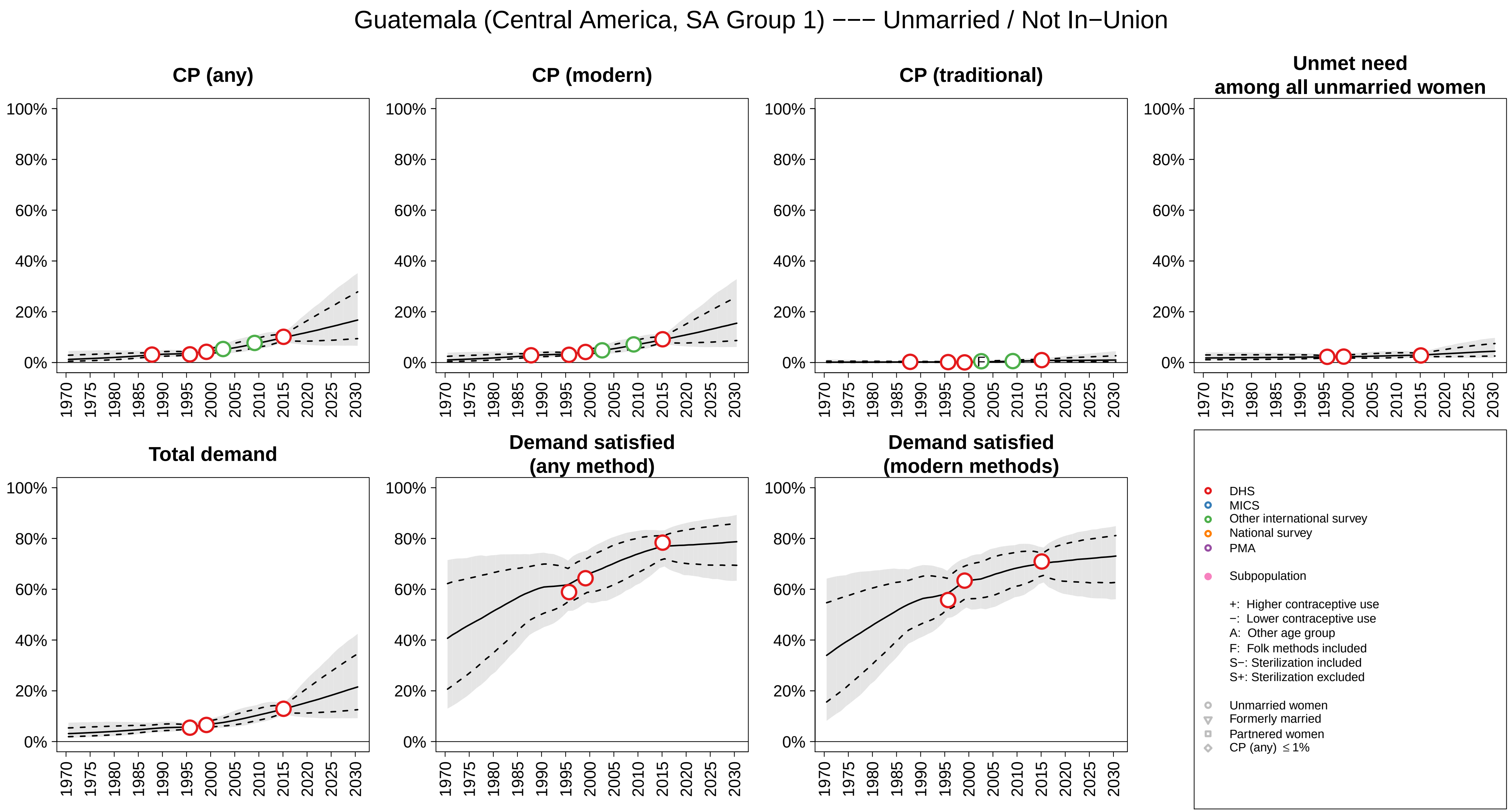
Guatemala (Central America, SA Group 1) --- Unmarried / Not In−Union
CP (any)
CP (modern)
CP (traditional)
Unmet need among all unmarried women
Total demand
Demand satisfied (any method)
Demand satisfied (modern methods)
0%
20%
40%
60%
80%
100%
1970
1975
1980
1985
1990
1995
2000
2005
2010
2015
2020
2025
2030
F
DHS
MICS
Other international survey
National survey
PMA
Subpopulation
+: Higher contraceptive use
−: Lower contraceptive use
A: Other age group
F: Folk methods included
S−: Sterilization included
S+: Sterilization excluded
Unmarried women
Formerly married
Partnered women
CP (any) ≤ 1%

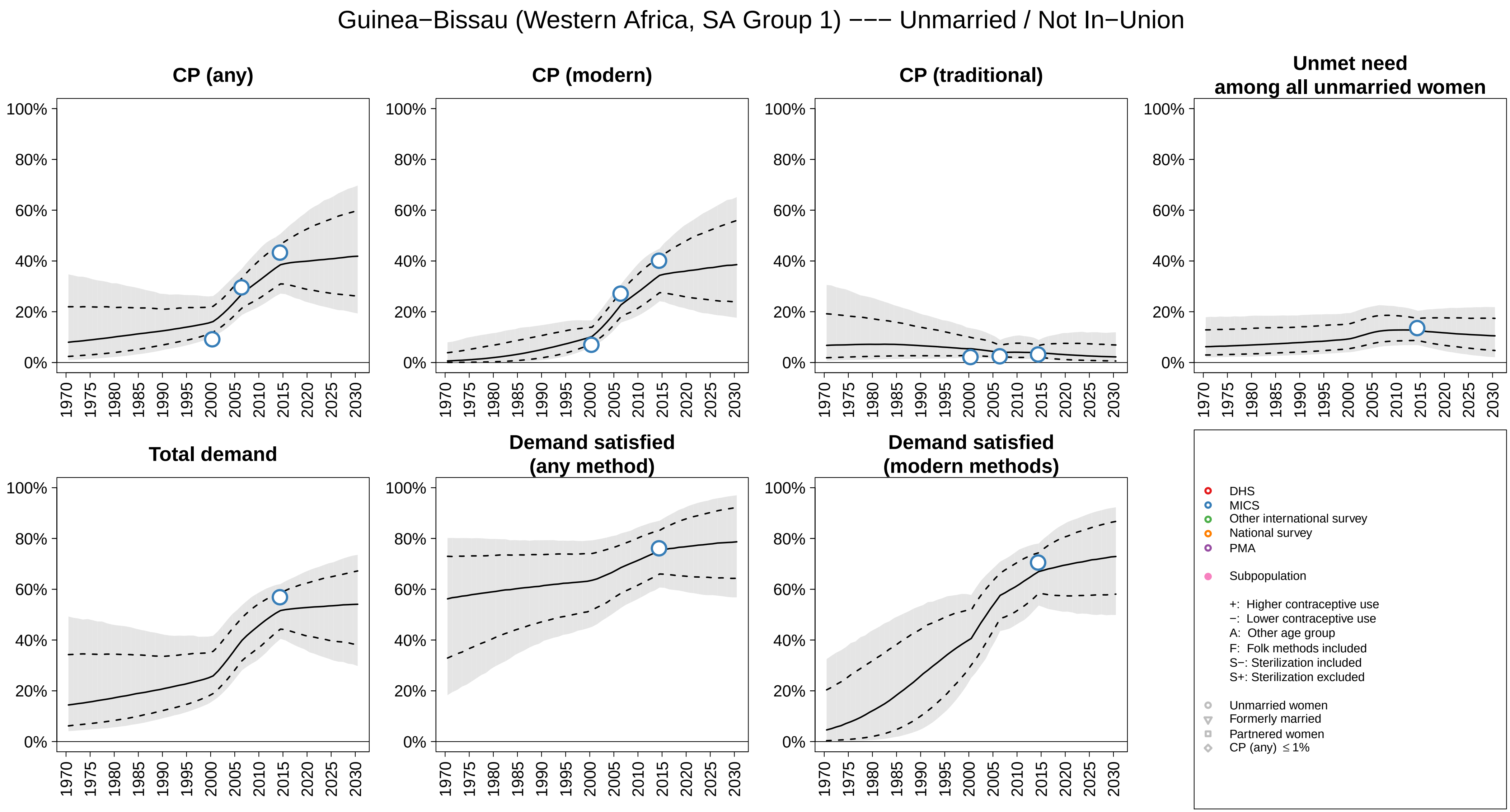
Guinea−Bissau (Western Africa, SA Group 1) −−− Unmarried / Not In−Union
CP (any)
CP (modern)
CP (traditional)
Unmet need among all unmarried women
Total demand
Demand satisfied (any method)
Demand satisfied (modern methods)
0%
20%
40%
60%
80%
100%
1970
1975
1980
1985
1990
1995
2000
2005
2010
2015
2020
2025
2030
DHS
MICS
Other international survey
National survey
PMA
Subpopulation
+: Higher contraceptive use
−: Lower contraceptive use
A: Other age group
F: Folk methods included
S−: Sterilization included
S+: Sterilization excluded
Unmarried women
Formerly married
Partnered women
CP (any) ≤ 1%

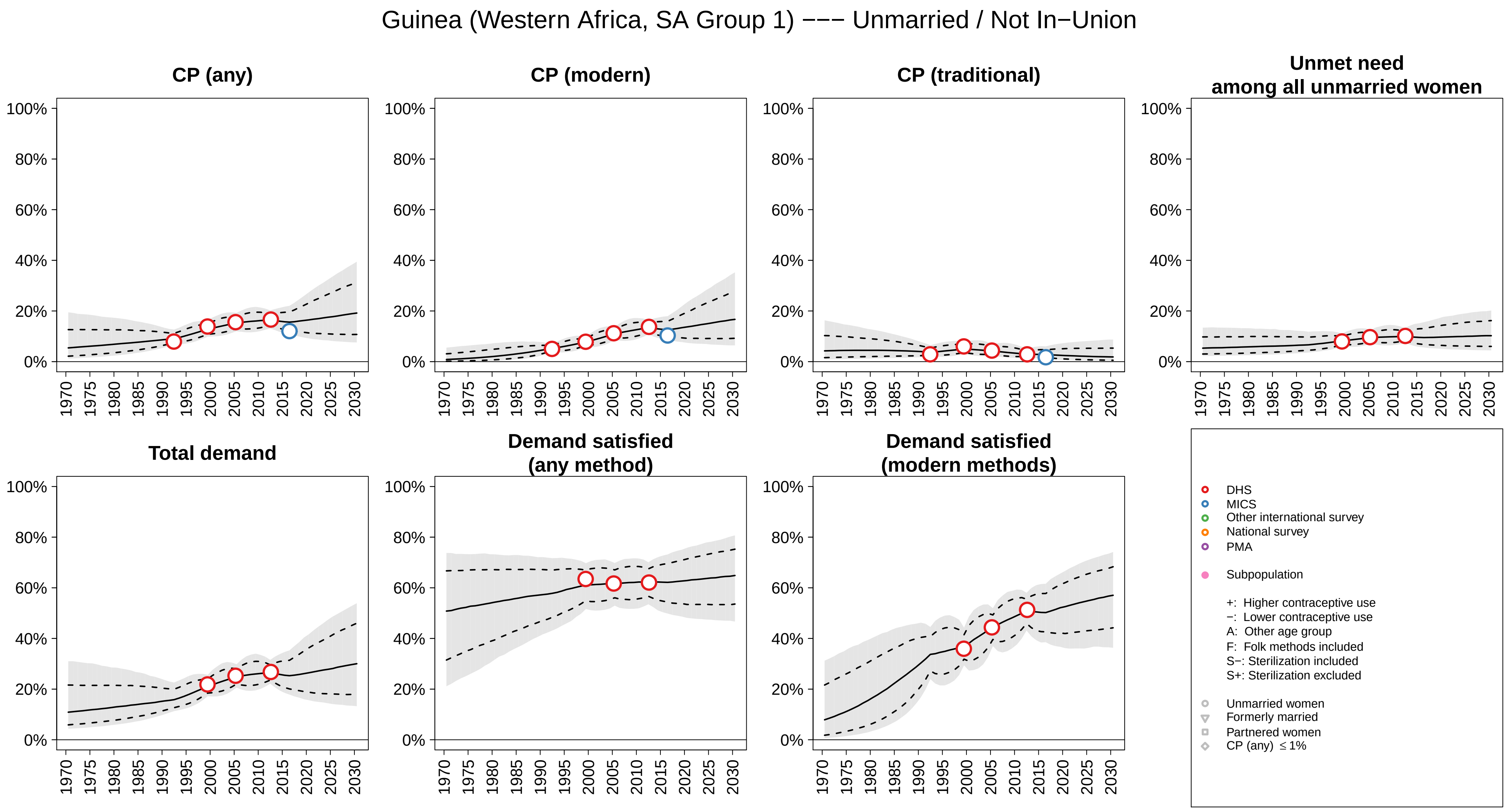
Guinea (Western Africa, SA Group 1) −−− Unmarried / Not In−Union
CP (any)
CP (modern)
CP (traditional)
Unmet need among all unmarried women
Total demand
Demand satisfied (any method)
Demand satisfied (modern methods)
100%
80%
60%
40%
20%
0%
1970
1975
1980
1985
1990
1995
2000
2005
2010
2015
2020
2025
2030
DHS
MICS
Other international survey
National survey
PMA
Subpopulation
+: Higher contraceptive use
−: Lower contraceptive use
A: Other age group
F: Folk methods included
S−: Sterilization included
S+: Sterilization excluded
Unmarried women
Formerly married
Partnered women
CP (any) ≤ 1%

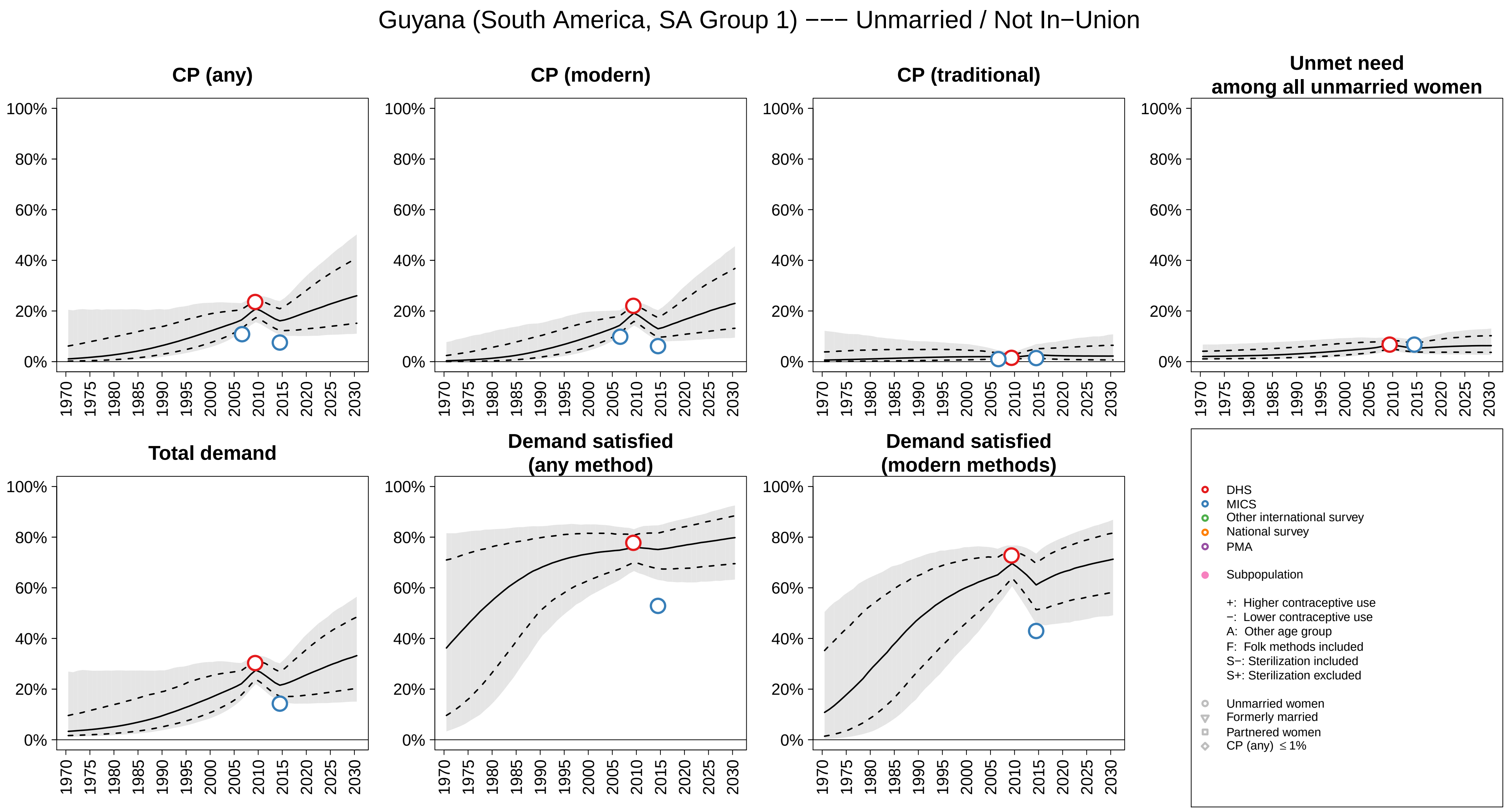
Guyana (South America, SA Group 1) −−− Unmarried / Not In−Union
CP (any)
CP (modern)
CP (traditional)
Unmet need among all unmarried women
Total demand
Demand satisfied (any method)
Demand satisfied (modern methods)
100%
80%
60%
40%
20%
0%
1970 1975 1980 1985 1990 1995 2000 2005 2010 2015 2020 2025 2030
DHS
MICS
Other international survey
National survey
PMA
Subpopulation
+: Higher contraceptive use
−: Lower contraceptive use
A: Other age group
F: Folk methods included
S−: Sterilization included
S+: Sterilization excluded
Unmarried women
Formerly married
Partnered women
CP (any) ≤ 1%

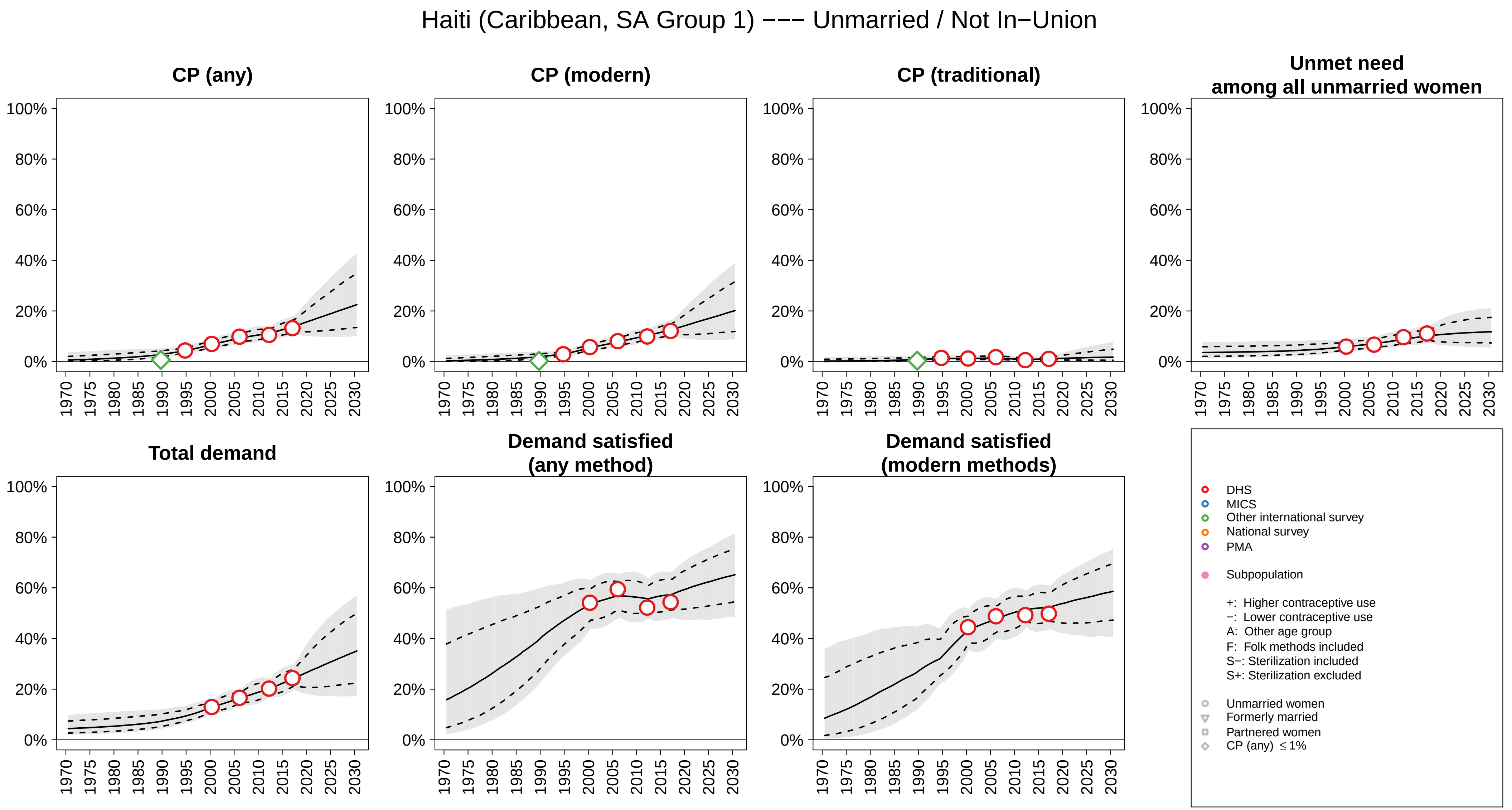
Haiti (Caribbean, SA Group 1) −−− Unmarried / Not In−Union
CP (any)
CP (modern)
CP (traditional)
Unmet need
among all unmarried women
Total demand
Demand satisfied
(any method)
Demand satisfied
(modern methods)
0%
20%
40%
60%
80%
100%
1970
1975
1980
1985
1990
1995
2000
2005
2010
2015
2020
2025
2030
DHS
MICS
Other international survey
National survey
PMA
Subpopulation
+: Higher contraceptive use
−: Lower contraceptive use
A: Other age group
F: Folk methods included
S−: Sterilization included
S+: Sterilization excluded
Unmarried women
Formerly married
Partnered women
CP (any) ≤ 1%

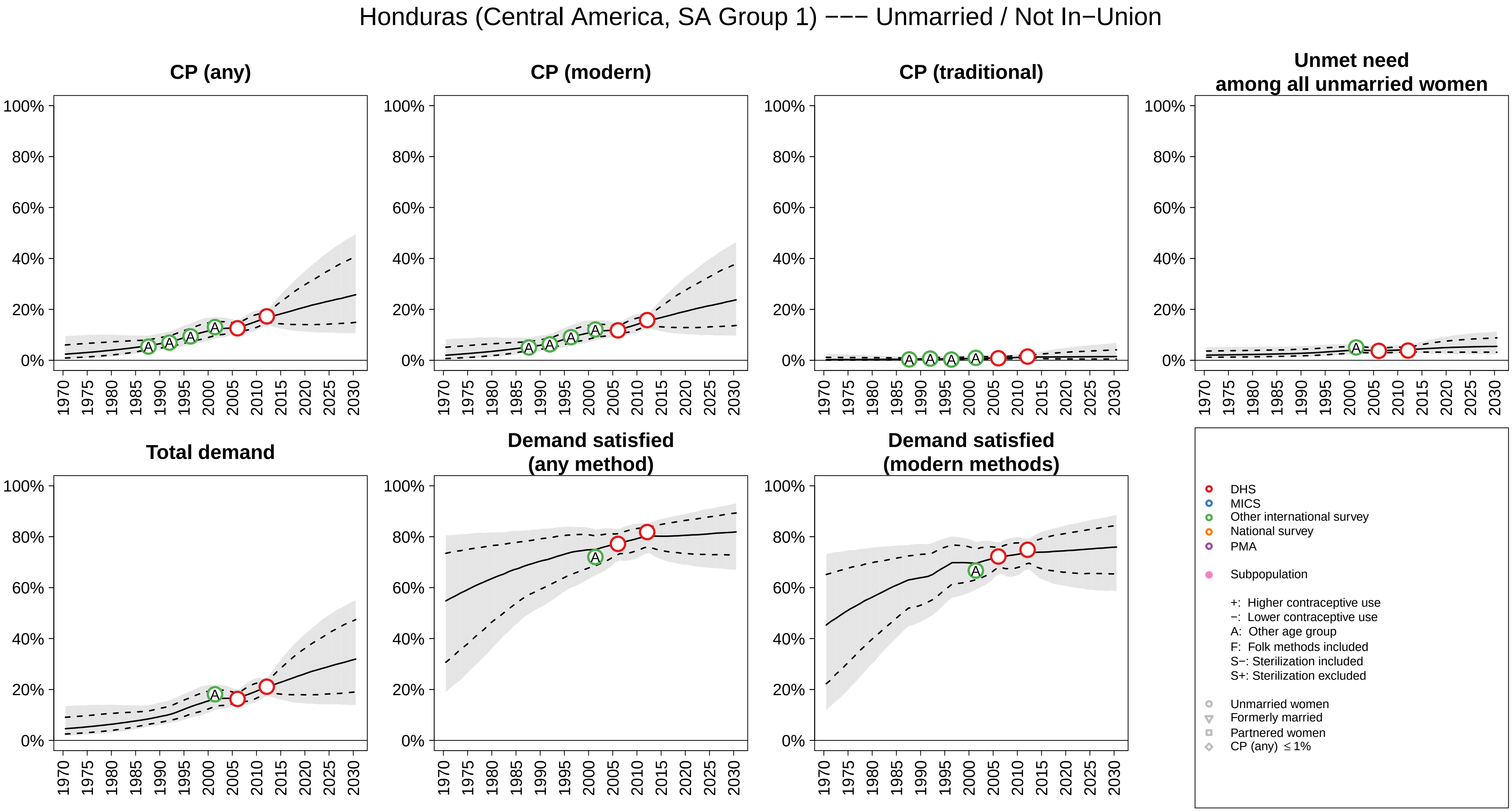
Honduras (Central America, SA Group 1) −−− Unmarried / Not In−Union
CP (any)
CP (modern)
CP (traditional)
Unmet need among all unmarried women
Total demand
Demand satisfied (any method)
Demand satisfied (modern methods)
0%
20%
40%
60%
80%
100%
1970
1975
1980
1985
1990
1995
2000
2005
2010
2015
2020
2025
2030
DHS
MICS
Other international survey
National survey
PMA
Subpopulation
+: Higher contraceptive use
−: Lower contraceptive use
A: Other age group
F: Folk methods included
S−: Sterilization included
S+: Sterilization excluded
Unmarried women
Formerly married
Partnered women
CP (any) ≤ 1%

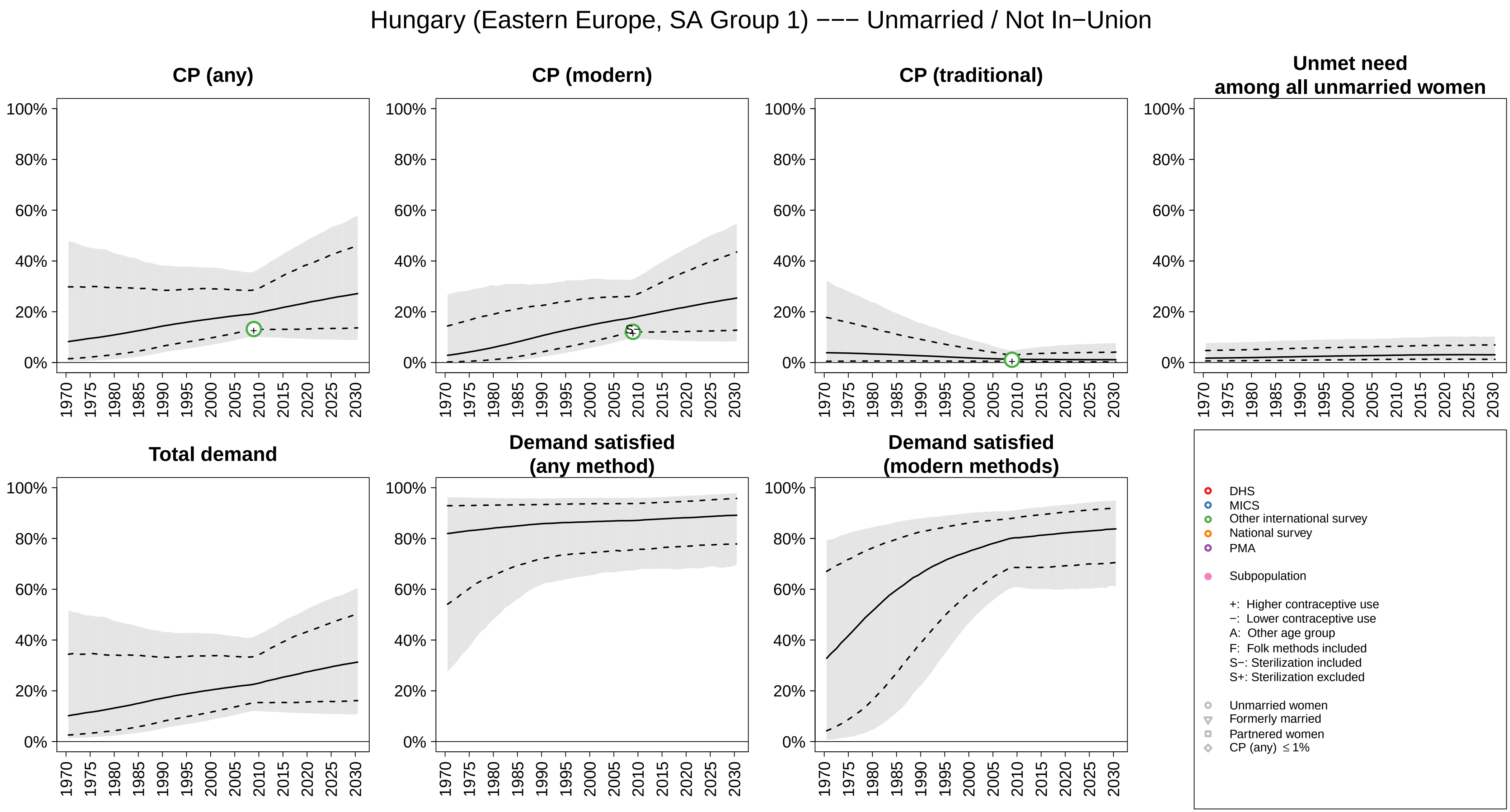

Hungary (Eastern Europe, SA Group 1) −−− Unmarried / Not In−Union
CP (any)
CP (modern)
CP (traditional)
Unmet need among all unmarried women
Total demand
Demand satisfied (any method)
Demand satisfied (modern methods)
0%
20%
40%
60%
80%
100%
1970
1975
1980
1985
1990
1995
2000
2005
2010
2015
2020
2025
2030
DHS
MICS
Other international survey
National survey
PMA
Subpopulation
+: Higher contraceptive use
−: Lower contraceptive use
A: Other age group
F: Folk methods included
S−: Sterilization included
S+: Sterilization excluded
Unmarried women
Formerly married
Partnered women
CP (any) ≤ 1%

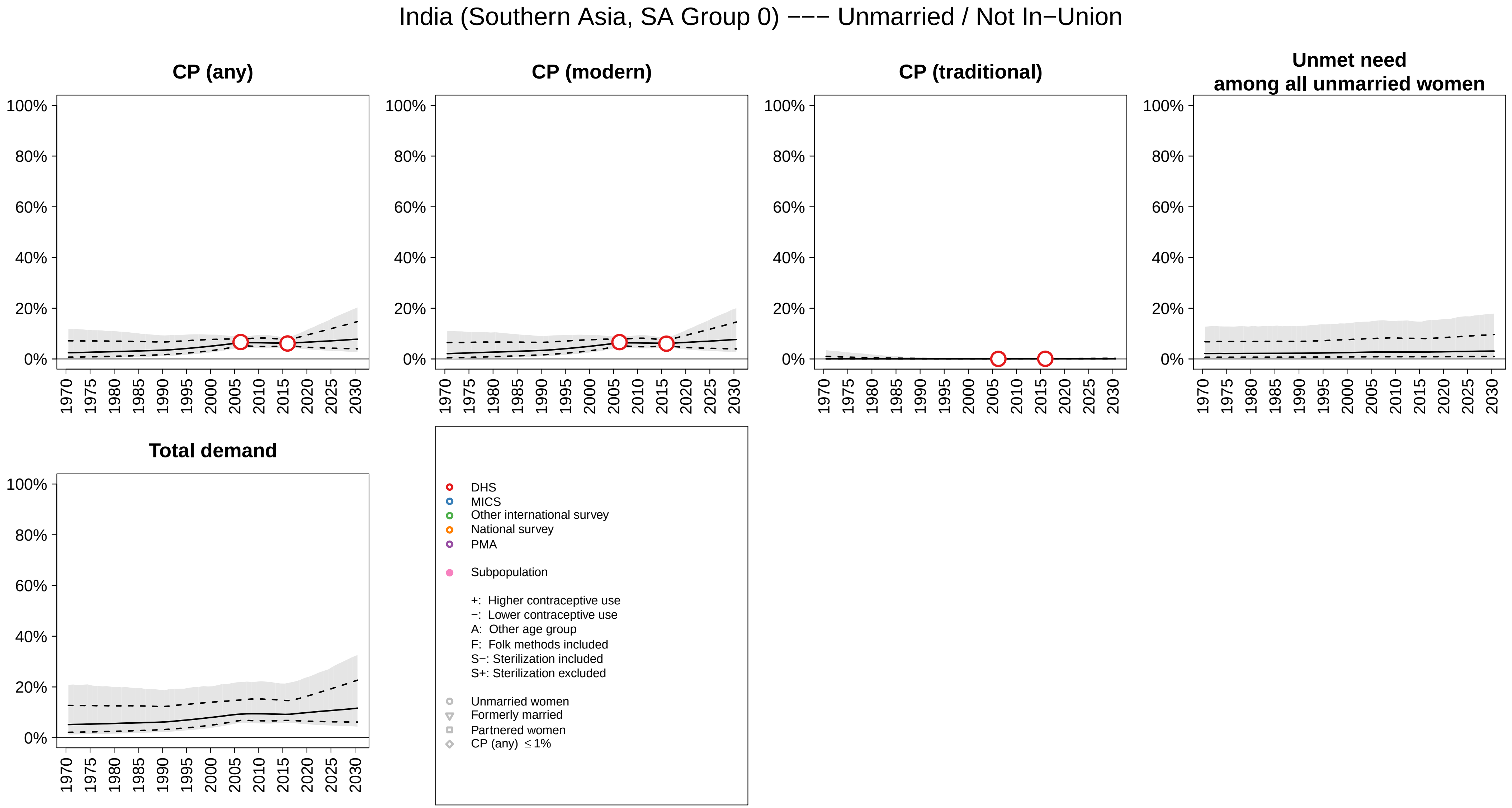
India (Southern Asia, SA Group 0) −−− Unmarried / Not In−Union
CP (any)
CP (modern)
CP (traditional)
Unmet need among all unmarried women
Total demand
100%
80%
60%
40%
20%
0%
1970 1975 1980 1985 1990 1995 2000 2005 2010 2015 2020 2025 2030
DHS
MICS
Other international survey
National survey
PMA
Subpopulation
+: Higher contraceptive use
−: Lower contraceptive use
A: Other age group
F: Folk methods included
S−: Sterilization included
S+: Sterilization excluded
Unmarried women
Formerly married
Partnered women
CP (any) ≤ 1%

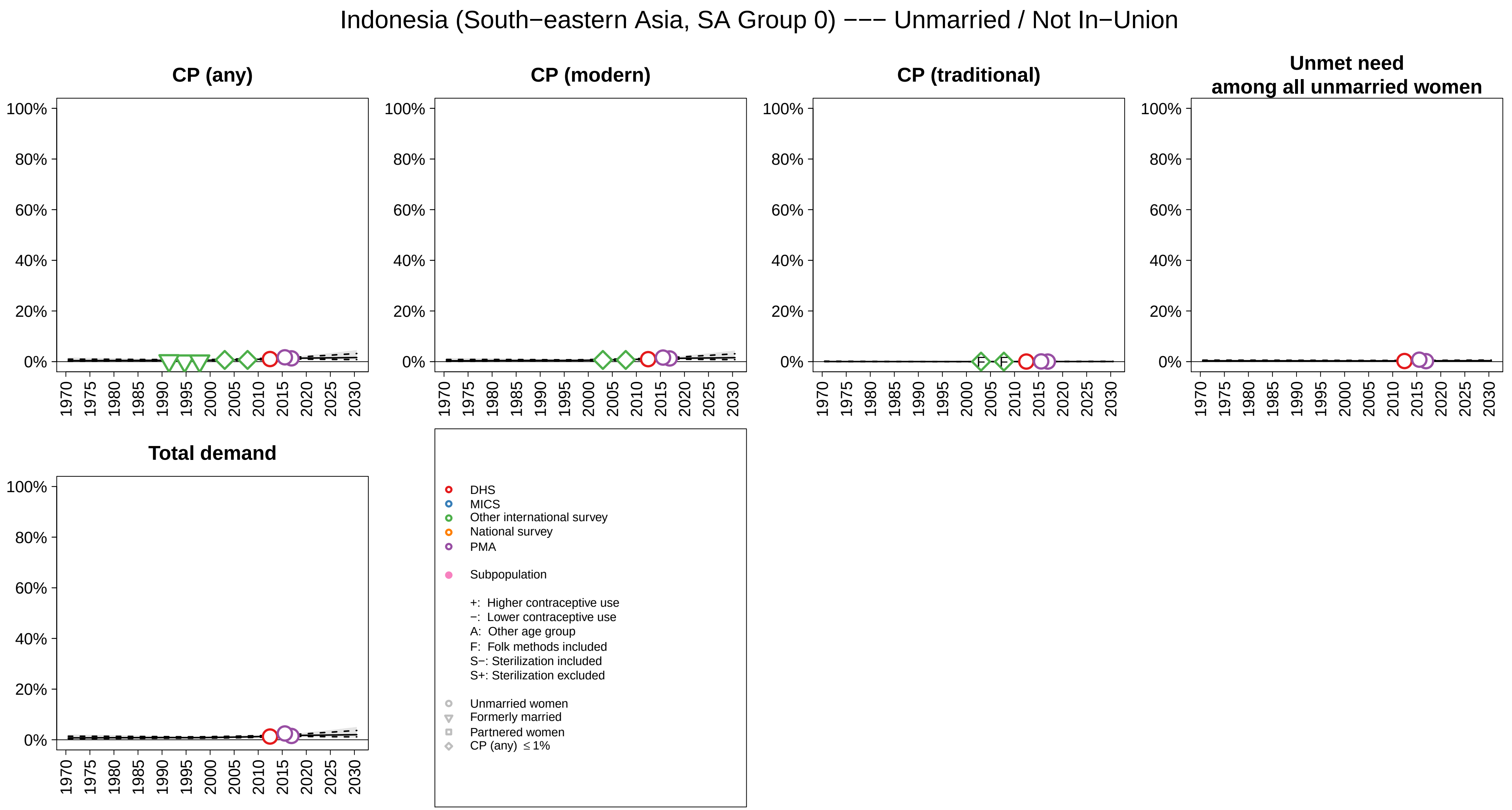
Indonesia (South−eastern Asia, SA Group 0) −−− Unmarried / Not In−Union
CP (any)
CP (modern)
CP (traditional)
Unmet need among all unmarried women
Total demand
0%
20%
40%
60%
80%
100%
1970
1975
1980
1985
1990
1995
2000
2005
2010
2015
2020
2025
2030
F
DHS
MICS
Other international survey
National survey
PMA
Subpopulation
+: Higher contraceptive use
−: Lower contraceptive use
A: Other age group
F: Folk methods included
S−: Sterilization included
S+: Sterilization excluded
Unmarried women
Formerly married
Partnered women
CP (any) ≤ 1%

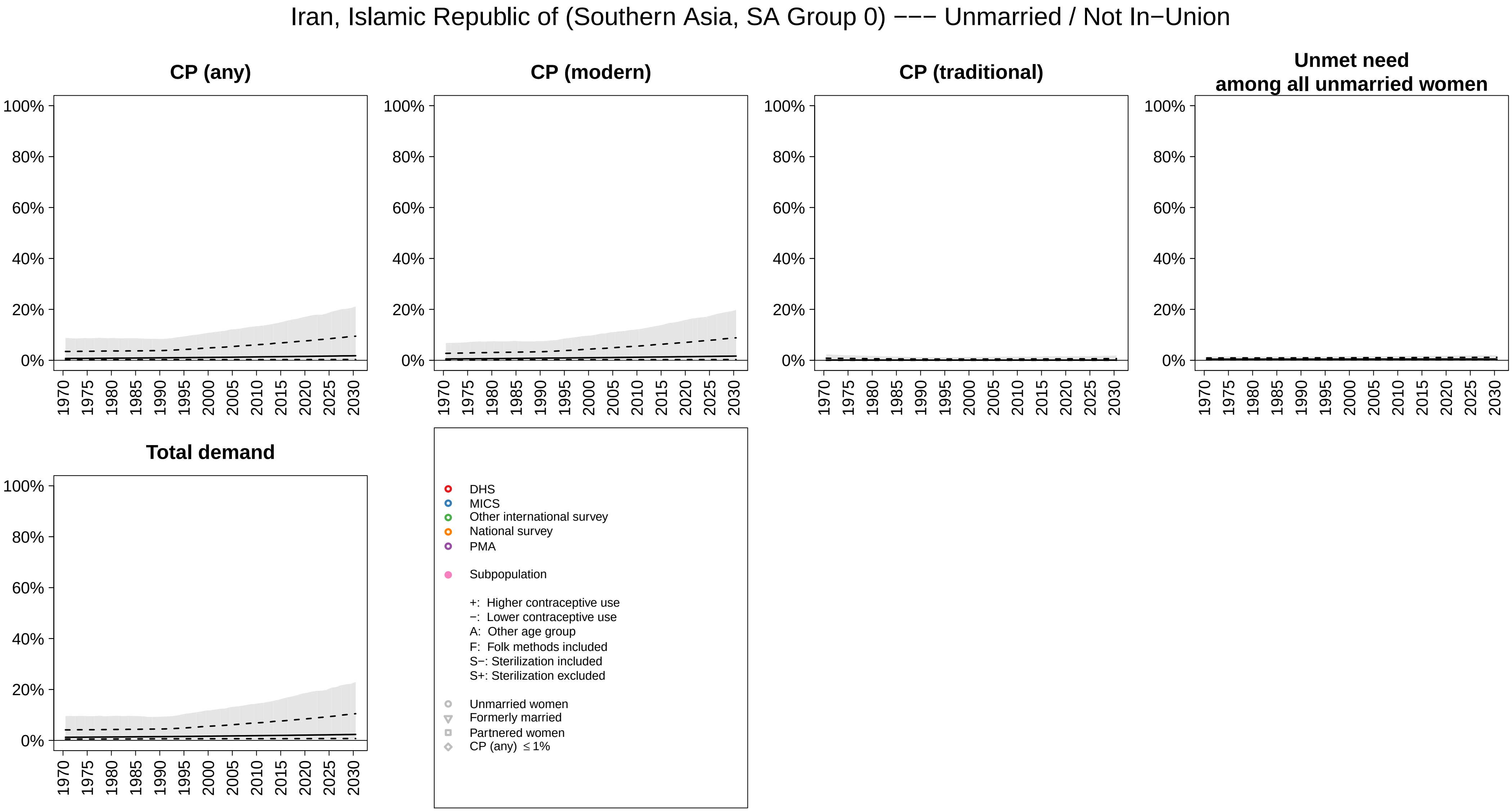
Iran, Islamic Republic of (Southern Asia, SA Group 0) −−− Unmarried / Not In−Union
CP (any)
CP (modern)
CP (traditional)
Unmet need among all unmarried women
Total demand
0%
20%
40%
60%
80%
100%
1970
1975
1980
1985
1990
1995
2000
2005
2010
2015
2020
2025
2030
DHS
MICS
Other international survey
National survey
PMA
Subpopulation
+: Higher contraceptive use
−: Lower contraceptive use
A: Other age group
F: Folk methods included
S−: Sterilization included
S+: Sterilization excluded
Unmarried women
Formerly married
Partnered women
CP (any) ≤ 1%

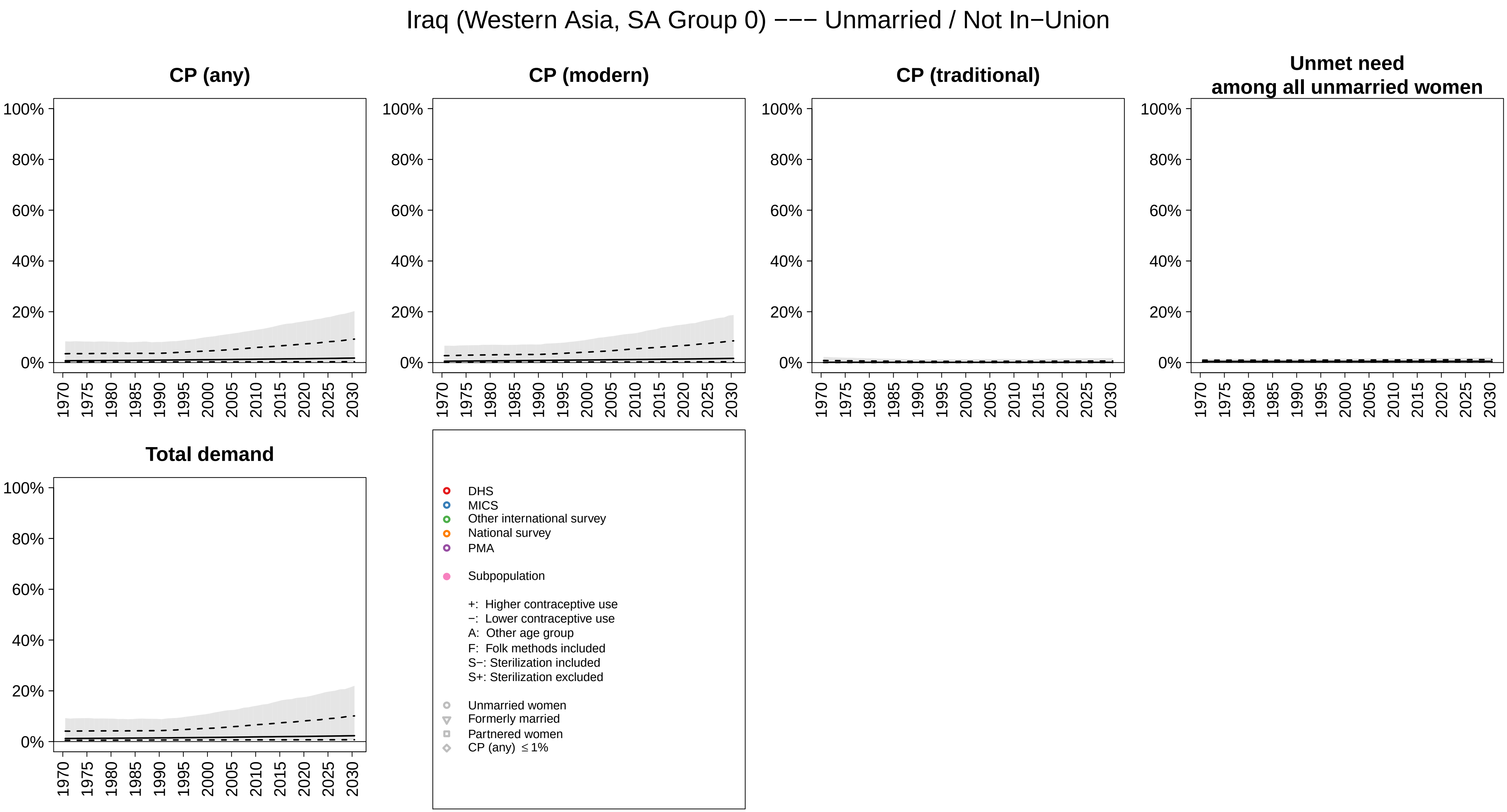
Iraq (Western Asia, SA Group 0) −−− Unmarried / Not In−Union
CP (any)
CP (modern)
CP (traditional)
Unmet need among all unmarried women
Total demand
100%
80%
60%
40%
20%
0%
1970
1975
1980
1985
1990
1995
2000
2005
2010
2015
2020
2025
2030
DHS
MICS
Other international survey
National survey
PMA
Subpopulation
+: Higher contraceptive use
−: Lower contraceptive use
A: Other age group
F: Folk methods included
S−: Sterilization included
S+: Sterilization excluded
Unmarried women
Formerly married
Partnered women
CP (any) ≤ 1%

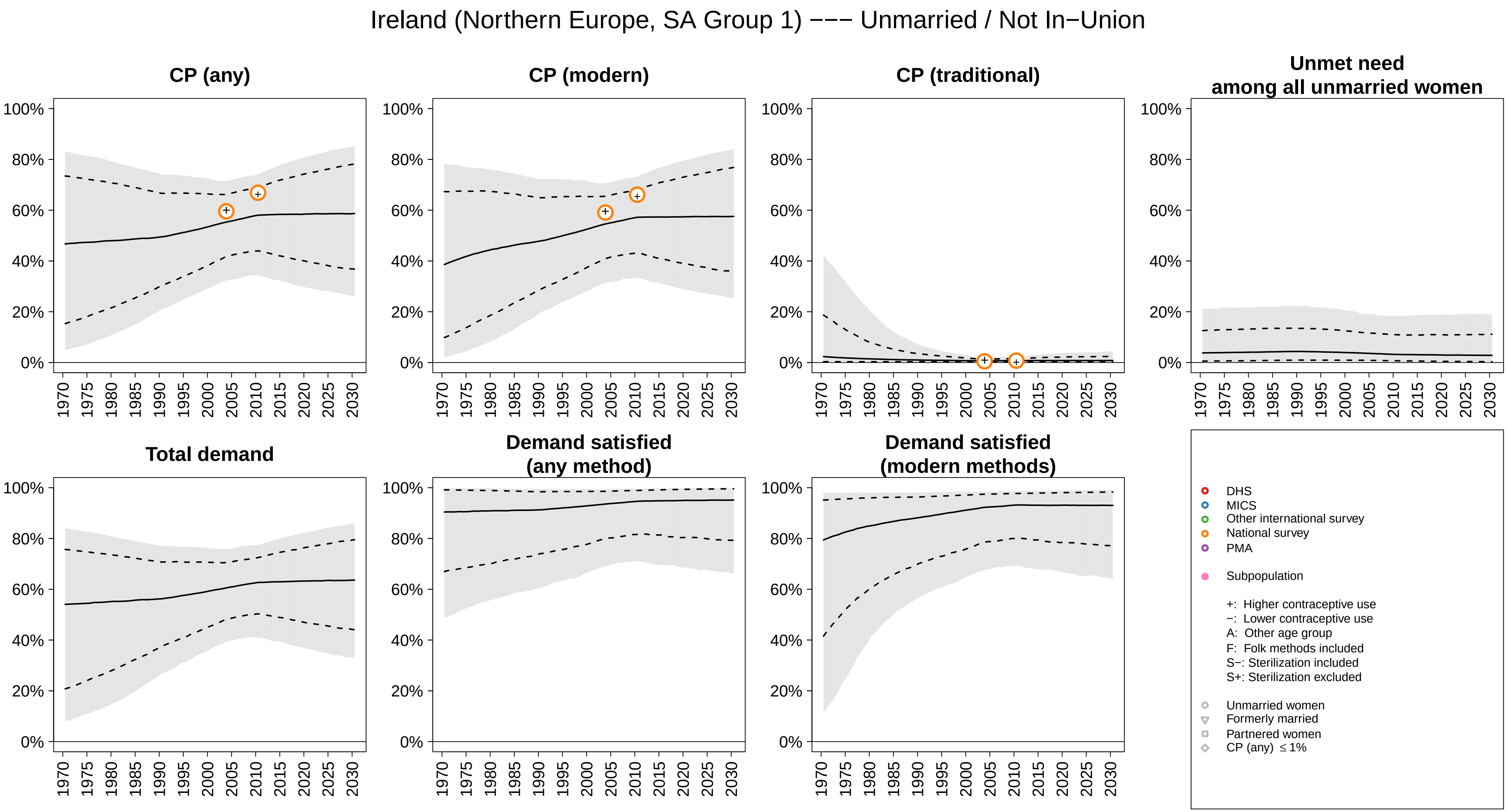
Ireland (Northern Europe, SA Group 1) −−− Unmarried / Not In−Union
CP (any)
CP (modern)
CP (traditional)
Unmet need among all unmarried women
Total demand
Demand satisfied (any method)
Demand satisfied (modern methods)
0%
20%
40%
60%
80%
100%
1970
1975
1980
1985
1990
1995
2000
2005
2010
2015
2020
2025
2030
DHS
MICS
Other international survey
National survey
PMA
Subpopulation
+: Higher contraceptive use
−: Lower contraceptive use
A: Other age group
F: Folk methods included
S−: Sterilization included
S+: Sterilization excluded
Unmarried women
Formerly married
Partnered women
CP (any) ≤ 1%

Israel (Western Asia, SA Group 1) −−− Unmarried / Not In−Union

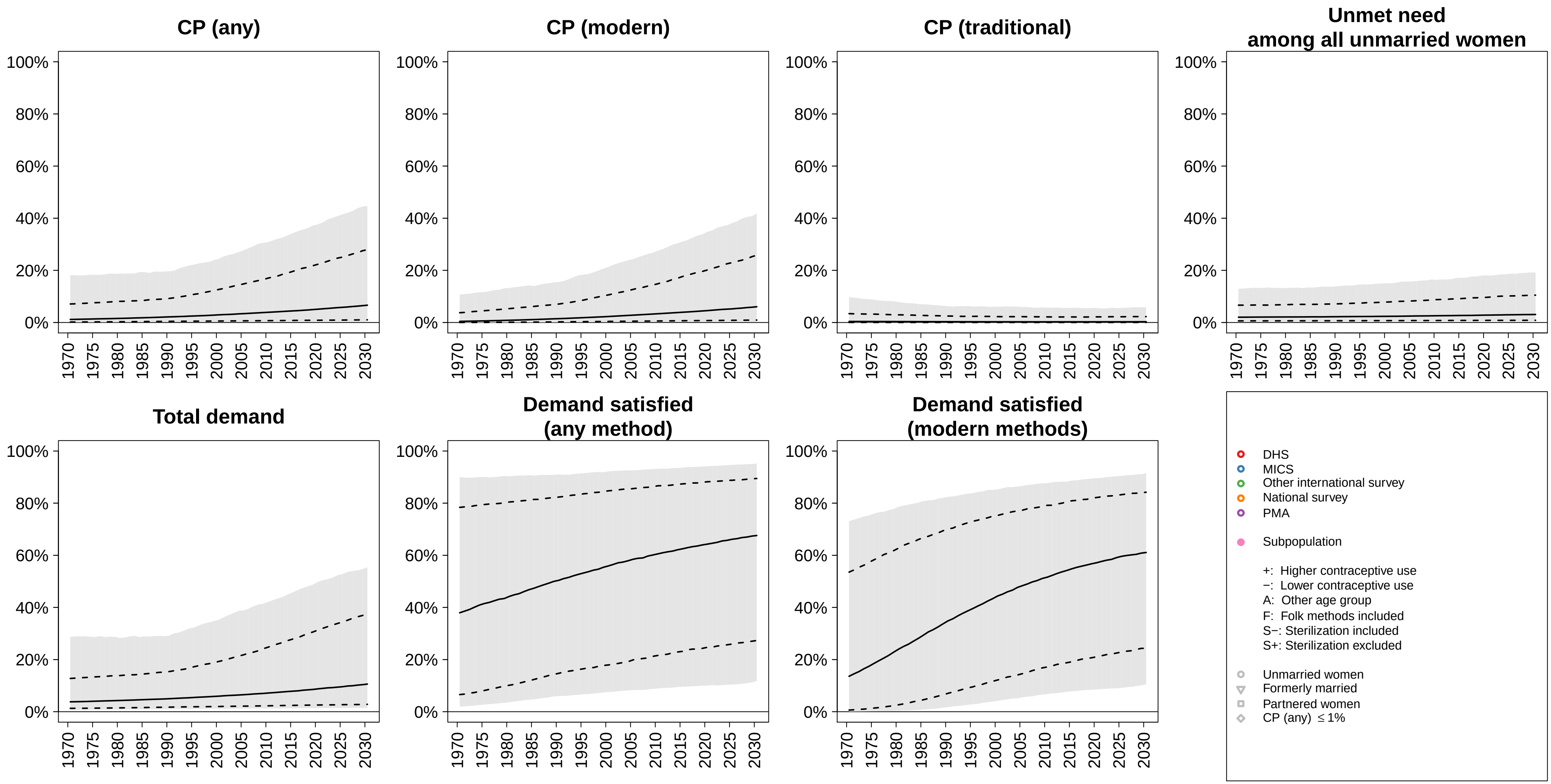

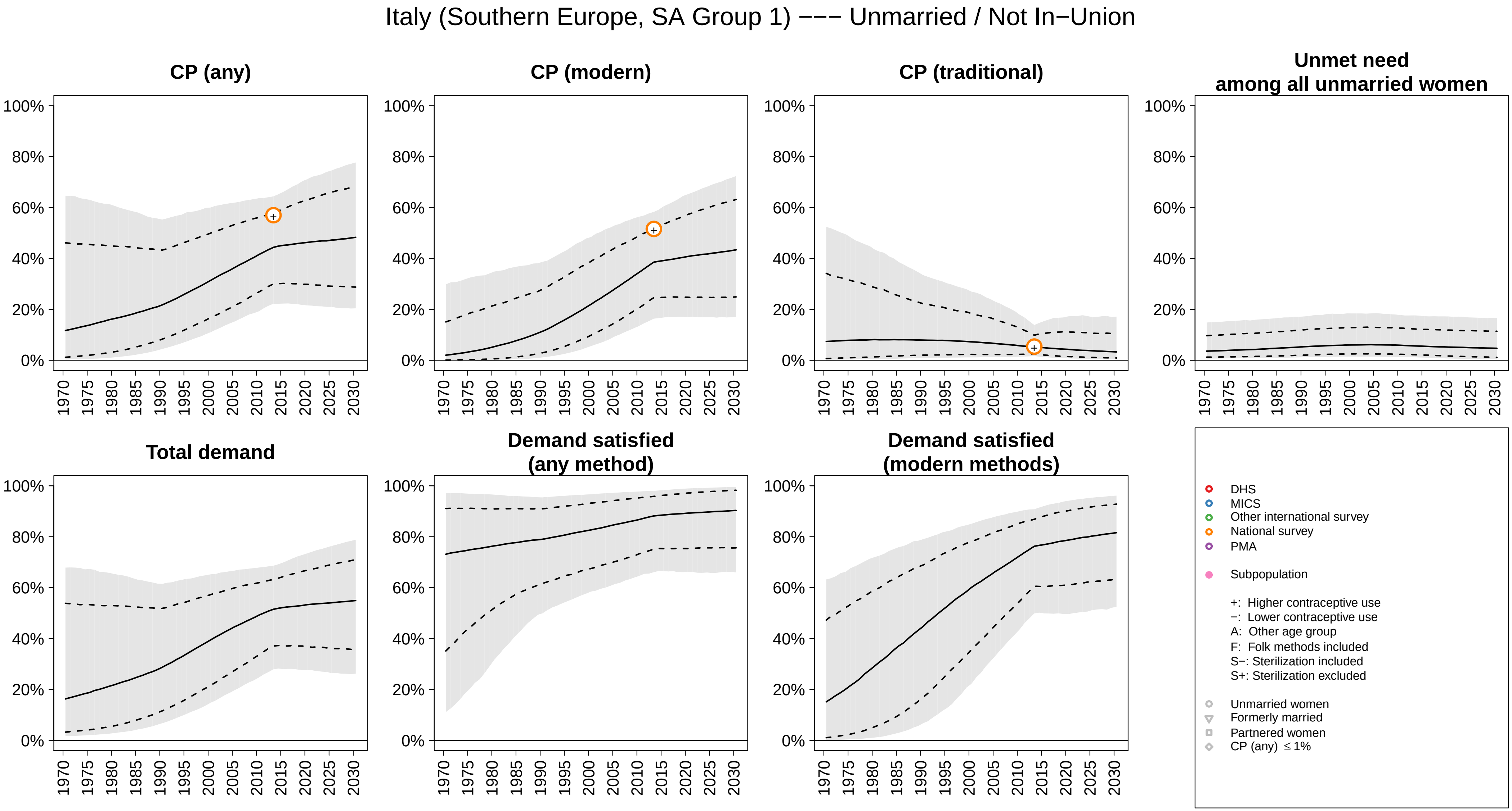
Italy (Southern Europe, SA Group 1) −−− Unmarried / Not In−Union
CP (any)
CP (modern)
CP (traditional)
Unmet need
among all unmarried women
Total demand
Demand satisfied
(any method)
Demand satisfied
(modern methods)
0%
20%
40%
60%
80%
100%
1970
1975
1980
1985
1990
1995
2000
2005
2010
2015
2020
2025
2030
DHS
MICS
Other international survey
National survey
PMA
Subpopulation
+: Higher contraceptive use
−: Lower contraceptive use
A: Other age group
F: Folk methods included
S−: Sterilization included
S+: Sterilization excluded
Unmarried women
Formerly married
Partnered women
CP (any) ≤ 1%

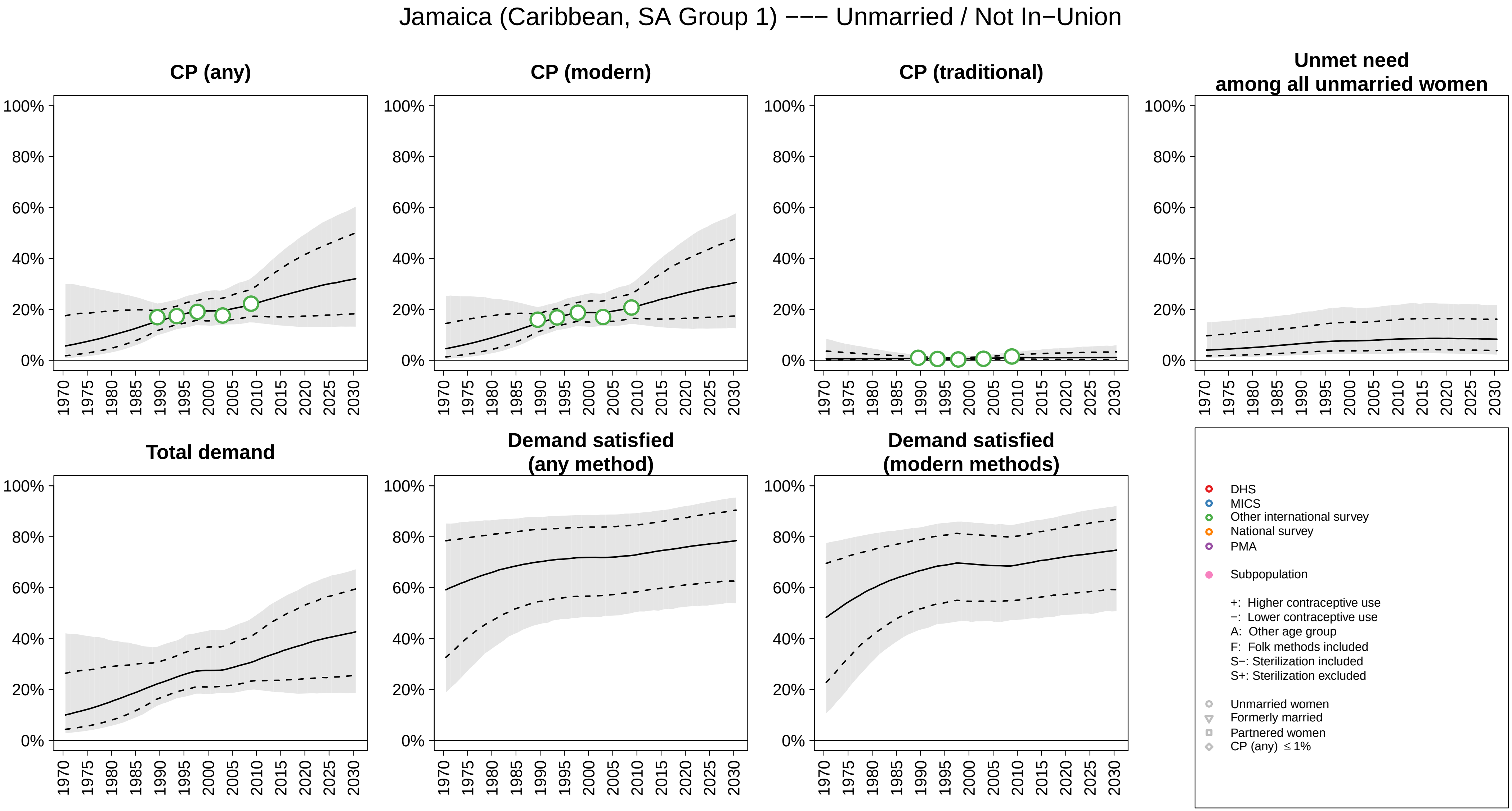
Jamaica (Caribbean, SA Group 1) −−− Unmarried / Not In−Union
CP (any)
CP (modern)
CP (traditional)
Unmet need among all unmarried women
Total demand
Demand satisfied (any method)
Demand satisfied (modern methods)
100%
80%
60%
40%
20%
0%
1970
1975
1980
1985
1990
1995
2000
2005
2010
2015
2020
2025
2030
DHS
MICS
Other international survey
National survey
PMA
Subpopulation
+: Higher contraceptive use
−: Lower contraceptive use
A: Other age group
F: Folk methods included
S−: Sterilization included
S+: Sterilization excluded
Unmarried women
Formerly married
Partnered women
CP (any) ≤ 1%

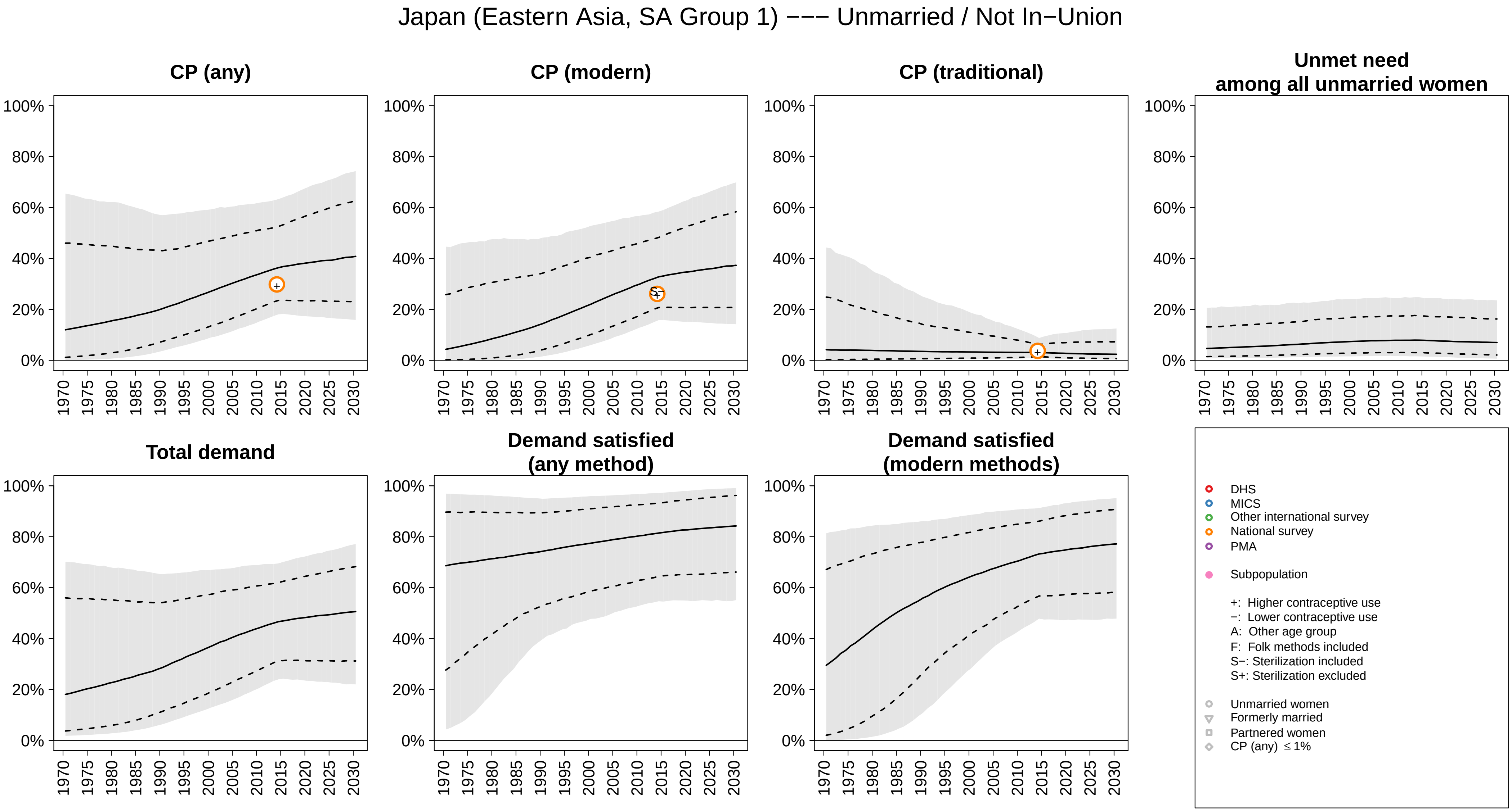
Japan (Eastern Asia, SA Group 1) −−− Unmarried / Not In−Union
CP (any)
CP (modern)
CP (traditional)
Unmet need among all unmarried women
Total demand
Demand satisfied (any method)
Demand satisfied (modern methods)
DHS
MICS
Other international survey
National survey
PMA
Subpopulation
+: Higher contraceptive use
−: Lower contraceptive use
A: Other age group
F: Folk methods included
S−: Sterilization included
S+: Sterilization excluded
Unmarried women
Formerly married
Partnered women
CP (any) ≤ 1%

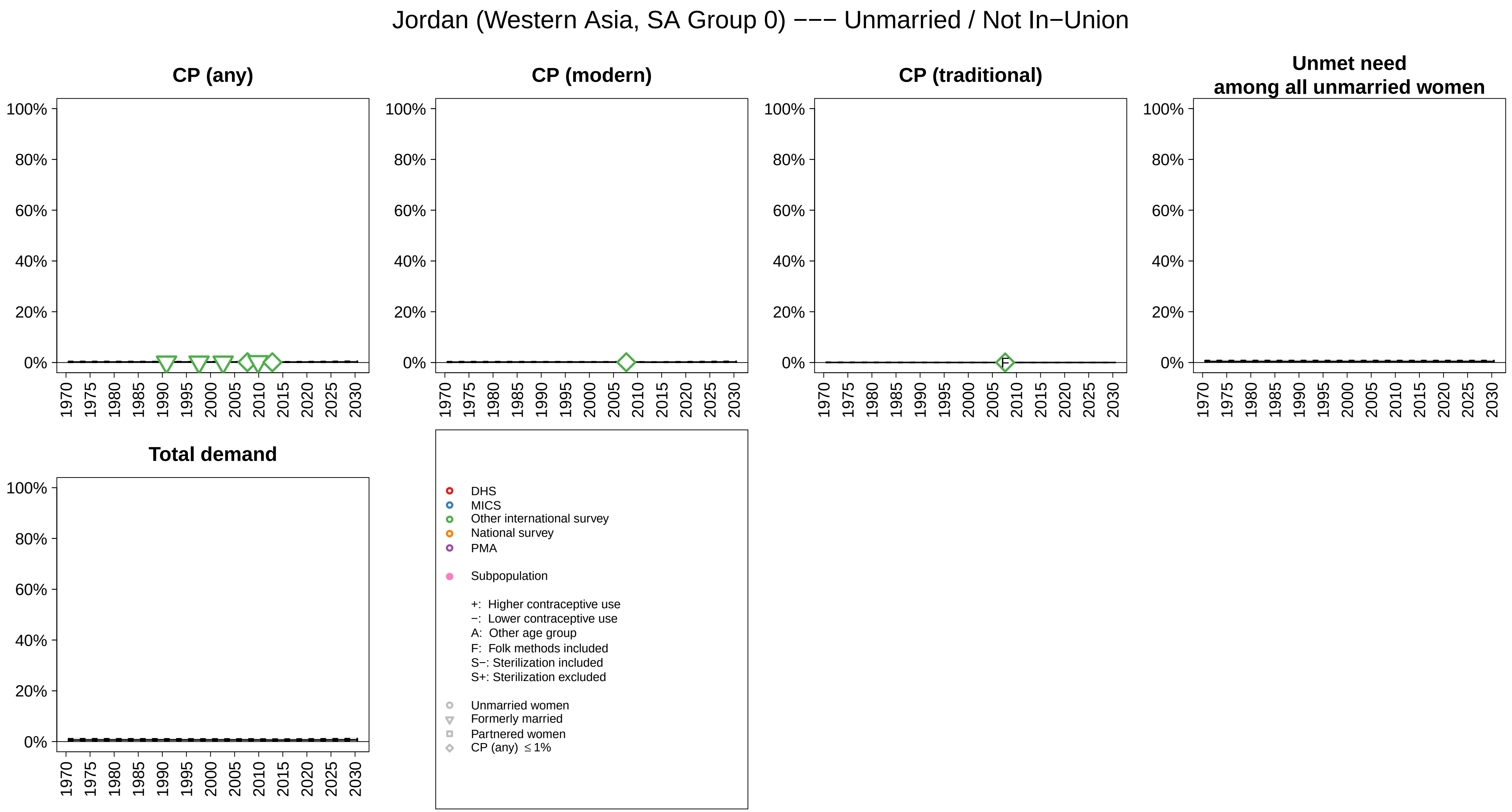
Jordan (Western Asia, SA Group 0) −−− Unmarried / Not In−Union
CP (any)
CP (modern)
CP (traditional)
Unmet need among all unmarried women
Total demand
100%
80%
60%
40%
20%
0%
1970
1975
1980
1985
1990
1995
2000
2005
2010
2015
2020
2025
2030
F
DHS
MICS
Other international survey
National survey
PMA
Subpopulation
+: Higher contraceptive use
−: Lower contraceptive use
A: Other age group
F: Folk methods included
S−: Sterilization included
S+: Sterilization excluded
Unmarried women
Formerly married
Partnered women
CP (any) ≤ 1%

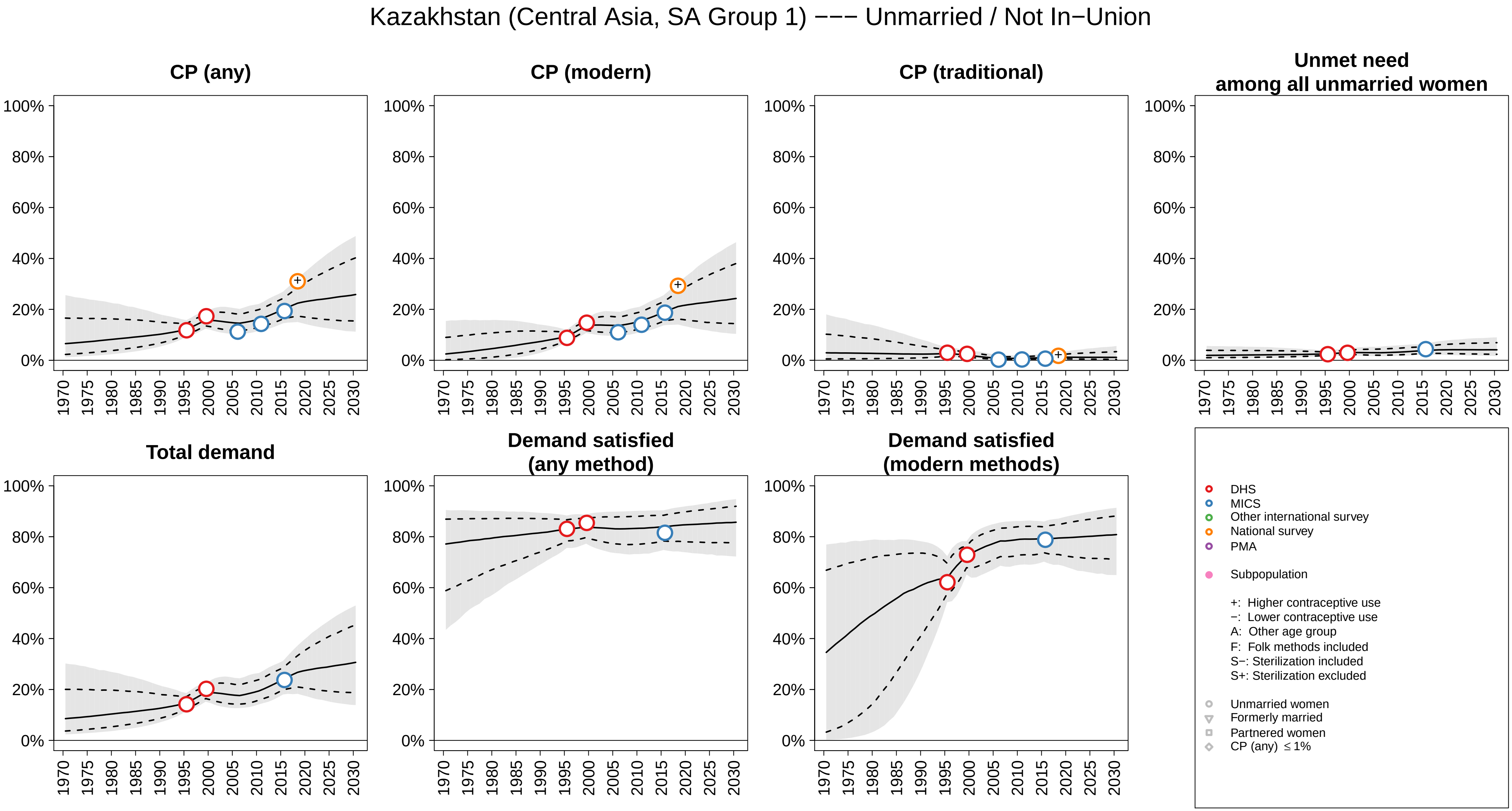
Kazakhstan (Central Asia, SA Group 1) −−− Unmarried / Not In−Union
CP (any)
CP (modern)
CP (traditional)
Unmet need among all unmarried women
Total demand
Demand satisfied (any method)
Demand satisfied (modern methods)
0%
20%
40%
60%
80%
100%
1970
1975
1980
1985
1990
1995
2000
2005
2010
2015
2020
2025
2030
DHS
MICS
Other international survey
National survey
PMA
Subpopulation
+: Higher contraceptive use
−: Lower contraceptive use
A: Other age group
F: Folk methods included
S−: Sterilization included
S+: Sterilization excluded
Unmarried women
Formerly married
Partnered women
CP (any) ≤ 1%

Kenya (Eastern Africa, SA Group 1) −−− Unmarried / Not In−Union

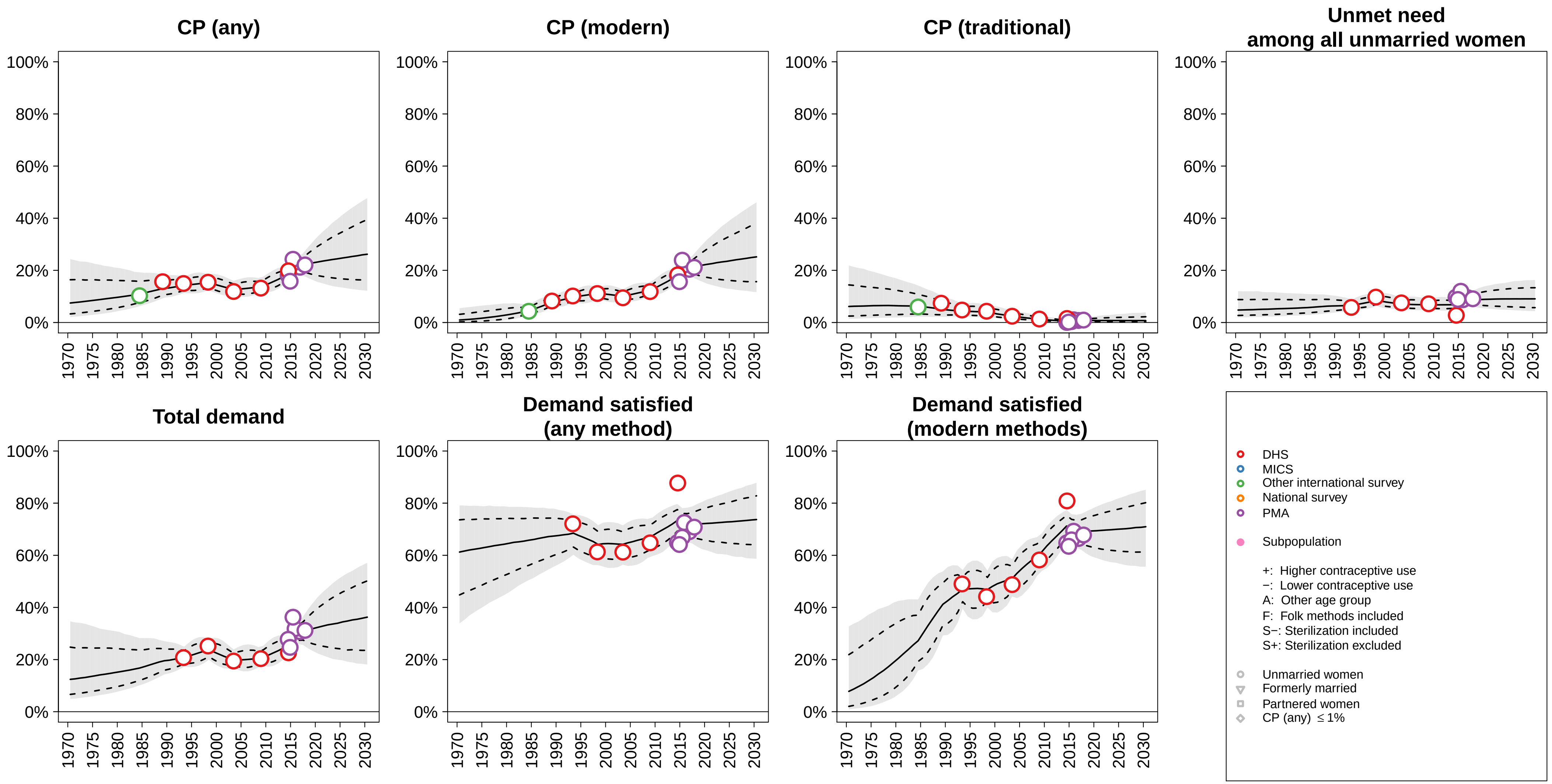

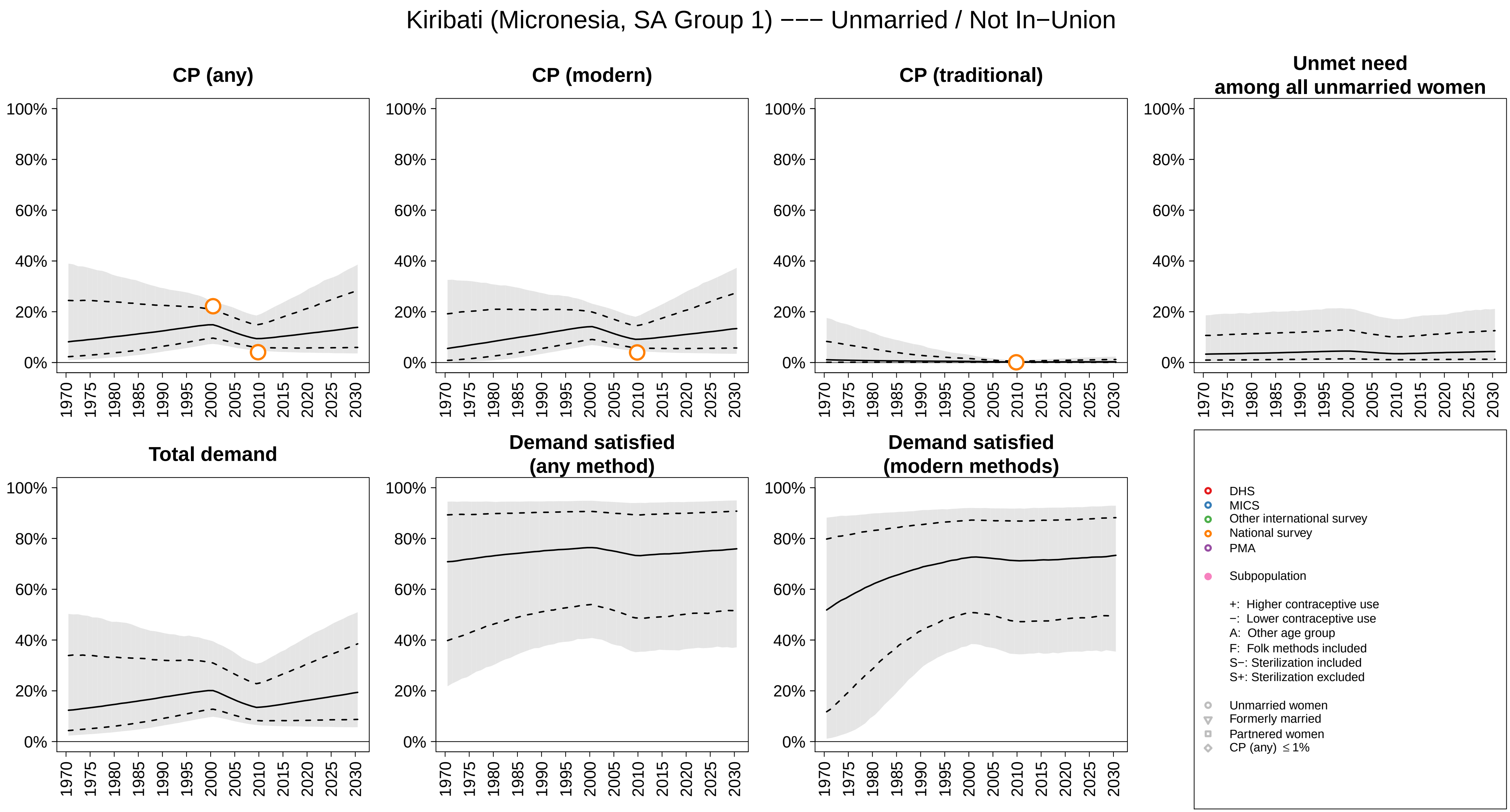
Kiribati (Micronesia, SA Group 1) −−− Unmarried / Not In−Union
CP (any)
CP (modern)
CP (traditional)
Unmet need among all unmarried women
Total demand
Demand satisfied (any method)
Demand satisfied (modern methods)
100%
80%
60%
40%
20%
0%
1970 1975 1980 1985 1990 1995 2000 2005 2010 2015 2020 2025 2030
DHS
MICS
Other international survey
National survey
PMA
Subpopulation
+: Higher contraceptive use
−: Lower contraceptive use
A: Other age group
F: Folk methods included
S−: Sterilization included
S+: Sterilization excluded
Unmarried women
Formerly married
Partnered women
CP (any) ≤ 1%

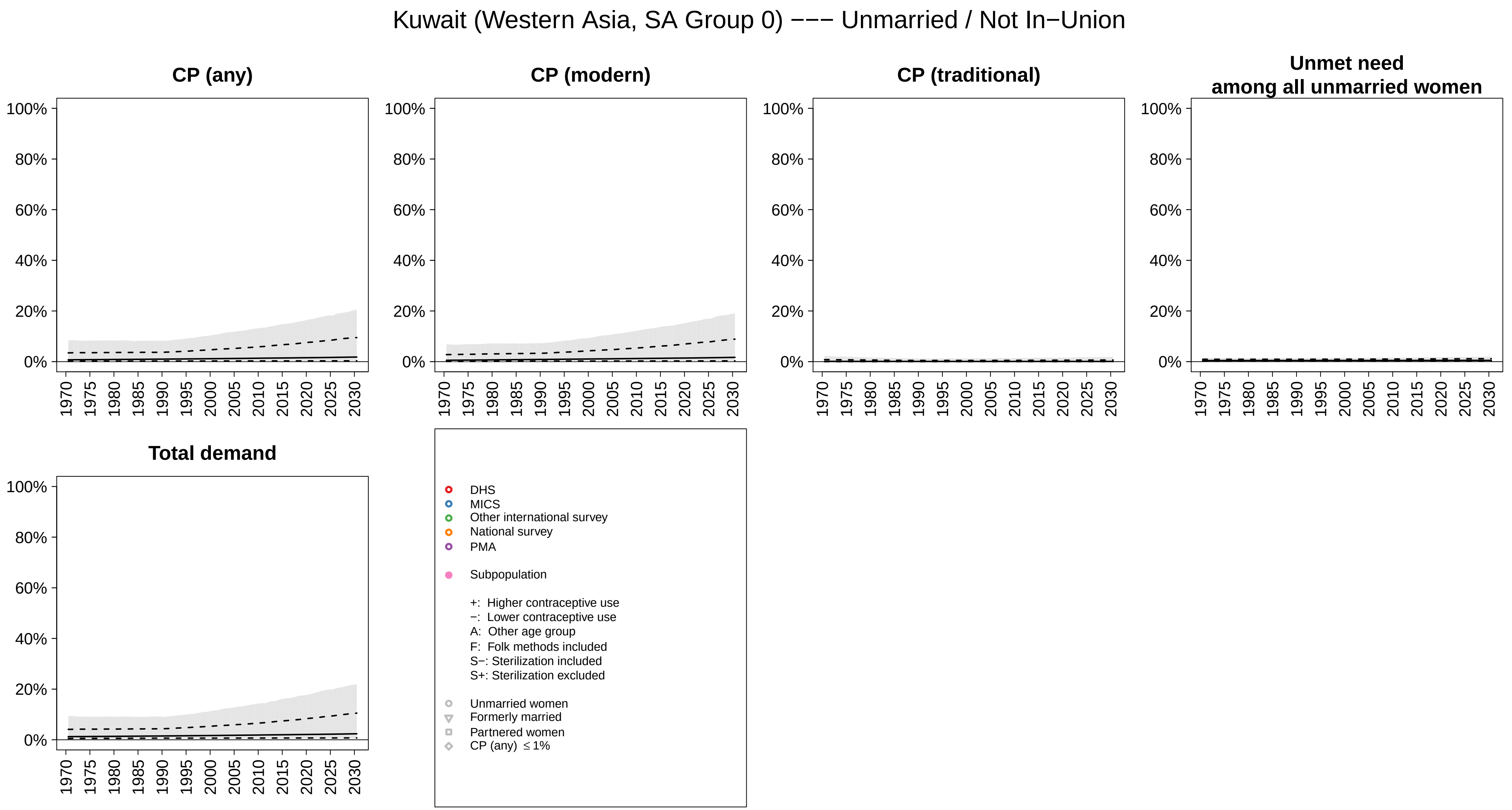
Kuwait (Western Asia, SA Group 0) −−− Unmarried / Not In−Union
CP (any)
CP (modern)
CP (traditional)
Unmet need among all unmarried women
Total demand
100%
80%
60%
40%
20%
0%
1970
1975
1980
1985
1990
1995
2000
2005
2010
2015
2020
2025
2030
DHS
MICS
Other international survey
National survey
PMA
Subpopulation
+: Higher contraceptive use
−: Lower contraceptive use
A: Other age group
F: Folk methods included
S−: Sterilization included
S+: Sterilization excluded
Unmarried women
Formerly married
Partnered women
CP (any) ≤ 1%

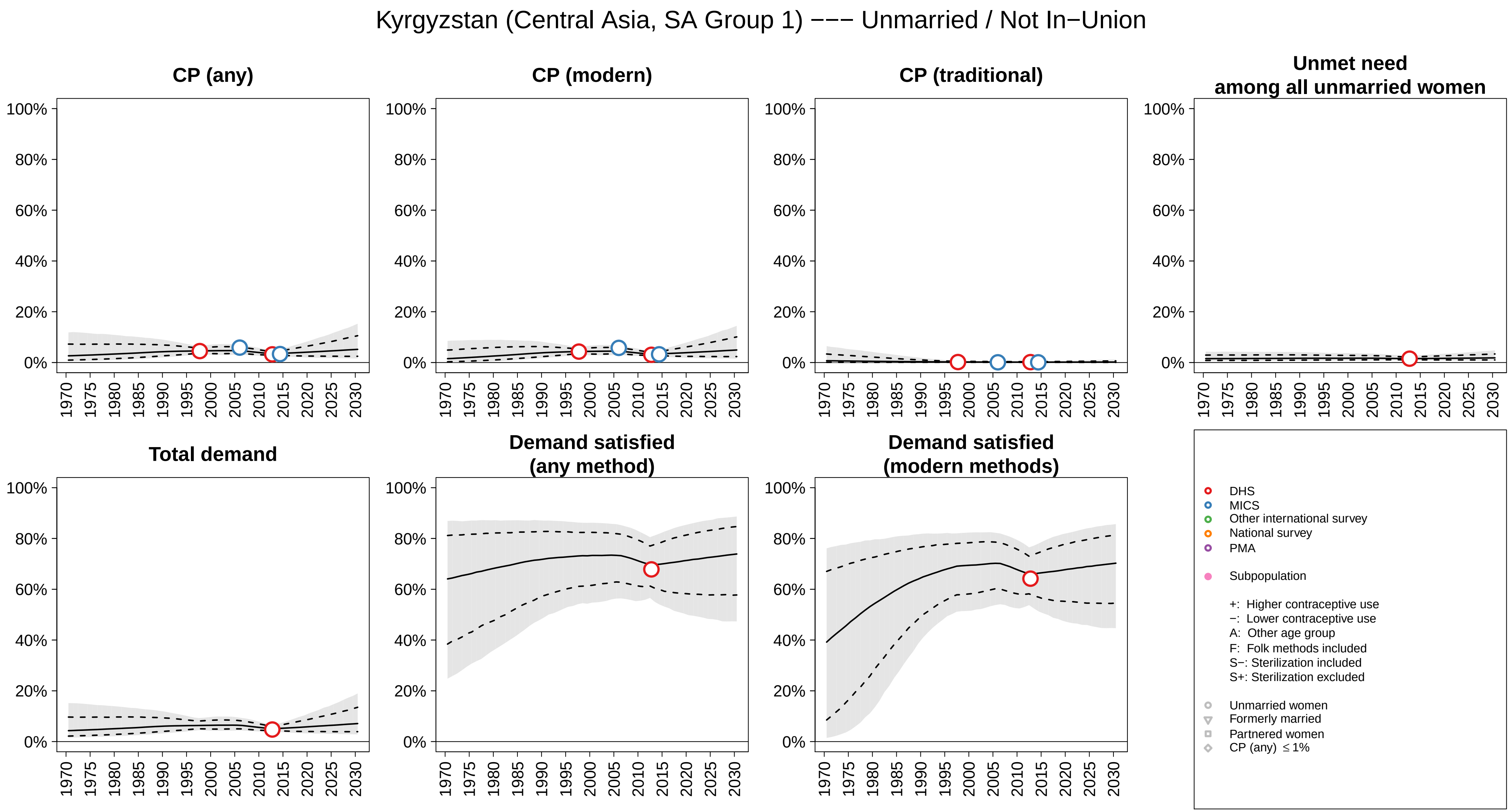
Kyrgyzstan (Central Asia, SA Group 1) −−− Unmarried / Not In−Union
CP (any)
CP (modern)
CP (traditional)
Unmet need among all unmarried women
Total demand
Demand satisfied (any method)
Demand satisfied (modern methods)
0%
20%
40%
60%
80%
100%
1970
1975
1980
1985
1990
1995
2000
2005
2010
2015
2020
2025
2030
DHS
MICS
Other international survey
National survey
PMA
Subpopulation
+: Higher contraceptive use
−: Lower contraceptive use
A: Other age group
F: Folk methods included
S−: Sterilization included
S+: Sterilization excluded
Unmarried women
Formerly married
Partnered women
CP (any) ≤ 1%

Lao People's Dem. Republic (South−eastern Asia, SA Group 0) −−− Unmarried / Not In−Union

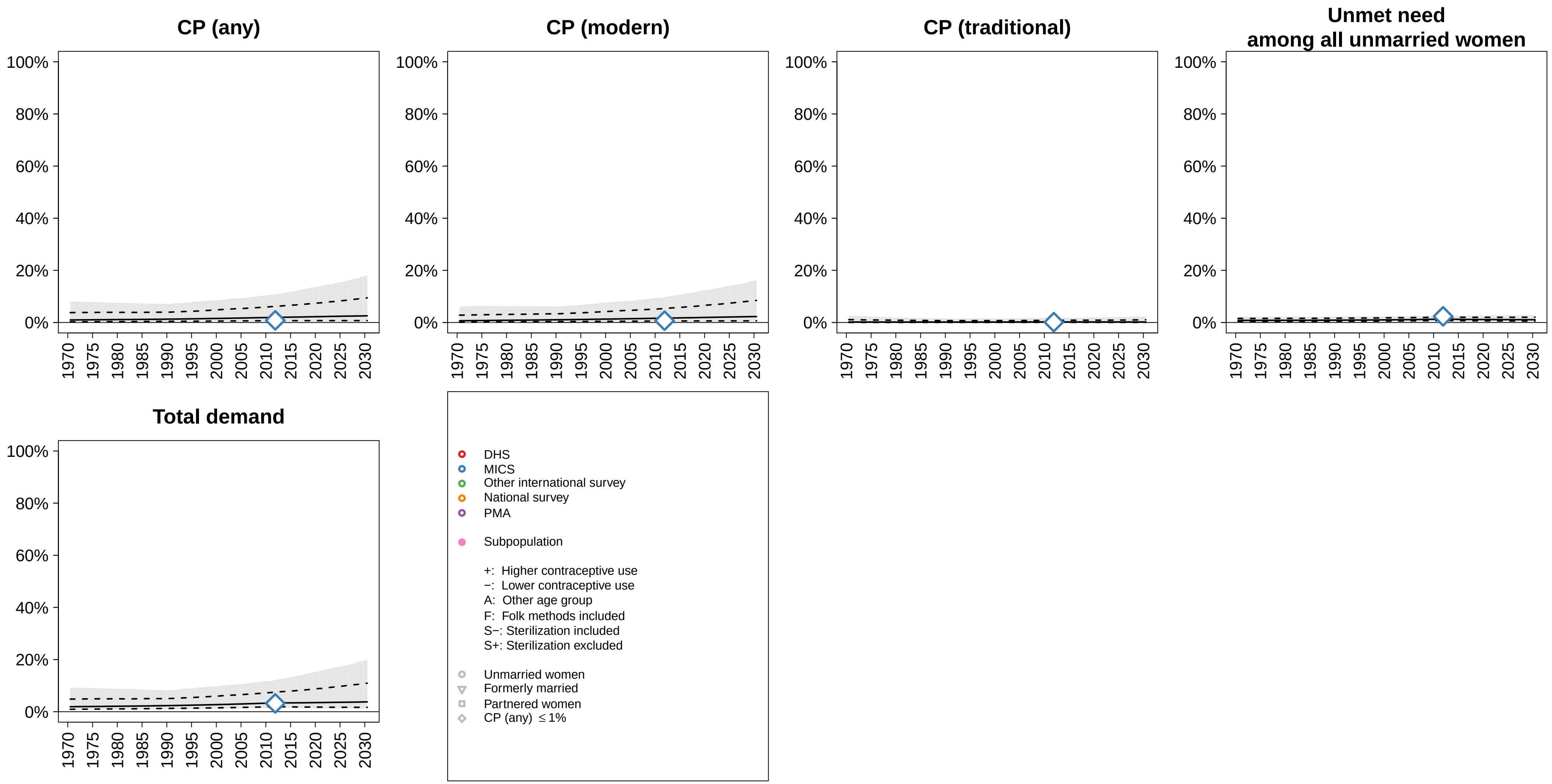

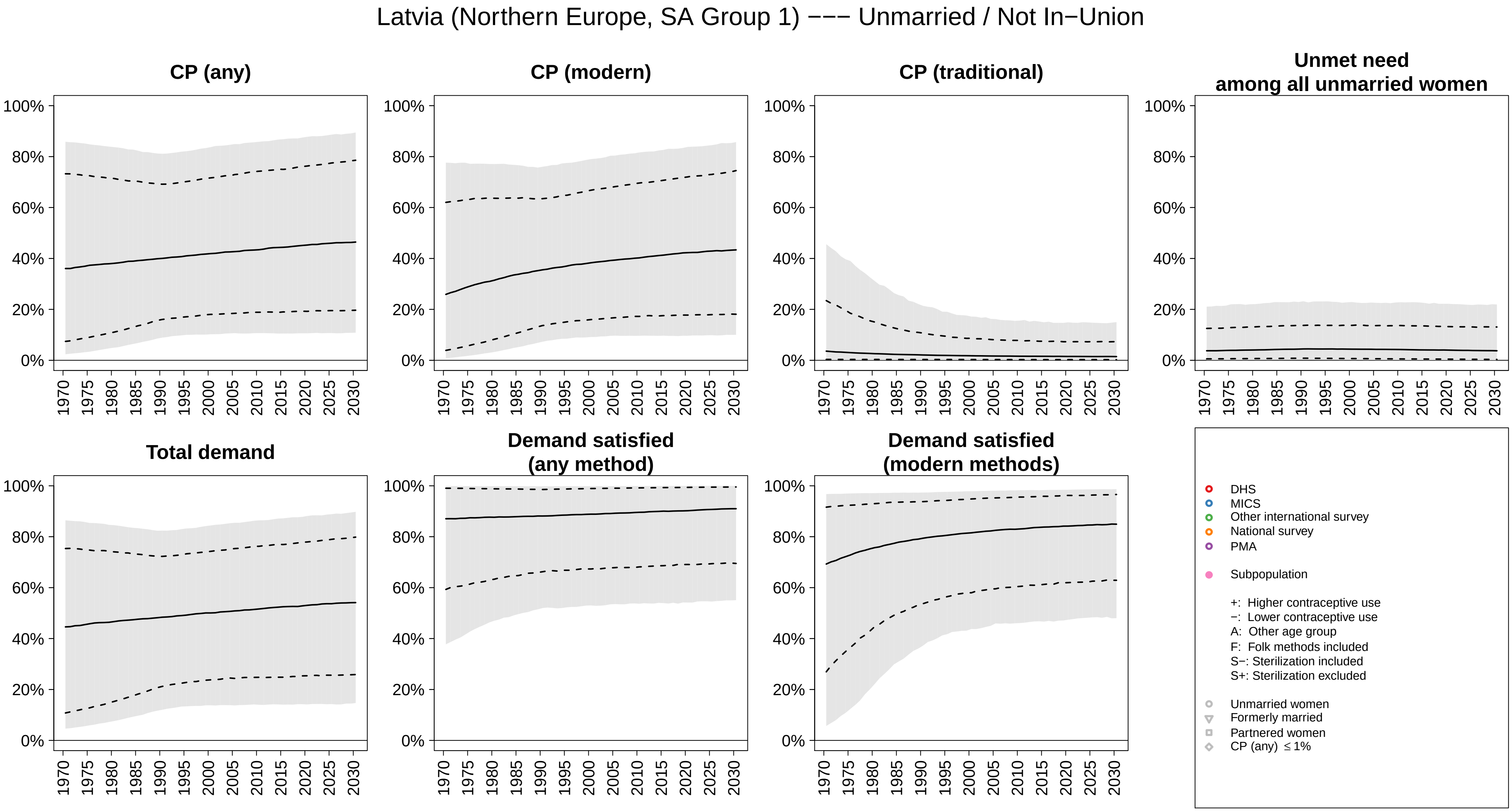
Latvia (Northern Europe, SA Group 1) −−− Unmarried / Not In−Union
CP (any)
CP (modern)
CP (traditional)
Unmet need among all unmarried women
Total demand
Demand satisfied (any method)
Demand satisfied (modern methods)
0%
20%
40%
60%
80%
100%
1970
1975
1980
1985
1990
1995
2000
2005
2010
2015
2020
2025
2030
DHS
MICS
Other international survey
National survey
PMA
Subpopulation
+: Higher contraceptive use
−: Lower contraceptive use
A: Other age group
F: Folk methods included
S−: Sterilization included
S+: Sterilization excluded
Unmarried women
Formerly married
Partnered women
CP (any) ≤ 1%

Lebanon (Western Asia, SA Group 0) −−− Unmarried / Not In−Union

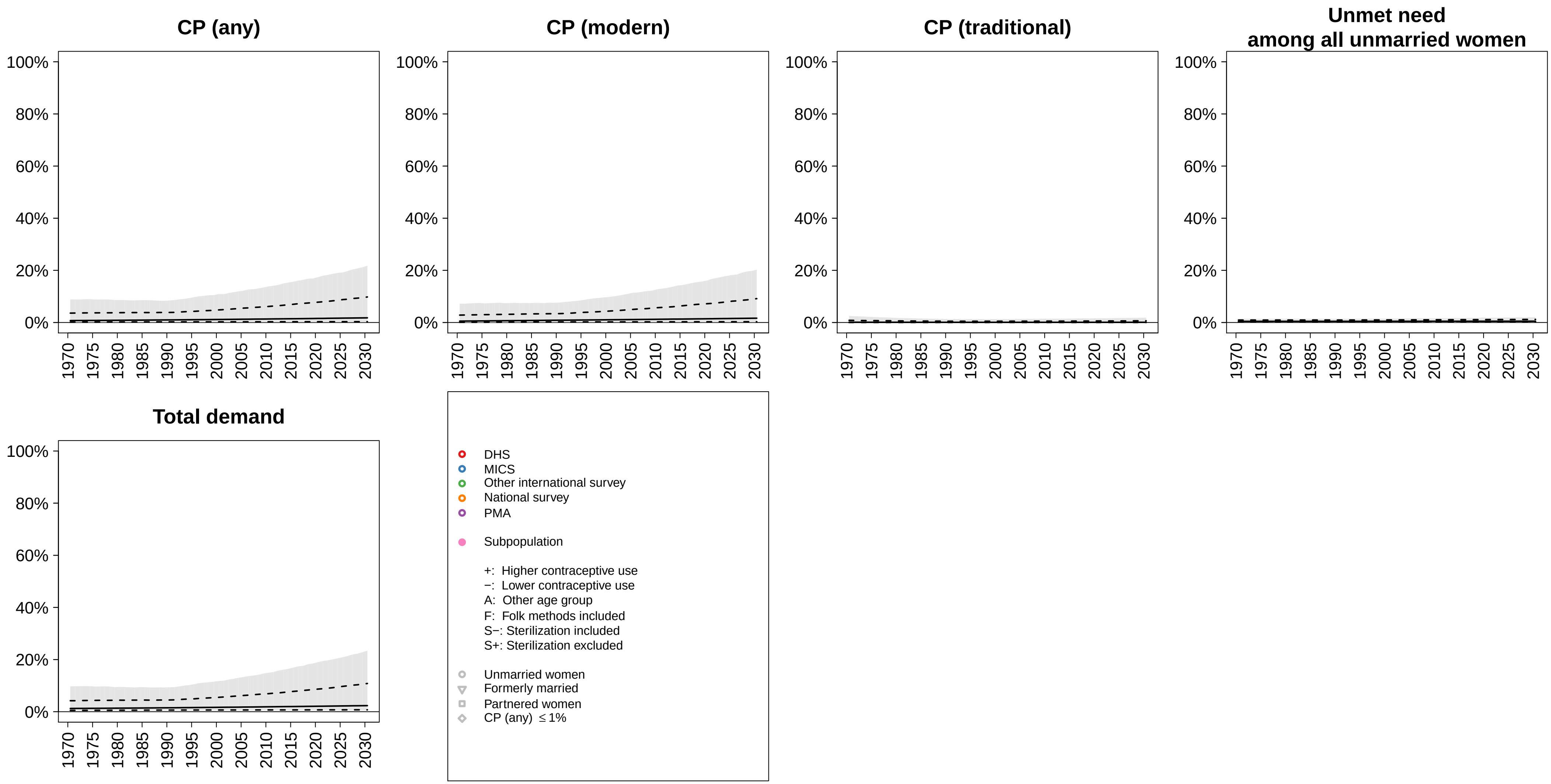

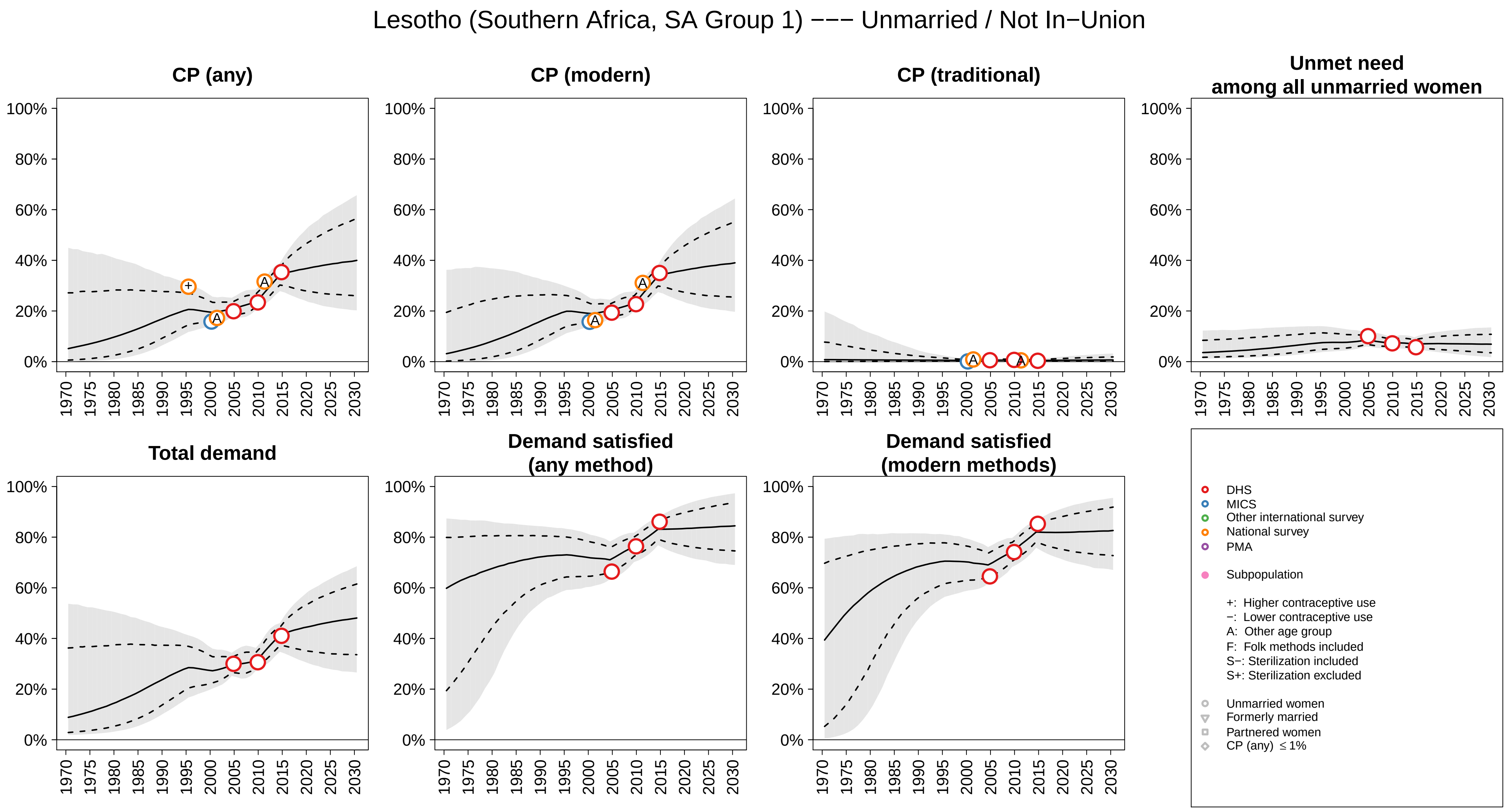
Lesotho (Southern Africa, SA Group 1) --- Unmarried / Not In−Union
CP (any)
CP (modern)
CP (traditional)
Unmet need among all unmarried women
Total demand
Demand satisfied (any method)
Demand satisfied (modern methods)
0%
20%
40%
60%
80%
100%
1970
1975
1980
1985
1990
1995
2000
2005
2010
2015
2020
2025
2030
DHS
MICS
Other international survey
National survey
PMA
Subpopulation
+: Higher contraceptive use
−: Lower contraceptive use
A: Other age group
F: Folk methods included
S−: Sterilization included
S+: Sterilization excluded
Unmarried women
Formerly married
Partnered women
CP (any) ≤ 1%

Liberia (Western Africa, SA Group 1) −−− Unmarried / Not In−Union

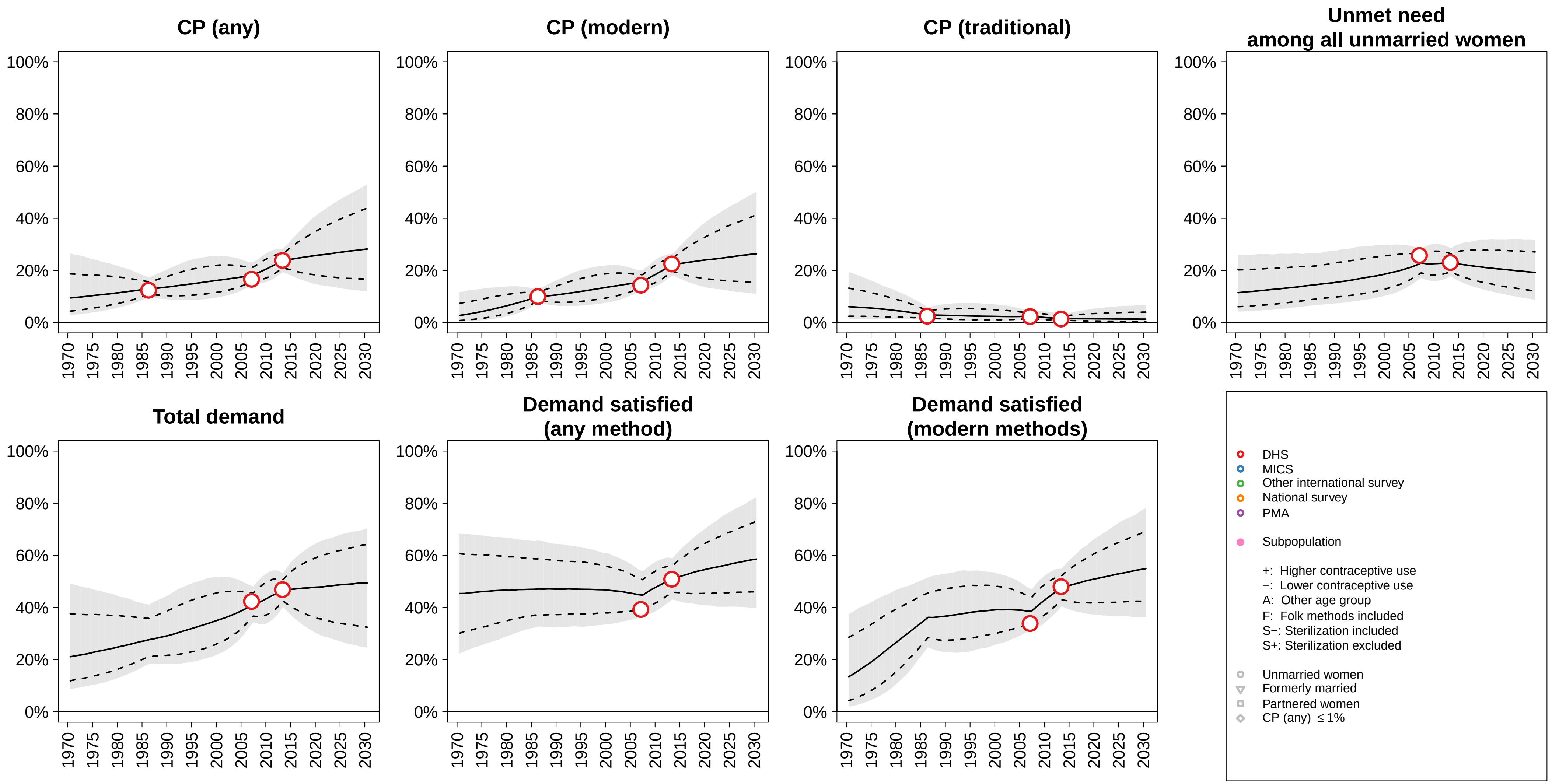

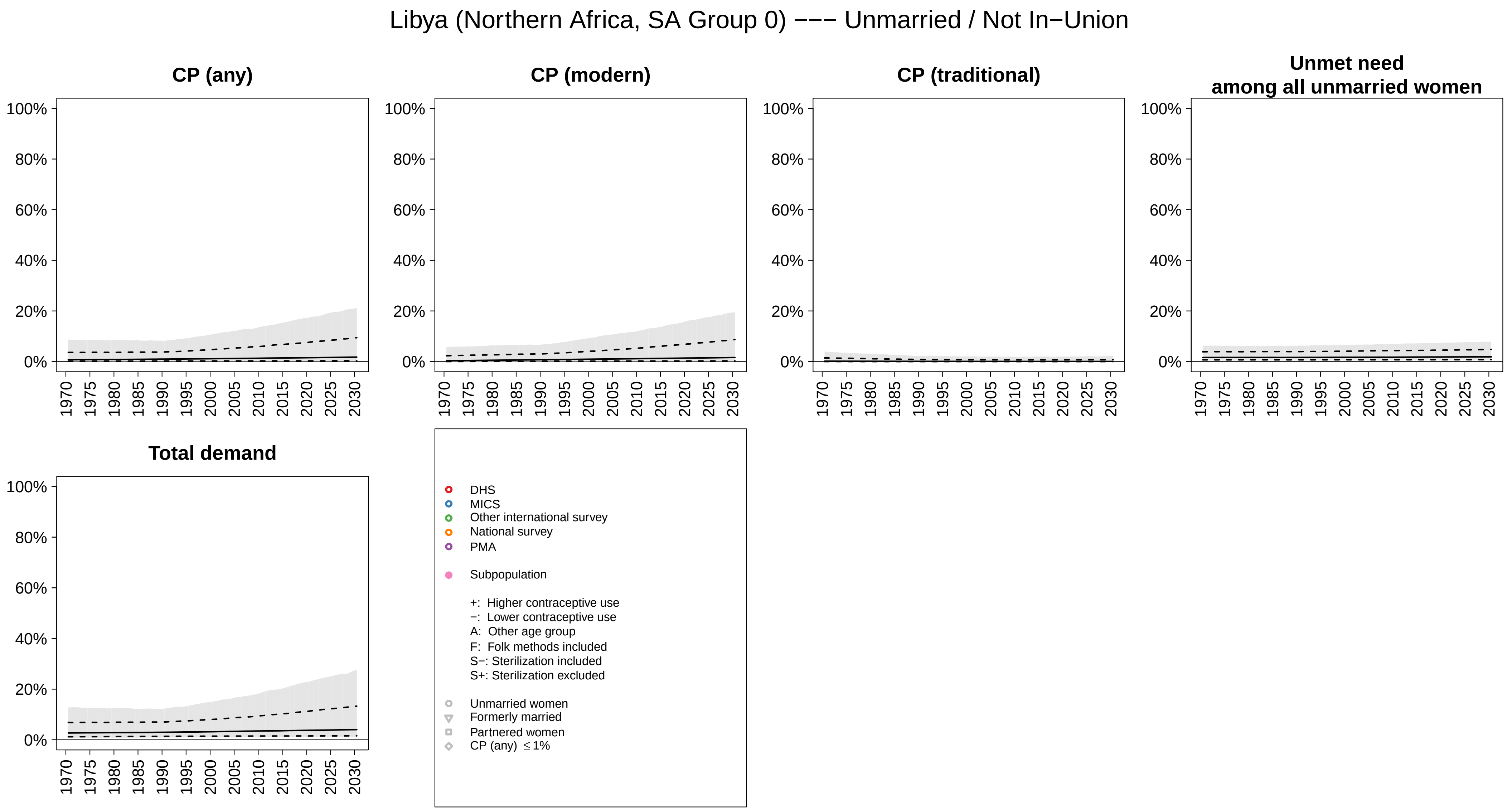
Libya (Northern Africa, SA Group 0) −−− Unmarried / Not In−Union
CP (any)
CP (modern)
CP (traditional)
Unmet need among all unmarried women
Total demand
100%
80%
60%
40%
20%
0%
1970 1975 1980 1985 1990 1995 2000 2005 2010 2015 2020 2025 2030
DHS
MICS
Other international survey
National survey
PMA
Subpopulation
+: Higher contraceptive use
−: Lower contraceptive use
A: Other age group
F: Folk methods included
S−: Sterilization included
S+: Sterilization excluded
Unmarried women
Formerly married
Partnered women
CP (any) ≤ 1%

Lithuania (Northern Europe, SA Group 1) −−− Unmarried / Not In−Union

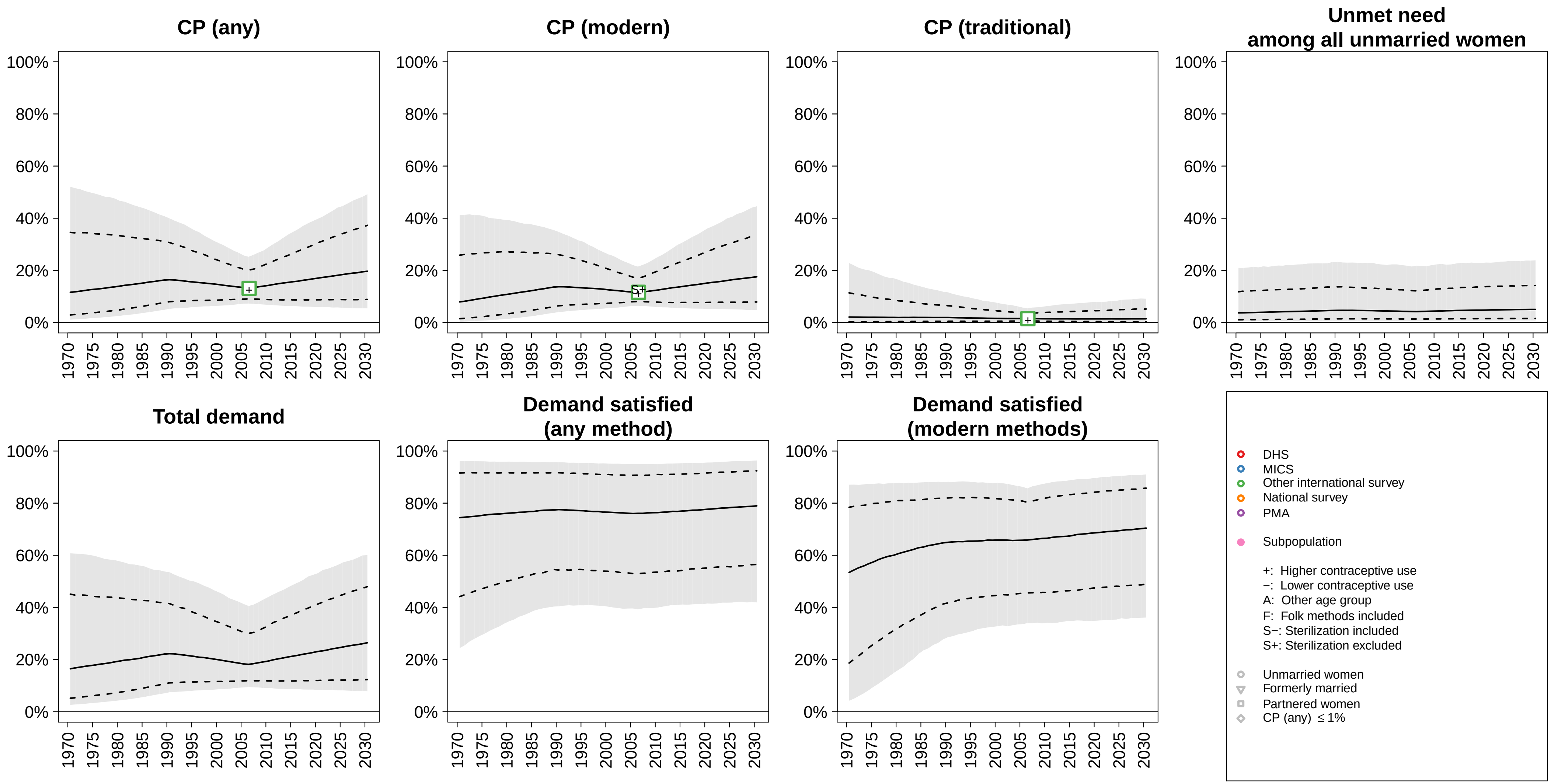

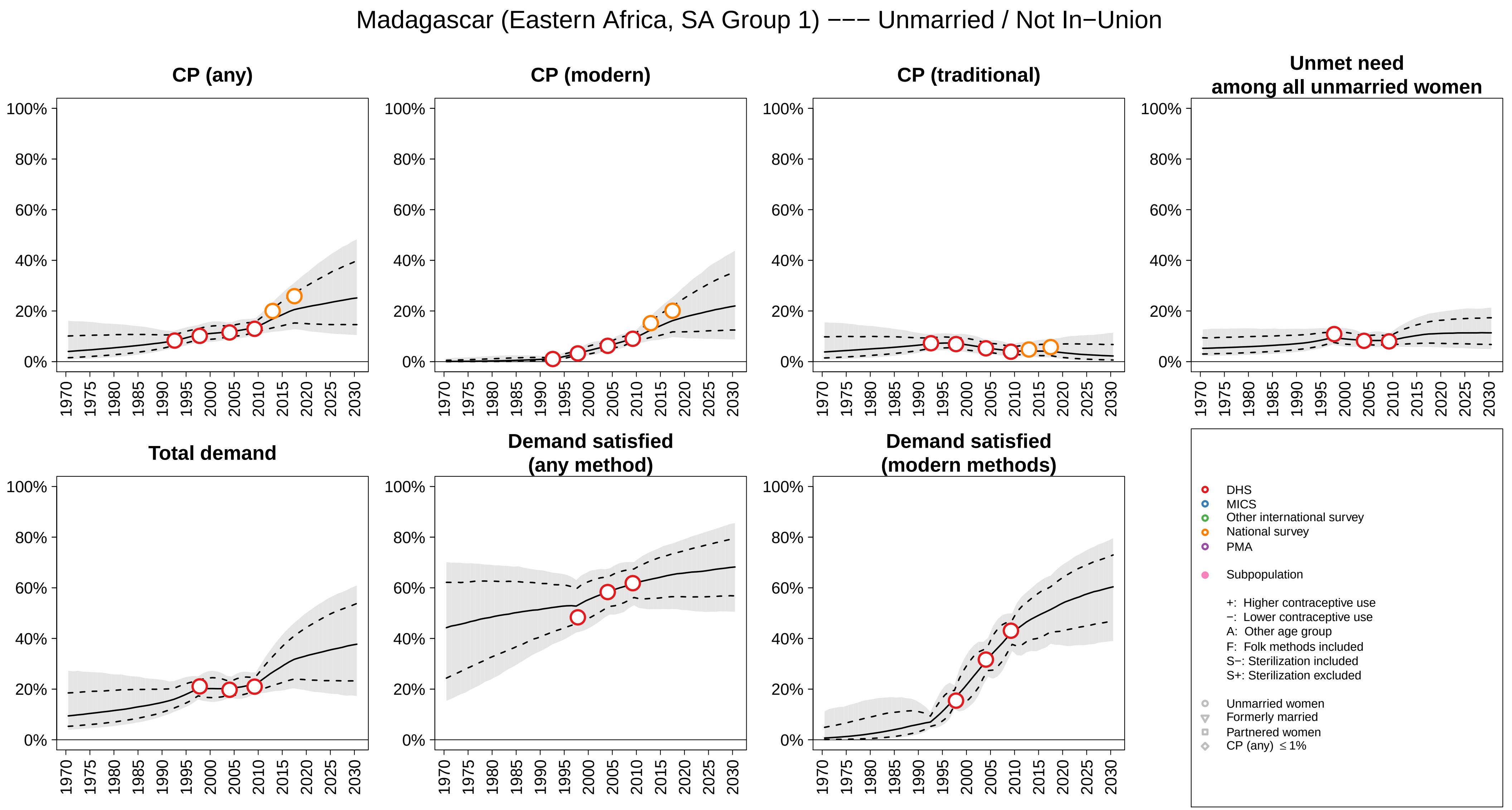
Madagascar (Eastern Africa, SA Group 1) −−− Unmarried / Not In−Union
CP (any)
CP (modern)
CP (traditional)
Unmet need among all unmarried women
Total demand
Demand satisfied (any method)
Demand satisfied (modern methods)
0%
20%
40%
60%
80%
100%
1970
1975
1980
1985
1990
1995
2000
2005
2010
2015
2020
2025
2030
DHS
MICS
Other international survey
National survey
PMA
Subpopulation
+: Higher contraceptive use
−: Lower contraceptive use
A: Other age group
F: Folk methods included
S−: Sterilization included
S+: Sterilization excluded
Unmarried women
Formerly married
Partnered women
CP (any) ≤ 1%

Malawi (Eastern Africa, SA Group 1) −−− Unmarried / Not In−Union

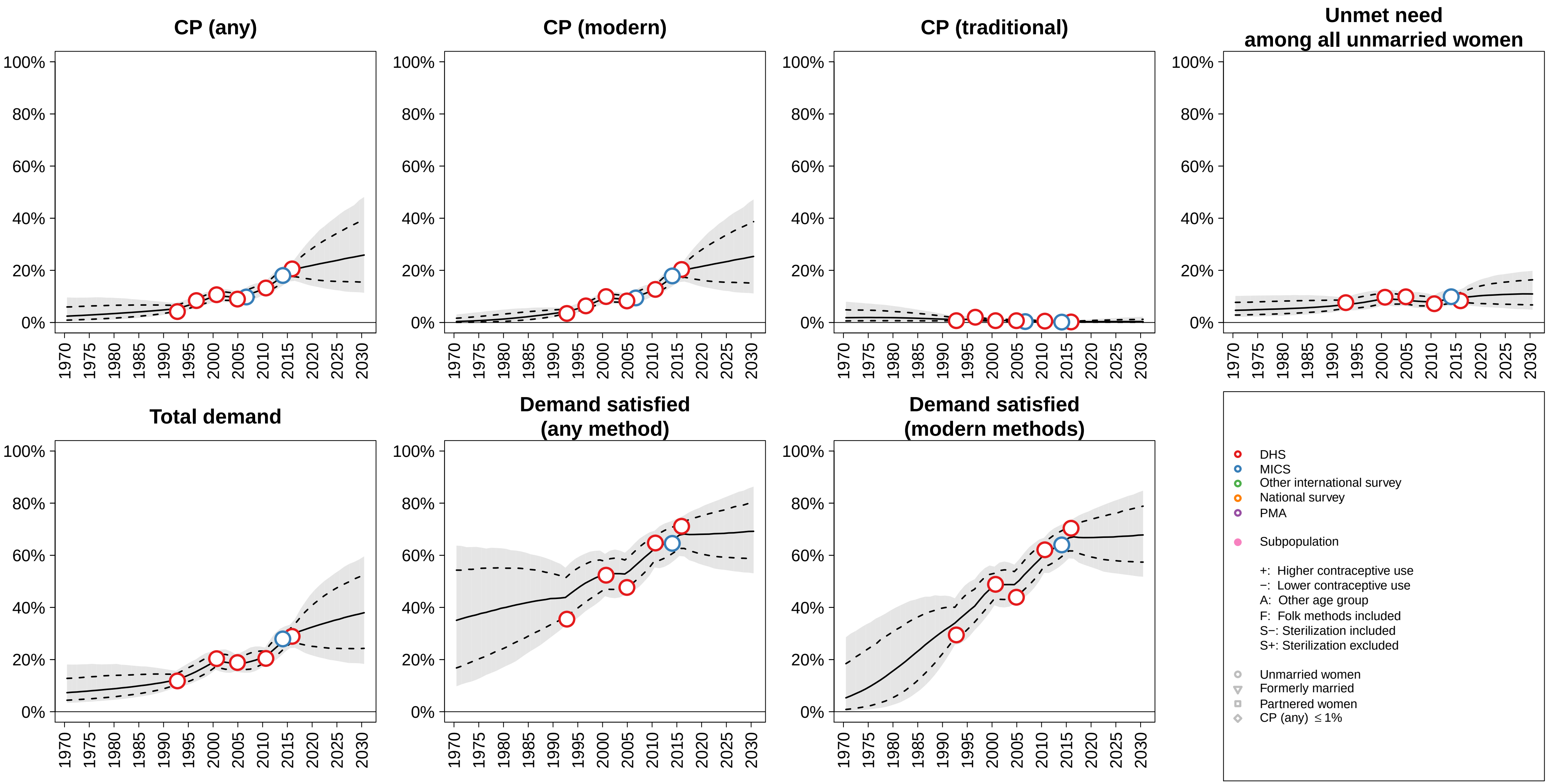

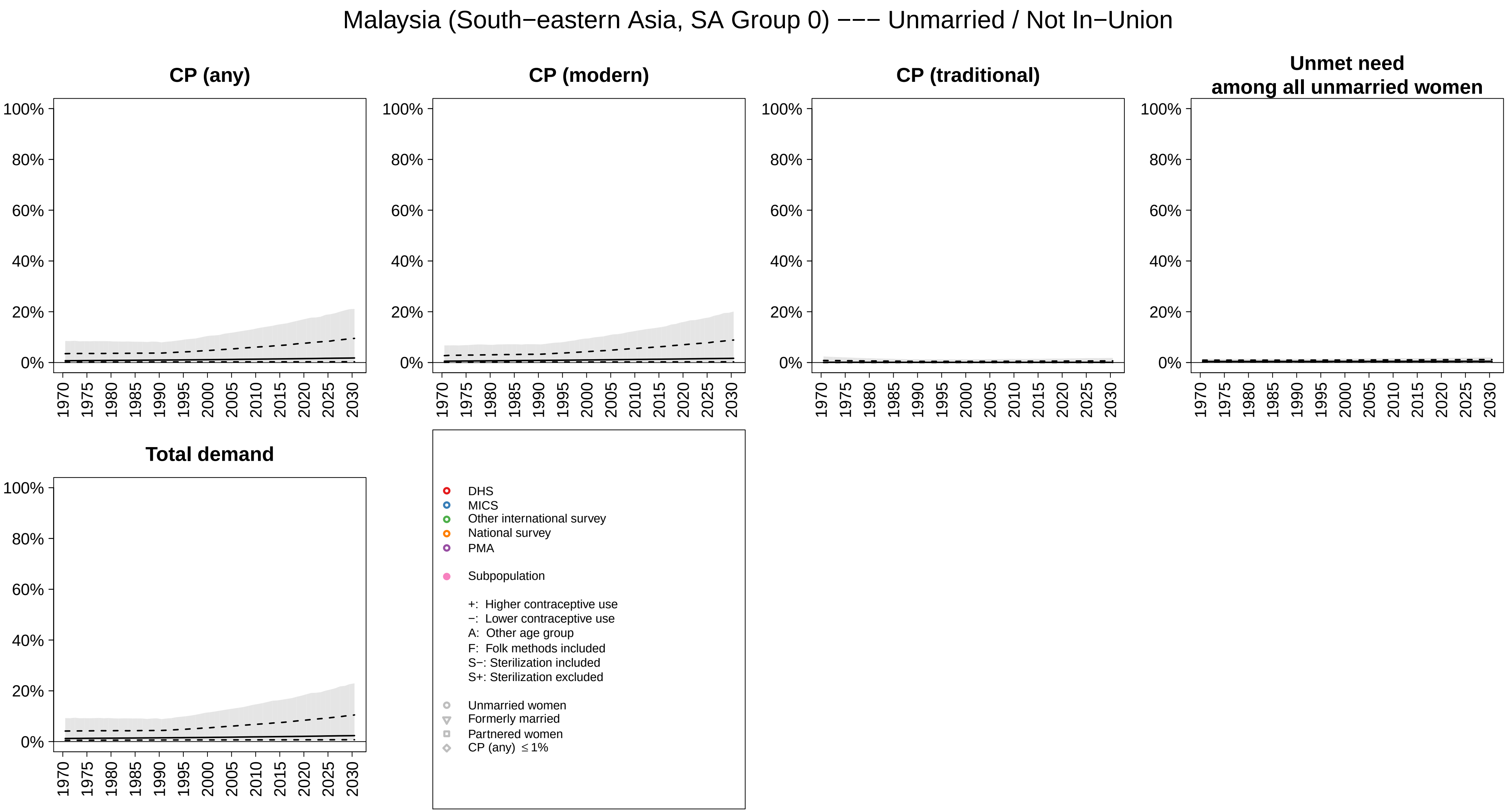
Malaysia (South−eastern Asia, SA Group 0) −−− Unmarried / Not In−Union
CP (any)
CP (modern)
CP (traditional)
Unmet need among all unmarried women
Total demand
100%
80%
60%
40%
20%
0%
1970 1975 1980 1985 1990 1995 2000 2005 2010 2015 2020 2025 2030
DHS
MICS
Other international survey
National survey
PMA
Subpopulation
+: Higher contraceptive use
−: Lower contraceptive use
A: Other age group
F: Folk methods included
S−: Sterilization included
S+: Sterilization excluded
Unmarried women
Formerly married
Partnered women
CP (any) ≤ 1%

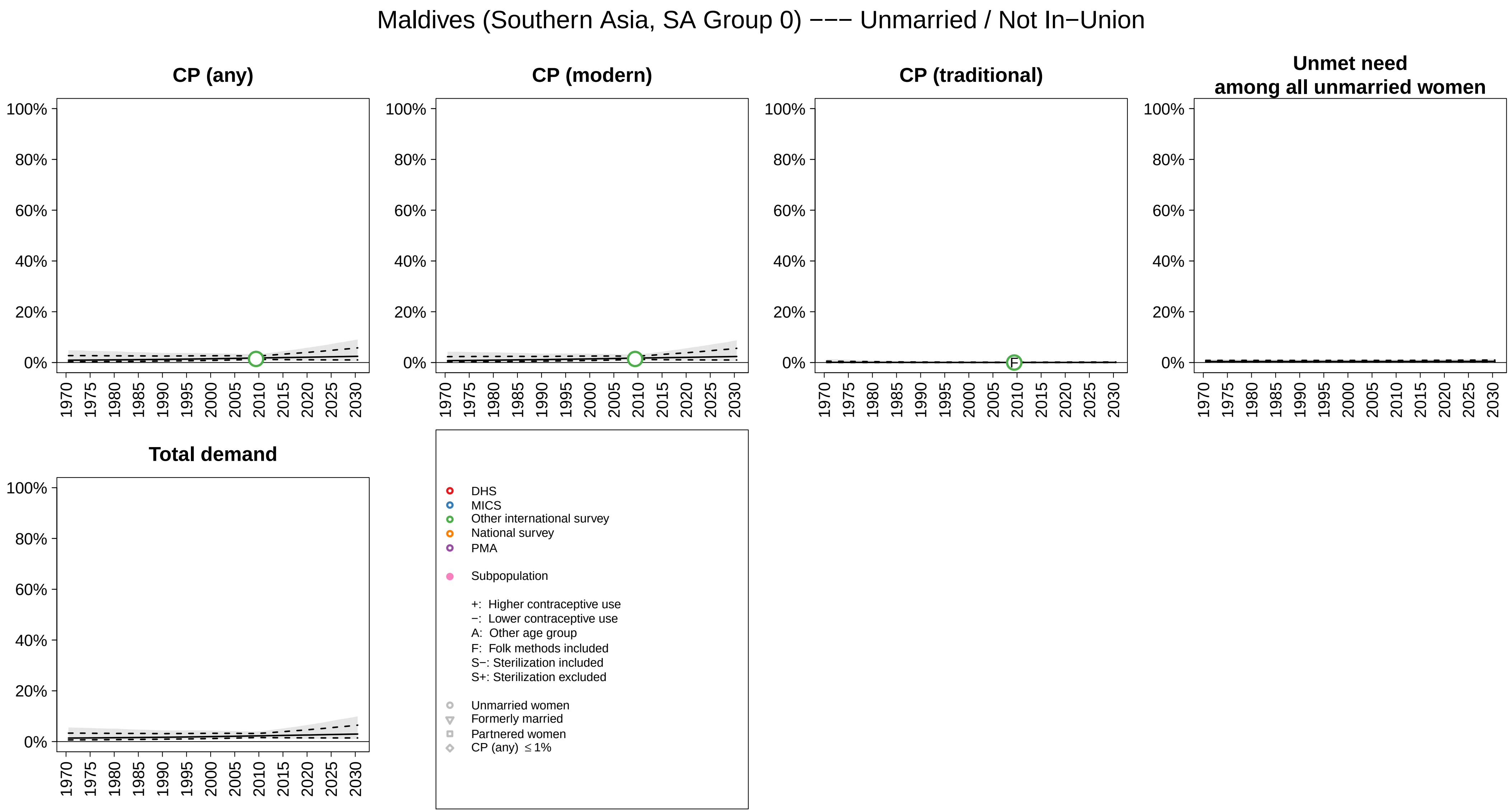
Maldives (Southern Asia, SA Group 0) −−− Unmarried / Not In−Union
CP (any)
CP (modern)
CP (traditional)
Unmet need among all unmarried women
Total demand
100%
80%
60%
40%
20%
0%
1970 1975 1980 1985 1990 1995 2000 2005 2010 2015 2020 2025 2030
F
DHS
MICS
Other international survey
National survey
PMA
Subpopulation
+: Higher contraceptive use
−: Lower contraceptive use
A: Other age group
F: Folk methods included
S−: Sterilization included
S+: Sterilization excluded
Unmarried women
Formerly married
Partnered women
CP (any) ≤ 1%

Mali (Western Africa, SA Group 1) −−− Unmarried / Not In−Union

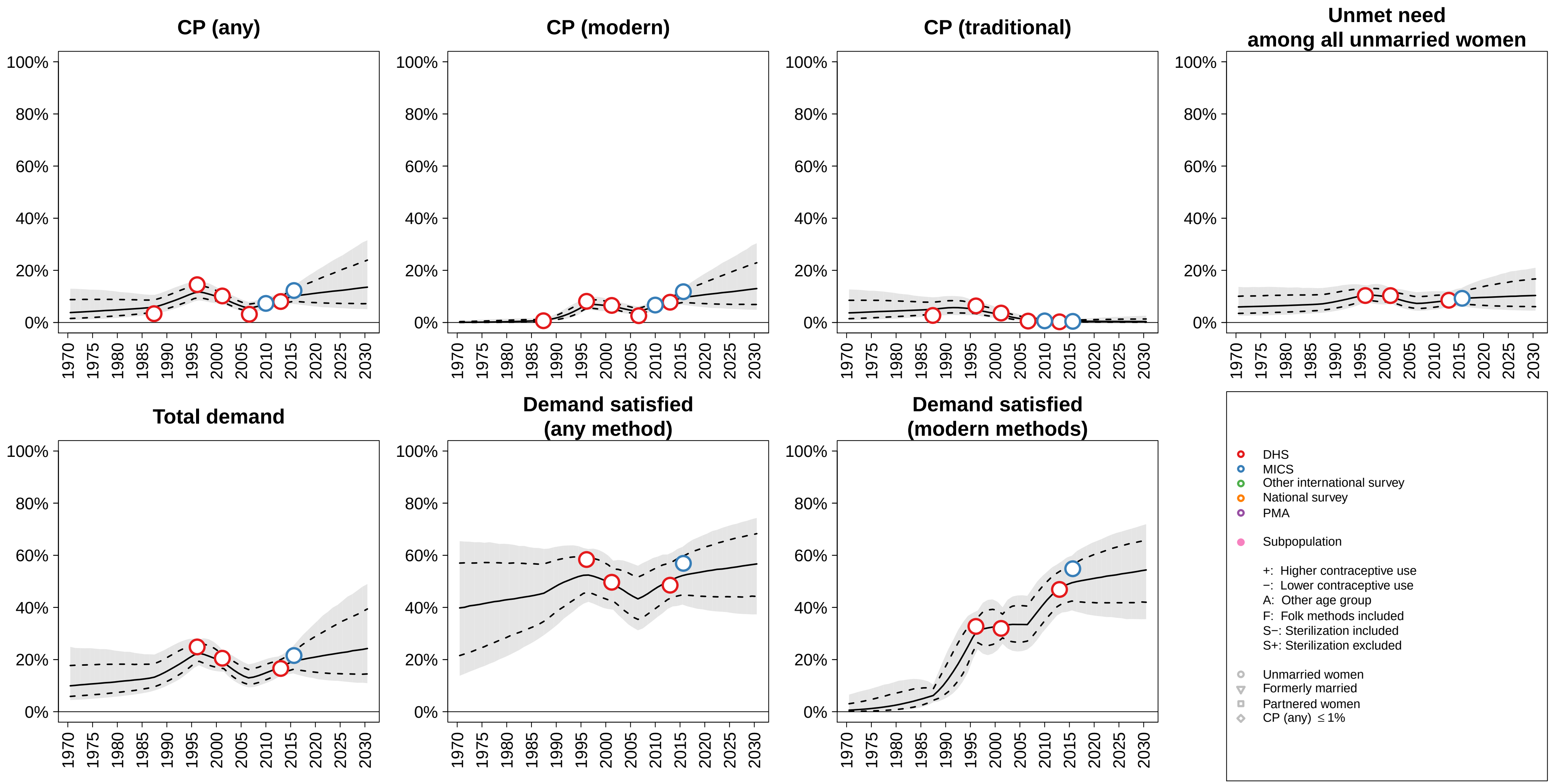

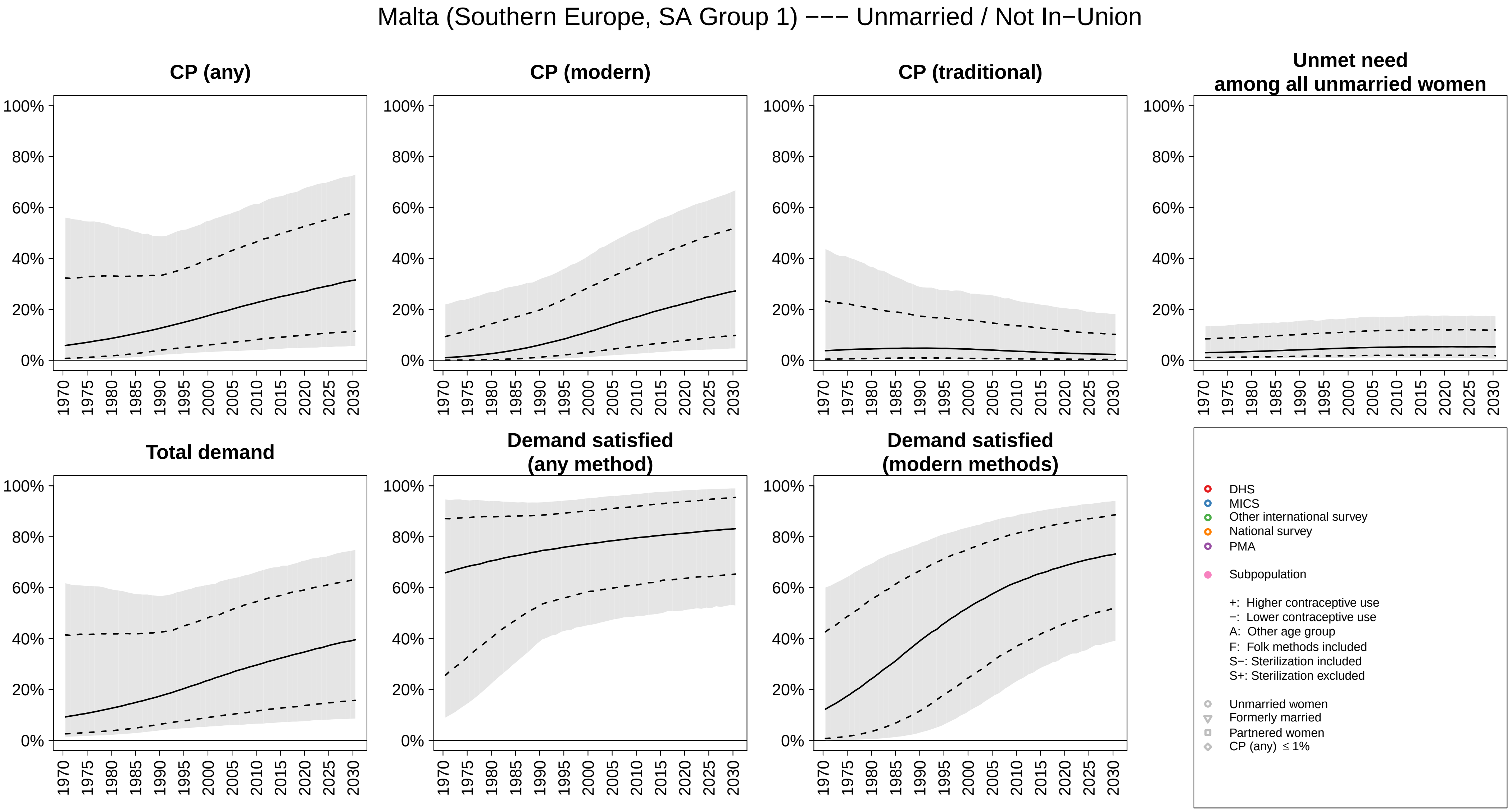
Malta (Southern Europe, SA Group 1) −−− Unmarried / Not In−Union
CP (any)
CP (modern)
CP (traditional)
Unmet need
among all unmarried women
Total demand
Demand satisfied
(any method)
Demand satisfied
(modern methods)
0%
20%
40%
60%
80%
100%
1970
1975
1980
1985
1990
1995
2000
2005
2010
2015
2020
2025
2030
DHS
MICS
Other international survey
National survey
PMA
Subpopulation
+: Higher contraceptive use
−: Lower contraceptive use
A: Other age group
F: Folk methods included
S−: Sterilization included
S+: Sterilization excluded
Unmarried women
Formerly married
Partnered women
CP (any) ≤ 1%

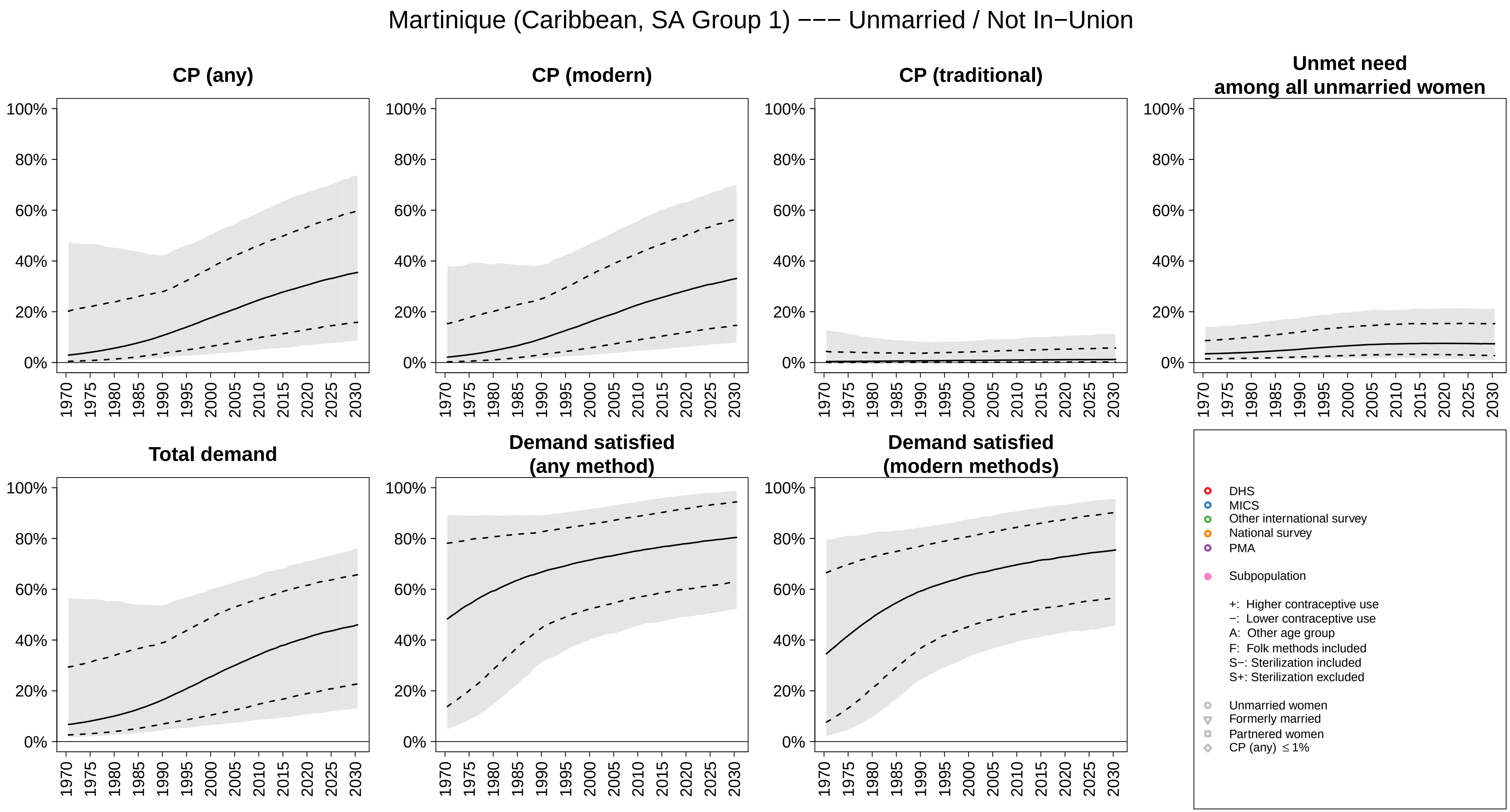
Martinique (Caribbean, SA Group 1) −−− Unmarried / Not In−Union
CP (any)
CP (modern)
CP (traditional)
Unmet need among all unmarried women
Total demand
Demand satisfied (any method)
Demand satisfied (modern methods)
0%
20%
40%
60%
80%
100%
1970
1975
1980
1985
1990
1995
2000
2005
2010
2015
2020
2025
2030
DHS
MICS
Other international survey
National survey
PMA
Subpopulation
+: Higher contraceptive use
−: Lower contraceptive use
A: Other age group
F: Folk methods included
S−: Sterilization included
S+: Sterilization excluded
Unmarried women
Formerly married
Partnered women
CP (any) ≤ 1%

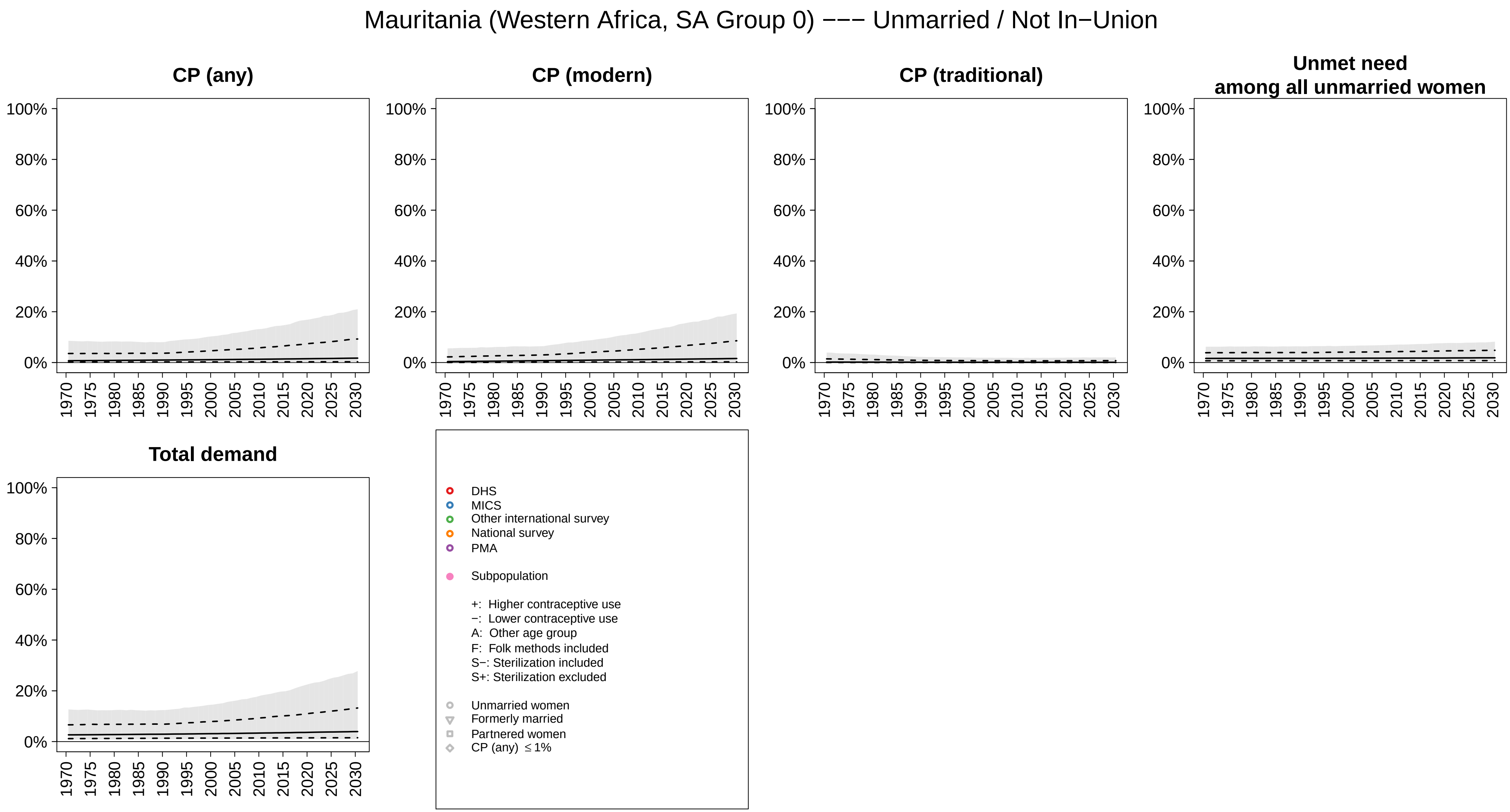
Mauritania (Western Africa, SA Group 0) −−− Unmarried / Not In−Union
CP (any)
CP (modern)
CP (traditional)
Unmet need among all unmarried women
Total demand
100%
80%
60%
40%
20%
0%
1970 1975 1980 1985 1990 1995 2000 2005 2010 2015 2020 2025 2030
DHS
MICS
Other international survey
National survey
PMA
Subpopulation
+: Higher contraceptive use
−: Lower contraceptive use
A: Other age group
F: Folk methods included
S−: Sterilization included
S+: Sterilization excluded
Unmarried women
Formerly married
Partnered women
CP (any) ≤ 1%

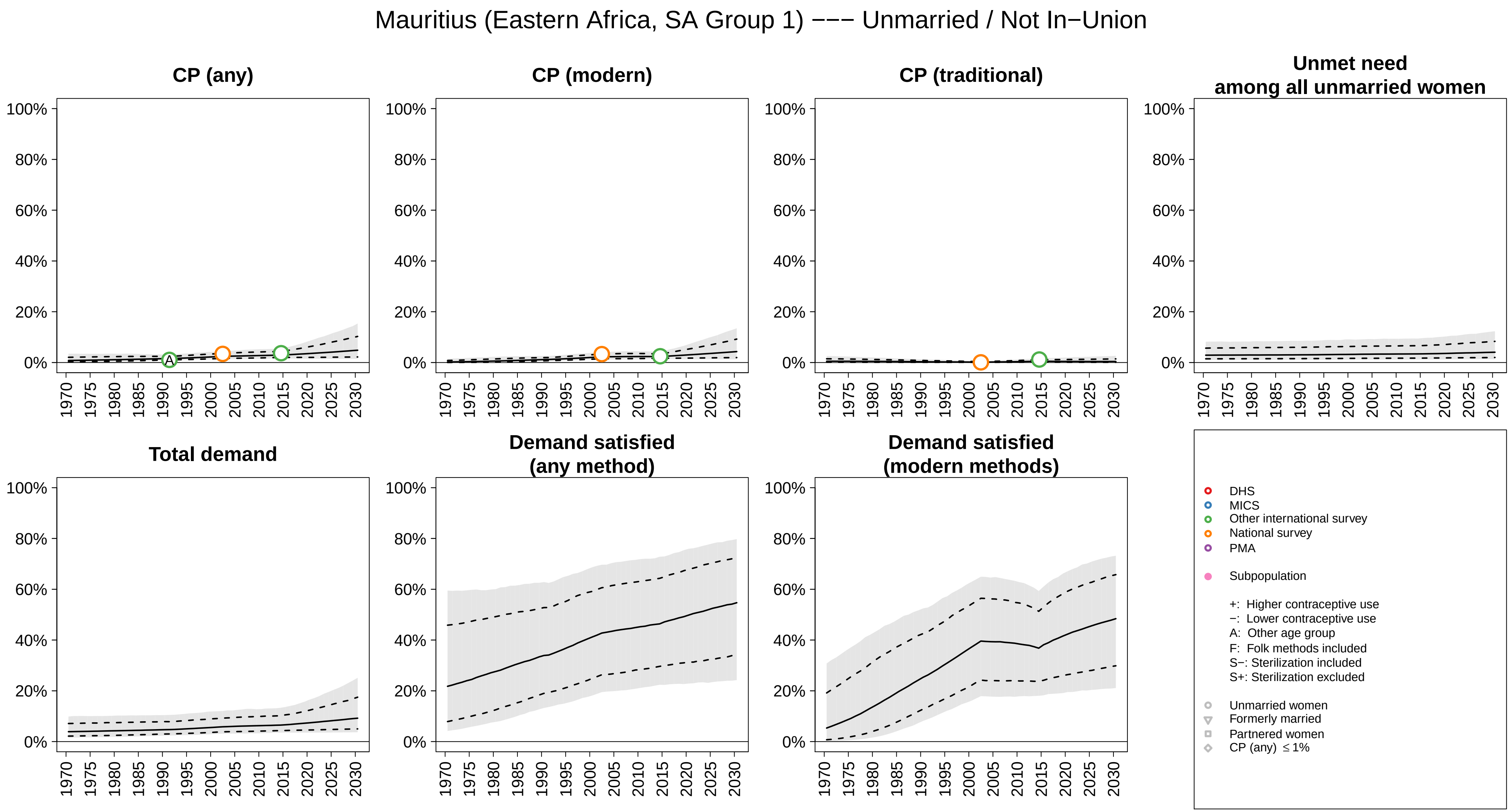
Mauritius (Eastern Africa, SA Group 1) −−− Unmarried / Not In−Union
CP (any)
CP (modern)
CP (traditional)
Unmet need among all unmarried women
Total demand
Demand satisfied (any method)
Demand satisfied (modern methods)
100%
80%
60%
40%
20%
0%
1970 1975 1980 1985 1990 1995 2000 2005 2010 2015 2020 2025 2030
DHS
MICS
Other international survey
National survey
PMA
Subpopulation
+: Higher contraceptive use
−: Lower contraceptive use
A: Other age group
F: Folk methods included
S−: Sterilization included
S+: Sterilization excluded
Unmarried women
Formerly married
Partnered women
CP (any) ≤ 1%

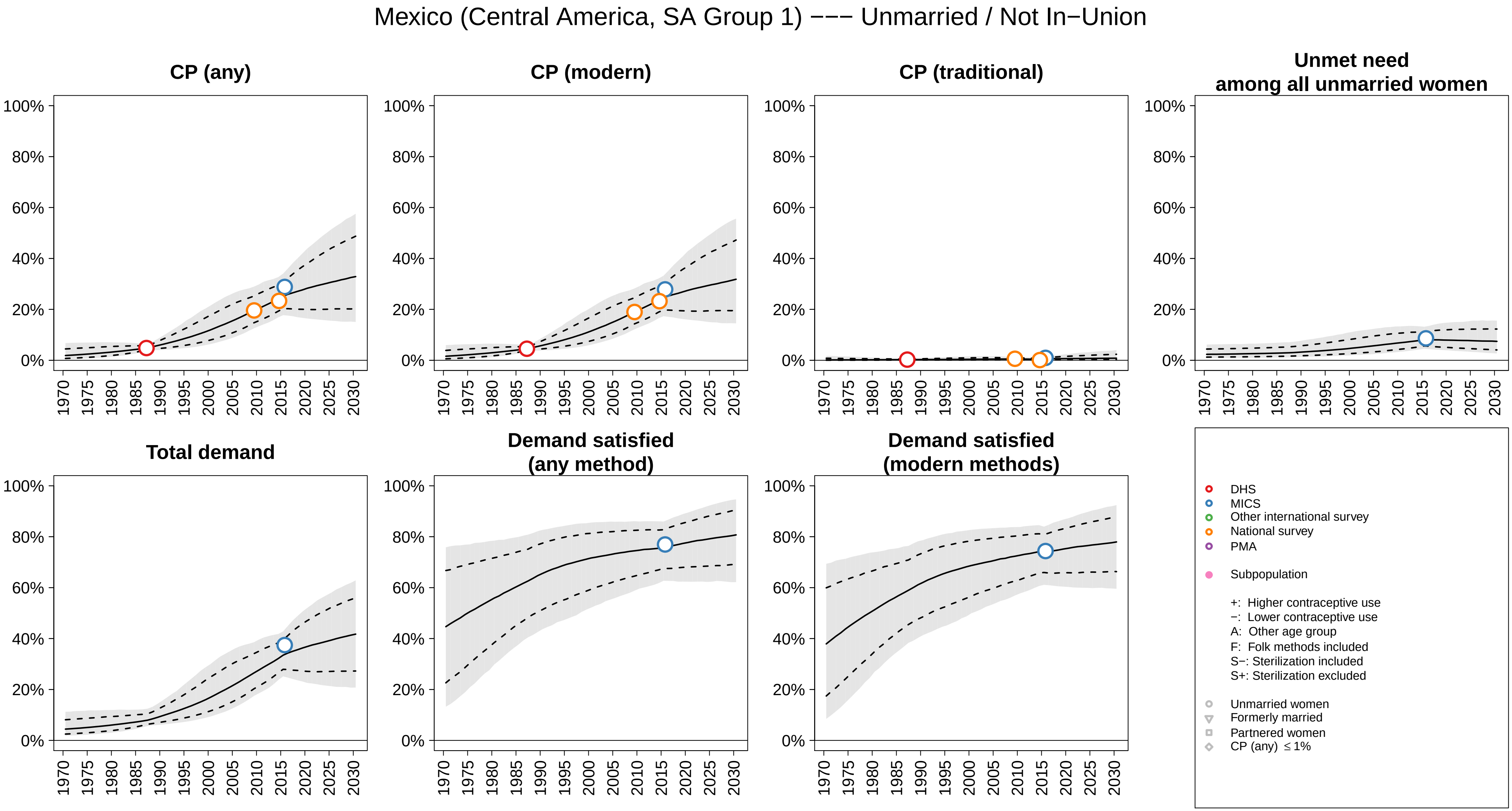
Mexico (Central America, SA Group 1) −−− Unmarried / Not In−Union
CP (any)
CP (modern)
CP (traditional)
Unmet need among all unmarried women
Total demand
Demand satisfied (any method)
Demand satisfied (modern methods)
100%
80%
60%
40%
20%
0%
1970
1975
1980
1985
1990
1995
2000
2005
2010
2015
2020
2025
2030
DHS
MICS
Other international survey
National survey
PMA
Subpopulation
+: Higher contraceptive use
−: Lower contraceptive use
A: Other age group
F: Folk methods included
S−: Sterilization included
S+: Sterilization excluded
Unmarried women
Formerly married
Partnered women
CP (any) ≤ 1%

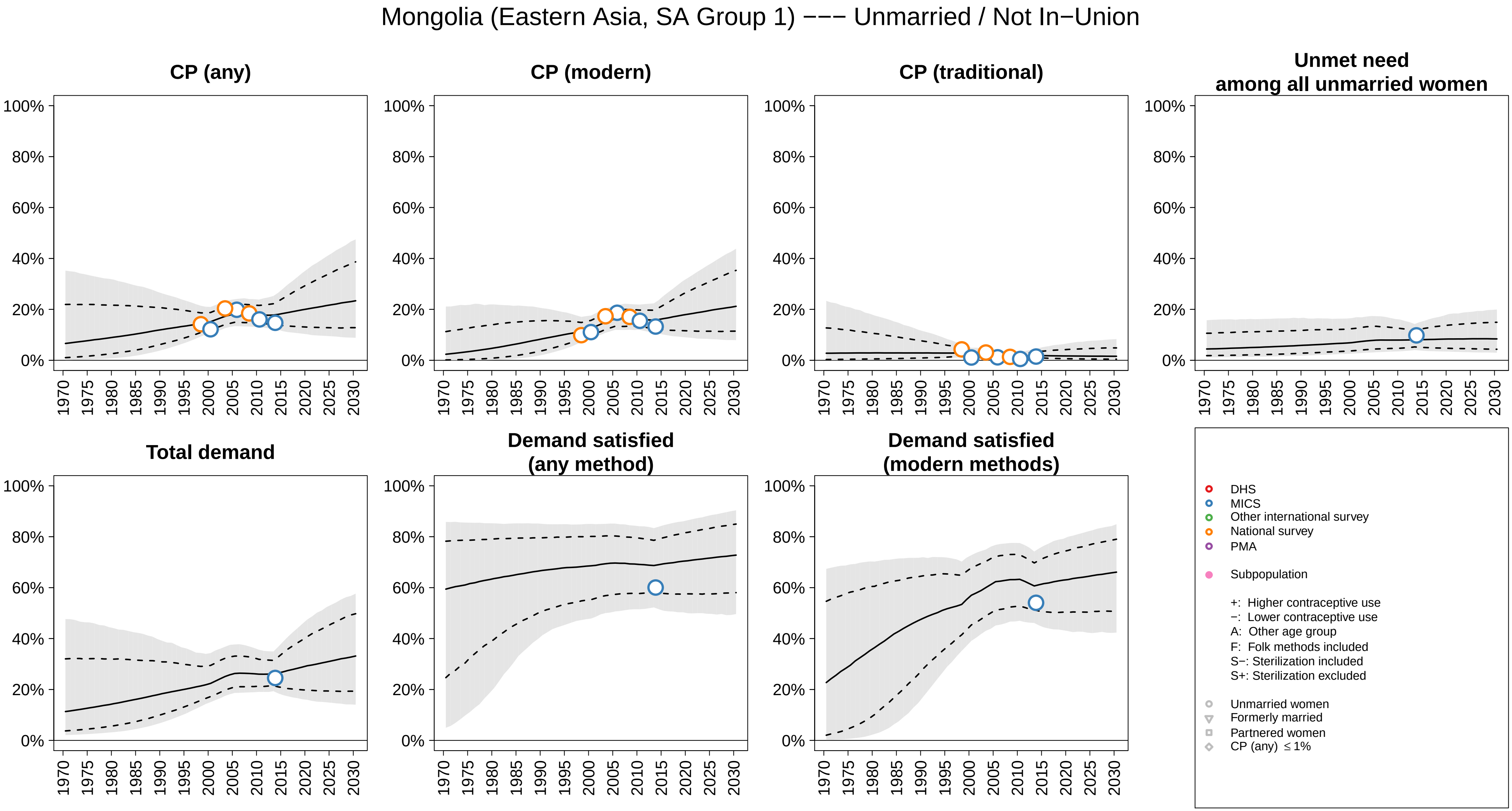
Mongolia (Eastern Asia, SA Group 1) −−− Unmarried / Not In−Union
CP (any)
CP (modern)
CP (traditional)
Unmet need among all unmarried women
Total demand
Demand satisfied (any method)
Demand satisfied (modern methods)
100%
80%
60%
40%
20%
0%
1970 1975 1980 1985 1990 1995 2000 2005 2010 2015 2020 2025 2030
DHS
MICS
Other international survey
National survey
PMA
Subpopulation
+: Higher contraceptive use
−: Lower contraceptive use
A: Other age group
F: Folk methods included
S−: Sterilization included
S+: Sterilization excluded
Unmarried women
Formerly married
Partnered women
CP (any) ≤ 1%

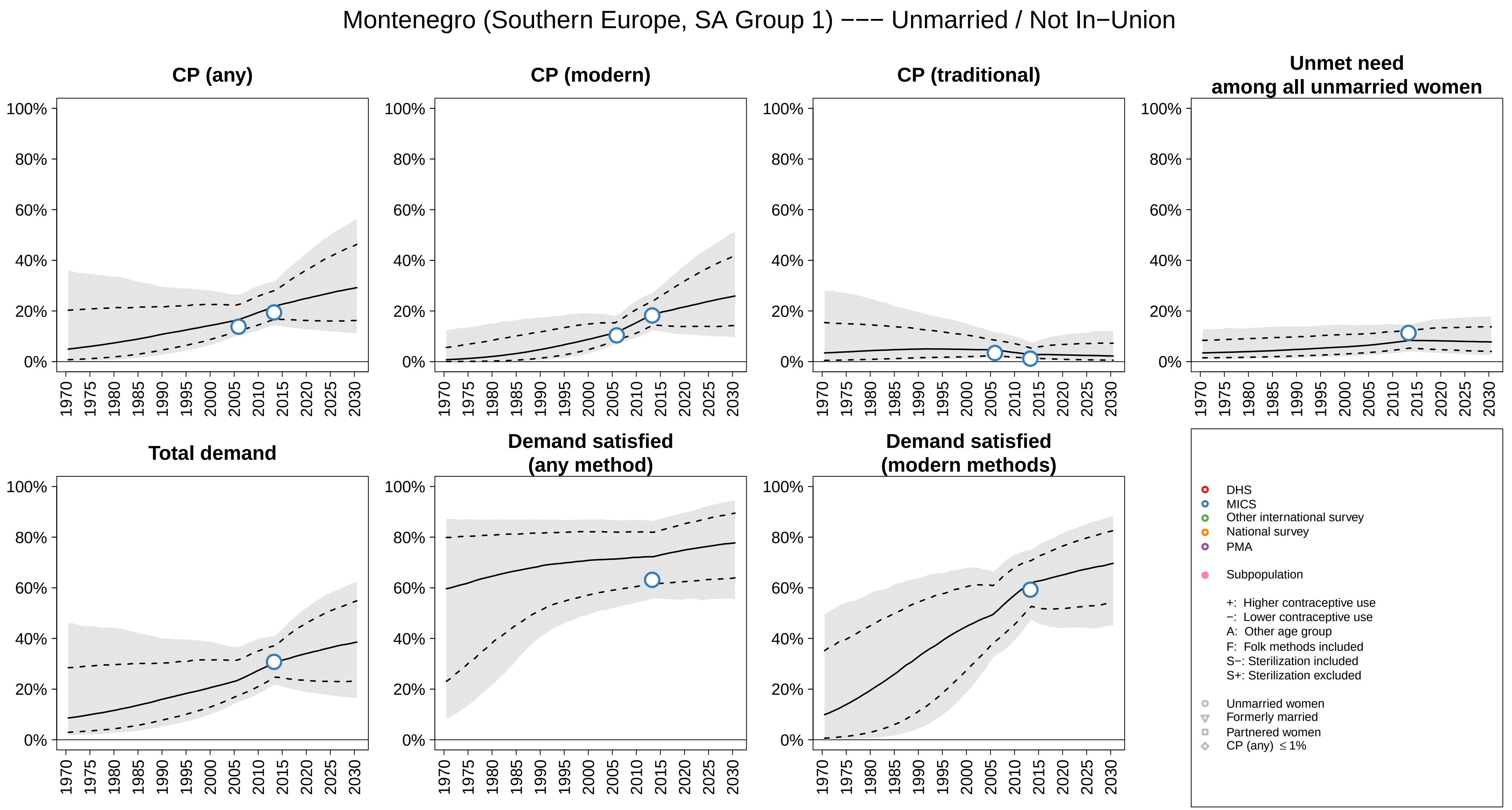

Montenegro (Southern Europe, SA Group 1) −−− Unmarried / Not In−Union
CP (any)
CP (modern)
CP (traditional)
Unmet need among all unmarried women
Total demand
Demand satisfied (any method)
Demand satisfied (modern methods)
0%
20%
40%
60%
80%
100%
1970
1975
1980
1985
1990
1995
2000
2005
2010
2015
2020
2025
2030
DHS
MICS
Other international survey
National survey
PMA
Subpopulation
+: Higher contraceptive use
−: Lower contraceptive use
A: Other age group
F: Folk methods included
S−: Sterilization included
S+: Sterilization excluded
Unmarried women
Formerly married
Partnered women
CP (any) ≤ 1%

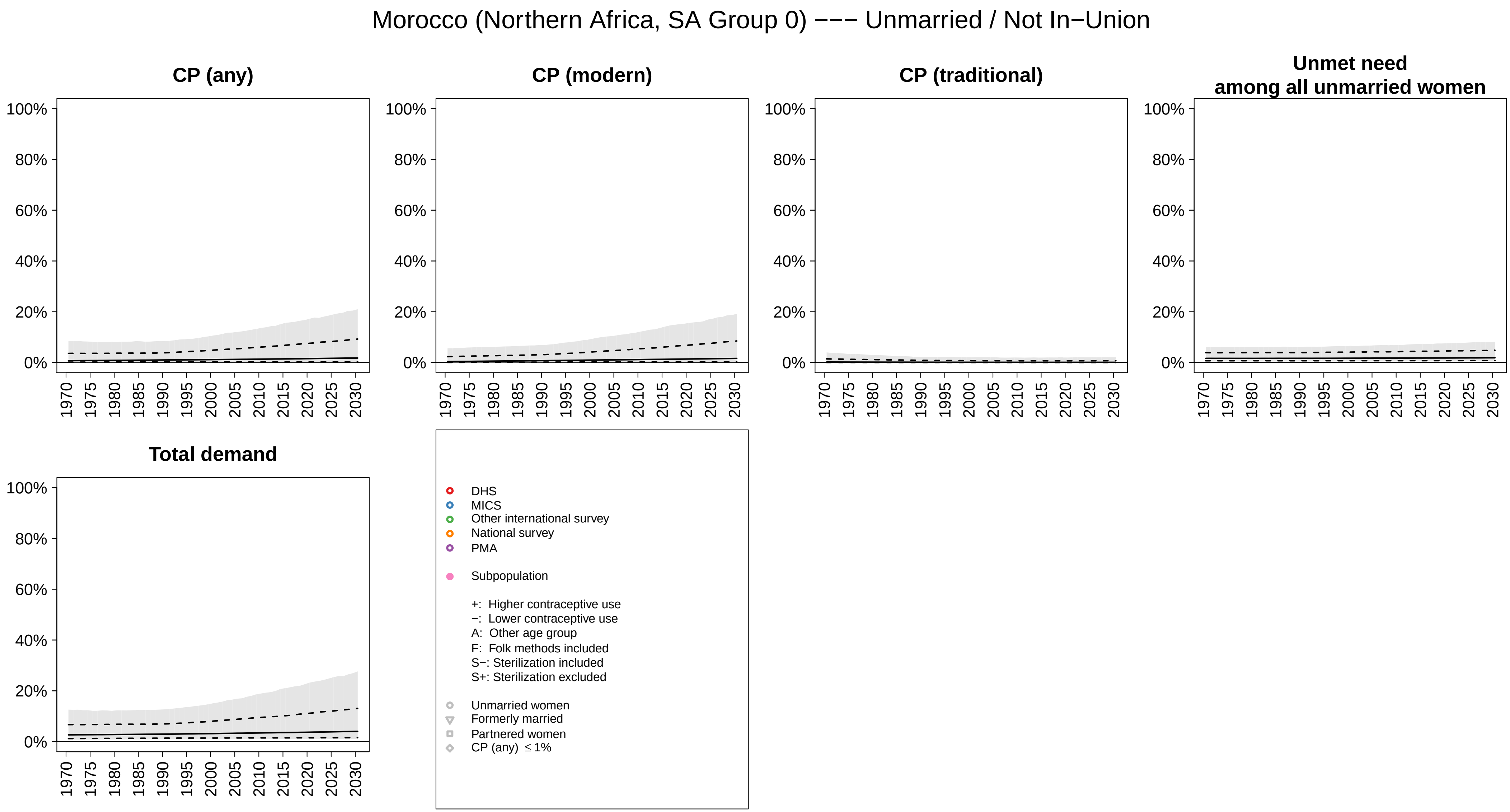
Morocco (Northern Africa, SA Group 0) −−− Unmarried / Not In−Union
CP (any)
CP (modern)
CP (traditional)
Unmet need among all unmarried women
Total demand
100%
80%
60%
40%
20%
0%
1970 1975 1980 1985 1990 1995 2000 2005 2010 2015 2020 2025 2030
DHS
MICS
Other international survey
National survey
PMA
Subpopulation
+: Higher contraceptive use
−: Lower contraceptive use
A: Other age group
F: Folk methods included
S−: Sterilization included
S+: Sterilization excluded
Unmarried women
Formerly married
Partnered women
CP (any) ≤ 1%

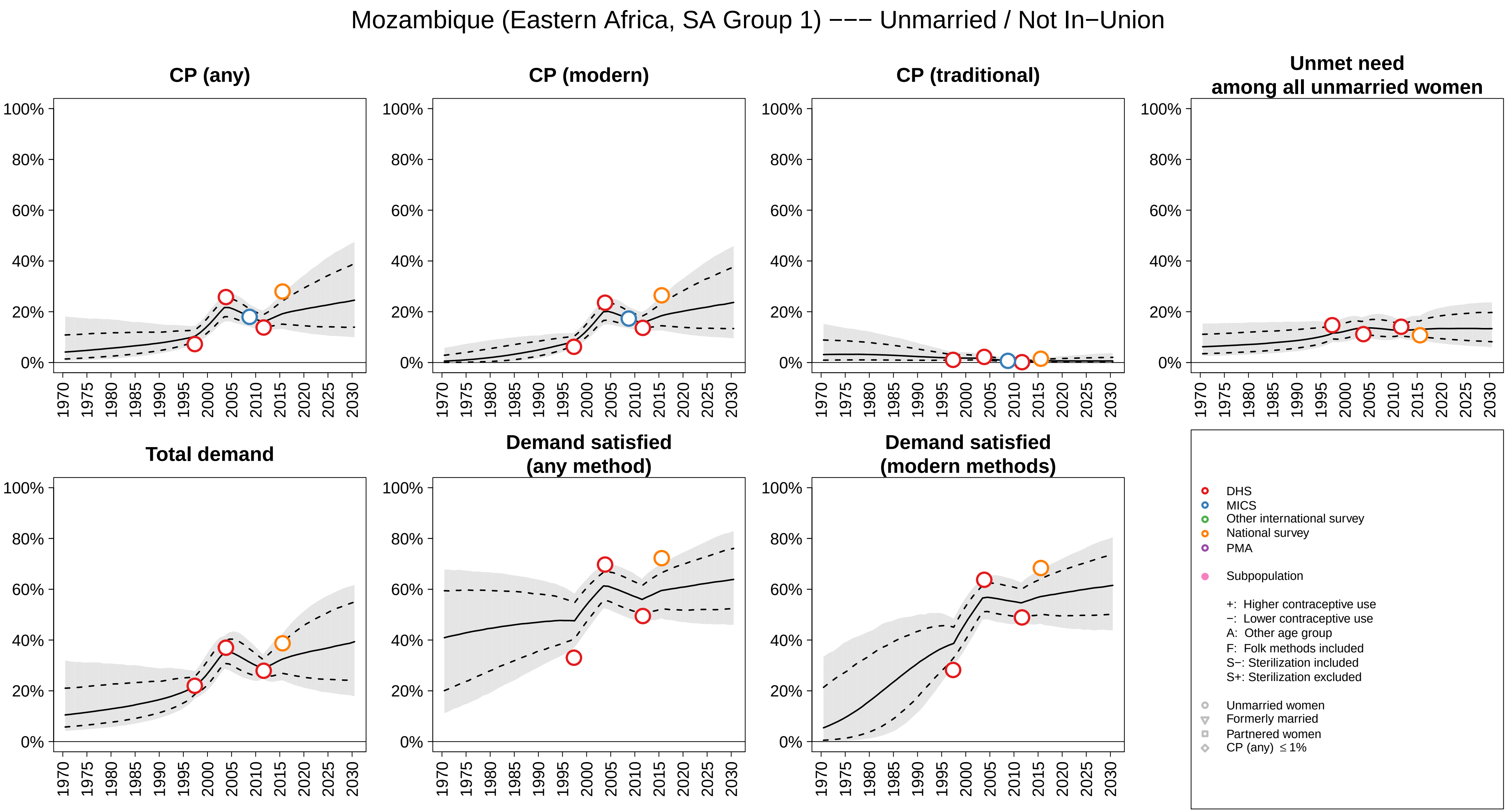
Mozambique (Eastern Africa, SA Group 1) --- Unmarried / Not In-Union
CP (any)
CP (modern)
CP (traditional)
Unmet need among all unmarried women
Total demand
Demand satisfied (any method)
Demand satisfied (modern methods)
0%
20%
40%
60%
80%
100%
1970
1975
1980
1985
1990
1995
2000
2005
2010
2015
2020
2025
2030
DHS
MICS
Other international survey
National survey
PMA
Subpopulation
+: Higher contraceptive use
−: Lower contraceptive use
A: Other age group
F: Folk methods included
S−: Sterilization included
S+: Sterilization excluded
Unmarried women
Formerly married
Partnered women
CP (any) ≤ 1%

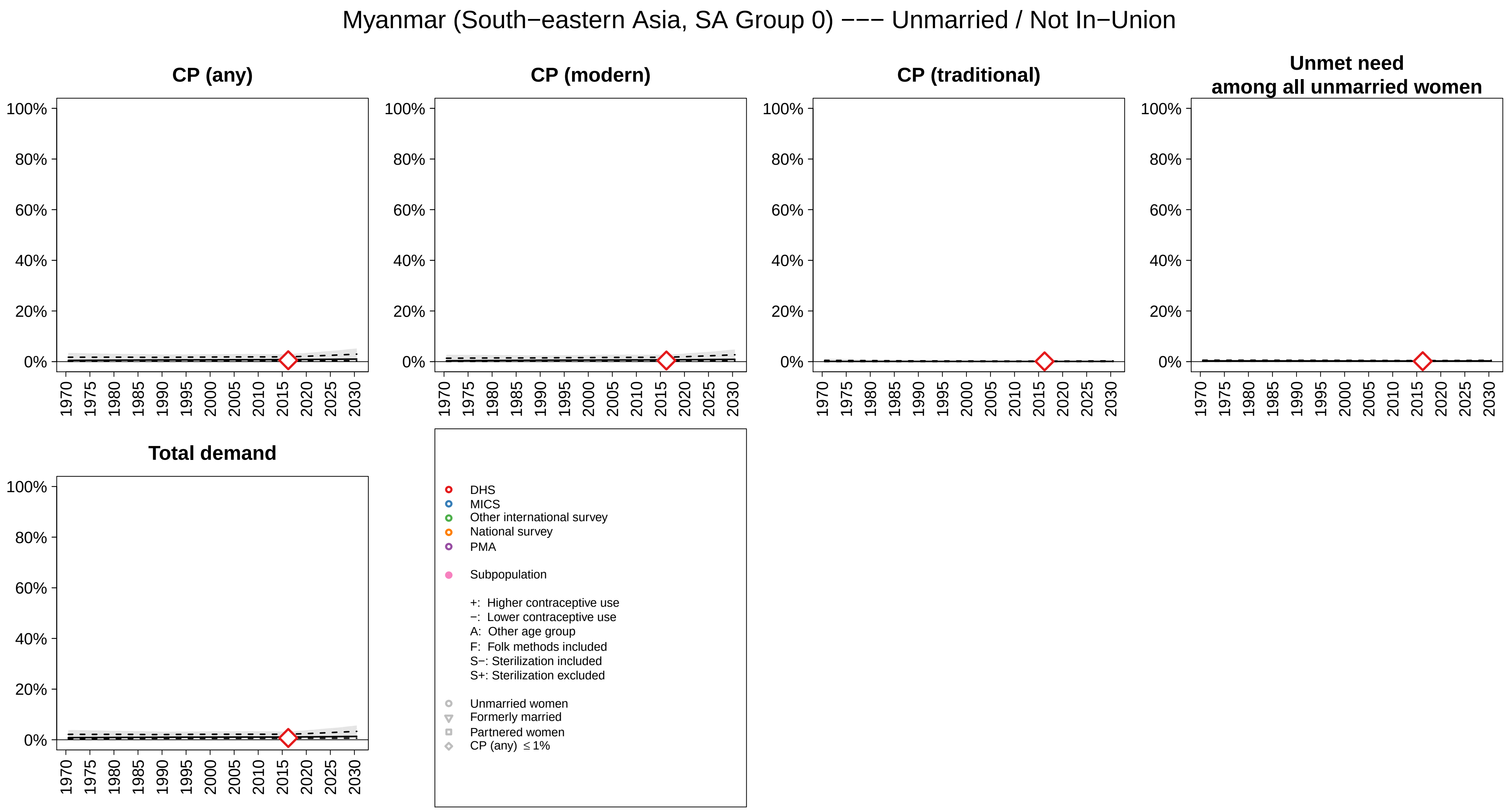
Myanmar (South−eastern Asia, SA Group 0) −−− Unmarried / Not In−Union
CP (any)
CP (modern)
CP (traditional)
Unmet need among all unmarried women
Total demand
100%
80%
60%
40%
20%
0%
1970 1975 1980 1985 1990 1995 2000 2005 2010 2015 2020 2025 2030
DHS
MICS
Other international survey
National survey
PMA
Subpopulation
+: Higher contraceptive use
−: Lower contraceptive use
A: Other age group
F: Folk methods included
S−: Sterilization included
S+: Sterilization excluded
Unmarried women
Formerly married
Partnered women
CP (any) ≤ 1%

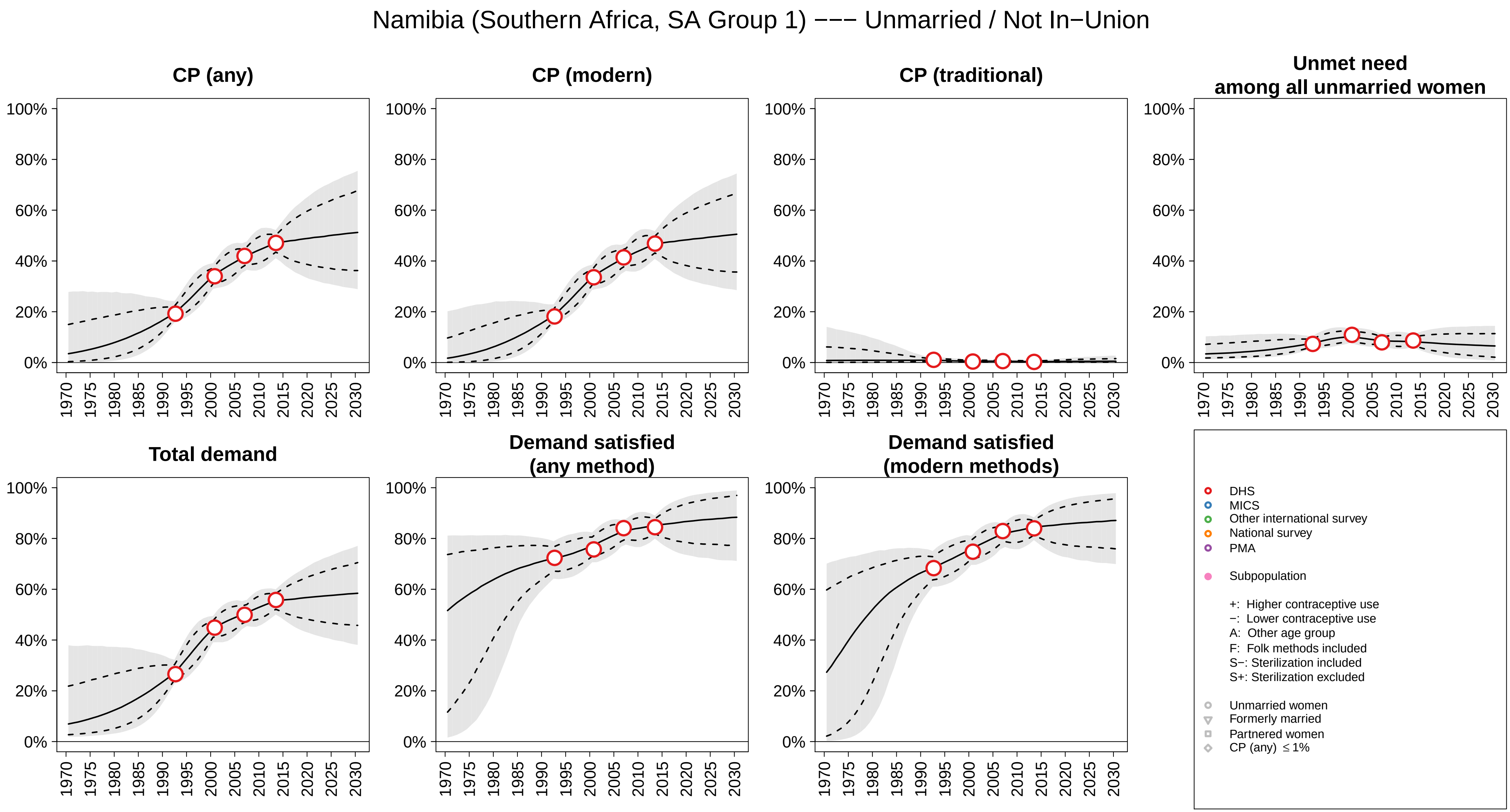
Namibia (Southern Africa, SA Group 1) −−− Unmarried / Not In−Union
CP (any)
CP (modern)
CP (traditional)
Unmet need among all unmarried women
Total demand
Demand satisfied (any method)
Demand satisfied (modern methods)
0%
20%
40%
60%
80%
100%
1970
1975
1980
1985
1990
1995
2000
2005
2010
2015
2020
2025
2030
DHS
MICS
Other international survey
National survey
PMA
Subpopulation
+: Higher contraceptive use
−: Lower contraceptive use
A: Other age group
F: Folk methods included
S−: Sterilization included
S+: Sterilization excluded
Unmarried women
Formerly married
Partnered women
CP (any) ≤ 1%

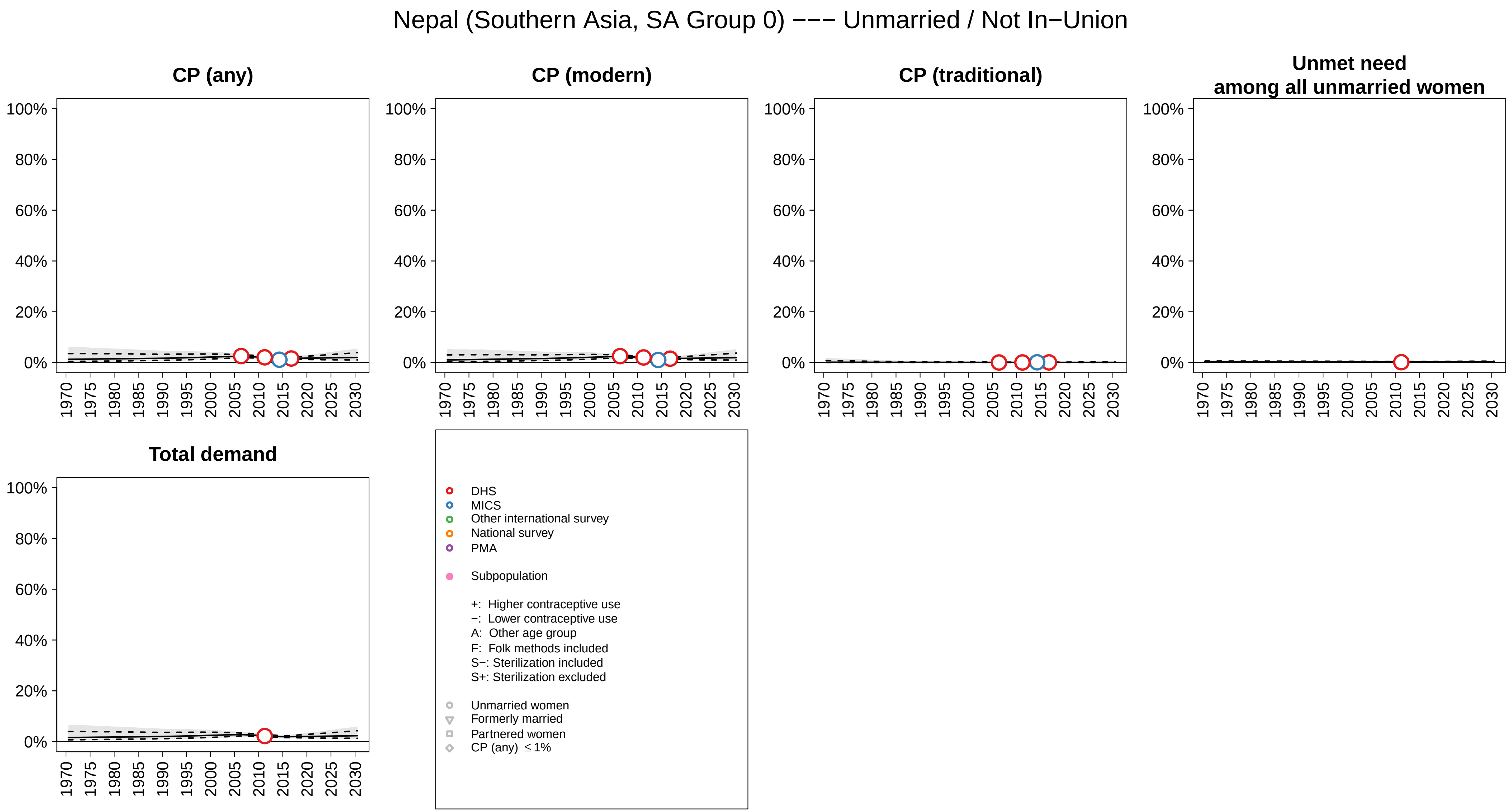
Nepal (Southern Asia, SA Group 0) −−− Unmarried / Not In−Union
CP (any)
CP (modern)
CP (traditional)
Unmet need
among all unmarried women
Total demand
100%
80%
60%
40%
20%
0%
1970
1975
1980
1985
1990
1995
2000
2005
2010
2015
2020
2025
2030
DHS
MICS
Other international survey
National survey
PMA
Subpopulation
+: Higher contraceptive use
−: Lower contraceptive use
A: Other age group
F: Folk methods included
S−: Sterilization included
S+: Sterilization excluded
Unmarried women
Formerly married
Partnered women
CP (any) ≤ 1%

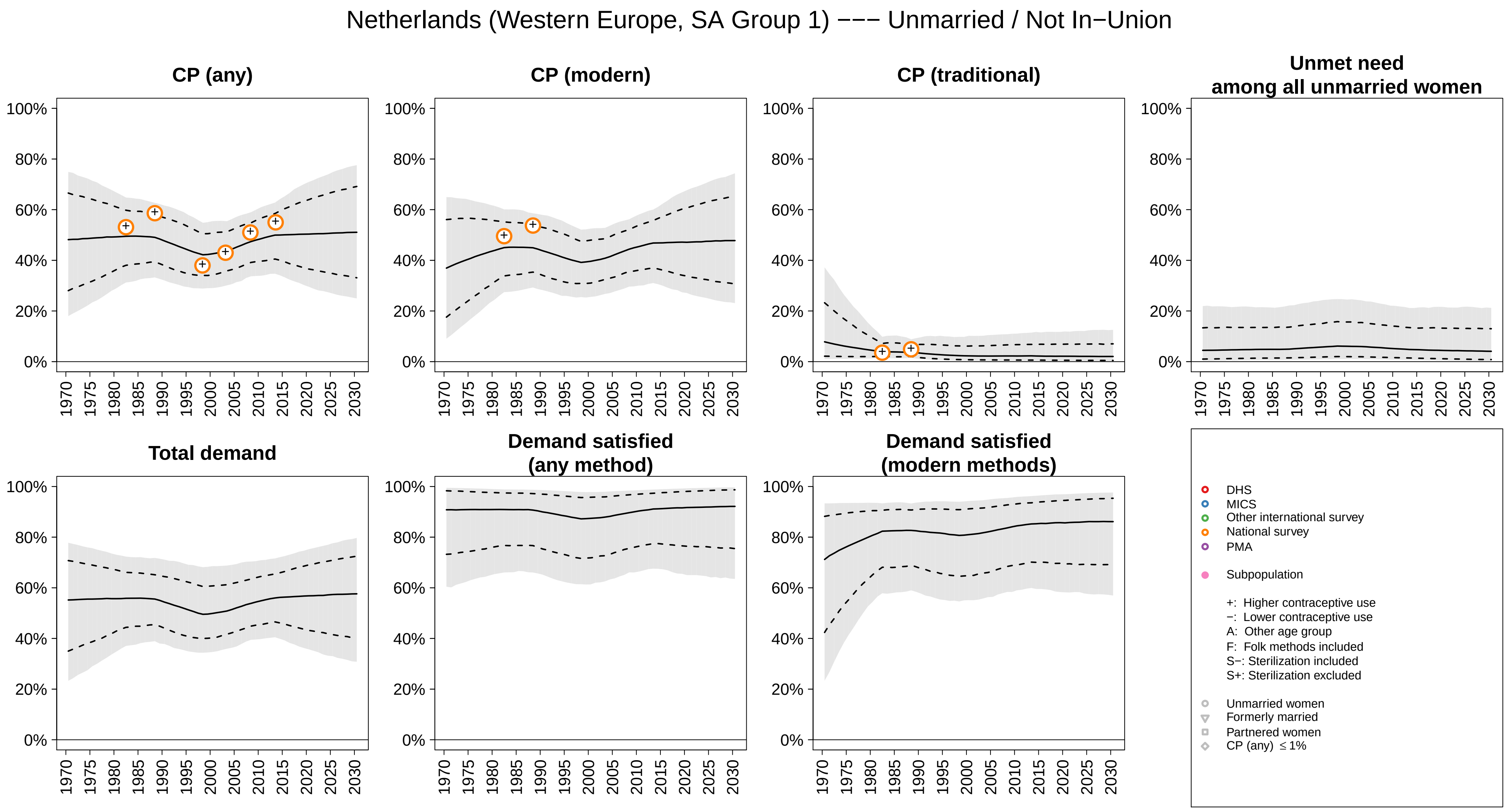

Netherlands (Western Europe, SA Group 1) −−− Unmarried / Not In−Union
CP (any)
CP (modern)
CP (traditional)
Unmet need among all unmarried women
Total demand
Demand satisfied (any method)
Demand satisfied (modern methods)
0%
20%
40%
60%
80%
100%
1970
1975
1980
1985
1990
1995
2000
2005
2010
2015
2020
2025
2030
DHS
MICS
Other international survey
National survey
PMA
Subpopulation
+: Higher contraceptive use
−: Lower contraceptive use
A: Other age group
F: Folk methods included
S−: Sterilization included
S+: Sterilization excluded
Unmarried women
Formerly married
Partnered women
CP (any) ≤ 1%

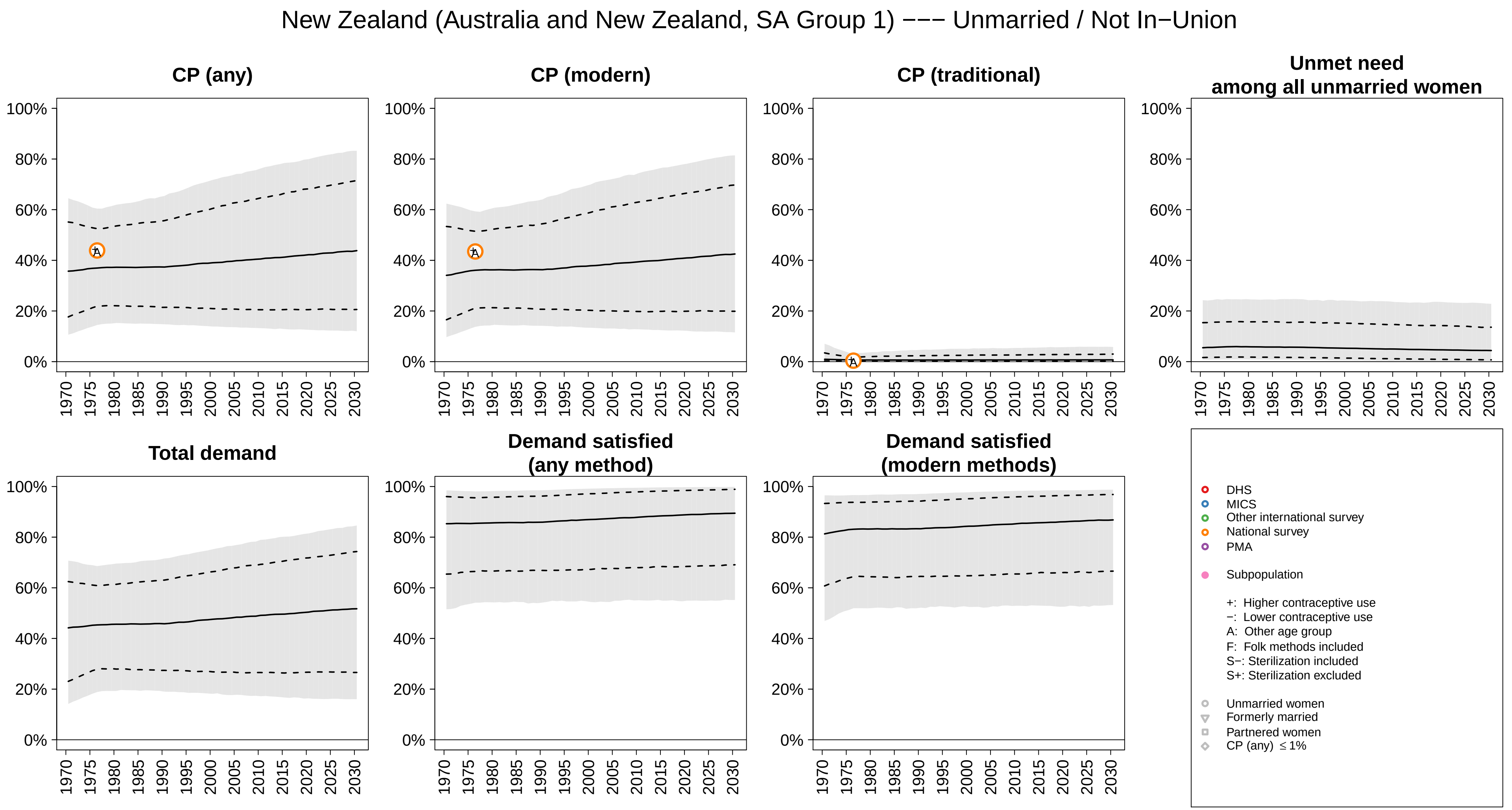
New Zealand (Australia and New Zealand, SA Group 1) −−− Unmarried / Not In−Union
CP (any)
CP (modern)
CP (traditional)
Unmet need among all unmarried women
Total demand
Demand satisfied (any method)
Demand satisfied (modern methods)
100%
80%
60%
40%
20%
0%
1970
1975
1980
1985
1990
1995
2000
2005
2010
2015
2020
2025
2030
DHS
MICS
Other international survey
National survey
PMA
Subpopulation
+: Higher contraceptive use
−: Lower contraceptive use
A: Other age group
F: Folk methods included
S−: Sterilization included
S+: Sterilization excluded
Unmarried women
Formerly married
Partnered women
CP (any) ≤ 1%

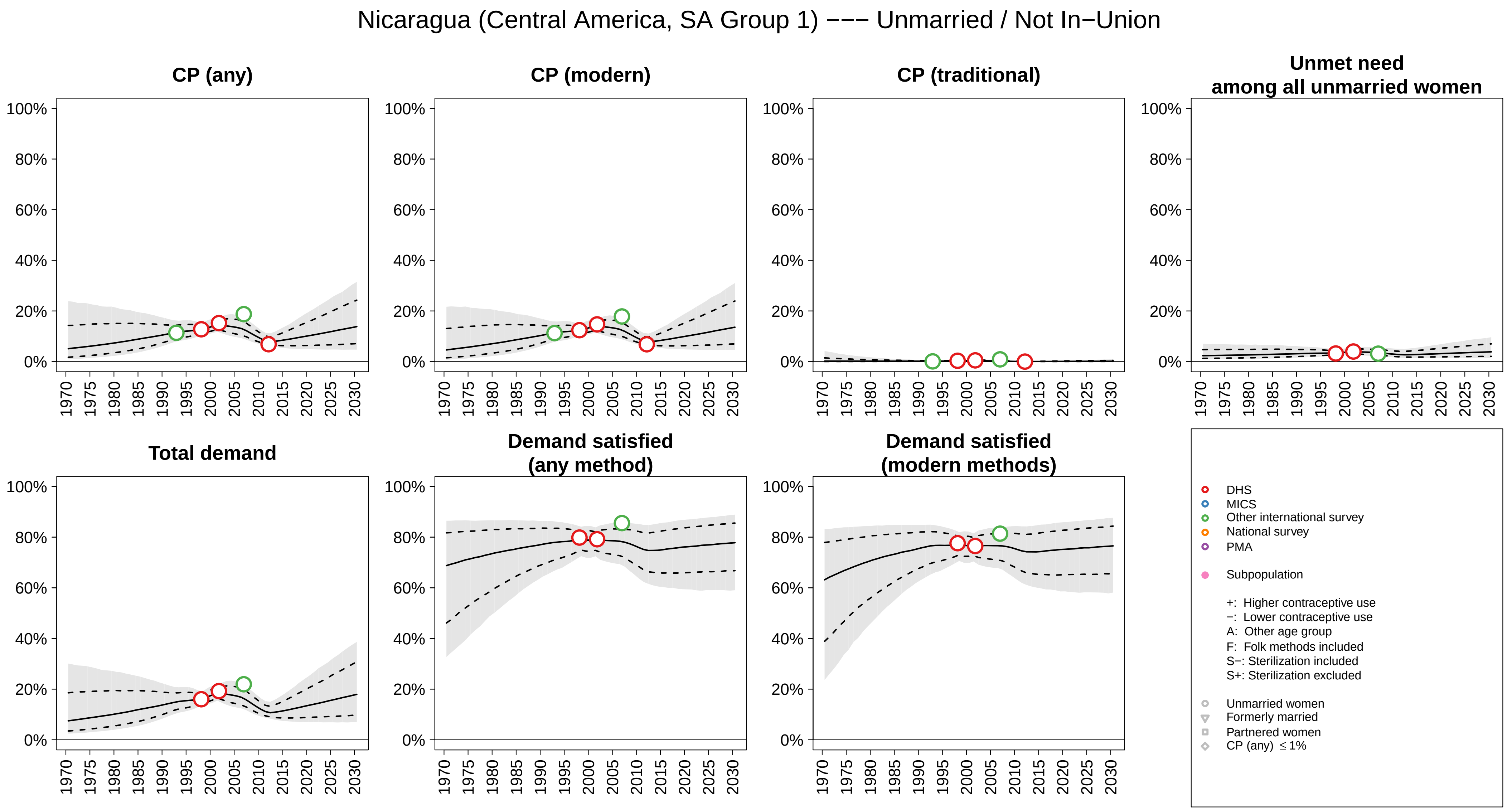

Nicaragua (Central America, SA Group 1) −−− Unmarried / Not In−Union
CP (any)
CP (modern)
CP (traditional)
Unmet need among all unmarried women
Total demand
Demand satisfied (any method)
Demand satisfied (modern methods)
DHS
MICS
Other international survey
National survey
PMA
Subpopulation
+: Higher contraceptive use
−: Lower contraceptive use
A: Other age group
F: Folk methods included
S−: Sterilization included
S+: Sterilization excluded
Unmarried women
Formerly married
Partnered women
CP (any) ≤ 1%

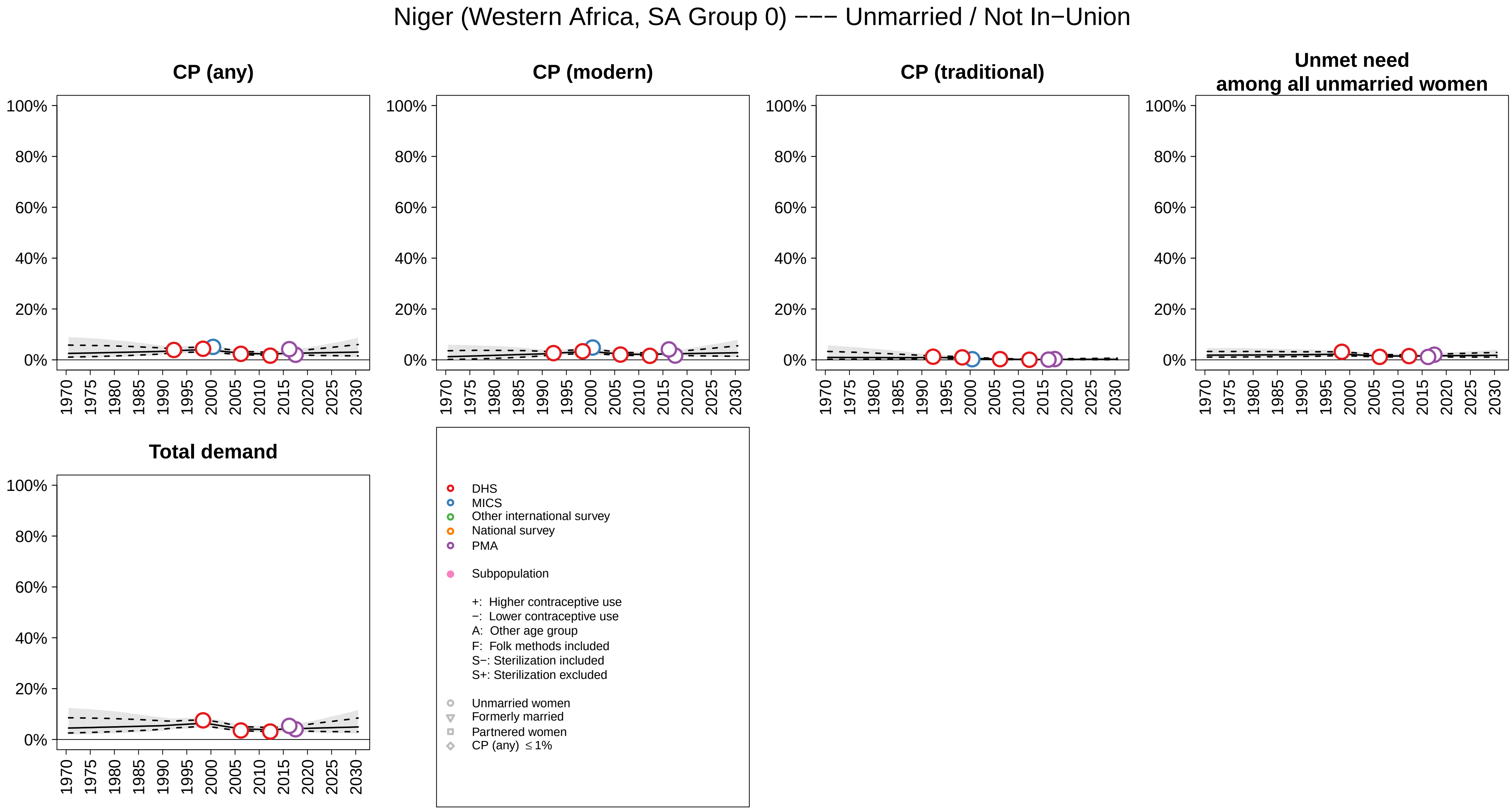

Niger (Western Africa, SA Group 0) −−− Unmarried / Not In−Union
CP (any)
CP (modern)
CP (traditional)
Unmet need
among all unmarried women
Total demand
100%
80%
60%
40%
20%
0%
1970
1975
1980
1985
1990
1995
2000
2005
2010
2015
2020
2025
2030
DHS
MICS
Other international survey
National survey
PMA
Subpopulation
+: Higher contraceptive use
−: Lower contraceptive use
A: Other age group
F: Folk methods included
S−: Sterilization included
S+: Sterilization excluded
Unmarried women
Formerly married
Partnered women
CP (any) ≤ 1%

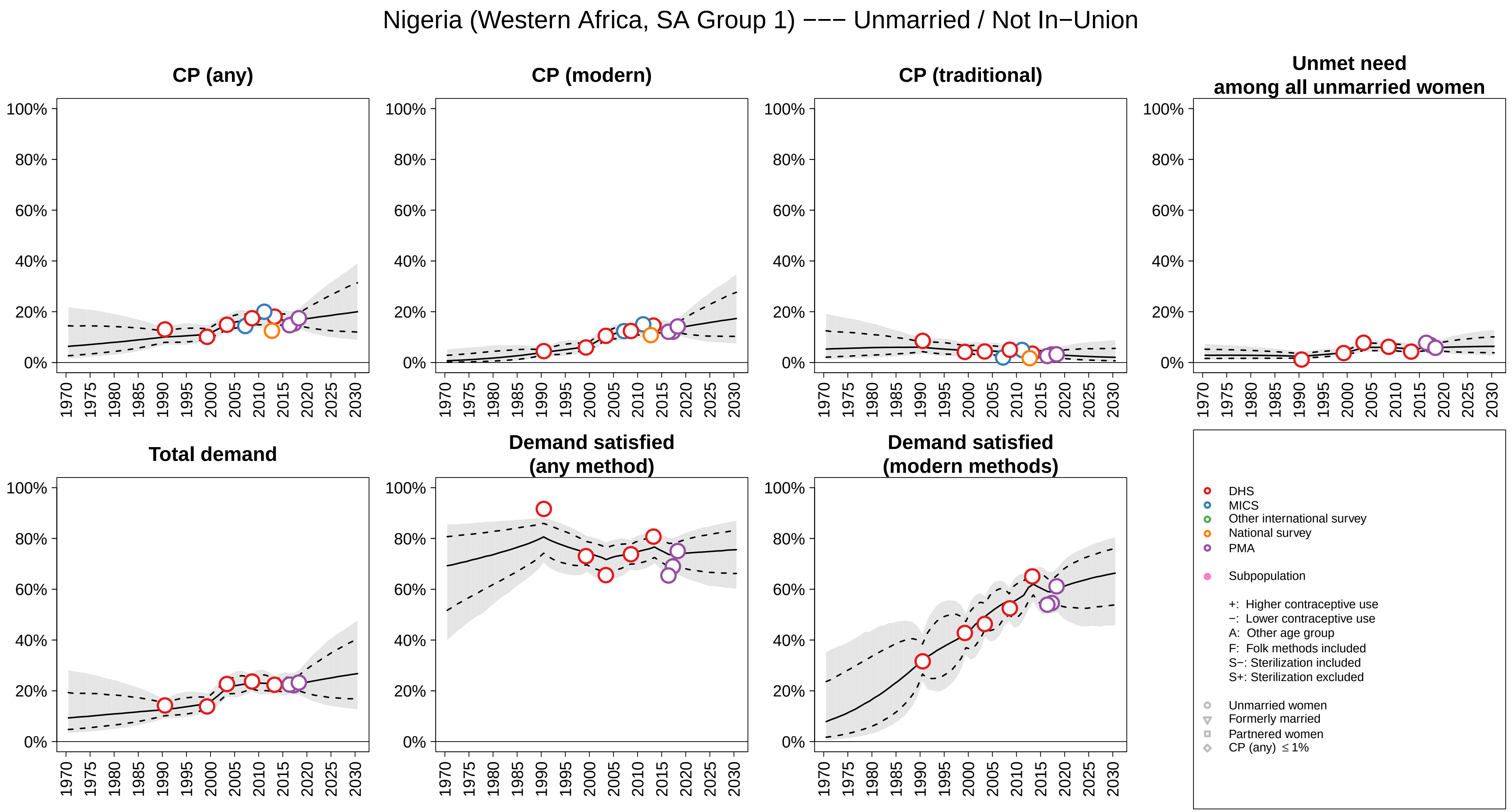

Nigeria (Western Africa, SA Group 1) --- Unmarried / Not In−Union
CP (any)
CP (modern)
CP (traditional)
Unmet need among all unmarried women
Total demand
Demand satisfied (any method)
Demand satisfied (modern methods)
0%
20%
40%
60%
80%
100%
1970
1975
1980
1985
1990
1995
2000
2005
2010
2015
2020
2025
2030
DHS
MICS
Other international survey
National survey
PMA
Subpopulation
+: Higher contraceptive use
−: Lower contraceptive use
A: Other age group
F: Folk methods included
S−: Sterilization included
S+: Sterilization excluded
Unmarried women
Formerly married
Partnered women
CP (any) ≤ 1%

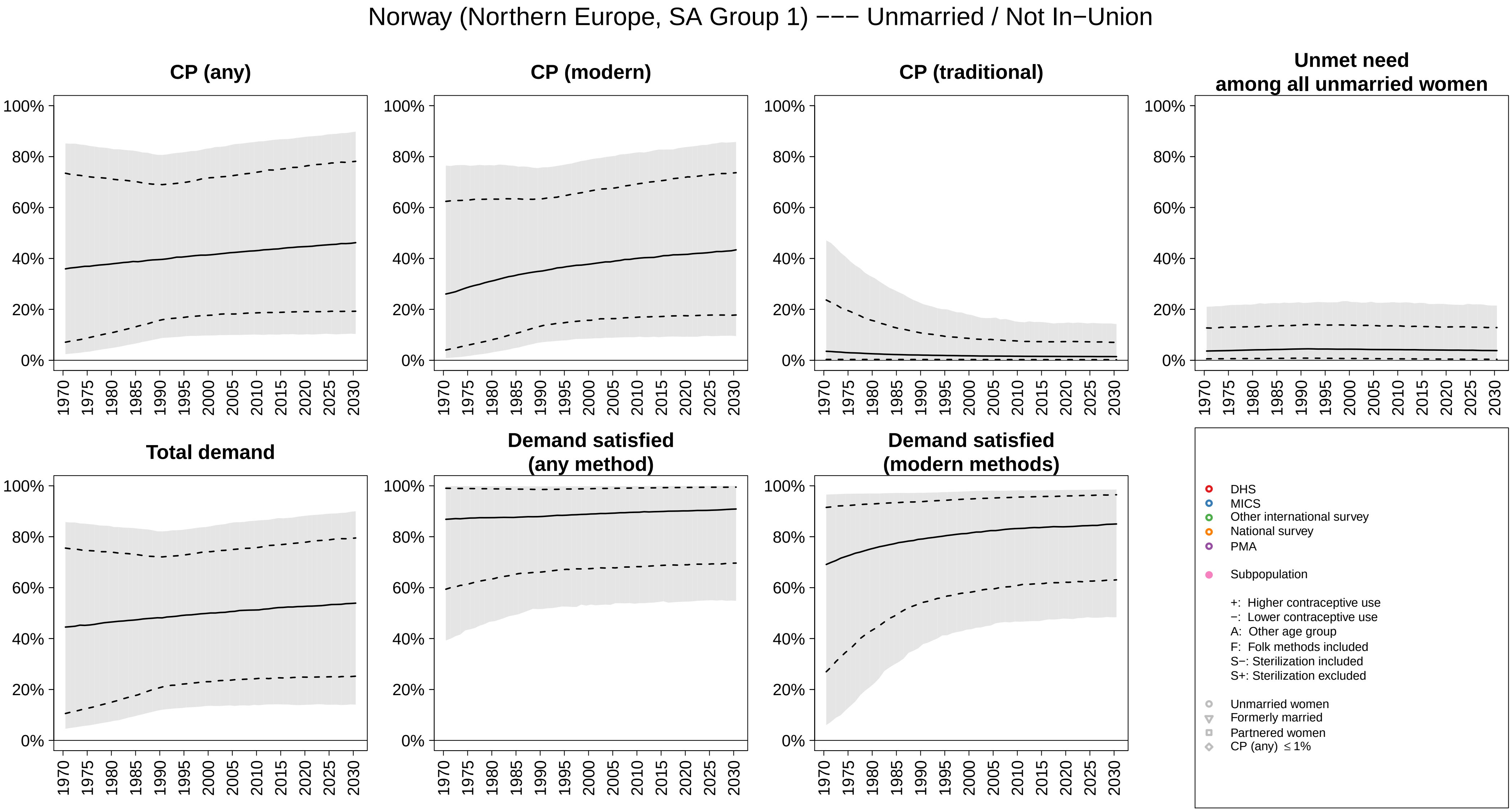

Norway (Northern Europe, SA Group 1) −−− Unmarried / Not In−Union
CP (any)
CP (modern)
CP (traditional)
Unmet need among all unmarried women
Total demand
Demand satisfied (any method)
Demand satisfied (modern methods)
100%
80%
60%
40%
20%
0%
1970 1975 1980 1985 1990 1995 2000 2005 2010 2015 2020 2025 2030
DHS
MICS
Other international survey
National survey
PMA
Subpopulation
+: Higher contraceptive use
−: Lower contraceptive use
A: Other age group
F: Folk methods included
S−: Sterilization included
S+: Sterilization excluded
Unmarried women
Formerly married
Partnered women
CP (any) ≤ 1%

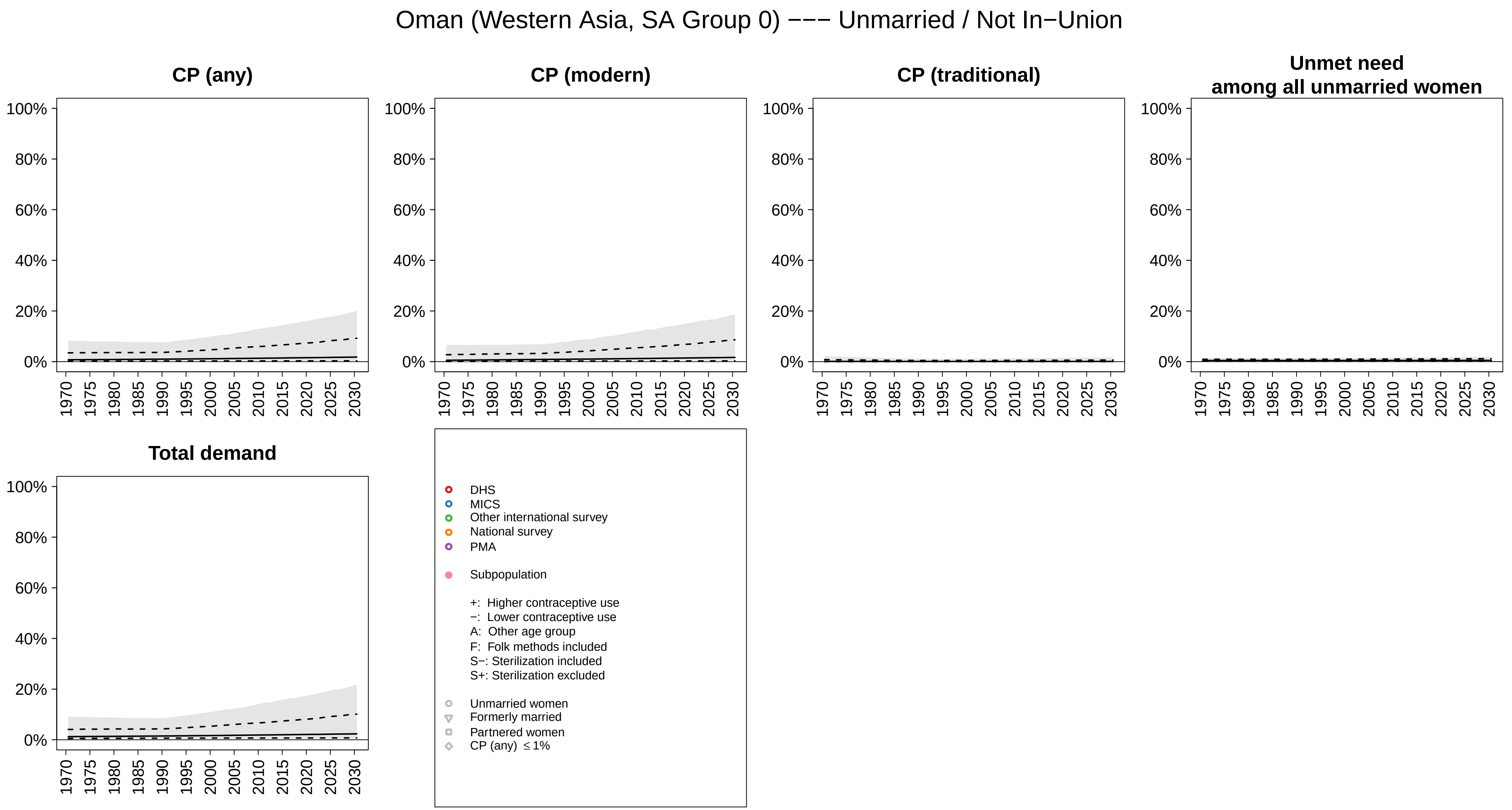
Oman (Western Asia, SA Group 0) −−− Unmarried / Not In−Union
CP (any)
CP (modern)
CP (traditional)
Unmet need among all unmarried women
Total demand
100%
80%
60%
40%
20%
0%
1970
1975
1980
1985
1990
1995
2000
2005
2010
2015
2020
2025
2030
DHS
MICS
Other international survey
National survey
PMA
Subpopulation
+: Higher contraceptive use
−: Lower contraceptive use
A: Other age group
F: Folk methods included
S−: Sterilization included
S+: Sterilization excluded
Unmarried women
Formerly married
Partnered women
CP (any) ≤ 1%

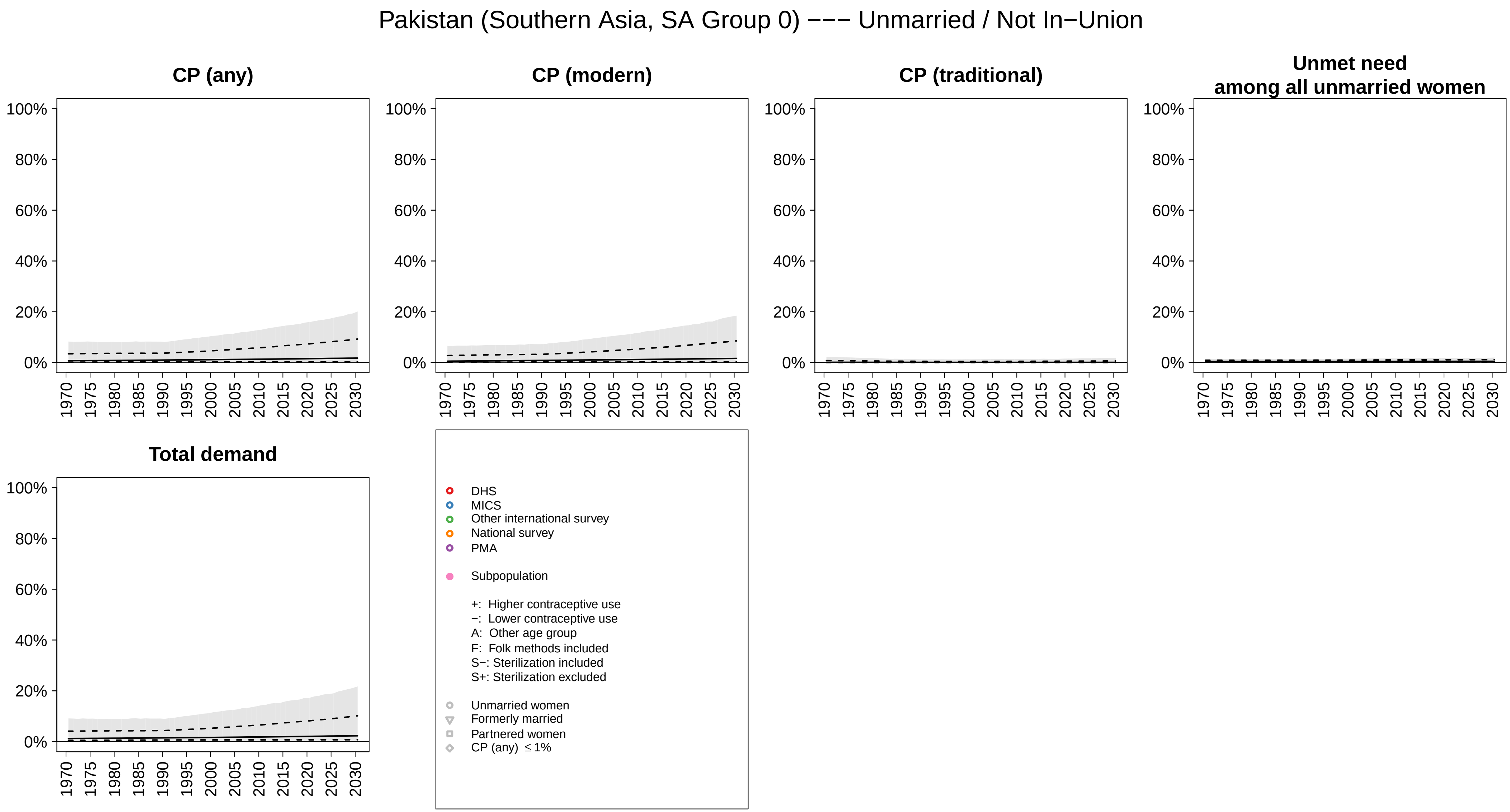
Pakistan (Southern Asia, SA Group 0) −−− Unmarried / Not In−Union
CP (any)
CP (modern)
CP (traditional)
Unmet need among all unmarried women
Total demand
100%
80%
60%
40%
20%
0%
1970 1975 1980 1985 1990 1995 2000 2005 2010 2015 2020 2025 2030
DHS
MICS
Other international survey
National survey
PMA
Subpopulation
+: Higher contraceptive use
−: Lower contraceptive use
A: Other age group
F: Folk methods included
S−: Sterilization included
S+: Sterilization excluded
Unmarried women
Formerly married
Partnered women
CP (any) ≤ 1%

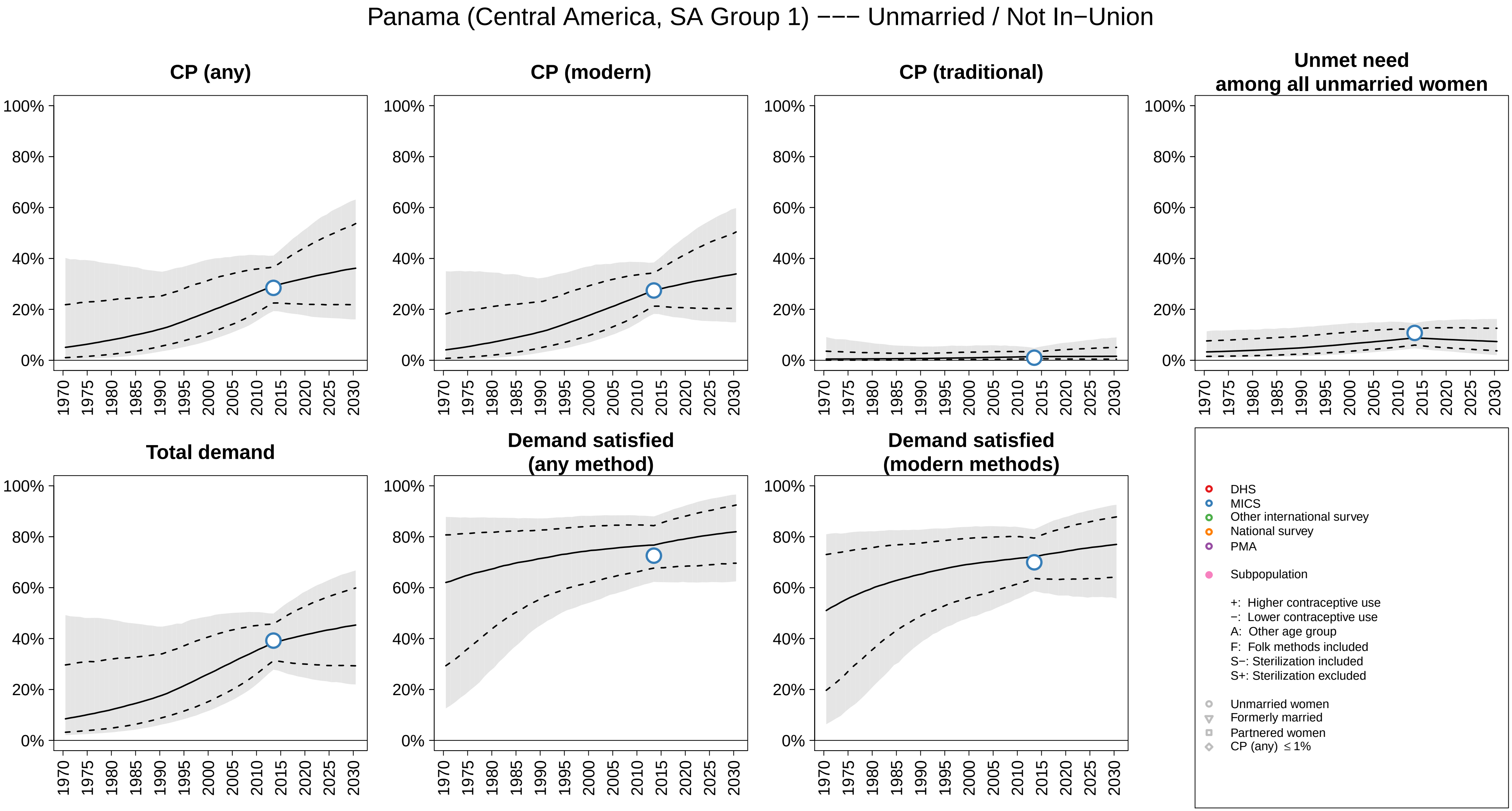
Panama (Central America, SA Group 1) −−− Unmarried / Not In−Union
CP (any)
CP (modern)
CP (traditional)
Unmet need among all unmarried women
Total demand
Demand satisfied (any method)
Demand satisfied (modern methods)
100%
80%
60%
40%
20%
0%
1970 1975 1980 1985 1990 1995 2000 2005 2010 2015 2020 2025 2030
DHS
MICS
Other international survey
National survey
PMA
Subpopulation
+: Higher contraceptive use
−: Lower contraceptive use
A: Other age group
F: Folk methods included
S−: Sterilization included
S+: Sterilization excluded
Unmarried women
Formerly married
Partnered women
CP (any) ≤ 1%

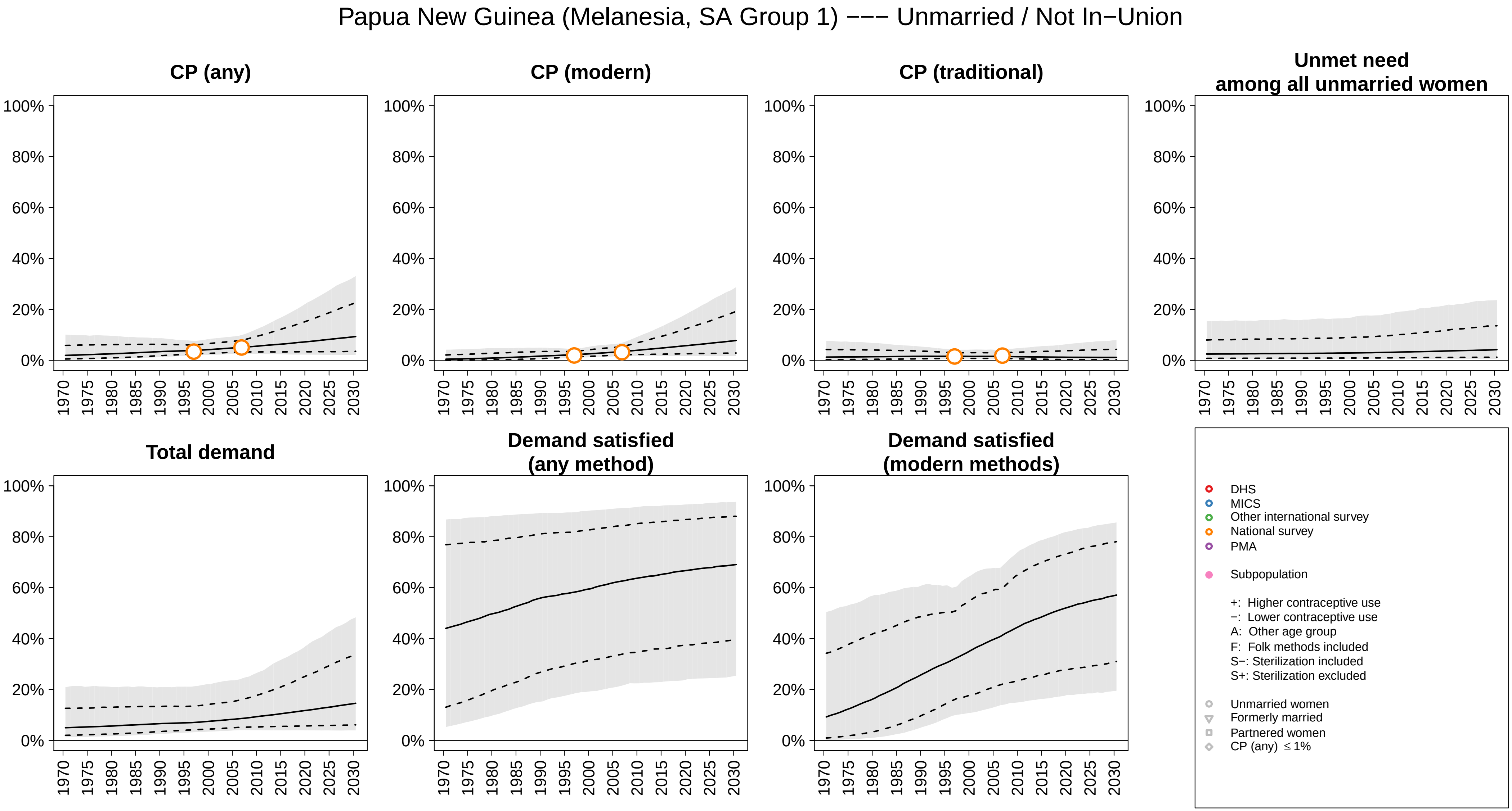
Papua New Guinea (Melanesia, SA Group 1) −−− Unmarried / Not In−Union
CP (any)
CP (modern)
CP (traditional)
Unmet need among all unmarried women
Total demand
Demand satisfied (any method)
Demand satisfied (modern methods)
100%
80%
60%
40%
20%
0%
1970
1975
1980
1985
1990
1995
2000
2005
2010
2015
2020
2025
2030
DHS
MICS
Other international survey
National survey
PMA
Subpopulation
+: Higher contraceptive use
−: Lower contraceptive use
A: Other age group
F: Folk methods included
S−: Sterilization included
S+: Sterilization excluded
Unmarried women
Formerly married
Partnered women
CP (any) ≤ 1%

Paraguay (South America, SA Group 1) −−− Unmarried / Not In−Union

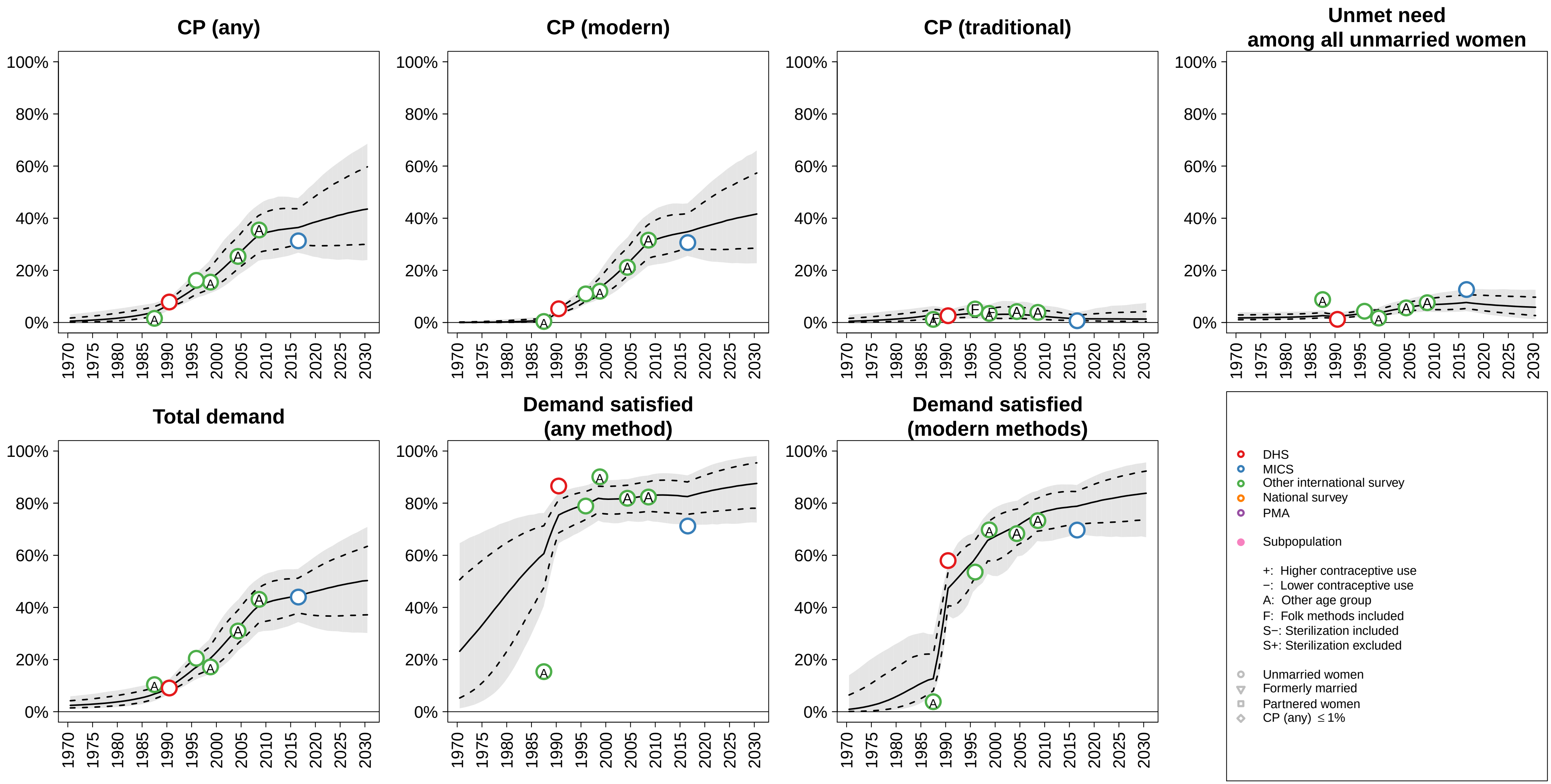

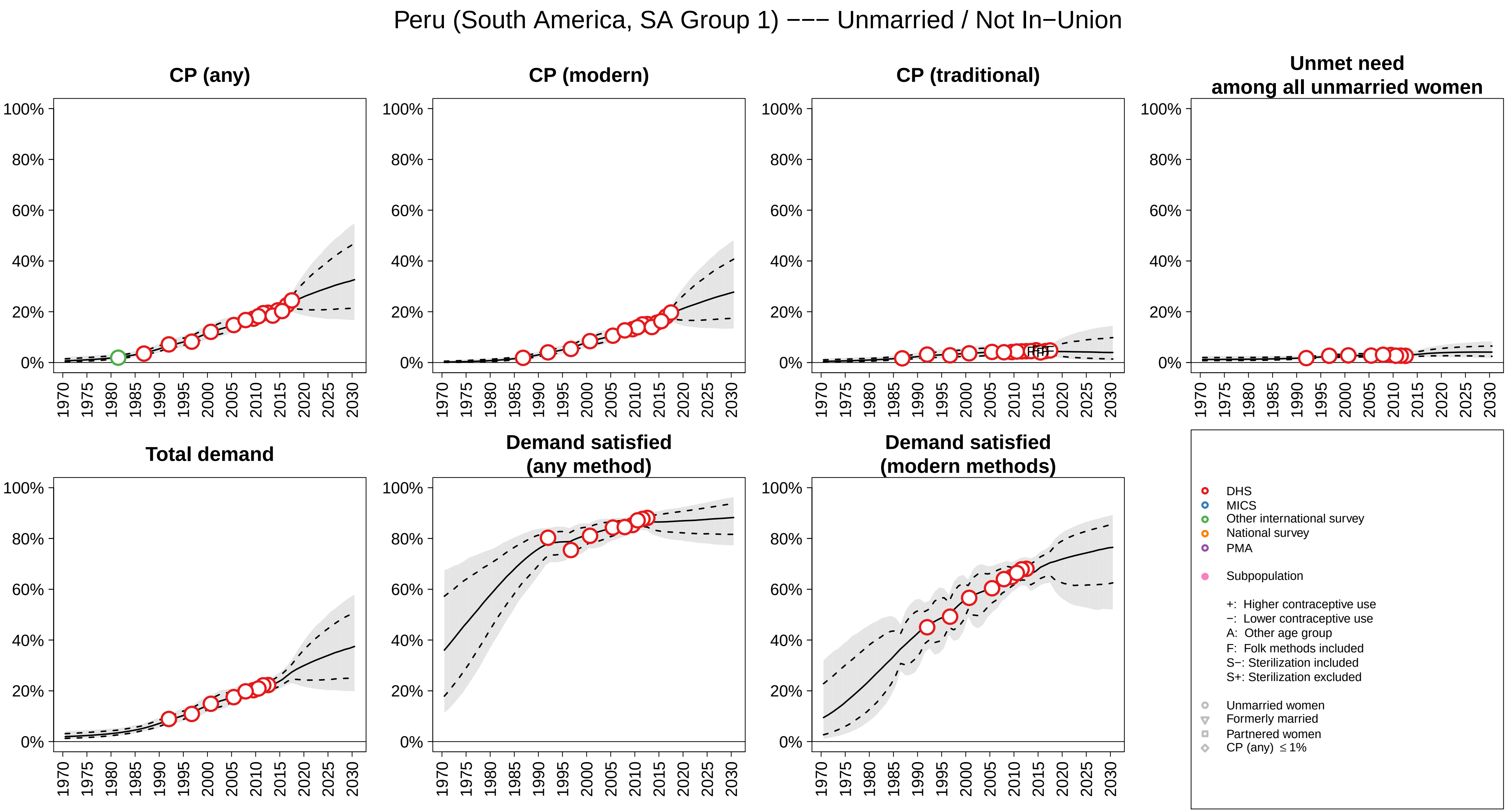
Peru (South America, SA Group 1) −−− Unmarried / Not In−Union
CP (any)
CP (modern)
CP (traditional)
Unmet need among all unmarried women
Total demand
Demand satisfied (any method)
Demand satisfied (modern methods)
100%
80%
60%
40%
20%
0%
1970 1975 1980 1985 1990 1995 2000 2005 2010 2015 2020 2025 2030
DHS
MICS
Other international survey
National survey
PMA
Subpopulation
+: Higher contraceptive use
−: Lower contraceptive use
A: Other age group
F: Folk methods included
S−: Sterilization included
S+: Sterilization excluded
Unmarried women
Formerly married
Partnered women
CP (any) ≤ 1%

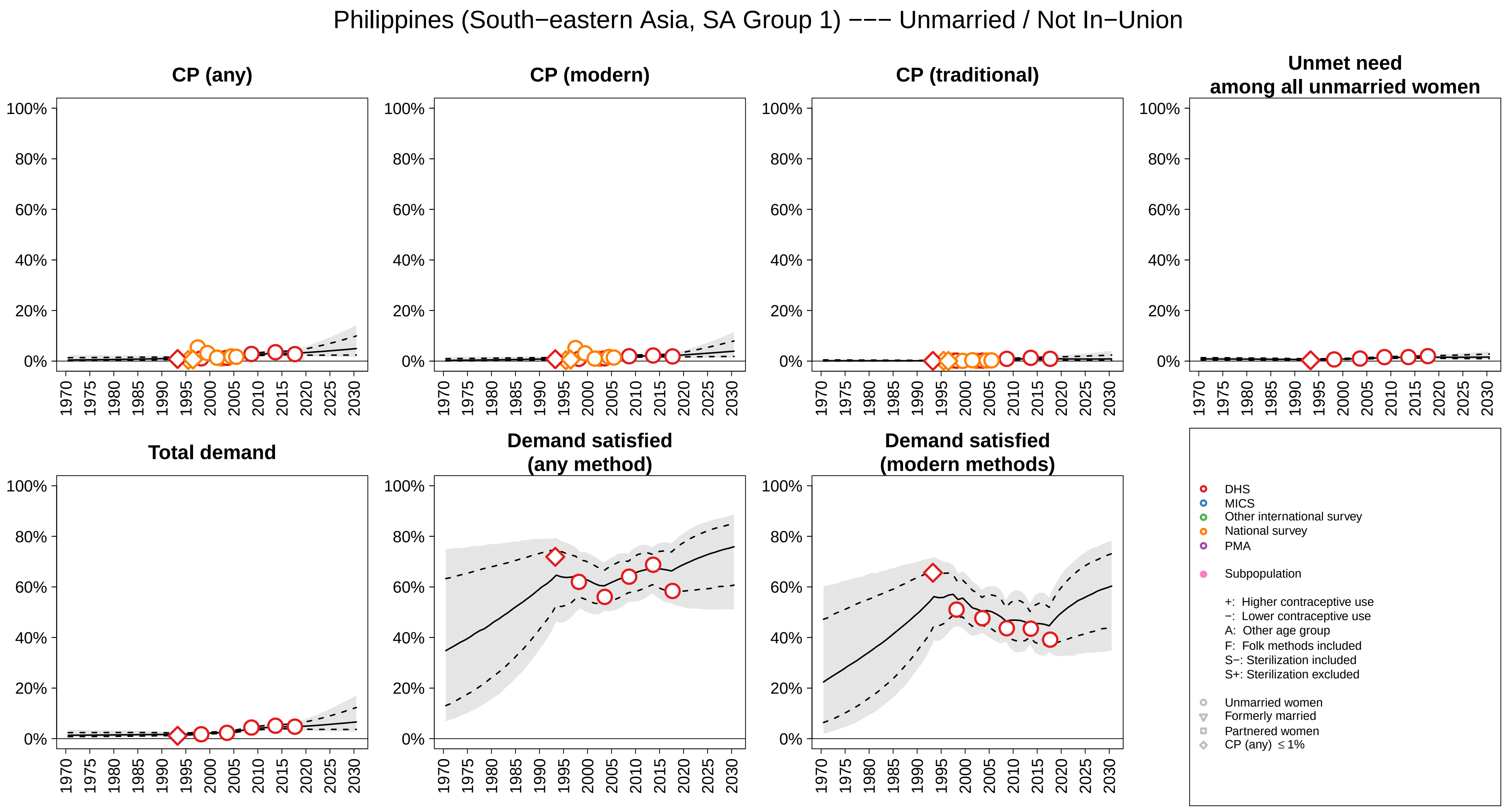
Philippines (South−eastern Asia, SA Group 1) −−− Unmarried / Not In−Union
CP (any)
CP (modern)
CP (traditional)
Unmet need among all unmarried women
Total demand
Demand satisfied (any method)
Demand satisfied (modern methods)
100%
80%
60%
40%
20%
0%
1970 1975 1980 1985 1990 1995 2000 2005 2010 2015 2020 2025 2030
DHS
MICS
Other international survey
National survey
PMA
Subpopulation
+: Higher contraceptive use
−: Lower contraceptive use
A: Other age group
F: Folk methods included
S−: Sterilization included
S+: Sterilization excluded
Unmarried women
Formerly married
Partnered women
CP (any) ≤ 1%

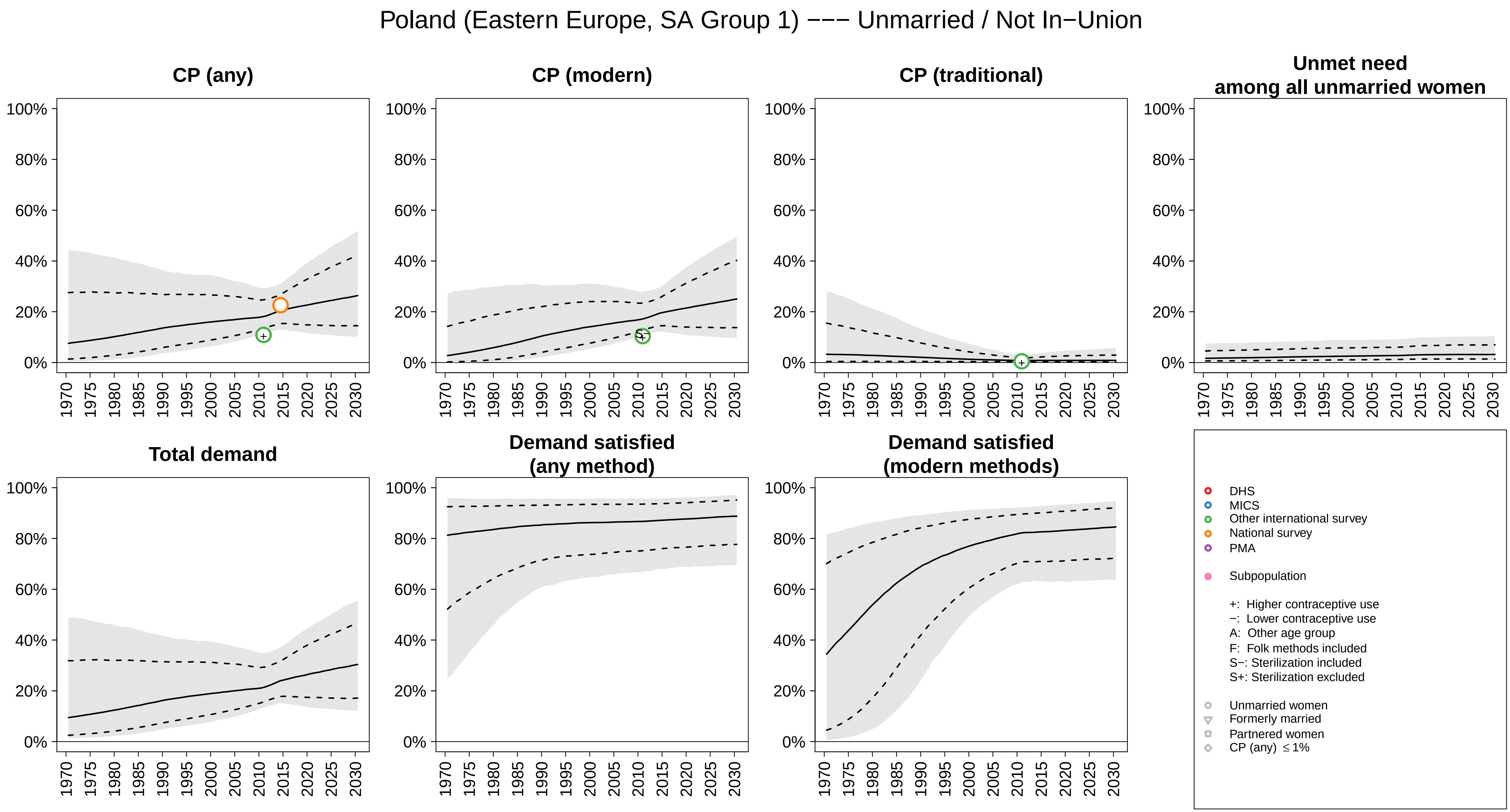
Poland (Eastern Europe, SA Group 1) −−− Unmarried / Not In−Union
CP (any)
CP (modern)
CP (traditional)
Unmet need among all unmarried women
Total demand
Demand satisfied (any method)
Demand satisfied (modern methods)
DHS
MICS
Other international survey
National survey
PMA
Subpopulation
+: Higher contraceptive use
−: Lower contraceptive use
A: Other age group
F: Folk methods included
S−: Sterilization included
S+: Sterilization excluded
Unmarried women
Formerly married
Partnered women
CP (any) ≤ 1%

Portugal (Southern Europe, SA Group 1) --- Unmarried / Not In−Union

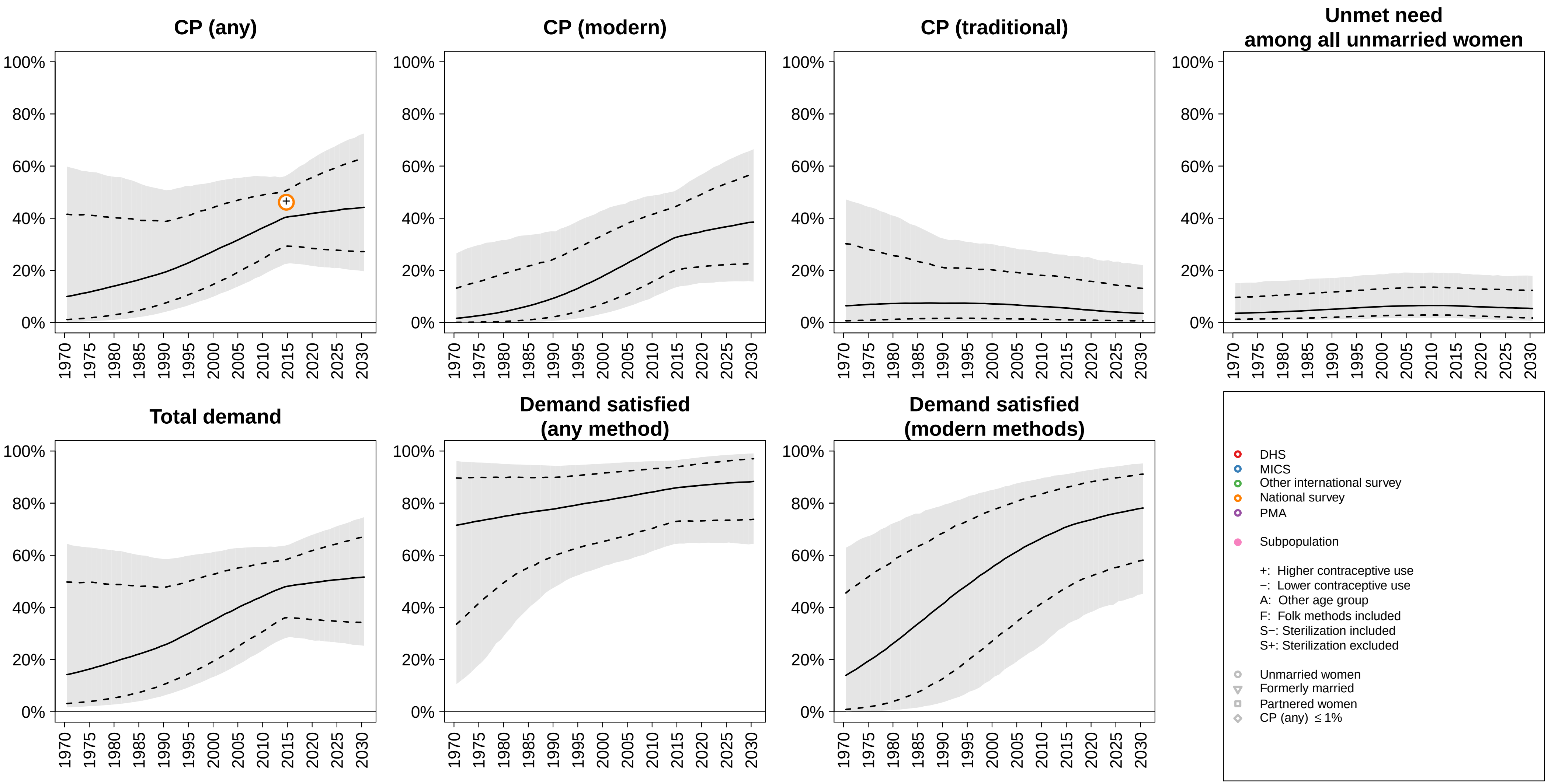

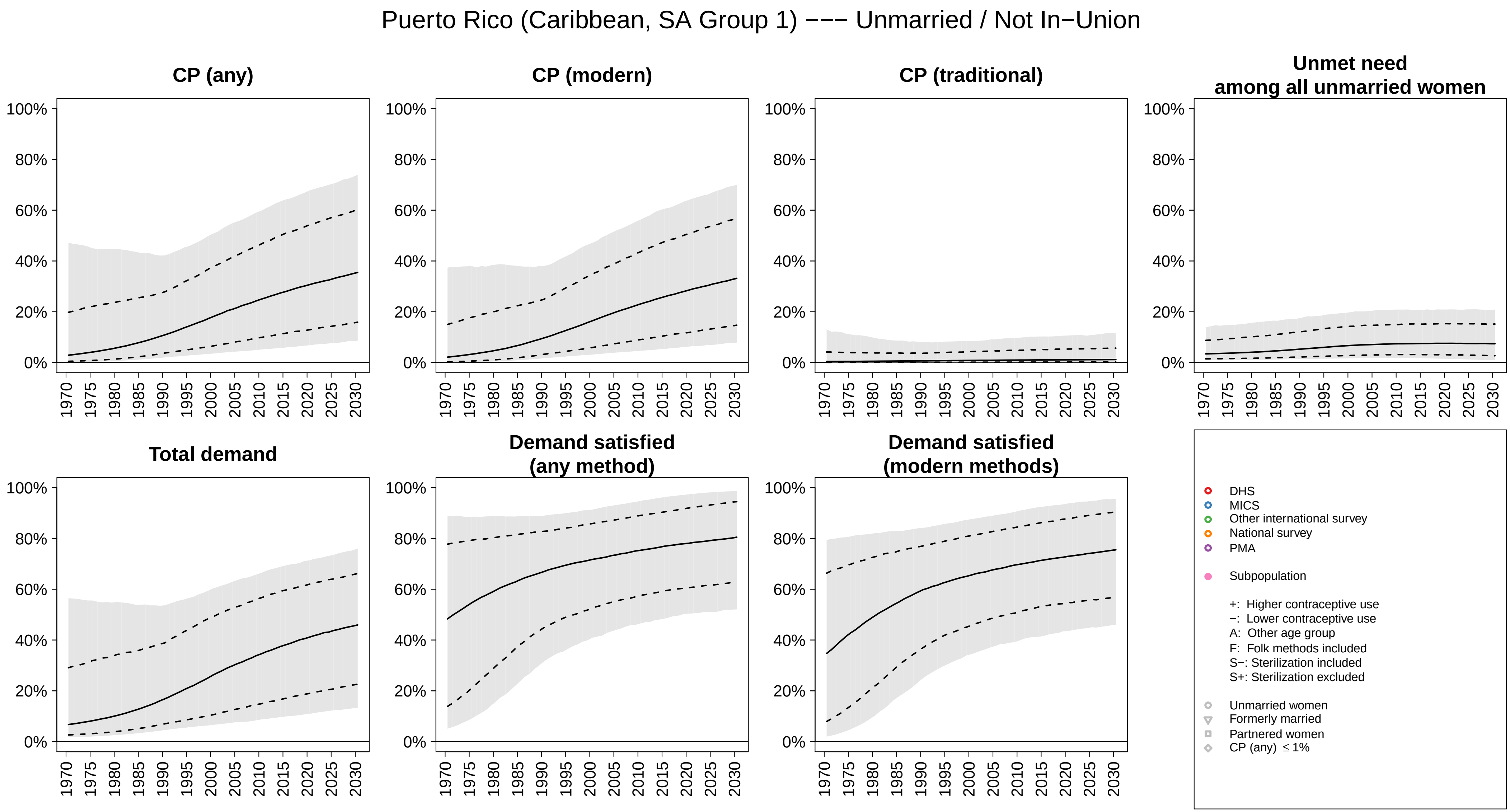
Puerto Rico (Caribbean, SA Group 1) −−− Unmarried / Not In−Union
CP (any)
CP (modern)
CP (traditional)
Unmet need among all unmarried women
Total demand
Demand satisfied (any method)
Demand satisfied (modern methods)
100%
80%
60%
40%
20%
0%
1970
1975
1980
1985
1990
1995
2000
2005
2010
2015
2020
2025
2030
DHS
MICS
Other international survey
National survey
PMA
Subpopulation
+: Higher contraceptive use
−: Lower contraceptive use
A: Other age group
F: Folk methods included
S−: Sterilization included
S+: Sterilization excluded
Unmarried women
Formerly married
Partnered women
CP (any) ≤ 1%

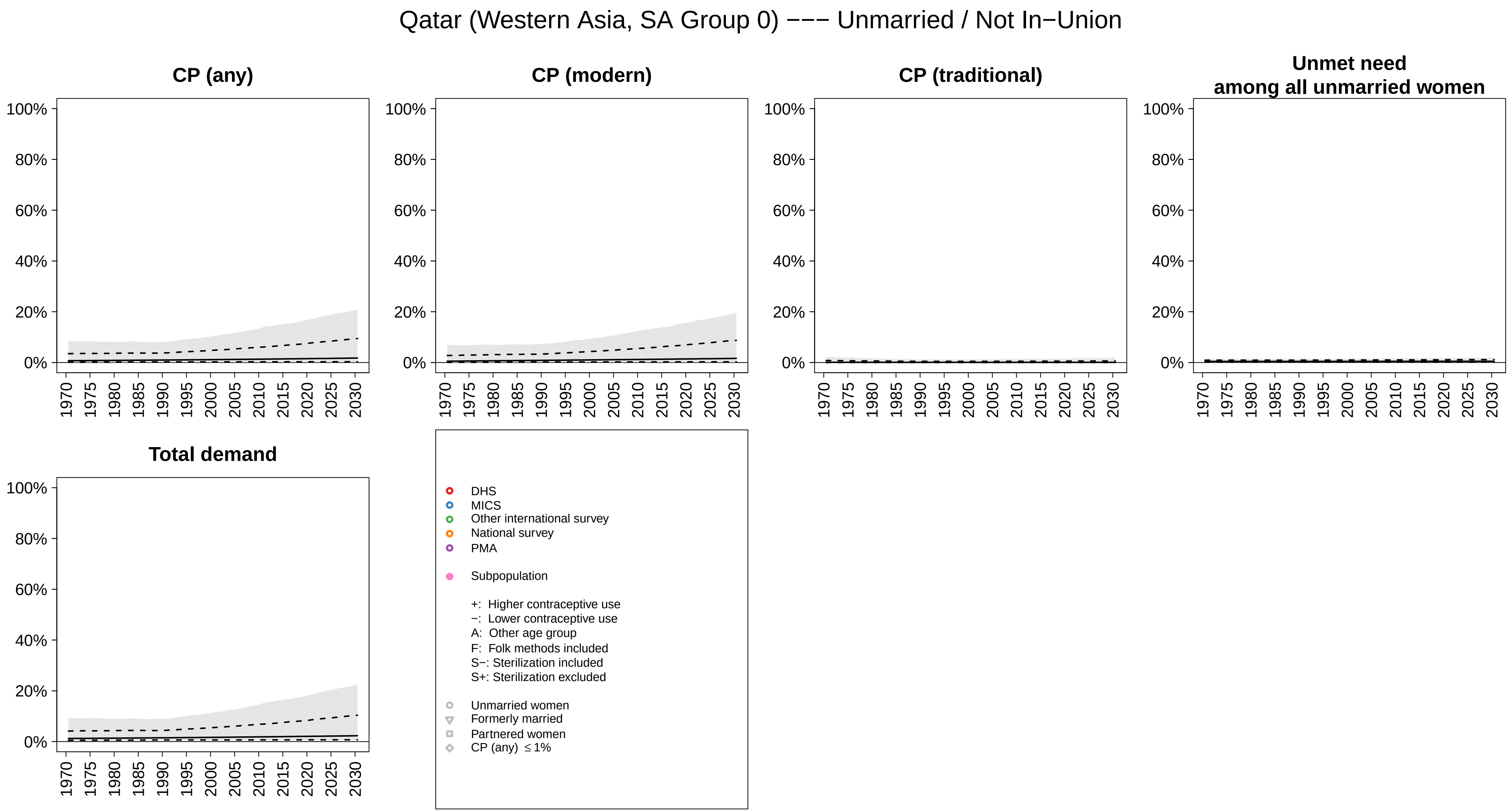
Qatar (Western Asia, SA Group 0) −−− Unmarried / Not In−Union
CP (any)
CP (modern)
CP (traditional)
Unmet need among all unmarried women
Total demand
100%
80%
60%
40%
20%
0%
1970 1975 1980 1985 1990 1995 2000 2005 2010 2015 2020 2025 2030
DHS
MICS
Other international survey
National survey
PMA
Subpopulation
+: Higher contraceptive use
−: Lower contraceptive use
A: Other age group
F: Folk methods included
S−: Sterilization included
S+: Sterilization excluded
Unmarried women
Formerly married
Partnered women
CP (any) ≤ 1%

Republic of Korea (Eastern Asia, SA Group 1) −−− Unmarried / Not In−Union

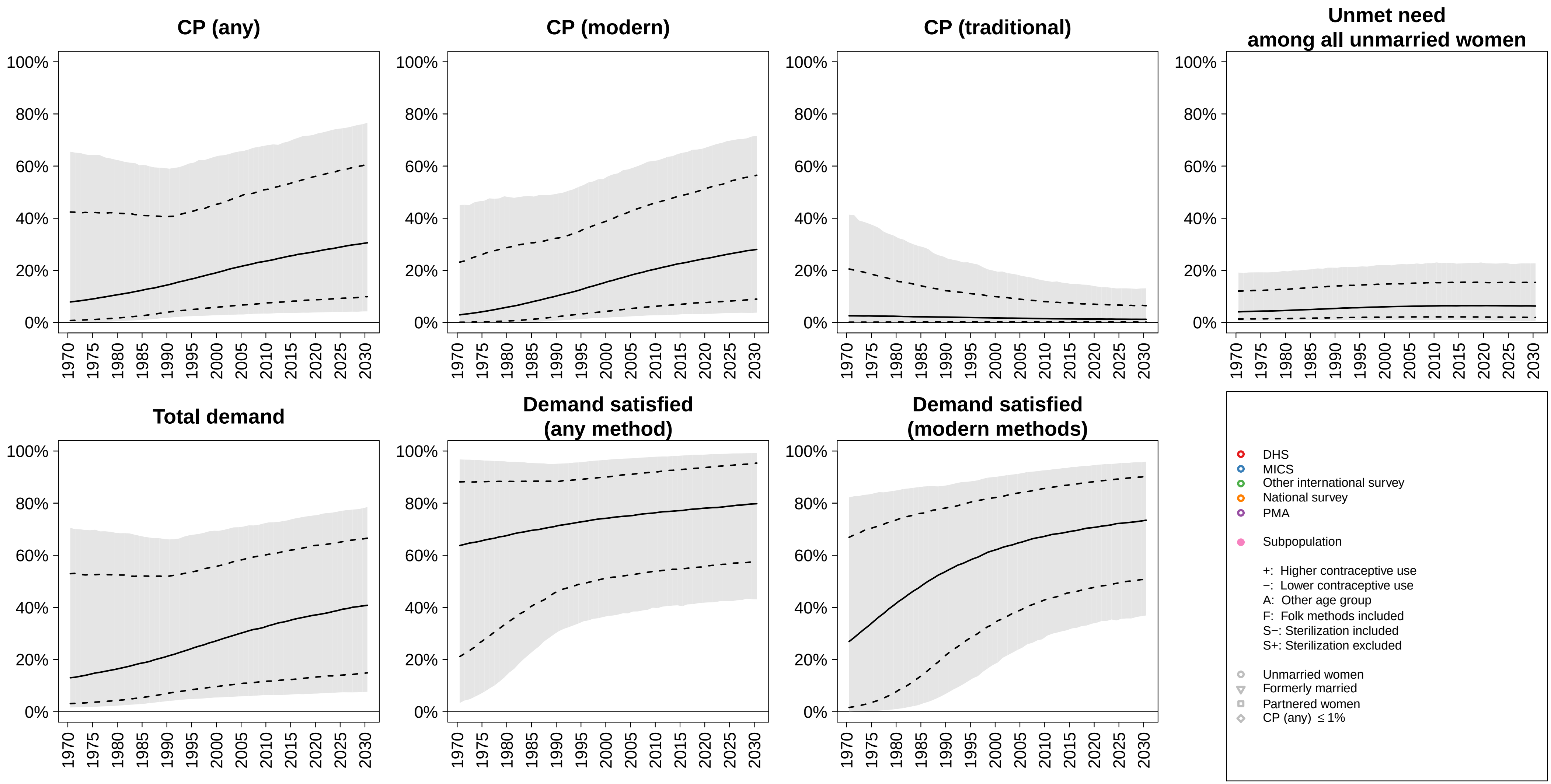

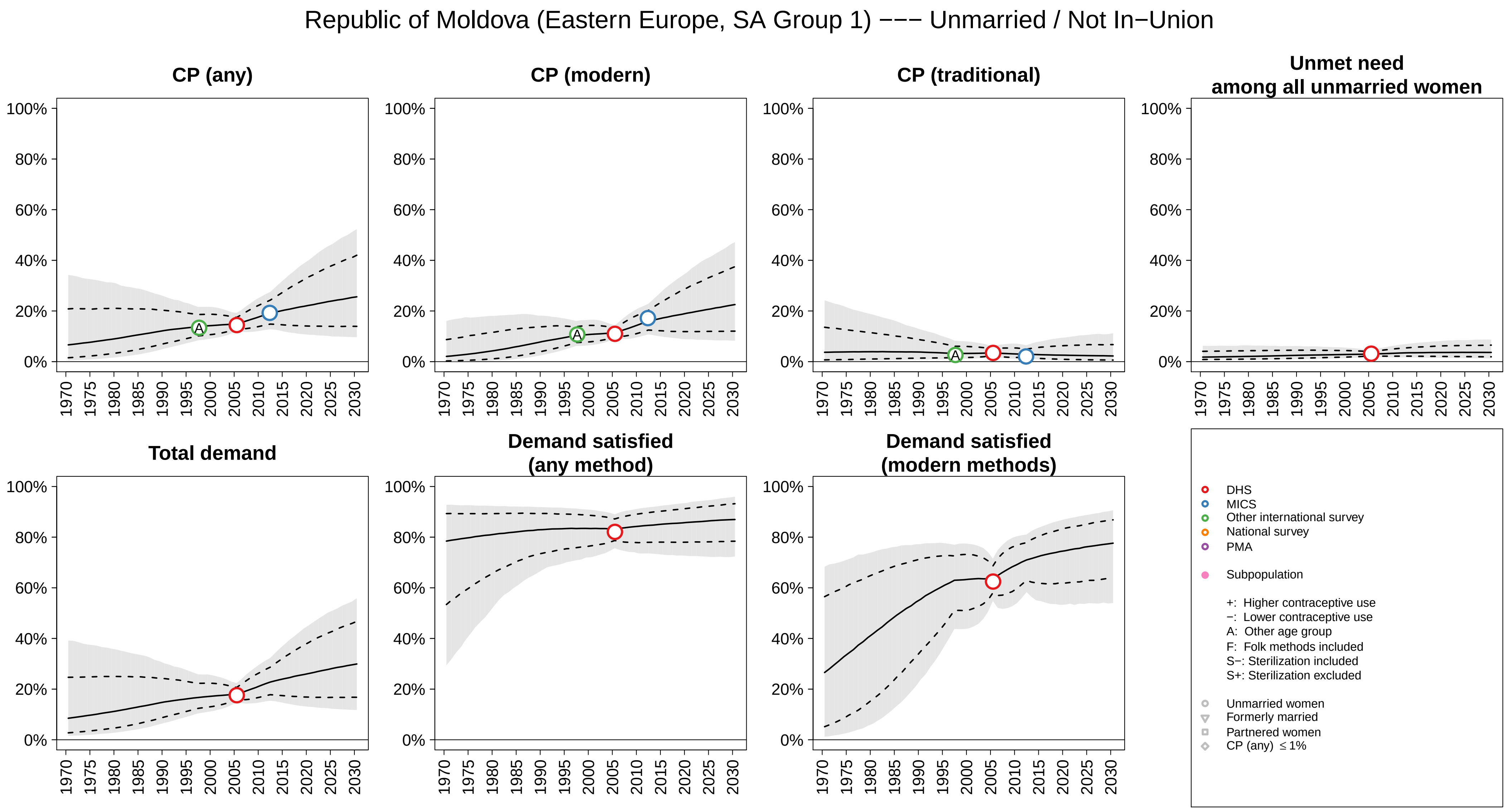
Republic of Moldova (Eastern Europe, SA Group 1) −−− Unmarried / Not In−Union
CP (any)
CP (modern)
CP (traditional)
Unmet need among all unmarried women
Total demand
Demand satisfied (any method)
Demand satisfied (modern methods)
0%
20%
40%
60%
80%
100%
1970
1975
1980
1985
1990
1995
2000
2005
2010
2015
2020
2025
2030
A
DHS
MICS
Other international survey
National survey
PMA
Subpopulation
+: Higher contraceptive use
−: Lower contraceptive use
A: Other age group
F: Folk methods included
S−: Sterilization included
S+: Sterilization excluded
Unmarried women
Formerly married
Partnered women
CP (any) ≤ 1%

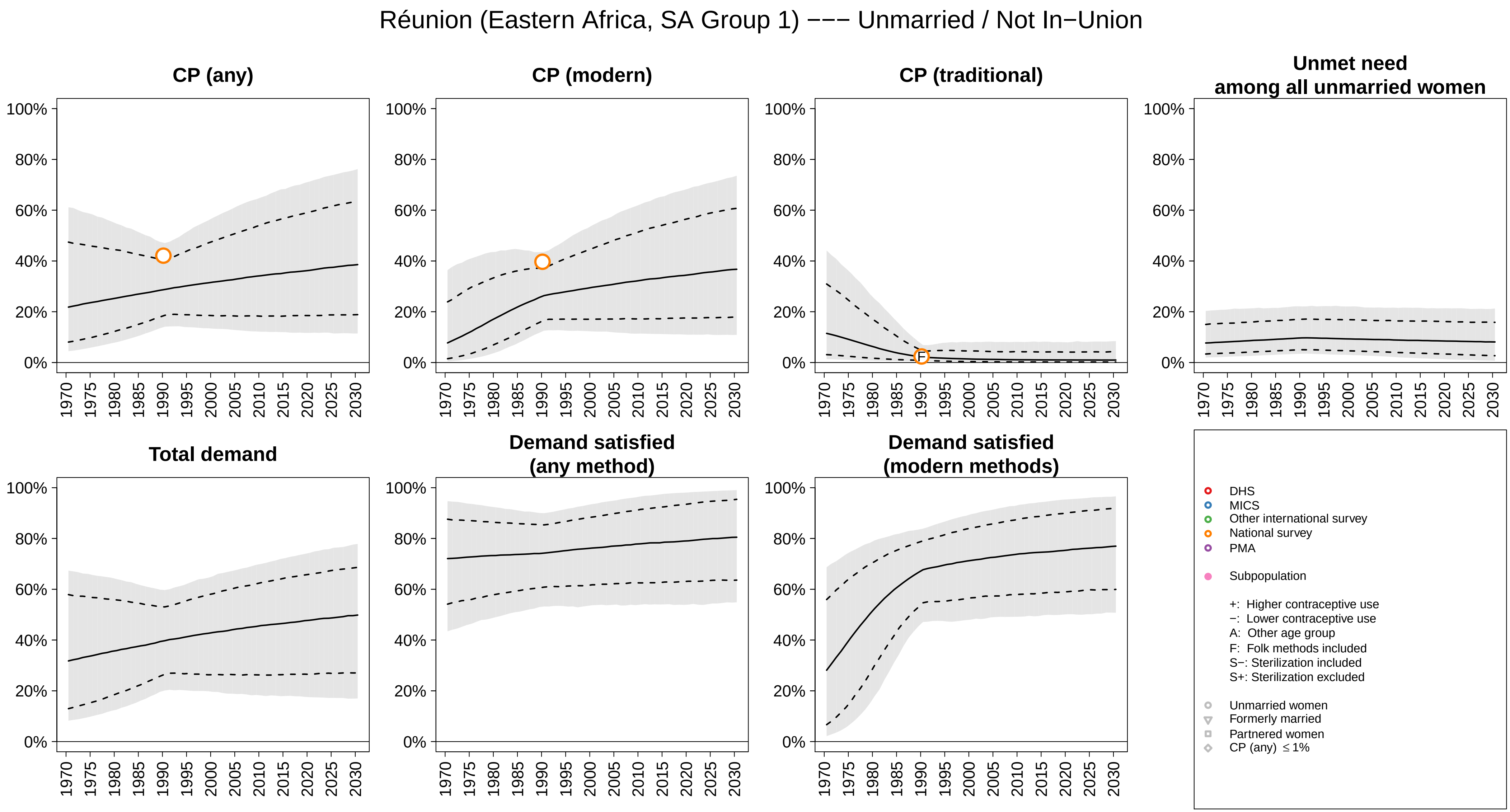
Réunion (Eastern Africa, SA Group 1) −−− Unmarried / Not In−Union
CP (any)
CP (modern)
CP (traditional)
Unmet need among all unmarried women
Total demand
Demand satisfied (any method)
Demand satisfied (modern methods)
100%
80%
60%
40%
20%
0%
1970
1975
1980
1985
1990
1995
2000
2005
2010
2015
2020
2025
2030
DHS
MICS
Other international survey
National survey
PMA
Subpopulation
+: Higher contraceptive use
−: Lower contraceptive use
A: Other age group
F: Folk methods included
S−: Sterilization included
S+: Sterilization excluded
Unmarried women
Formerly married
Partnered women
CP (any) ≤ 1%

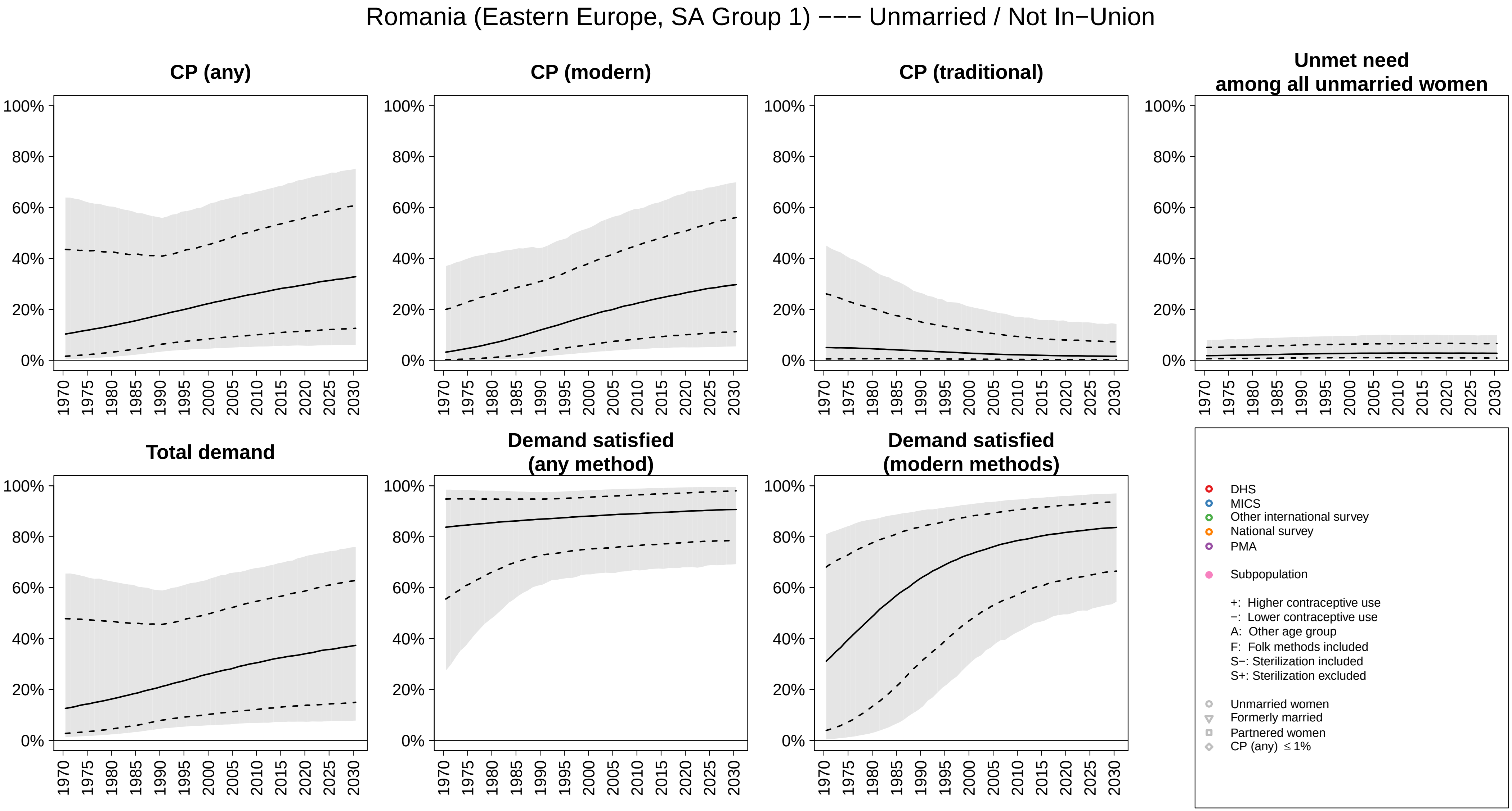
Romania (Eastern Europe, SA Group 1) −−− Unmarried / Not In−Union
CP (any)
CP (modern)
CP (traditional)
Unmet need among all unmarried women
Total demand
Demand satisfied (any method)
Demand satisfied (modern methods)
100%
80%
60%
40%
20%
0%
1970
1975
1980
1985
1990
1995
2000
2005
2010
2015
2020
2025
2030
DHS
MICS
Other international survey
National survey
PMA
Subpopulation
+: Higher contraceptive use
−: Lower contraceptive use
A: Other age group
F: Folk methods included
S−: Sterilization included
S+: Sterilization excluded
Unmarried women
Formerly married
Partnered women
CP (any) ≤ 1%

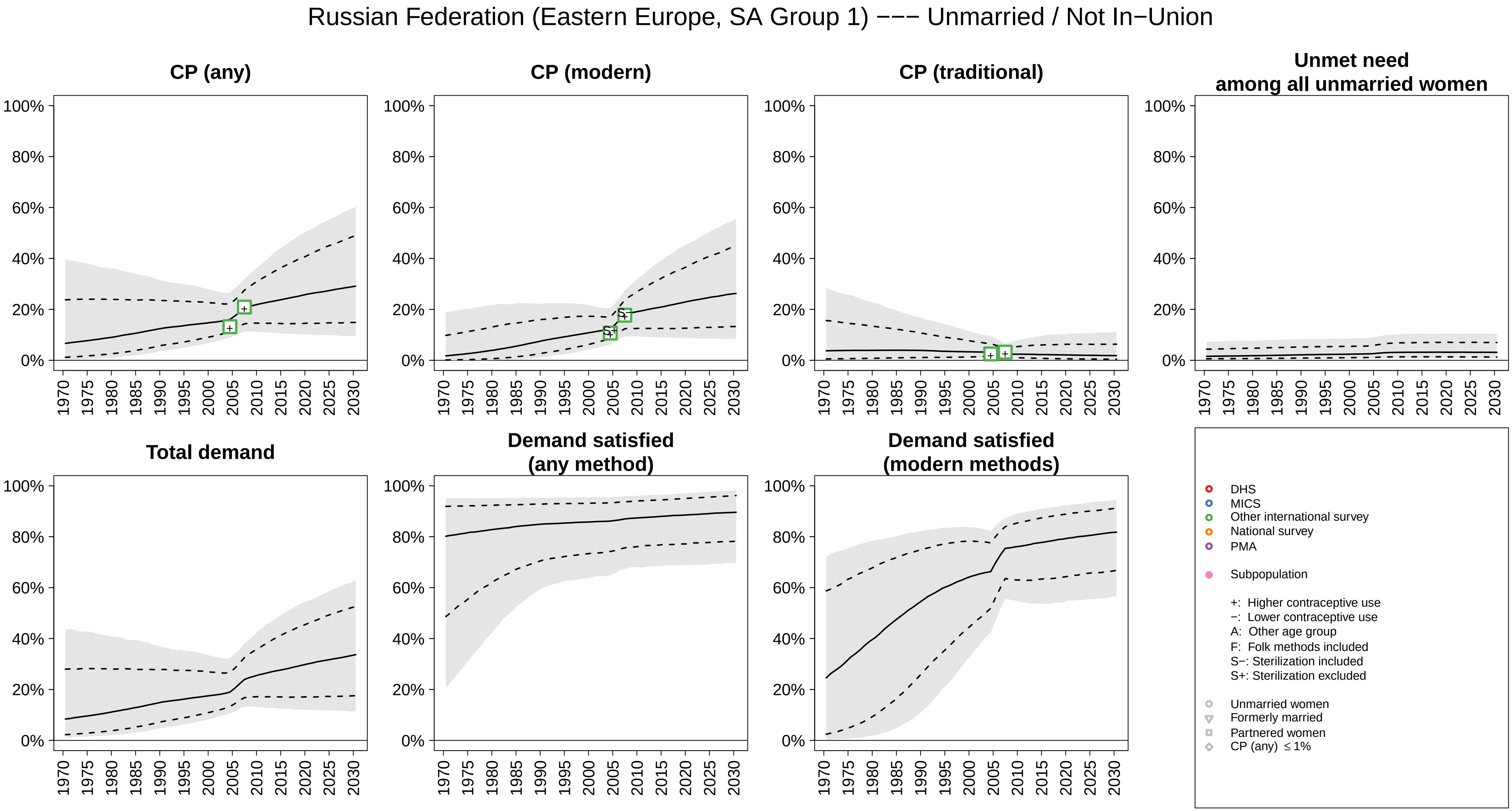
Russian Federation (Eastern Europe, SA Group 1) −−− Unmarried / Not In−Union
CP (any)
CP (modern)
CP (traditional)
Unmet need among all unmarried women
Total demand
Demand satisfied (any method)
Demand satisfied (modern methods)
0%
20%
40%
60%
80%
100%
1970
1975
1980
1985
1990
1995
2000
2005
2010
2015
2020
2025
2030
DHS
MICS
Other international survey
National survey
PMA
Subpopulation
+: Higher contraceptive use
−: Lower contraceptive use
A: Other age group
F: Folk methods included
S−: Sterilization included
S+: Sterilization excluded
Unmarried women
Formerly married
Partnered women
CP (any) ≤ 1%

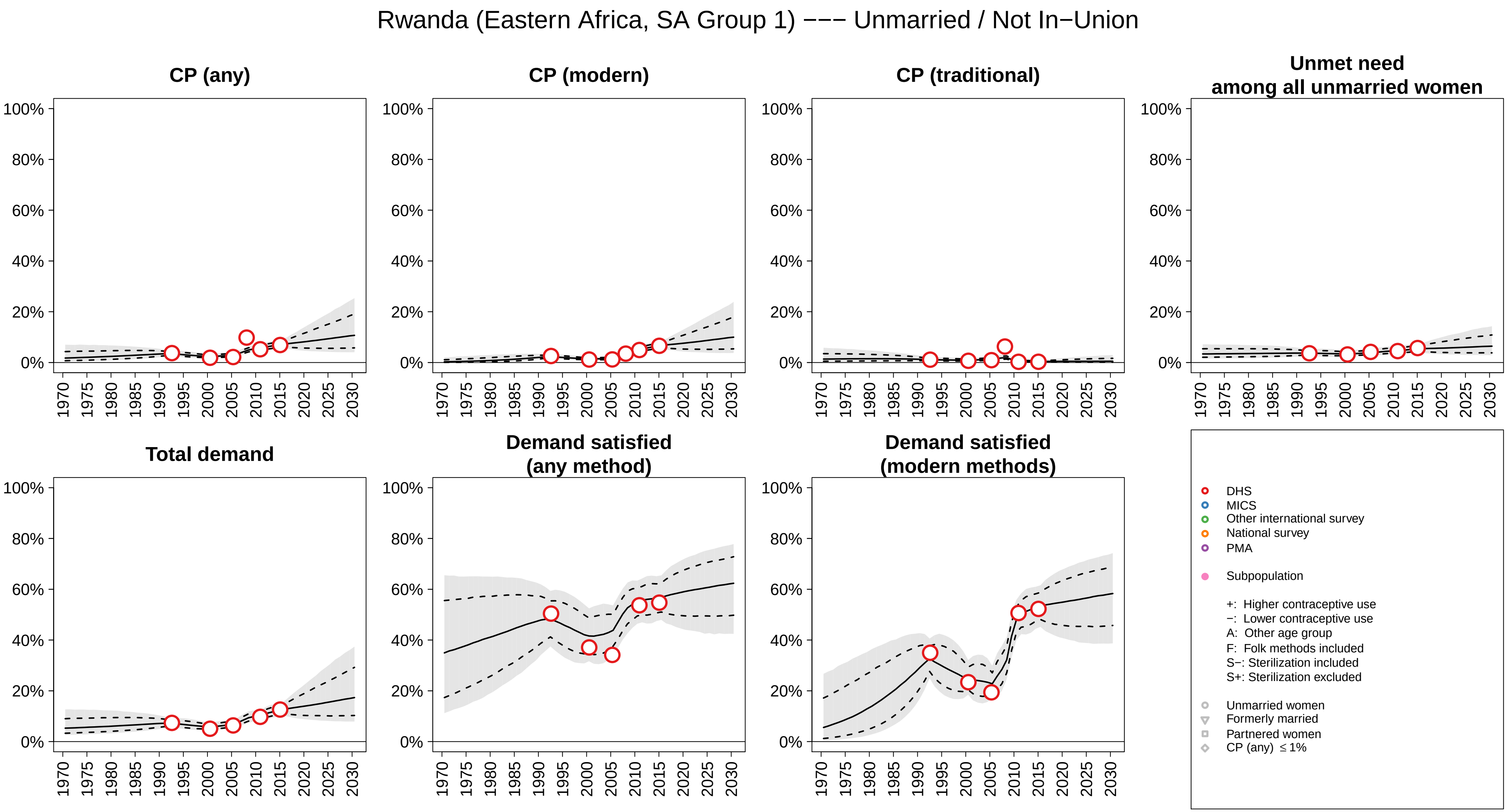
Rwanda (Eastern Africa, SA Group 1) −−− Unmarried / Not In−Union
CP (any)
CP (modern)
CP (traditional)
Unmet need among all unmarried women
Total demand
Demand satisfied (any method)
Demand satisfied (modern methods)
100%
80%
60%
40%
20%
0%
1970 1975 1980 1985 1990 1995 2000 2005 2010 2015 2020 2025 2030
DHS
MICS
Other international survey
National survey
PMA
Subpopulation
+: Higher contraceptive use
−: Lower contraceptive use
A: Other age group
F: Folk methods included
S−: Sterilization included
S+: Sterilization excluded
Unmarried women
Formerly married
Partnered women
CP (any) ≤ 1%

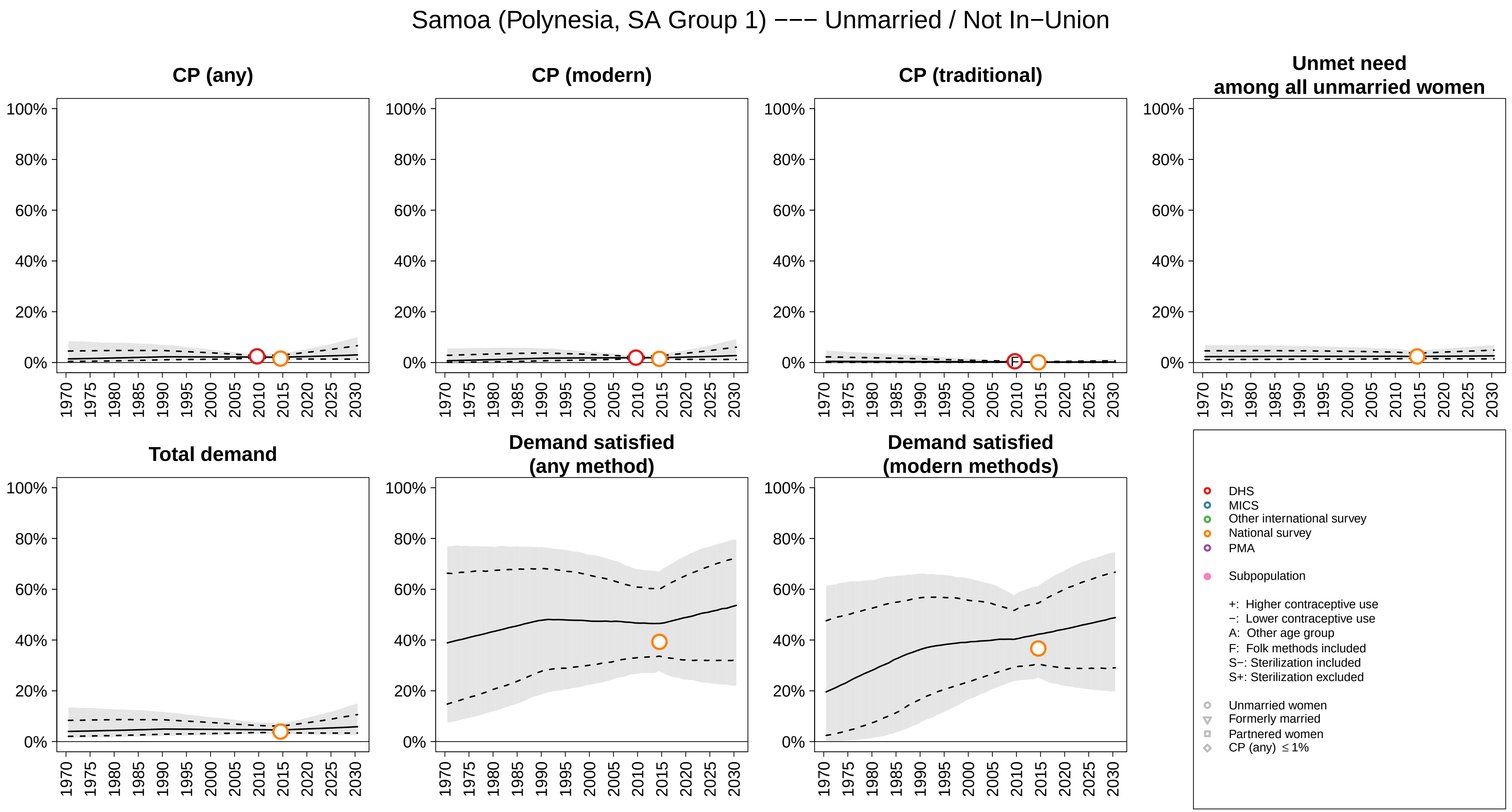
Samoa (Polynesia, SA Group 1) −−− Unmarried / Not In−Union
CP (any)
CP (modern)
CP (traditional)
Unmet need among all unmarried women
Total demand
Demand satisfied (any method)
Demand satisfied (modern methods)
0%
20%
40%
60%
80%
100%
1970
1975
1980
1985
1990
1995
2000
2005
2010
2015
2020
2025
2030
F
DHS
MICS
Other international survey
National survey
PMA
Subpopulation
+: Higher contraceptive use
−: Lower contraceptive use
A: Other age group
F: Folk methods included
S−: Sterilization included
S+: Sterilization excluded
Unmarried women
Formerly married
Partnered women
CP (any) ≤ 1%

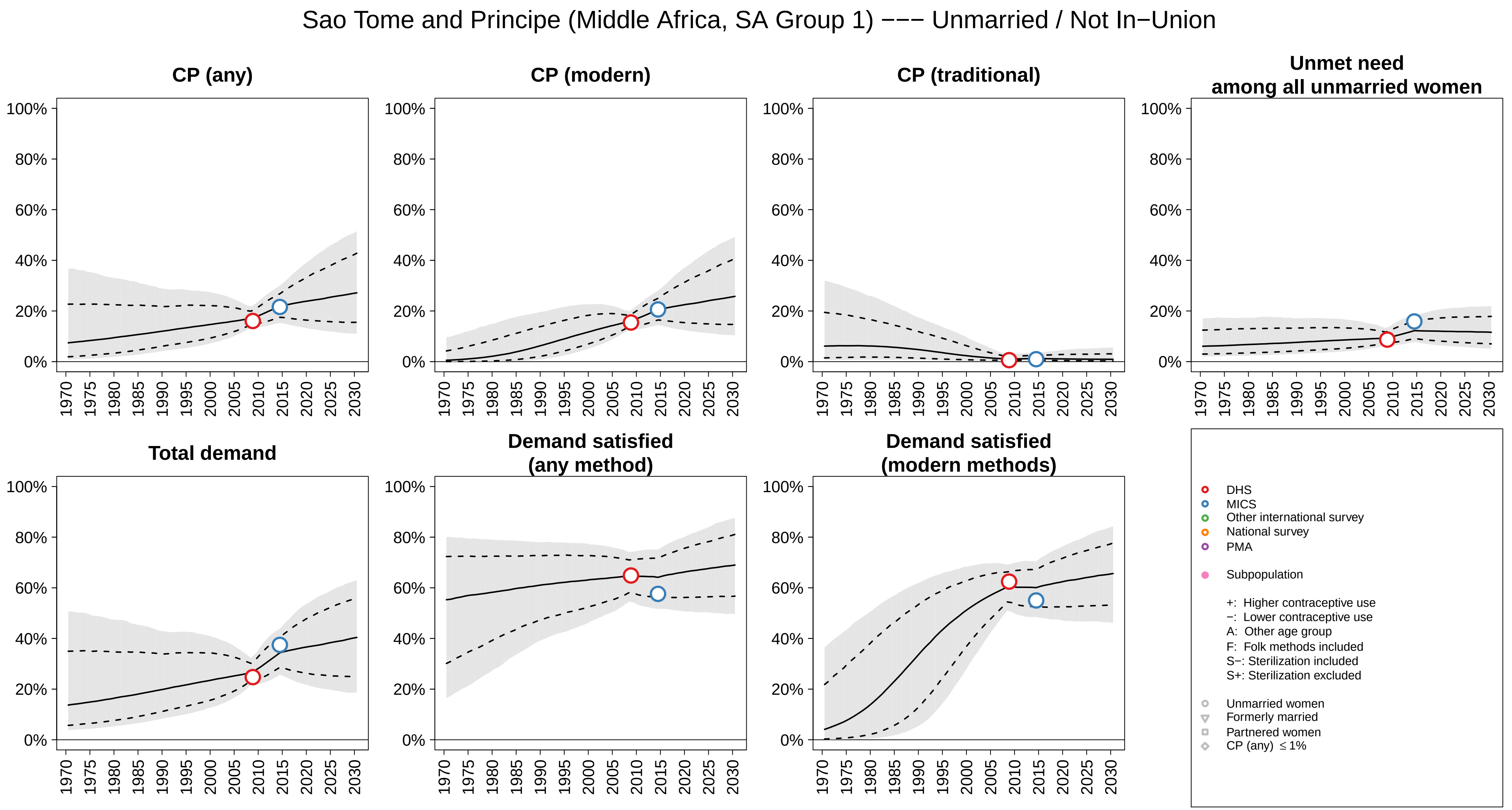
Sao Tome and Principe (Middle Africa, SA Group 1) −−− Unmarried / Not In−Union
CP (any)
CP (modern)
CP (traditional)
Unmet need among all unmarried women
Total demand
Demand satisfied (any method)
Demand satisfied (modern methods)
100%
80%
60%
40%
20%
0%
1970
1975
1980
1985
1990
1995
2000
2005
2010
2015
2020
2025
2030
DHS
MICS
Other international survey
National survey
PMA
Subpopulation
+: Higher contraceptive use
−: Lower contraceptive use
A: Other age group
F: Folk methods included
S−: Sterilization included
S+: Sterilization excluded
Unmarried women
Formerly married
Partnered women
CP (any) ≤ 1%

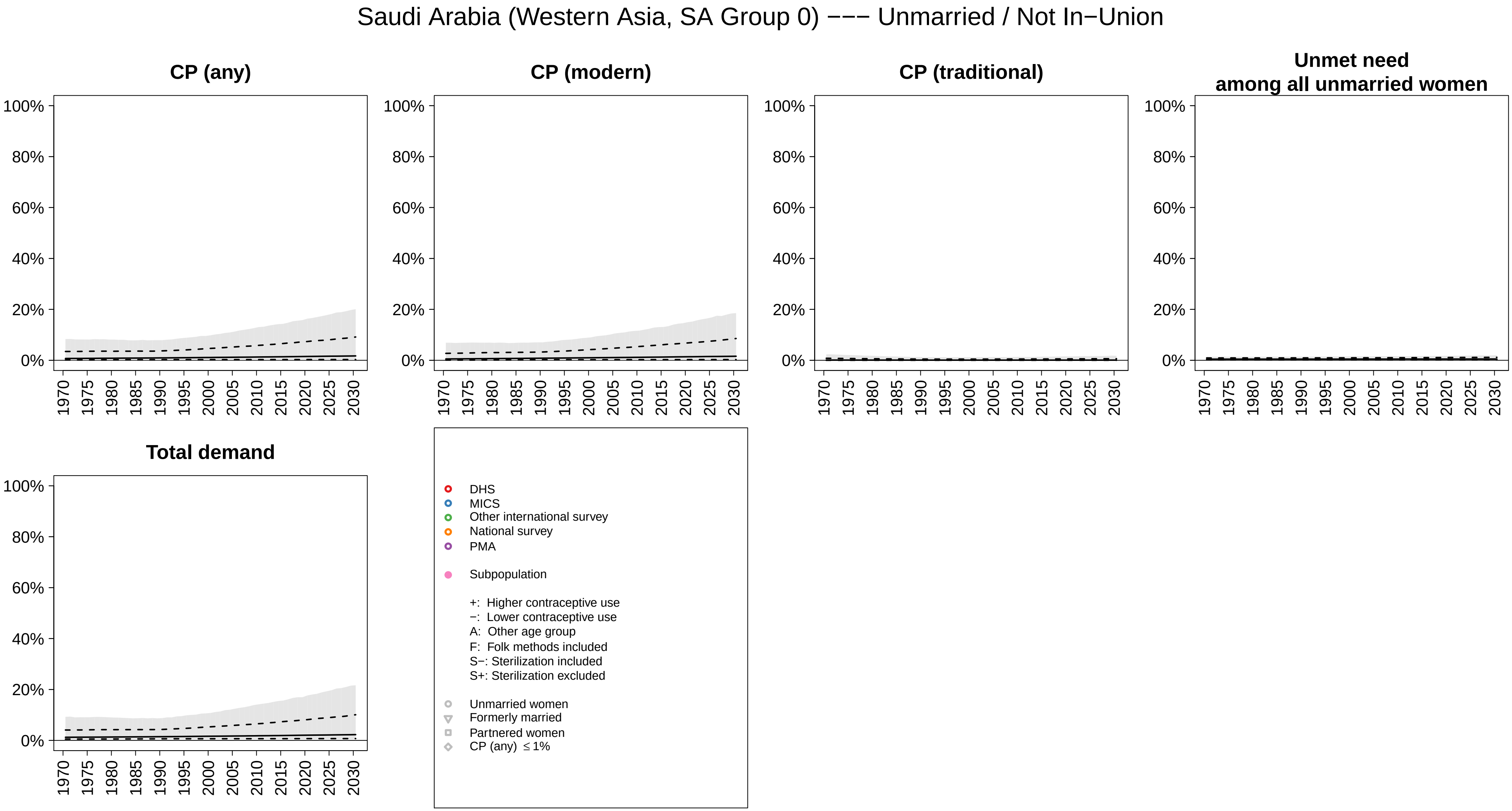
Saudi Arabia (Western Asia, SA Group 0) −−− Unmarried / Not In−Union
CP (any)
CP (modern)
CP (traditional)
Unmet need among all unmarried women
Total demand
100%
80%
60%
40%
20%
0%
1970 1975 1980 1985 1990 1995 2000 2005 2010 2015 2020 2025 2030
DHS
MICS
Other international survey
National survey
PMA
Subpopulation
+: Higher contraceptive use
−: Lower contraceptive use
A: Other age group
F: Folk methods included
S−: Sterilization included
S+: Sterilization excluded
Unmarried women
Formerly married
Partnered women
CP (any) ≤ 1%

Senegal (Western Africa, SA Group 1) −−− Unmarried / Not In−Union

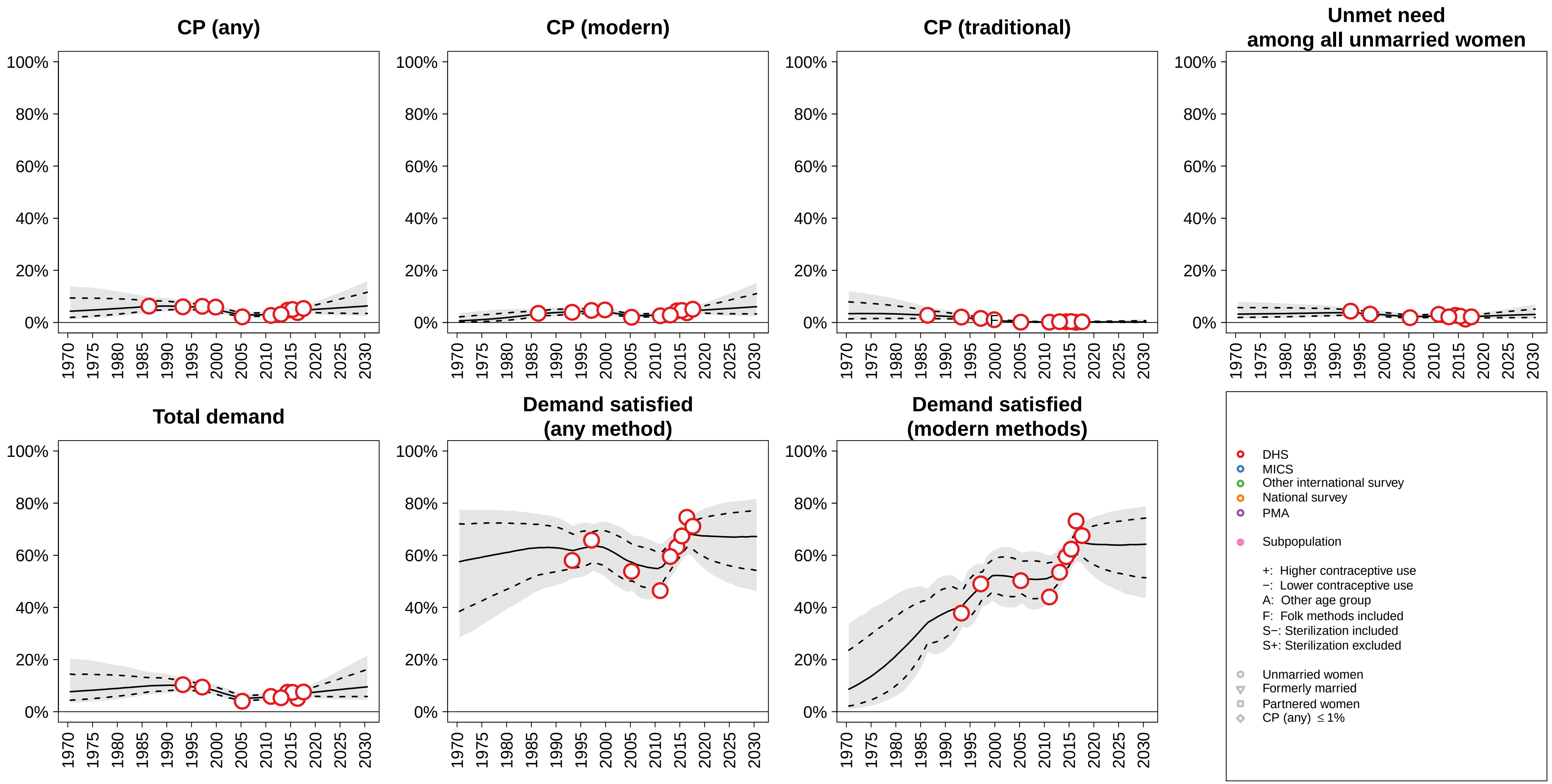

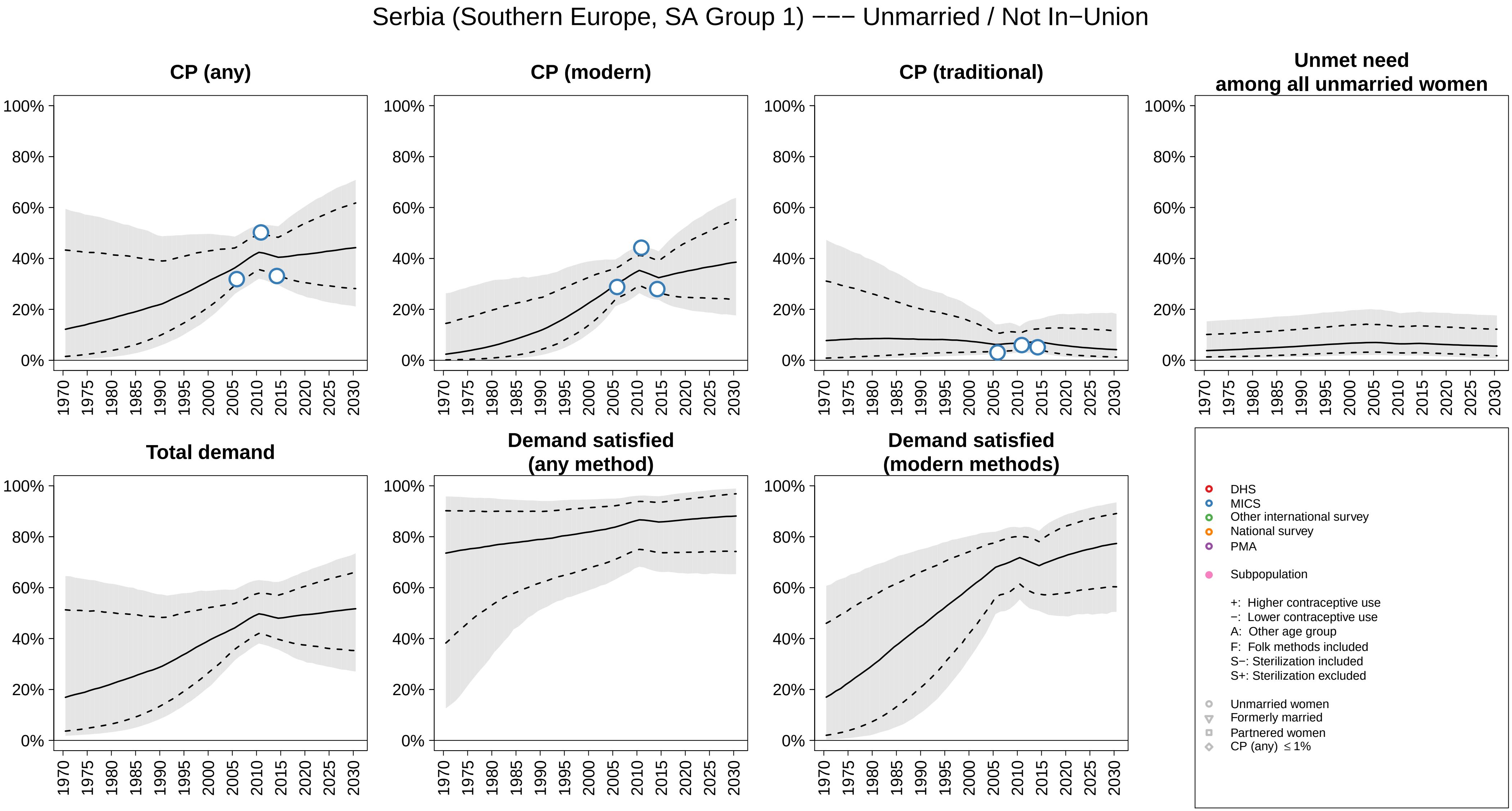
Serbia (Southern Europe, SA Group 1) −−− Unmarried / Not In−Union
CP (any)
CP (modern)
CP (traditional)
Unmet need among all unmarried women
Total demand
Demand satisfied (any method)
Demand satisfied (modern methods)
0%
20%
40%
60%
80%
100%
1970
1975
1980
1985
1990
1995
2000
2005
2010
2015
2020
2025
2030
DHS
MICS
Other international survey
National survey
PMA
Subpopulation
+: Higher contraceptive use
−: Lower contraceptive use
A: Other age group
F: Folk methods included
S−: Sterilization included
S+: Sterilization excluded
Unmarried women
Formerly married
Partnered women
CP (any) ≤ 1%

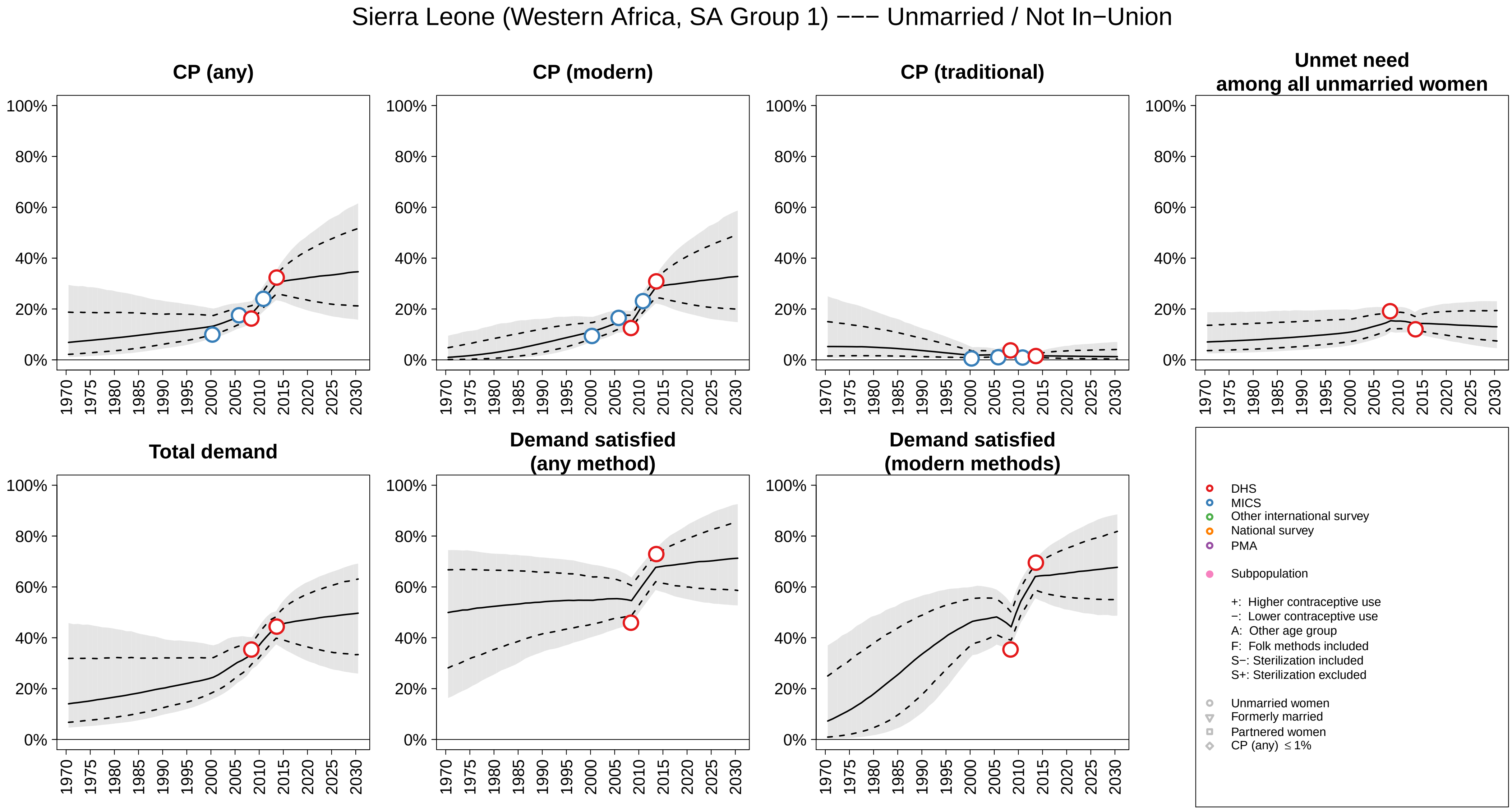
Sierra Leone (Western Africa, SA Group 1) −−− Unmarried / Not In−Union
CP (any)
CP (modern)
CP (traditional)
Unmet need among all unmarried women
Total demand
Demand satisfied (any method)
Demand satisfied (modern methods)
DHS
MICS
Other international survey
National survey
PMA
Subpopulation
+: Higher contraceptive use
−: Lower contraceptive use
A: Other age group
F: Folk methods included
S−: Sterilization included
S+: Sterilization excluded
Unmarried women
Formerly married
Partnered women
CP (any) ≤ 1%

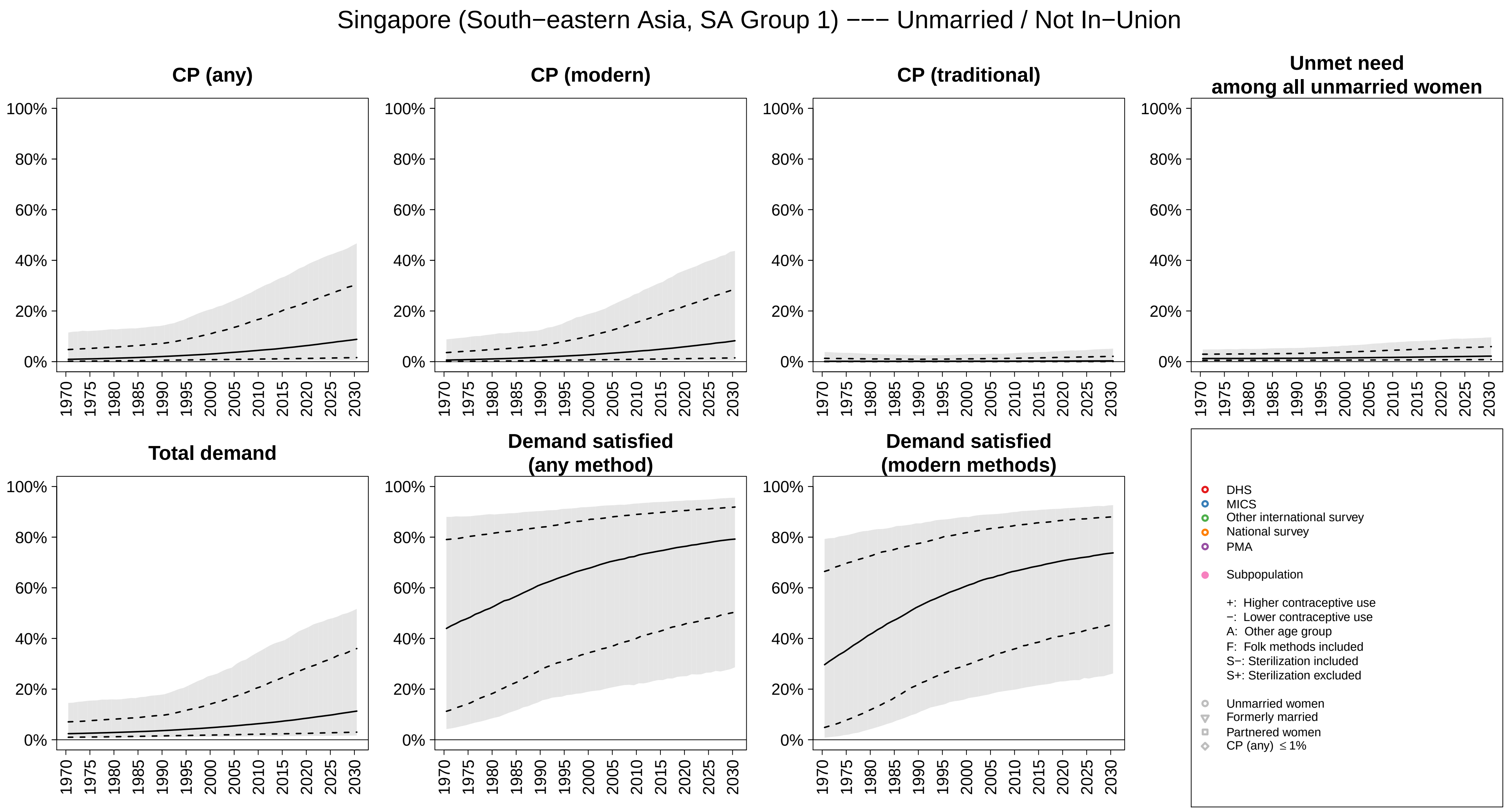
Singapore (South−eastern Asia, SA Group 1) −−− Unmarried / Not In−Union
CP (any)
CP (modern)
CP (traditional)
Unmet need among all unmarried women
Total demand
Demand satisfied (any method)
Demand satisfied (modern methods)
0%
20%
40%
60%
80%
100%
1970
1975
1980
1985
1990
1995
2000
2005
2010
2015
2020
2025
2030
DHS
MICS
Other international survey
National survey
PMA
Subpopulation
+: Higher contraceptive use
−: Lower contraceptive use
A: Other age group
F: Folk methods included
S−: Sterilization included
S+: Sterilization excluded
Unmarried women
Formerly married
Partnered women
CP (any) ≤ 1%

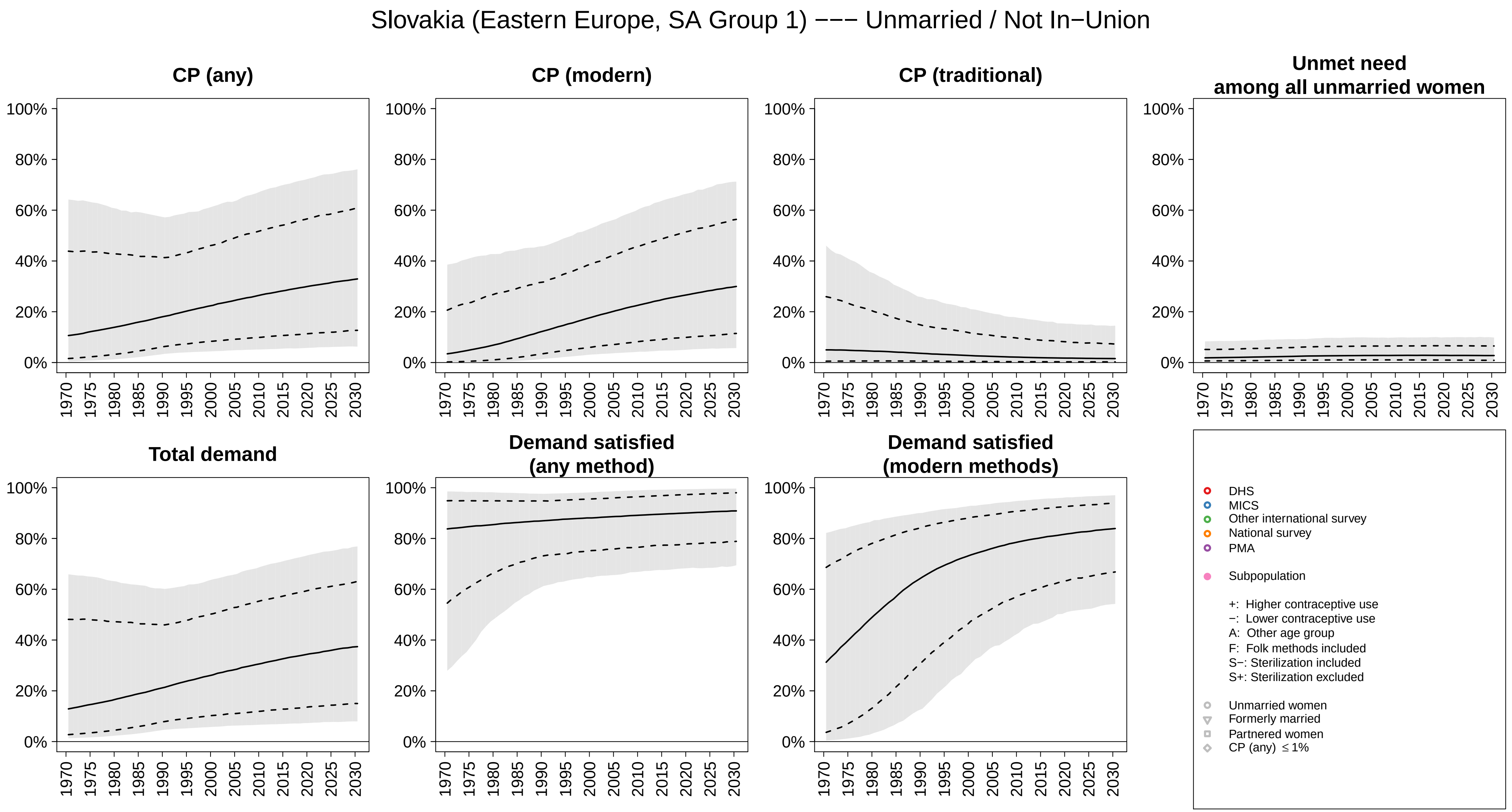
Slovakia (Eastern Europe, SA Group 1) −−− Unmarried / Not In−Union
CP (any)
CP (modern)
CP (traditional)
Unmet need among all unmarried women
Total demand
Demand satisfied (any method)
Demand satisfied (modern methods)
0%
20%
40%
60%
80%
100%
1970
1975
1980
1985
1990
1995
2000
2005
2010
2015
2020
2025
2030
DHS
MICS
Other international survey
National survey
PMA
Subpopulation
+: Higher contraceptive use
−: Lower contraceptive use
A: Other age group
F: Folk methods included
S−: Sterilization included
S+: Sterilization excluded
Unmarried women
Formerly married
Partnered women
CP (any) ≤ 1%

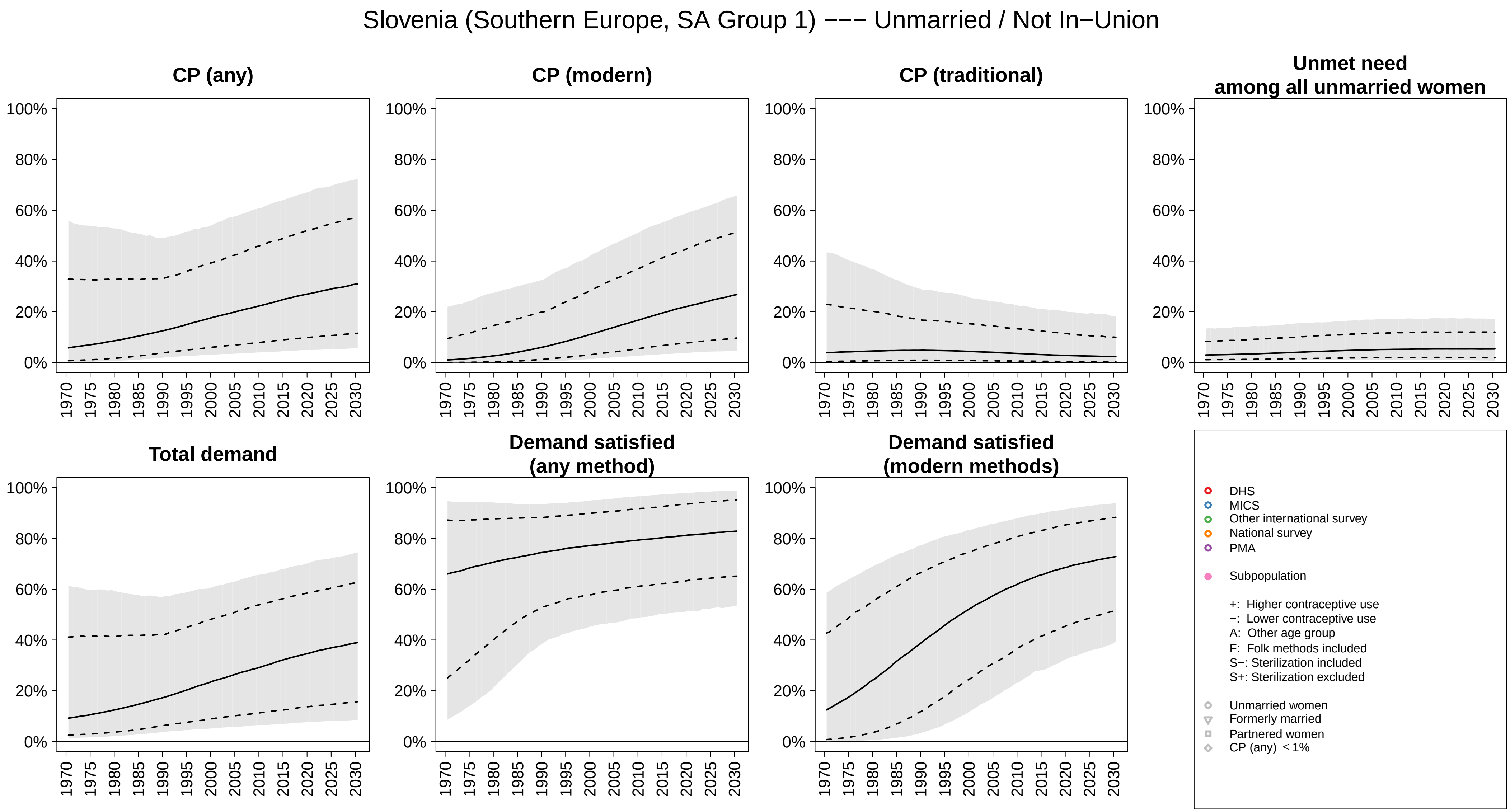
Slovenia (Southern Europe, SA Group 1) −−− Unmarried / Not In−Union
CP (any)
CP (modern)
CP (traditional)
Unmet need among all unmarried women
Total demand
Demand satisfied (any method)
Demand satisfied (modern methods)
DHS
MICS
Other international survey
National survey
PMA
Subpopulation
+: Higher contraceptive use
−: Lower contraceptive use
A: Other age group
F: Folk methods included
S−: Sterilization included
S+: Sterilization excluded
Unmarried women
Formerly married
Partnered women
CP (any) ≤ 1%

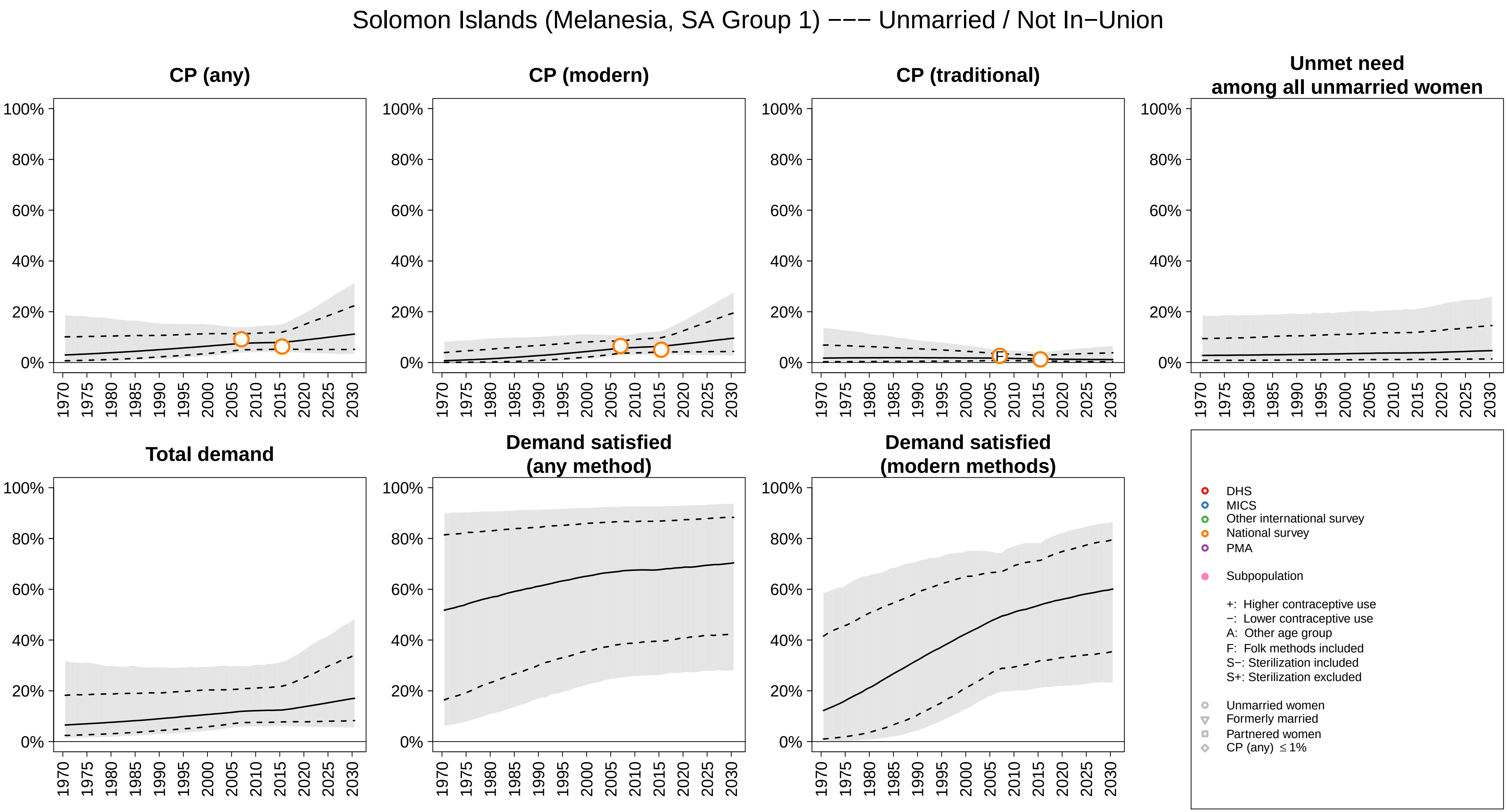
Solomon Islands (Melanesia, SA Group 1) --- Unmarried / Not In−Union
CP (any)
CP (modern)
CP (traditional)
Unmet need among all unmarried women
Total demand
Demand satisfied (any method)
Demand satisfied (modern methods)
0%
20%
40%
60%
80%
100%
1970
1975
1980
1985
1990
1995
2000
2005
2010
2015
2020
2025
2030
F
DHS
MICS
Other international survey
National survey
PMA
Subpopulation
+: Higher contraceptive use
−: Lower contraceptive use
A: Other age group
F: Folk methods included
S−: Sterilization included
S+: Sterilization excluded
Unmarried women
Formerly married
Partnered women
CP (any) ≤ 1%

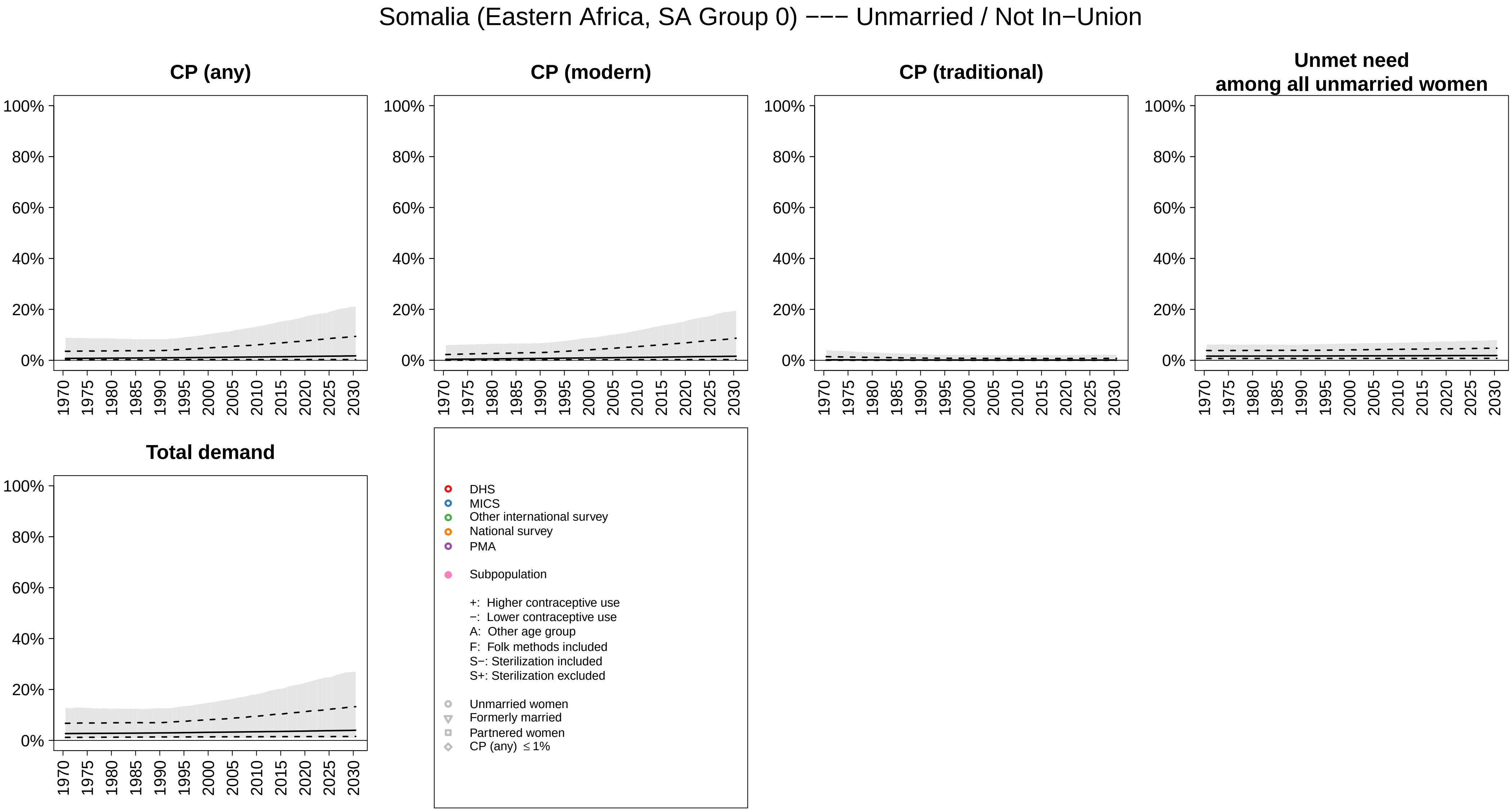
Somalia (Eastern Africa, SA Group 0) −−− Unmarried / Not In−Union
CP (any)
CP (modern)
CP (traditional)
Unmet need among all unmarried women
Total demand
100%
80%
60%
40%
20%
0%
1970
1975
1980
1985
1990
1995
2000
2005
2010
2015
2020
2025
2030
DHS
MICS
Other international survey
National survey
PMA
Subpopulation
+: Higher contraceptive use
−: Lower contraceptive use
A: Other age group
F: Folk methods included
S−: Sterilization included
S+: Sterilization excluded
Unmarried women
Formerly married
Partnered women
CP (any) ≤ 1%

South Africa (Southern Africa, SA Group 1) −−− Unmarried / Not In−Union

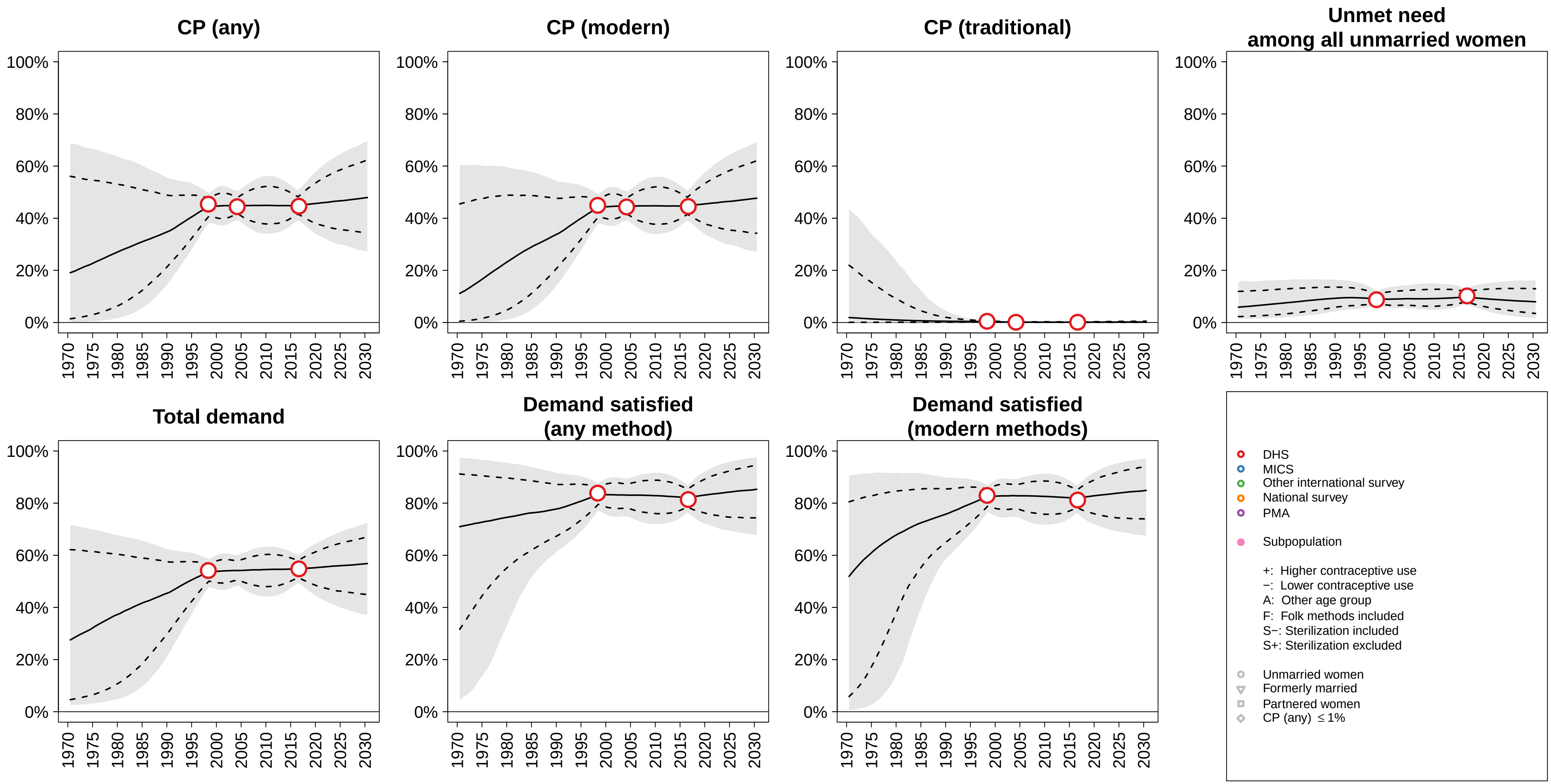

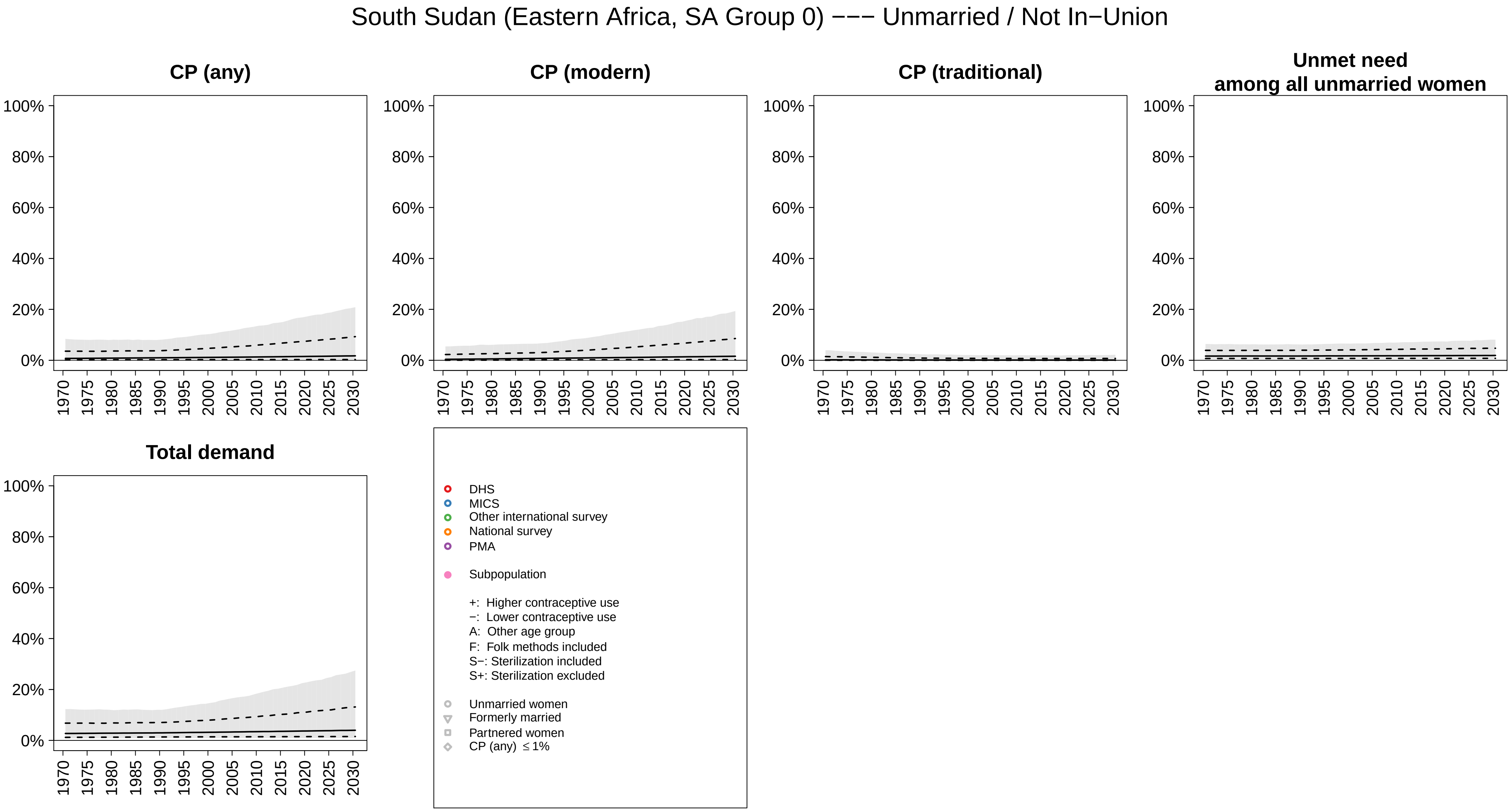
South Sudan (Eastern Africa, SA Group 0) −−− Unmarried / Not In−Union
CP (any)
CP (modern)
CP (traditional)
Unmet need among all unmarried women
Total demand
0%
20%
40%
60%
80%
100%
1970
1975
1980
1985
1990
1995
2000
2005
2010
2015
2020
2025
2030
DHS
MICS
Other international survey
National survey
PMA
Subpopulation
+: Higher contraceptive use
−: Lower contraceptive use
A: Other age group
F: Folk methods included
S−: Sterilization included
S+: Sterilization excluded
Unmarried women
Formerly married
Partnered women
CP (any) ≤ 1%

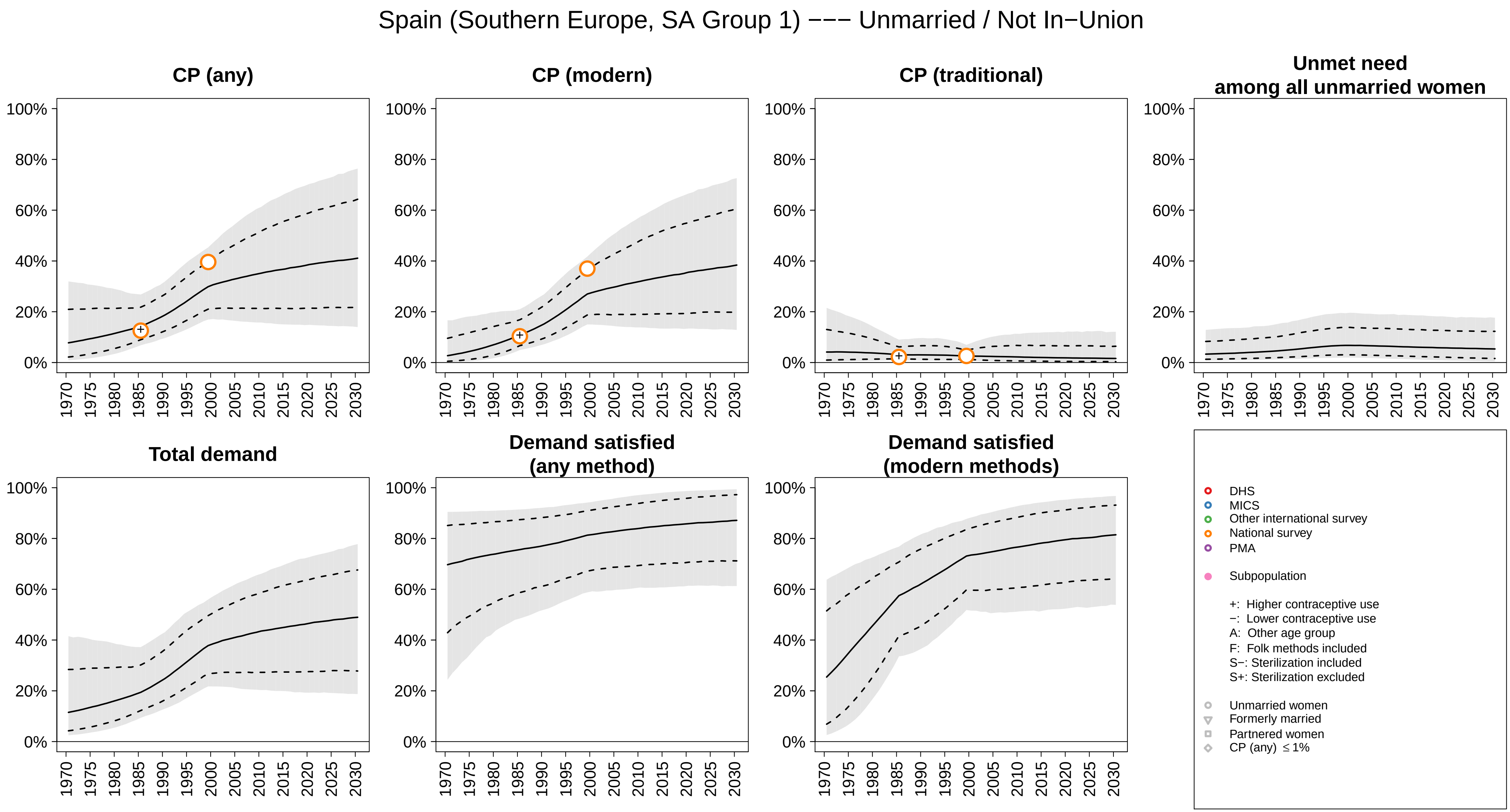
Spain (Southern Europe, SA Group 1) −−− Unmarried / Not In−Union
CP (any)
CP (modern)
CP (traditional)
Unmet need among all unmarried women
Total demand
Demand satisfied (any method)
Demand satisfied (modern methods)
100%
80%
60%
40%
20%
0%
1970
1975
1980
1985
1990
1995
2000
2005
2010
2015
2020
2025
2030
DHS
MICS
Other international survey
National survey
PMA
Subpopulation
+: Higher contraceptive use
−: Lower contraceptive use
A: Other age group
F: Folk methods included
S−: Sterilization included
S+: Sterilization excluded
Unmarried women
Formerly married
Partnered women
CP (any) ≤ 1%

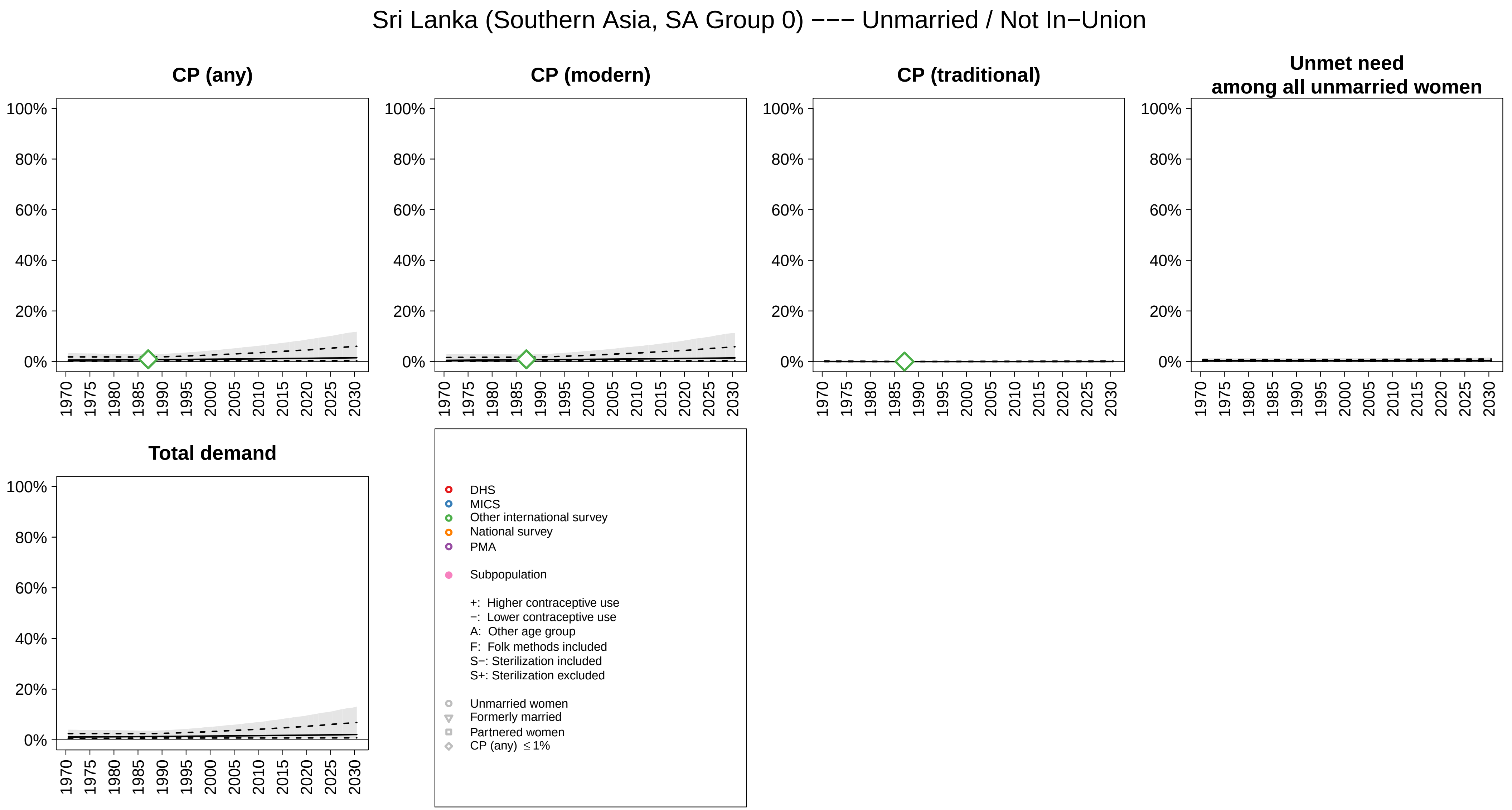
Sri Lanka (Southern Asia, SA Group 0) −−− Unmarried / Not In−Union
CP (any)
CP (modern)
CP (traditional)
Unmet need among all unmarried women
Total demand
100%
80%
60%
40%
20%
0%
1970 1975 1980 1985 1990 1995 2000 2005 2010 2015 2020 2025 2030
DHS
MICS
Other international survey
National survey
PMA
Subpopulation
+: Higher contraceptive use
−: Lower contraceptive use
A: Other age group
F: Folk methods included
S−: Sterilization included
S+: Sterilization excluded
Unmarried women
Formerly married
Partnered women
CP (any) ≤ 1%

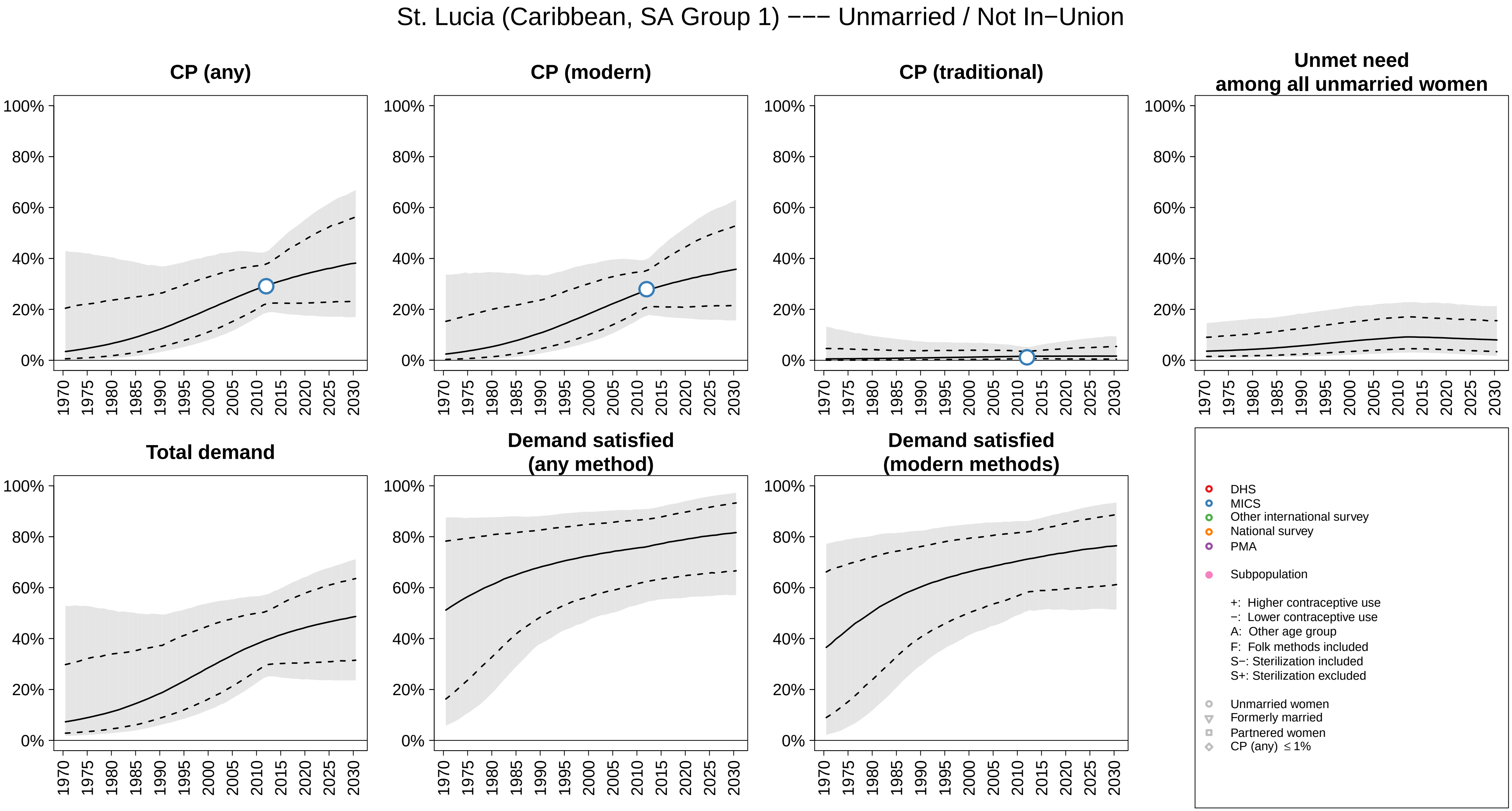
St. Lucia (Caribbean, SA Group 1) −−− Unmarried / Not In−Union
CP (any)
CP (modern)
CP (traditional)
Unmet need among all unmarried women
Total demand
Demand satisfied (any method)
Demand satisfied (modern methods)
100%
80%
60%
40%
20%
0%
1970 1975 1980 1985 1990 1995 2000 2005 2010 2015 2020 2025 2030
DHS
MICS
Other international survey
National survey
PMA
Subpopulation
+: Higher contraceptive use
−: Lower contraceptive use
A: Other age group
F: Folk methods included
S−: Sterilization included
S+: Sterilization excluded
Unmarried women
Formerly married
Partnered women
CP (any) ≤ 1%

St. Vincent and the Grenadines (Caribbean, SA Group 1) −−− Unmarried / Not In−Union

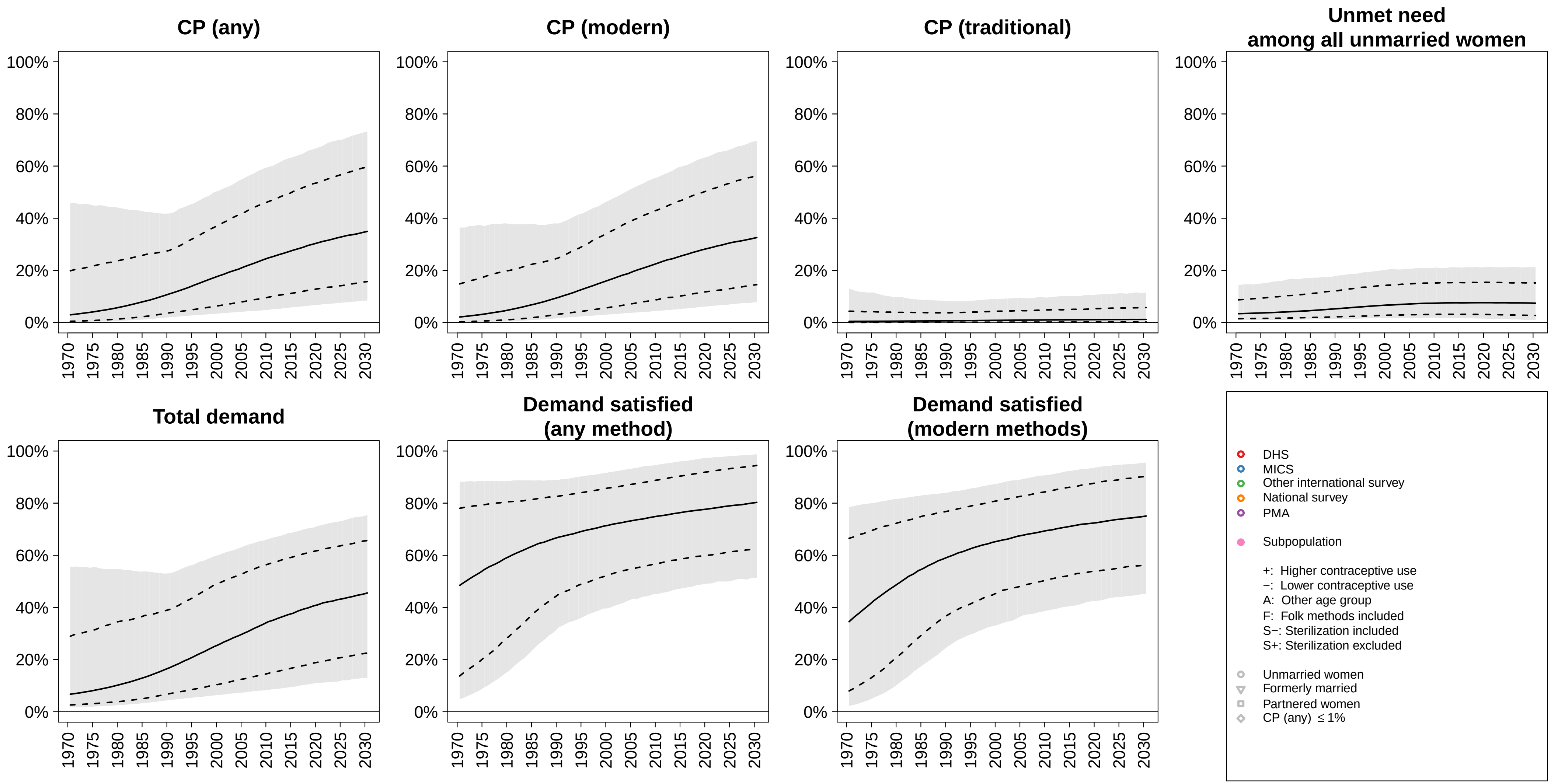

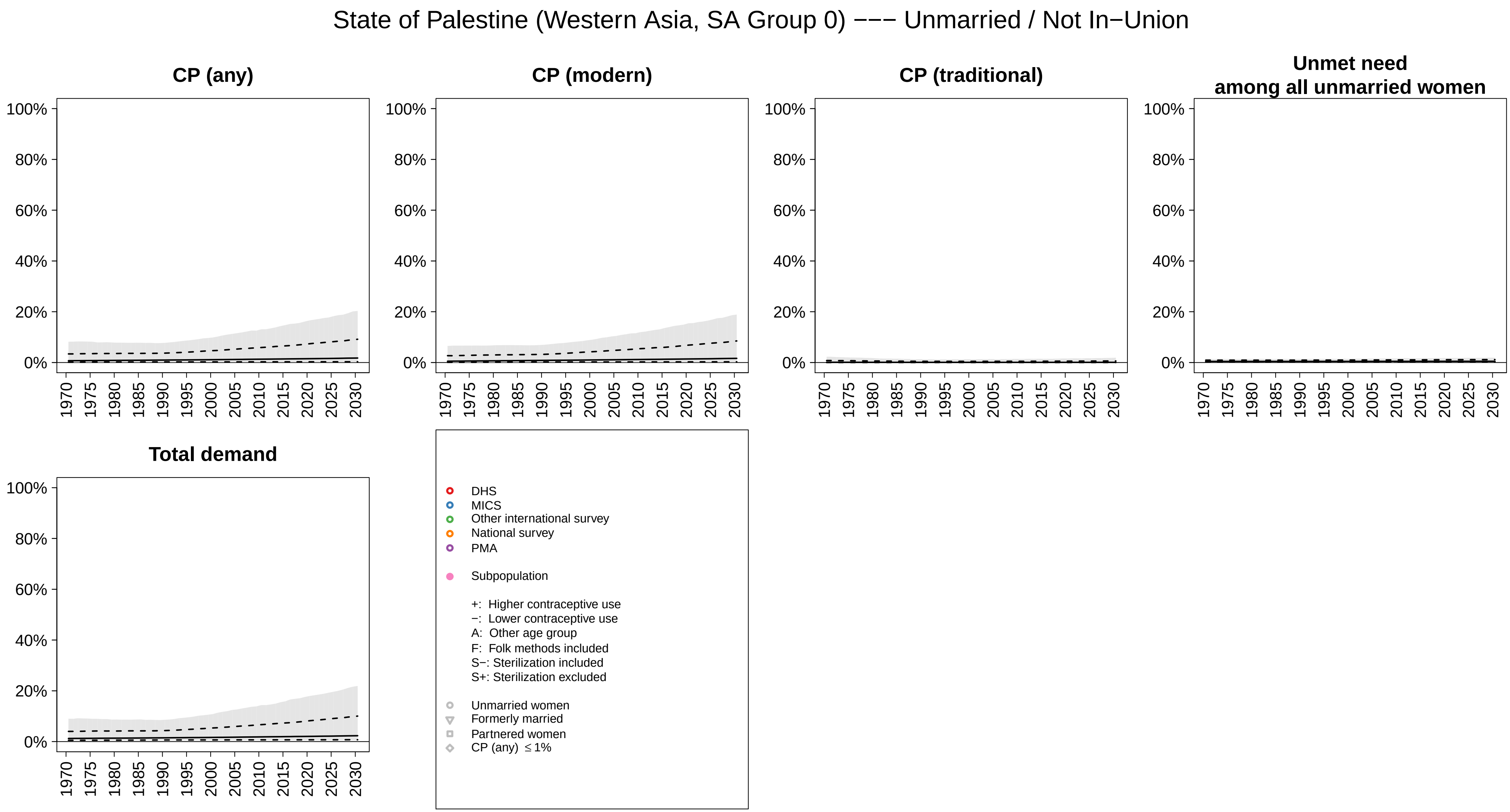
State of Palestine (Western Asia, SA Group 0) −−− Unmarried / Not In−Union
CP (any)
CP (modern)
CP (traditional)
Unmet need among all unmarried women
Total demand
100%
80%
60%
40%
20%
0%
1970
1975
1980
1985
1990
1995
2000
2005
2010
2015
2020
2025
2030
DHS
MICS
Other international survey
National survey
PMA
Subpopulation
+: Higher contraceptive use
−: Lower contraceptive use
A: Other age group
F: Folk methods included
S−: Sterilization included
S+: Sterilization excluded
Unmarried women
Formerly married
Partnered women
CP (any) ≤ 1%

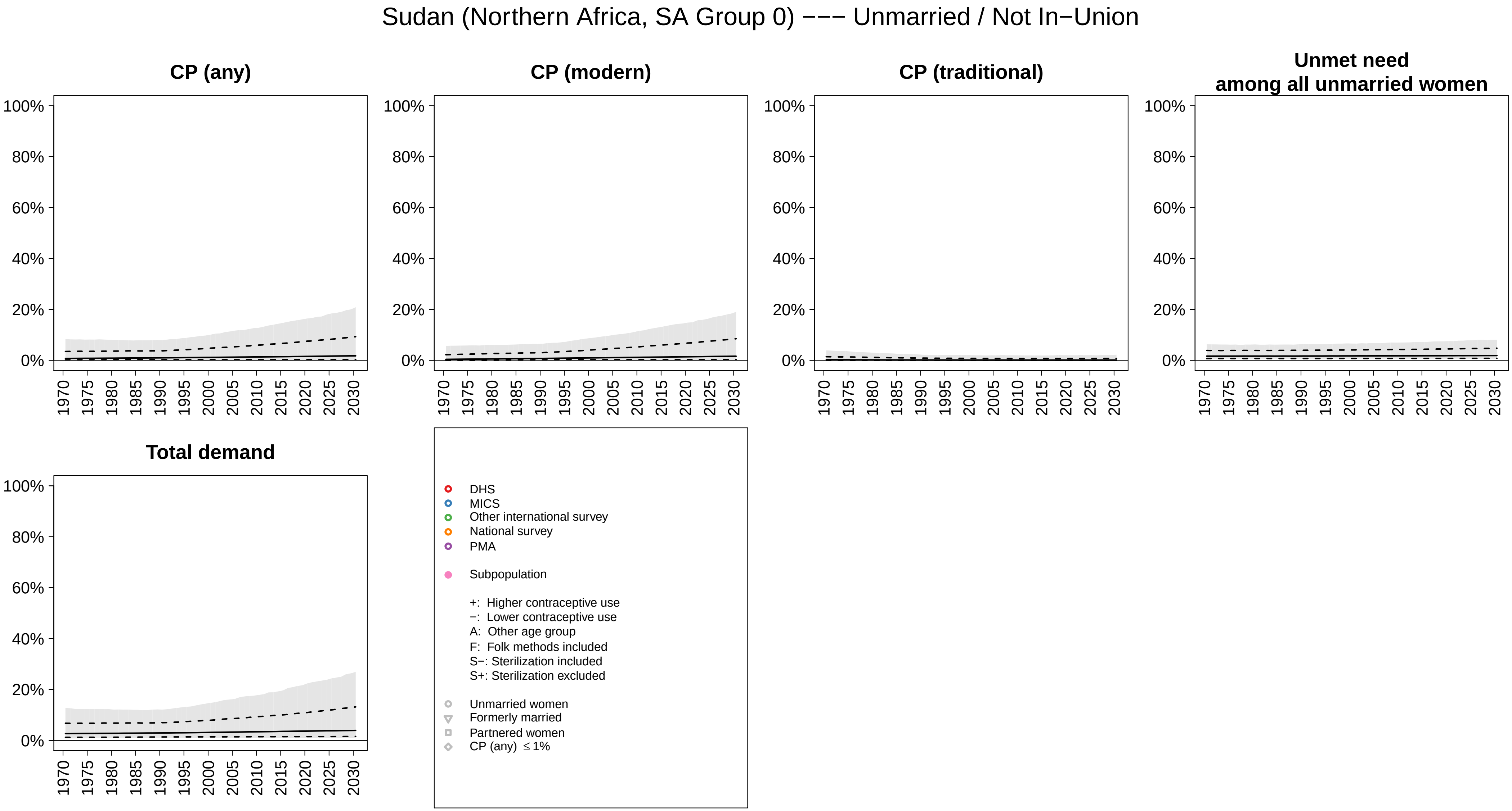
Sudan (Northern Africa, SA Group 0) −−− Unmarried / Not In−Union
CP (any)
CP (modern)
CP (traditional)
Unmet need among all unmarried women
Total demand
0%
20%
40%
60%
80%
100%
1970
1975
1980
1985
1990
1995
2000
2005
2010
2015
2020
2025
2030
DHS
MICS
Other international survey
National survey
PMA
Subpopulation
+: Higher contraceptive use
−: Lower contraceptive use
A: Other age group
F: Folk methods included
S−: Sterilization included
S+: Sterilization excluded
Unmarried women
Formerly married
Partnered women
CP (any) ≤ 1%

Suriname (South America, SA Group 1) −−− Unmarried / Not In−Union

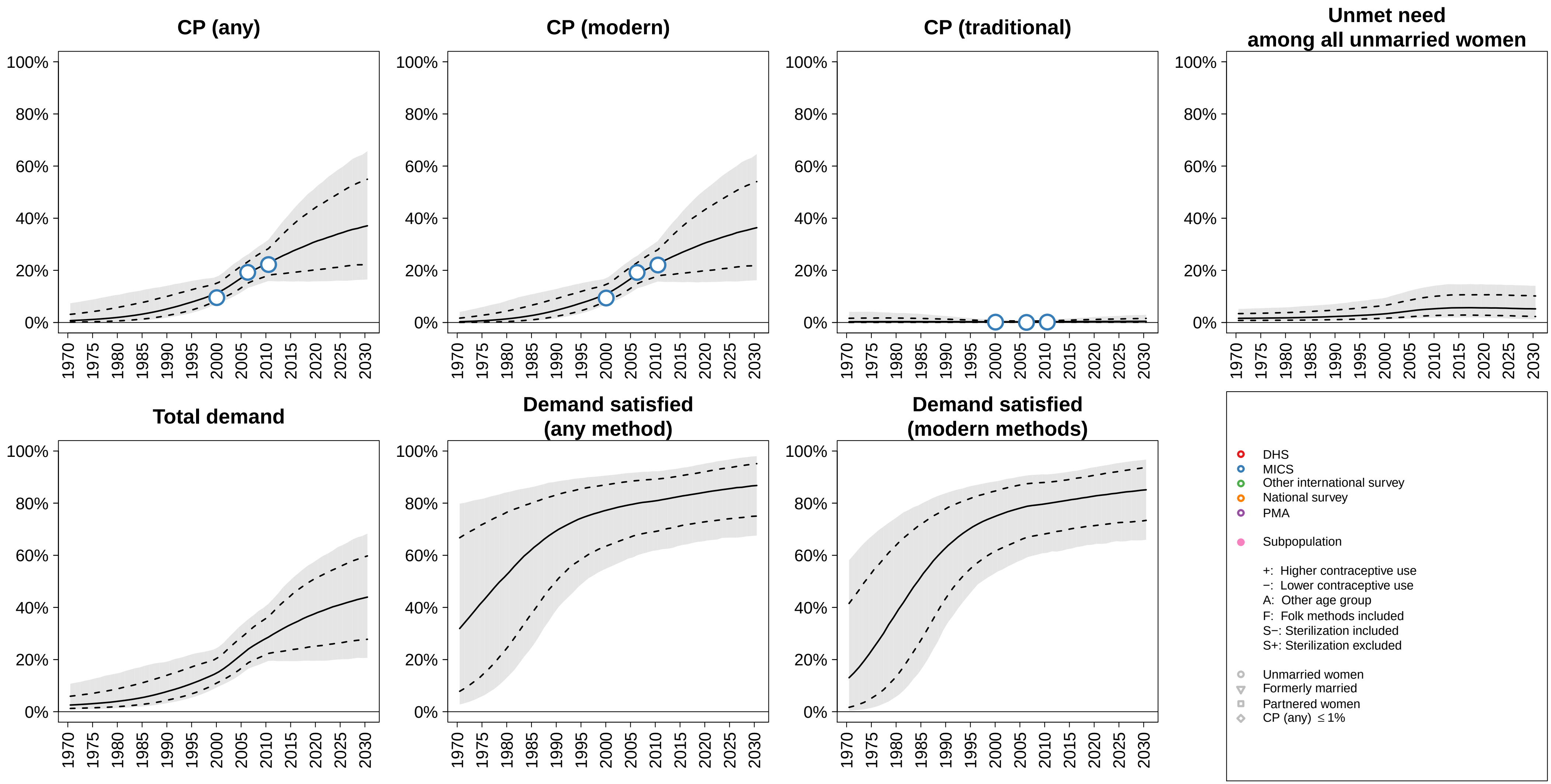

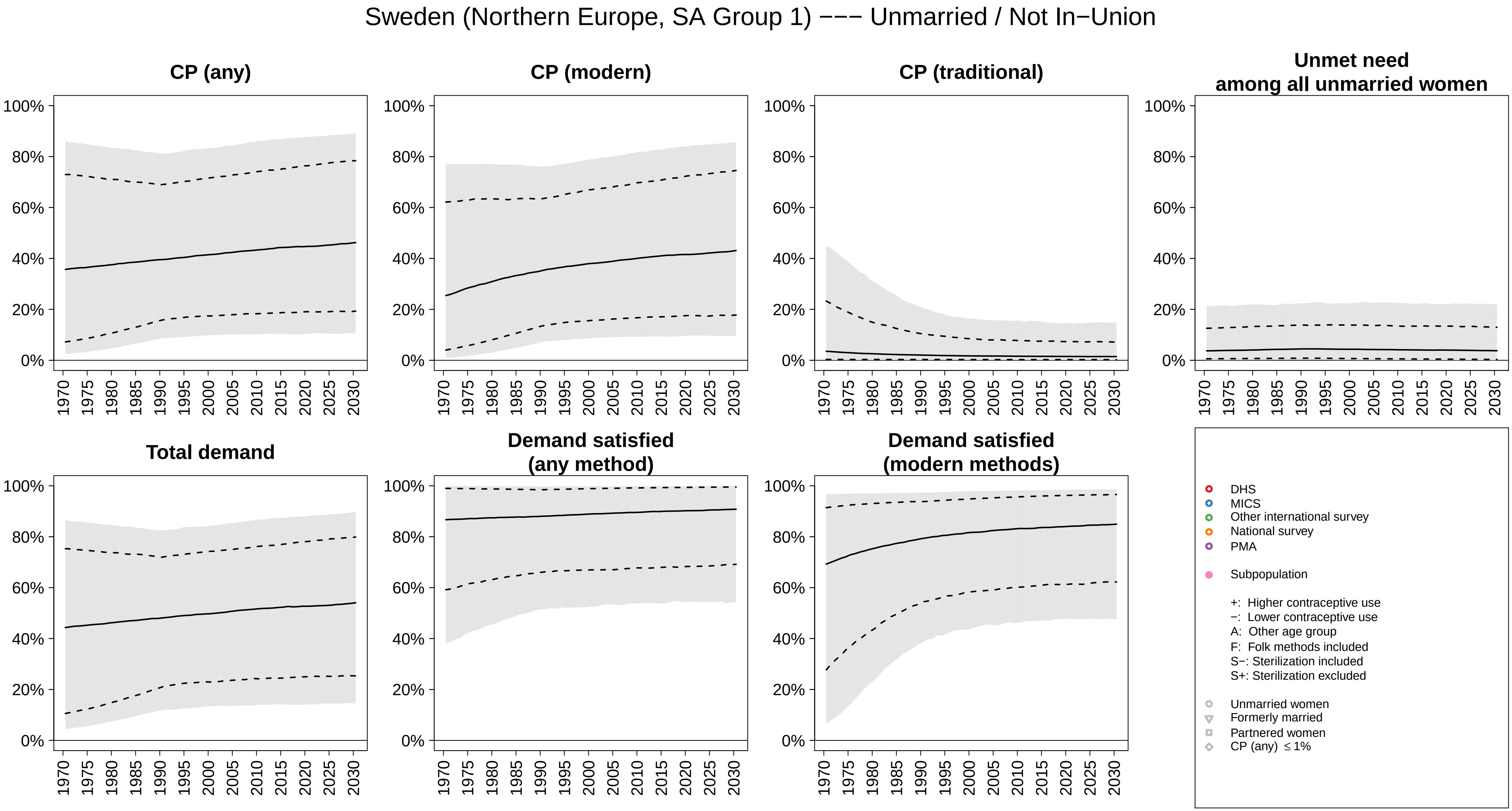
Sweden (Northern Europe, SA Group 1) −−− Unmarried / Not In−Union
CP (any)
CP (modern)
CP (traditional)
Unmet need among all unmarried women
Total demand
Demand satisfied (any method)
Demand satisfied (modern methods)
0%
20%
40%
60%
80%
100%
1970
1975
1980
1985
1990
1995
2000
2005
2010
2015
2020
2025
2030
DHS
MICS
Other international survey
National survey
PMA
Subpopulation
+: Higher contraceptive use
−: Lower contraceptive use
A: Other age group
F: Folk methods included
S−: Sterilization included
S+: Sterilization excluded
Unmarried women
Formerly married
Partnered women
CP (any) ≤ 1%

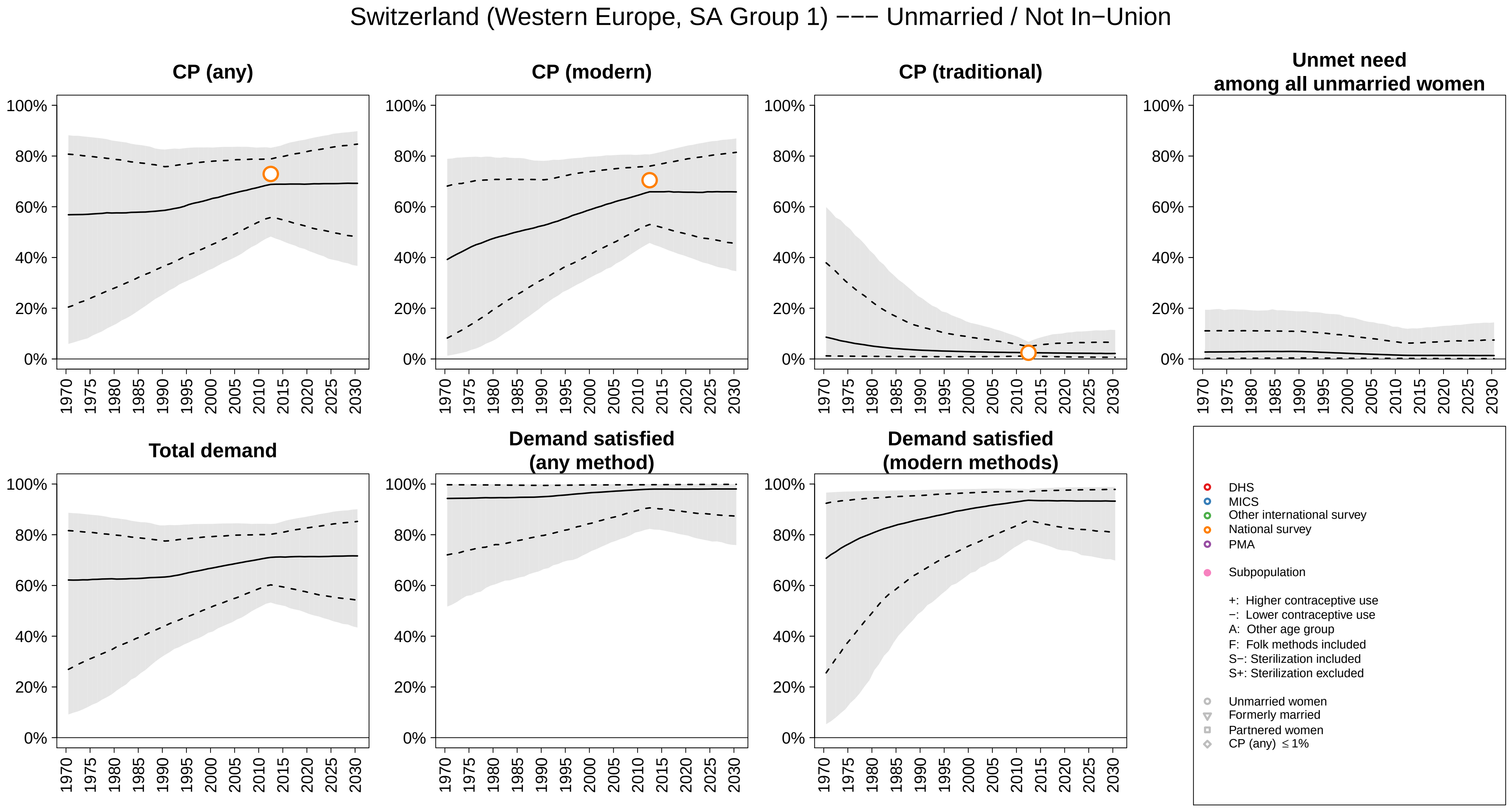
Switzerland (Western Europe, SA Group 1) −−− Unmarried / Not In−Union
CP (any)
CP (modern)
CP (traditional)
Unmet need among all unmarried women
Total demand
Demand satisfied (any method)
Demand satisfied (modern methods)
0%
20%
40%
60%
80%
100%
1970
1975
1980
1985
1990
1995
2000
2005
2010
2015
2020
2025
2030
DHS
MICS
Other international survey
National survey
PMA
Subpopulation
+: Higher contraceptive use
−: Lower contraceptive use
A: Other age group
F: Folk methods included
S−: Sterilization included
S+: Sterilization excluded
Unmarried women
Formerly married
Partnered women
CP (any) ≤ 1%

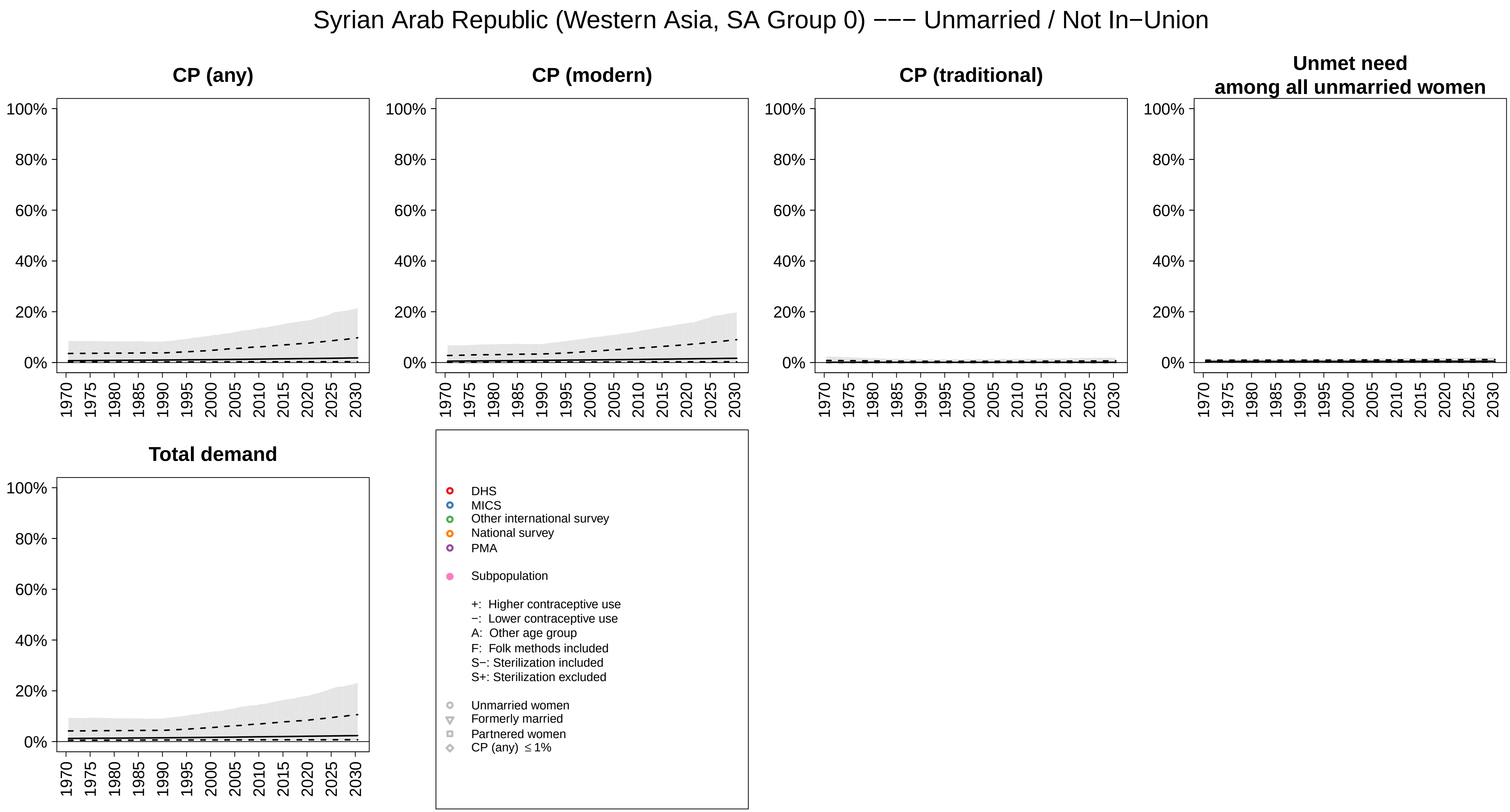
Syrian Arab Republic (Western Asia, SA Group 0) --- Unmarried / Not In-Union
CP (any)
CP (modern)
CP (traditional)
Unmet need among all unmarried women
Total demand
100%
80%
60%
40%
20%
0%
1970 1975 1980 1985 1990 1995 2000 2005 2010 2015 2020 2025 2030
DHS
MICS
Other international survey
National survey
PMA
Subpopulation
+: Higher contraceptive use
−: Lower contraceptive use
A: Other age group
F: Folk methods included
S−: Sterilization included
S+: Sterilization excluded
Unmarried women
Formerly married
Partnered women
CP (any) ≤ 1%

Tajikistan (Central Asia, SA Group 0) −−− Unmarried / Not In−Union

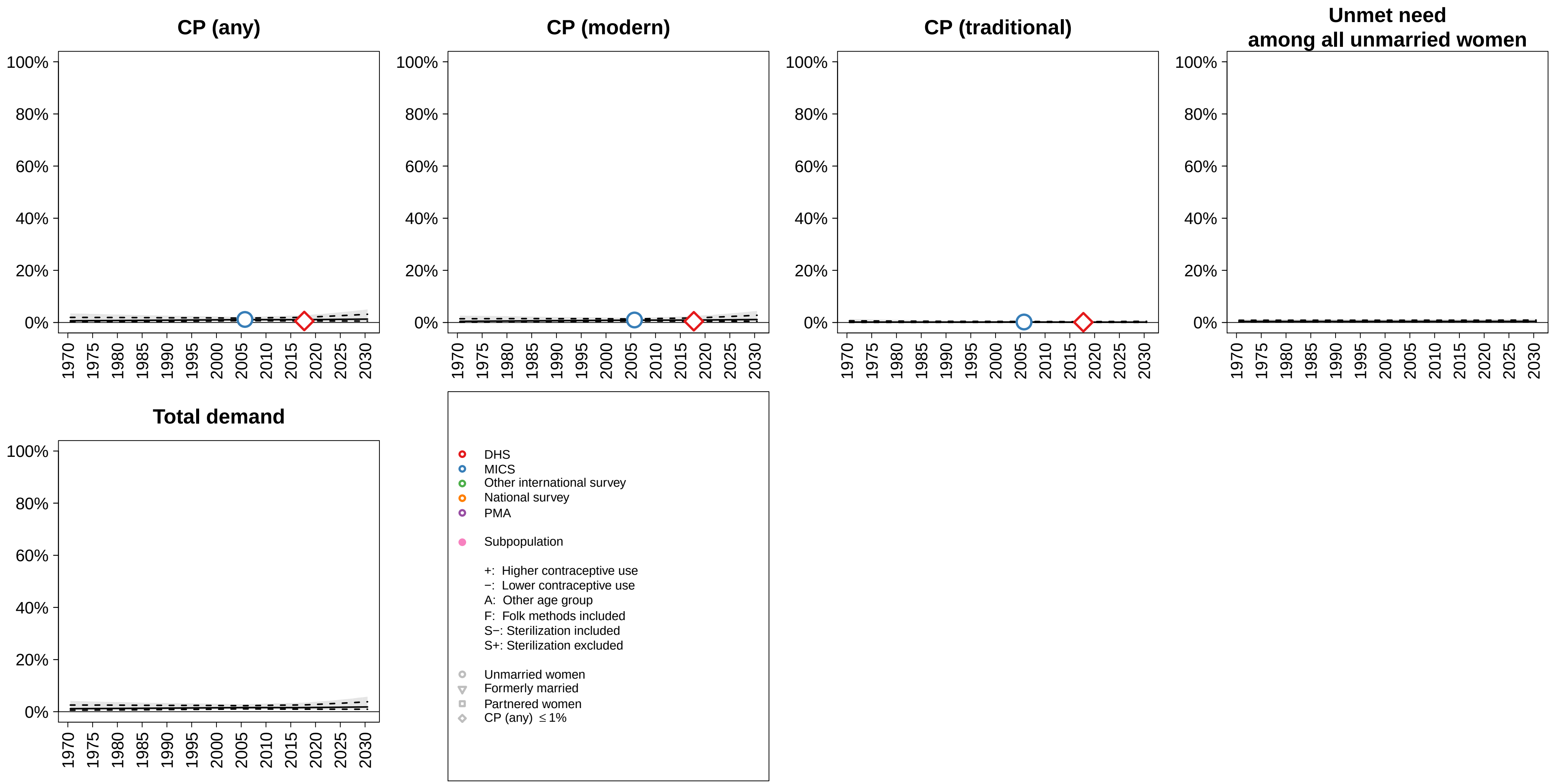

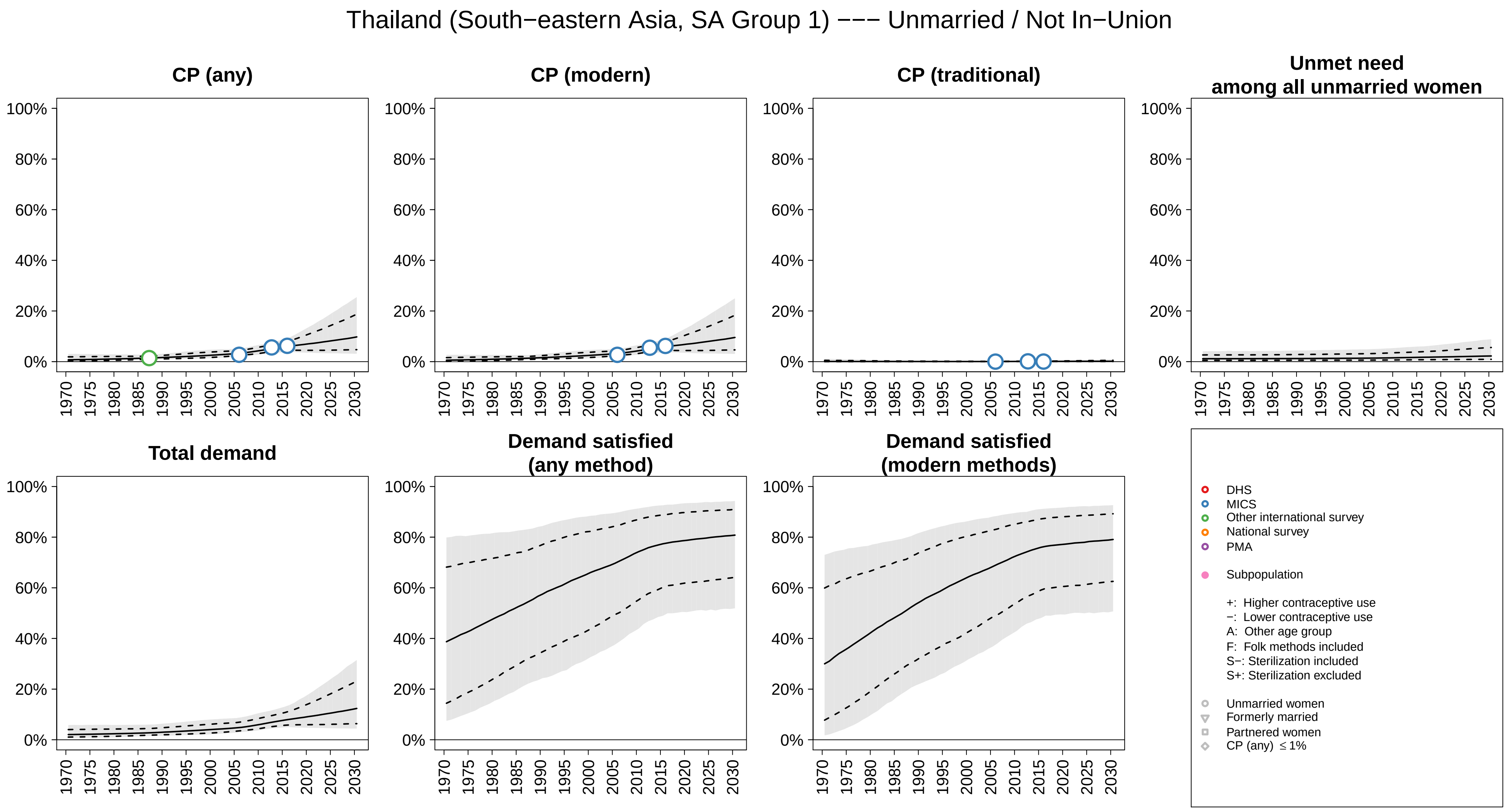
Thailand (South−eastern Asia, SA Group 1) −−− Unmarried / Not In−Union
CP (any)
CP (modern)
CP (traditional)
Unmet need among all unmarried women
Total demand
Demand satisfied (any method)
Demand satisfied (modern methods)
100%
80%
60%
40%
20%
0%
1970
1975
1980
1985
1990
1995
2000
2005
2010
2015
2020
2025
2030
DHS
MICS
Other international survey
National survey
PMA
Subpopulation
+: Higher contraceptive use
−: Lower contraceptive use
A: Other age group
F: Folk methods included
S−: Sterilization included
S+: Sterilization excluded
Unmarried women
Formerly married
Partnered women
CP (any) ≤ 1%

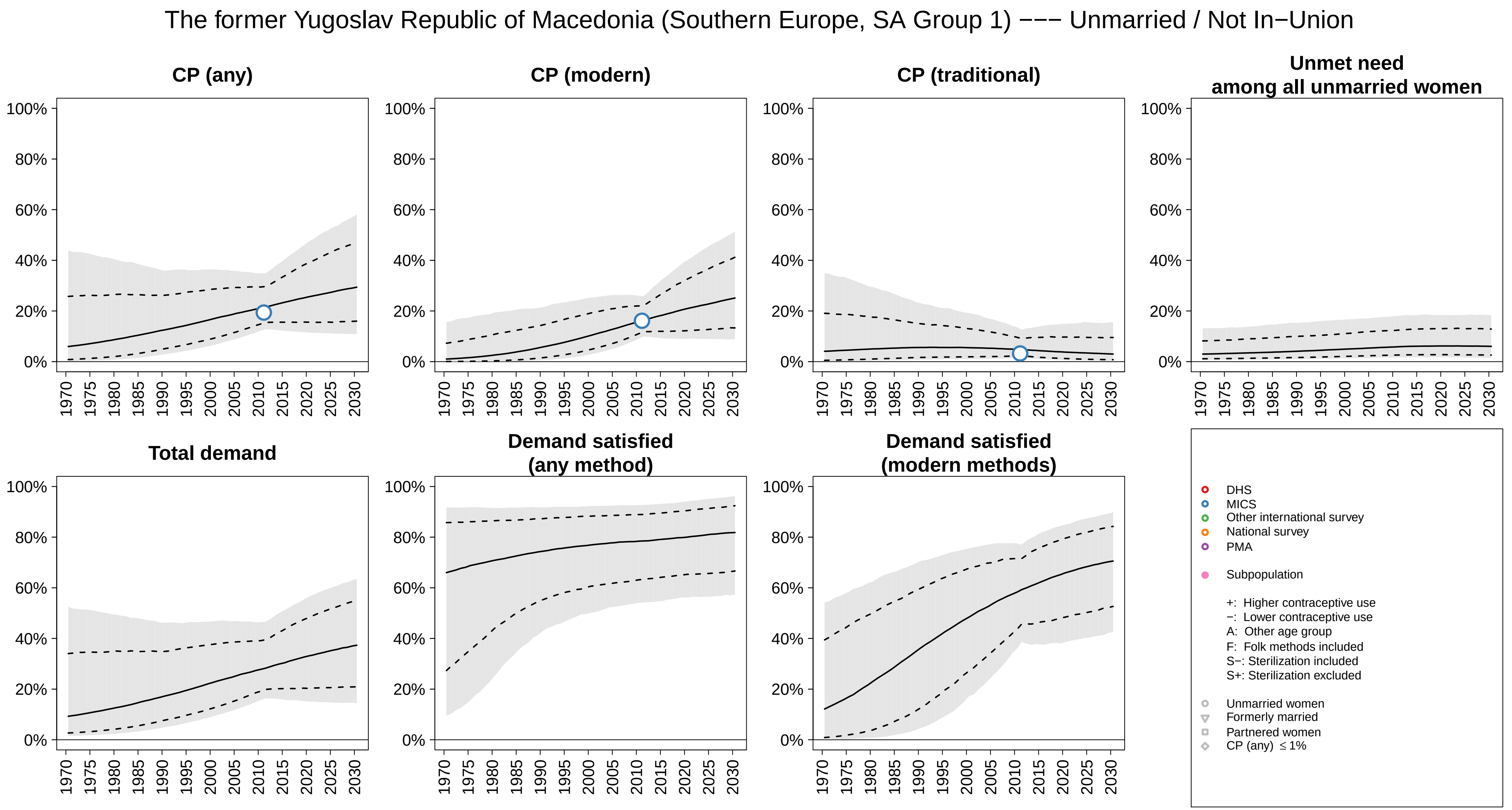
The former Yugoslav Republic of Macedonia (Southern Europe, SA Group 1) −−− Unmarried / Not In−Union
CP (any)
CP (modern)
CP (traditional)
Unmet need
among all unmarried women
Total demand
Demand satisfied
(any method)
Demand satisfied
(modern methods)
100%
80%
60%
40%
20%
0%
1970
1975
1980
1985
1990
1995
2000
2005
2010
2015
2020
2025
2030
DHS
MICS
Other international survey
National survey
PMA
Subpopulation
+: Higher contraceptive use
−: Lower contraceptive use
A: Other age group
F: Folk methods included
S−: Sterilization included
S+: Sterilization excluded
Unmarried women
Formerly married
Partnered women
CP (any) ≤ 1%

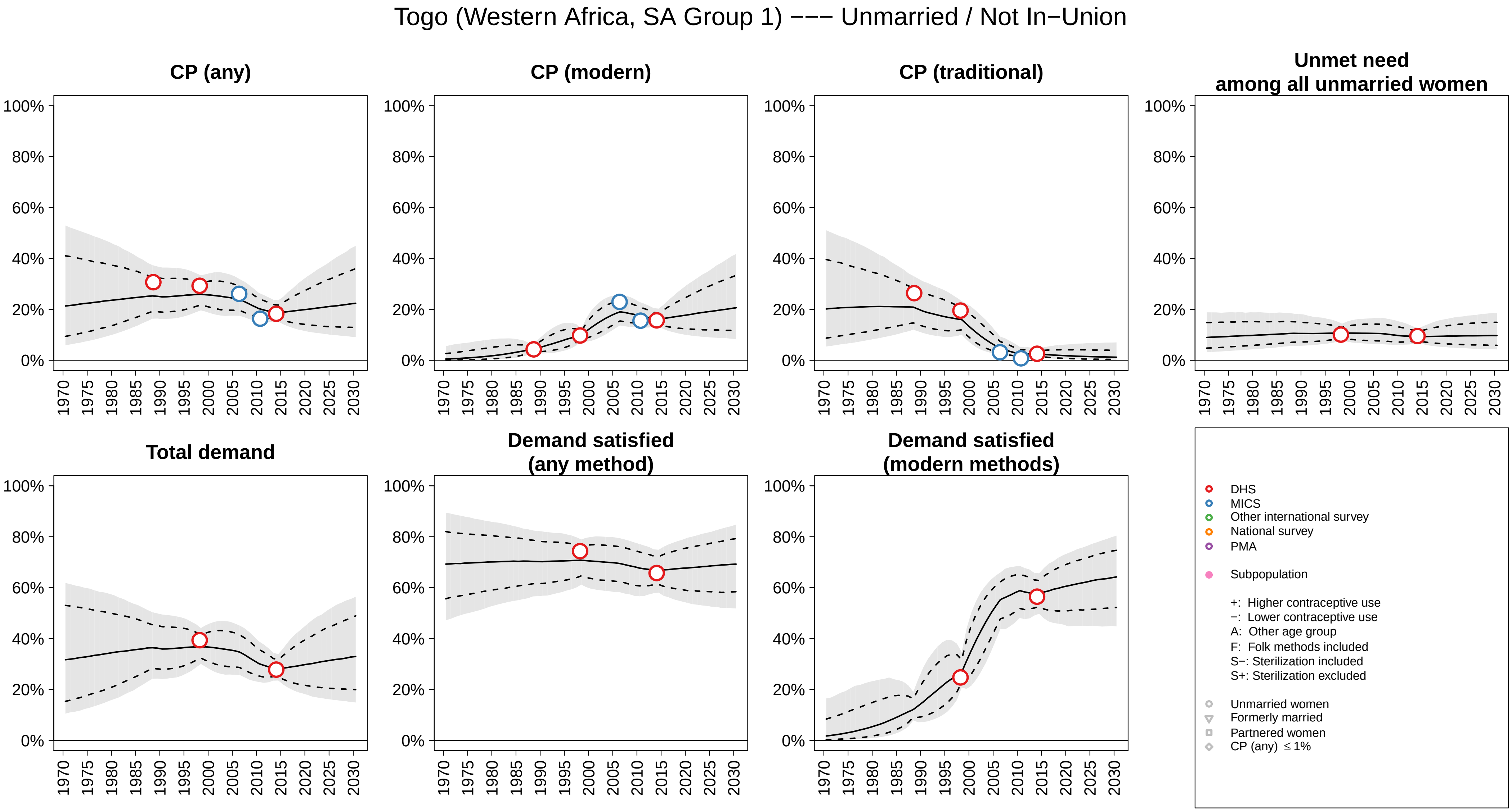

Togo (Western Africa, SA Group 1) −−− Unmarried / Not In−Union
CP (any)
CP (modern)
CP (traditional)
Unmet need among all unmarried women
Total demand
Demand satisfied (any method)
Demand satisfied (modern methods)
100%
80%
60%
40%
20%
0%
1970 1975 1980 1985 1990 1995 2000 2005 2010 2015 2020 2025 2030
DHS
MICS
Other international survey
National survey
PMA
Subpopulation
+: Higher contraceptive use
−: Lower contraceptive use
A: Other age group
F: Folk methods included
S−: Sterilization included
S+: Sterilization excluded
Unmarried women
Formerly married
Partnered women
CP (any) ≤ 1%

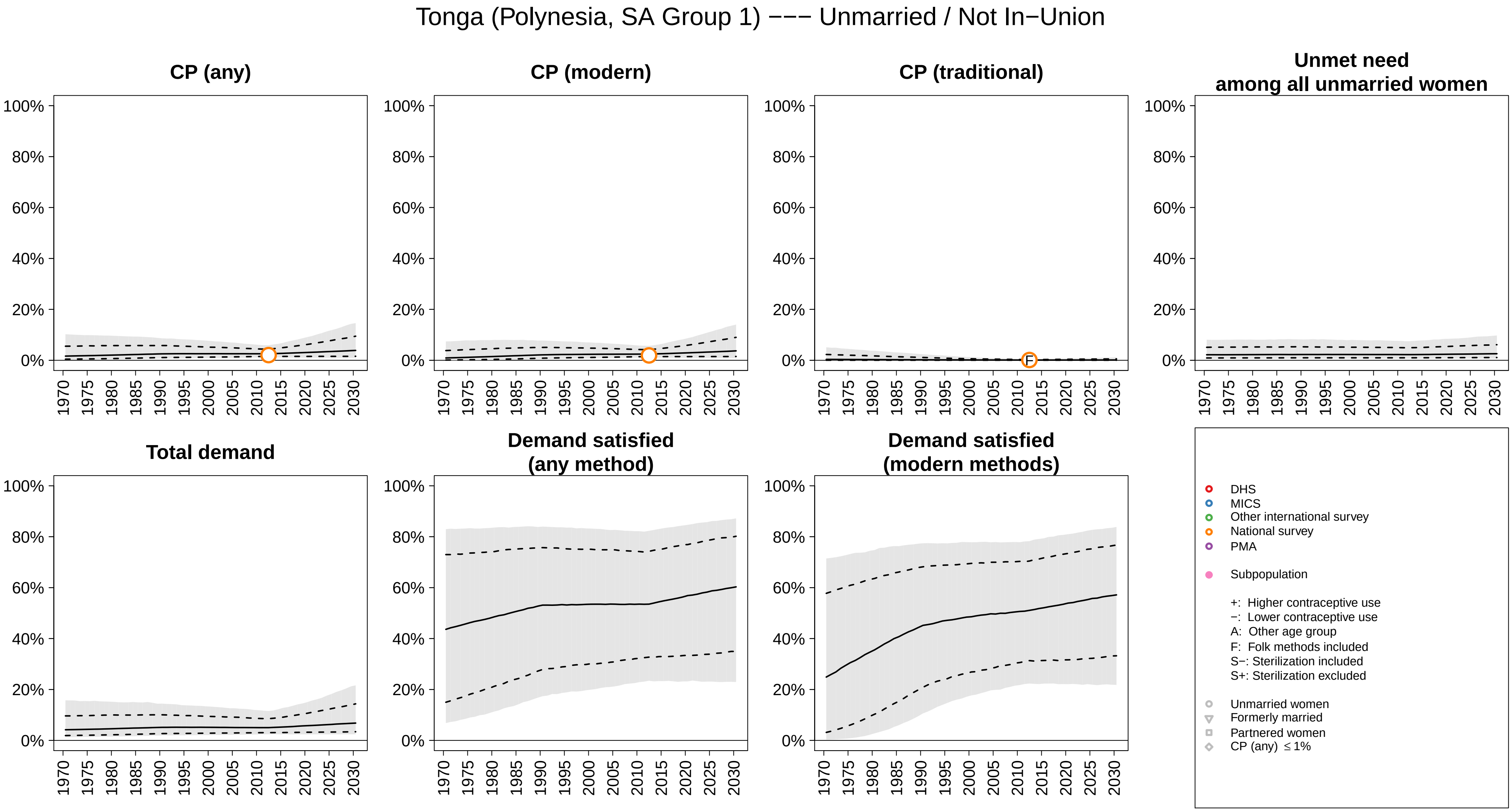
Tonga (Polynesia, SA Group 1) −−− Unmarried / Not In−Union
CP (any)
CP (modern)
CP (traditional)
Unmet need among all unmarried women
Total demand
Demand satisfied (any method)
Demand satisfied (modern methods)
0%
20%
40%
60%
80%
100%
1970 1975 1980 1985 1990 1995 2000 2005 2010 2015 2020 2025 2030
F
DHS
MICS
Other international survey
National survey
PMA
Subpopulation
+: Higher contraceptive use
−: Lower contraceptive use
A: Other age group
F: Folk methods included
S−: Sterilization included
S+: Sterilization excluded
Unmarried women
Formerly married
Partnered women
CP (any) ≤ 1%

Trinidad and Tobago (Caribbean, SA Group 1) --- Unmarried / Not In−Union

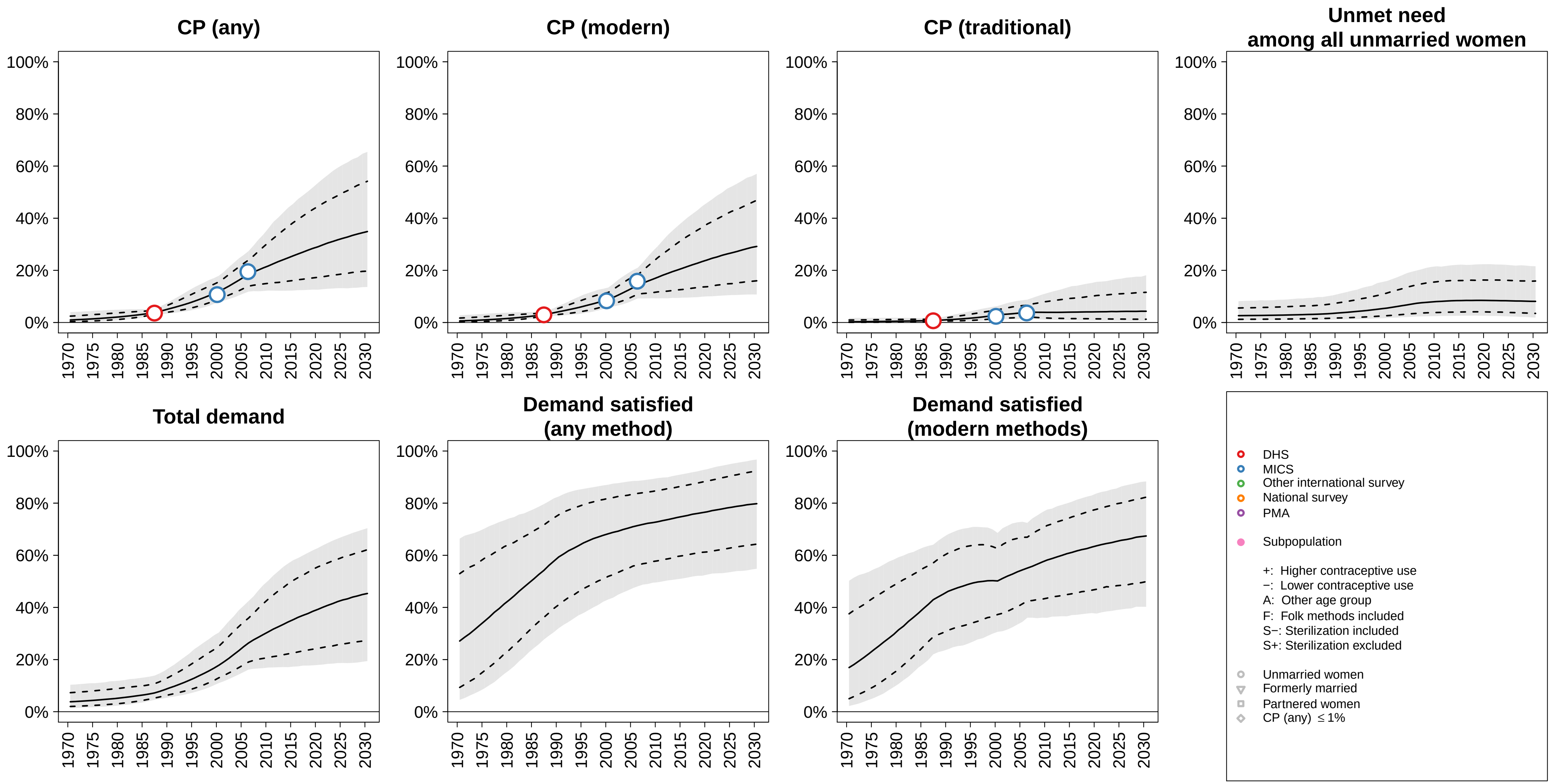

Tunisia (Northern Africa, SA Group 0) −−− Unmarried / Not In−Union

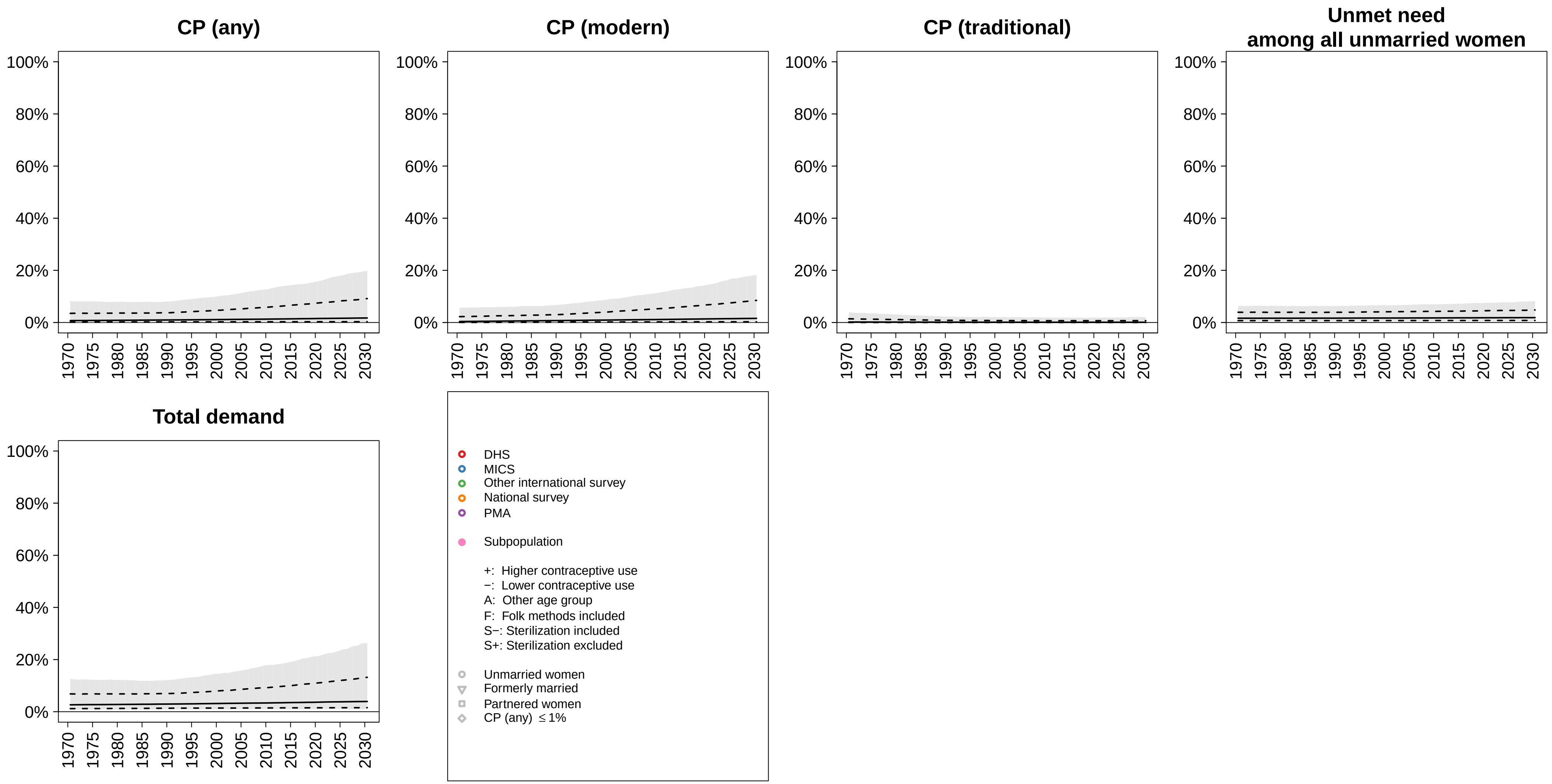

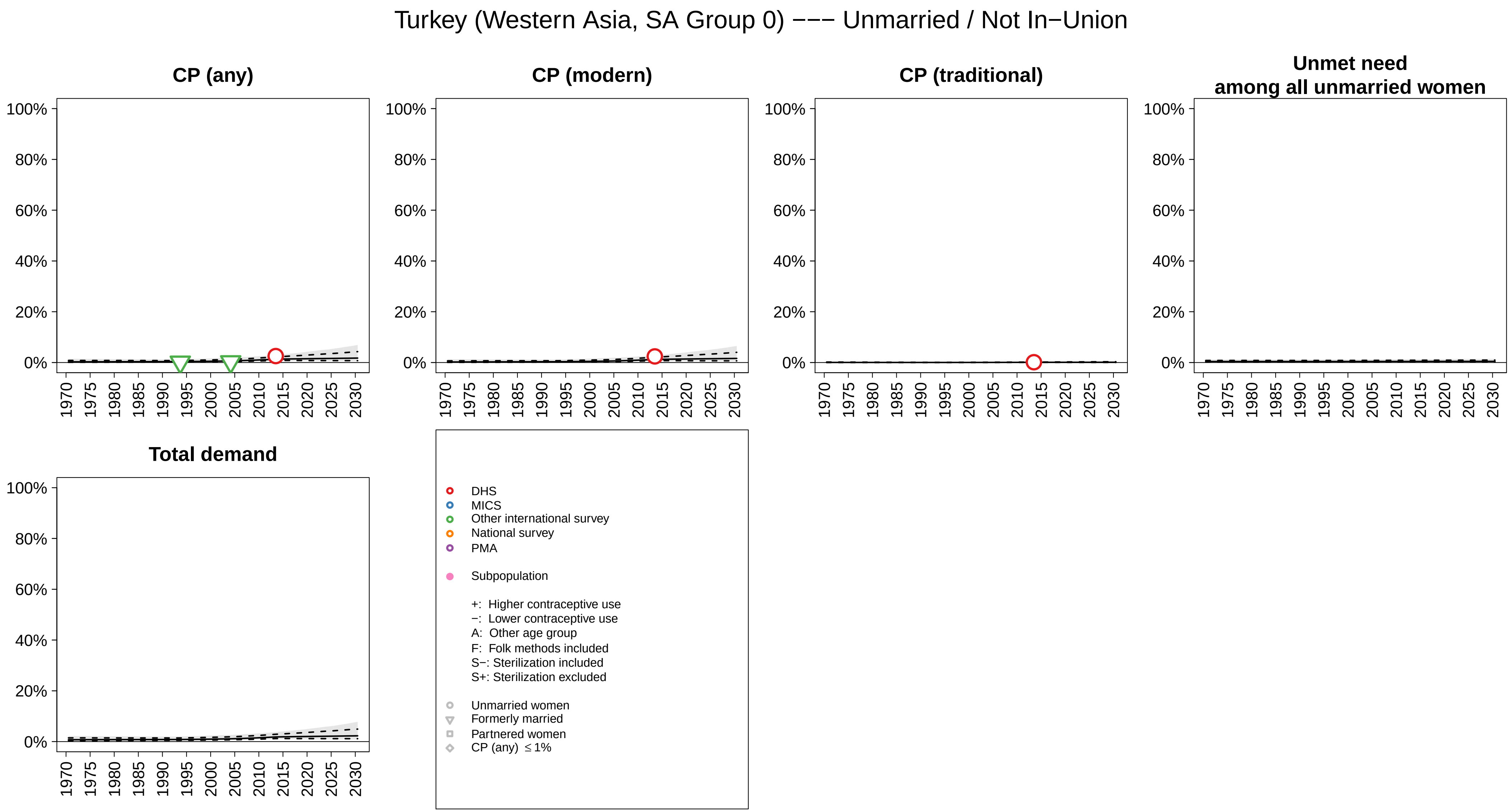
Turkey (Western Asia, SA Group 0) −−− Unmarried / Not In−Union
CP (any)
CP (modern)
CP (traditional)
Unmet need among all unmarried women
Total demand
100%
80%
60%
40%
20%
0%
1970 1975 1980 1985 1990 1995 2000 2005 2010 2015 2020 2025 2030
DHS
MICS
Other international survey
National survey
PMA
Subpopulation
+: Higher contraceptive use
−: Lower contraceptive use
A: Other age group
F: Folk methods included
S−: Sterilization included
S+: Sterilization excluded
Unmarried women
Formerly married
Partnered women
CP (any) ≤ 1%

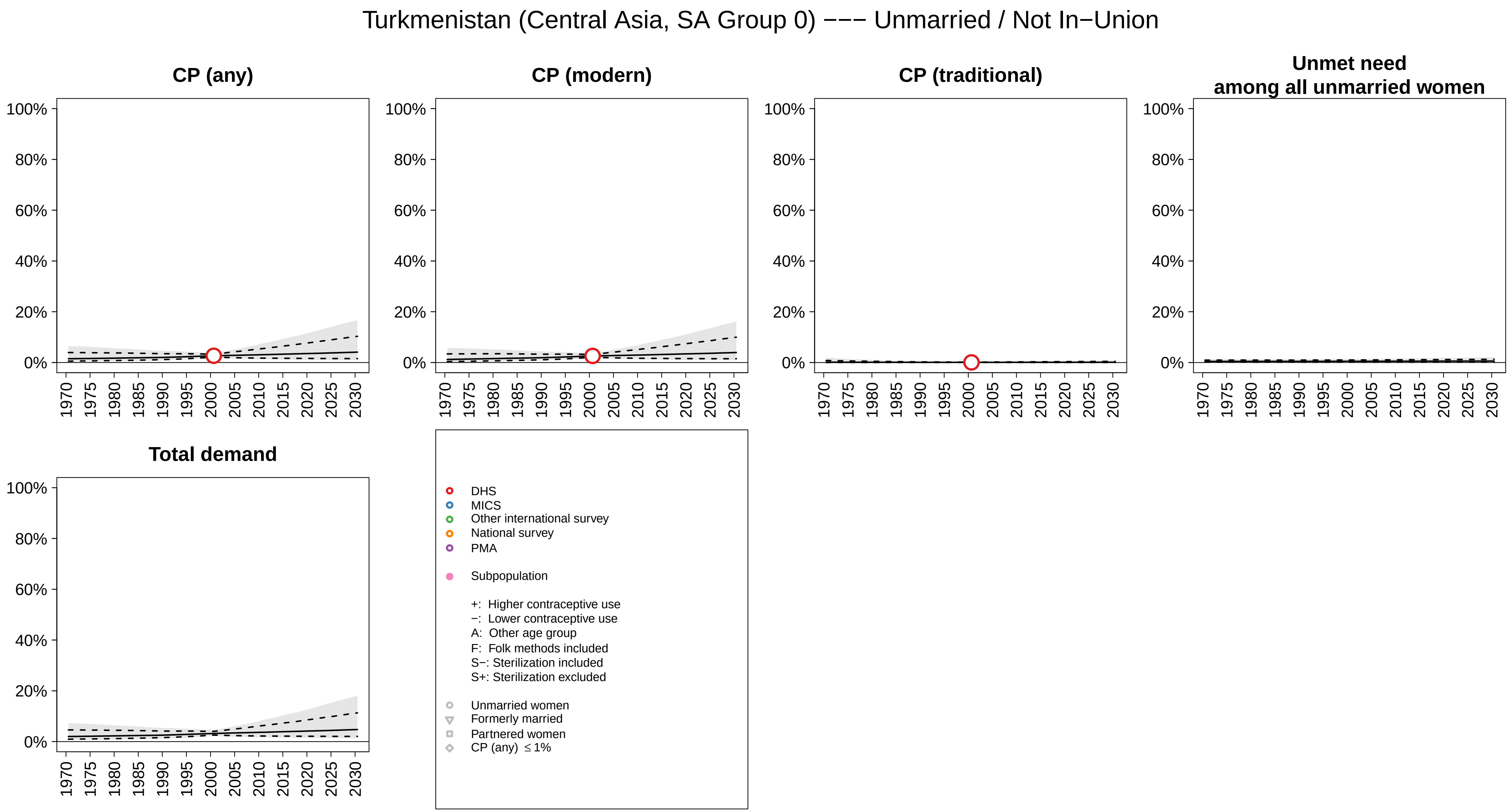
Turkmenistan (Central Asia, SA Group 0) −−− Unmarried / Not In−Union
CP (any)
CP (modern)
CP (traditional)
Unmet need among all unmarried women
Total demand
100%
80%
60%
40%
20%
0%
1970
1975
1980
1985
1990
1995
2000
2005
2010
2015
2020
2025
2030
DHS
MICS
Other international survey
National survey
PMA
Subpopulation
+: Higher contraceptive use
−: Lower contraceptive use
A: Other age group
F: Folk methods included
S−: Sterilization included
S+: Sterilization excluded
Unmarried women
Formerly married
Partnered women
CP (any) ≤ 1%

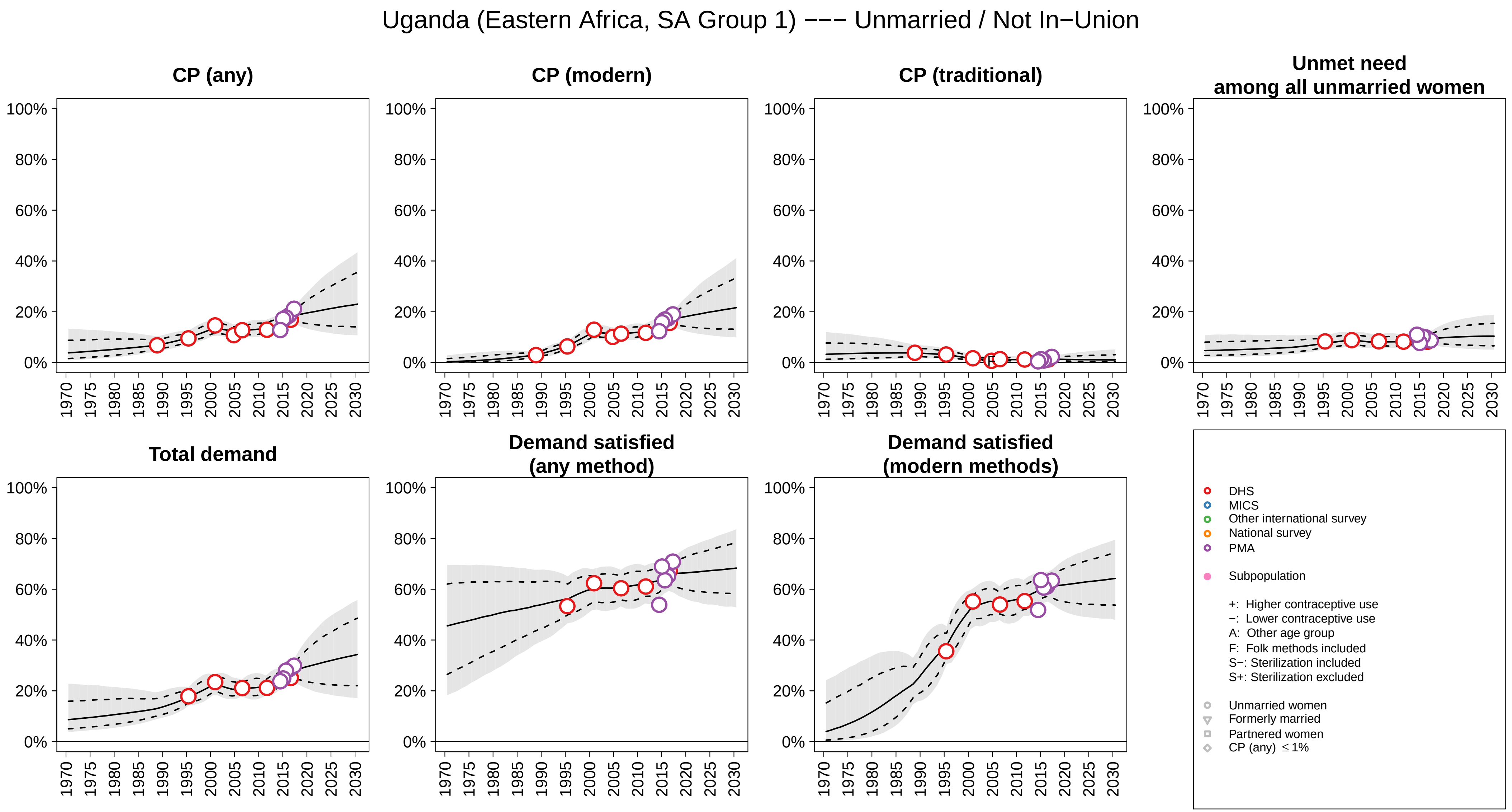

Uganda (Eastern Africa, SA Group 1) −−− Unmarried / Not In−Union
CP (any)
CP (modern)
CP (traditional)
Unmet need among all unmarried women
Total demand
Demand satisfied (any method)
Demand satisfied (modern methods)
0% 20% 40% 60% 80% 100%
1970 1975 1980 1985 1990 1995 2000 2005 2010 2015 2020 2025 2030
DHS
MICS
Other international survey
National survey
PMA
Subpopulation
+: Higher contraceptive use
−: Lower contraceptive use
A: Other age group
F: Folk methods included
S−: Sterilization included
S+: Sterilization excluded
Unmarried women
Formerly married
Partnered women
CP (any) ≤ 1%

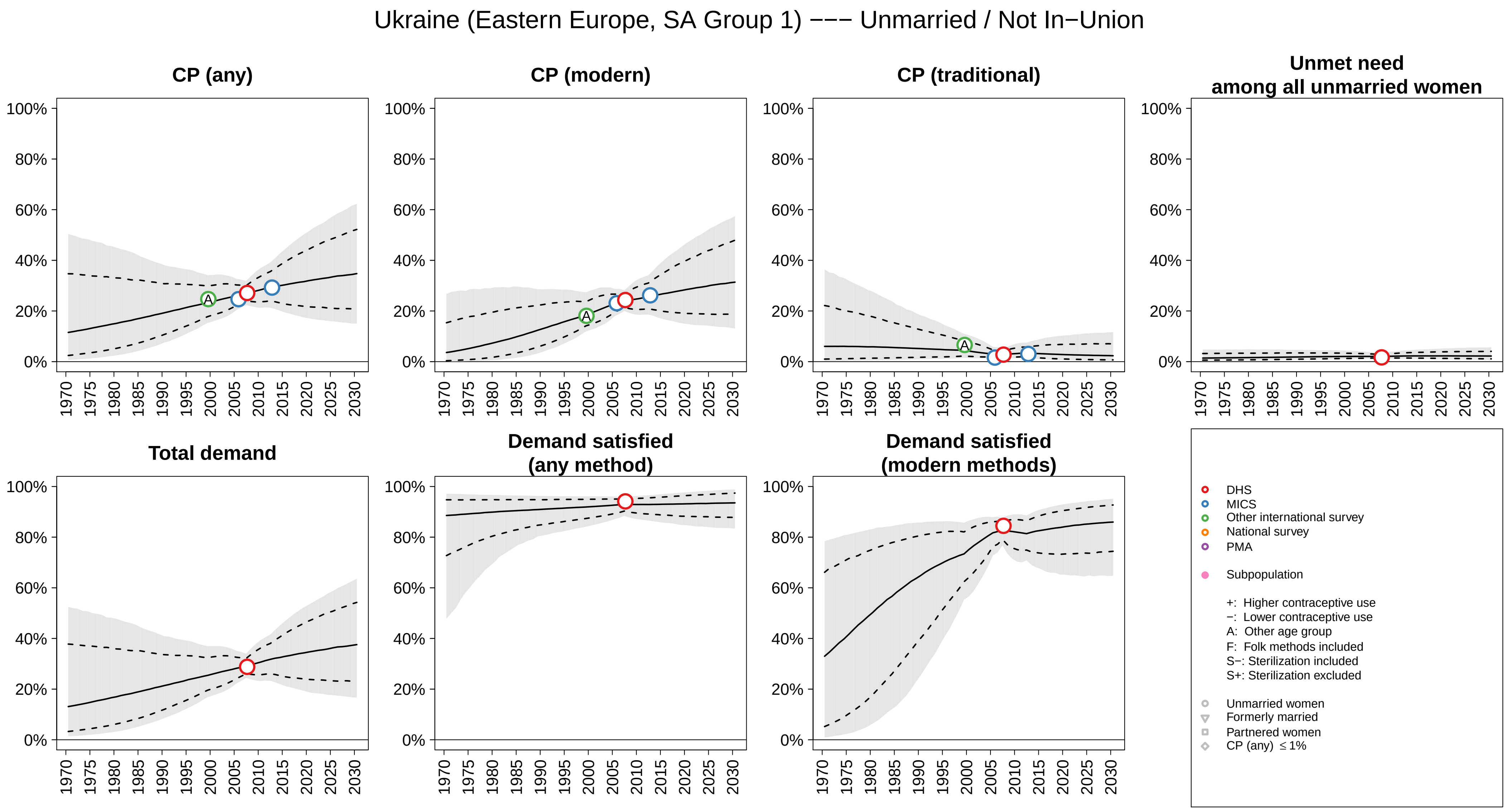
Ukraine (Eastern Europe, SA Group 1) −−− Unmarried / Not In−Union
CP (any)
CP (modern)
CP (traditional)
Unmet need among all unmarried women
Total demand
Demand satisfied (any method)
Demand satisfied (modern methods)
0%
20%
40%
60%
80%
100%
1970
1975
1980
1985
1990
1995
2000
2005
2010
2015
2020
2025
2030
DHS
MICS
Other international survey
National survey
PMA
Subpopulation
+: Higher contraceptive use
−: Lower contraceptive use
A: Other age group
F: Folk methods included
S−: Sterilization included
S+: Sterilization excluded
Unmarried women
Formerly married
Partnered women
CP (any) ≤ 1%

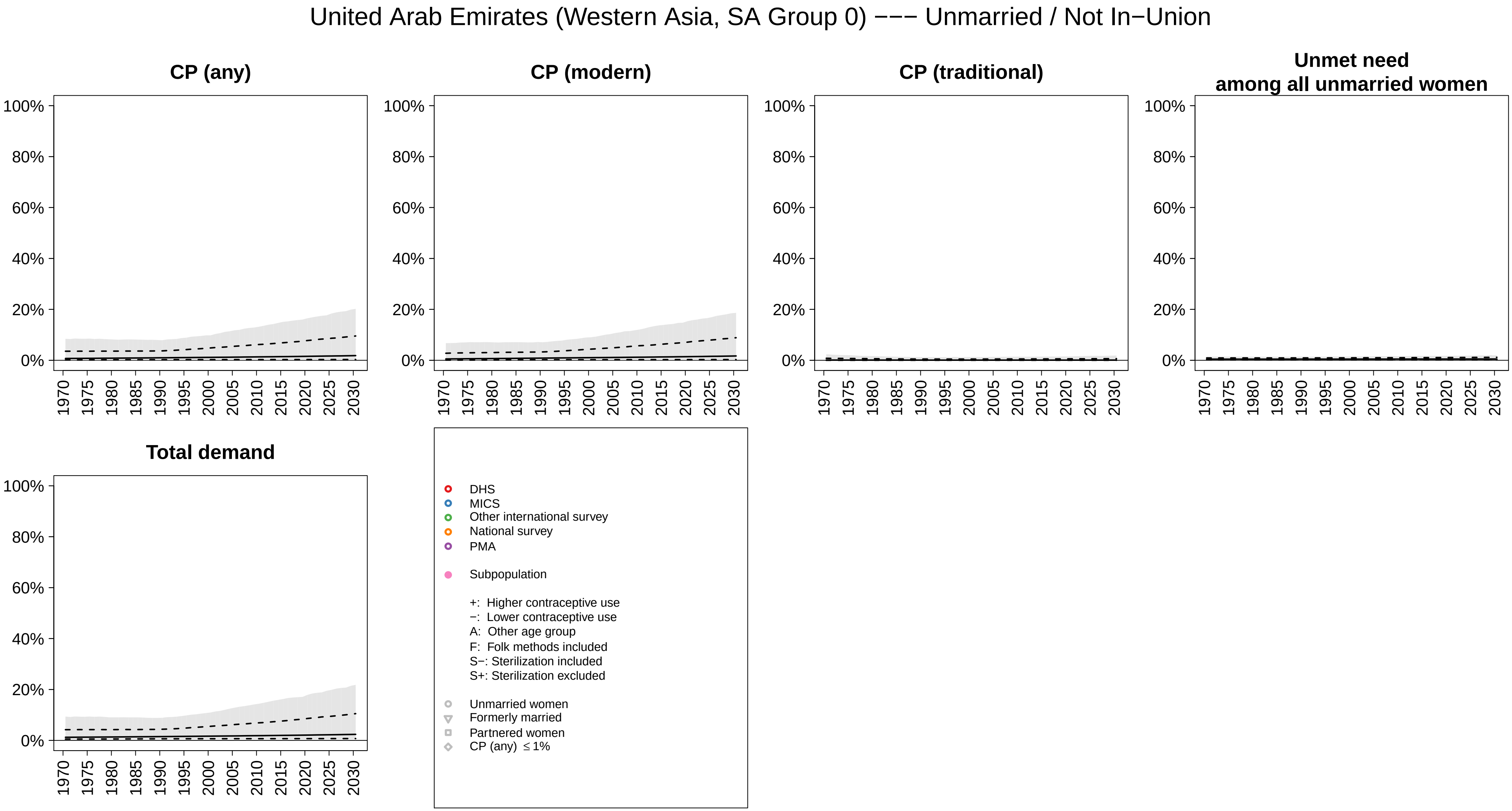
United Arab Emirates (Western Asia, SA Group 0) −−− Unmarried / Not In−Union
CP (any)
CP (modern)
CP (traditional)
Unmet need among all unmarried women
Total demand
100%
80%
60%
40%
20%
0%
1970 1975 1980 1985 1990 1995 2000 2005 2010 2015 2020 2025 2030
DHS
MICS
Other international survey
National survey
PMA
Subpopulation
+: Higher contraceptive use
−: Lower contraceptive use
A: Other age group
F: Folk methods included
S−: Sterilization included
S+: Sterilization excluded
Unmarried women
Formerly married
Partnered women
CP (any) ≤ 1%

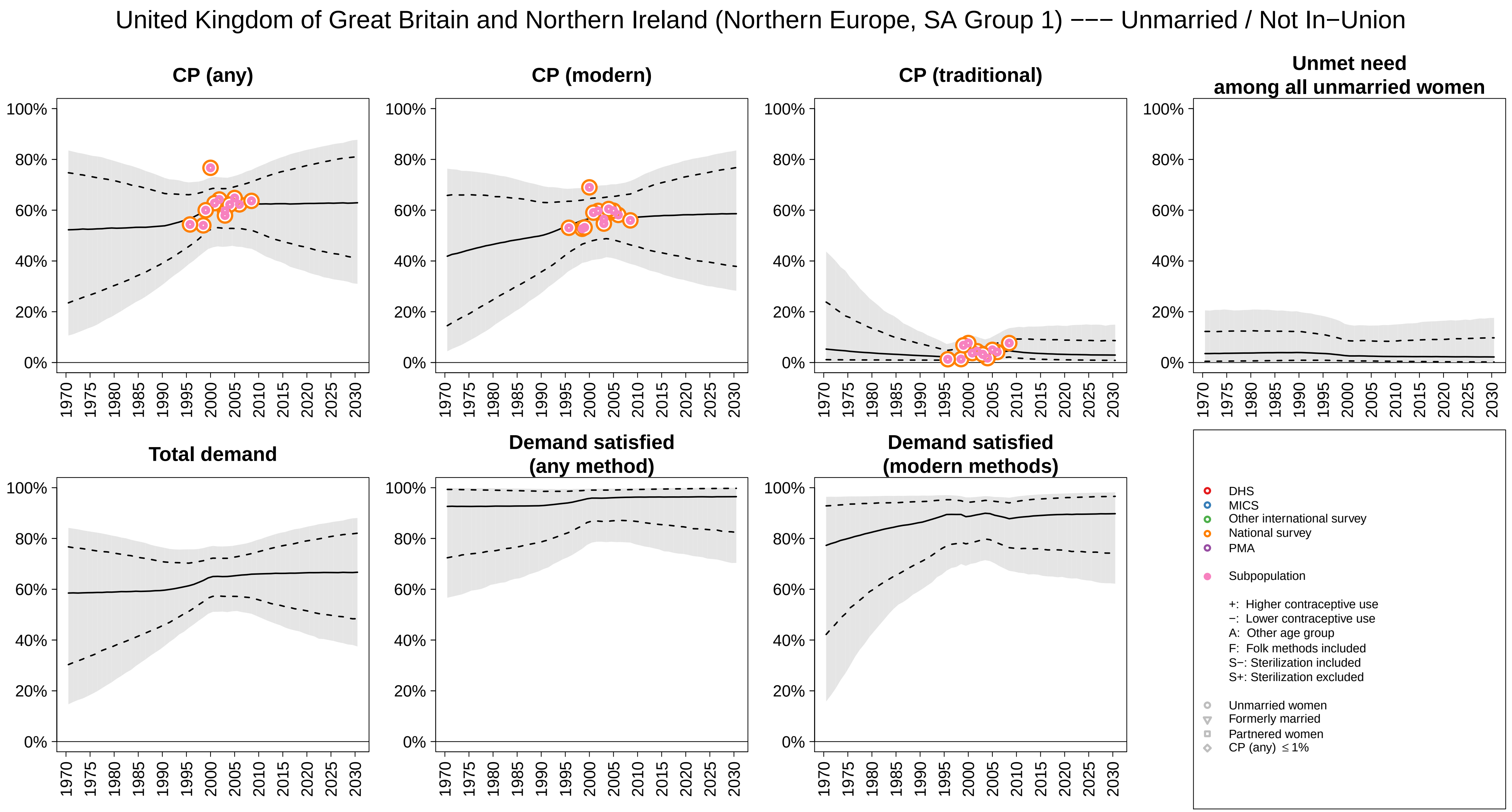
United Kingdom of Great Britain and Northern Ireland (Northern Europe, SA Group 1) −−− Unmarried / Not In−Union
CP (any)
CP (modern)
CP (traditional)
Unmet need among all unmarried women
Total demand
Demand satisfied (any method)
Demand satisfied (modern methods)
0%
20%
40%
60%
80%
100%
1970
1975
1980
1985
1990
1995
2000
2005
2010
2015
2020
2025
2030
DHS
MICS
Other international survey
National survey
PMA
Subpopulation
+: Higher contraceptive use
−: Lower contraceptive use
A: Other age group
F: Folk methods included
S−: Sterilization included
S+: Sterilization excluded
Unmarried women
Formerly married
Partnered women
CP (any) ≤ 1%

United Rep. of Tanzania (Eastern Africa, SA Group 1) −−− Unmarried / Not In−Union

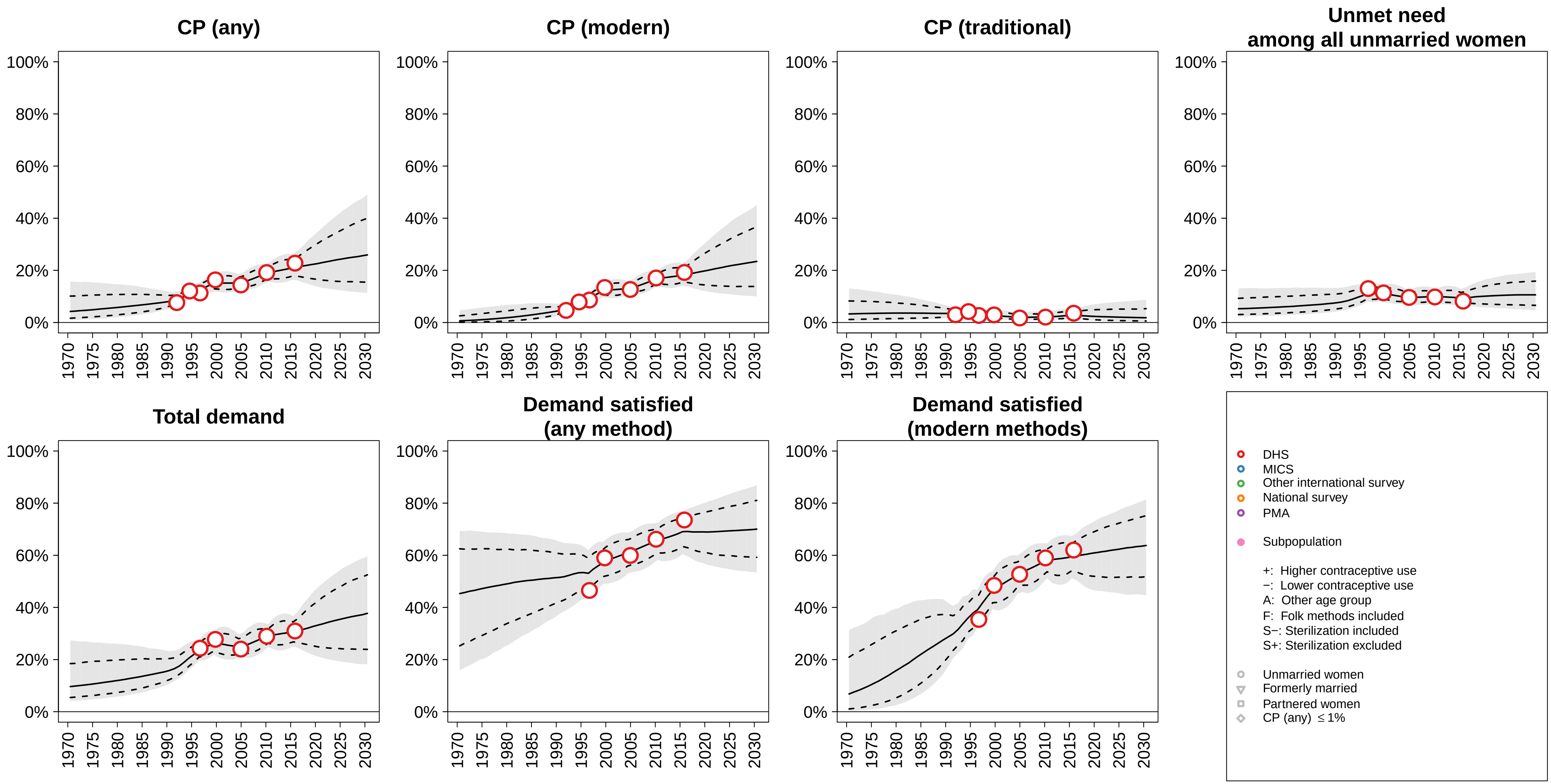

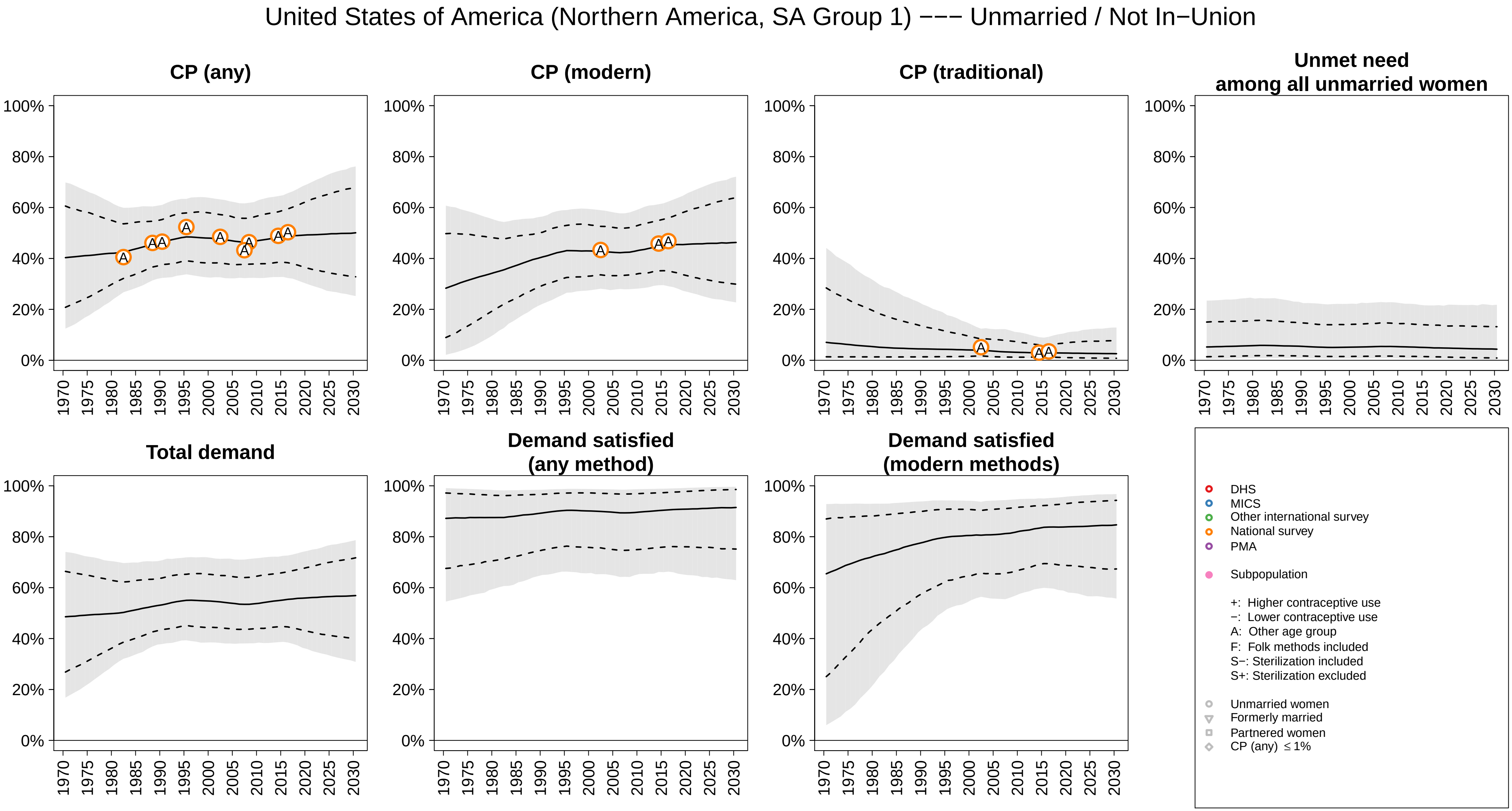
United States of America (Northern America, SA Group 1) −−− Unmarried / Not In−Union
CP (any)
CP (modern)
CP (traditional)
Unmet need among all unmarried women
Total demand
Demand satisfied (any method)
Demand satisfied (modern methods)
100%
80%
60%
40%
20%
0%
1970
1975
1980
1985
1990
1995
2000
2005
2010
2015
2020
2025
2030
DHS
MICS
Other international survey
National survey
PMA
Subpopulation
+: Higher contraceptive use
−: Lower contraceptive use
A: Other age group
F: Folk methods included
S−: Sterilization included
S+: Sterilization excluded
Unmarried women
Formerly married
Partnered women
CP (any) ≤ 1%

United States Virgin Islands (Caribbean, SA Group 1) −−− Unmarried / Not In−Union

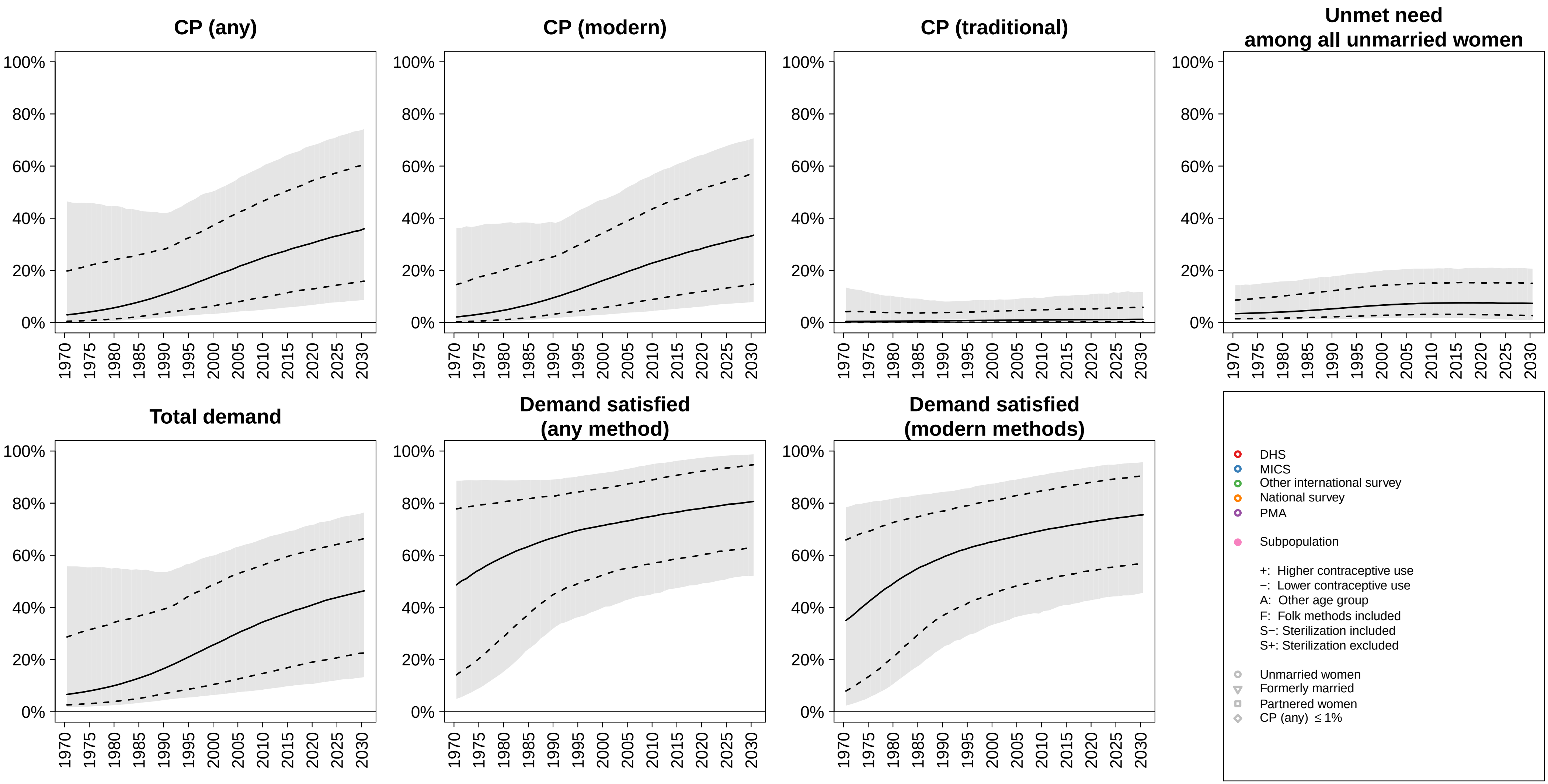

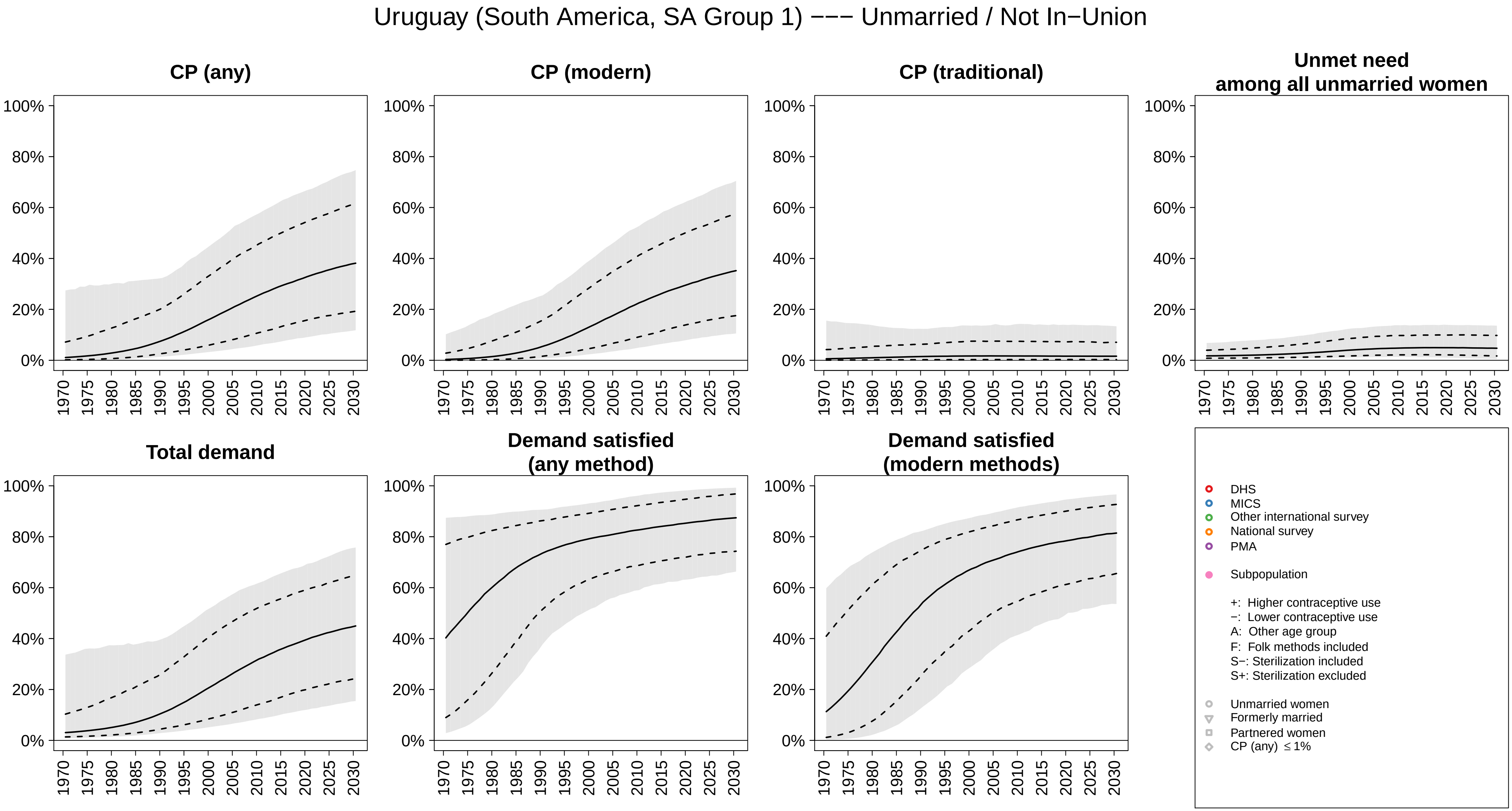
Uruguay (South America, SA Group 1) −−− Unmarried / Not In−Union
CP (any)
CP (modern)
CP (traditional)
Unmet need among all unmarried women
Total demand
Demand satisfied (any method)
Demand satisfied (modern methods)
0%
20%
40%
60%
80%
100%
1970
1975
1980
1985
1990
1995
2000
2005
2010
2015
2020
2025
2030
DHS
MICS
Other international survey
National survey
PMA
Subpopulation
+: Higher contraceptive use
−: Lower contraceptive use
A: Other age group
F: Folk methods included
S−: Sterilization included
S+: Sterilization excluded
Unmarried women
Formerly married
Partnered women
CP (any) ≤ 1%

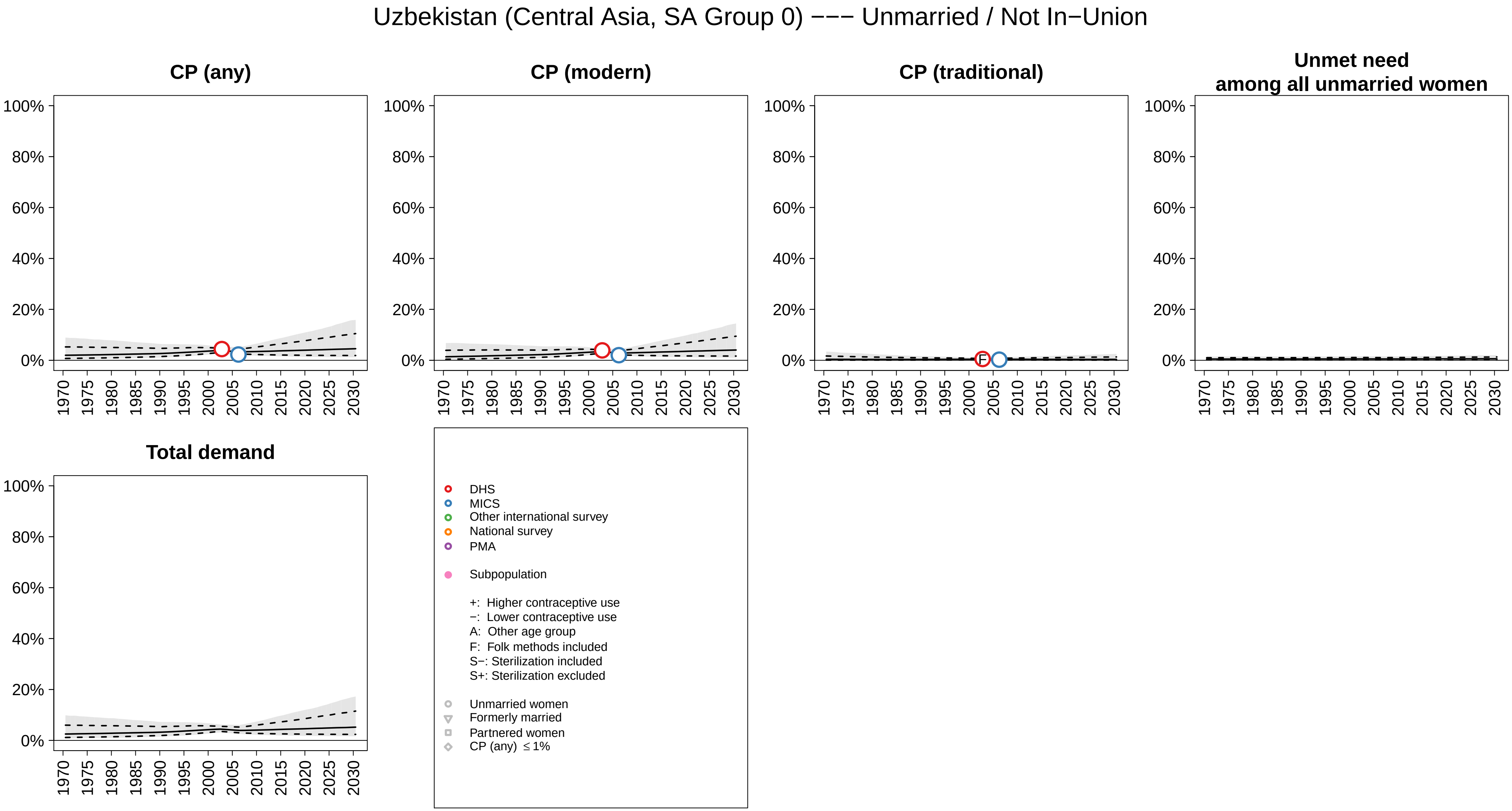
Uzbekistan (Central Asia, SA Group 0) --- Unmarried / Not In−Union
CP (any)
CP (modern)
CP (traditional)
Unmet need among all unmarried women
Total demand
100%
80%
60%
40%
20%
0%
1970 1975 1980 1985 1990 1995 2000 2005 2010 2015 2020 2025 2030
F
DHS
MICS
Other international survey
National survey
PMA
Subpopulation
+: Higher contraceptive use
−: Lower contraceptive use
A: Other age group
F: Folk methods included
S−: Sterilization included
S+: Sterilization excluded
Unmarried women
Formerly married
Partnered women
CP (any) ≤ 1%

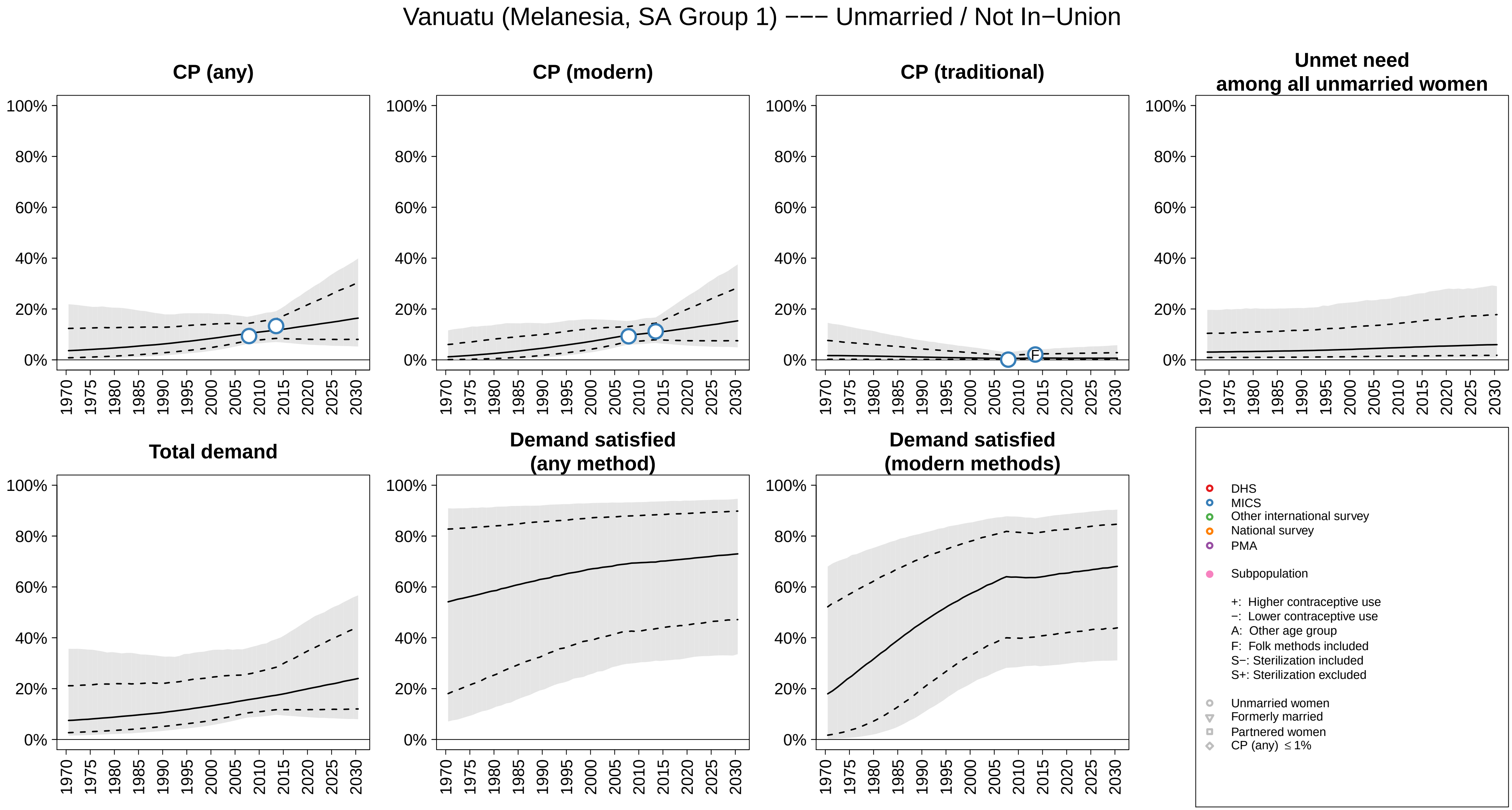
Vanuatu (Melanesia, SA Group 1) −−− Unmarried / Not In−Union
CP (any)
CP (modern)
CP (traditional)
Unmet need among all unmarried women
Total demand
Demand satisfied (any method)
Demand satisfied (modern methods)
100%
80%
60%
40%
20%
0%
1970
1975
1980
1985
1990
1995
2000
2005
2010
2015
2020
2025
2030
F
DHS
MICS
Other international survey
National survey
PMA
Subpopulation
+: Higher contraceptive use
−: Lower contraceptive use
A: Other age group
F: Folk methods included
S−: Sterilization included
S+: Sterilization excluded
Unmarried women
Formerly married
Partnered women
CP (any) ≤ 1%

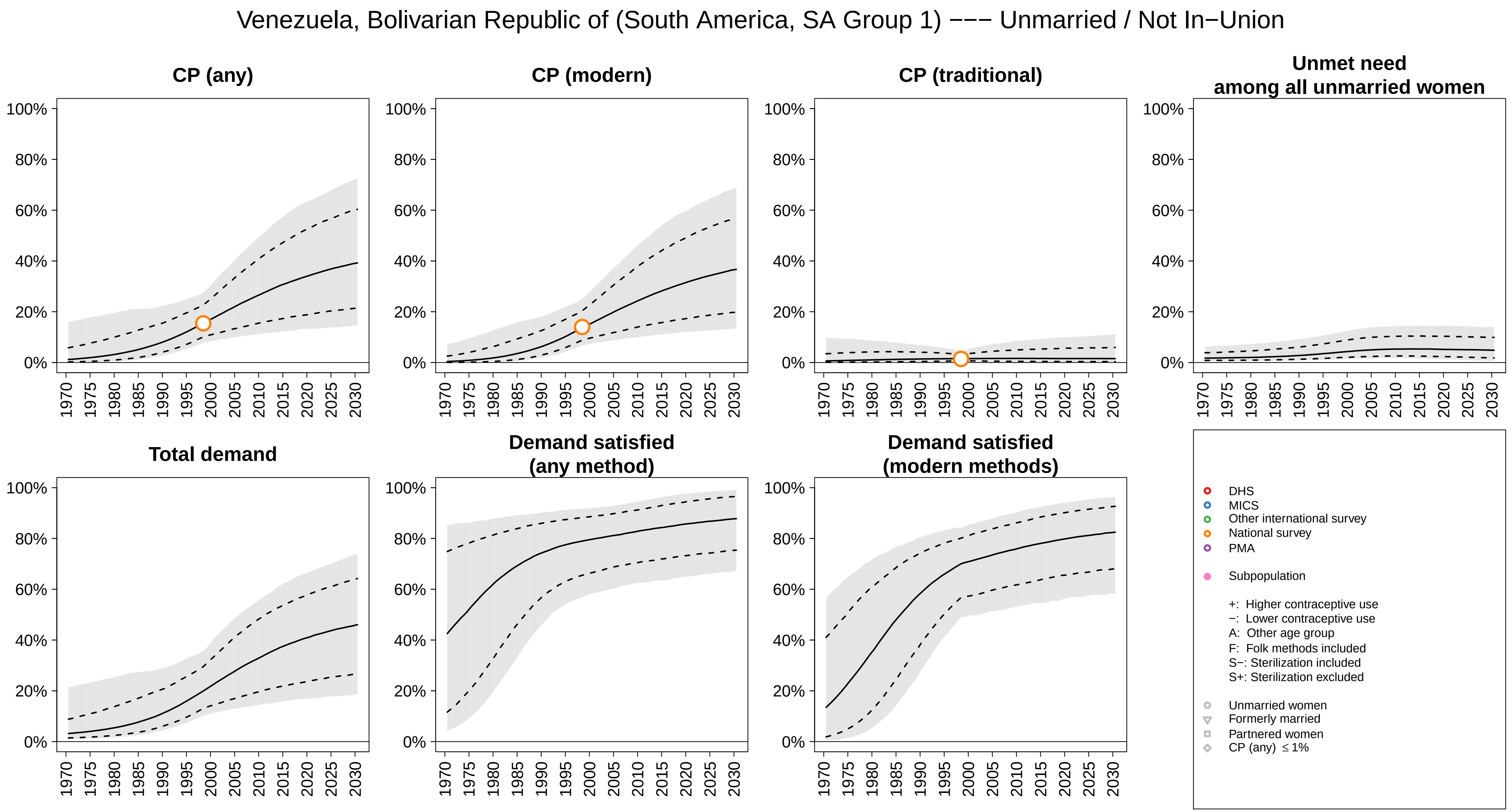
Venezuela, Bolivarian Republic of (South America, SA Group 1) −−− Unmarried / Not In−Union
CP (any)
CP (modern)
CP (traditional)
Unmet need among all unmarried women
Total demand
Demand satisfied (any method)
Demand satisfied (modern methods)
0%
20%
40%
60%
80%
100%
1970
1975
1980
1985
1990
1995
2000
2005
2010
2015
2020
2025
2030
DHS
MICS
Other international survey
National survey
PMA
Subpopulation
+: Higher contraceptive use
−: Lower contraceptive use
A: Other age group
F: Folk methods included
S−: Sterilization included
S+: Sterilization excluded
Unmarried women
Formerly married
Partnered women
CP (any) ≤ 1%

Viet Nam (South−eastern Asia, SA Group 0) −−− Unmarried / Not In−Union

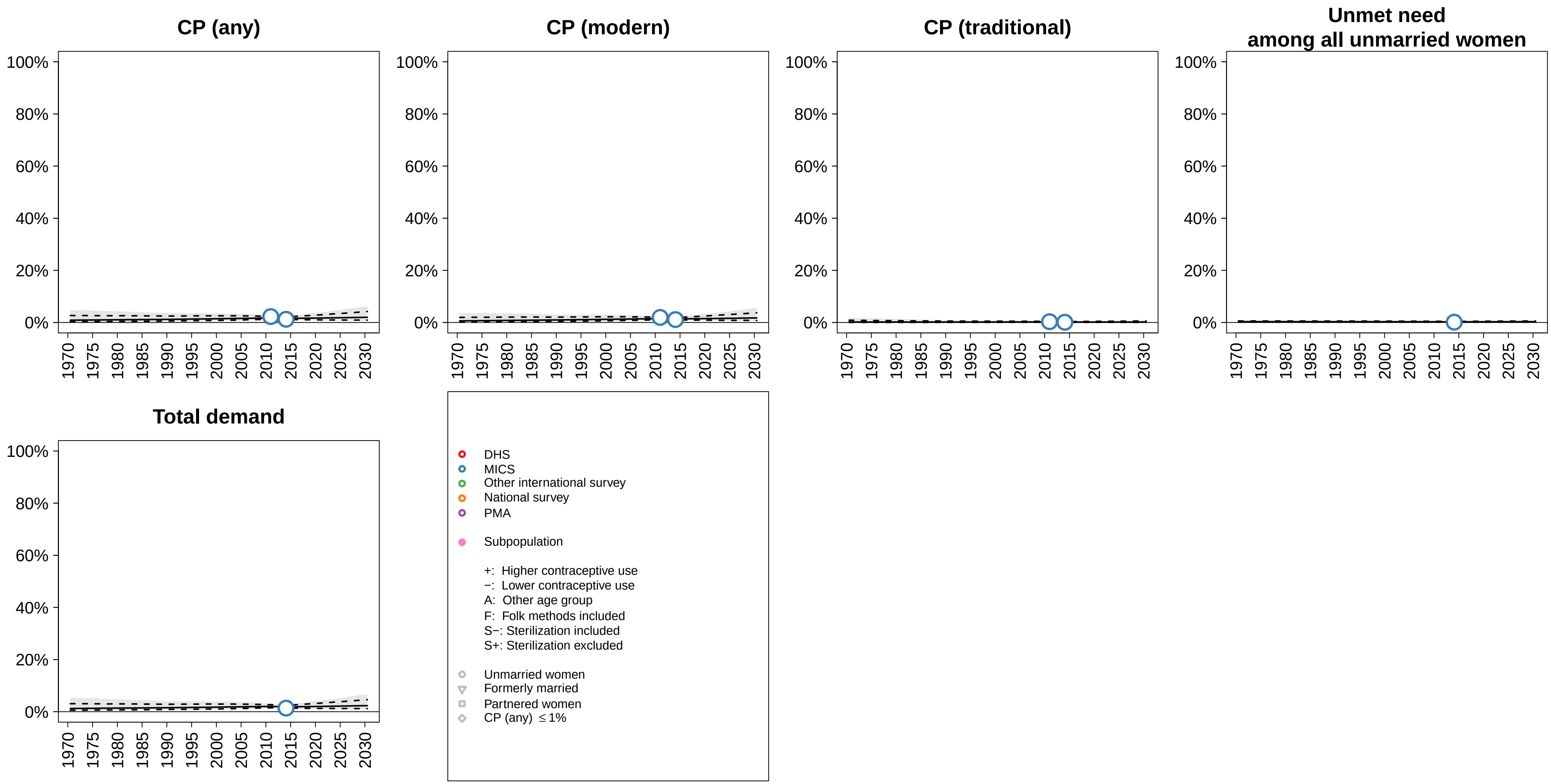

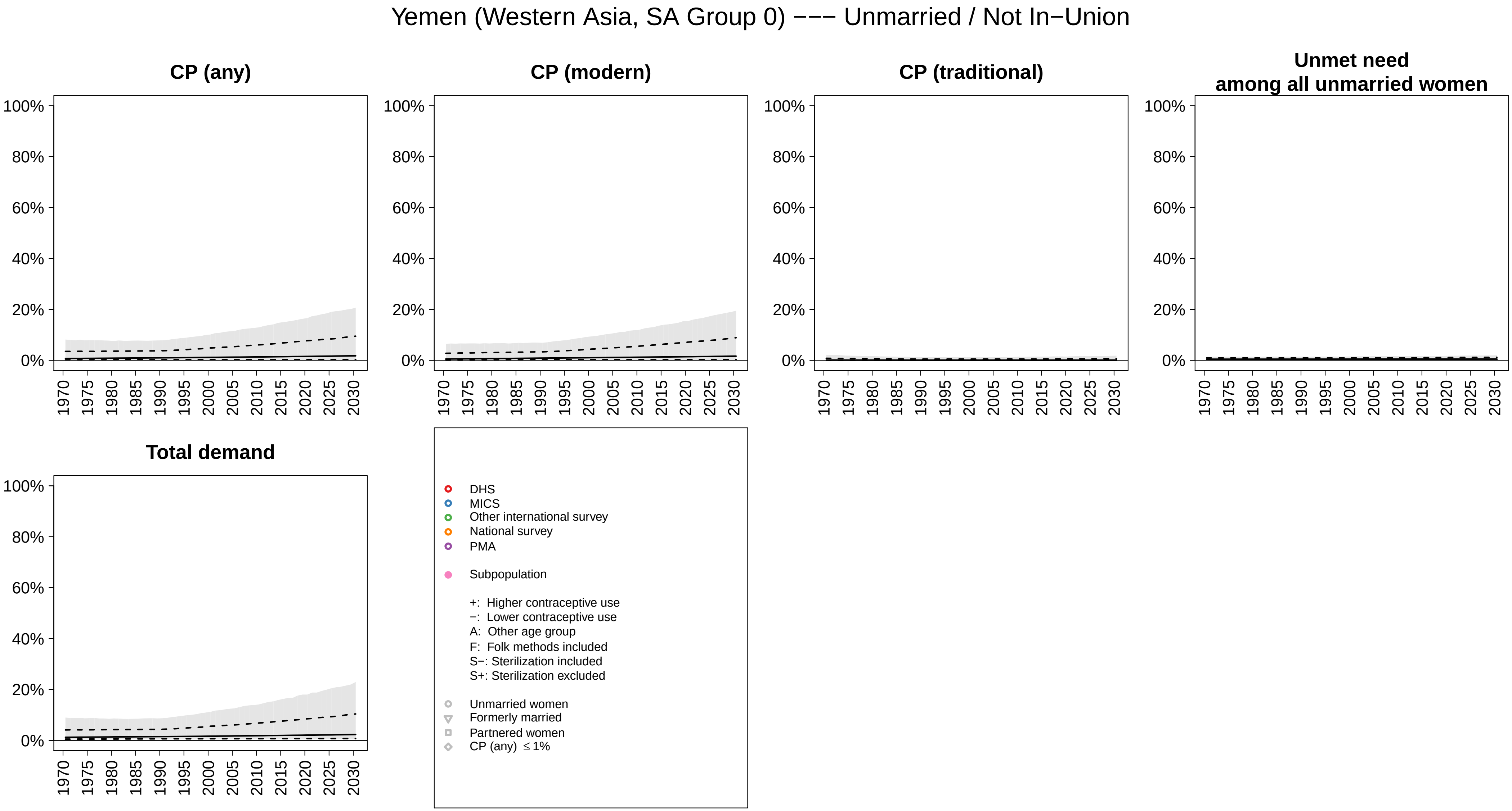
Yemen (Western Asia, SA Group 0) −−− Unmarried / Not In−Union
CP (any)
CP (modern)
CP (traditional)
Unmet need among all unmarried women
Total demand
0%
20%
40%
60%
80%
100%
1970
1975
1980
1985
1990
1995
2000
2005
2010
2015
2020
2025
2030
DHS
MICS
Other international survey
National survey
PMA
Subpopulation
+: Higher contraceptive use
−: Lower contraceptive use
A: Other age group
F: Folk methods included
S−: Sterilization included
S+: Sterilization excluded
Unmarried women
Formerly married
Partnered women
CP (any) ≤ 1%

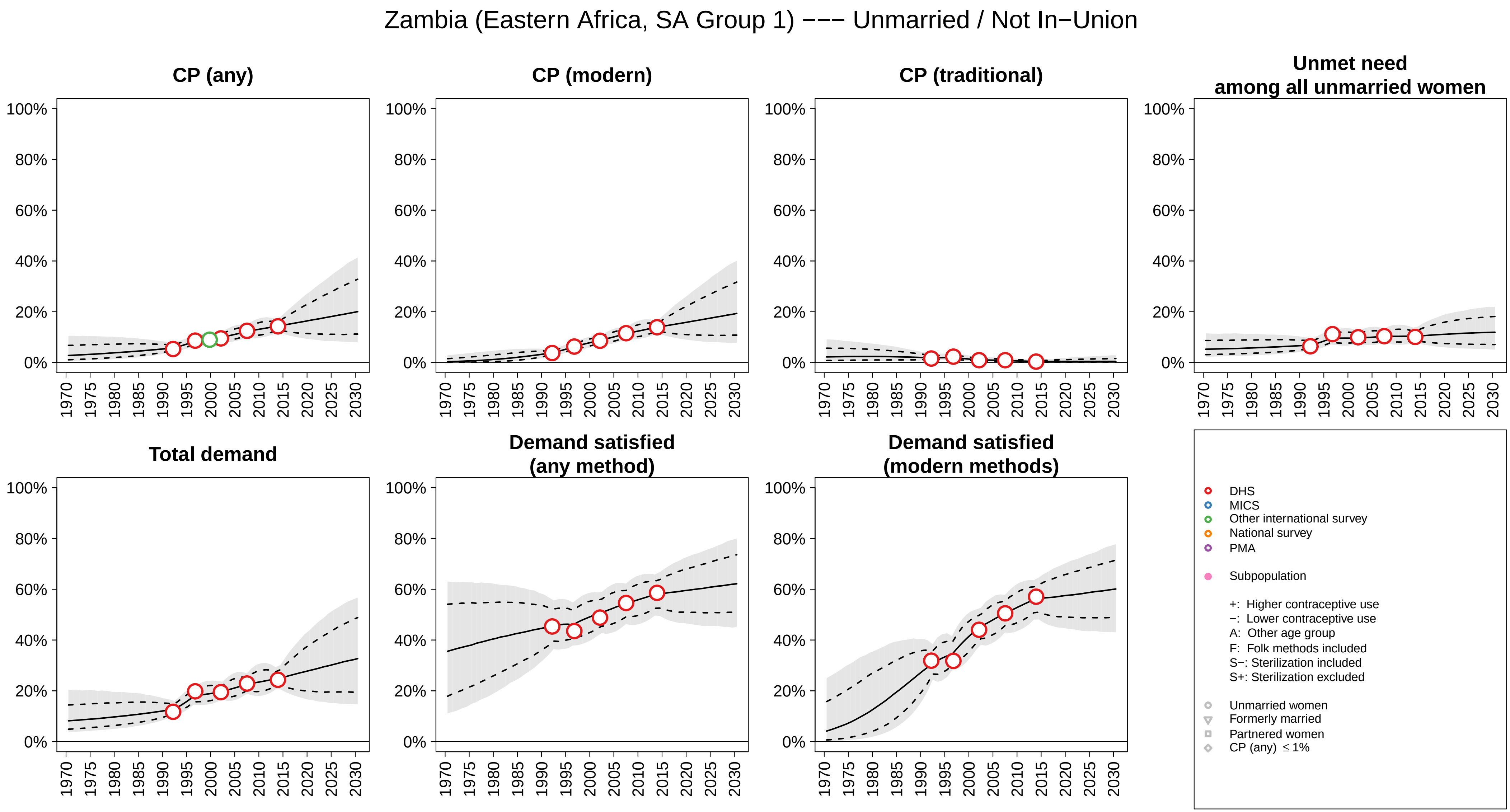
Zambia (Eastern Africa, SA Group 1) −−− Unmarried / Not In−Union
CP (any)
CP (modern)
CP (traditional)
Unmet need among all unmarried women
Total demand
Demand satisfied (any method)
Demand satisfied (modern methods)
DHS
MICS
Other international survey
National survey
PMA
Subpopulation
+: Higher contraceptive use
−: Lower contraceptive use
A: Other age group
F: Folk methods included
S−: Sterilization included
S+: Sterilization excluded
Unmarried women
Formerly married
Partnered women
CP (any) ≤ 1%

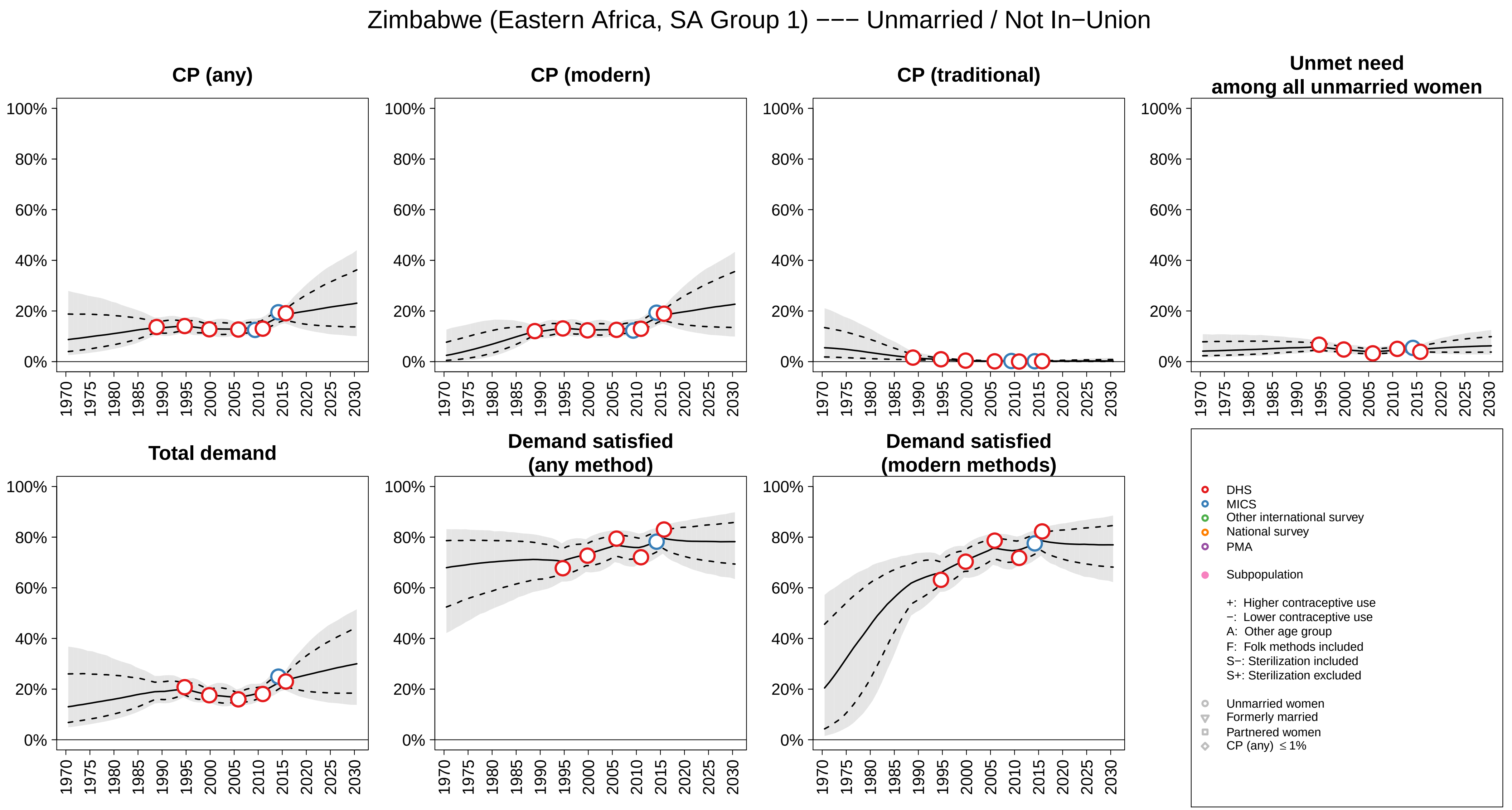

Zimbabwe (Eastern Africa, SA Group 1) --- Unmarried / Not In−Union
CP (any)
CP (modern)
CP (traditional)
Unmet need among all unmarried women
Total demand
Demand satisfied (any method)
Demand satisfied (modern methods)
100%
80%
60%
40%
20%
0%
1970
1975
1980
1985
1990
1995
2000
2005
2010
2015
2020
2025
2030
DHS
MICS
Other international survey
National survey
PMA
Subpopulation
+: Higher contraceptive use
−: Lower contraceptive use
A: Other age group
F: Folk methods included
S−: Sterilization included
S+: Sterilization excluded
Unmarried women
Formerly married
Partnered women
CP (any) ≤ 1%

1.2 Country aggregates

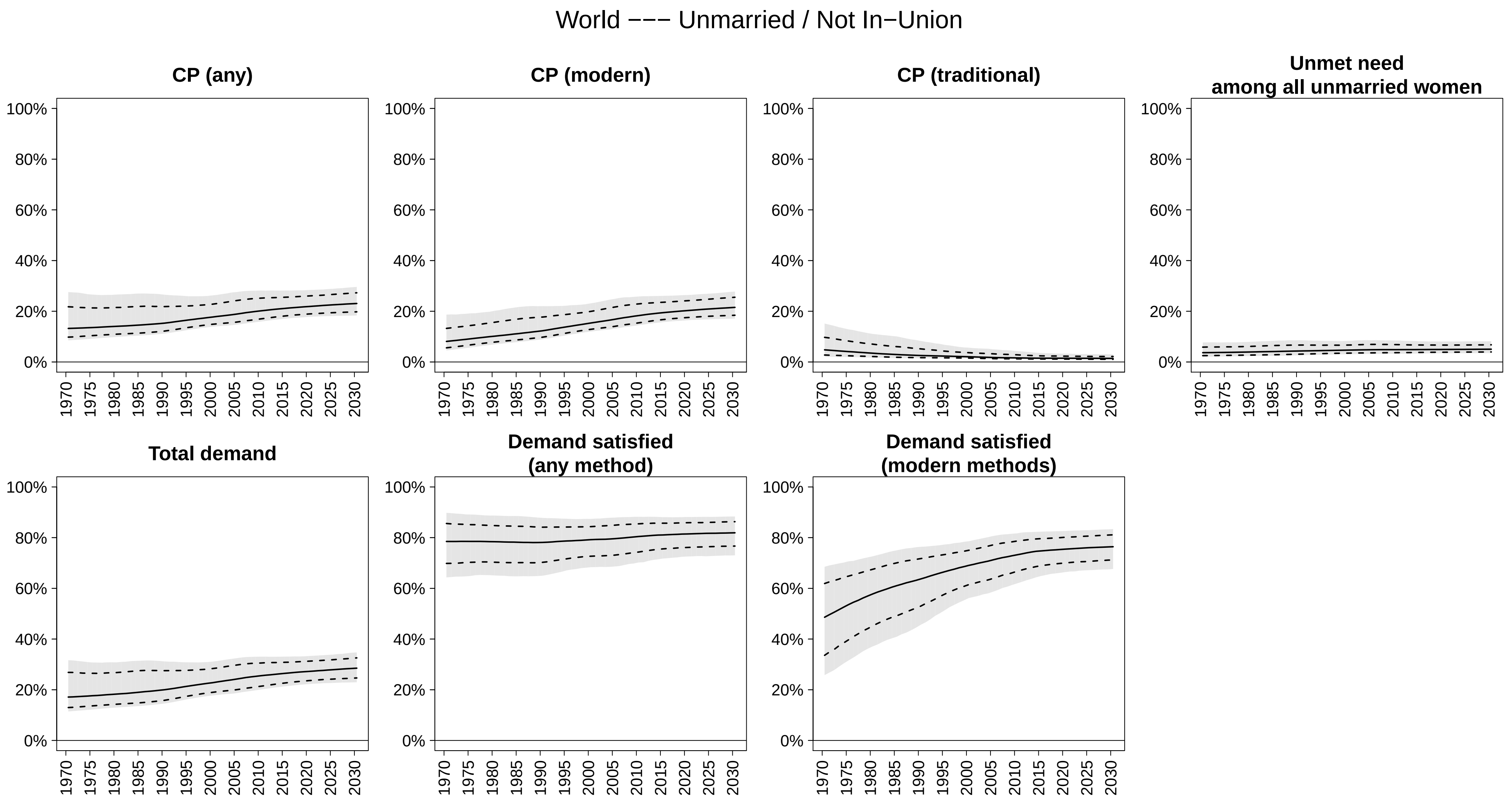
World −−− Unmarried / Not In−Union
CP (any)
CP (modern)
CP (traditional)
Unmet need among all unmarried women
Total demand
Demand satisfied (any method)
Demand satisfied (modern methods)
0%
20%
40%
60%
80%
100%
1970
1975
1980
1985
1990
1995
2000
2005
2010
2015
2020
2025
2030

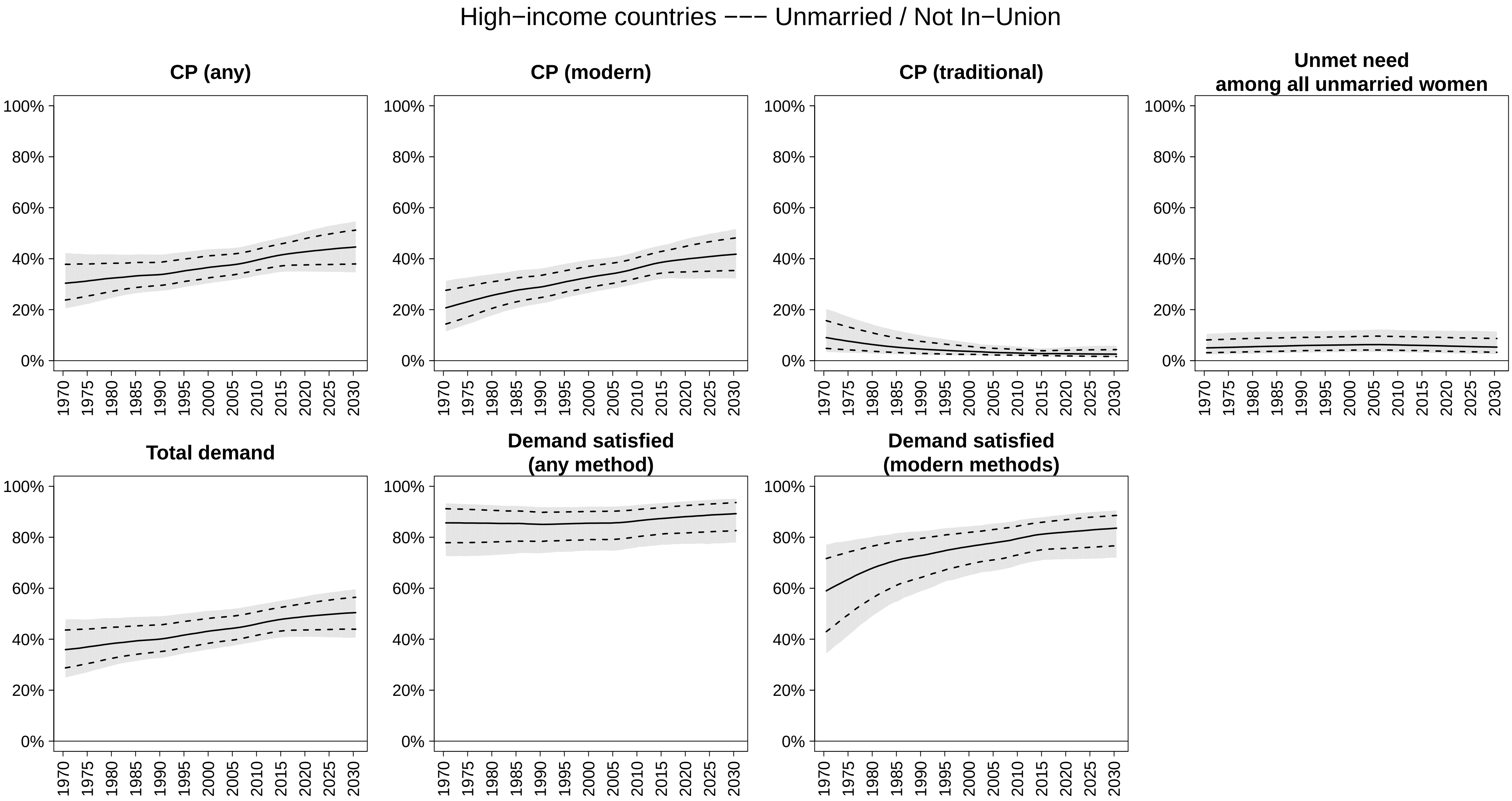
High−income countries −−− Unmarried / Not In−Union
CP (any)
CP (modern)
CP (traditional)
Unmet need among all unmarried women
Total demand
Demand satisfied (any method)
Demand satisfied (modern methods)
0%
20%
40%
60%
80%
100%
1970
1975
1980
1985
1990
1995
2000
2005
2010
2015
2020
2025
2030

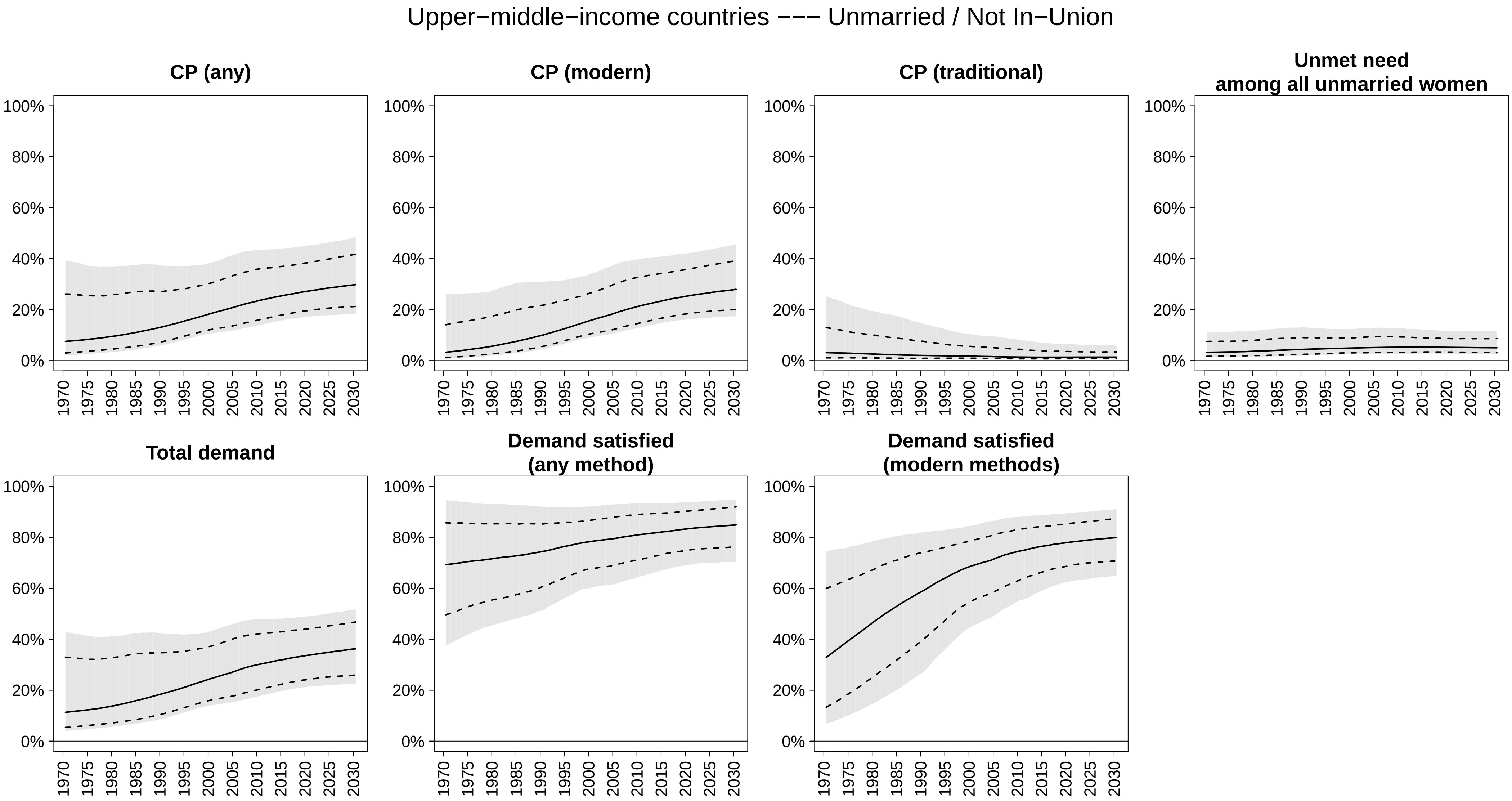
Upper−middle−income countries −−− Unmarried / Not In−Union
CP (any)
CP (modern)
CP (traditional)
Unmet need among all unmarried women
Total demand
Demand satisfied (any method)
Demand satisfied (modern methods)
0%
20%
40%
60%
80%
100%
1970
1975
1980
1985
1990
1995
2000
2005
2010
2015
2020
2025
2030

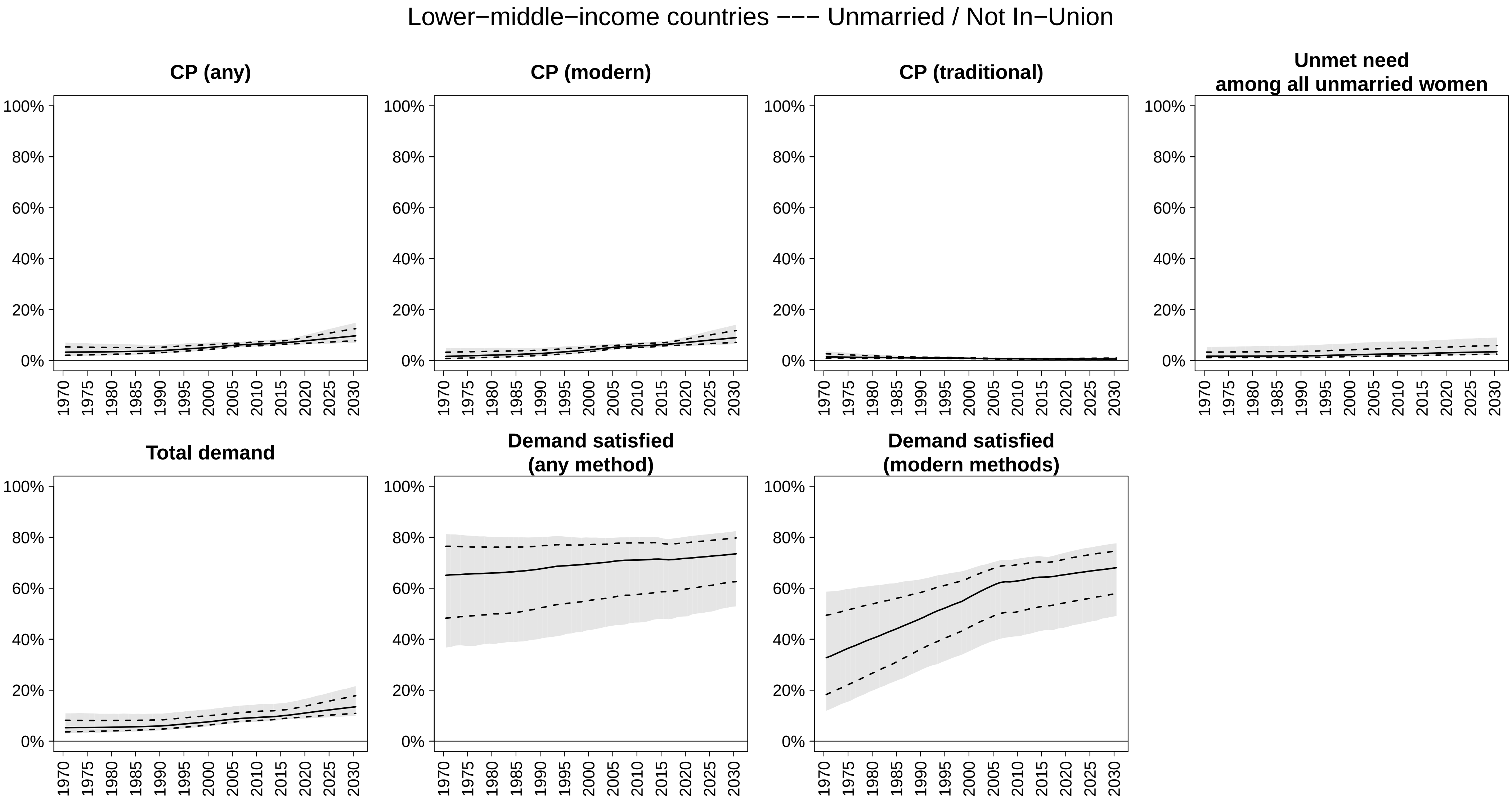
Lower−middle−income countries −−− Unmarried / Not In−Union
CP (any)
CP (modern)
CP (traditional)
Unmet need among all unmarried women
Total demand
Demand satisfied (any method)
Demand satisfied (modern methods)
0%
20%
40%
60%
80%
100%
1970
1975
1980
1985
1990
1995
2000
2005
2010
2015
2020
2025
2030

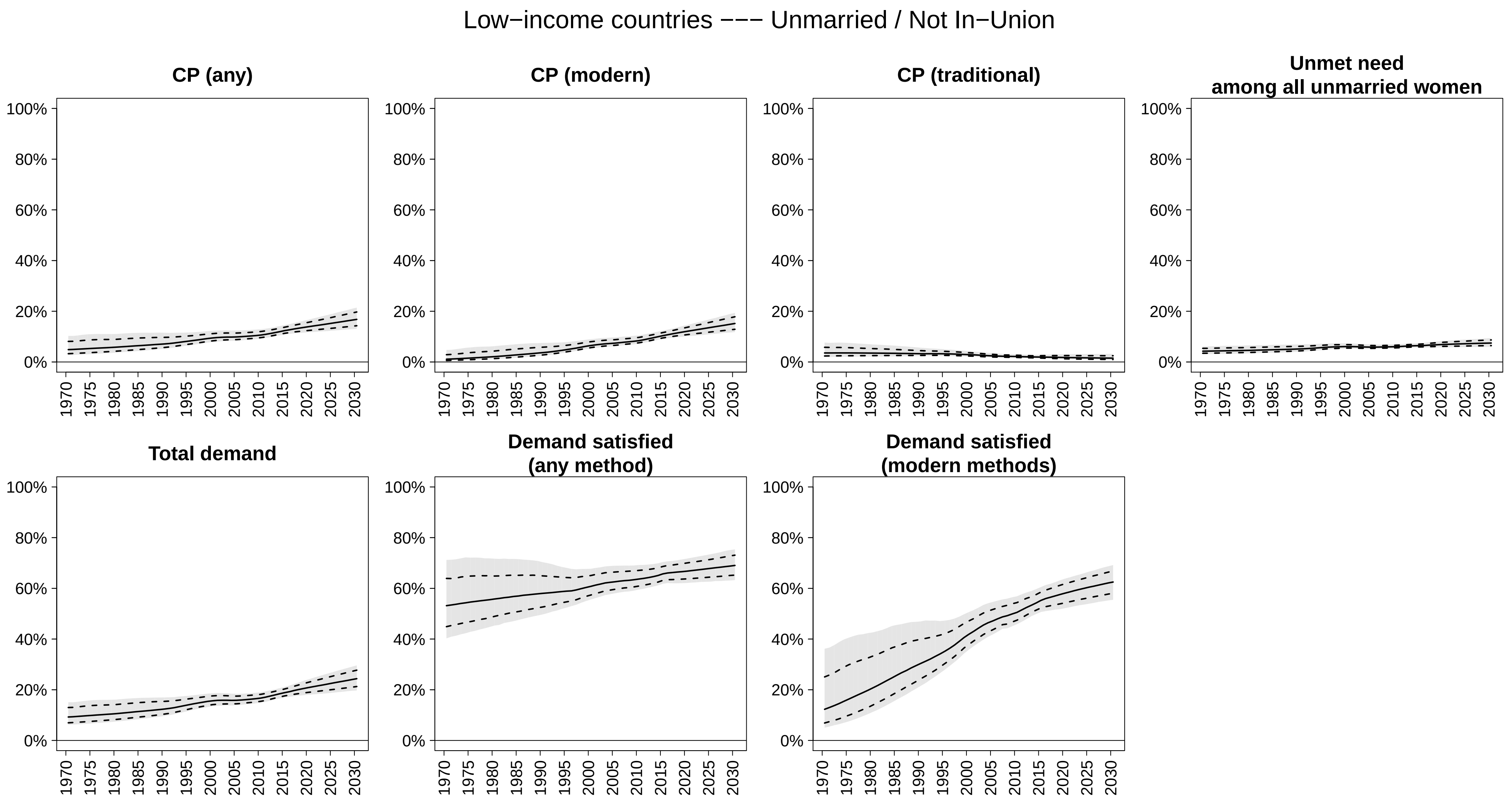
Low−income countries −−− Unmarried / Not In−Union
CP (any)
CP (modern)
CP (traditional)
Unmet need among all unmarried women
Total demand
Demand satisfied (any method)
Demand satisfied (modern methods)
0%
20%
40%
60%
80%
100%
1970
1975
1980
1985
1990
1995
2000
2005
2010
2015
2020
2025
2030

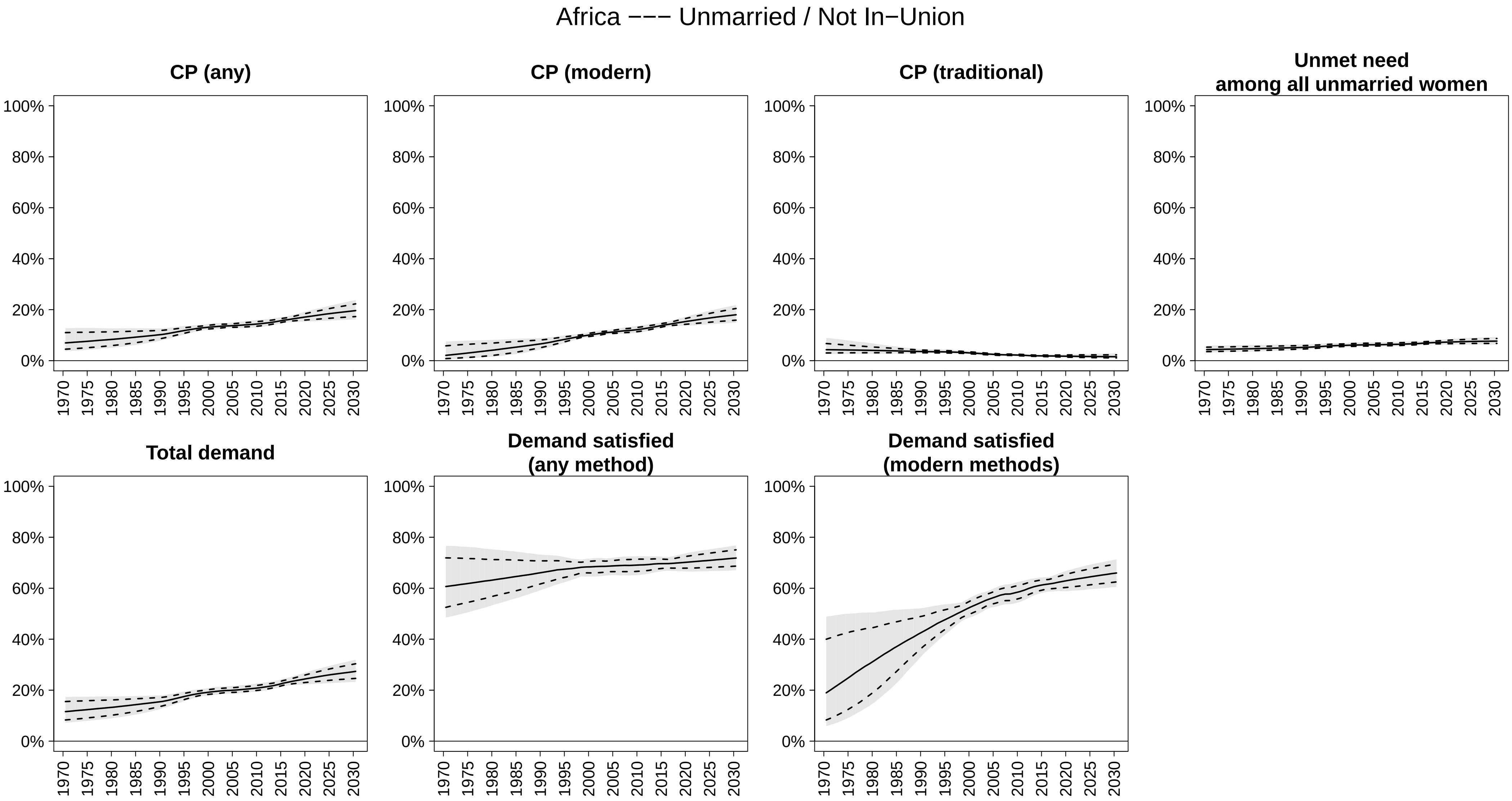
Africa −−− Unmarried / Not In−Union
CP (any)
CP (modern)
CP (traditional)
Unmet need among all unmarried women
Total demand
Demand satisfied (any method)
Demand satisfied (modern methods)
0%
20%
40%
60%
80%
100%
1970
1975
1980
1985
1990
1995
2000
2005
2010
2015
2020
2025
2030

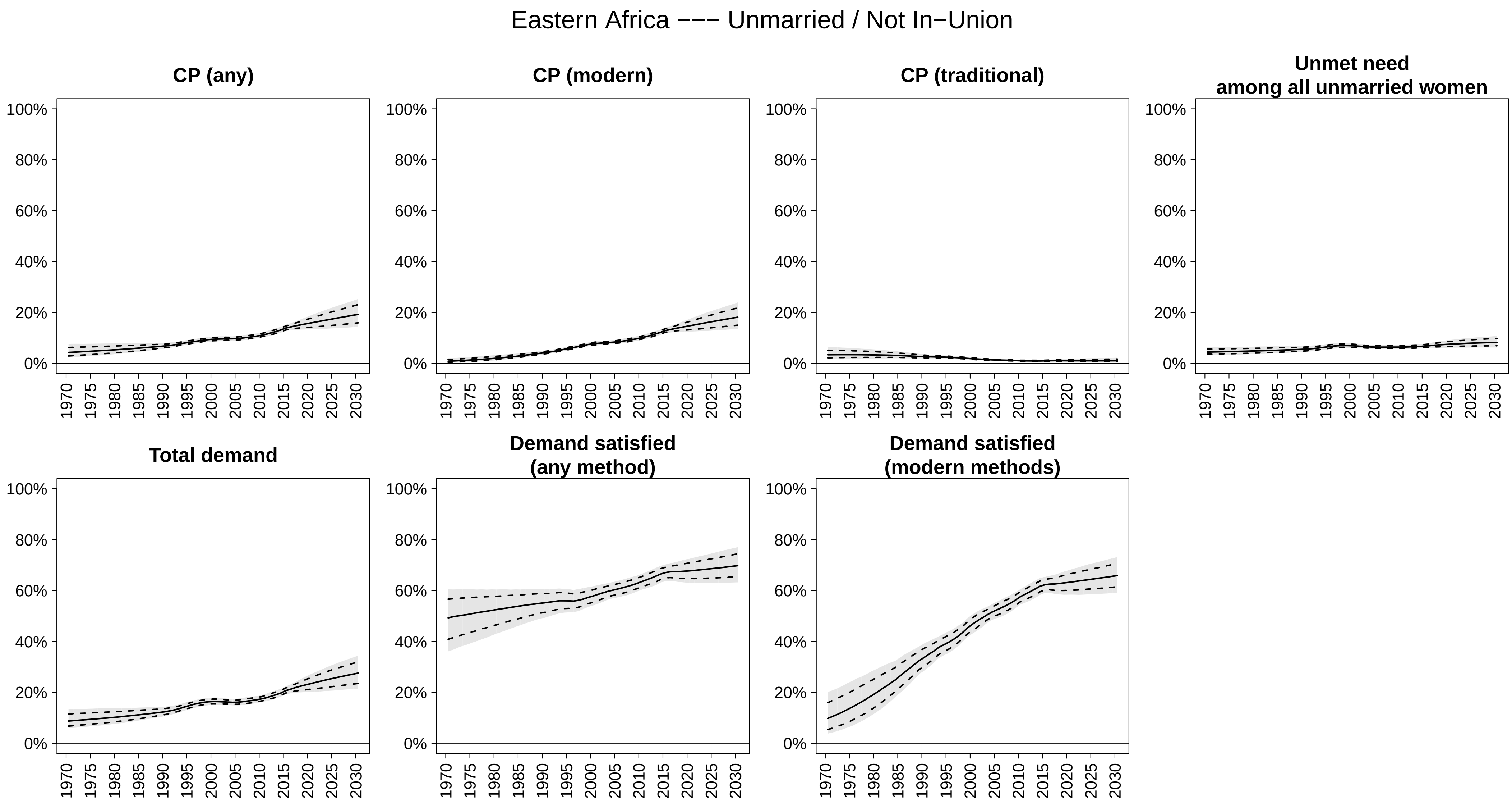
Eastern Africa −−− Unmarried / Not In−Union
CP (any)
CP (modern)
CP (traditional)
Unmet need among all unmarried women
Total demand
Demand satisfied (any method)
Demand satisfied (modern methods)
0%
20%
40%
60%
80%
100%
1970
1975
1980
1985
1990
1995
2000
2005
2010
2015
2020
2025
2030

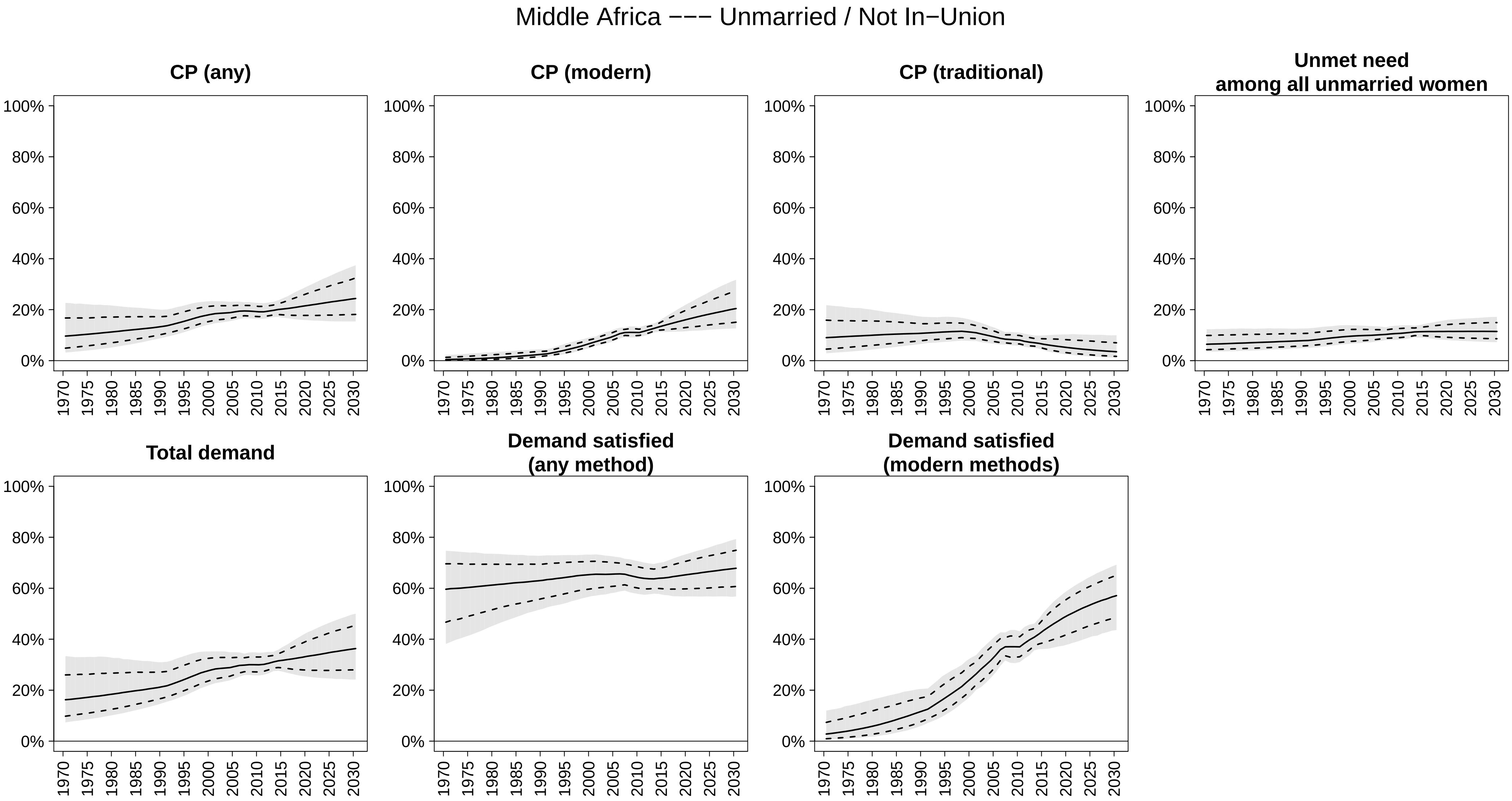
Middle Africa −−− Unmarried / Not In−Union
CP (any)
CP (modern)
CP (traditional)
Unmet need among all unmarried women
Total demand
Demand satisfied (any method)
Demand satisfied (modern methods)
0%
20%
40%
60%
80%
100%
1970
1975
1980
1985
1990
1995
2000
2005
2010
2015
2020
2025
2030

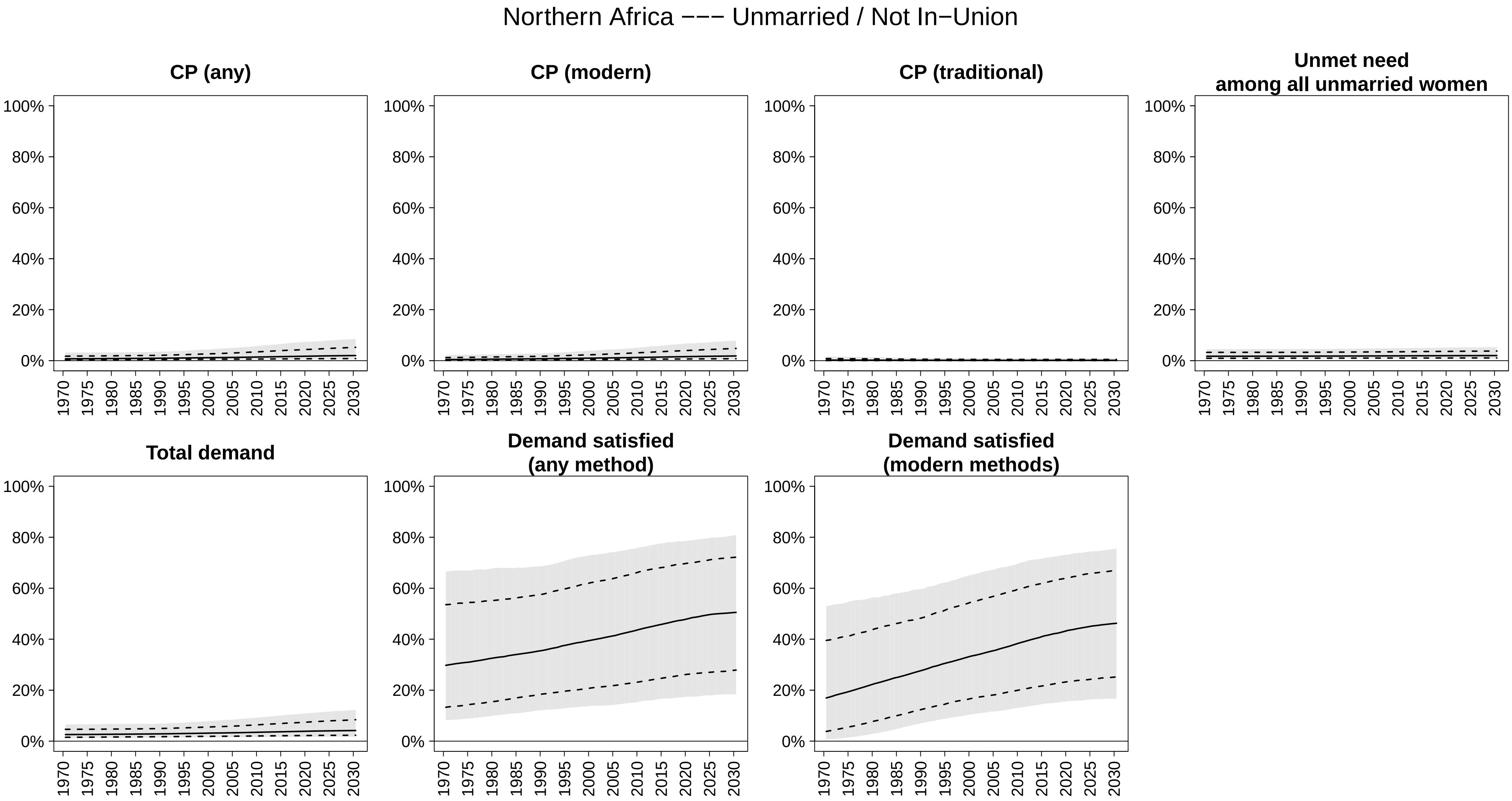
Northern Africa −−− Unmarried / Not In−Union
CP (any)
CP (modern)
CP (traditional)
Unmet need
among all unmarried women
Total demand
Demand satisfied
(any method)
Demand satisfied
(modern methods)
0%
20%
40%
60%
80%
100%
1970
1975
1980
1985
1990
1995
2000
2005
2010
2015
2020
2025
2030

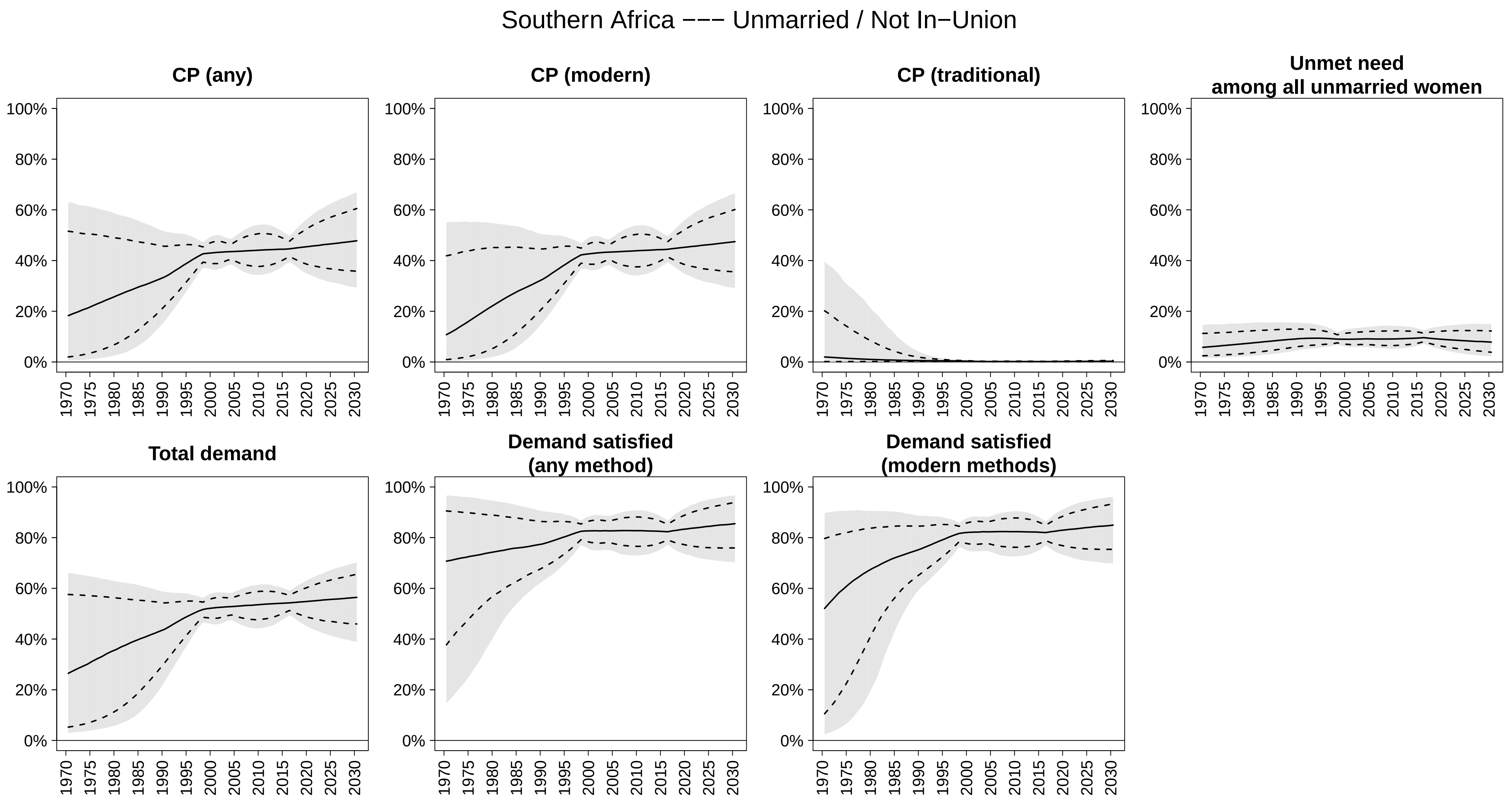
Southern Africa −−− Unmarried / Not In−Union
CP (any)
CP (modern)
CP (traditional)
Unmet need among all unmarried women
Total demand
Demand satisfied (any method)
Demand satisfied (modern methods)
0%
20%
40%
60%
80%
100%
1970
1975
1980
1985
1990
1995
2000
2005
2010
2015
2020
2025
2030

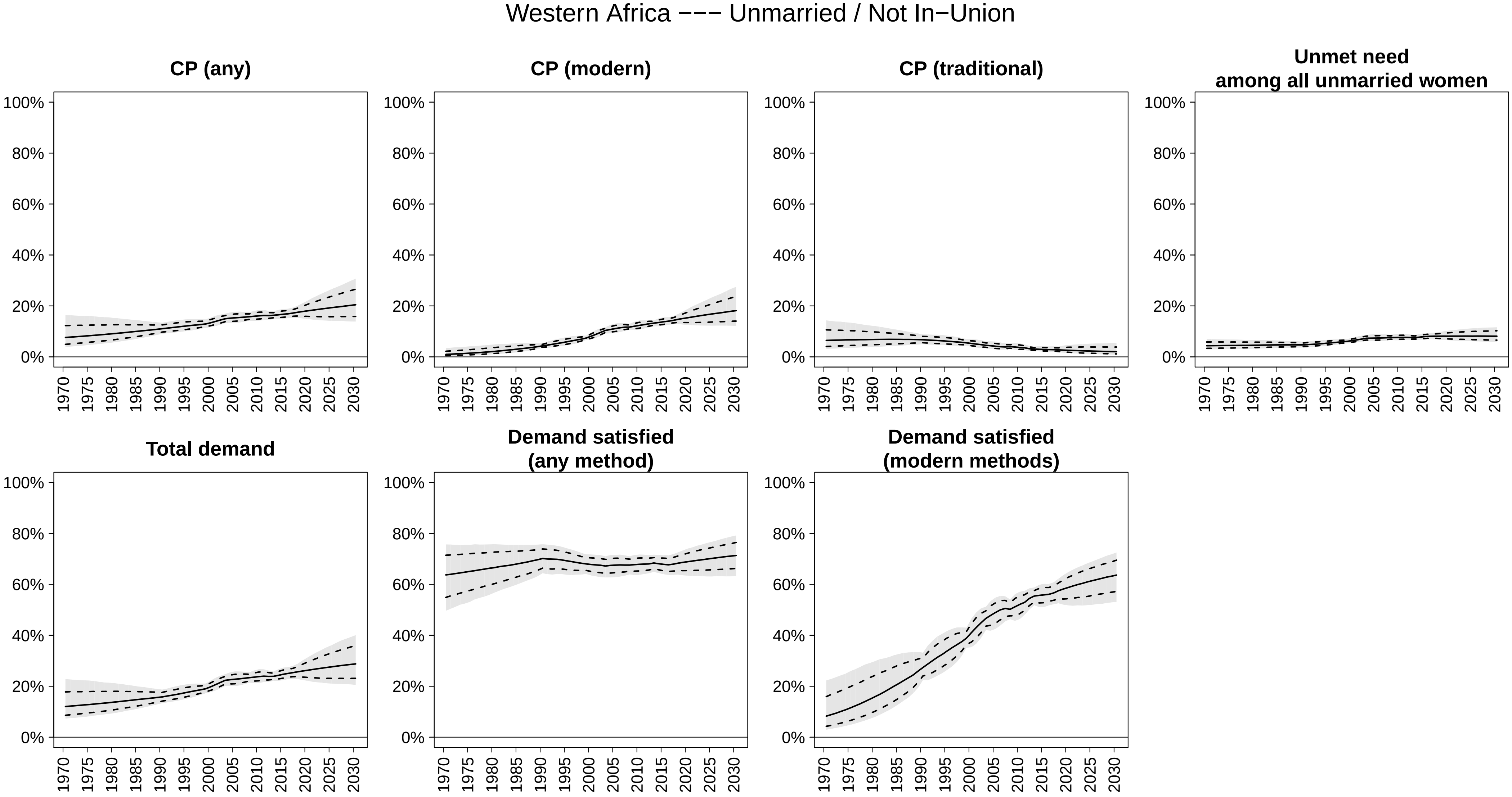
Western Africa −−− Unmarried / Not In−Union
CP (any)
CP (modern)
CP (traditional)
Unmet need among all unmarried women
Total demand
Demand satisfied (any method)
Demand satisfied (modern methods)
0%
20%
40%
60%
80%
100%
1970
1975
1980
1985
1990
1995
2000
2005
2010
2015
2020
2025
2030

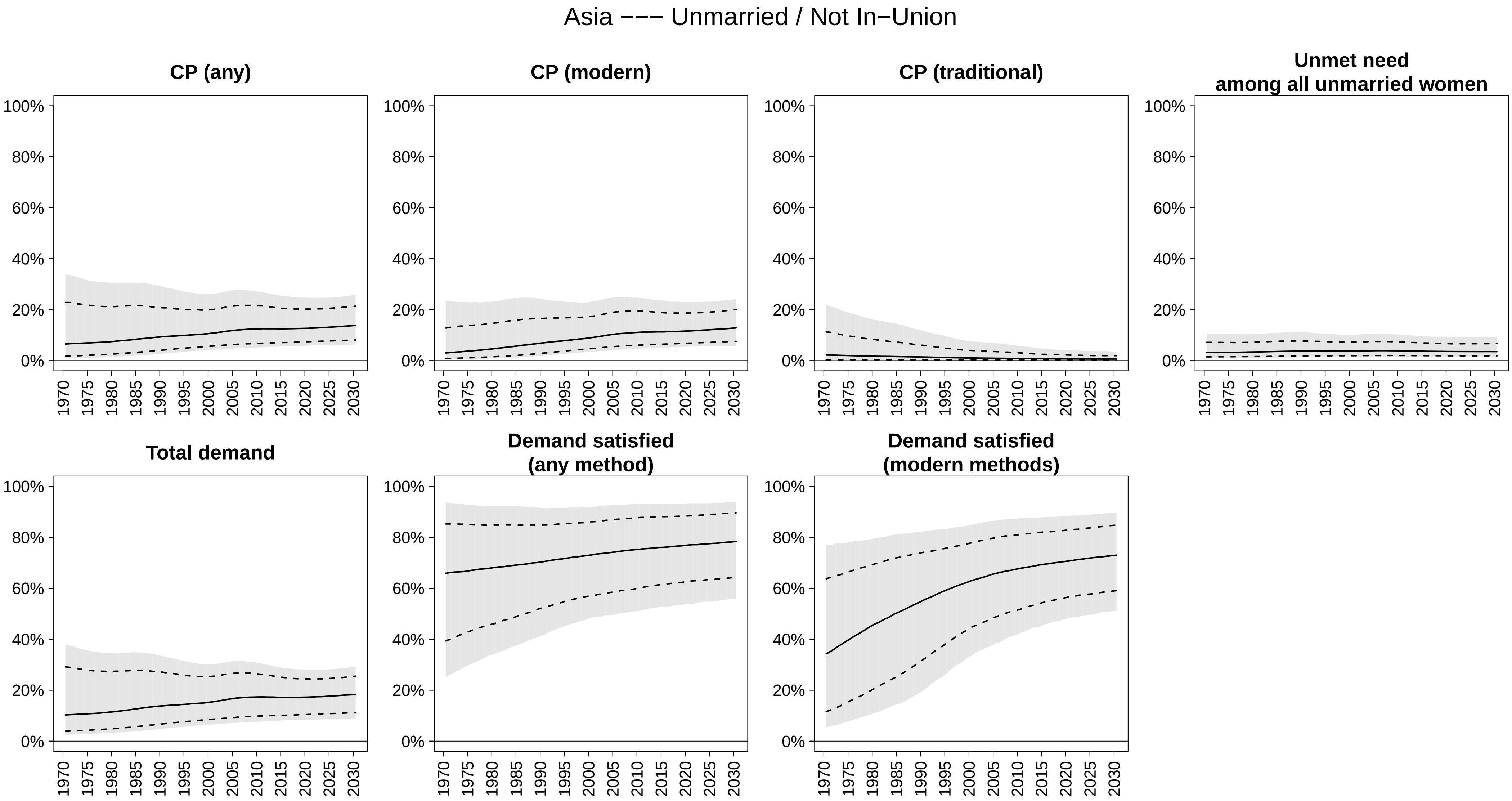
Asia --- Unmarried / Not In-Union
CP (any)
CP (modern)
CP (traditional)
Unmet need among all unmarried women
Total demand
Demand satisfied (any method)
Demand satisfied (modern methods)
0%
20%
40%
60%
80%
100%
1970
1975
1980
1985
1990
1995
2000
2005
2010
2015
2020
2025
2030

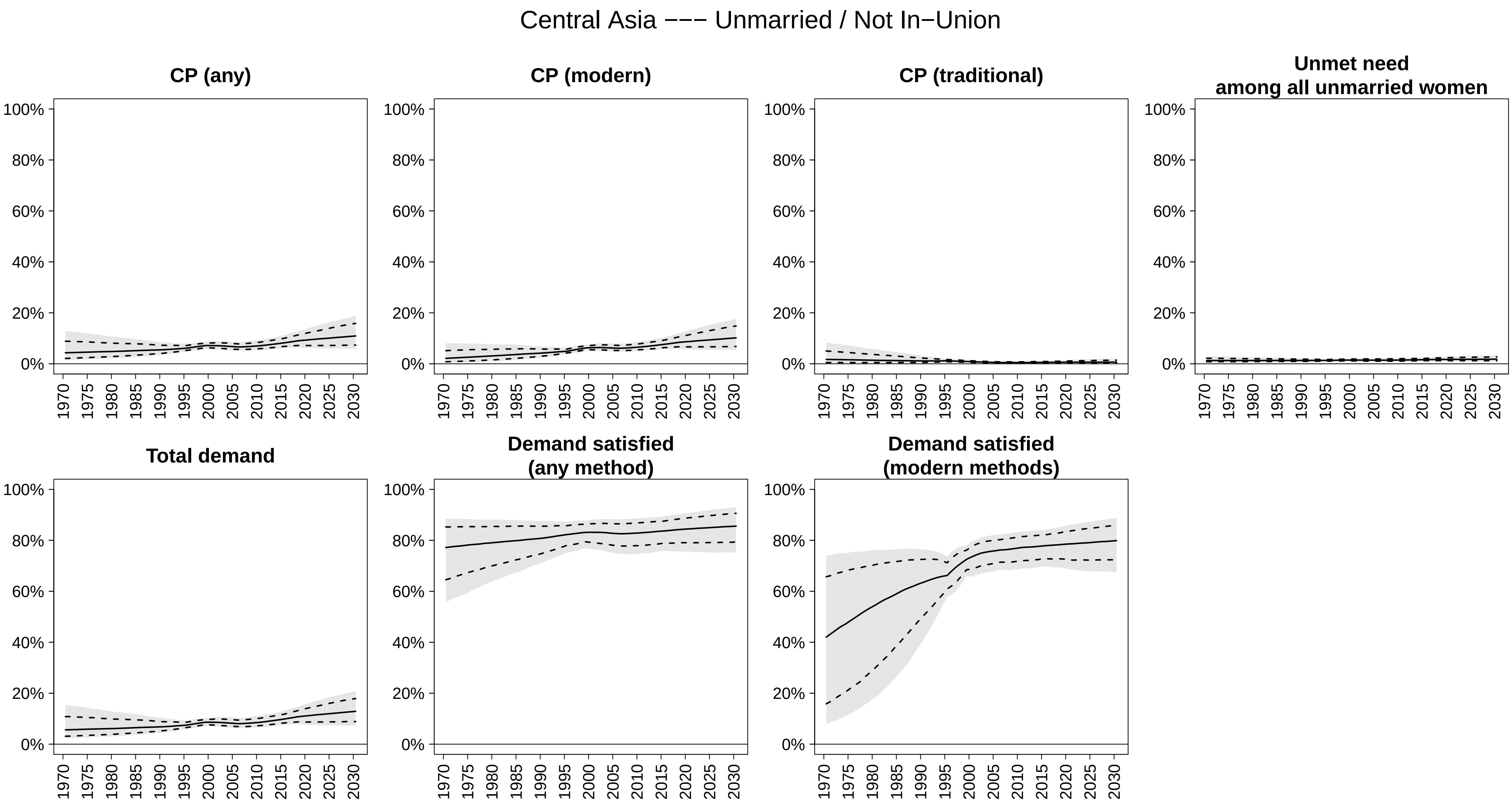
Central Asia --- Unmarried / Not In−Union
CP (any)
CP (modern)
CP (traditional)
Unmet need among all unmarried women
Total demand
Demand satisfied (any method)
Demand satisfied (modern methods)
0%
20%
40%
60%
80%
100%
1970
1975
1980
1985
1990
1995
2000
2005
2010
2015
2020
2025
2030

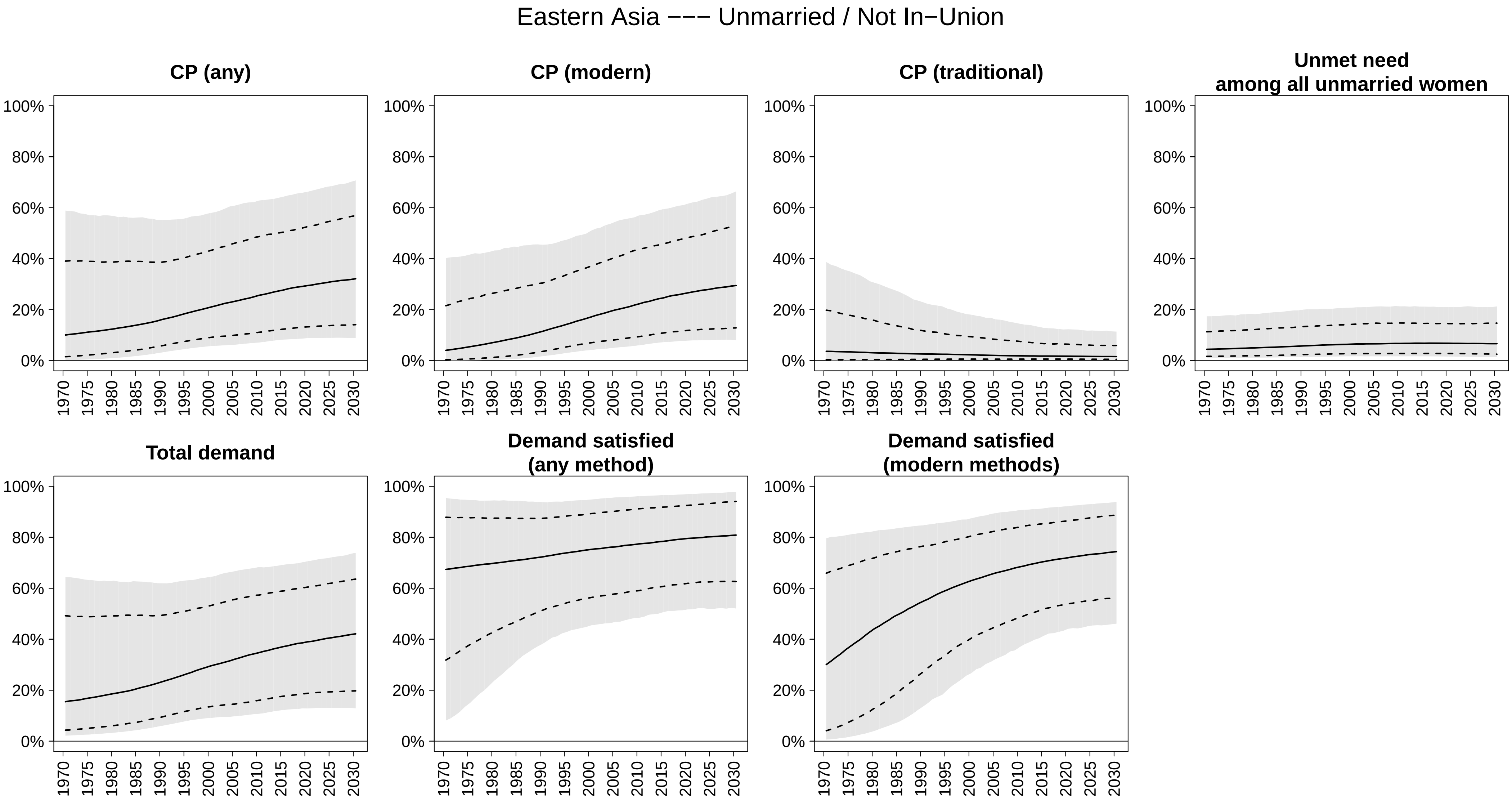
Eastern Asia −−− Unmarried / Not In−Union
CP (any)
CP (modern)
CP (traditional)
Unmet need among all unmarried women
Total demand
Demand satisfied (any method)
Demand satisfied (modern methods)
0%
20%
40%
60%
80%
100%
1970
1975
1980
1985
1990
1995
2000
2005
2010
2015
2020
2025
2030

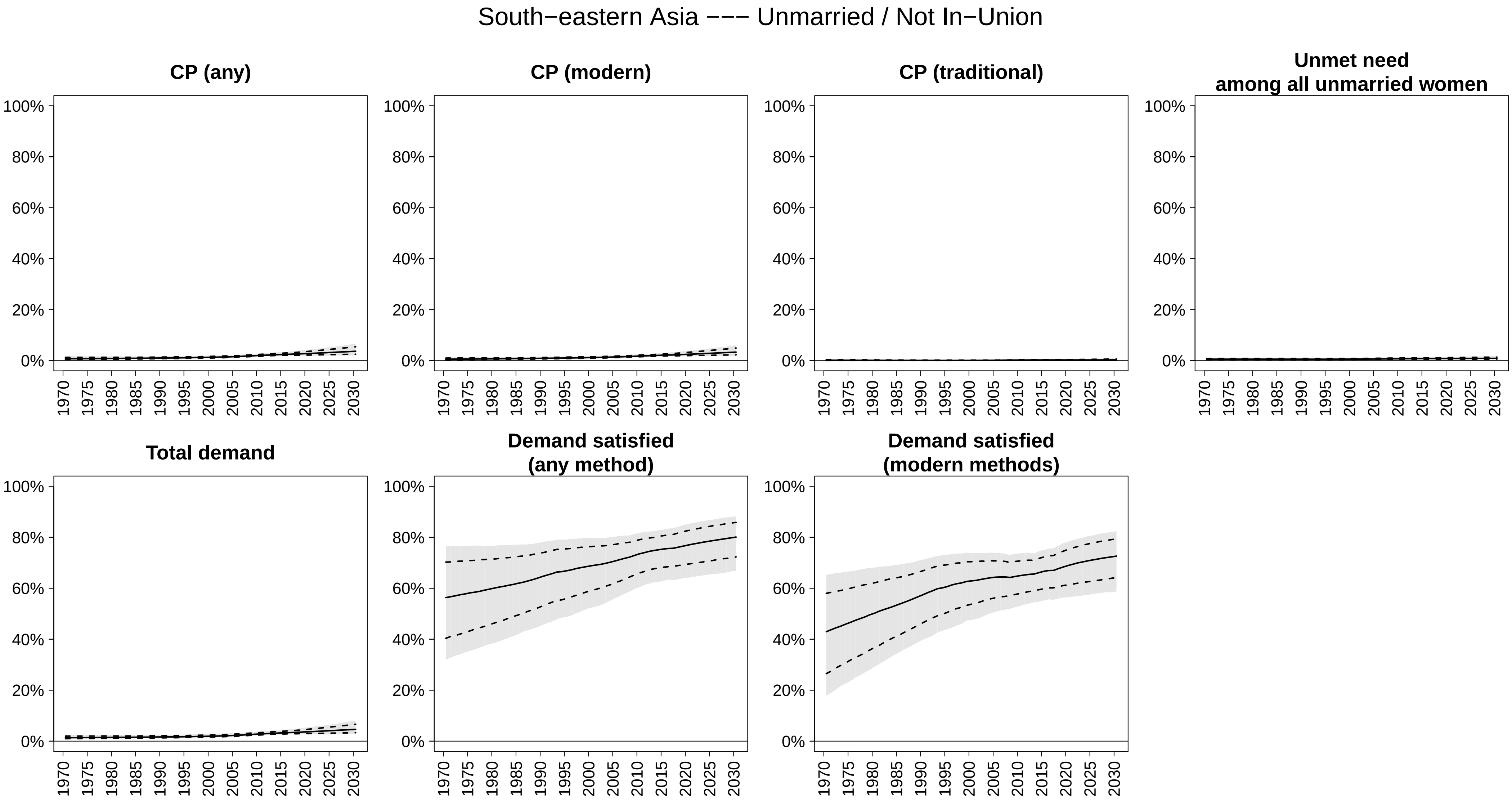
South−eastern Asia −−− Unmarried / Not In−Union
CP (any)
CP (modern)
CP (traditional)
Unmet need among all unmarried women
Total demand
Demand satisfied (any method)
Demand satisfied (modern methods)
0%
20%
40%
60%
80%
100%
1970
1975
1980
1985
1990
1995
2000
2005
2010
2015
2020
2025
2030

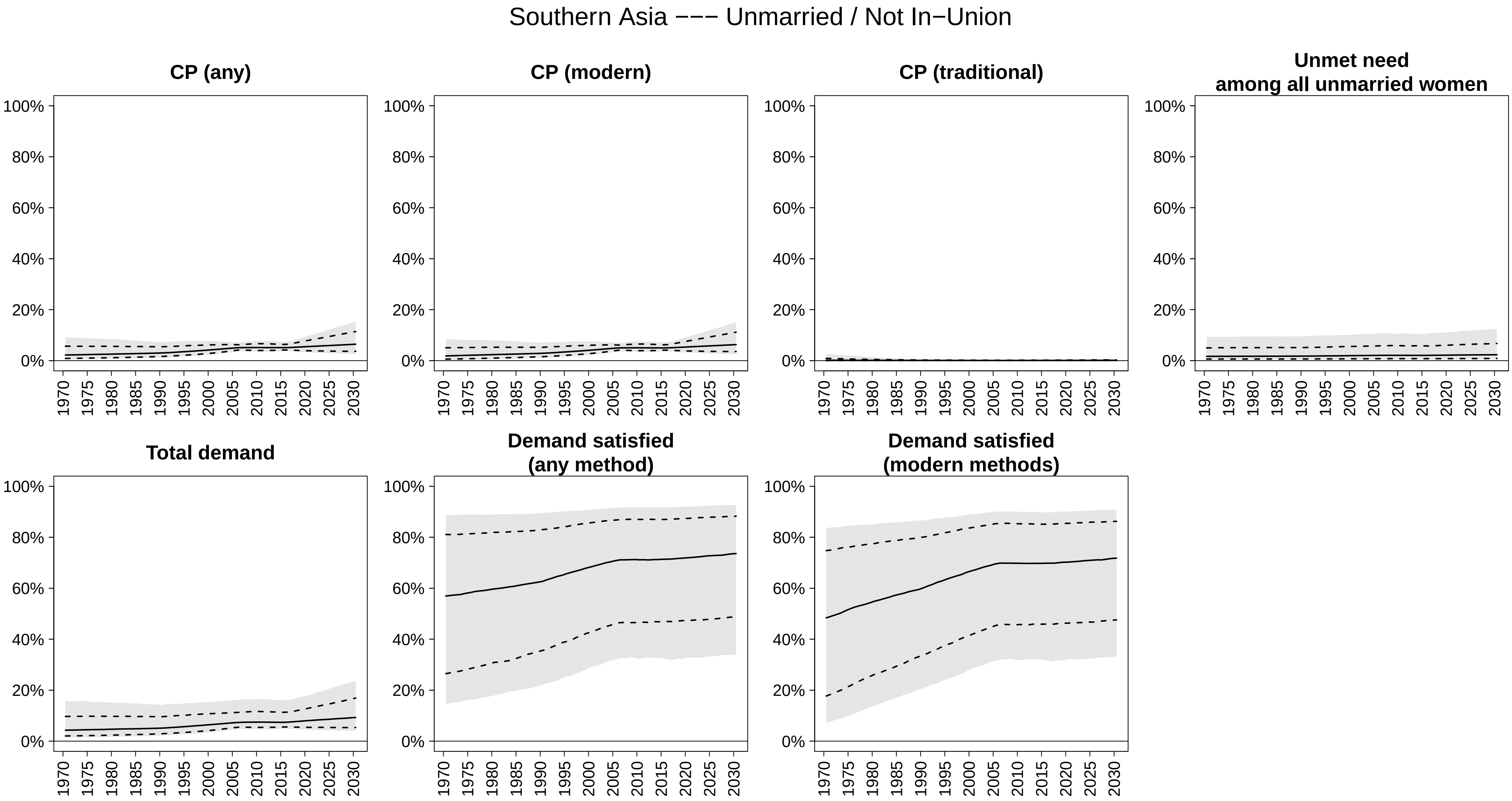
Southern Asia −−− Unmarried / Not In−Union
CP (any)
CP (modern)
CP (traditional)
Unmet need among all unmarried women
Total demand
Demand satisfied (any method)
Demand satisfied (modern methods)
0%
20%
40%
60%
80%
100%
1970
1975
1980
1985
1990
1995
2000
2005
2010
2015
2020
2025
2030

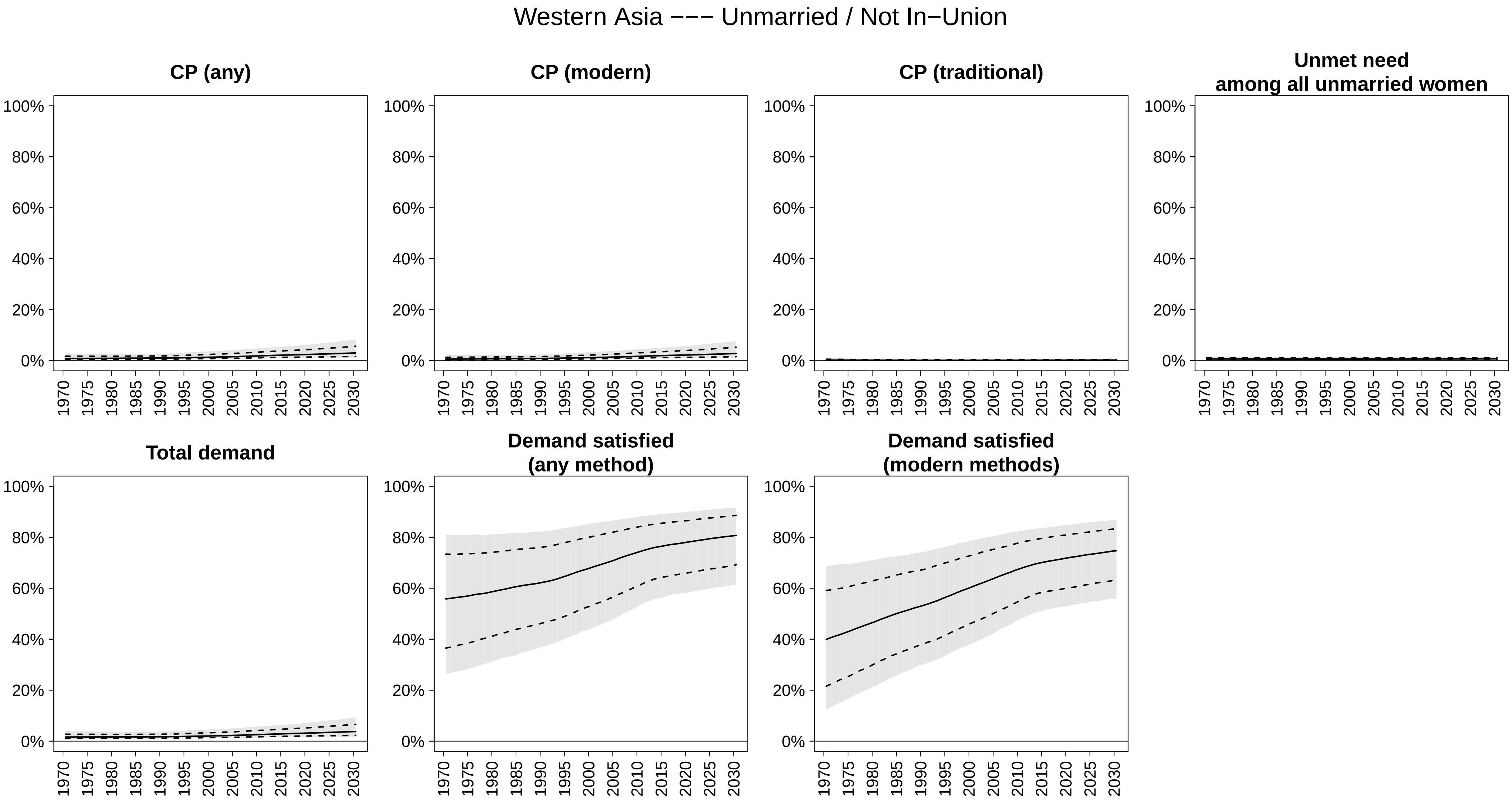
Western Asia −−− Unmarried / Not In−Union
CP (any)
CP (modern)
CP (traditional)
Unmet need
among all unmarried women
Total demand
Demand satisfied
(any method)
Demand satisfied
(modern methods)
100%
80%
60%
40%
20%
0%
1970
1975
1980
1985
1990
1995
2000
2005
2010
2015
2020
2025
2030

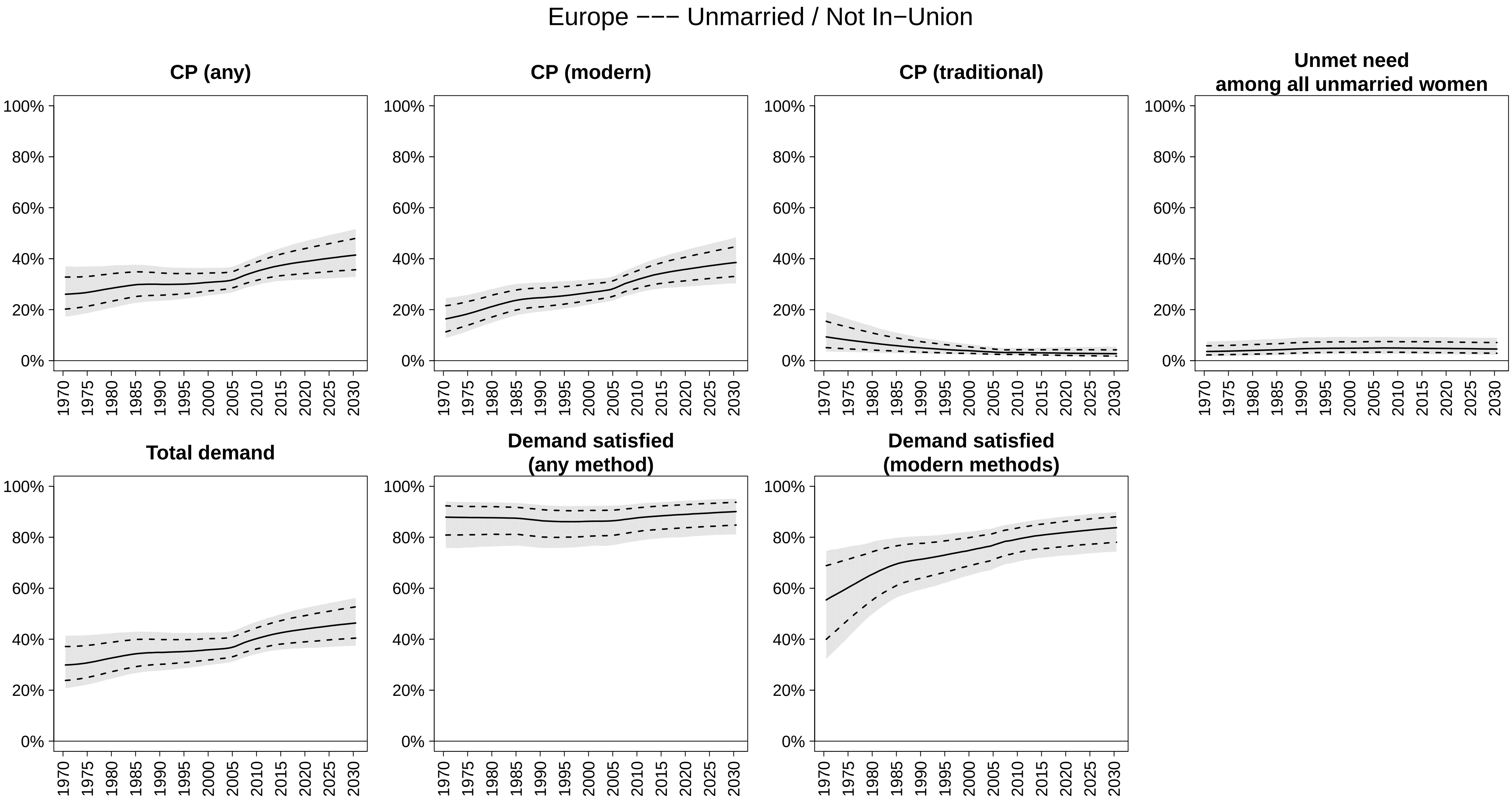
Europe −−− Unmarried / Not In−Union
CP (any)
CP (modern)
CP (traditional)
Unmet need among all unmarried women
Total demand
Demand satisfied (any method)
Demand satisfied (modern methods)
0%
20%
40%
60%
80%
100%
1970
1975
1980
1985
1990
1995
2000
2005
2010
2015
2020
2025
2030

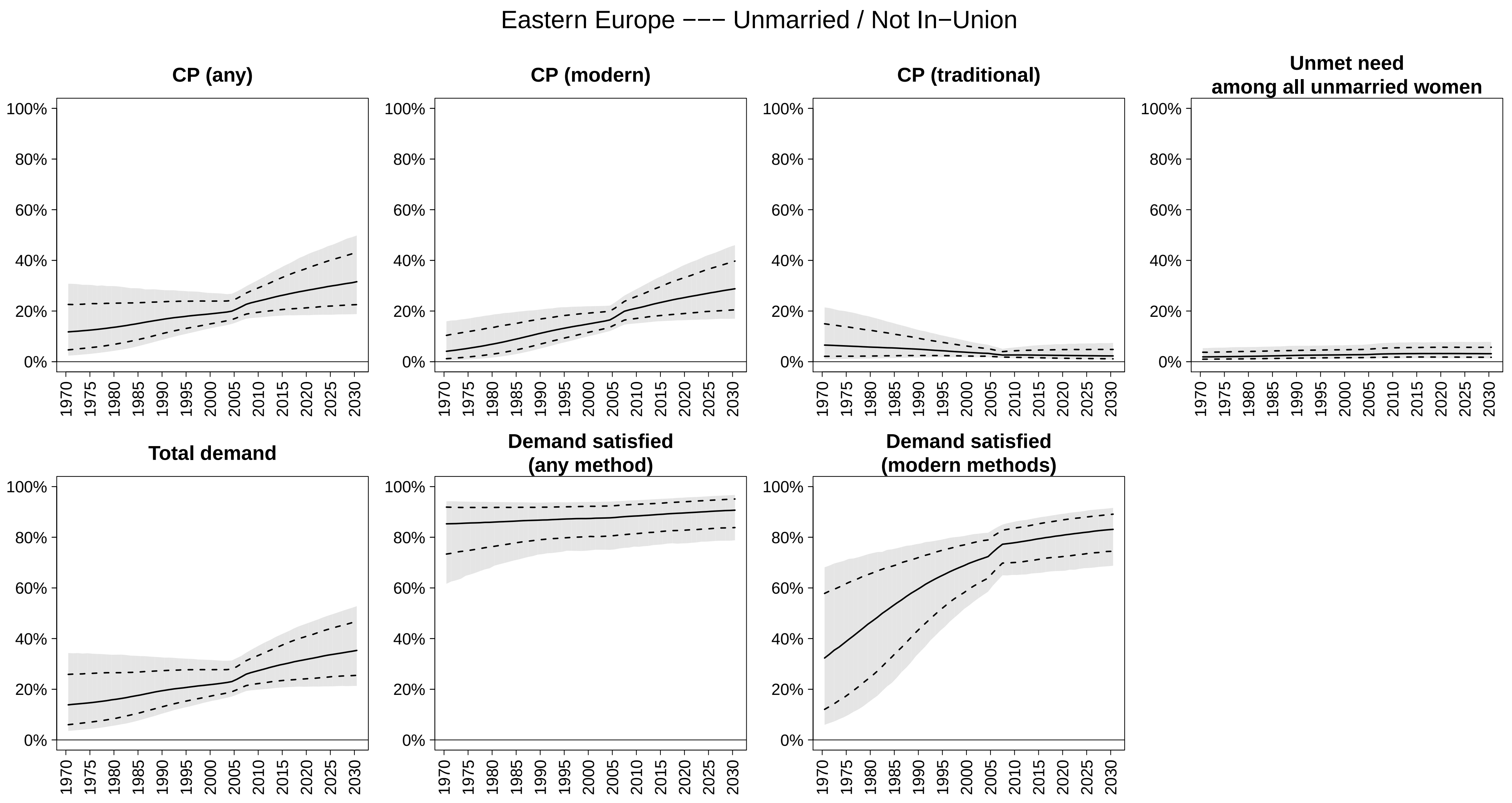
Eastern Europe −−− Unmarried / Not In−Union
CP (any)
CP (modern)
CP (traditional)
Unmet need
among all unmarried women
Total demand
Demand satisfied
(any method)
Demand satisfied
(modern methods)
0%
20%
40%
60%
80%
100%
1970
1975
1980
1985
1990
1995
2000
2005
2010
2015
2020
2025
2030

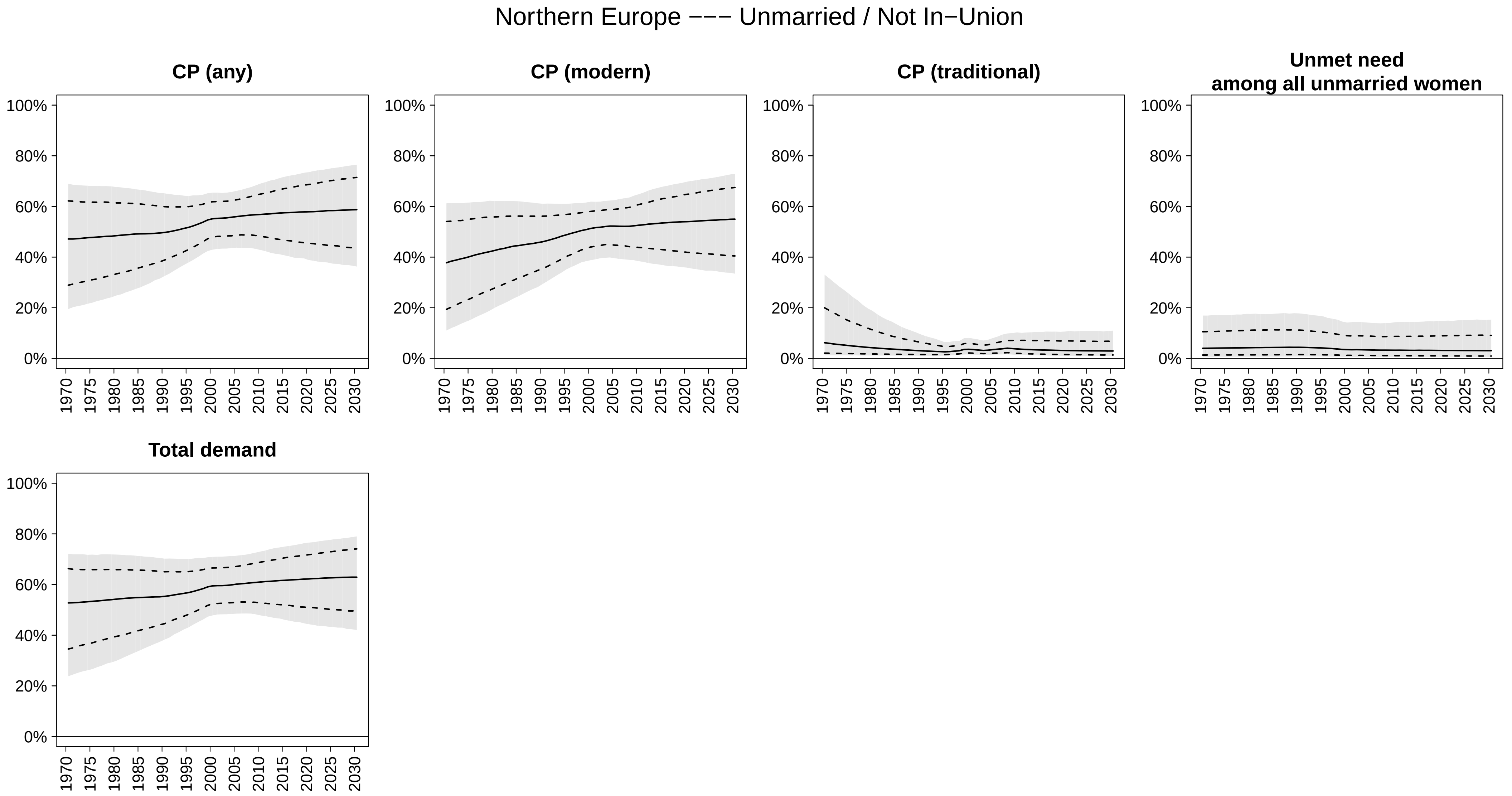
Northern Europe −−− Unmarried / Not In−Union
CP (any)
CP (modern)
CP (traditional)
Unmet need among all unmarried women
Total demand
100%
80%
60%
40%
20%
0%
1970
1975
1980
1985
1990
1995
2000
2005
2010
2015
2020
2025
2030

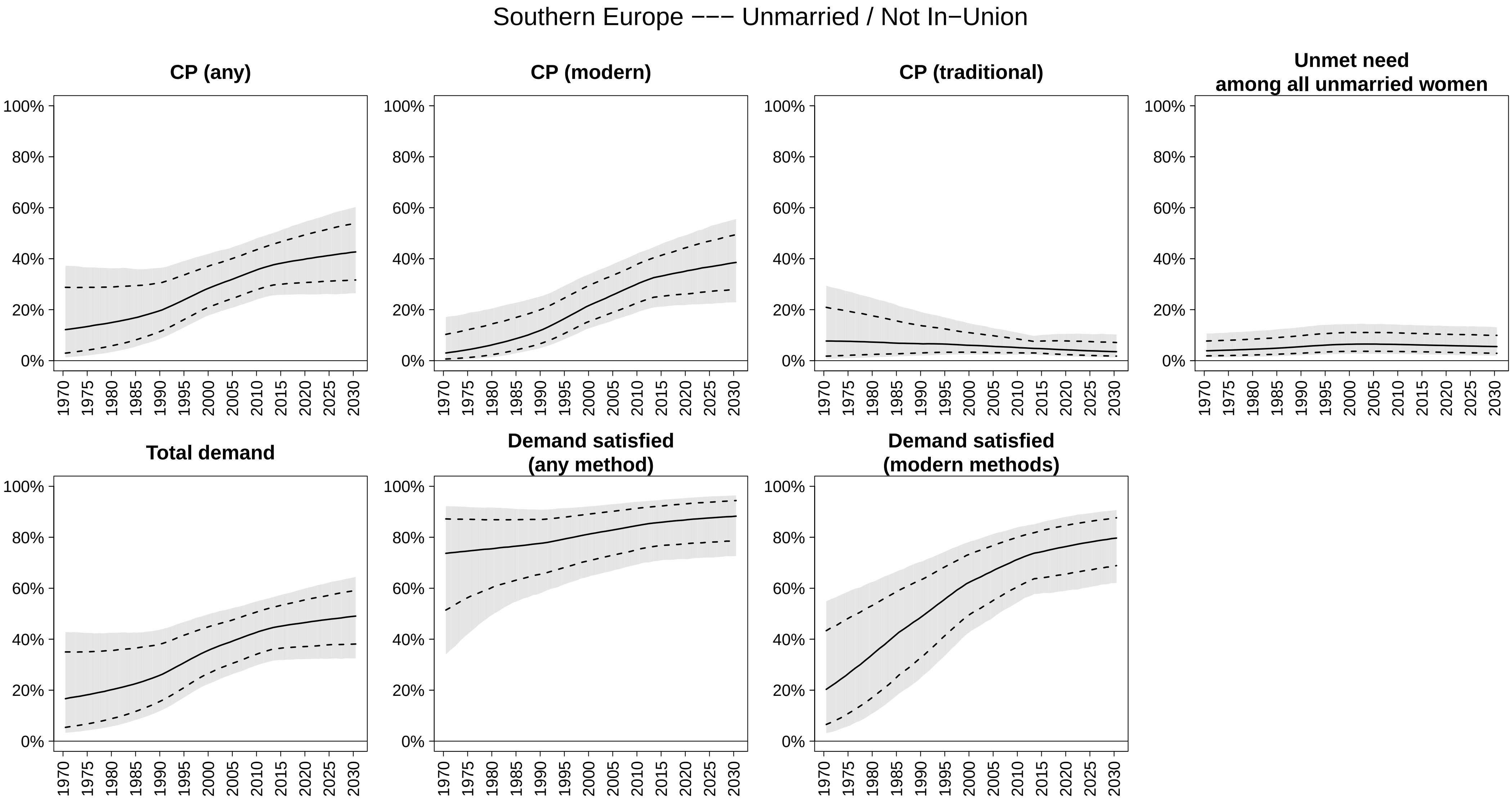
Southern Europe −−− Unmarried / Not In−Union
CP (any)
CP (modern)
CP (traditional)
Unmet need among all unmarried women
Total demand
Demand satisfied (any method)
Demand satisfied (modern methods)
0%
20%
40%
60%
80%
100%
1970
1975
1980
1985
1990
1995
2000
2005
2010
2015
2020
2025
2030

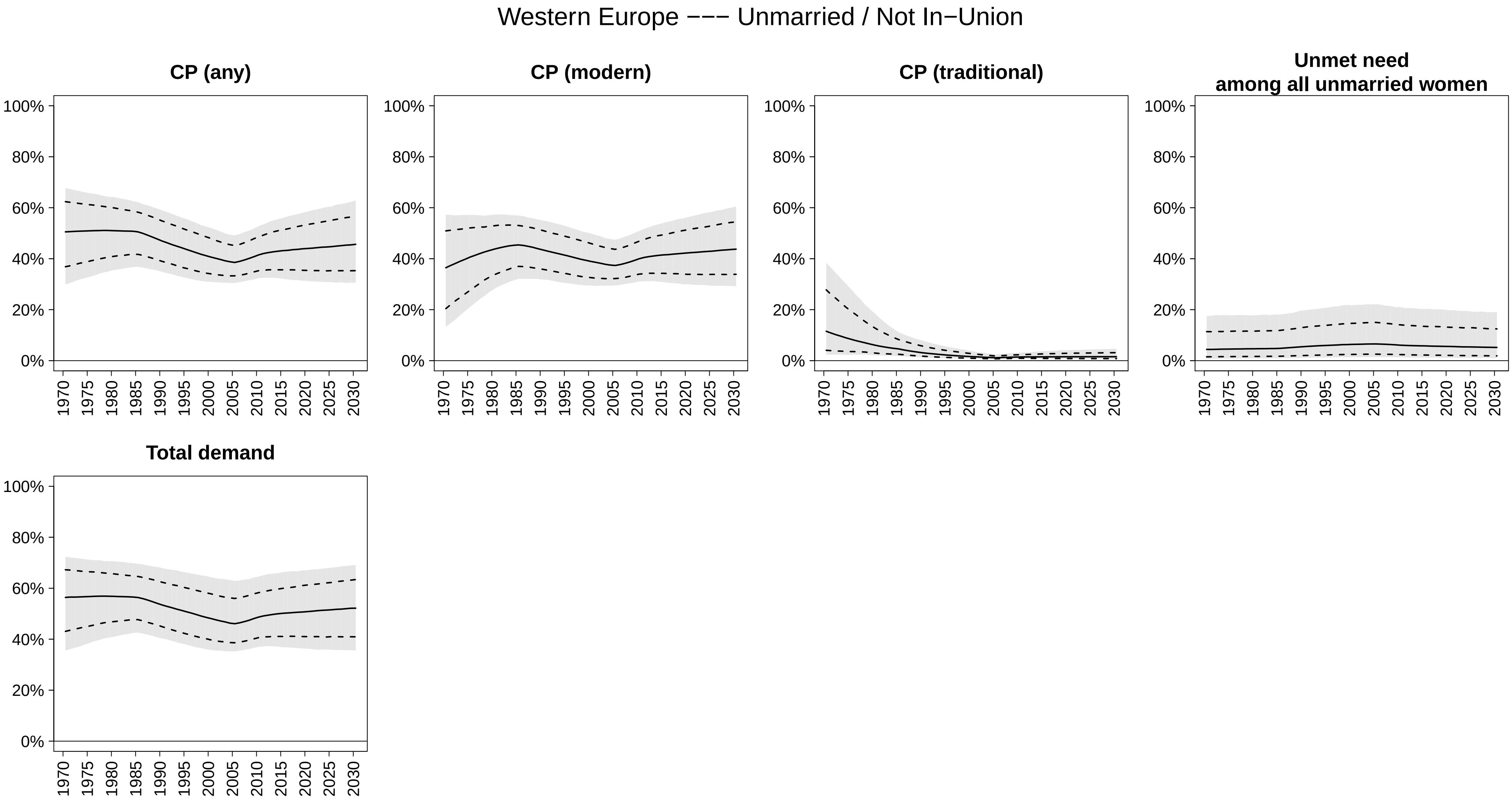
Western Europe −−− Unmarried / Not In−Union
CP (any)
CP (modern)
CP (traditional)
Unmet need among all unmarried women
Total demand
100%
80%
60%
40%
20%
0%
1970
1975
1980
1985
1990
1995
2000
2005
2010
2015
2020
2025
2030

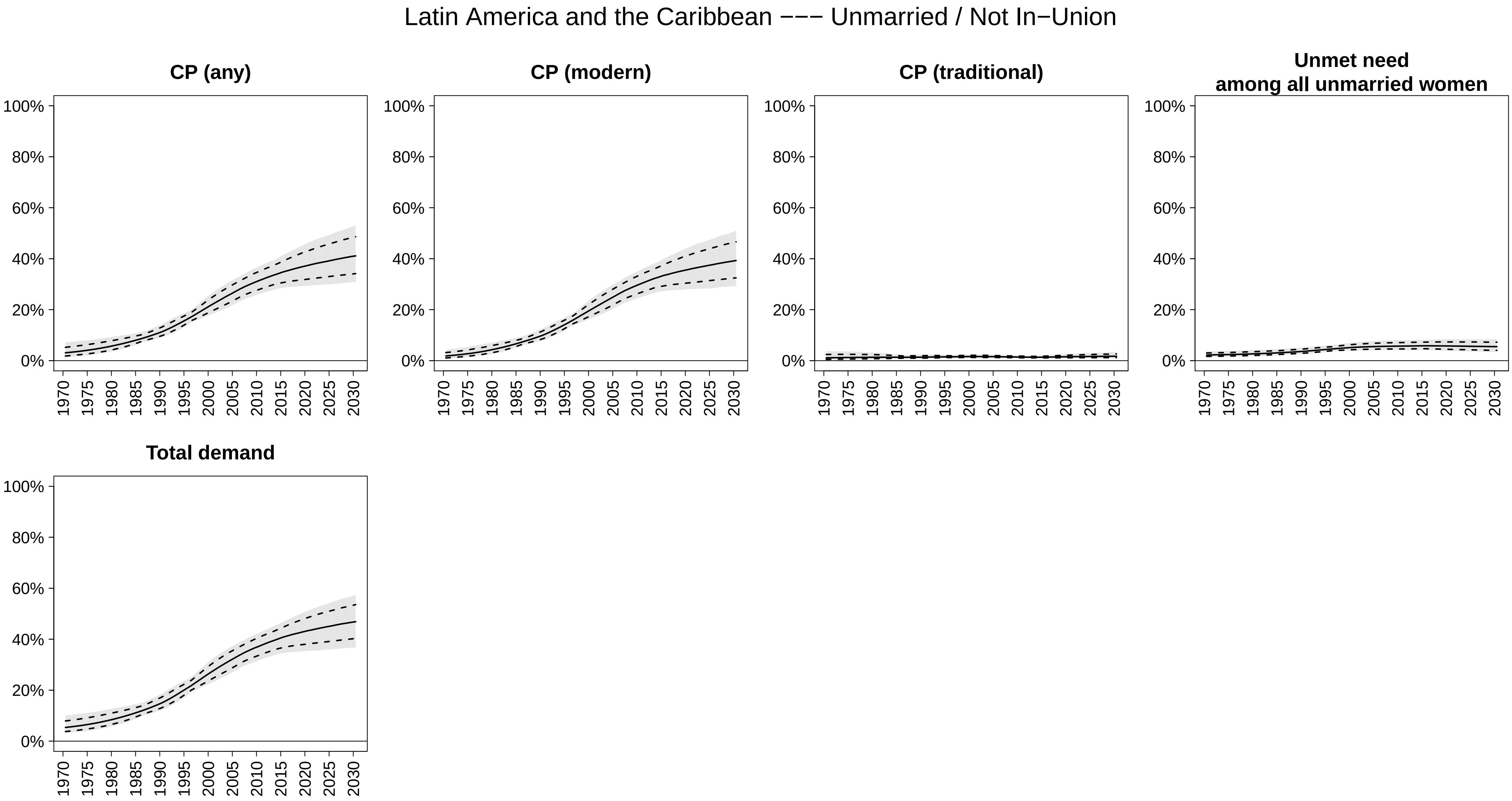
Latin America and the Caribbean −−− Unmarried / Not In−Union
CP (any)
CP (modern)
CP (traditional)
Unmet need
among all unmarried women
Total demand
0%
20%
40%
60%
80%
100%
1970
1975
1980
1985
1990
1995
2000
2005
2010
2015
2020
2025
2030

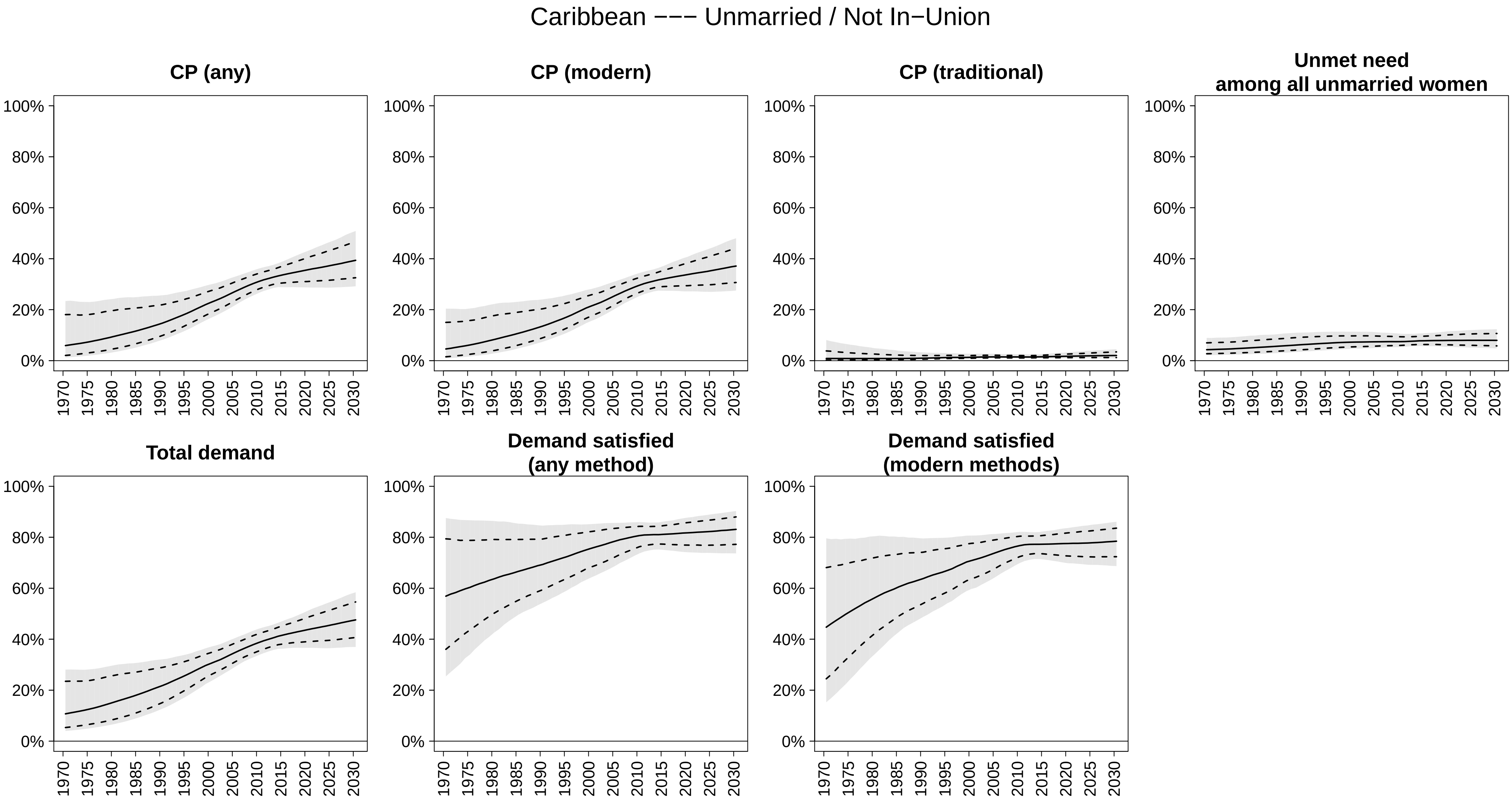
Caribbean −−− Unmarried / Not In−Union
CP (any)
CP (modern)
CP (traditional)
Unmet need
among all unmarried women
Total demand
Demand satisfied
(any method)
Demand satisfied
(modern methods)
0%
20%
40%
60%
80%
100%
1970
1975
1980
1985
1990
1995
2000
2005
2010
2015
2020
2025
2030

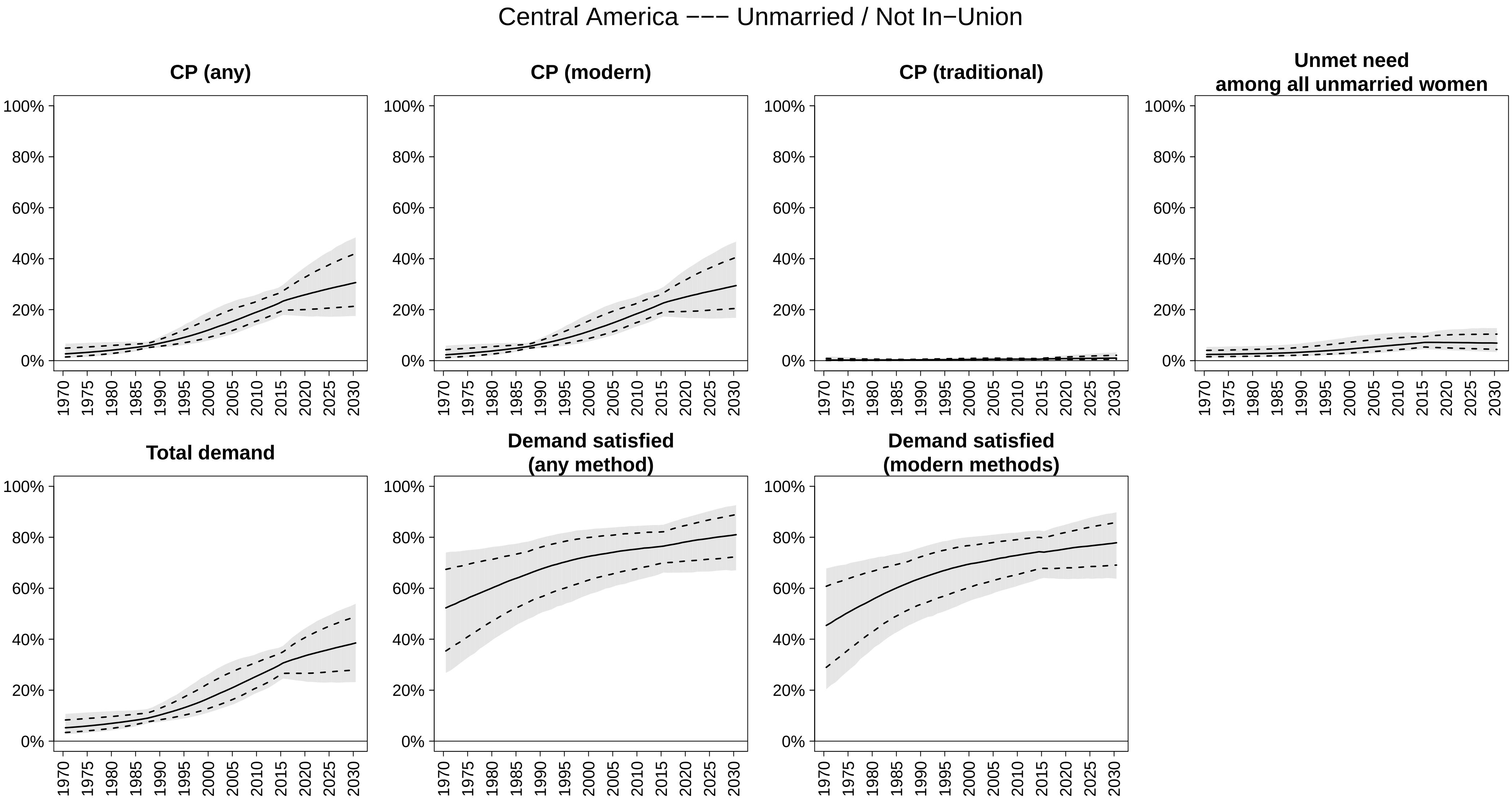
Central America --- Unmarried / Not In−Union
CP (any)
CP (modern)
CP (traditional)
Unmet need among all unmarried women
Total demand
Demand satisfied (any method)
Demand satisfied (modern methods)
0%
20%
40%
60%
80%
100%
1970
1975
1980
1985
1990
1995
2000
2005
2010
2015
2020
2025
2030

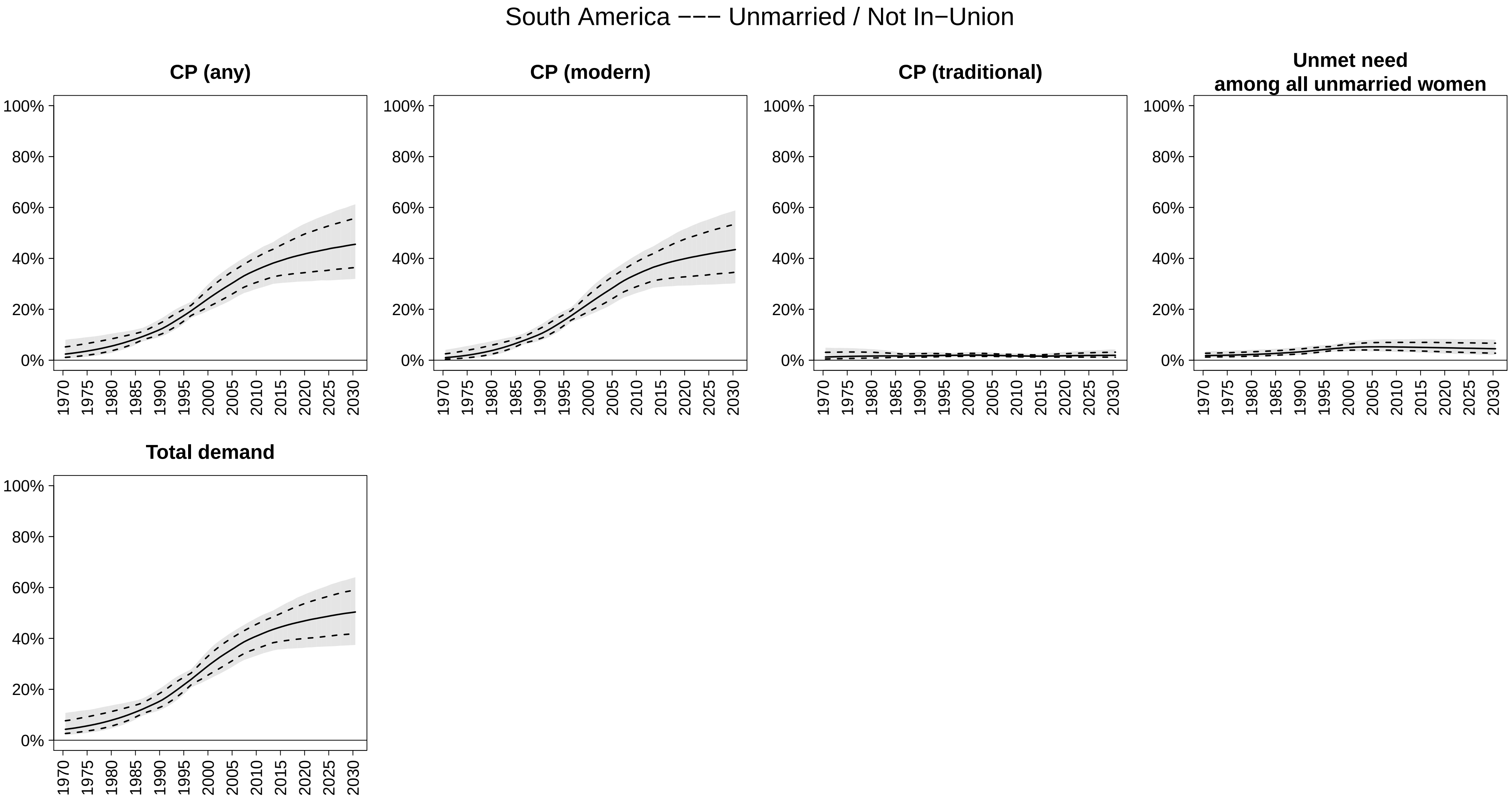
South America −−− Unmarried / Not In−Union
CP (any)
CP (modern)
CP (traditional)
Unmet need
among all unmarried women
Total demand
100%
80%
60%
40%
20%
0%
1970
1975
1980
1985
1990
1995
2000
2005
2010
2015
2020
2025
2030

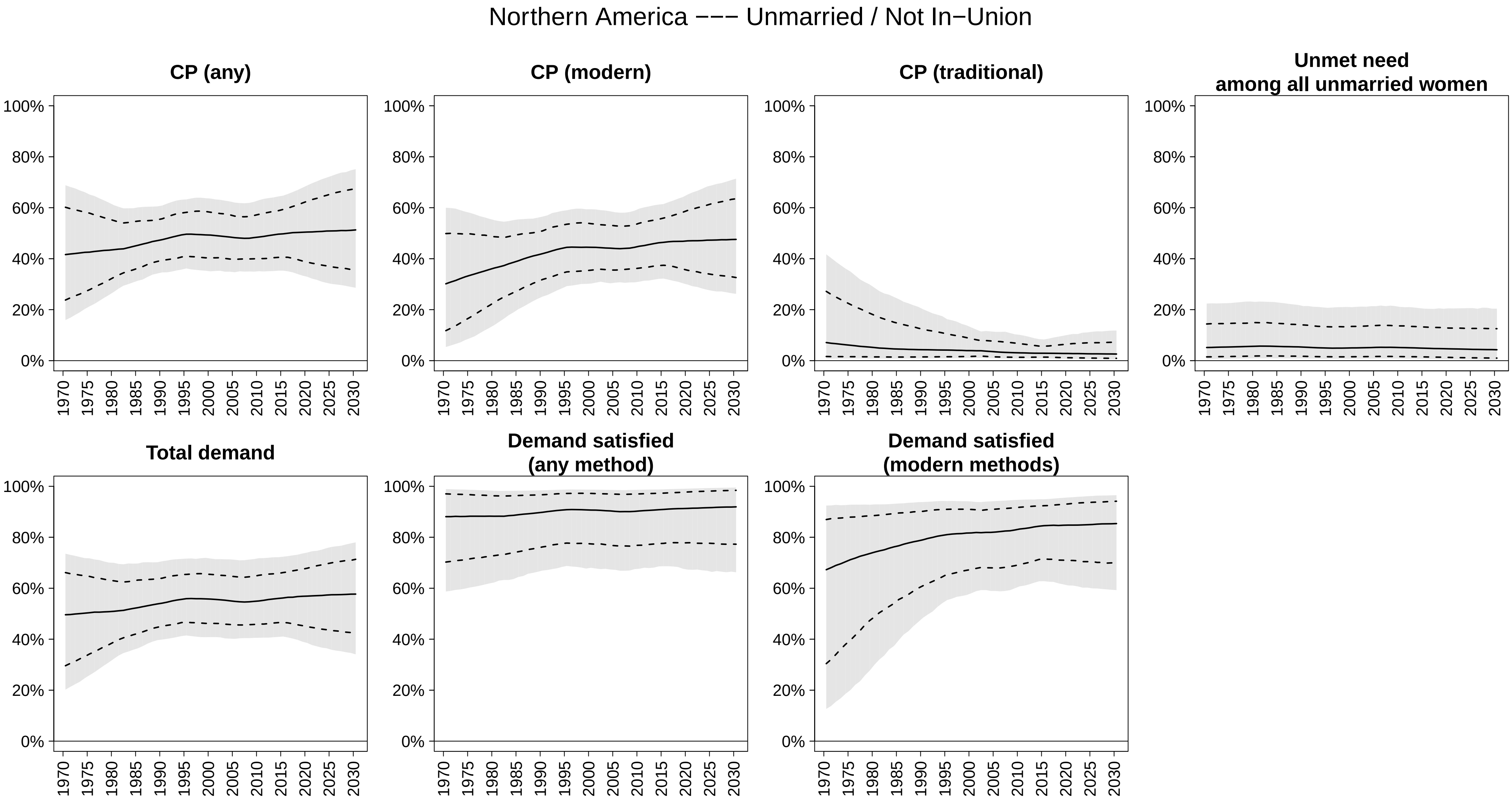
Northern America −−− Unmarried / Not In−Union
CP (any)
CP (modern)
CP (traditional)
Unmet need among all unmarried women
Total demand
Demand satisfied (any method)
Demand satisfied (modern methods)
0%
20%
40%
60%
80%
100%
1970
1975
1980
1985
1990
1995
2000
2005
2010
2015
2020
2025
2030

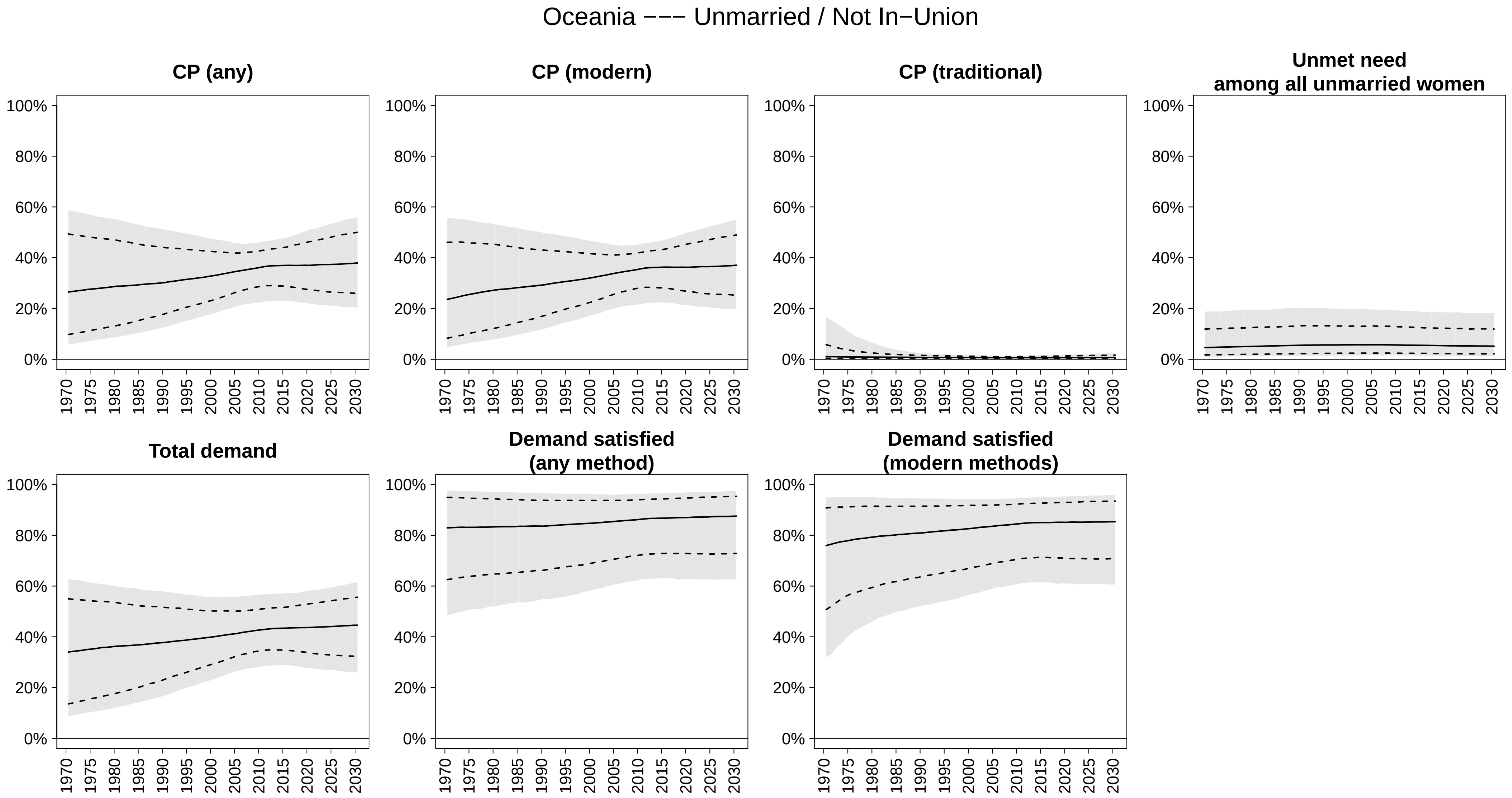
Oceania −−− Unmarried / Not In−Union
CP (any)
CP (modern)
CP (traditional)
Unmet need
among all unmarried women
Total demand
Demand satisfied
(any method)
Demand satisfied
(modern methods)
0%
20%
40%
60%
80%
100%
1970
1975
1980
1985
1990
1995
2000
2005
2010
2015
2020
2025
2030

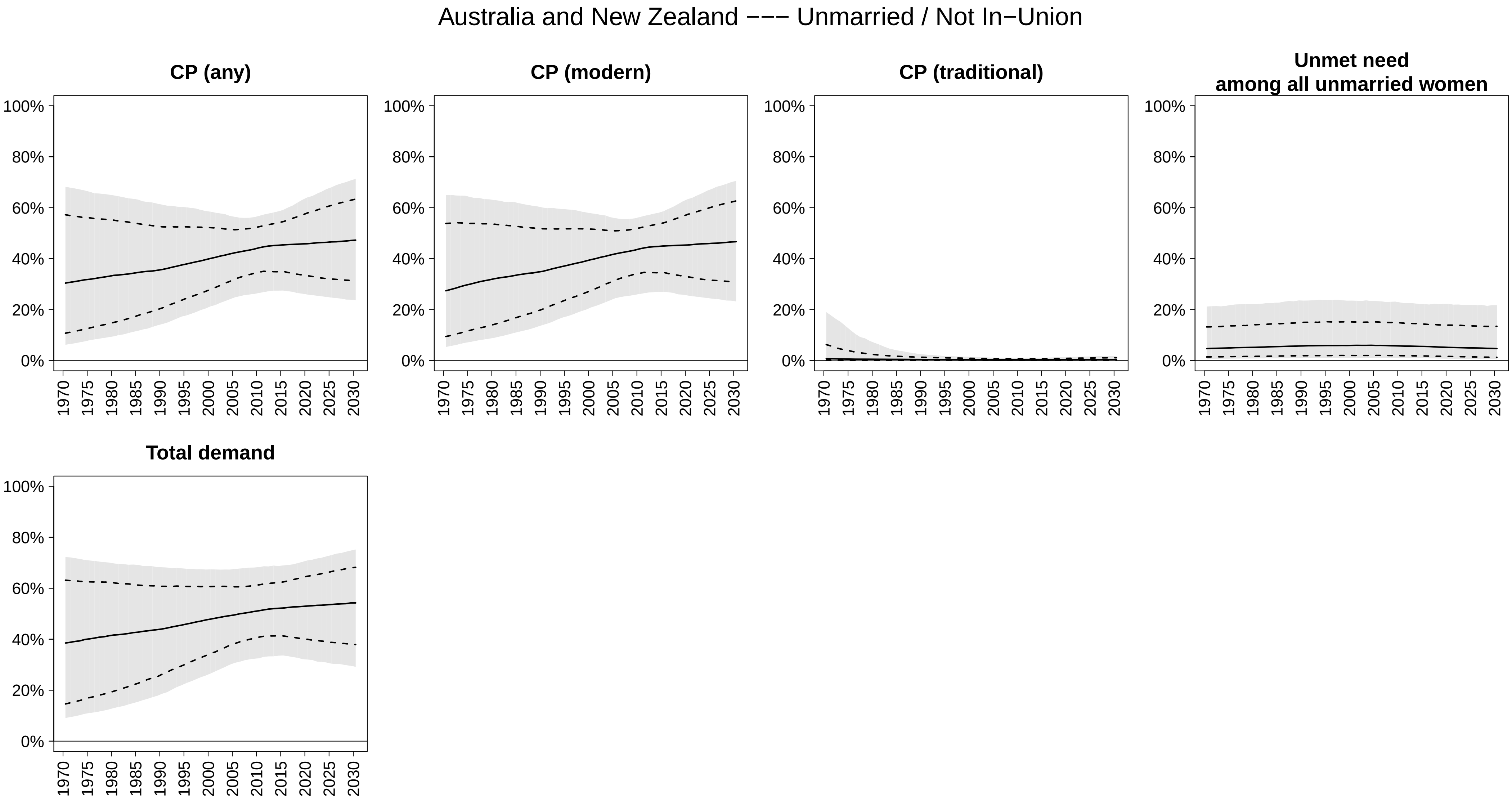
Australia and New Zealand −−− Unmarried / Not In−Union
CP (any)
CP (modern)
CP (traditional)
Unmet need
among all unmarried women
Total demand
100%
80%
60%
40%
20%
0%
1970
1975
1980
1985
1990
1995
2000
2005
2010
2015
2020
2025
2030

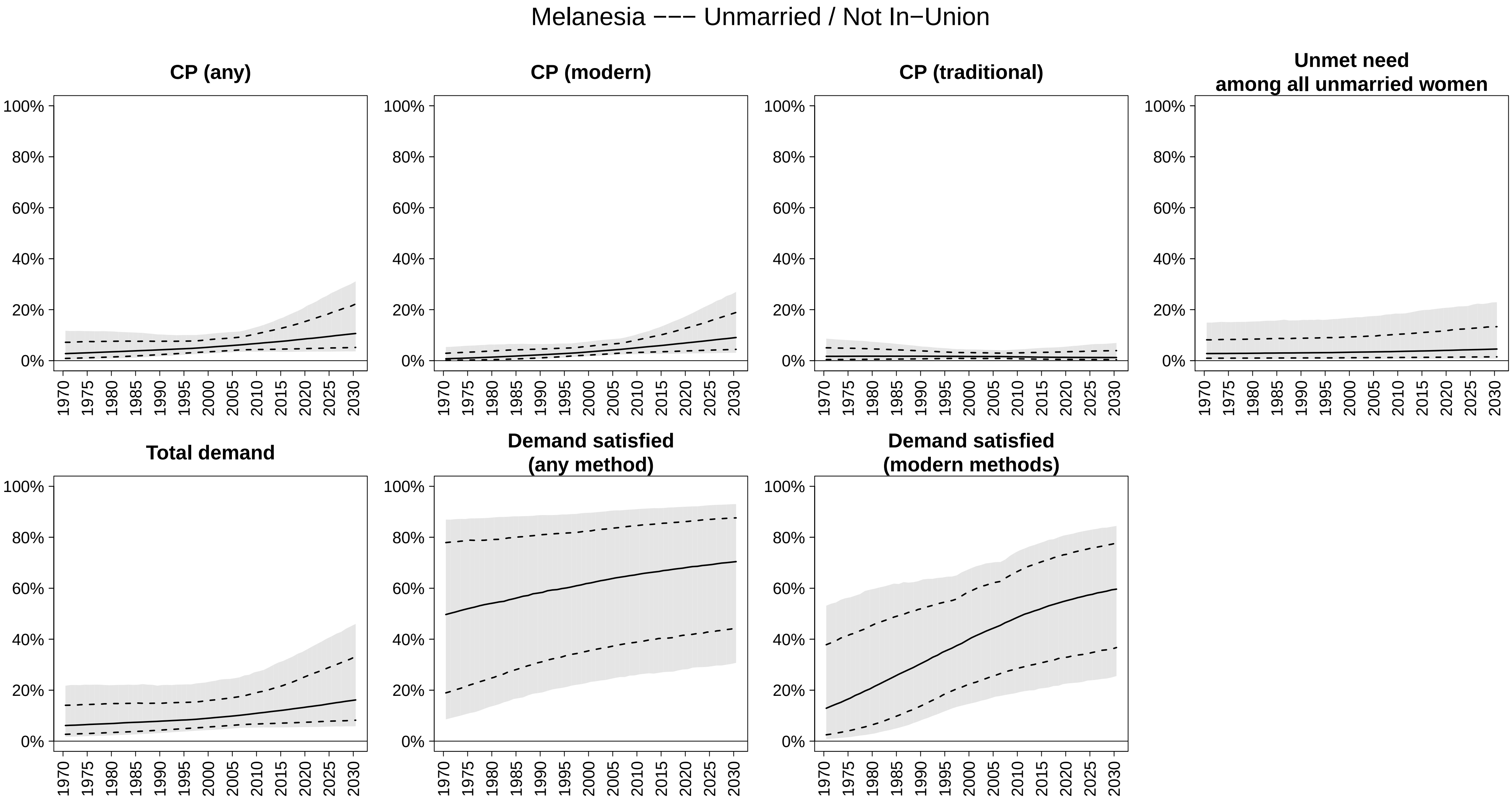
Melanesia −−− Unmarried / Not In−Union
CP (any)
CP (modern)
CP (traditional)
Unmet need among all unmarried women
Total demand
Demand satisfied (any method)
Demand satisfied (modern methods)
0%
20%
40%
60%
80%
100%
1970
1975
1980
1985
1990
1995
2000
2005
2010
2015
2020
2025
2030

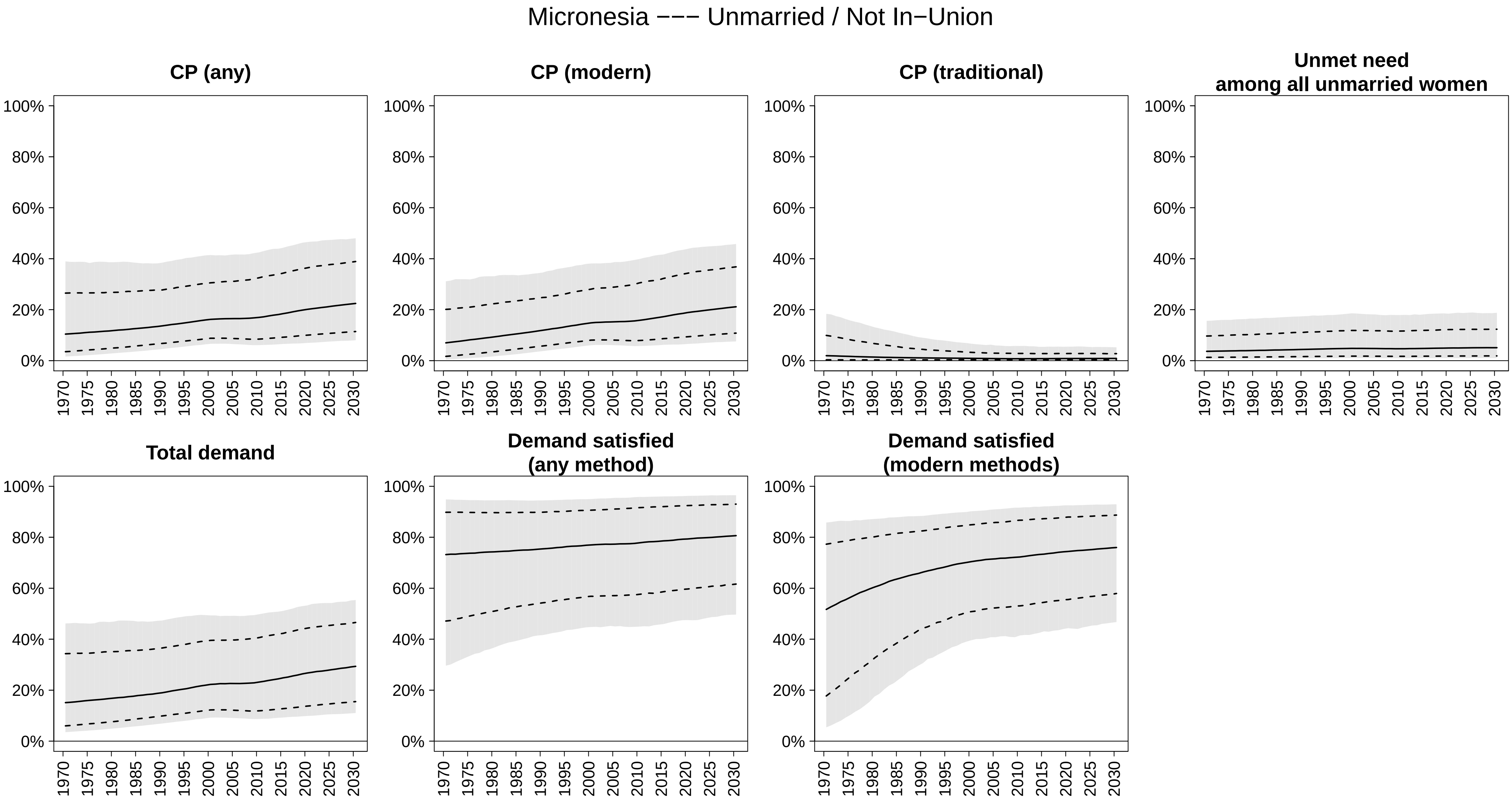
Micronesia −−− Unmarried / Not In−Union
CP (any)
CP (modern)
CP (traditional)
Unmet need
among all unmarried women
Total demand
Demand satisfied
(any method)
Demand satisfied
(modern methods)
0%
20%
40%
60%
80%
100%
1970
1975
1980
1985
1990
1995
2000
2005
2010
2015
2020
2025
2030

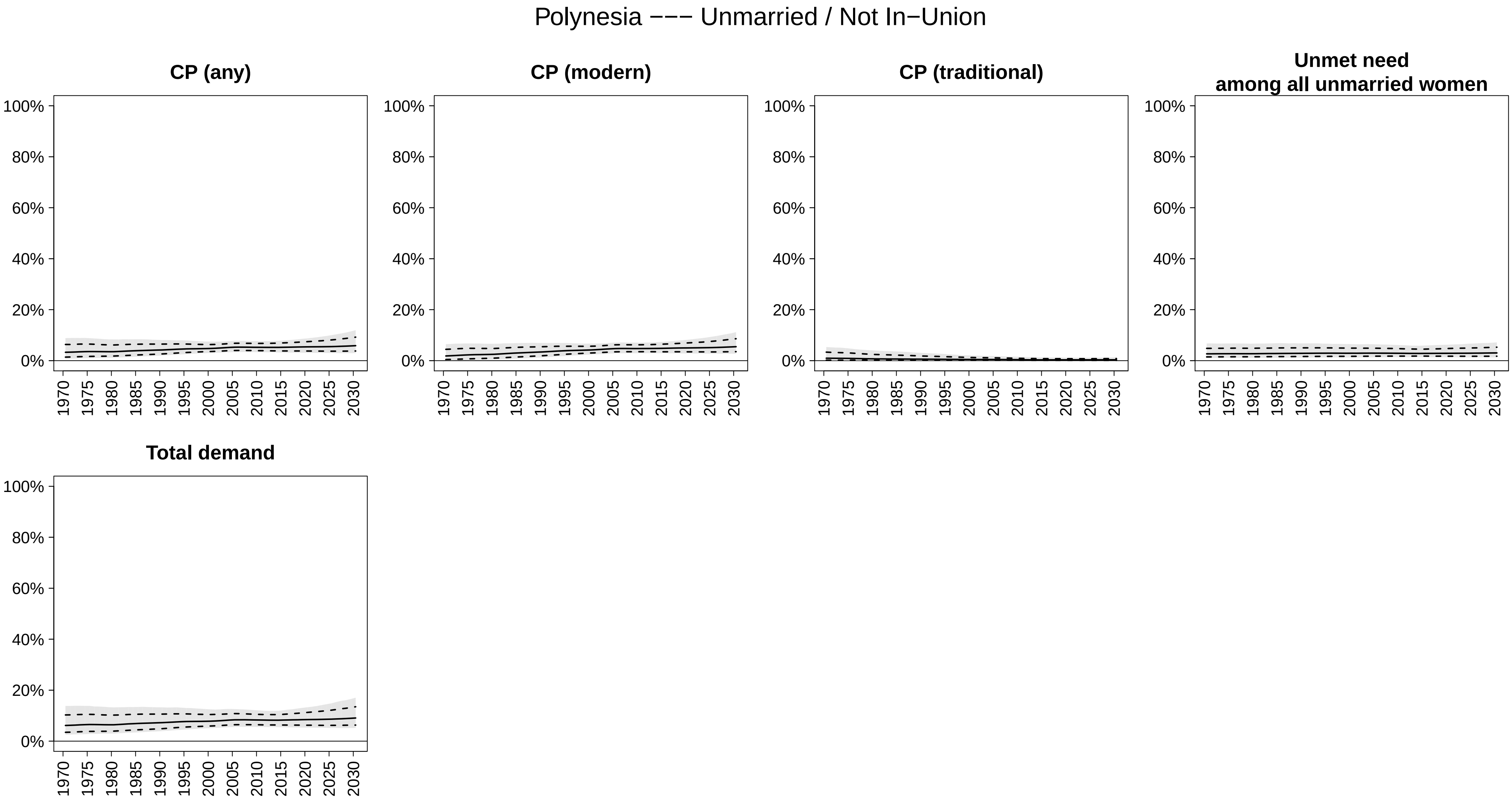
Polynesia −−− Unmarried / Not In−Union
CP (any)
CP (modern)
CP (traditional)
Unmet need among all unmarried women
Total demand
0%
20%
40%
60%
80%
100%
1970
1975
1980
1985
1990
1995
2000
2005
2010
2015
2020
2025
2030

2 Married or in a union women of reproductive age

2.1 Countries and areas

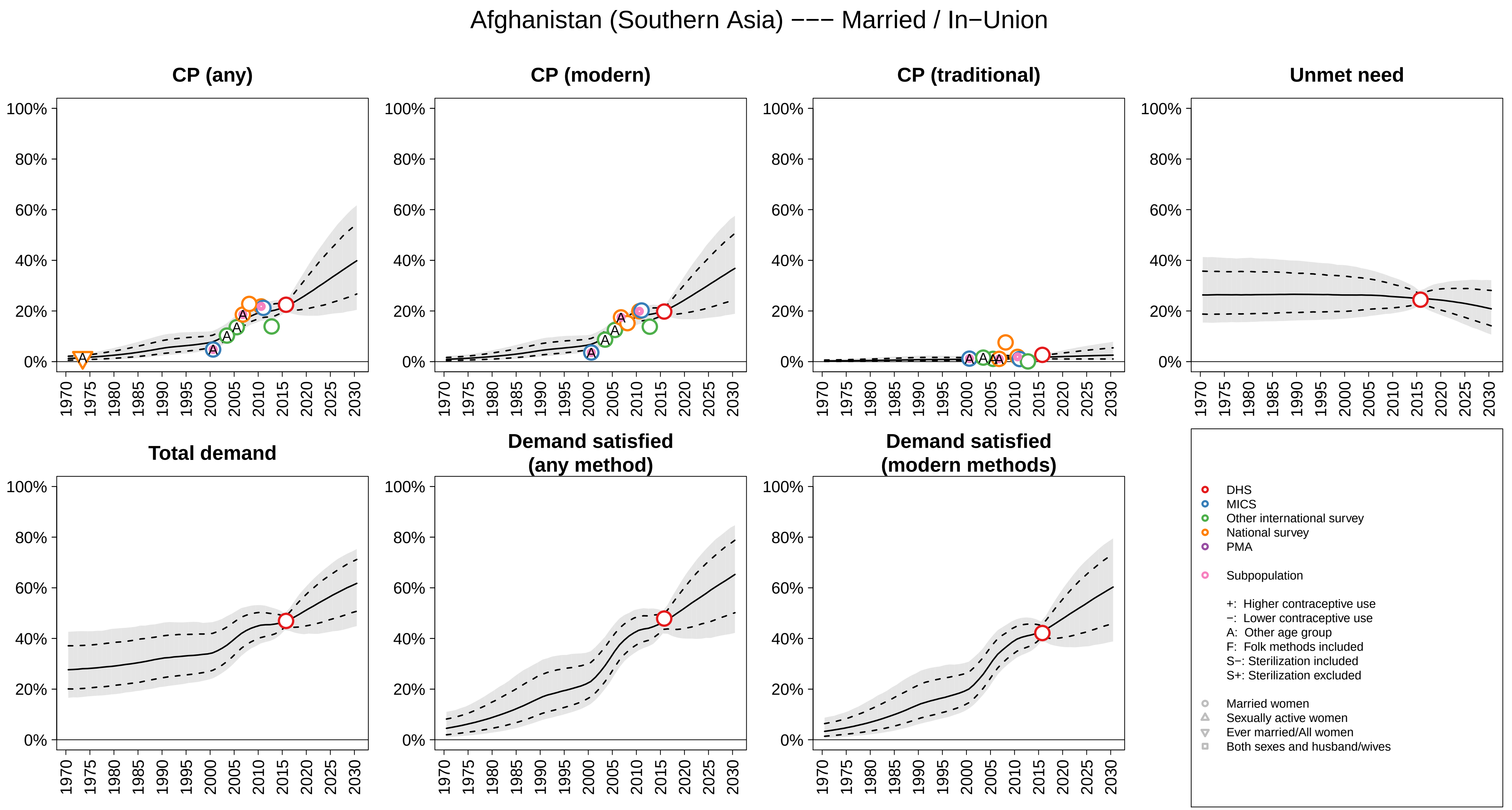
Afghanistan (Southern Asia) −−− Married / In−Union
CP (any)
CP (modern)
CP (traditional)
Unmet need
Total demand
Demand satisfied (any method)
Demand satisfied (modern methods)
DHS
MICS
Other international survey
National survey
PMA
Subpopulation
+: Higher contraceptive use
−: Lower contraceptive use
A: Other age group
F: Folk methods included
S−: Sterilization included
S+: Sterilization excluded
Married women
Sexually active women
Ever married/All women
Both sexes and husband/wives

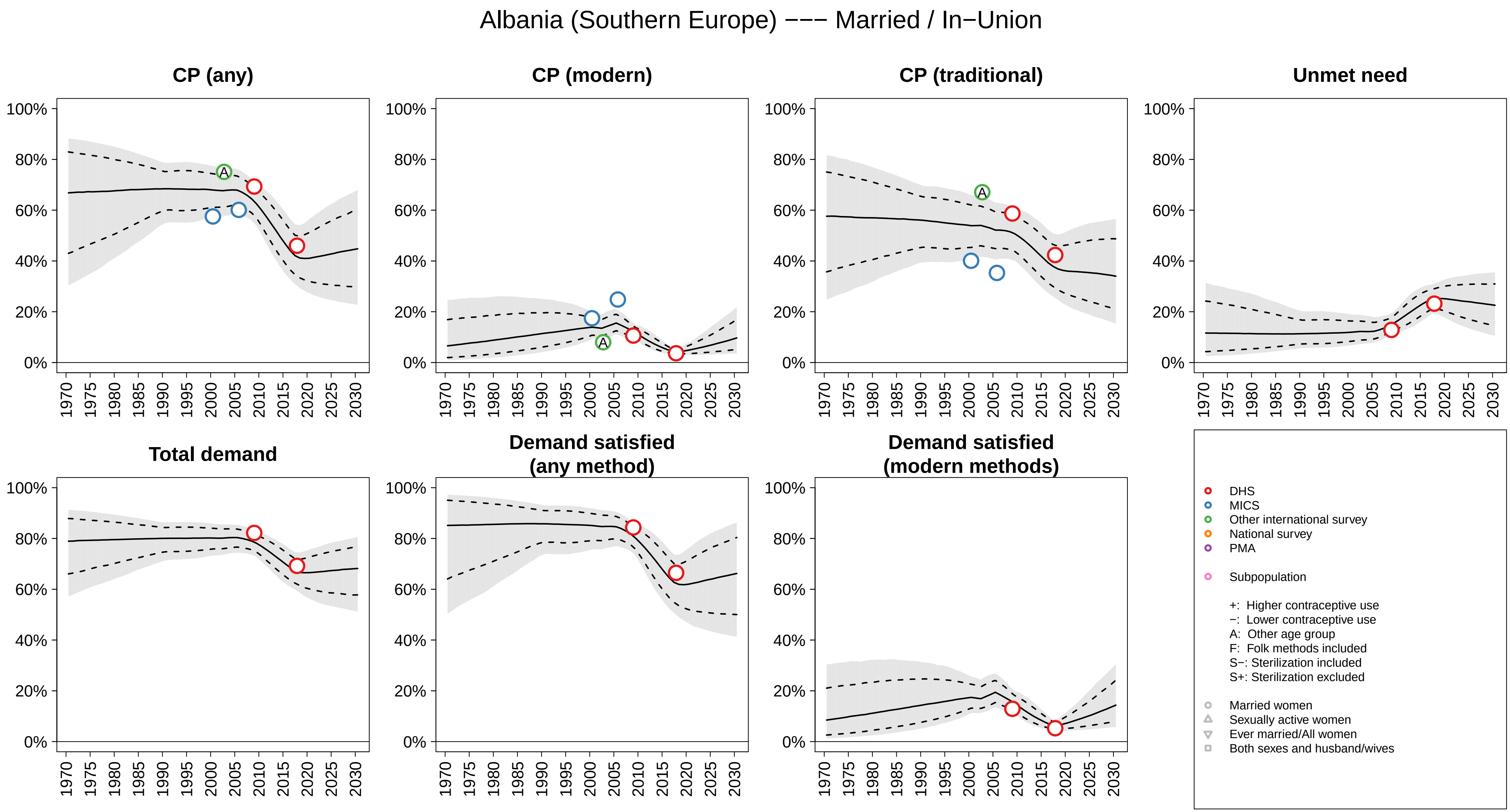
Albania (Southern Europe) −−− Married / In−Union
CP (any)
CP (modern)
CP (traditional)
Unmet need
Total demand
Demand satisfied (any method)
Demand satisfied (modern methods)
0%
20%
40%
60%
80%
100%
1970
1975
1980
1985
1990
1995
2000
2005
2010
2015
2020
2025
2030
A
DHS
MICS
Other international survey
National survey
PMA
Subpopulation
+: Higher contraceptive use
−: Lower contraceptive use
A: Other age group
F: Folk methods included
S−: Sterilization included
S+: Sterilization excluded
Married women
Sexually active women
Ever married/All women
Both sexes and husband/wives

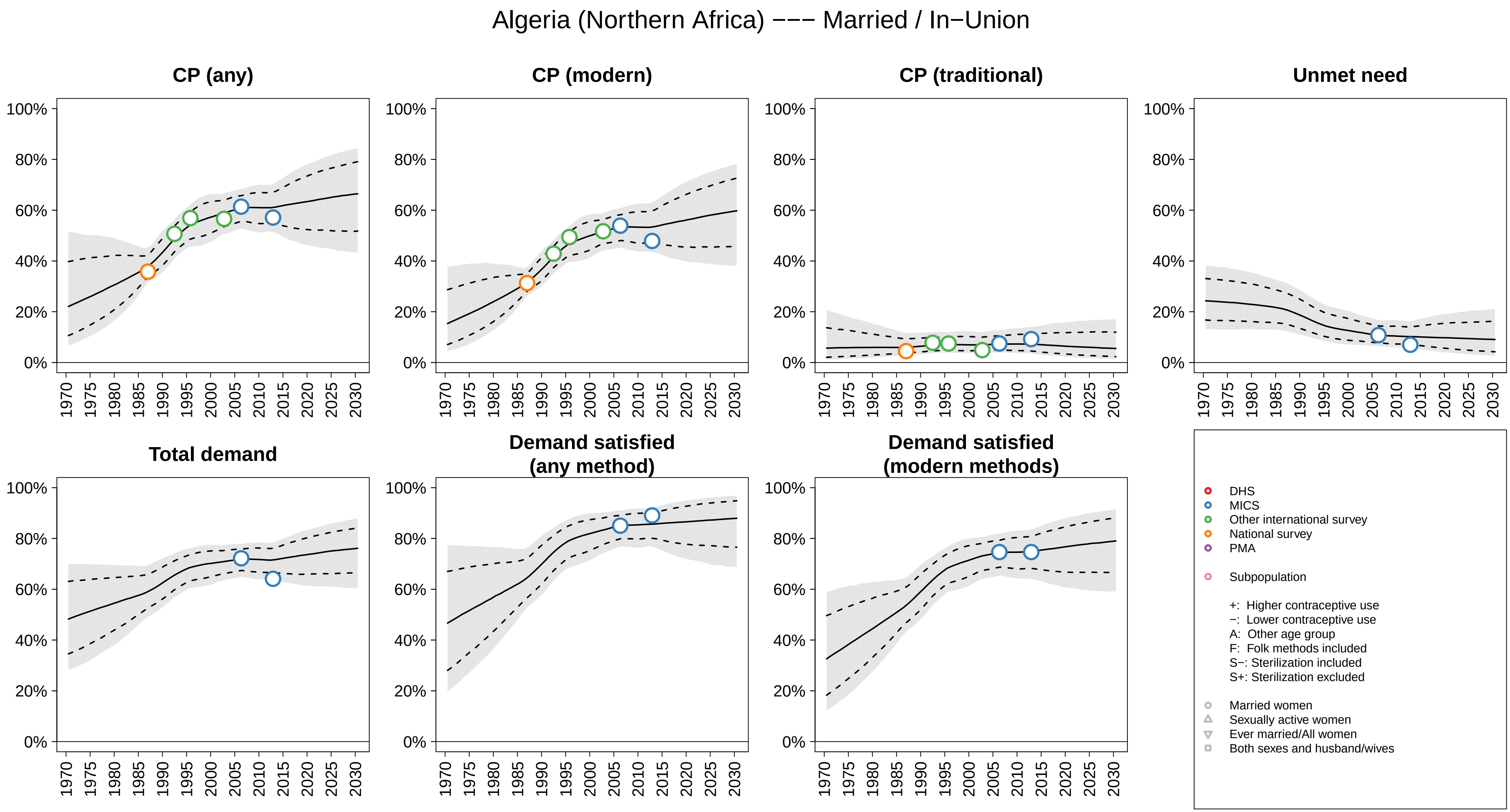
Algeria (Northern Africa) --- Married / In−Union
CP (any)
CP (modern)
CP (traditional)
Unmet need
Total demand
Demand satisfied (any method)
Demand satisfied (modern methods)
0%
20%
40%
60%
80%
100%
1970
1975
1980
1985
1990
1995
2000
2005
2010
2015
2020
2025
2030
DHS
MICS
Other international survey
National survey
PMA
Subpopulation
+: Higher contraceptive use
−: Lower contraceptive use
A: Other age group
F: Folk methods included
S−: Sterilization included
S+: Sterilization excluded
Married women
Sexually active women
Ever married/All women
Both sexes and husband/wives

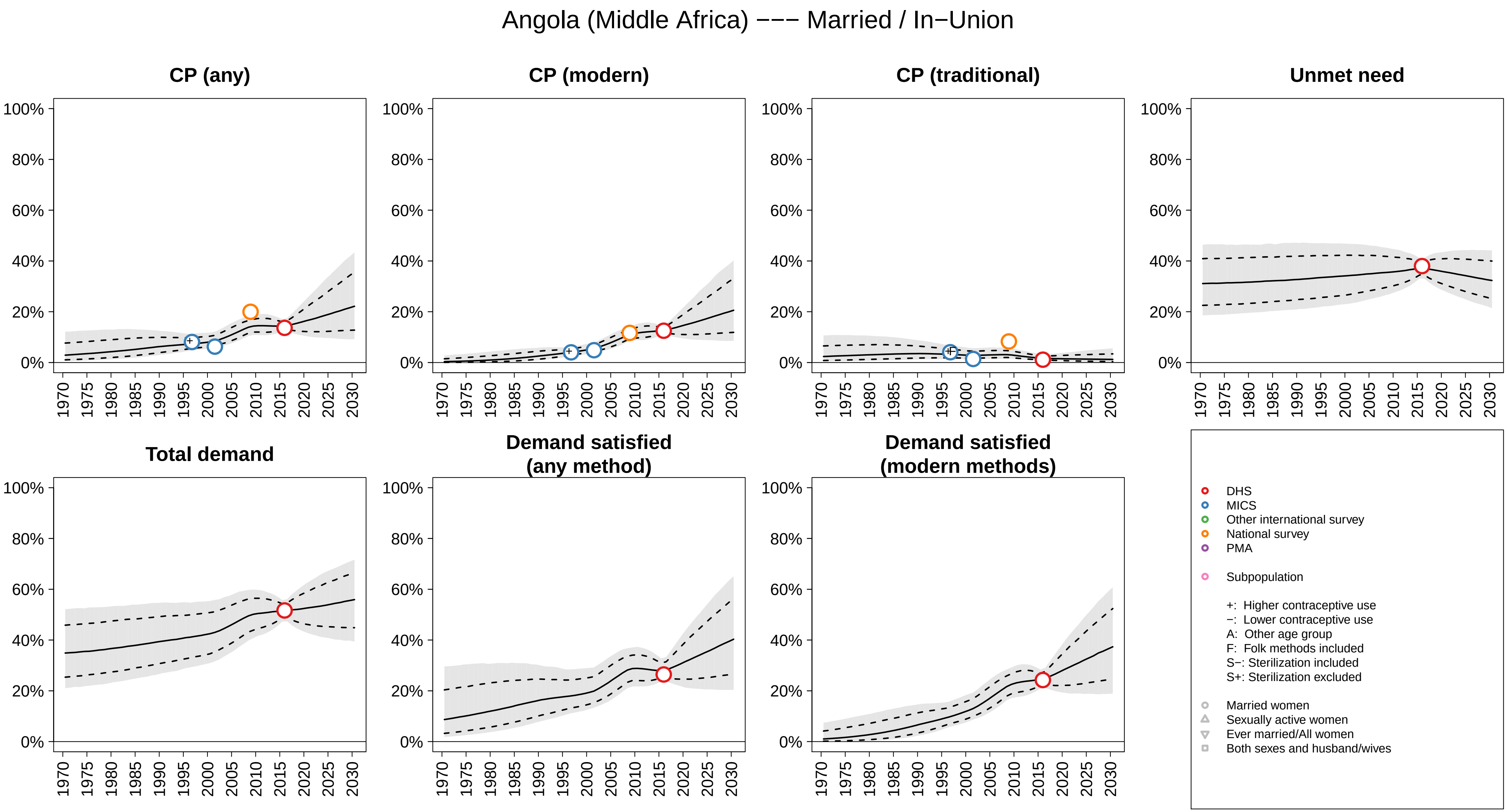
Angola (Middle Africa) --- Married / In−Union
CP (any)
CP (modern)
CP (traditional)
Unmet need
Total demand
Demand satisfied (any method)
Demand satisfied (modern methods)
100%
80%
60%
40%
20%
0%
1970
1975
1980
1985
1990
1995
2000
2005
2010
2015
2020
2025
2030
DHS
MICS
Other international survey
National survey
PMA
Subpopulation
+: Higher contraceptive use
−: Lower contraceptive use
A: Other age group
F: Folk methods included
S−: Sterilization included
S+: Sterilization excluded
Married women
Sexually active women
Ever married/All women
Both sexes and husband/wives

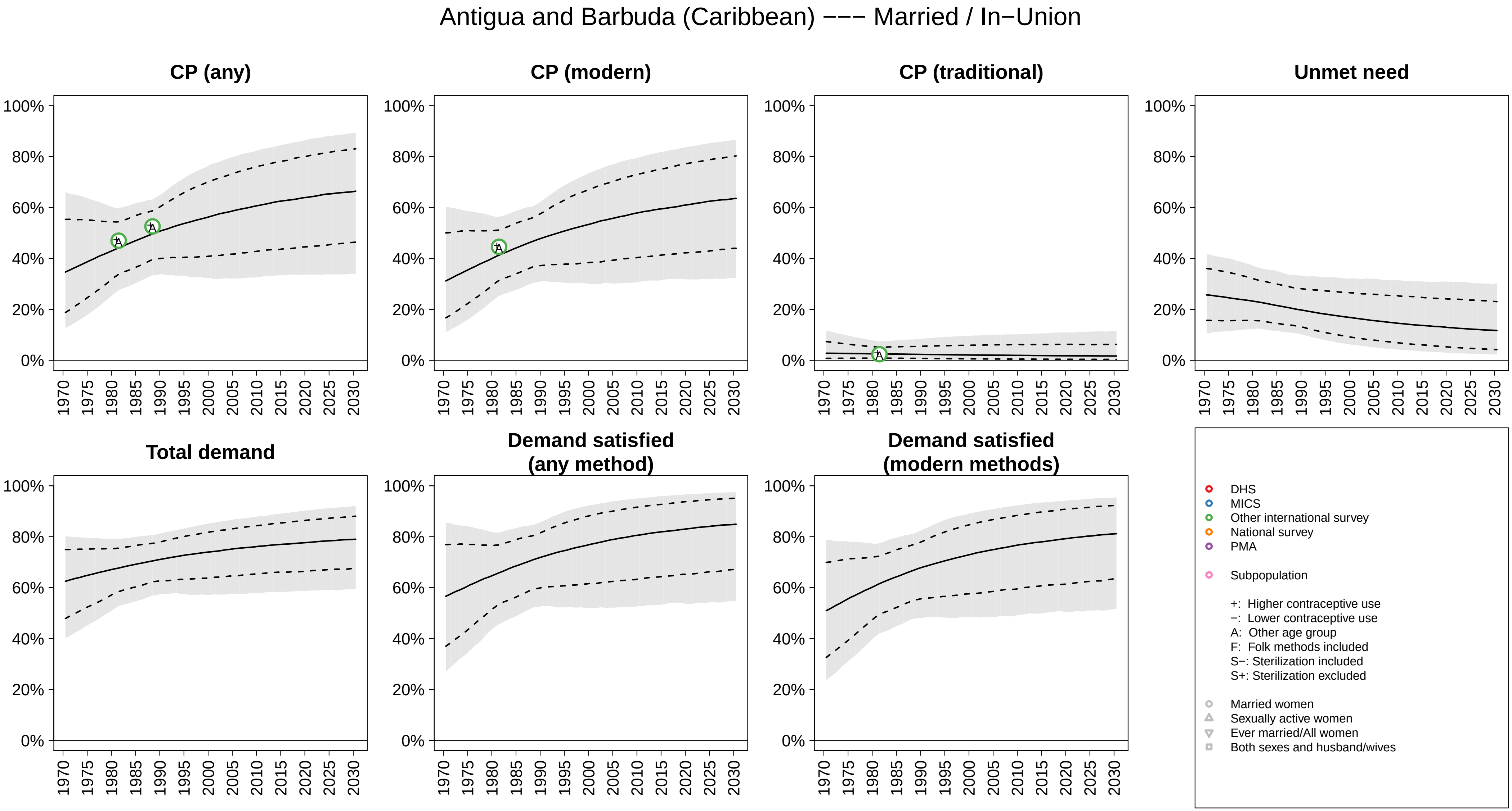
Antigua and Barbuda (Caribbean) −−− Married / In−Union
CP (any)
CP (modern)
CP (traditional)
Unmet need
Total demand
Demand satisfied (any method)
Demand satisfied (modern methods)
0%
20%
40%
60%
80%
100%
1970
1975
1980
1985
1990
1995
2000
2005
2010
2015
2020
2025
2030
A
DHS
MICS
Other international survey
National survey
PMA
Subpopulation
+: Higher contraceptive use
−: Lower contraceptive use
A: Other age group
F: Folk methods included
S−: Sterilization included
S+: Sterilization excluded
Married women
Sexually active women
Ever married/All women
Both sexes and husband/wives

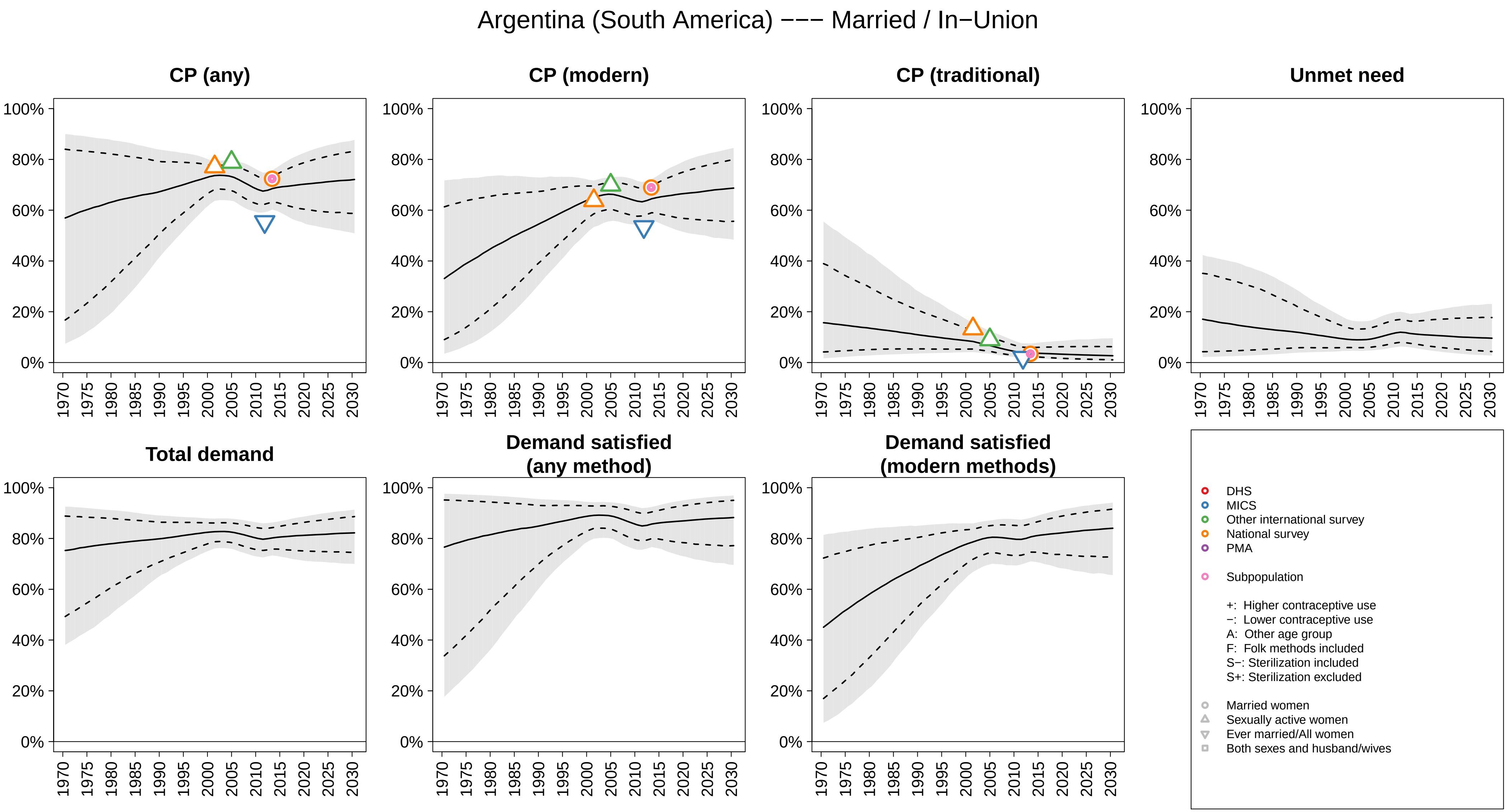
Argentina (South America) −−− Married / In−Union
CP (any)
CP (modern)
CP (traditional)
Unmet need
Total demand
Demand satisfied (any method)
Demand satisfied (modern methods)
0%
20%
40%
60%
80%
100%
1970
1975
1980
1985
1990
1995
2000
2005
2010
2015
2020
2025
2030
DHS
MICS
Other international survey
National survey
PMA
Subpopulation
+: Higher contraceptive use
−: Lower contraceptive use
A: Other age group
F: Folk methods included
S−: Sterilization included
S+: Sterilization excluded
Married women
Sexually active women
Ever married/All women
Both sexes and husband/wives

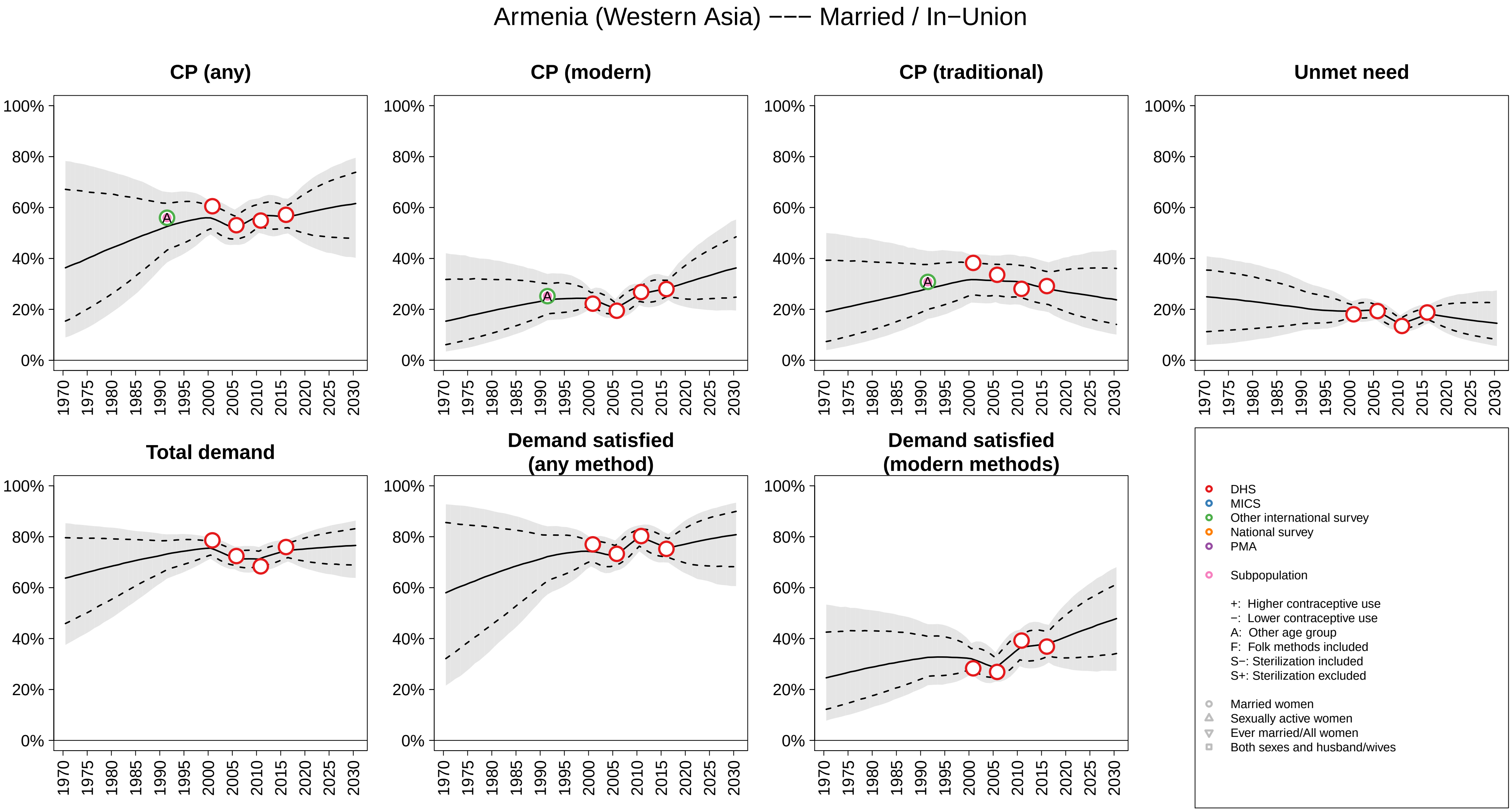
Armenia (Western Asia) ––– Married / In−Union
CP (any)
CP (modern)
CP (traditional)
Unmet need
Total demand
Demand satisfied (any method)
Demand satisfied (modern methods)
0%
20%
40%
60%
80%
100%
1970
1975
1980
1985
1990
1995
2000
2005
2010
2015
2020
2025
2030
A
DHS
MICS
Other international survey
National survey
PMA
Subpopulation
+: Higher contraceptive use
−: Lower contraceptive use
A: Other age group
F: Folk methods included
S−: Sterilization included
S+: Sterilization excluded
Married women
Sexually active women
Ever married/All women
Both sexes and husband/wives

Australia (Australia and New Zealand) −−− Married / In−Union

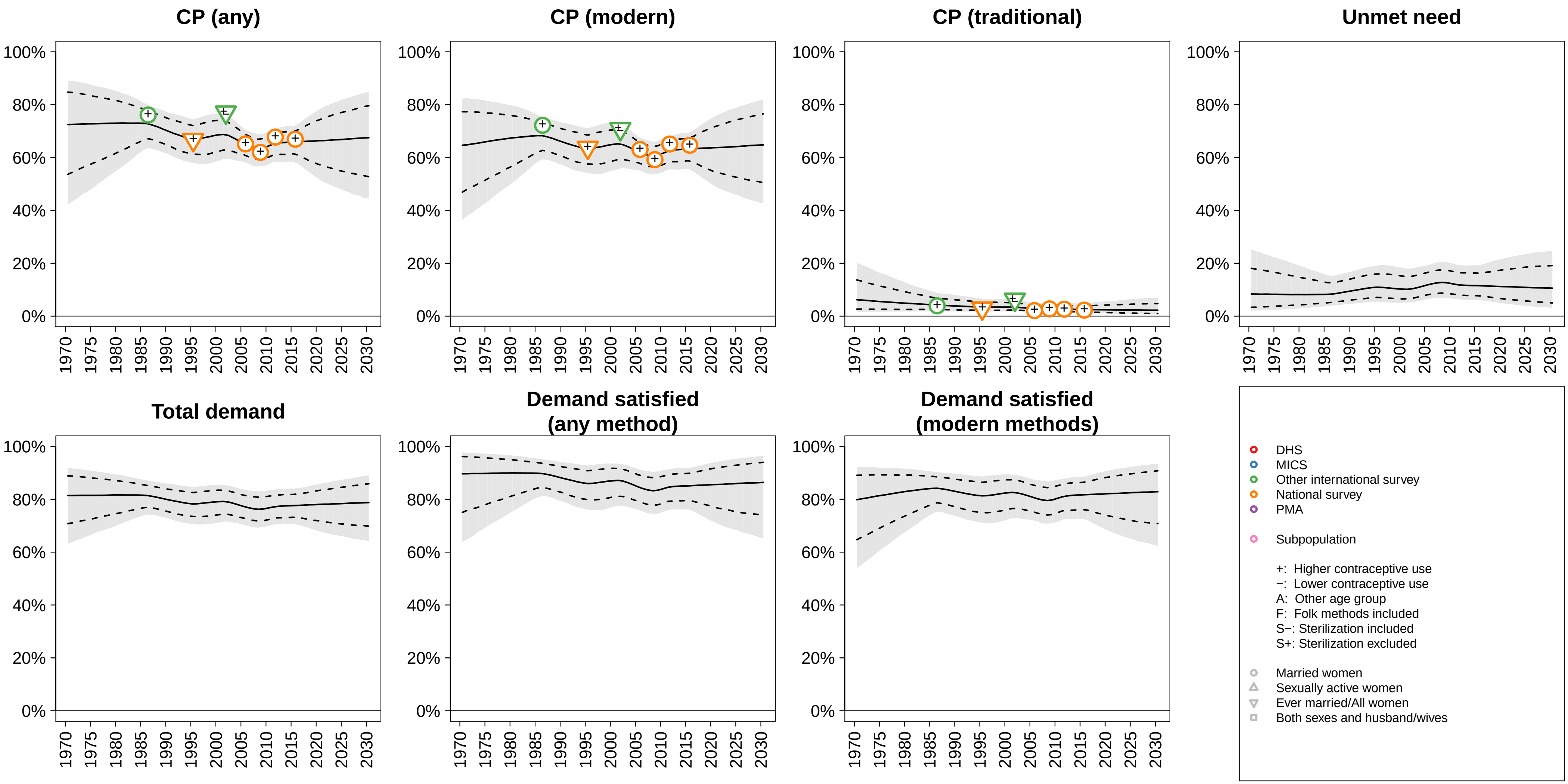

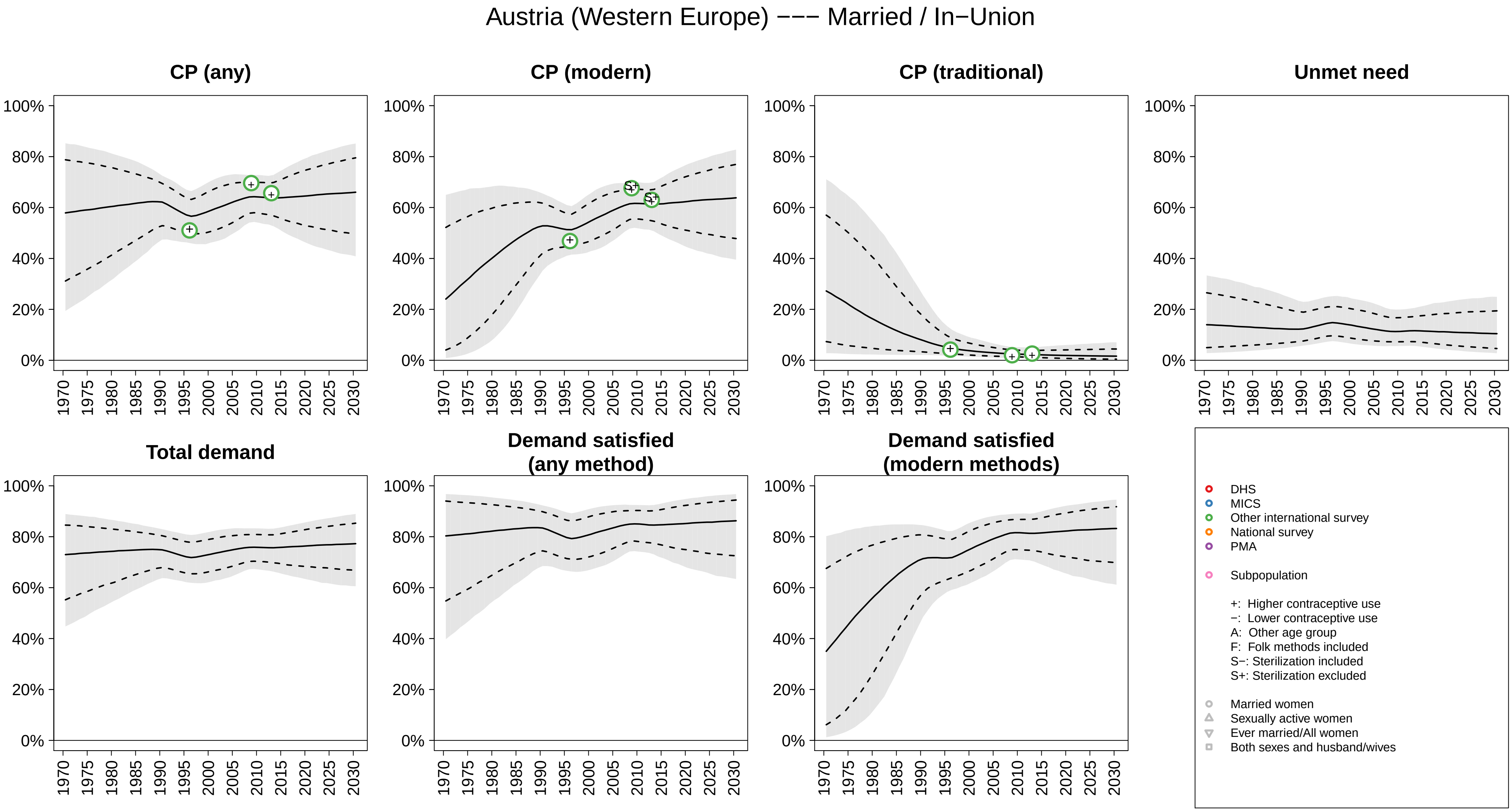
Austria (Western Europe) −−− Married / In−Union
CP (any)
CP (modern)
CP (traditional)
Unmet need
Total demand
Demand satisfied (any method)
Demand satisfied (modern methods)
0%
20%
40%
60%
80%
100%
1970
1975
1980
1985
1990
1995
2000
2005
2010
2015
2020
2025
2030
S+
DHS
MICS
Other international survey
National survey
PMA
Subpopulation
+: Higher contraceptive use
−: Lower contraceptive use
A: Other age group
F: Folk methods included
S−: Sterilization included
S+: Sterilization excluded
Married women
Sexually active women
Ever married/All women
Both sexes and husband/wives

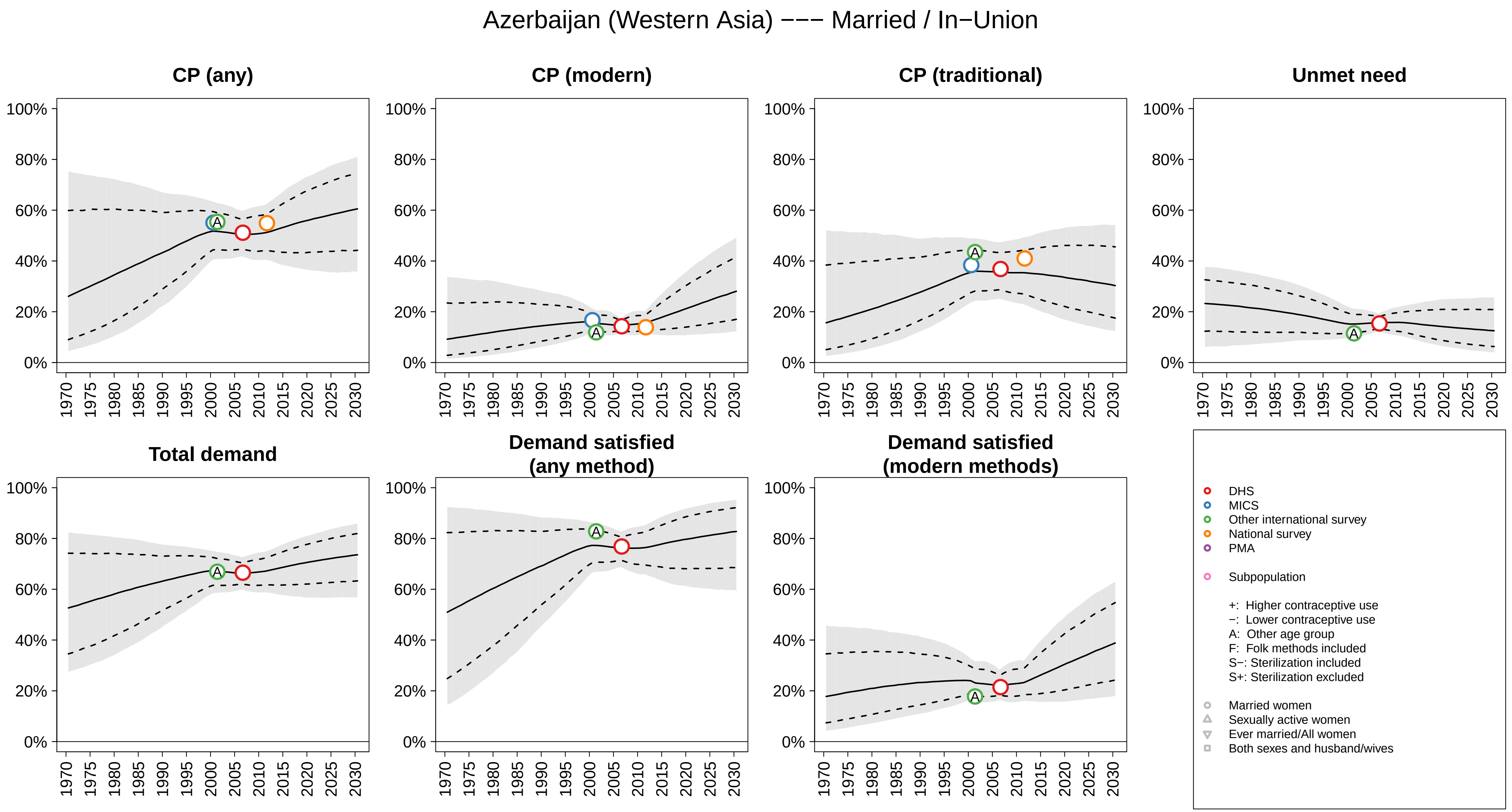
Azerbaijan (Western Asia) --- Married / In−Union
CP (any)
CP (modern)
CP (traditional)
Unmet need
Total demand
Demand satisfied (any method)
Demand satisfied (modern methods)
100%
80%
60%
40%
20%
0%
1970
1975
1980
1985
1990
1995
2000
2005
2010
2015
2020
2025
2030
A
DHS
MICS
Other international survey
National survey
PMA
Subpopulation
+: Higher contraceptive use
−: Lower contraceptive use
A: Other age group
F: Folk methods included
S−: Sterilization included
S+: Sterilization excluded
Married women
Sexually active women
Ever married/All women
Both sexes and husband/wives

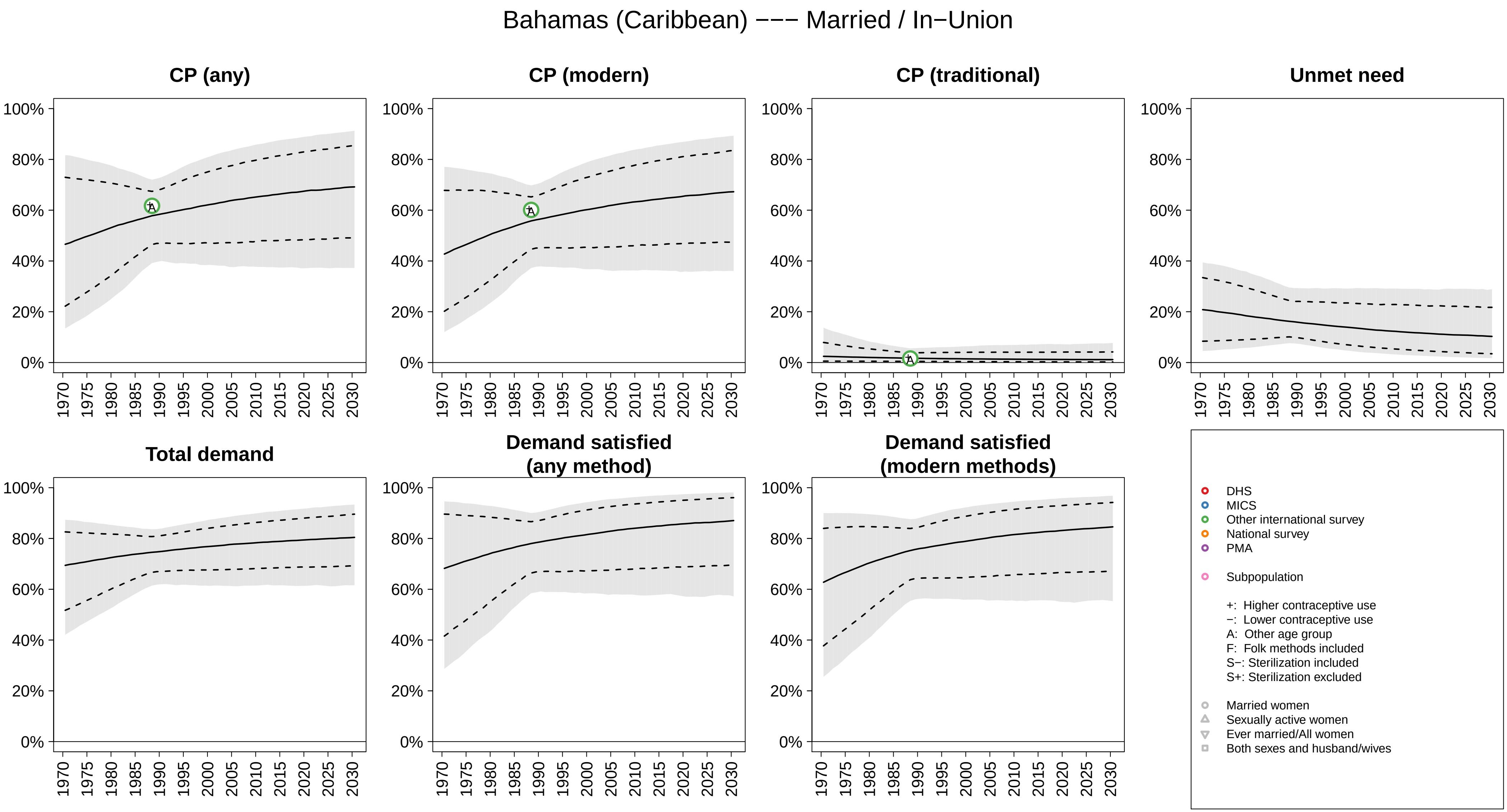
Bahamas (Caribbean) −−− Married / In−Union
CP (any)
CP (modern)
CP (traditional)
Unmet need
Total demand
Demand satisfied (any method)
Demand satisfied (modern methods)
0%
20%
40%
60%
80%
100%
1970
1975
1980
1985
1990
1995
2000
2005
2010
2015
2020
2025
2030
A
DHS
MICS
Other international survey
National survey
PMA
Subpopulation
+: Higher contraceptive use
−: Lower contraceptive use
A: Other age group
F: Folk methods included
S−: Sterilization included
S+: Sterilization excluded
Married women
Sexually active women
Ever married/All women
Both sexes and husband/wives

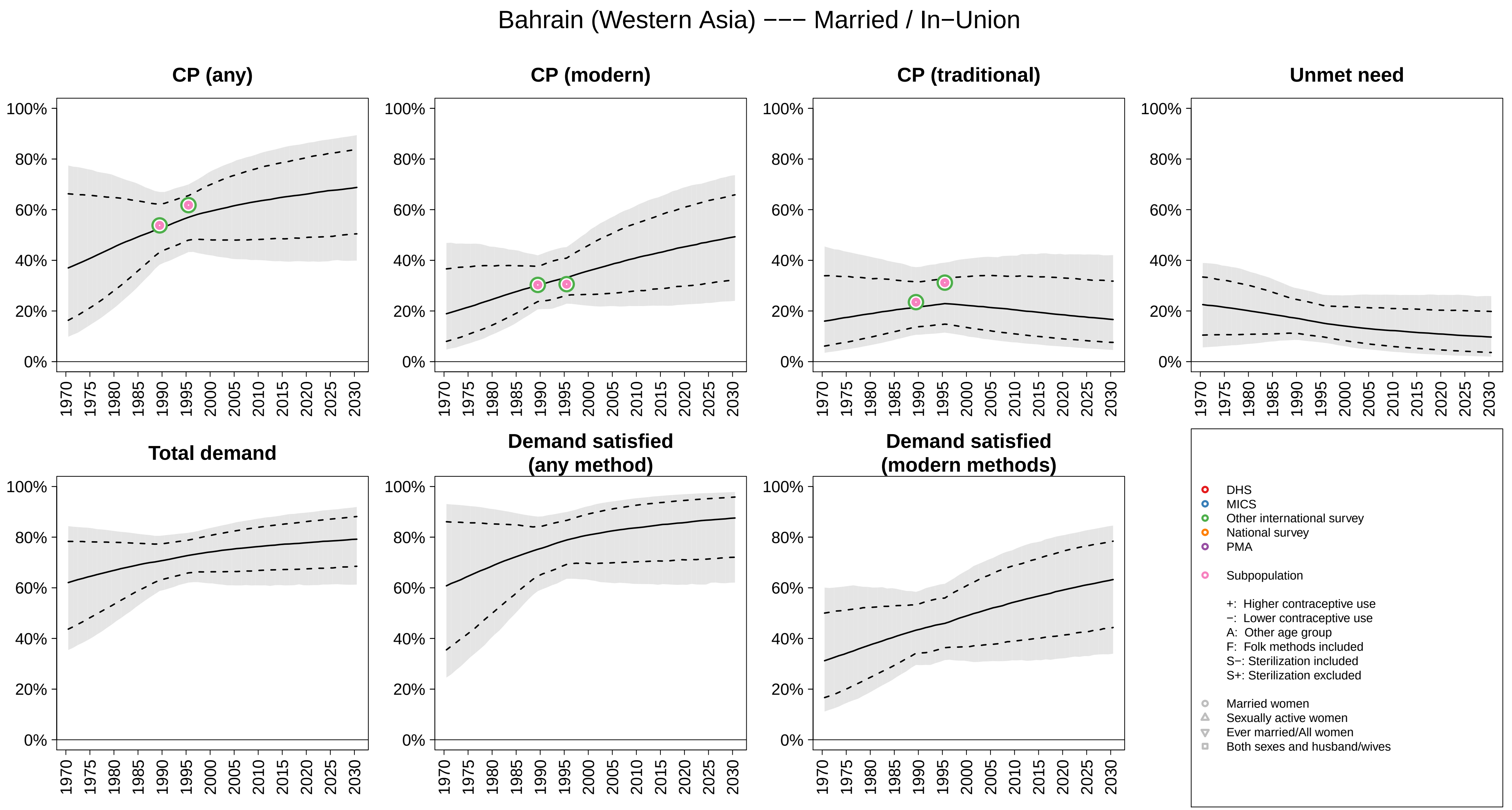
Bahrain (Western Asia) −−− Married / In−Union
CP (any)
CP (modern)
CP (traditional)
Unmet need
Total demand
Demand satisfied (any method)
Demand satisfied (modern methods)
0%
20%
40%
60%
80%
100%
1970
1975
1980
1985
1990
1995
2000
2005
2010
2015
2020
2025
2030
DHS
MICS
Other international survey
National survey
PMA
Subpopulation
+: Higher contraceptive use
−: Lower contraceptive use
A: Other age group
F: Folk methods included
S−: Sterilization included
S+: Sterilization excluded
Married women
Sexually active women
Ever married/All women
Both sexes and husband/wives

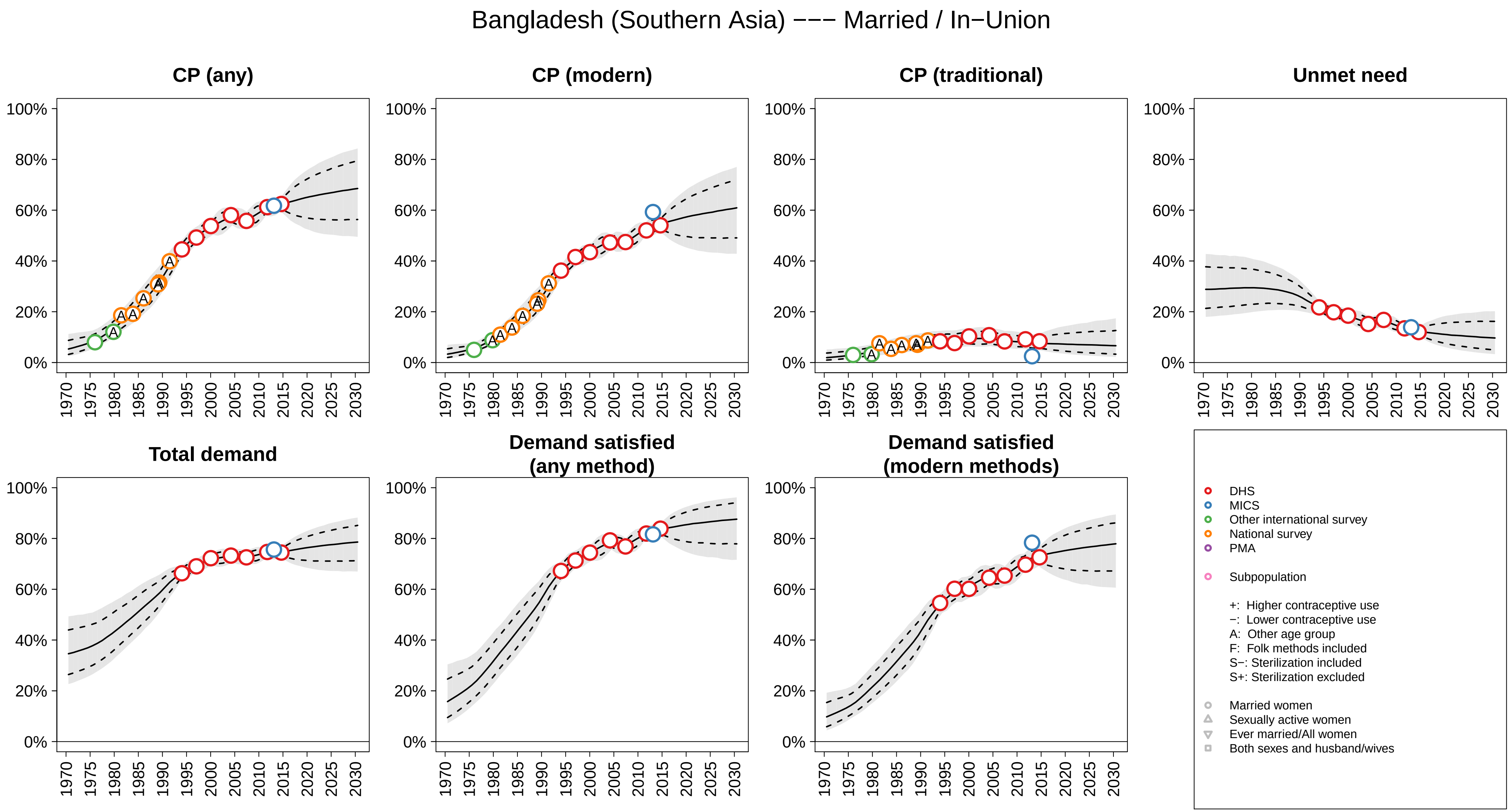
Bangladesh (Southern Asia) --- Married / In−Union
CP (any)
CP (modern)
CP (traditional)
Unmet need
Total demand
Demand satisfied (any method)
Demand satisfied (modern methods)
0%
20%
40%
60%
80%
100%
1970
1975
1980
1985
1990
1995
2000
2005
2010
2015
2020
2025
2030
DHS
MICS
Other international survey
National survey
PMA
Subpopulation
+: Higher contraceptive use
−: Lower contraceptive use
A: Other age group
F: Folk methods included
S−: Sterilization included
S+: Sterilization excluded
Married women
Sexually active women
Ever married/All women
Both sexes and husband/wives

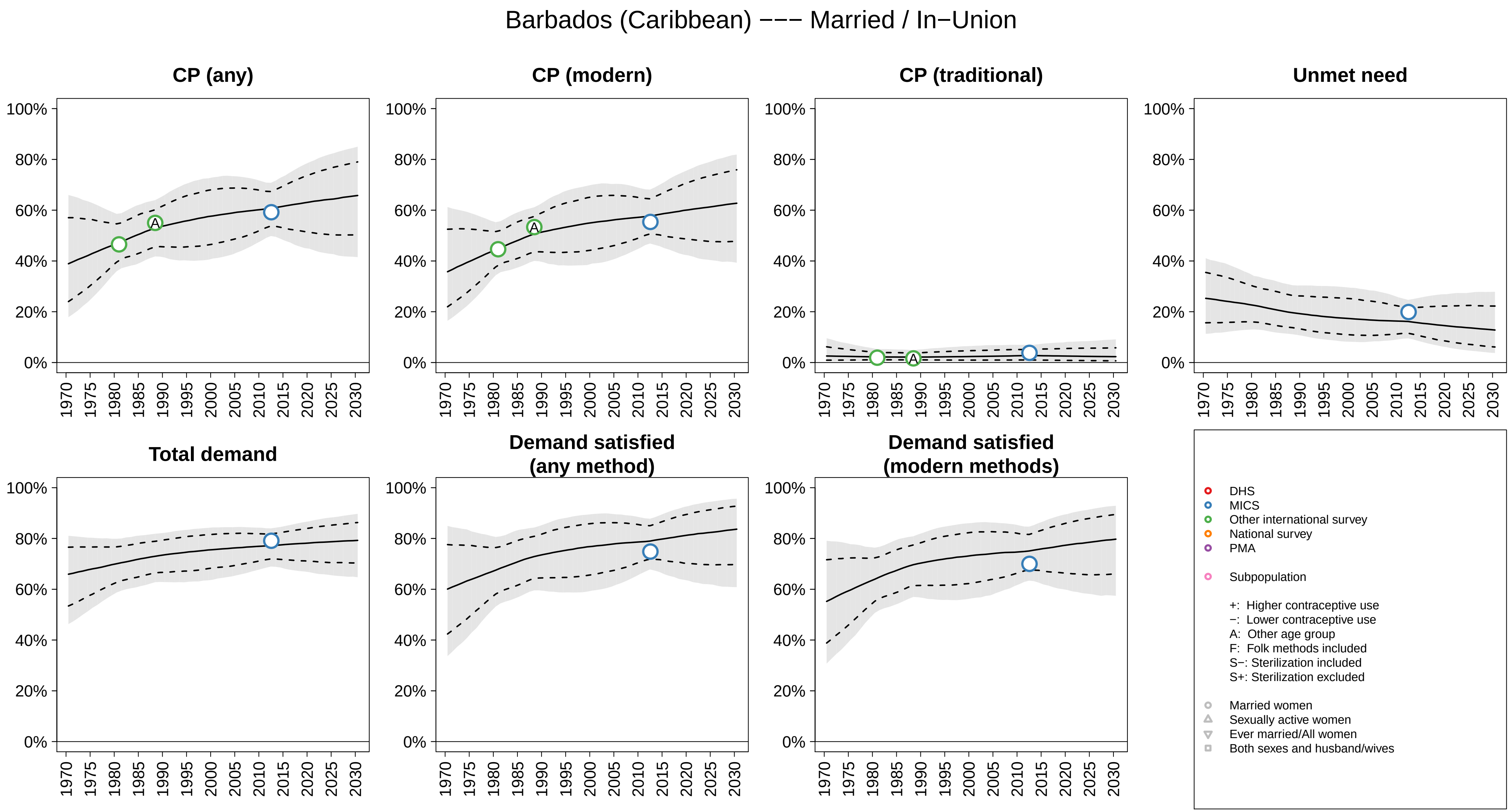
Barbados (Caribbean) −−− Married / In−Union
CP (any)
CP (modern)
CP (traditional)
Unmet need
Total demand
Demand satisfied (any method)
Demand satisfied (modern methods)
0%
20%
40%
60%
80%
100%
1970
1975
1980
1985
1990
1995
2000
2005
2010
2015
2020
2025
2030
DHS
MICS
Other international survey
National survey
PMA
Subpopulation
+: Higher contraceptive use
−: Lower contraceptive use
A: Other age group
F: Folk methods included
S−: Sterilization included
S+: Sterilization excluded
Married women
Sexually active women
Ever married/All women
Both sexes and husband/wives

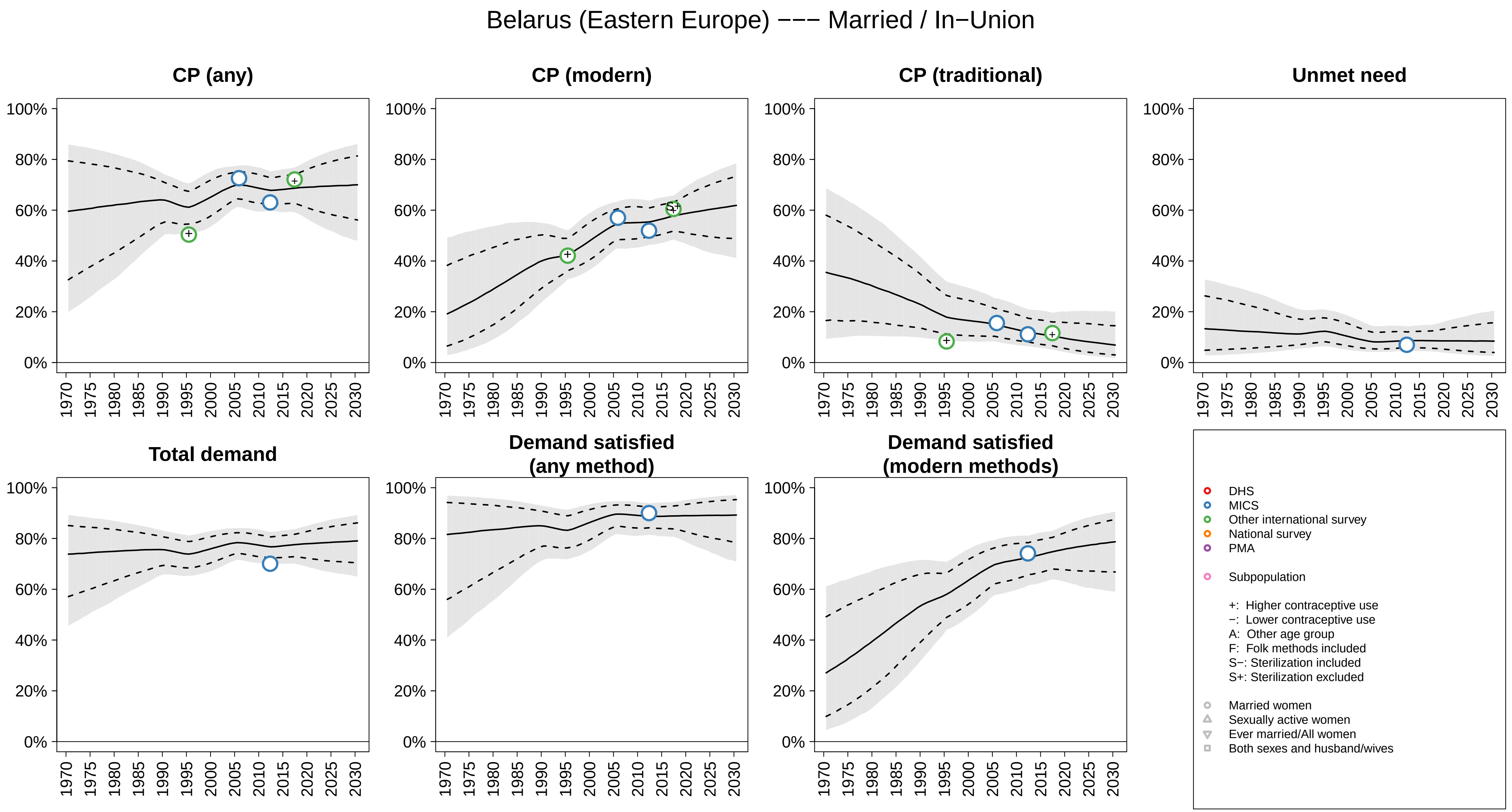
Belarus (Eastern Europe) −−− Married / In−Union
CP (any)
CP (modern)
CP (traditional)
Unmet need
Total demand
Demand satisfied (any method)
Demand satisfied (modern methods)
0%
20%
40%
60%
80%
100%
1970
1975
1980
1985
1990
1995
2000
2005
2010
2015
2020
2025
2030
DHS
MICS
Other international survey
National survey
PMA
Subpopulation
+: Higher contraceptive use
−: Lower contraceptive use
A: Other age group
F: Folk methods included
S−: Sterilization included
S+: Sterilization excluded
Married women
Sexually active women
Ever married/All women
Both sexes and husband/wives

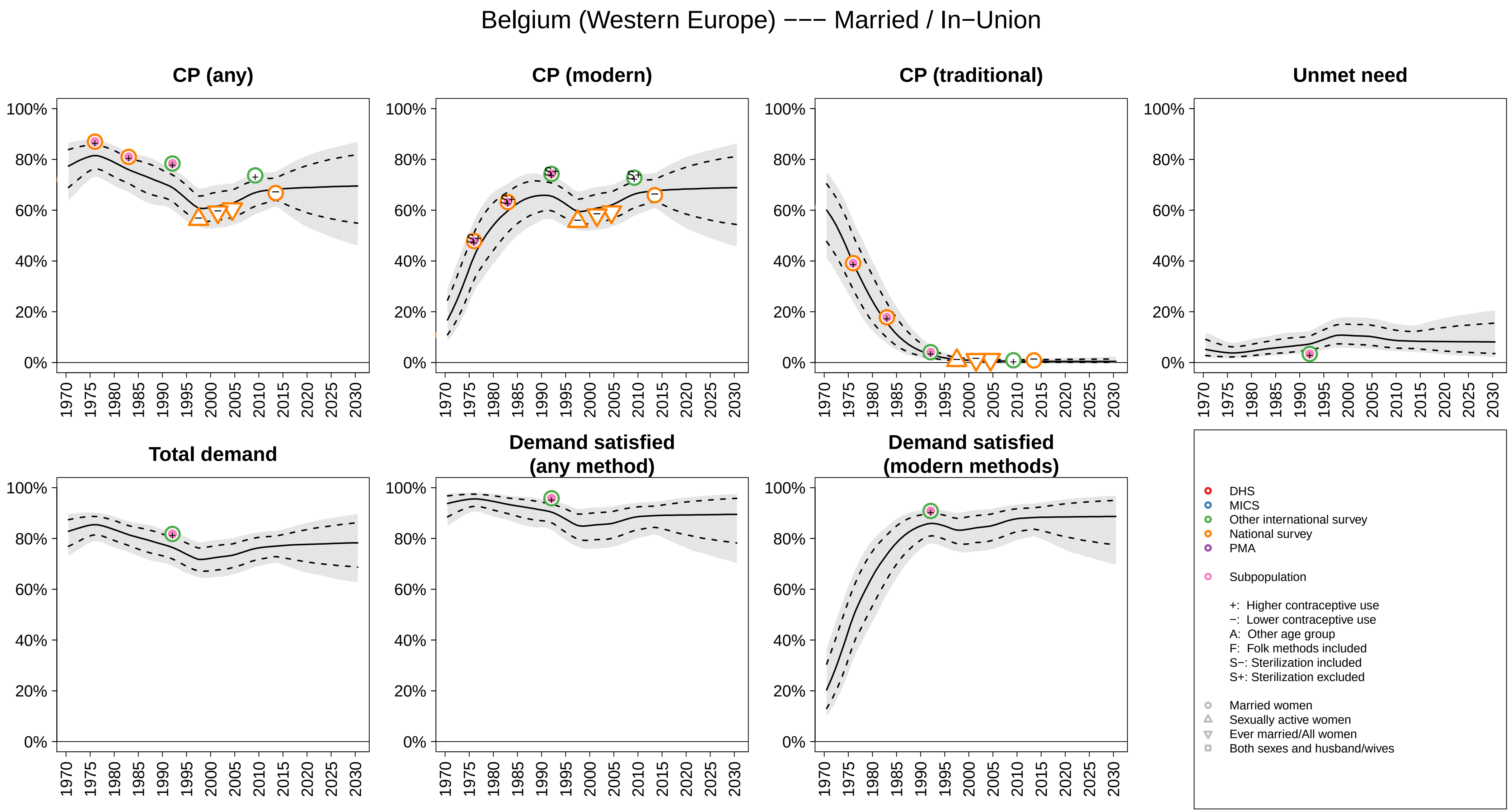
Belgium (Western Europe) --- Married / In−Union
CP (any)
CP (modern)
CP (traditional)
Unmet need
Total demand
Demand satisfied (any method)
Demand satisfied (modern methods)
100%
80%
60%
40%
20%
0%
1970 1975 1980 1985 1990 1995 2000 2005 2010 2015 2020 2025 2030
DHS
MICS
Other international survey
National survey
PMA
Subpopulation
+: Higher contraceptive use
−: Lower contraceptive use
A: Other age group
F: Folk methods included
S−: Sterilization included
S+: Sterilization excluded
Married women
Sexually active women
Ever married/All women
Both sexes and husband/wives

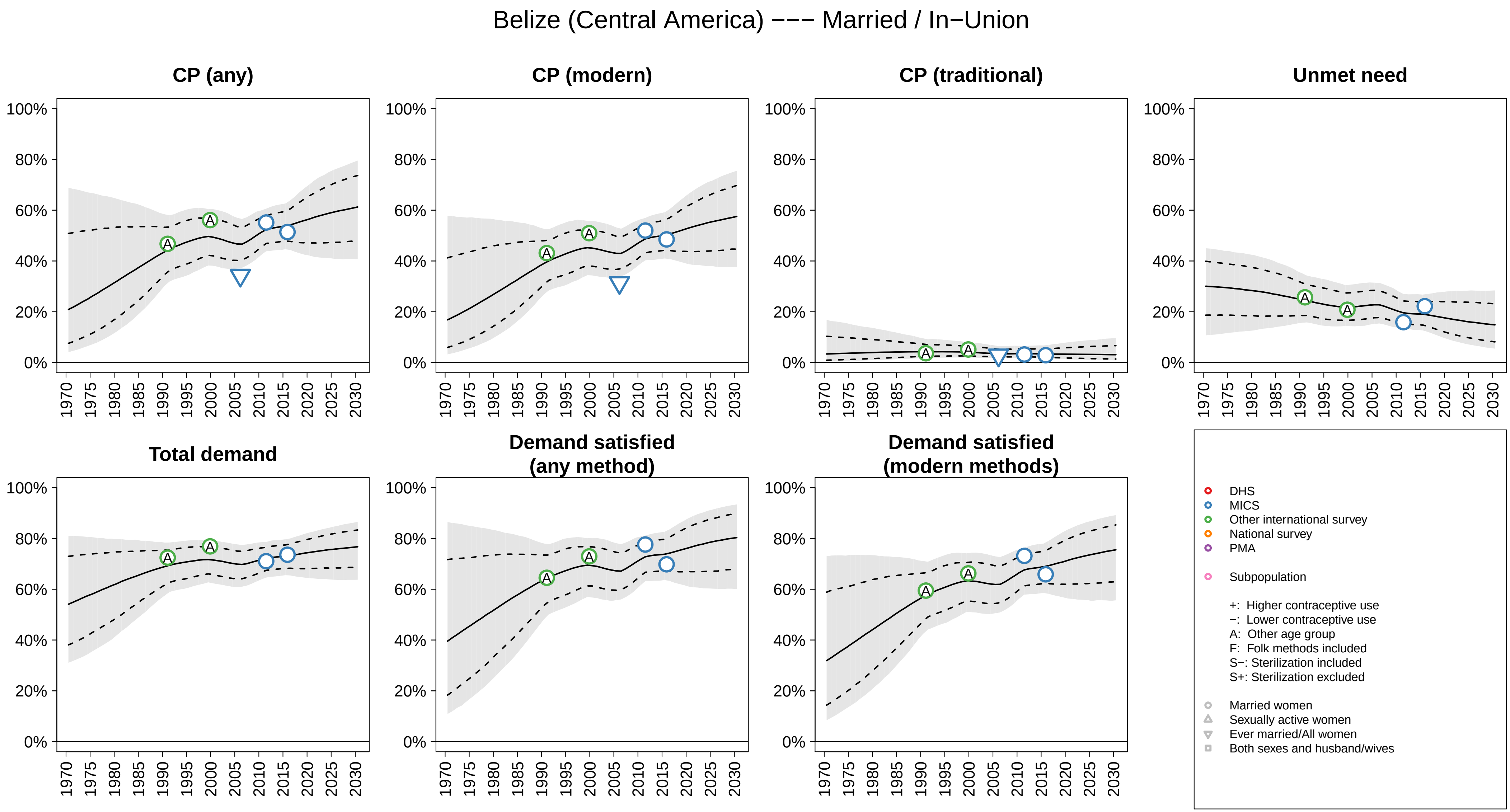
Belize (Central America) --- Married / In−Union
CP (any)
CP (modern)
CP (traditional)
Unmet need
Total demand
Demand satisfied (any method)
Demand satisfied (modern methods)
0%
20%
40%
60%
80%
100%
1970
1975
1980
1985
1990
1995
2000
2005
2010
2015
2020
2025
2030
A
DHS
MICS
Other international survey
National survey
PMA
Subpopulation
+: Higher contraceptive use
−: Lower contraceptive use
A: Other age group
F: Folk methods included
S−: Sterilization included
S+: Sterilization excluded
Married women
Sexually active women
Ever married/All women
Both sexes and husband/wives

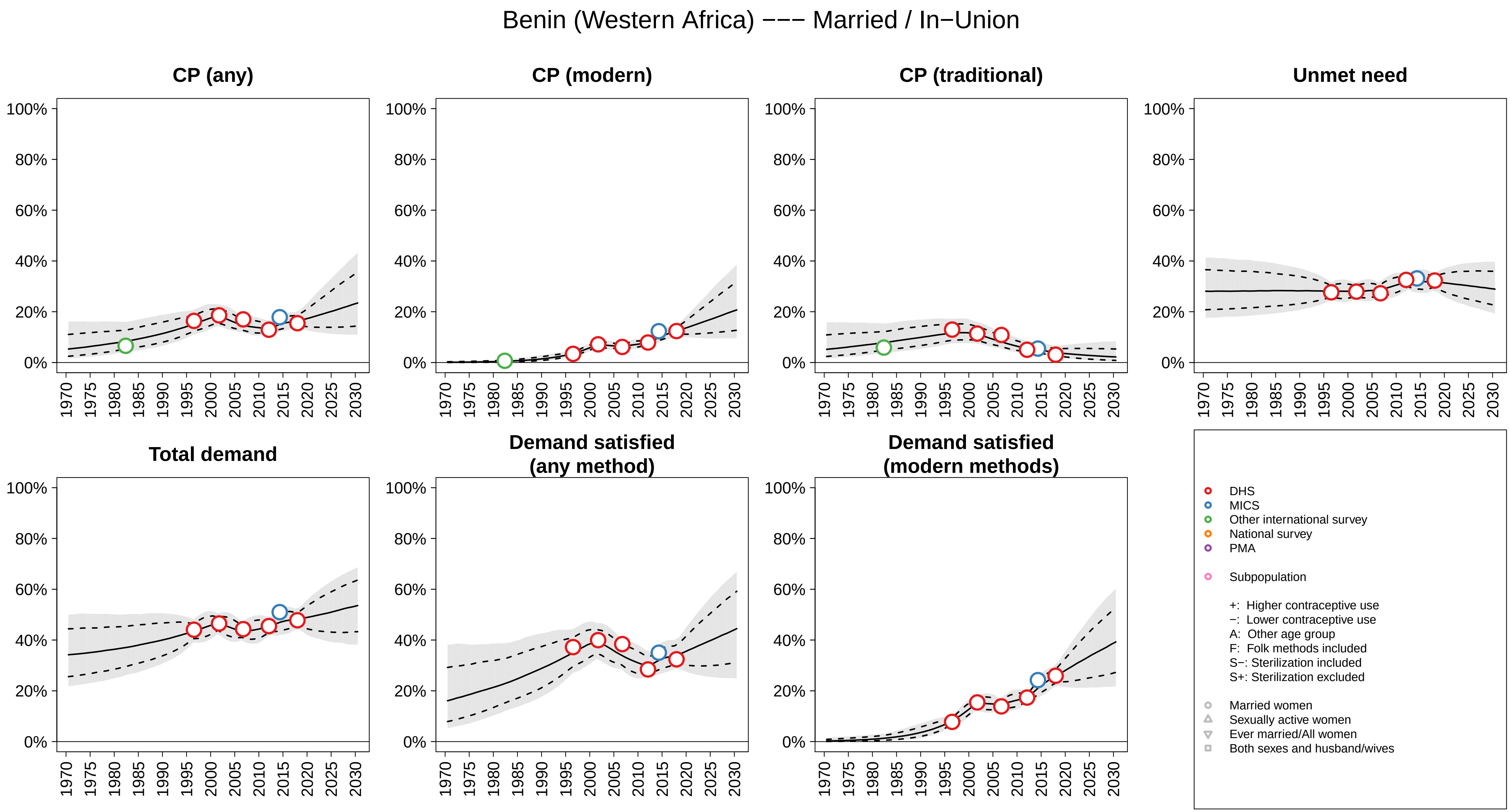
Benin (Western Africa) −−− Married / In−Union
CP (any)
CP (modern)
CP (traditional)
Unmet need
Total demand
Demand satisfied (any method)
Demand satisfied (modern methods)
100%
80%
60%
40%
20%
0%
1970 1975 1980 1985 1990 1995 2000 2005 2010 2015 2020 2025 2030
DHS
MICS
Other international survey
National survey
PMA
Subpopulation
+: Higher contraceptive use
−: Lower contraceptive use
A: Other age group
F: Folk methods included
S−: Sterilization included
S+: Sterilization excluded
Married women
Sexually active women
Ever married/All women
Both sexes and husband/wives

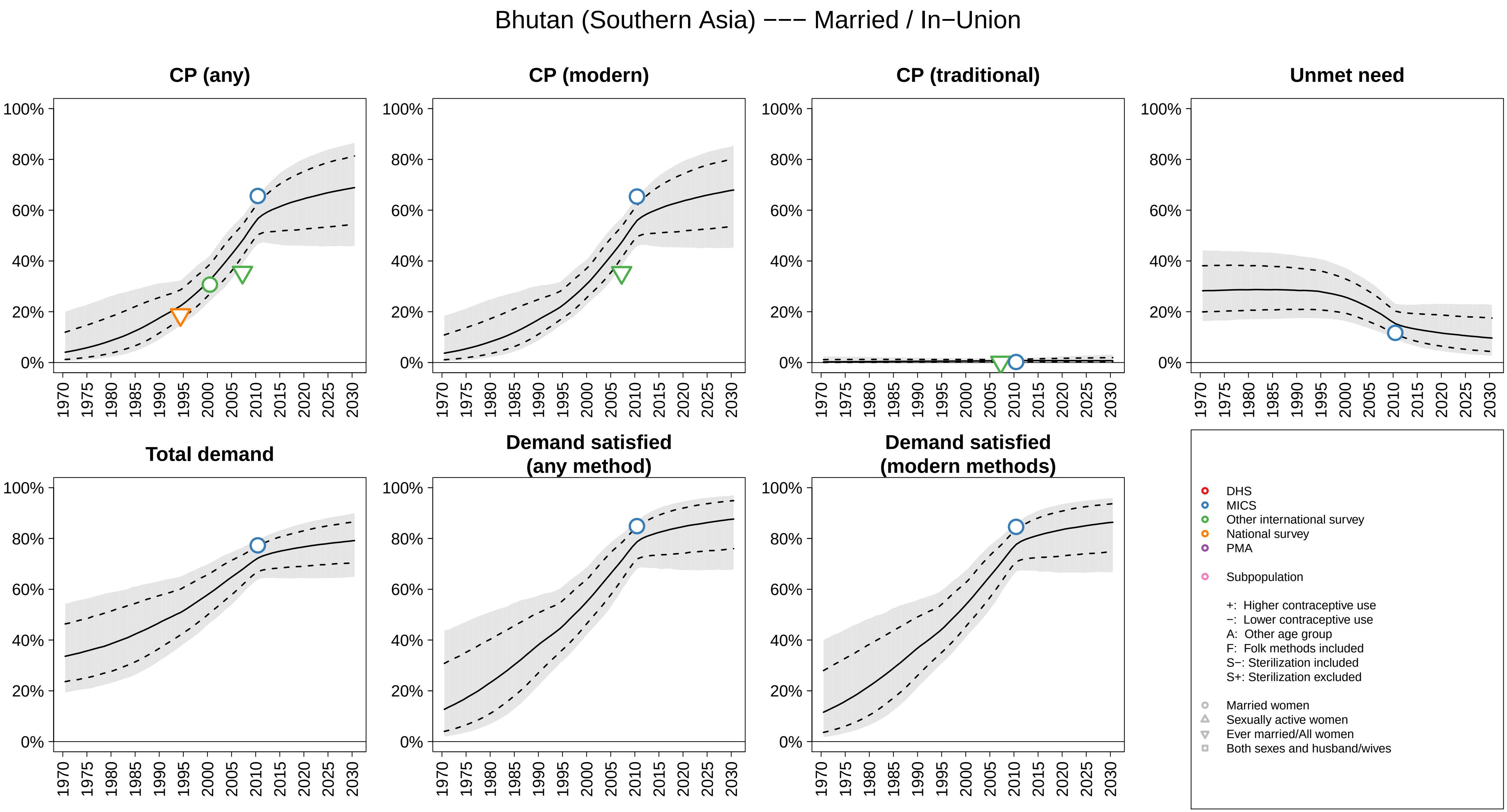
Bhutan (Southern Asia) −−− Married / In−Union
CP (any)
CP (modern)
CP (traditional)
Unmet need
Total demand
Demand satisfied (any method)
Demand satisfied (modern methods)
0%
20%
40%
60%
80%
100%
1970
1975
1980
1985
1990
1995
2000
2005
2010
2015
2020
2025
2030
DHS
MICS
Other international survey
National survey
PMA
Subpopulation
+: Higher contraceptive use
−: Lower contraceptive use
A: Other age group
F: Folk methods included
S−: Sterilization included
S+: Sterilization excluded
Married women
Sexually active women
Ever married/All women
Both sexes and husband/wives

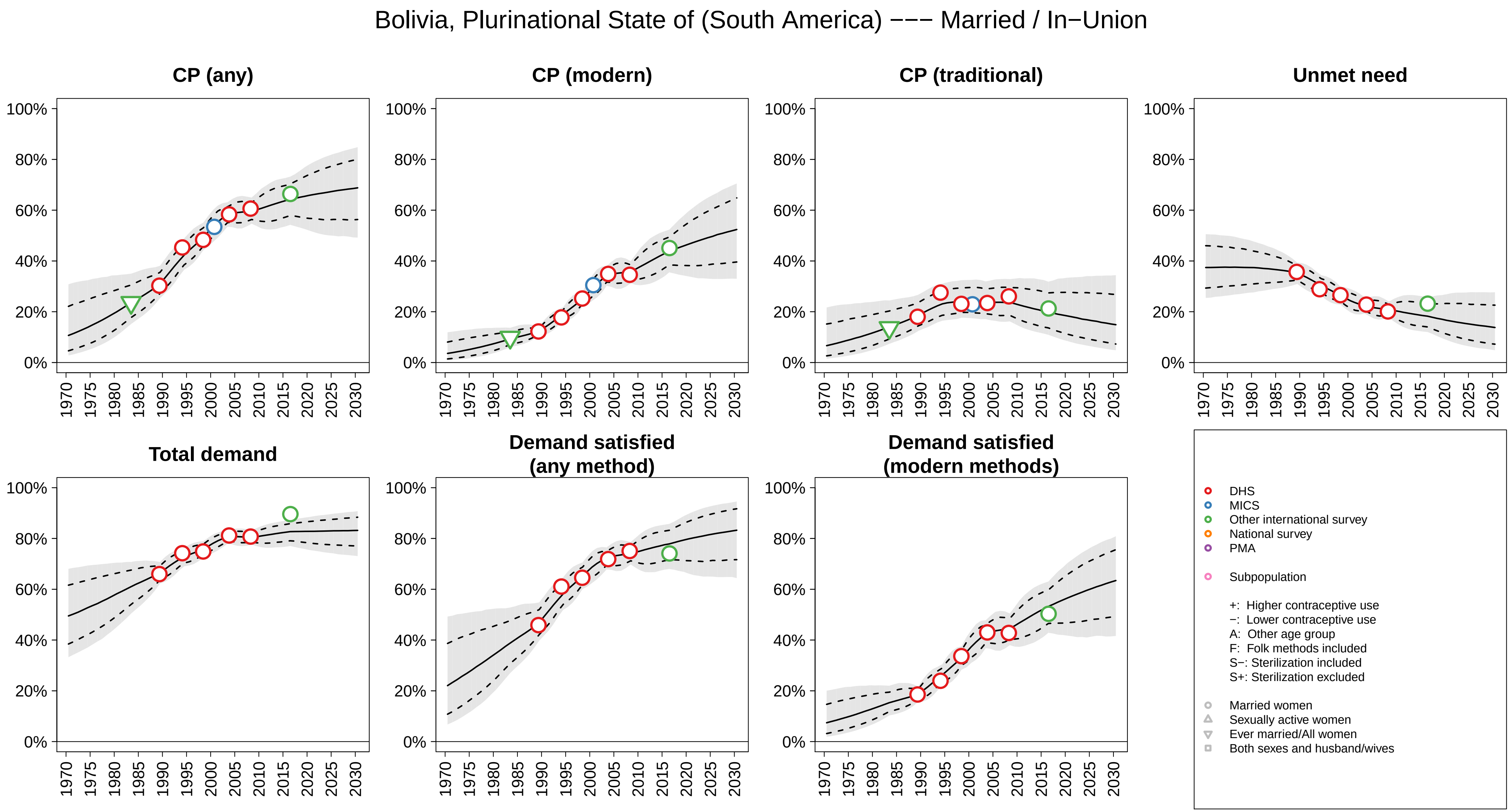
Bolivia, Plurinational State of (South America) −−− Married / In−Union
CP (any)
CP (modern)
CP (traditional)
Unmet need
Total demand
Demand satisfied (any method)
Demand satisfied (modern methods)
0%
20%
40%
60%
80%
100%
1970
1975
1980
1985
1990
1995
2000
2005
2010
2015
2020
2025
2030
DHS
MICS
Other international survey
National survey
PMA
Subpopulation
+: Higher contraceptive use
−: Lower contraceptive use
A: Other age group
F: Folk methods included
S−: Sterilization included
S+: Sterilization excluded
Married women
Sexually active women
Ever married/All women
Both sexes and husband/wives

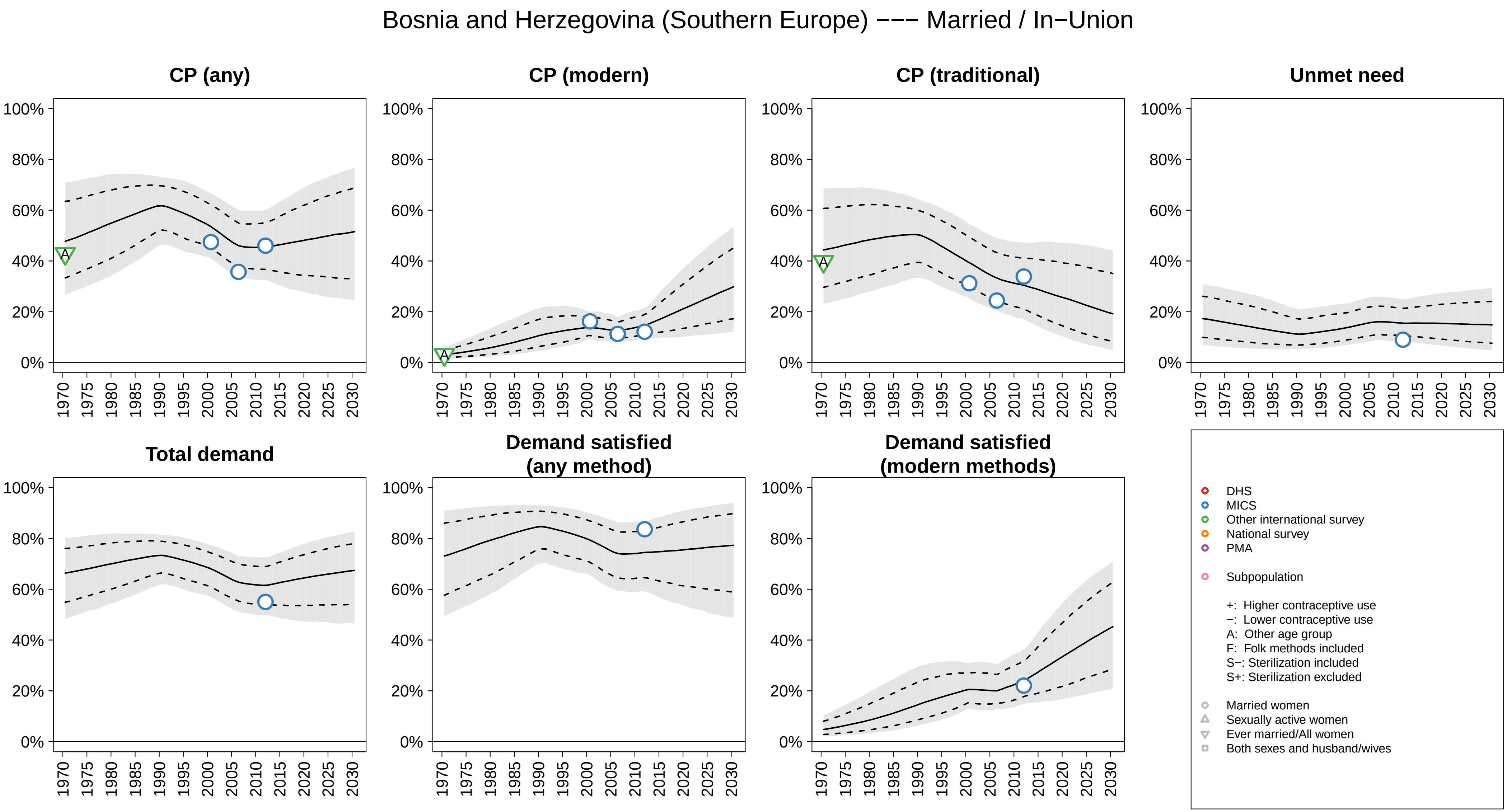
Bosnia and Herzegovina (Southern Europe) −−− Married / In−Union
CP (any)
CP (modern)
CP (traditional)
Unmet need
Total demand
Demand satisfied (any method)
Demand satisfied (modern methods)
100%
80%
60%
40%
20%
0%
1970 1975 1980 1985 1990 1995 2000 2005 2010 2015 2020 2025 2030
DHS
MICS
Other international survey
National survey
PMA
Subpopulation
+: Higher contraceptive use
−: Lower contraceptive use
A: Other age group
F: Folk methods included
S−: Sterilization included
S+: Sterilization excluded
Married women
Sexually active women
Ever married/All women
Both sexes and husband/wives

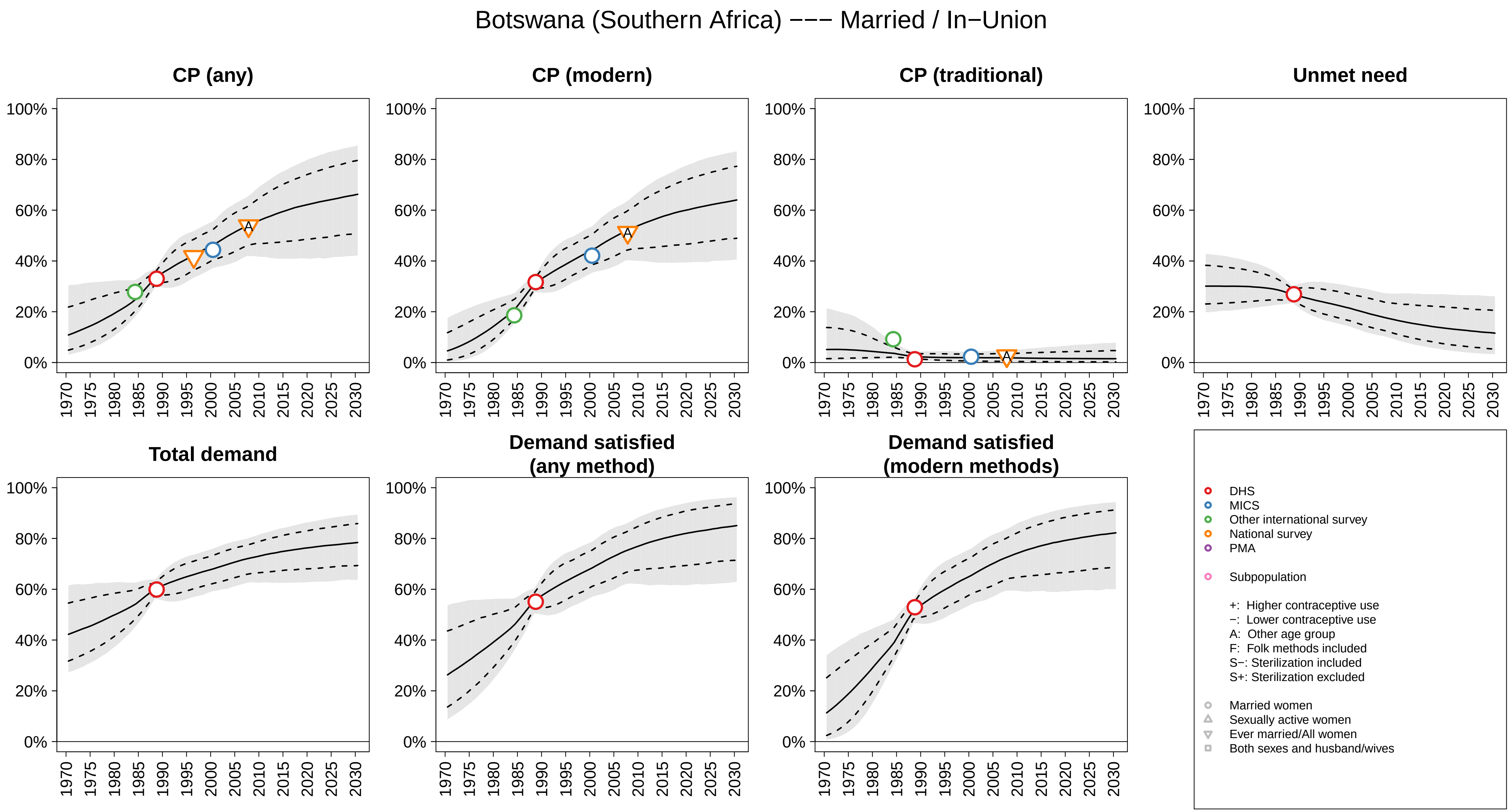
Botswana (Southern Africa) −−− Married / In−Union
CP (any)
CP (modern)
CP (traditional)
Unmet need
Total demand
Demand satisfied (any method)
Demand satisfied (modern methods)
0%
20%
40%
60%
80%
100%
1970
1975
1980
1985
1990
1995
2000
2005
2010
2015
2020
2025
2030
A
DHS
MICS
Other international survey
National survey
PMA
Subpopulation
+: Higher contraceptive use
−: Lower contraceptive use
A: Other age group
F: Folk methods included
S−: Sterilization included
S+: Sterilization excluded
Married women
Sexually active women
Ever married/All women
Both sexes and husband/wives

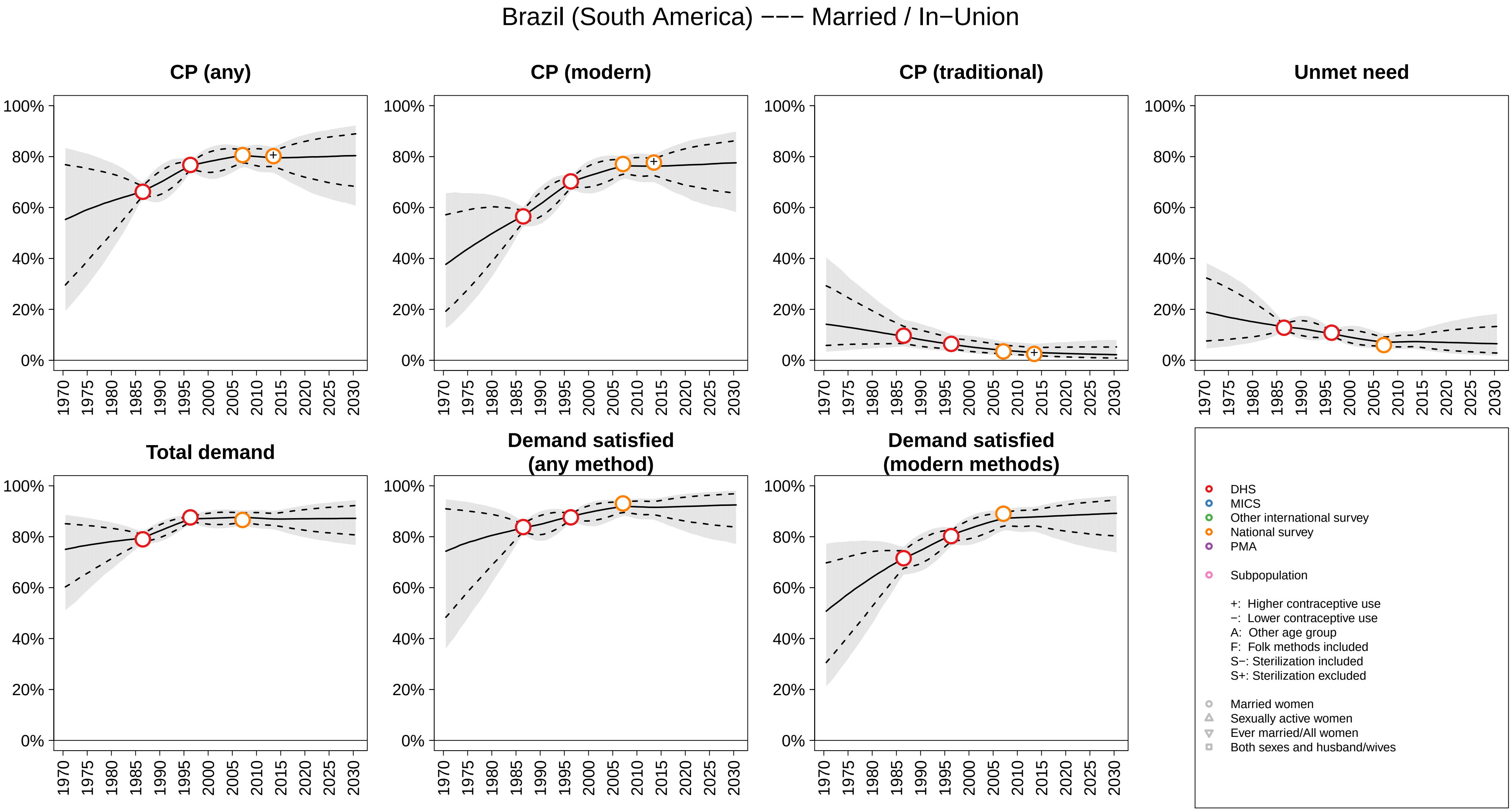
Brazil (South America) −−− Married / In−Union
CP (any)
CP (modern)
CP (traditional)
Unmet need
Total demand
Demand satisfied (any method)
Demand satisfied (modern methods)
0%
20%
40%
60%
80%
100%
1970
1975
1980
1985
1990
1995
2000
2005
2010
2015
2020
2025
2030
DHS
MICS
Other international survey
National survey
PMA
Subpopulation
+: Higher contraceptive use
−: Lower contraceptive use
A: Other age group
F: Folk methods included
S−: Sterilization included
S+: Sterilization excluded
Married women
Sexually active women
Ever married/All women
Both sexes and husband/wives

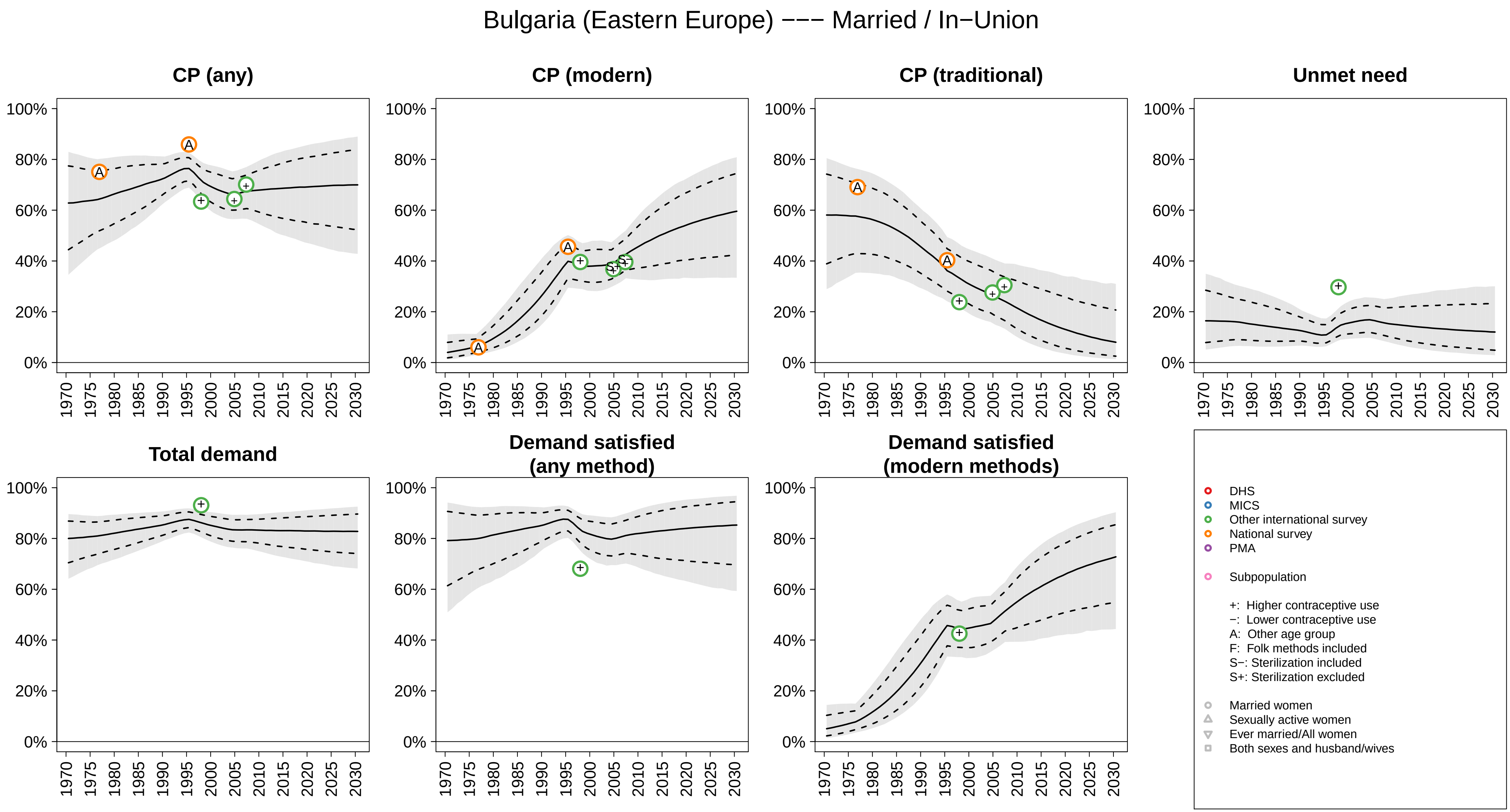
Bulgaria (Eastern Europe) --- Married / In−Union
CP (any)
CP (modern)
CP (traditional)
Unmet need
Total demand
Demand satisfied (any method)
Demand satisfied (modern methods)
100%
80%
60%
40%
20%
0%
1970 1975 1980 1985 1990 1995 2000 2005 2010 2015 2020 2025 2030
DHS
MICS
Other international survey
National survey
PMA
Subpopulation
+: Higher contraceptive use
−: Lower contraceptive use
A: Other age group
F: Folk methods included
S−: Sterilization included
S+: Sterilization excluded
Married women
Sexually active women
Ever married/All women
Both sexes and husband/wives

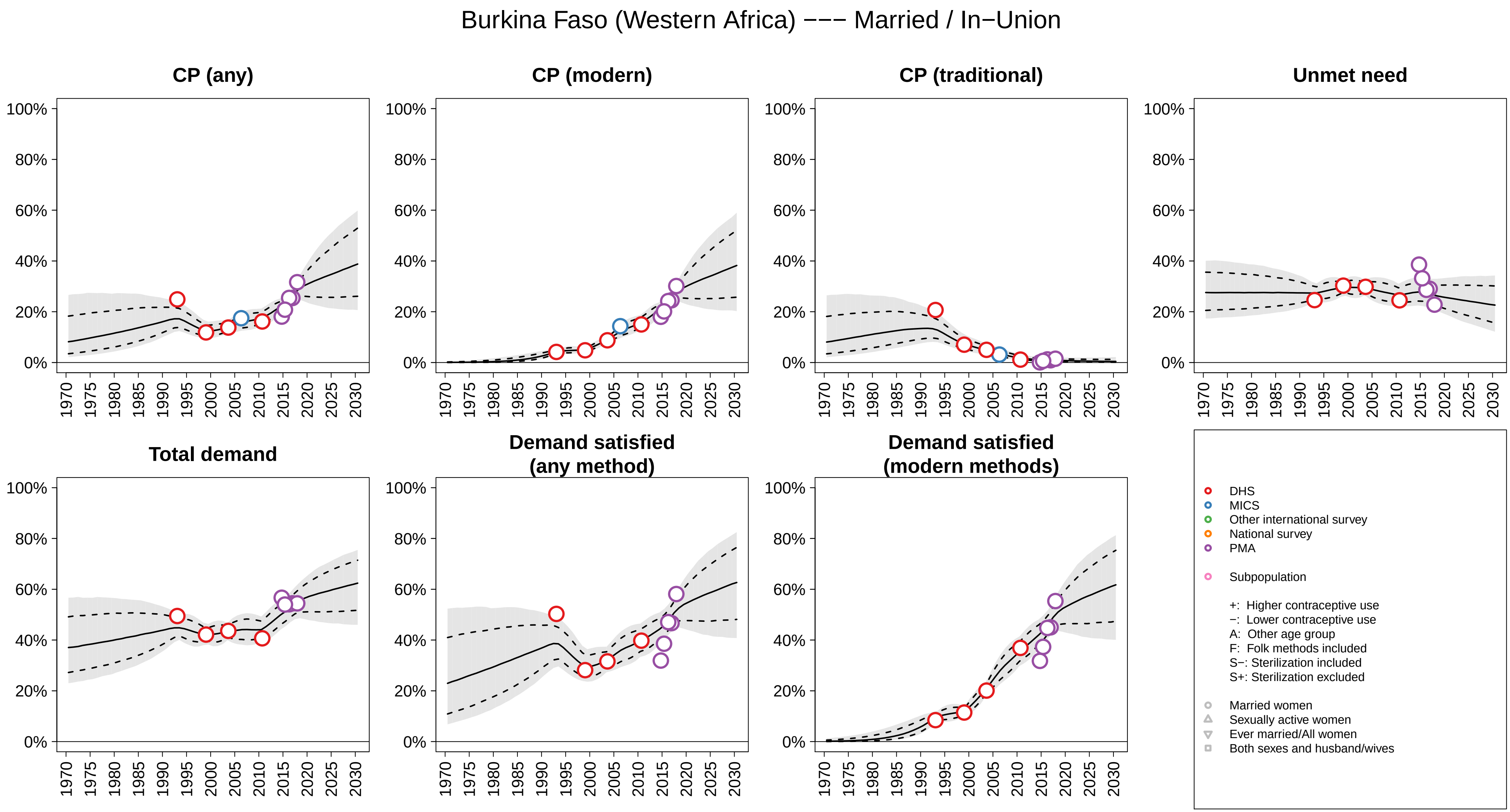
Burkina Faso (Western Africa) −−− Married / In−Union
CP (any)
CP (modern)
CP (traditional)
Unmet need
Total demand
Demand satisfied (any method)
Demand satisfied (modern methods)
100%
80%
60%
40%
20%
0%
1970
1975
1980
1985
1990
1995
2000
2005
2010
2015
2020
2025
2030
DHS
MICS
Other international survey
National survey
PMA
Subpopulation
+: Higher contraceptive use
−: Lower contraceptive use
A: Other age group
F: Folk methods included
S−: Sterilization included
S+: Sterilization excluded
Married women
Sexually active women
Ever married/All women
Both sexes and husband/wives

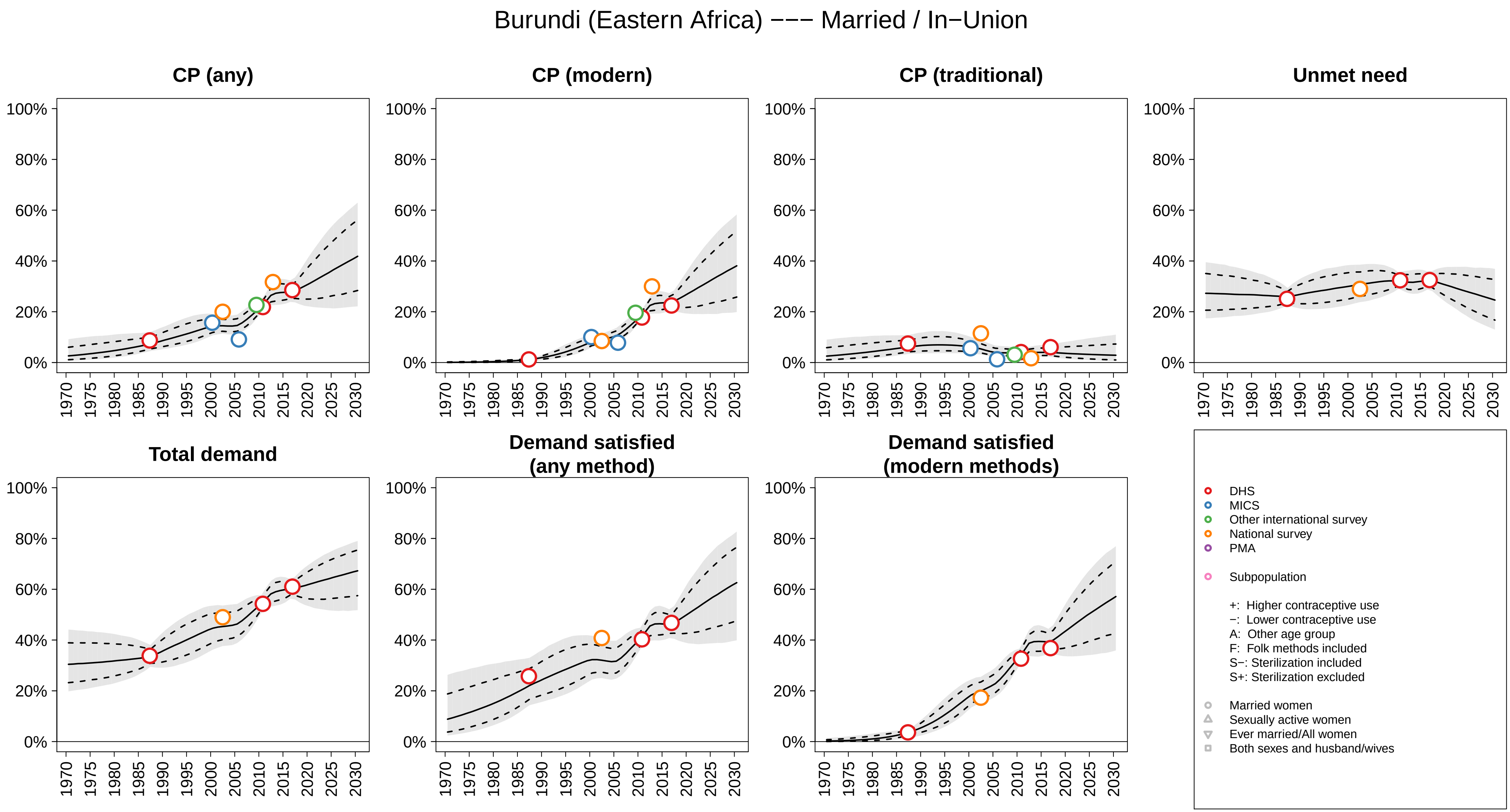
Burundi (Eastern Africa) --- Married / In−Union
CP (any)
CP (modern)
CP (traditional)
Unmet need
Total demand
Demand satisfied (any method)
Demand satisfied (modern methods)
0%
20%
40%
60%
80%
100%
1970
1975
1980
1985
1990
1995
2000
2005
2010
2015
2020
2025
2030
DHS
MICS
Other international survey
National survey
PMA
Subpopulation
+: Higher contraceptive use
−: Lower contraceptive use
A: Other age group
F: Folk methods included
S−: Sterilization included
S+: Sterilization excluded
Married women
Sexually active women
Ever married/All women
Both sexes and husband/wives

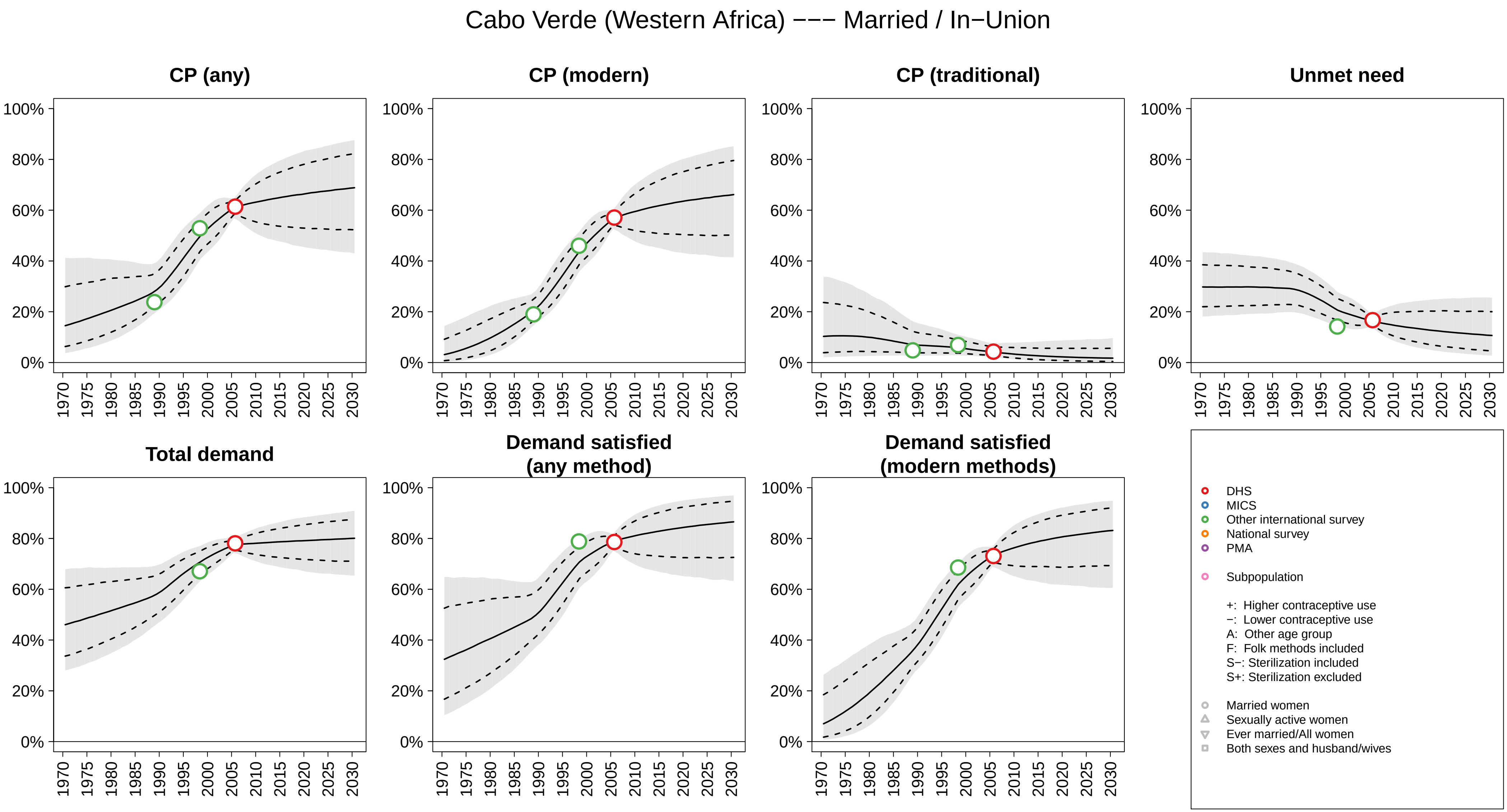
Cabo Verde (Western Africa) −−− Married / In−Union
CP (any)
CP (modern)
CP (traditional)
Unmet need
Total demand
Demand satisfied (any method)
Demand satisfied (modern methods)
0%
20%
40%
60%
80%
100%
1970
1975
1980
1985
1990
1995
2000
2005
2010
2015
2020
2025
2030
DHS
MICS
Other international survey
National survey
PMA
Subpopulation
+: Higher contraceptive use
−: Lower contraceptive use
A: Other age group
F: Folk methods included
S−: Sterilization included
S+: Sterilization excluded
Married women
Sexually active women
Ever married/All women
Both sexes and husband/wives

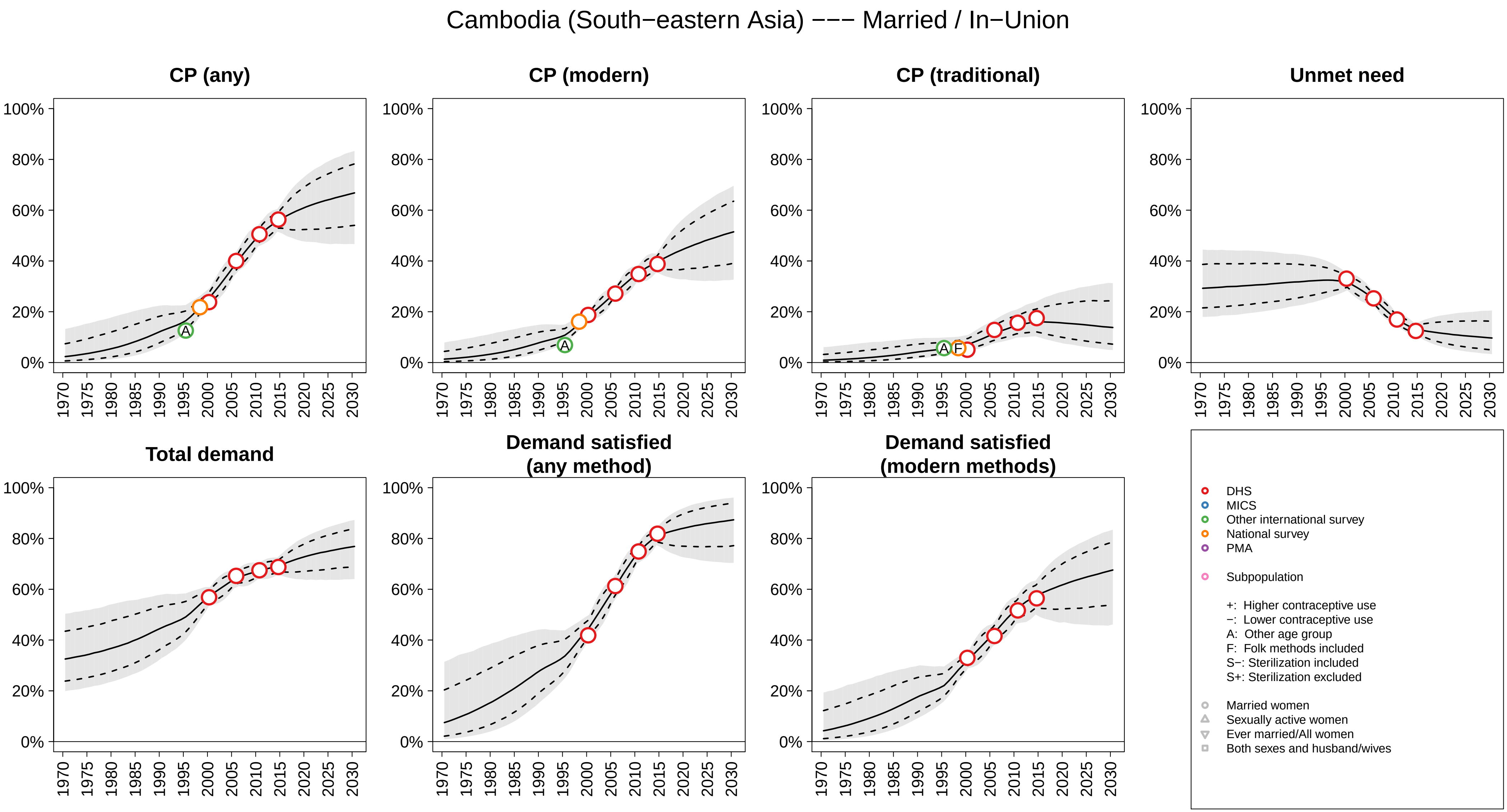
Cambodia (South−eastern Asia) −−− Married / In−Union
CP (any)
CP (modern)
CP (traditional)
Unmet need
Total demand
Demand satisfied (any method)
Demand satisfied (modern methods)
0%
20%
40%
60%
80%
100%
1970
1975
1980
1985
1990
1995
2000
2005
2010
2015
2020
2025
2030
A
F
DHS
MICS
Other international survey
National survey
PMA
Subpopulation
+: Higher contraceptive use
−: Lower contraceptive use
A: Other age group
F: Folk methods included
S−: Sterilization included
S+: Sterilization excluded
Married women
Sexually active women
Ever married/All women
Both sexes and husband/wives

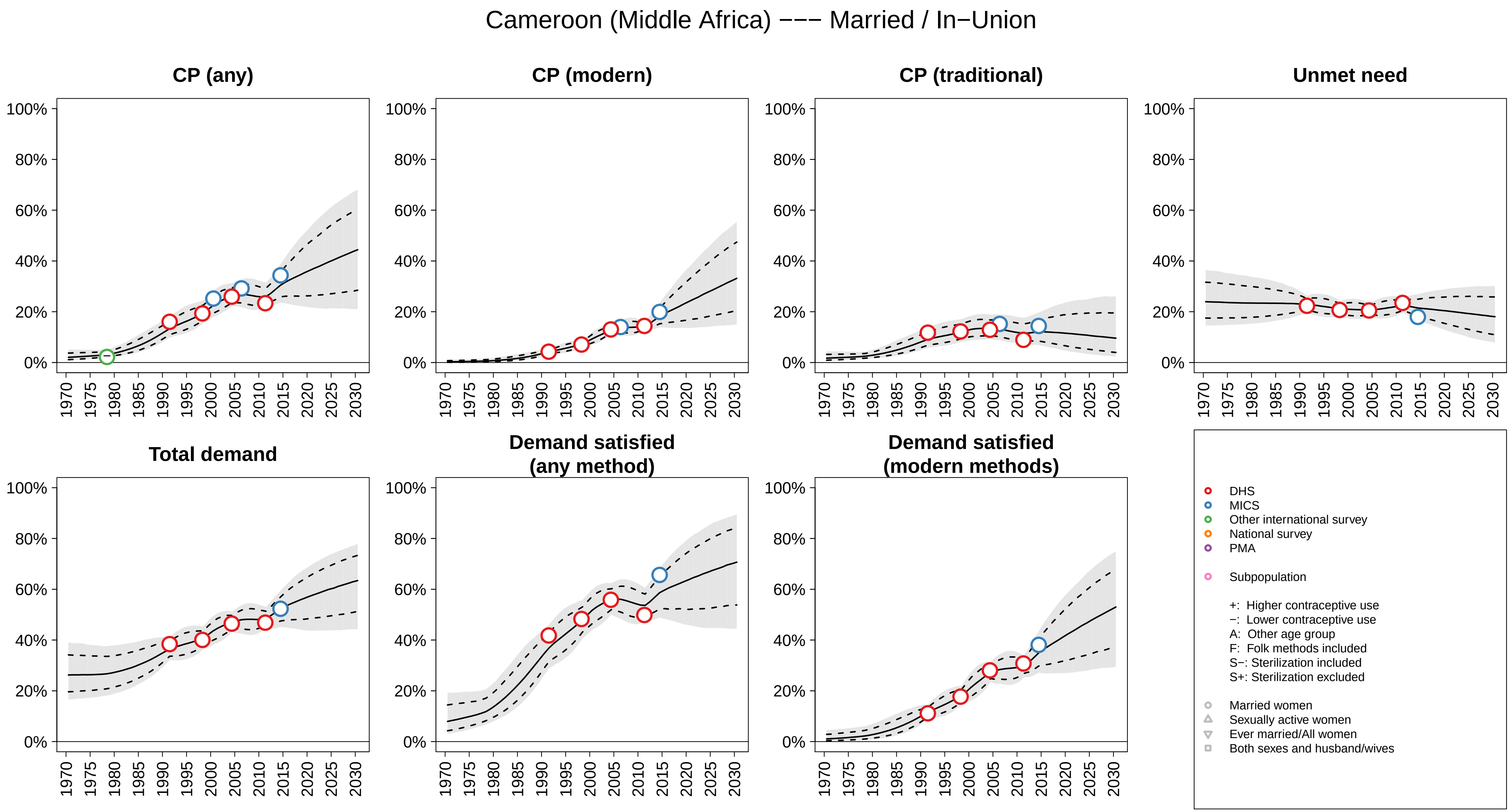
Cameroon (Middle Africa) --- Married / In-Union
CP (any)
CP (modern)
CP (traditional)
Unmet need
Total demand
Demand satisfied (any method)
Demand satisfied (modern methods)
0%
20%
40%
60%
80%
100%
1970
1975
1980
1985
1990
1995
2000
2005
2010
2015
2020
2025
2030
DHS
MICS
Other international survey
National survey
PMA
Subpopulation
+: Higher contraceptive use
−: Lower contraceptive use
A: Other age group
F: Folk methods included
S−: Sterilization included
S+: Sterilization excluded
Married women
Sexually active women
Ever married/All women
Both sexes and husband/wives

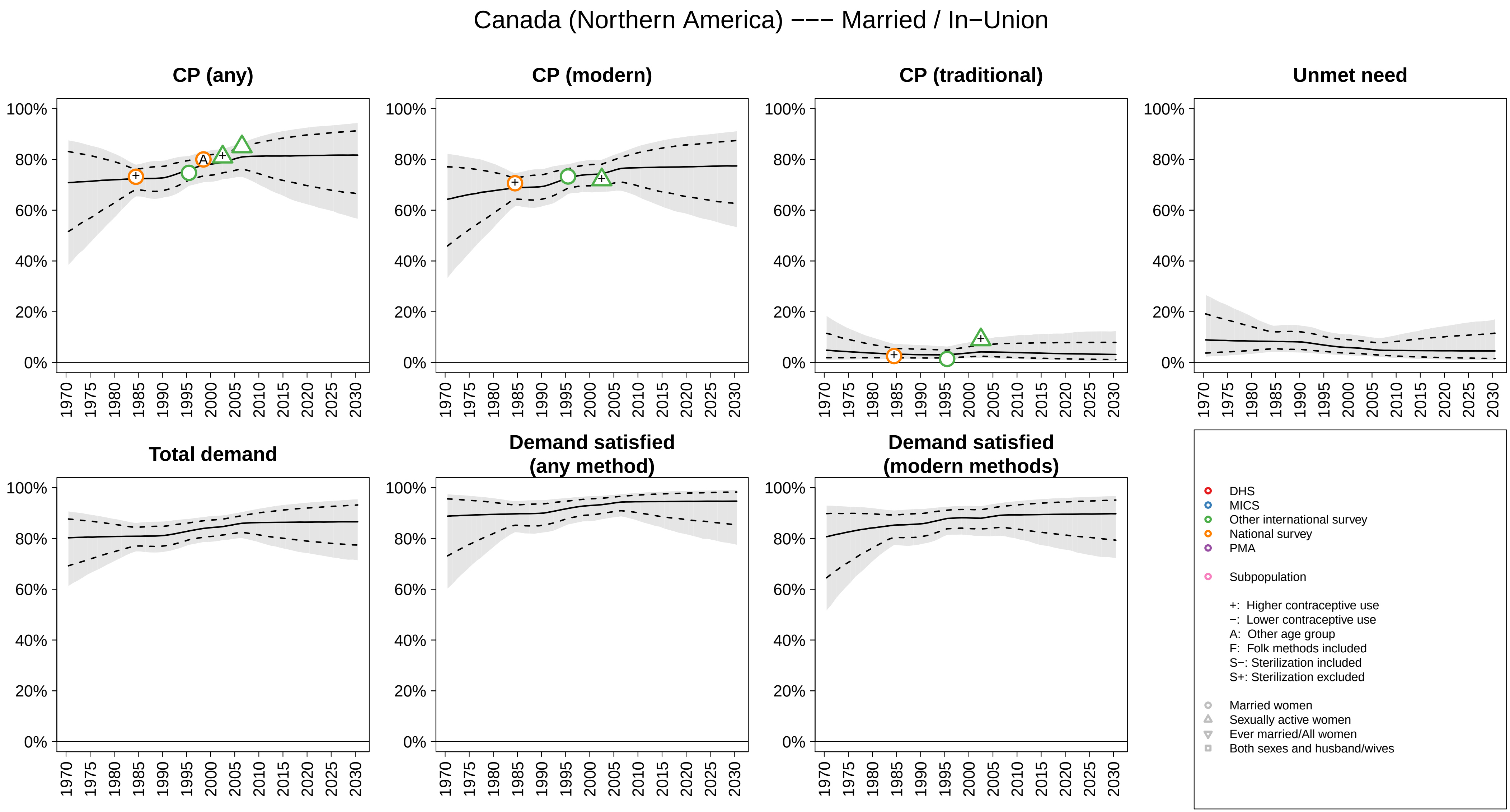
Canada (Northern America) −−− Married / In−Union
CP (any)
CP (modern)
CP (traditional)
Unmet need
Total demand
Demand satisfied (any method)
Demand satisfied (modern methods)
100%
80%
60%
40%
20%
0%
1970
1975
1980
1985
1990
1995
2000
2005
2010
2015
2020
2025
2030
DHS
MICS
Other international survey
National survey
PMA
Subpopulation
+: Higher contraceptive use
−: Lower contraceptive use
A: Other age group
F: Folk methods included
S−: Sterilization included
S+: Sterilization excluded
Married women
Sexually active women
Ever married/All women
Both sexes and husband/wives

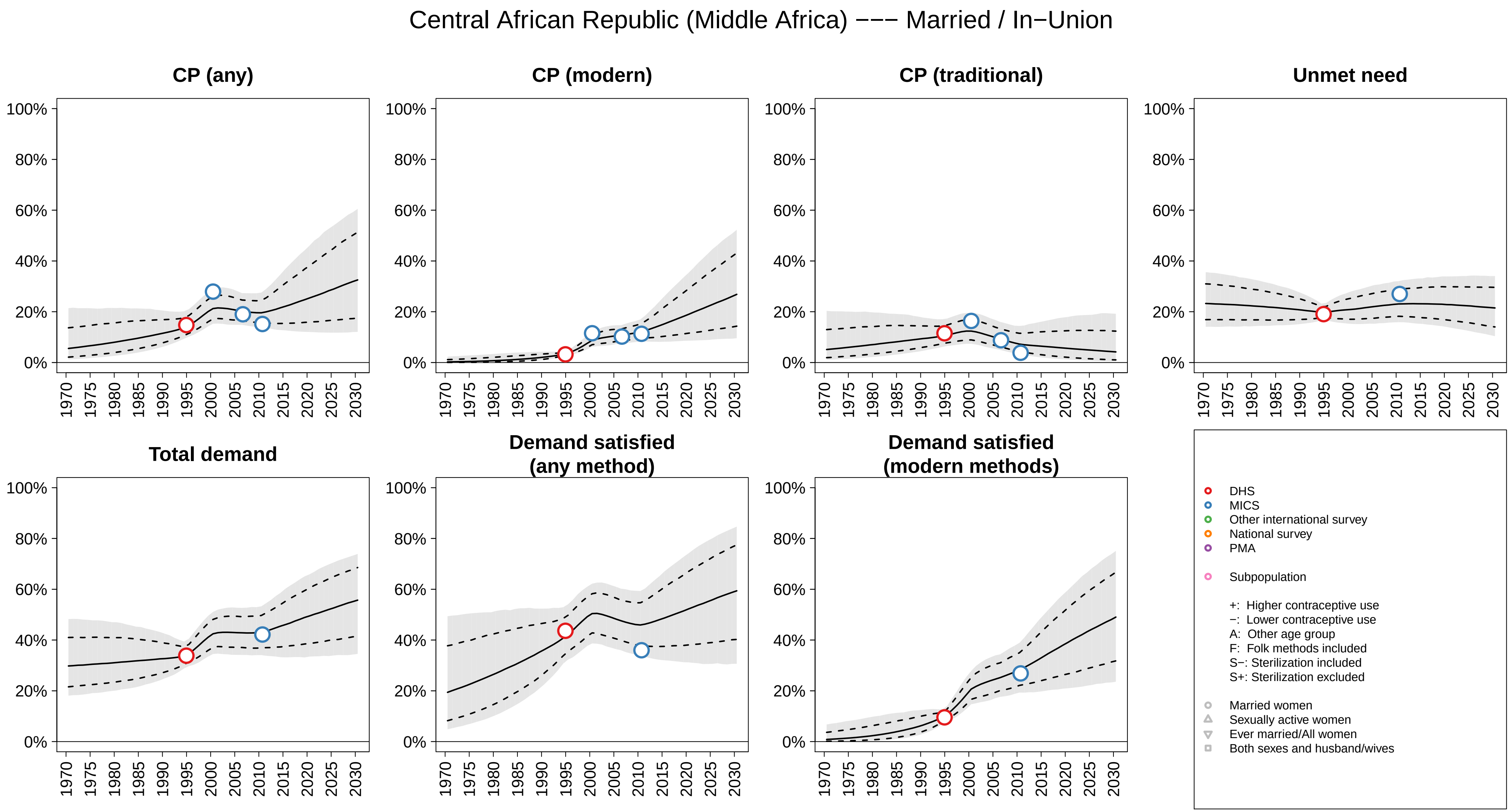
Central African Republic (Middle Africa) --- Married / In−Union
CP (any)
CP (modern)
CP (traditional)
Unmet need
Total demand
Demand satisfied (any method)
Demand satisfied (modern methods)
100%
80%
60%
40%
20%
0%
1970
1975
1980
1985
1990
1995
2000
2005
2010
2015
2020
2025
2030
DHS
MICS
Other international survey
National survey
PMA
Subpopulation
+: Higher contraceptive use
−: Lower contraceptive use
A: Other age group
F: Folk methods included
S−: Sterilization included
S+: Sterilization excluded
Married women
Sexually active women
Ever married/All women
Both sexes and husband/wives

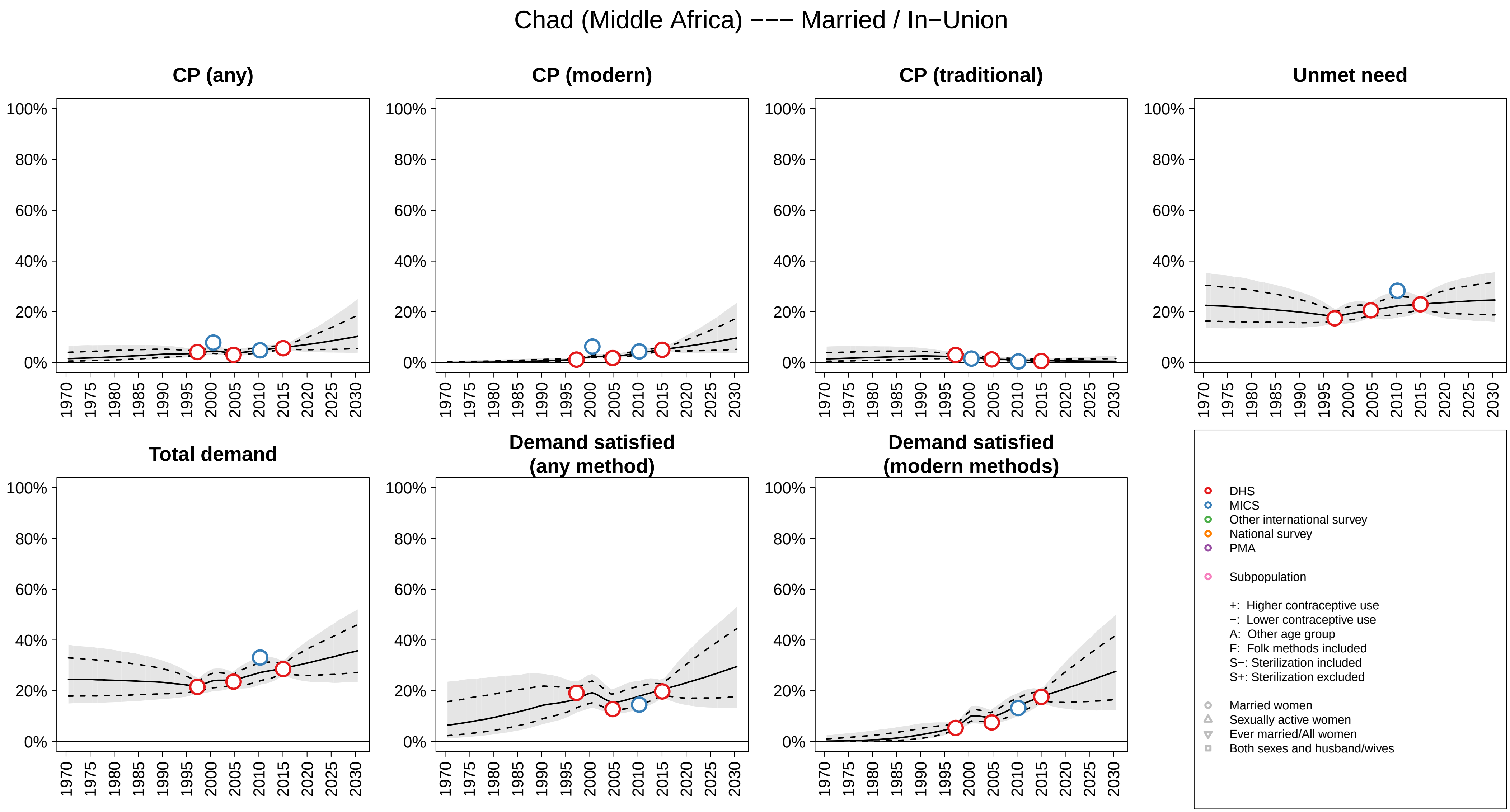
Chad (Middle Africa) −−− Married / In−Union
CP (any)
CP (modern)
CP (traditional)
Unmet need
Total demand
Demand satisfied (any method)
Demand satisfied (modern methods)
0%
20%
40%
60%
80%
100%
1970
1975
1980
1985
1990
1995
2000
2005
2010
2015
2020
2025
2030
DHS
MICS
Other international survey
National survey
PMA
Subpopulation
+: Higher contraceptive use
−: Lower contraceptive use
A: Other age group
F: Folk methods included
S−: Sterilization included
S+: Sterilization excluded
Married women
Sexually active women
Ever married/All women
Both sexes and husband/wives

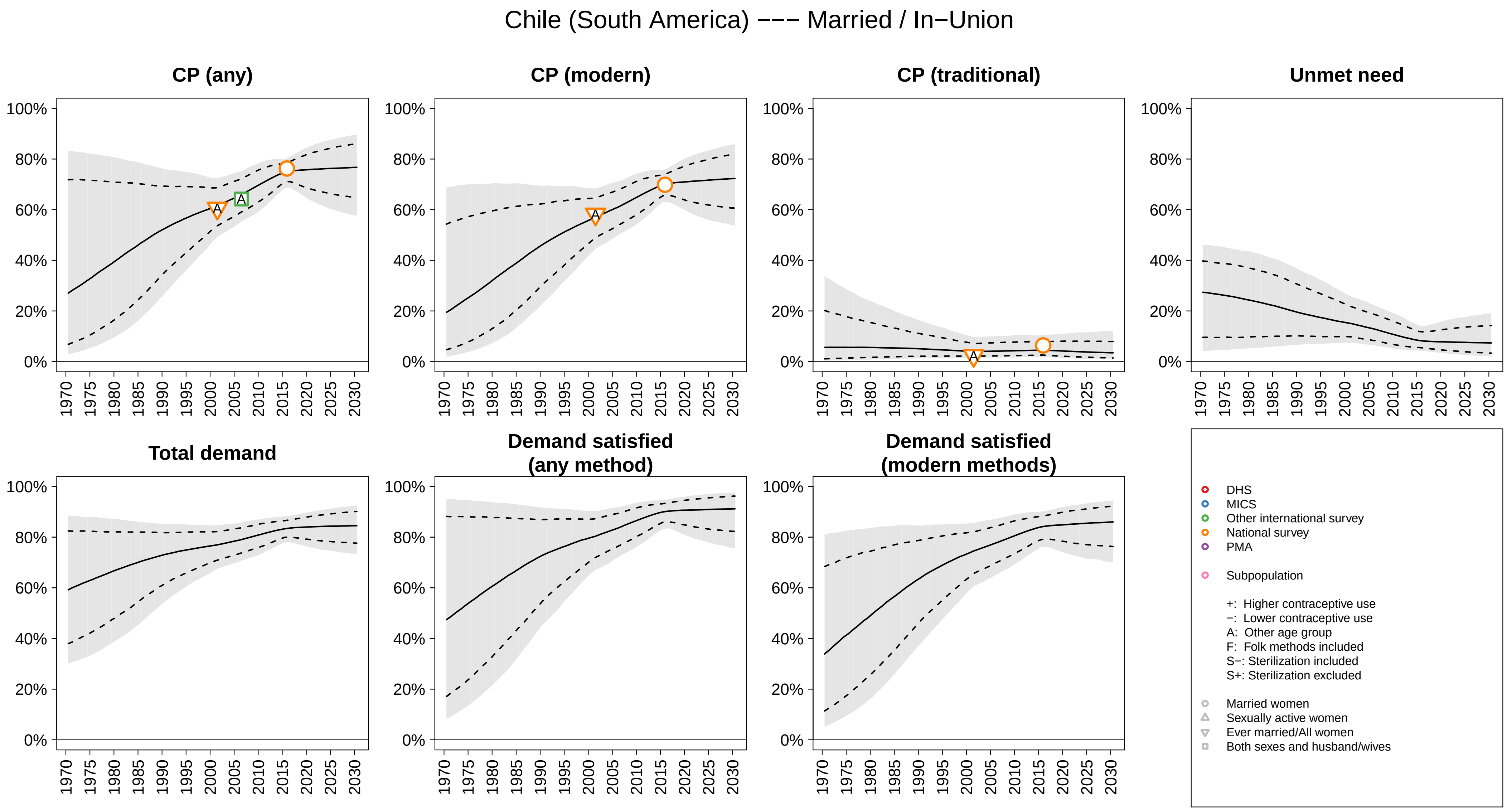
Chile (South America) --- Married / In−Union
CP (any)
CP (modern)
CP (traditional)
Unmet need
Total demand
Demand satisfied (any method)
Demand satisfied (modern methods)
0%
20%
40%
60%
80%
100%
1970
1975
1980
1985
1990
1995
2000
2005
2010
2015
2020
2025
2030
A
DHS
MICS
Other international survey
National survey
PMA
Subpopulation
+: Higher contraceptive use
−: Lower contraceptive use
A: Other age group
F: Folk methods included
S−: Sterilization included
S+: Sterilization excluded
Married women
Sexually active women
Ever married/All women
Both sexes and husband/wives

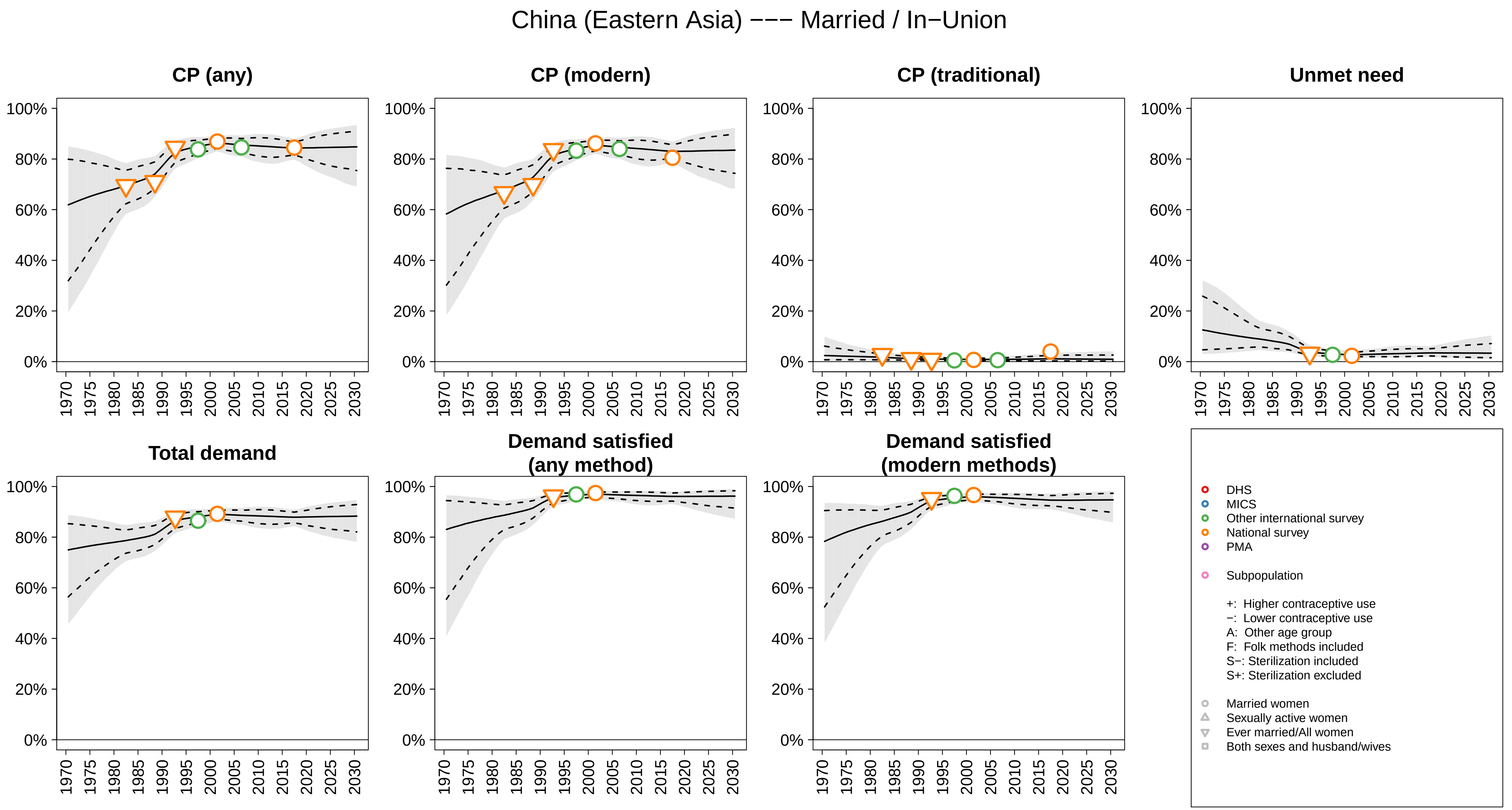
China (Eastern Asia) --- Married / In−Union
CP (any)
CP (modern)
CP (traditional)
Unmet need
Total demand
Demand satisfied (any method)
Demand satisfied (modern methods)
100%
80%
60%
40%
20%
0%
1970 1975 1980 1985 1990 1995 2000 2005 2010 2015 2020 2025 2030
DHS
MICS
Other international survey
National survey
PMA
Subpopulation
+: Higher contraceptive use
−: Lower contraceptive use
A: Other age group
F: Folk methods included
S−: Sterilization included
S+: Sterilization excluded
Married women
Sexually active women
Ever married/All women
Both sexes and husband/wives

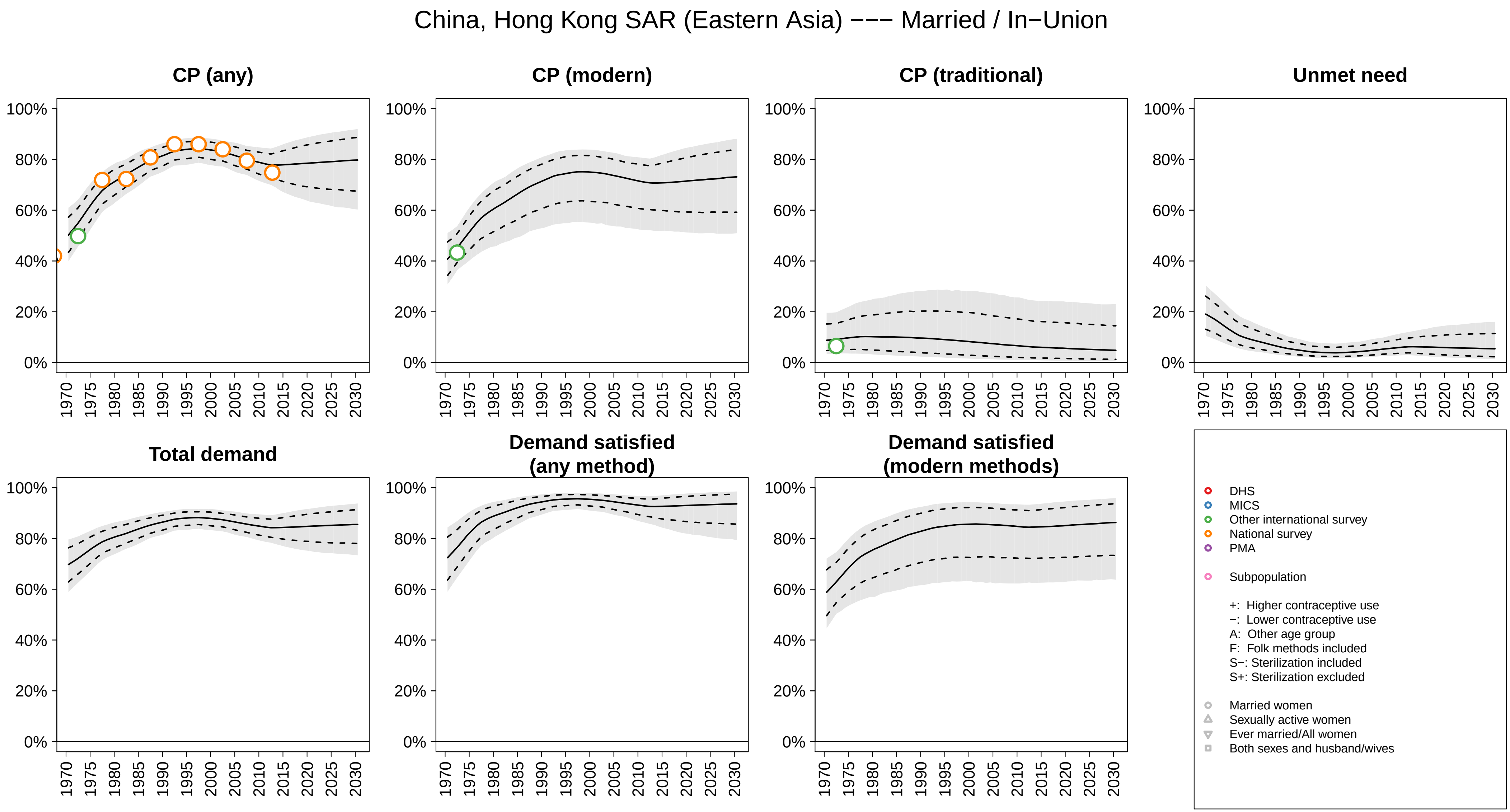
China, Hong Kong SAR (Eastern Asia) --- Married / In−Union
CP (any)
CP (modern)
CP (traditional)
Unmet need
Total demand
Demand satisfied (any method)
Demand satisfied (modern methods)
0%
20%
40%
60%
80%
100%
1970
1975
1980
1985
1990
1995
2000
2005
2010
2015
2020
2025
2030
DHS
MICS
Other international survey
National survey
PMA
Subpopulation
+: Higher contraceptive use
−: Lower contraceptive use
A: Other age group
F: Folk methods included
S−: Sterilization included
S+: Sterilization excluded
Married women
Sexually active women
Ever married/All women
Both sexes and husband/wives

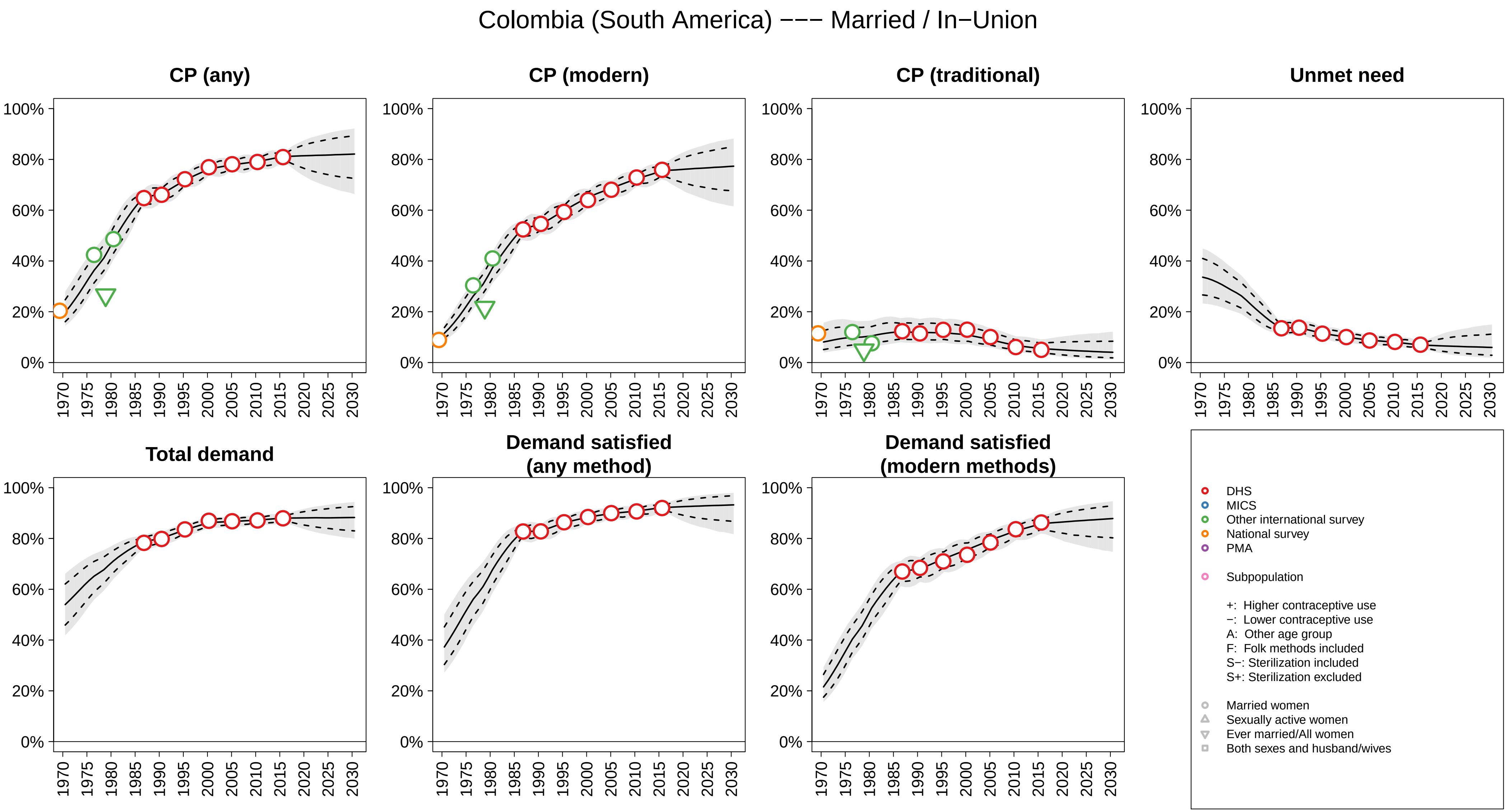
Colombia (South America) −−− Married / In−Union
CP (any)
CP (modern)
CP (traditional)
Unmet need
Total demand
Demand satisfied
(any method)
Demand satisfied
(modern methods)
100%
80%
60%
40%
20%
0%
1970
1975
1980
1985
1990
1995
2000
2005
2010
2015
2020
2025
2030
DHS
MICS
Other international survey
National survey
PMA
Subpopulation
+: Higher contraceptive use
−: Lower contraceptive use
A: Other age group
F: Folk methods included
S−: Sterilization included
S+: Sterilization excluded
Married women
Sexually active women
Ever married/All women
Both sexes and husband/wives

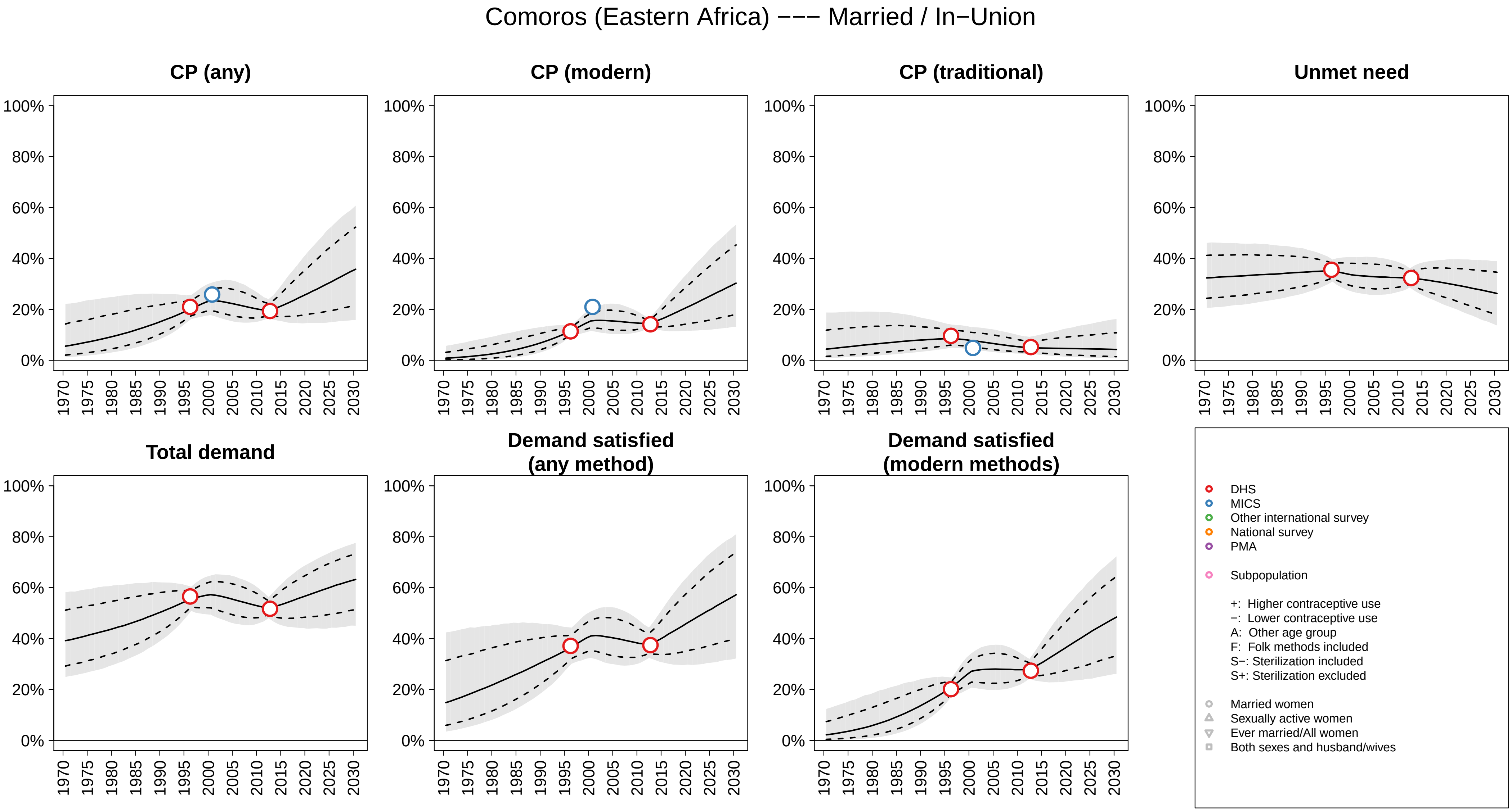
Comoros (Eastern Africa) −−− Married / In−Union
CP (any)
CP (modern)
CP (traditional)
Unmet need
Total demand
Demand satisfied (any method)
Demand satisfied (modern methods)
100%
80%
60%
40%
20%
0%
1970 1975 1980 1985 1990 1995 2000 2005 2010 2015 2020 2025 2030
DHS
MICS
Other international survey
National survey
PMA
Subpopulation
+: Higher contraceptive use
−: Lower contraceptive use
A: Other age group
F: Folk methods included
S−: Sterilization included
S+: Sterilization excluded
Married women
Sexually active women
Ever married/All women
Both sexes and husband/wives

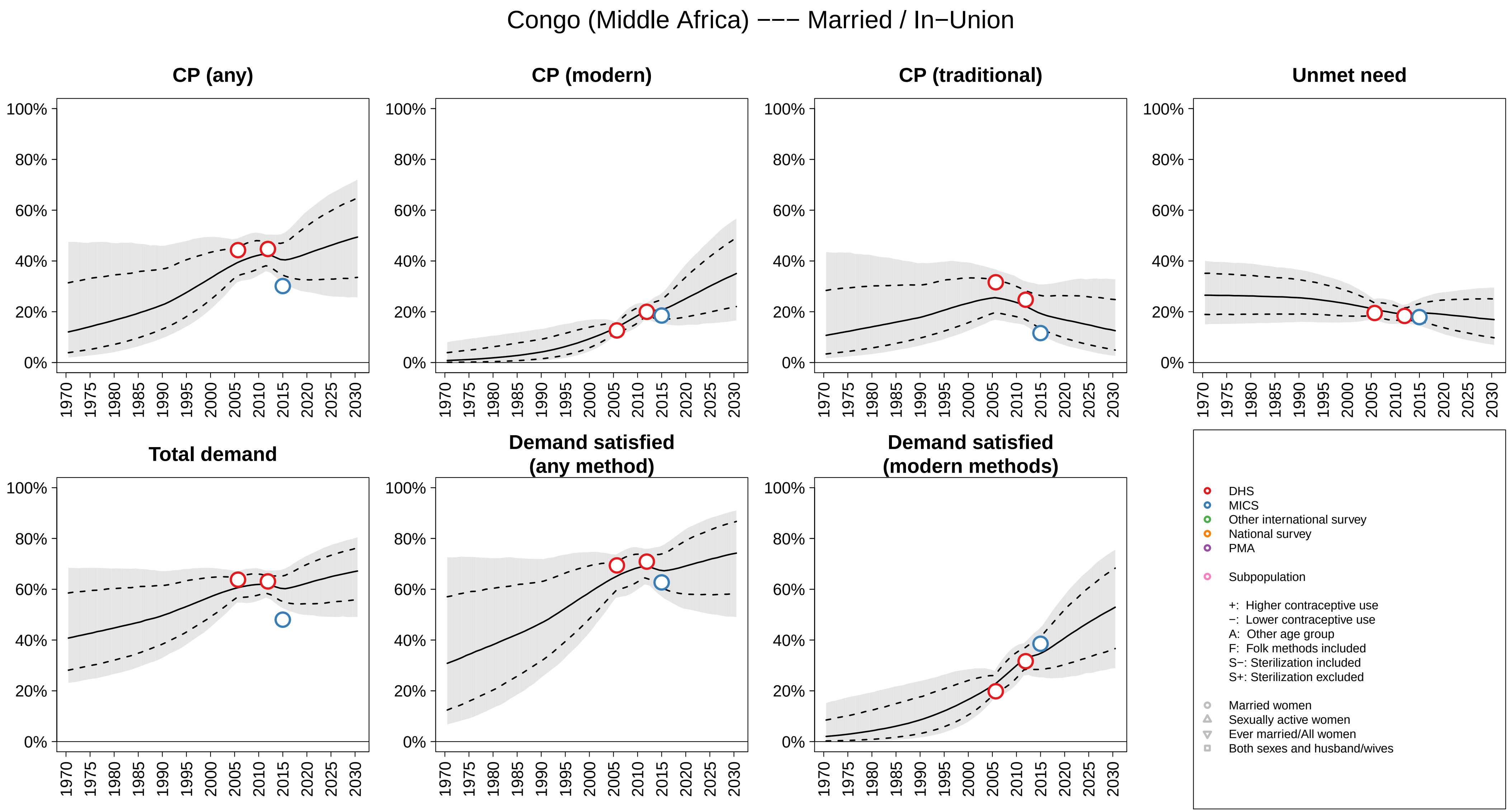

Congo (Middle Africa) --- Married / In−Union
CP (any)
CP (modern)
CP (traditional)
Unmet need
Total demand
Demand satisfied (any method)
Demand satisfied (modern methods)
0%
20%
40%
60%
80%
100%
1970
1975
1980
1985
1990
1995
2000
2005
2010
2015
2020
2025
2030
DHS
MICS
Other international survey
National survey
PMA
Subpopulation
+: Higher contraceptive use
−: Lower contraceptive use
A: Other age group
F: Folk methods included
S−: Sterilization included
S+: Sterilization excluded
Married women
Sexually active women
Ever married/All women
Both sexes and husband/wives

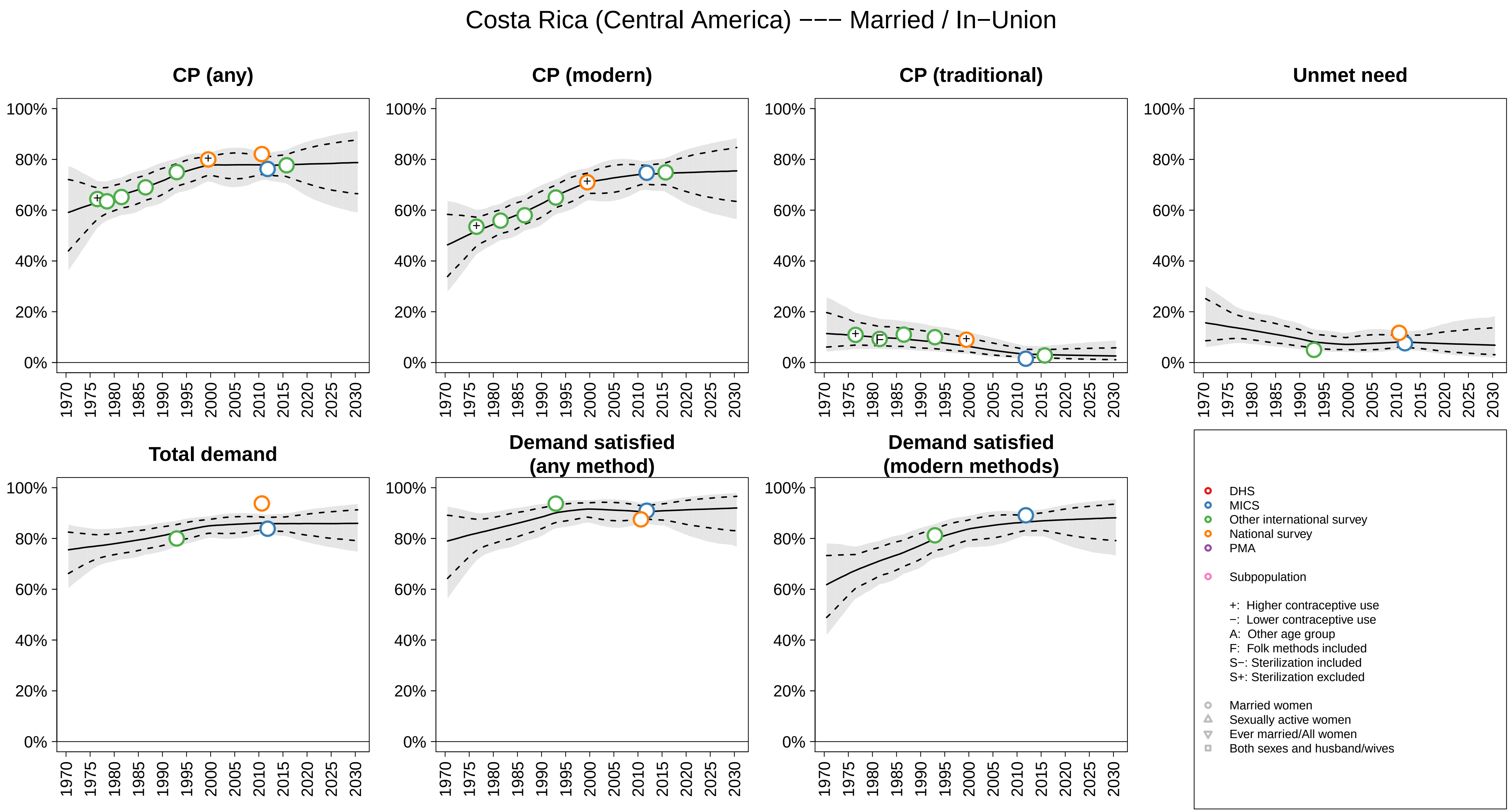
Costa Rica (Central America) --- Married / In−Union
CP (any)
CP (modern)
CP (traditional)
Unmet need
Total demand
Demand satisfied (any method)
Demand satisfied (modern methods)
0%
20%
40%
60%
80%
100%
1970
1975
1980
1985
1990
1995
2000
2005
2010
2015
2020
2025
2030
DHS
MICS
Other international survey
National survey
PMA
Subpopulation
+: Higher contraceptive use
−: Lower contraceptive use
A: Other age group
F: Folk methods included
S−: Sterilization included
S+: Sterilization excluded
Married women
Sexually active women
Ever married/All women
Both sexes and husband/wives

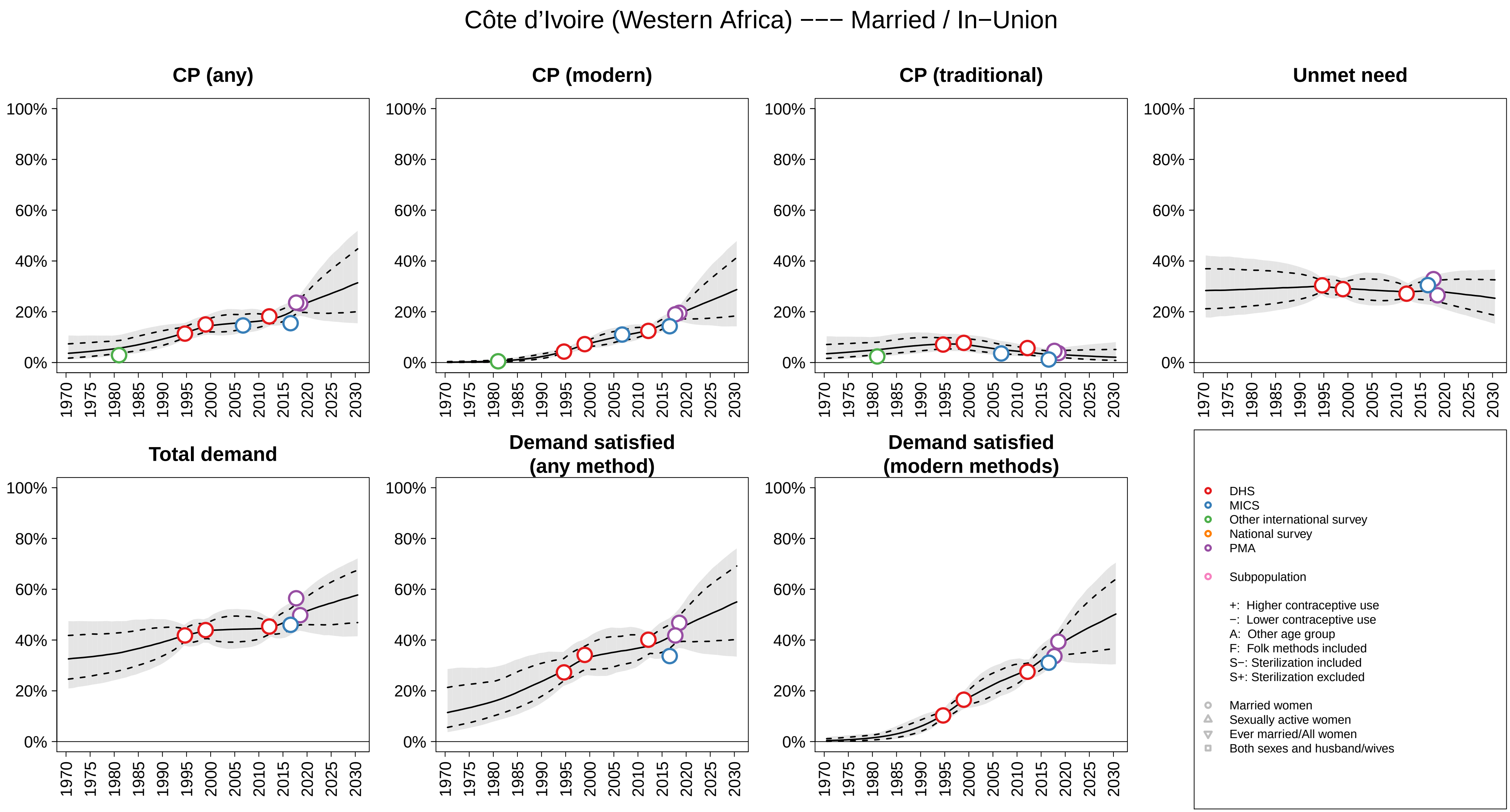

Côte d'Ivoire (Western Africa) −−− Married / In−Union
CP (any)
CP (modern)
CP (traditional)
Unmet need
Total demand
Demand satisfied (any method)
Demand satisfied (modern methods)
DHS
MICS
Other international survey
National survey
PMA
Subpopulation
+: Higher contraceptive use
−: Lower contraceptive use
A: Other age group
F: Folk methods included
S−: Sterilization included
S+: Sterilization excluded
Married women
Sexually active women
Ever married/All women
Both sexes and husband/wives

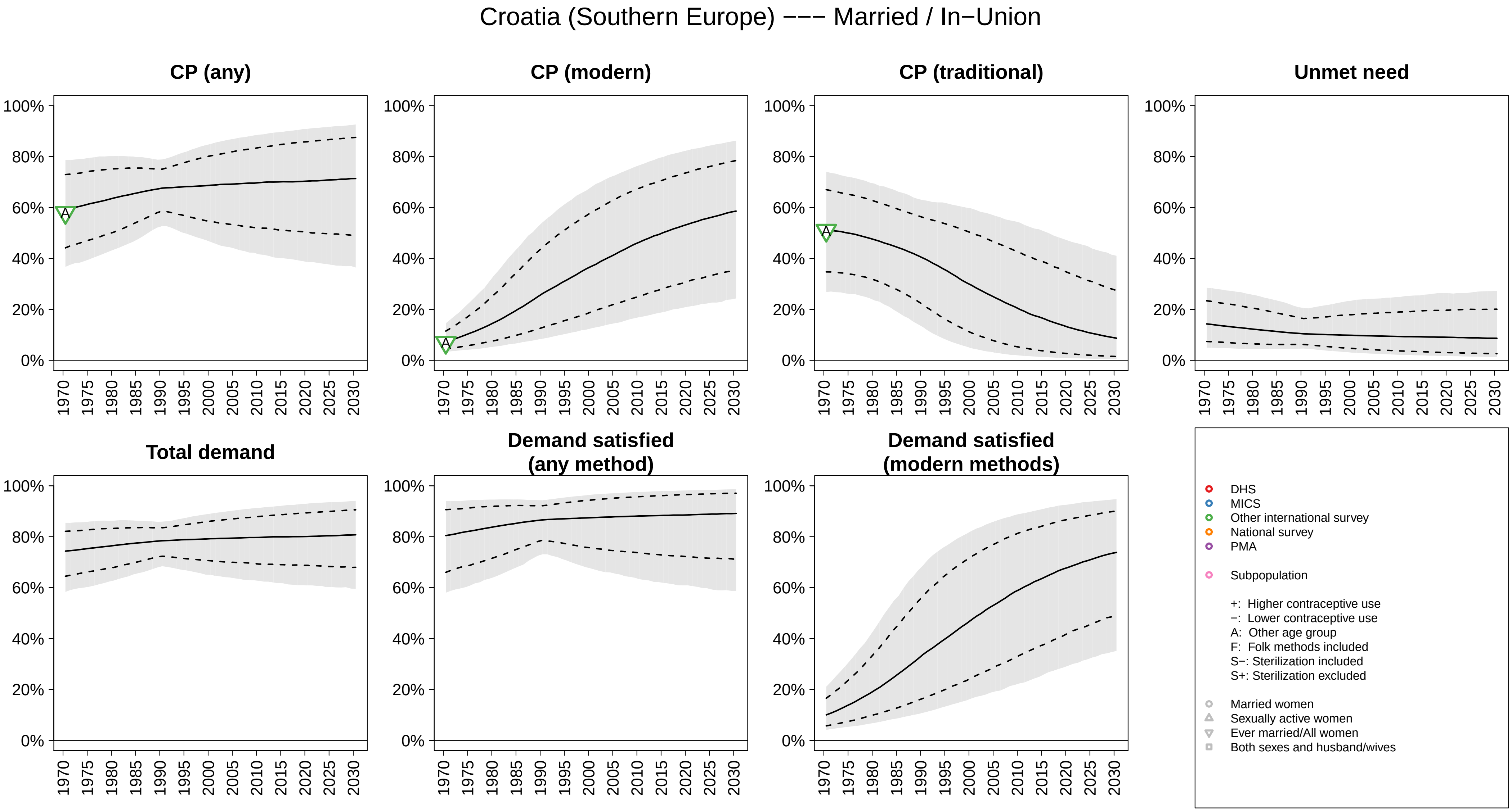

Croatia (Southern Europe) −−− Married / In−Union
CP (any)
CP (modern)
CP (traditional)
Unmet need
Total demand
Demand satisfied (any method)
Demand satisfied (modern methods)
100%
80%
60%
40%
20%
0%
1970 1975 1980 1985 1990 1995 2000 2005 2010 2015 2020 2025 2030
A
DHS
MICS
Other international survey
National survey
PMA
Subpopulation
+: Higher contraceptive use
−: Lower contraceptive use
A: Other age group
F: Folk methods included
S−: Sterilization included
S+: Sterilization excluded
Married women
Sexually active women
Ever married/All women
Both sexes and husband/wives

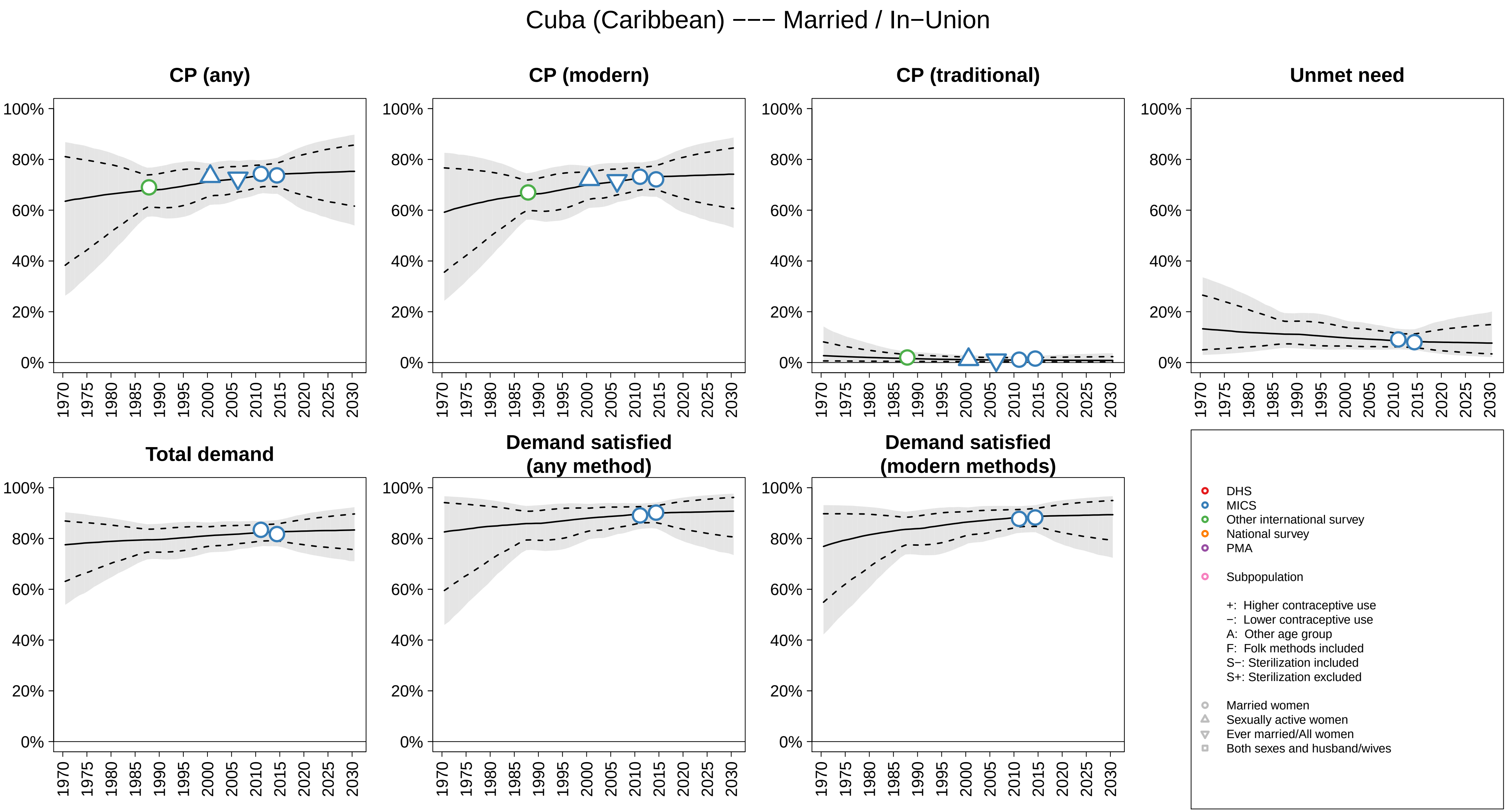
Cuba (Caribbean) ––– Married / In–Union
CP (any)
CP (modern)
CP (traditional)
Unmet need
Total demand
Demand satisfied (any method)
Demand satisfied (modern methods)
100%
80%
60%
40%
20%
0%
1970 1975 1980 1985 1990 1995 2000 2005 2010 2015 2020 2025 2030
DHS
MICS
Other international survey
National survey
PMA
Subpopulation
+: Higher contraceptive use
−: Lower contraceptive use
A: Other age group
F: Folk methods included
S−: Sterilization included
S+: Sterilization excluded
Married women
Sexually active women
Ever married/All women
Both sexes and husband/wives

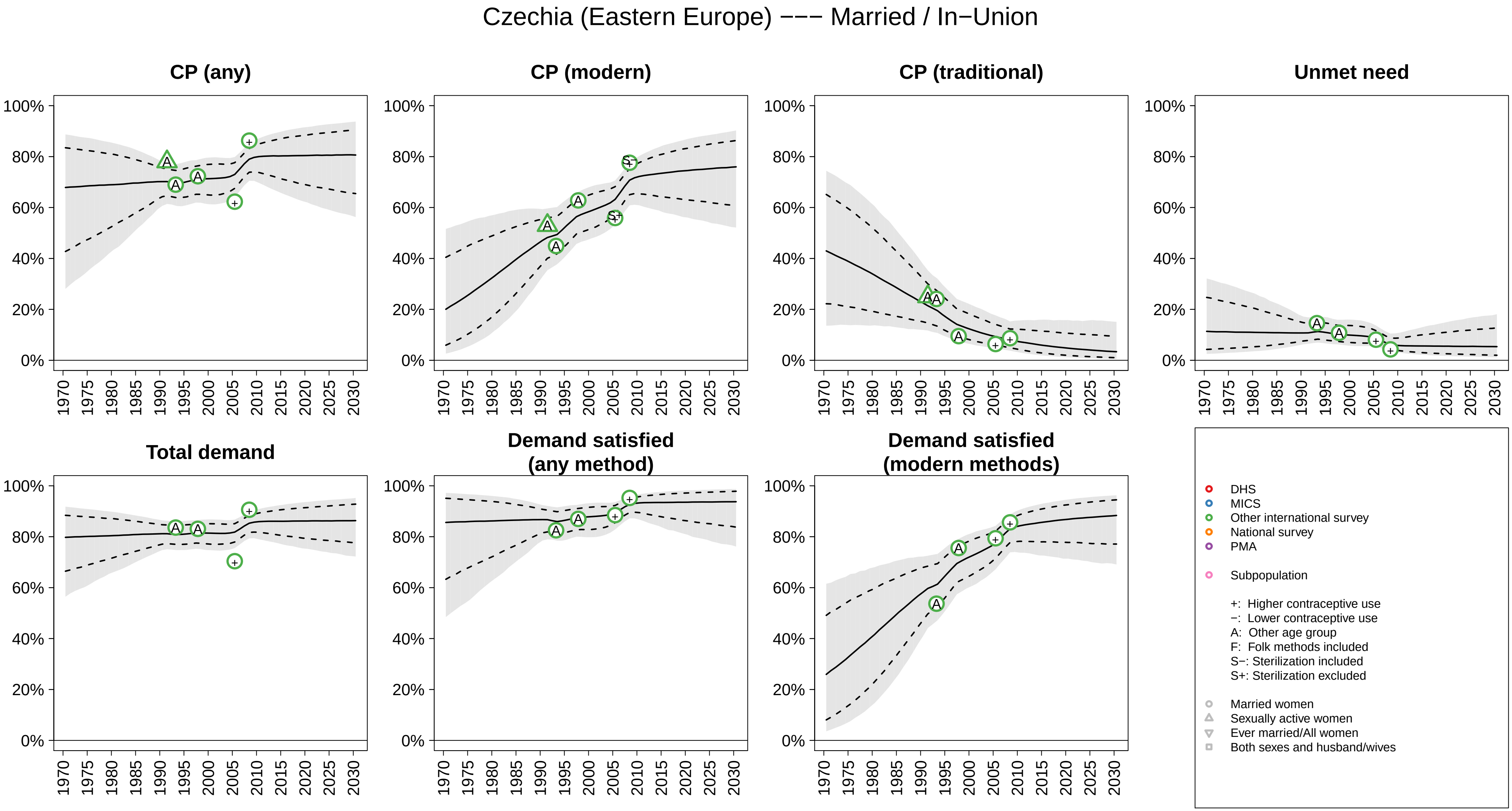

Czechia (Eastern Europe) −−− Married / In−Union
CP (any)
CP (modern)
CP (traditional)
Unmet need
Total demand
Demand satisfied (any method)
Demand satisfied (modern methods)
100%
80%
60%
40%
20%
0%
1970
1975
1980
1985
1990
1995
2000
2005
2010
2015
2020
2025
2030
DHS
MICS
Other international survey
National survey
PMA
Subpopulation
+: Higher contraceptive use
−: Lower contraceptive use
A: Other age group
F: Folk methods included
S−: Sterilization included
S+: Sterilization excluded
Married women
Sexually active women
Ever married/All women
Both sexes and husband/wives

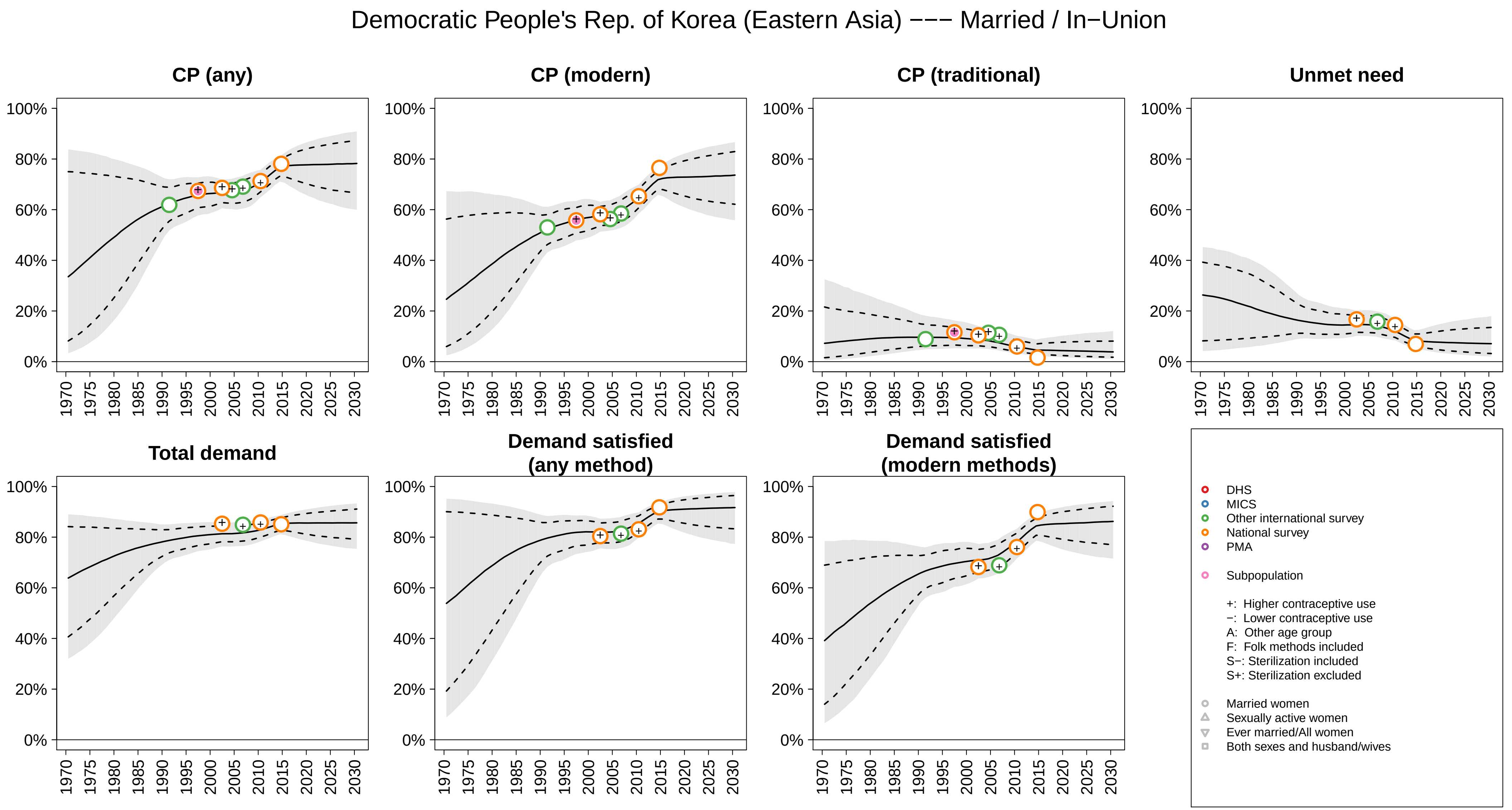

Democratic People's Rep. of Korea (Eastern Asia) −−− Married / In−Union
CP (any)
CP (modern)
CP (traditional)
Unmet need
Total demand
Demand satisfied (any method)
Demand satisfied (modern methods)
100%
80%
60%
40%
20%
0%
1970 1975 1980 1985 1990 1995 2000 2005 2010 2015 2020 2025 2030
DHS
MICS
Other international survey
National survey
PMA
Subpopulation
+: Higher contraceptive use
−: Lower contraceptive use
A: Other age group
F: Folk methods included
S−: Sterilization included
S+: Sterilization excluded
Married women
Sexually active women
Ever married/All women
Both sexes and husband/wives

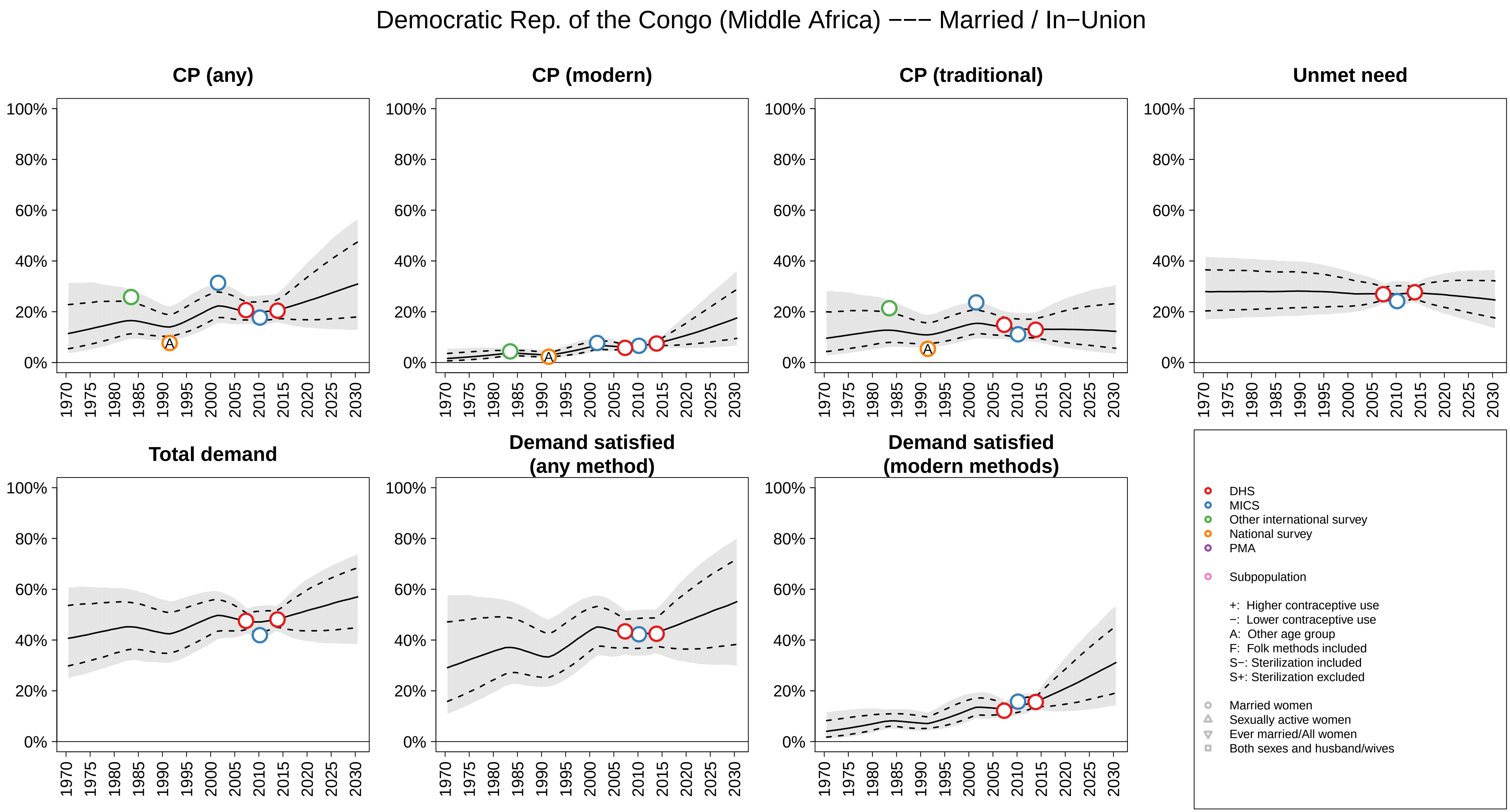
Democratic Rep. of the Congo (Middle Africa) −−− Married / In−Union
CP (any)
CP (modern)
CP (traditional)
Unmet need
Total demand
Demand satisfied (any method)
Demand satisfied (modern methods)
0%
20%
40%
60%
80%
100%
1970
1975
1980
1985
1990
1995
2000
2005
2010
2015
2020
2025
2030
A
DHS
MICS
Other international survey
National survey
PMA
Subpopulation
+: Higher contraceptive use
−: Lower contraceptive use
A: Other age group
F: Folk methods included
S−: Sterilization included
S+: Sterilization excluded
Married women
Sexually active women
Ever married/All women
Both sexes and husband/wives

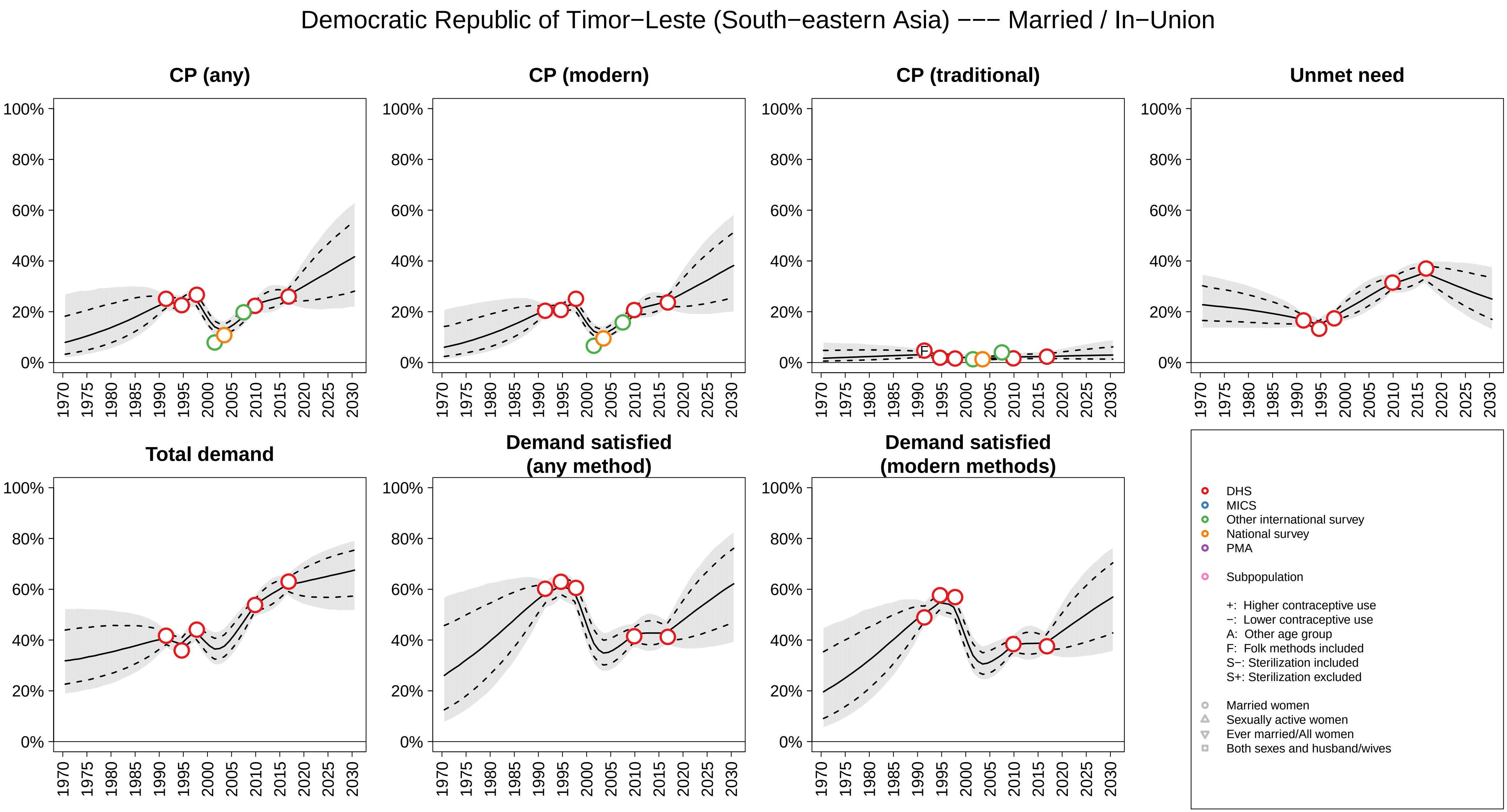
Democratic Republic of Timor−Leste (South−eastern Asia) −−− Married / In−Union
CP (any)
CP (modern)
CP (traditional)
Unmet need
Total demand
Demand satisfied (any method)
Demand satisfied (modern methods)
100%
80%
60%
40%
20%
0%
1970
1975
1980
1985
1990
1995
2000
2005
2010
2015
2020
2025
2030
F
DHS
MICS
Other international survey
National survey
PMA
Subpopulation
+: Higher contraceptive use
−: Lower contraceptive use
A: Other age group
F: Folk methods included
S−: Sterilization included
S+: Sterilization excluded
Married women
Sexually active women
Ever married/All women
Both sexes and husband/wives

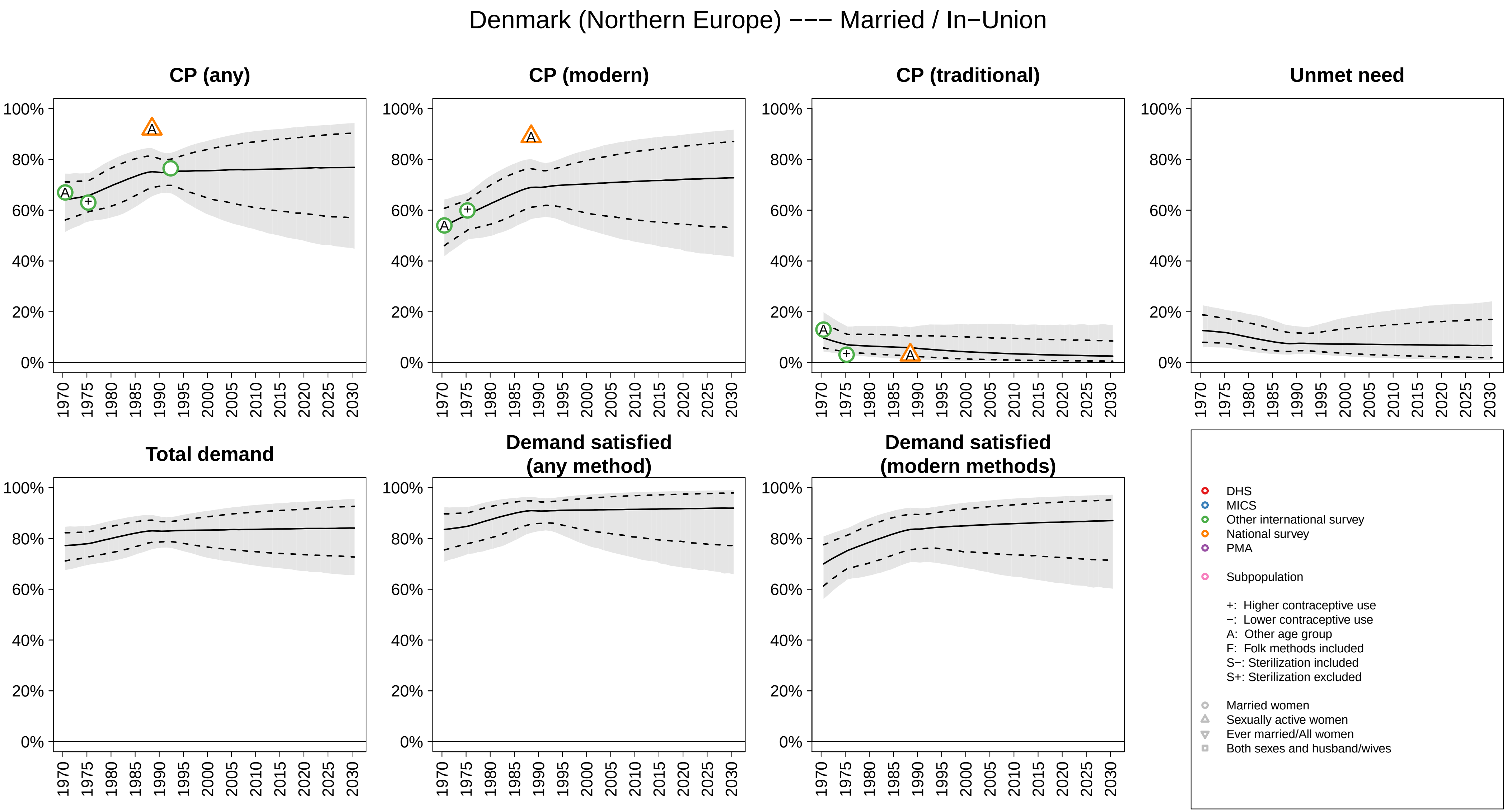
Denmark (Northern Europe) --- Married / In−Union
CP (any)
CP (modern)
CP (traditional)
Unmet need
Total demand
Demand satisfied (any method)
Demand satisfied (modern methods)
DHS
MICS
Other international survey
National survey
PMA
Subpopulation
+: Higher contraceptive use
−: Lower contraceptive use
A: Other age group
F: Folk methods included
S−: Sterilization included
S+: Sterilization excluded
Married women
Sexually active women
Ever married/All women
Both sexes and husband/wives

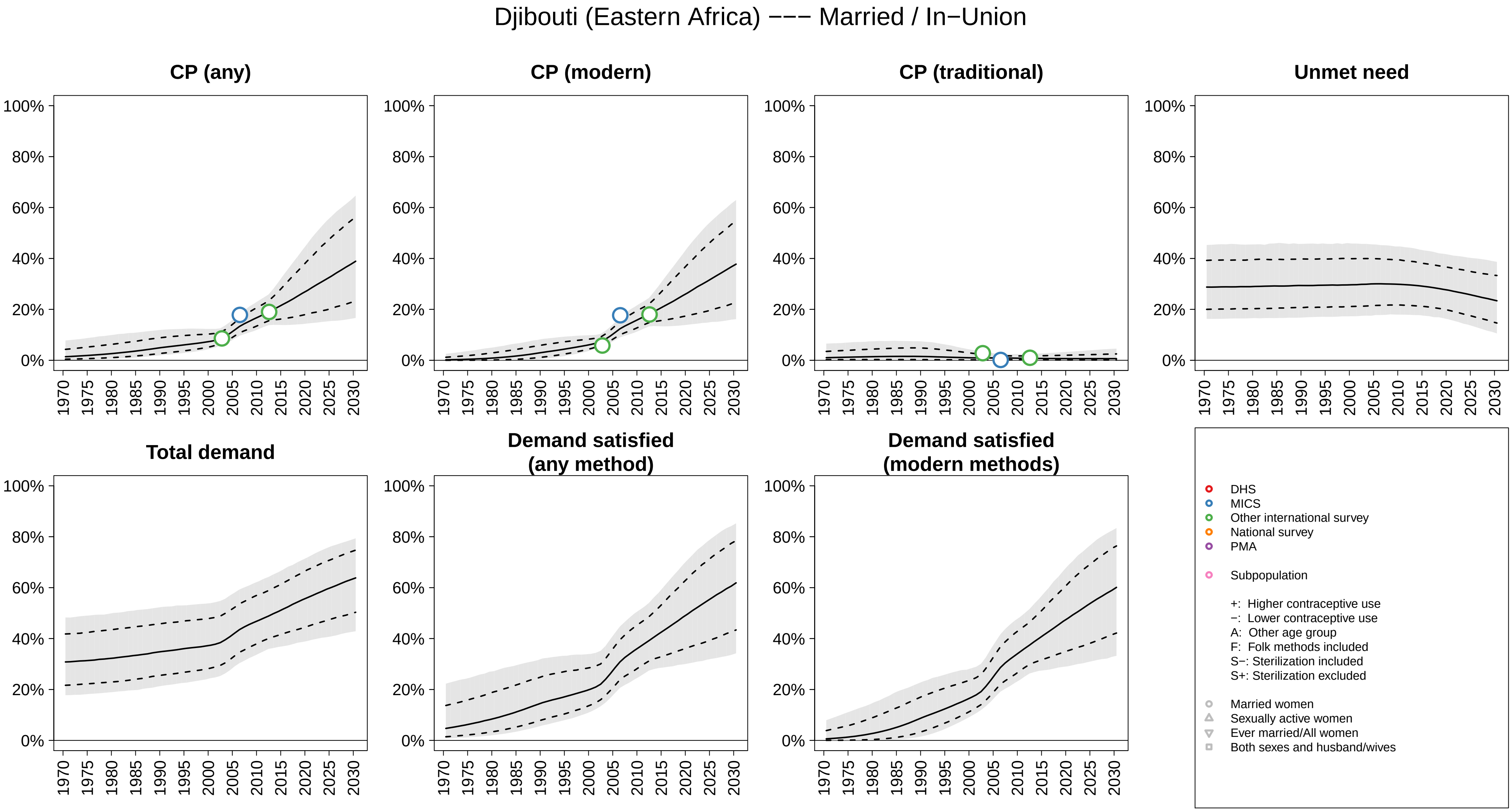

Djibouti (Eastern Africa) --- Married / In-Union
CP (any)
CP (modern)
CP (traditional)
Unmet need
Total demand
Demand satisfied (any method)
Demand satisfied (modern methods)
100%
80%
60%
40%
20%
0%
1970 1975 1980 1985 1990 1995 2000 2005 2010 2015 2020 2025 2030
DHS
MICS
Other international survey
National survey
PMA
Subpopulation
+: Higher contraceptive use
−: Lower contraceptive use
A: Other age group
F: Folk methods included
S−: Sterilization included
S+: Sterilization excluded
Married women
Sexually active women
Ever married/All women
Both sexes and husband/wives

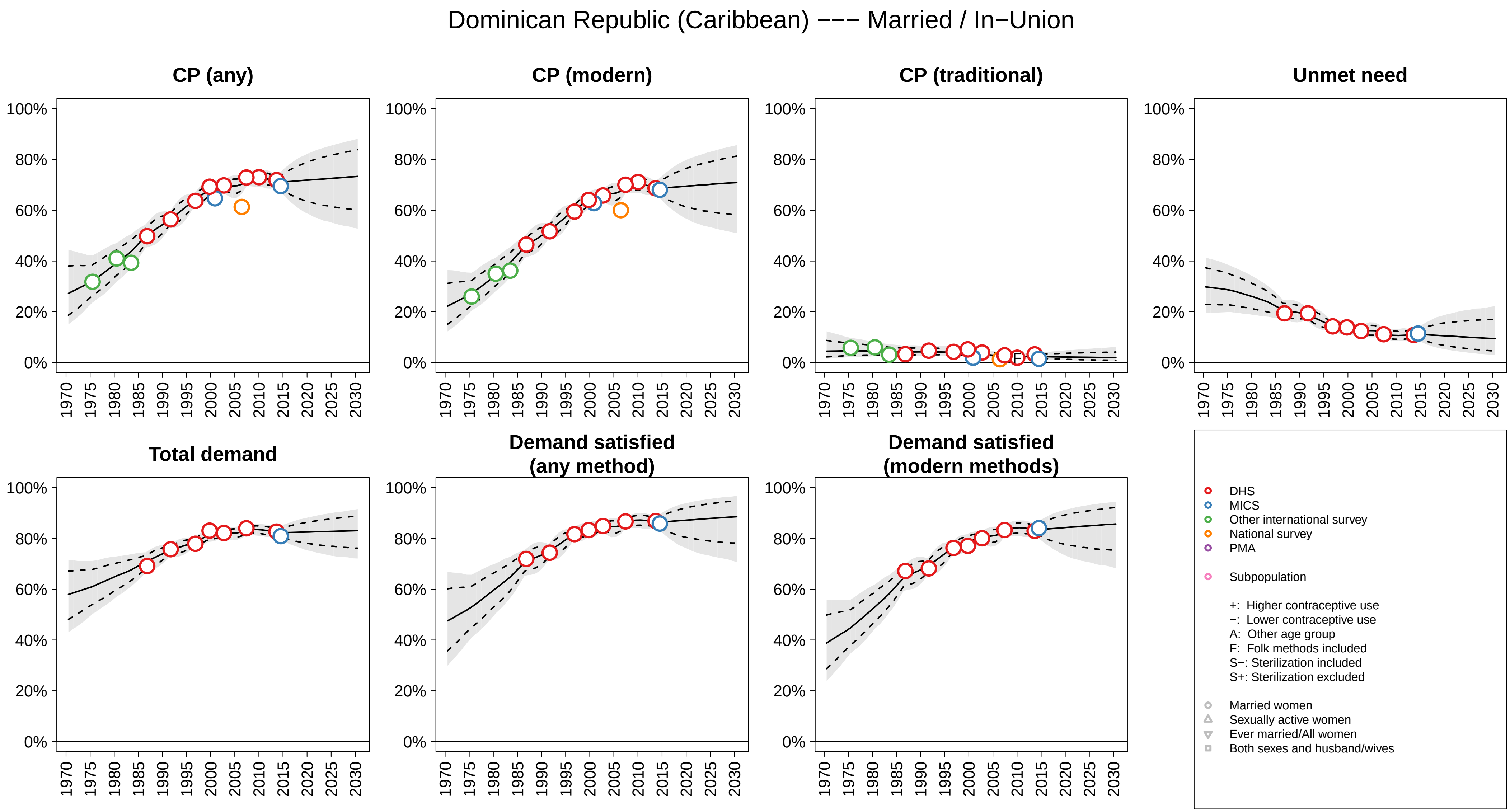
Dominican Republic (Caribbean) −−− Married / In−Union
CP (any)
CP (modern)
CP (traditional)
Unmet need
Total demand
Demand satisfied (any method)
Demand satisfied (modern methods)
0%
20%
40%
60%
80%
100%
1970
1975
1980
1985
1990
1995
2000
2005
2010
2015
2020
2025
2030
F
DHS
MICS
Other international survey
National survey
PMA
Subpopulation
+: Higher contraceptive use
−: Lower contraceptive use
A: Other age group
F: Folk methods included
S−: Sterilization included
S+: Sterilization excluded
Married women
Sexually active women
Ever married/All women
Both sexes and husband/wives

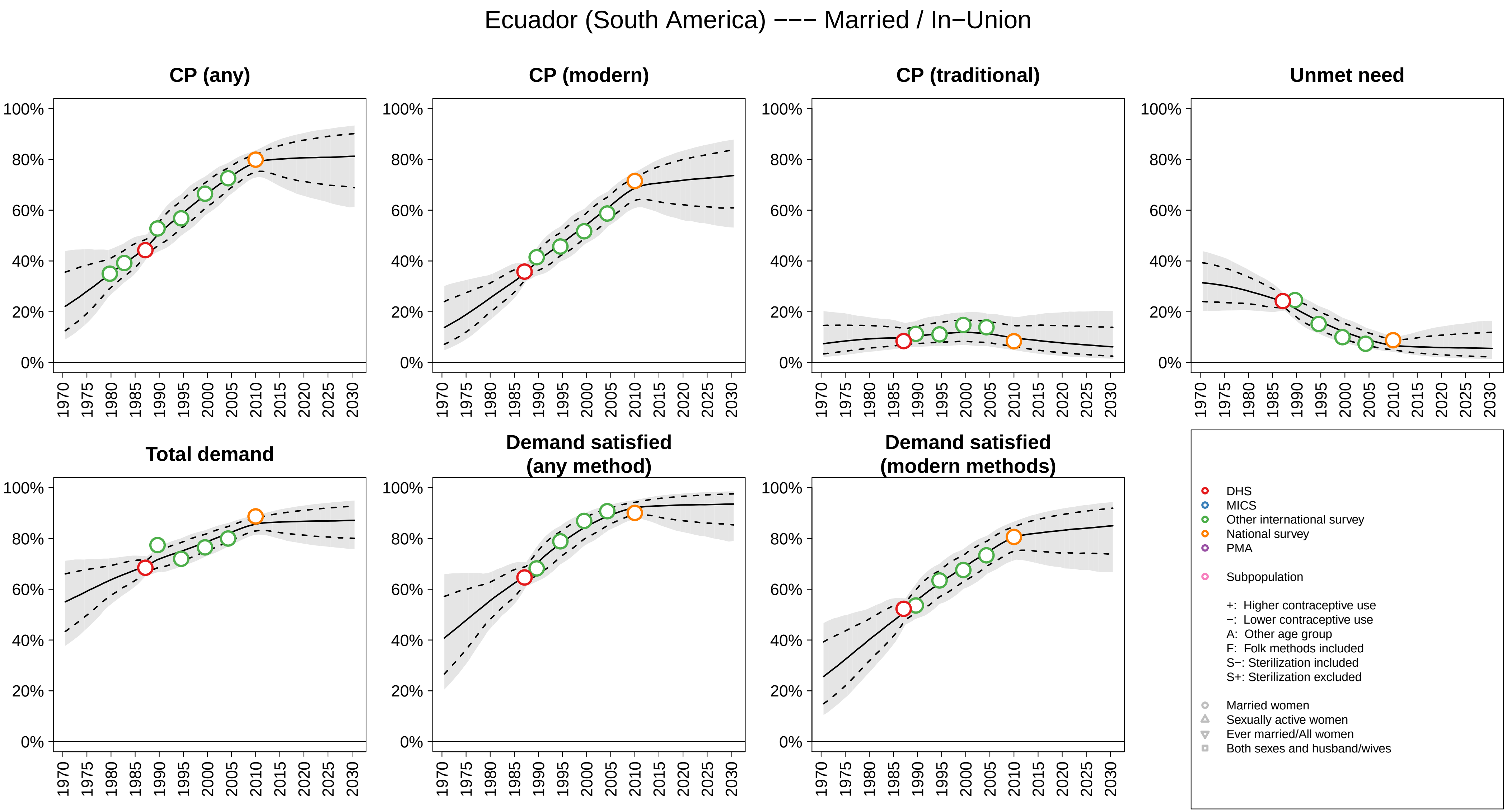
Ecuador (South America) --- Married / In−Union
CP (any)
CP (modern)
CP (traditional)
Unmet need
Total demand
Demand satisfied (any method)
Demand satisfied (modern methods)
0%
20%
40%
60%
80%
100%
1970
1975
1980
1985
1990
1995
2000
2005
2010
2015
2020
2025
2030
DHS
MICS
Other international survey
National survey
PMA
Subpopulation
+: Higher contraceptive use
−: Lower contraceptive use
A: Other age group
F: Folk methods included
S−: Sterilization included
S+: Sterilization excluded
Married women
Sexually active women
Ever married/All women
Both sexes and husband/wives

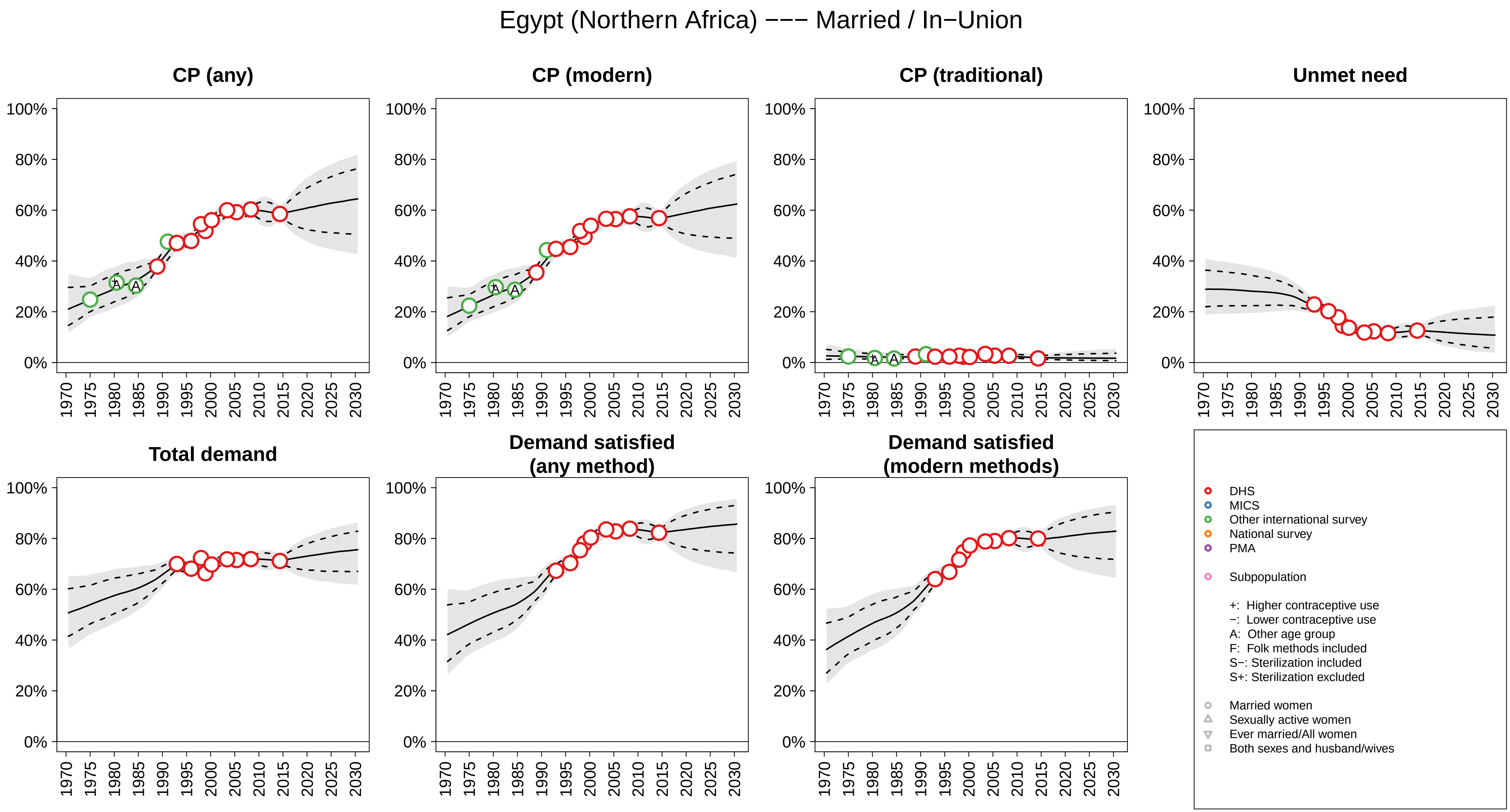
Egypt (Northern Africa) --- Married / In−Union
CP (any)
CP (modern)
CP (traditional)
Unmet need
Total demand
Demand satisfied (any method)
Demand satisfied (modern methods)
0%
20%
40%
60%
80%
100%
1970
1975
1980
1985
1990
1995
2000
2005
2010
2015
2020
2025
2030
DHS
MICS
Other international survey
National survey
PMA
Subpopulation
+: Higher contraceptive use
−: Lower contraceptive use
A: Other age group
F: Folk methods included
S−: Sterilization included
S+: Sterilization excluded
Married women
Sexually active women
Ever married/All women
Both sexes and husband/wives

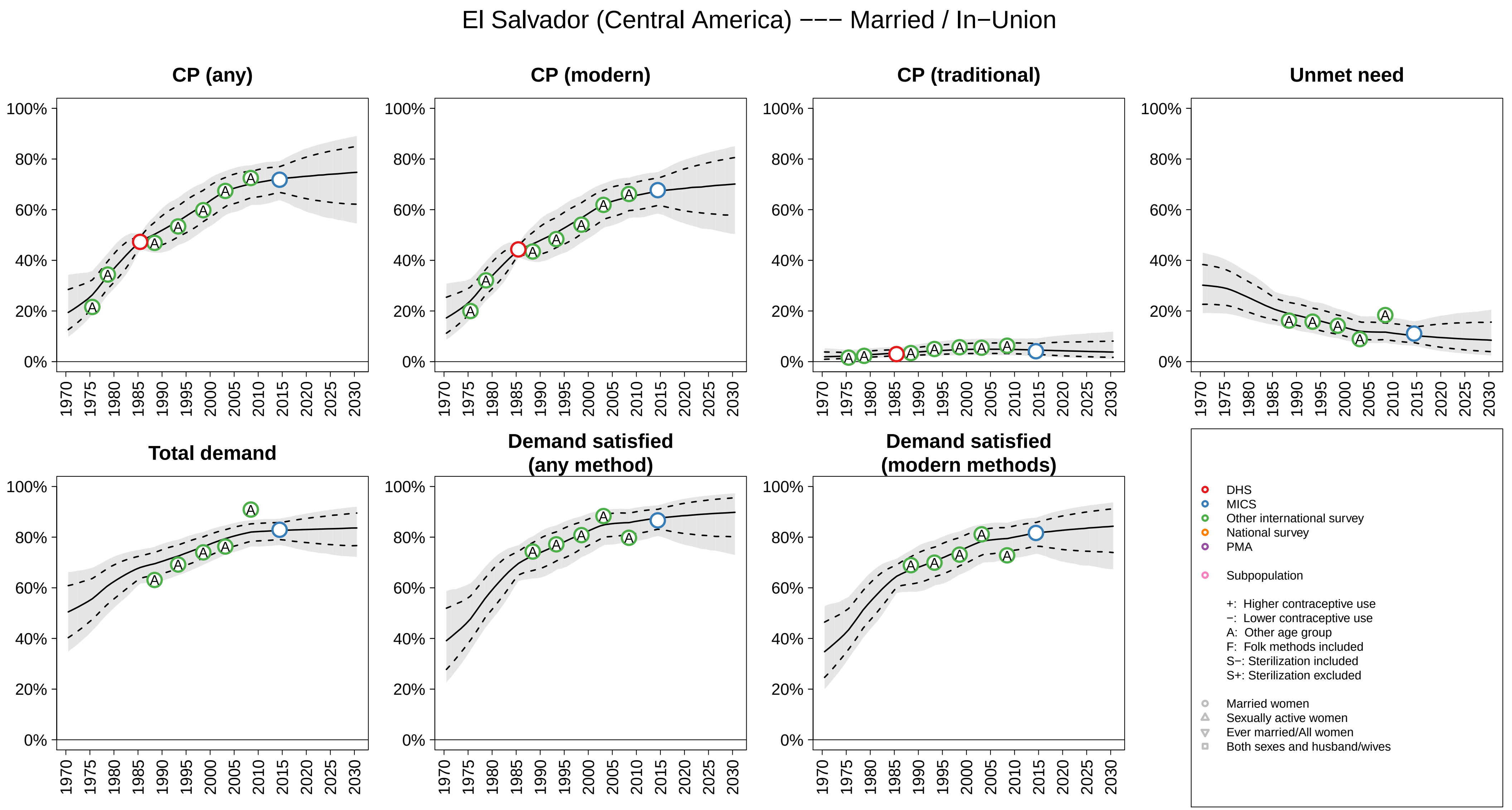
El Salvador (Central America) −−− Married / In−Union
CP (any)
CP (modern)
CP (traditional)
Unmet need
Total demand
Demand satisfied (any method)
Demand satisfied (modern methods)
100%
80%
60%
40%
20%
0%
1970 1975 1980 1985 1990 1995 2000 2005 2010 2015 2020 2025 2030
DHS
MICS
Other international survey
National survey
PMA
Subpopulation
+: Higher contraceptive use
−: Lower contraceptive use
A: Other age group
F: Folk methods included
S−: Sterilization included
S+: Sterilization excluded
Married women
Sexually active women
Ever married/All women
Both sexes and husband/wives

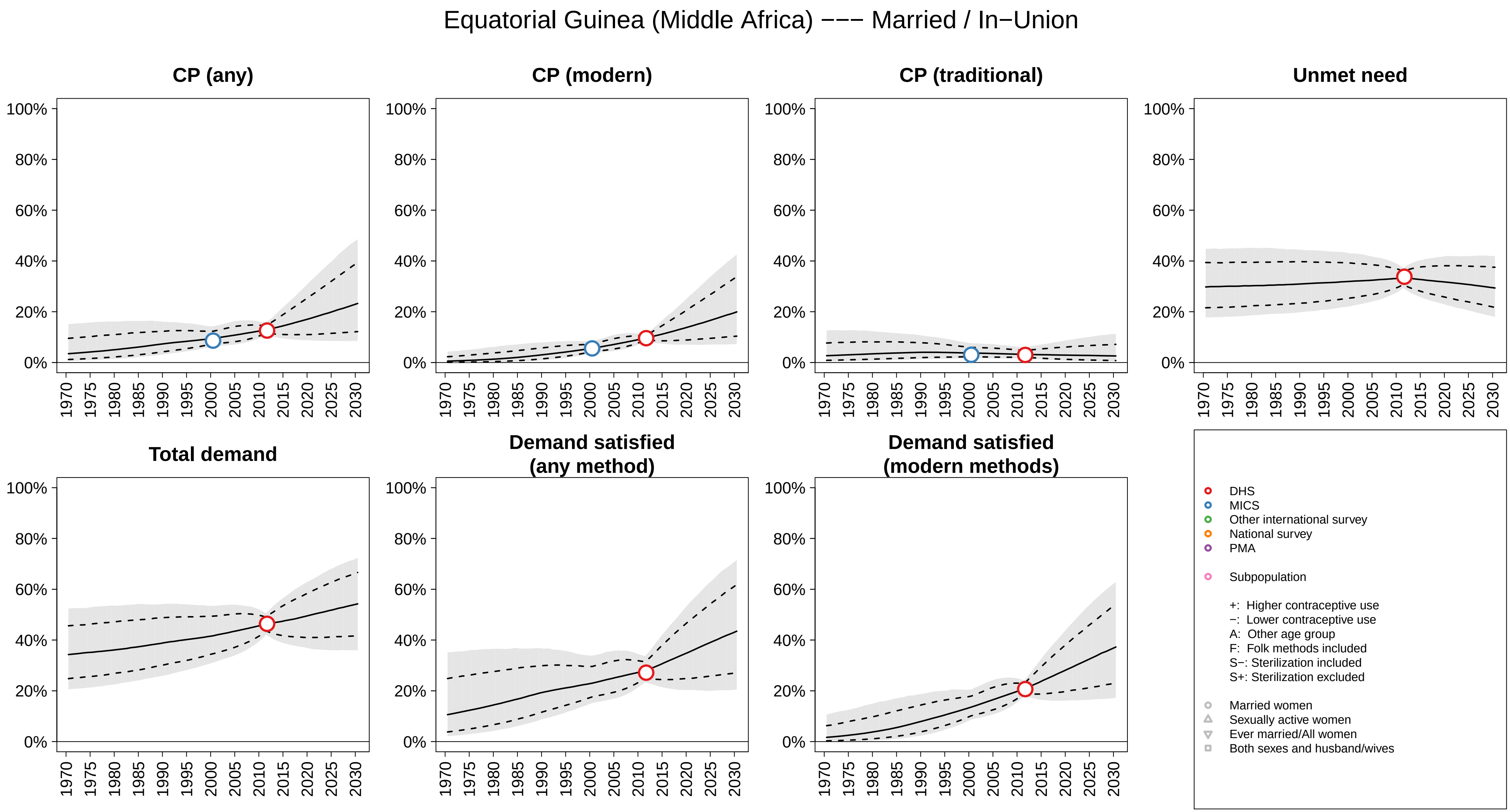
Equatorial Guinea (Middle Africa) −−− Married / In−Union
CP (any)
CP (modern)
CP (traditional)
Unmet need
Total demand
Demand satisfied (any method)
Demand satisfied (modern methods)
100%
80%
60%
40%
20%
0%
1970 1975 1980 1985 1990 1995 2000 2005 2010 2015 2020 2025 2030
DHS
MICS
Other international survey
National survey
PMA
Subpopulation
+: Higher contraceptive use
−: Lower contraceptive use
A: Other age group
F: Folk methods included
S−: Sterilization included
S+: Sterilization excluded
Married women
Sexually active women
Ever married/All women
Both sexes and husband/wives

Eritrea (Eastern Africa) ––– Married / In−Union

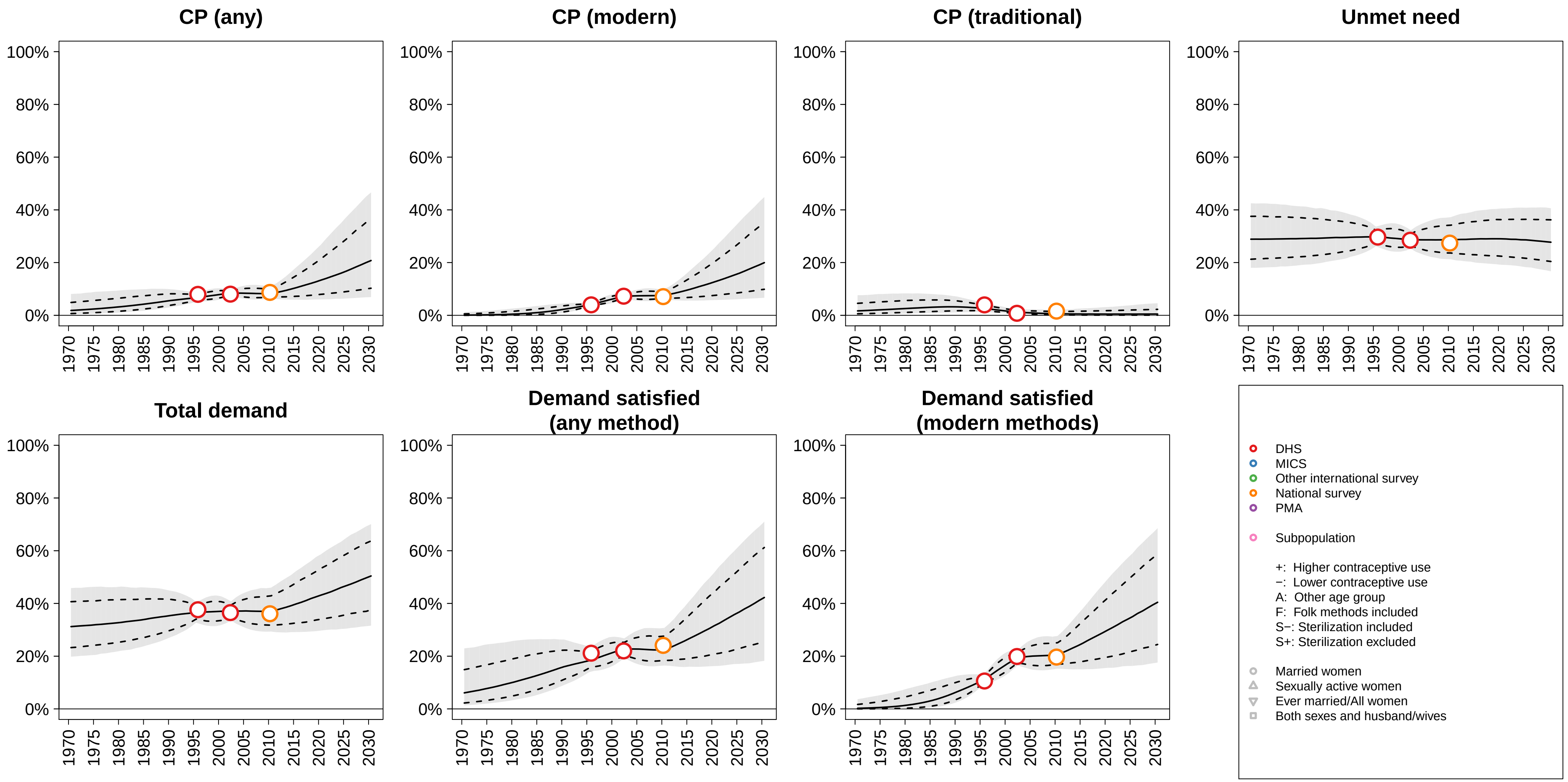

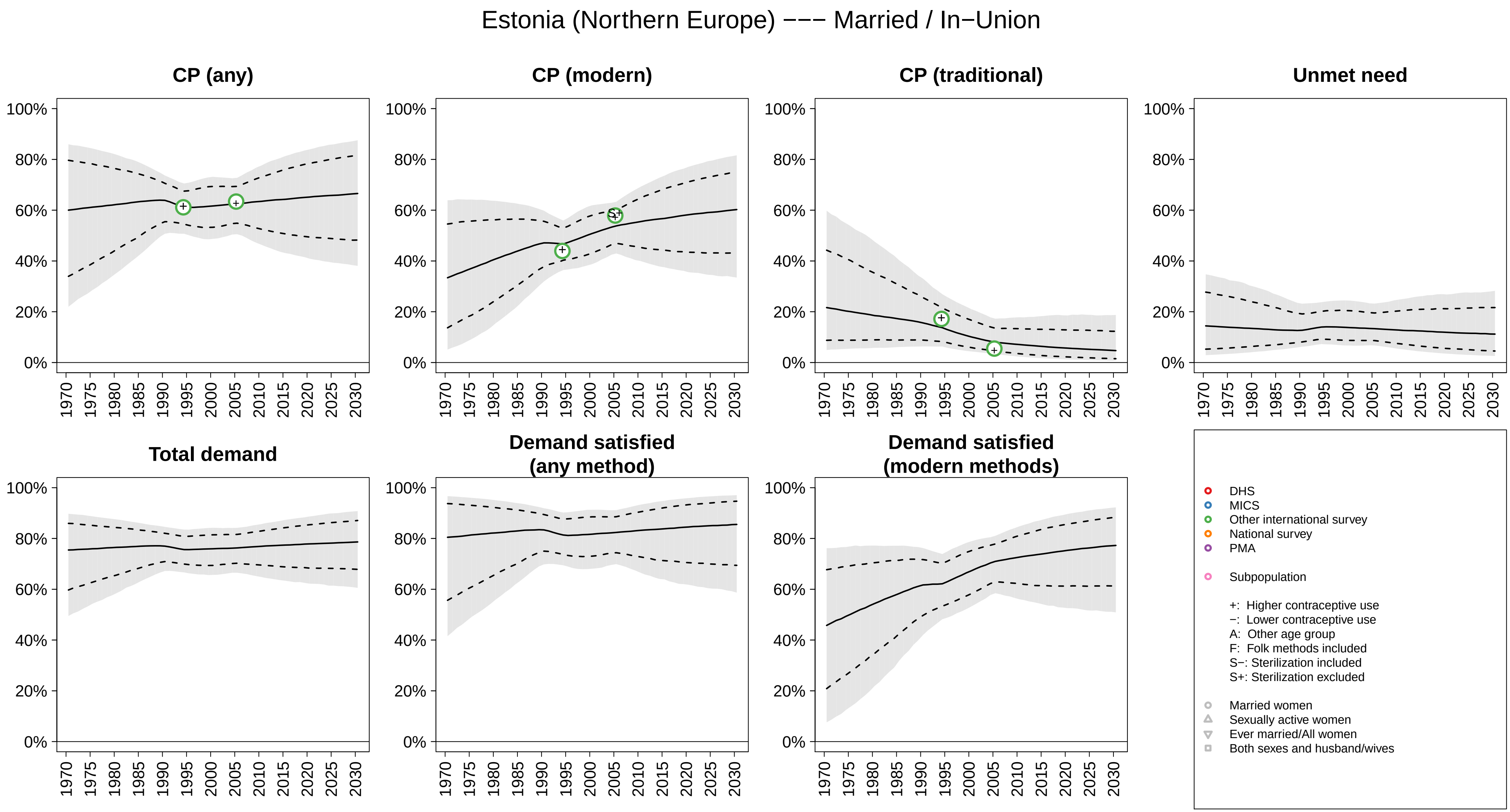

Estonia (Northern Europe) −−− Married / In−Union
CP (any)
CP (modern)
CP (traditional)
Unmet need
Total demand
Demand satisfied (any method)
Demand satisfied (modern methods)
100%
80%
60%
40%
20%
0%
1970 1975 1980 1985 1990 1995 2000 2005 2010 2015 2020 2025 2030
DHS
MICS
Other international survey
National survey
PMA
Subpopulation
+: Higher contraceptive use
−: Lower contraceptive use
A: Other age group
F: Folk methods included
S−: Sterilization included
S+: Sterilization excluded
Married women
Sexually active women
Ever married/All women
Both sexes and husband/wives

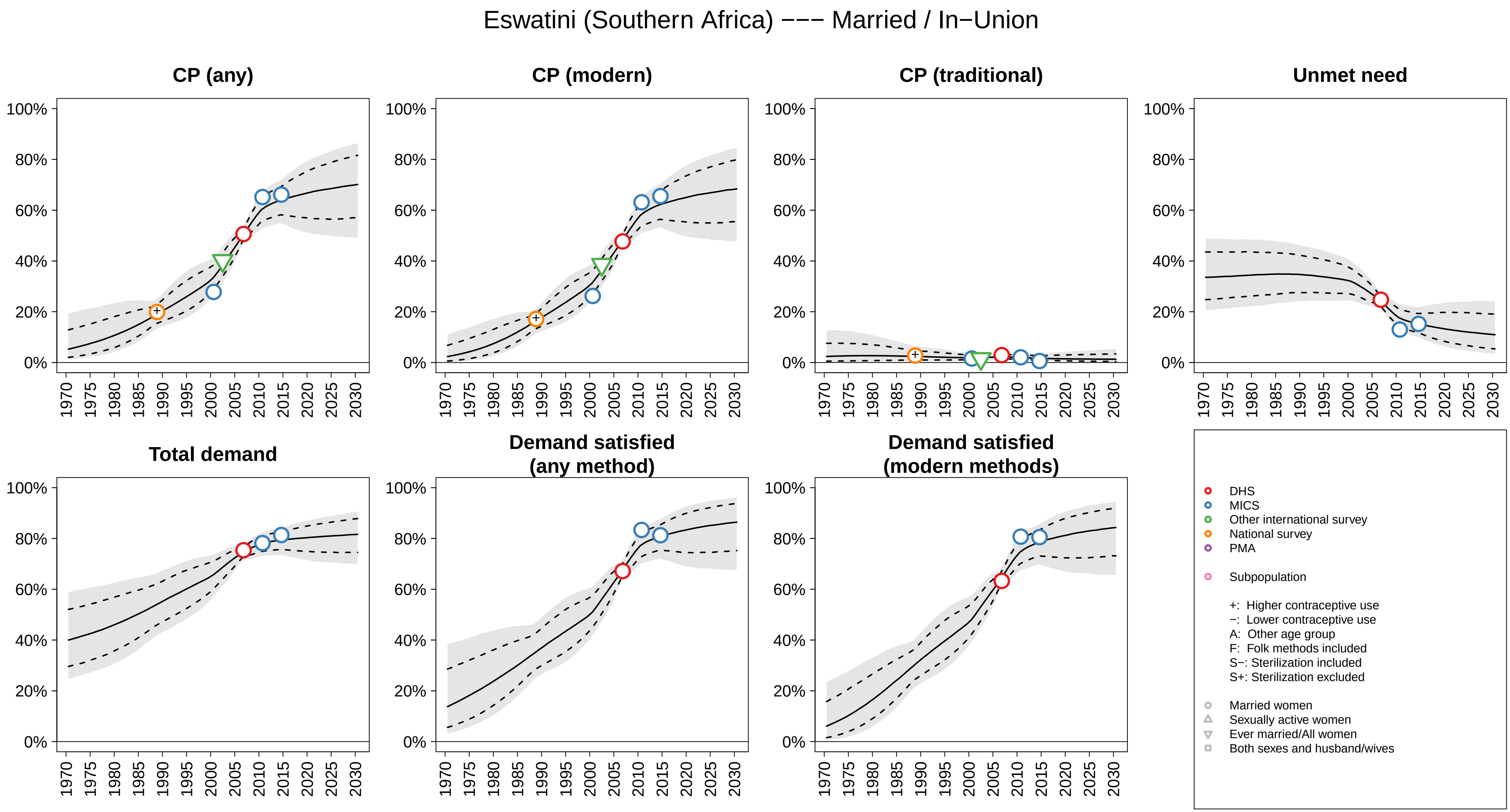
Eswatini (Southern Africa) −−− Married / In−Union
CP (any)
CP (modern)
CP (traditional)
Unmet need
Total demand
Demand satisfied (any method)
Demand satisfied (modern methods)
0%
20%
40%
60%
80%
100%
1970
1975
1980
1985
1990
1995
2000
2005
2010
2015
2020
2025
2030
DHS
MICS
Other international survey
National survey
PMA
Subpopulation
+: Higher contraceptive use
−: Lower contraceptive use
A: Other age group
F: Folk methods included
S−: Sterilization included
S+: Sterilization excluded
Married women
Sexually active women
Ever married/All women
Both sexes and husband/wives

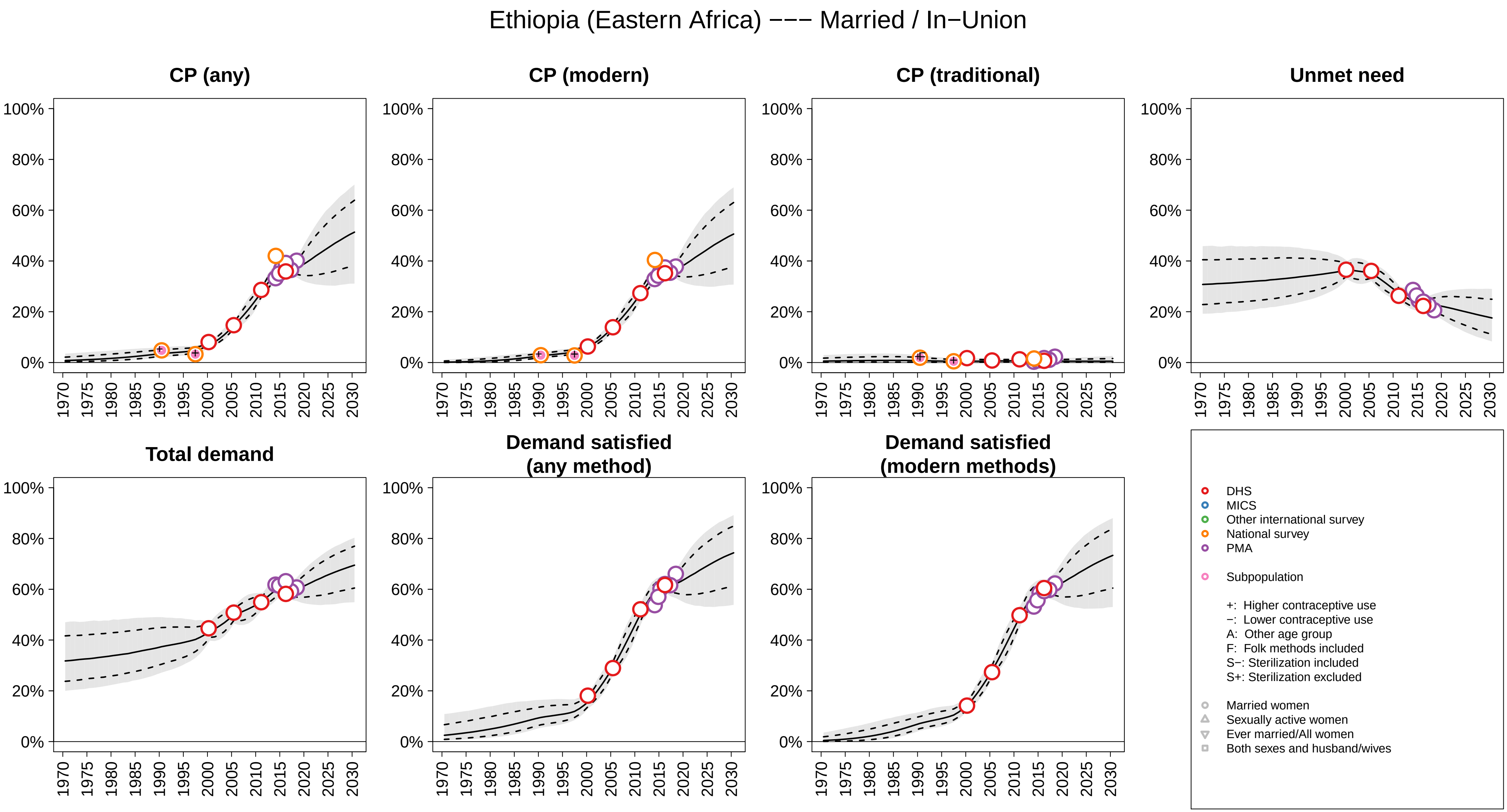
Ethiopia (Eastern Africa) --- Married / In−Union
CP (any)
CP (modern)
CP (traditional)
Unmet need
Total demand
Demand satisfied (any method)
Demand satisfied (modern methods)
100%
80%
60%
40%
20%
0%
1970 1975 1980 1985 1990 1995 2000 2005 2010 2015 2020 2025 2030
DHS
MICS
Other international survey
National survey
PMA
Subpopulation
+: Higher contraceptive use
−: Lower contraceptive use
A: Other age group
F: Folk methods included
S−: Sterilization included
S+: Sterilization excluded
Married women
Sexually active women
Ever married/All women
Both sexes and husband/wives

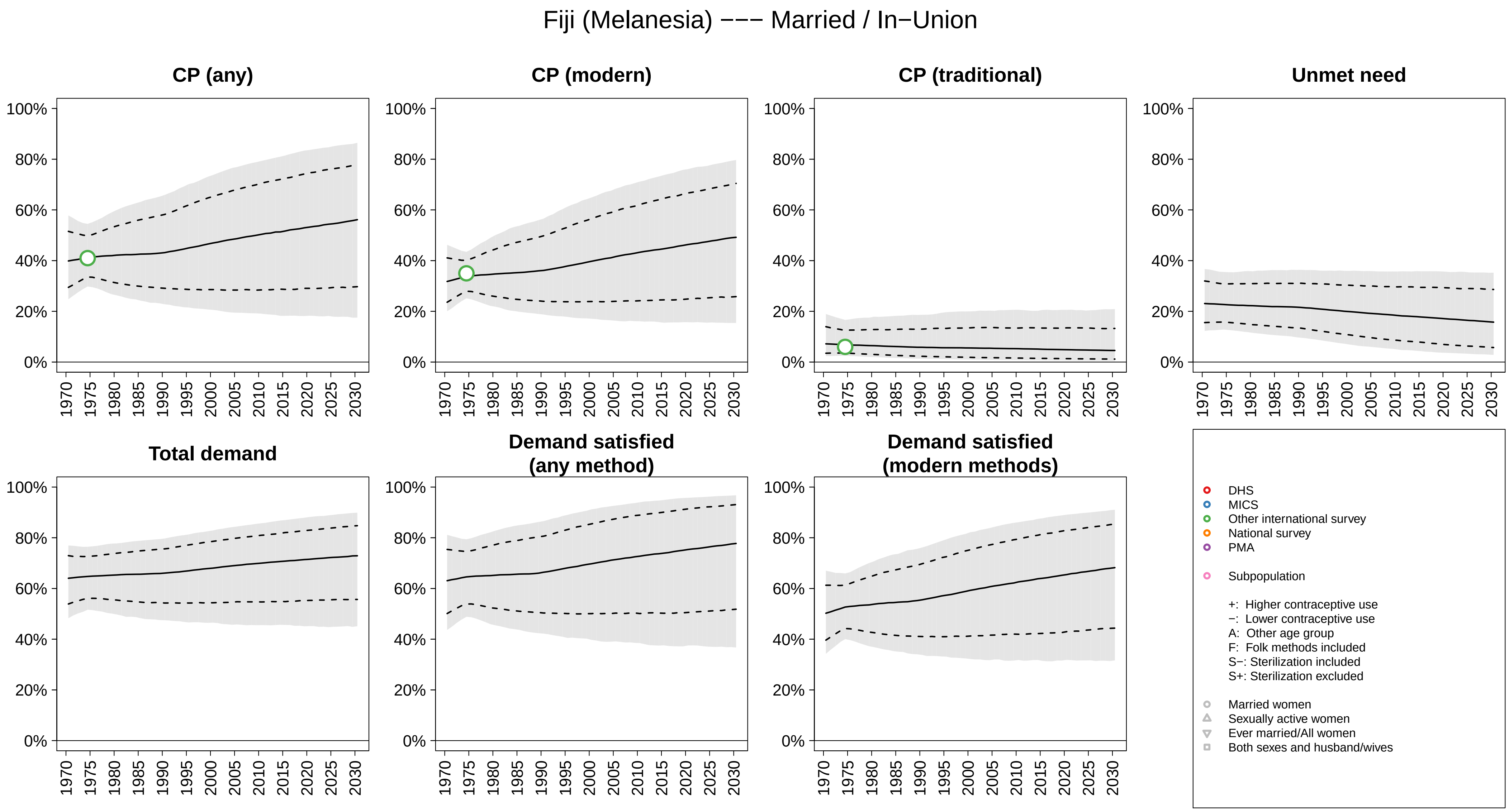
Fiji (Melanesia) −−− Married / In−Union
CP (any)
CP (modern)
CP (traditional)
Unmet need
Total demand
Demand satisfied (any method)
Demand satisfied (modern methods)
0%
20%
40%
60%
80%
100%
1970
1975
1980
1985
1990
1995
2000
2005
2010
2015
2020
2025
2030
DHS
MICS
Other international survey
National survey
PMA
Subpopulation
+: Higher contraceptive use
−: Lower contraceptive use
A: Other age group
F: Folk methods included
S−: Sterilization included
S+: Sterilization excluded
Married women
Sexually active women
Ever married/All women
Both sexes and husband/wives

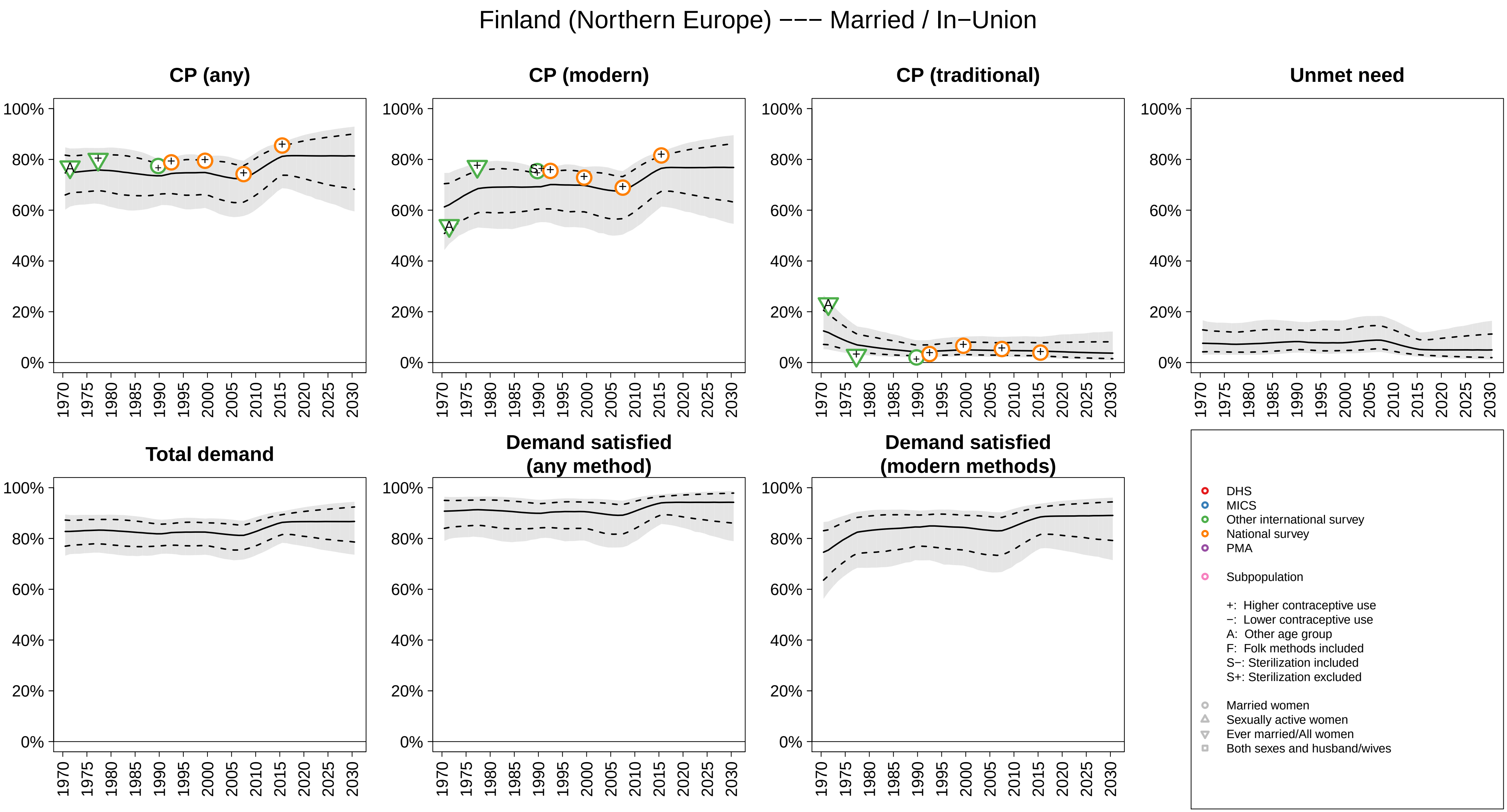

Finland (Northern Europe) --- Married / In−Union
CP (any)
CP (modern)
CP (traditional)
Unmet need
Total demand
Demand satisfied (any method)
Demand satisfied (modern methods)
100%
80%
60%
40%
20%
0%
1970 1975 1980 1985 1990 1995 2000 2005 2010 2015 2020 2025 2030
DHS
MICS
Other international survey
National survey
PMA
Subpopulation
+: Higher contraceptive use
−: Lower contraceptive use
A: Other age group
F: Folk methods included
S−: Sterilization included
S+: Sterilization excluded
Married women
Sexually active women
Ever married/All women
Both sexes and husband/wives

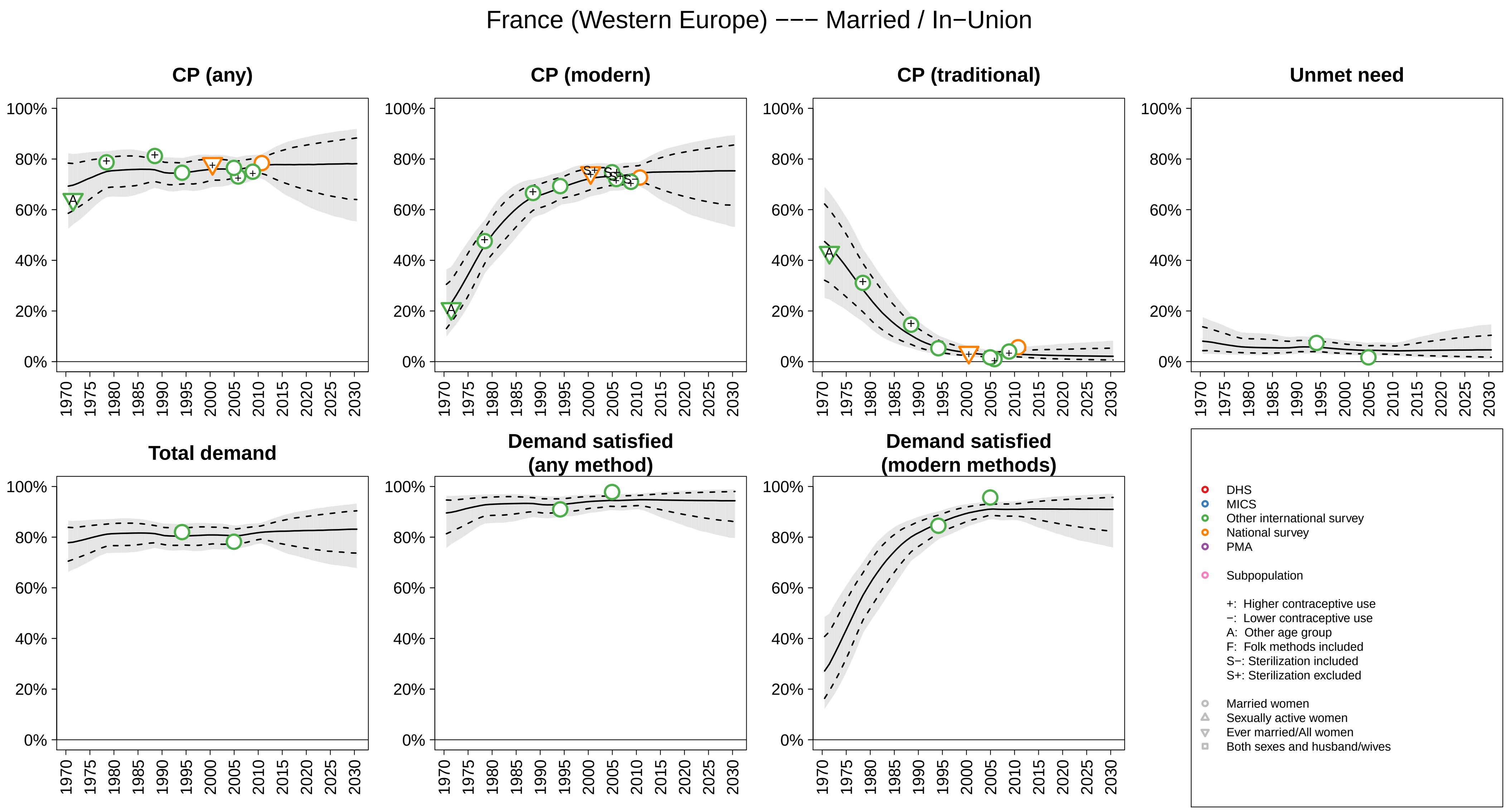
France (Western Europe) −−− Married / In−Union
CP (any)
CP (modern)
CP (traditional)
Unmet need
Total demand
Demand satisfied (any method)
Demand satisfied (modern methods)
0%
20%
40%
60%
80%
100%
1970
1975
1980
1985
1990
1995
2000
2005
2010
2015
2020
2025
2030
DHS
MICS
Other international survey
National survey
PMA
Subpopulation
+: Higher contraceptive use
−: Lower contraceptive use
A: Other age group
F: Folk methods included
S−: Sterilization included
S+: Sterilization excluded
Married women
Sexually active women
Ever married/All women
Both sexes and husband/wives

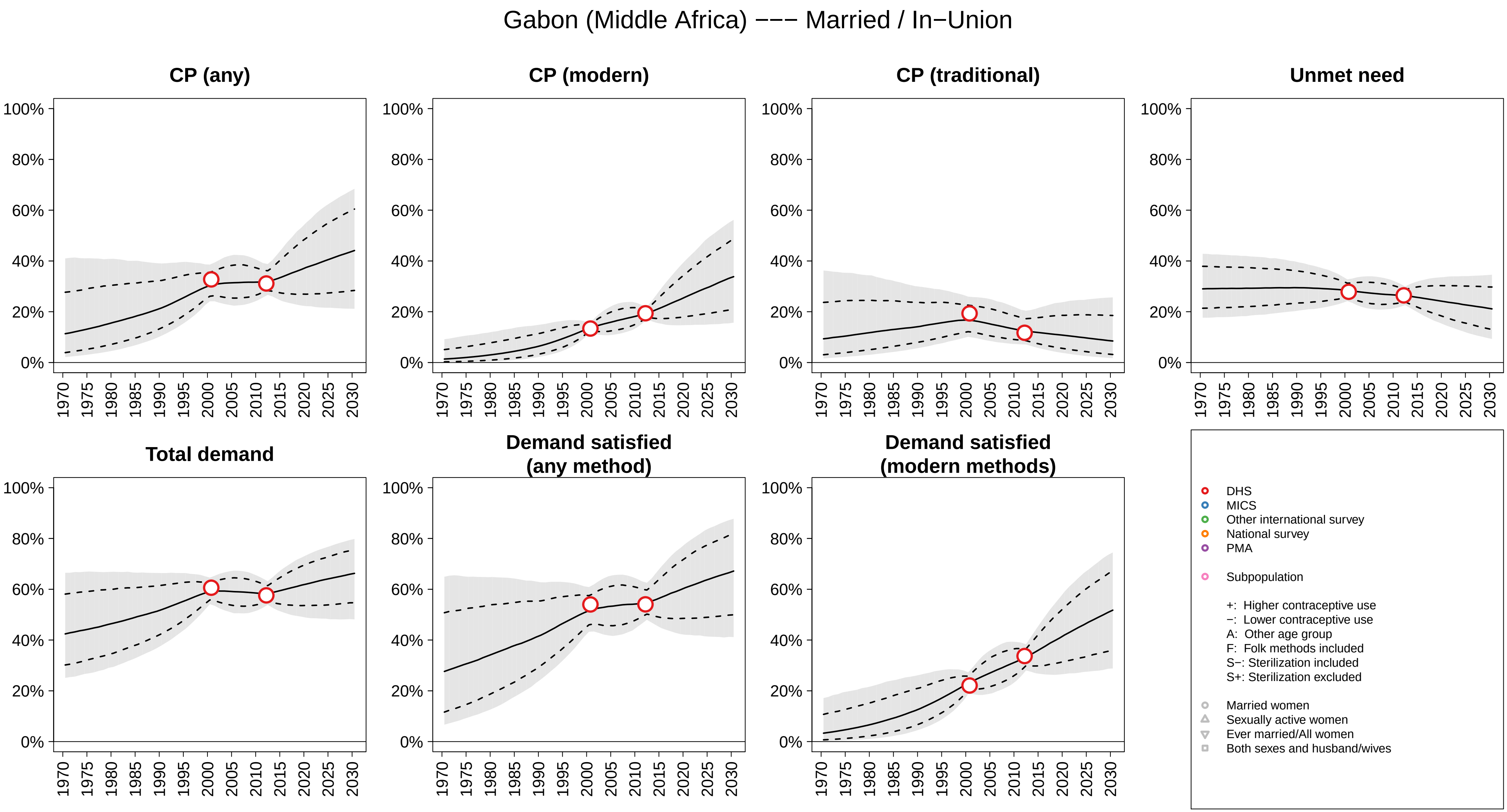

Gabon (Middle Africa) −−− Married / In−Union
CP (any)
CP (modern)
CP (traditional)
Unmet need
Total demand
Demand satisfied (any method)
Demand satisfied (modern methods)
0%
20%
40%
60%
80%
100%
1970
1975
1980
1985
1990
1995
2000
2005
2010
2015
2020
2025
2030
DHS
MICS
Other international survey
National survey
PMA
Subpopulation
+: Higher contraceptive use
−: Lower contraceptive use
A: Other age group
F: Folk methods included
S−: Sterilization included
S+: Sterilization excluded
Married women
Sexually active women
Ever married/All women
Both sexes and husband/wives

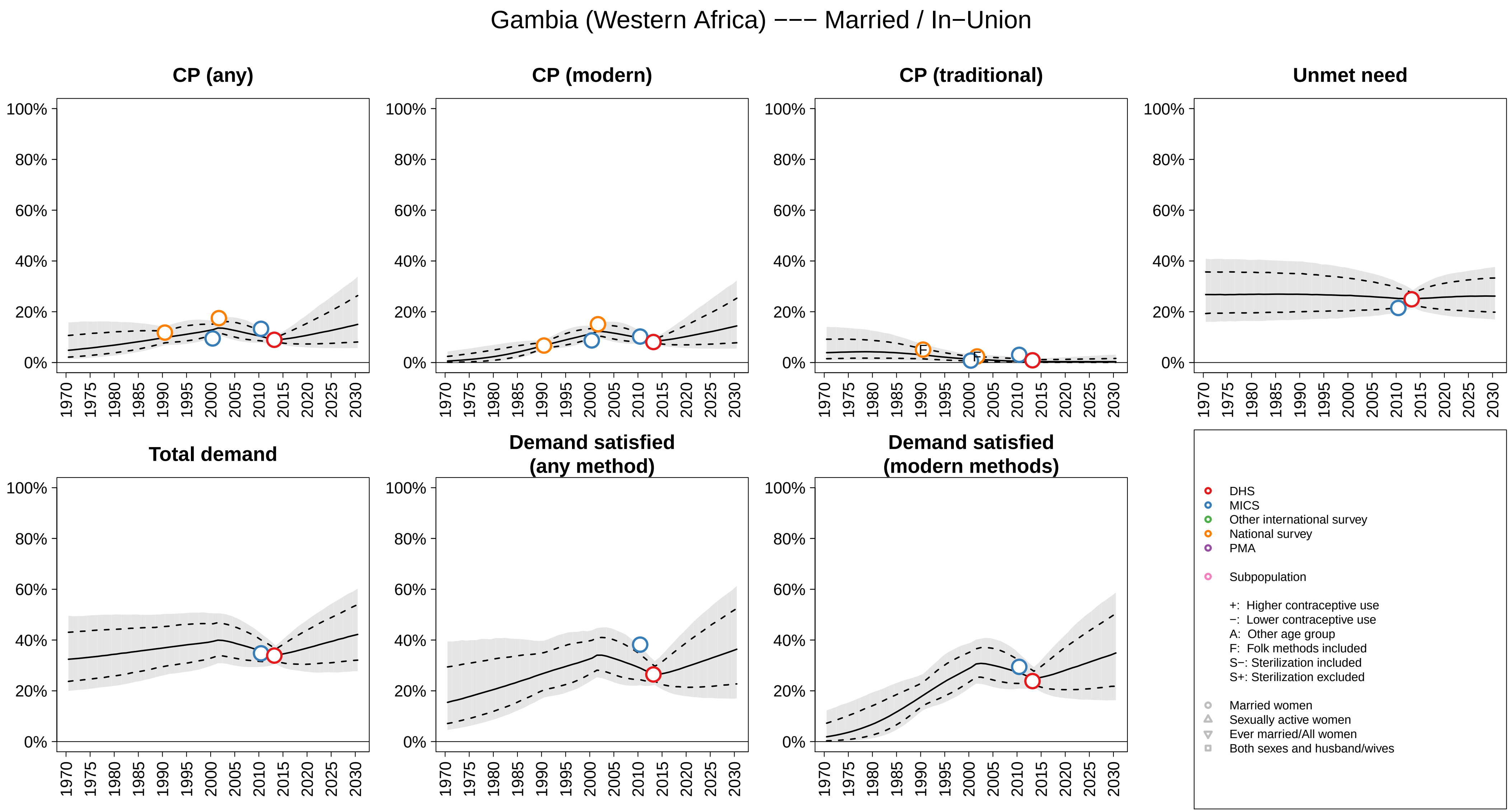
Gambia (Western Africa) --- Married / In−Union
CP (any)
CP (modern)
CP (traditional)
Unmet need
Total demand
Demand satisfied (any method)
Demand satisfied (modern methods)
100%
80%
60%
40%
20%
0%
1970 1975 1980 1985 1990 1995 2000 2005 2010 2015 2020 2025 2030
F
DHS
MICS
Other international survey
National survey
PMA
Subpopulation
+: Higher contraceptive use
−: Lower contraceptive use
A: Other age group
F: Folk methods included
S−: Sterilization included
S+: Sterilization excluded
Married women
Sexually active women
Ever married/All women
Both sexes and husband/wives

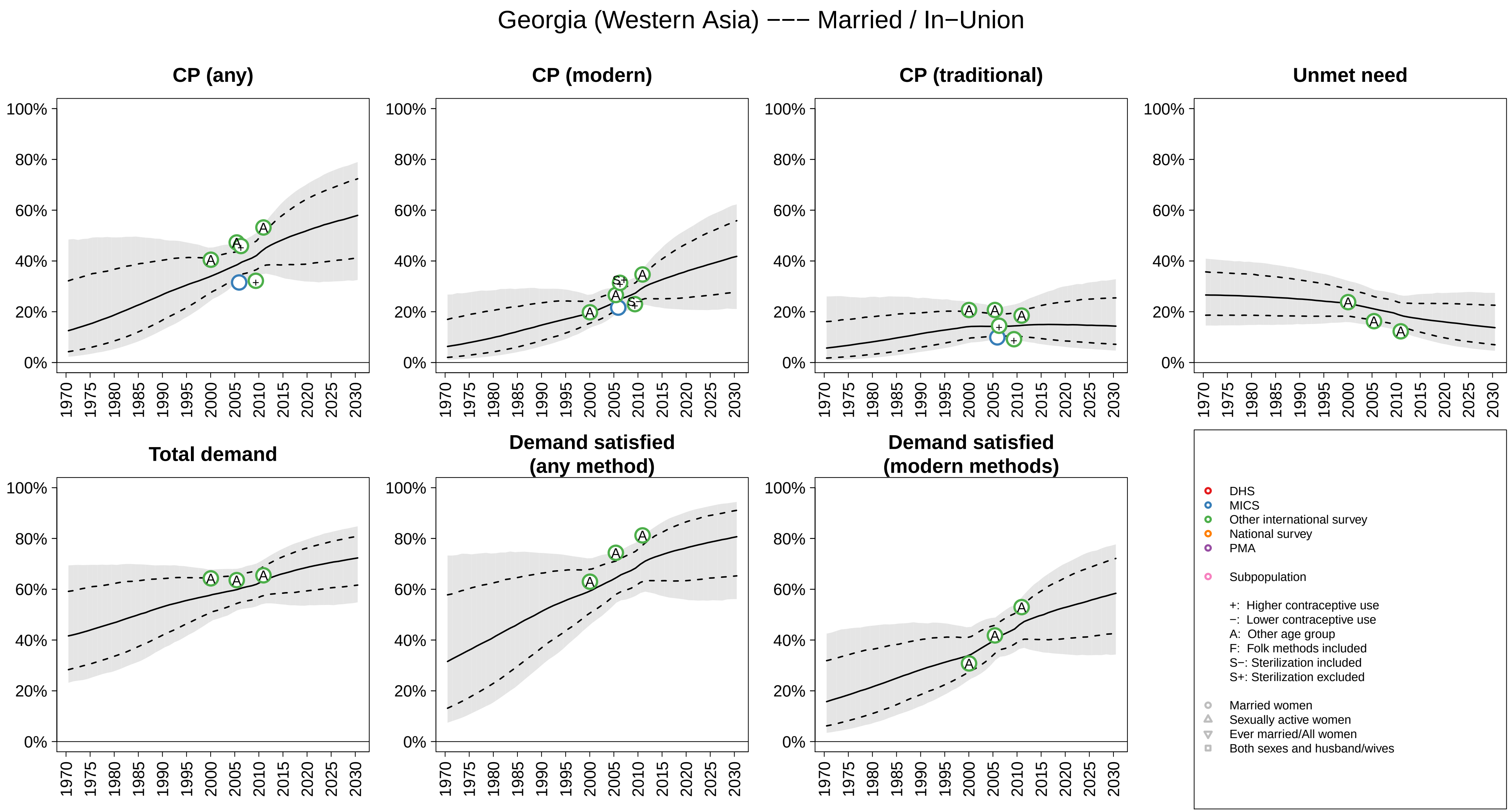
Georgia (Western Asia) −−− Married / In−Union
CP (any)
CP (modern)
CP (traditional)
Unmet need
Total demand
Demand satisfied (any method)
Demand satisfied (modern methods)
DHS
MICS
Other international survey
National survey
PMA
Subpopulation
+: Higher contraceptive use
−: Lower contraceptive use
A: Other age group
F: Folk methods included
S−: Sterilization included
S+: Sterilization excluded
Married women
Sexually active women
Ever married/All women
Both sexes and husband/wives

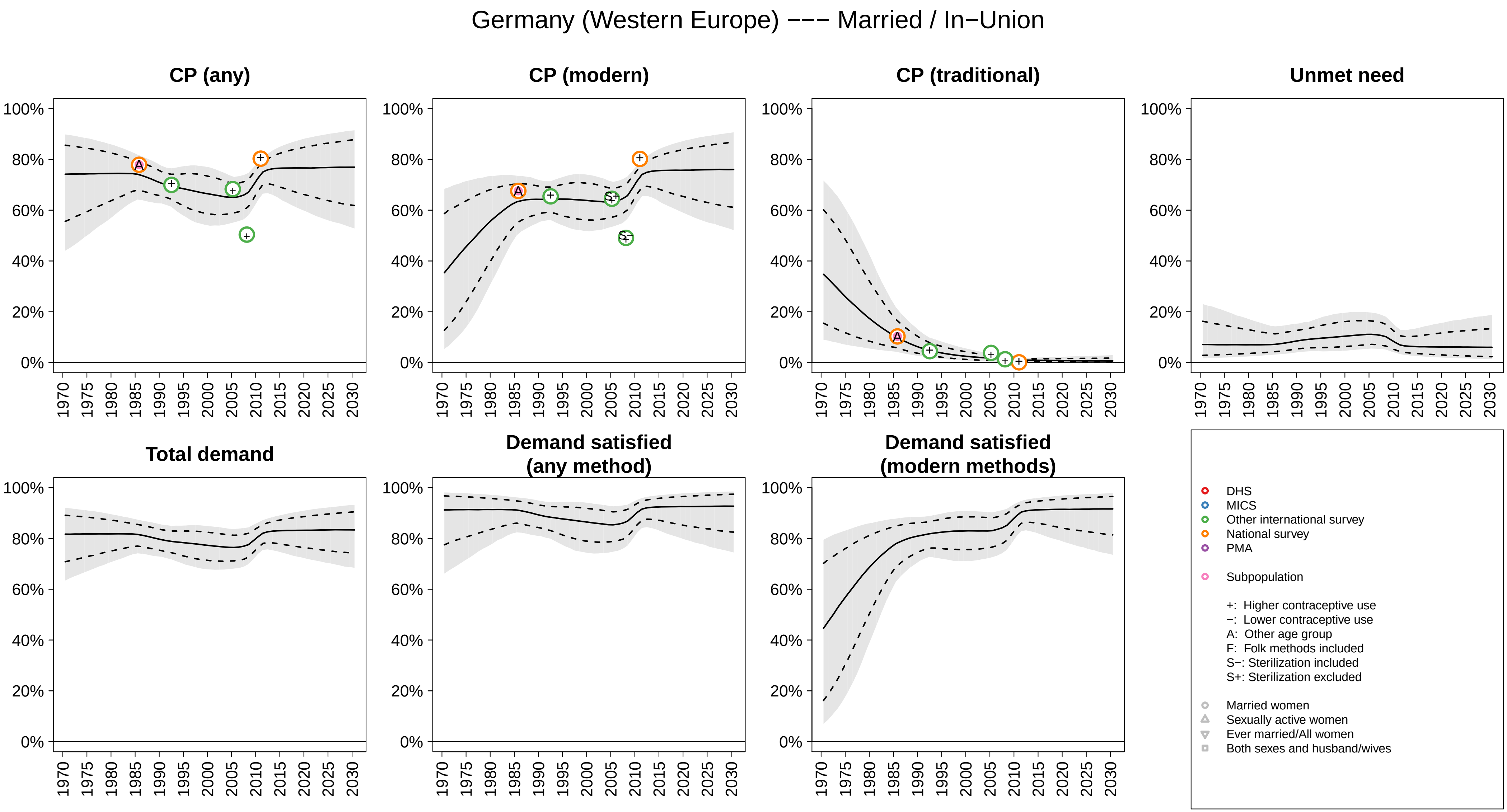
Germany (Western Europe) −−− Married / In−Union
CP (any)
CP (modern)
CP (traditional)
Unmet need
Total demand
Demand satisfied (any method)
Demand satisfied (modern methods)
DHS
MICS
Other international survey
National survey
PMA
Subpopulation
+: Higher contraceptive use
−: Lower contraceptive use
A: Other age group
F: Folk methods included
S−: Sterilization included
S+: Sterilization excluded
Married women
Sexually active women
Ever married/All women
Both sexes and husband/wives

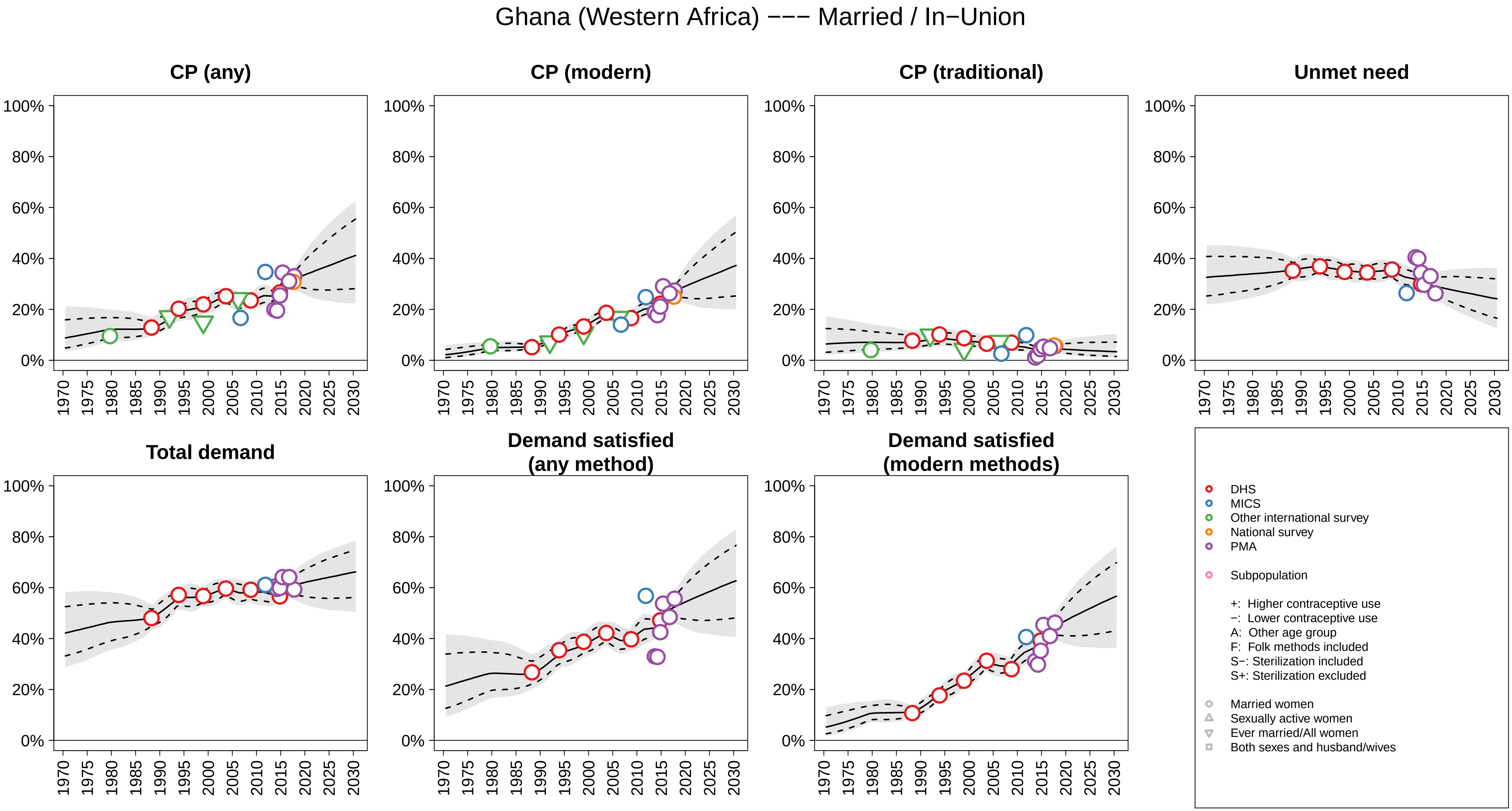
Ghana (Western Africa) −−− Married / In−Union
CP (any)
CP (modern)
CP (traditional)
Unmet need
Total demand
Demand satisfied (any method)
Demand satisfied (modern methods)
0%
20%
40%
60%
80%
100%
1970
1975
1980
1985
1990
1995
2000
2005
2010
2015
2020
2025
2030
DHS
MICS
Other international survey
National survey
PMA
Subpopulation
+: Higher contraceptive use
−: Lower contraceptive use
A: Other age group
F: Folk methods included
S−: Sterilization included
S+: Sterilization excluded
Married women
Sexually active women
Ever married/All women
Both sexes and husband/wives

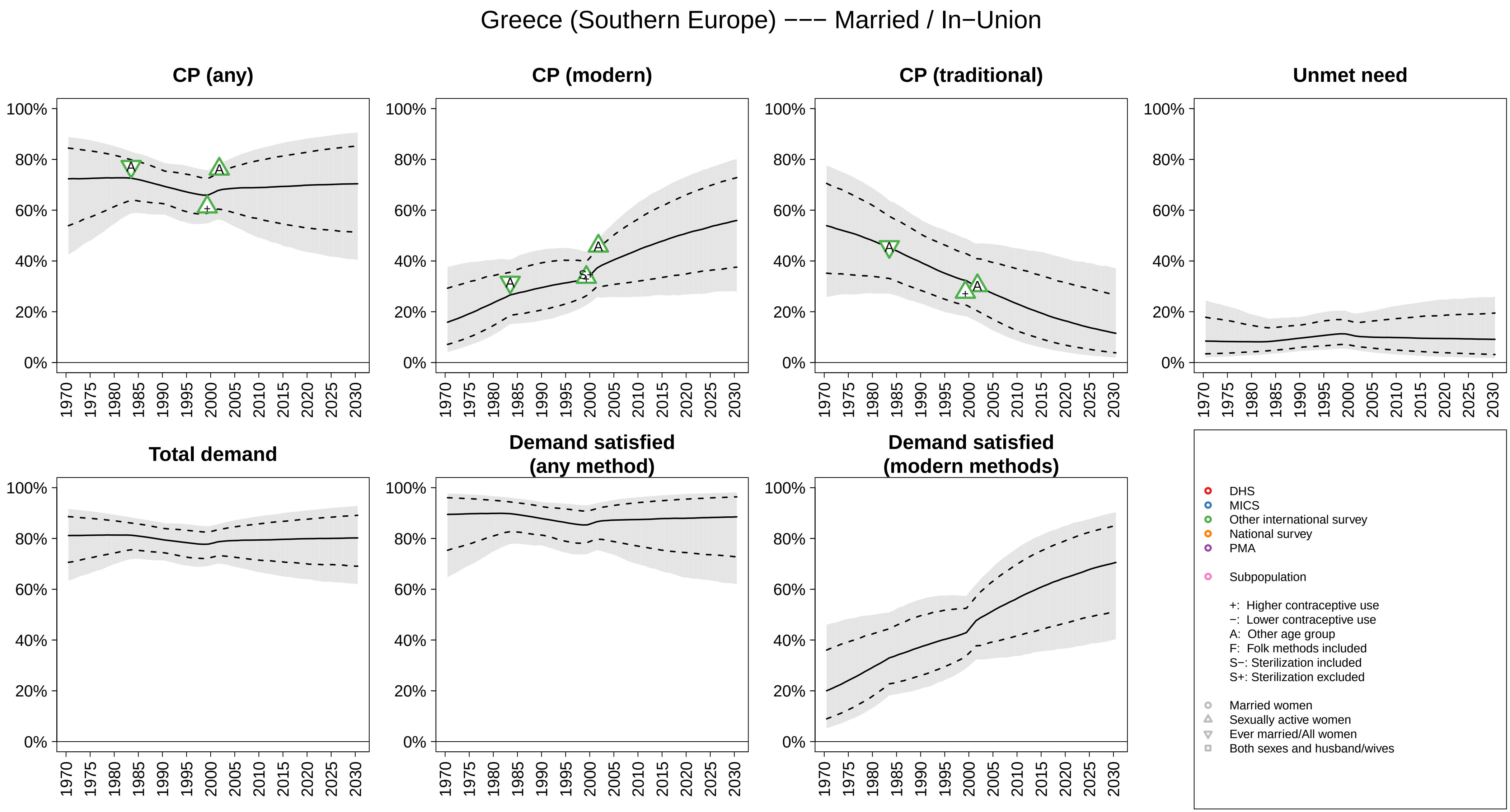
Greece (Southern Europe) −−− Married / In−Union
CP (any)
CP (modern)
CP (traditional)
Unmet need
Total demand
Demand satisfied (any method)
Demand satisfied (modern methods)
DHS
MICS
Other international survey
National survey
PMA
Subpopulation
+: Higher contraceptive use
−: Lower contraceptive use
A: Other age group
F: Folk methods included
S−: Sterilization included
S+: Sterilization excluded
Married women
Sexually active women
Ever married/All women
Both sexes and husband/wives

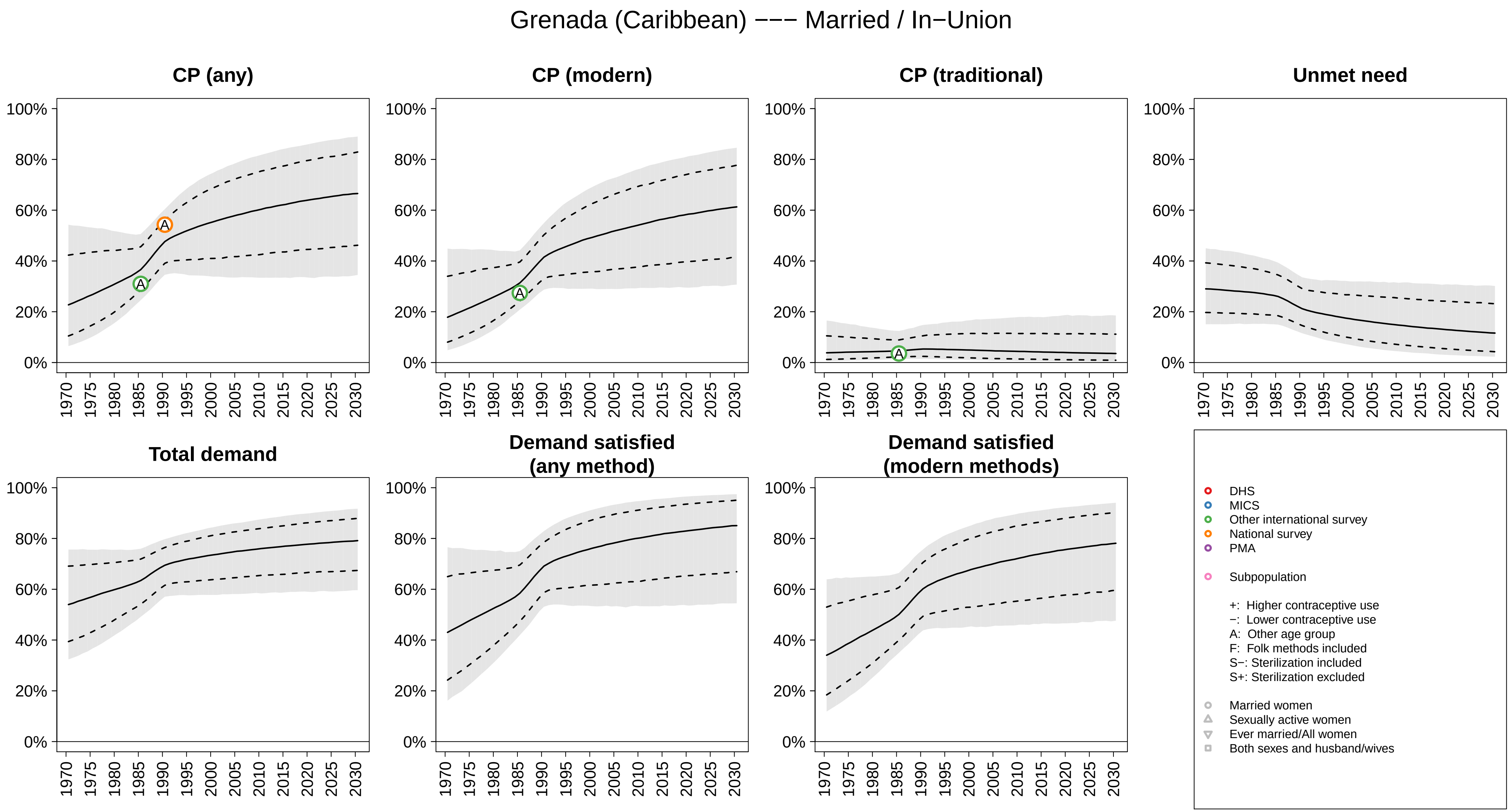

Grenada (Caribbean) −−− Married / In−Union
CP (any)
CP (modern)
CP (traditional)
Unmet need
Total demand
Demand satisfied (any method)
Demand satisfied (modern methods)
DHS
MICS
Other international survey
National survey
PMA
Subpopulation
+: Higher contraceptive use
−: Lower contraceptive use
A: Other age group
F: Folk methods included
S−: Sterilization included
S+: Sterilization excluded
Married women
Sexually active women
Ever married/All women
Both sexes and husband/wives

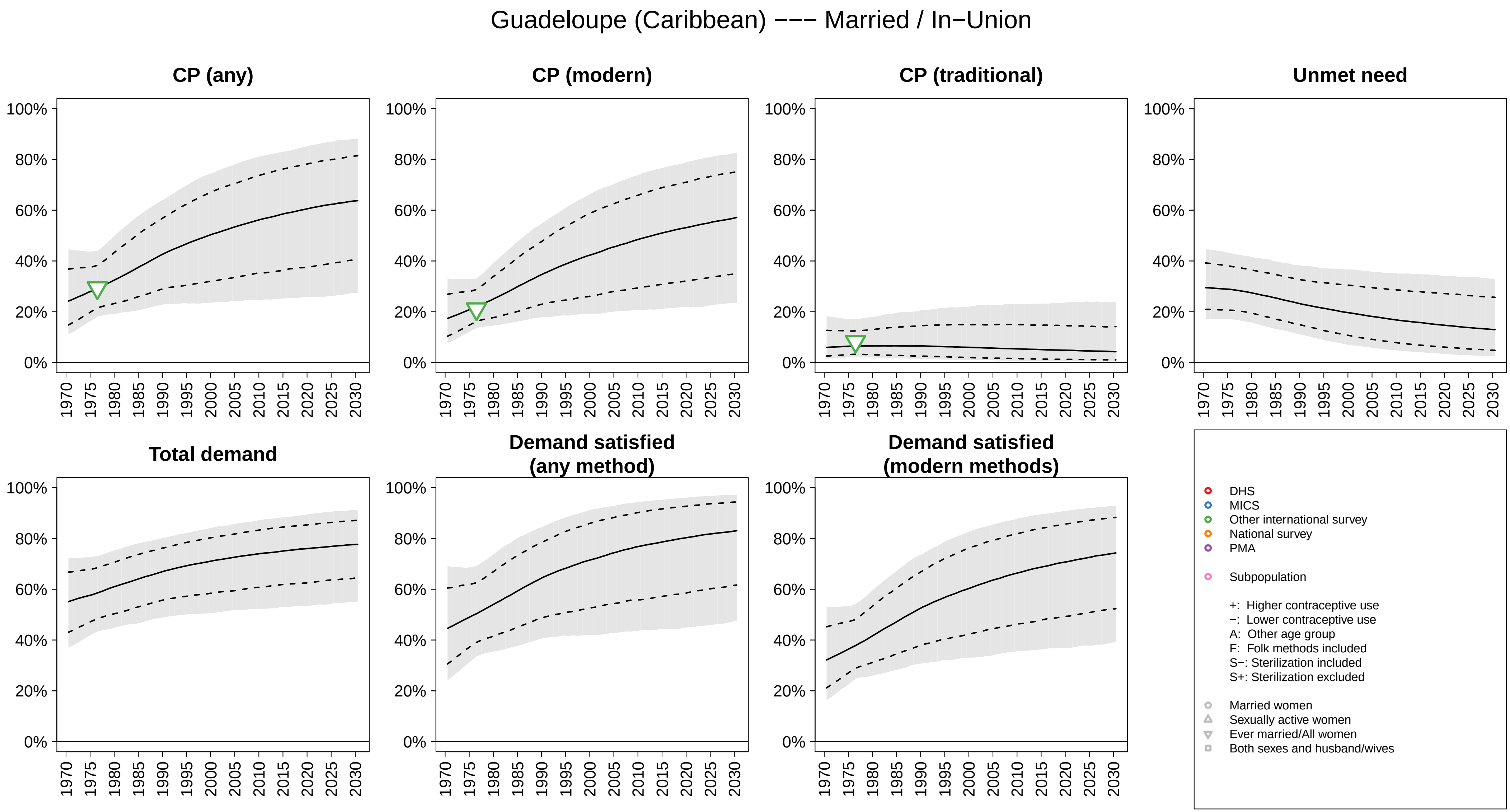
Guadeloupe (Caribbean) −−− Married / In−Union
CP (any)
CP (modern)
CP (traditional)
Unmet need
Total demand
Demand satisfied (any method)
Demand satisfied (modern methods)
0%
20%
40%
60%
80%
100%
1970
1975
1980
1985
1990
1995
2000
2005
2010
2015
2020
2025
2030
DHS
MICS
Other international survey
National survey
PMA
Subpopulation
+: Higher contraceptive use
−: Lower contraceptive use
A: Other age group
F: Folk methods included
S−: Sterilization included
S+: Sterilization excluded
Married women
Sexually active women
Ever married/All women
Both sexes and husband/wives

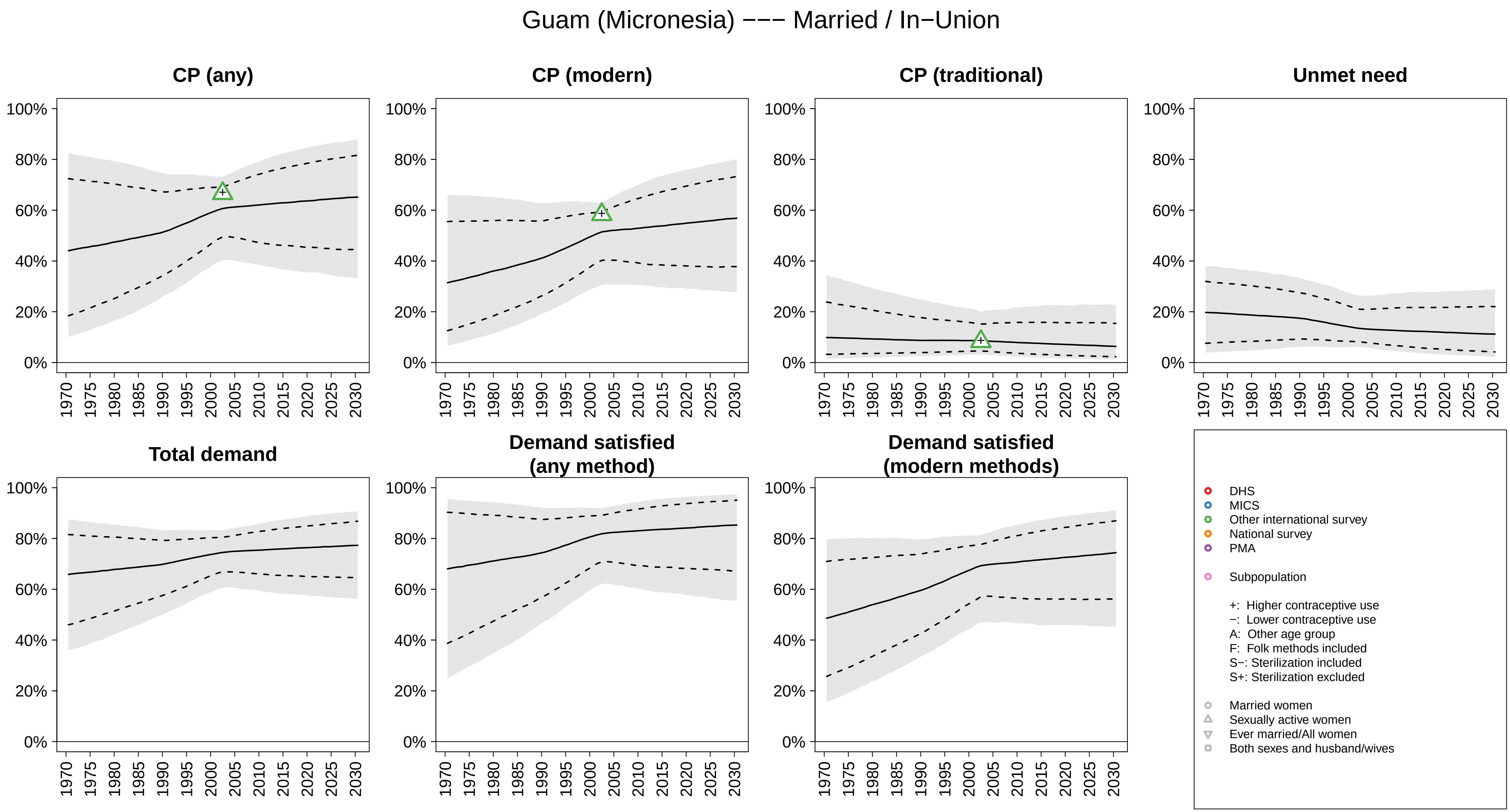
Guam (Micronesia) −−− Married / In−Union
CP (any)
CP (modern)
CP (traditional)
Unmet need
Total demand
Demand satisfied (any method)
Demand satisfied (modern methods)
100%
80%
60%
40%
20%
0%
1970
1975
1980
1985
1990
1995
2000
2005
2010
2015
2020
2025
2030
DHS
MICS
Other international survey
National survey
PMA
Subpopulation
+: Higher contraceptive use
−: Lower contraceptive use
A: Other age group
F: Folk methods included
S−: Sterilization included
S+: Sterilization excluded
Married women
Sexually active women
Ever married/All women
Both sexes and husband/wives

Guatemala (Central America) −−− Married / In−Union

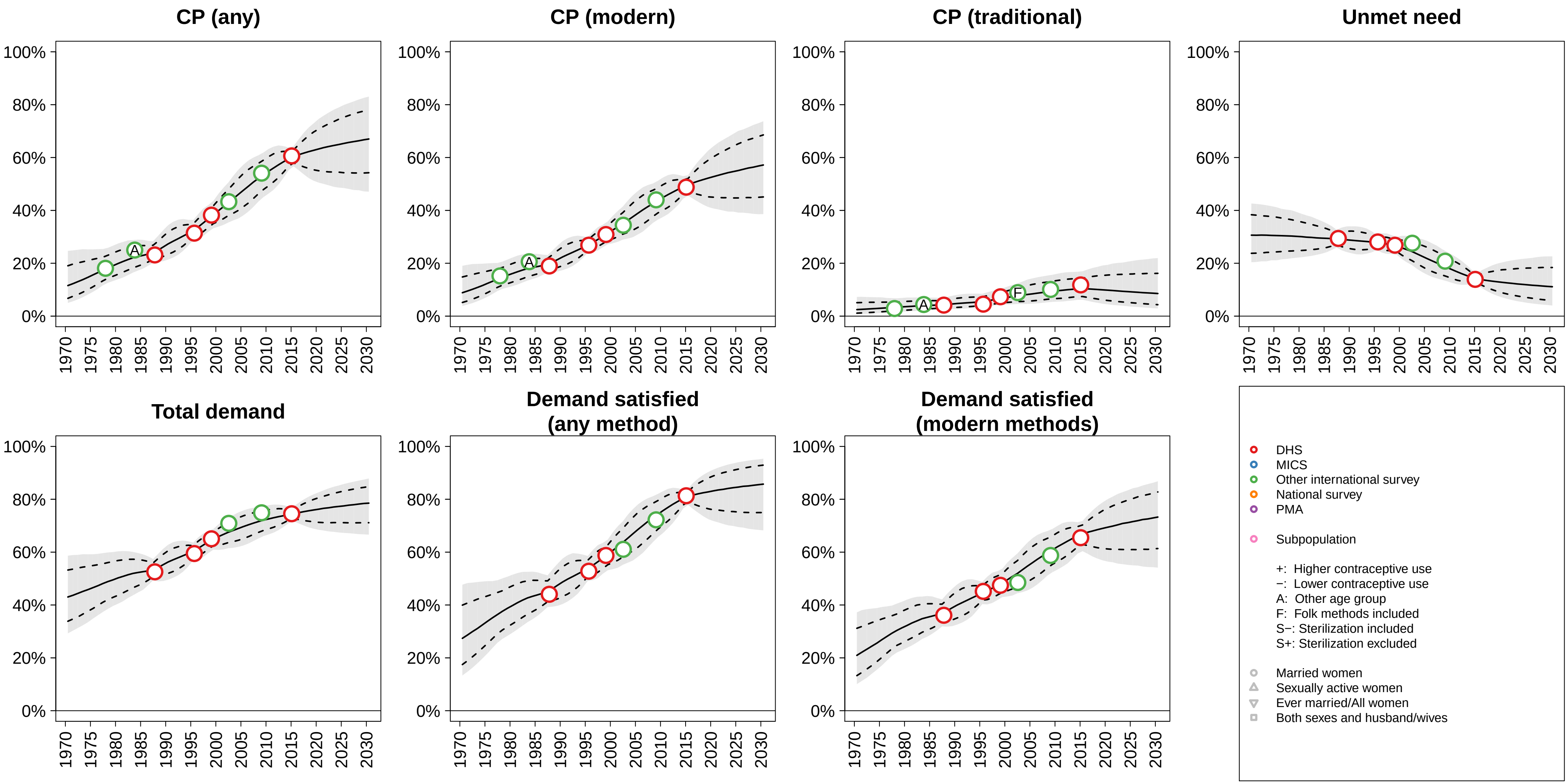

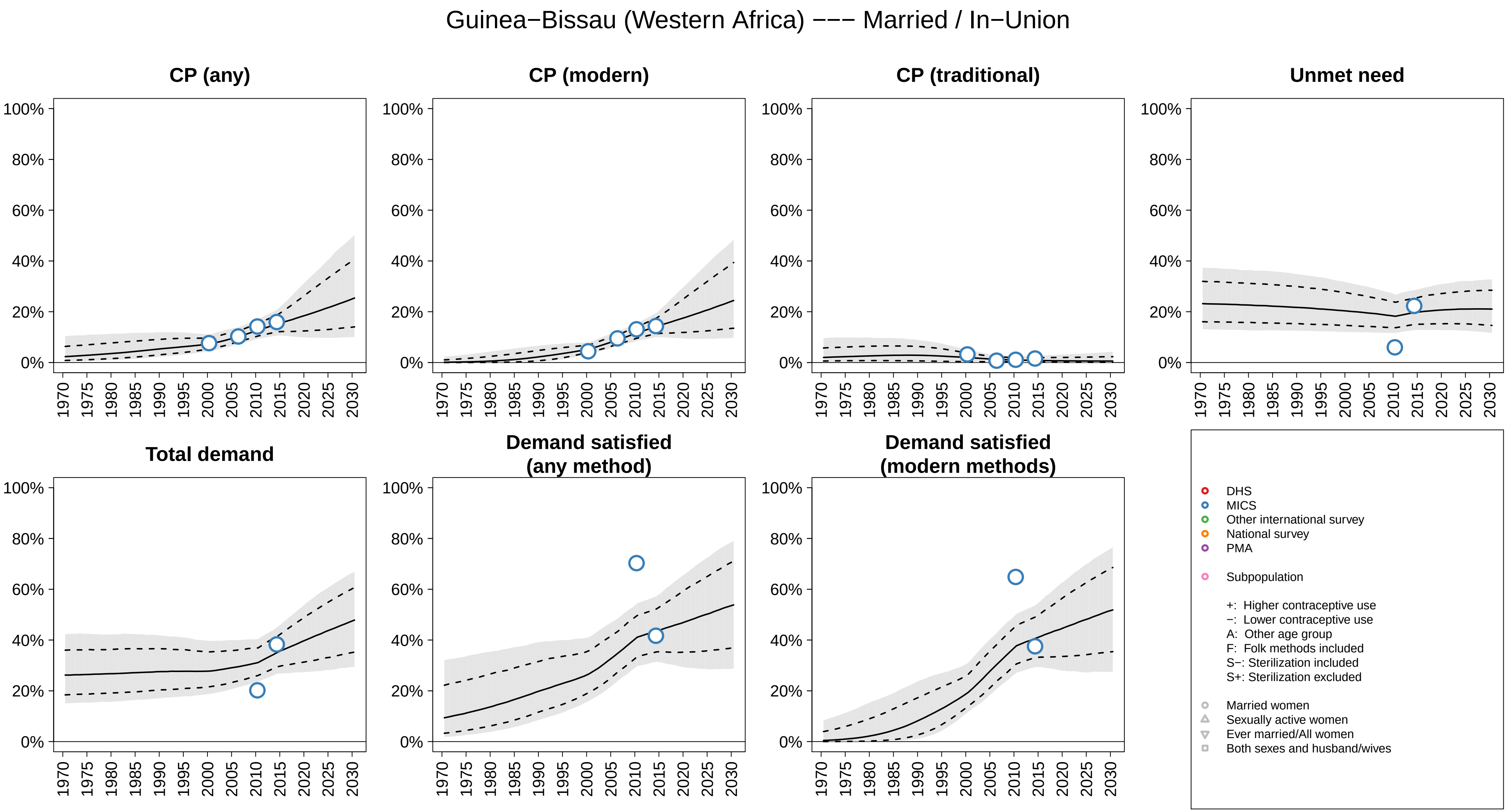
Guinea−Bissau (Western Africa) −−− Married / In−Union
CP (any)
CP (modern)
CP (traditional)
Unmet need
Total demand
Demand satisfied (any method)
Demand satisfied (modern methods)
100%
80%
60%
40%
20%
0%
1970
1975
1980
1985
1990
1995
2000
2005
2010
2015
2020
2025
2030
DHS
MICS
Other international survey
National survey
PMA
Subpopulation
+: Higher contraceptive use
−: Lower contraceptive use
A: Other age group
F: Folk methods included
S−: Sterilization included
S+: Sterilization excluded
Married women
Sexually active women
Ever married/All women
Both sexes and husband/wives

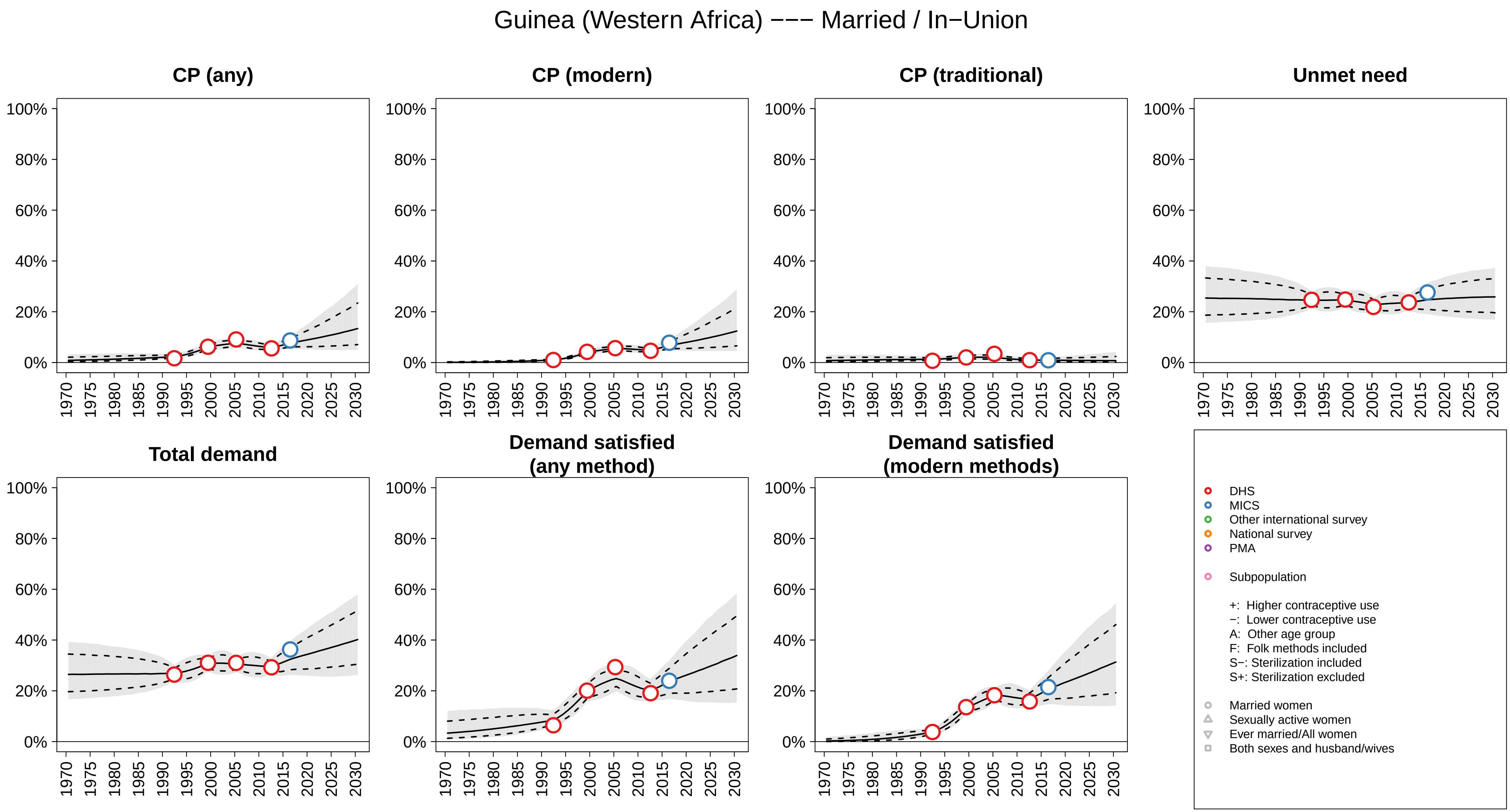
Guinea (Western Africa) −−− Married / In−Union
CP (any)
CP (modern)
CP (traditional)
Unmet need
Total demand
Demand satisfied (any method)
Demand satisfied (modern methods)
0%
20%
40%
60%
80%
100%
1970
1975
1980
1985
1990
1995
2000
2005
2010
2015
2020
2025
2030
DHS
MICS
Other international survey
National survey
PMA
Subpopulation
+: Higher contraceptive use
−: Lower contraceptive use
A: Other age group
F: Folk methods included
S−: Sterilization included
S+: Sterilization excluded
Married women
Sexually active women
Ever married/All women
Both sexes and husband/wives

Guyana (South America) −−− Married / In−Union

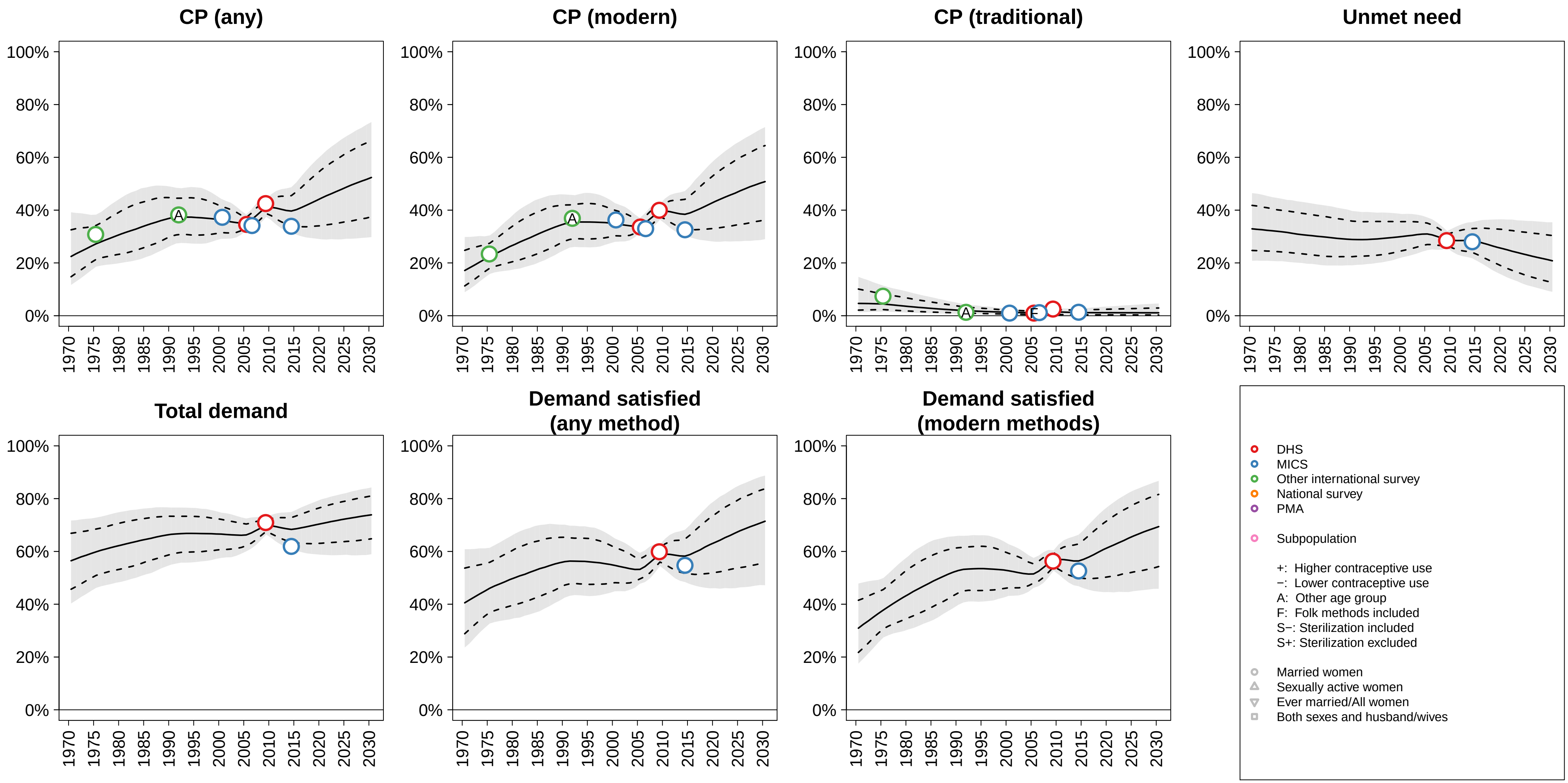

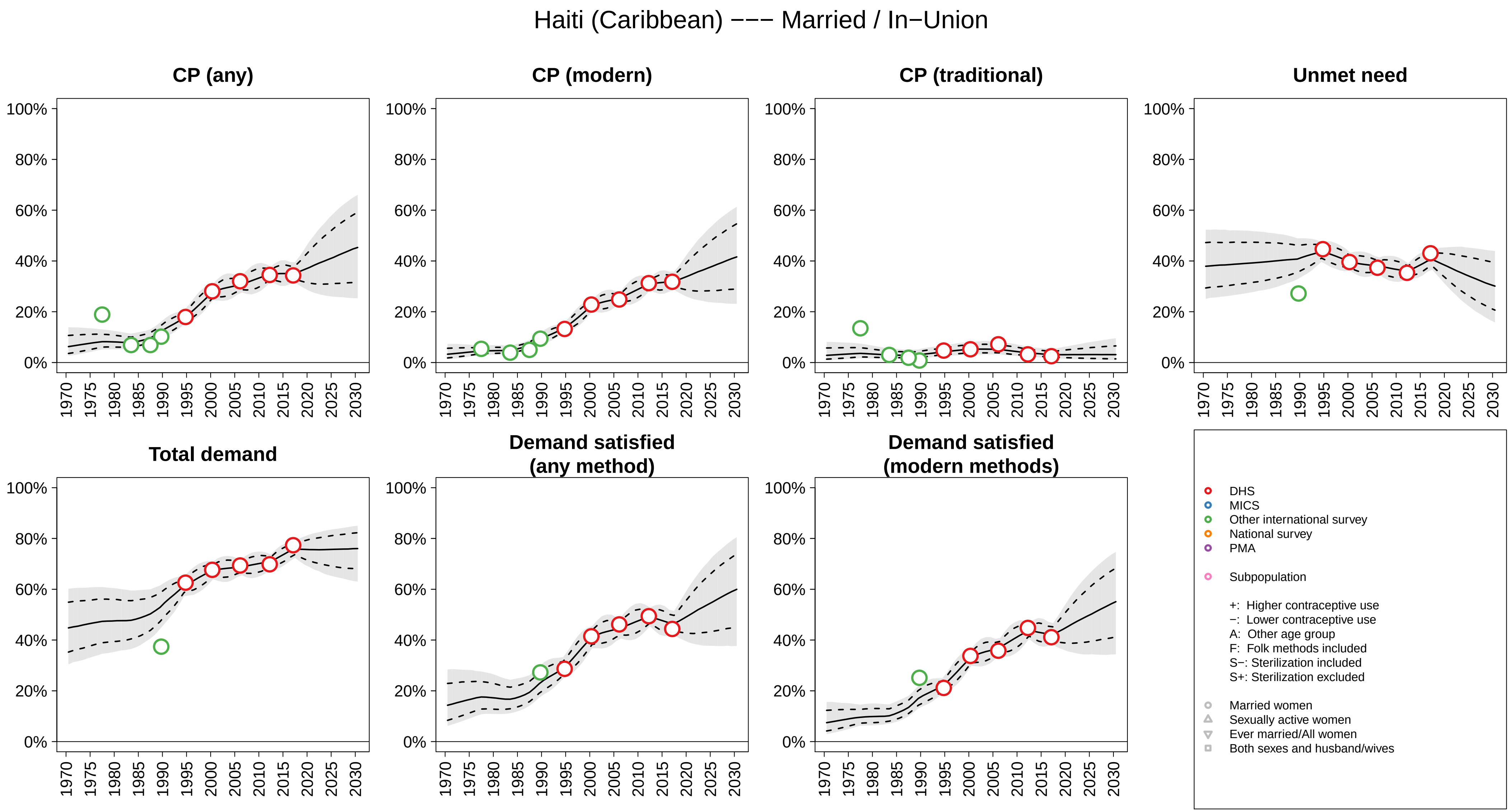
Haiti (Caribbean) --- Married / In−Union
CP (any)
CP (modern)
CP (traditional)
Unmet need
Total demand
Demand satisfied (any method)
Demand satisfied (modern methods)
0%
20%
40%
60%
80%
100%
1970
1975
1980
1985
1990
1995
2000
2005
2010
2015
2020
2025
2030
DHS
MICS
Other international survey
National survey
PMA
Subpopulation
+: Higher contraceptive use
−: Lower contraceptive use
A: Other age group
F: Folk methods included
S−: Sterilization included
S+: Sterilization excluded
Married women
Sexually active women
Ever married/All women
Both sexes and husband/wives

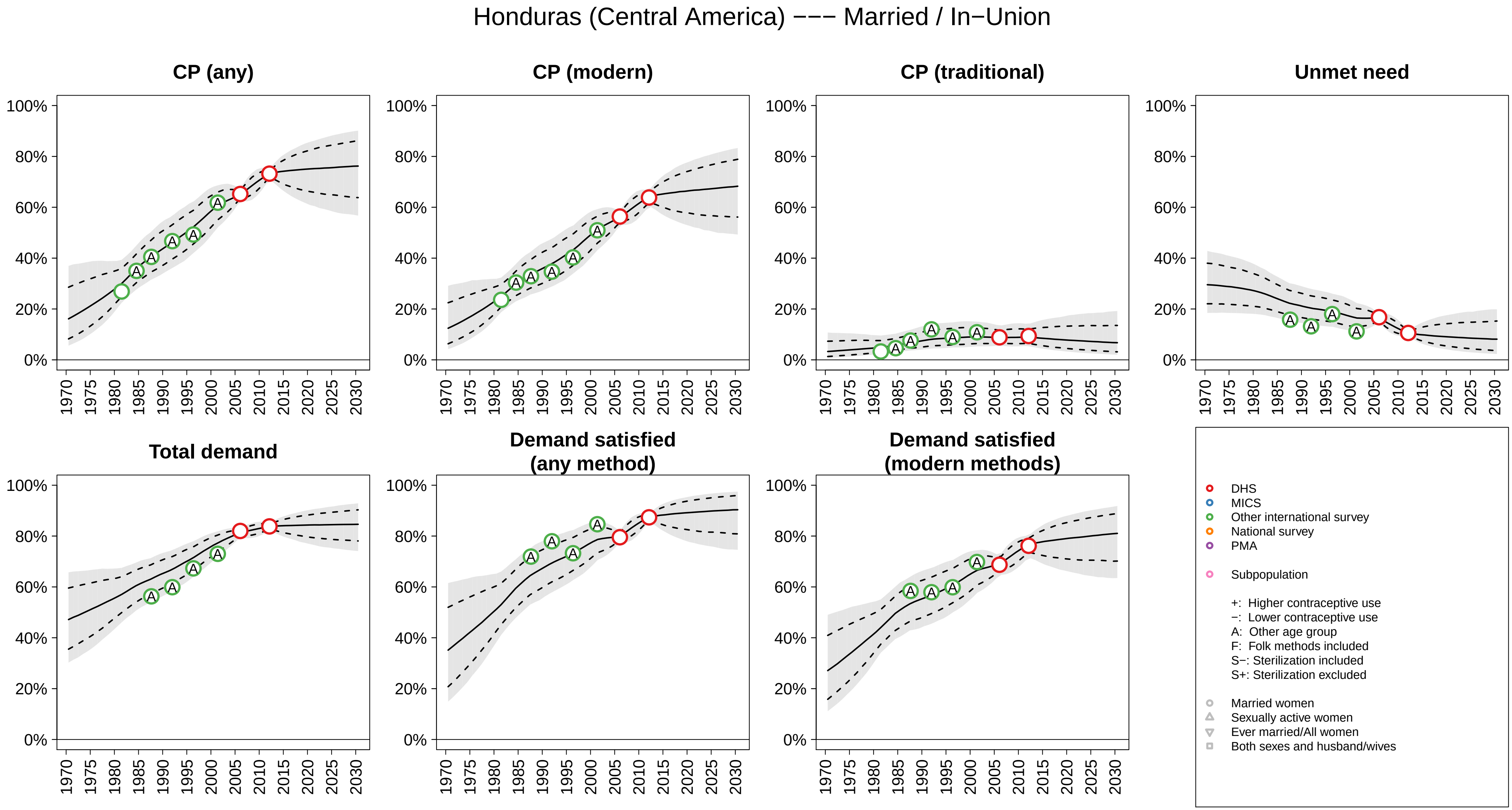
Honduras (Central America) −−− Married / In−Union
CP (any)
CP (modern)
CP (traditional)
Unmet need
Total demand
Demand satisfied (any method)
Demand satisfied (modern methods)
100%
80%
60%
40%
20%
0%
1970
1975
1980
1985
1990
1995
2000
2005
2010
2015
2020
2025
2030
DHS
MICS
Other international survey
National survey
PMA
Subpopulation
+: Higher contraceptive use
−: Lower contraceptive use
A: Other age group
F: Folk methods included
S−: Sterilization included
S+: Sterilization excluded
Married women
Sexually active women
Ever married/All women
Both sexes and husband/wives

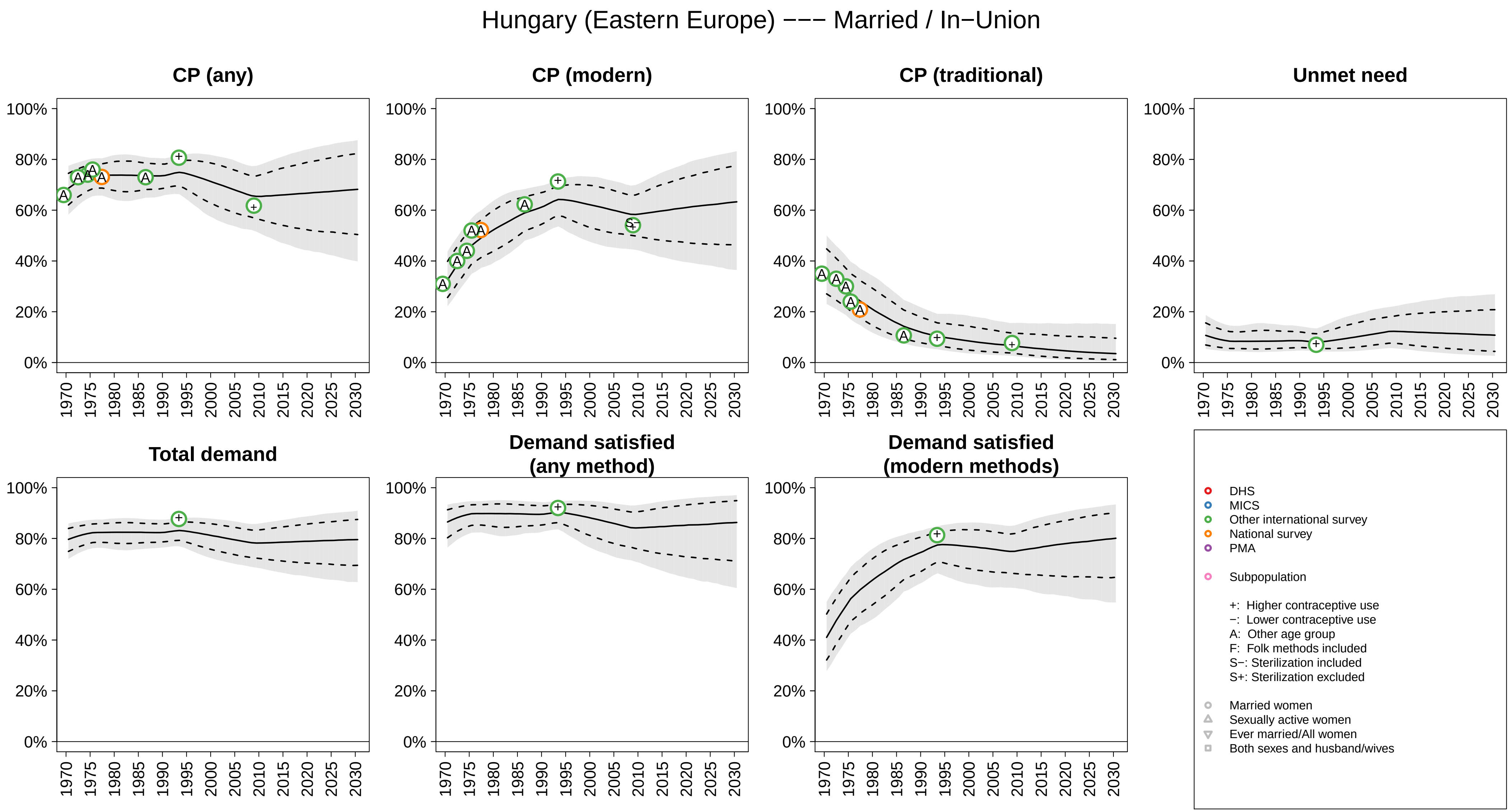

Hungary (Eastern Europe) --- Married / In−Union
CP (any)
CP (modern)
CP (traditional)
Unmet need
Total demand
Demand satisfied (any method)
Demand satisfied (modern methods)
100%
80%
60%
40%
20%
0%
1970 1975 1980 1985 1990 1995 2000 2005 2010 2015 2020 2025 2030
DHS
MICS
Other international survey
National survey
PMA
Subpopulation
+: Higher contraceptive use
−: Lower contraceptive use
A: Other age group
F: Folk methods included
S−: Sterilization included
S+: Sterilization excluded
Married women
Sexually active women
Ever married/All women
Both sexes and husband/wives

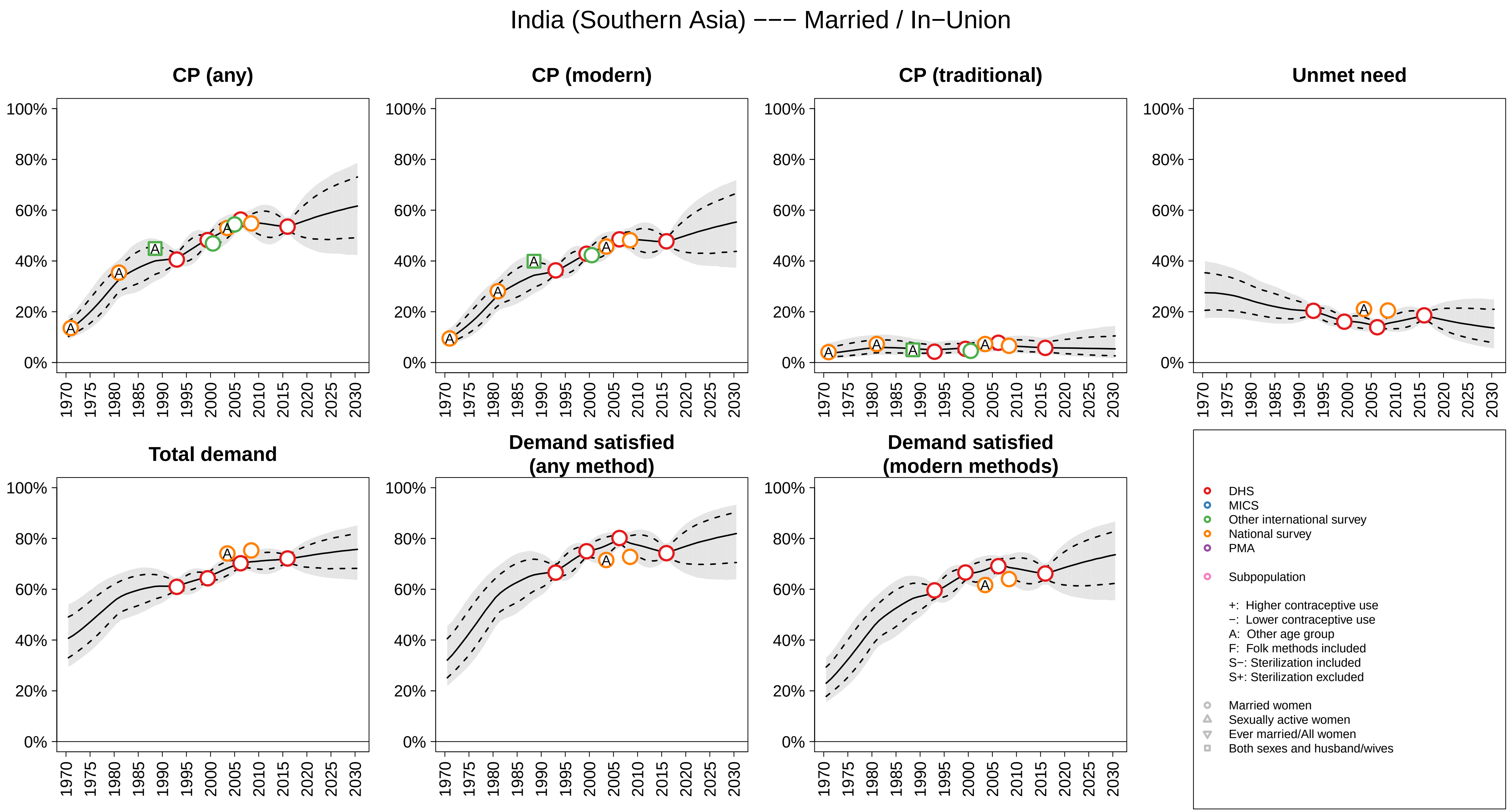
India (Southern Asia) −−− Married / In−Union
CP (any)
CP (modern)
CP (traditional)
Unmet need
Total demand
Demand satisfied (any method)
Demand satisfied (modern methods)
100%
80%
60%
40%
20%
0%
1970
1975
1980
1985
1990
1995
2000
2005
2010
2015
2020
2025
2030
A
DHS
MICS
Other international survey
National survey
PMA
Subpopulation
+: Higher contraceptive use
−: Lower contraceptive use
A: Other age group
F: Folk methods included
S−: Sterilization included
S+: Sterilization excluded
Married women
Sexually active women
Ever married/All women
Both sexes and husband/wives

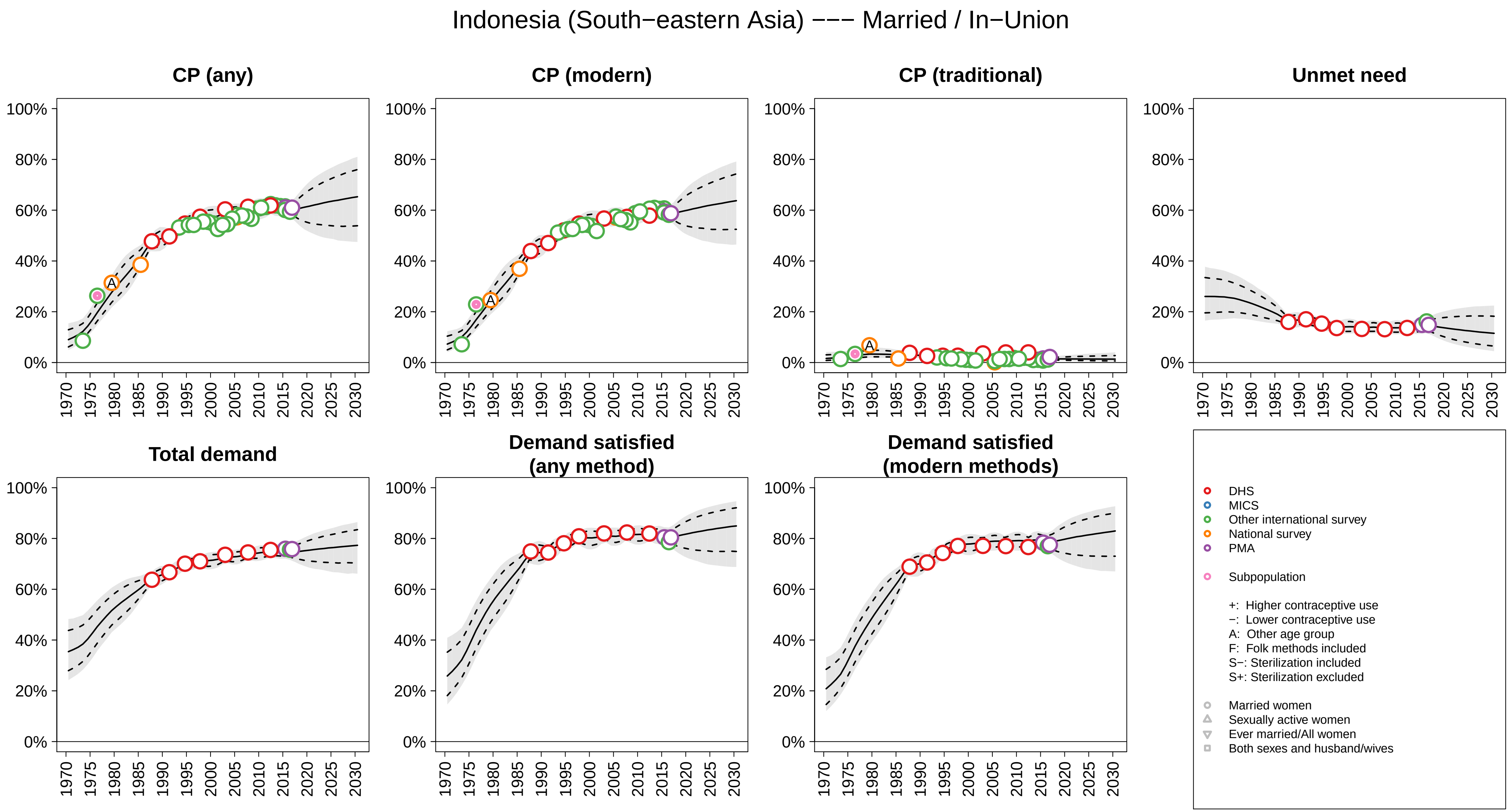
Indonesia (South−eastern Asia) −−− Married / In−Union
CP (any)
CP (modern)
CP (traditional)
Unmet need
Total demand
Demand satisfied (any method)
Demand satisfied (modern methods)
0%
20%
40%
60%
80%
100%
1970
1975
1980
1985
1990
1995
2000
2005
2010
2015
2020
2025
2030
A
DHS
MICS
Other international survey
National survey
PMA
Subpopulation
+: Higher contraceptive use
−: Lower contraceptive use
A: Other age group
F: Folk methods included
S−: Sterilization included
S+: Sterilization excluded
Married women
Sexually active women
Ever married/All women
Both sexes and husband/wives

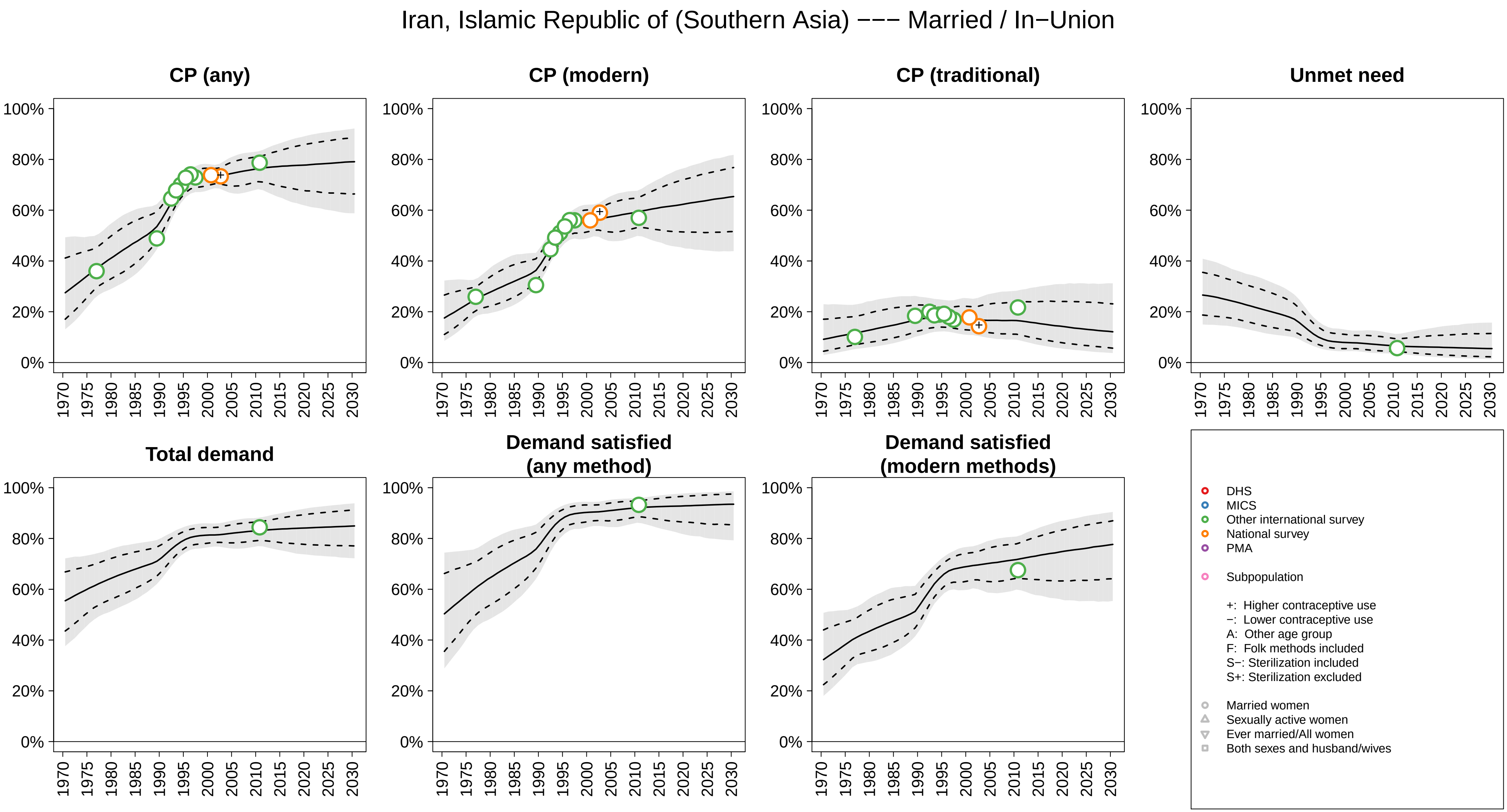
Iran, Islamic Republic of (Southern Asia) −−− Married / In−Union
CP (any)
CP (modern)
CP (traditional)
Unmet need
Total demand
Demand satisfied (any method)
Demand satisfied (modern methods)
0%
20%
40%
60%
80%
100%
1970
1975
1980
1985
1990
1995
2000
2005
2010
2015
2020
2025
2030
DHS
MICS
Other international survey
National survey
PMA
Subpopulation
+: Higher contraceptive use
−: Lower contraceptive use
A: Other age group
F: Folk methods included
S−: Sterilization included
S+: Sterilization excluded
Married women
Sexually active women
Ever married/All women
Both sexes and husband/wives

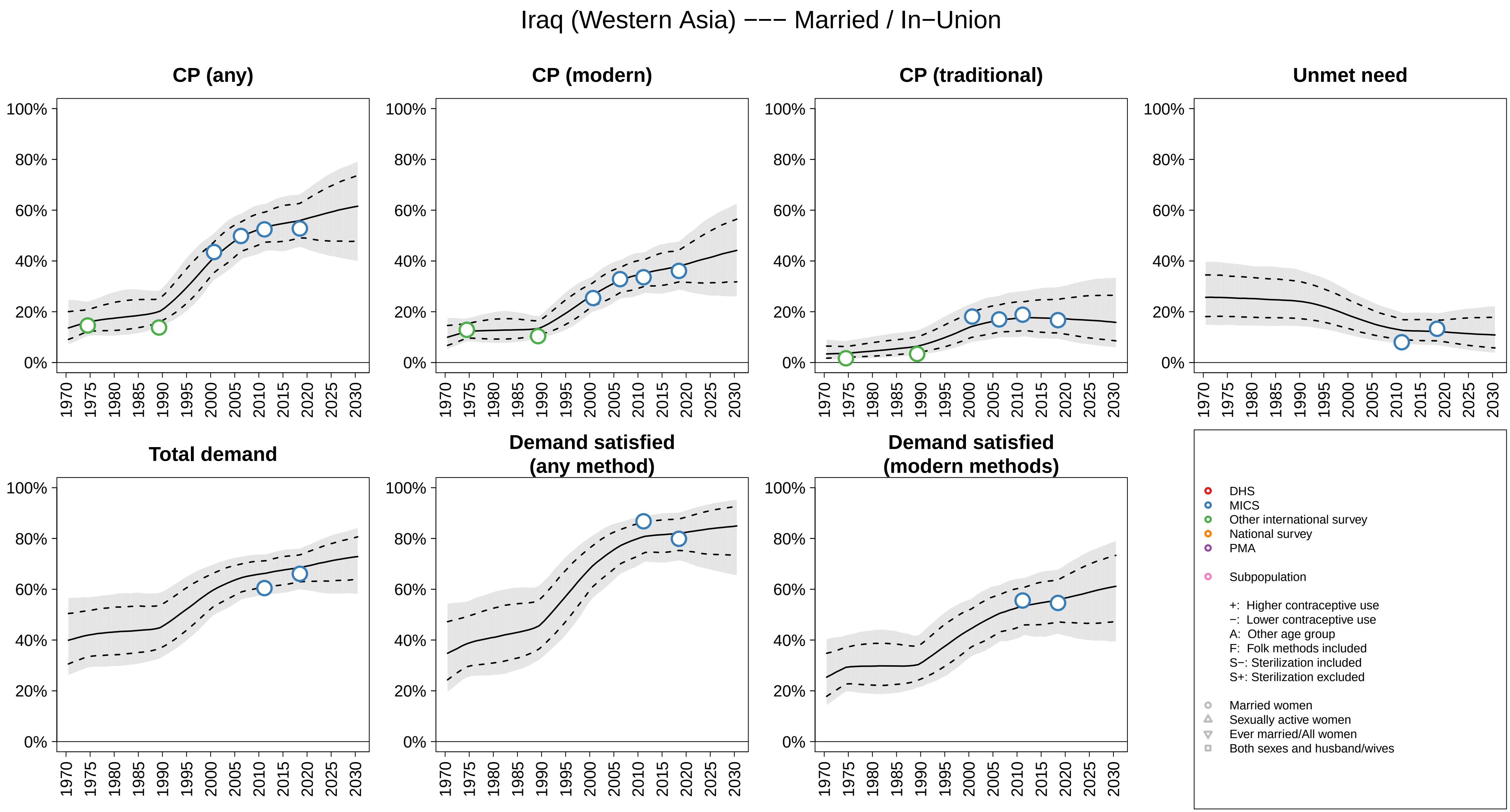
Iraq (Western Asia) −−− Married / In−Union
CP (any)
CP (modern)
CP (traditional)
Unmet need
Total demand
Demand satisfied (any method)
Demand satisfied (modern methods)
0%
20%
40%
60%
80%
100%
1970
1975
1980
1985
1990
1995
2000
2005
2010
2015
2020
2025
2030
DHS
MICS
Other international survey
National survey
PMA
Subpopulation
+: Higher contraceptive use
−: Lower contraceptive use
A: Other age group
F: Folk methods included
S−: Sterilization included
S+: Sterilization excluded
Married women
Sexually active women
Ever married/All women
Both sexes and husband/wives

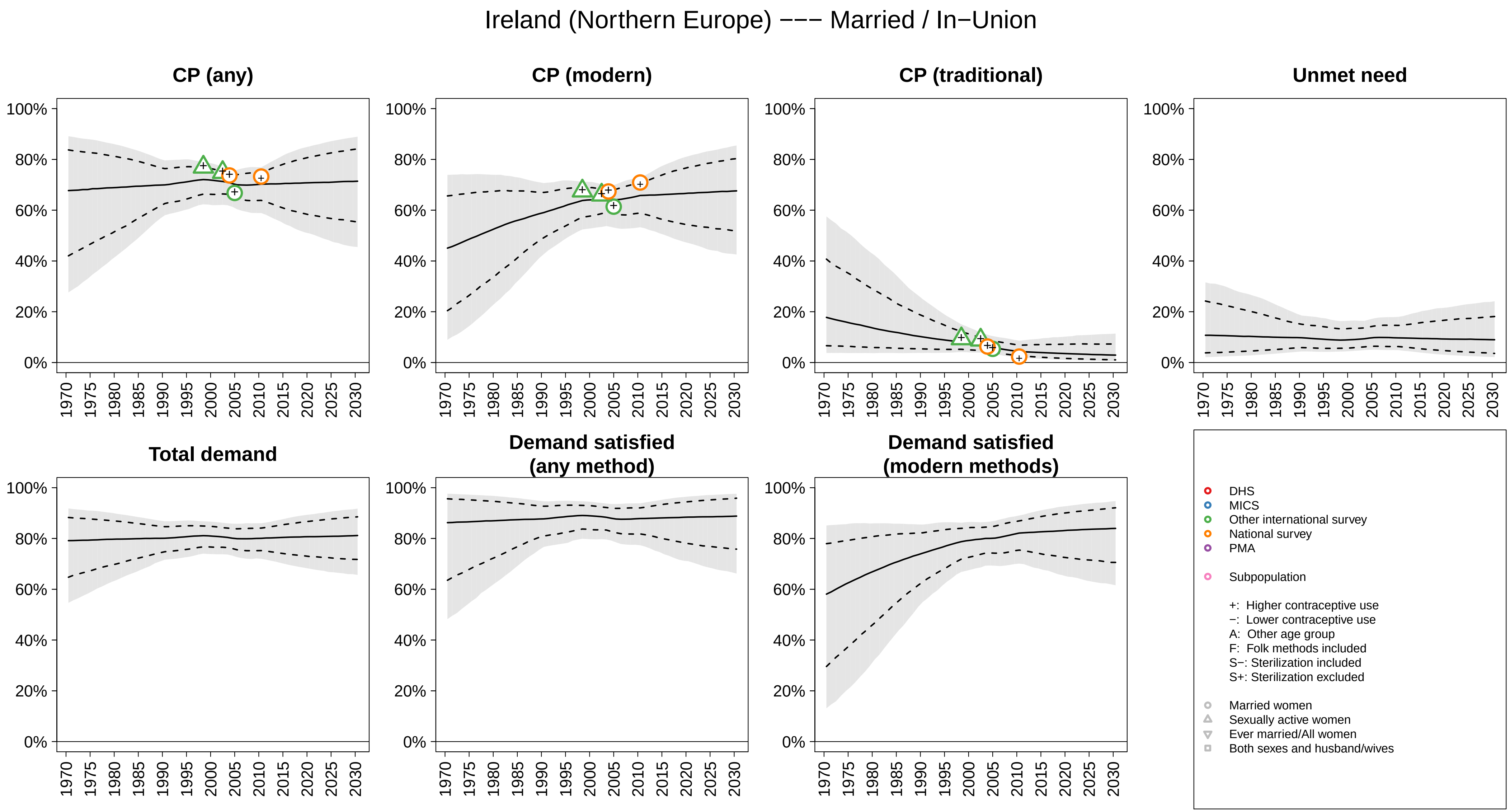
Ireland (Northern Europe) --- Married / In−Union
CP (any)
CP (modern)
CP (traditional)
Unmet need
Total demand
Demand satisfied (any method)
Demand satisfied (modern methods)
0%
20%
40%
60%
80%
100%
1970
1975
1980
1985
1990
1995
2000
2005
2010
2015
2020
2025
2030
DHS
MICS
Other international survey
National survey
PMA
Subpopulation
+: Higher contraceptive use
−: Lower contraceptive use
A: Other age group
F: Folk methods included
S−: Sterilization included
S+: Sterilization excluded
Married women
Sexually active women
Ever married/All women
Both sexes and husband/wives

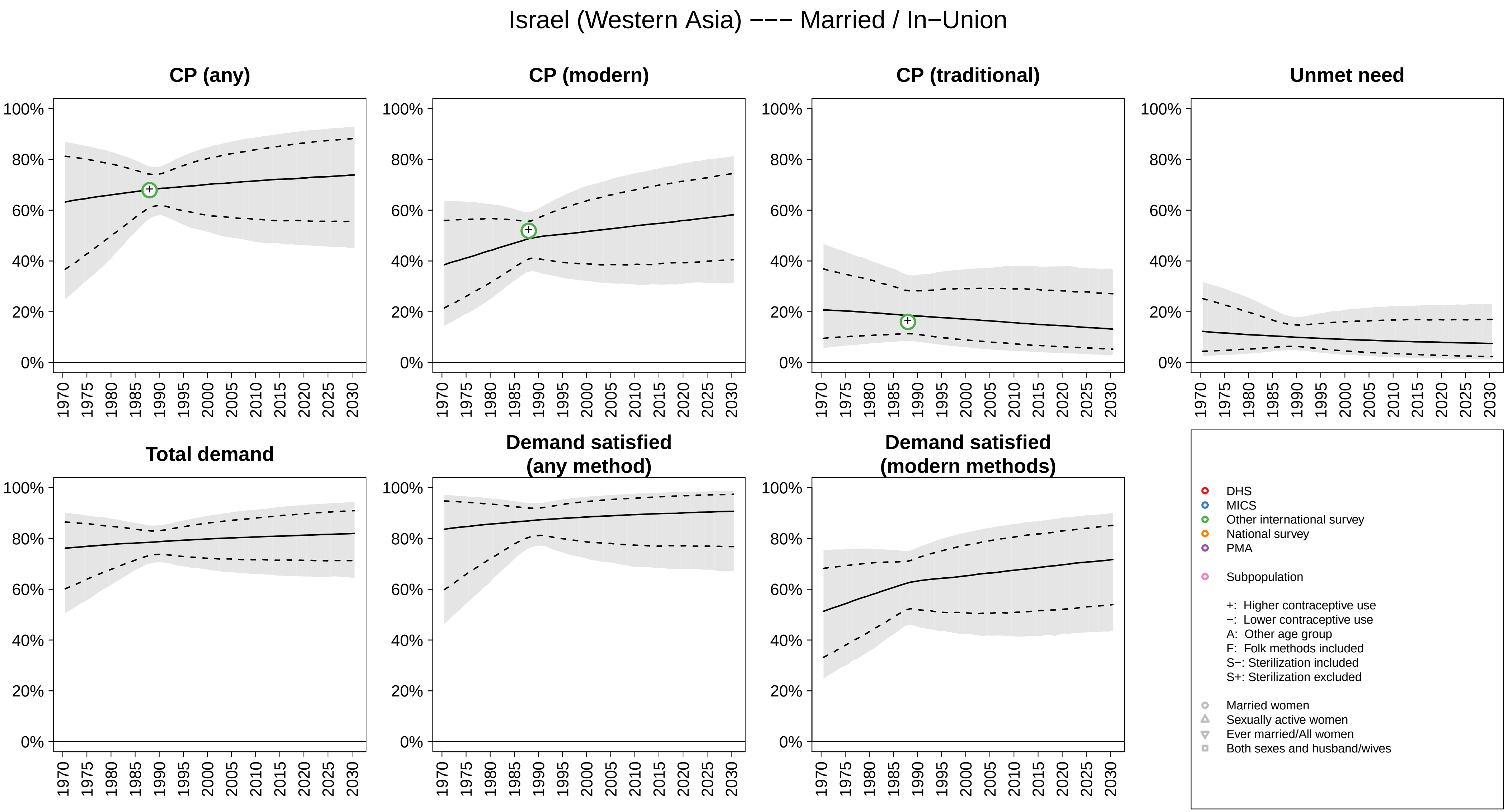
Israel (Western Asia) --- Married / In−Union
CP (any)
CP (modern)
CP (traditional)
Unmet need
Total demand
Demand satisfied (any method)
Demand satisfied (modern methods)
0%
20%
40%
60%
80%
100%
1970
1975
1980
1985
1990
1995
2000
2005
2010
2015
2020
2025
2030
DHS
MICS
Other international survey
National survey
PMA
Subpopulation
+: Higher contraceptive use
−: Lower contraceptive use
A: Other age group
F: Folk methods included
S−: Sterilization included
S+: Sterilization excluded
Married women
Sexually active women
Ever married/All women
Both sexes and husband/wives

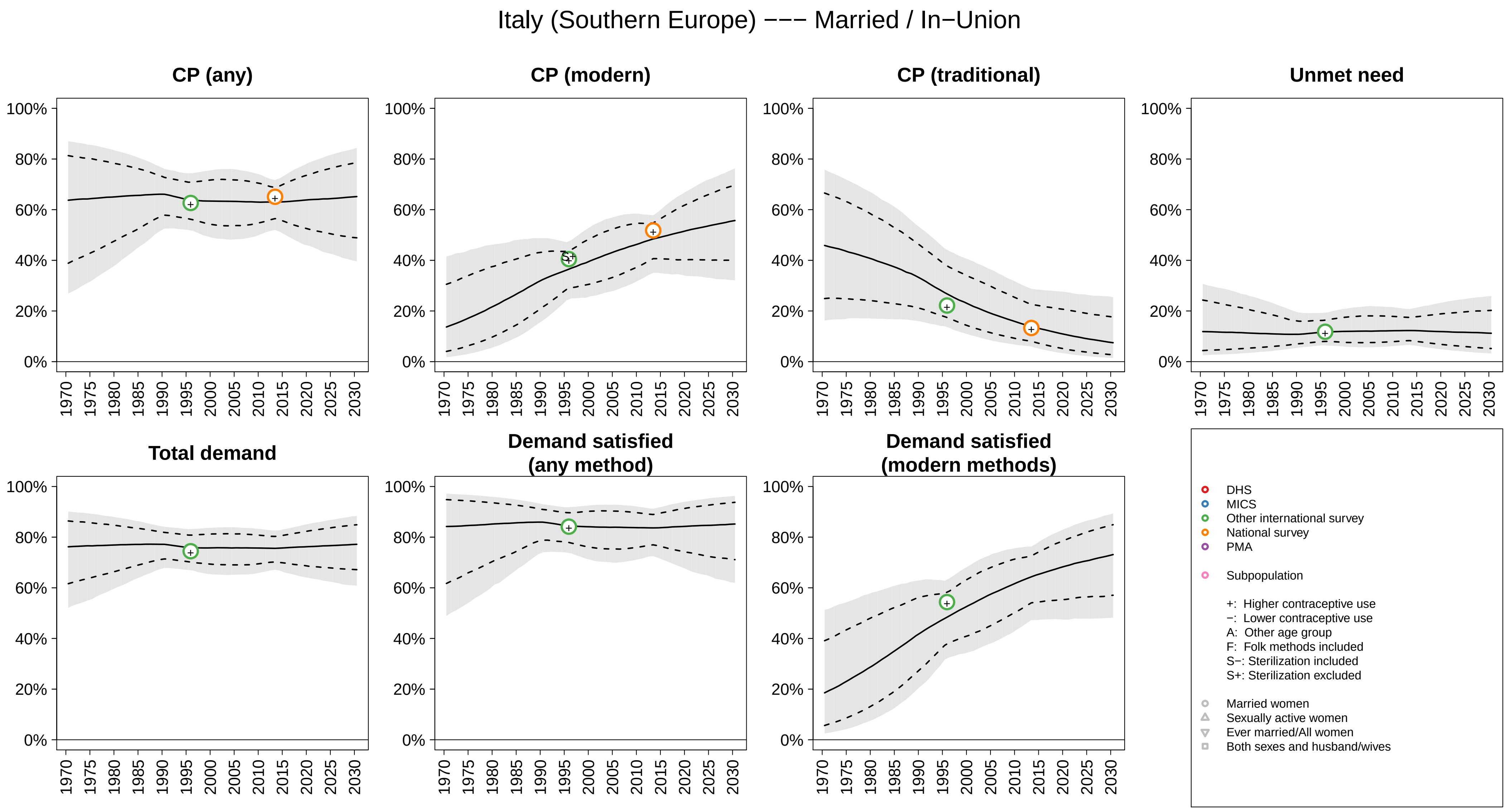
Italy (Southern Europe) −−− Married / In−Union
CP (any)
CP (modern)
CP (traditional)
Unmet need
Total demand
Demand satisfied (any method)
Demand satisfied (modern methods)
0%
20%
40%
60%
80%
100%
1970
1975
1980
1985
1990
1995
2000
2005
2010
2015
2020
2025
2030
DHS
MICS
Other international survey
National survey
PMA
Subpopulation
+: Higher contraceptive use
−: Lower contraceptive use
A: Other age group
F: Folk methods included
S−: Sterilization included
S+: Sterilization excluded
Married women
Sexually active women
Ever married/All women
Both sexes and husband/wives

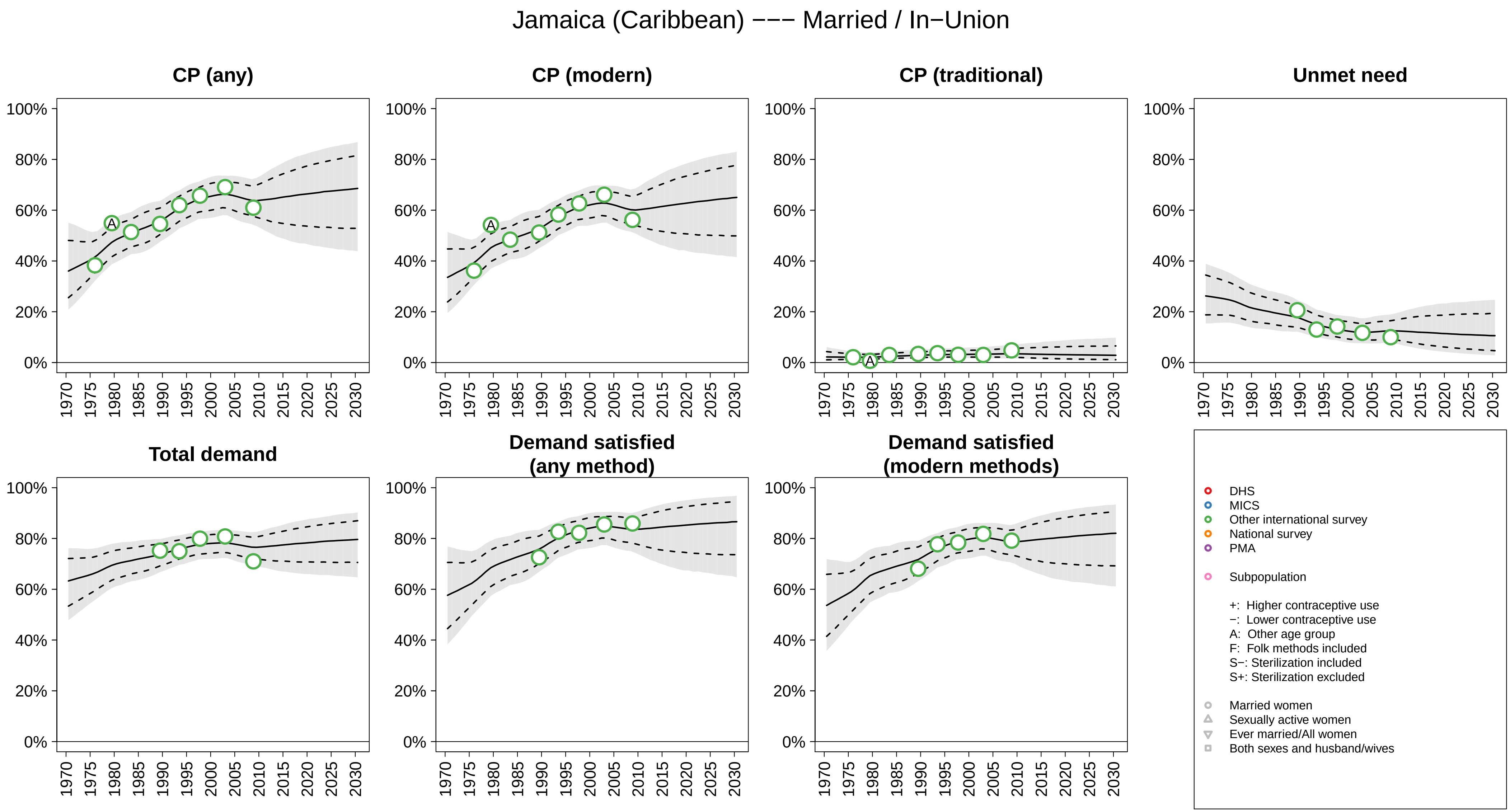

Jamaica (Caribbean) --- Married / In-Union
CP (any)
CP (modern)
CP (traditional)
Unmet need
Total demand
Demand satisfied (any method)
Demand satisfied (modern methods)
DHS
MICS
Other international survey
National survey
PMA
Subpopulation
+: Higher contraceptive use
−: Lower contraceptive use
A: Other age group
F: Folk methods included
S−: Sterilization included
S+: Sterilization excluded
Married women
Sexually active women
Ever married/All women
Both sexes and husband/wives

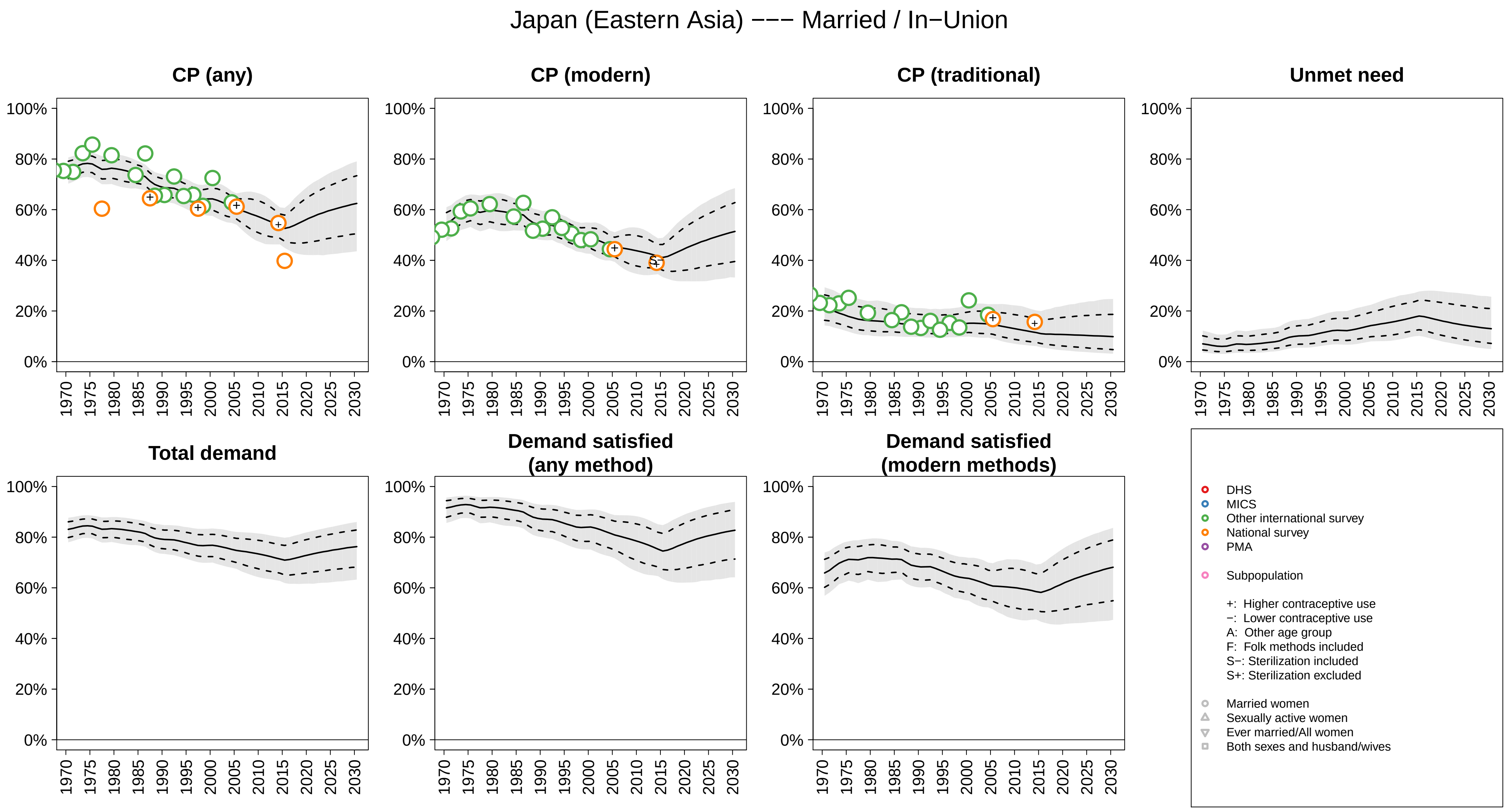
Japan (Eastern Asia) --- Married / In−Union
CP (any)
CP (modern)
CP (traditional)
Unmet need
Total demand
Demand satisfied (any method)
Demand satisfied (modern methods)
0%
20%
40%
60%
80%
100%
1970
1975
1980
1985
1990
1995
2000
2005
2010
2015
2020
2025
2030
DHS
MICS
Other international survey
National survey
PMA
Subpopulation
+: Higher contraceptive use
−: Lower contraceptive use
A: Other age group
F: Folk methods included
S−: Sterilization included
S+: Sterilization excluded
Married women
Sexually active women
Ever married/All women
Both sexes and husband/wives

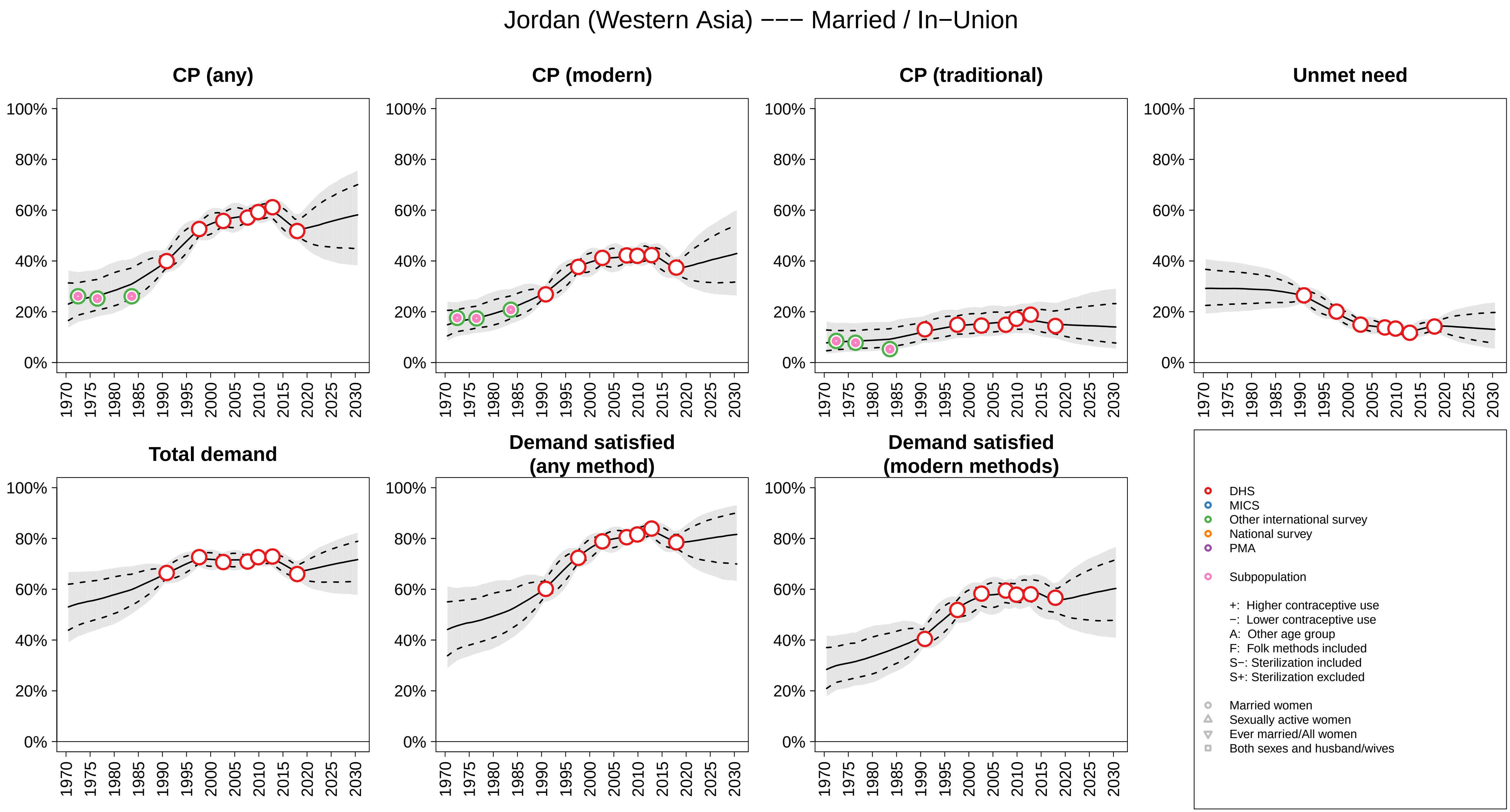
Jordan (Western Asia) --- Married / In−Union
CP (any)
CP (modern)
CP (traditional)
Unmet need
Total demand
Demand satisfied (any method)
Demand satisfied (modern methods)
100%
80%
60%
40%
20%
0%
1970 1975 1980 1985 1990 1995 2000 2005 2010 2015 2020 2025 2030
DHS
MICS
Other international survey
National survey
PMA
Subpopulation
+: Higher contraceptive use
−: Lower contraceptive use
A: Other age group
F: Folk methods included
S−: Sterilization included
S+: Sterilization excluded
Married women
Sexually active women
Ever married/All women
Both sexes and husband/wives

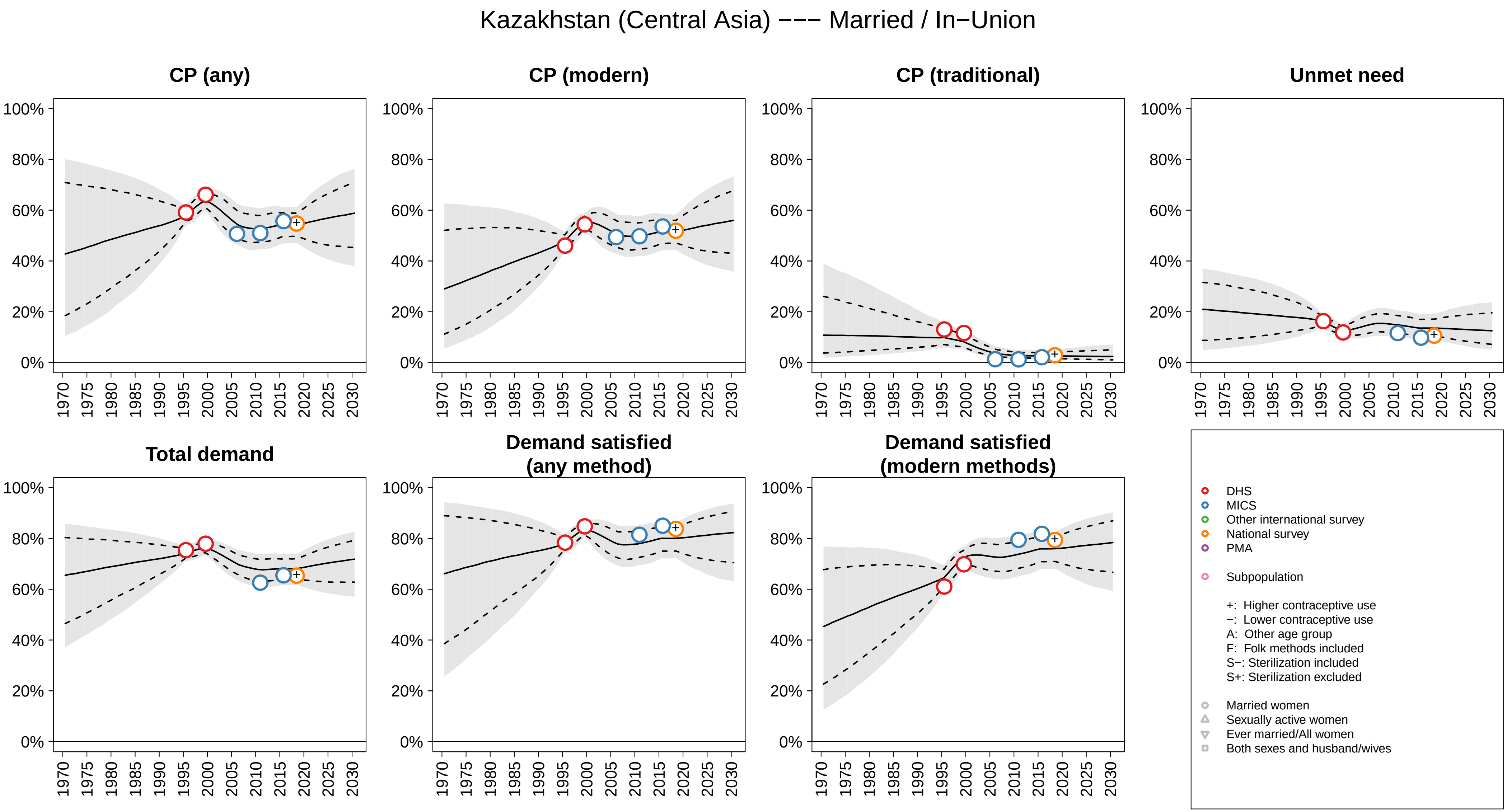

Kazakhstan (Central Asia) −−− Married / In−Union
CP (any)
CP (modern)
CP (traditional)
Unmet need
Total demand
Demand satisfied (any method)
Demand satisfied (modern methods)
100%
80%
60%
40%
20%
0%
1970
1975
1980
1985
1990
1995
2000
2005
2010
2015
2020
2025
2030
DHS
MICS
Other international survey
National survey
PMA
Subpopulation
+: Higher contraceptive use
−: Lower contraceptive use
A: Other age group
F: Folk methods included
S−: Sterilization included
S+: Sterilization excluded
Married women
Sexually active women
Ever married/All women
Both sexes and husband/wives

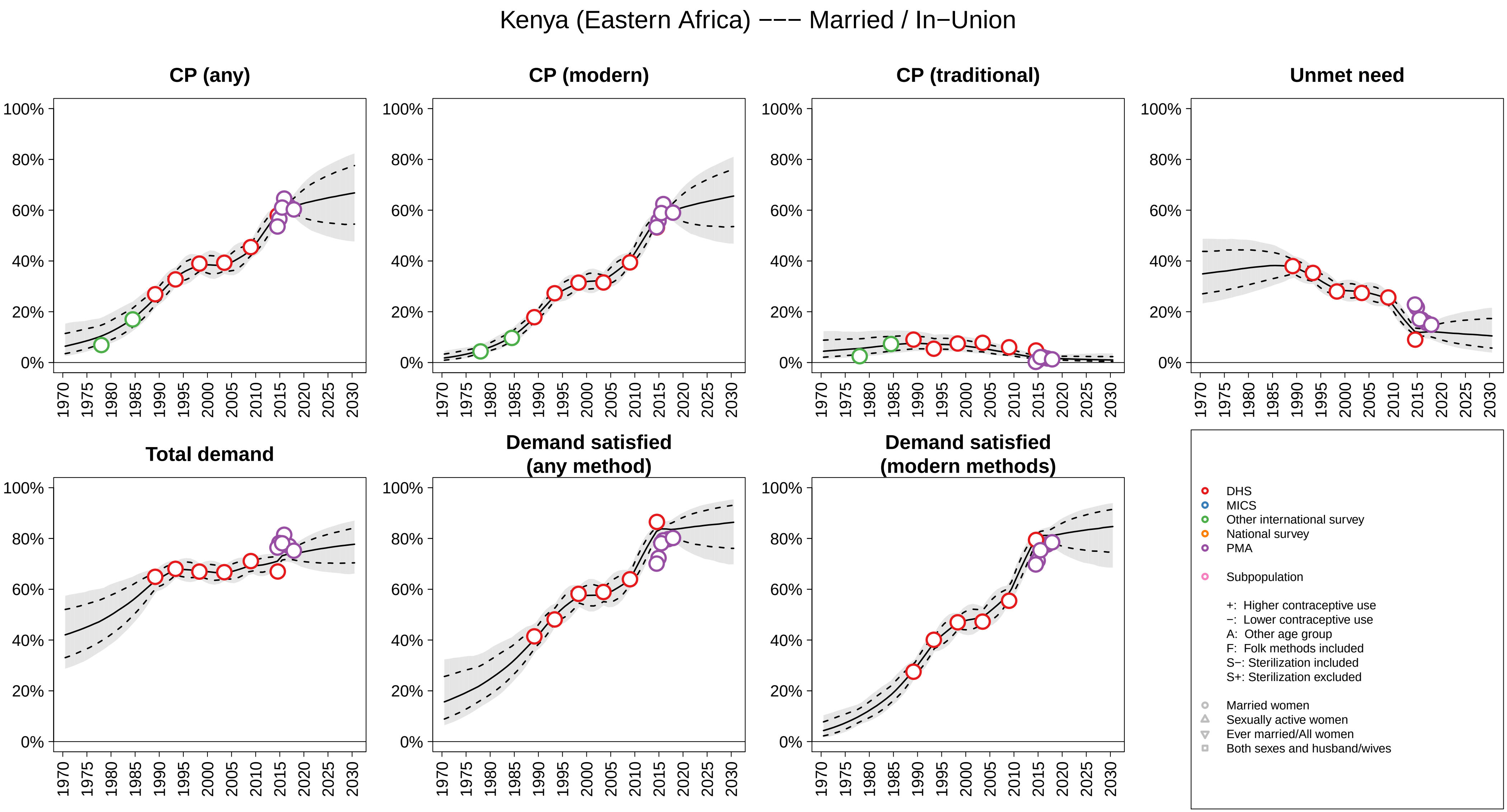
Kenya (Eastern Africa) --- Married / In−Union
CP (any)
CP (modern)
CP (traditional)
Unmet need
Total demand
Demand satisfied (any method)
Demand satisfied (modern methods)
0%
20%
40%
60%
80%
100%
1970
1975
1980
1985
1990
1995
2000
2005
2010
2015
2020
2025
2030
DHS
MICS
Other international survey
National survey
PMA
Subpopulation
+: Higher contraceptive use
−: Lower contraceptive use
A: Other age group
F: Folk methods included
S−: Sterilization included
S+: Sterilization excluded
Married women
Sexually active women
Ever married/All women
Both sexes and husband/wives

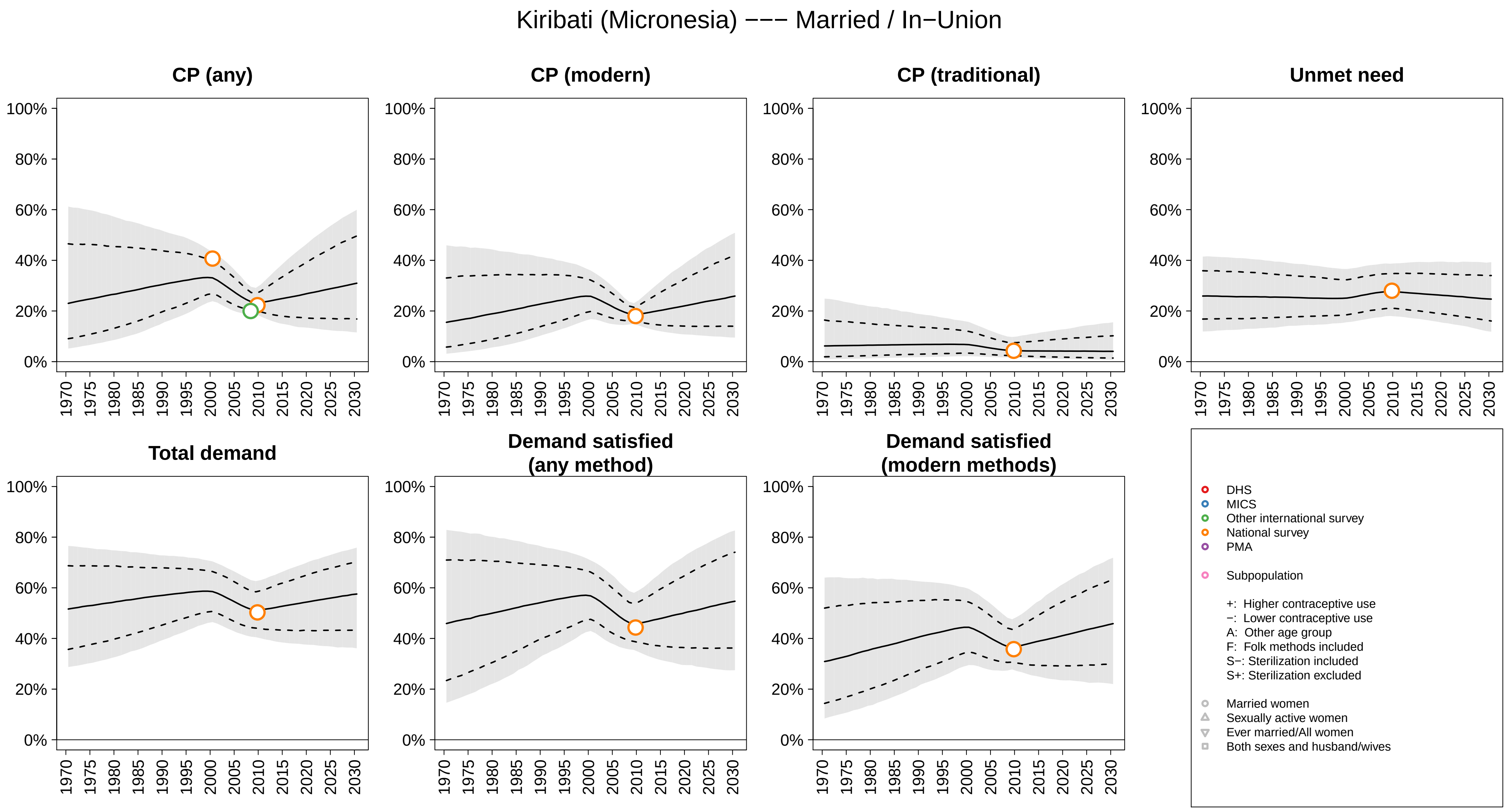
Kiribati (Micronesia) −−− Married / In−Union
CP (any)
CP (modern)
CP (traditional)
Unmet need
Total demand
Demand satisfied (any method)
Demand satisfied (modern methods)
0%
20%
40%
60%
80%
100%
1970
1975
1980
1985
1990
1995
2000
2005
2010
2015
2020
2025
2030
DHS
MICS
Other international survey
National survey
PMA
Subpopulation
+: Higher contraceptive use
−: Lower contraceptive use
A: Other age group
F: Folk methods included
S−: Sterilization included
S+: Sterilization excluded
Married women
Sexually active women
Ever married/All women
Both sexes and husband/wives

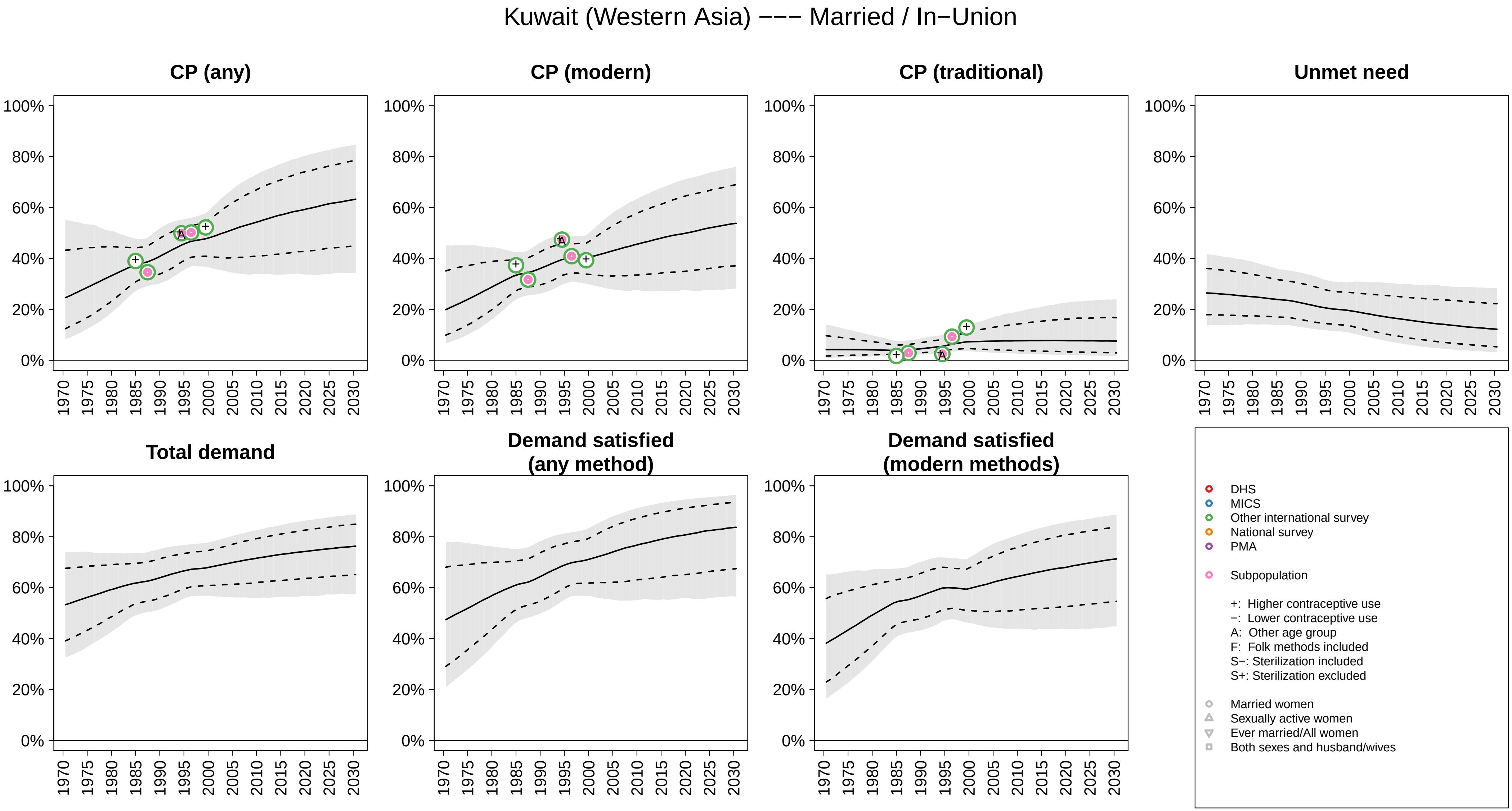

Kuwait (Western Asia) −−− Married / In−Union
CP (any)
CP (modern)
CP (traditional)
Unmet need
Total demand
Demand satisfied (any method)
Demand satisfied (modern methods)
0%
20%
40%
60%
80%
100%
1970
1975
1980
1985
1990
1995
2000
2005
2010
2015
2020
2025
2030
DHS
MICS
Other international survey
National survey
PMA
Subpopulation
+: Higher contraceptive use
−: Lower contraceptive use
A: Other age group
F: Folk methods included
S−: Sterilization included
S+: Sterilization excluded
Married women
Sexually active women
Ever married/All women
Both sexes and husband/wives

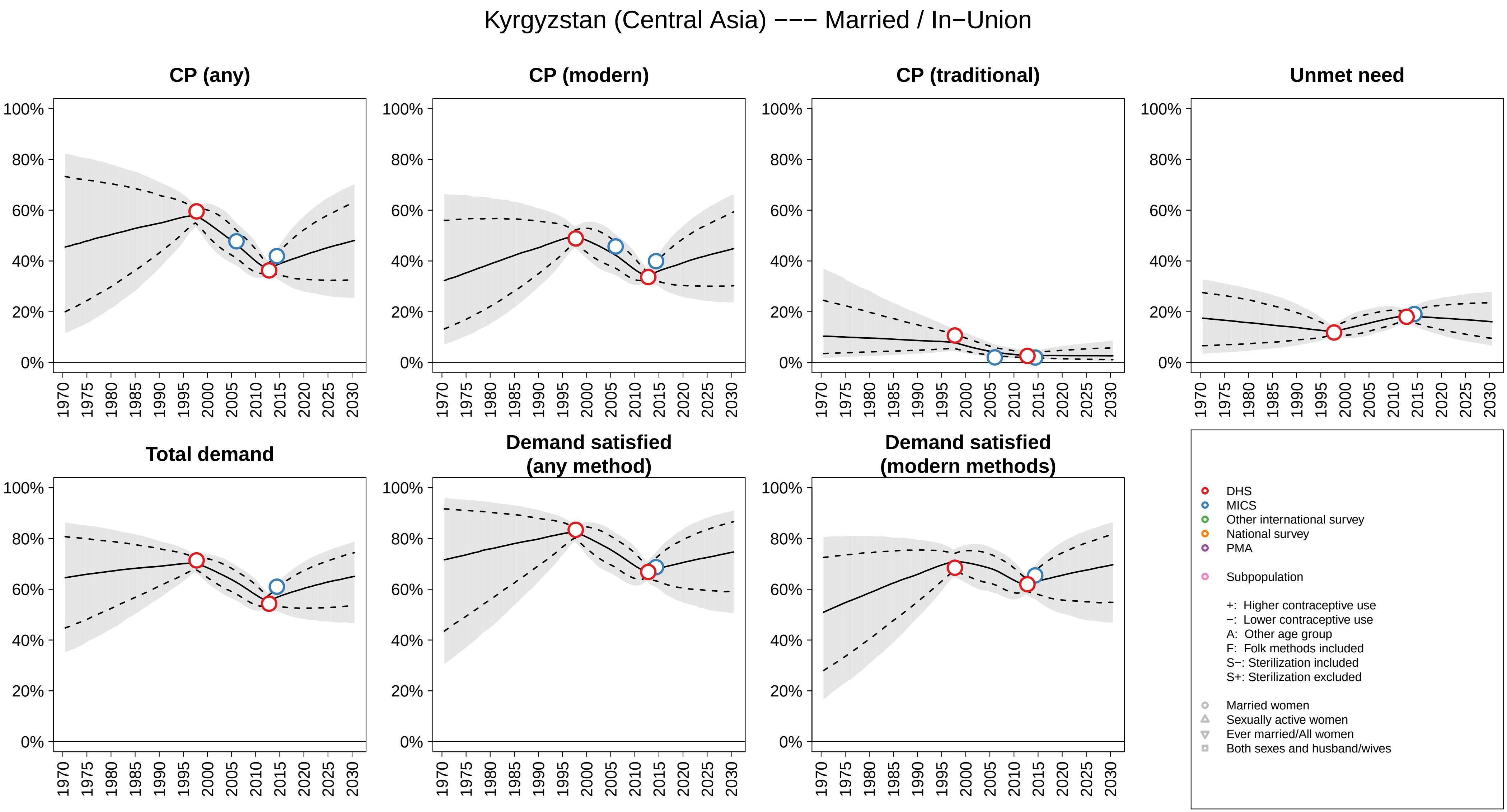
Kyrgyzstan (Central Asia) −−− Married / In−Union
CP (any)
CP (modern)
CP (traditional)
Unmet need
Total demand
Demand satisfied (any method)
Demand satisfied (modern methods)
0%
20%
40%
60%
80%
100%
1970
1975
1980
1985
1990
1995
2000
2005
2010
2015
2020
2025
2030
DHS
MICS
Other international survey
National survey
PMA
Subpopulation
+: Higher contraceptive use
−: Lower contraceptive use
A: Other age group
F: Folk methods included
S−: Sterilization included
S+: Sterilization excluded
Married women
Sexually active women
Ever married/All women
Both sexes and husband/wives

Lao People's Dem. Republic (South−eastern Asia) −−− Married / In−Union

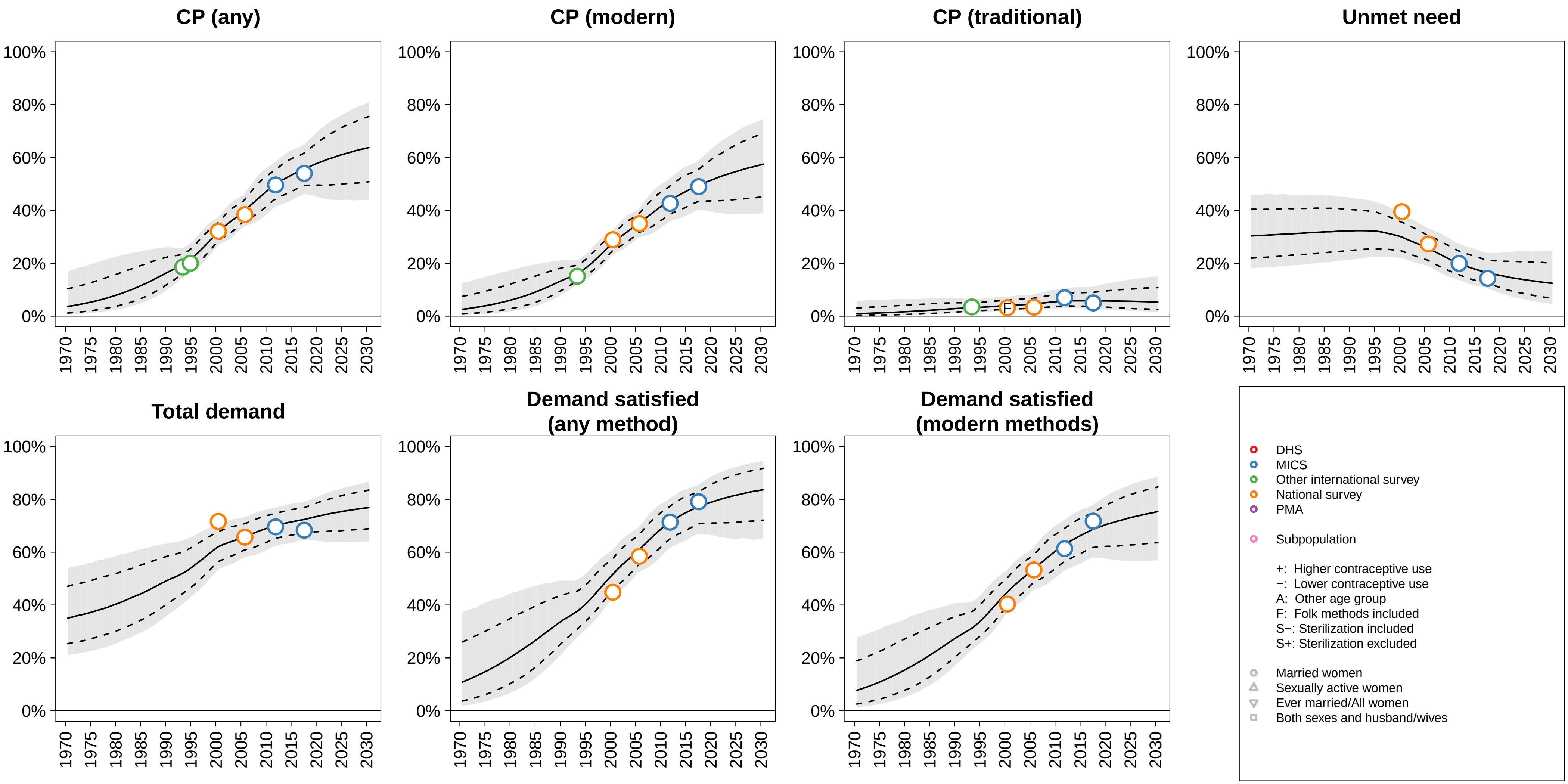

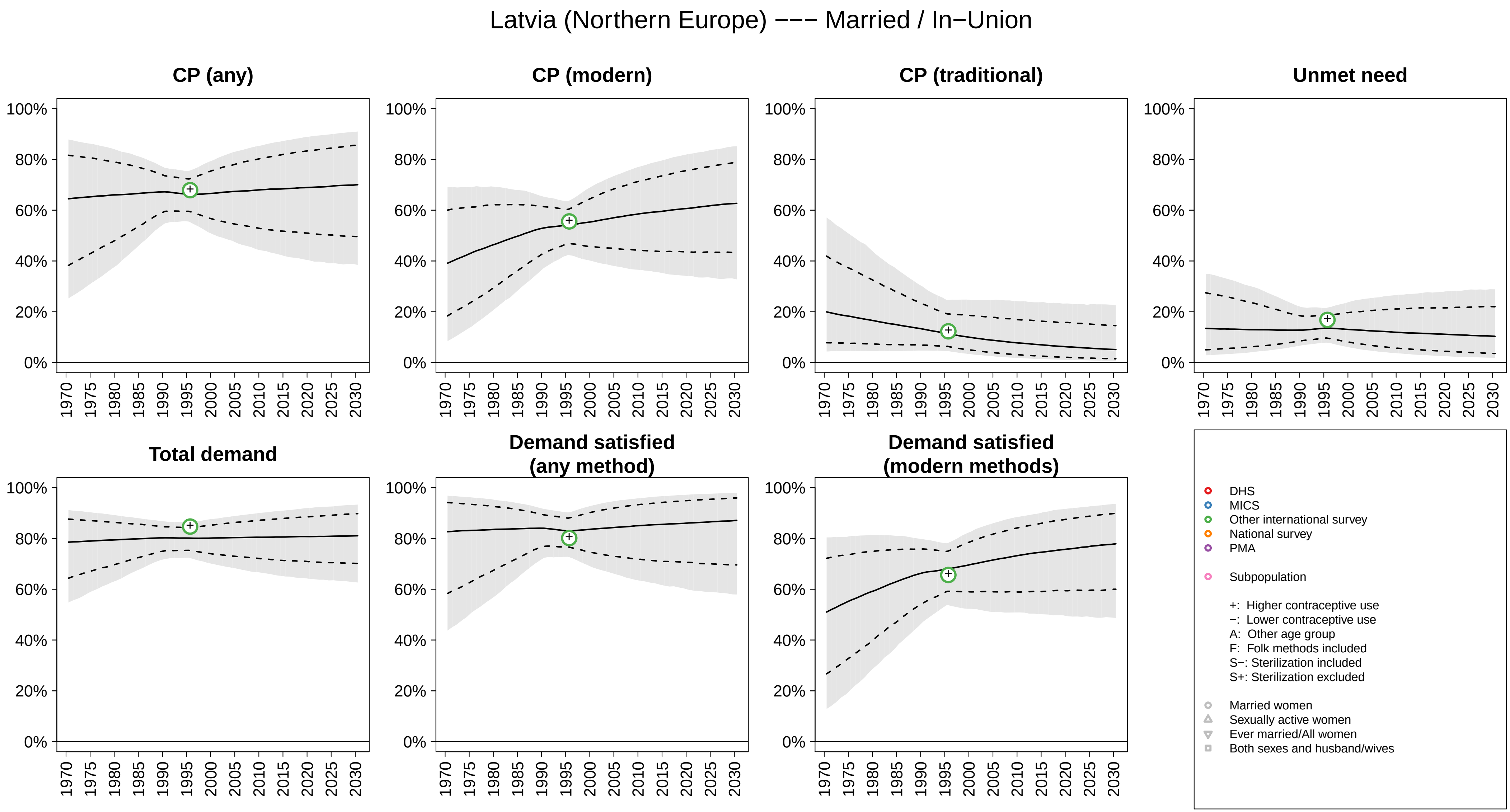
Latvia (Northern Europe) −−− Married / In−Union
CP (any)
CP (modern)
CP (traditional)
Unmet need
Total demand
Demand satisfied (any method)
Demand satisfied (modern methods)
100%
80%
60%
40%
20%
0%
1970
1975
1980
1985
1990
1995
2000
2005
2010
2015
2020
2025
2030
DHS
MICS
Other international survey
National survey
PMA
Subpopulation
+: Higher contraceptive use
−: Lower contraceptive use
A: Other age group
F: Folk methods included
S−: Sterilization included
S+: Sterilization excluded
Married women
Sexually active women
Ever married/All women
Both sexes and husband/wives

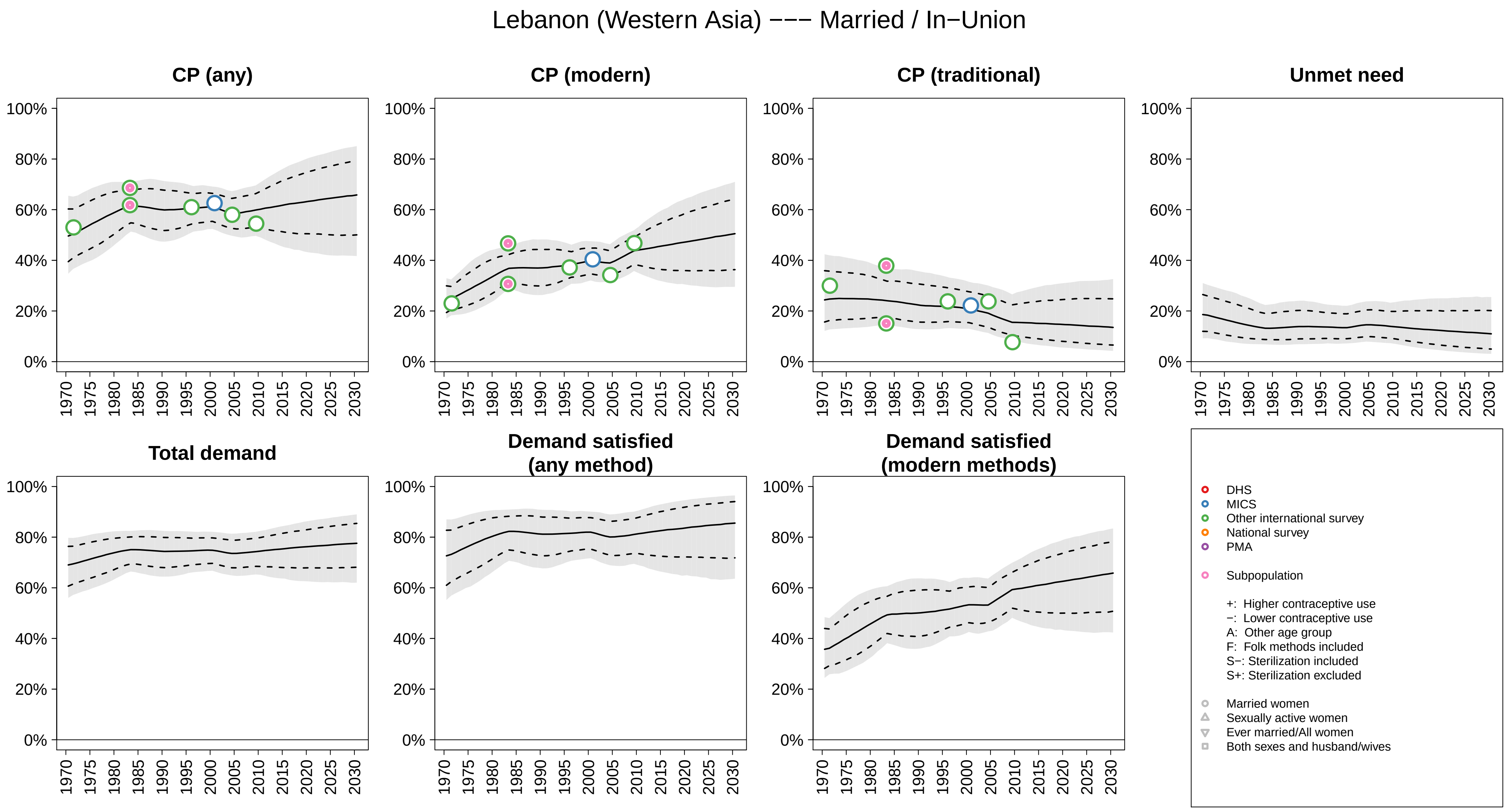
Lebanon (Western Asia) −−− Married / In−Union
CP (any)
CP (modern)
CP (traditional)
Unmet need
Total demand
Demand satisfied (any method)
Demand satisfied (modern methods)
0%
20%
40%
60%
80%
100%
1970
1975
1980
1985
1990
1995
2000
2005
2010
2015
2020
2025
2030
DHS
MICS
Other international survey
National survey
PMA
Subpopulation
+: Higher contraceptive use
−: Lower contraceptive use
A: Other age group
F: Folk methods included
S−: Sterilization included
S+: Sterilization excluded
Married women
Sexually active women
Ever married/All women
Both sexes and husband/wives

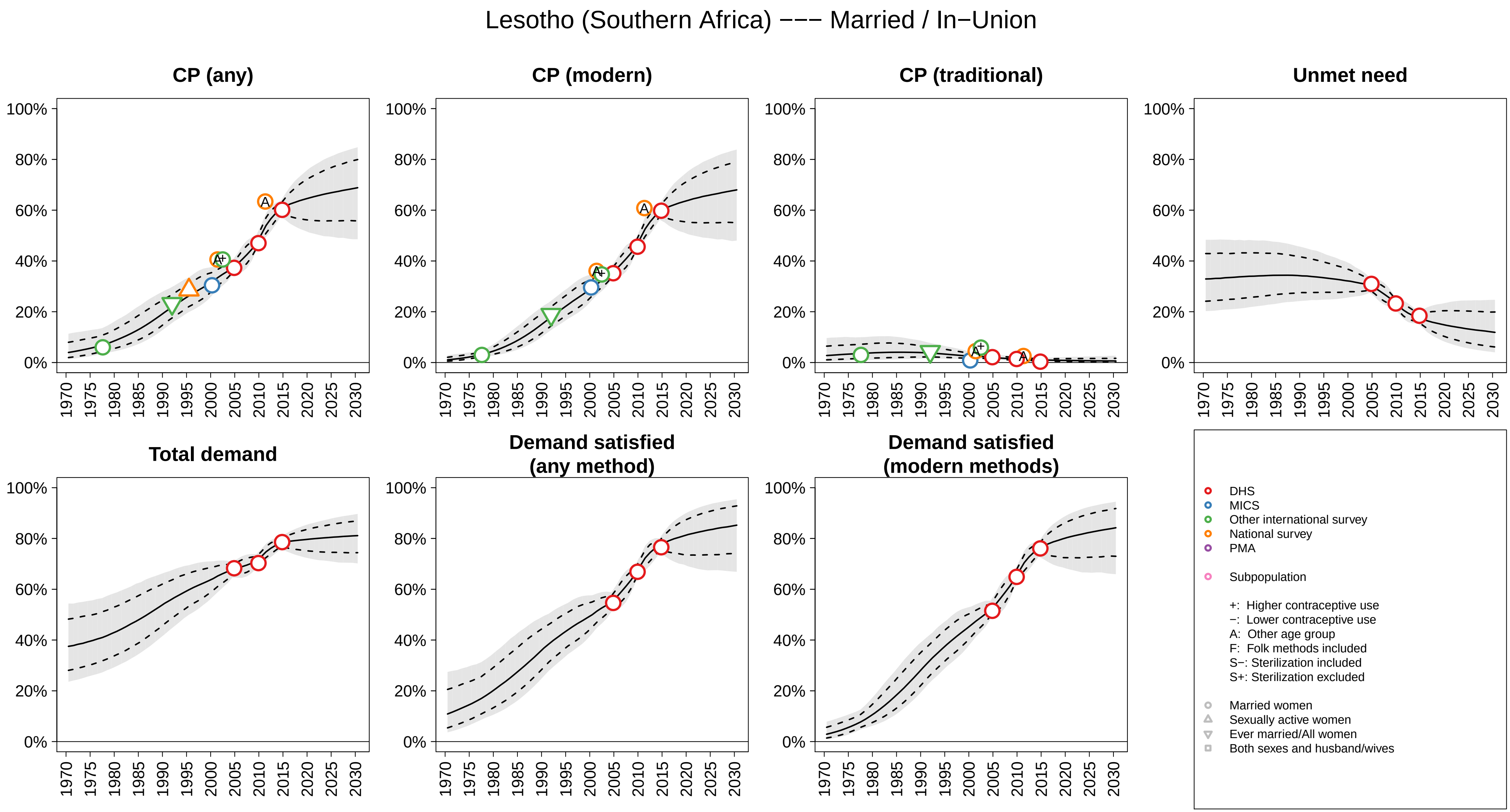
Lesotho (Southern Africa) −−− Married / In−Union
CP (any)
CP (modern)
CP (traditional)
Unmet need
Total demand
Demand satisfied (any method)
Demand satisfied (modern methods)
100%
80%
60%
40%
20%
0%
1970 1975 1980 1985 1990 1995 2000 2005 2010 2015 2020 2025 2030
DHS
MICS
Other international survey
National survey
PMA
Subpopulation
+: Higher contraceptive use
−: Lower contraceptive use
A: Other age group
F: Folk methods included
S−: Sterilization included
S+: Sterilization excluded
Married women
Sexually active women
Ever married/All women
Both sexes and husband/wives

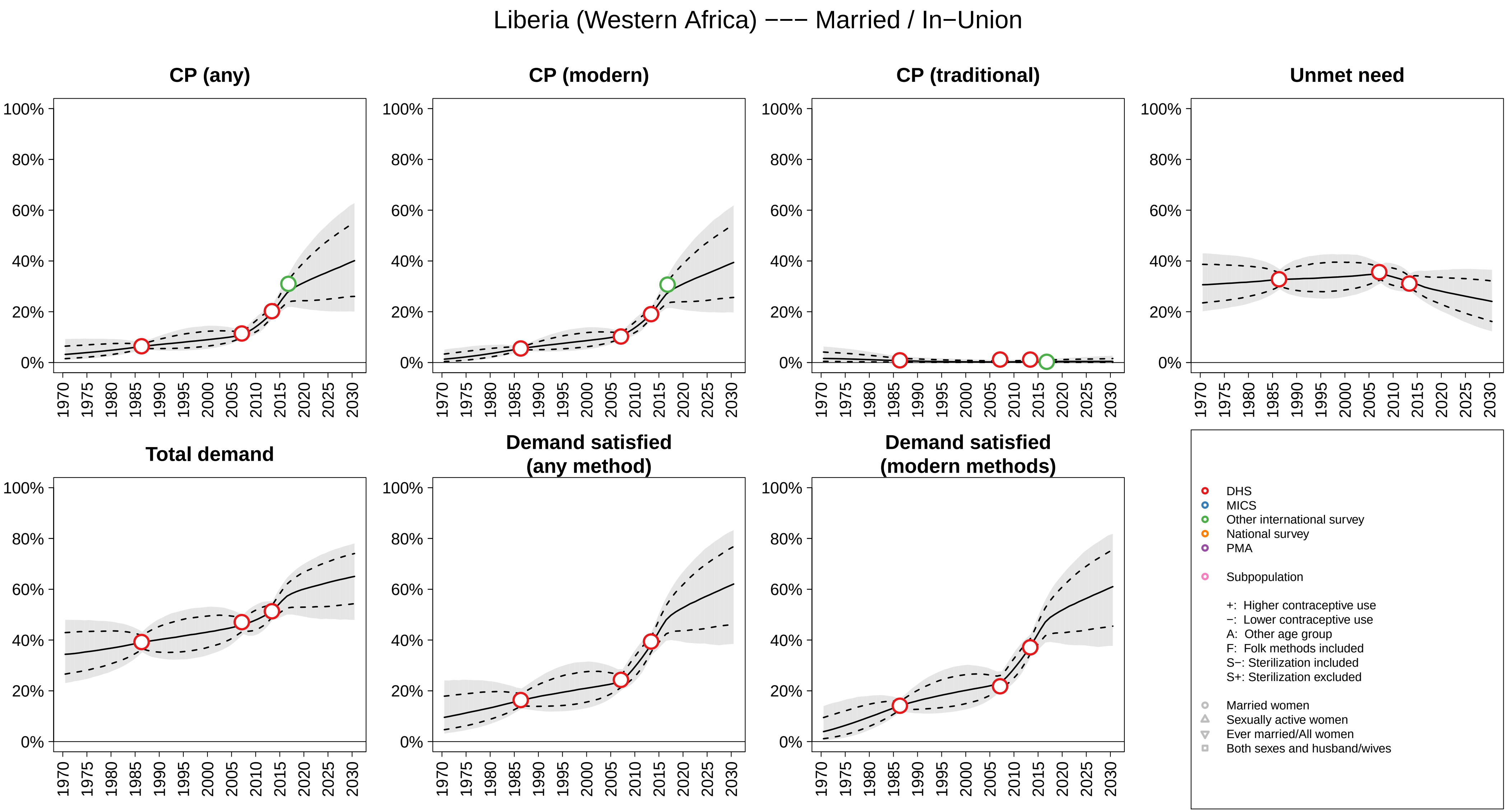
Liberia (Western Africa) −−− Married / In−Union
CP (any)
CP (modern)
CP (traditional)
Unmet need
Total demand
Demand satisfied (any method)
Demand satisfied (modern methods)
0%
20%
40%
60%
80%
100%
1970
1975
1980
1985
1990
1995
2000
2005
2010
2015
2020
2025
2030
DHS
MICS
Other international survey
National survey
PMA
Subpopulation
+: Higher contraceptive use
−: Lower contraceptive use
A: Other age group
F: Folk methods included
S−: Sterilization included
S+: Sterilization excluded
Married women
Sexually active women
Ever married/All women
Both sexes and husband/wives

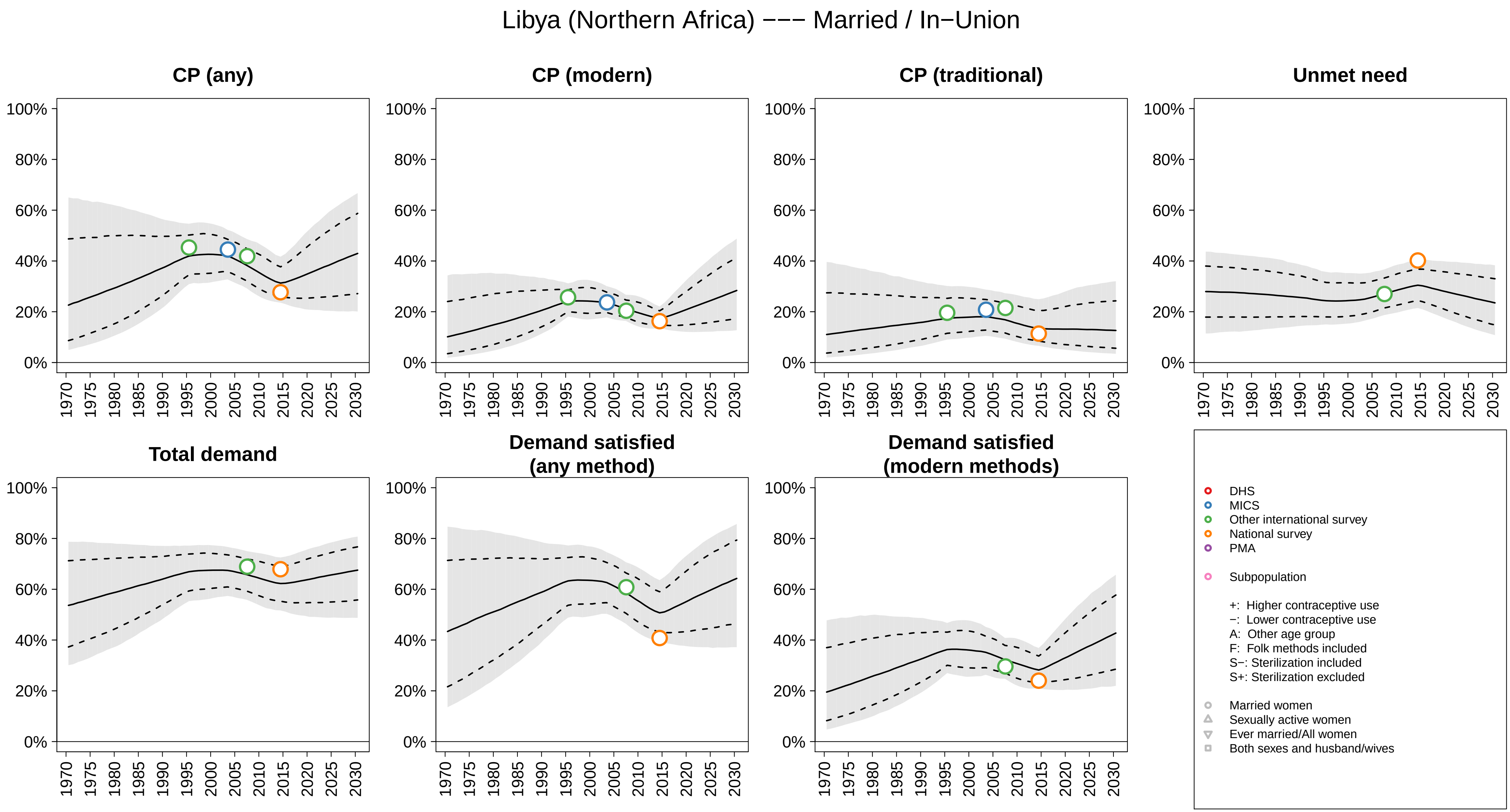
Libya (Northern Africa) −−− Married / In−Union
CP (any)
CP (modern)
CP (traditional)
Unmet need
Total demand
Demand satisfied (any method)
Demand satisfied (modern methods)
0%
20%
40%
60%
80%
100%
1970
1975
1980
1985
1990
1995
2000
2005
2010
2015
2020
2025
2030
DHS
MICS
Other international survey
National survey
PMA
Subpopulation
+: Higher contraceptive use
−: Lower contraceptive use
A: Other age group
F: Folk methods included
S−: Sterilization included
S+: Sterilization excluded
Married women
Sexually active women
Ever married/All women
Both sexes and husband/wives

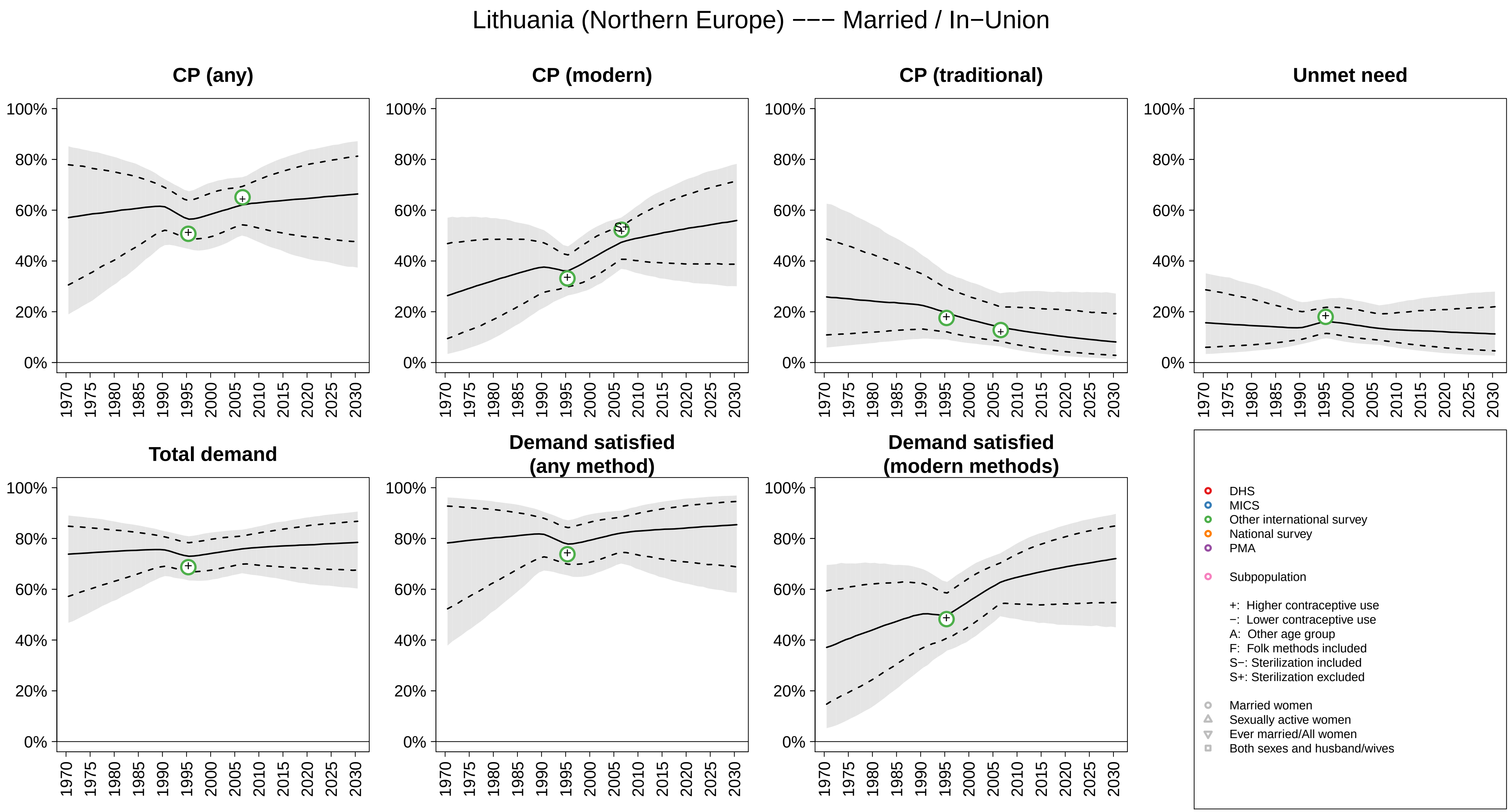
Lithuania (Northern Europe) −−− Married / In−Union
CP (any)
CP (modern)
CP (traditional)
Unmet need
Total demand
Demand satisfied (any method)
Demand satisfied (modern methods)
100%
80%
60%
40%
20%
0%
1970 1975 1980 1985 1990 1995 2000 2005 2010 2015 2020 2025 2030
S+
DHS
MICS
Other international survey
National survey
PMA
Subpopulation
+: Higher contraceptive use
−: Lower contraceptive use
A: Other age group
F: Folk methods included
S−: Sterilization included
S+: Sterilization excluded
Married women
Sexually active women
Ever married/All women
Both sexes and husband/wives

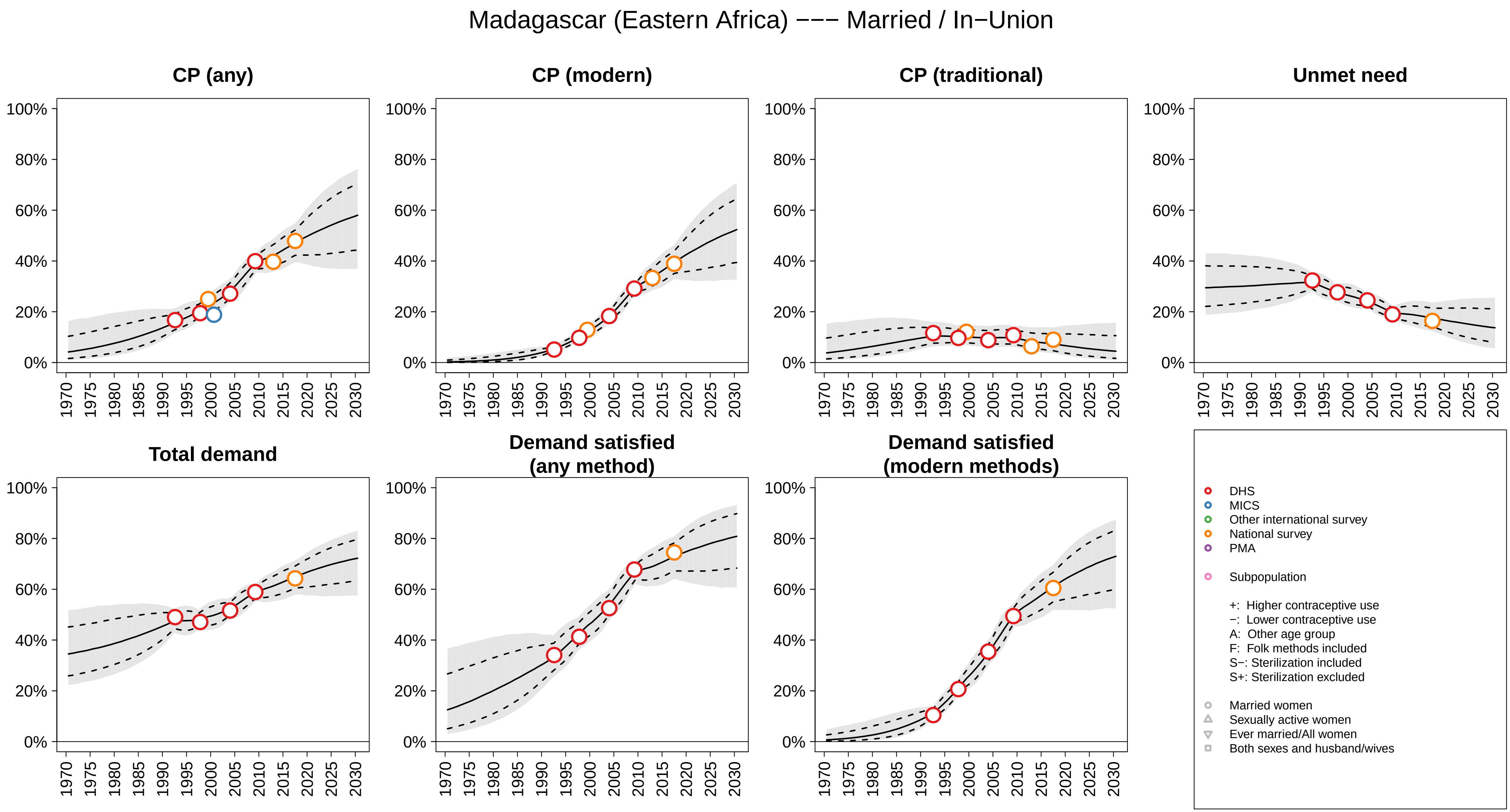
Madagascar (Eastern Africa) --- Married / In−Union
CP (any)
CP (modern)
CP (traditional)
Unmet need
Total demand
Demand satisfied (any method)
Demand satisfied (modern methods)
0%
20%
40%
60%
80%
100%
1970
1975
1980
1985
1990
1995
2000
2005
2010
2015
2020
2025
2030
DHS
MICS
Other international survey
National survey
PMA
Subpopulation
+: Higher contraceptive use
−: Lower contraceptive use
A: Other age group
F: Folk methods included
S−: Sterilization included
S+: Sterilization excluded
Married women
Sexually active women
Ever married/All women
Both sexes and husband/wives

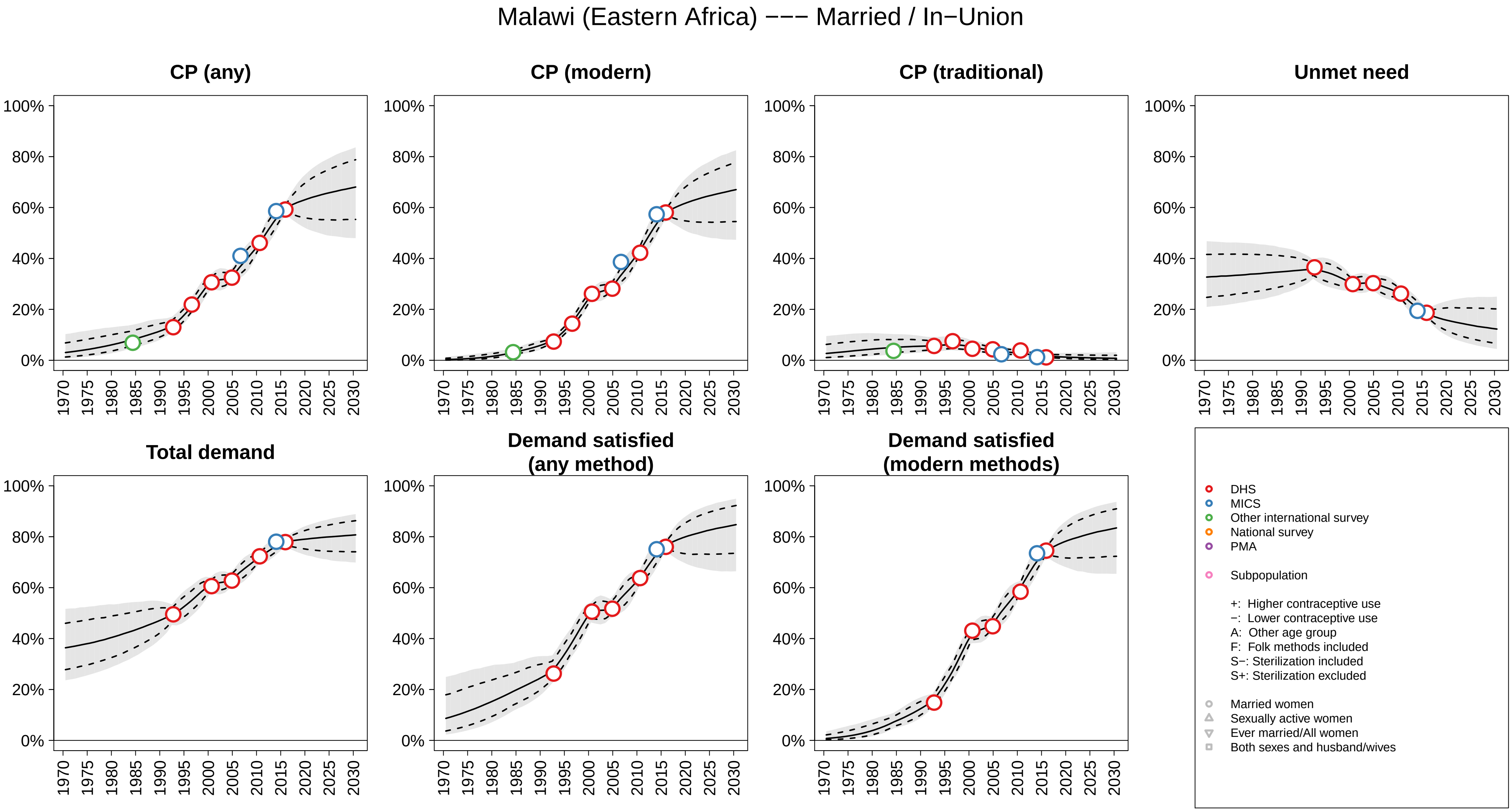
Malawi (Eastern Africa) −−− Married / In−Union
CP (any)
CP (modern)
CP (traditional)
Unmet need
Total demand
Demand satisfied (any method)
Demand satisfied (modern methods)
0%
20%
40%
60%
80%
100%
1970
1975
1980
1985
1990
1995
2000
2005
2010
2015
2020
2025
2030
DHS
MICS
Other international survey
National survey
PMA
Subpopulation
+: Higher contraceptive use
−: Lower contraceptive use
A: Other age group
F: Folk methods included
S−: Sterilization included
S+: Sterilization excluded
Married women
Sexually active women
Ever married/All women
Both sexes and husband/wives

Malaysia (South−eastern Asia) −−− Married / In−Union

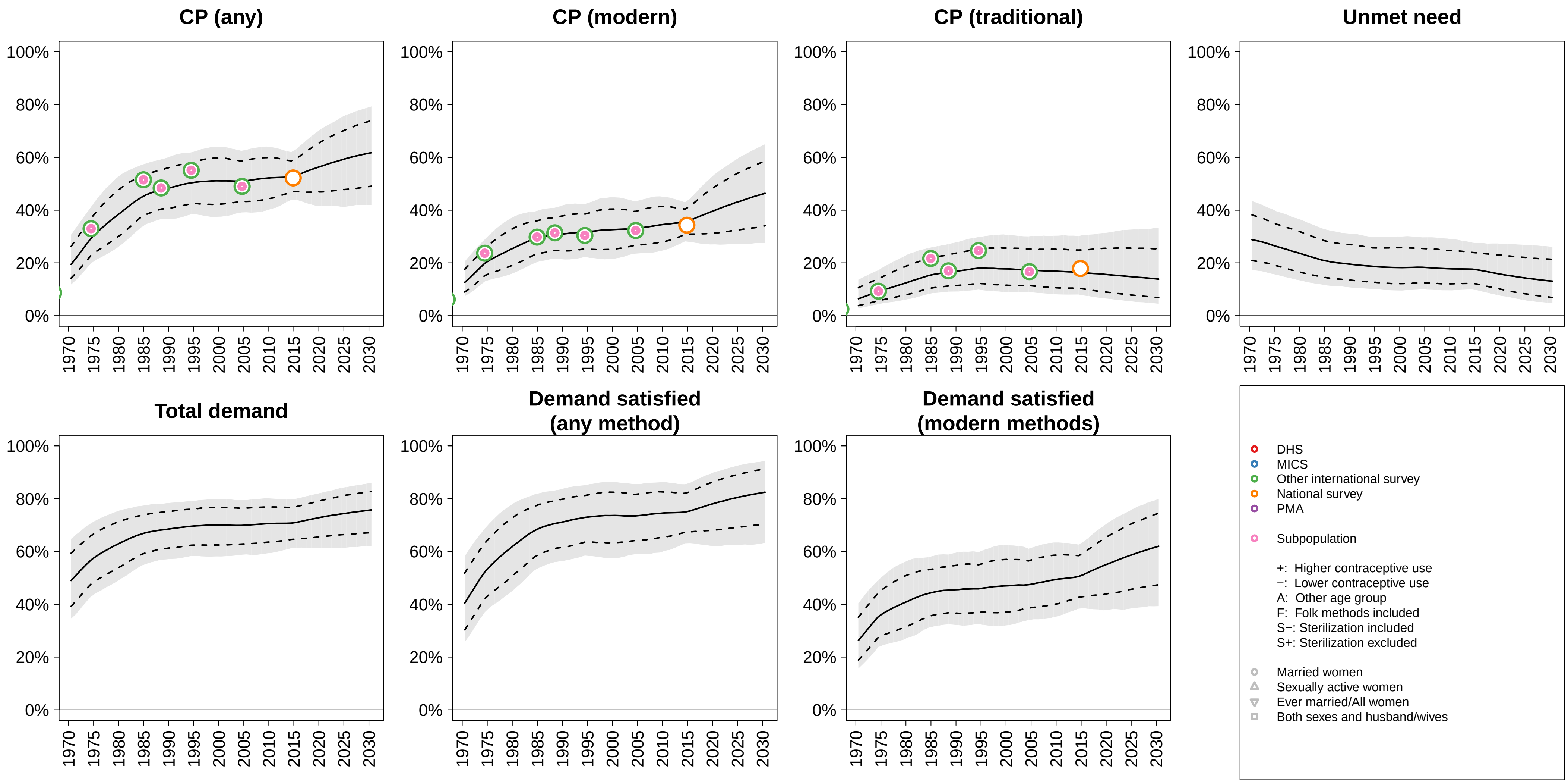

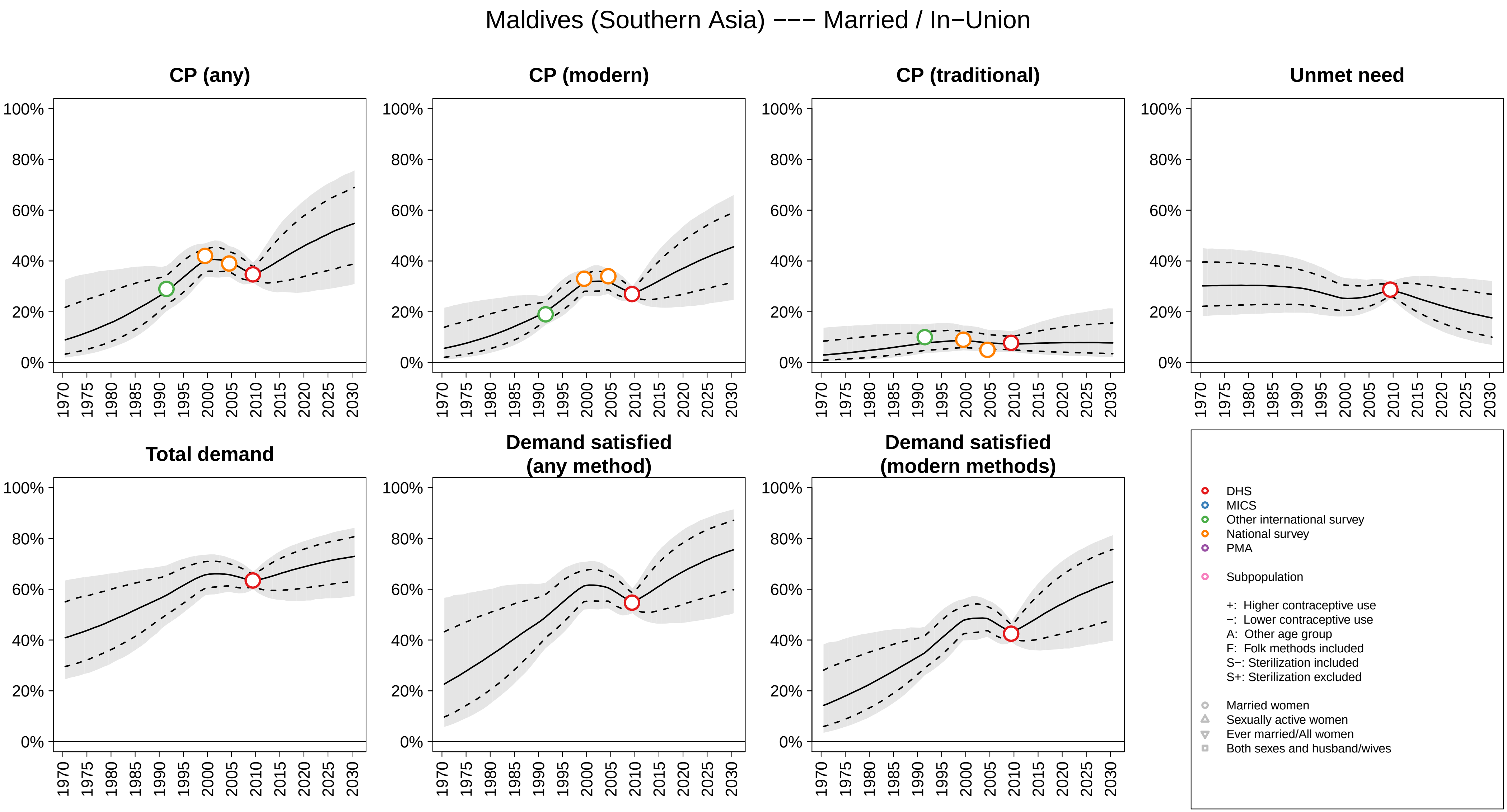
Maldives (Southern Asia) --- Married / In−Union
CP (any)
CP (modern)
CP (traditional)
Unmet need
Total demand
Demand satisfied (any method)
Demand satisfied (modern methods)
100%
80%
60%
40%
20%
0%
1970
1975
1980
1985
1990
1995
2000
2005
2010
2015
2020
2025
2030
DHS
MICS
Other international survey
National survey
PMA
Subpopulation
+: Higher contraceptive use
−: Lower contraceptive use
A: Other age group
F: Folk methods included
S−: Sterilization included
S+: Sterilization excluded
Married women
Sexually active women
Ever married/All women
Both sexes and husband/wives

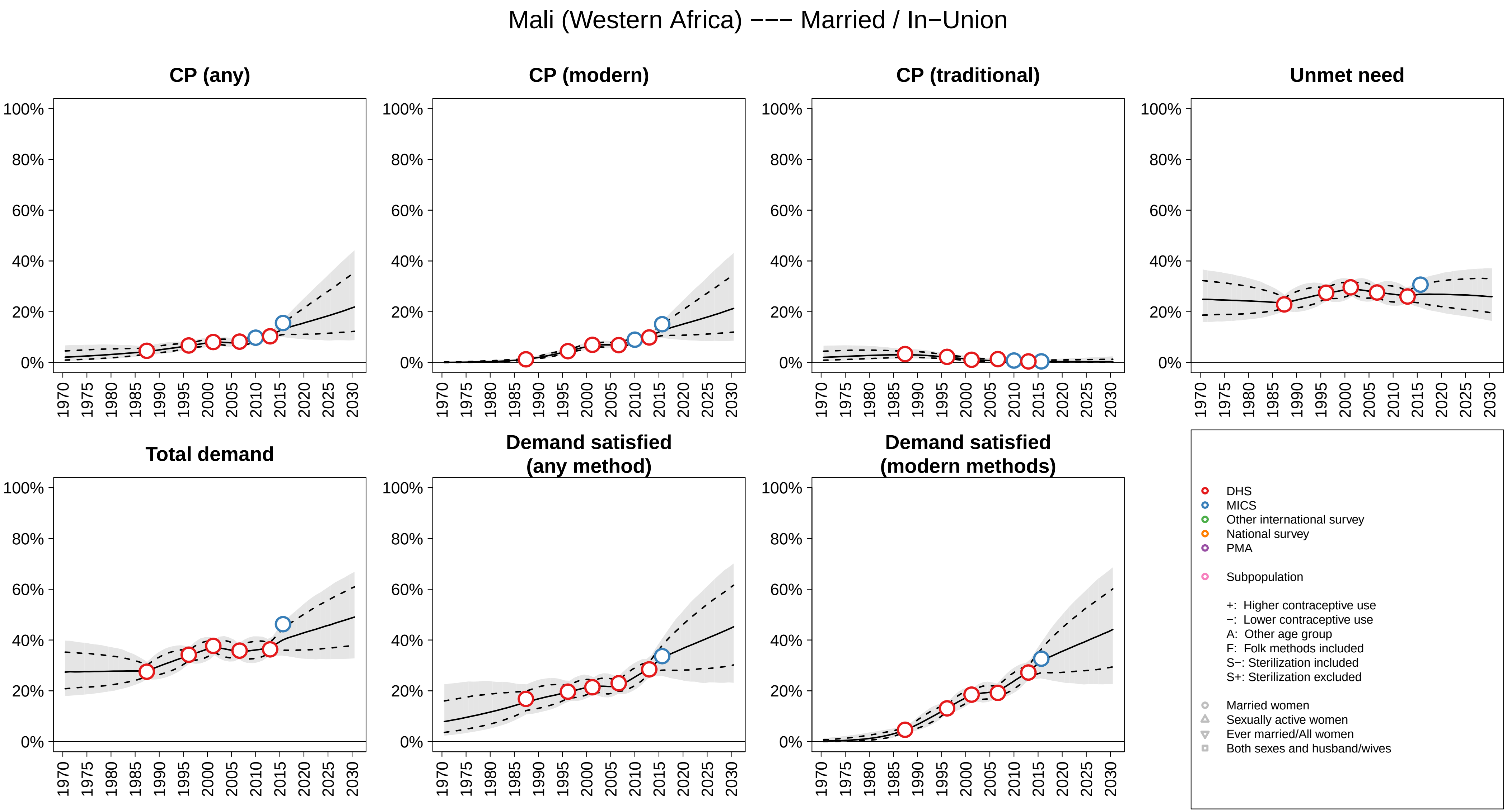

Mali (Western Africa) --- Married / In−Union
CP (any)
CP (modern)
CP (traditional)
Unmet need
Total demand
Demand satisfied (any method)
Demand satisfied (modern methods)
0%
20%
40%
60%
80%
100%
1970
1975
1980
1985
1990
1995
2000
2005
2010
2015
2020
2025
2030
DHS
MICS
Other international survey
National survey
PMA
Subpopulation
+: Higher contraceptive use
−: Lower contraceptive use
A: Other age group
F: Folk methods included
S−: Sterilization included
S+: Sterilization excluded
Married women
Sexually active women
Ever married/All women
Both sexes and husband/wives

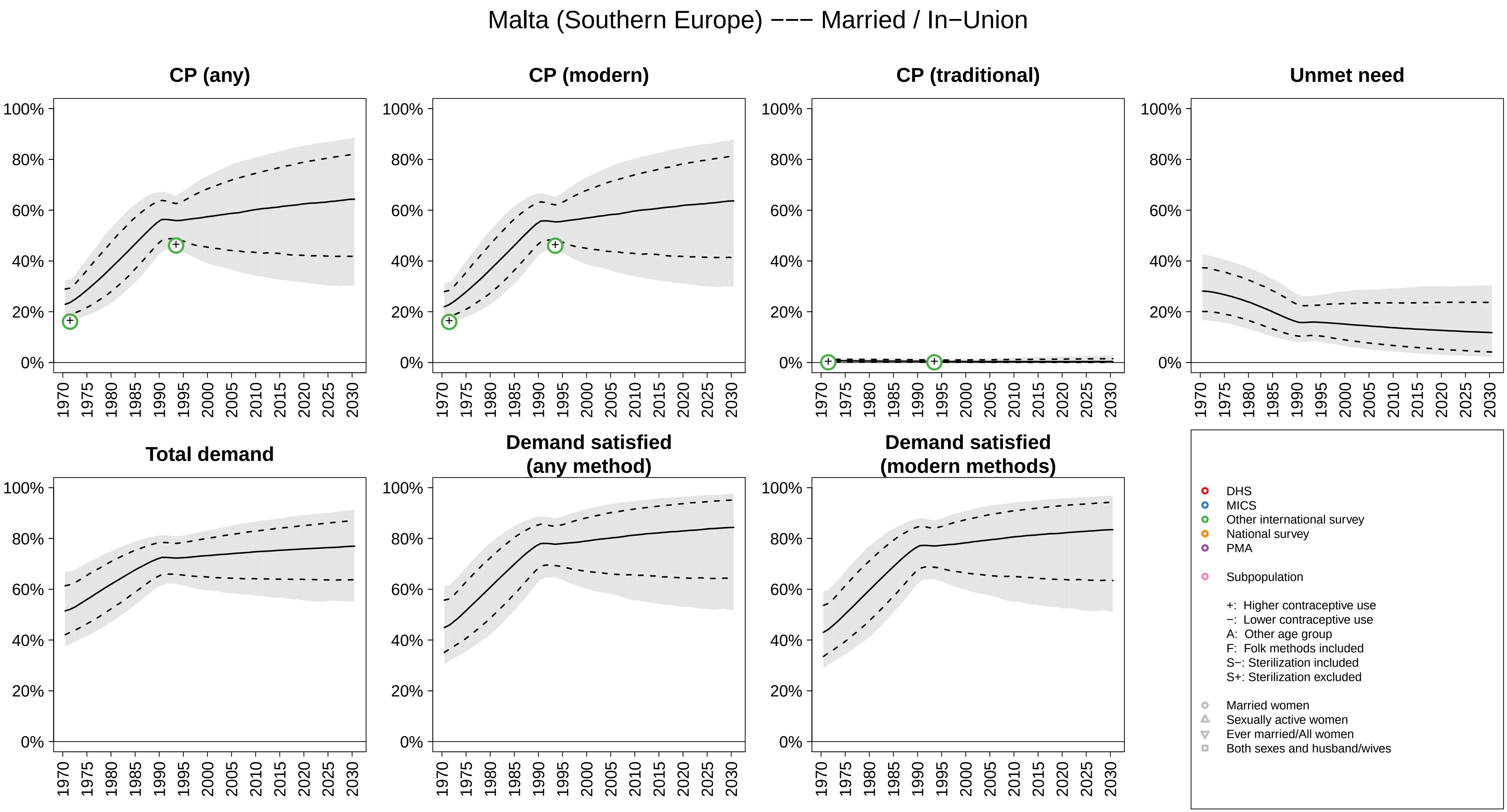

Malta (Southern Europe) --- Married / In−Union
CP (any)
CP (modern)
CP (traditional)
Unmet need
Total demand
Demand satisfied (any method)
Demand satisfied (modern methods)
0%
20%
40%
60%
80%
100%
1970
1975
1980
1985
1990
1995
2000
2005
2010
2015
2020
2025
2030
DHS
MICS
Other international survey
National survey
PMA
Subpopulation
+: Higher contraceptive use
−: Lower contraceptive use
A: Other age group
F: Folk methods included
S−: Sterilization included
S+: Sterilization excluded
Married women
Sexually active women
Ever married/All women
Both sexes and husband/wives

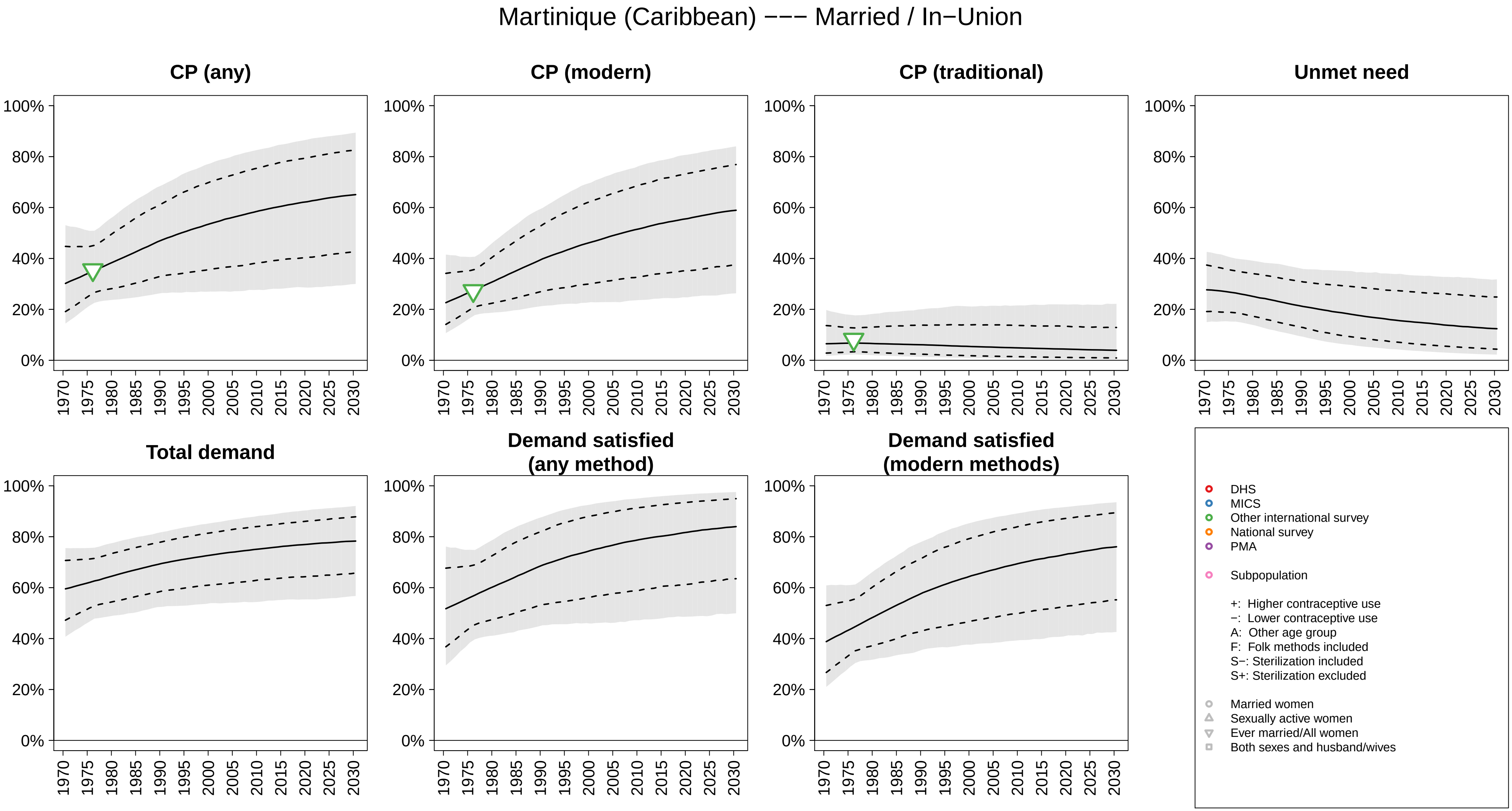

Martinique (Caribbean) −−− Married / In−Union
CP (any)
CP (modern)
CP (traditional)
Unmet need
Total demand
Demand satisfied (any method)
Demand satisfied (modern methods)
0% 20% 40% 60% 80% 100%
1970 1975 1980 1985 1990 1995 2000 2005 2010 2015 2020 2025 2030
DHS
MICS
Other international survey
National survey
PMA
Subpopulation
+: Higher contraceptive use
−: Lower contraceptive use
A: Other age group
F: Folk methods included
S−: Sterilization included
S+: Sterilization excluded
Married women
Sexually active women
Ever married/All women
Both sexes and husband/wives

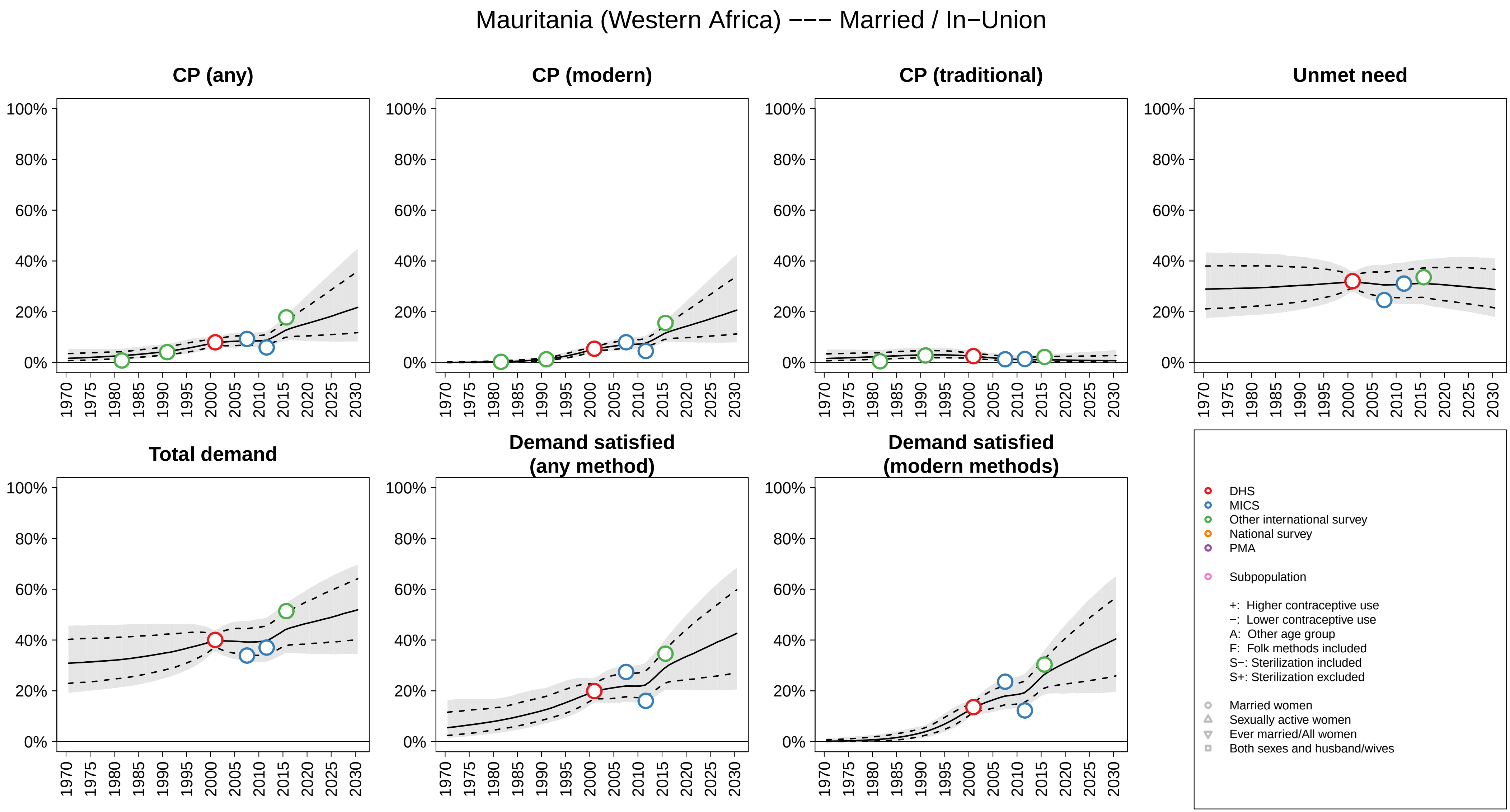
Mauritania (Western Africa) −−− Married / In−Union
CP (any)
CP (modern)
CP (traditional)
Unmet need
Total demand
Demand satisfied (any method)
Demand satisfied (modern methods)
100%
80%
60%
40%
20%
0%
1970
1975
1980
1985
1990
1995
2000
2005
2010
2015
2020
2025
2030
DHS
MICS
Other international survey
National survey
PMA
Subpopulation
+: Higher contraceptive use
−: Lower contraceptive use
A: Other age group
F: Folk methods included
S−: Sterilization included
S+: Sterilization excluded
Married women
Sexually active women
Ever married/All women
Both sexes and husband/wives

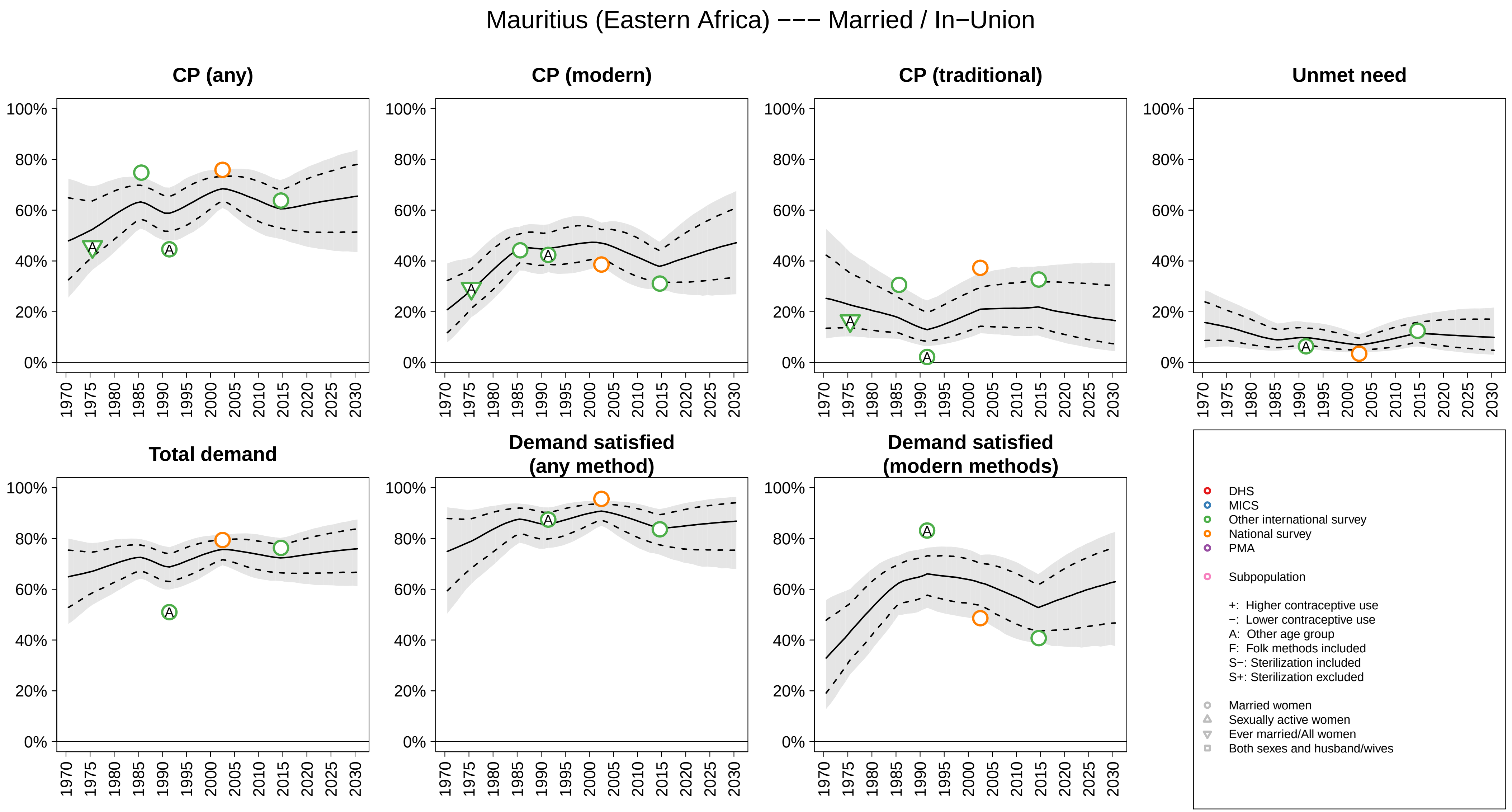
Mauritius (Eastern Africa) −−− Married / In−Union
CP (any)
CP (modern)
CP (traditional)
Unmet need
Total demand
Demand satisfied (any method)
Demand satisfied (modern methods)
0%
20%
40%
60%
80%
100%
1970
1975
1980
1985
1990
1995
2000
2005
2010
2015
2020
2025
2030
DHS
MICS
Other international survey
National survey
PMA
Subpopulation
+: Higher contraceptive use
−: Lower contraceptive use
A: Other age group
F: Folk methods included
S−: Sterilization included
S+: Sterilization excluded
Married women
Sexually active women
Ever married/All women
Both sexes and husband/wives

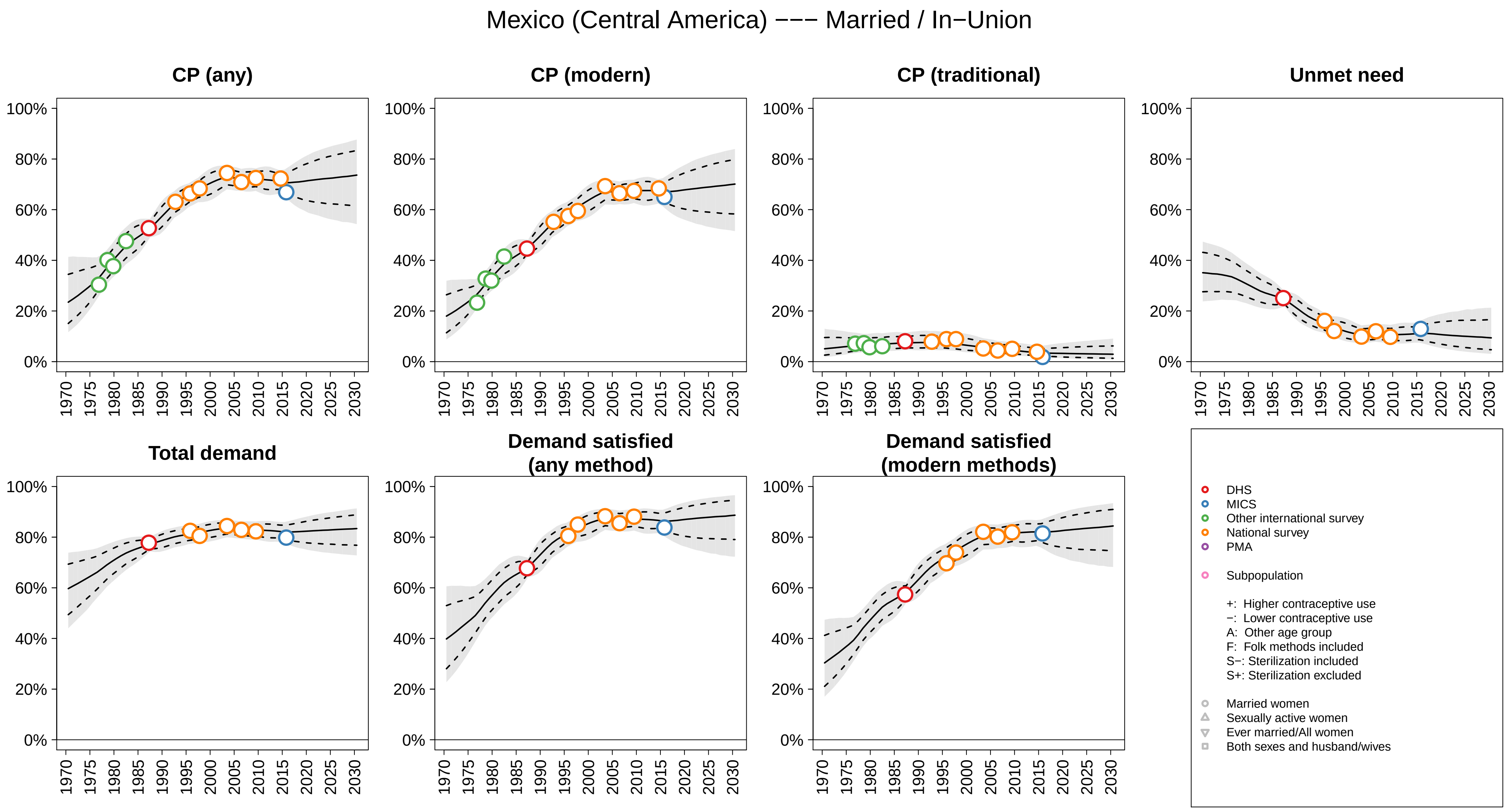
Mexico (Central America) −−− Married / In−Union
CP (any)
CP (modern)
CP (traditional)
Unmet need
Total demand
Demand satisfied (any method)
Demand satisfied (modern methods)
100%
80%
60%
40%
20%
0%
1970
1975
1980
1985
1990
1995
2000
2005
2010
2015
2020
2025
2030
DHS
MICS
Other international survey
National survey
PMA
Subpopulation
+: Higher contraceptive use
−: Lower contraceptive use
A: Other age group
F: Folk methods included
S−: Sterilization included
S+: Sterilization excluded
Married women
Sexually active women
Ever married/All women
Both sexes and husband/wives

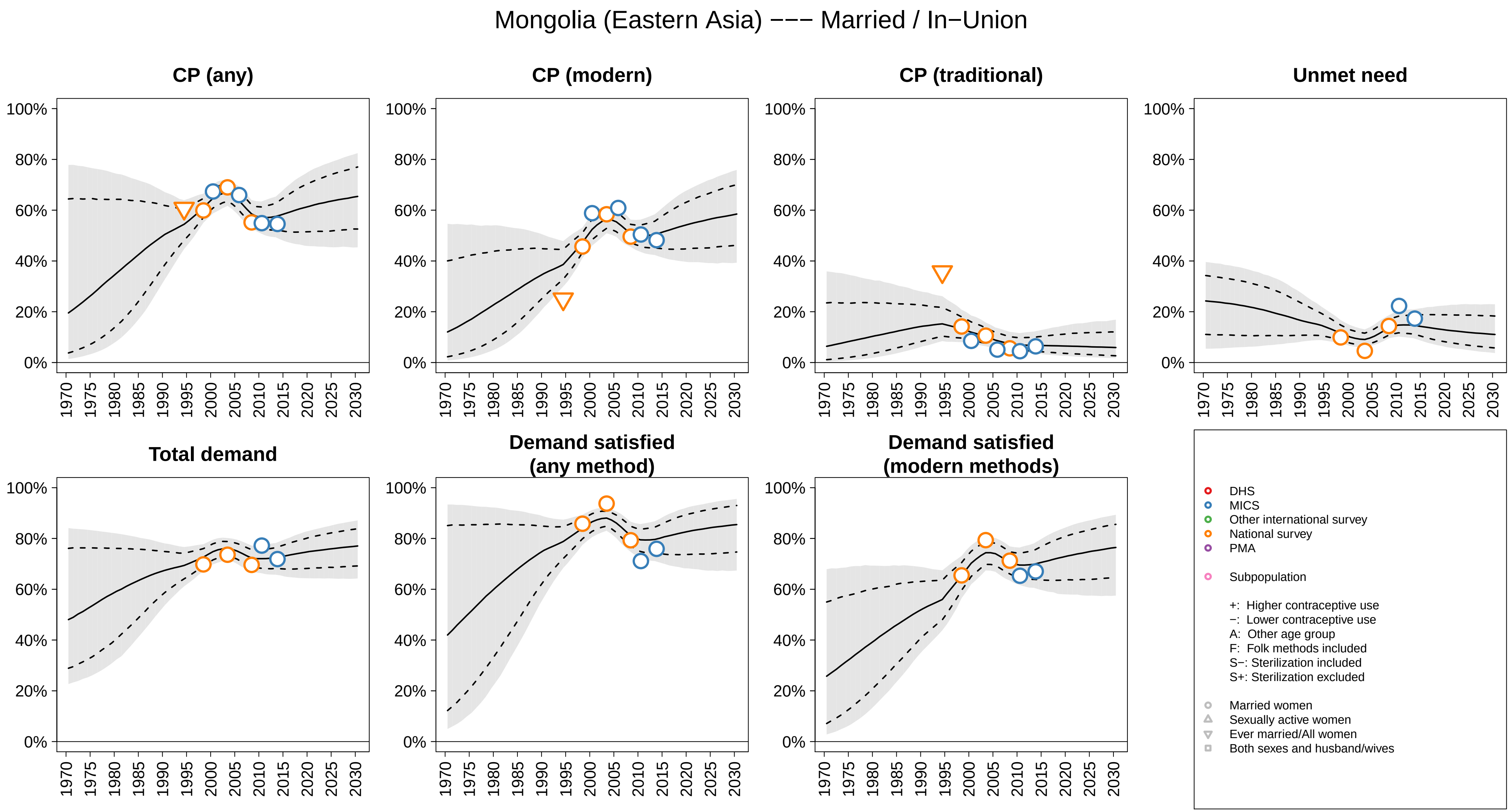
Mongolia (Eastern Asia) --- Married / In−Union
CP (any)
CP (modern)
CP (traditional)
Unmet need
Total demand
Demand satisfied (any method)
Demand satisfied (modern methods)
DHS
MICS
Other international survey
National survey
PMA
Subpopulation
+: Higher contraceptive use
−: Lower contraceptive use
A: Other age group
F: Folk methods included
S−: Sterilization included
S+: Sterilization excluded
Married women
Sexually active women
Ever married/All women
Both sexes and husband/wives

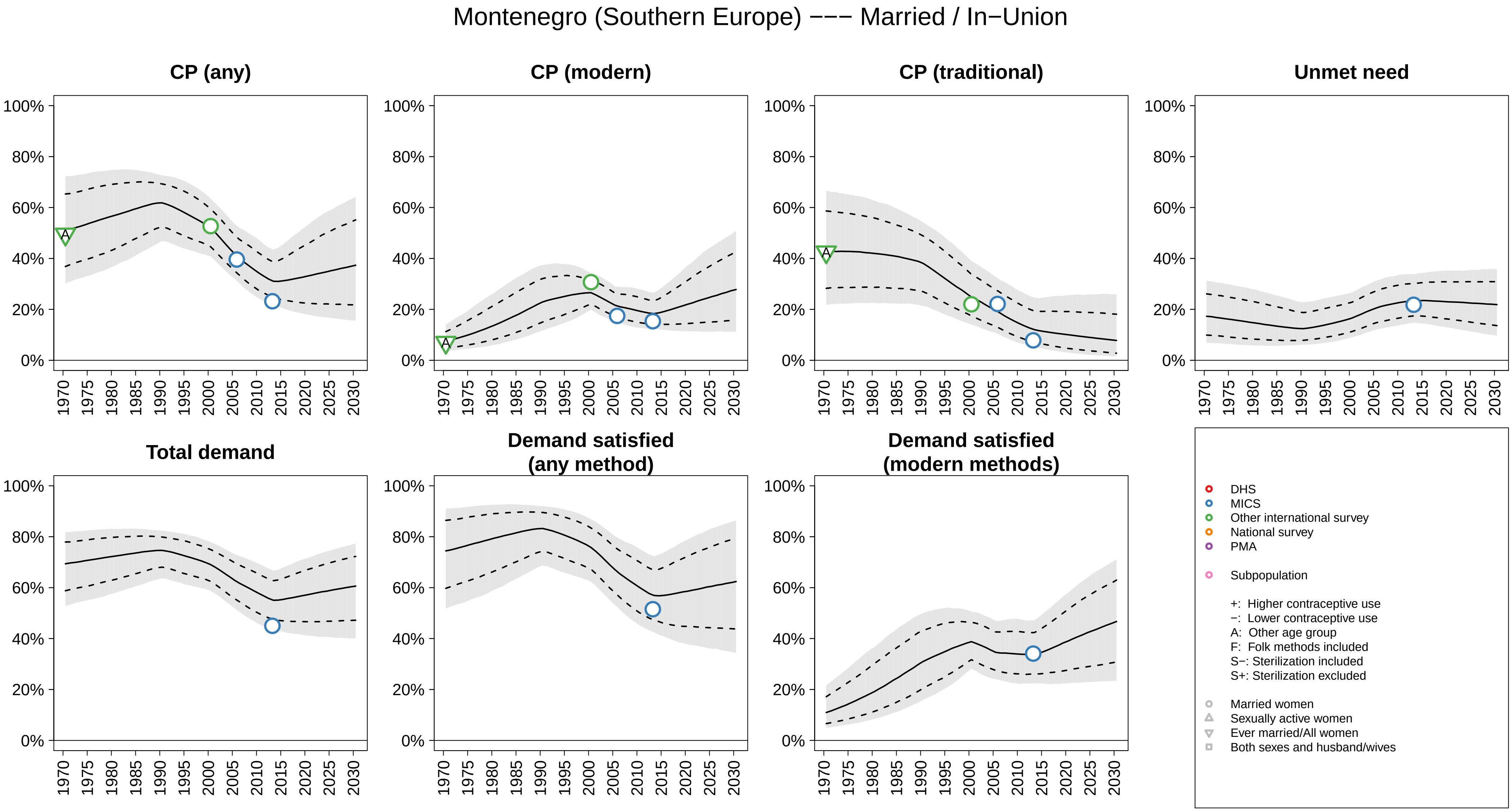
Montenegro (Southern Europe) --- Married / In−Union
CP (any)
CP (modern)
CP (traditional)
Unmet need
Total demand
Demand satisfied (any method)
Demand satisfied (modern methods)
0%
20%
40%
60%
80%
100%
1970
1975
1980
1985
1990
1995
2000
2005
2010
2015
2020
2025
2030
DHS
MICS
Other international survey
National survey
PMA
Subpopulation
+: Higher contraceptive use
−: Lower contraceptive use
A: Other age group
F: Folk methods included
S−: Sterilization included
S+: Sterilization excluded
Married women
Sexually active women
Ever married/All women
Both sexes and husband/wives

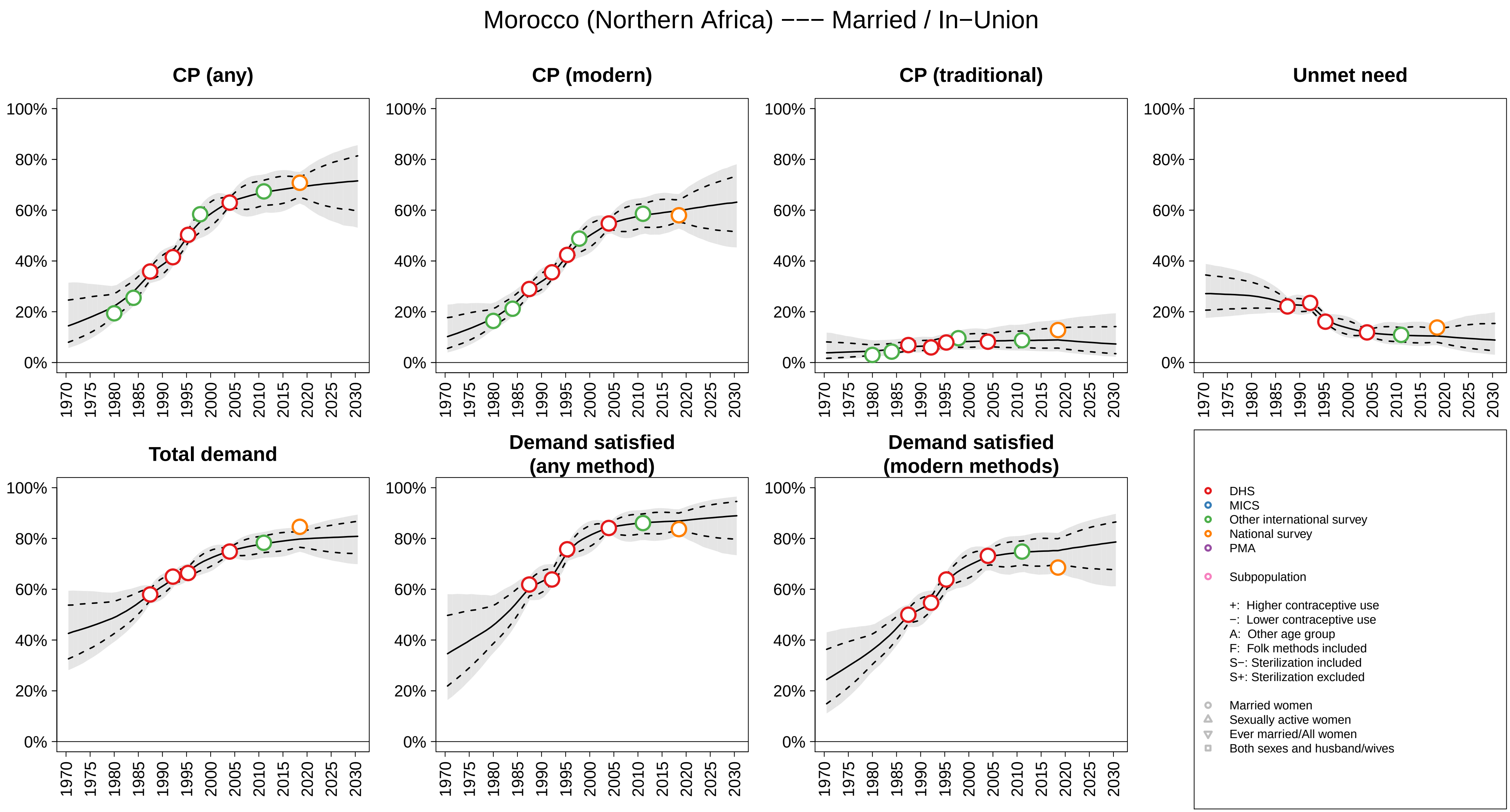

Morocco (Northern Africa) --- Married / In−Union
CP (any)
CP (modern)
CP (traditional)
Unmet need
Total demand
Demand satisfied (any method)
Demand satisfied (modern methods)
0%
20%
40%
60%
80%
100%
1970
1975
1980
1985
1990
1995
2000
2005
2010
2015
2020
2025
2030
DHS
MICS
Other international survey
National survey
PMA
Subpopulation
+: Higher contraceptive use
−: Lower contraceptive use
A: Other age group
F: Folk methods included
S−: Sterilization included
S+: Sterilization excluded
Married women
Sexually active women
Ever married/All women
Both sexes and husband/wives

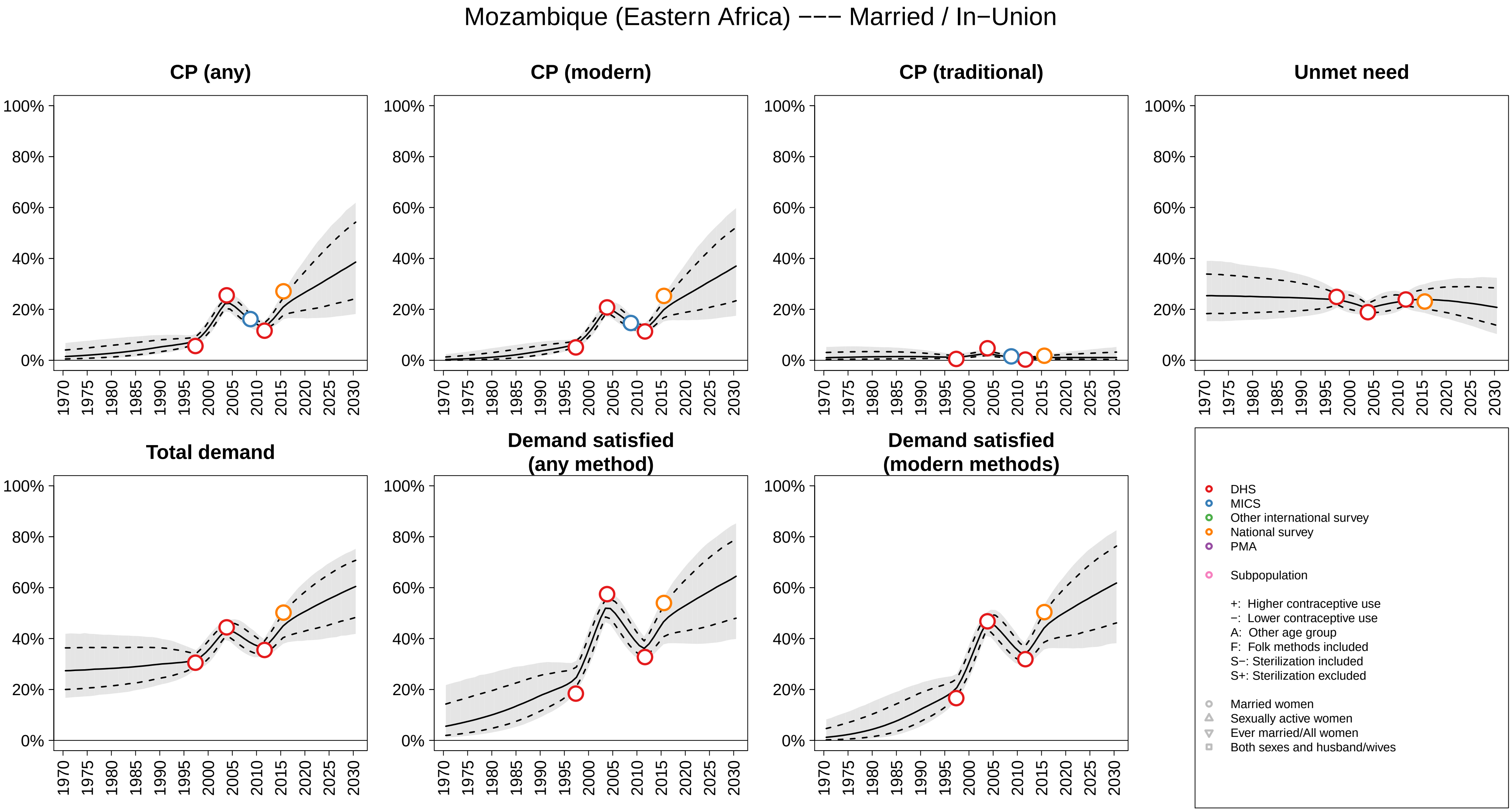
Mozambique (Eastern Africa) −−− Married / In−Union
CP (any)
CP (modern)
CP (traditional)
Unmet need
Total demand
Demand satisfied (any method)
Demand satisfied (modern methods)
100%
80%
60%
40%
20%
0%
1970 1975 1980 1985 1990 1995 2000 2005 2010 2015 2020 2025 2030
DHS
MICS
Other international survey
National survey
PMA
Subpopulation
+: Higher contraceptive use
−: Lower contraceptive use
A: Other age group
F: Folk methods included
S−: Sterilization included
S+: Sterilization excluded
Married women
Sexually active women
Ever married/All women
Both sexes and husband/wives

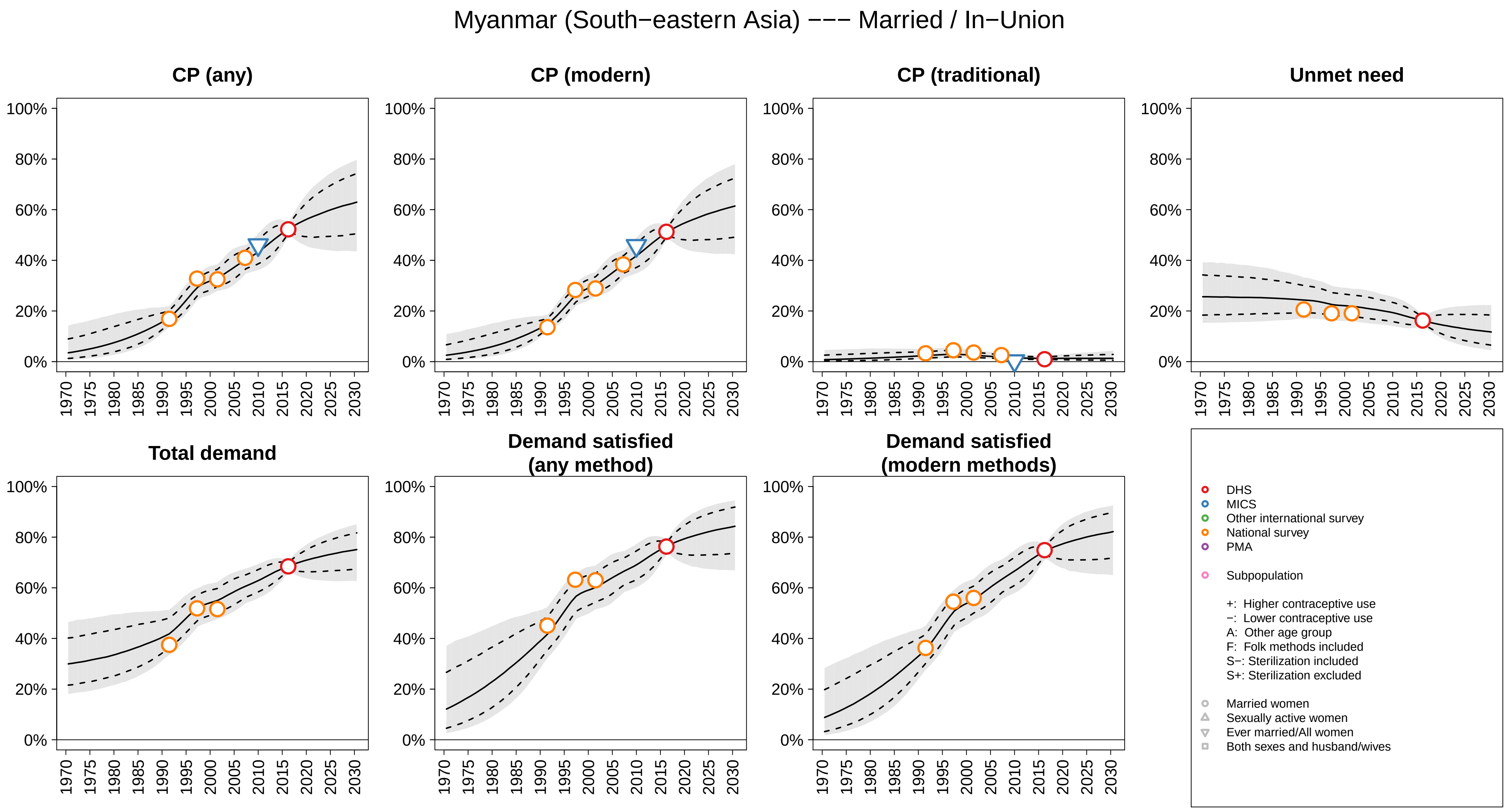

Myanmar (South−eastern Asia) −−− Married / In−Union
CP (any)
CP (modern)
CP (traditional)
Unmet need
Total demand
Demand satisfied (any method)
Demand satisfied (modern methods)
0%
20%
40%
60%
80%
100%
1970
1975
1980
1985
1990
1995
2000
2005
2010
2015
2020
2025
2030
DHS
MICS
Other international survey
National survey
PMA
Subpopulation
+: Higher contraceptive use
−: Lower contraceptive use
A: Other age group
F: Folk methods included
S−: Sterilization included
S+: Sterilization excluded
Married women
Sexually active women
Ever married/All women
Both sexes and husband/wives

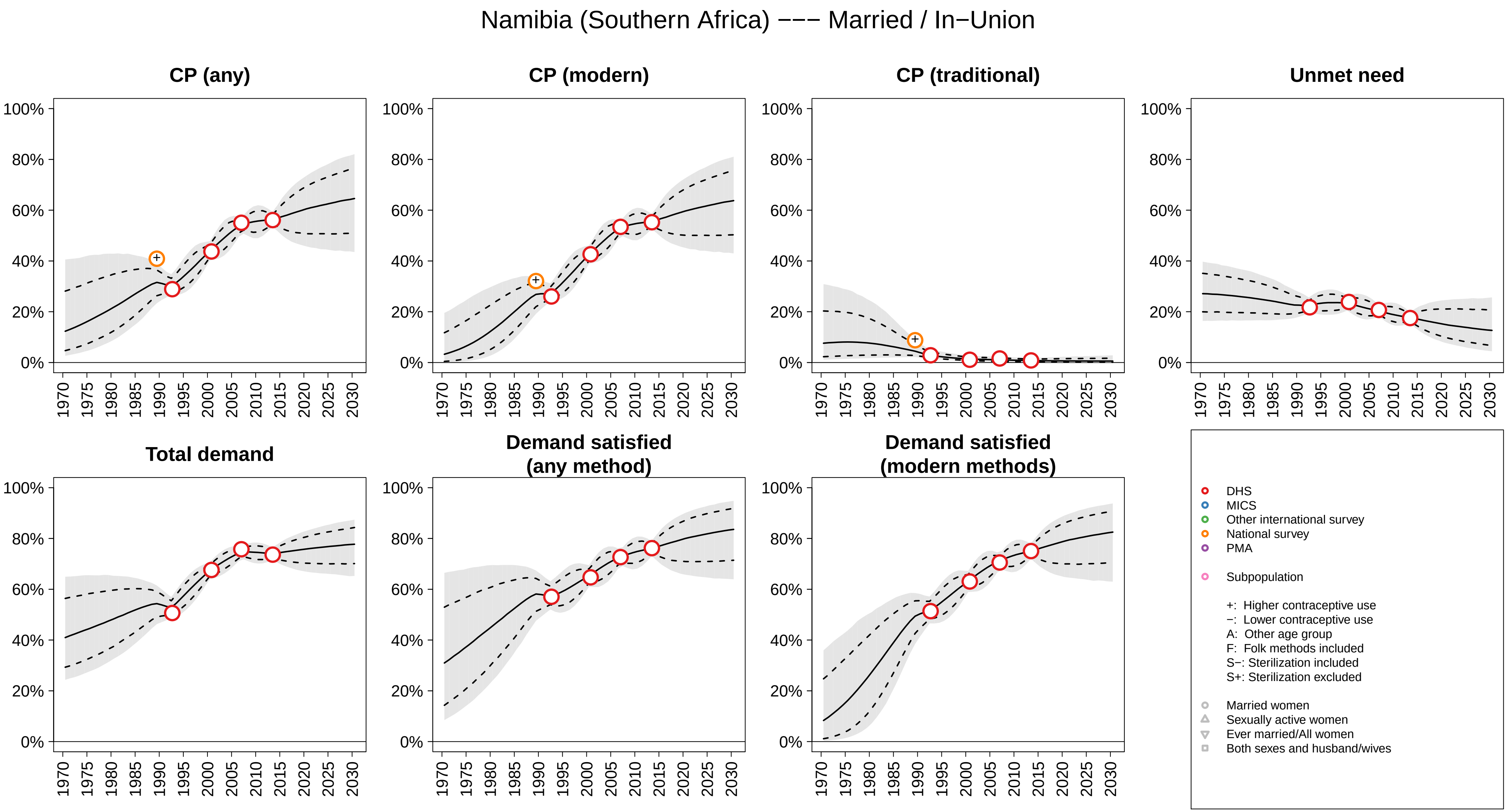

Namibia (Southern Africa) --- Married / In−Union
CP (any)
CP (modern)
CP (traditional)
Unmet need
Total demand
Demand satisfied (any method)
Demand satisfied (modern methods)
0%
20%
40%
60%
80%
100%
1970
1975
1980
1985
1990
1995
2000
2005
2010
2015
2020
2025
2030
DHS
MICS
Other international survey
National survey
PMA
Subpopulation
+: Higher contraceptive use
−: Lower contraceptive use
A: Other age group
F: Folk methods included
S−: Sterilization included
S+: Sterilization excluded
Married women
Sexually active women
Ever married/All women
Both sexes and husband/wives

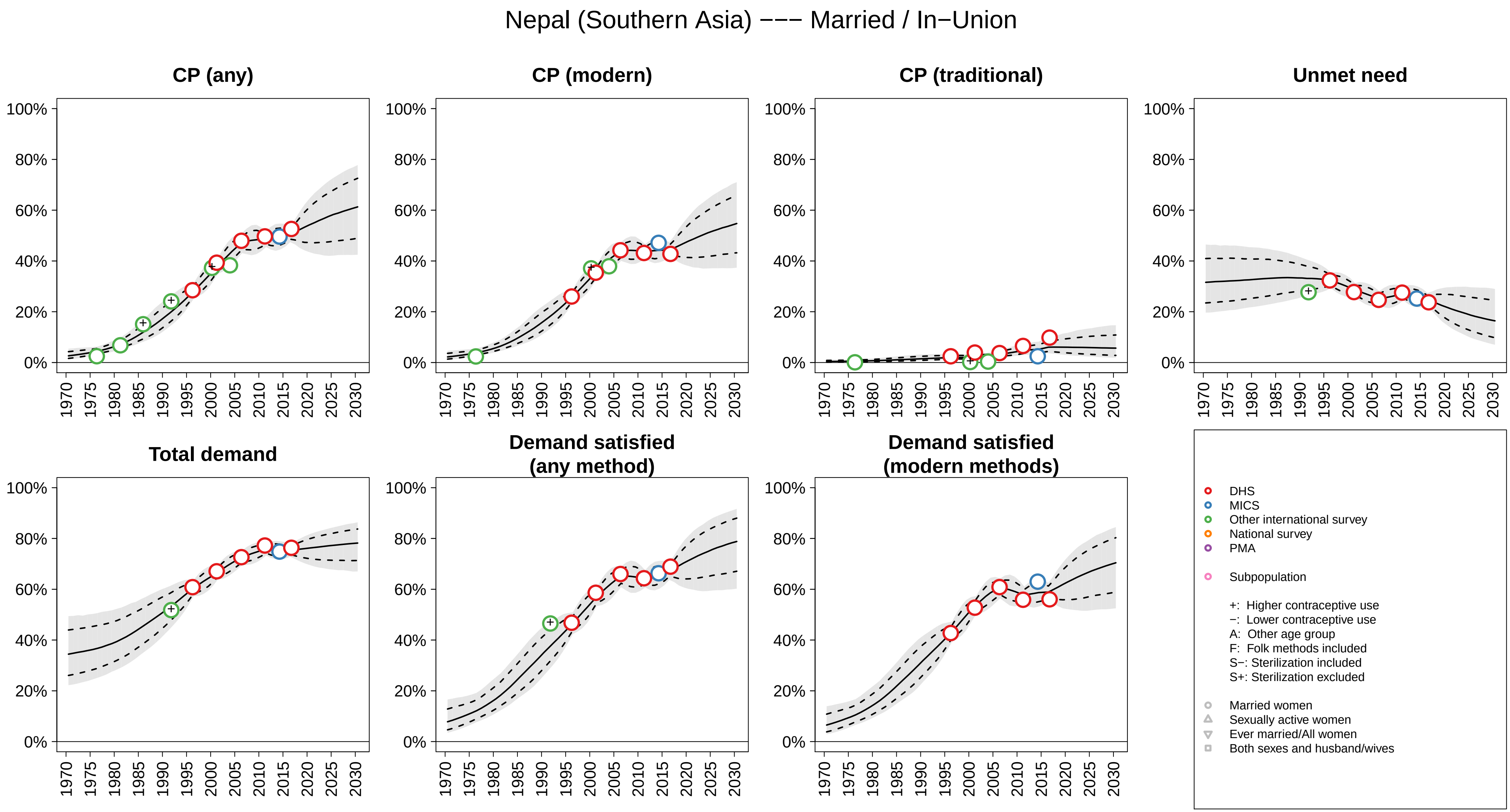
Nepal (Southern Asia) −−− Married / In−Union
CP (any)
CP (modern)
CP (traditional)
Unmet need
Total demand
Demand satisfied (any method)
Demand satisfied (modern methods)
DHS
MICS
Other international survey
National survey
PMA
Subpopulation
+: Higher contraceptive use
−: Lower contraceptive use
A: Other age group
F: Folk methods included
S−: Sterilization included
S+: Sterilization excluded
Married women
Sexually active women
Ever married/All women
Both sexes and husband/wives

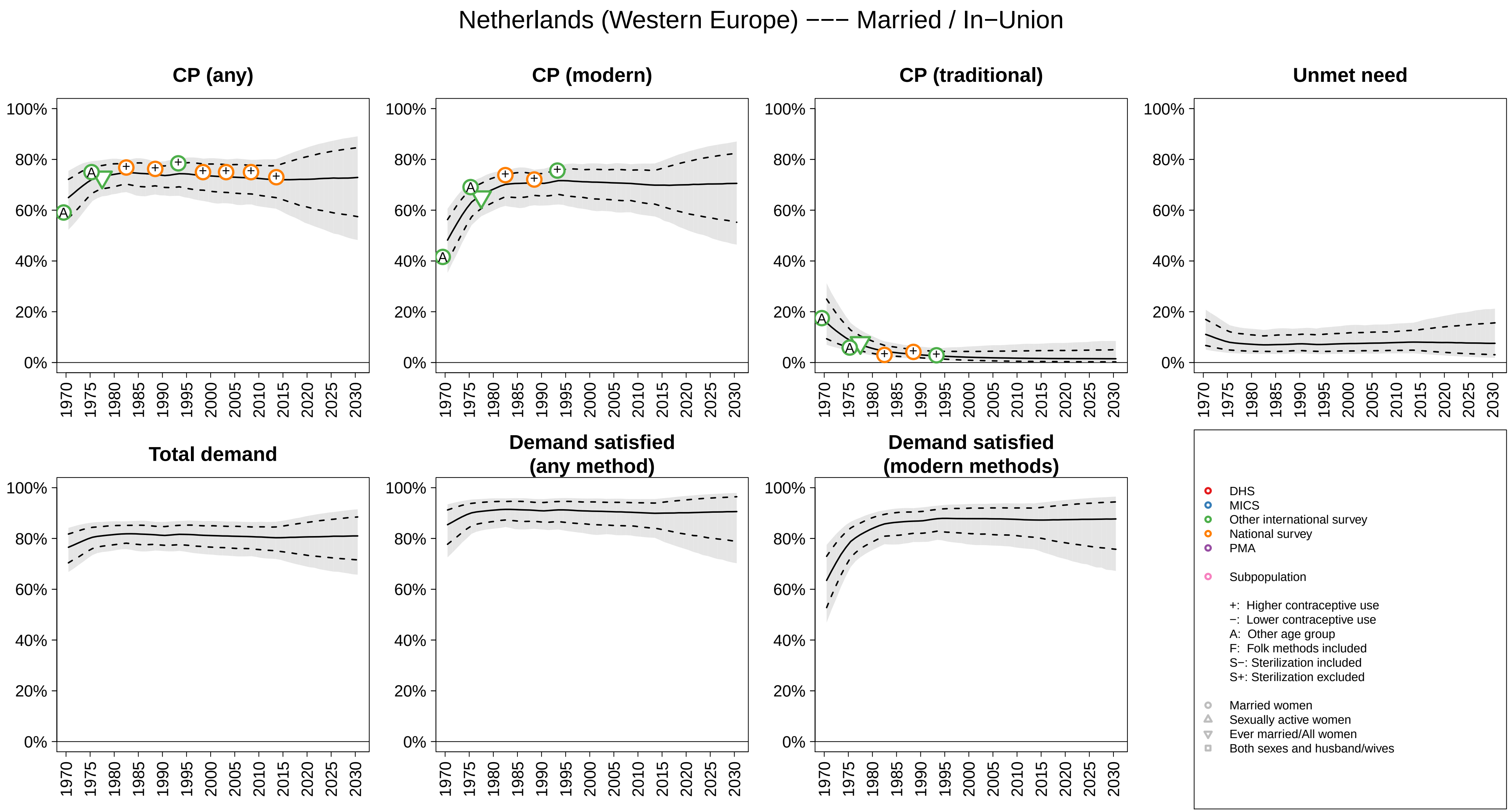
Netherlands (Western Europe) −−− Married / In−Union
CP (any)
CP (modern)
CP (traditional)
Unmet need
Total demand
Demand satisfied (any method)
Demand satisfied (modern methods)
100%
80%
60%
40%
20%
0%
1970
1975
1980
1985
1990
1995
2000
2005
2010
2015
2020
2025
2030
DHS
MICS
Other international survey
National survey
PMA
Subpopulation
+: Higher contraceptive use
−: Lower contraceptive use
A: Other age group
F: Folk methods included
S−: Sterilization included
S+: Sterilization excluded
Married women
Sexually active women
Ever married/All women
Both sexes and husband/wives

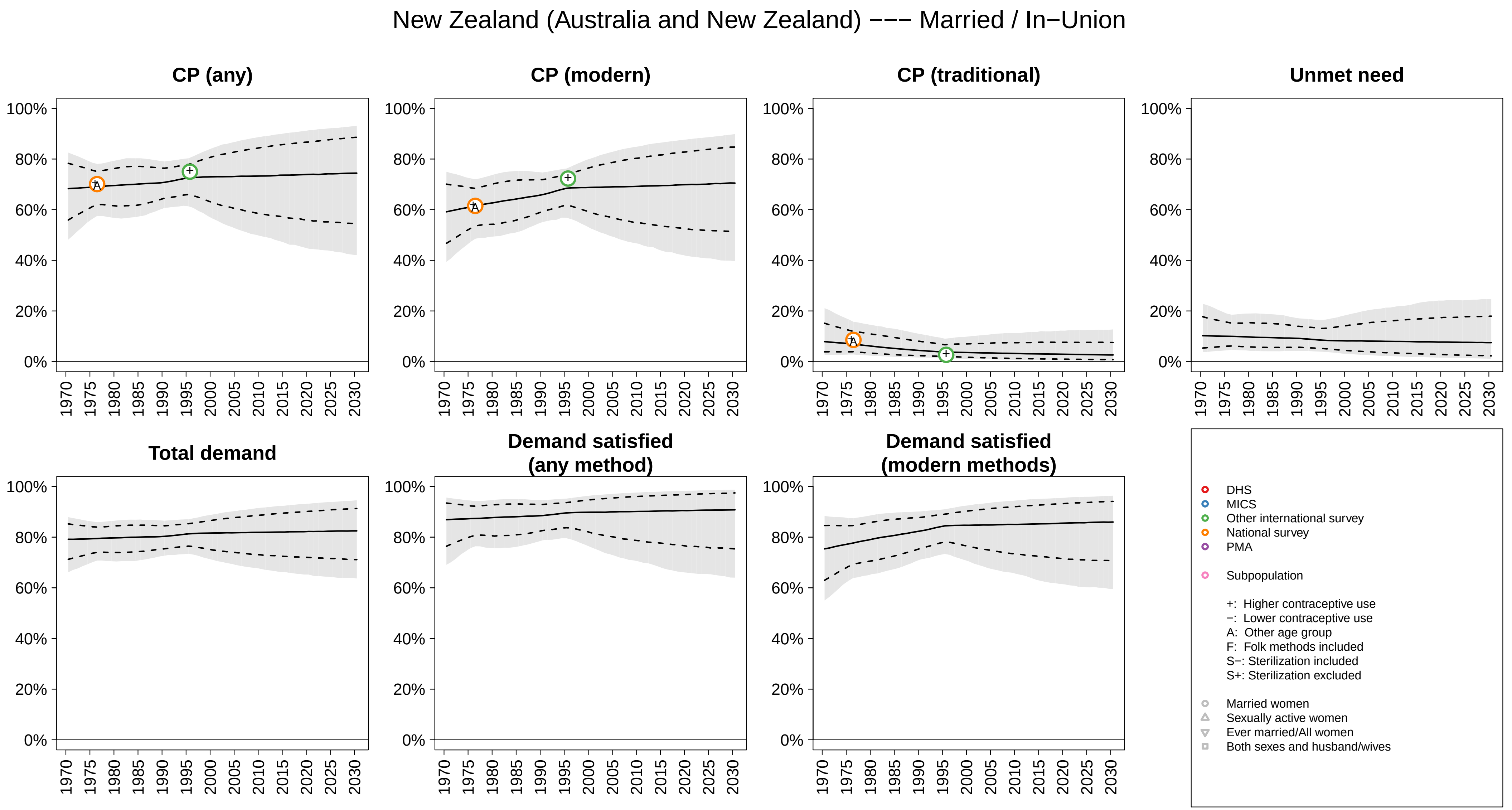

New Zealand (Australia and New Zealand) ––– Married / In−Union
CP (any)
CP (modern)
CP (traditional)
Unmet need
Total demand
Demand satisfied (any method)
Demand satisfied (modern methods)
100%
80%
60%
40%
20%
0%
1970 1975 1980 1985 1990 1995 2000 2005 2010 2015 2020 2025 2030
DHS
MICS
Other international survey
National survey
PMA
Subpopulation
+: Higher contraceptive use
−: Lower contraceptive use
A: Other age group
F: Folk methods included
S−: Sterilization included
S+: Sterilization excluded
Married women
Sexually active women
Ever married/All women
Both sexes and husband/wives

Nicaragua (Central America) −−− Married / In−Union

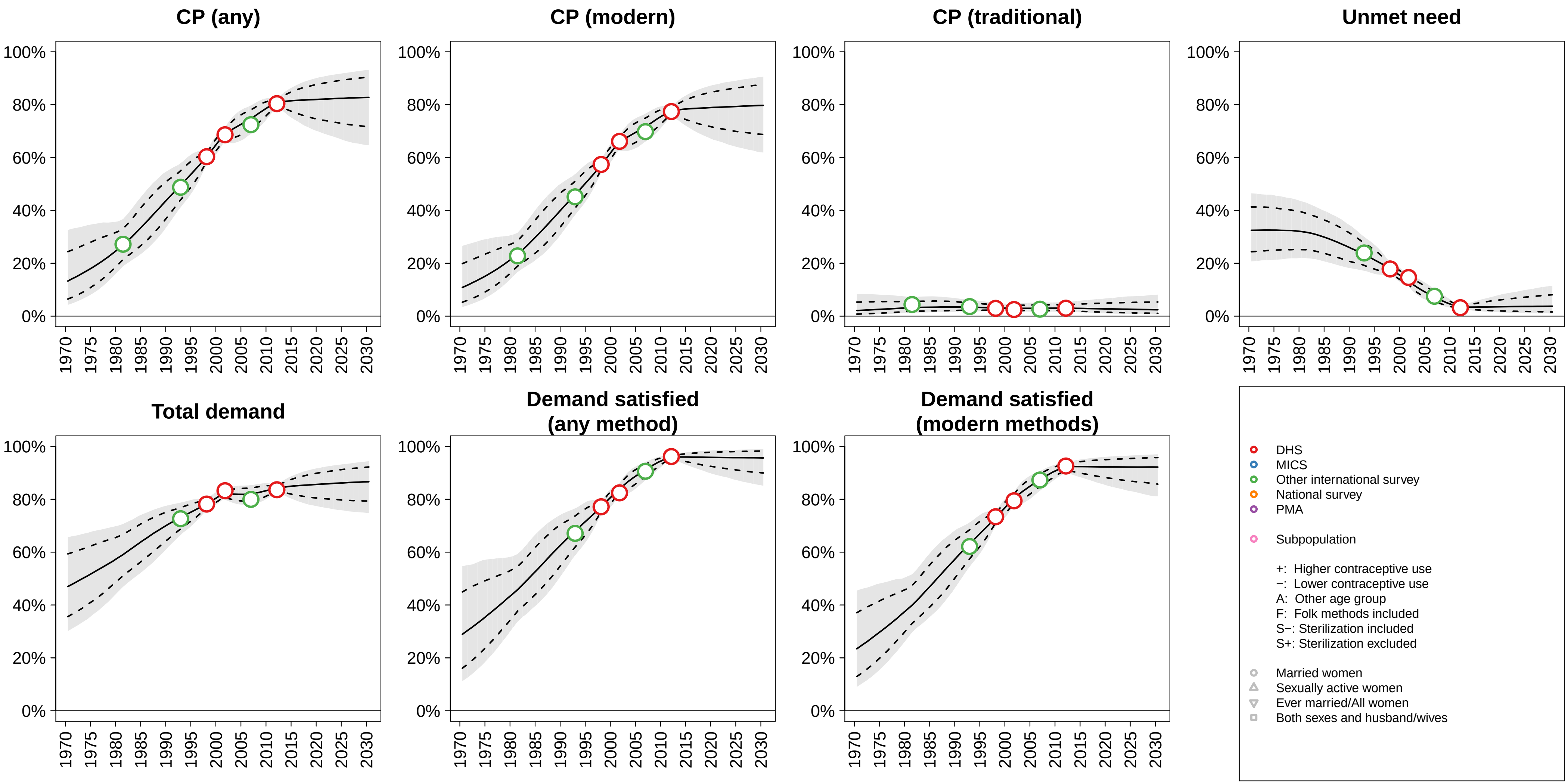

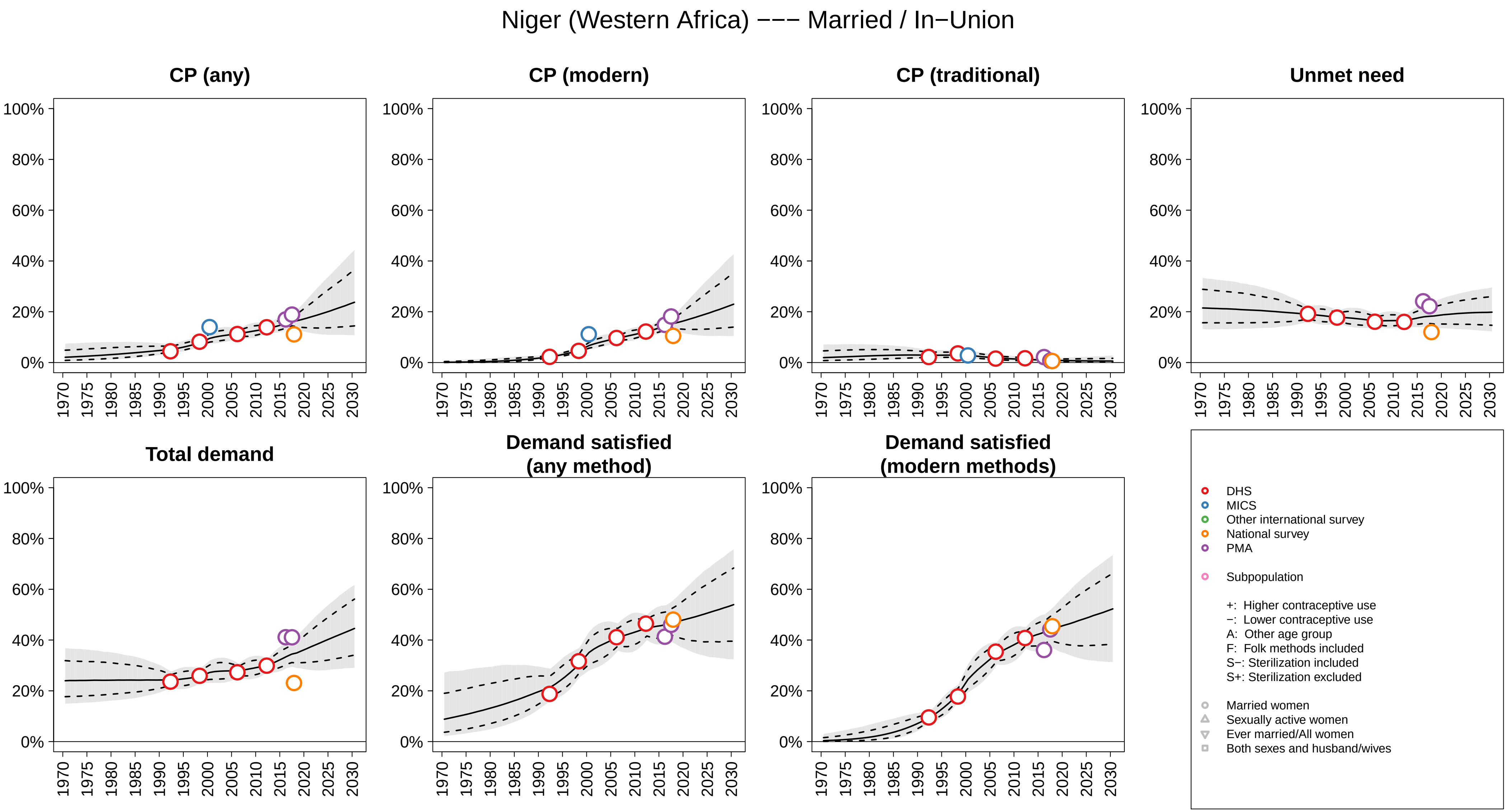
Niger (Western Africa) --- Married / In−Union
CP (any)
CP (modern)
CP (traditional)
Unmet need
Total demand
Demand satisfied (any method)
Demand satisfied (modern methods)
0%
20%
40%
60%
80%
100%
1970
1975
1980
1985
1990
1995
2000
2005
2010
2015
2020
2025
2030
DHS
MICS
Other international survey
National survey
PMA
Subpopulation
+: Higher contraceptive use
−: Lower contraceptive use
A: Other age group
F: Folk methods included
S−: Sterilization included
S+: Sterilization excluded
Married women
Sexually active women
Ever married/All women
Both sexes and husband/wives

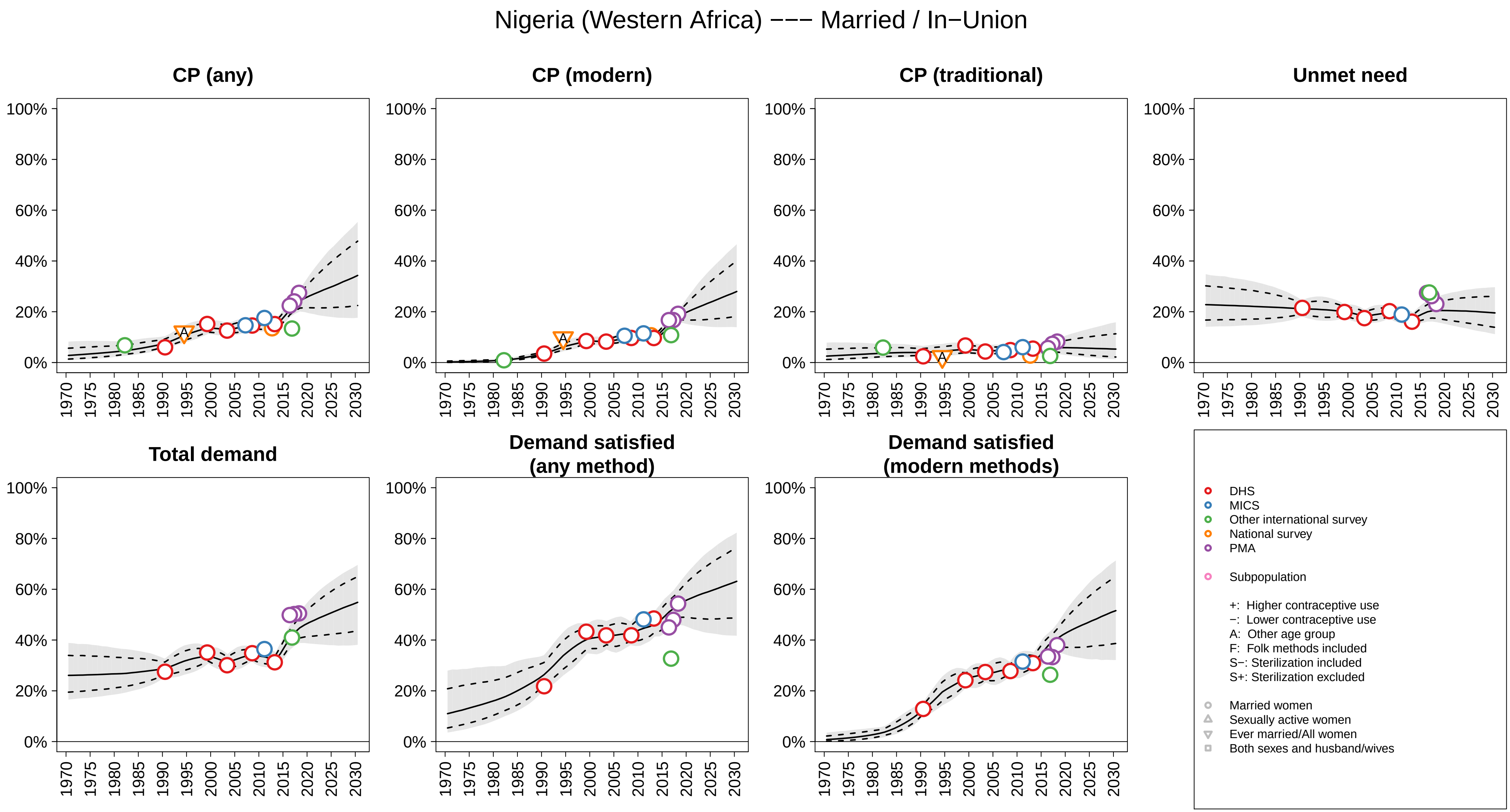
Nigeria (Western Africa) −−− Married / In−Union
CP (any)
CP (modern)
CP (traditional)
Unmet need
Total demand
Demand satisfied (any method)
Demand satisfied (modern methods)
100%
80%
60%
40%
20%
0%
1970 1975 1980 1985 1990 1995 2000 2005 2010 2015 2020 2025 2030
DHS
MICS
Other international survey
National survey
PMA
Subpopulation
+: Higher contraceptive use
−: Lower contraceptive use
A: Other age group
F: Folk methods included
S−: Sterilization included
S+: Sterilization excluded
Married women
Sexually active women
Ever married/All women
Both sexes and husband/wives

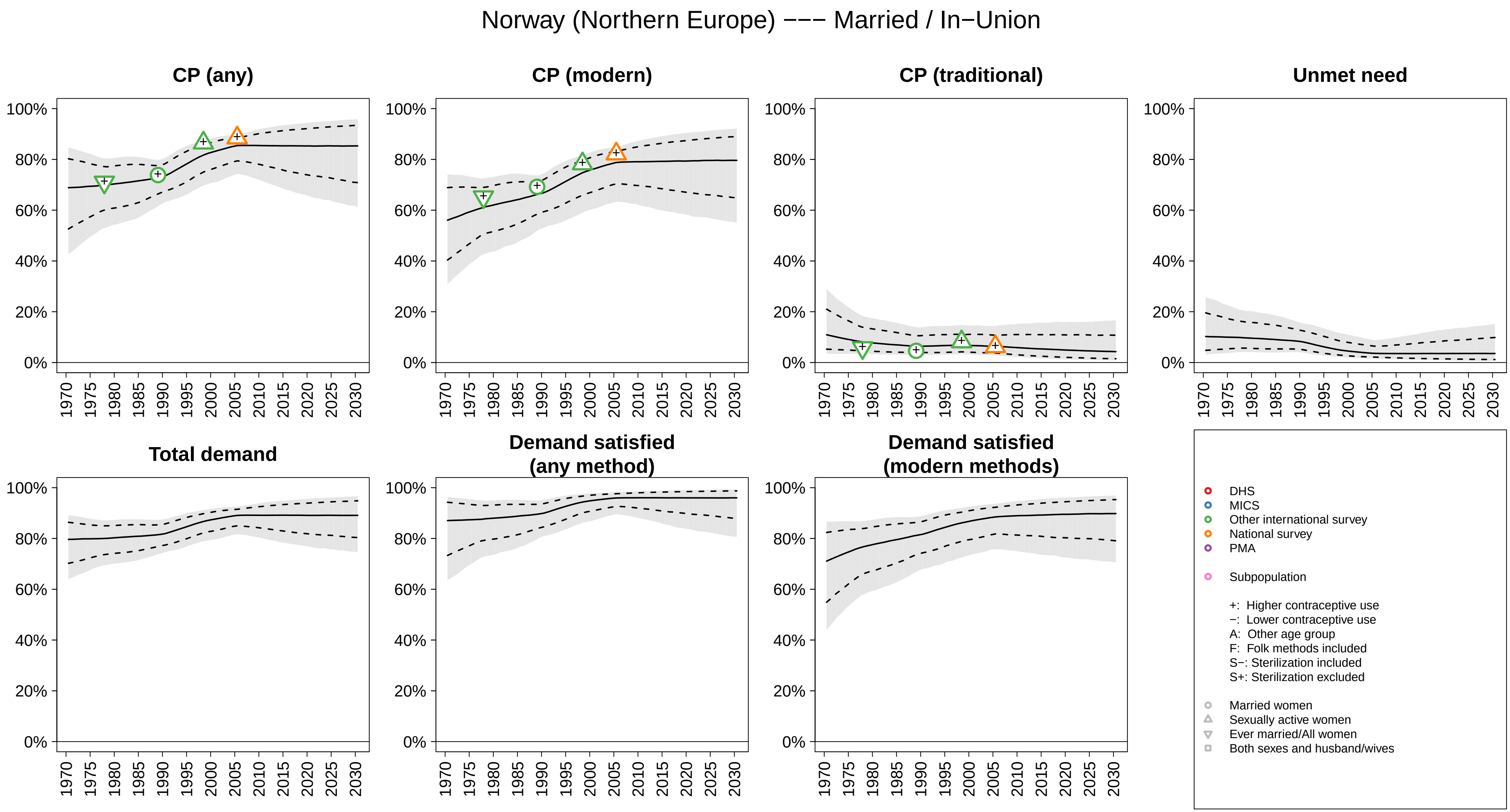
Norway (Northern Europe) --- Married / In−Union
CP (any)
CP (modern)
CP (traditional)
Unmet need
Total demand
Demand satisfied (any method)
Demand satisfied (modern methods)
DHS
MICS
Other international survey
National survey
PMA
Subpopulation
+: Higher contraceptive use
−: Lower contraceptive use
A: Other age group
F: Folk methods included
S−: Sterilization included
S+: Sterilization excluded
Married women
Sexually active women
Ever married/All women
Both sexes and husband/wives

Oman (Western Asia) −−− Married / In−Union

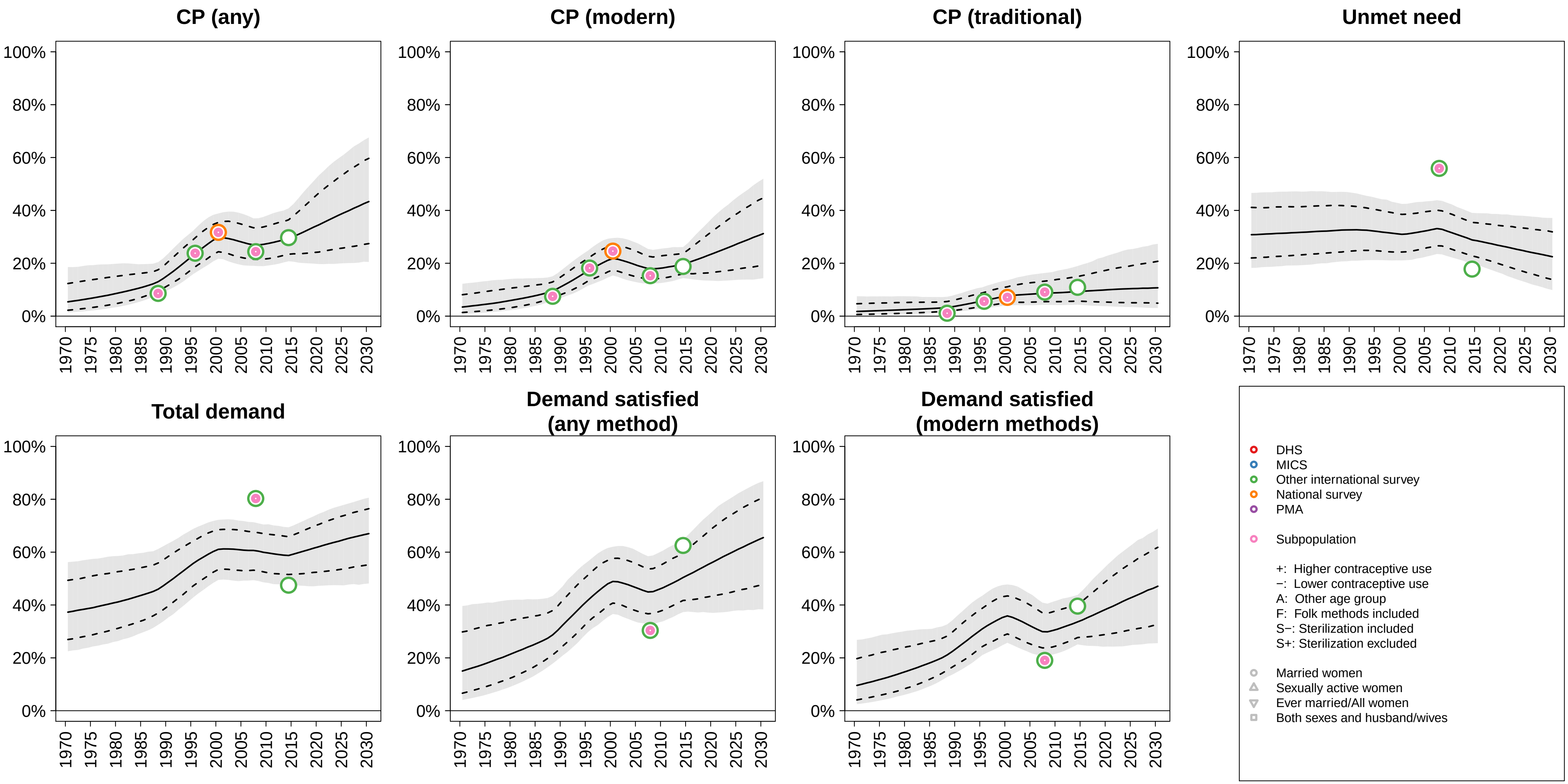

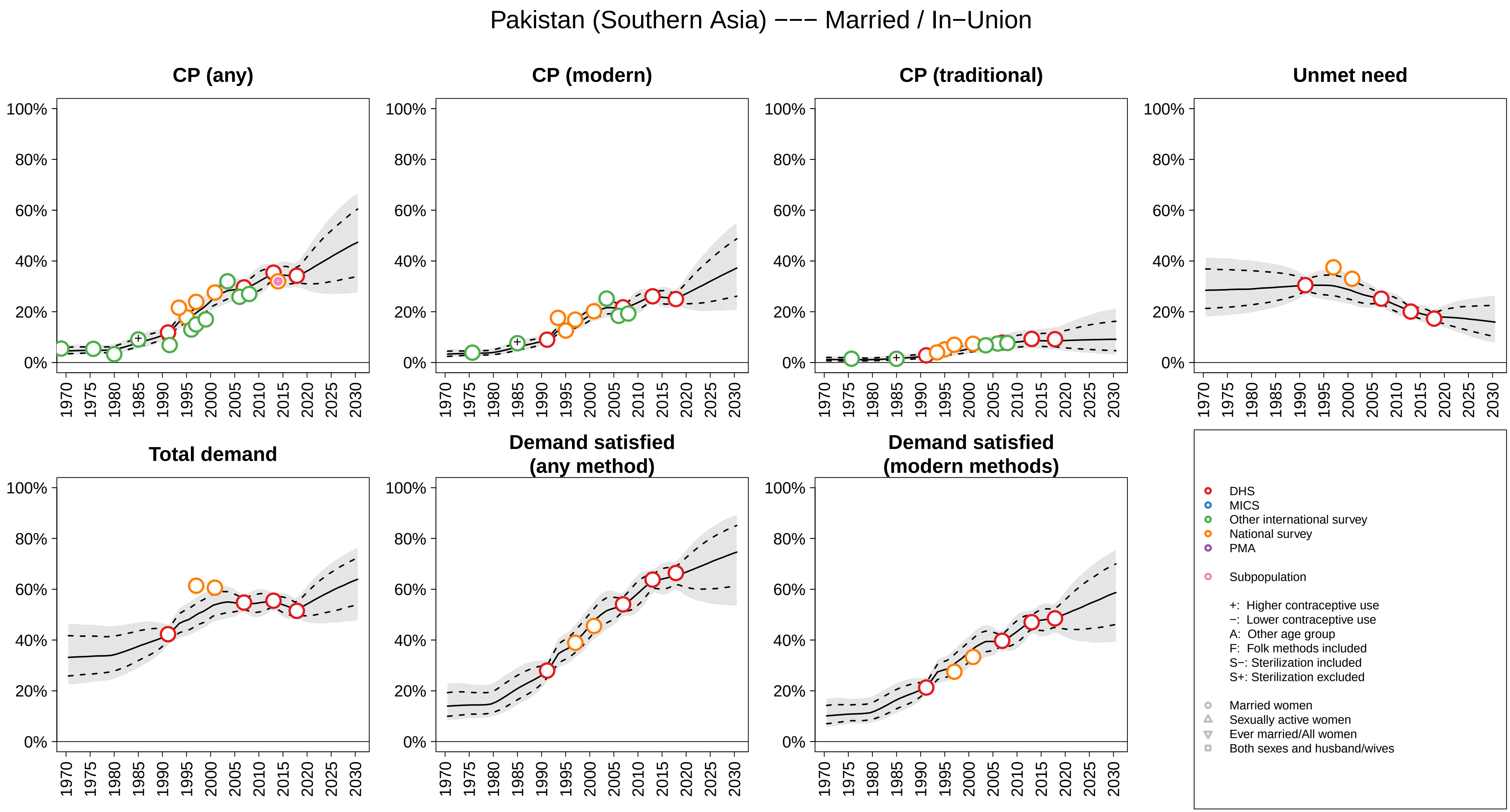
Pakistan (Southern Asia) --- Married / In−Union
CP (any)
CP (modern)
CP (traditional)
Unmet need
Total demand
Demand satisfied (any method)
Demand satisfied (modern methods)
0%
20%
40%
60%
80%
100%
1970
1975
1980
1985
1990
1995
2000
2005
2010
2015
2020
2025
2030
DHS
MICS
Other international survey
National survey
PMA
Subpopulation
+: Higher contraceptive use
−: Lower contraceptive use
A: Other age group
F: Folk methods included
S−: Sterilization included
S+: Sterilization excluded
Married women
Sexually active women
Ever married/All women
Both sexes and husband/wives

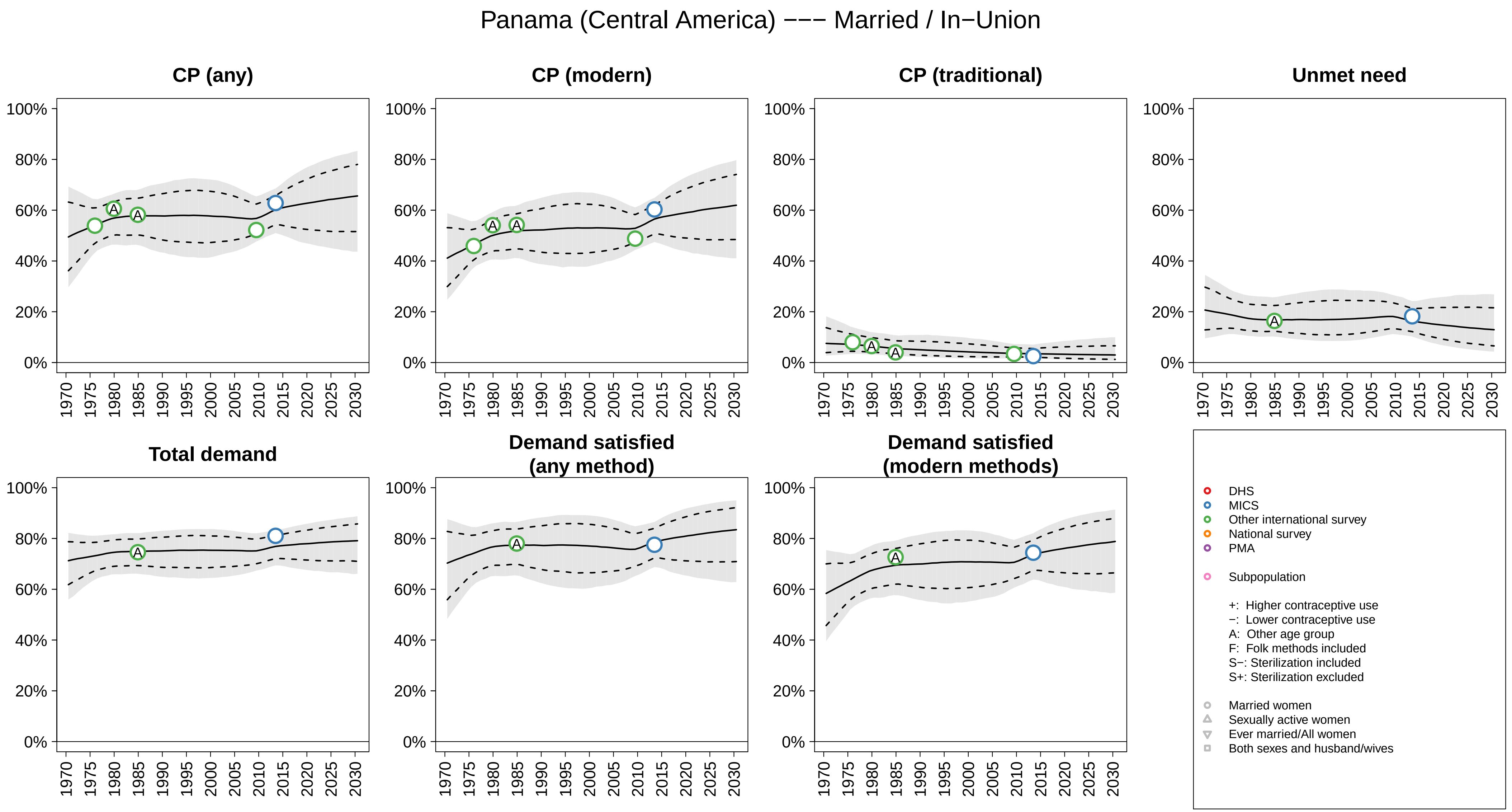
Panama (Central America) −−− Married / In−Union
CP (any)
CP (modern)
CP (traditional)
Unmet need
Total demand
Demand satisfied (any method)
Demand satisfied (modern methods)
100%
80%
60%
40%
20%
0%
1970 1975 1980 1985 1990 1995 2000 2005 2010 2015 2020 2025 2030
DHS
MICS
Other international survey
National survey
PMA
Subpopulation
+: Higher contraceptive use
−: Lower contraceptive use
A: Other age group
F: Folk methods included
S−: Sterilization included
S+: Sterilization excluded
Married women
Sexually active women
Ever married/All women
Both sexes and husband/wives

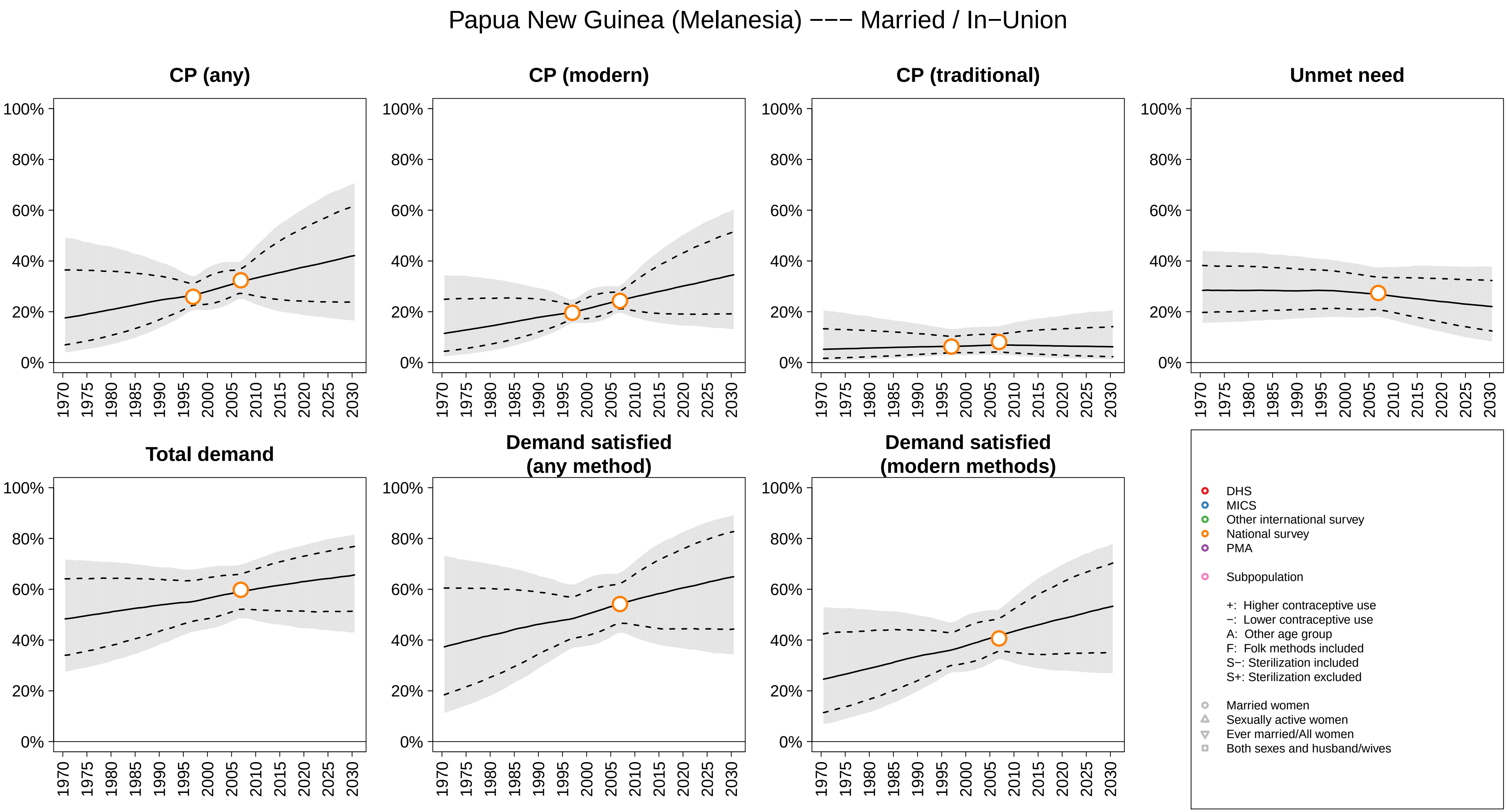
Papua New Guinea (Melanesia) −−− Married / In−Union
CP (any)
CP (modern)
CP (traditional)
Unmet need
Total demand
Demand satisfied (any method)
Demand satisfied (modern methods)
0%
20%
40%
60%
80%
100%
1970
1975
1980
1985
1990
1995
2000
2005
2010
2015
2020
2025
2030
DHS
MICS
Other international survey
National survey
PMA
Subpopulation
+: Higher contraceptive use
−: Lower contraceptive use
A: Other age group
F: Folk methods included
S−: Sterilization included
S+: Sterilization excluded
Married women
Sexually active women
Ever married/All women
Both sexes and husband/wives

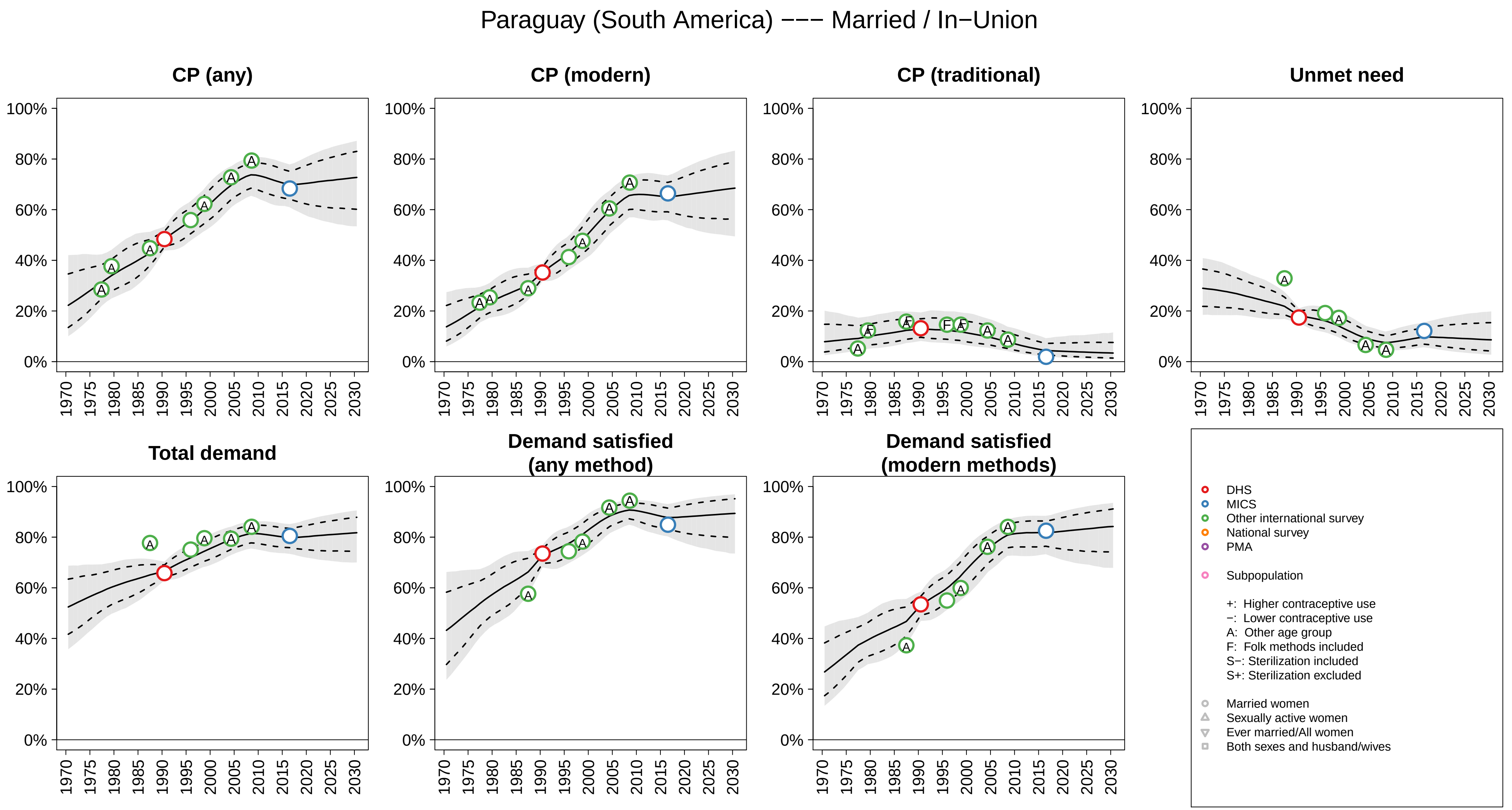
Paraguay (South America) −−− Married / In−Union
CP (any)
CP (modern)
CP (traditional)
Unmet need
Total demand
Demand satisfied (any method)
Demand satisfied (modern methods)
DHS
MICS
Other international survey
National survey
PMA
Subpopulation
+: Higher contraceptive use
−: Lower contraceptive use
A: Other age group
F: Folk methods included
S−: Sterilization included
S+: Sterilization excluded
Married women
Sexually active women
Ever married/All women
Both sexes and husband/wives

Peru (South America) −−− Married / In−Union

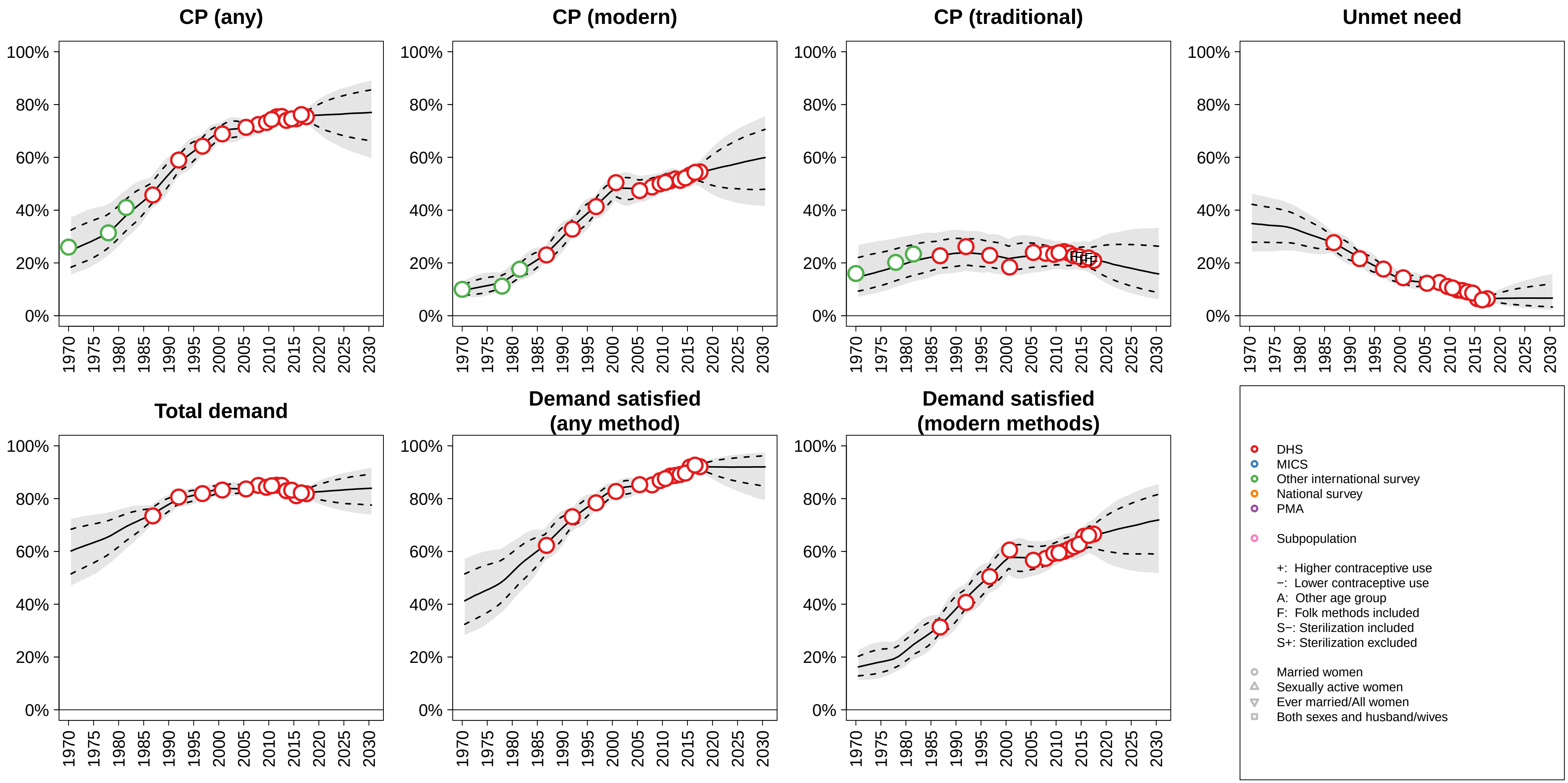

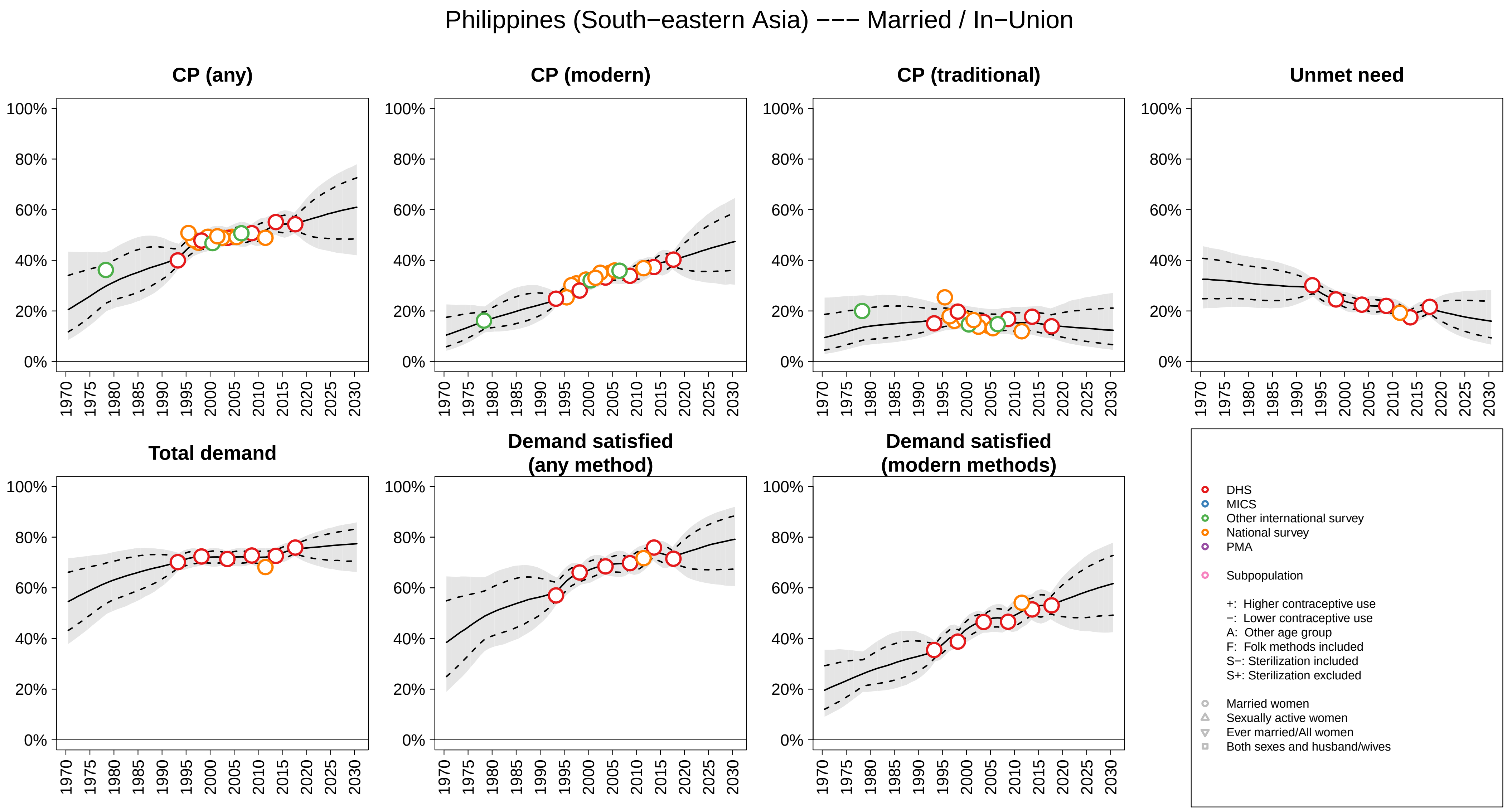

Philippines (South−eastern Asia) −−− Married / In−Union
CP (any)
CP (modern)
CP (traditional)
Unmet need
Total demand
Demand satisfied (any method)
Demand satisfied (modern methods)
0%
20%
40%
60%
80%
100%
1970
1975
1980
1985
1990
1995
2000
2005
2010
2015
2020
2025
2030
DHS
MICS
Other international survey
National survey
PMA
Subpopulation
+: Higher contraceptive use
−: Lower contraceptive use
A: Other age group
F: Folk methods included
S−: Sterilization included
S+: Sterilization excluded
Married women
Sexually active women
Ever married/All women
Both sexes and husband/wives

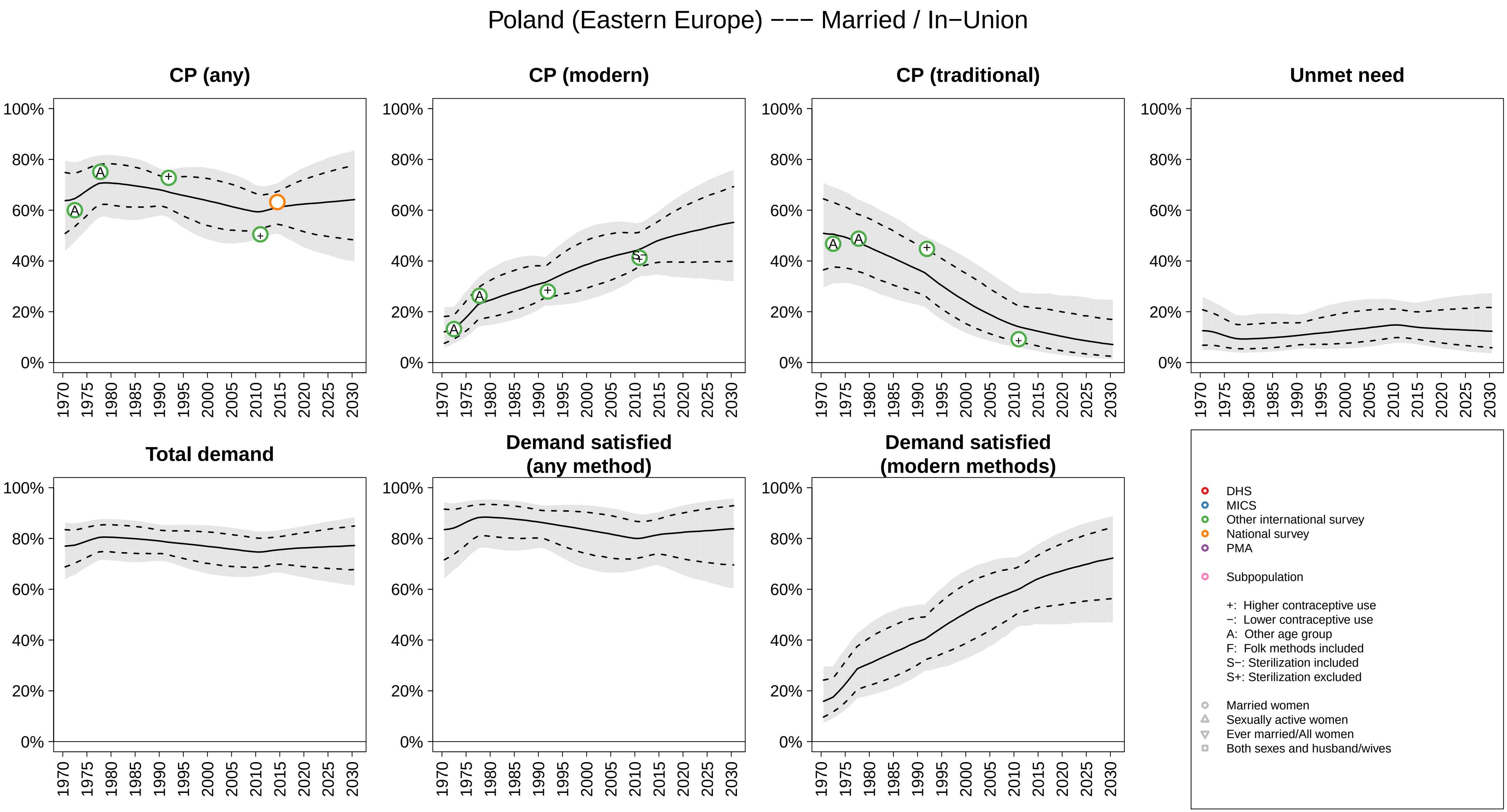
Poland (Eastern Europe) −−− Married / In−Union
CP (any)
CP (modern)
CP (traditional)
Unmet need
Total demand
Demand satisfied (any method)
Demand satisfied (modern methods)
0%
20%
40%
60%
80%
100%
1970
1975
1980
1985
1990
1995
2000
2005
2010
2015
2020
2025
2030
DHS
MICS
Other international survey
National survey
PMA
Subpopulation
+: Higher contraceptive use
−: Lower contraceptive use
A: Other age group
F: Folk methods included
S−: Sterilization included
S+: Sterilization excluded
Married women
Sexually active women
Ever married/All women
Both sexes and husband/wives

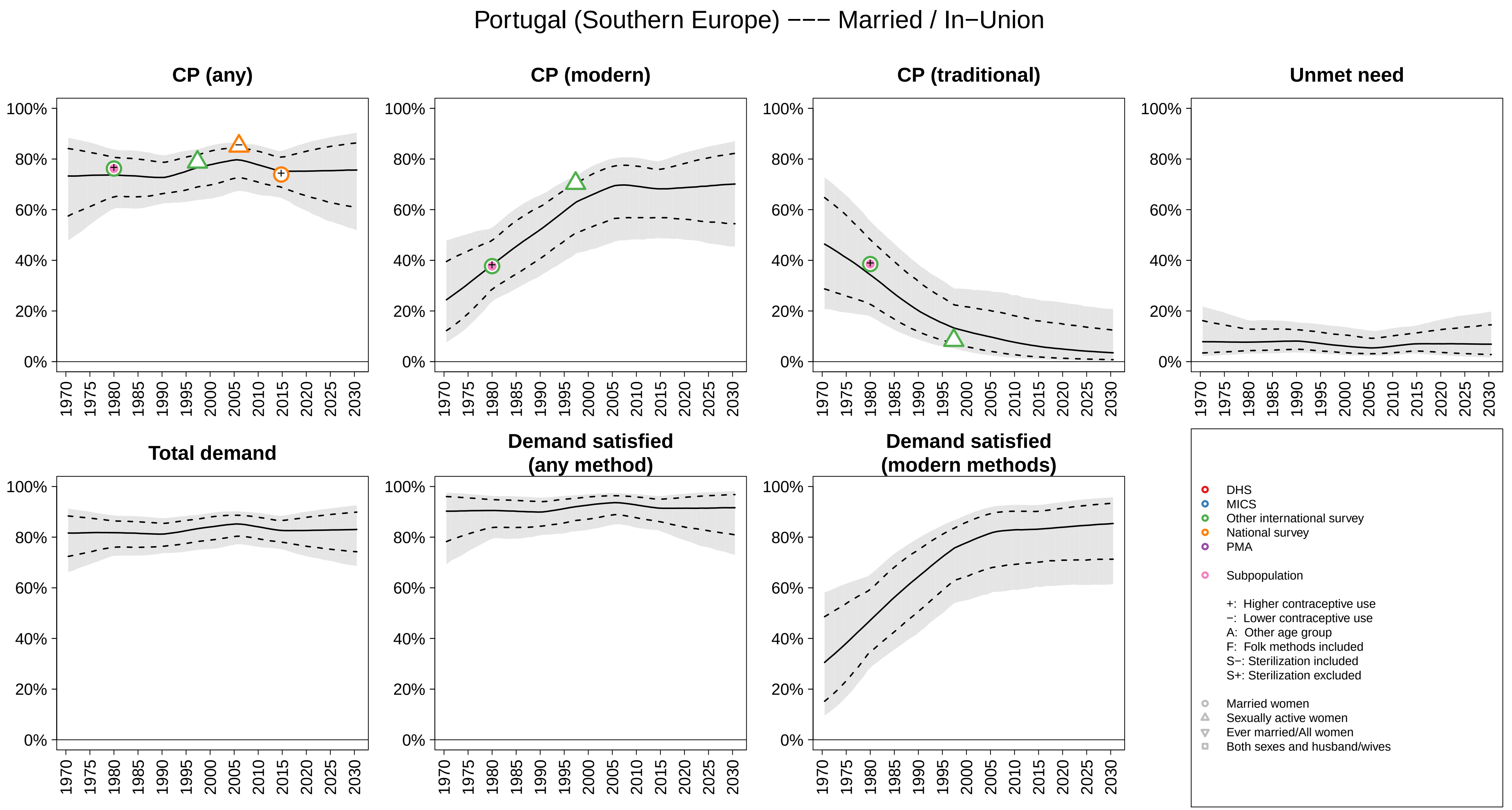
Portugal (Southern Europe) −−− Married / In−Union
CP (any)
CP (modern)
CP (traditional)
Unmet need
Total demand
Demand satisfied (any method)
Demand satisfied (modern methods)
DHS
MICS
Other international survey
National survey
PMA
Subpopulation
+: Higher contraceptive use
−: Lower contraceptive use
A: Other age group
F: Folk methods included
S−: Sterilization included
S+: Sterilization excluded
Married women
Sexually active women
Ever married/All women
Both sexes and husband/wives

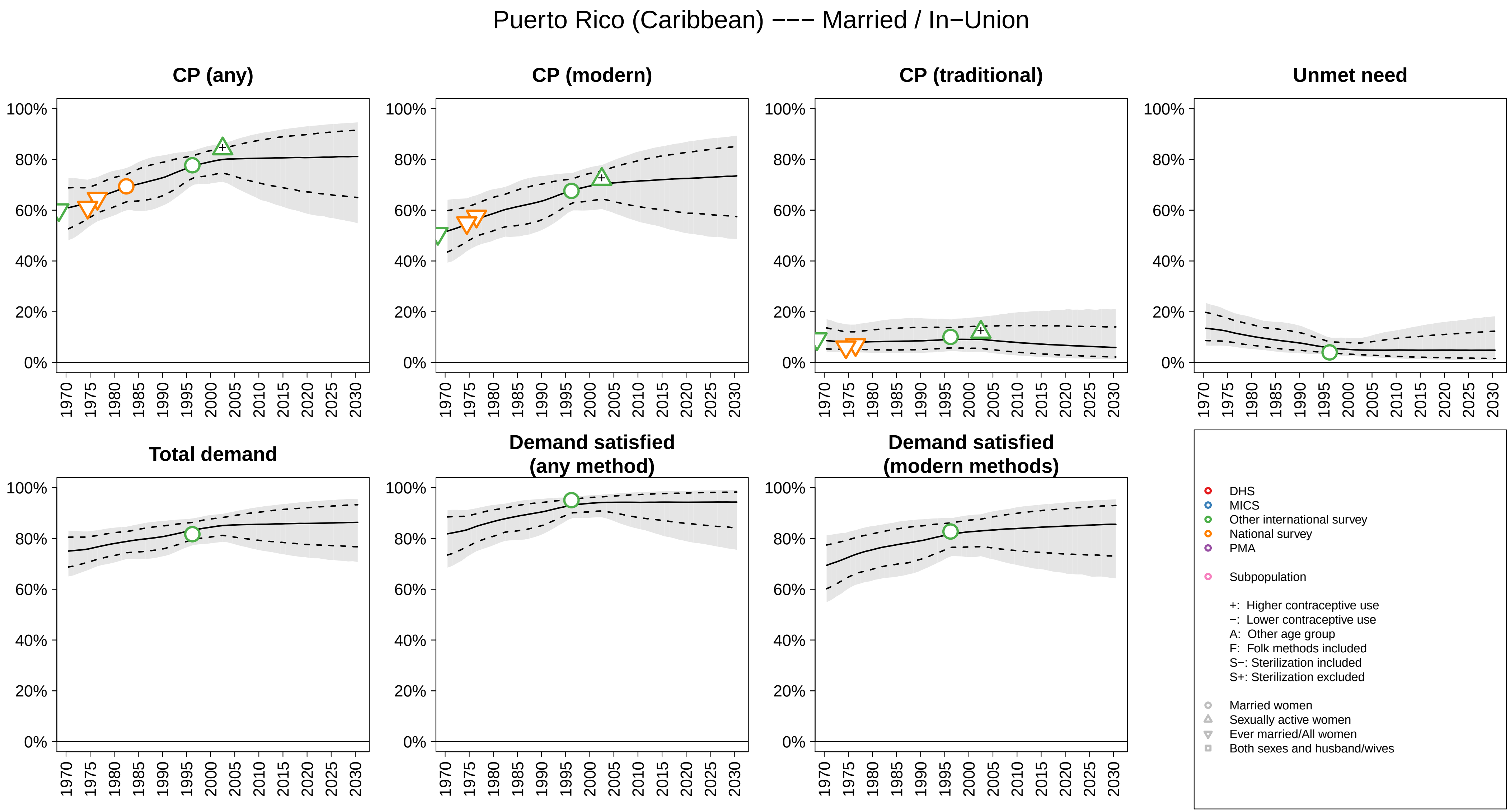
Puerto Rico (Caribbean) --- Married / In−Union
CP (any)
CP (modern)
CP (traditional)
Unmet need
Total demand
Demand satisfied (any method)
Demand satisfied (modern methods)
DHS
MICS
Other international survey
National survey
PMA
Subpopulation
+: Higher contraceptive use
−: Lower contraceptive use
A: Other age group
F: Folk methods included
S−: Sterilization included
S+: Sterilization excluded
Married women
Sexually active women
Ever married/All women
Both sexes and husband/wives

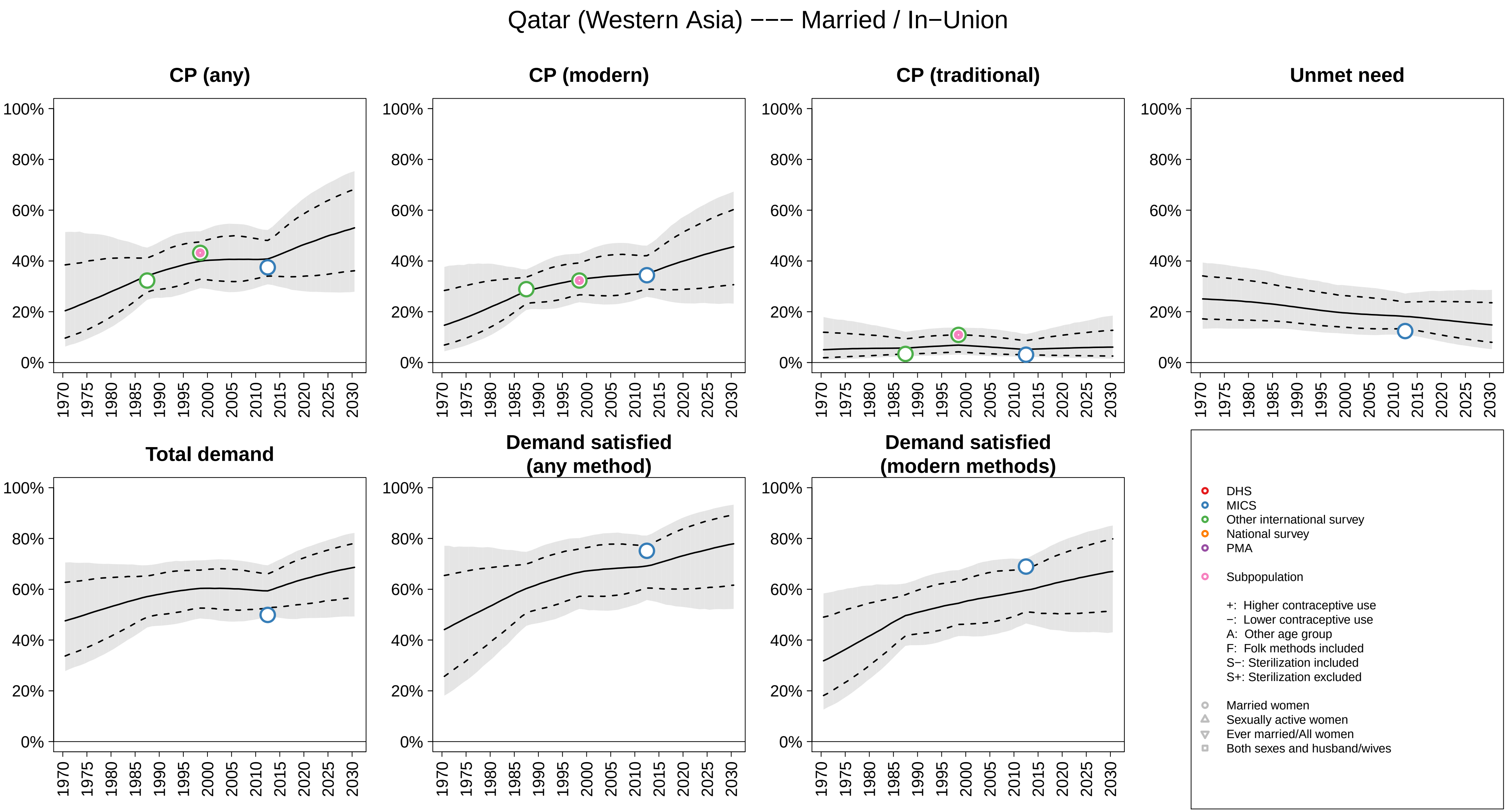
Qatar (Western Asia) −−− Married / In−Union
CP (any)
CP (modern)
CP (traditional)
Unmet need
Total demand
Demand satisfied (any method)
Demand satisfied (modern methods)
100%
80%
60%
40%
20%
0%
1970 1975 1980 1985 1990 1995 2000 2005 2010 2015 2020 2025 2030
DHS
MICS
Other international survey
National survey
PMA
Subpopulation
+: Higher contraceptive use
−: Lower contraceptive use
A: Other age group
F: Folk methods included
S−: Sterilization included
S+: Sterilization excluded
Married women
Sexually active women
Ever married/All women
Both sexes and husband/wives

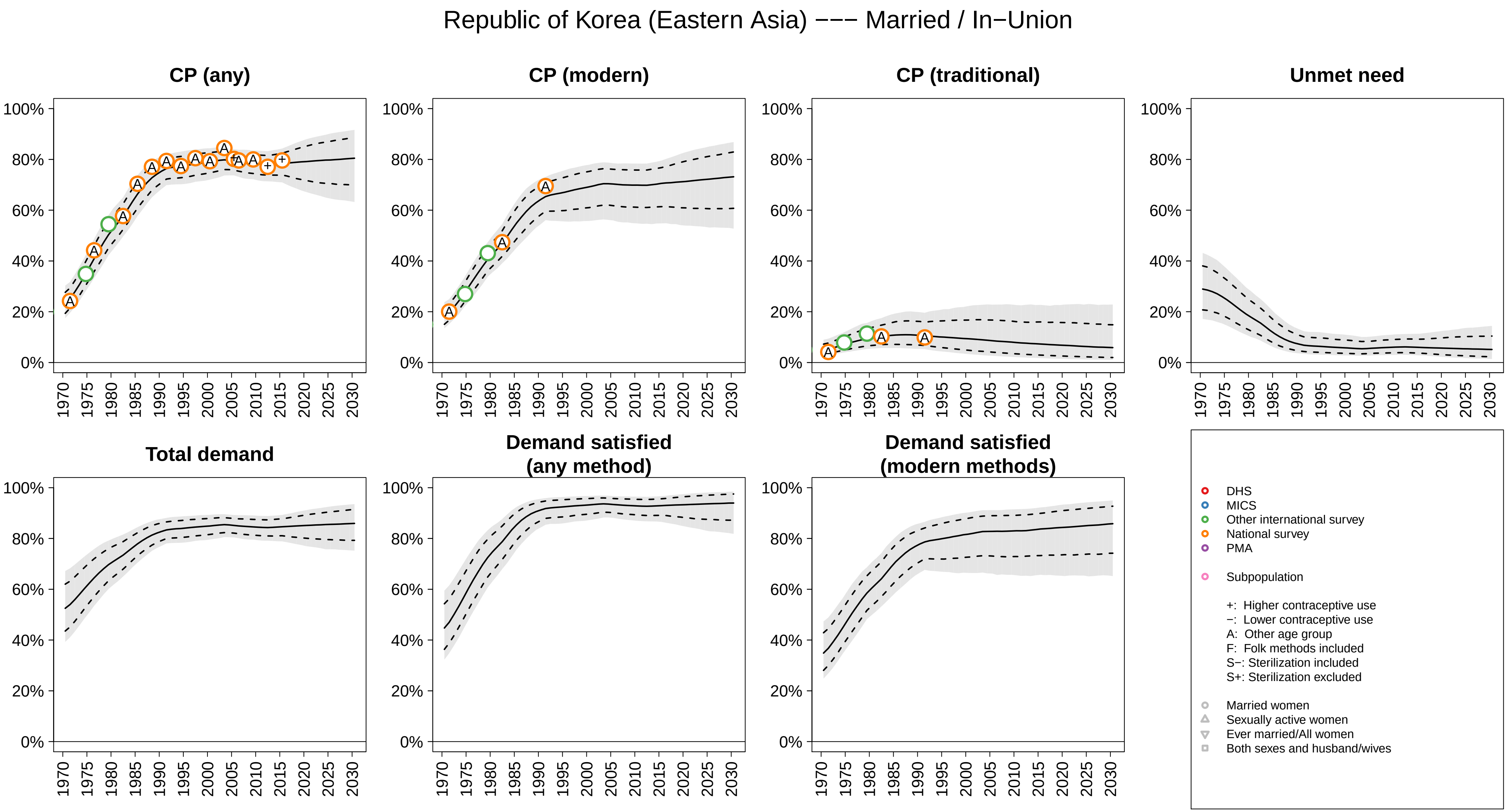
Republic of Korea (Eastern Asia) −−− Married / In−Union
CP (any)
CP (modern)
CP (traditional)
Unmet need
Total demand
Demand satisfied (any method)
Demand satisfied (modern methods)
0%
20%
40%
60%
80%
100%
1970
1975
1980
1985
1990
1995
2000
2005
2010
2015
2020
2025
2030
DHS
MICS
Other international survey
National survey
PMA
Subpopulation
+: Higher contraceptive use
−: Lower contraceptive use
A: Other age group
F: Folk methods included
S−: Sterilization included
S+: Sterilization excluded
Married women
Sexually active women
Ever married/All women
Both sexes and husband/wives

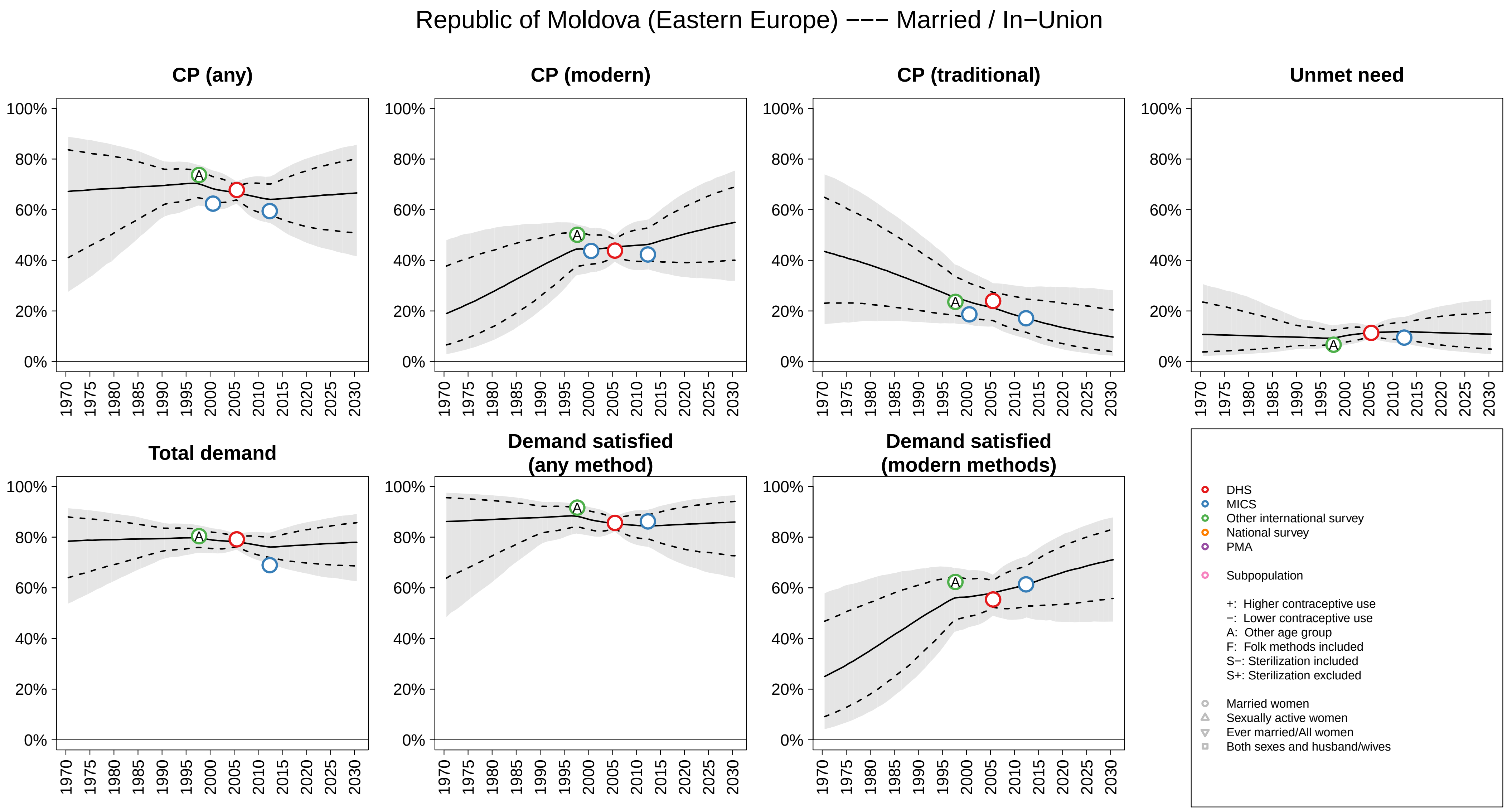

Republic of Moldova (Eastern Europe) --- Married / In−Union
CP (any)
CP (modern)
CP (traditional)
Unmet need
Total demand
Demand satisfied (any method)
Demand satisfied (modern methods)
DHS
MICS
Other international survey
National survey
PMA
Subpopulation
+: Higher contraceptive use
−: Lower contraceptive use
A: Other age group
F: Folk methods included
S−: Sterilization included
S+: Sterilization excluded
Married women
Sexually active women
Ever married/All women
Both sexes and husband/wives

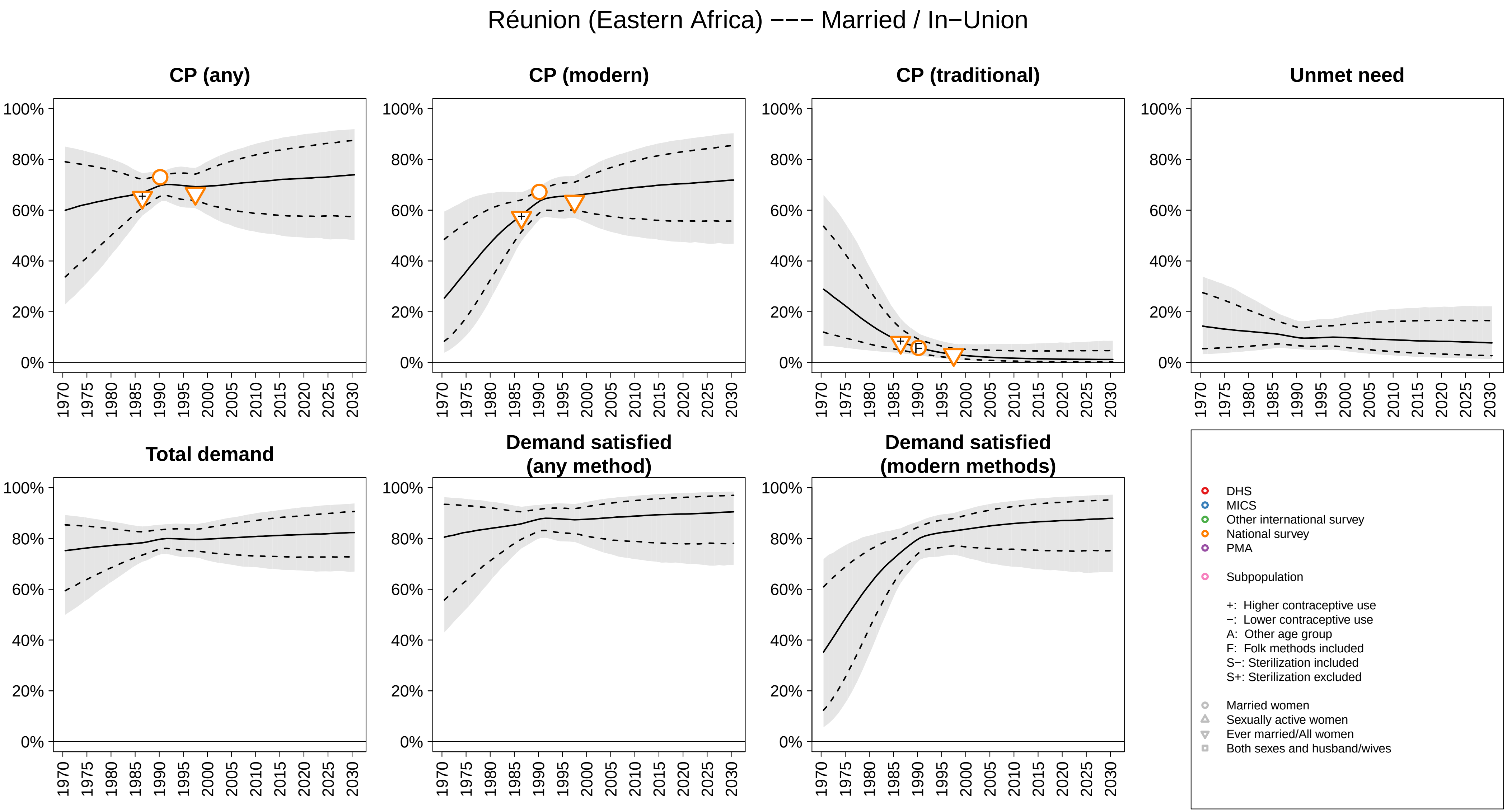
Réunion (Eastern Africa) −−− Married / In−Union
CP (any)
CP (modern)
CP (traditional)
Unmet need
Total demand
Demand satisfied (any method)
Demand satisfied (modern methods)
DHS
MICS
Other international survey
National survey
PMA
Subpopulation
+: Higher contraceptive use
−: Lower contraceptive use
A: Other age group
F: Folk methods included
S−: Sterilization included
S+: Sterilization excluded
Married women
Sexually active women
Ever married/All women
Both sexes and husband/wives

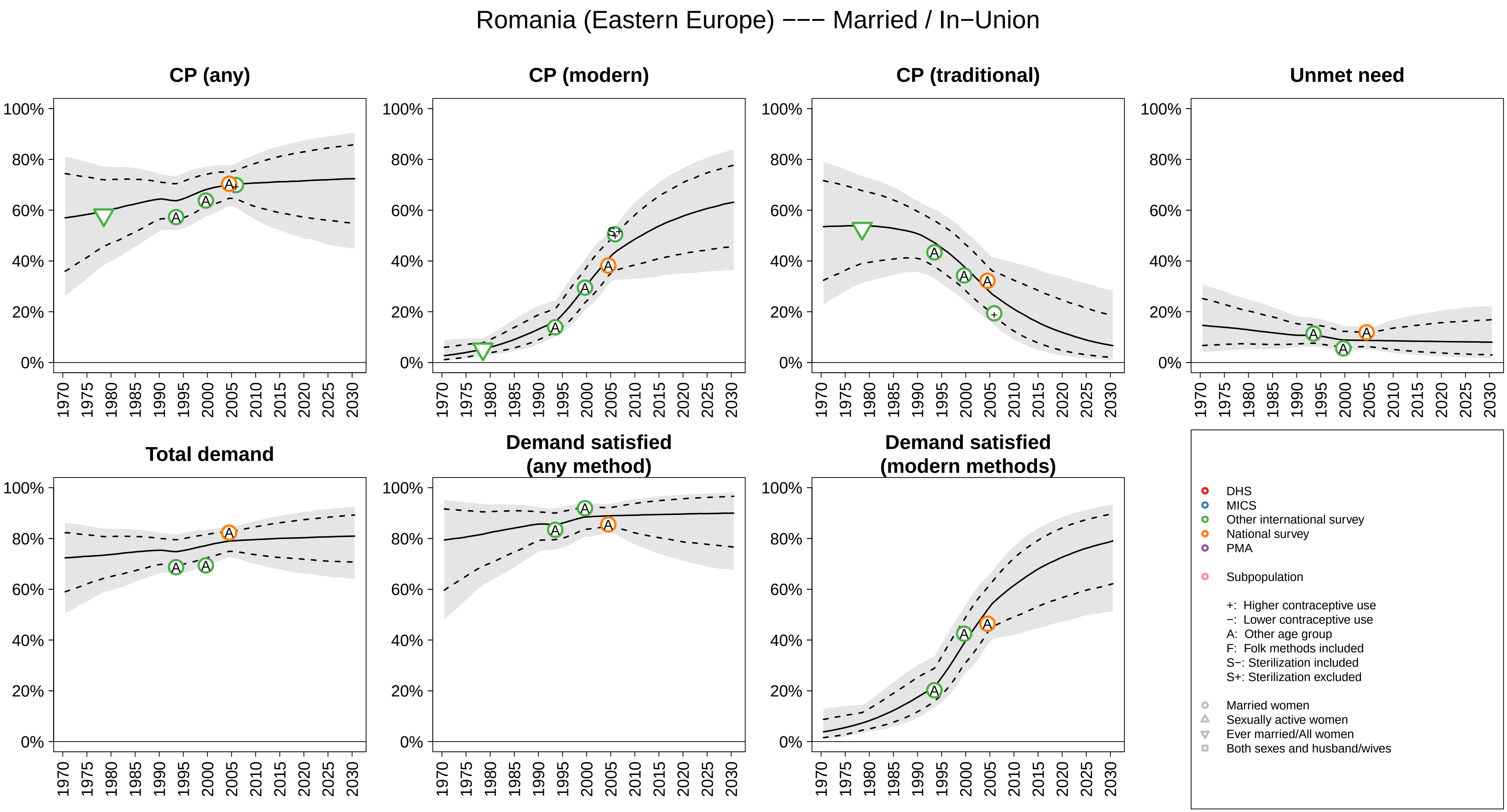
Romania (Eastern Europe) −−− Married / In−Union
CP (any)
CP (modern)
CP (traditional)
Unmet need
Total demand
Demand satisfied (any method)
Demand satisfied (modern methods)
DHS
MICS
Other international survey
National survey
PMA
Subpopulation
+: Higher contraceptive use
−: Lower contraceptive use
A: Other age group
F: Folk methods included
S−: Sterilization included
S+: Sterilization excluded
Married women
Sexually active women
Ever married/All women
Both sexes and husband/wives

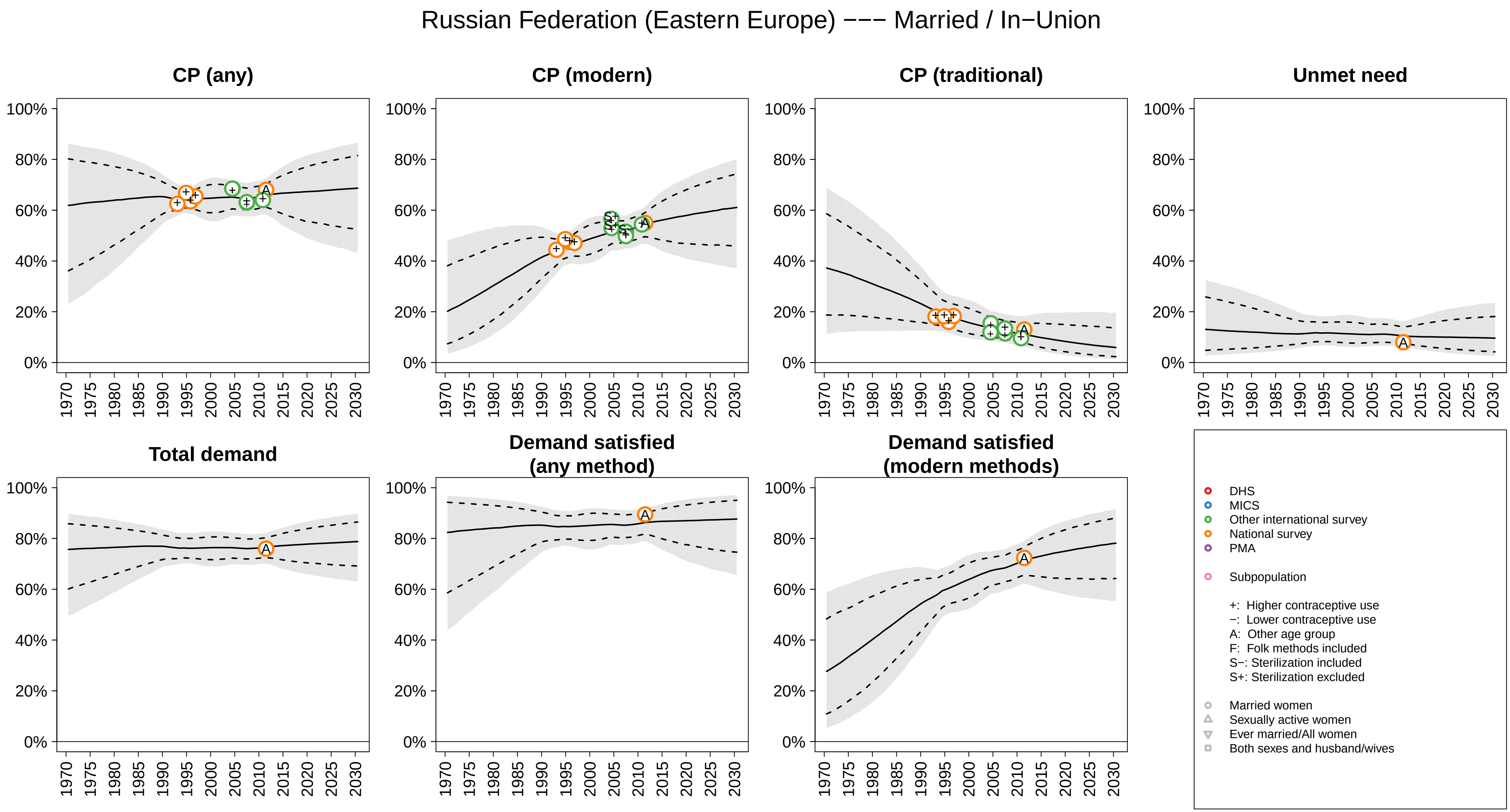
Russian Federation (Eastern Europe) −−− Married / In−Union
CP (any)
CP (modern)
CP (traditional)
Unmet need
Total demand
Demand satisfied (any method)
Demand satisfied (modern methods)
DHS
MICS
Other international survey
National survey
PMA
Subpopulation
+: Higher contraceptive use
−: Lower contraceptive use
A: Other age group
F: Folk methods included
S−: Sterilization included
S+: Sterilization excluded
Married women
Sexually active women
Ever married/All women
Both sexes and husband/wives

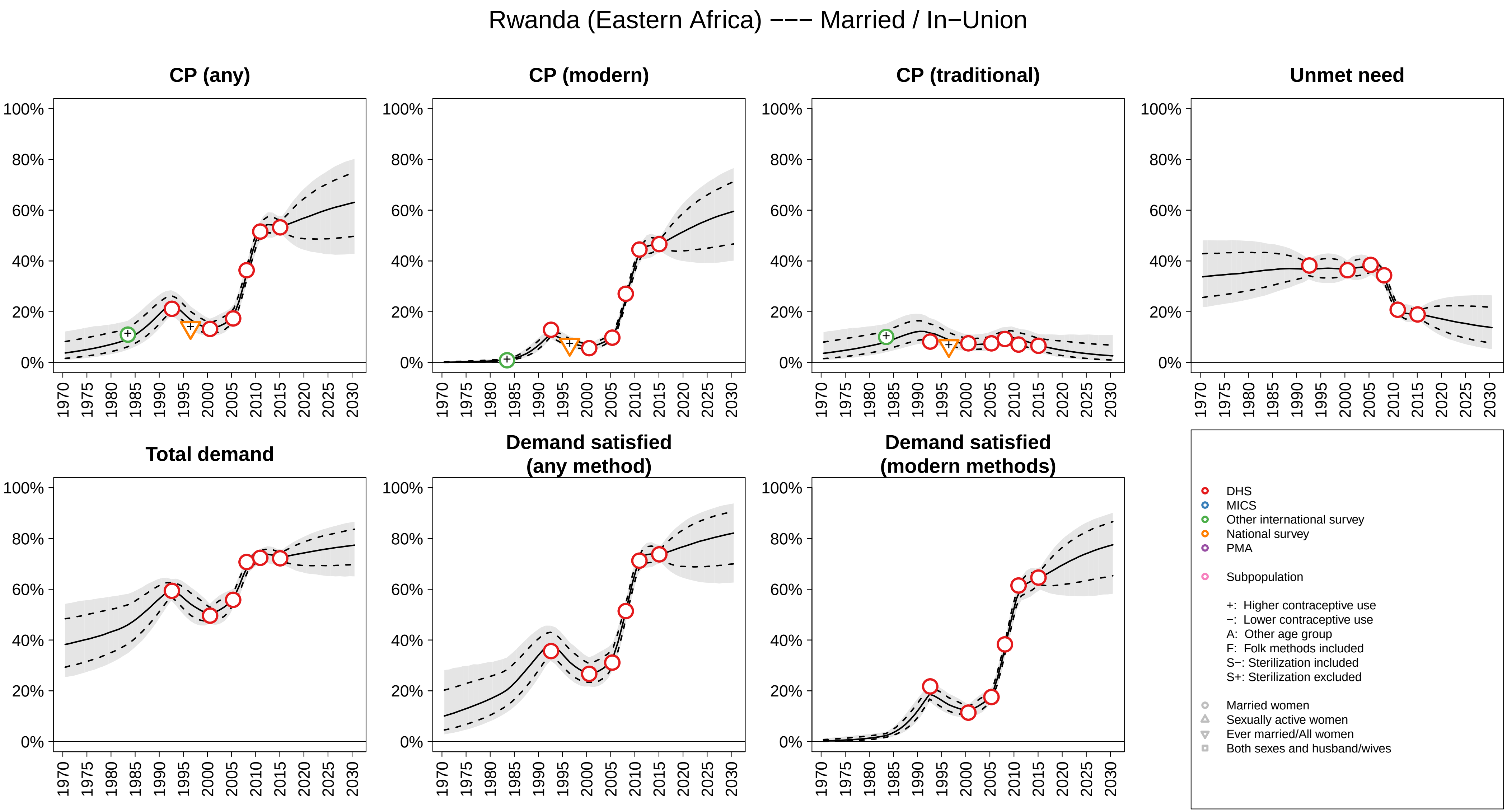

Rwanda (Eastern Africa) --- Married / In−Union
CP (any)
CP (modern)
CP (traditional)
Unmet need
Total demand
Demand satisfied (any method)
Demand satisfied (modern methods)
0%
20%
40%
60%
80%
100%
1970
1975
1980
1985
1990
1995
2000
2005
2010
2015
2020
2025
2030
DHS
MICS
Other international survey
National survey
PMA
Subpopulation
+: Higher contraceptive use
−: Lower contraceptive use
A: Other age group
F: Folk methods included
S−: Sterilization included
S+: Sterilization excluded
Married women
Sexually active women
Ever married/All women
Both sexes and husband/wives

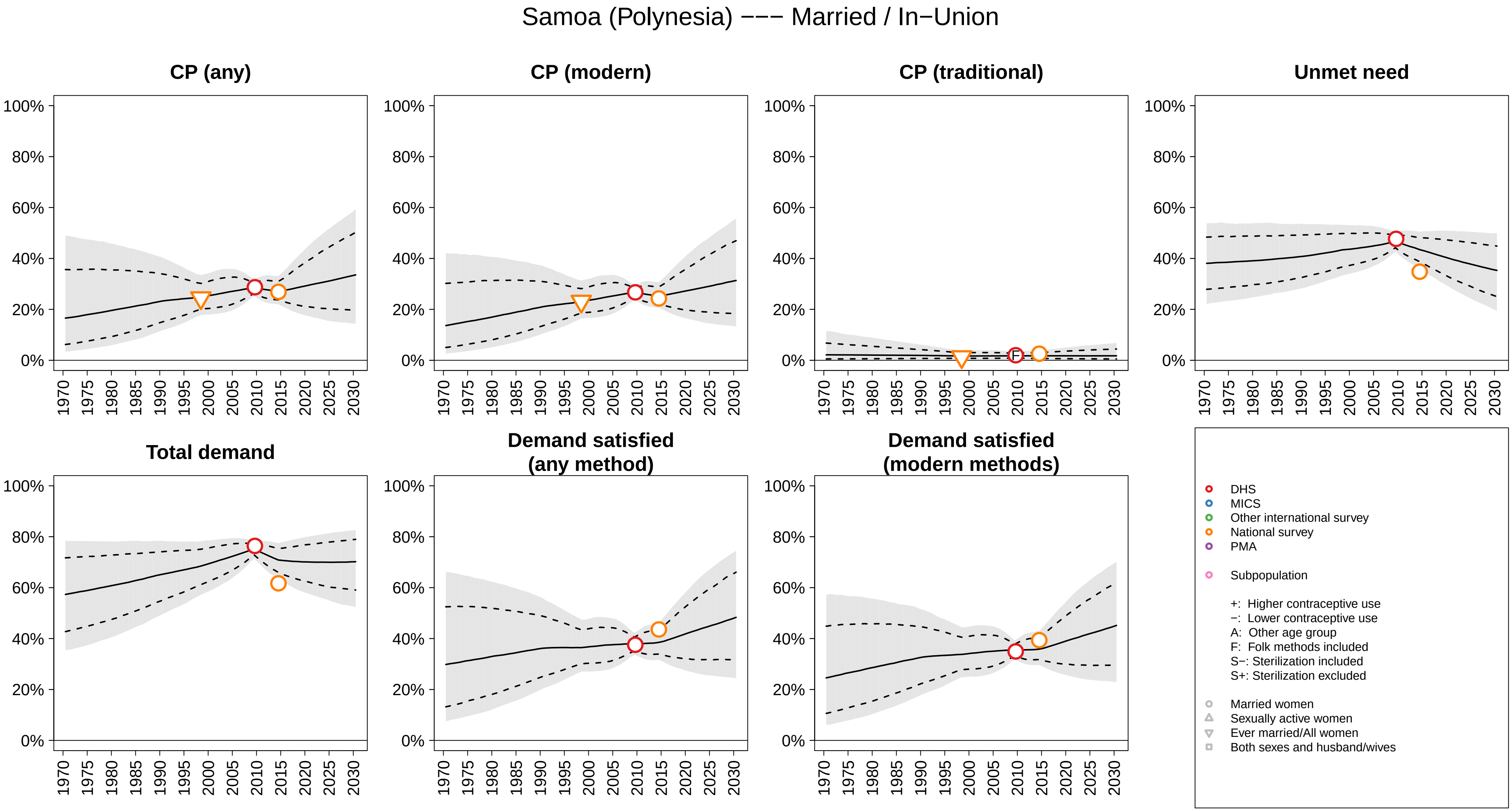

Samoa (Polynesia) ––– Married / In−Union
CP (any)
CP (modern)
CP (traditional)
Unmet need
Total demand
Demand satisfied (any method)
Demand satisfied (modern methods)
100%
80%
60%
40%
20%
0%
1970 1975 1980 1985 1990 1995 2000 2005 2010 2015 2020 2025 2030
F
DHS
MICS
Other international survey
National survey
PMA
Subpopulation
+: Higher contraceptive use
−: Lower contraceptive use
A: Other age group
F: Folk methods included
S−: Sterilization included
S+: Sterilization excluded
Married women
Sexually active women
Ever married/All women
Both sexes and husband/wives

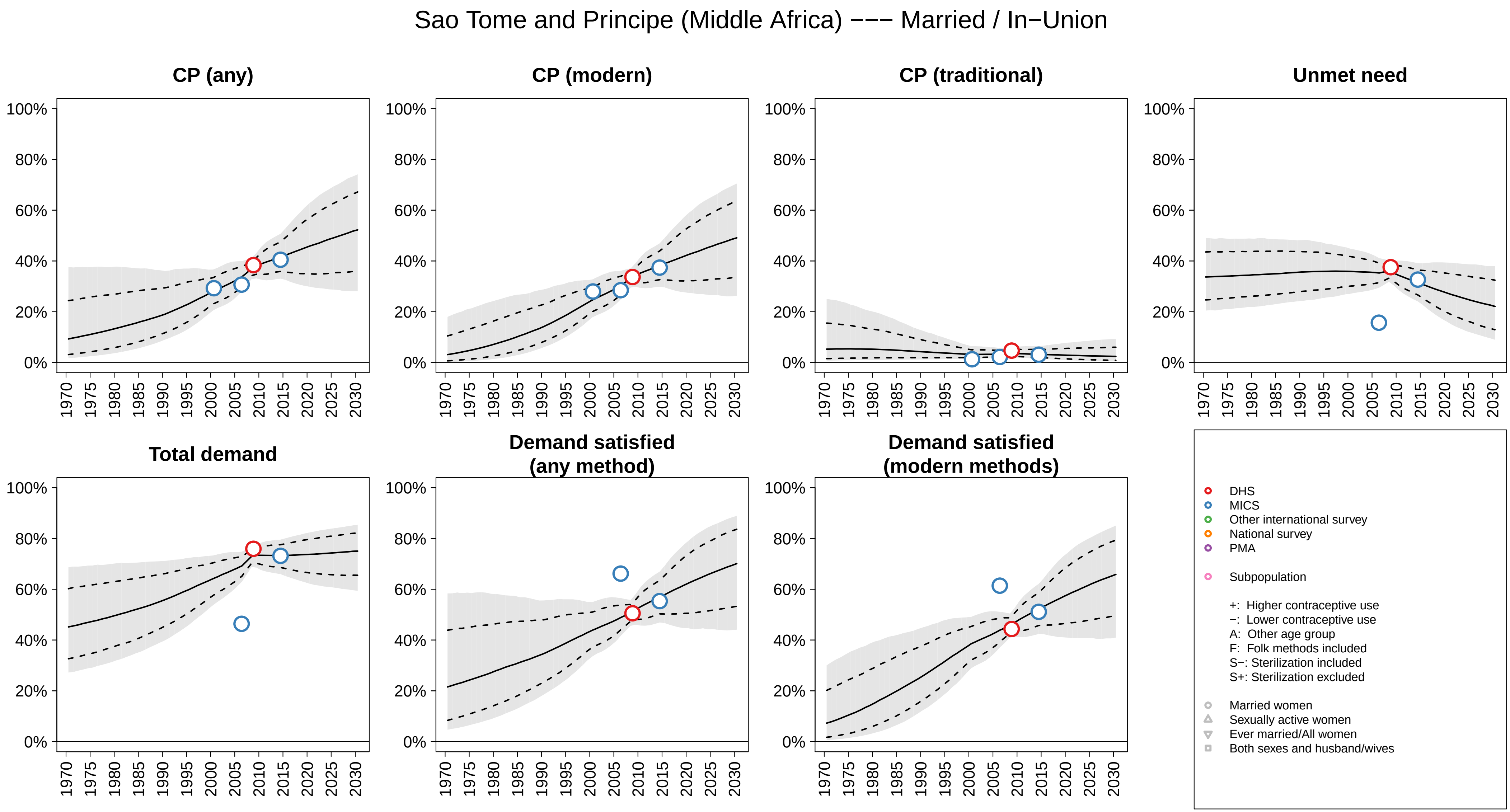
Sao Tome and Principe (Middle Africa) --- Married / In−Union
CP (any)
CP (modern)
CP (traditional)
Unmet need
Total demand
Demand satisfied (any method)
Demand satisfied (modern methods)
100%
80%
60%
40%
20%
0%
1970 1975 1980 1985 1990 1995 2000 2005 2010 2015 2020 2025 2030
DHS
MICS
Other international survey
National survey
PMA
Subpopulation
+: Higher contraceptive use
−: Lower contraceptive use
A: Other age group
F: Folk methods included
S−: Sterilization included
S+: Sterilization excluded
Married women
Sexually active women
Ever married/All women
Both sexes and husband/wives

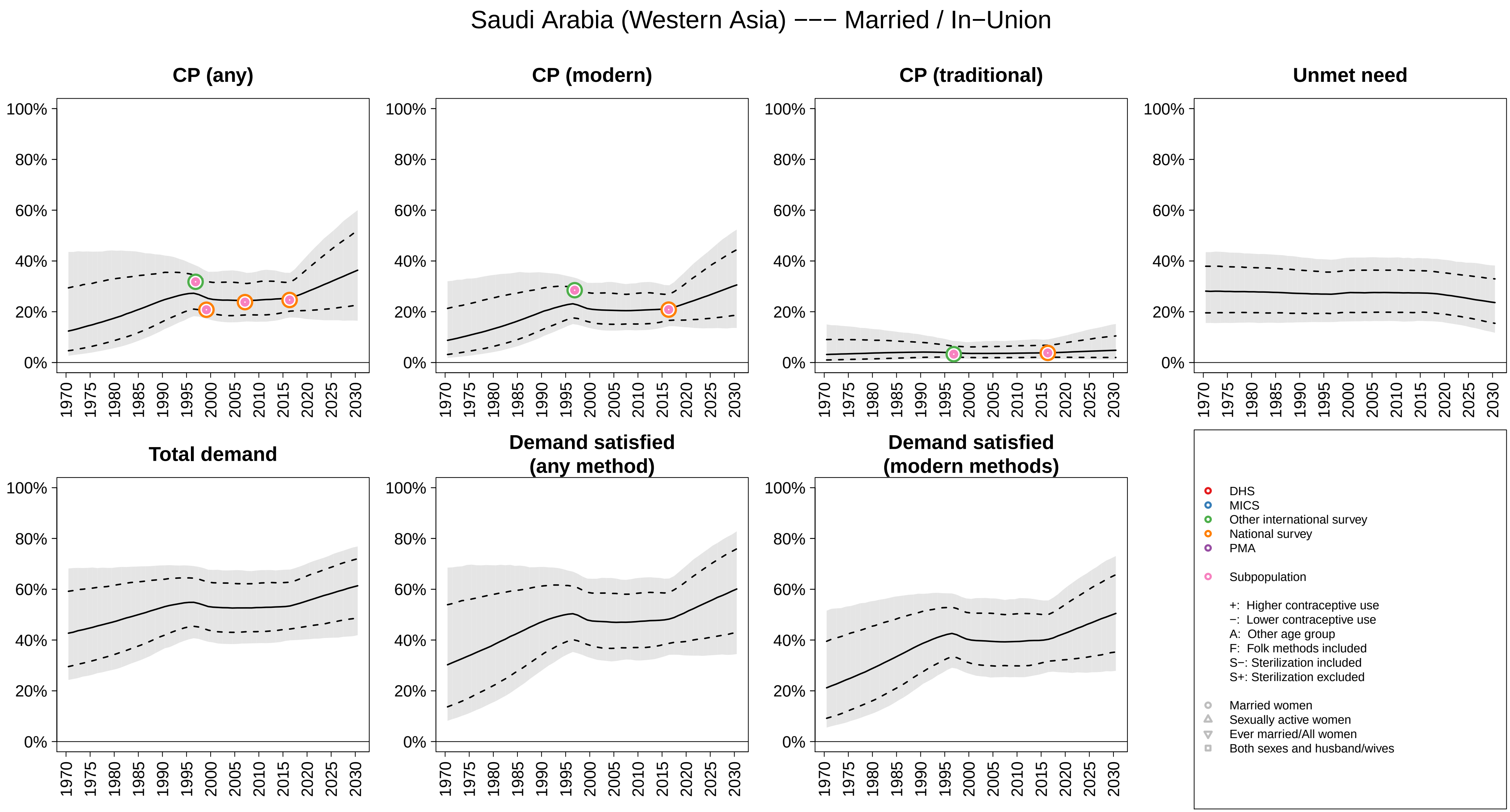
Saudi Arabia (Western Asia) −−− Married / In−Union
CP (any)
CP (modern)
CP (traditional)
Unmet need
Total demand
Demand satisfied (any method)
Demand satisfied (modern methods)
0%
20%
40%
60%
80%
100%
1970
1975
1980
1985
1990
1995
2000
2005
2010
2015
2020
2025
2030
DHS
MICS
Other international survey
National survey
PMA
Subpopulation
+: Higher contraceptive use
−: Lower contraceptive use
A: Other age group
F: Folk methods included
S−: Sterilization included
S+: Sterilization excluded
Married women
Sexually active women
Ever married/All women
Both sexes and husband/wives

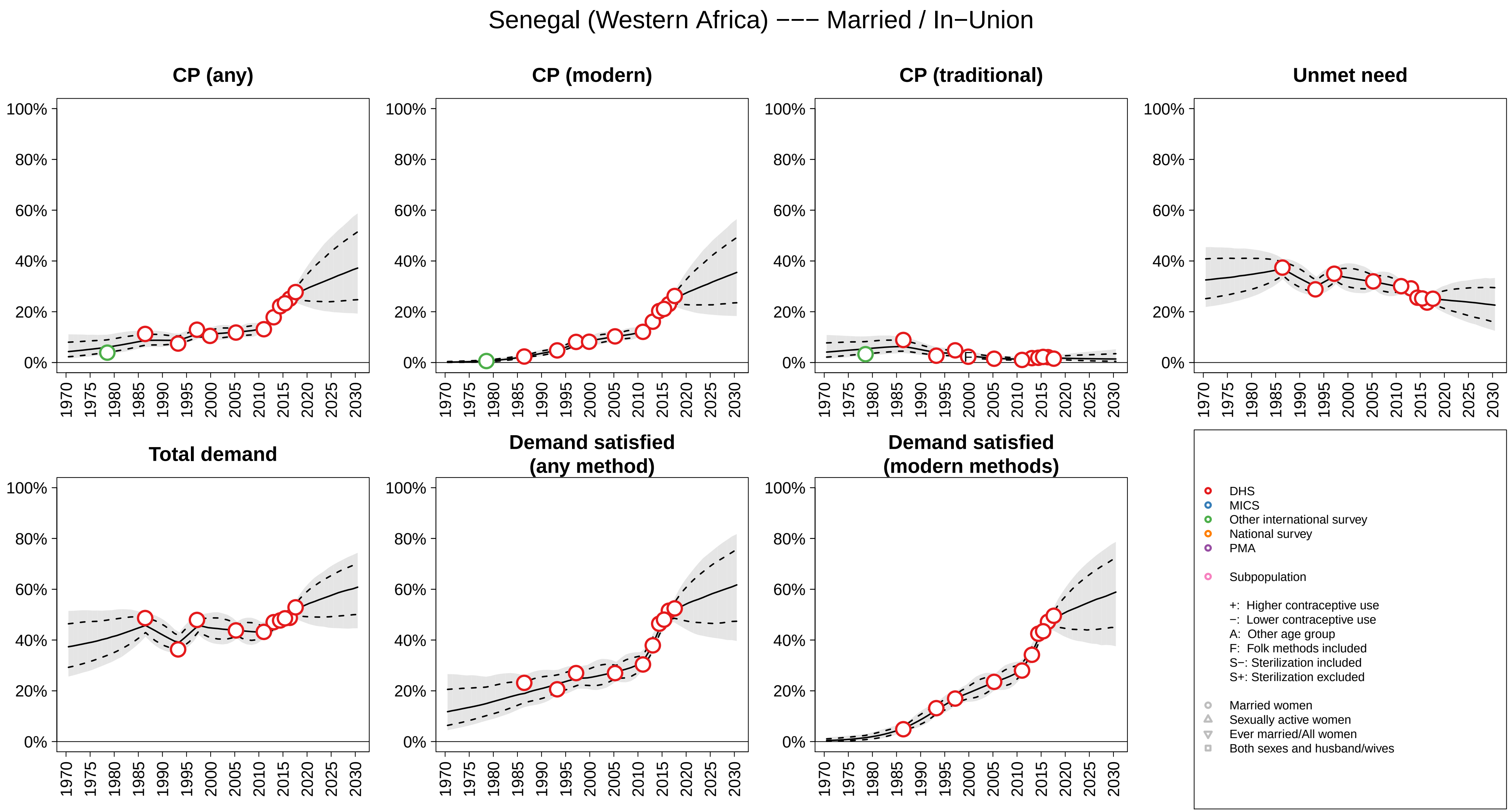
Senegal (Western Africa) −−− Married / In−Union
CP (any)
CP (modern)
CP (traditional)
Unmet need
Total demand
Demand satisfied (any method)
Demand satisfied (modern methods)
0%
20%
40%
60%
80%
100%
1970
1975
1980
1985
1990
1995
2000
2005
2010
2015
2020
2025
2030
F
DHS
MICS
Other international survey
National survey
PMA
Subpopulation
+: Higher contraceptive use
−: Lower contraceptive use
A: Other age group
F: Folk methods included
S−: Sterilization included
S+: Sterilization excluded
Married women
Sexually active women
Ever married/All women
Both sexes and husband/wives

Serbia (Southern Europe) −−− Married / In−Union

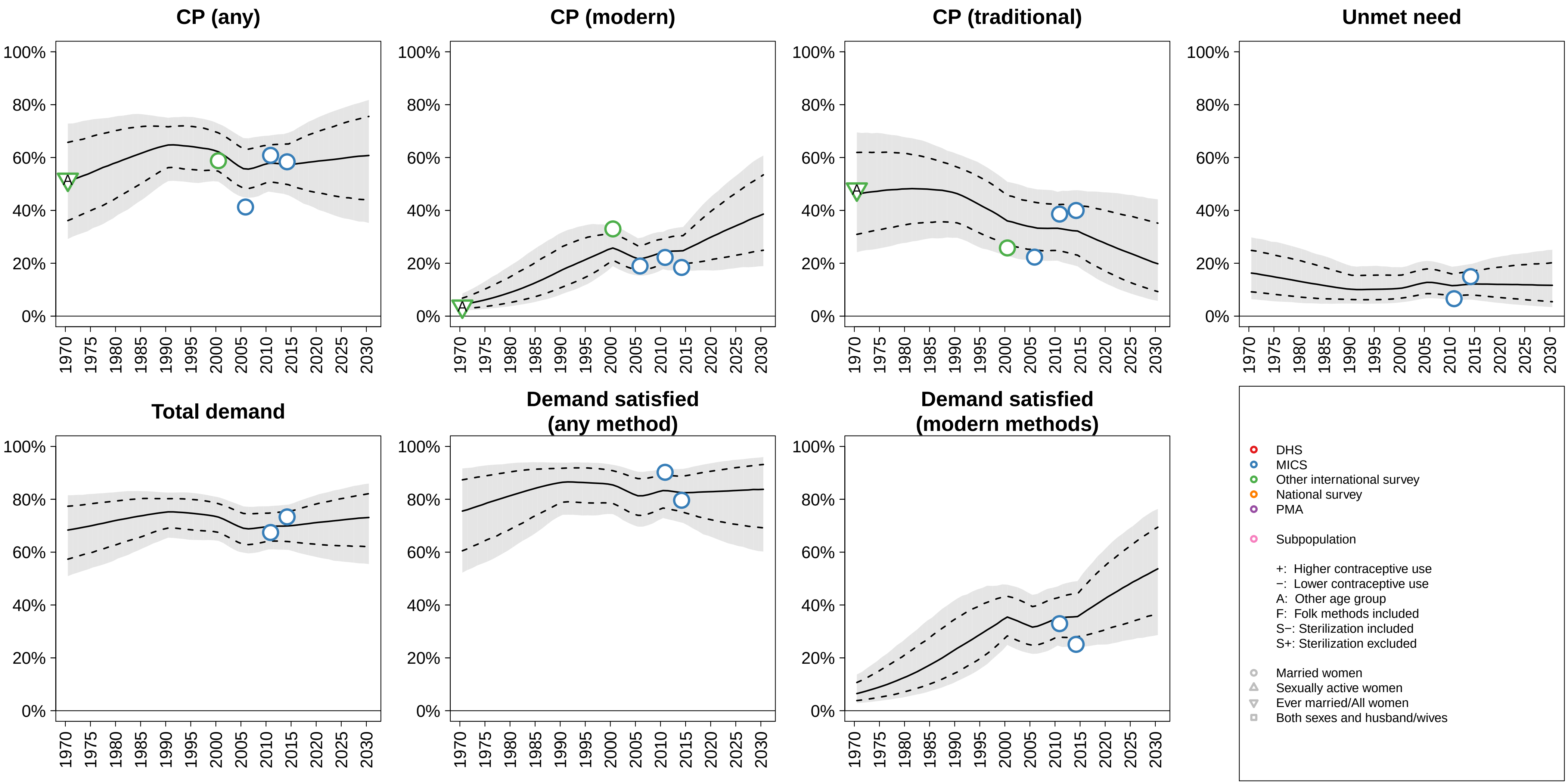

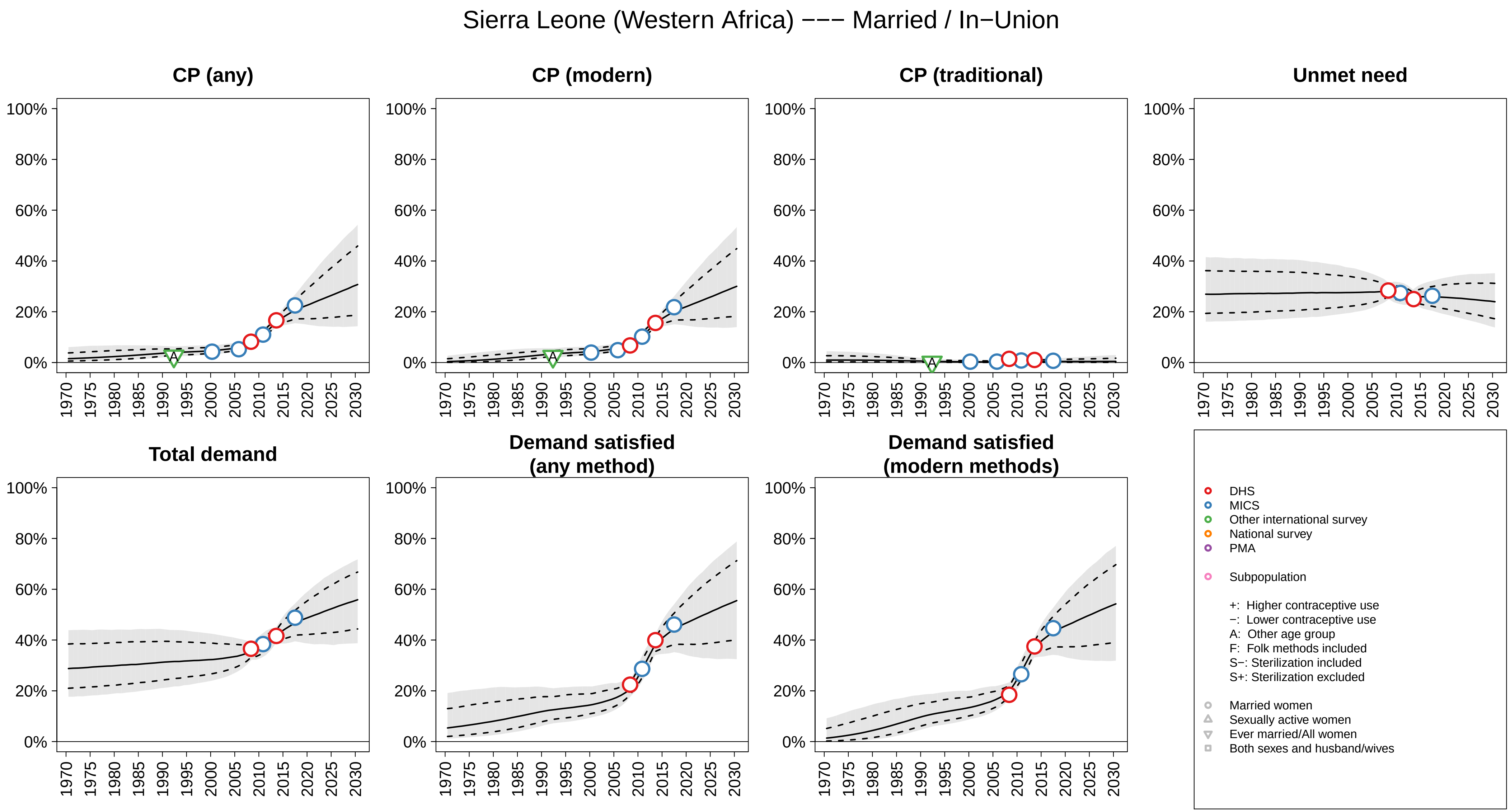
Sierra Leone (Western Africa) −−− Married / In−Union
CP (any)
CP (modern)
CP (traditional)
Unmet need
Total demand
Demand satisfied (any method)
Demand satisfied (modern methods)
0%
20%
40%
60%
80%
100%
1970
1975
1980
1985
1990
1995
2000
2005
2010
2015
2020
2025
2030
A
DHS
MICS
Other international survey
National survey
PMA
Subpopulation
+: Higher contraceptive use
−: Lower contraceptive use
A: Other age group
F: Folk methods included
S−: Sterilization included
S+: Sterilization excluded
Married women
Sexually active women
Ever married/All women
Both sexes and husband/wives

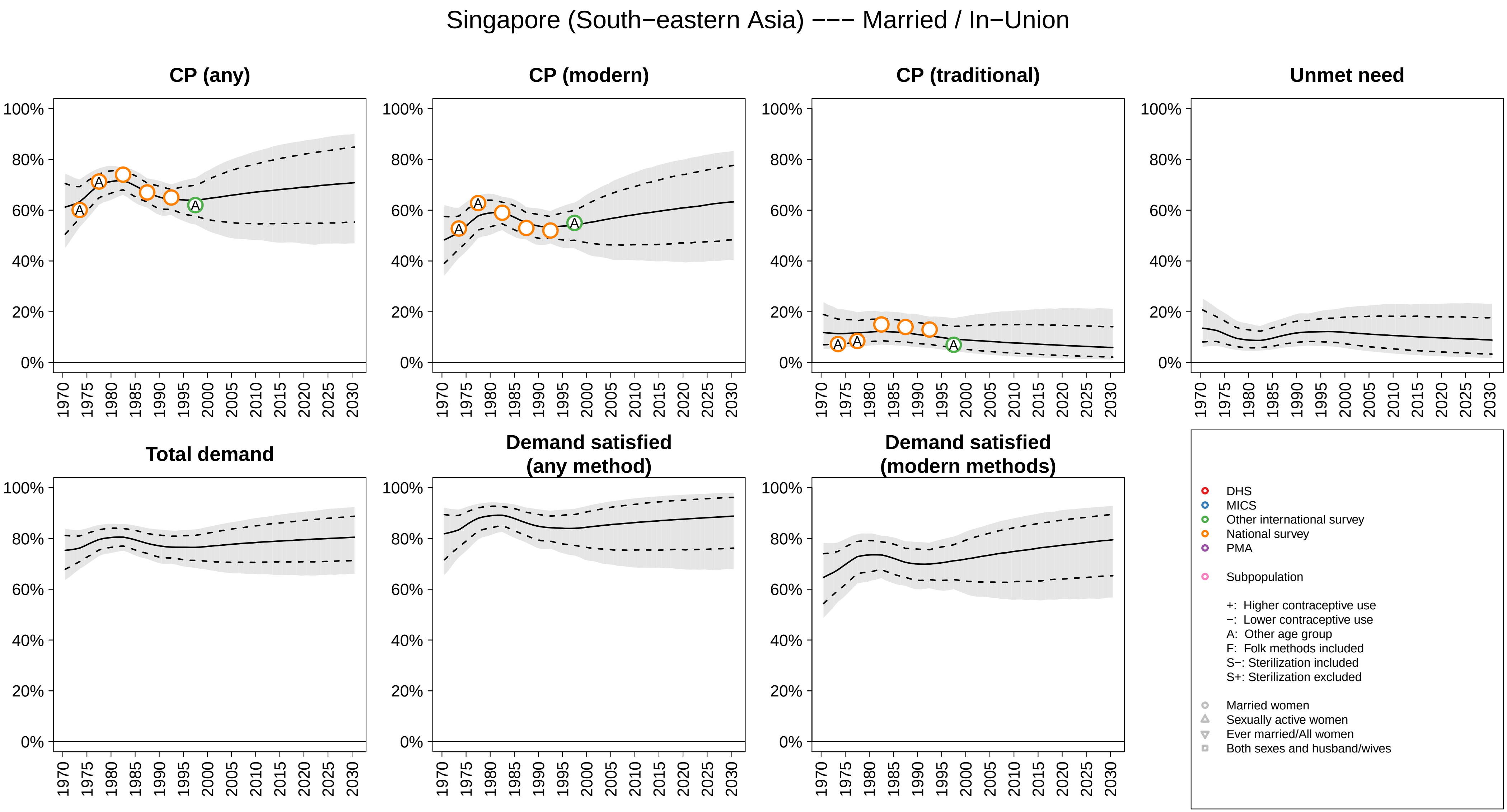
Singapore (South−eastern Asia) −−− Married / In−Union
CP (any)
CP (modern)
CP (traditional)
Unmet need
Total demand
Demand satisfied (any method)
Demand satisfied (modern methods)
0%
20%
40%
60%
80%
100%
1970
1975
1980
1985
1990
1995
2000
2005
2010
2015
2020
2025
2030
A
DHS
MICS
Other international survey
National survey
PMA
Subpopulation
+: Higher contraceptive use
−: Lower contraceptive use
A: Other age group
F: Folk methods included
S−: Sterilization included
S+: Sterilization excluded
Married women
Sexually active women
Ever married/All women
Both sexes and husband/wives

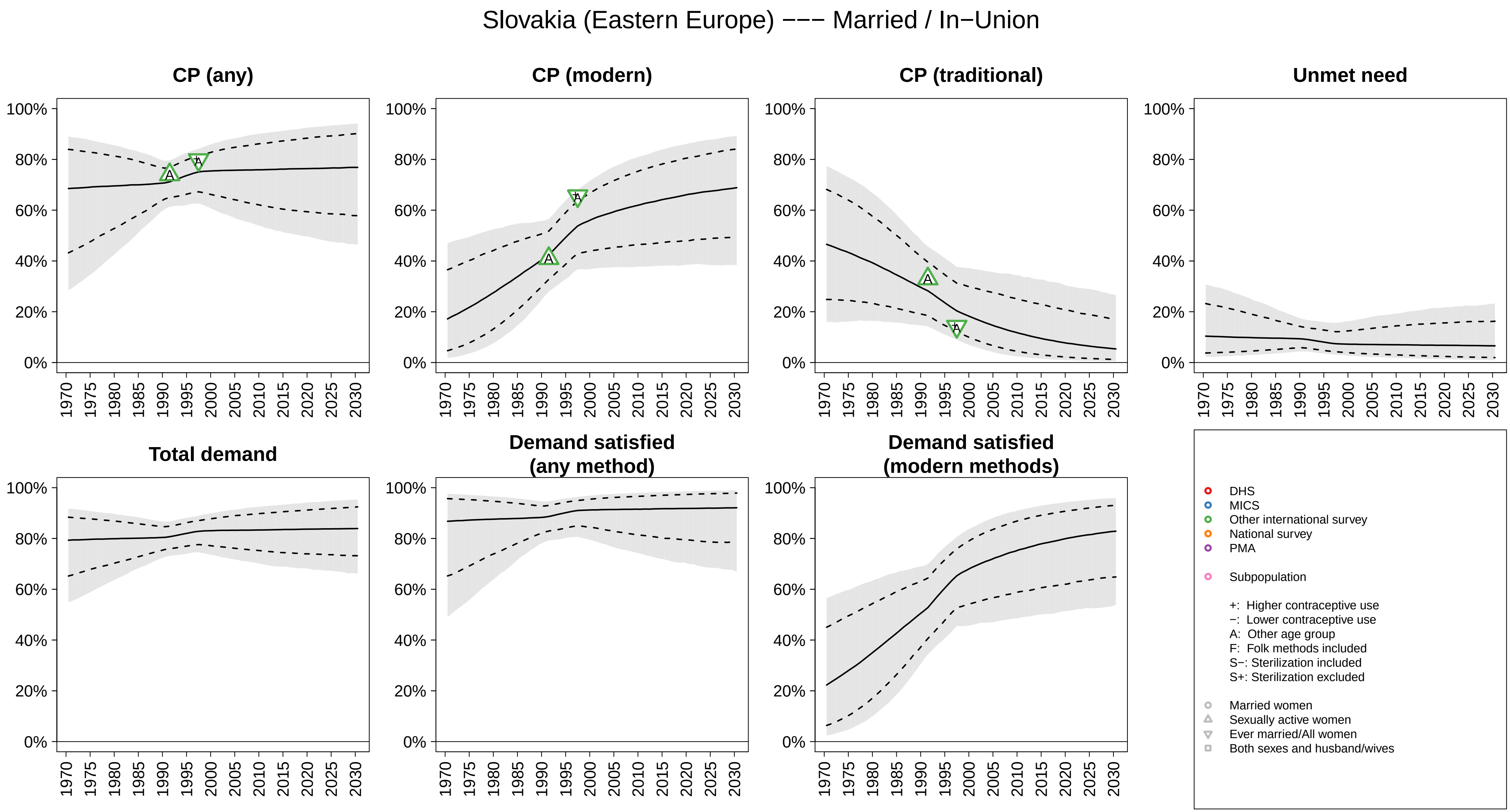
Slovakia (Eastern Europe) --- Married / In−Union
CP (any)
CP (modern)
CP (traditional)
Unmet need
Total demand
Demand satisfied (any method)
Demand satisfied (modern methods)
DHS
MICS
Other international survey
National survey
PMA
Subpopulation
+: Higher contraceptive use
−: Lower contraceptive use
A: Other age group
F: Folk methods included
S−: Sterilization included
S+: Sterilization excluded
Married women
Sexually active women
Ever married/All women
Both sexes and husband/wives

Slovenia (Southern Europe) −−− Married / In−Union

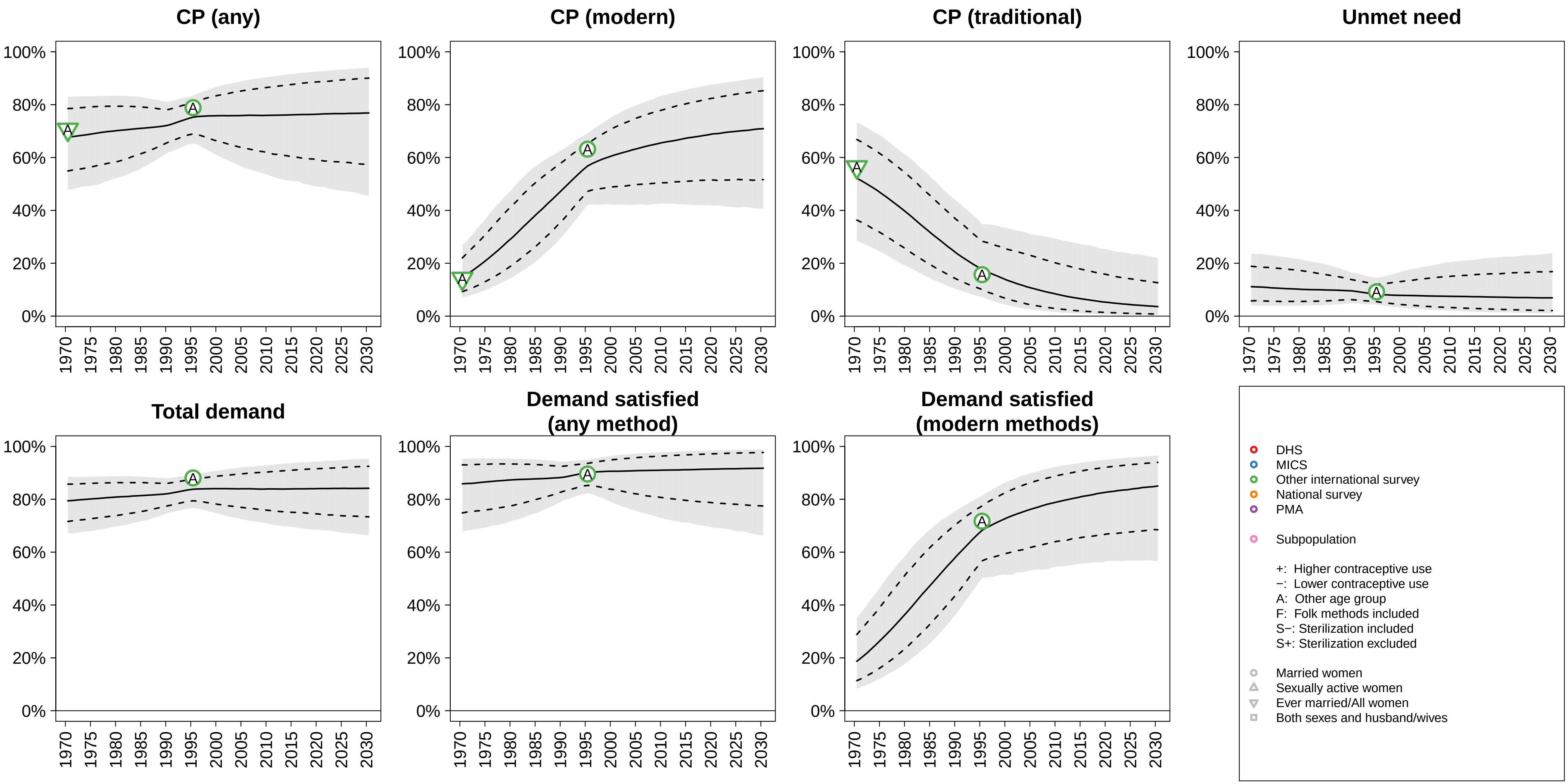

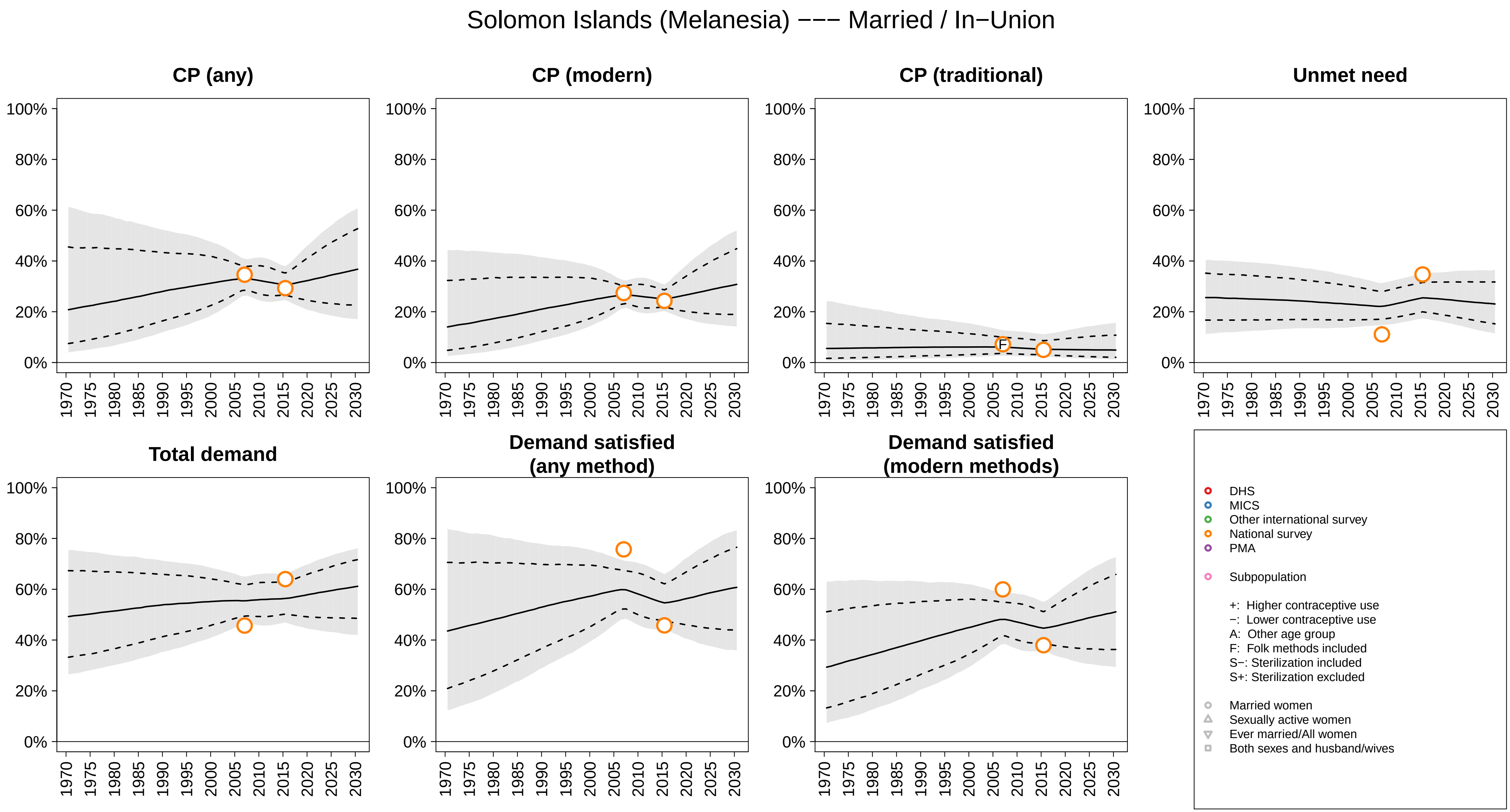

Solomon Islands (Melanesia) --- Married / In−Union
CP (any)
CP (modern)
CP (traditional)
Unmet need
Total demand
Demand satisfied (any method)
Demand satisfied (modern methods)
0%
20%
40%
60%
80%
100%
1970
1975
1980
1985
1990
1995
2000
2005
2010
2015
2020
2025
2030
F
DHS
MICS
Other international survey
National survey
PMA
Subpopulation
+: Higher contraceptive use
−: Lower contraceptive use
A: Other age group
F: Folk methods included
S−: Sterilization included
S+: Sterilization excluded
Married women
Sexually active women
Ever married/All women
Both sexes and husband/wives

Somalia (Eastern Africa) −−− Married / In−Union

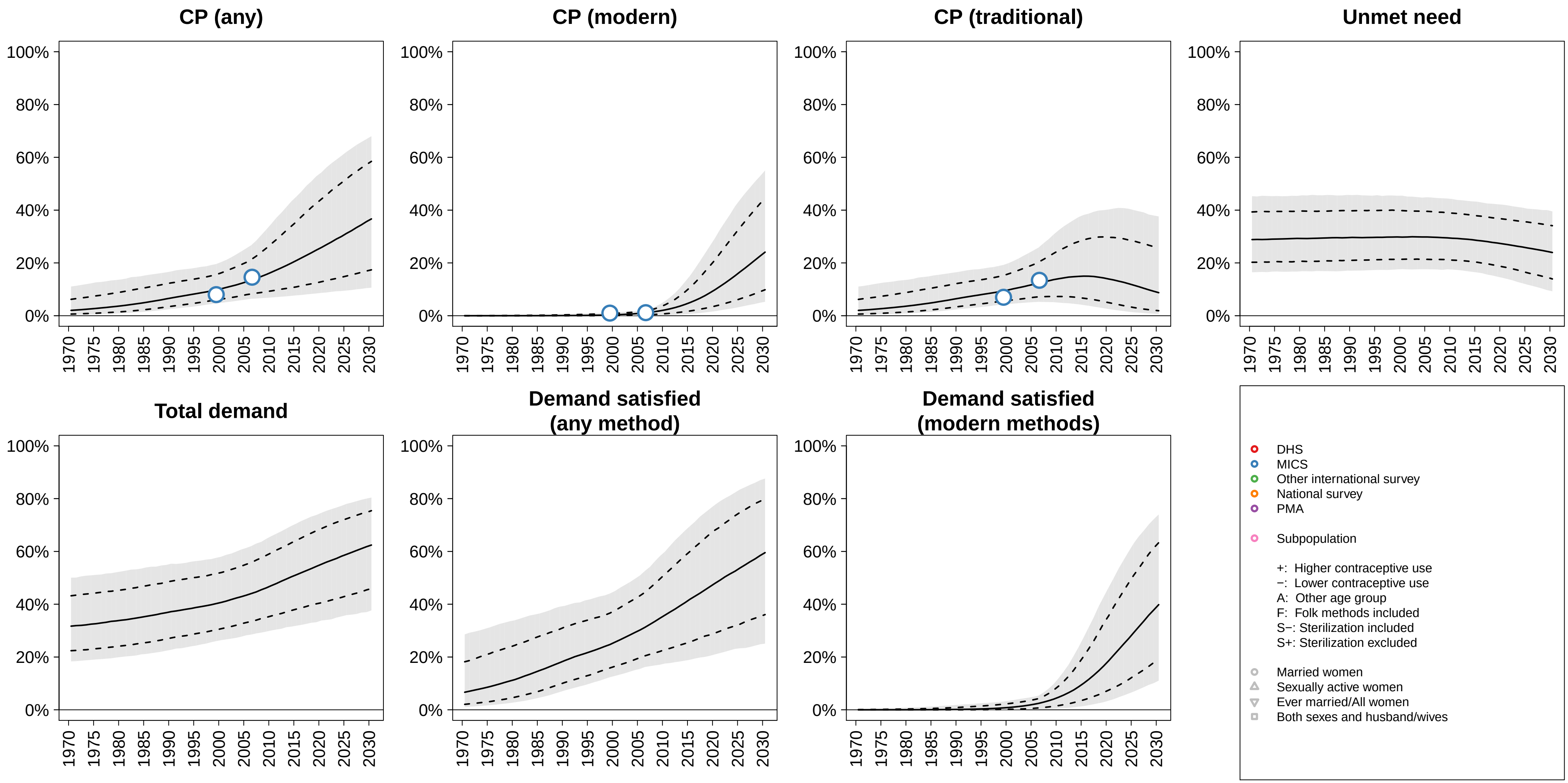

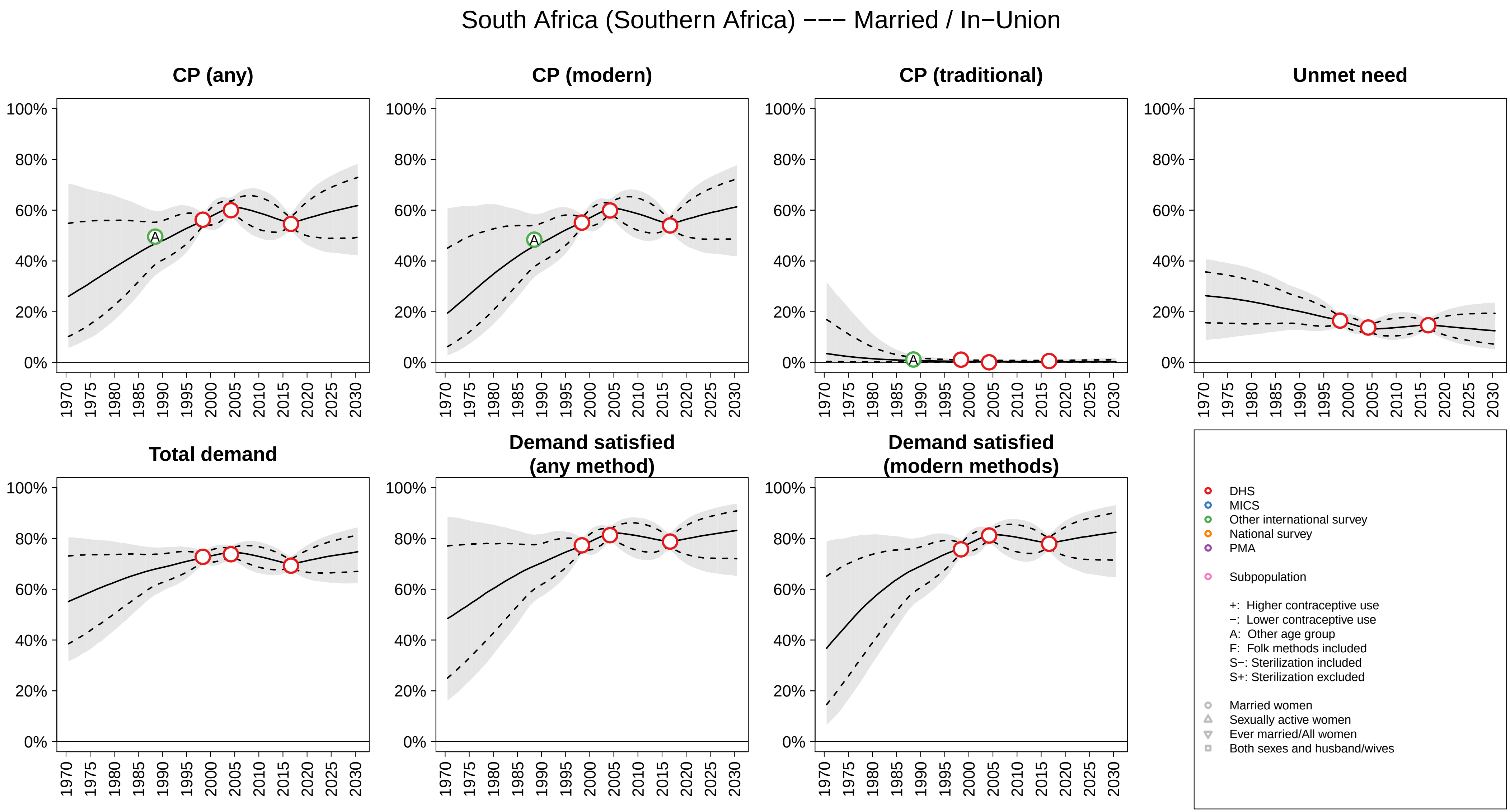
South Africa (Southern Africa) −−− Married / In−Union
CP (any)
CP (modern)
CP (traditional)
Unmet need
Total demand
Demand satisfied (any method)
Demand satisfied (modern methods)
DHS
MICS
Other international survey
National survey
PMA
Subpopulation
+: Higher contraceptive use
−: Lower contraceptive use
A: Other age group
F: Folk methods included
S−: Sterilization included
S+: Sterilization excluded
Married women
Sexually active women
Ever married/All women
Both sexes and husband/wives

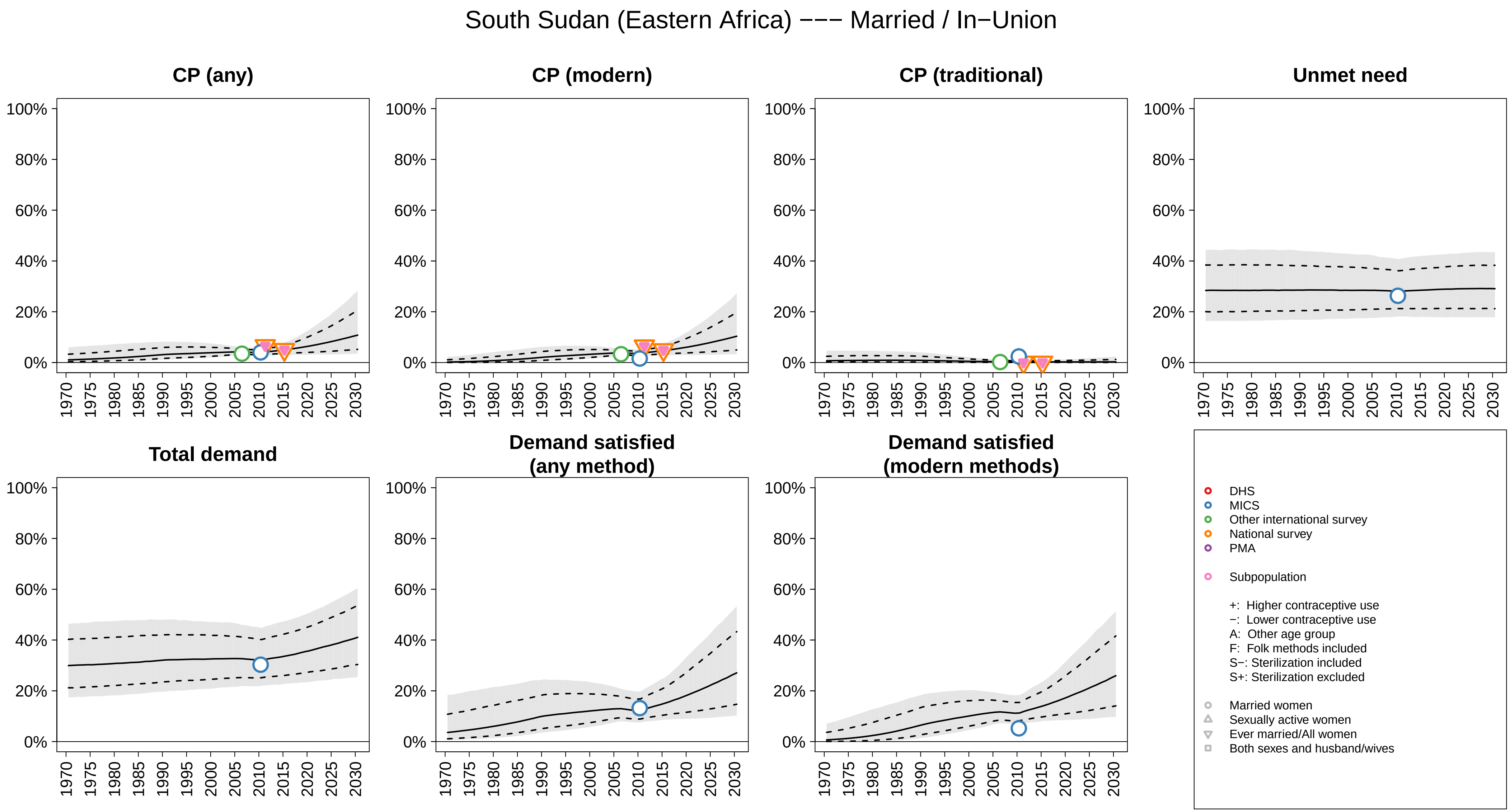
South Sudan (Eastern Africa) −−− Married / In−Union
CP (any)
CP (modern)
CP (traditional)
Unmet need
Total demand
Demand satisfied (any method)
Demand satisfied (modern methods)
0%
20%
40%
60%
80%
100%
1970
1975
1980
1985
1990
1995
2000
2005
2010
2015
2020
2025
2030
DHS
MICS
Other international survey
National survey
PMA
Subpopulation
+: Higher contraceptive use
−: Lower contraceptive use
A: Other age group
F: Folk methods included
S−: Sterilization included
S+: Sterilization excluded
Married women
Sexually active women
Ever married/All women
Both sexes and husband/wives

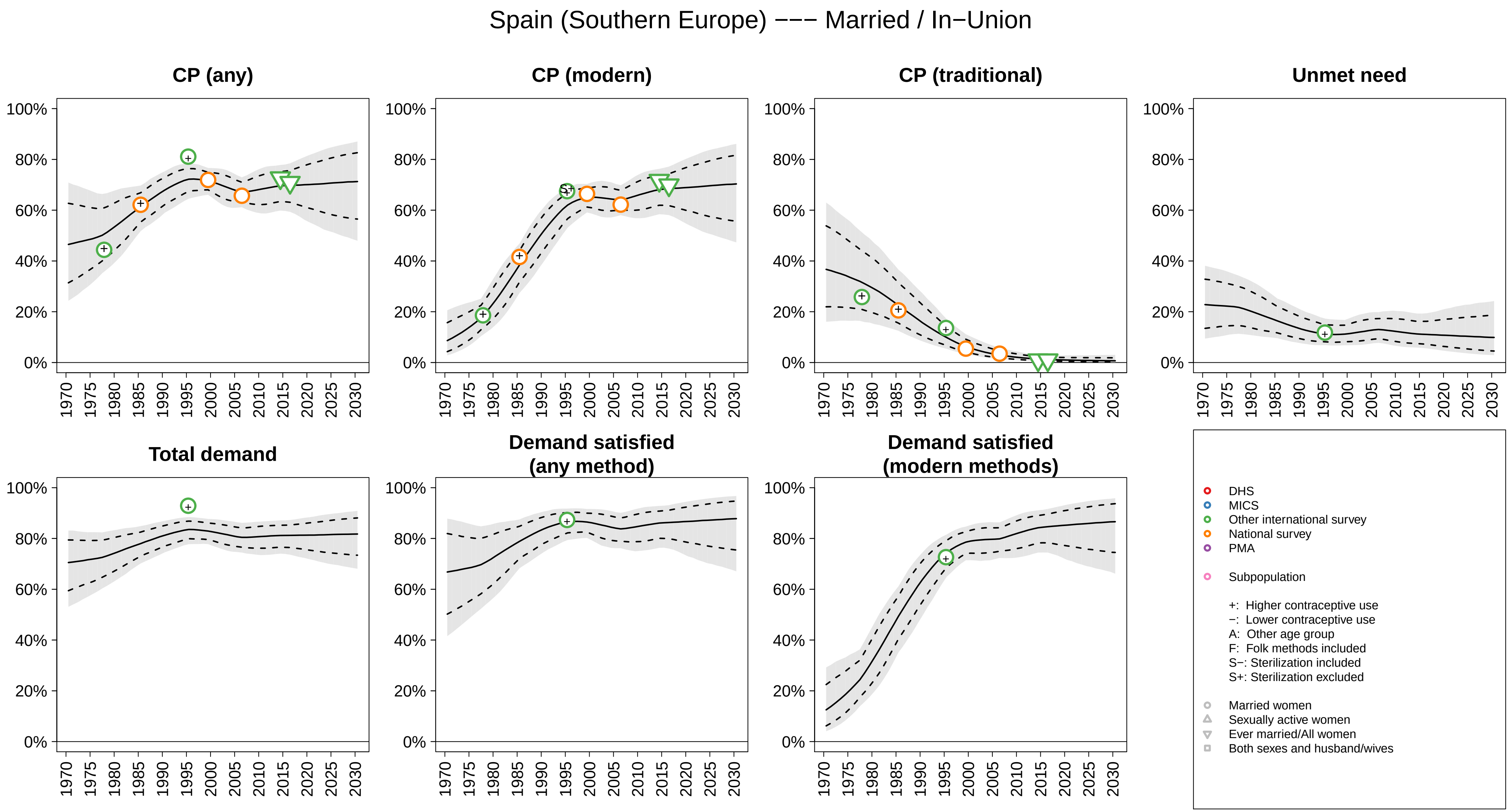
Spain (Southern Europe) −−− Married / In−Union
CP (any)
CP (modern)
CP (traditional)
Unmet need
Total demand
Demand satisfied (any method)
Demand satisfied (modern methods)
0%
20%
40%
60%
80%
100%
1970
1975
1980
1985
1990
1995
2000
2005
2010
2015
2020
2025
2030
DHS
MICS
Other international survey
National survey
PMA
Subpopulation
+: Higher contraceptive use
−: Lower contraceptive use
A: Other age group
F: Folk methods included
S−: Sterilization included
S+: Sterilization excluded
Married women
Sexually active women
Ever married/All women
Both sexes and husband/wives

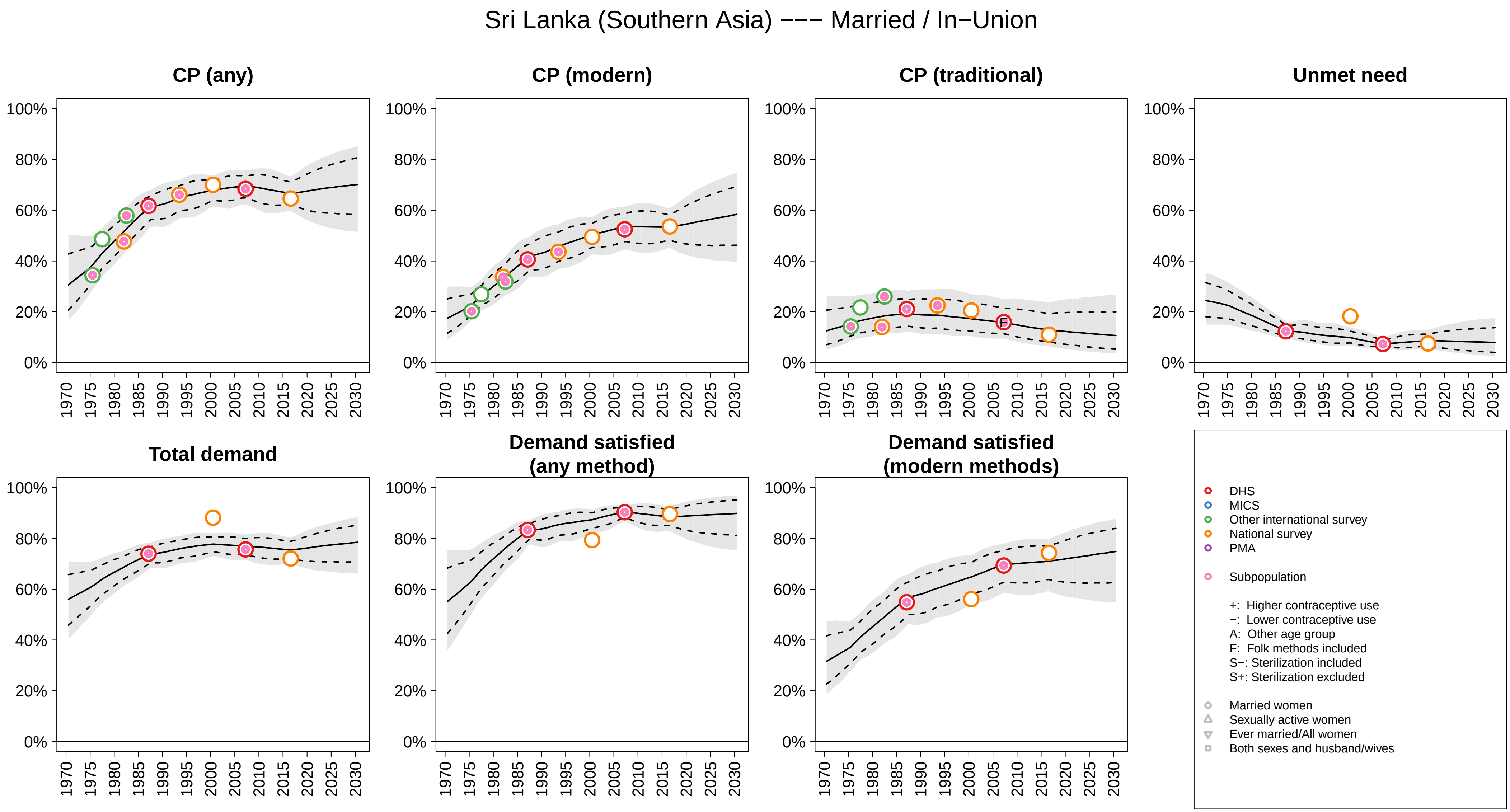
Sri Lanka (Southern Asia) −−− Married / In−Union
CP (any)
CP (modern)
CP (traditional)
Unmet need
Total demand
Demand satisfied (any method)
Demand satisfied (modern methods)
100%
80%
60%
40%
20%
0%
1970 1975 1980 1985 1990 1995 2000 2005 2010 2015 2020 2025 2030
F
DHS
MICS
Other international survey
National survey
PMA
Subpopulation
+: Higher contraceptive use
−: Lower contraceptive use
A: Other age group
F: Folk methods included
S−: Sterilization included
S+: Sterilization excluded
Married women
Sexually active women
Ever married/All women
Both sexes and husband/wives

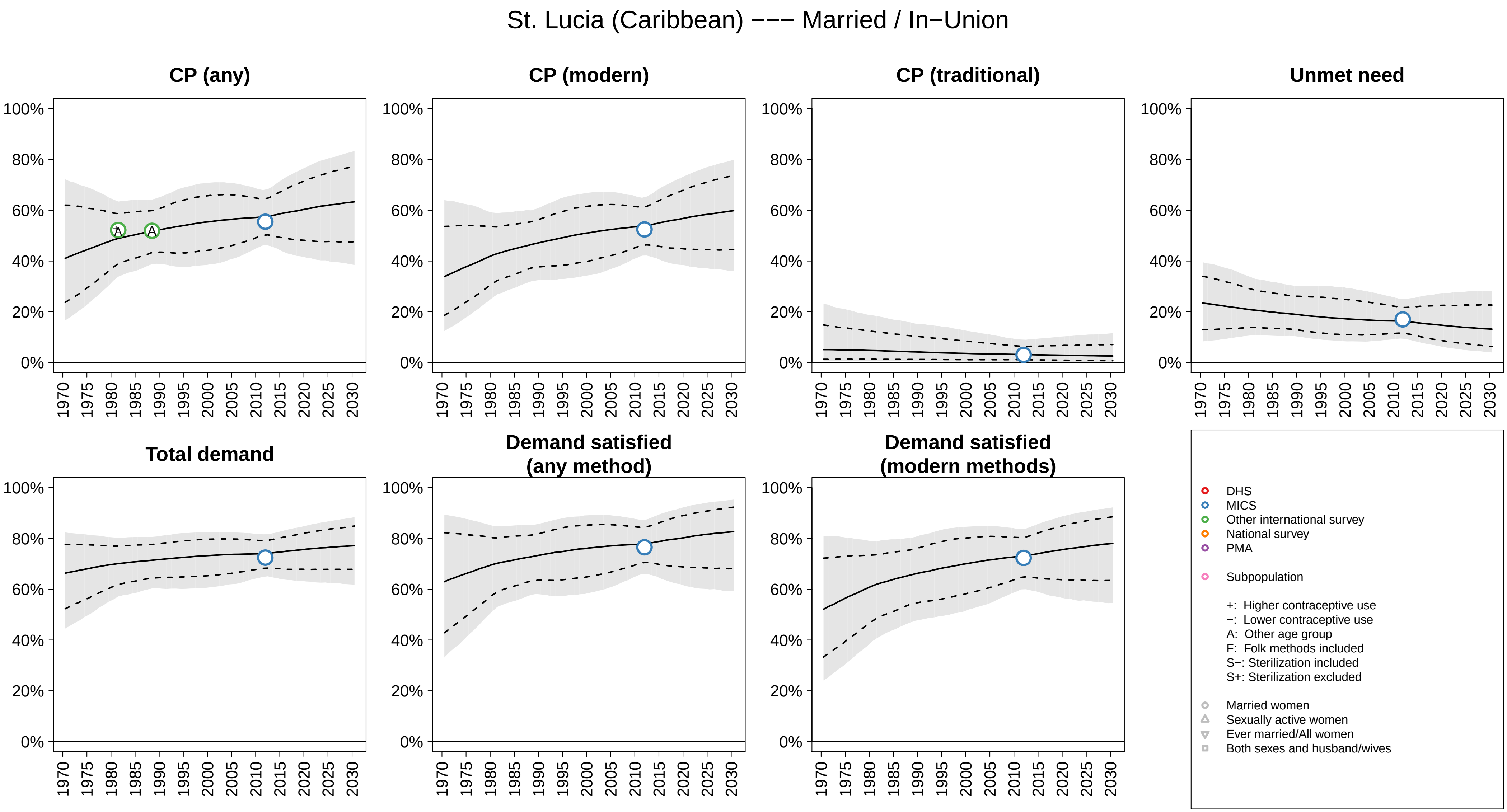
St. Lucia (Caribbean) --- Married / In−Union
CP (any)
CP (modern)
CP (traditional)
Unmet need
Total demand
Demand satisfied (any method)
Demand satisfied (modern methods)
DHS
MICS
Other international survey
National survey
PMA
Subpopulation
+: Higher contraceptive use
−: Lower contraceptive use
A: Other age group
F: Folk methods included
S−: Sterilization included
S+: Sterilization excluded
Married women
Sexually active women
Ever married/All women
Both sexes and husband/wives

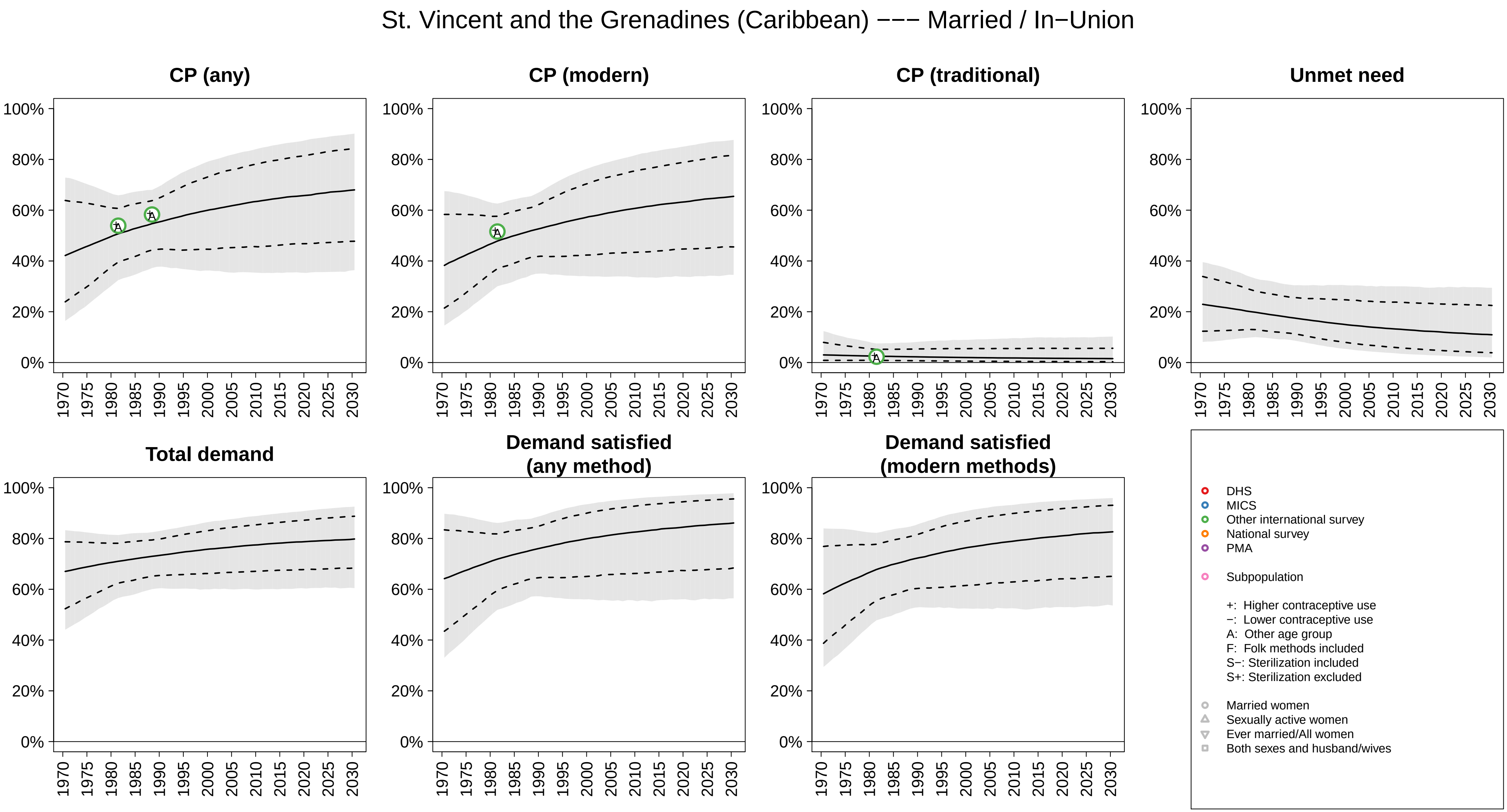
St. Vincent and the Grenadines (Caribbean) −−− Married / In−Union
CP (any)
CP (modern)
CP (traditional)
Unmet need
Total demand
Demand satisfied (any method)
Demand satisfied (modern methods)
100%
80%
60%
40%
20%
0%
1970 1975 1980 1985 1990 1995 2000 2005 2010 2015 2020 2025 2030
DHS
MICS
Other international survey
National survey
PMA
Subpopulation
+: Higher contraceptive use
−: Lower contraceptive use
A: Other age group
F: Folk methods included
S−: Sterilization included
S+: Sterilization excluded
Married women
Sexually active women
Ever married/All women
Both sexes and husband/wives

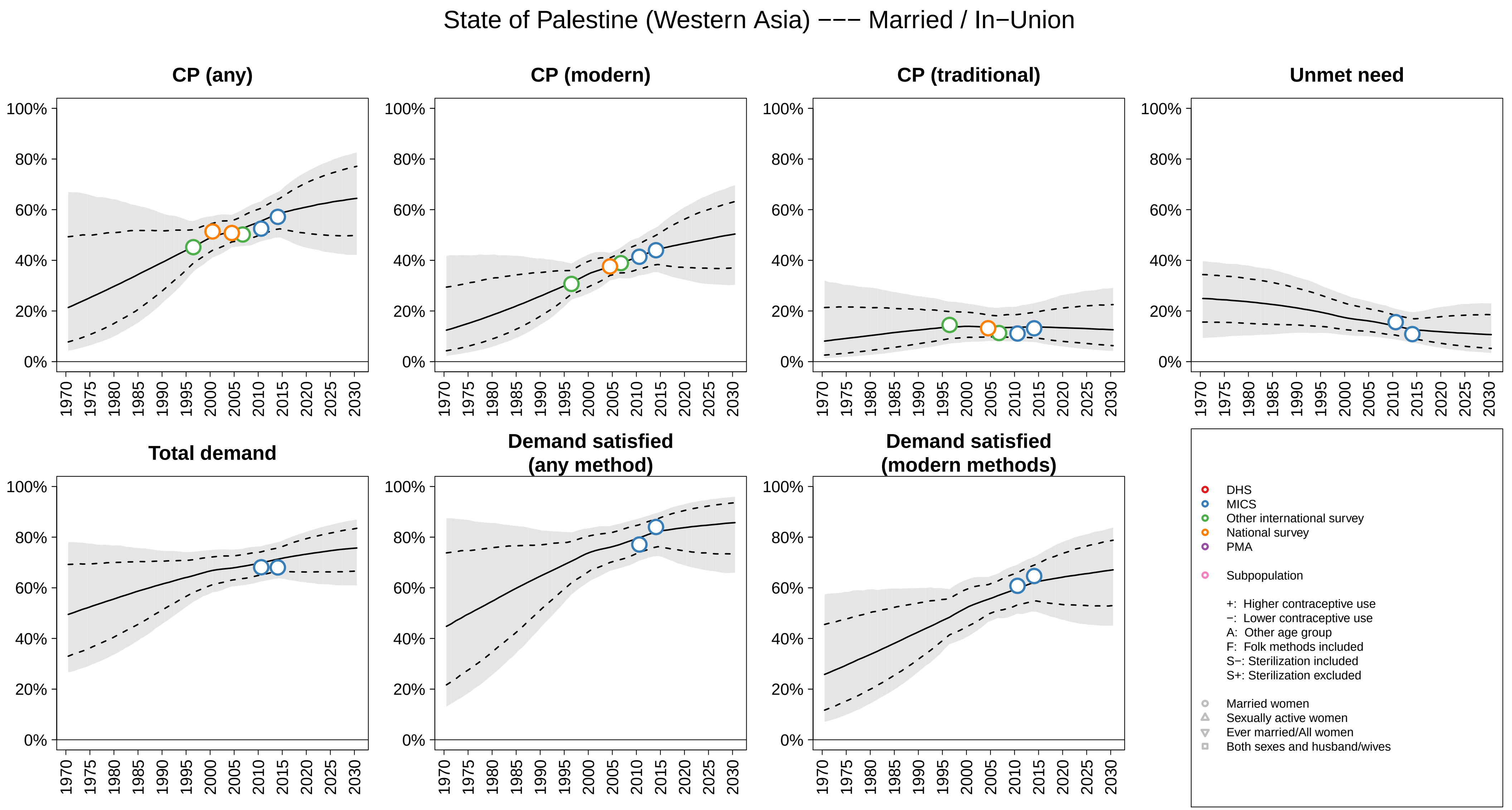

State of Palestine (Western Asia) --- Married / In−Union
CP (any)
CP (modern)
CP (traditional)
Unmet need
Total demand
Demand satisfied (any method)
Demand satisfied (modern methods)
0%
20%
40%
60%
80%
100%
1970
1975
1980
1985
1990
1995
2000
2005
2010
2015
2020
2025
2030
DHS
MICS
Other international survey
National survey
PMA
Subpopulation
+: Higher contraceptive use
−: Lower contraceptive use
A: Other age group
F: Folk methods included
S−: Sterilization included
S+: Sterilization excluded
Married women
Sexually active women
Ever married/All women
Both sexes and husband/wives

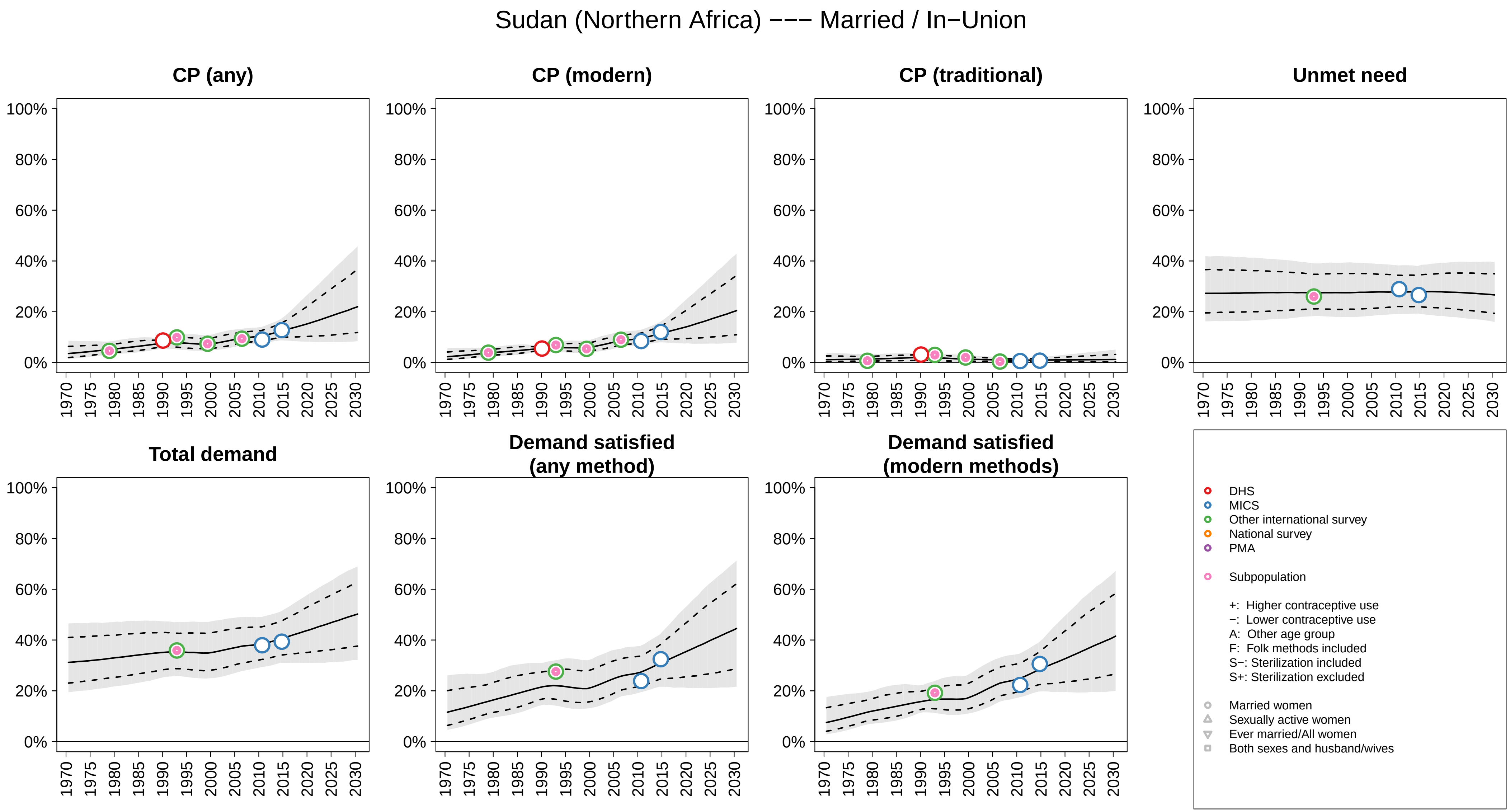
Sudan (Northern Africa) --- Married / In−Union
CP (any)
CP (modern)
CP (traditional)
Unmet need
Total demand
Demand satisfied (any method)
Demand satisfied (modern methods)
100%
80%
60%
40%
20%
0%
1970 1975 1980 1985 1990 1995 2000 2005 2010 2015 2020 2025 2030
DHS
MICS
Other international survey
National survey
PMA
Subpopulation
+: Higher contraceptive use
−: Lower contraceptive use
A: Other age group
F: Folk methods included
S−: Sterilization included
S+: Sterilization excluded
Married women
Sexually active women
Ever married/All women
Both sexes and husband/wives

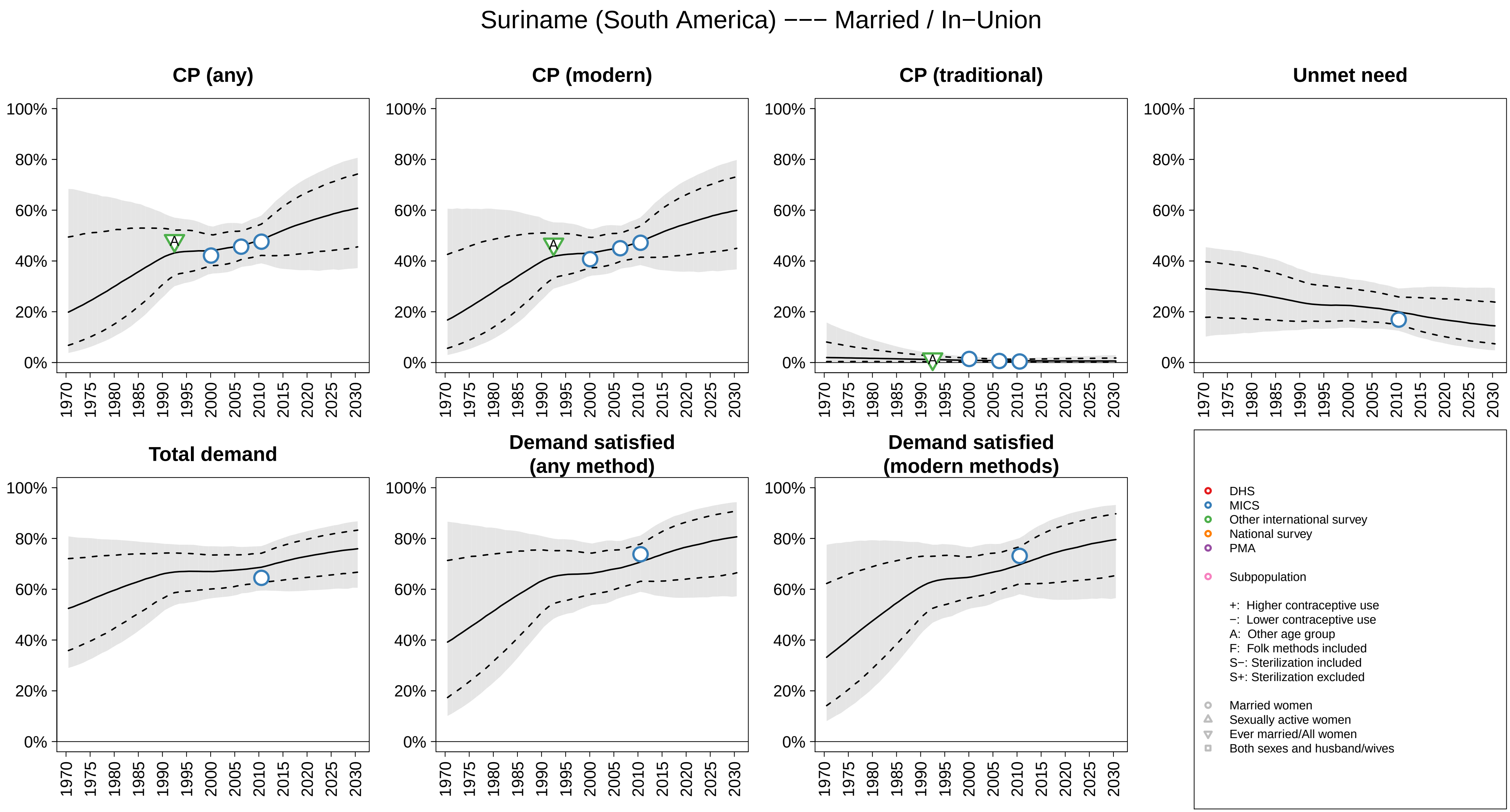

Suriname (South America) ––– Married / In−Union
CP (any)
CP (modern)
CP (traditional)
Unmet need
Total demand
Demand satisfied (any method)
Demand satisfied (modern methods)
100%
80%
60%
40%
20%
0%
1970 1975 1980 1985 1990 1995 2000 2005 2010 2015 2020 2025 2030
A
DHS
MICS
Other international survey
National survey
PMA
Subpopulation
+: Higher contraceptive use
−: Lower contraceptive use
A: Other age group
F: Folk methods included
S−: Sterilization included
S+: Sterilization excluded
Married women
Sexually active women
Ever married/All women
Both sexes and husband/wives

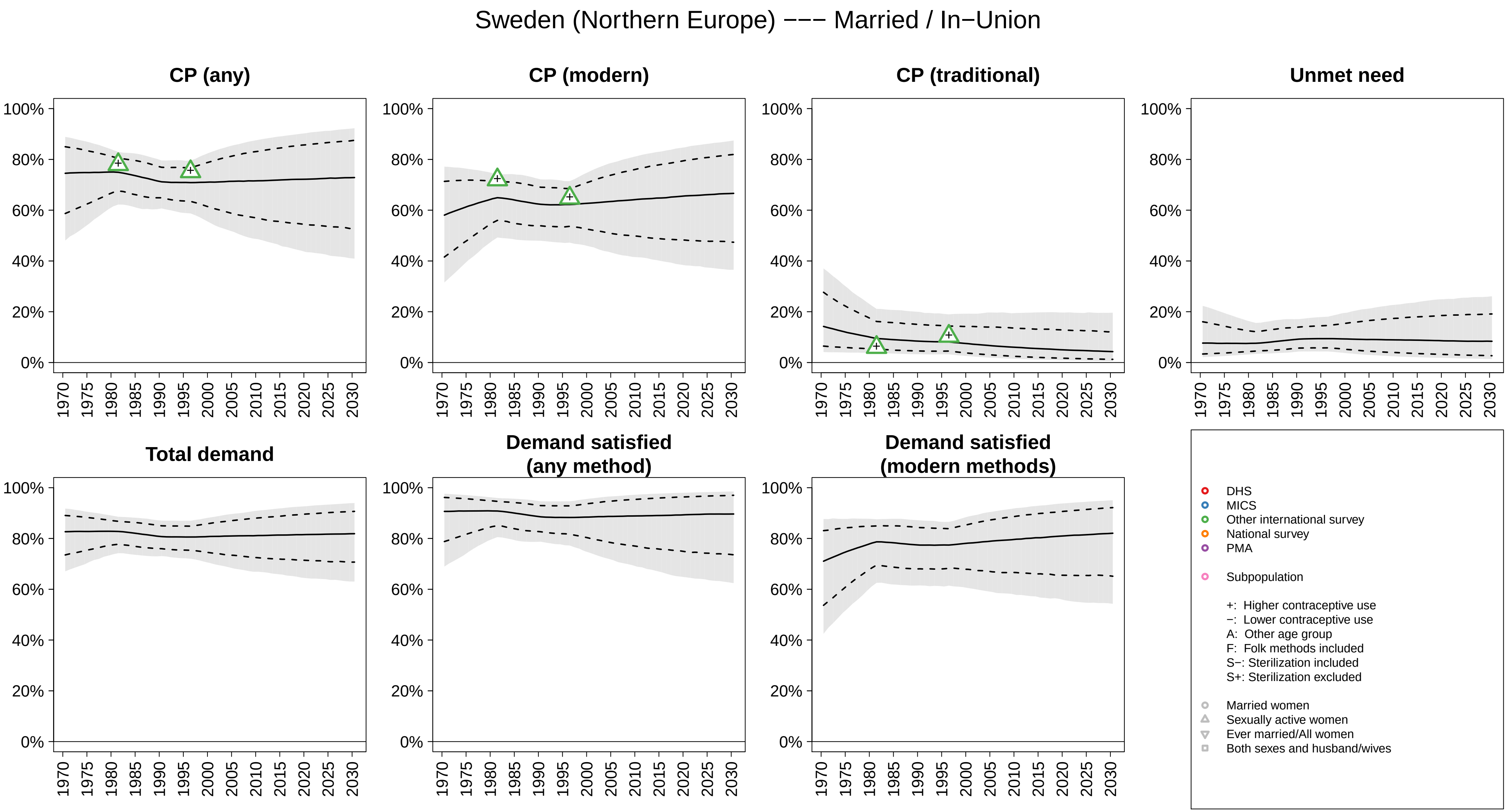
Sweden (Northern Europe) −−− Married / In−Union
CP (any)
CP (modern)
CP (traditional)
Unmet need
Total demand
Demand satisfied (any method)
Demand satisfied (modern methods)
0%
20%
40%
60%
80%
100%
1970
1975
1980
1985
1990
1995
2000
2005
2010
2015
2020
2025
2030
DHS
MICS
Other international survey
National survey
PMA
Subpopulation
+: Higher contraceptive use
−: Lower contraceptive use
A: Other age group
F: Folk methods included
S−: Sterilization included
S+: Sterilization excluded
Married women
Sexually active women
Ever married/All women
Both sexes and husband/wives

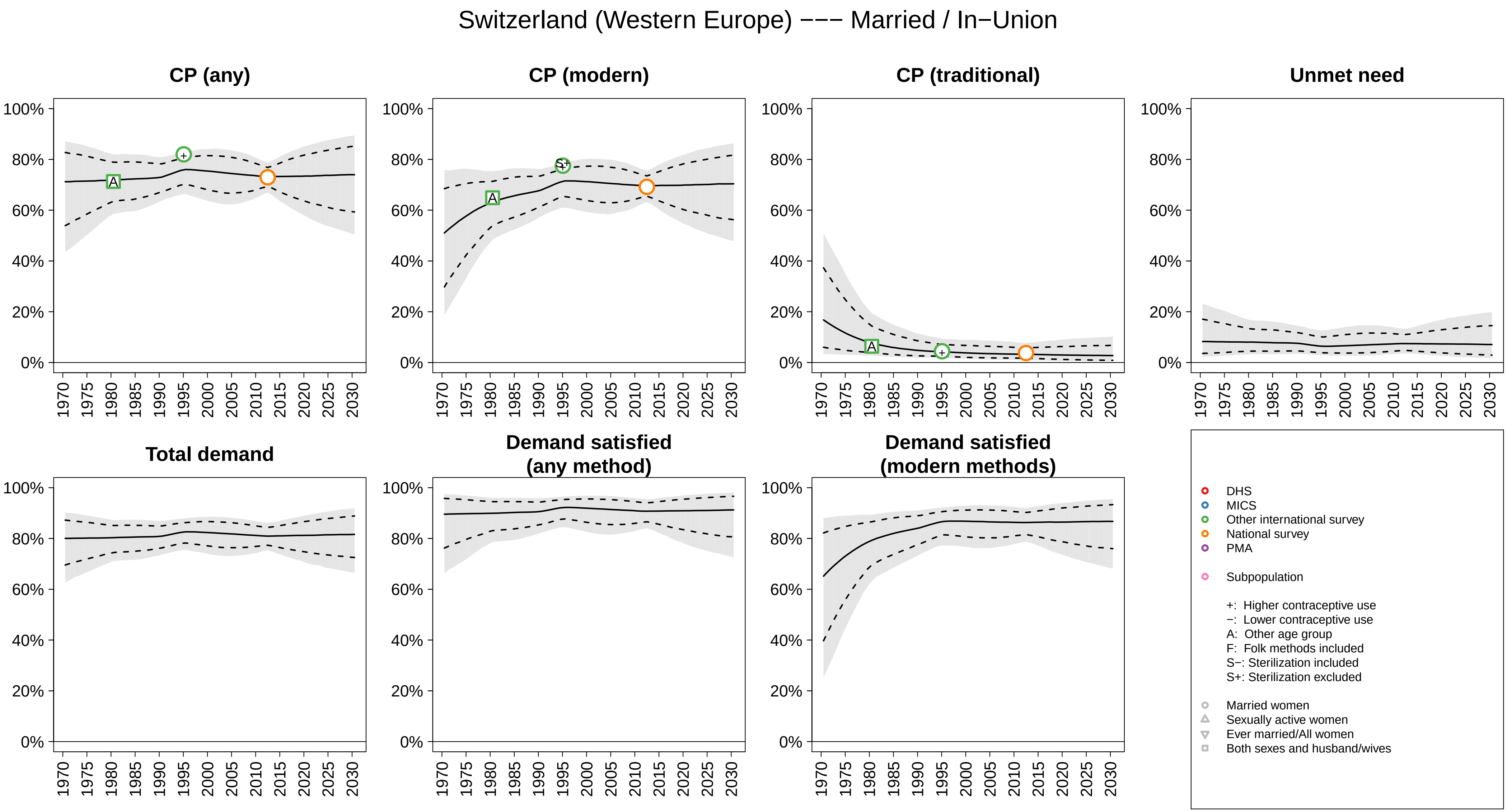
Switzerland (Western Europe) −−− Married / In−Union
CP (any)
CP (modern)
CP (traditional)
Unmet need
Total demand
Demand satisfied (any method)
Demand satisfied (modern methods)
0%
20%
40%
60%
80%
100%
1970
1975
1980
1985
1990
1995
2000
2005
2010
2015
2020
2025
2030
A
S+
+
DHS
MICS
Other international survey
National survey
PMA
Subpopulation
+: Higher contraceptive use
−: Lower contraceptive use
A: Other age group
F: Folk methods included
S−: Sterilization included
S+: Sterilization excluded
Married women
Sexually active women
Ever married/All women
Both sexes and husband/wives

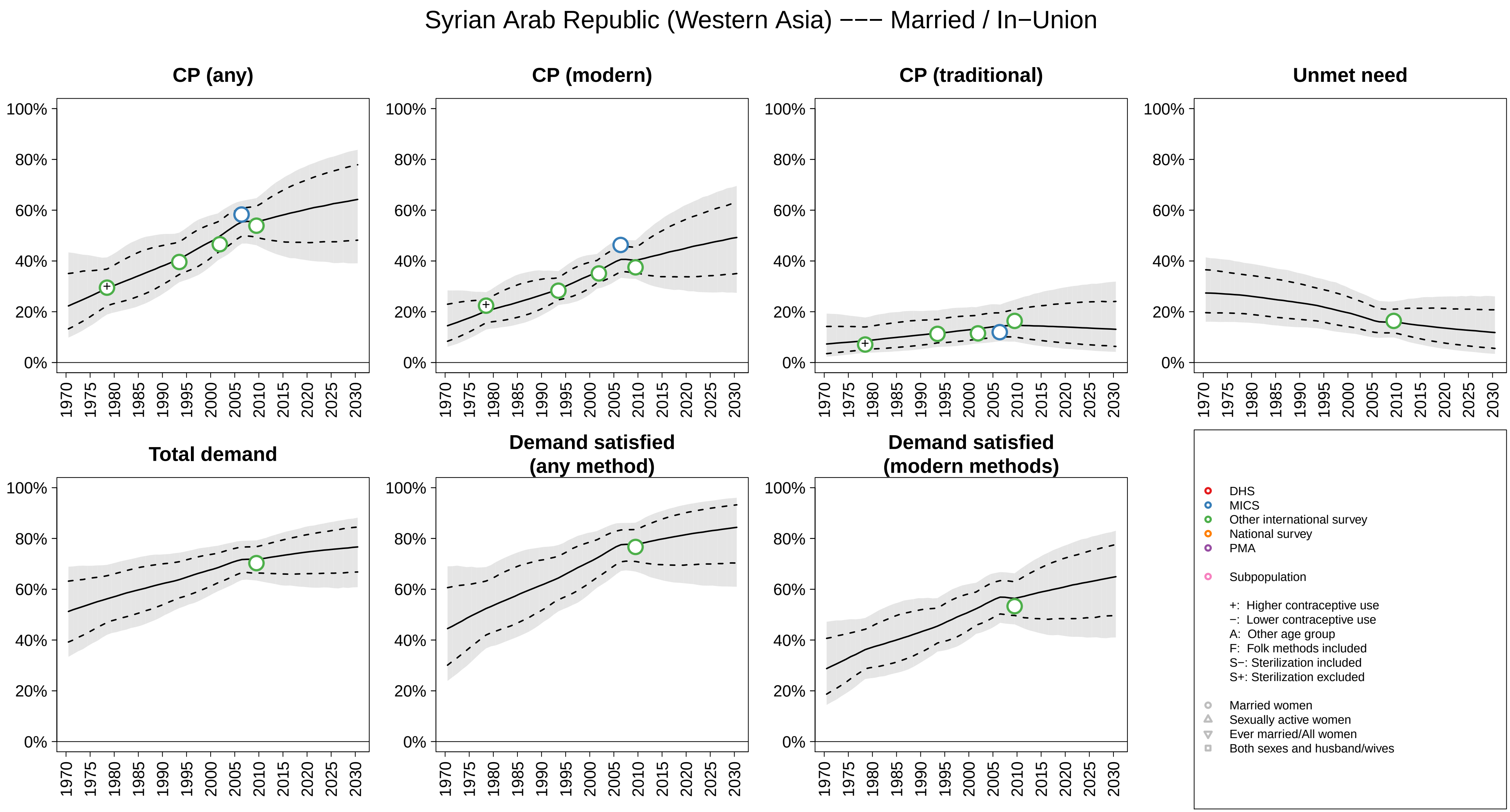
Syrian Arab Republic (Western Asia) --- Married / In−Union
CP (any)
CP (modern)
CP (traditional)
Unmet need
Total demand
Demand satisfied (any method)
Demand satisfied (modern methods)
0%
20%
40%
60%
80%
100%
1970
1975
1980
1985
1990
1995
2000
2005
2010
2015
2020
2025
2030
DHS
MICS
Other international survey
National survey
PMA
Subpopulation
+: Higher contraceptive use
−: Lower contraceptive use
A: Other age group
F: Folk methods included
S−: Sterilization included
S+: Sterilization excluded
Married women
Sexually active women
Ever married/All women
Both sexes and husband/wives

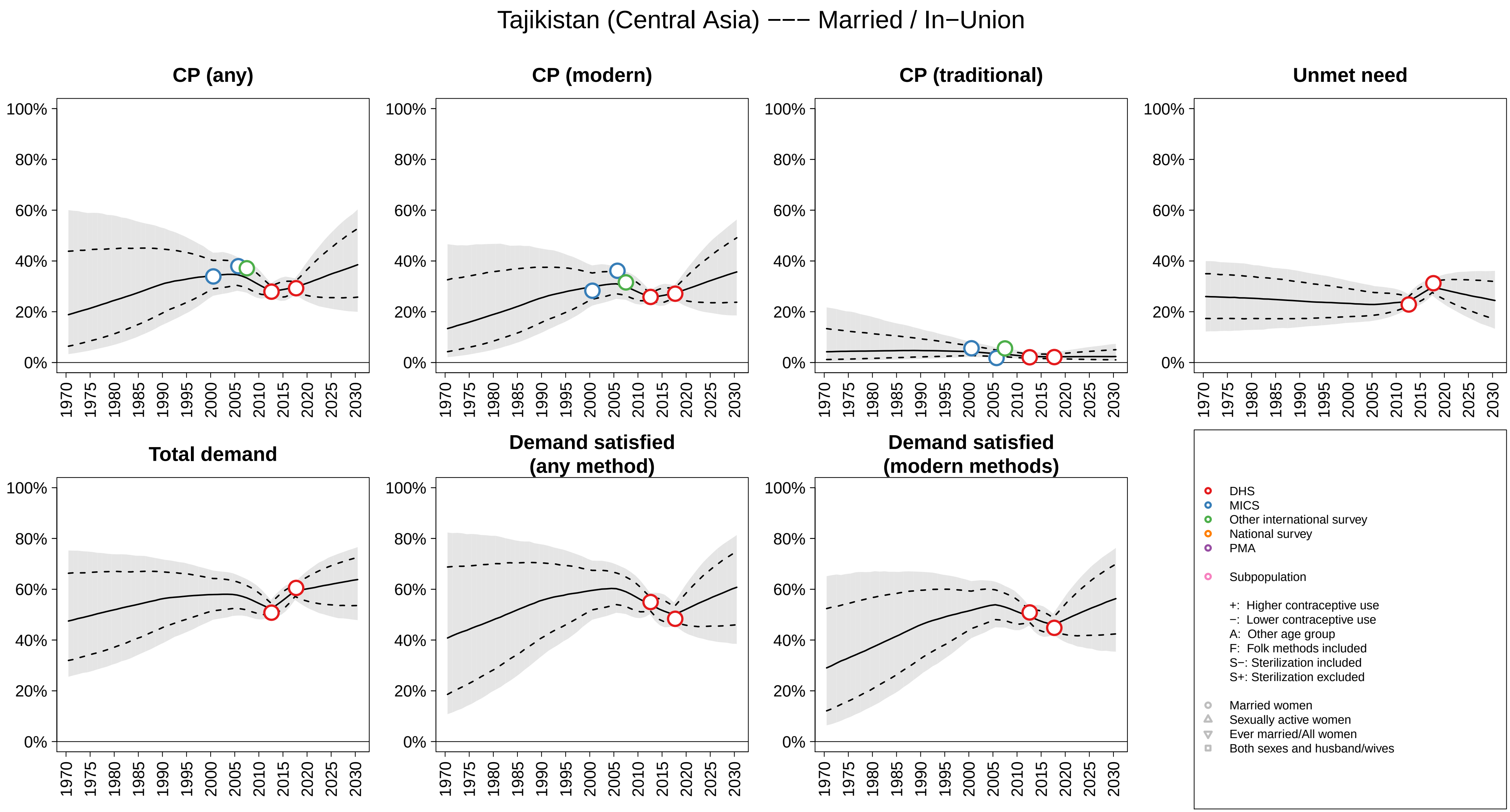
Tajikistan (Central Asia) --- Married / In−Union
CP (any)
CP (modern)
CP (traditional)
Unmet need
Total demand
Demand satisfied (any method)
Demand satisfied (modern methods)
0%
20%
40%
60%
80%
100%
1970
1975
1980
1985
1990
1995
2000
2005
2010
2015
2020
2025
2030
DHS
MICS
Other international survey
National survey
PMA
Subpopulation
+: Higher contraceptive use
−: Lower contraceptive use
A: Other age group
F: Folk methods included
S−: Sterilization included
S+: Sterilization excluded
Married women
Sexually active women
Ever married/All women
Both sexes and husband/wives

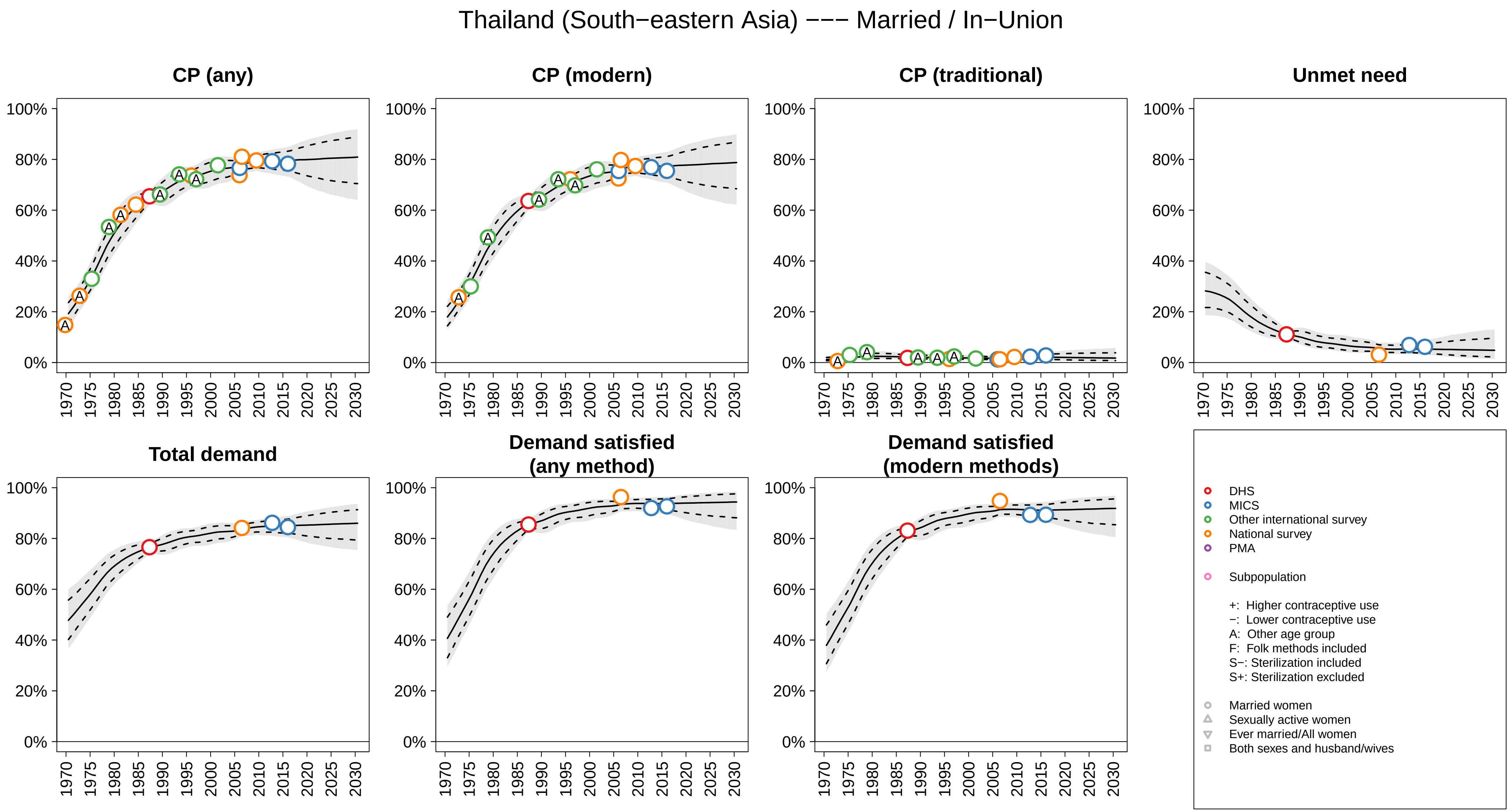
Thailand (South−eastern Asia) −−− Married / In−Union
CP (any)
CP (modern)
CP (traditional)
Unmet need
Total demand
Demand satisfied (any method)
Demand satisfied (modern methods)
DHS
MICS
Other international survey
National survey
PMA
Subpopulation
+: Higher contraceptive use
−: Lower contraceptive use
A: Other age group
F: Folk methods included
S−: Sterilization included
S+: Sterilization excluded
Married women
Sexually active women
Ever married/All women
Both sexes and husband/wives

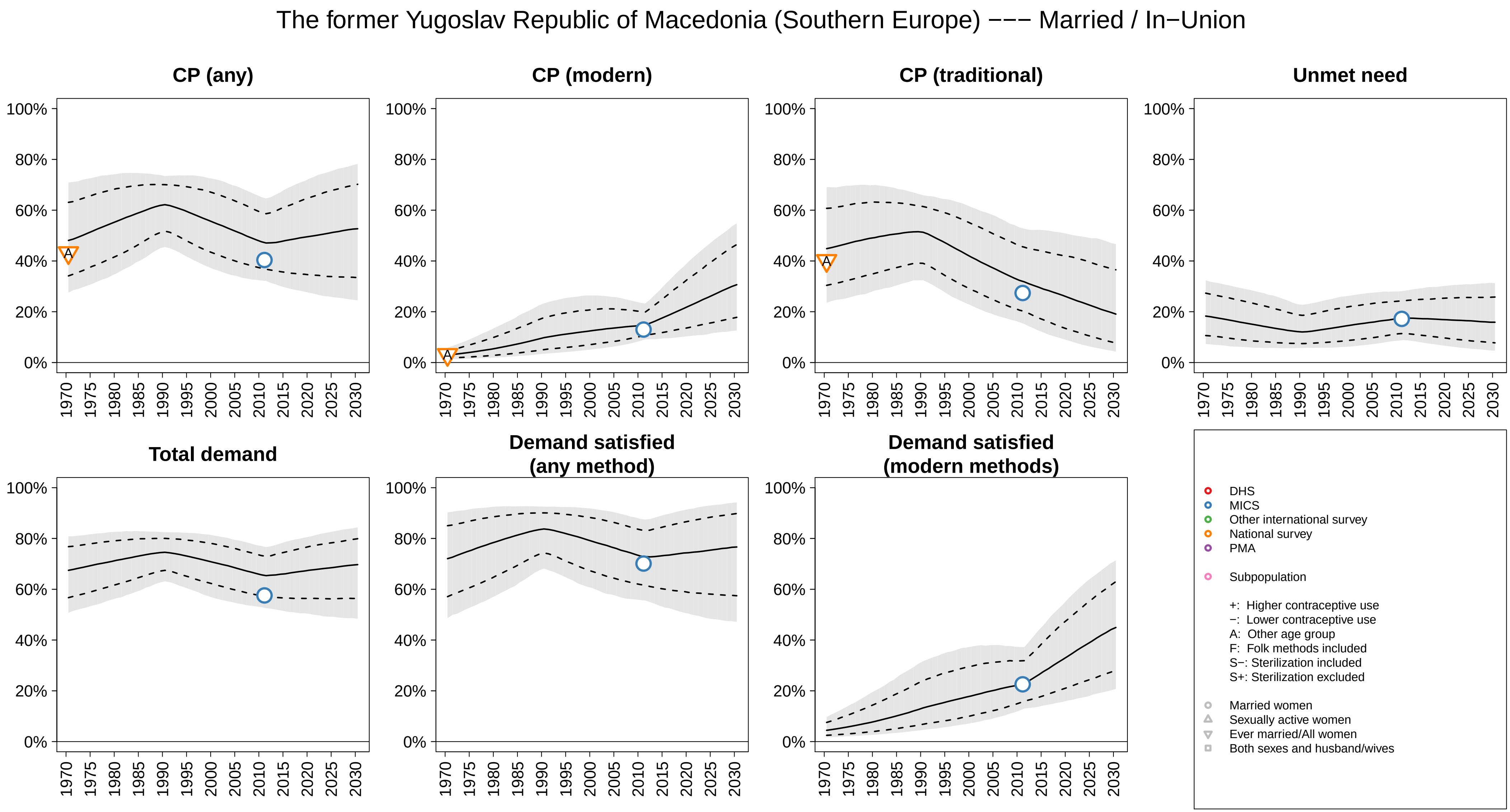
The former Yugoslav Republic of Macedonia (Southern Europe) −−− Married / In−Union
CP (any)
CP (modern)
CP (traditional)
Unmet need
Total demand
Demand satisfied (any method)
Demand satisfied (modern methods)
0%
20%
40%
60%
80%
100%
1970
1975
1980
1985
1990
1995
2000
2005
2010
2015
2020
2025
2030
A
DHS
MICS
Other international survey
National survey
PMA
Subpopulation
+: Higher contraceptive use
−: Lower contraceptive use
A: Other age group
F: Folk methods included
S−: Sterilization included
S+: Sterilization excluded
Married women
Sexually active women
Ever married/All women
Both sexes and husband/wives

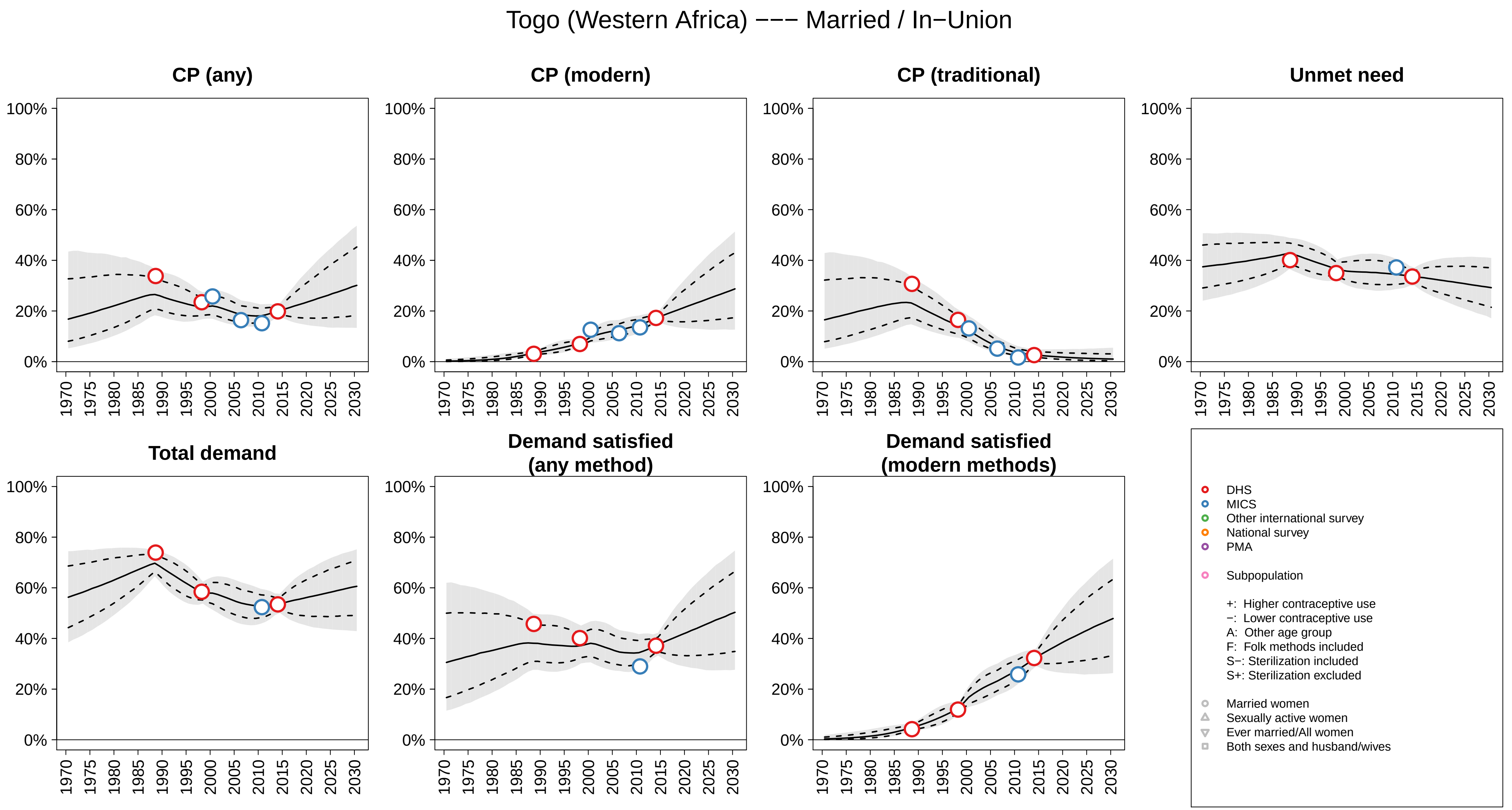
Togo (Western Africa) --- Married / In−Union
CP (any)
CP (modern)
CP (traditional)
Unmet need
Total demand
Demand satisfied (any method)
Demand satisfied (modern methods)
100%
80%
60%
40%
20%
0%
1970
1975
1980
1985
1990
1995
2000
2005
2010
2015
2020
2025
2030
DHS
MICS
Other international survey
National survey
PMA
Subpopulation
+: Higher contraceptive use
−: Lower contraceptive use
A: Other age group
F: Folk methods included
S−: Sterilization included
S+: Sterilization excluded
Married women
Sexually active women
Ever married/All women
Both sexes and husband/wives

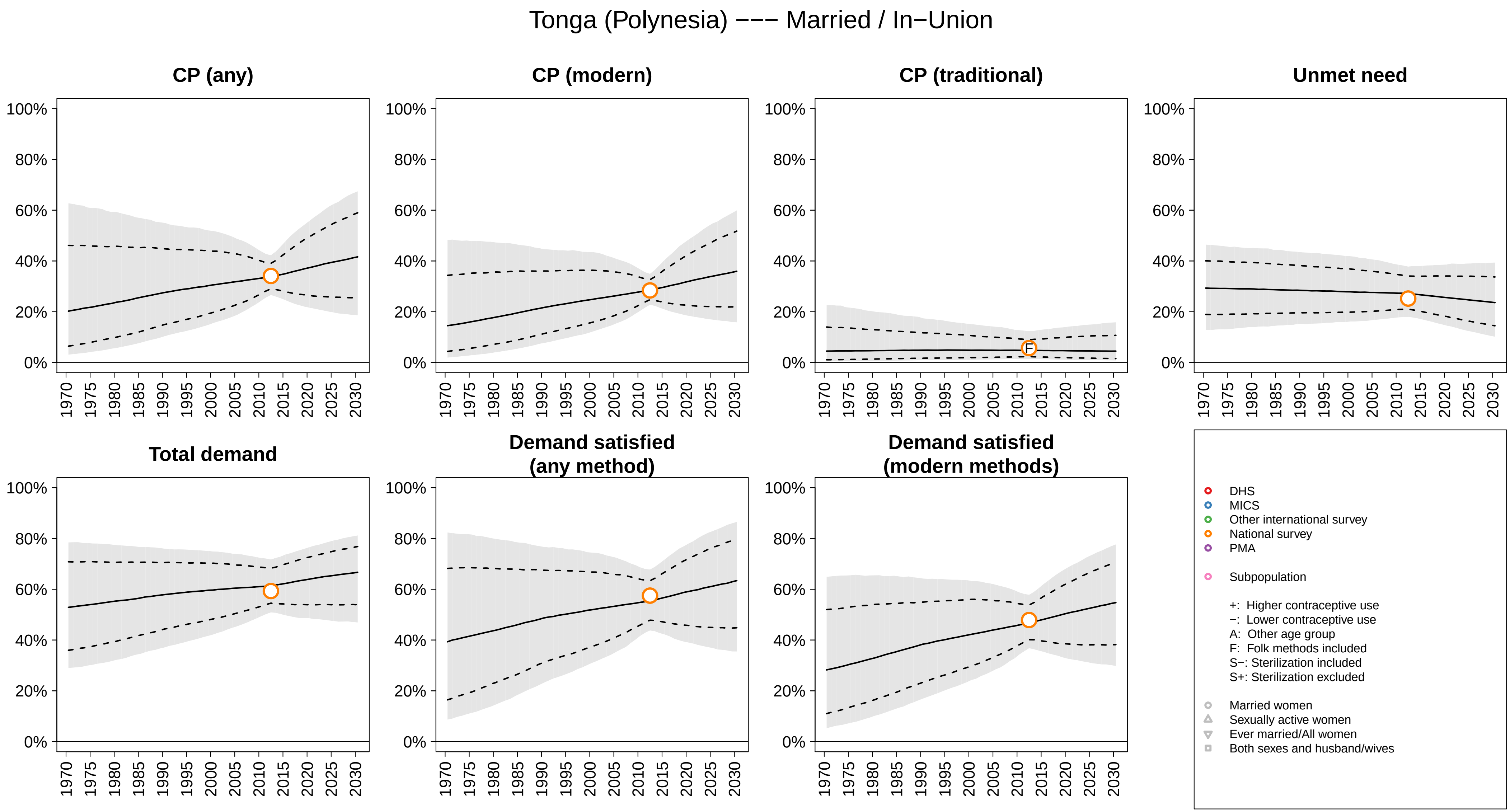
Tonga (Polynesia) −−− Married / In−Union
CP (any)
CP (modern)
CP (traditional)
Unmet need
Total demand
Demand satisfied (any method)
Demand satisfied (modern methods)
100%
80%
60%
40%
20%
0%
1970
1975
1980
1985
1990
1995
2000
2005
2010
2015
2020
2025
2030
F
DHS
MICS
Other international survey
National survey
PMA
Subpopulation
+: Higher contraceptive use
−: Lower contraceptive use
A: Other age group
F: Folk methods included
S−: Sterilization included
S+: Sterilization excluded
Married women
Sexually active women
Ever married/All women
Both sexes and husband/wives

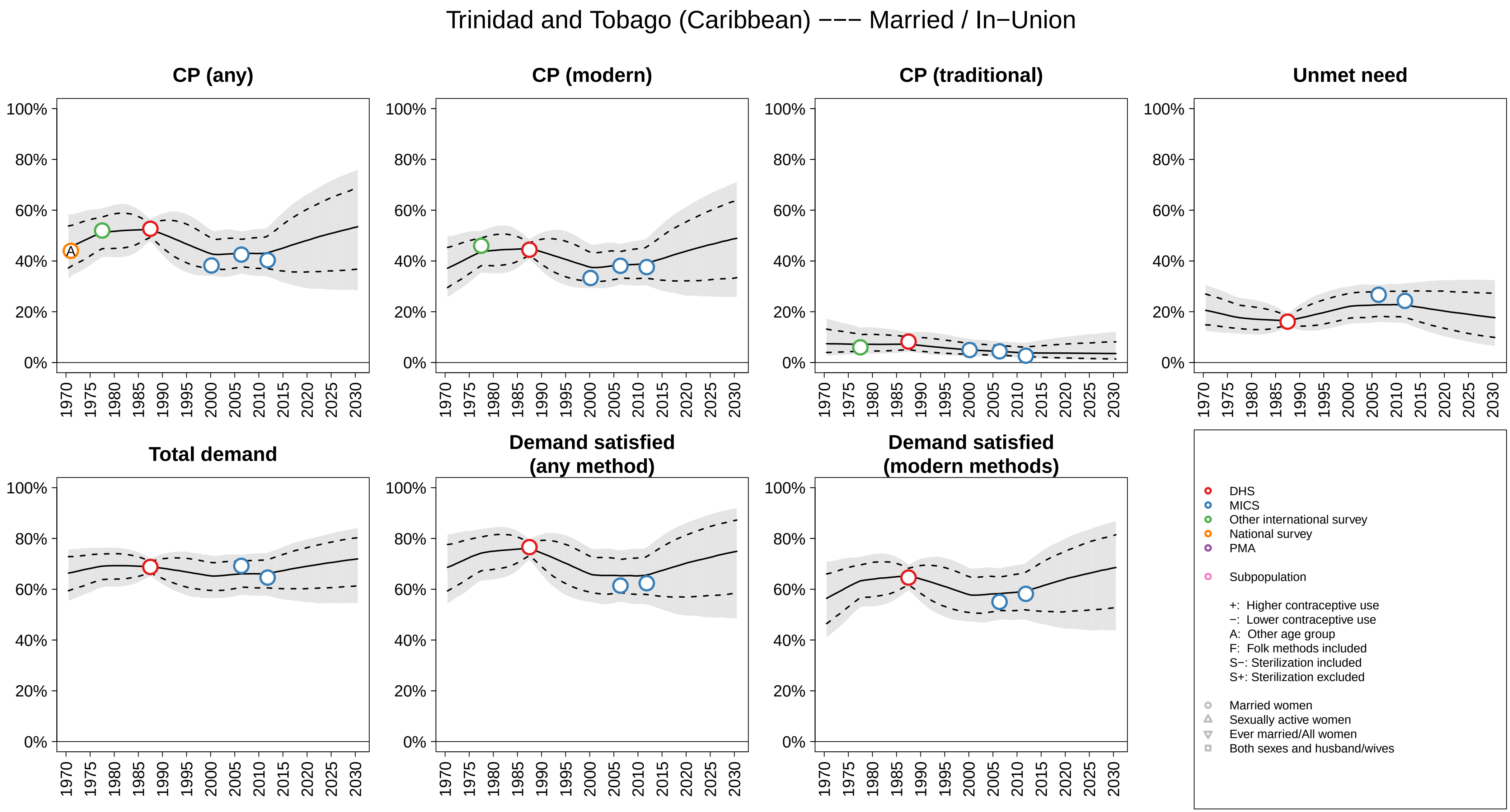
Trinidad and Tobago (Caribbean) --- Married / In−Union
CP (any)
CP (modern)
CP (traditional)
Unmet need
Total demand
Demand satisfied (any method)
Demand satisfied (modern methods)
100%
80%
60%
40%
20%
0%
1970 1975 1980 1985 1990 1995 2000 2005 2010 2015 2020 2025 2030
A
DHS
MICS
Other international survey
National survey
PMA
Subpopulation
+: Higher contraceptive use
−: Lower contraceptive use
A: Other age group
F: Folk methods included
S−: Sterilization included
S+: Sterilization excluded
Married women
Sexually active women
Ever married/All women
Both sexes and husband/wives

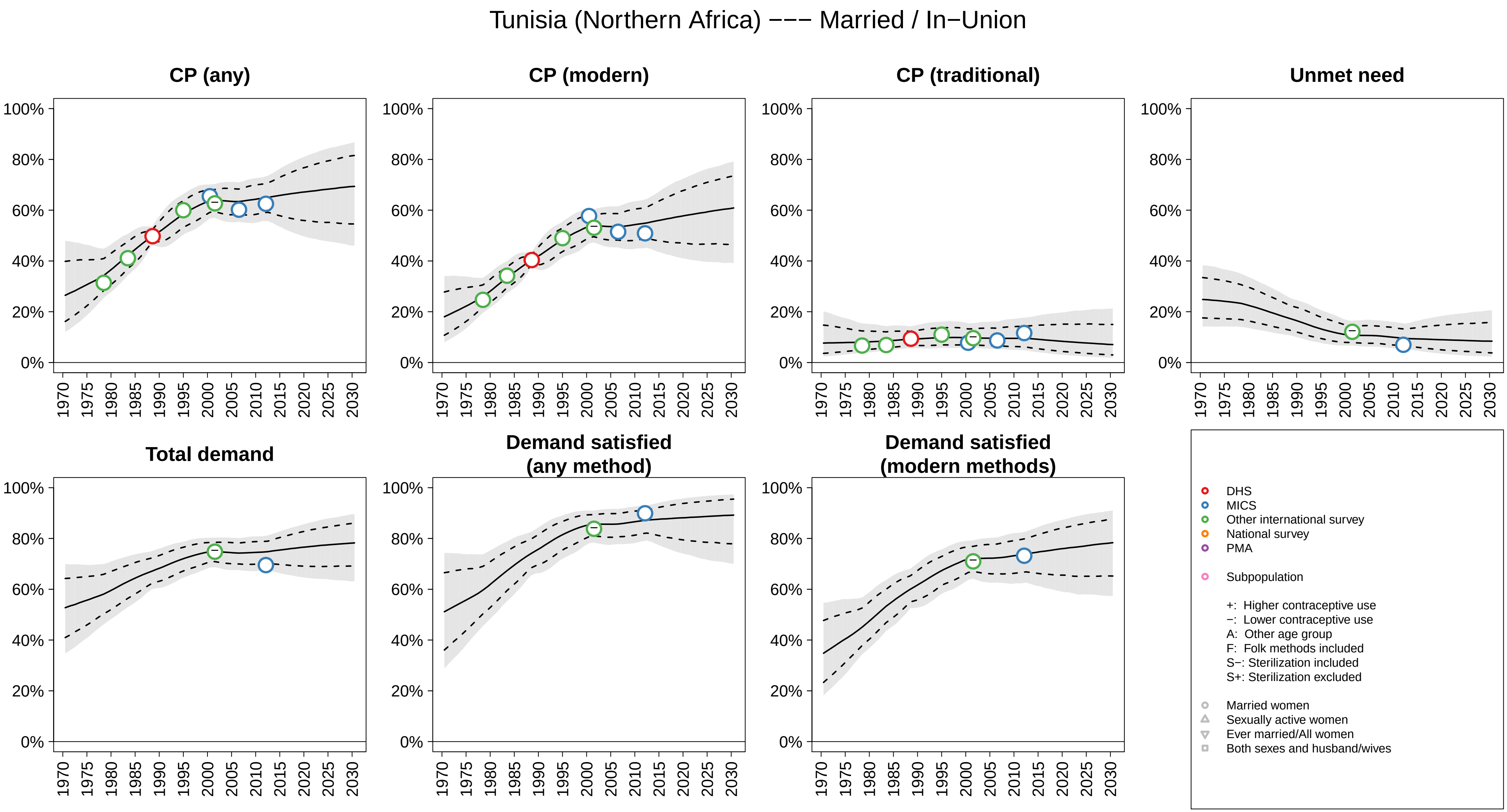
Tunisia (Northern Africa) −−− Married / In−Union
CP (any)
CP (modern)
CP (traditional)
Unmet need
Total demand
Demand satisfied (any method)
Demand satisfied (modern methods)
0%
20%
40%
60%
80%
100%
1970
1975
1980
1985
1990
1995
2000
2005
2010
2015
2020
2025
2030
DHS
MICS
Other international survey
National survey
PMA
Subpopulation
+: Higher contraceptive use
−: Lower contraceptive use
A: Other age group
F: Folk methods included
S−: Sterilization included
S+: Sterilization excluded
Married women
Sexually active women
Ever married/All women
Both sexes and husband/wives

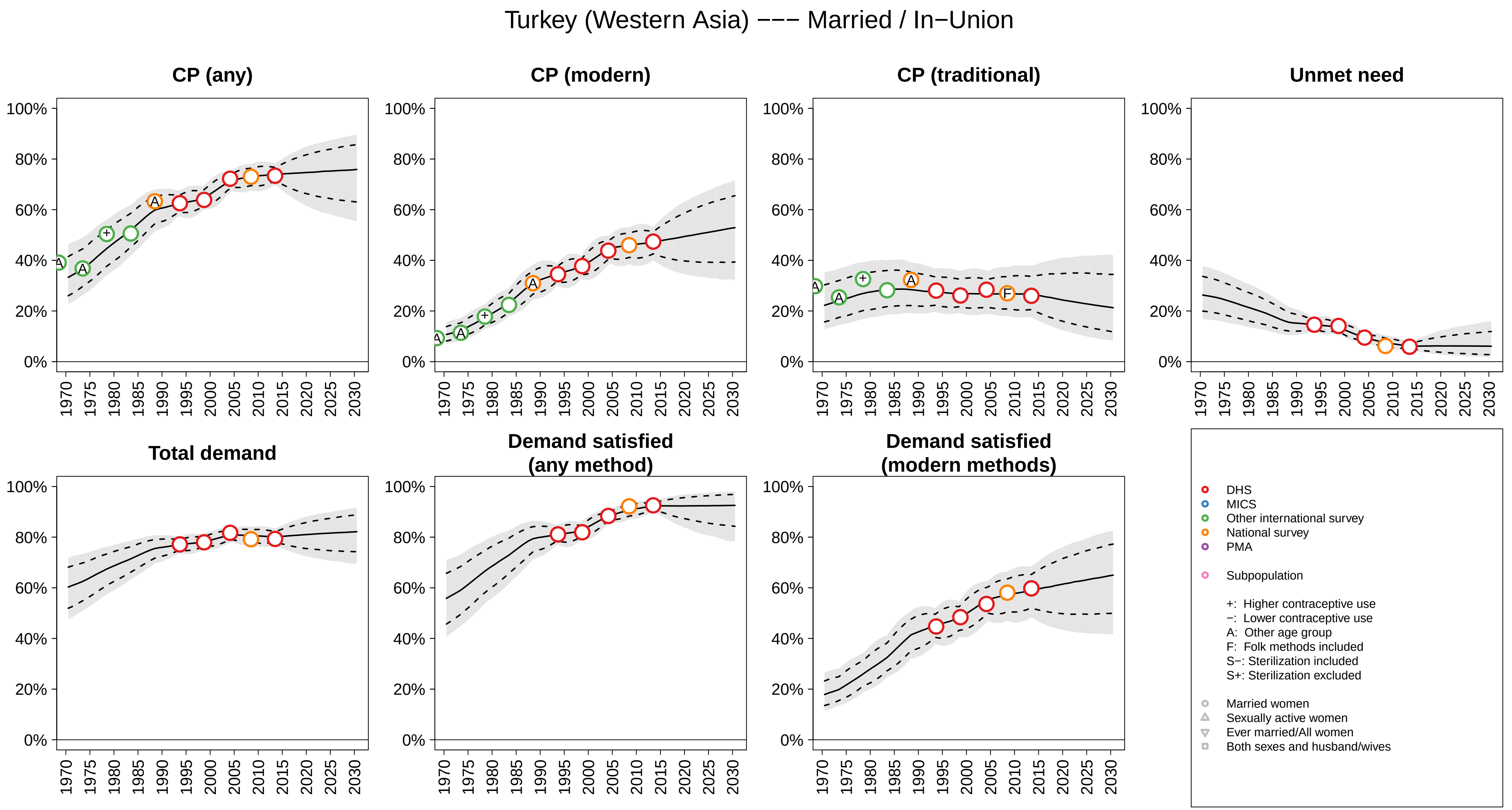
Turkey (Western Asia) −−− Married / In−Union
CP (any)
CP (modern)
CP (traditional)
Unmet need
Total demand
Demand satisfied (any method)
Demand satisfied (modern methods)
0%
20%
40%
60%
80%
100%
1970
1975
1980
1985
1990
1995
2000
2005
2010
2015
2020
2025
2030
DHS
MICS
Other international survey
National survey
PMA
Subpopulation
+: Higher contraceptive use
−: Lower contraceptive use
A: Other age group
F: Folk methods included
S−: Sterilization included
S+: Sterilization excluded
Married women
Sexually active women
Ever married/All women
Both sexes and husband/wives

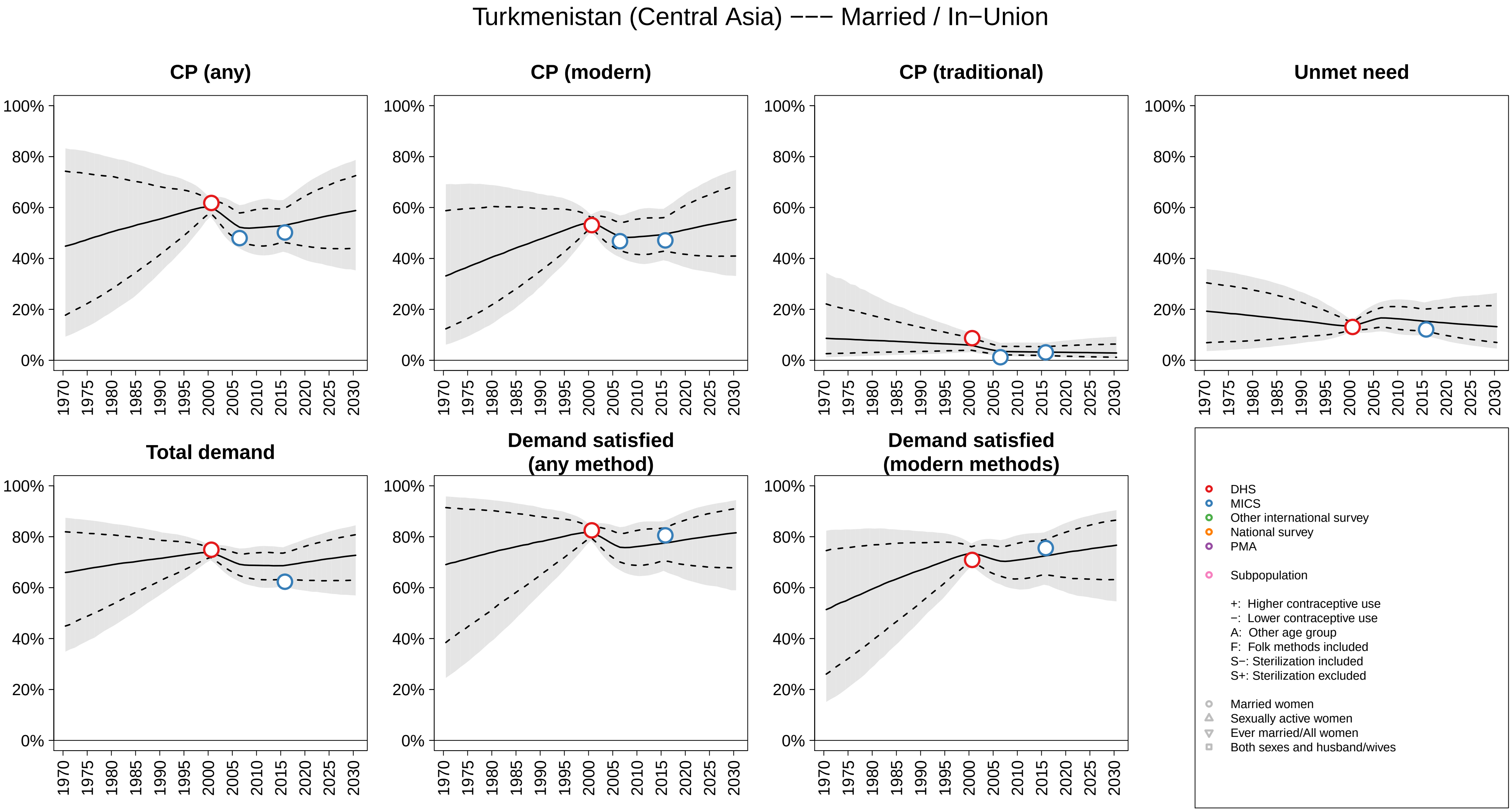
Turkmenistan (Central Asia) −−− Married / In−Union
CP (any)
CP (modern)
CP (traditional)
Unmet need
Total demand
Demand satisfied (any method)
Demand satisfied (modern methods)
0%
20%
40%
60%
80%
100%
1970
1975
1980
1985
1990
1995
2000
2005
2010
2015
2020
2025
2030
DHS
MICS
Other international survey
National survey
PMA
Subpopulation
+: Higher contraceptive use
−: Lower contraceptive use
A: Other age group
F: Folk methods included
S−: Sterilization included
S+: Sterilization excluded
Married women
Sexually active women
Ever married/All women
Both sexes and husband/wives

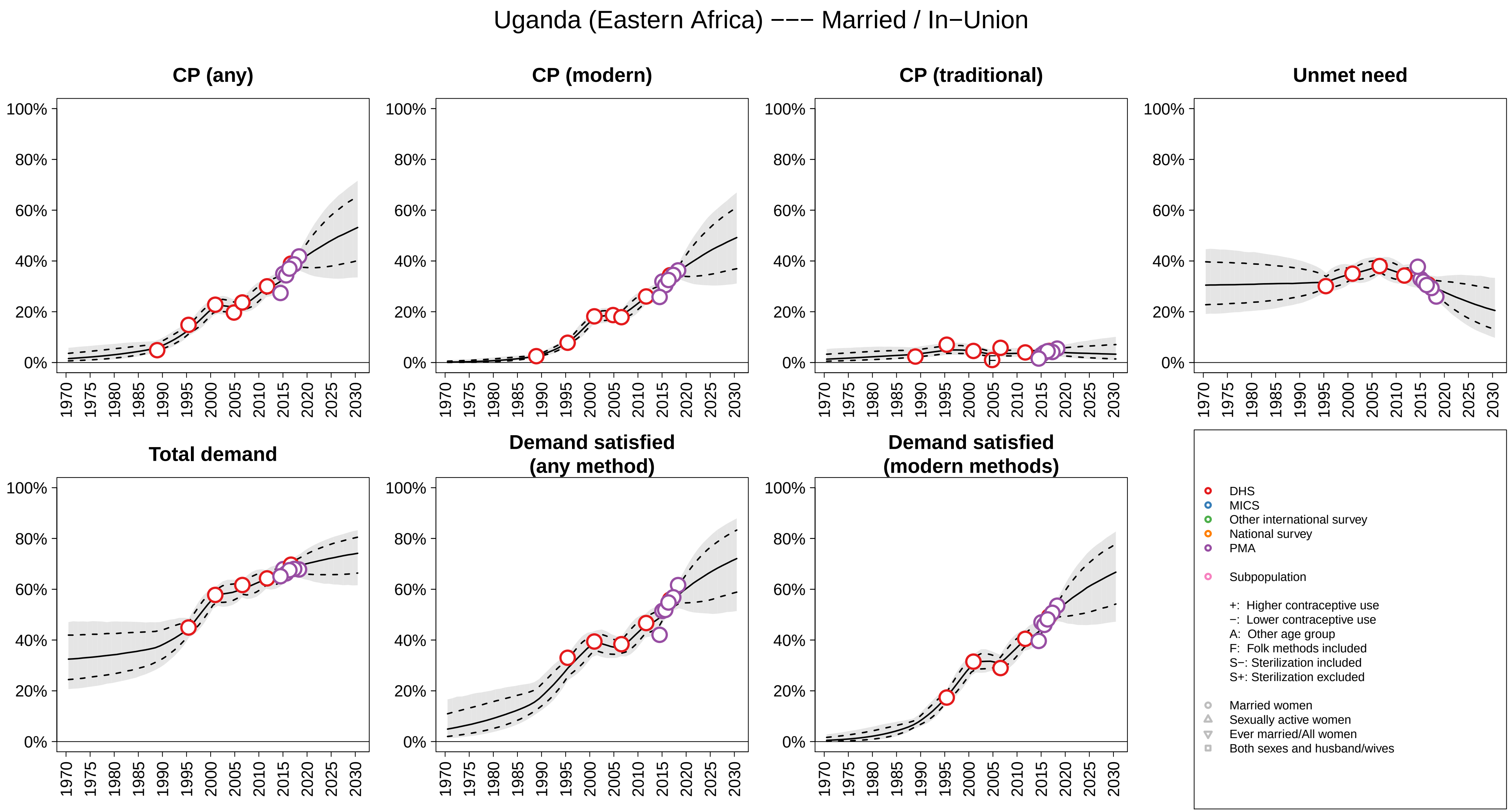
Uganda (Eastern Africa) --- Married / In−Union
CP (any)
CP (modern)
CP (traditional)
Unmet need
Total demand
Demand satisfied (any method)
Demand satisfied (modern methods)
DHS
MICS
Other international survey
National survey
PMA
Subpopulation
+: Higher contraceptive use
−: Lower contraceptive use
A: Other age group
F: Folk methods included
S−: Sterilization included
S+: Sterilization excluded
Married women
Sexually active women
Ever married/All women
Both sexes and husband/wives

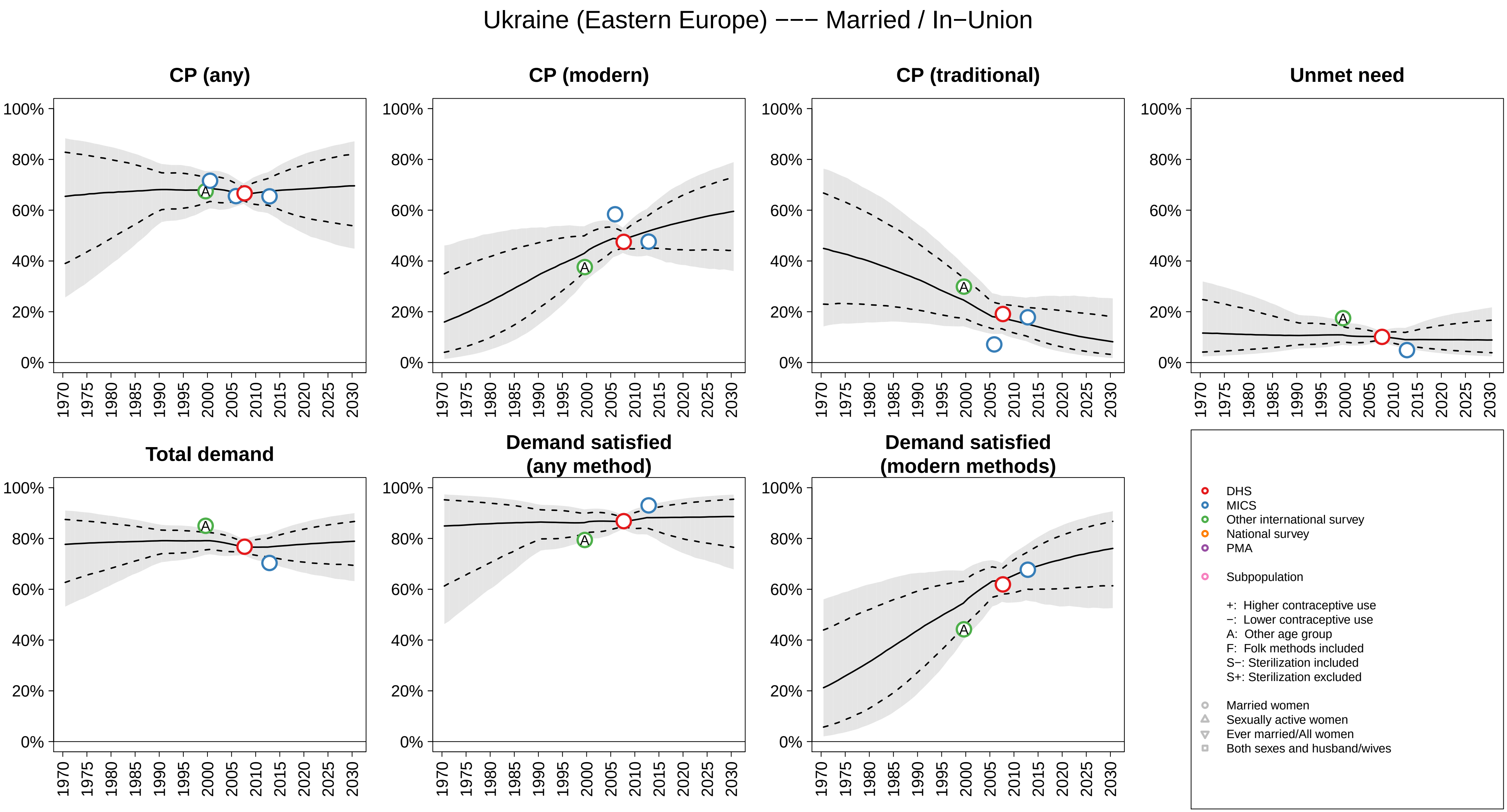
Ukraine (Eastern Europe) −−− Married / In−Union
CP (any)
CP (modern)
CP (traditional)
Unmet need
Total demand
Demand satisfied (any method)
Demand satisfied (modern methods)
DHS
MICS
Other international survey
National survey
PMA
Subpopulation
+: Higher contraceptive use
−: Lower contraceptive use
A: Other age group
F: Folk methods included
S−: Sterilization included
S+: Sterilization excluded
Married women
Sexually active women
Ever married/All women
Both sexes and husband/wives

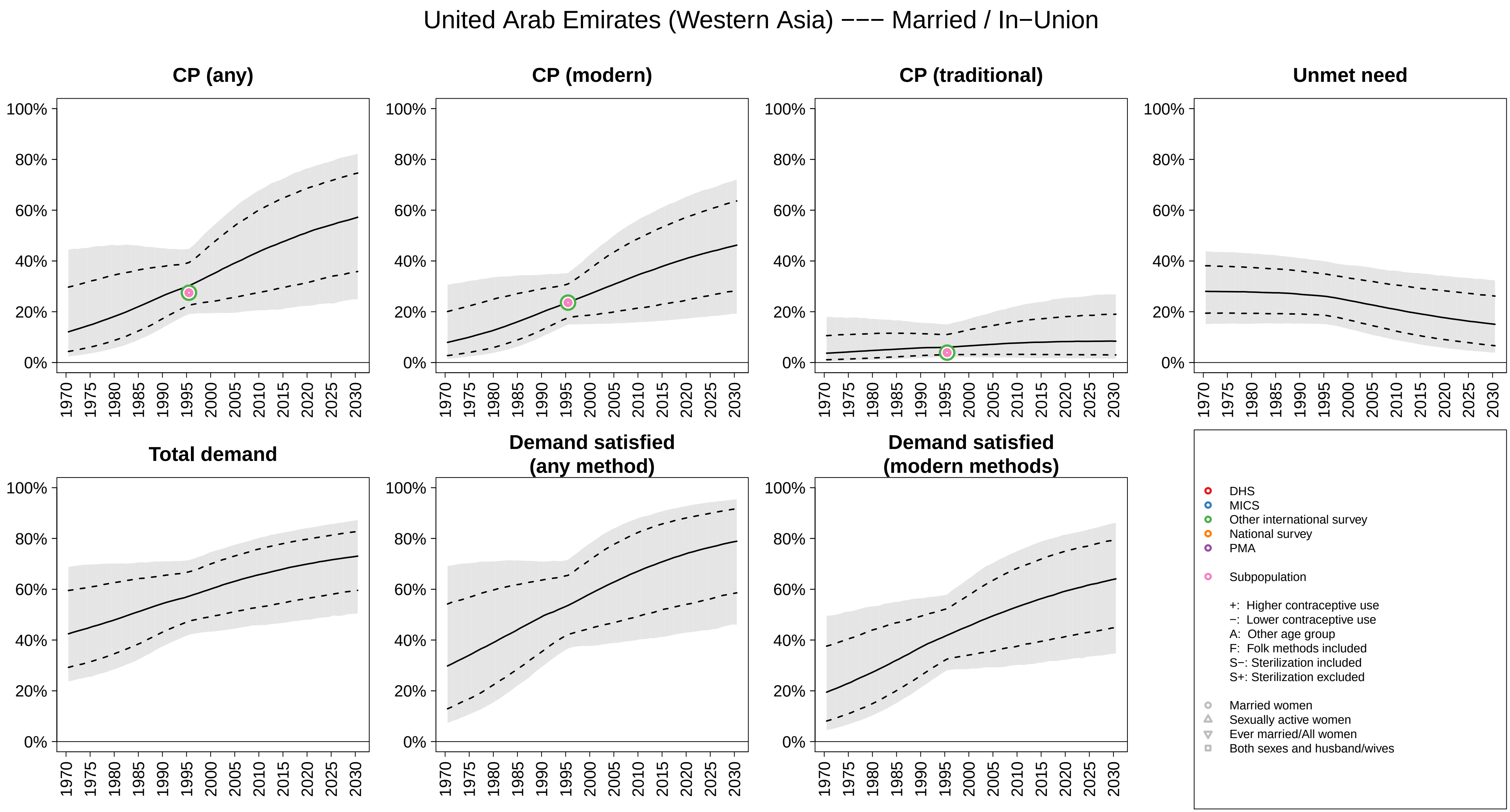
United Arab Emirates (Western Asia) −−− Married / In−Union
CP (any)
CP (modern)
CP (traditional)
Unmet need
Total demand
Demand satisfied (any method)
Demand satisfied (modern methods)
0%
20%
40%
60%
80%
100%
1970
1975
1980
1985
1990
1995
2000
2005
2010
2015
2020
2025
2030
DHS
MICS
Other international survey
National survey
PMA
Subpopulation
+: Higher contraceptive use
−: Lower contraceptive use
A: Other age group
F: Folk methods included
S−: Sterilization included
S+: Sterilization excluded
Married women
Sexually active women
Ever married/All women
Both sexes and husband/wives

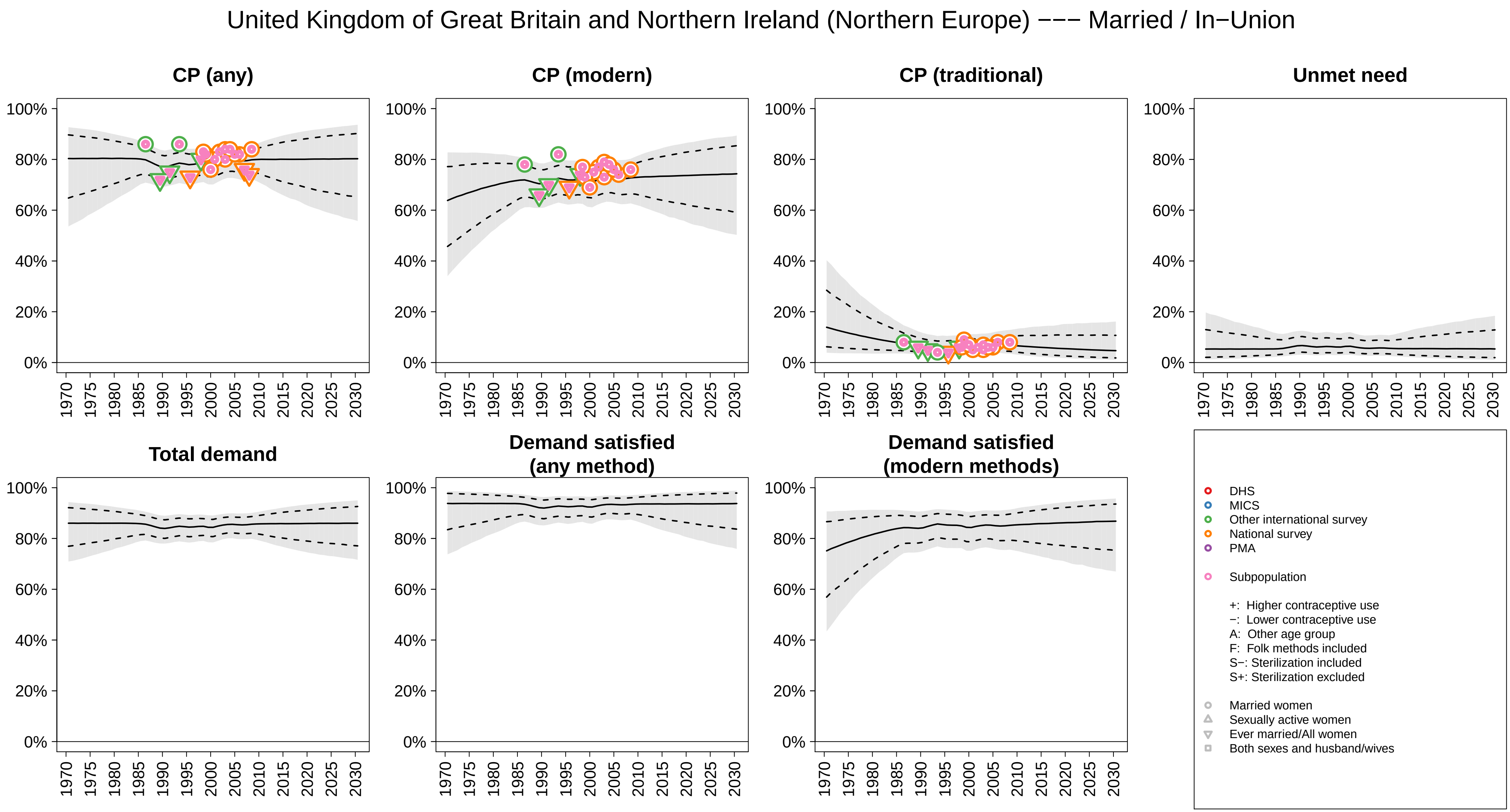
United Kingdom of Great Britain and Northern Ireland (Northern Europe) --- Married / In-Union
CP (any)
CP (modern)
CP (traditional)
Unmet need
Total demand
Demand satisfied (any method)
Demand satisfied (modern methods)
100%
80%
60%
40%
20%
0%
1970 1975 1980 1985 1990 1995 2000 2005 2010 2015 2020 2025 2030
DHS
MICS
Other international survey
National survey
PMA
Subpopulation
+: Higher contraceptive use
−: Lower contraceptive use
A: Other age group
F: Folk methods included
S−: Sterilization included
S+: Sterilization excluded
Married women
Sexually active women
Ever married/All women
Both sexes and husband/wives

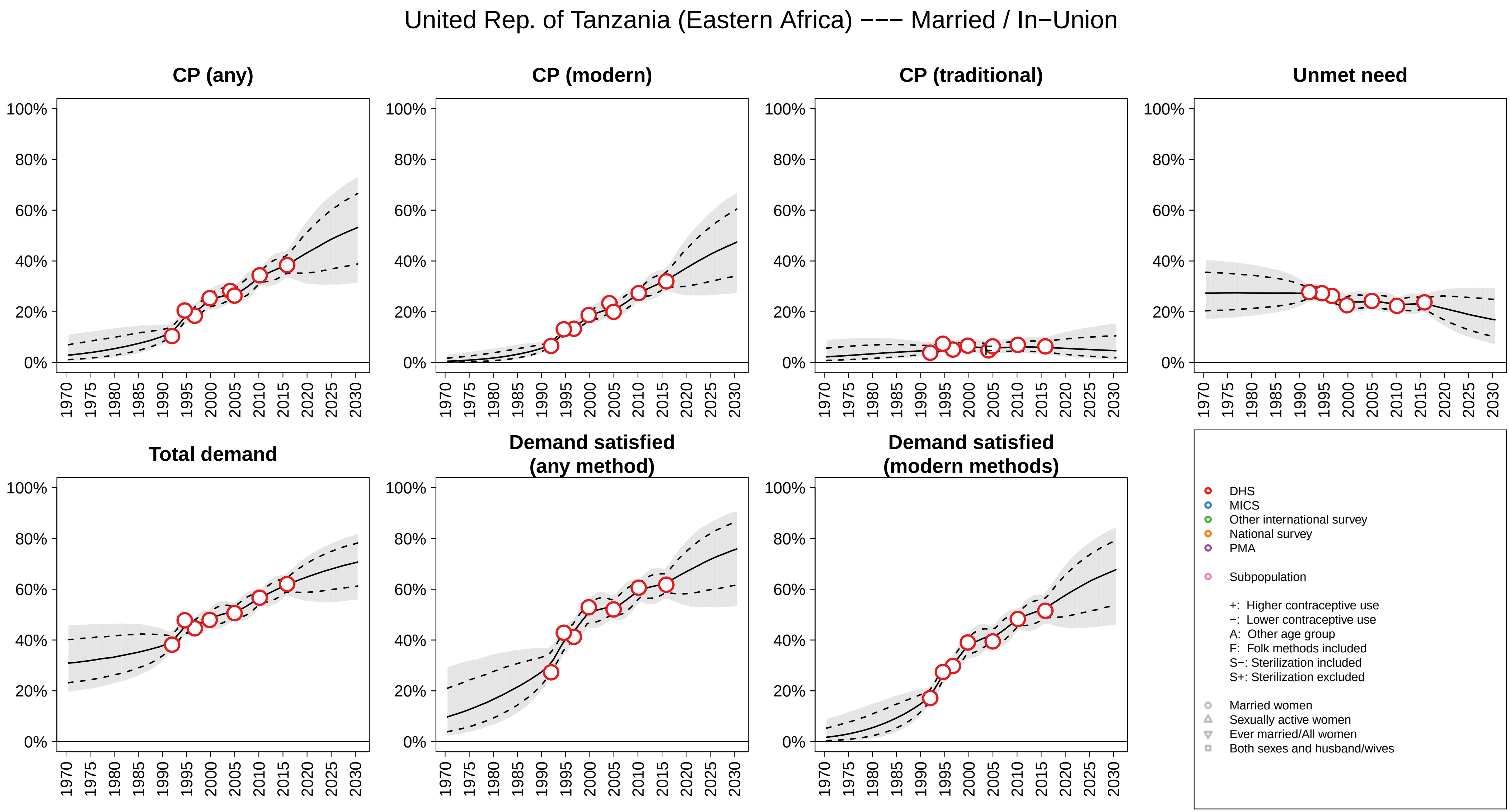
United Rep. of Tanzania (Eastern Africa) --- Married / In−Union
CP (any)
CP (modern)
CP (traditional)
Unmet need
Total demand
Demand satisfied (any method)
Demand satisfied (modern methods)
DHS
MICS
Other international survey
National survey
PMA
Subpopulation
+: Higher contraceptive use
−: Lower contraceptive use
A: Other age group
F: Folk methods included
S−: Sterilization included
S+: Sterilization excluded
Married women
Sexually active women
Ever married/All women
Both sexes and husband/wives

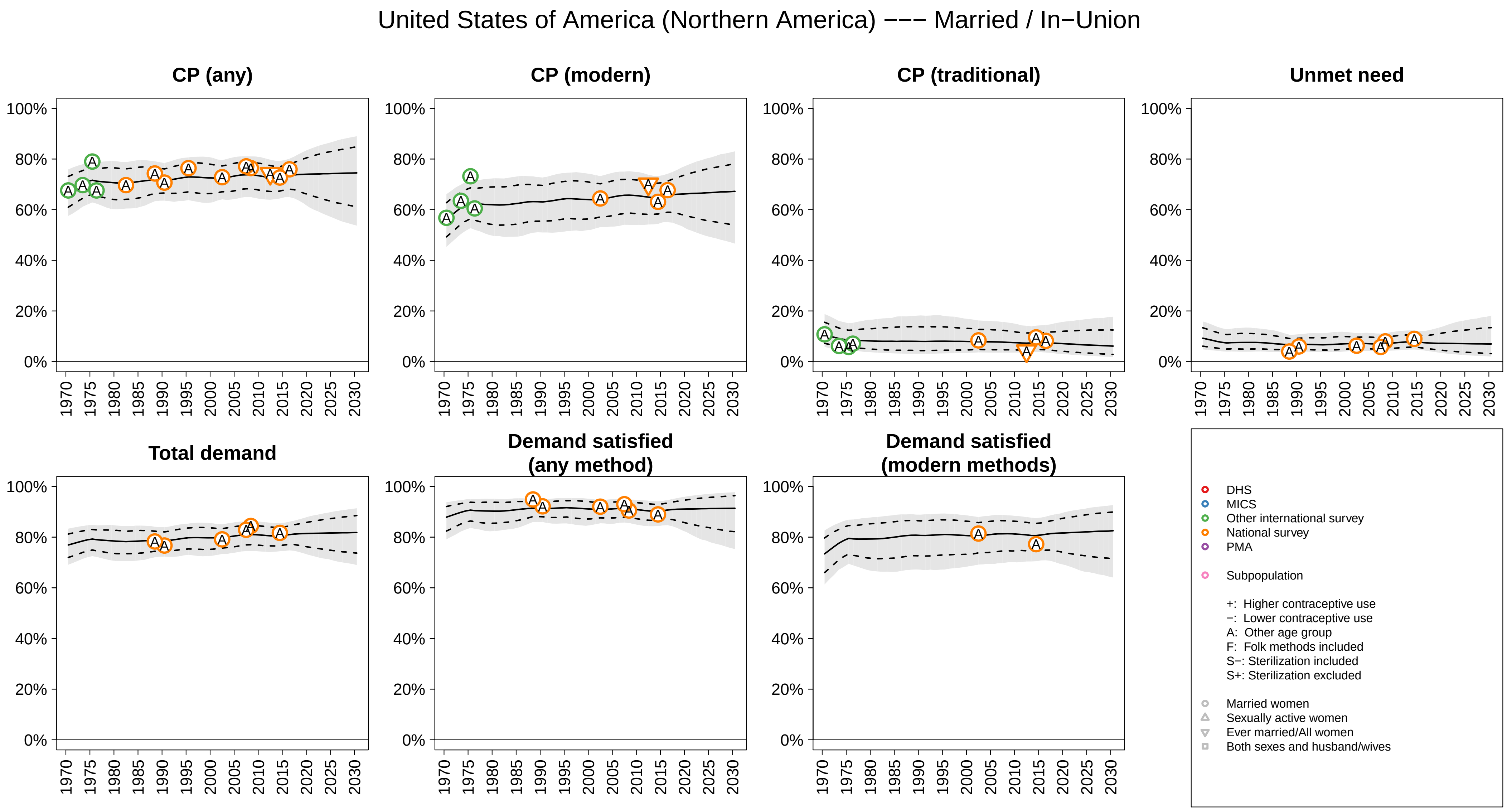
United States of America (Northern America) −−− Married / In−Union
CP (any)
CP (modern)
CP (traditional)
Unmet need
Total demand
Demand satisfied (any method)
Demand satisfied (modern methods)
DHS
MICS
Other international survey
National survey
PMA
Subpopulation
+: Higher contraceptive use
−: Lower contraceptive use
A: Other age group
F: Folk methods included
S−: Sterilization included
S+: Sterilization excluded
Married women
Sexually active women
Ever married/All women
Both sexes and husband/wives

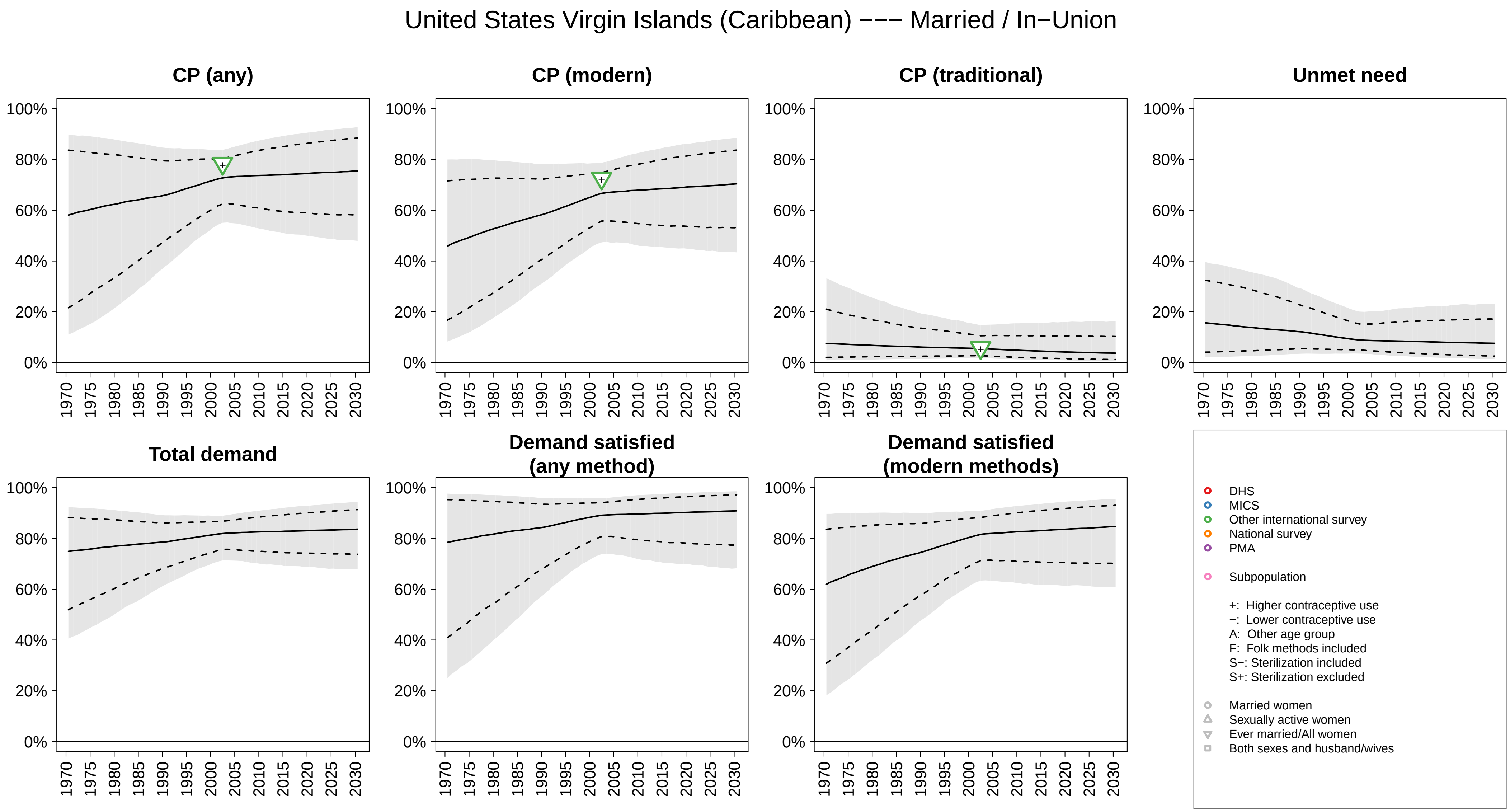
United States Virgin Islands (Caribbean) −−− Married / In−Union
CP (any)
CP (modern)
CP (traditional)
Unmet need
Total demand
Demand satisfied (any method)
Demand satisfied (modern methods)
100%
80%
60%
40%
20%
0%
1970 1975 1980 1985 1990 1995 2000 2005 2010 2015 2020 2025 2030
DHS
MICS
Other international survey
National survey
PMA
Subpopulation
+: Higher contraceptive use
−: Lower contraceptive use
A: Other age group
F: Folk methods included
S−: Sterilization included
S+: Sterilization excluded
Married women
Sexually active women
Ever married/All women
Both sexes and husband/wives

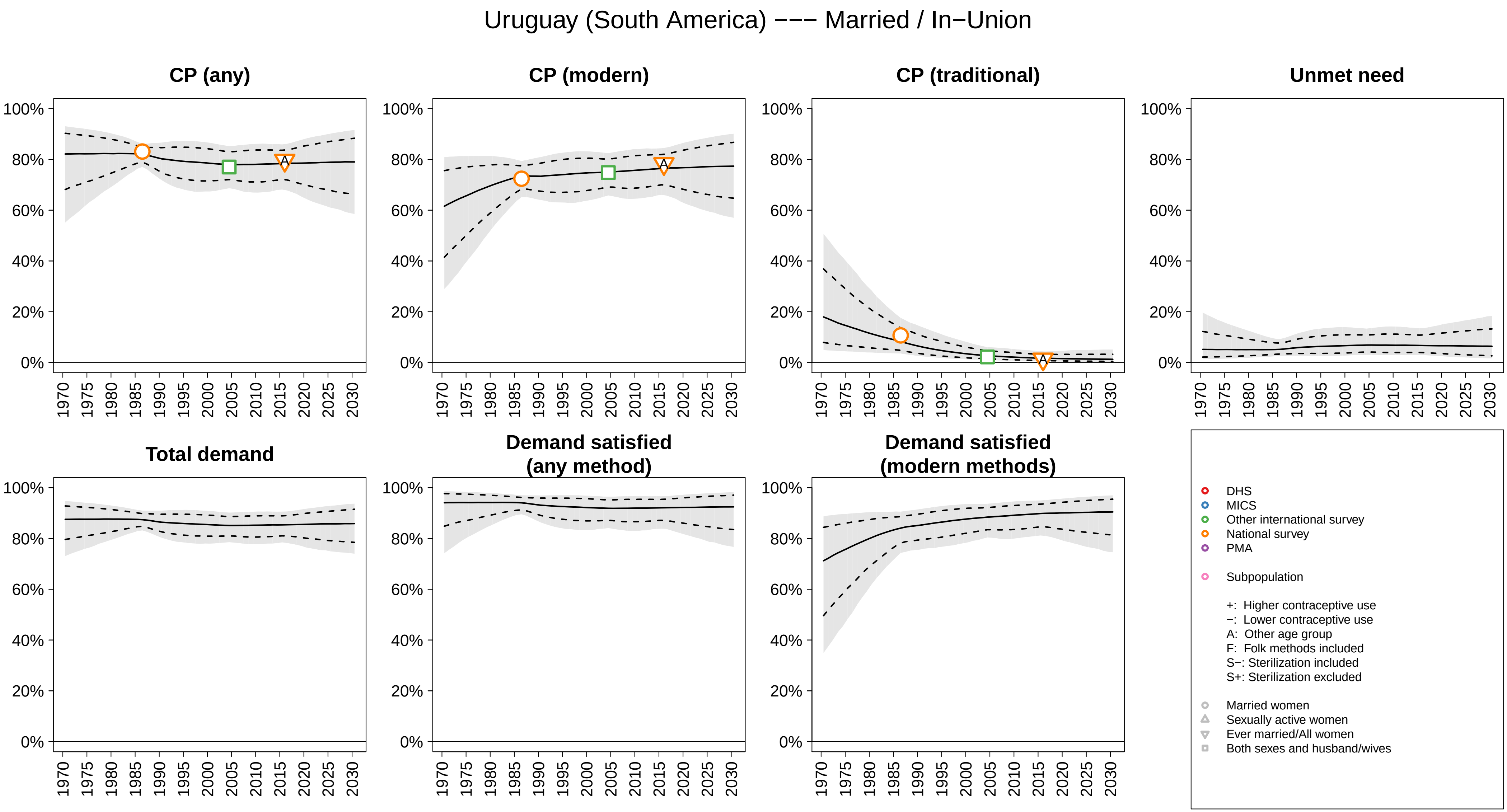
Uruguay (South America) --- Married / In−Union
CP (any)
CP (modern)
CP (traditional)
Unmet need
Total demand
Demand satisfied (any method)
Demand satisfied (modern methods)
0%
20%
40%
60%
80%
100%
1970
1975
1980
1985
1990
1995
2000
2005
2010
2015
2020
2025
2030
DHS
MICS
Other international survey
National survey
PMA
Subpopulation
+: Higher contraceptive use
−: Lower contraceptive use
A: Other age group
F: Folk methods included
S−: Sterilization included
S+: Sterilization excluded
Married women
Sexually active women
Ever married/All women
Both sexes and husband/wives

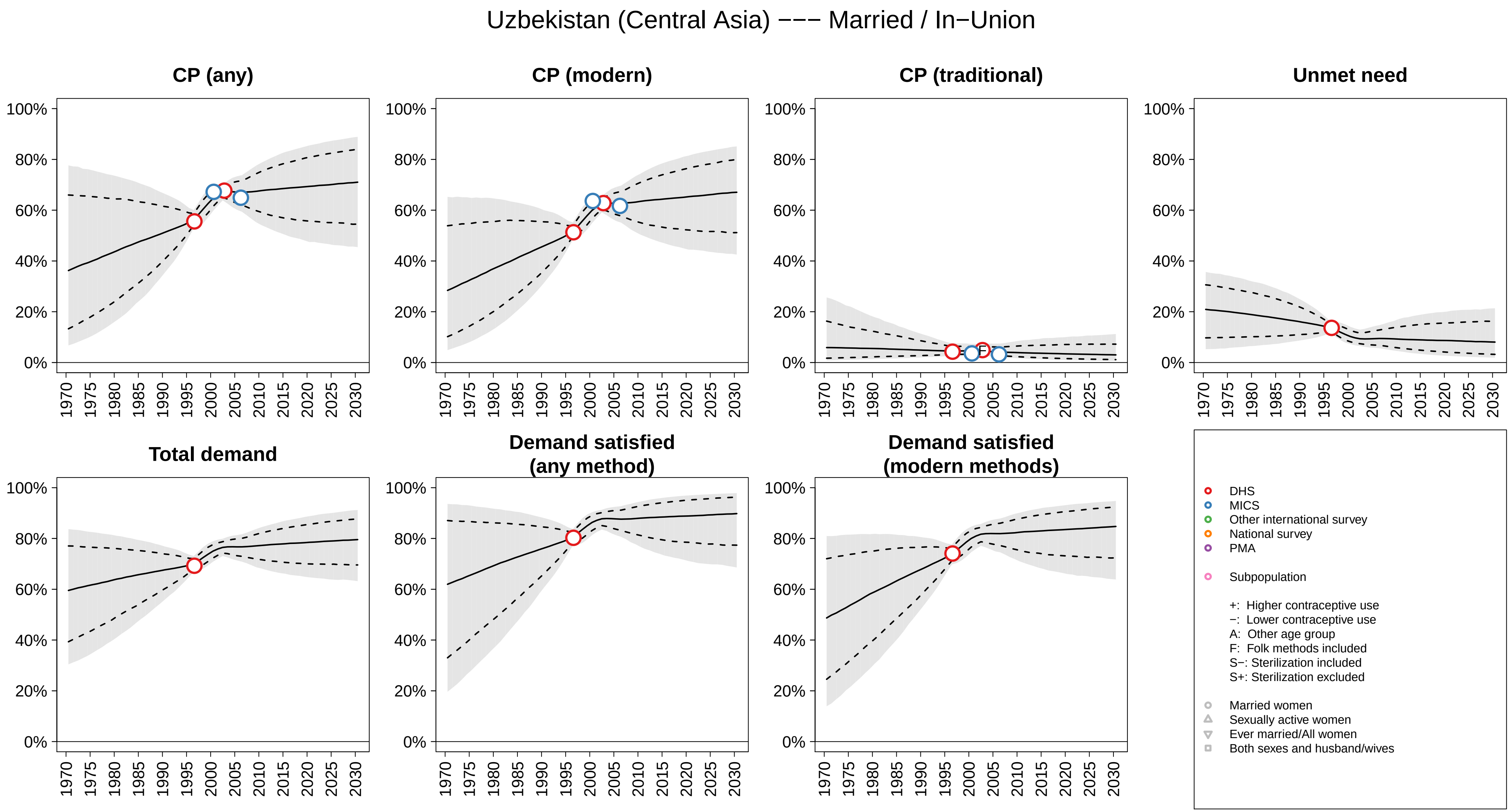

Uzbekistan (Central Asia) --- Married / In−Union
CP (any)
CP (modern)
CP (traditional)
Unmet need
Total demand
Demand satisfied (any method)
Demand satisfied (modern methods)
0%
20%
40%
60%
80%
100%
1970
1975
1980
1985
1990
1995
2000
2005
2010
2015
2020
2025
2030
F
DHS
MICS
Other international survey
National survey
PMA
Subpopulation
+: Higher contraceptive use
−: Lower contraceptive use
A: Other age group
F: Folk methods included
S−: Sterilization included
S+: Sterilization excluded
Married women
Sexually active women
Ever married/All women
Both sexes and husband/wives

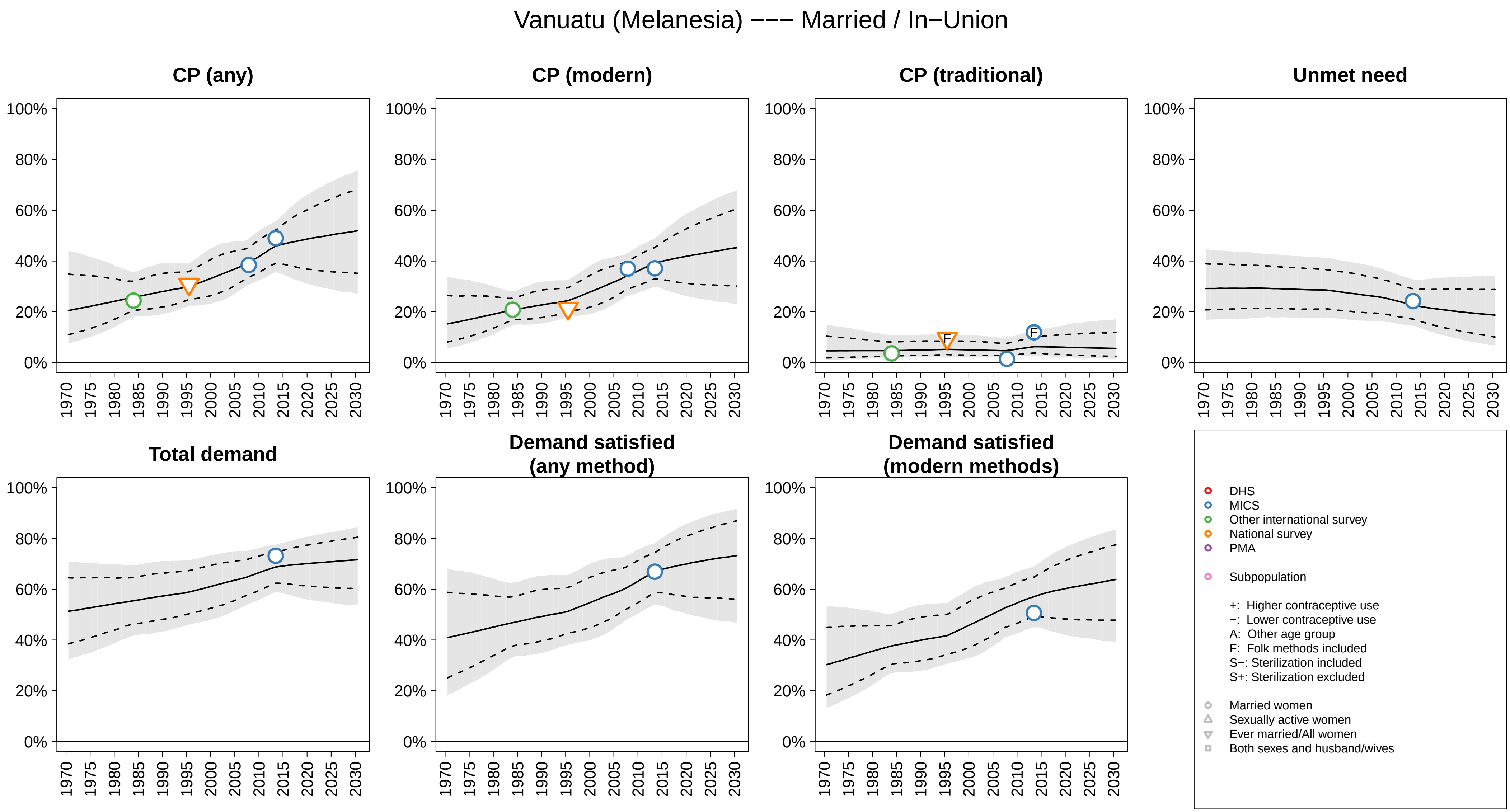
Vanuatu (Melanesia) −−− Married / In−Union
CP (any)
CP (modern)
CP (traditional)
Unmet need
Total demand
Demand satisfied (any method)
Demand satisfied (modern methods)
0%
20%
40%
60%
80%
100%
1970
1975
1980
1985
1990
1995
2000
2005
2010
2015
2020
2025
2030
F
DHS
MICS
Other international survey
National survey
PMA
Subpopulation
+: Higher contraceptive use
−: Lower contraceptive use
A: Other age group
F: Folk methods included
S−: Sterilization included
S+: Sterilization excluded
Married women
Sexually active women
Ever married/All women
Both sexes and husband/wives

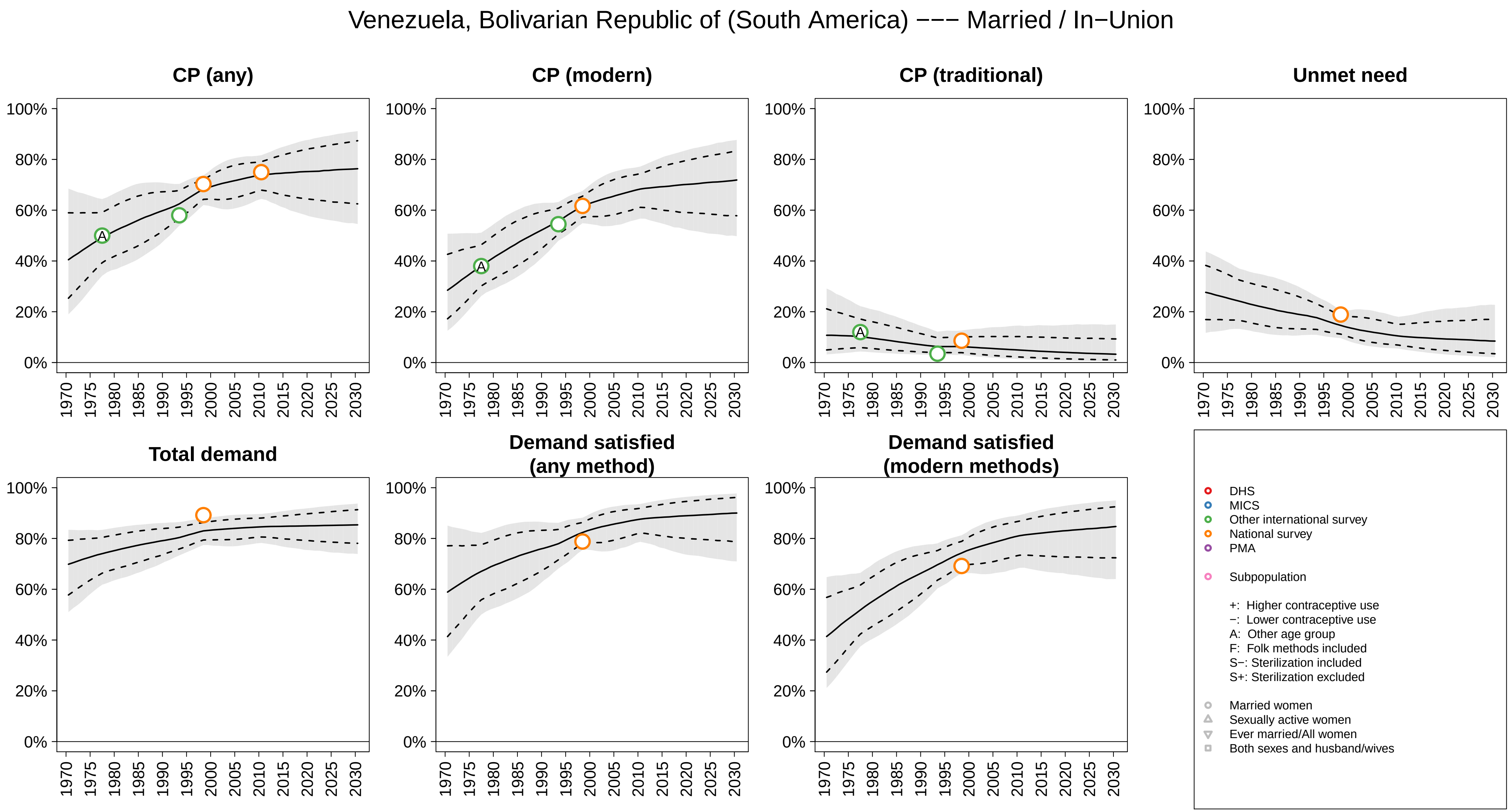
Venezuela, Bolivarian Republic of (South America) −−− Married / In−Union
CP (any)
CP (modern)
CP (traditional)
Unmet need
Total demand
Demand satisfied (any method)
Demand satisfied (modern methods)
0%
20%
40%
60%
80%
100%
1970
1975
1980
1985
1990
1995
2000
2005
2010
2015
2020
2025
2030
DHS
MICS
Other international survey
National survey
PMA
Subpopulation
+: Higher contraceptive use
−: Lower contraceptive use
A: Other age group
F: Folk methods included
S−: Sterilization included
S+: Sterilization excluded
Married women
Sexually active women
Ever married/All women
Both sexes and husband/wives

Viet Nam (South−eastern Asia) −−− Married / In−Union

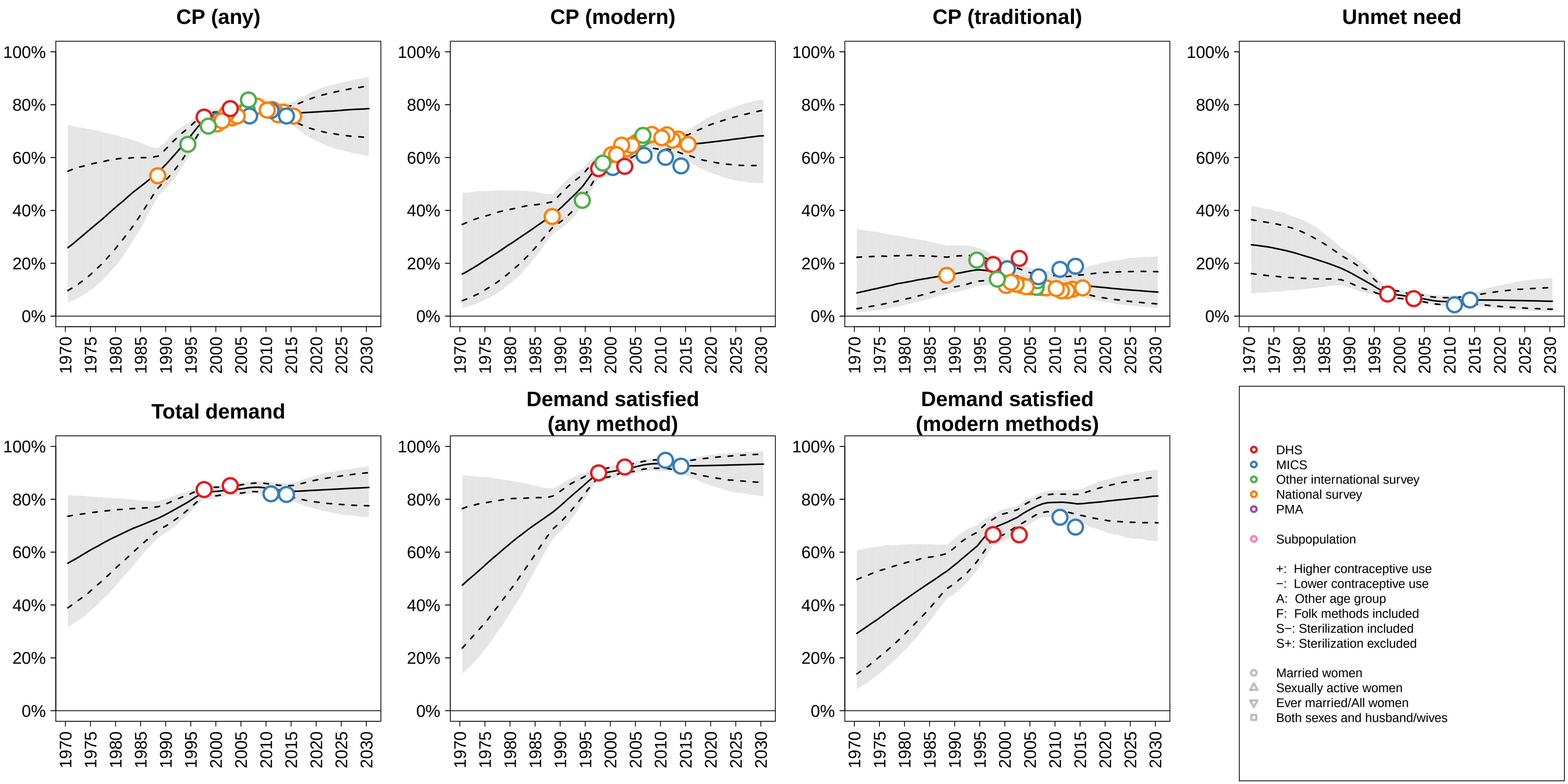

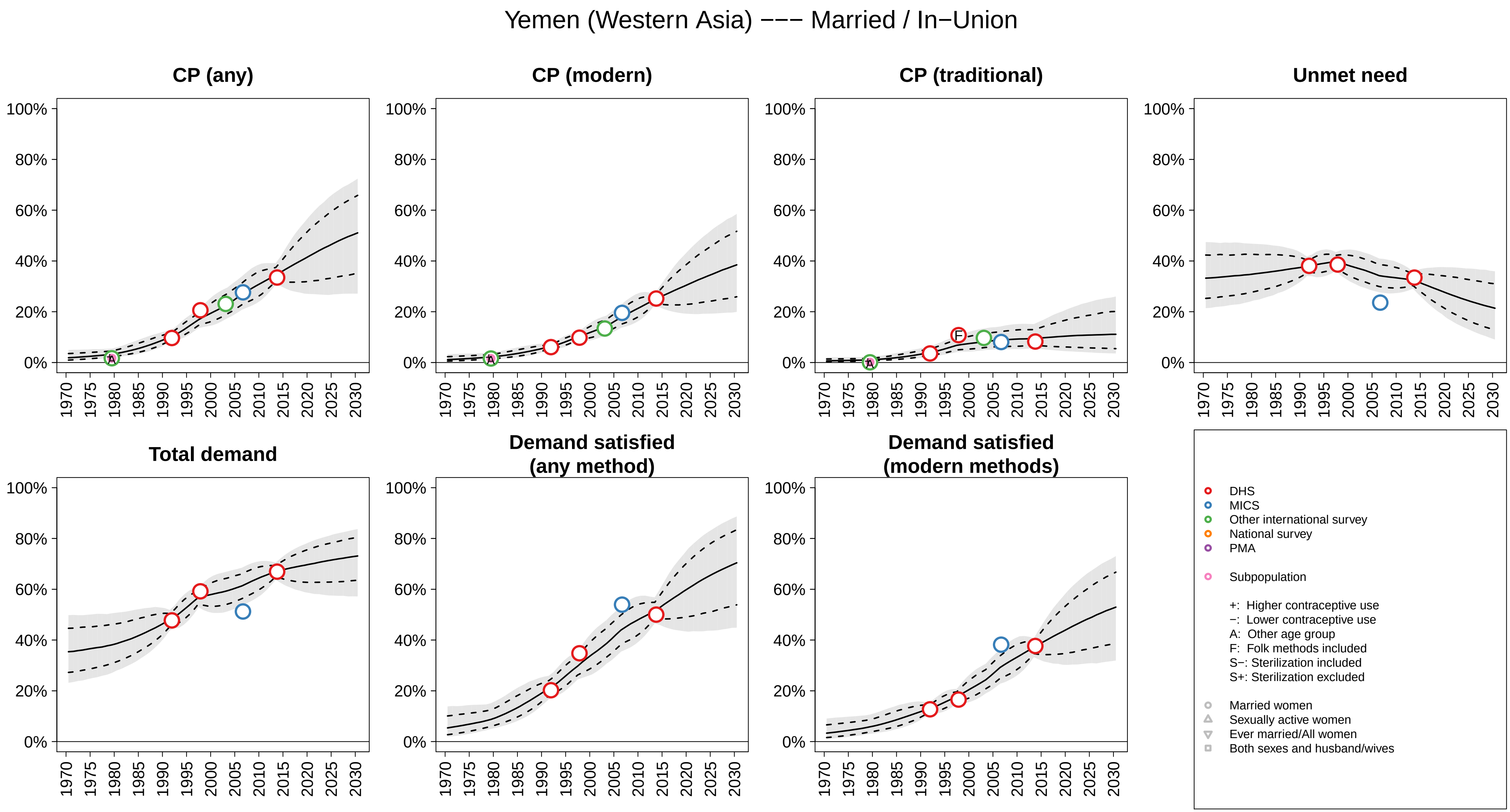

Yemen (Western Asia) −−− Married / In−Union
CP (any)
CP (modern)
CP (traditional)
Unmet need
Total demand
Demand satisfied (any method)
Demand satisfied (modern methods)
0%
20%
40%
60%
80%
100%
1970
1975
1980
1985
1990
1995
2000
2005
2010
2015
2020
2025
2030
A
F
DHS
MICS
Other international survey
National survey
PMA
Subpopulation
+: Higher contraceptive use
−: Lower contraceptive use
A: Other age group
F: Folk methods included
S−: Sterilization included
S+: Sterilization excluded
Married women
Sexually active women
Ever married/All women
Both sexes and husband/wives

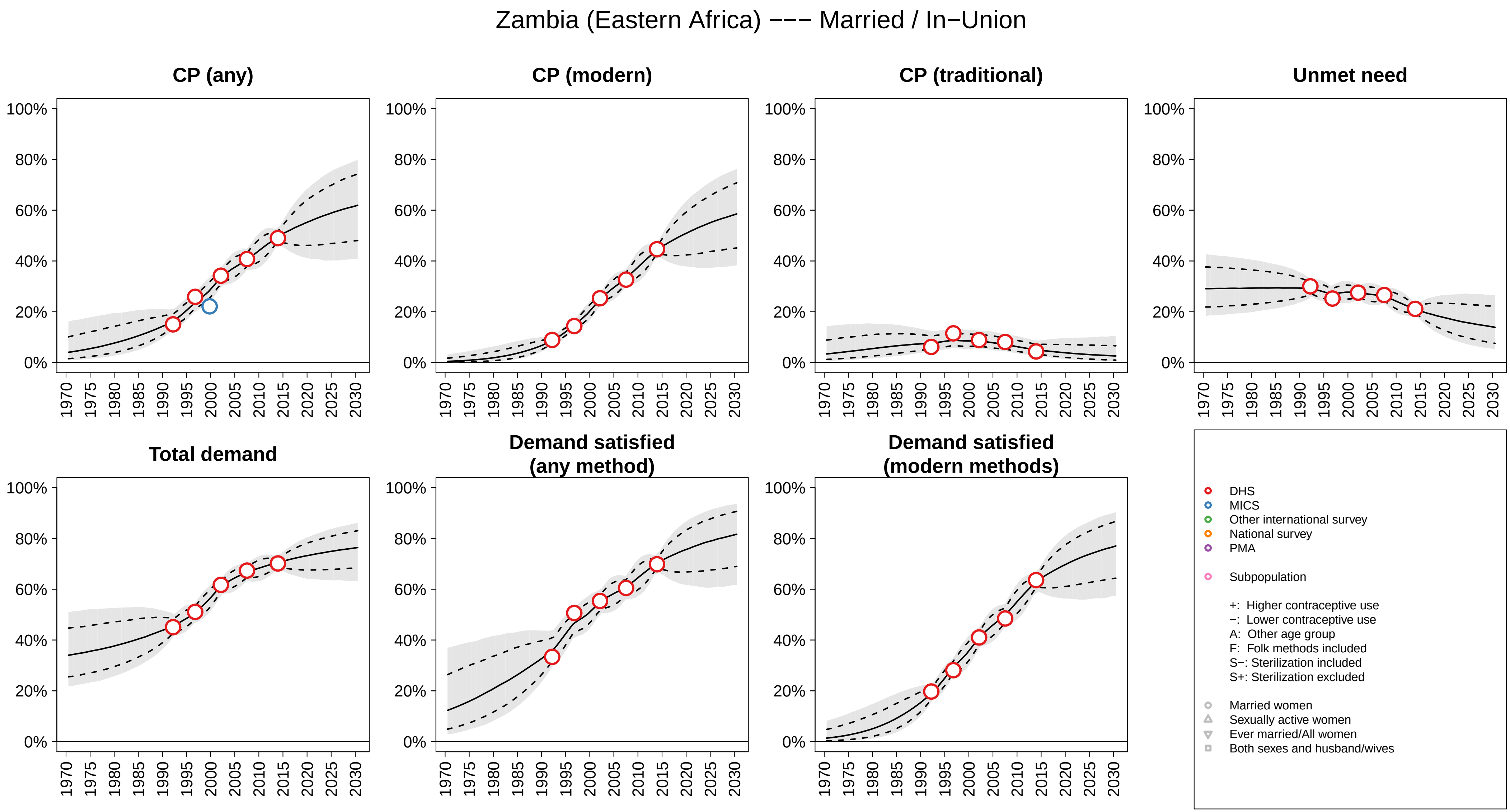

Zambia (Eastern Africa) −−− Married / In−Union
CP (any)
CP (modern)
CP (traditional)
Unmet need
Total demand
Demand satisfied (any method)
Demand satisfied (modern methods)
100%
80%
60%
40%
20%
0%
1970 1975 1980 1985 1990 1995 2000 2005 2010 2015 2020 2025 2030
DHS
MICS
Other international survey
National survey
PMA
Subpopulation
+: Higher contraceptive use
−: Lower contraceptive use
A: Other age group
F: Folk methods included
S−: Sterilization included
S+: Sterilization excluded
Married women
Sexually active women
Ever married/All women
Both sexes and husband/wives

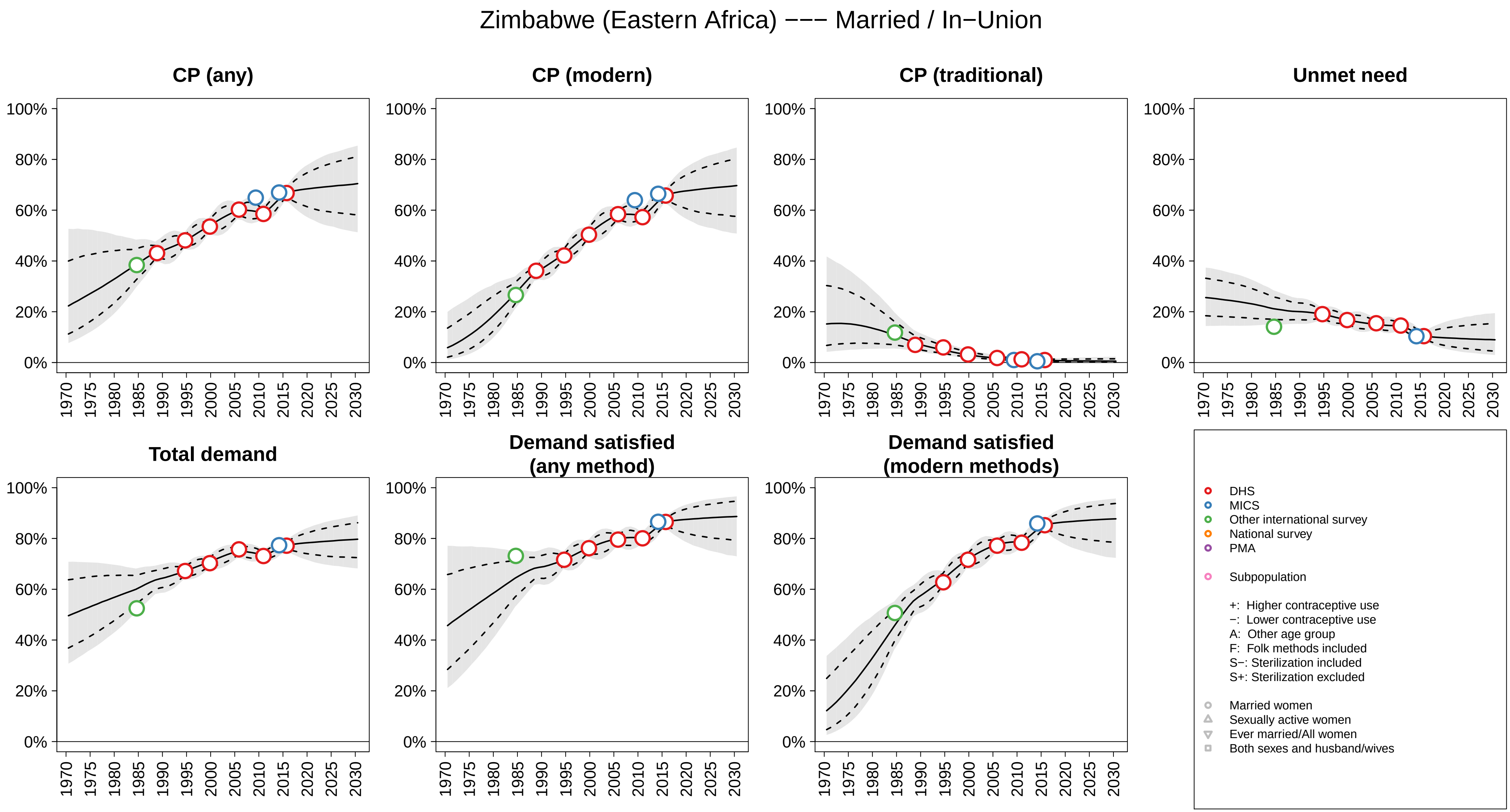
Zimbabwe (Eastern Africa) −−− Married / In−Union
CP (any)
CP (modern)
CP (traditional)
Unmet need
Total demand
Demand satisfied (any method)
Demand satisfied (modern methods)
0%
20%
40%
60%
80%
100%
1970
1975
1980
1985
1990
1995
2000
2005
2010
2015
2020
2025
2030
DHS
MICS
Other international survey
National survey
PMA
Subpopulation
+: Higher contraceptive use
−: Lower contraceptive use
A: Other age group
F: Folk methods included
S−: Sterilization included
S+: Sterilization excluded
Married women
Sexually active women
Ever married/All women
Both sexes and husband/wives

2.2 Country aggregates

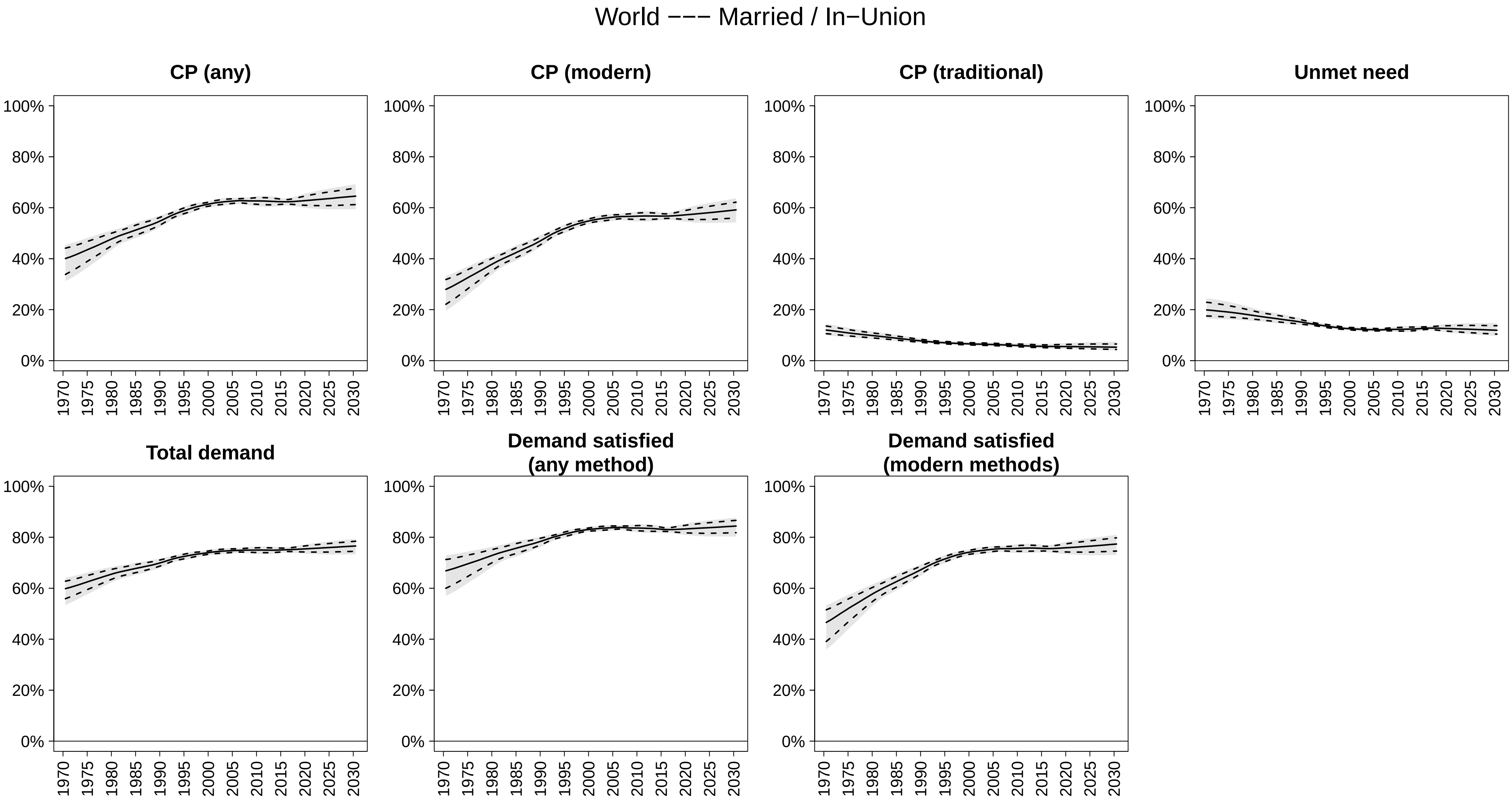
World −−− Married / In−Union
CP (any)
CP (modern)
CP (traditional)
Unmet need
Total demand
Demand satisfied (any method)
Demand satisfied (modern methods)
0%
20%
40%
60%
80%
100%
1970
1975
1980
1985
1990
1995
2000
2005
2010
2015
2020
2025
2030

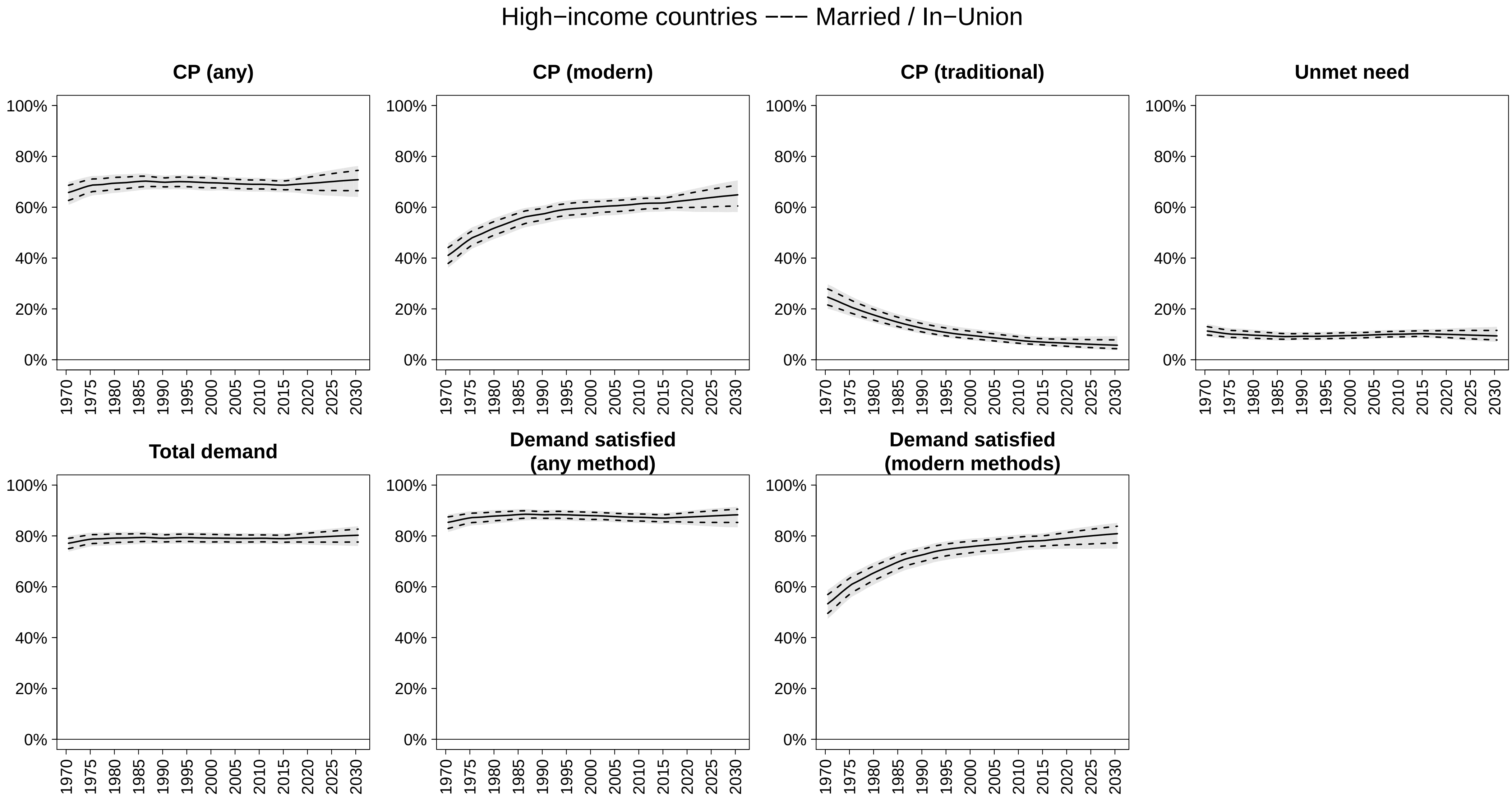
High−income countries −−− Married / In−Union
CP (any)
CP (modern)
CP (traditional)
Unmet need
Total demand
Demand satisfied (any method)
Demand satisfied (modern methods)
100%
80%
60%
40%
20%
0%
1970
1975
1980
1985
1990
1995
2000
2005
2010
2015
2020
2025
2030

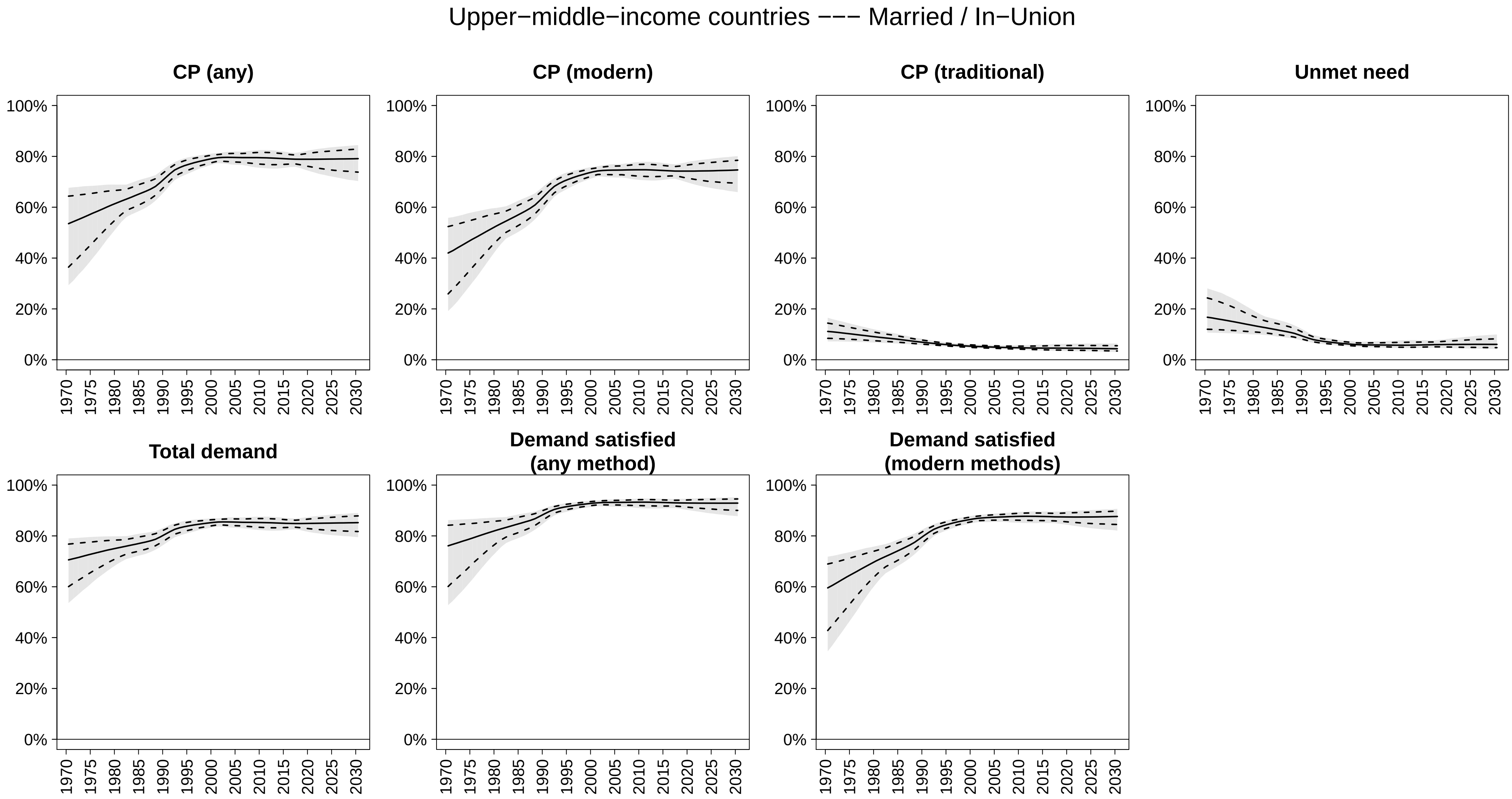
Upper−middle−income countries −−− Married / In−Union
CP (any)
CP (modern)
CP (traditional)
Unmet need
Total demand
Demand satisfied (any method)
Demand satisfied (modern methods)
0%
20%
40%
60%
80%
100%
1970
1975
1980
1985
1990
1995
2000
2005
2010
2015
2020
2025
2030

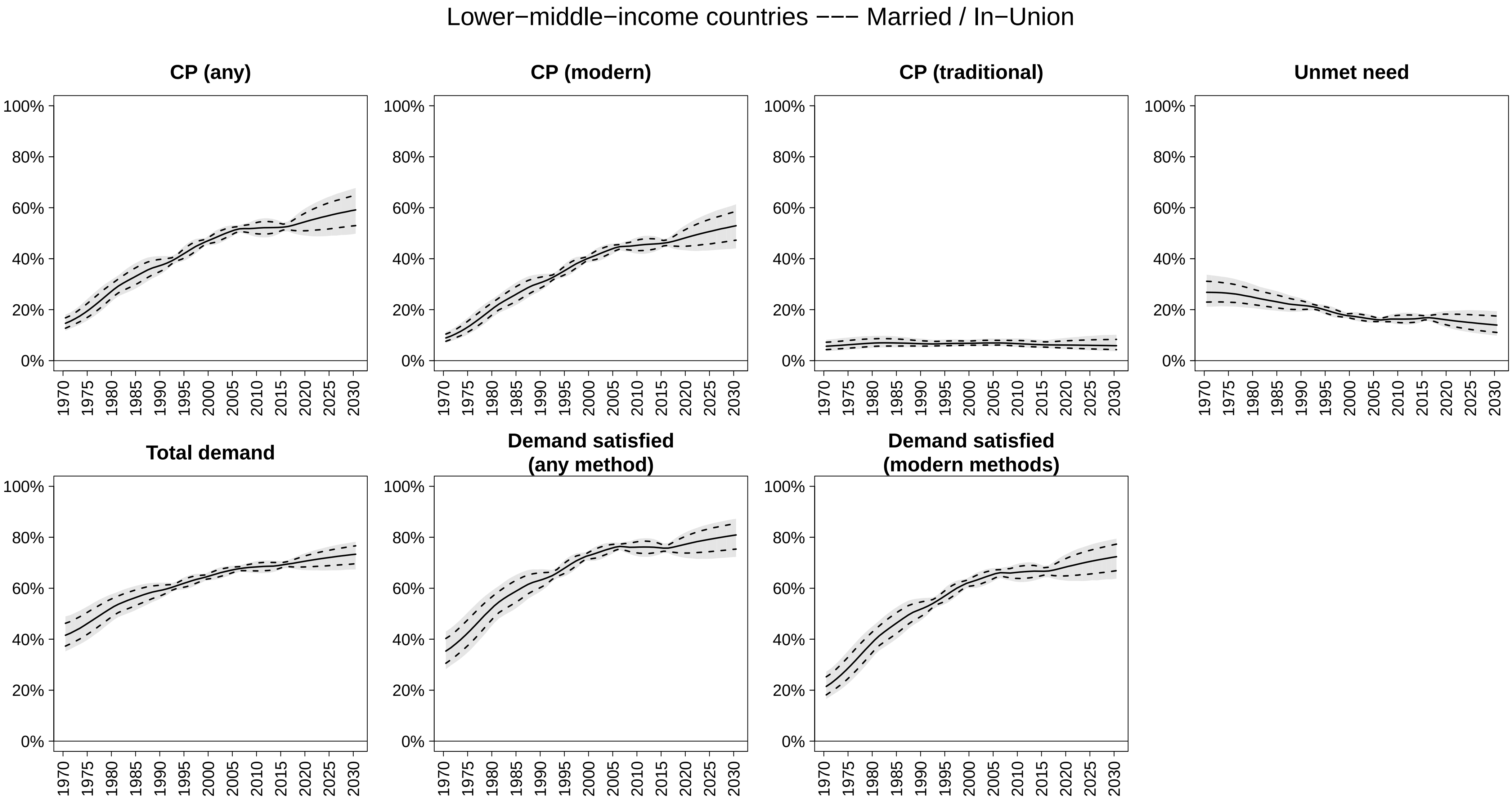
Lower−middle−income countries −−− Married / In−Union
CP (any)
CP (modern)
CP (traditional)
Unmet need
Total demand
Demand satisfied (any method)
Demand satisfied (modern methods)
0%
20%
40%
60%
80%
100%
1970
1975
1980
1985
1990
1995
2000
2005
2010
2015
2020
2025
2030

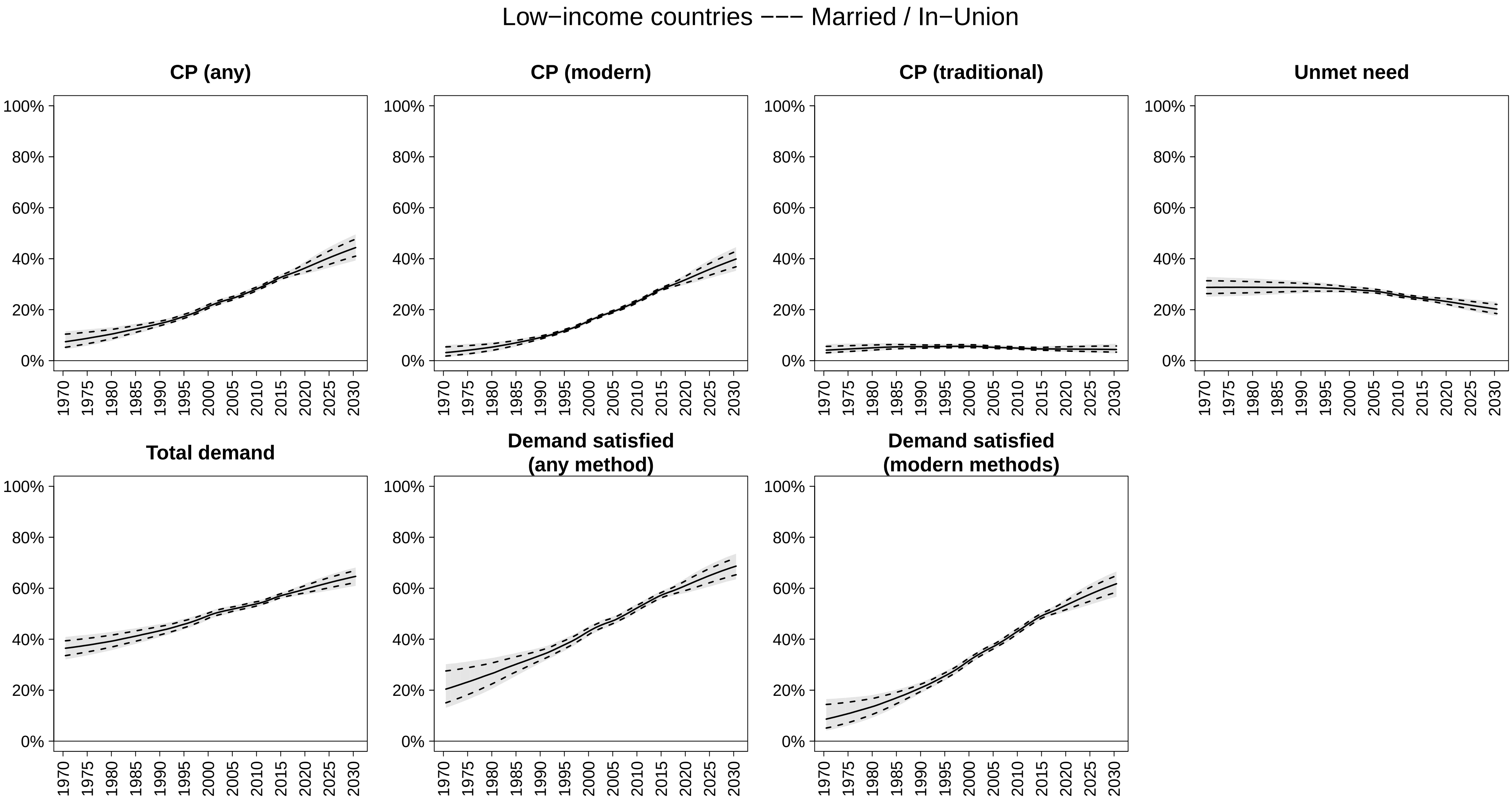
Low−income countries −−− Married / In−Union
CP (any)
CP (modern)
CP (traditional)
Unmet need
Total demand
Demand satisfied (any method)
Demand satisfied (modern methods)
0%
20%
40%
60%
80%
100%
1970
1975
1980
1985
1990
1995
2000
2005
2010
2015
2020
2025
2030

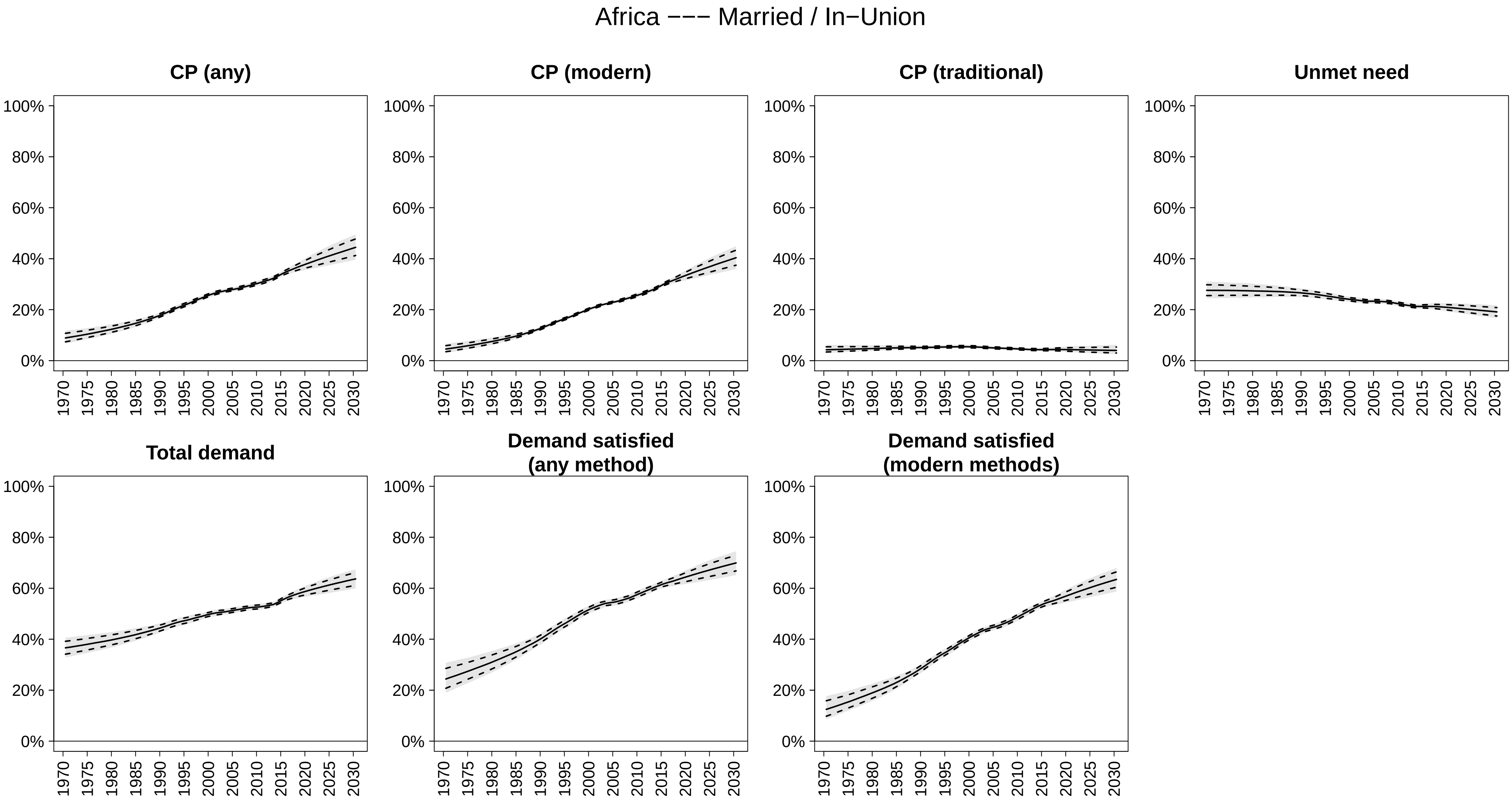
Africa −−− Married / In−Union
CP (any)
CP (modern)
CP (traditional)
Unmet need
Total demand
Demand satisfied (any method)
Demand satisfied (modern methods)
0%
20%
40%
60%
80%
100%
1970
1975
1980
1985
1990
1995
2000
2005
2010
2015
2020
2025
2030

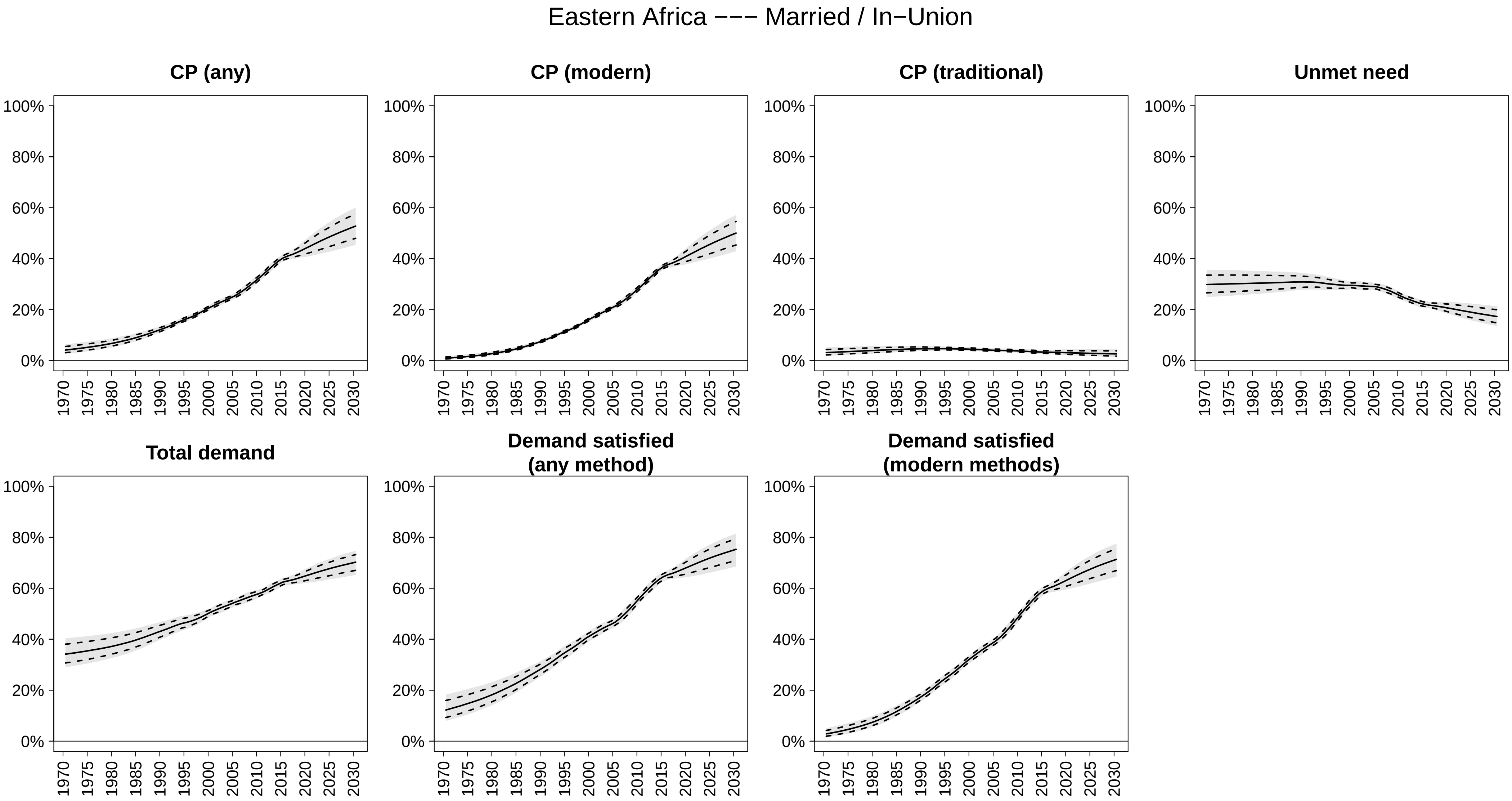
Eastern Africa −−− Married / In−Union
CP (any)
CP (modern)
CP (traditional)
Unmet need
Total demand
Demand satisfied (any method)
Demand satisfied (modern methods)
0%
20%
40%
60%
80%
100%
1970
1975
1980
1985
1990
1995
2000
2005
2010
2015
2020
2025
2030

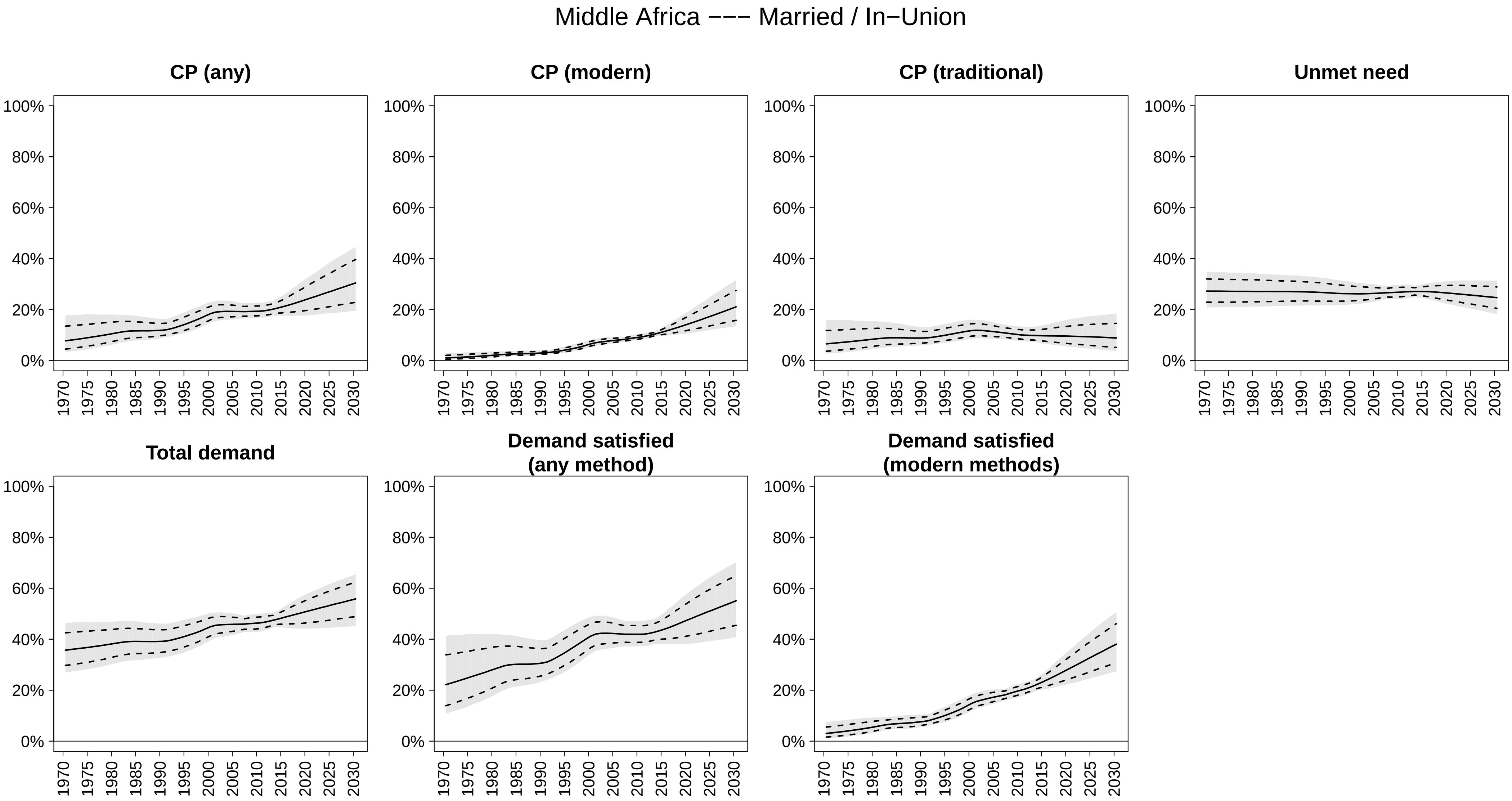
Middle Africa −−− Married / In−Union
CP (any)
CP (modern)
CP (traditional)
Unmet need
Total demand
Demand satisfied (any method)
Demand satisfied (modern methods)
0%
20%
40%
60%
80%
100%
1970
1975
1980
1985
1990
1995
2000
2005
2010
2015
2020
2025
2030

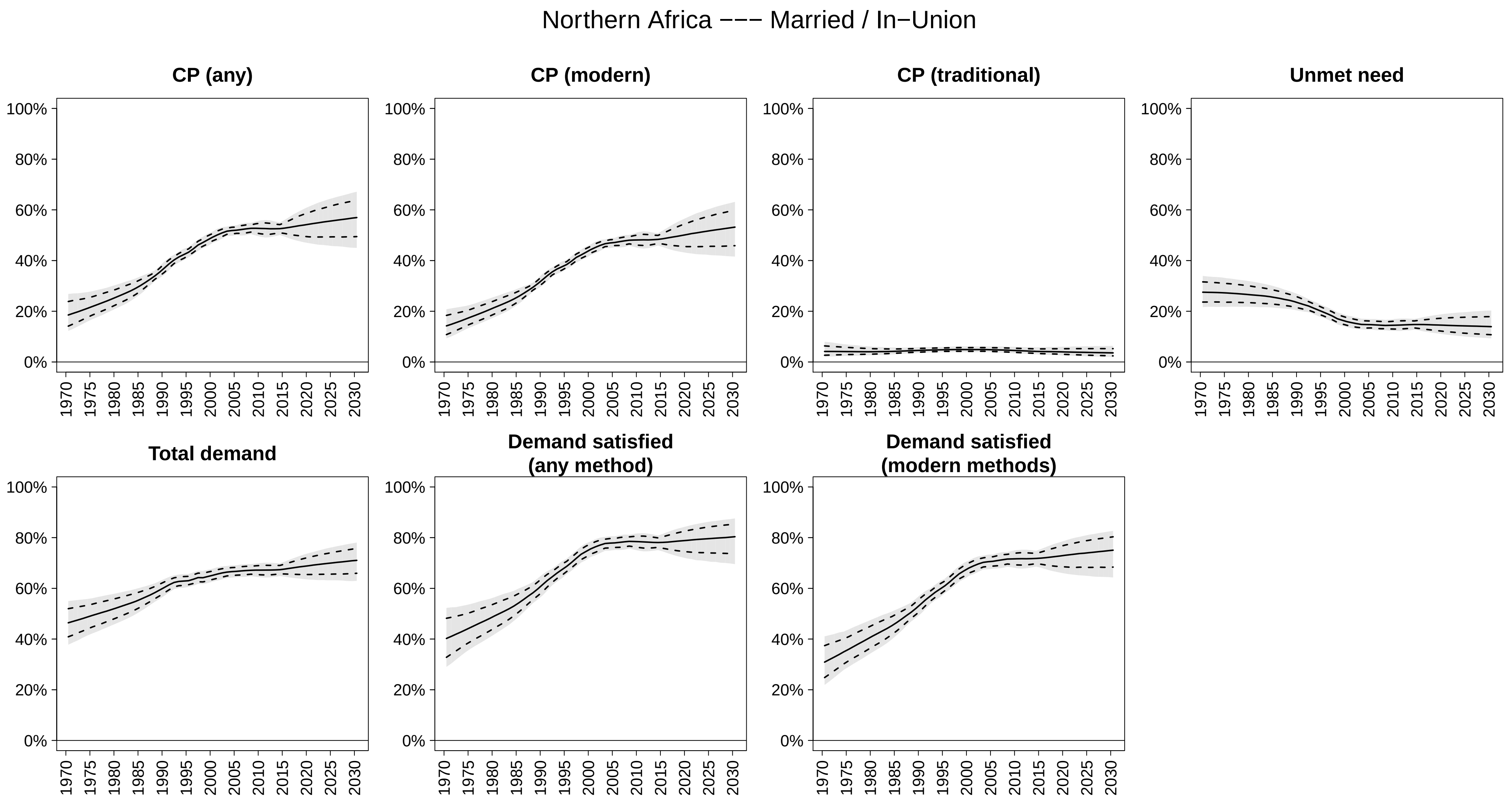
Northern Africa −−− Married / In−Union
CP (any)
CP (modern)
CP (traditional)
Unmet need
Total demand
Demand satisfied (any method)
Demand satisfied (modern methods)
0%
20%
40%
60%
80%
100%
1970
1975
1980
1985
1990
1995
2000
2005
2010
2015
2020
2025
2030

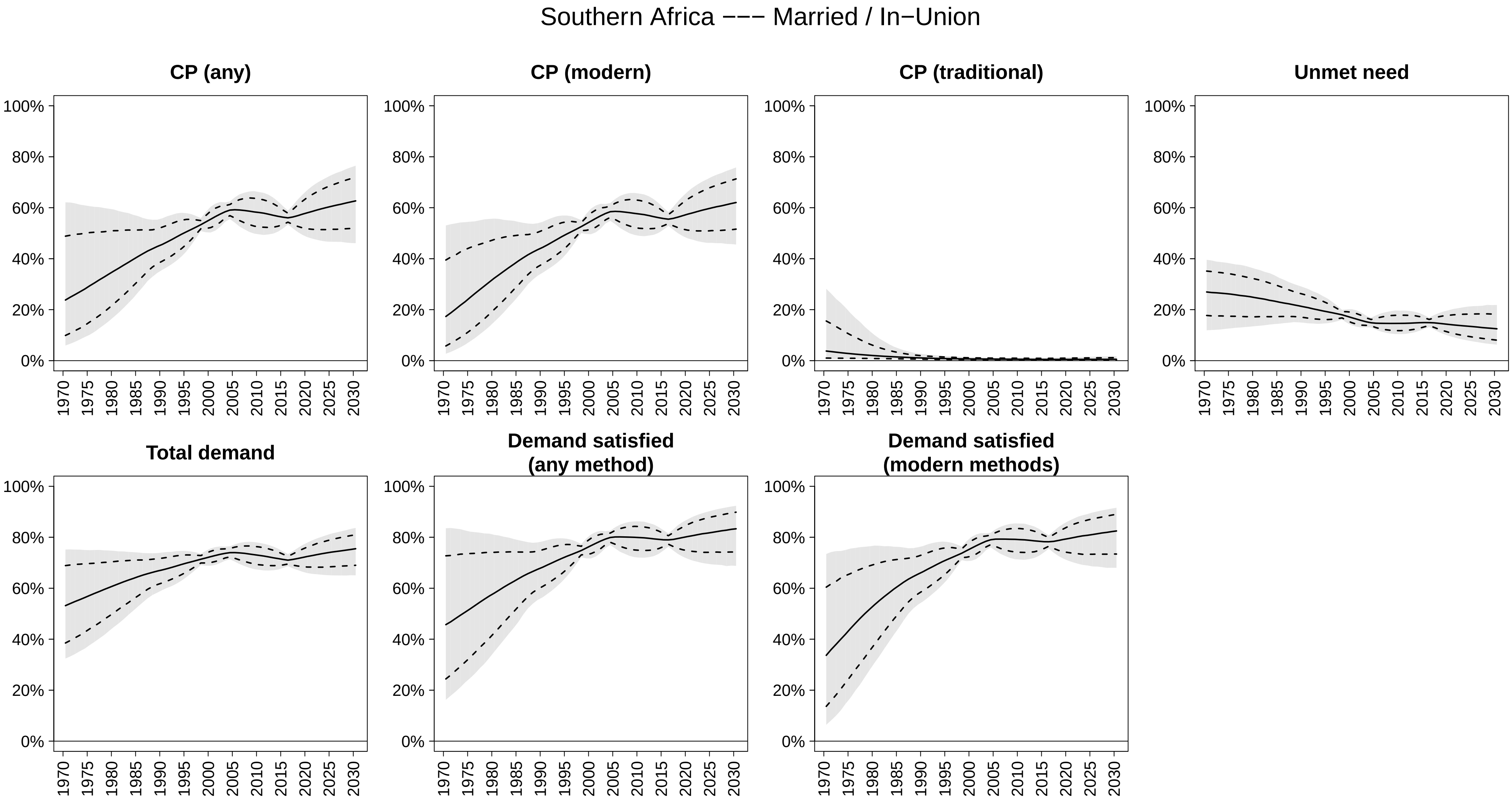
Southern Africa −−− Married / In−Union
CP (any)
CP (modern)
CP (traditional)
Unmet need
Total demand
Demand satisfied (any method)
Demand satisfied (modern methods)
0%
20%
40%
60%
80%
100%
1970
1975
1980
1985
1990
1995
2000
2005
2010
2015
2020
2025
2030

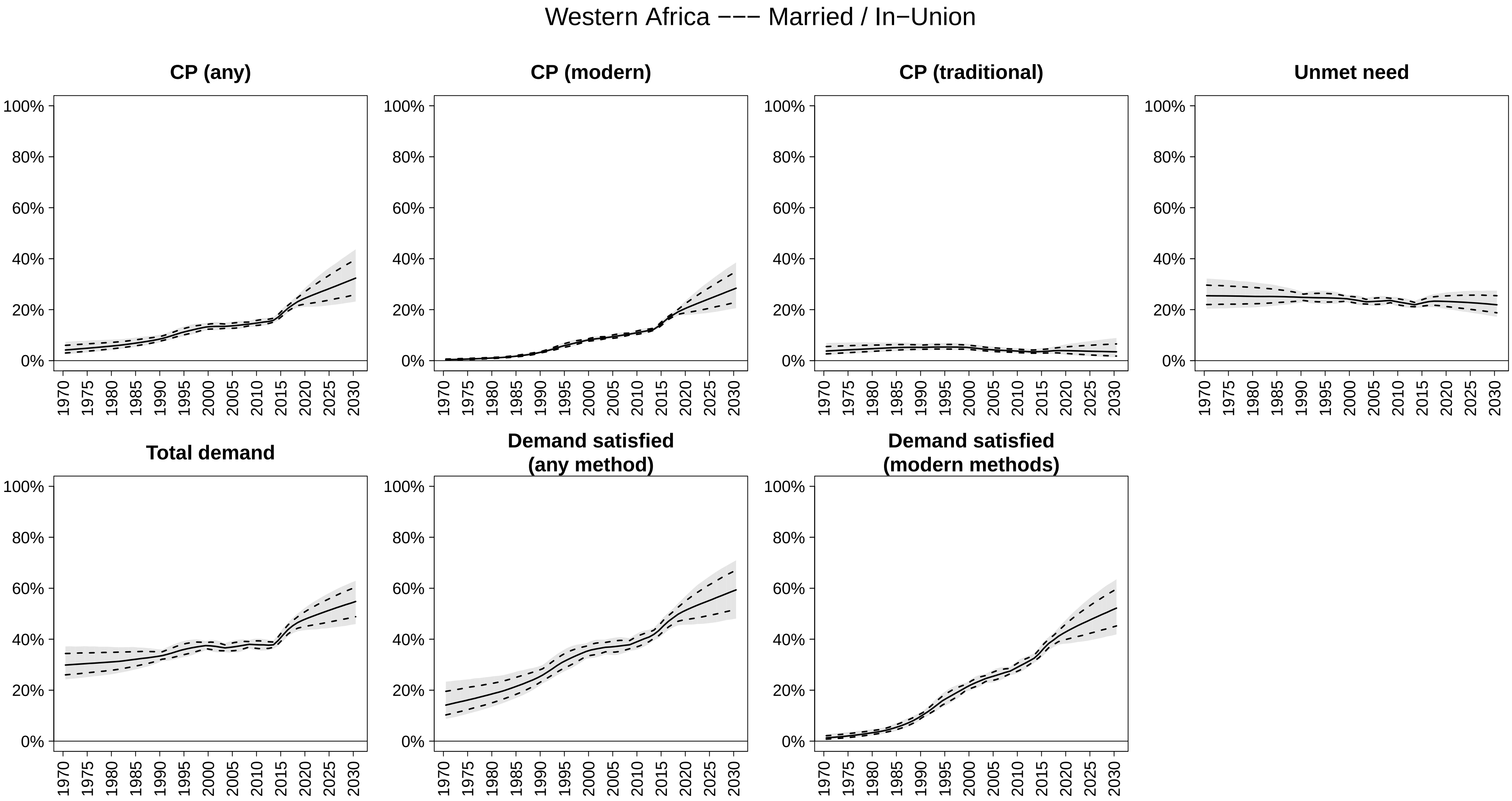
Western Africa −−− Married / In−Union
CP (any)
CP (modern)
CP (traditional)
Unmet need
Total demand
Demand satisfied (any method)
Demand satisfied (modern methods)
0%
20%
40%
60%
80%
100%
1970
1975
1980
1985
1990
1995
2000
2005
2010
2015
2020
2025
2030

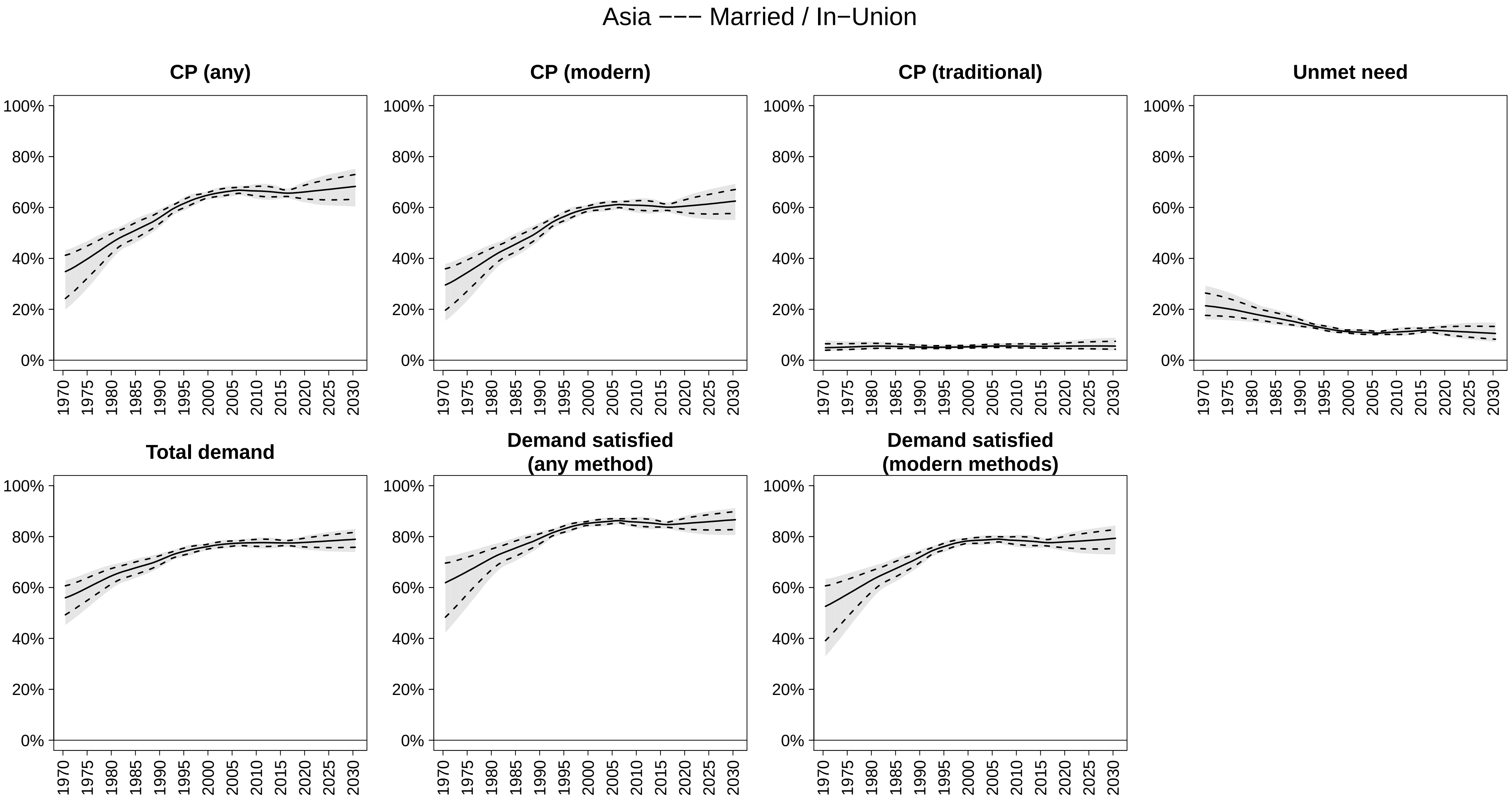
Asia −−− Married / In−Union
CP (any)
CP (modern)
CP (traditional)
Unmet need
Total demand
Demand satisfied (any method)
Demand satisfied (modern methods)
0%
20%
40%
60%
80%
100%
1970
1975
1980
1985
1990
1995
2000
2005
2010
2015
2020
2025
2030

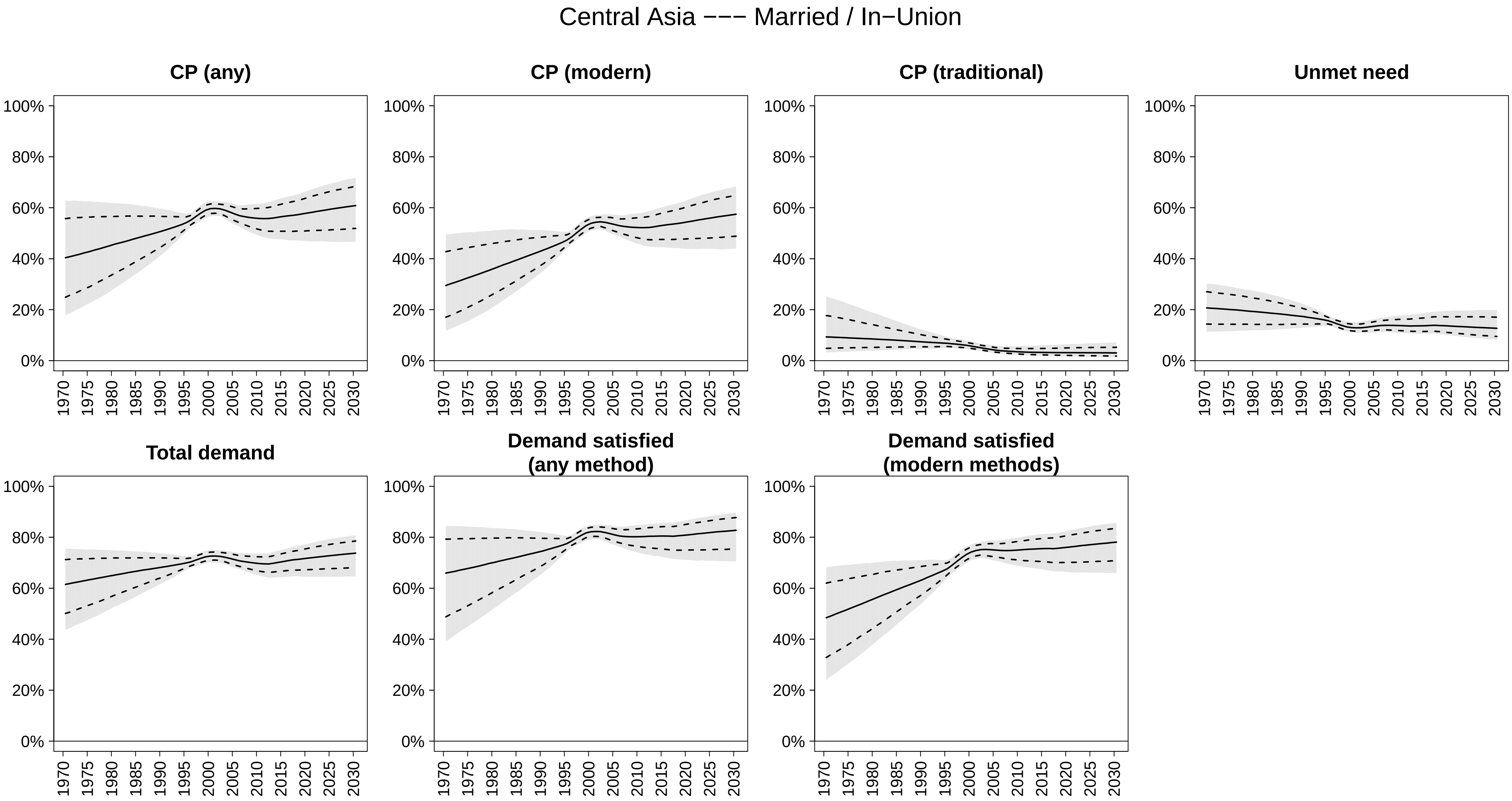
Central Asia −−− Married / In−Union
CP (any)
CP (modern)
CP (traditional)
Unmet need
Total demand
Demand satisfied (any method)
Demand satisfied (modern methods)
0%
20%
40%
60%
80%
100%
1970
1975
1980
1985
1990
1995
2000
2005
2010
2015
2020
2025
2030

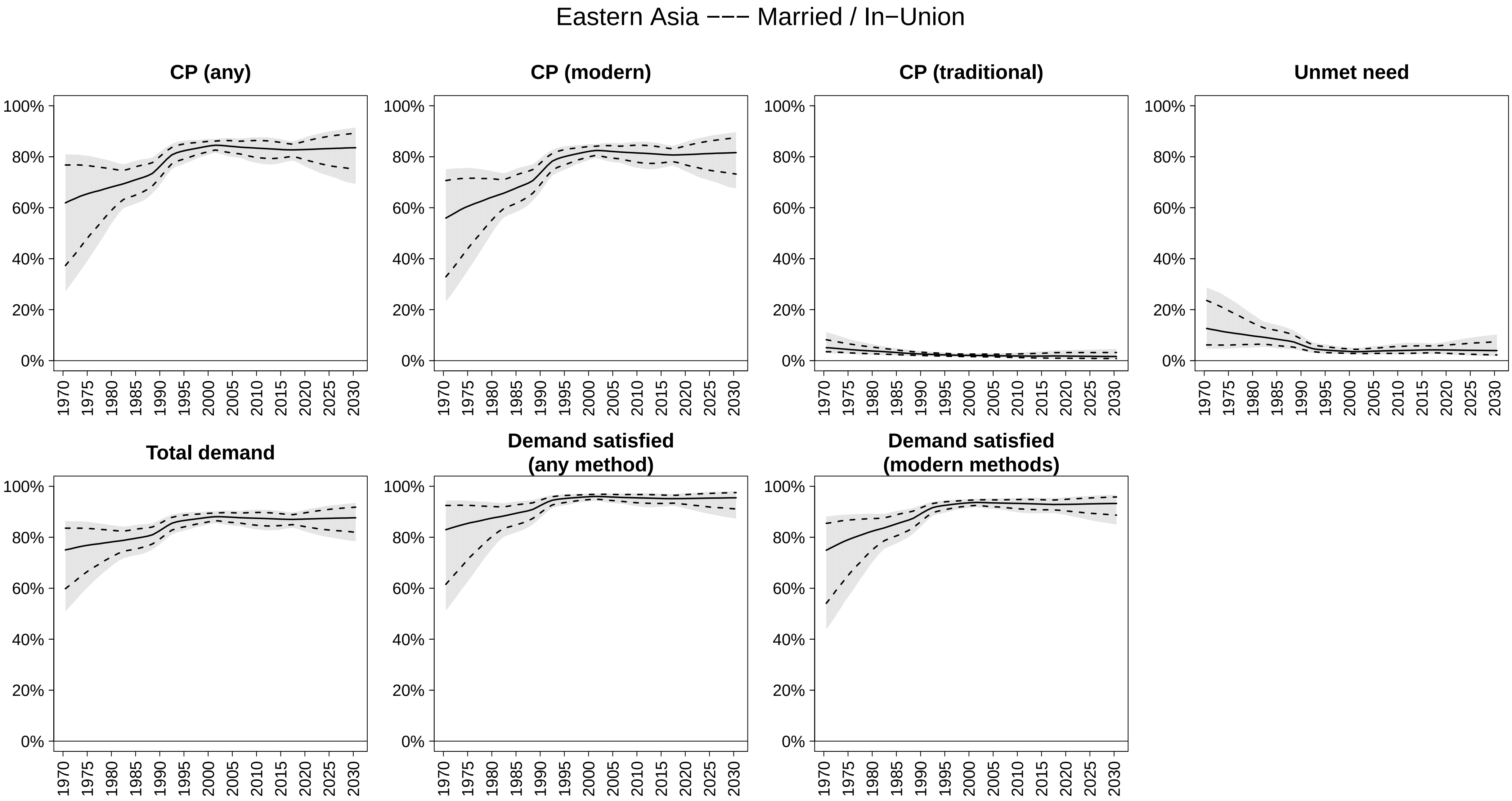

Eastern Asia −−− Married / In−Union
CP (any)
CP (modern)
CP (traditional)
Unmet need
Total demand
Demand satisfied (any method)
Demand satisfied (modern methods)
0%
20%
40%
60%
80%
100%
1970
1975
1980
1985
1990
1995
2000
2005
2010
2015
2020
2025
2030

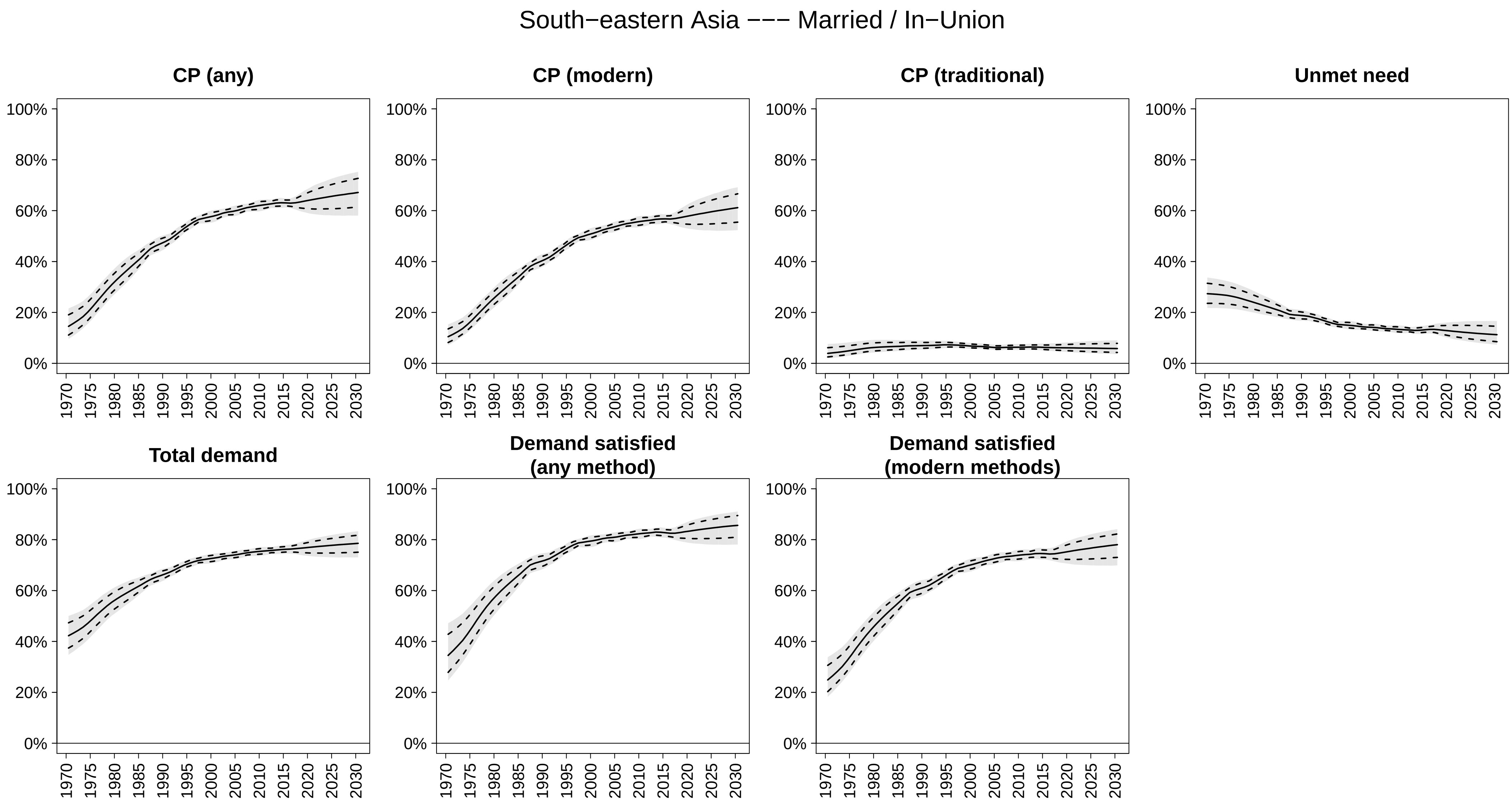
South−eastern Asia −−− Married / In−Union
CP (any)
CP (modern)
CP (traditional)
Unmet need
Total demand
Demand satisfied (any method)
Demand satisfied (modern methods)
0%
20%
40%
60%
80%
100%
1970
1975
1980
1985
1990
1995
2000
2005
2010
2015
2020
2025
2030

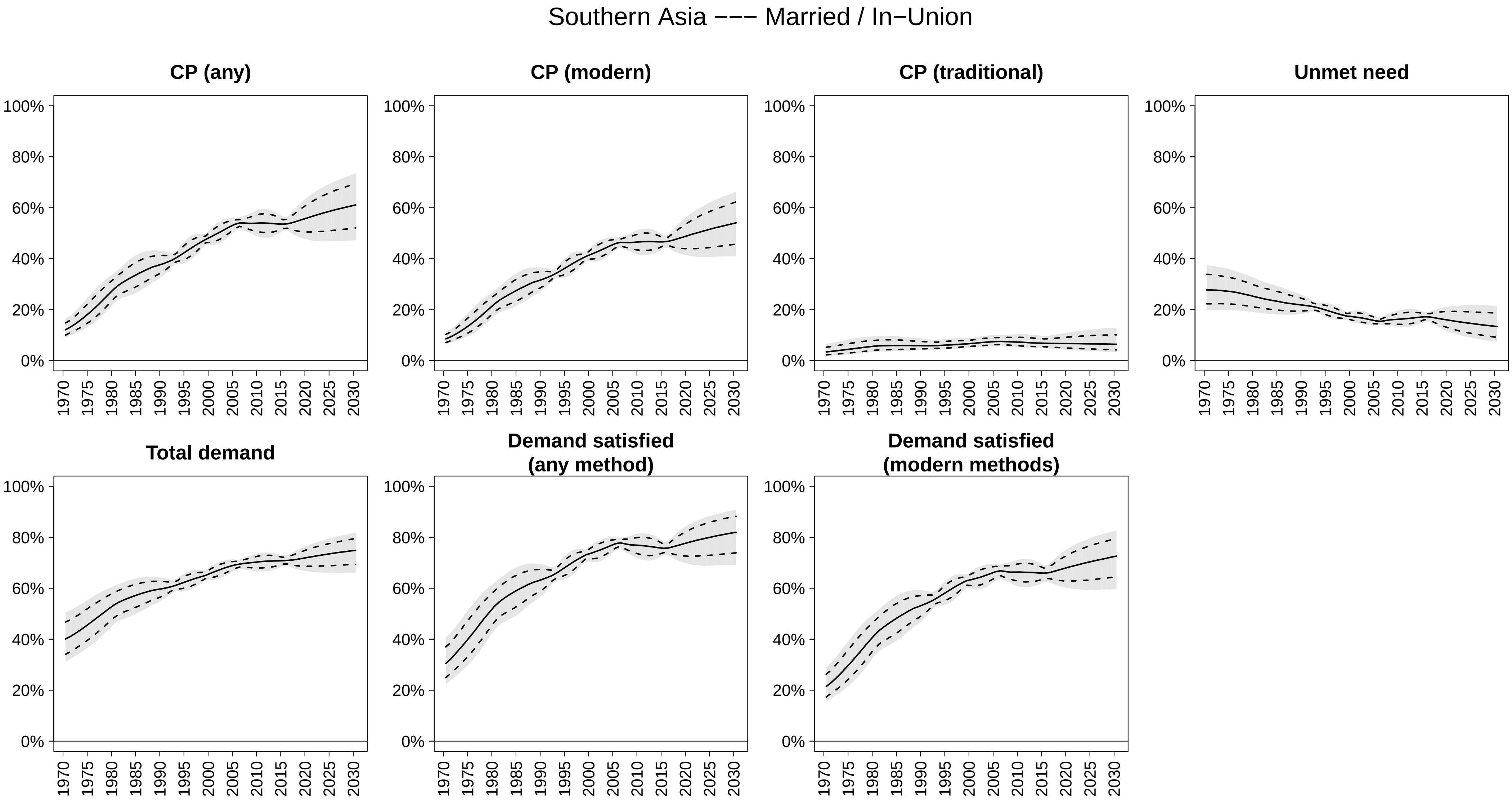
Southern Asia −−− Married / In−Union
CP (any)
CP (modern)
CP (traditional)
Unmet need
Total demand
Demand satisfied (any method)
Demand satisfied (modern methods)
0%
20%
40%
60%
80%
100%
1970
1975
1980
1985
1990
1995
2000
2005
2010
2015
2020
2025
2030

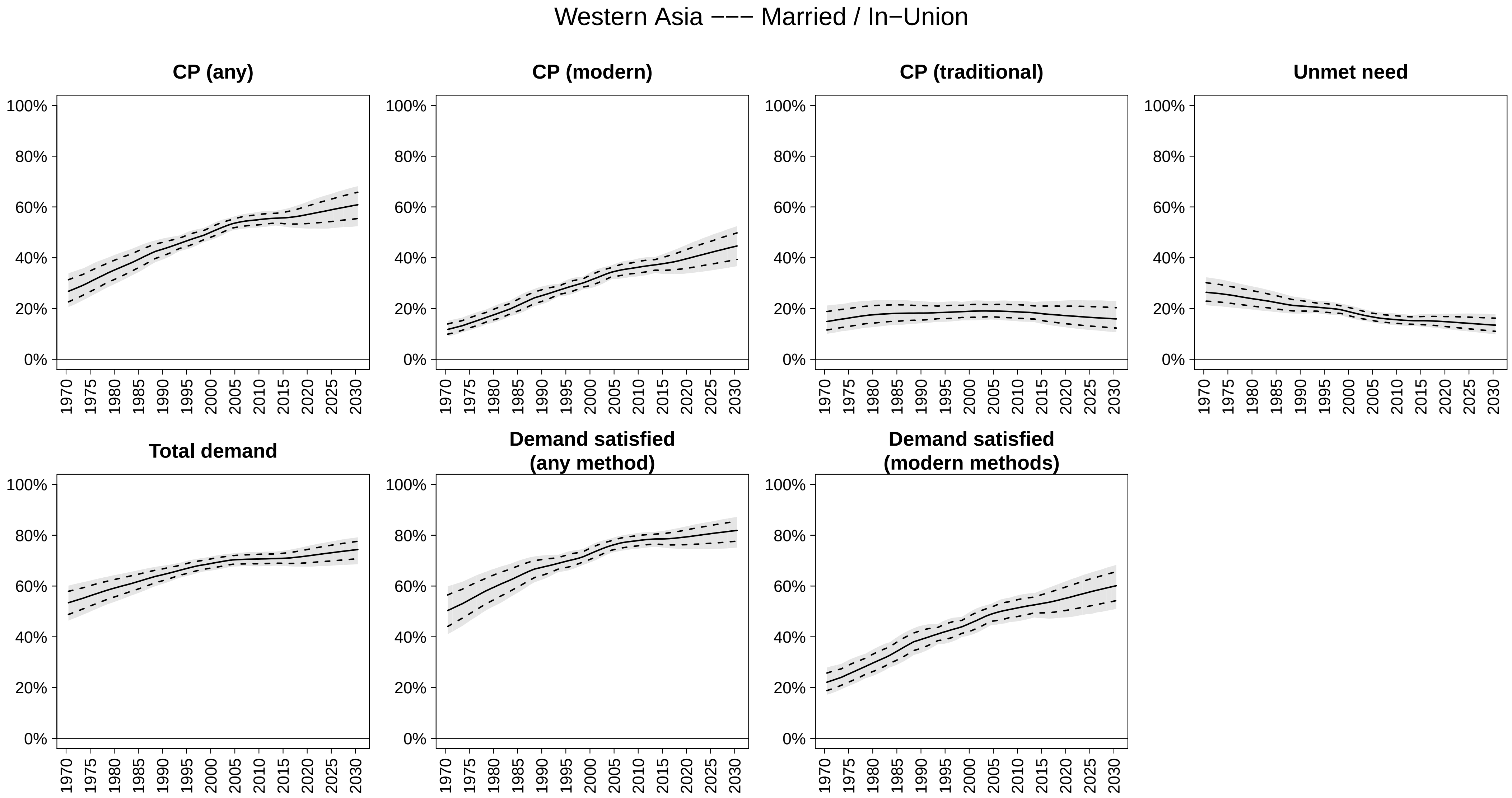
Western Asia −−− Married / In−Union
CP (any)
CP (modern)
CP (traditional)
Unmet need
Total demand
Demand satisfied
(any method)
Demand satisfied
(modern methods)
0%
20%
40%
60%
80%
100%
1970
1975
1980
1985
1990
1995
2000
2005
2010
2015
2020
2025
2030

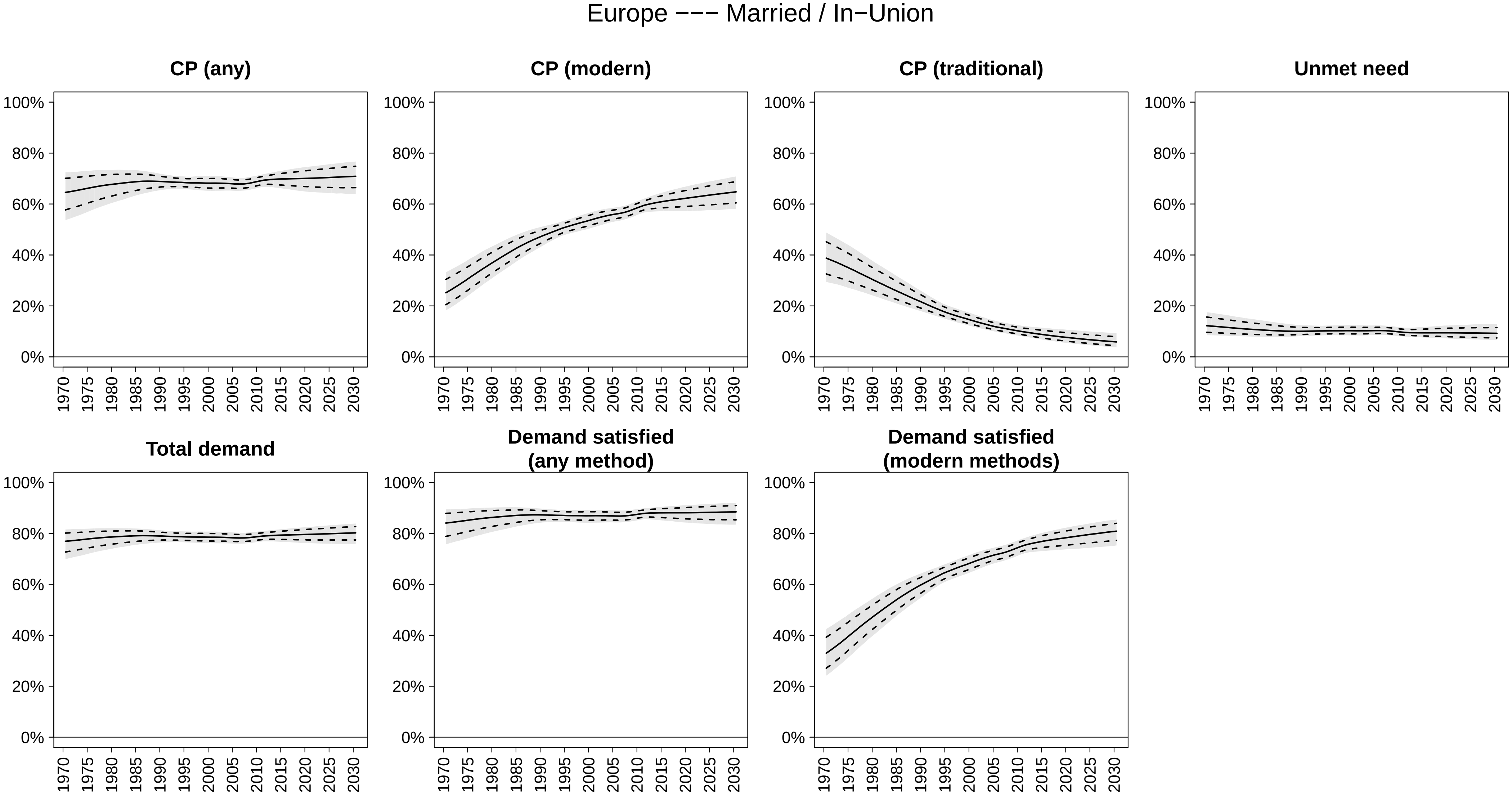
Europe −−− Married / In−Union
CP (any)
CP (modern)
CP (traditional)
Unmet need
Total demand
Demand satisfied (any method)
Demand satisfied (modern methods)
0%
20%
40%
60%
80%
100%
1970
1975
1980
1985
1990
1995
2000
2005
2010
2015
2020
2025
2030

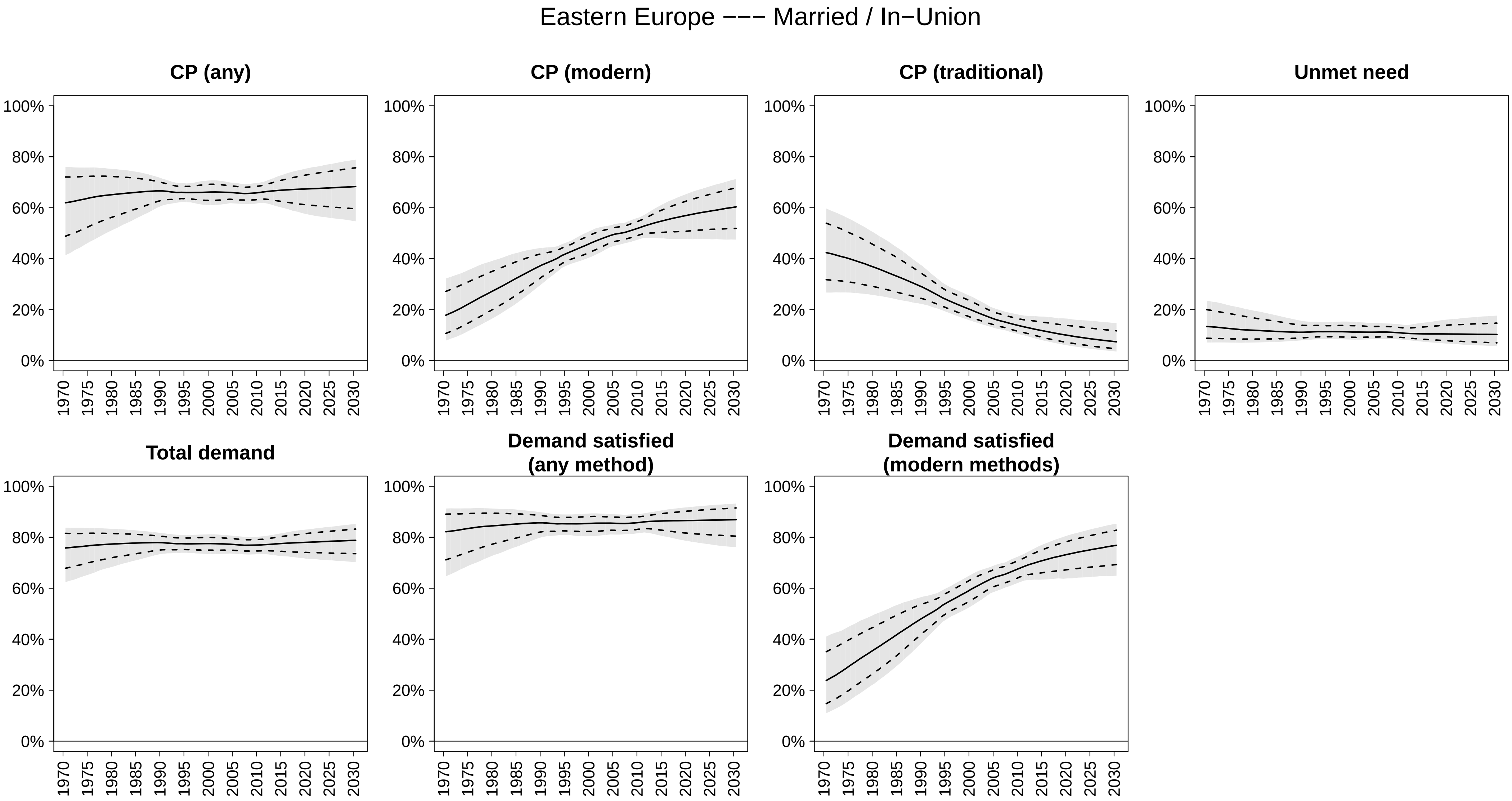
Eastern Europe −−− Married / In−Union
CP (any)
CP (modern)
CP (traditional)
Unmet need
Total demand
Demand satisfied (any method)
Demand satisfied (modern methods)
0%
20%
40%
60%
80%
100%
1970
1975
1980
1985
1990
1995
2000
2005
2010
2015
2020
2025
2030

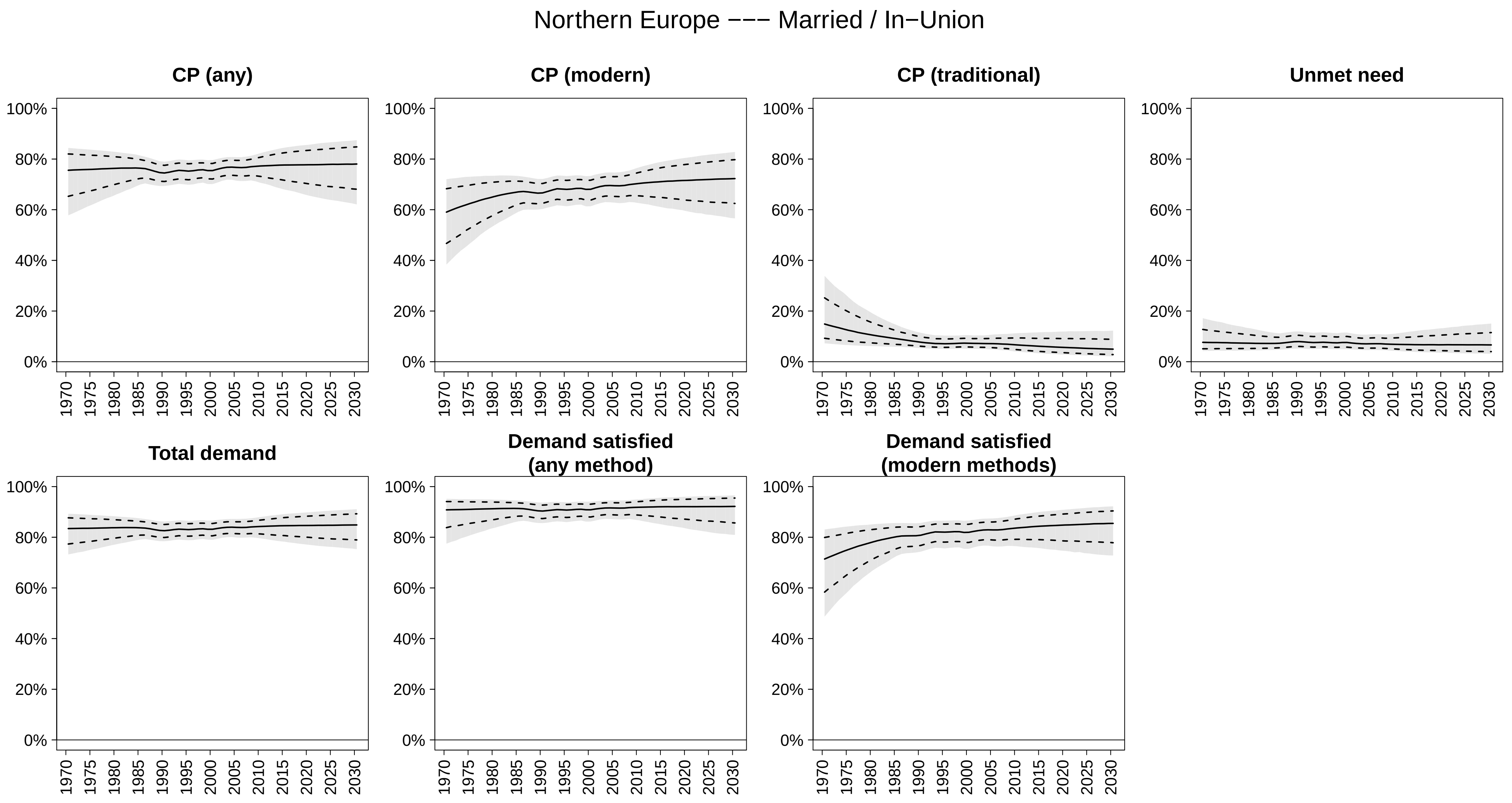

Northern Europe --- Married / In−Union
CP (any)
CP (modern)
CP (traditional)
Unmet need
Total demand
Demand satisfied (any method)
Demand satisfied (modern methods)
0%
20%
40%
60%
80%
100%
1970
1975
1980
1985
1990
1995
2000
2005
2010
2015
2020
2025
2030

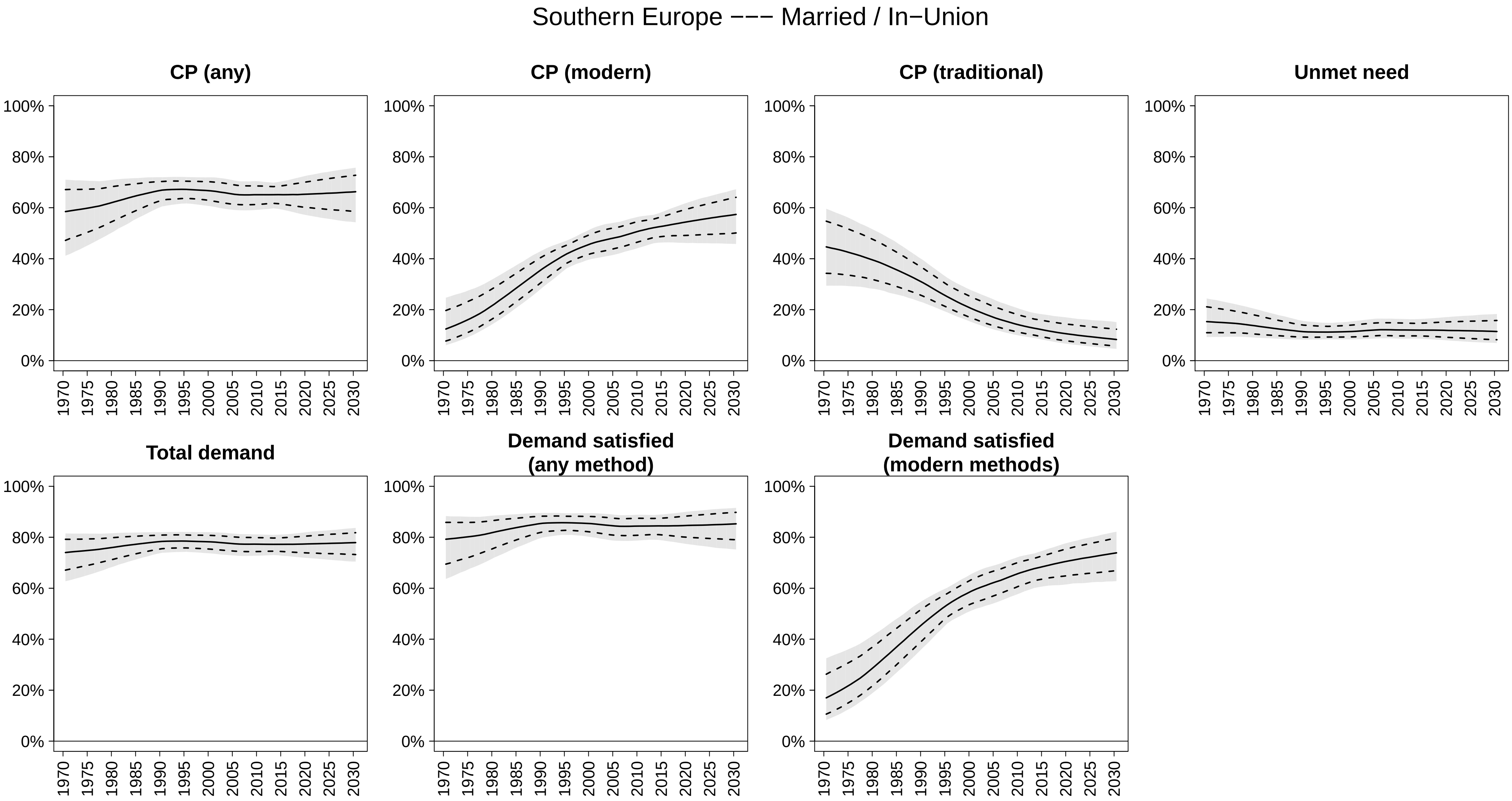
Southern Europe −−− Married / In−Union
CP (any)
CP (modern)
CP (traditional)
Unmet need
Total demand
Demand satisfied (any method)
Demand satisfied (modern methods)
0%
20%
40%
60%
80%
100%
1970
1975
1980
1985
1990
1995
2000
2005
2010
2015
2020
2025
2030

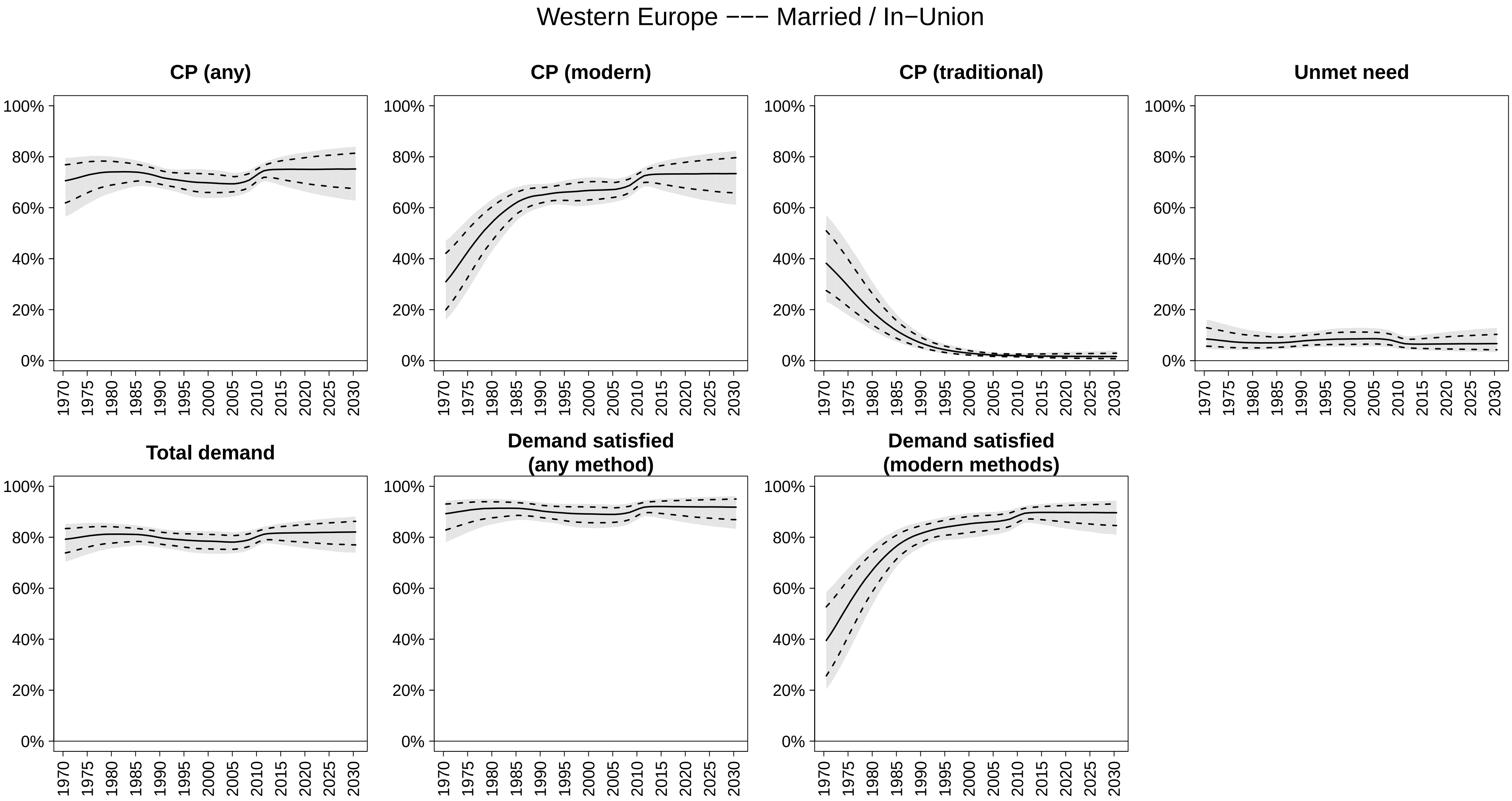
Western Europe −−− Married / In−Union
CP (any)
CP (modern)
CP (traditional)
Unmet need
Total demand
Demand satisfied (any method)
Demand satisfied (modern methods)
0%
20%
40%
60%
80%
100%
1970
1975
1980
1985
1990
1995
2000
2005
2010
2015
2020
2025
2030

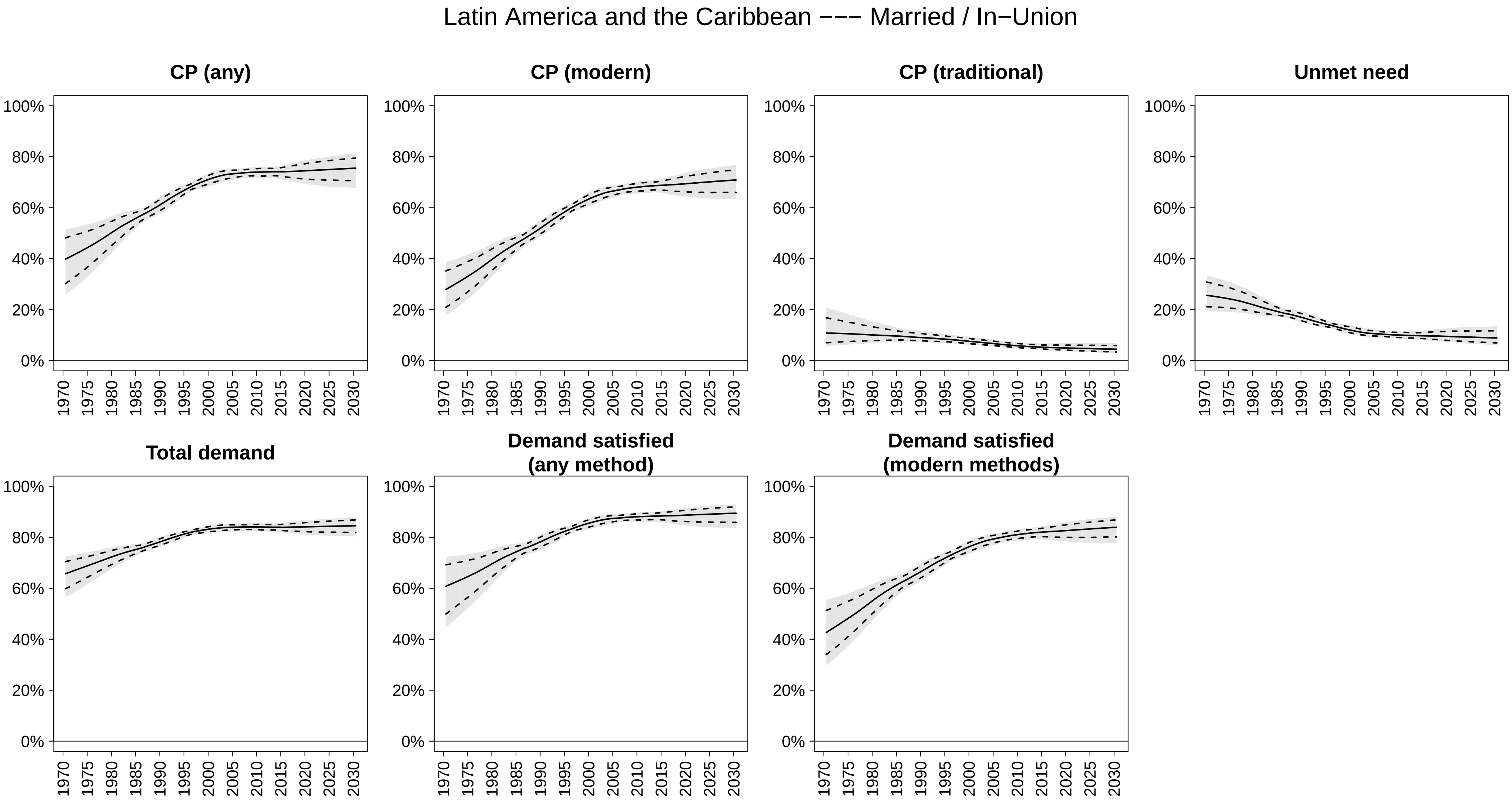
Latin America and the Caribbean −−− Married / In−Union
CP (any)
CP (modern)
CP (traditional)
Unmet need
Total demand
Demand satisfied (any method)
Demand satisfied (modern methods)
0%
20%
40%
60%
80%
100%
1970
1975
1980
1985
1990
1995
2000
2005
2010
2015
2020
2025
2030

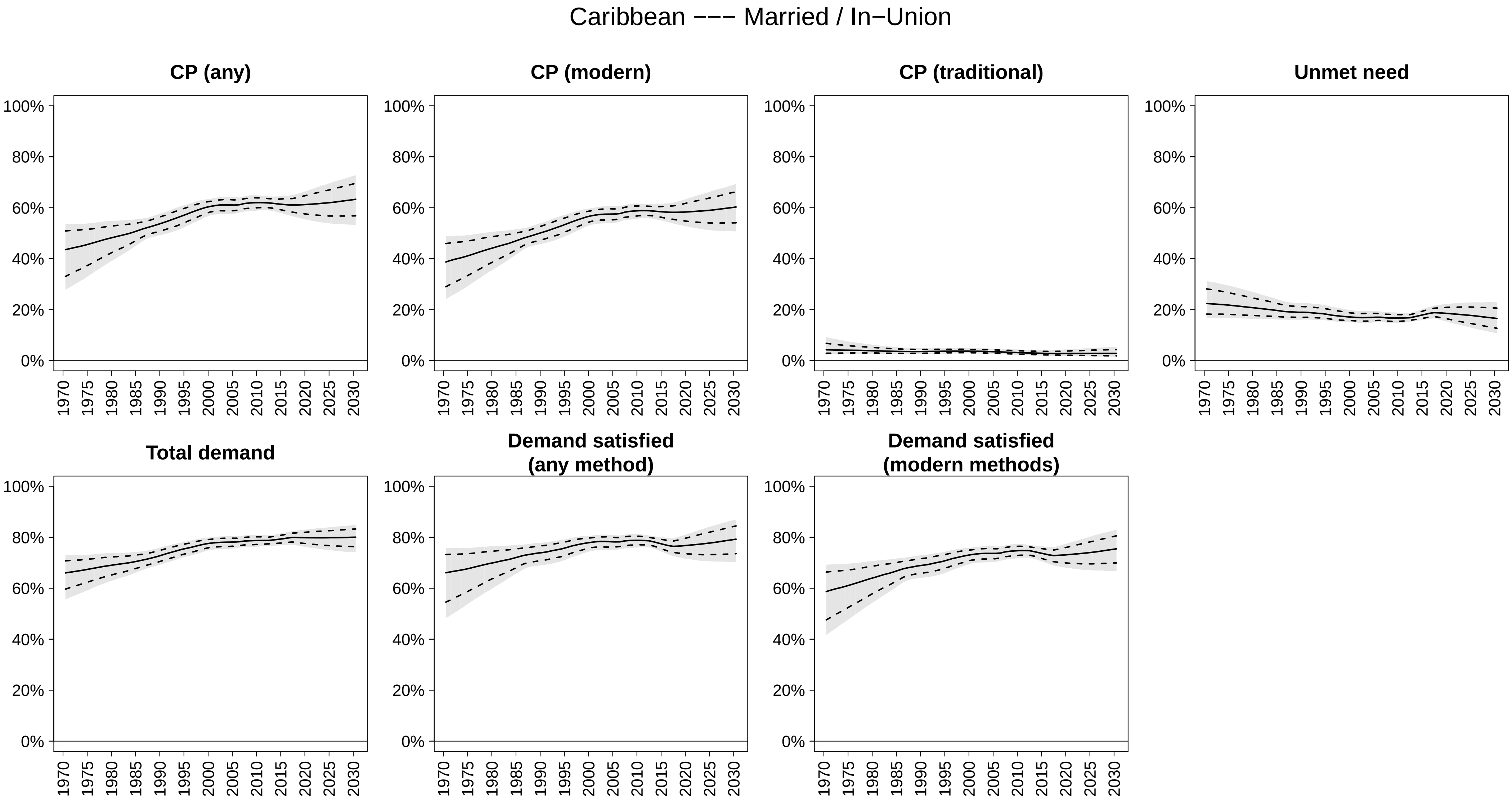
Caribbean −−− Married / In−Union
CP (any)
CP (modern)
CP (traditional)
Unmet need
Total demand
Demand satisfied (any method)
Demand satisfied (modern methods)
0%
20%
40%
60%
80%
100%
1970
1975
1980
1985
1990
1995
2000
2005
2010
2015
2020
2025
2030

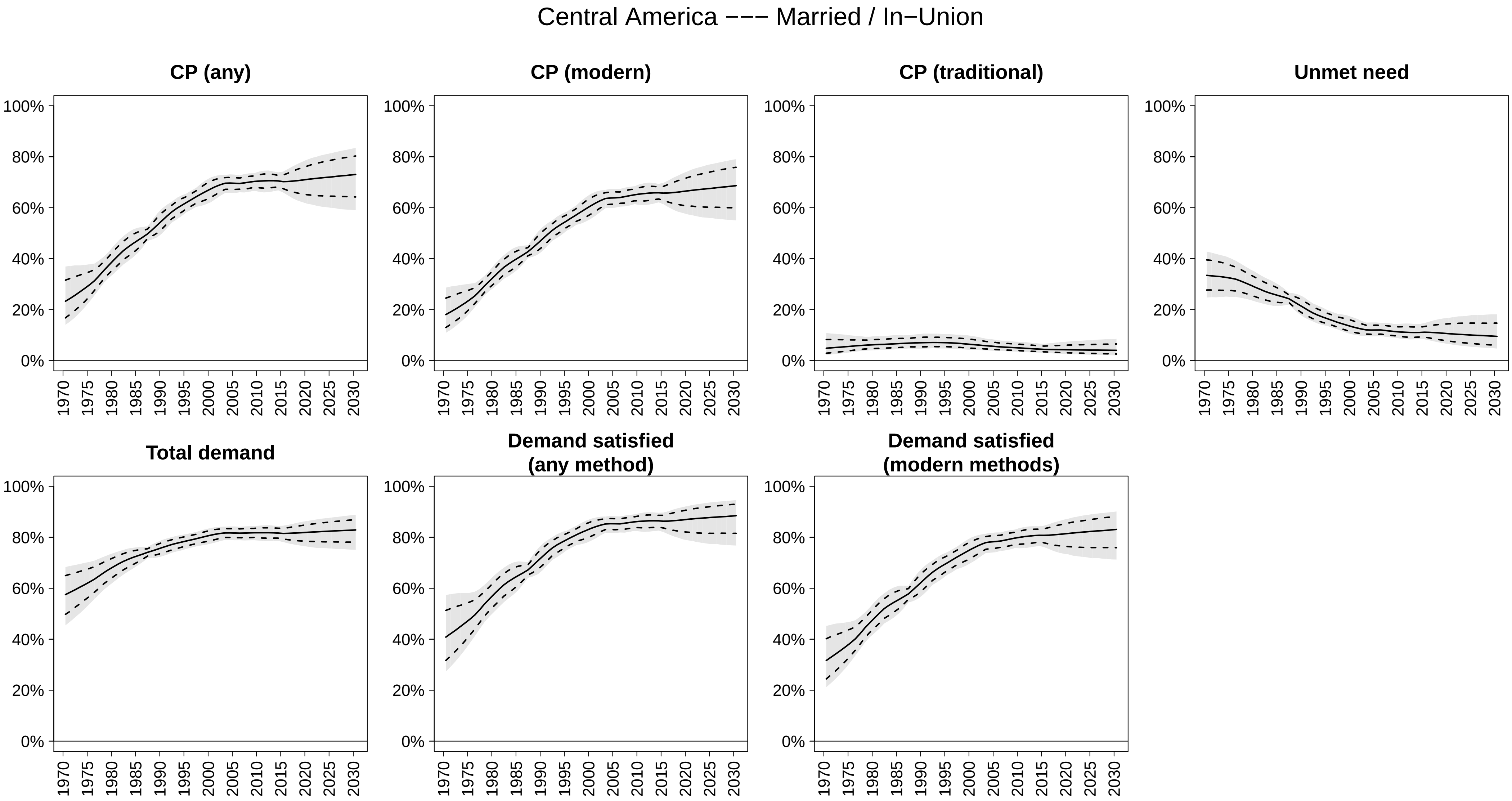
Central America −−− Married / In−Union
CP (any)
CP (modern)
CP (traditional)
Unmet need
Total demand
Demand satisfied (any method)
Demand satisfied (modern methods)
0%
20%
40%
60%
80%
100%
1970
1975
1980
1985
1990
1995
2000
2005
2010
2015
2020
2025
2030

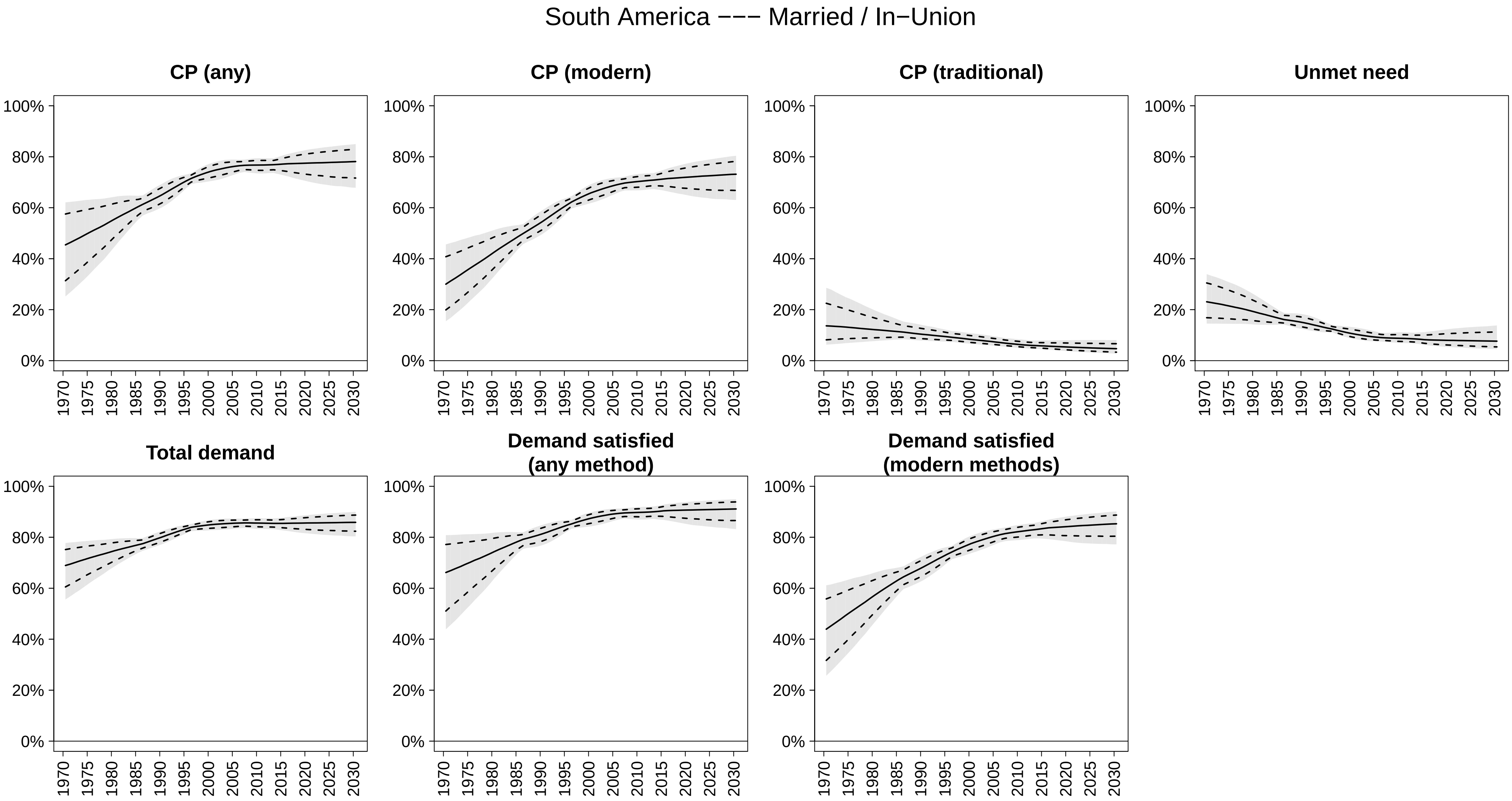
South America −−− Married / In−Union
CP (any)
CP (modern)
CP (traditional)
Unmet need
Total demand
Demand satisfied (any method)
Demand satisfied (modern methods)
0%
20%
40%
60%
80%
100%
1970
1975
1980
1985
1990
1995
2000
2005
2010
2015
2020
2025
2030

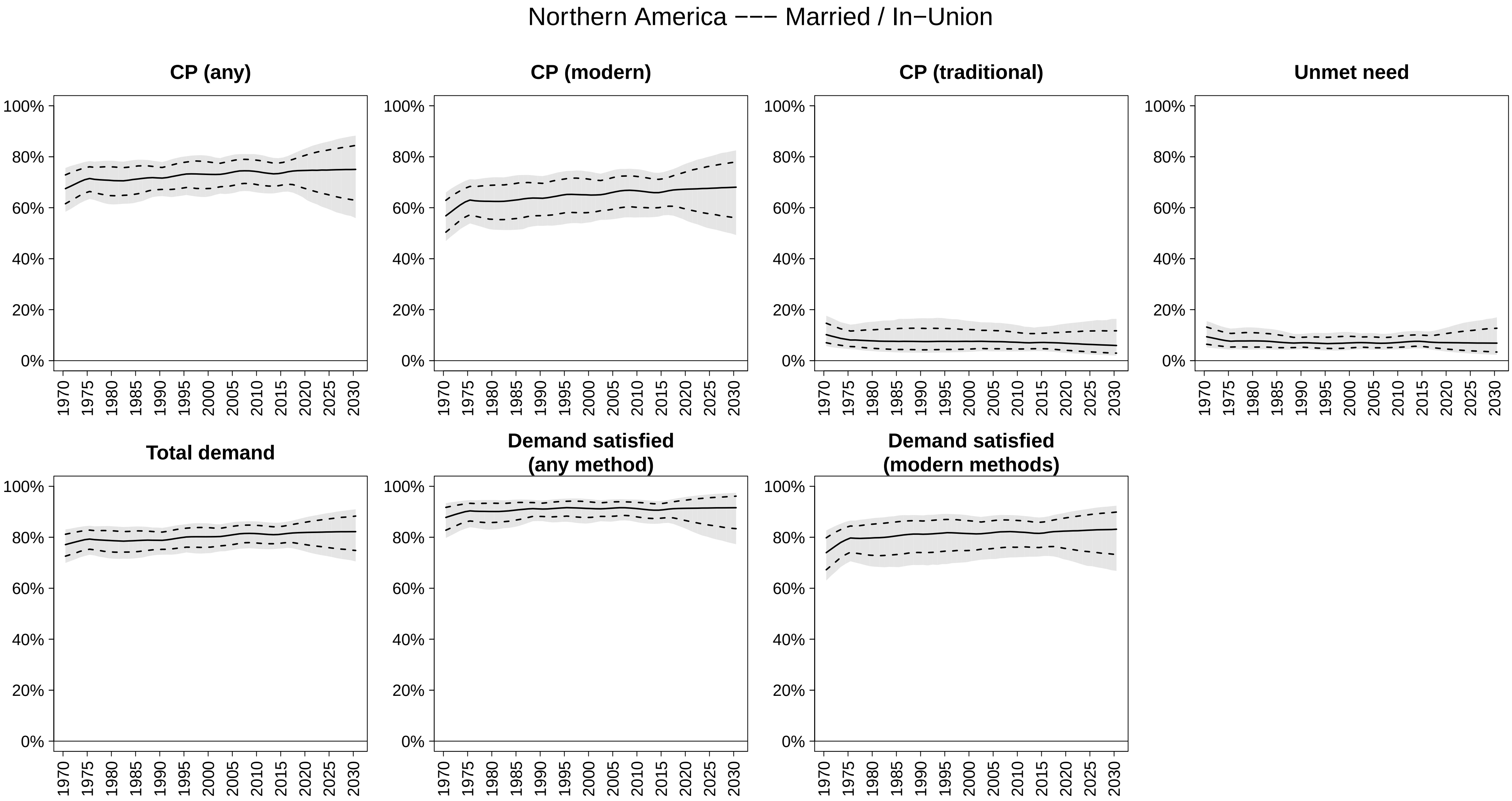
Northern America −−− Married / In−Union
CP (any)
CP (modern)
CP (traditional)
Unmet need
Total demand
Demand satisfied (any method)
Demand satisfied (modern methods)
100%
80%
60%
40%
20%
0%
1970
1975
1980
1985
1990
1995
2000
2005
2010
2015
2020
2025
2030

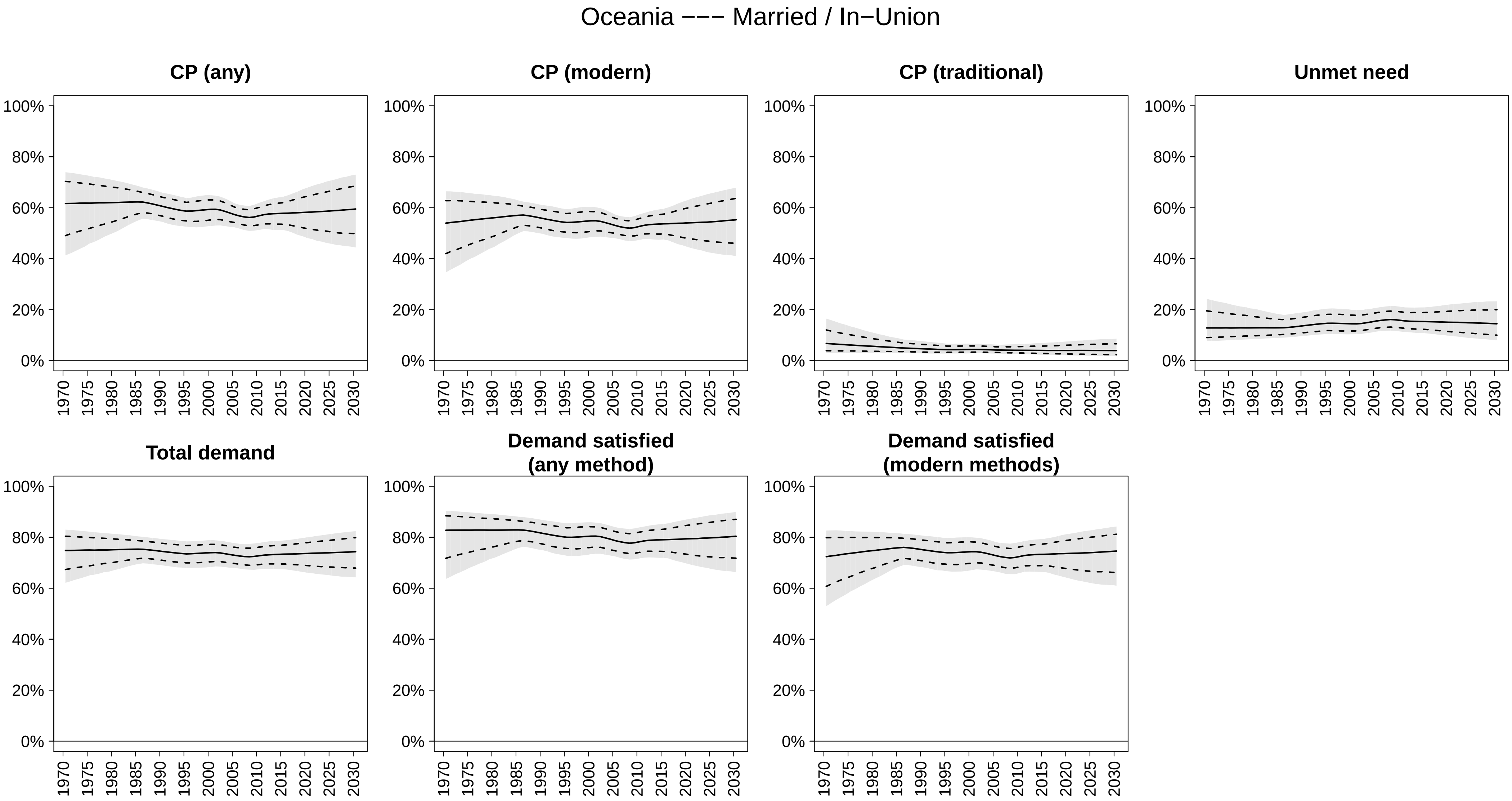
Oceania --- Married / In−Union
CP (any)
CP (modern)
CP (traditional)
Unmet need
Total demand
Demand satisfied (any method)
Demand satisfied (modern methods)
100%
80%
60%
40%
20%
0%
1970 1975 1980 1985 1990 1995 2000 2005 2010 2015 2020 2025 2030

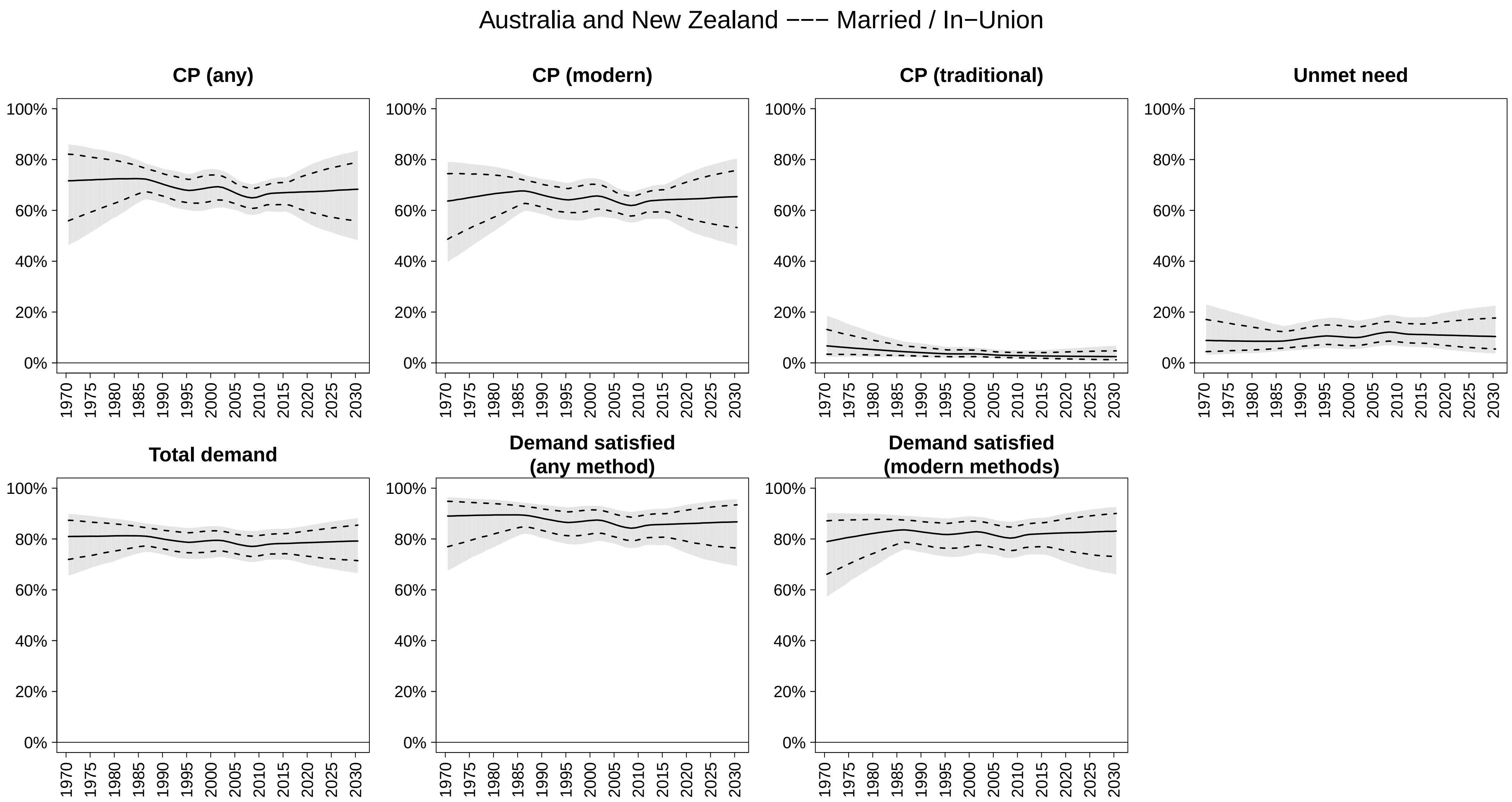
Australia and New Zealand −−− Married / In−Union
CP (any)
CP (modern)
CP (traditional)
Unmet need
Total demand
Demand satisfied (any method)
Demand satisfied (modern methods)
0%
20%
40%
60%
80%
100%
1970
1975
1980
1985
1990
1995
2000
2005
2010
2015
2020
2025
2030

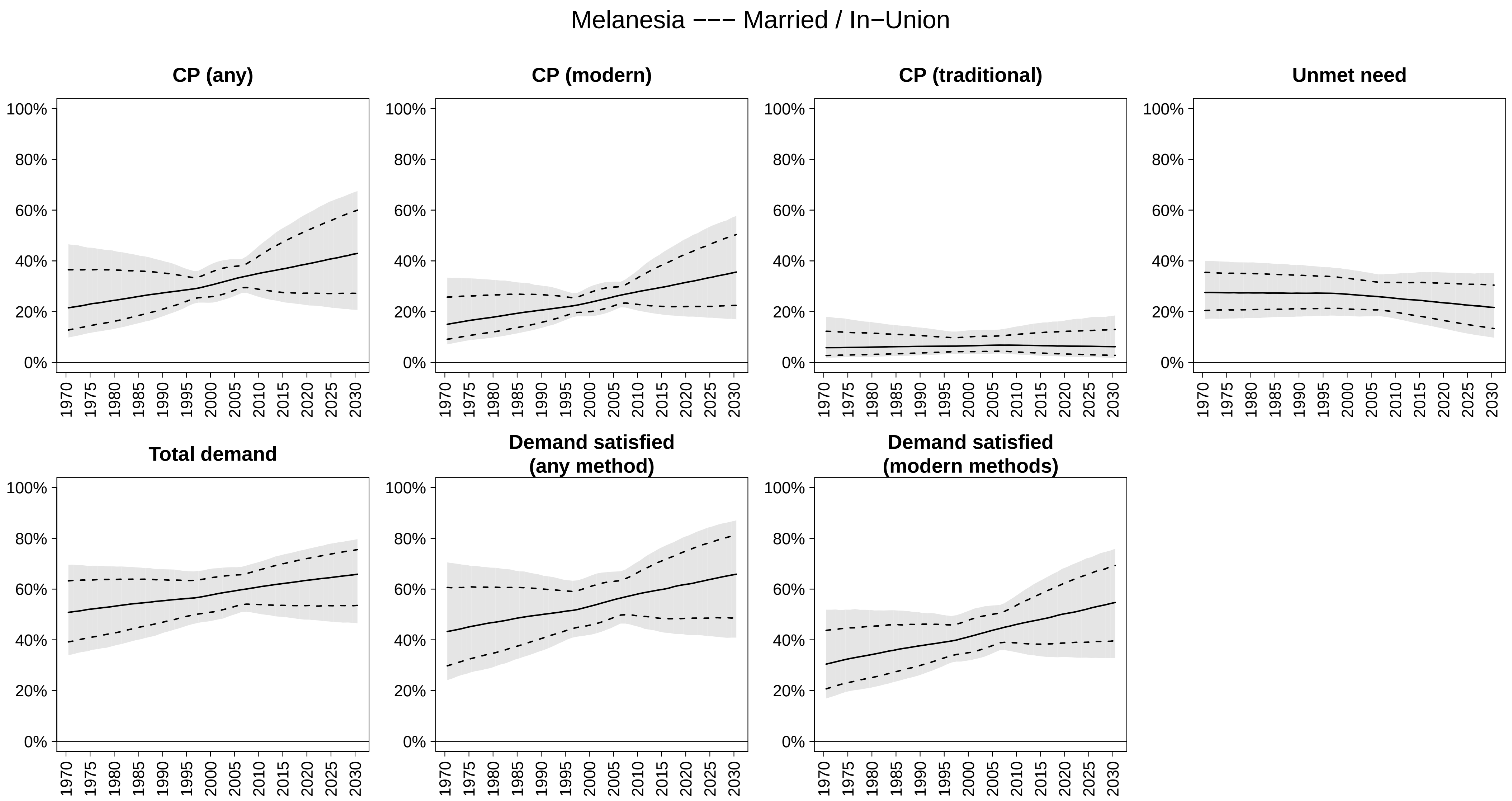
Melanesia −−− Married / In−Union
CP (any)
CP (modern)
CP (traditional)
Unmet need
Total demand
Demand satisfied (any method)
Demand satisfied (modern methods)
0%
20%
40%
60%
80%
100%
1970
1975
1980
1985
1990
1995
2000
2005
2010
2015
2020
2025
2030

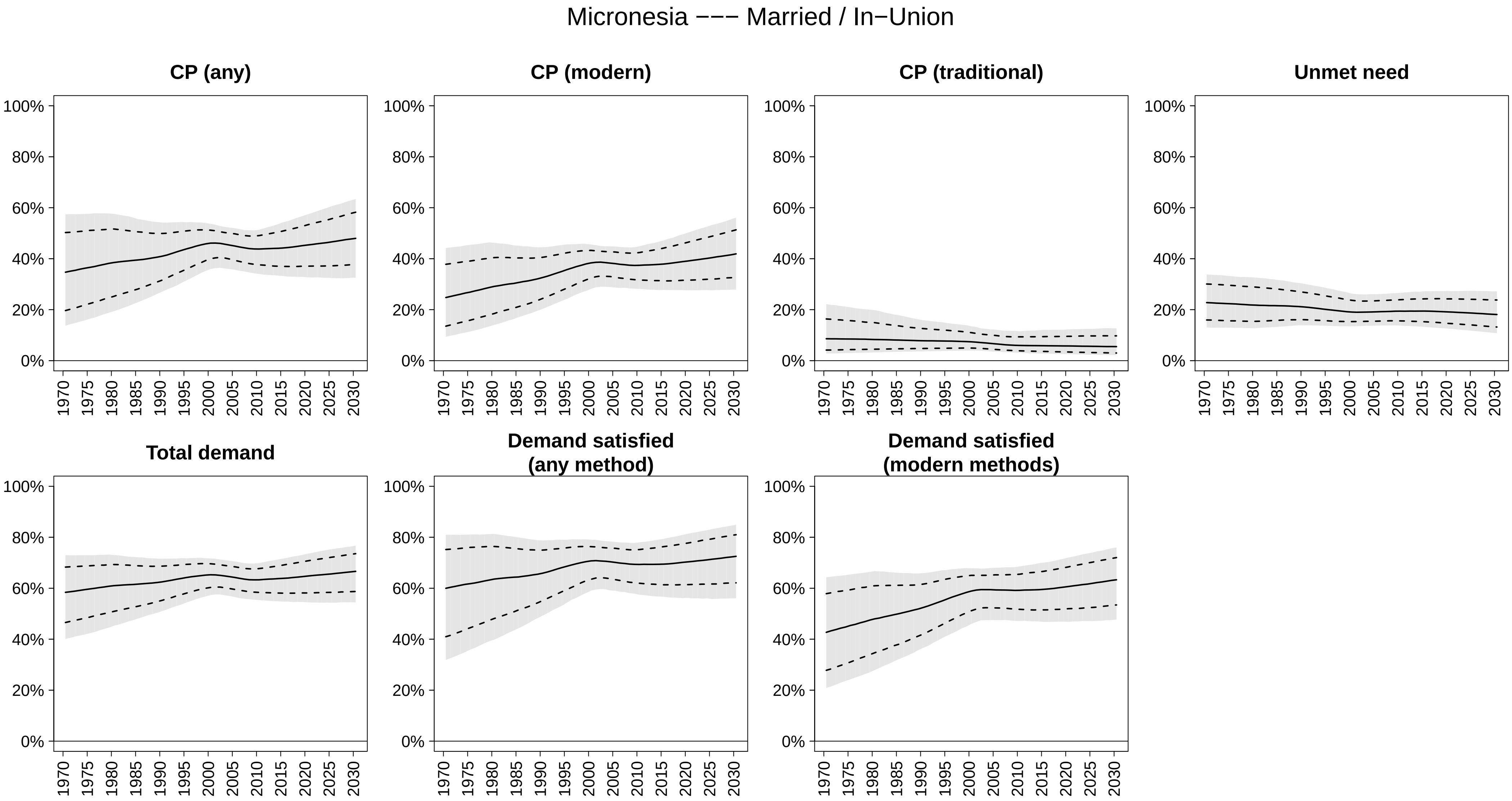
Micronesia −−− Married / In−Union
CP (any)
CP (modern)
CP (traditional)
Unmet need
Total demand
Demand satisfied (any method)
Demand satisfied (modern methods)
0%
20%
40%
60%
80%
100%
1970
1975
1980
1985
1990
1995
2000
2005
2010
2015
2020
2025
2030

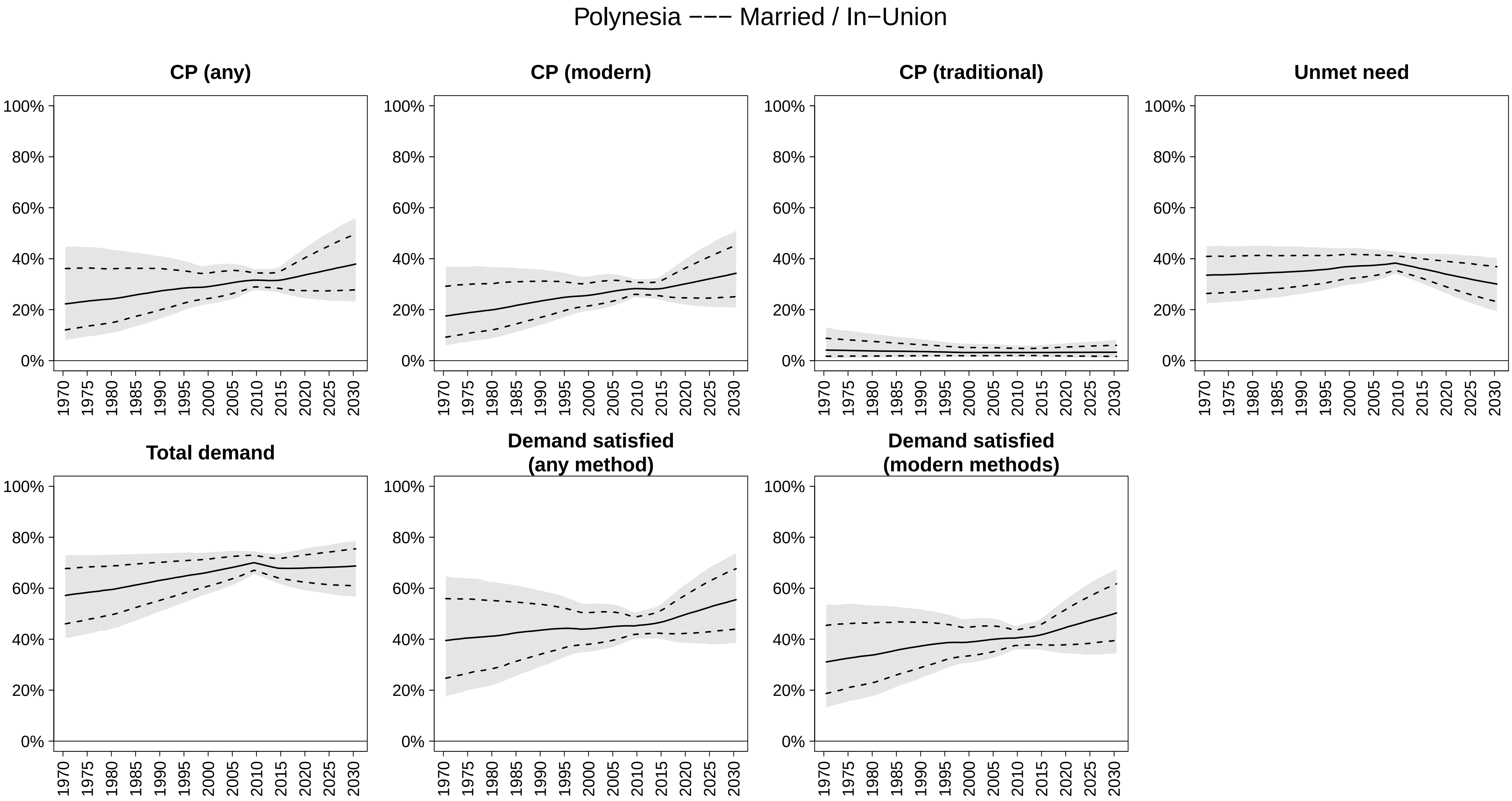

Polynesia −−− Married / In−Union
CP (any)
CP (modern)
CP (traditional)
Unmet need
Total demand
Demand satisfied (any method)
Demand satisfied (modern methods)
0%
20%
40%
60%
80%
100%
1970
1975
1980
1985
1990
1995
2000
2005
2010
2015
2020
2025
2030

3 Women of reproductive age irrespective of marital status

3.1 Countries and areas

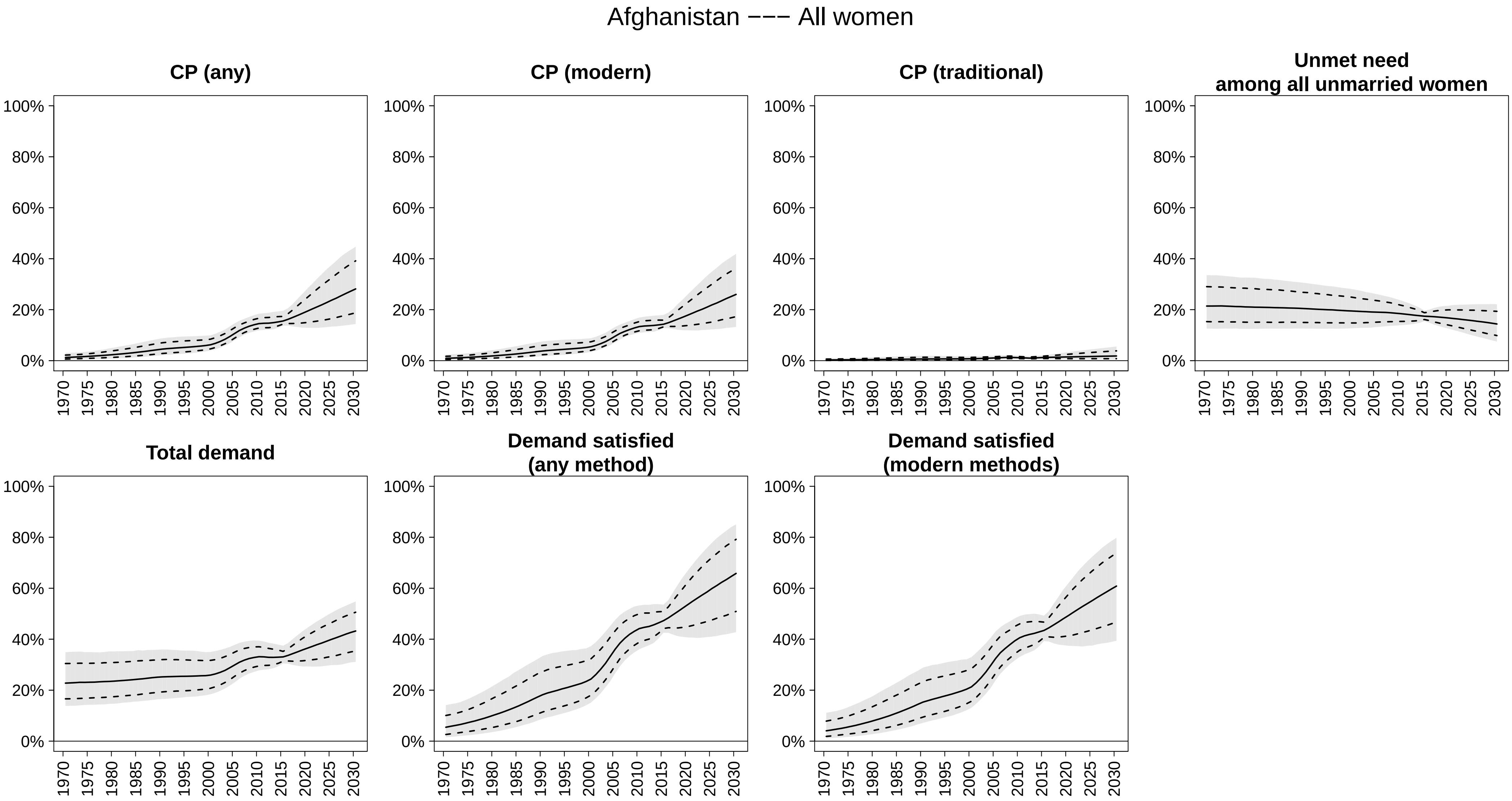
Afghanistan −−− All women
CP (any)
CP (modern)
CP (traditional)
Unmet need among all unmarried women
Total demand
Demand satisfied (any method)
Demand satisfied (modern methods)
0%
20%
40%
60%
80%
100%
1970
1975
1980
1985
1990
1995
2000
2005
2010
2015
2020
2025
2030

Albania −−− All women

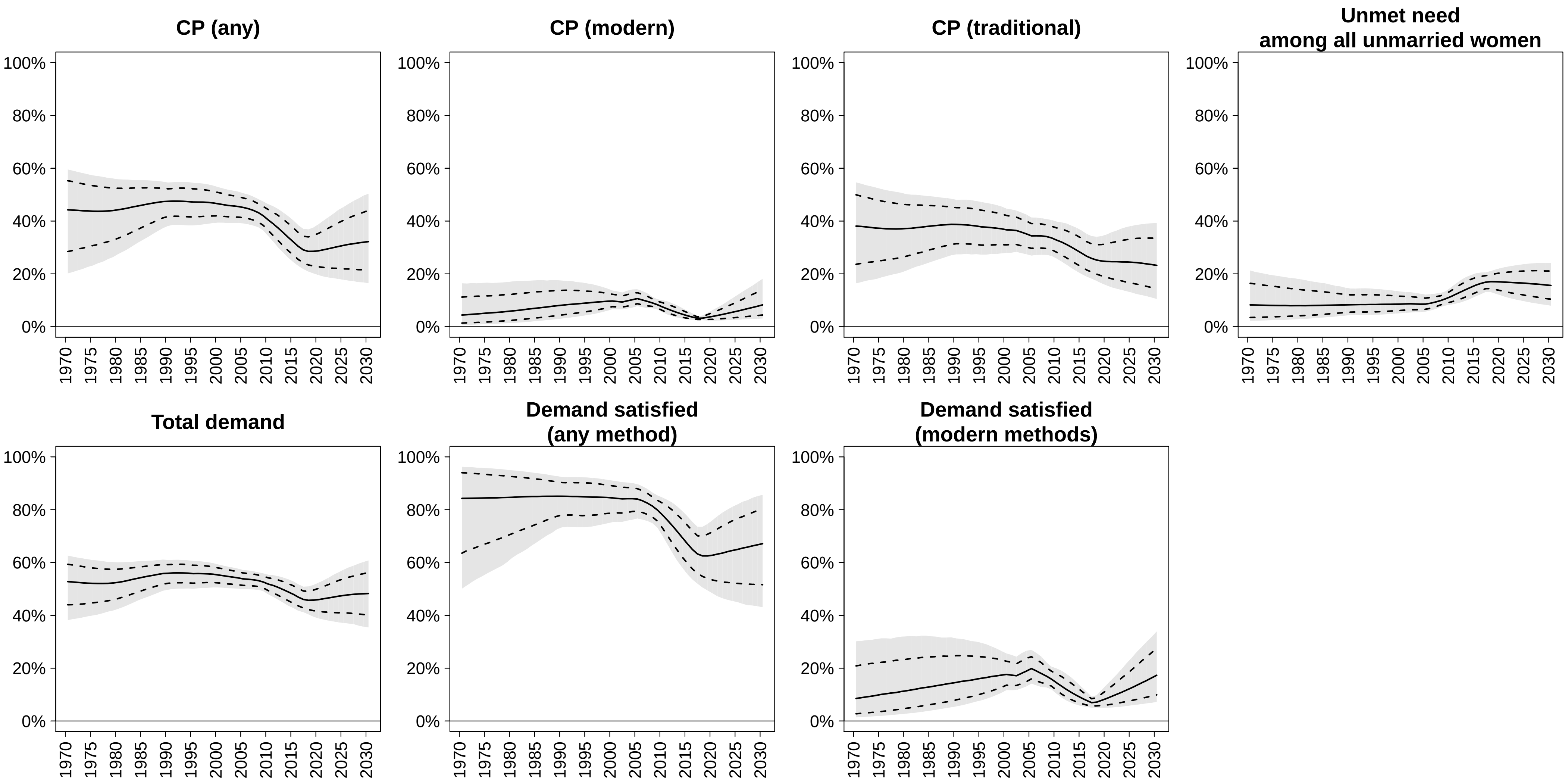

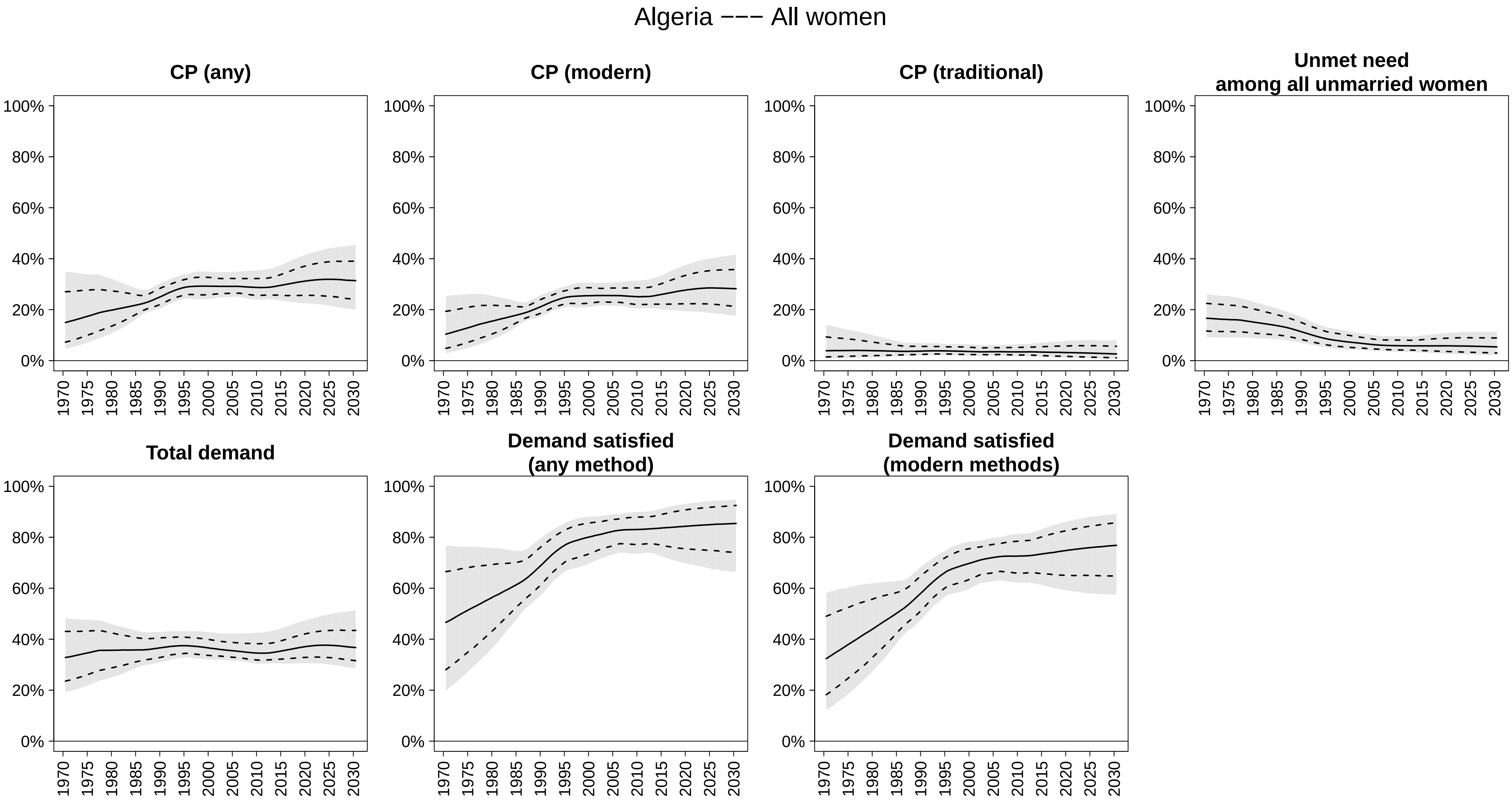
Algeria −−− All women
CP (any)
CP (modern)
CP (traditional)
Unmet need
among all unmarried women
Total demand
Demand satisfied
(any method)
Demand satisfied
(modern methods)
100%
80%
60%
40%
20%
0%
1970
1975
1980
1985
1990
1995
2000
2005
2010
2015
2020
2025
2030

Angola −−− All women

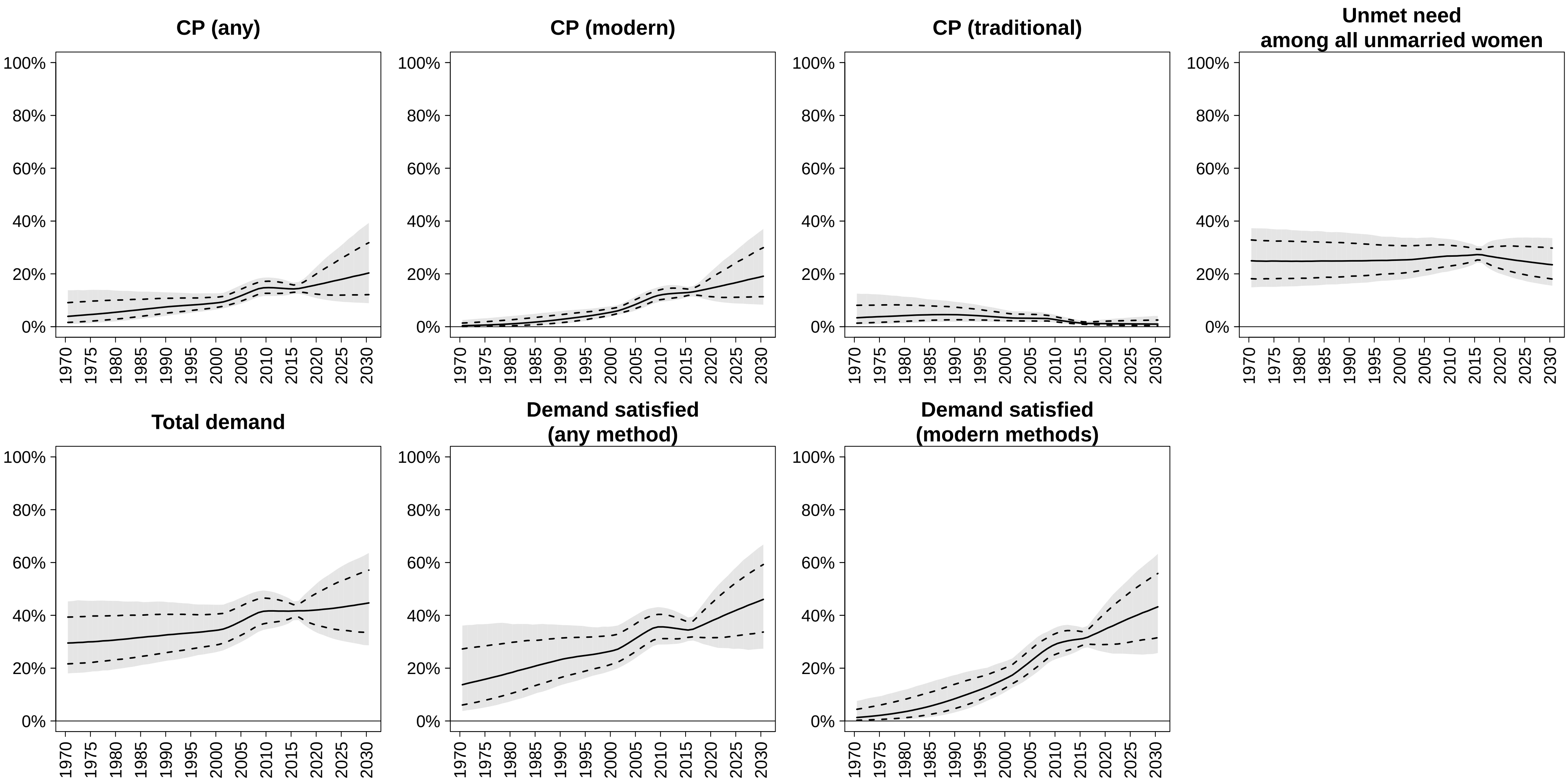

Antigua and Barbuda −−− All women

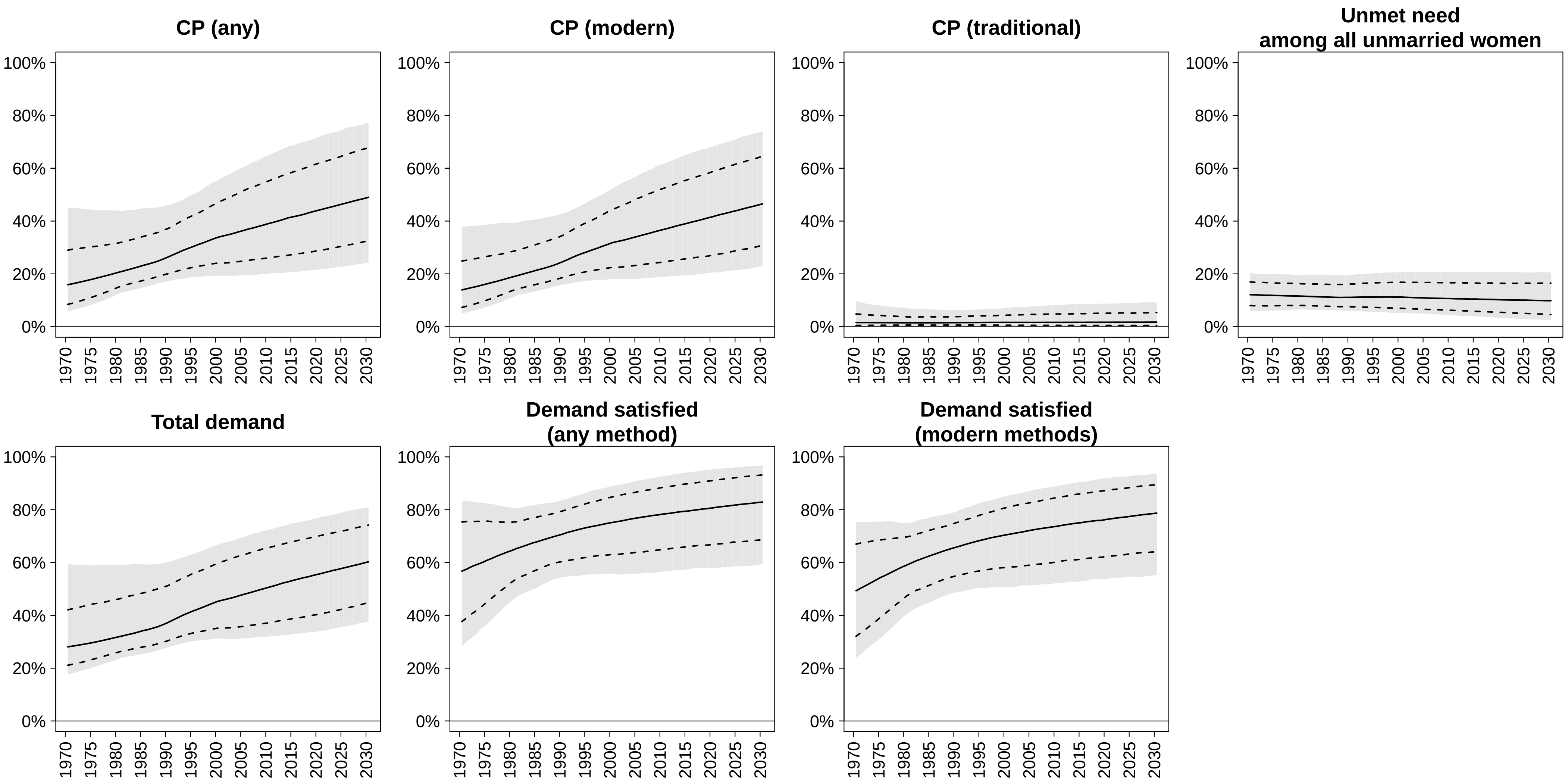

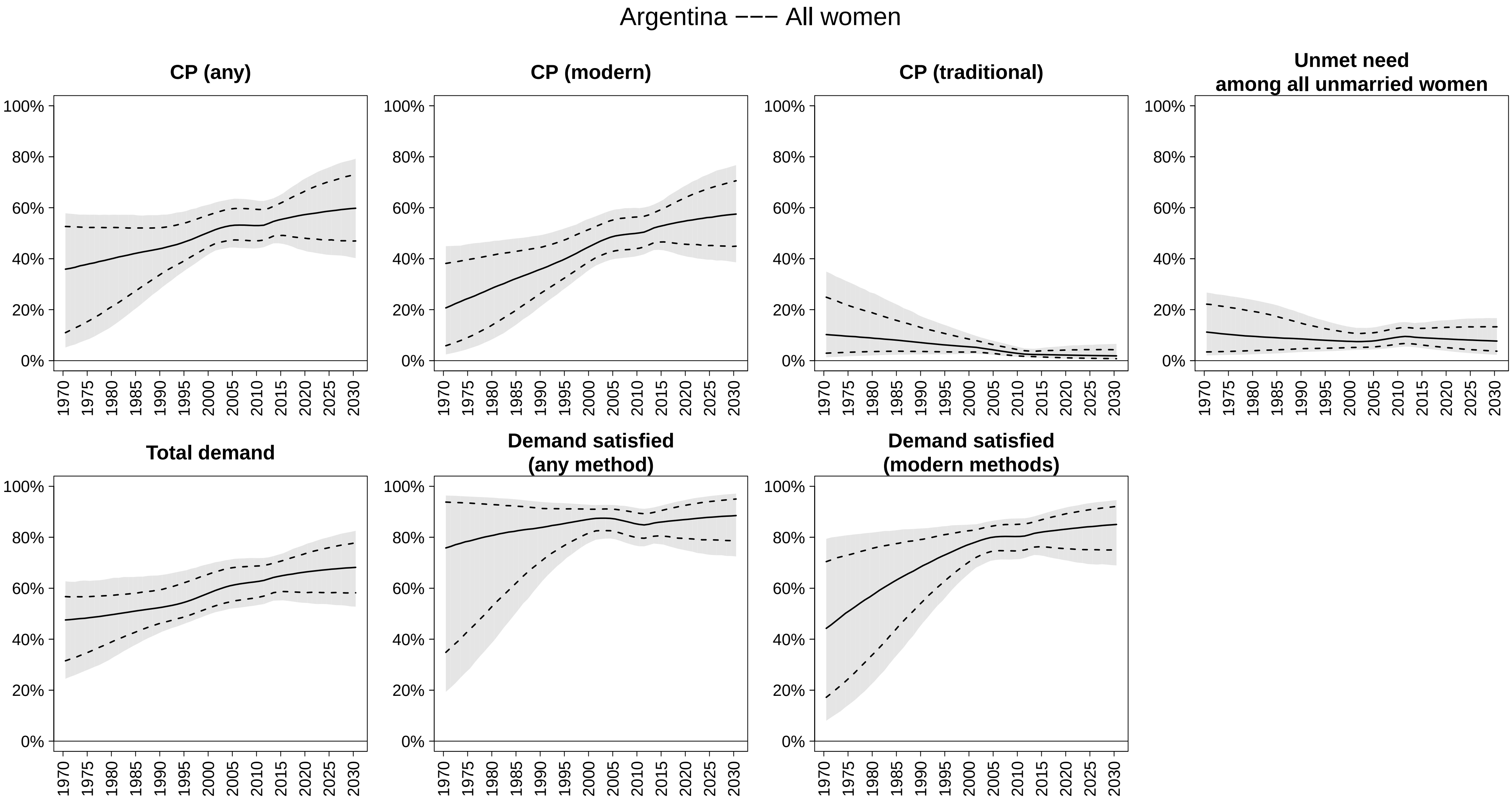
Argentina −−− All women
CP (any)
CP (modern)
CP (traditional)
Unmet need among all unmarried women
Total demand
Demand satisfied (any method)
Demand satisfied (modern methods)
0%
20%
40%
60%
80%
100%
1970
1975
1980
1985
1990
1995
2000
2005
2010
2015
2020
2025
2030

Armenia −−− All women

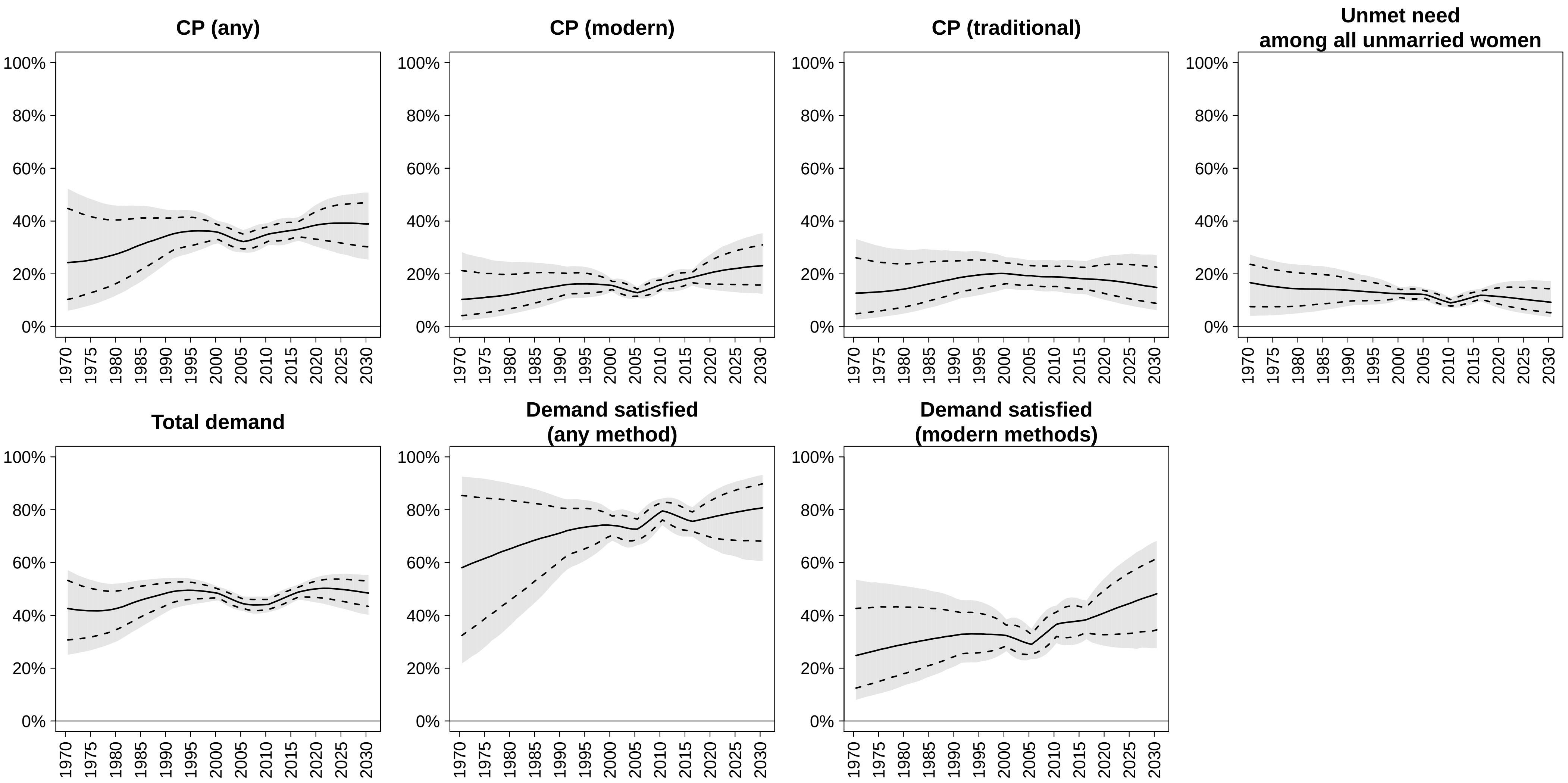

Australia −−− All women

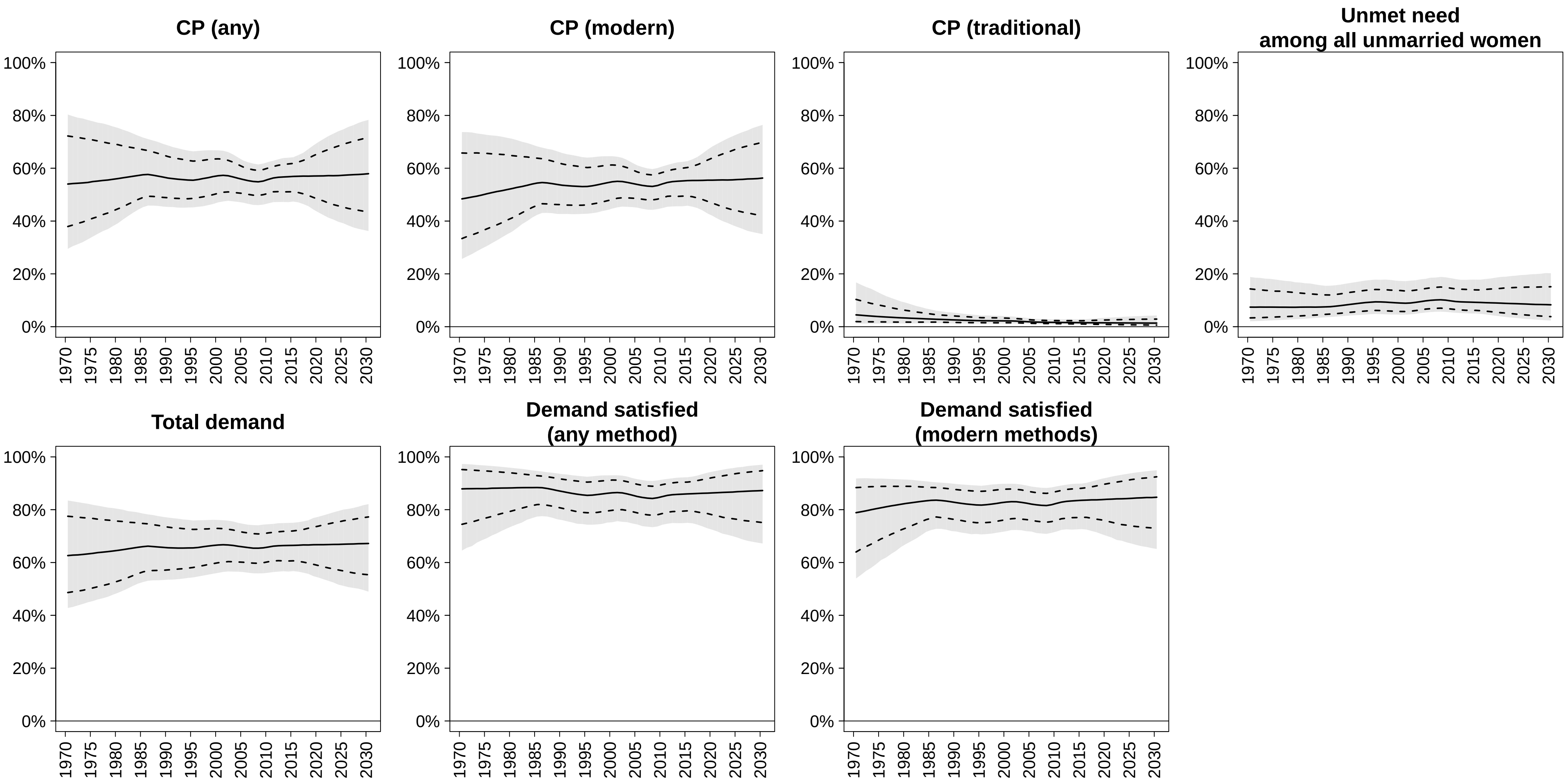

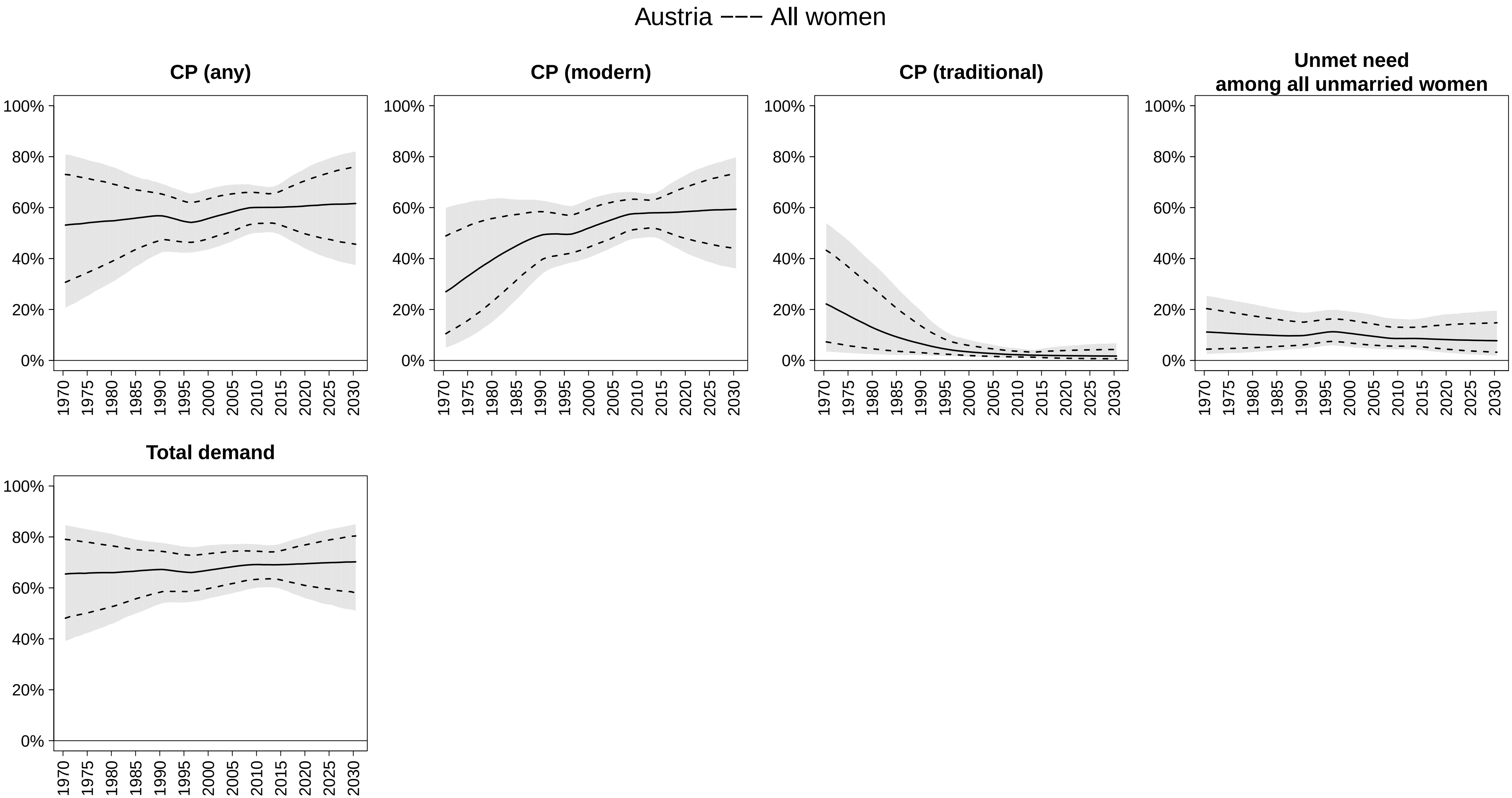
Austria −−− All women
CP (any)
CP (modern)
CP (traditional)
Unmet need
among all unmarried women
Total demand
100%
80%
60%
40%
20%
0%
1970
1975
1980
1985
1990
1995
2000
2005
2010
2015
2020
2025
2030

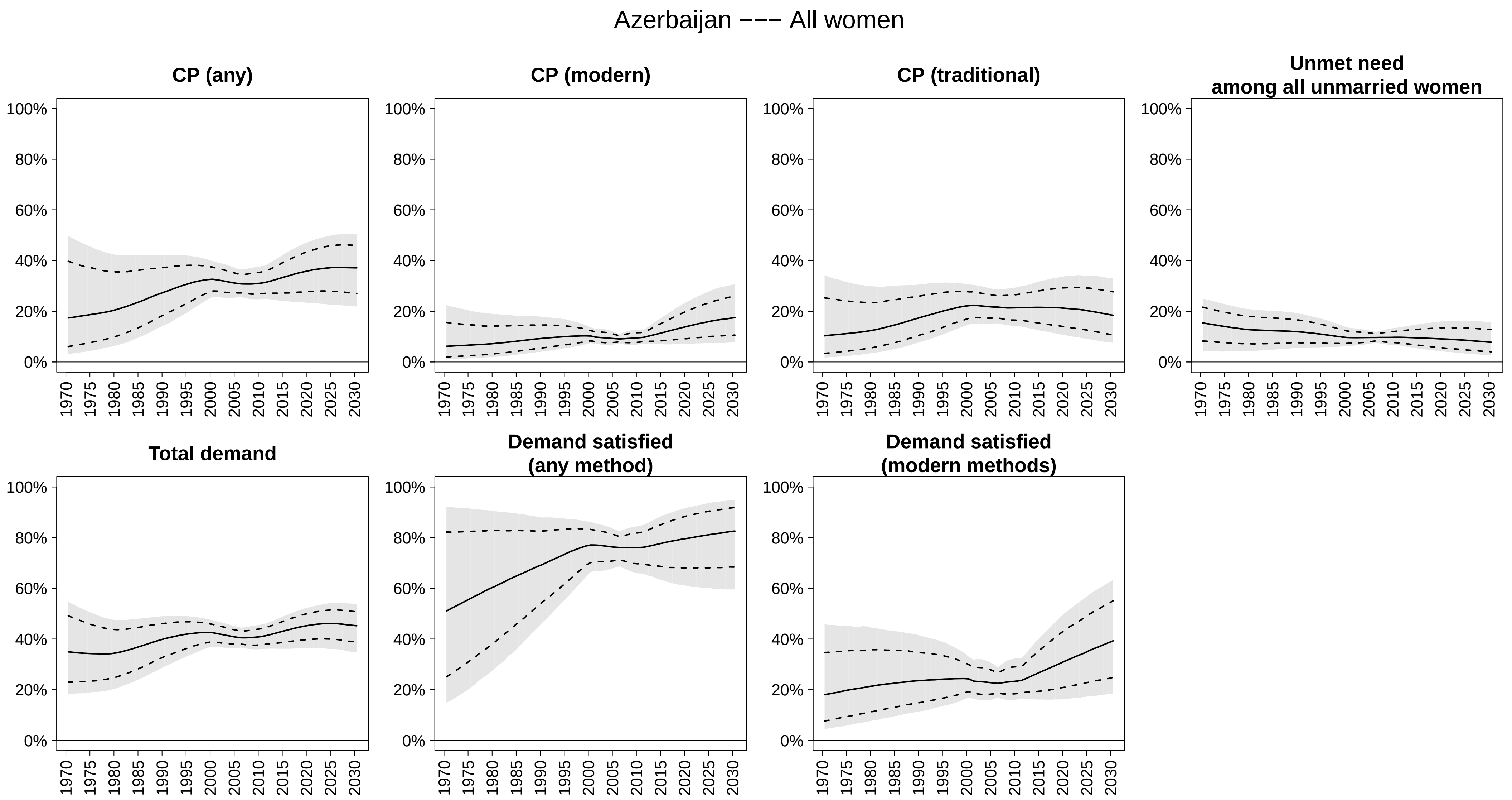
Azerbaijan −−− All women
CP (any)
CP (modern)
CP (traditional)
Unmet need
among all unmarried women
Total demand
Demand satisfied
(any method)
Demand satisfied
(modern methods)
100%
80%
60%
40%
20%
0%
1970
1975
1980
1985
1990
1995
2000
2005
2010
2015
2020
2025
2030

Bahamas ––– All women

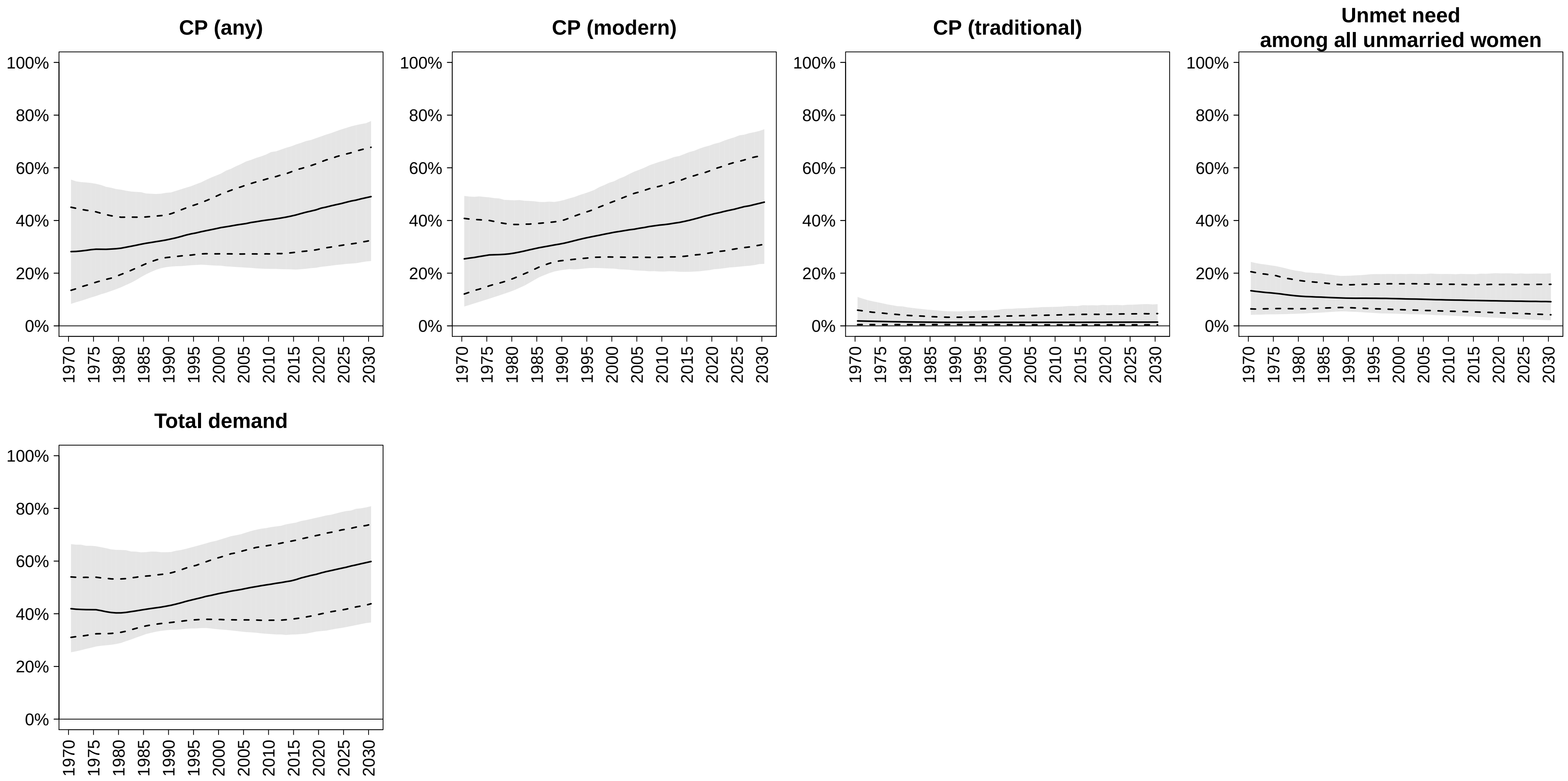

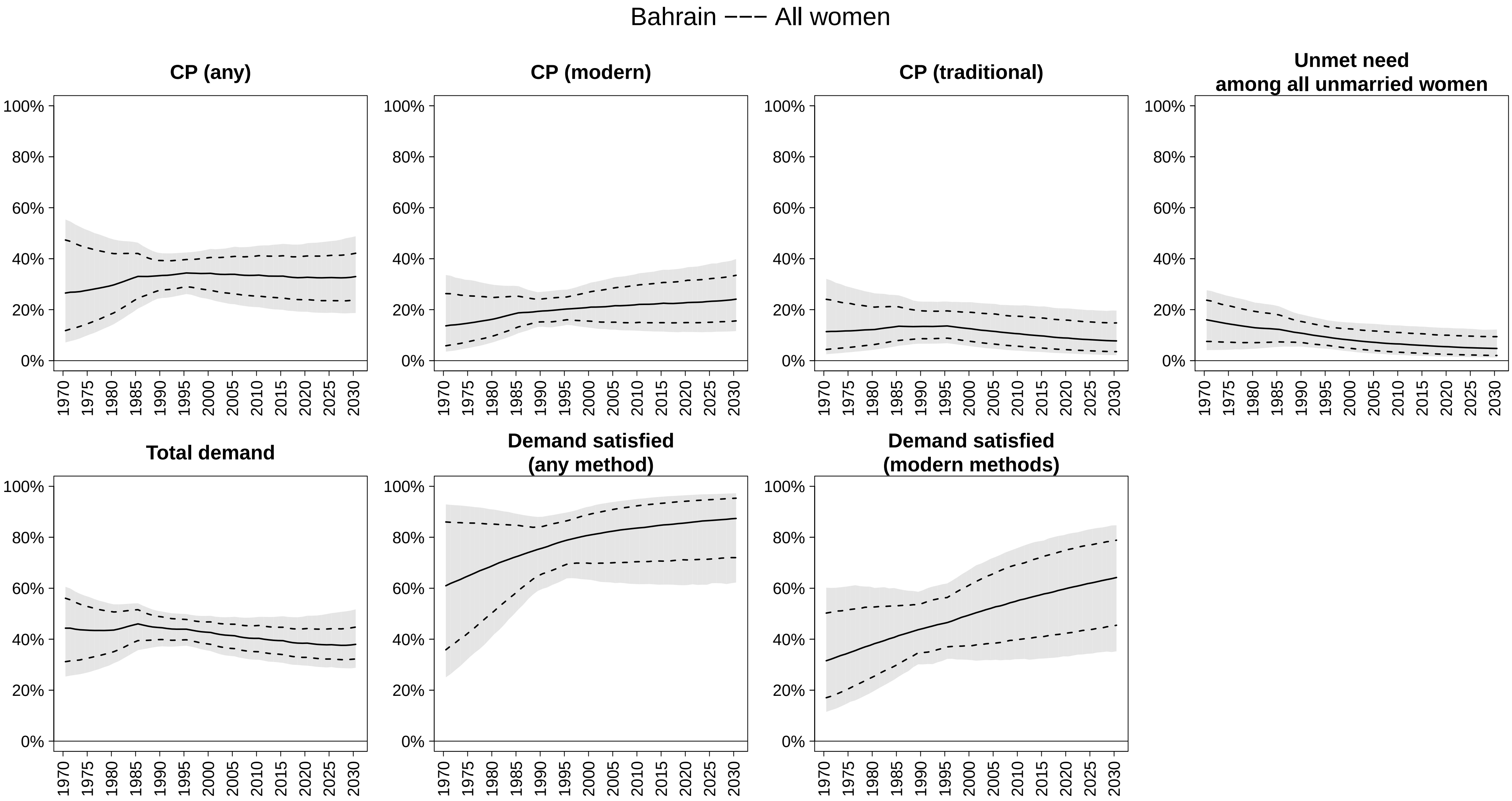
Bahrain −−− All women
CP (any)
CP (modern)
CP (traditional)
Unmet need
among all unmarried women
Total demand
Demand satisfied
(any method)
Demand satisfied
(modern methods)
0%
20%
40%
60%
80%
100%
1970
1975
1980
1985
1990
1995
2000
2005
2010
2015
2020
2025
2030

Bangladesh ––– All women

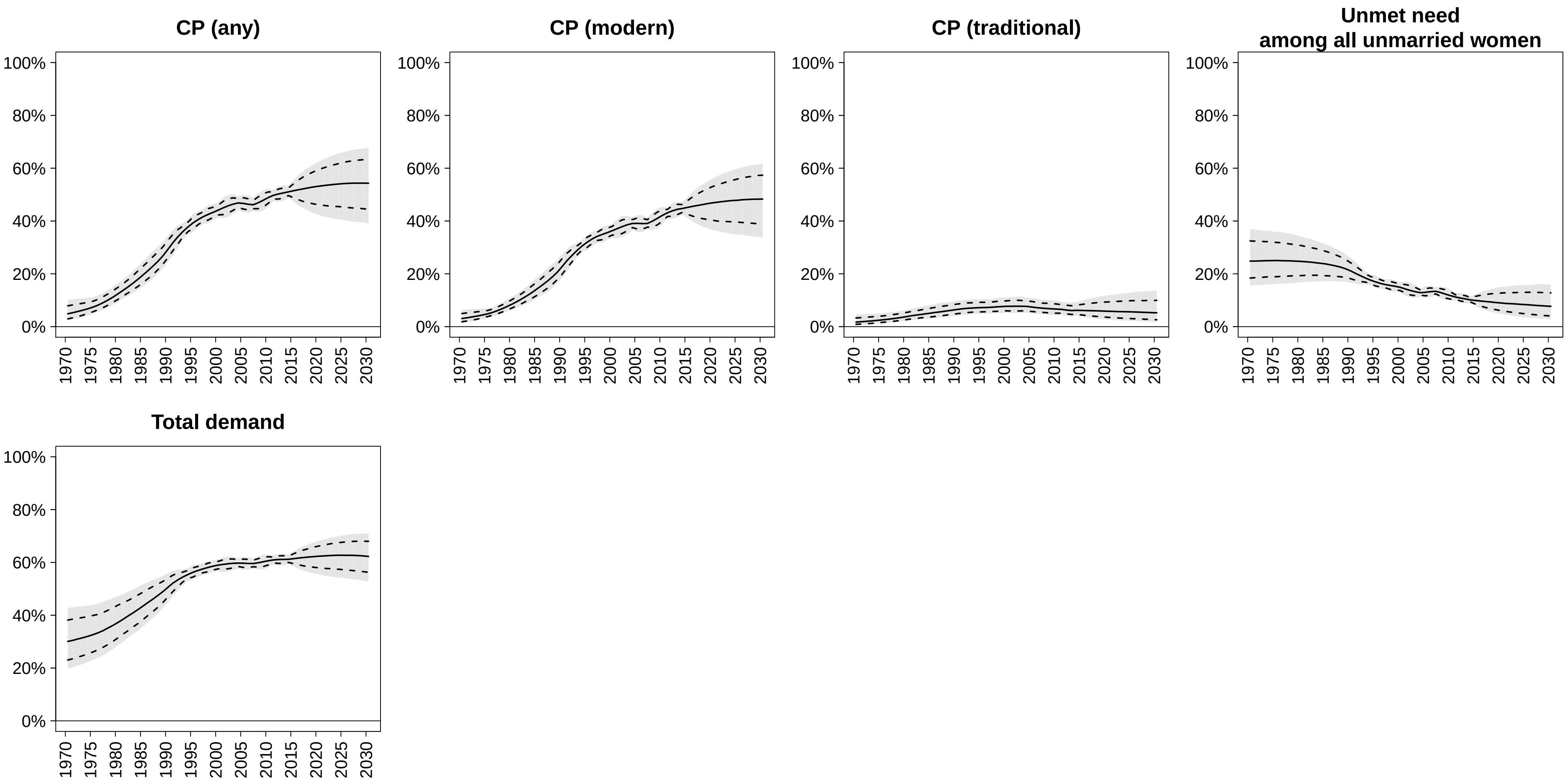

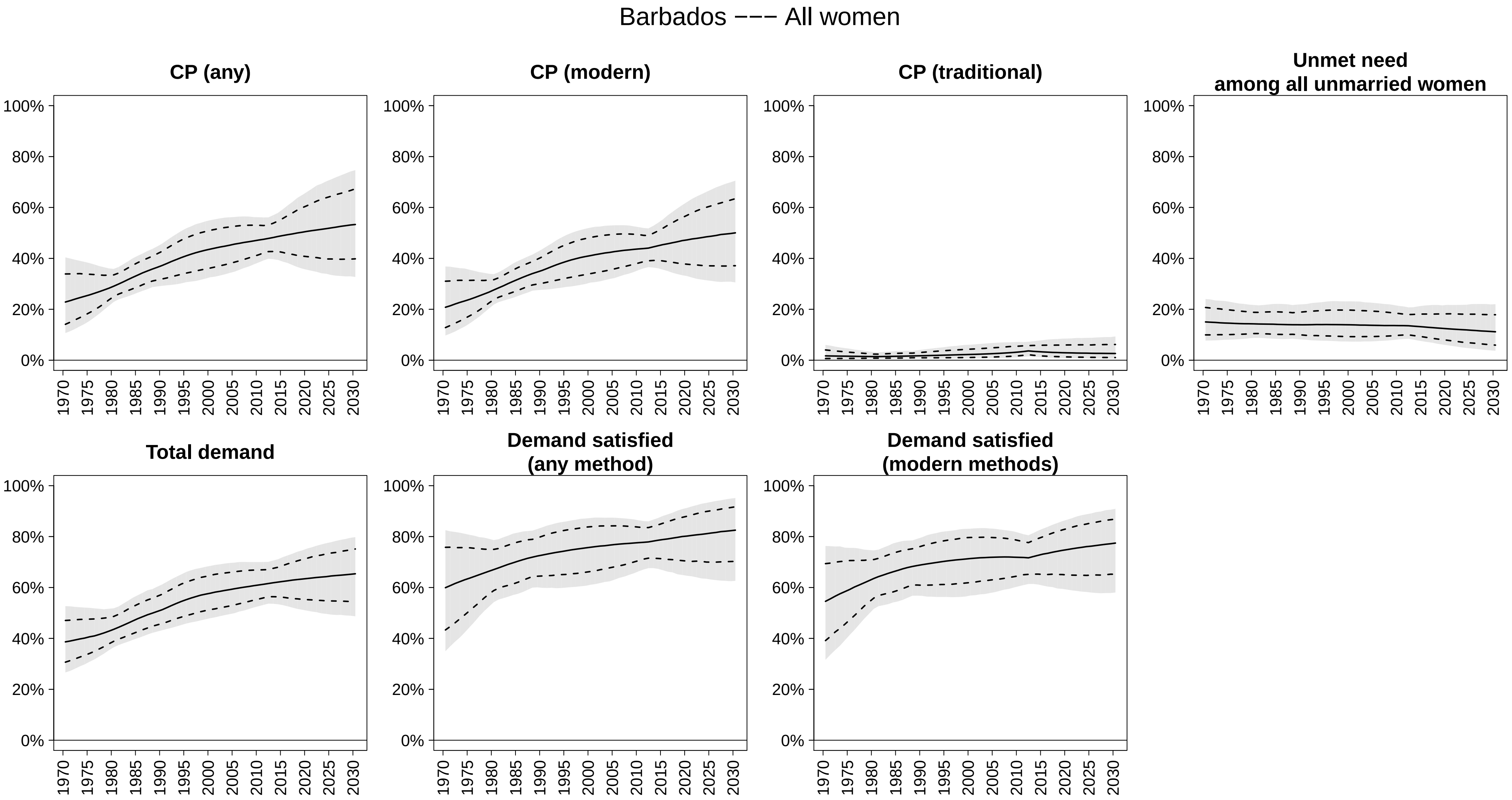
Barbados −−− All women
CP (any)
CP (modern)
CP (traditional)
Unmet need
among all unmarried women
Total demand
Demand satisfied
(any method)
Demand satisfied
(modern methods)
0%
20%
40%
60%
80%
100%
1970
1975
1980
1985
1990
1995
2000
2005
2010
2015
2020
2025
2030

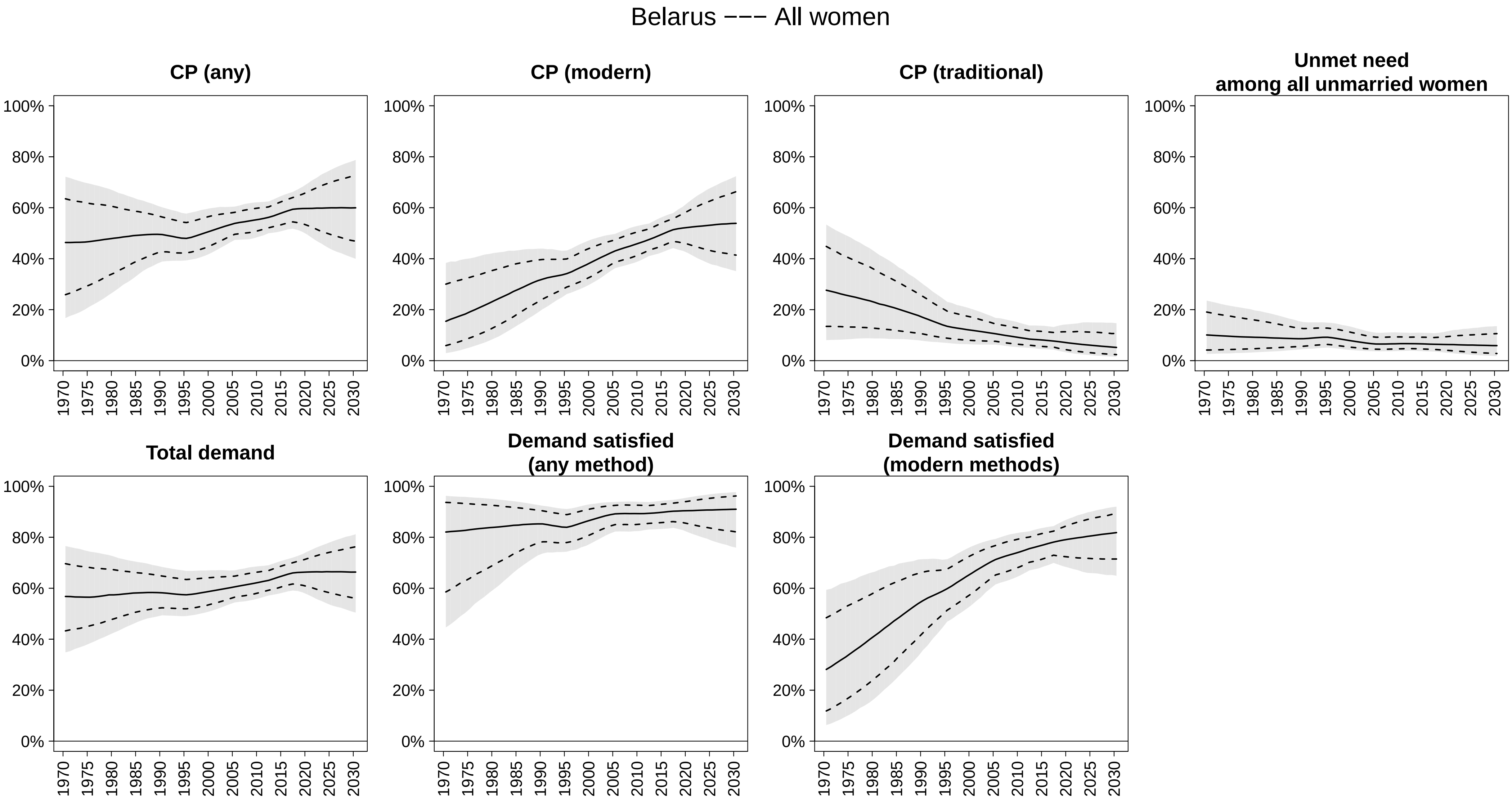
Belarus −−− All women
CP (any)
CP (modern)
CP (traditional)
Unmet need
among all unmarried women
Total demand
Demand satisfied
(any method)
Demand satisfied
(modern methods)
100%
80%
60%
40%
20%
0%
1970
1975
1980
1985
1990
1995
2000
2005
2010
2015
2020
2025
2030

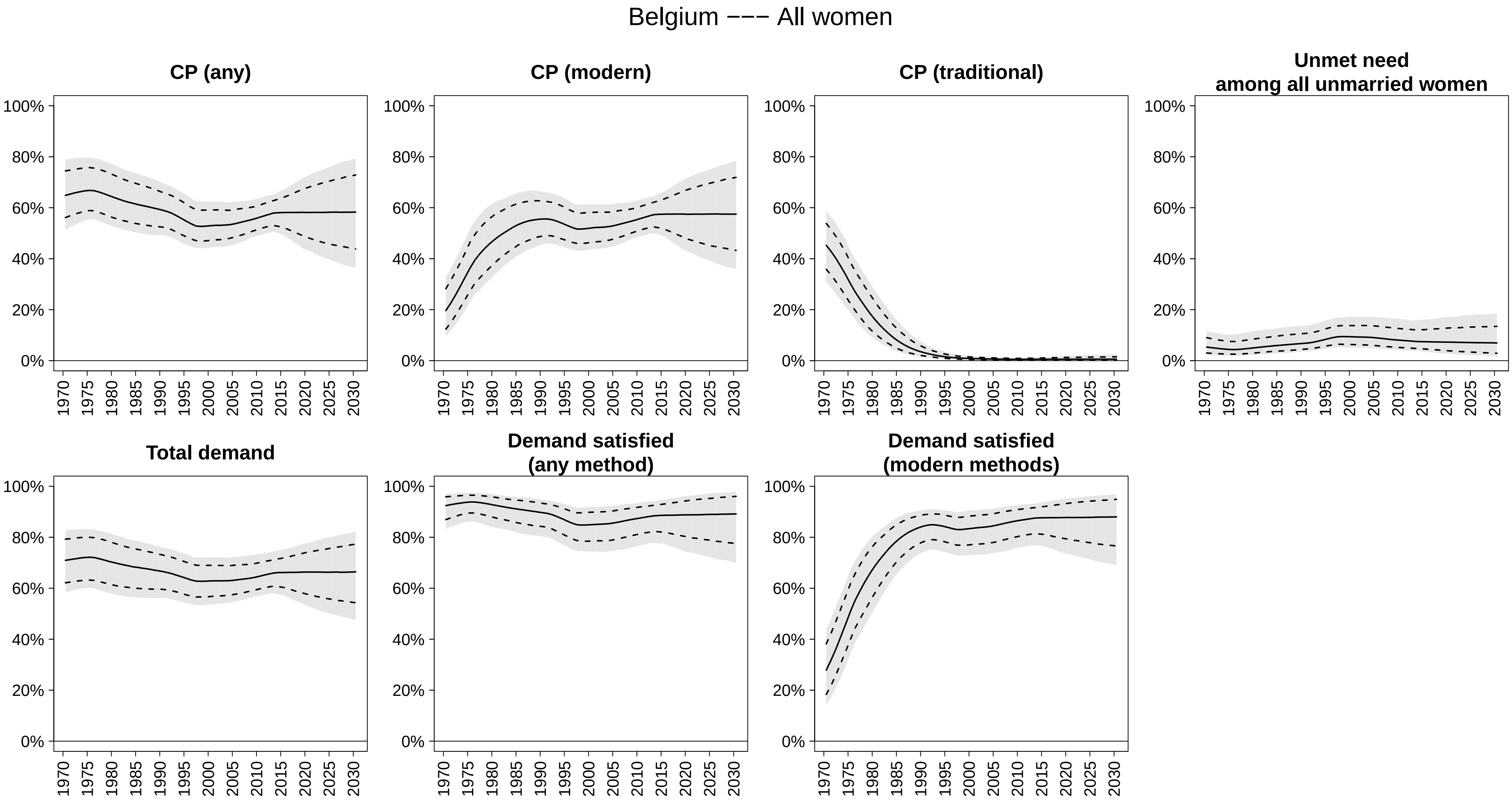
Belgium −−− All women
CP (any)
CP (modern)
CP (traditional)
Unmet need among all unmarried women
Total demand
Demand satisfied (any method)
Demand satisfied (modern methods)
0%
20%
40%
60%
80%
100%
1970
1975
1980
1985
1990
1995
2000
2005
2010
2015
2020
2025
2030

Belize −−− All women

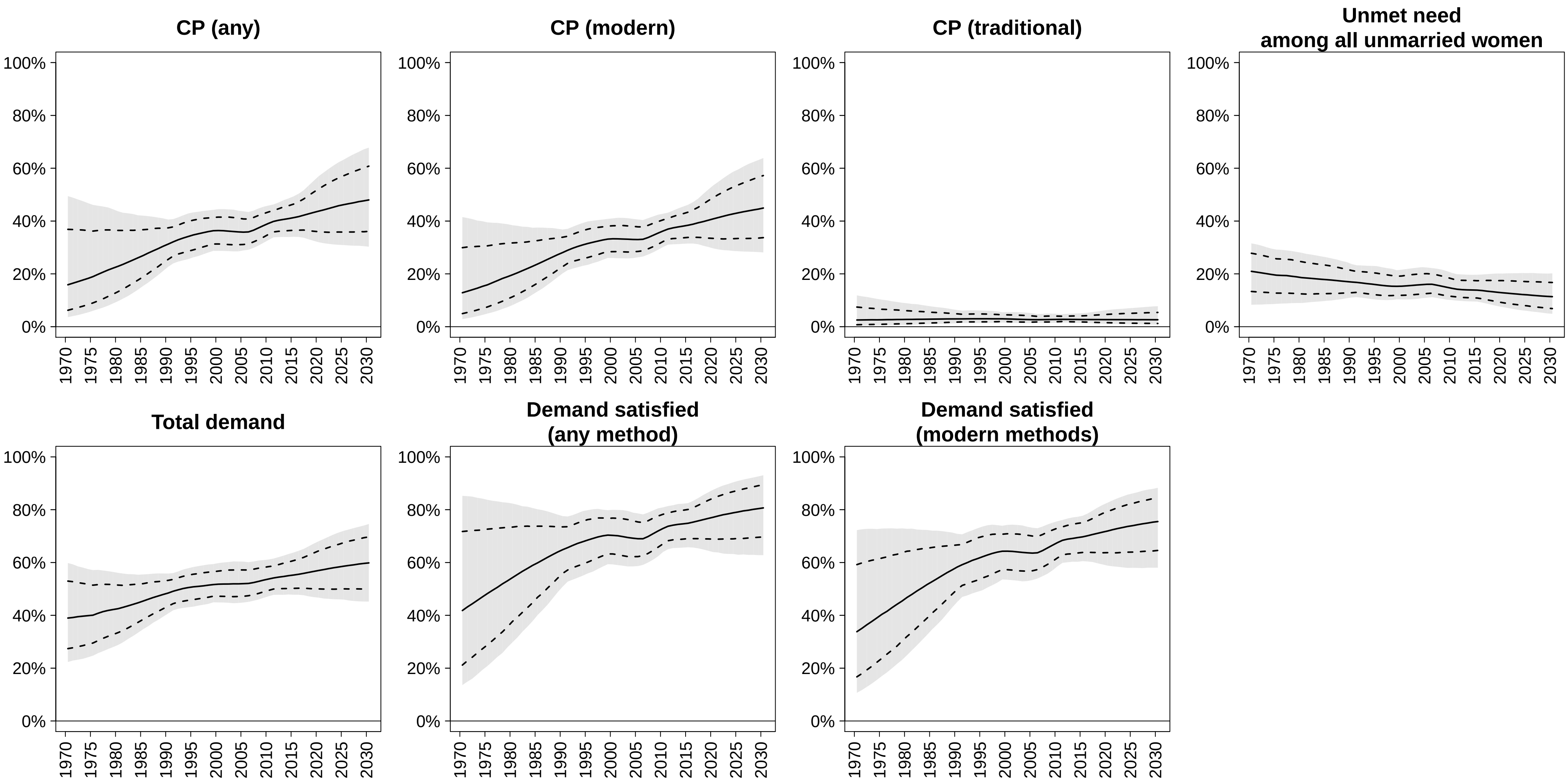

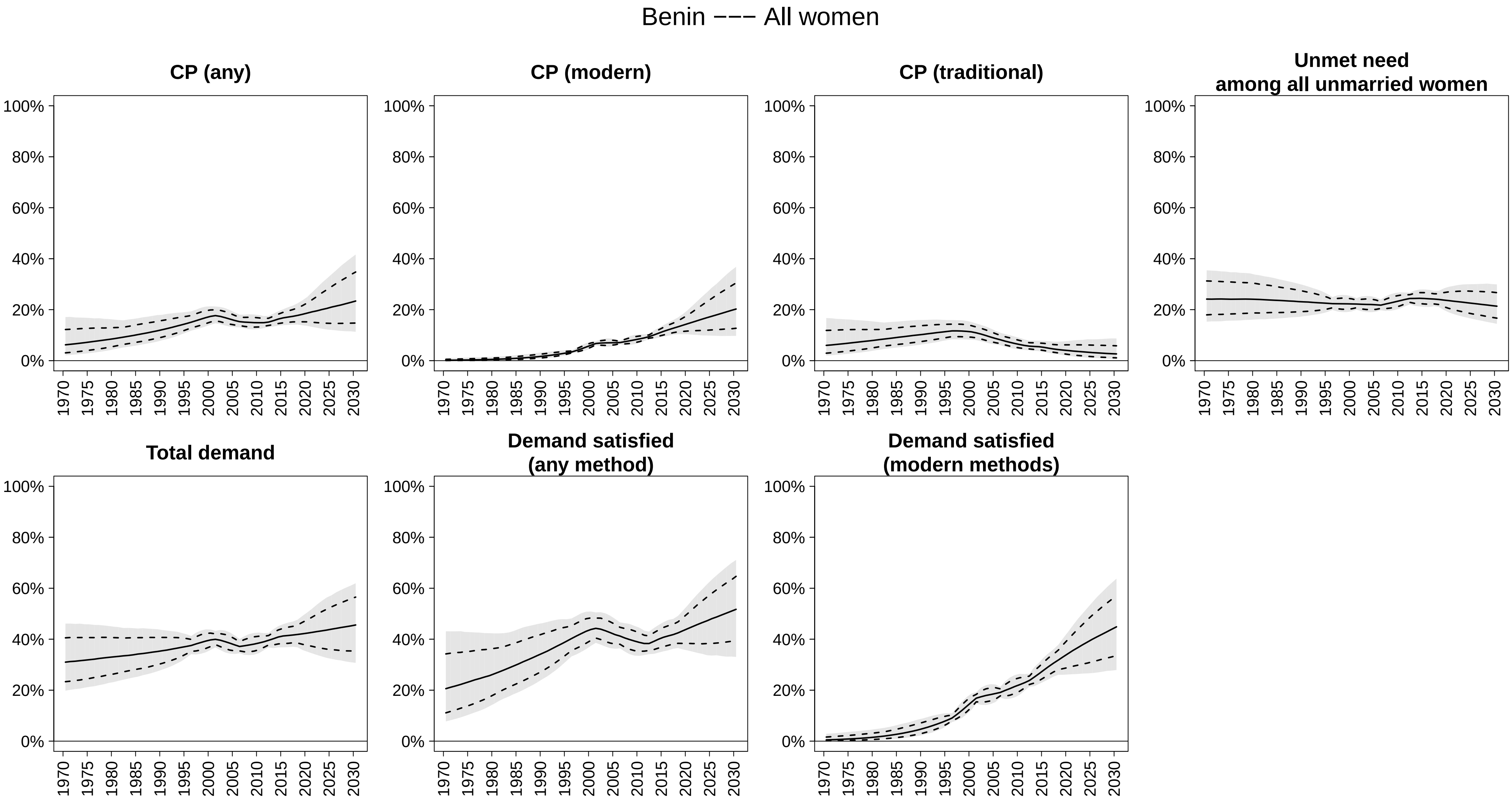
Benin −−− All women
CP (any)
CP (modern)
CP (traditional)
Unmet need among all unmarried women
Total demand
Demand satisfied (any method)
Demand satisfied (modern methods)
0%
20%
40%
60%
80%
100%
1970
1975
1980
1985
1990
1995
2000
2005
2010
2015
2020
2025
2030

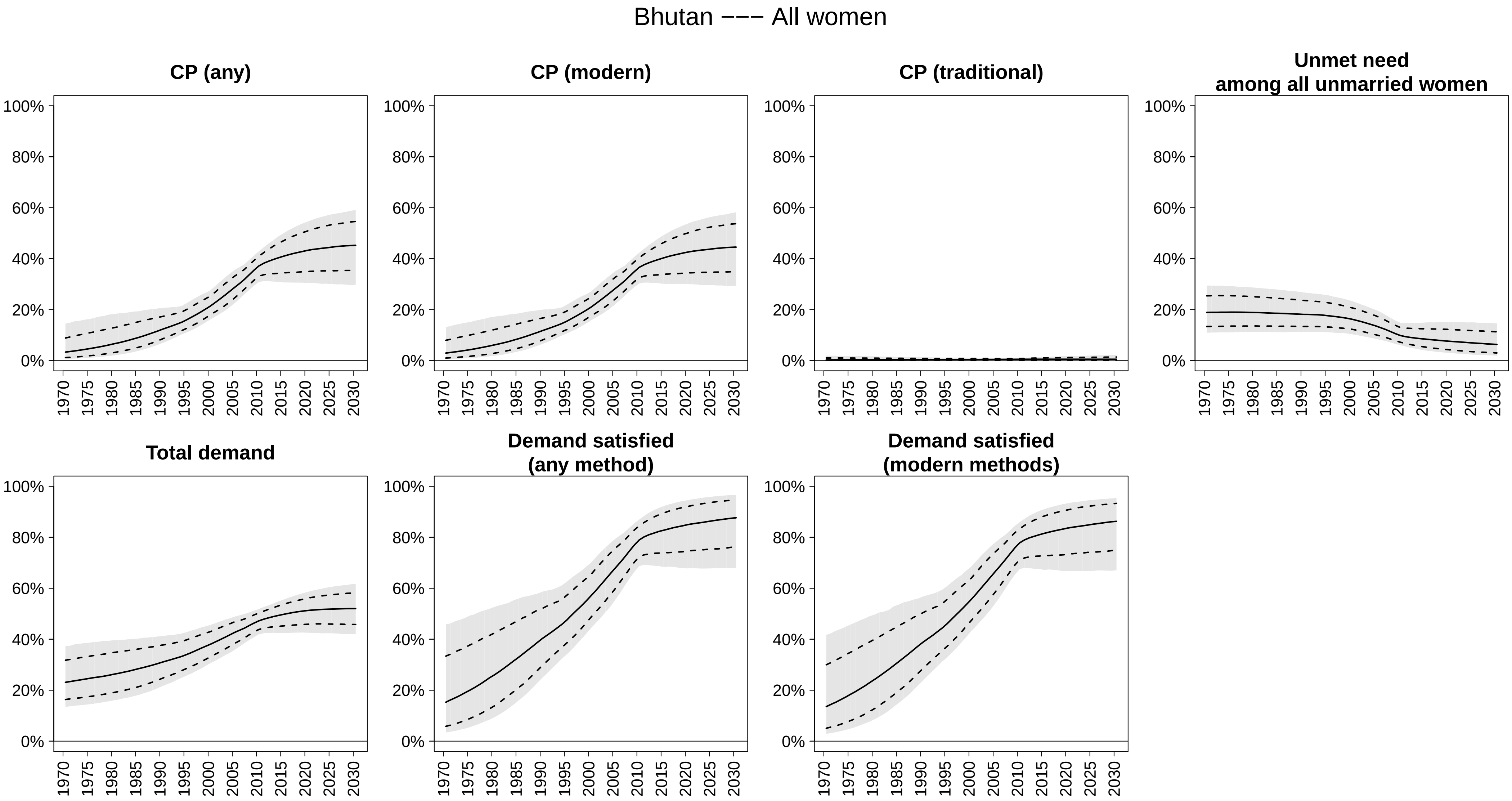
Bhutan --- All women
CP (any)
CP (modern)
CP (traditional)
Unmet need among all unmarried women
Total demand
Demand satisfied (any method)
Demand satisfied (modern methods)
0%
20%
40%
60%
80%
100%
1970
1975
1980
1985
1990
1995
2000
2005
2010
2015
2020
2025
2030

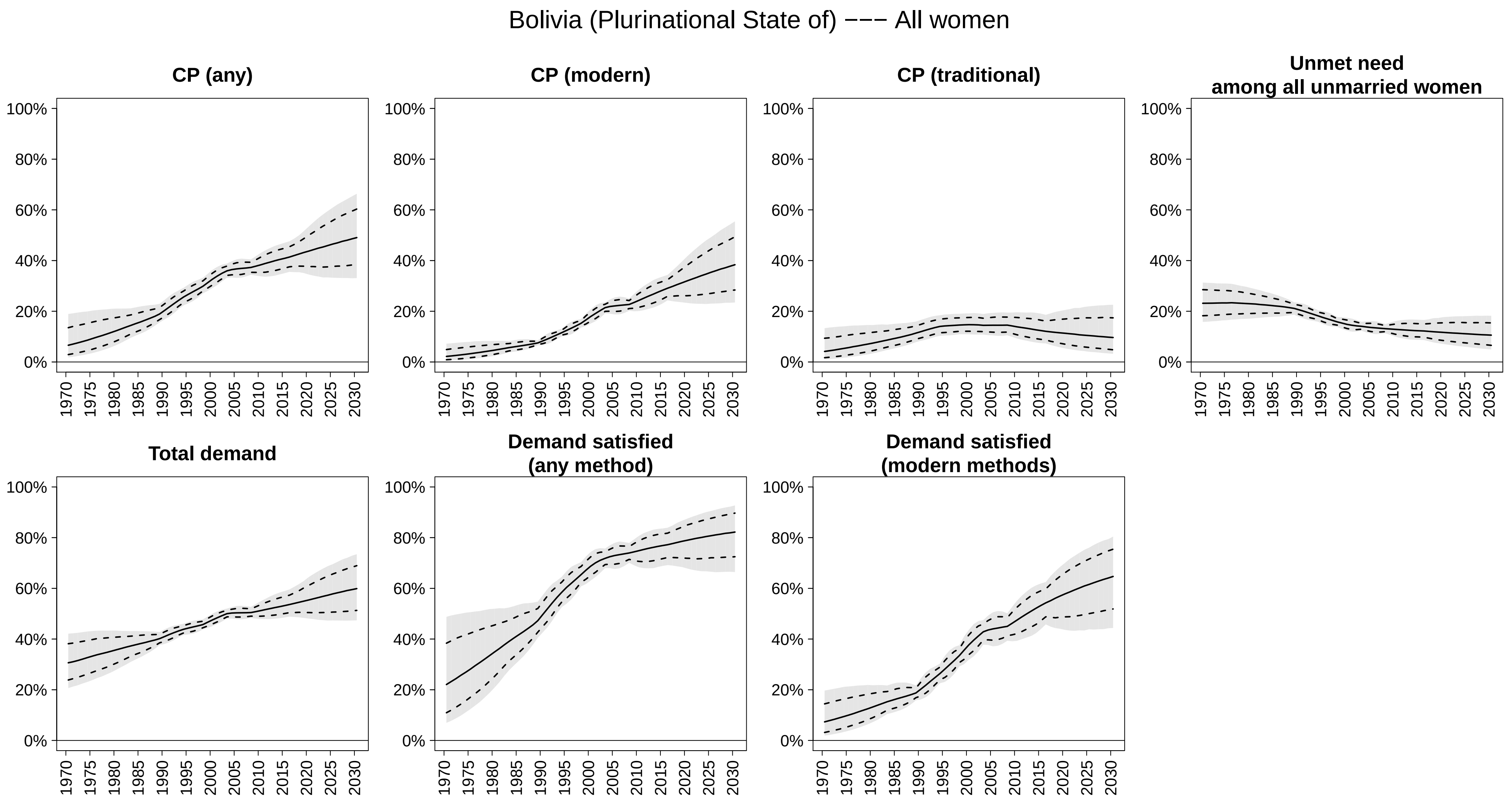
Bolivia (Plurinational State of) −−− All women
CP (any)
CP (modern)
CP (traditional)
Unmet need among all unmarried women
Total demand
Demand satisfied (any method)
Demand satisfied (modern methods)
0%
20%
40%
60%
80%
100%
1970
1975
1980
1985
1990
1995
2000
2005
2010
2015
2020
2025
2030

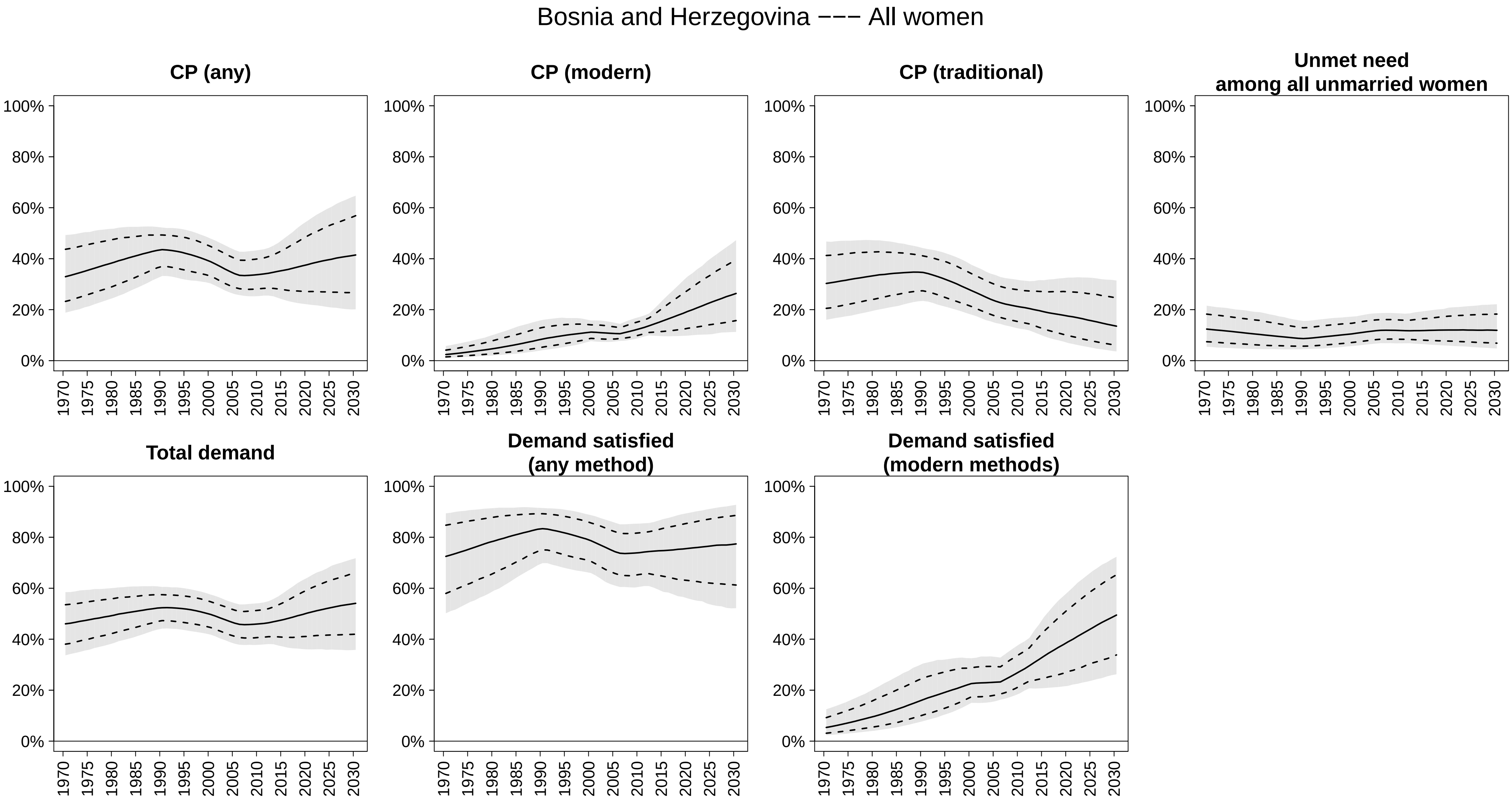
Bosnia and Herzegovina −−− All women
CP (any)
CP (modern)
CP (traditional)
Unmet need
among all unmarried women
Total demand
Demand satisfied
(any method)
Demand satisfied
(modern methods)
0%
20%
40%
60%
80%
100%
1970
1975
1980
1985
1990
1995
2000
2005
2010
2015
2020
2025
2030

Botswana −−− All women

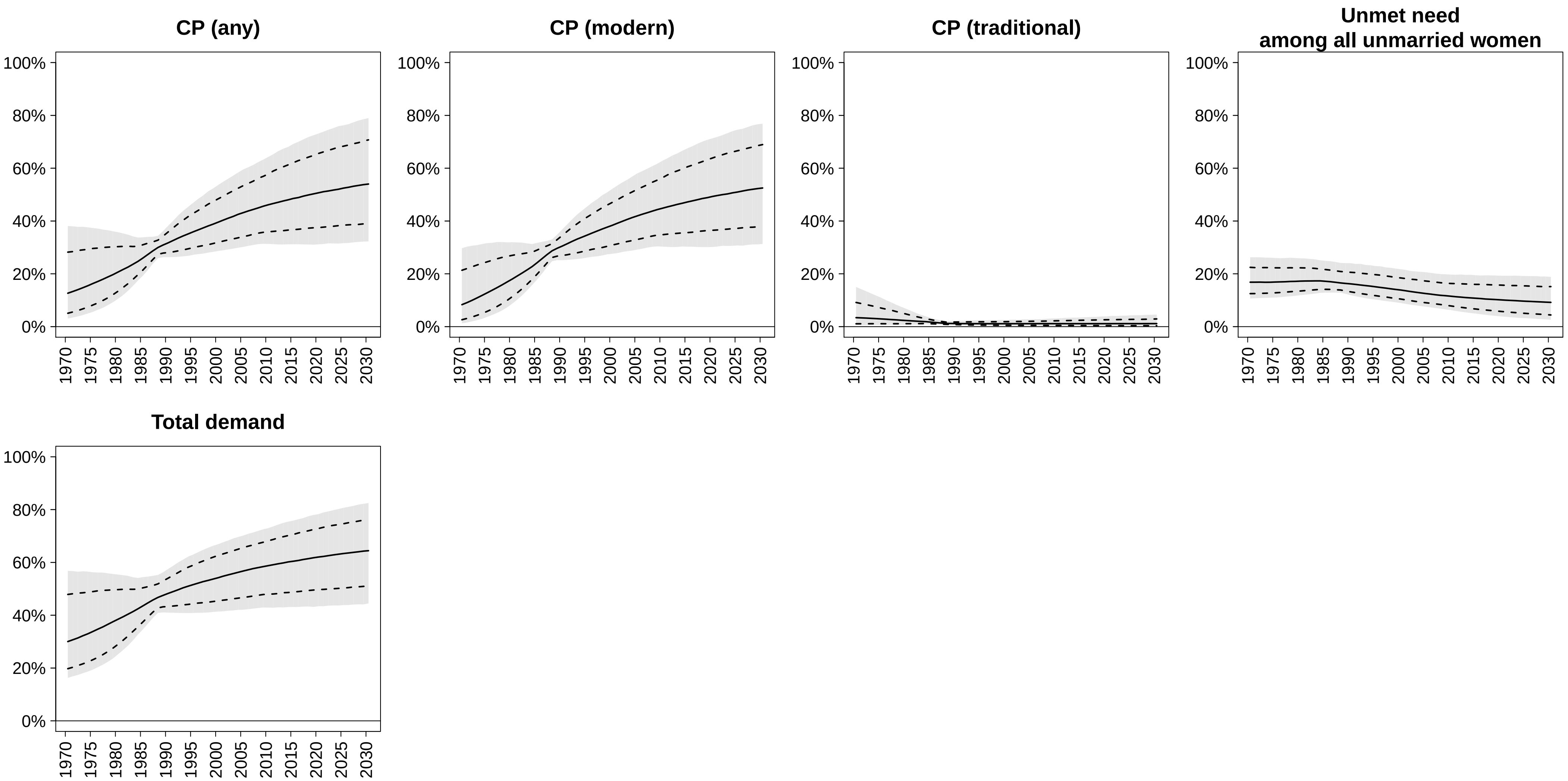

Brazil −−− All women

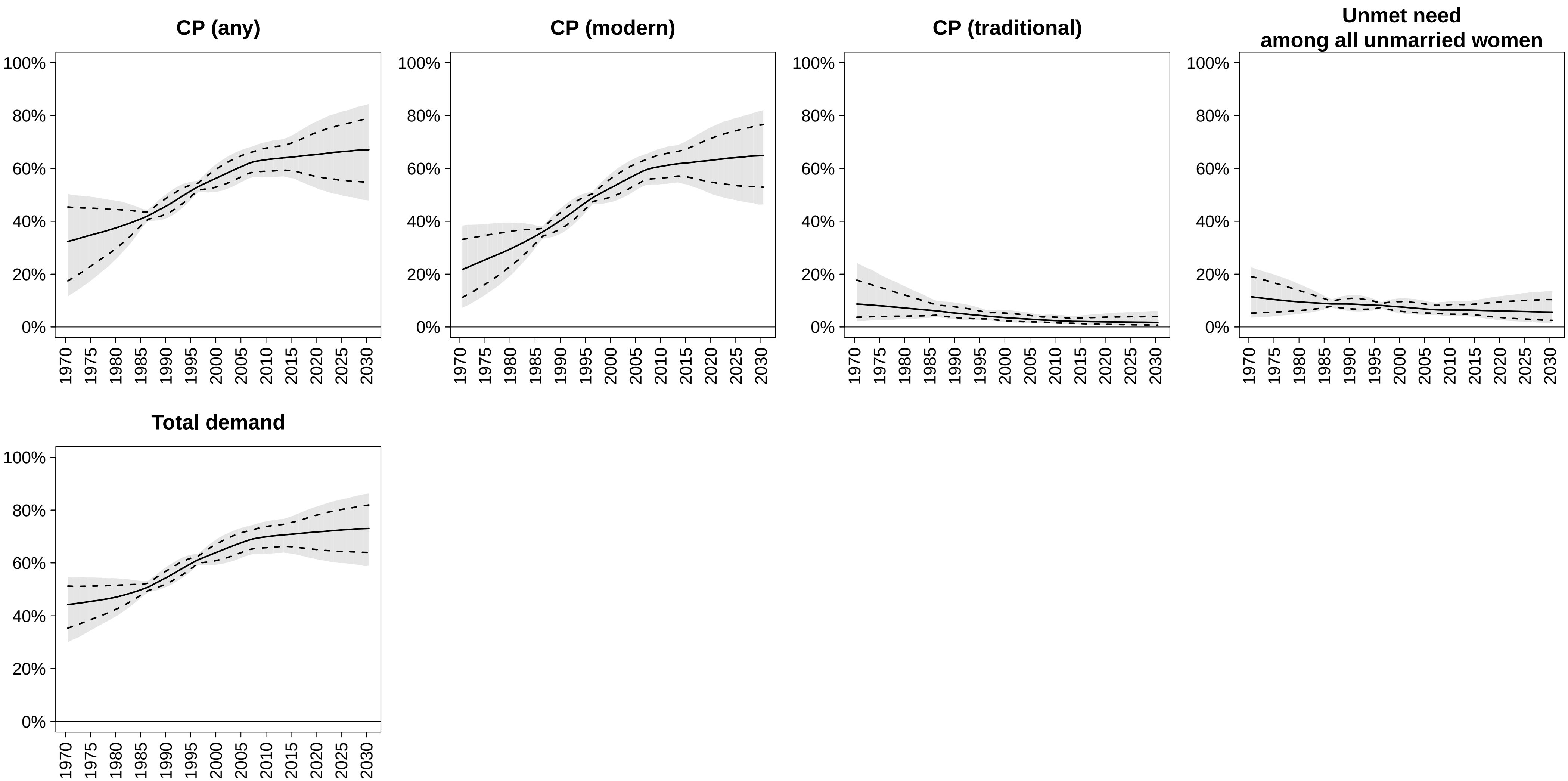

Bulgaria −−− All women

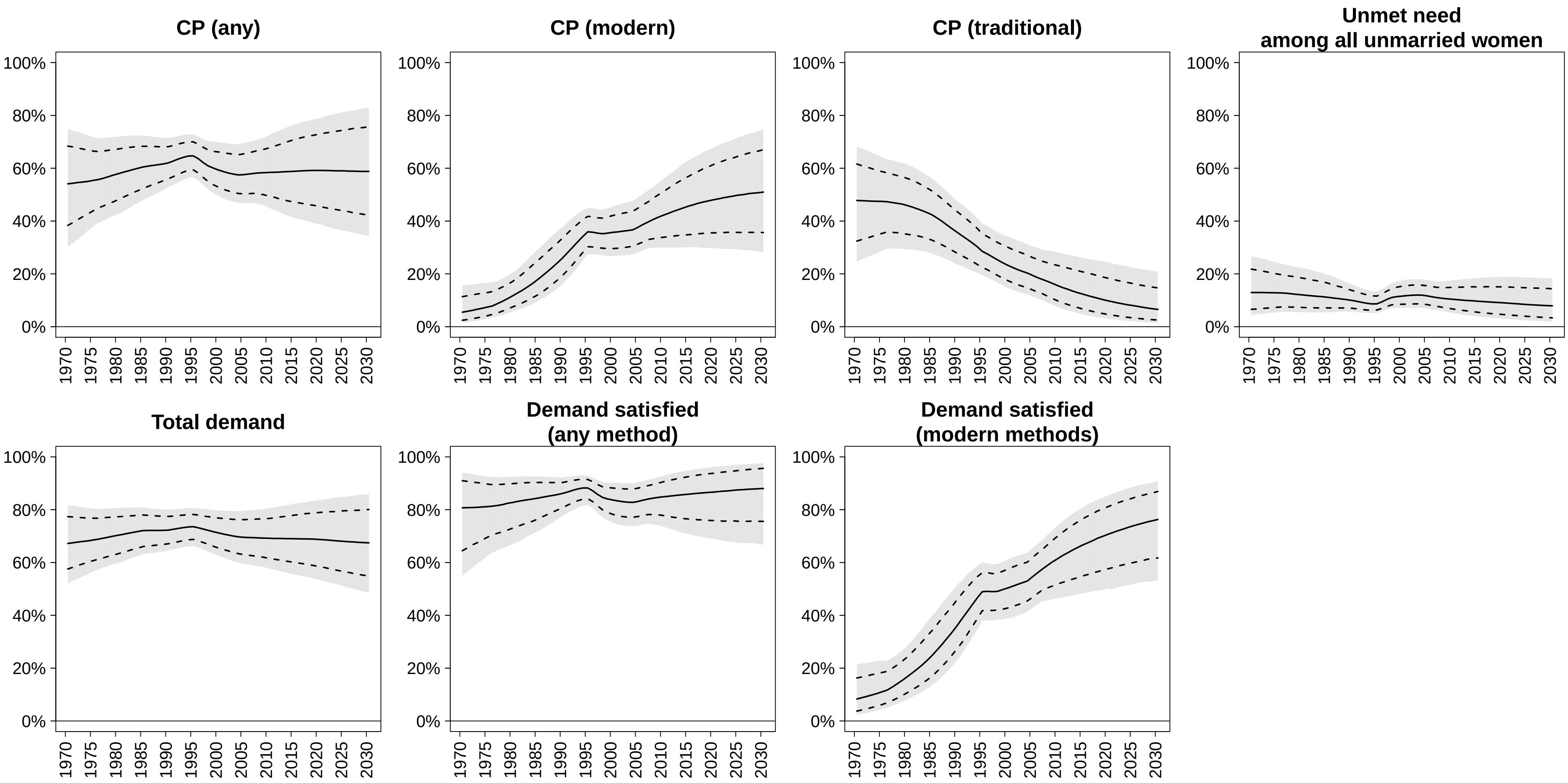

Burkina Faso −−− All women

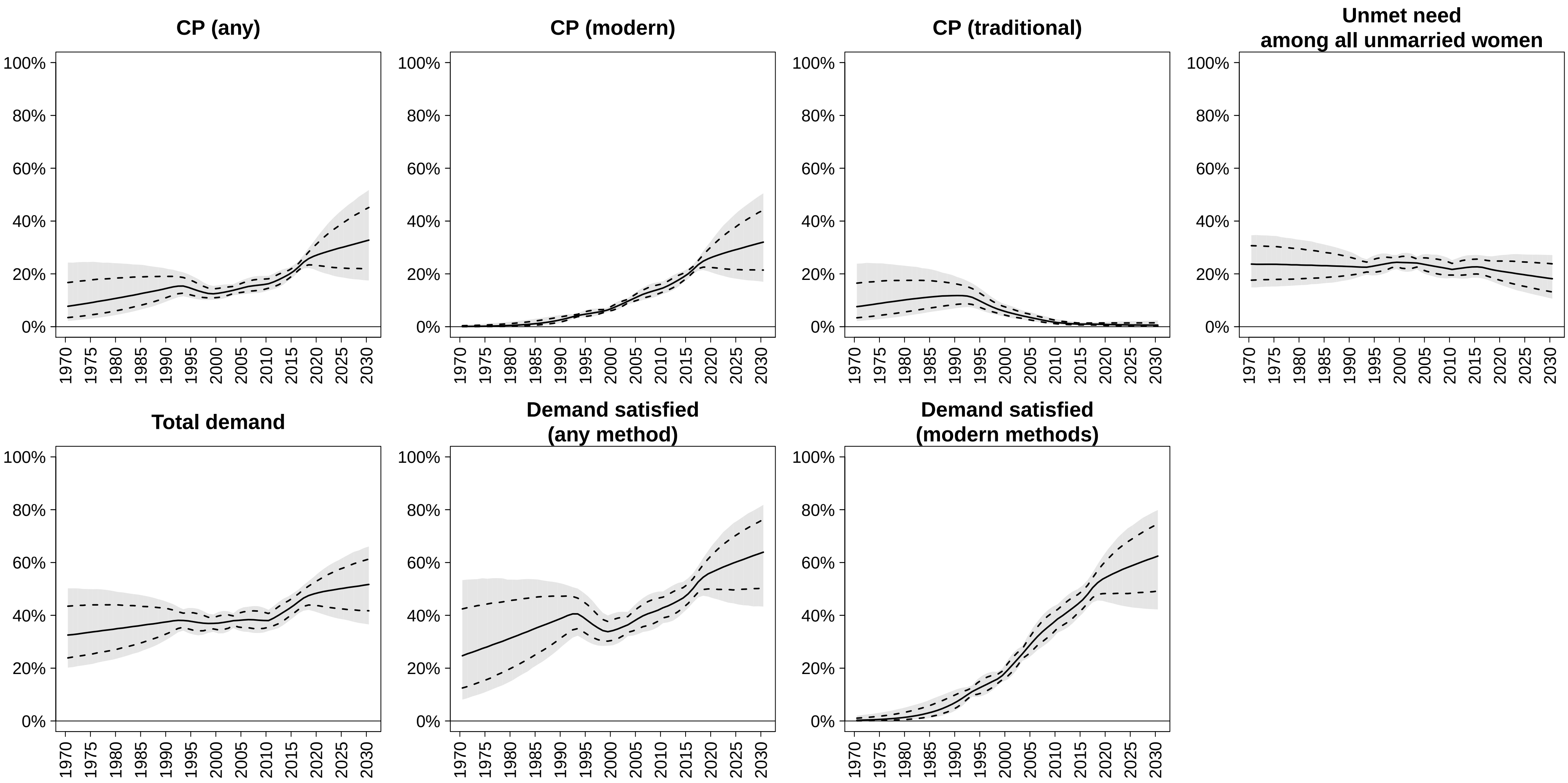

Burundi −−− All women

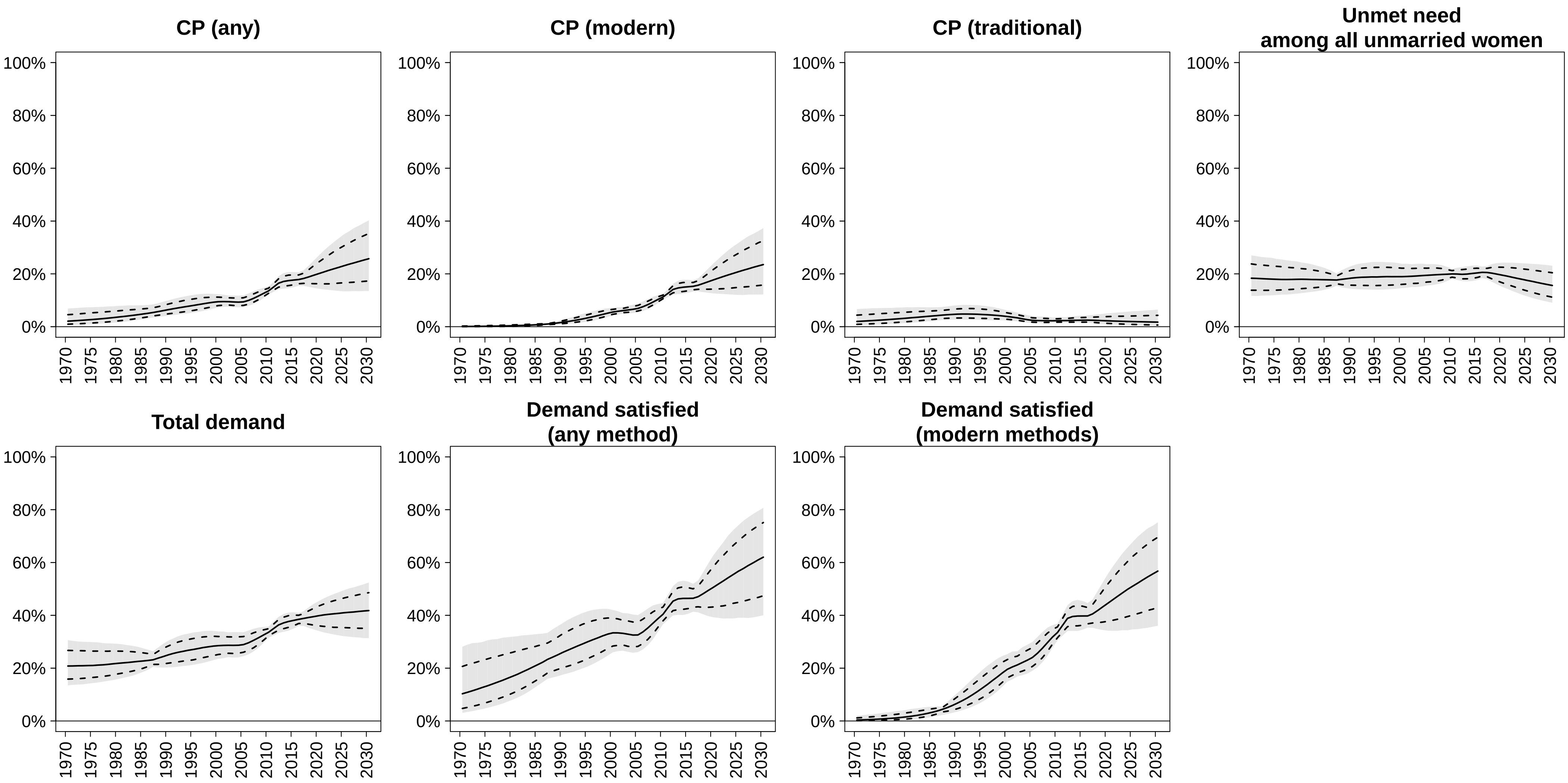

Cabo Verde --- All women

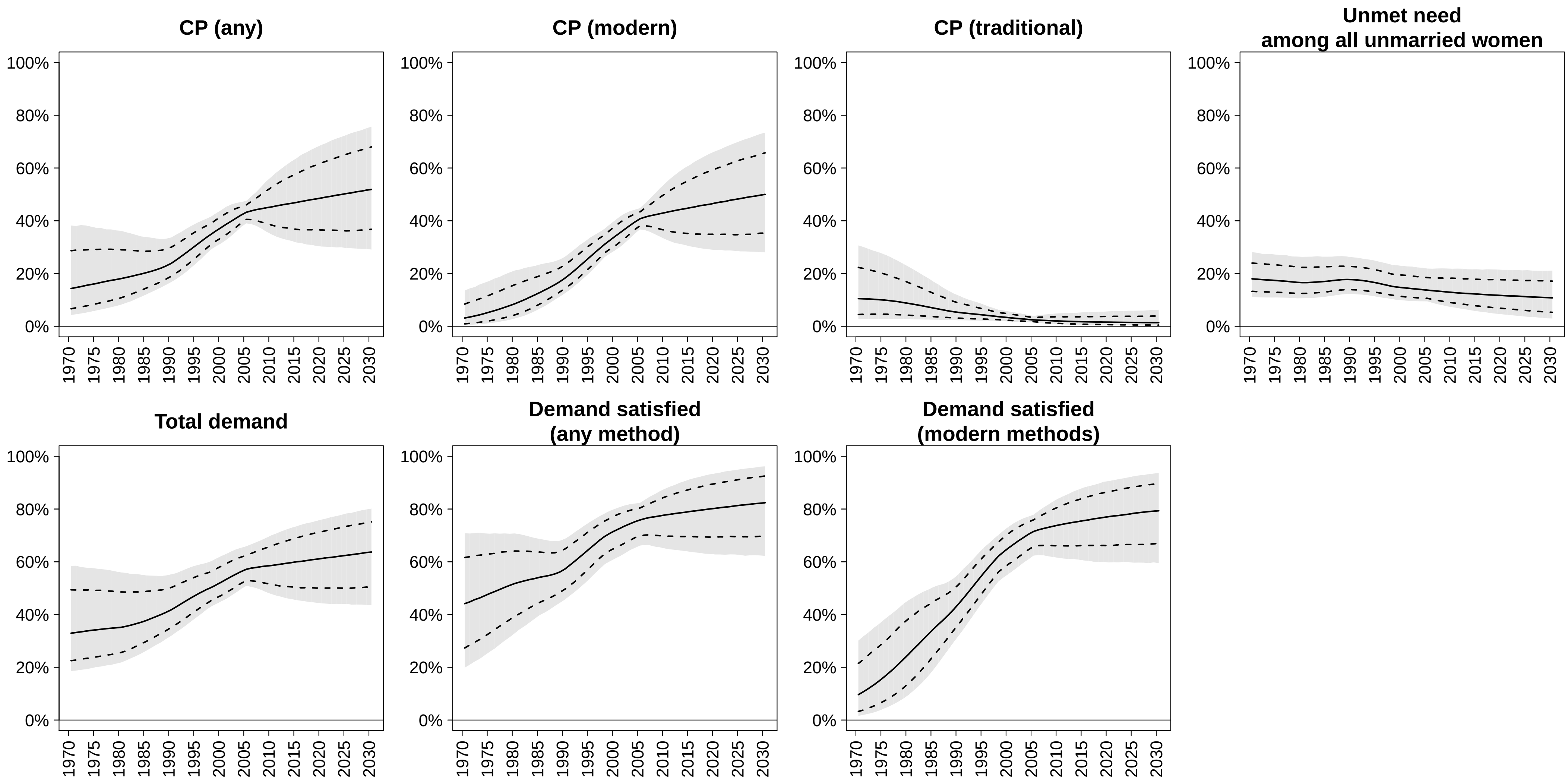

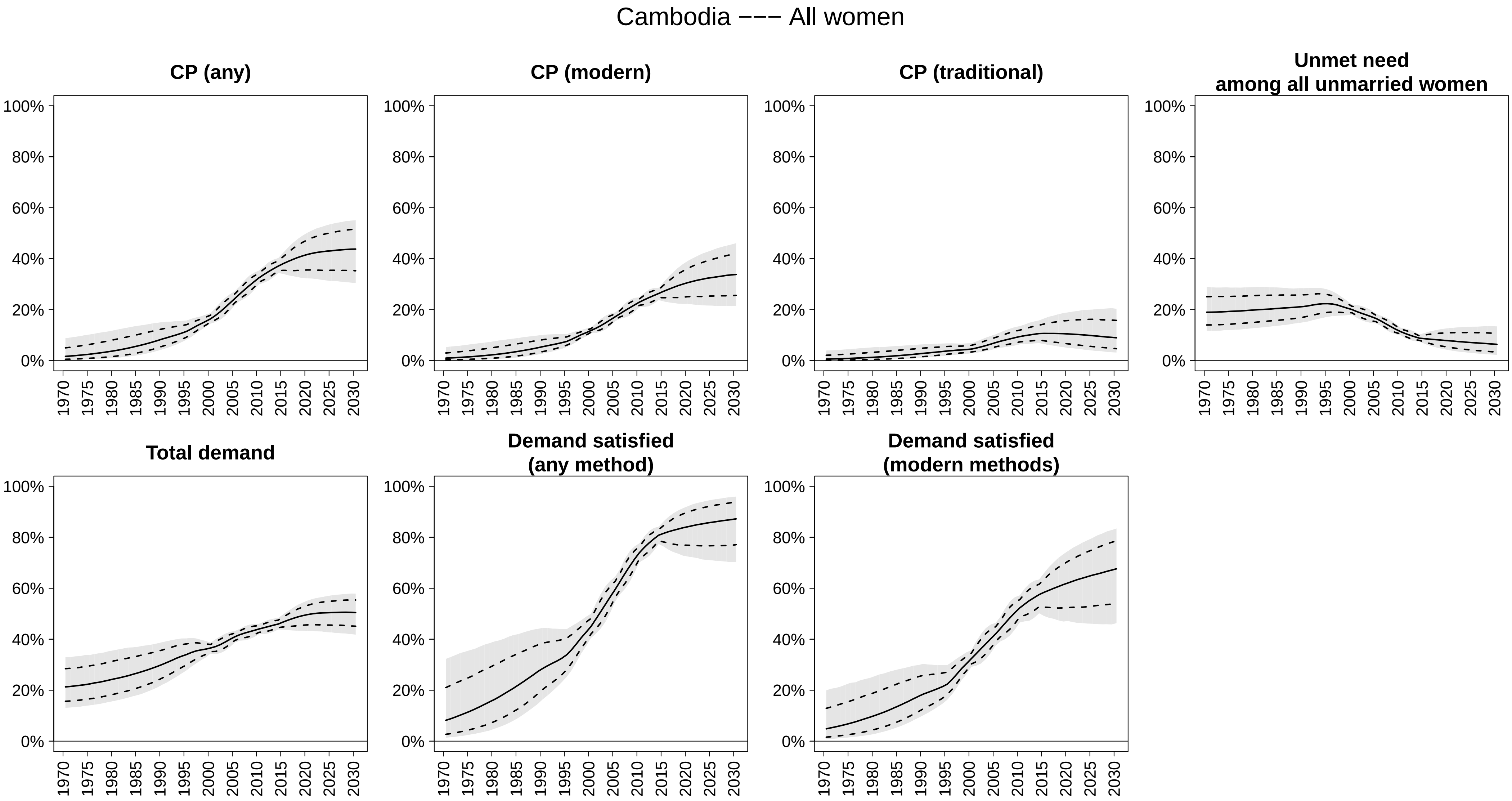
Cambodia −−− All women
CP (any)
CP (modern)
CP (traditional)
Unmet need
among all unmarried women
Total demand
Demand satisfied
(any method)
Demand satisfied
(modern methods)
0%
20%
40%
60%
80%
100%
1970
1975
1980
1985
1990
1995
2000
2005
2010
2015
2020
2025
2030

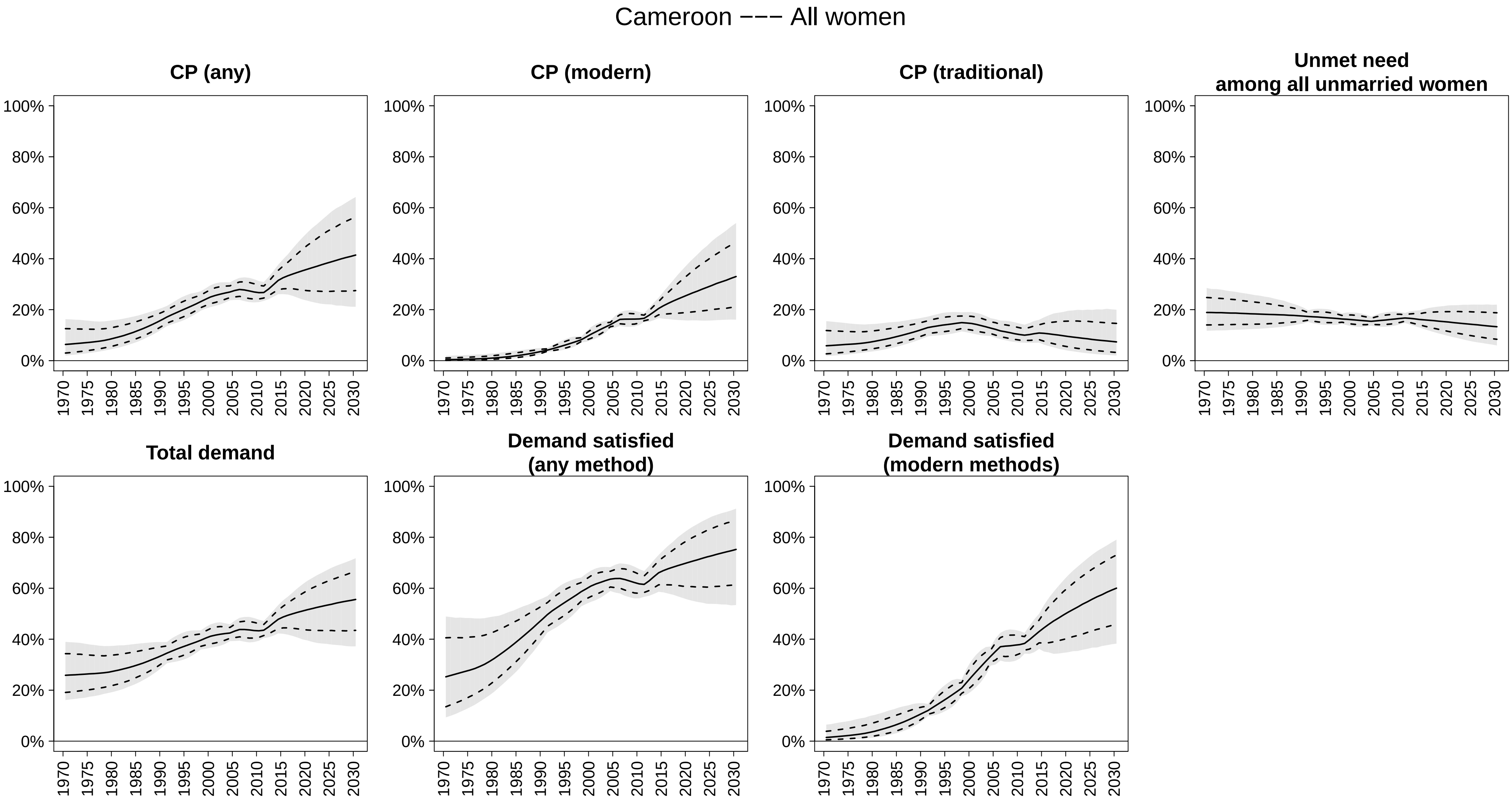
Cameroon −−− All women
CP (any)
CP (modern)
CP (traditional)
Unmet need
among all unmarried women
Total demand
Demand satisfied
(any method)
Demand satisfied
(modern methods)
0%
20%
40%
60%
80%
100%
1970
1975
1980
1985
1990
1995
2000
2005
2010
2015
2020
2025
2030

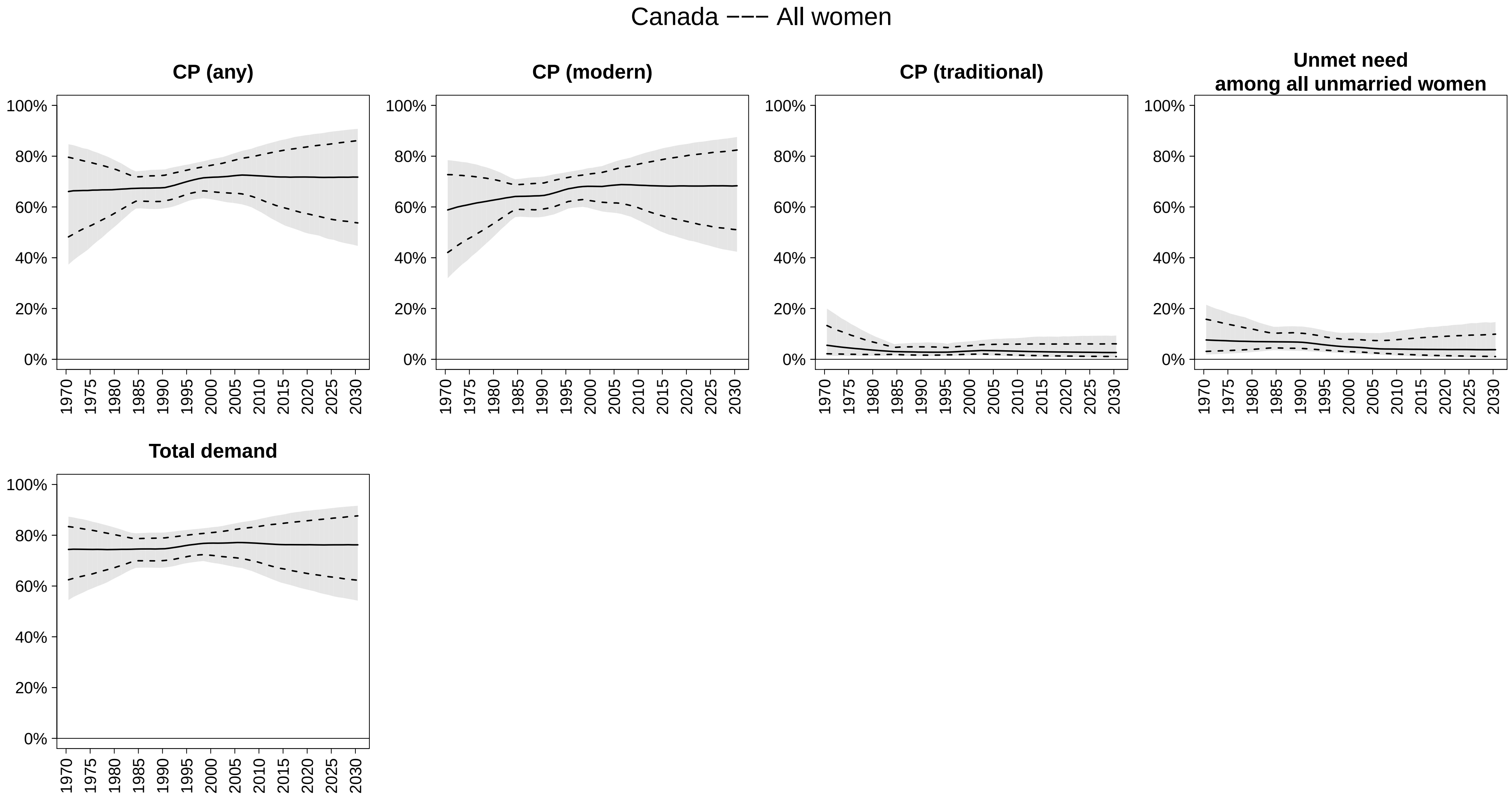
Canada −−− All women
CP (any)
CP (modern)
CP (traditional)
Unmet need
among all unmarried women
Total demand
0%
20%
40%
60%
80%
100%
1970
1975
1980
1985
1990
1995
2000
2005
2010
2015
2020
2025
2030

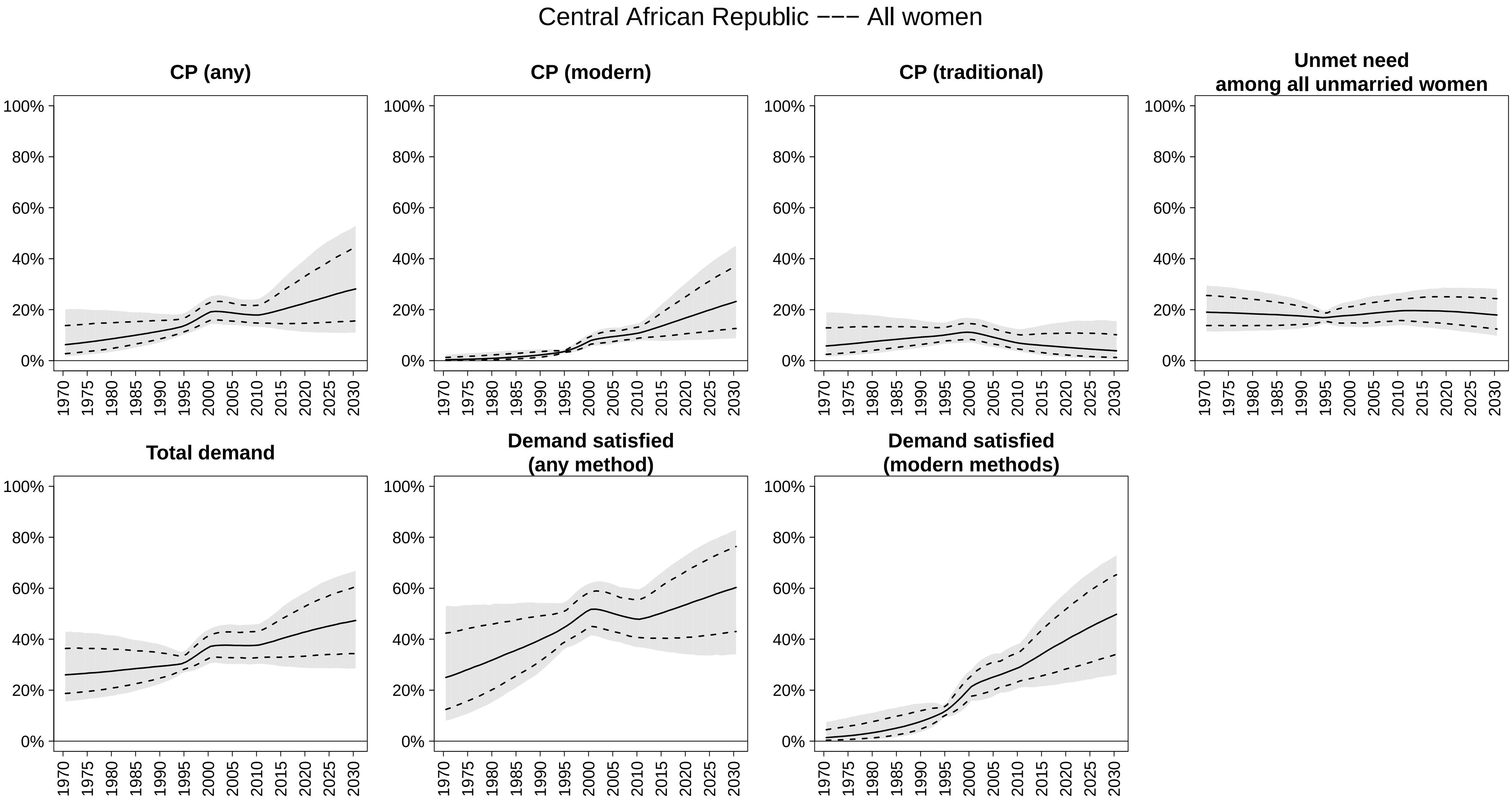
Central African Republic −−− All women
CP (any)
CP (modern)
CP (traditional)
Unmet need
among all unmarried women
Total demand
Demand satisfied
(any method)
Demand satisfied
(modern methods)
0%
20%
40%
60%
80%
100%
1970
1975
1980
1985
1990
1995
2000
2005
2010
2015
2020
2025
2030

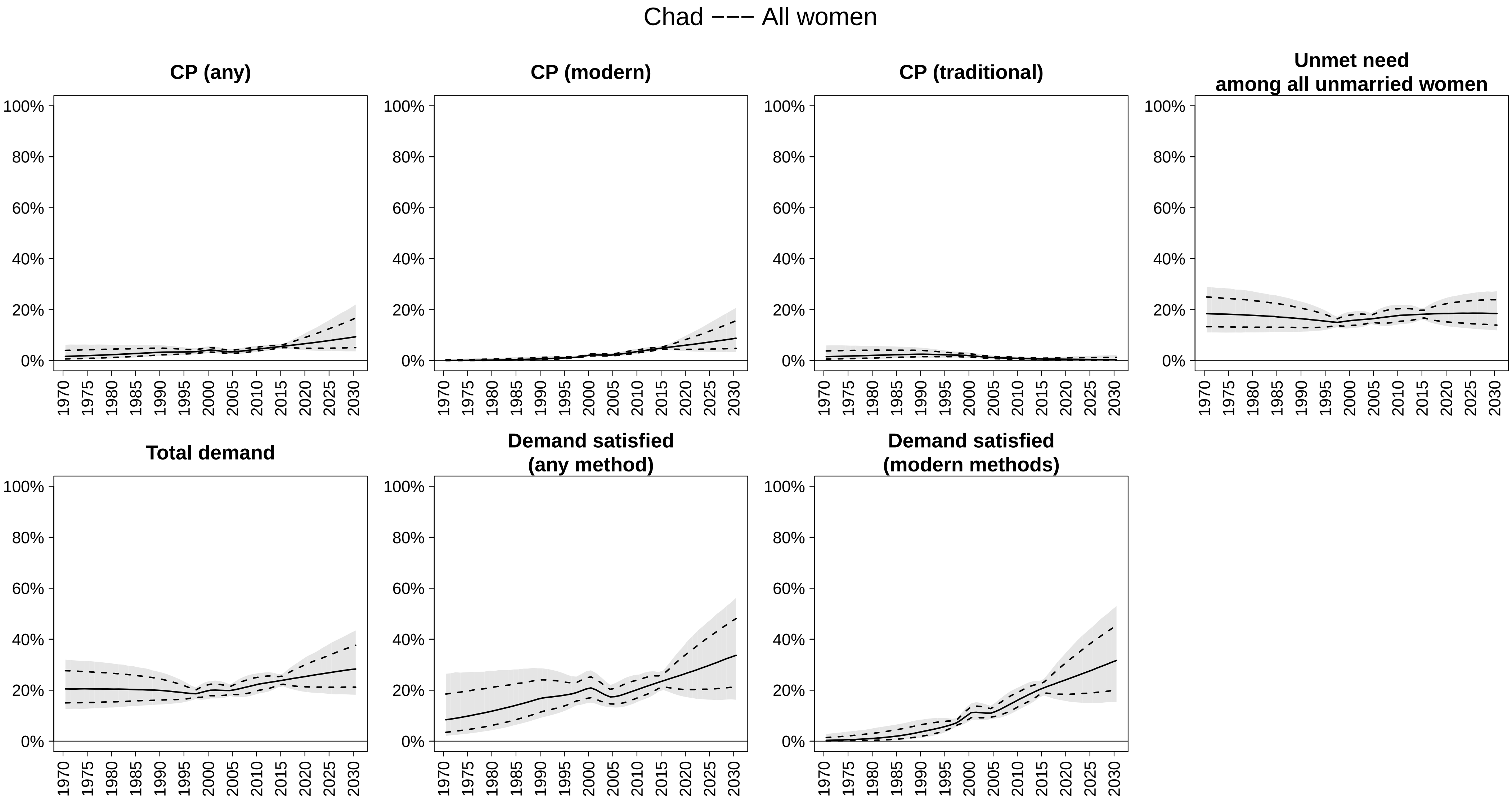
Chad −−− All women
CP (any)
CP (modern)
CP (traditional)
Unmet need
among all unmarried women
Total demand
Demand satisfied
(any method)
Demand satisfied
(modern methods)
100%
80%
60%
40%
20%
0%
1970
1975
1980
1985
1990
1995
2000
2005
2010
2015
2020
2025
2030

Chile −−− All women

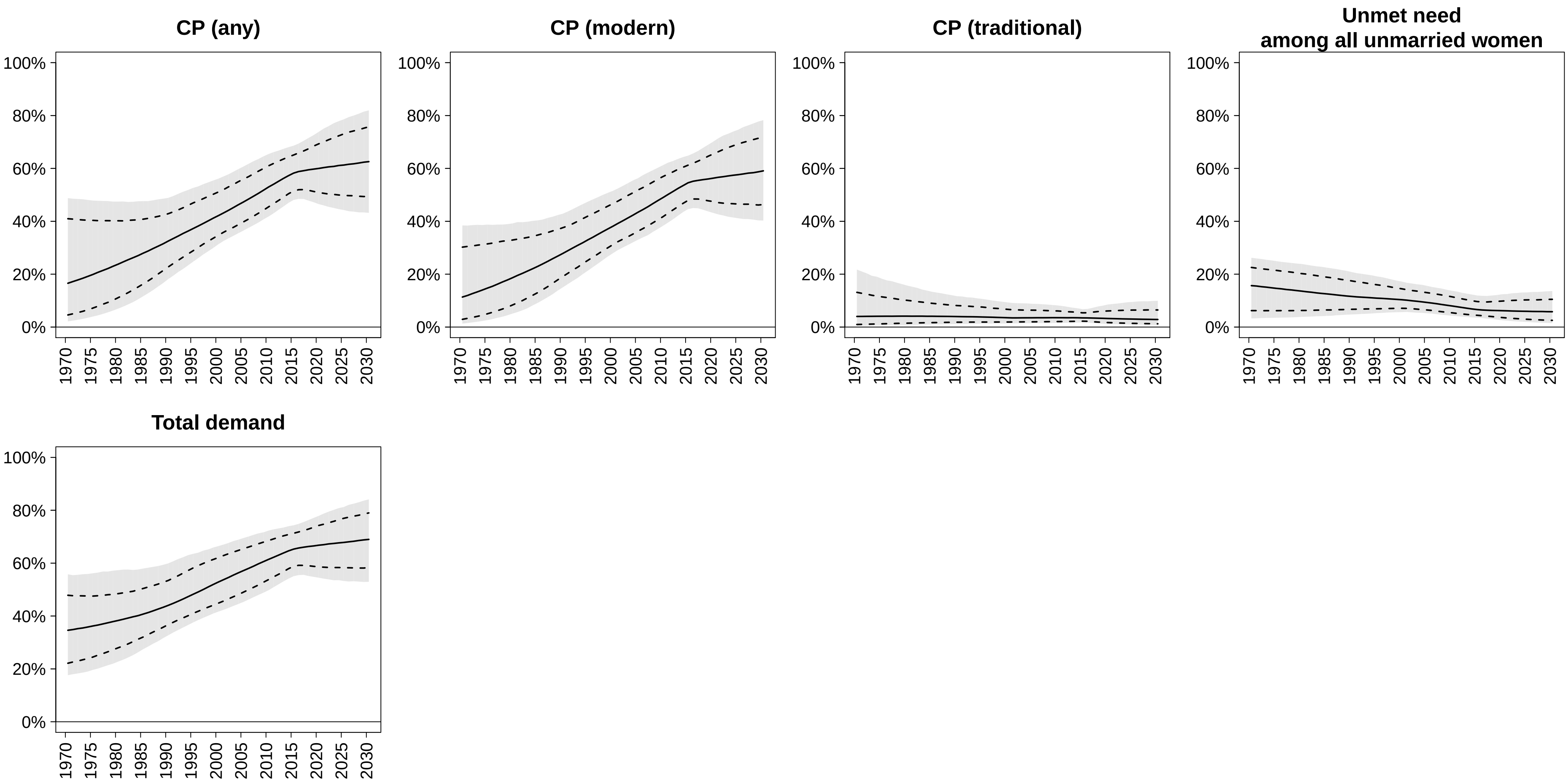

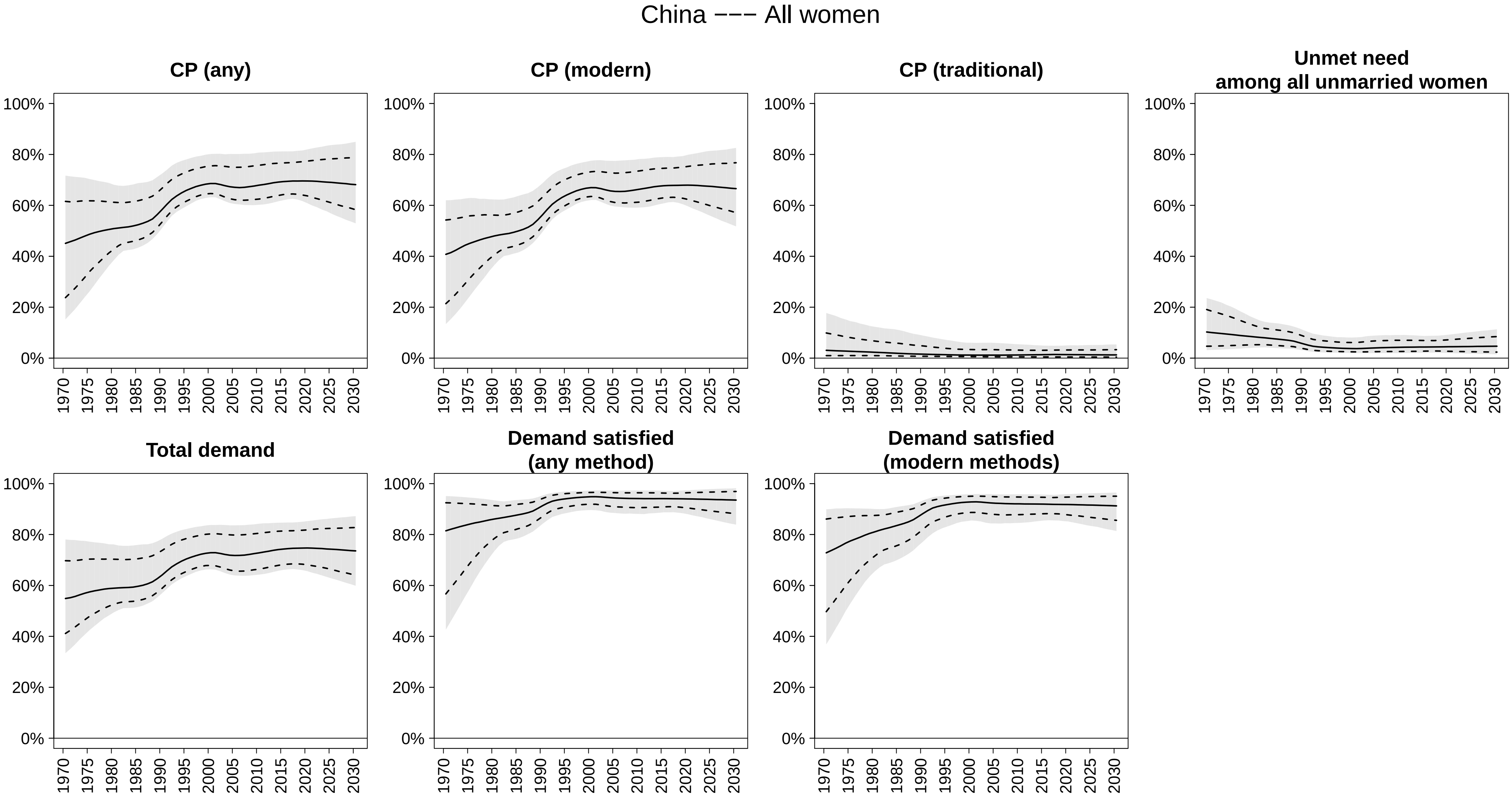
China −−− All women
CP (any)
CP (modern)
CP (traditional)
Unmet need
among all unmarried women
Total demand
Demand satisfied
(any method)
Demand satisfied
(modern methods)
0%
20%
40%
60%
80%
100%
1970
1975
1980
1985
1990
1995
2000
2005
2010
2015
2020
2025
2030

China, Hong Kong Special Administrative Region −−− All women

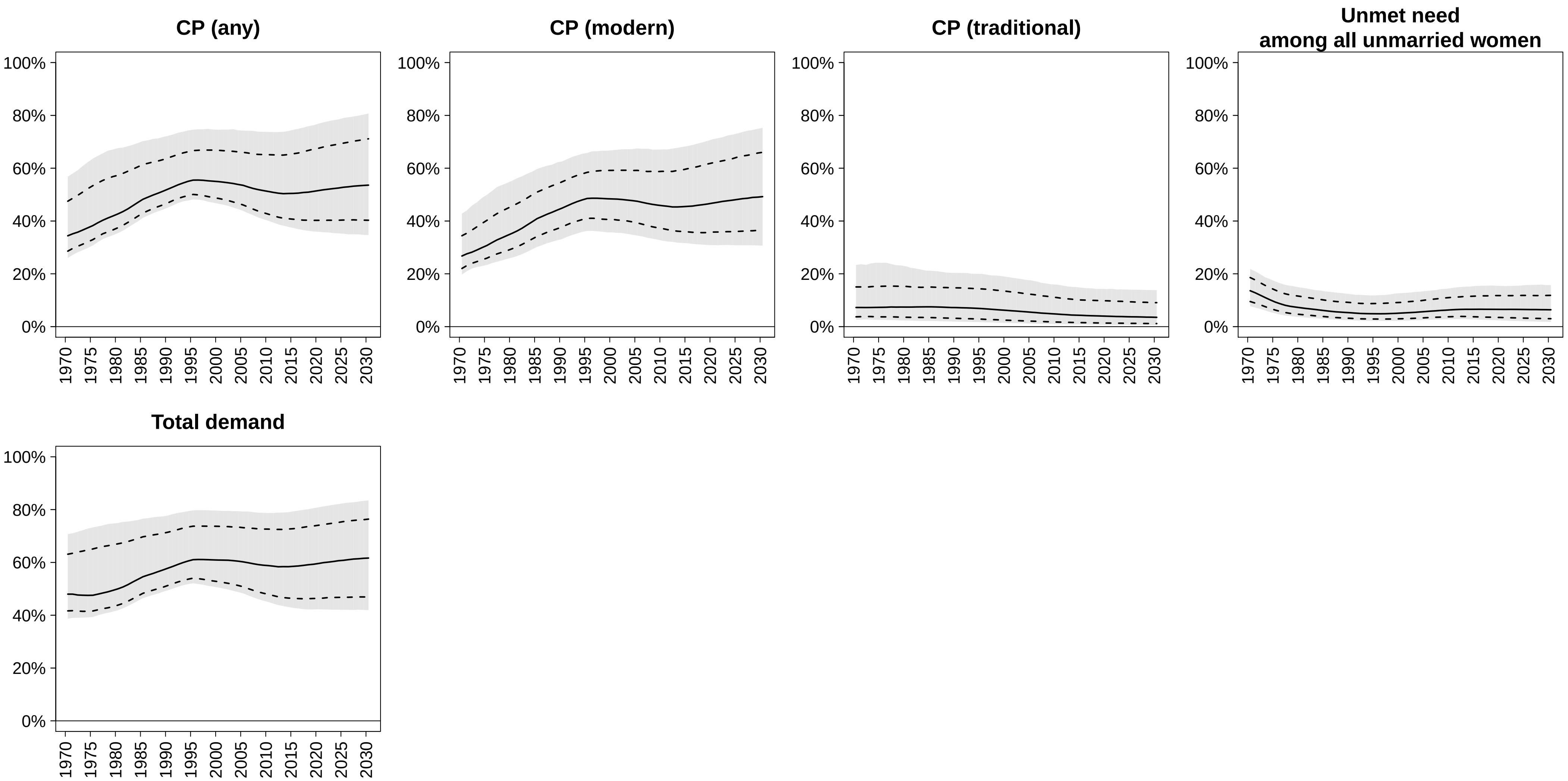

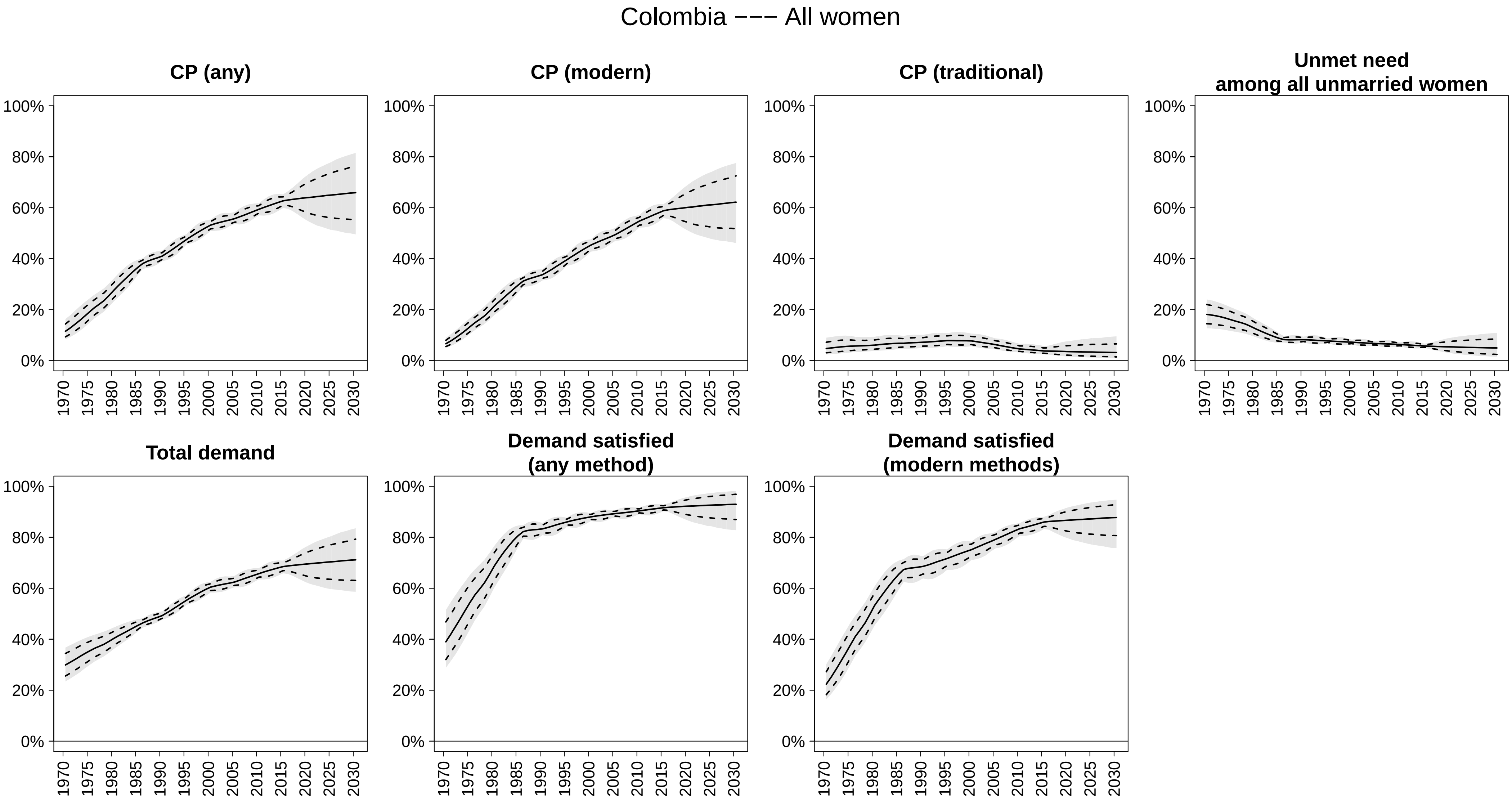
Colombia −−− All women
CP (any)
CP (modern)
CP (traditional)
Unmet need among all unmarried women
Total demand
Demand satisfied (any method)
Demand satisfied (modern methods)
0%
20%
40%
60%
80%
100%
1970
1975
1980
1985
1990
1995
2000
2005
2010
2015
2020
2025
2030

Comoros −−− All women

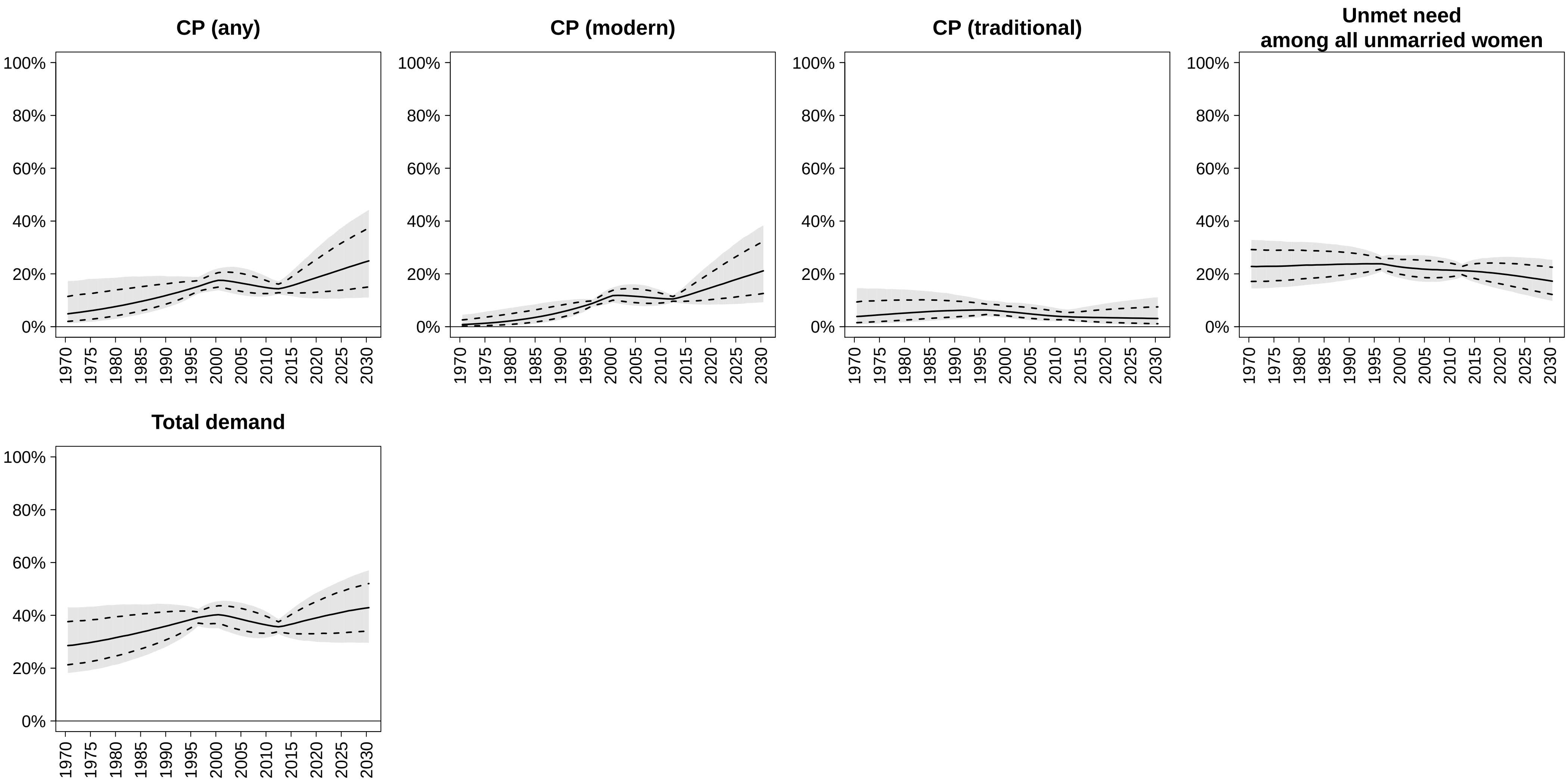

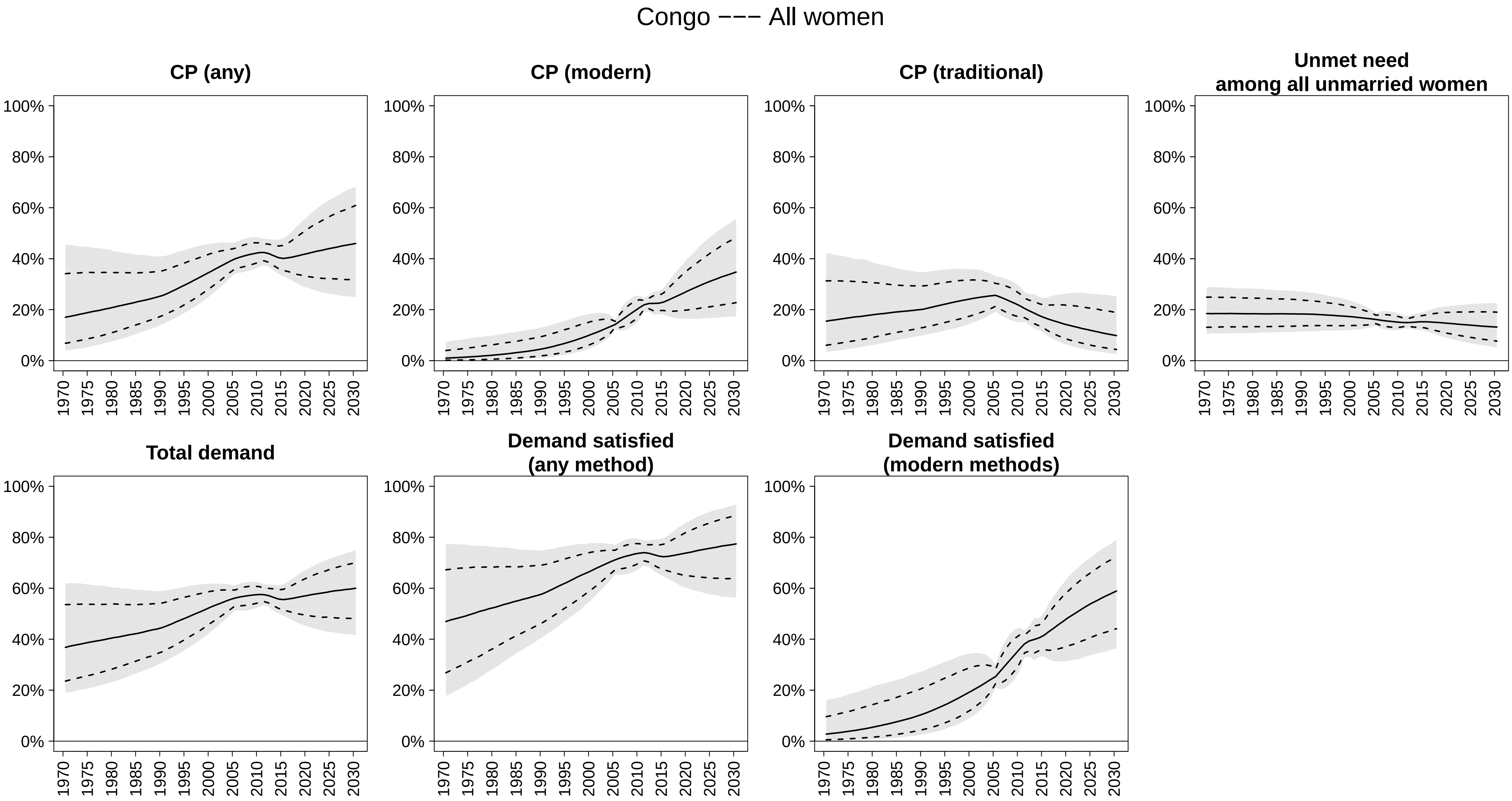
Congo −−− All women
CP (any)
CP (modern)
CP (traditional)
Unmet need
among all unmarried women
Total demand
Demand satisfied
(any method)
Demand satisfied
(modern methods)
0%
20%
40%
60%
80%
100%
1970
1975
1980
1985
1990
1995
2000
2005
2010
2015
2020
2025
2030

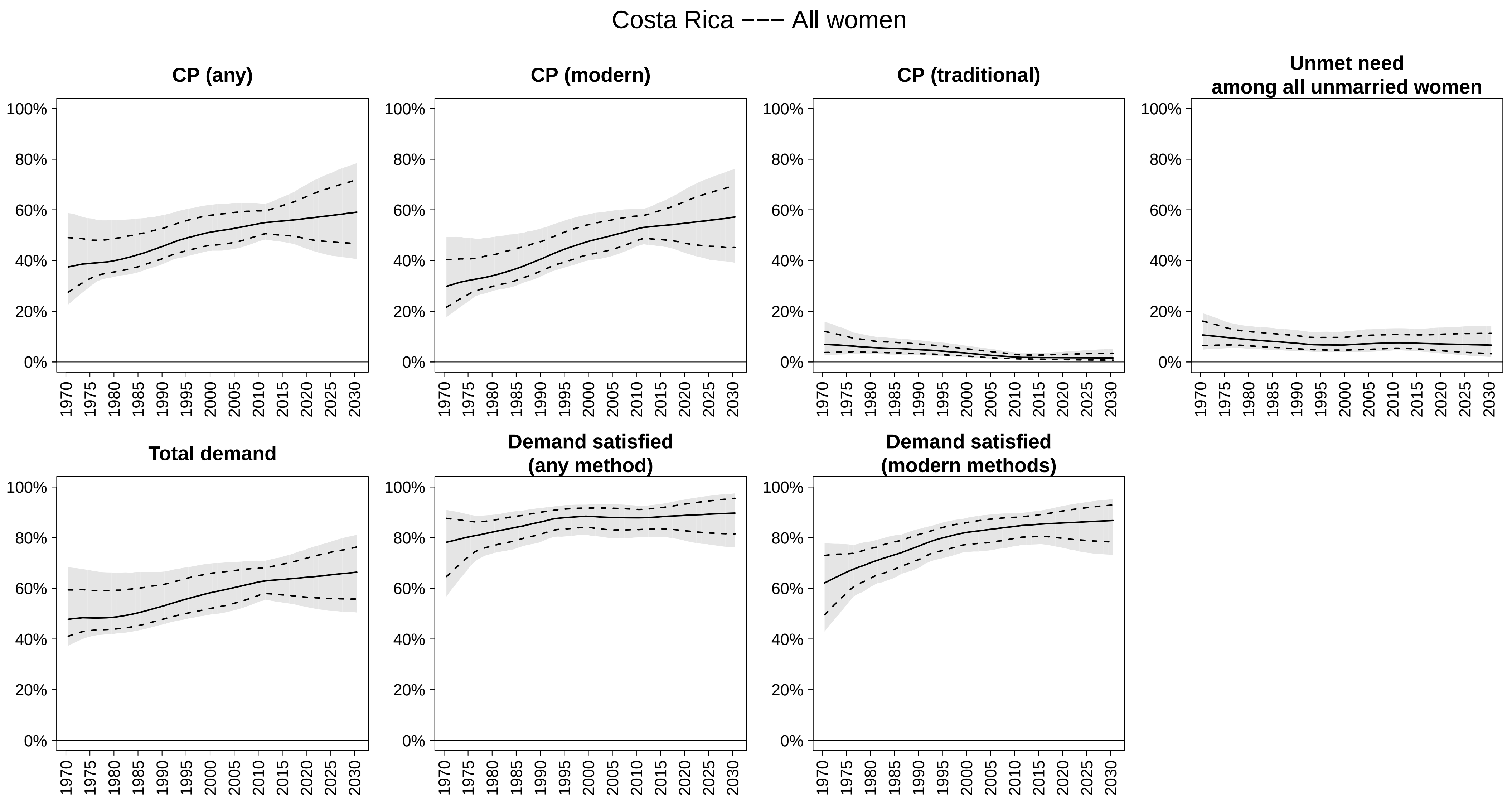
Costa Rica −−− All women
CP (any)
CP (modern)
CP (traditional)
Unmet need among all unmarried women
Total demand
Demand satisfied (any method)
Demand satisfied (modern methods)
0%
20%
40%
60%
80%
100%
1970
1975
1980
1985
1990
1995
2000
2005
2010
2015
2020
2025
2030

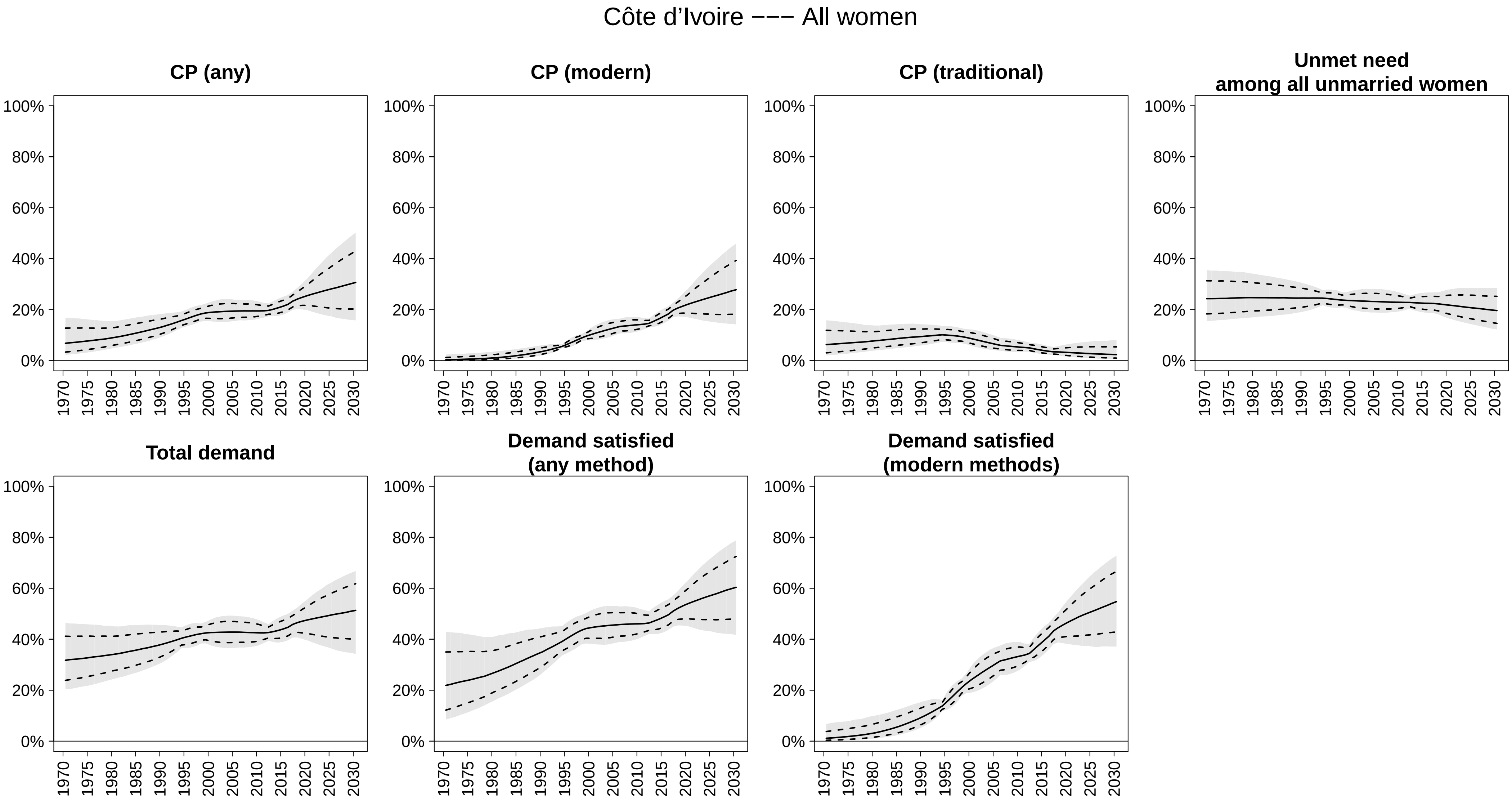
Côte d'Ivoire −−− All women
CP (any)
CP (modern)
CP (traditional)
Unmet need among all unmarried women
Total demand
Demand satisfied (any method)
Demand satisfied (modern methods)
0%
20%
40%
60%
80%
100%
1970
1975
1980
1985
1990
1995
2000
2005
2010
2015
2020
2025
2030

Croatia −−− All women

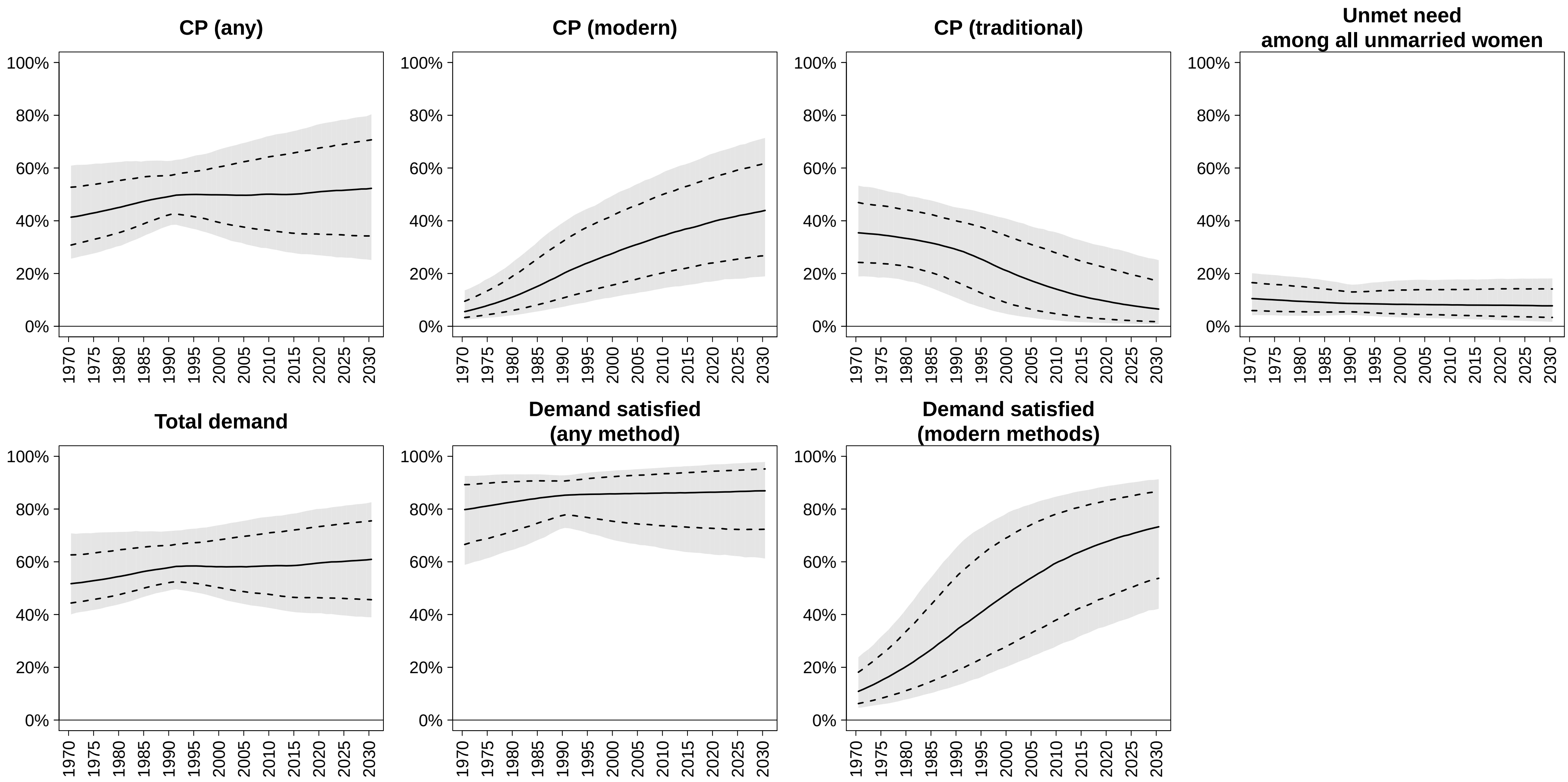

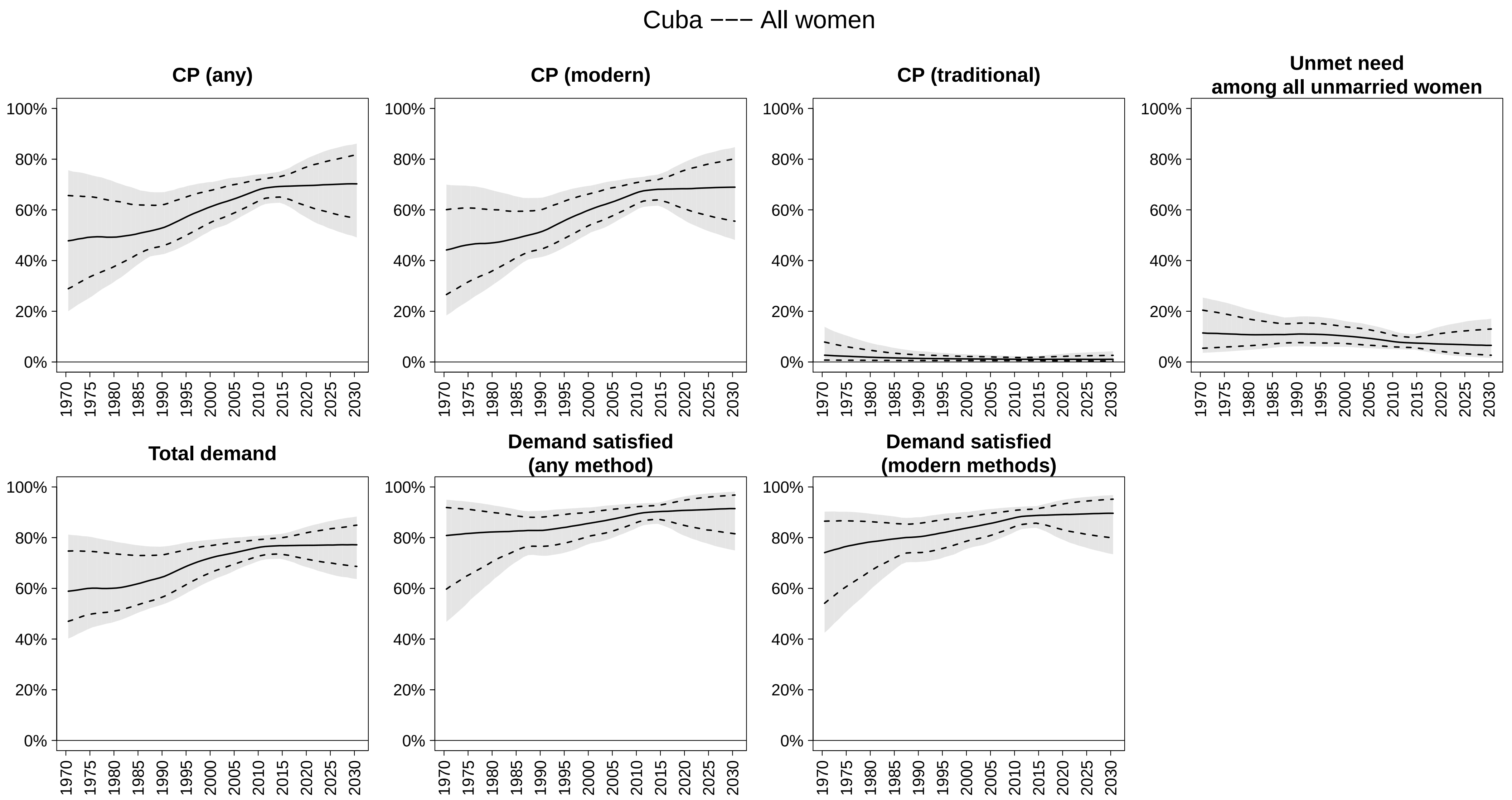
Cuba −−− All women
CP (any)
CP (modern)
CP (traditional)
Unmet need among all unmarried women
Total demand
Demand satisfied (any method)
Demand satisfied (modern methods)
0%
20%
40%
60%
80%
100%
1970
1975
1980
1985
1990
1995
2000
2005
2010
2015
2020
2025
2030

Czechia −−− All women

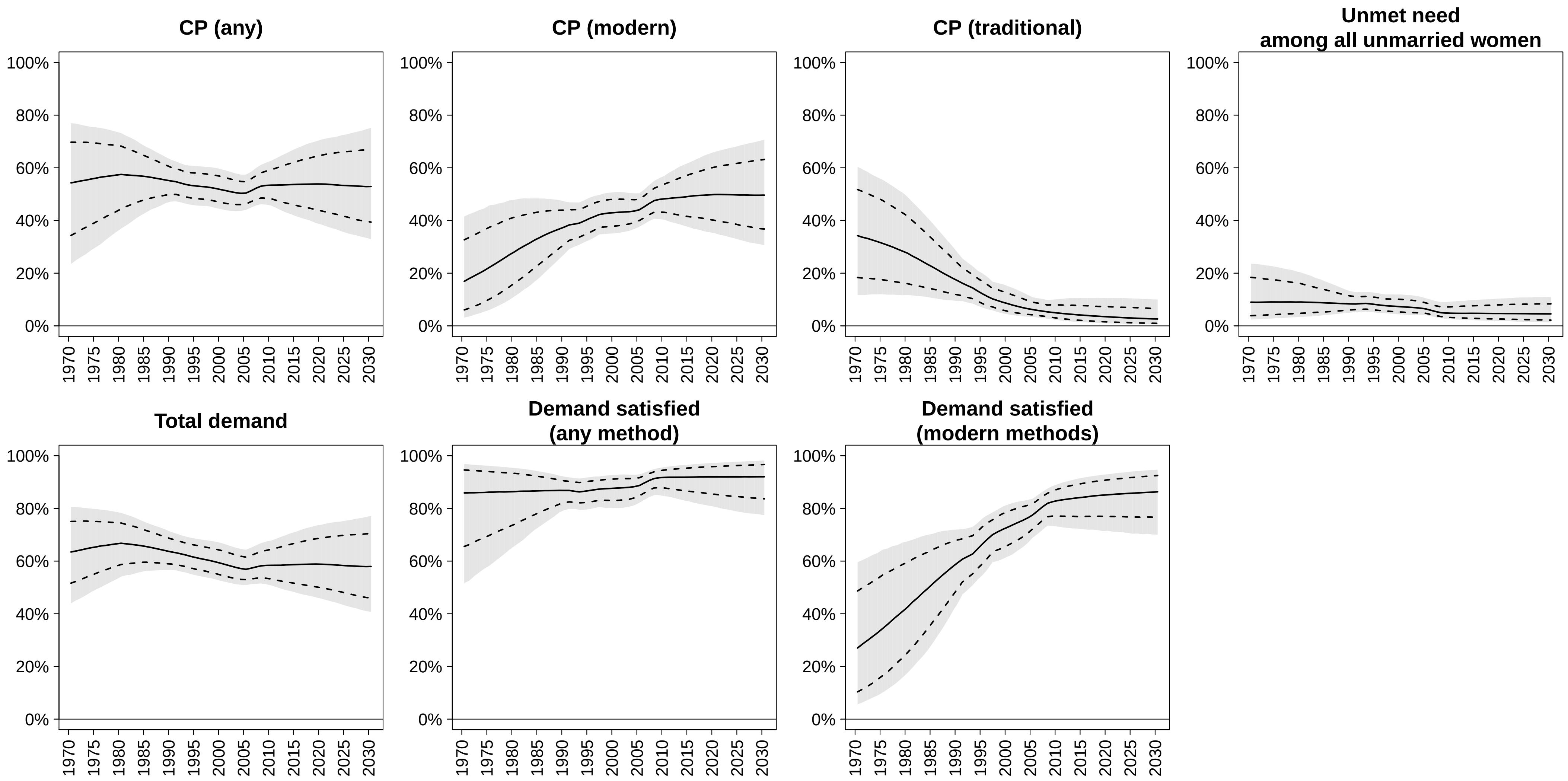

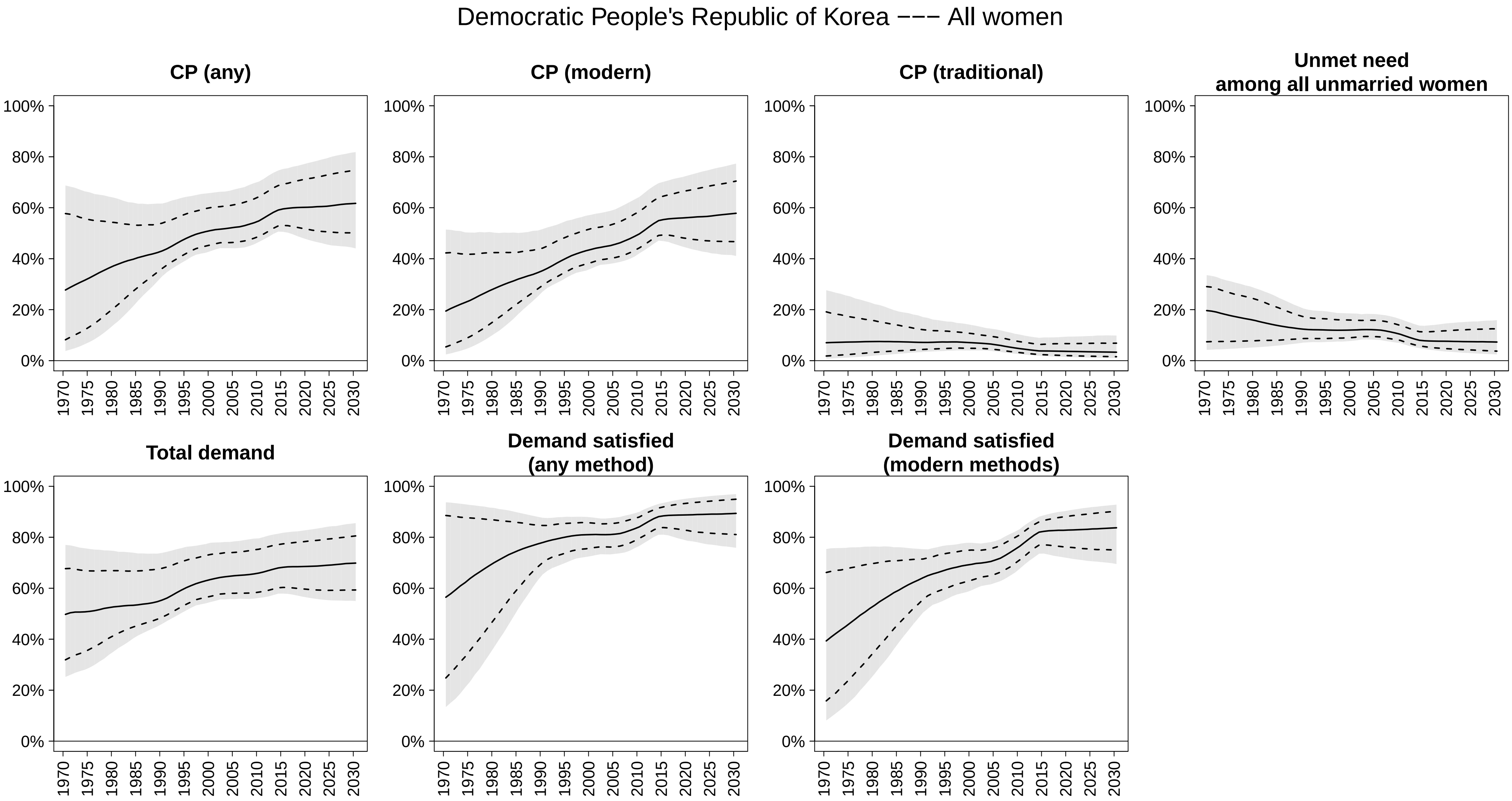
Democratic People's Republic of Korea −−− All women
CP (any)
CP (modern)
CP (traditional)
Unmet need among all unmarried women
Total demand
Demand satisfied (any method)
Demand satisfied (modern methods)
100%
80%
60%
40%
20%
0%
1970
1975
1980
1985
1990
1995
2000
2005
2010
2015
2020
2025
2030

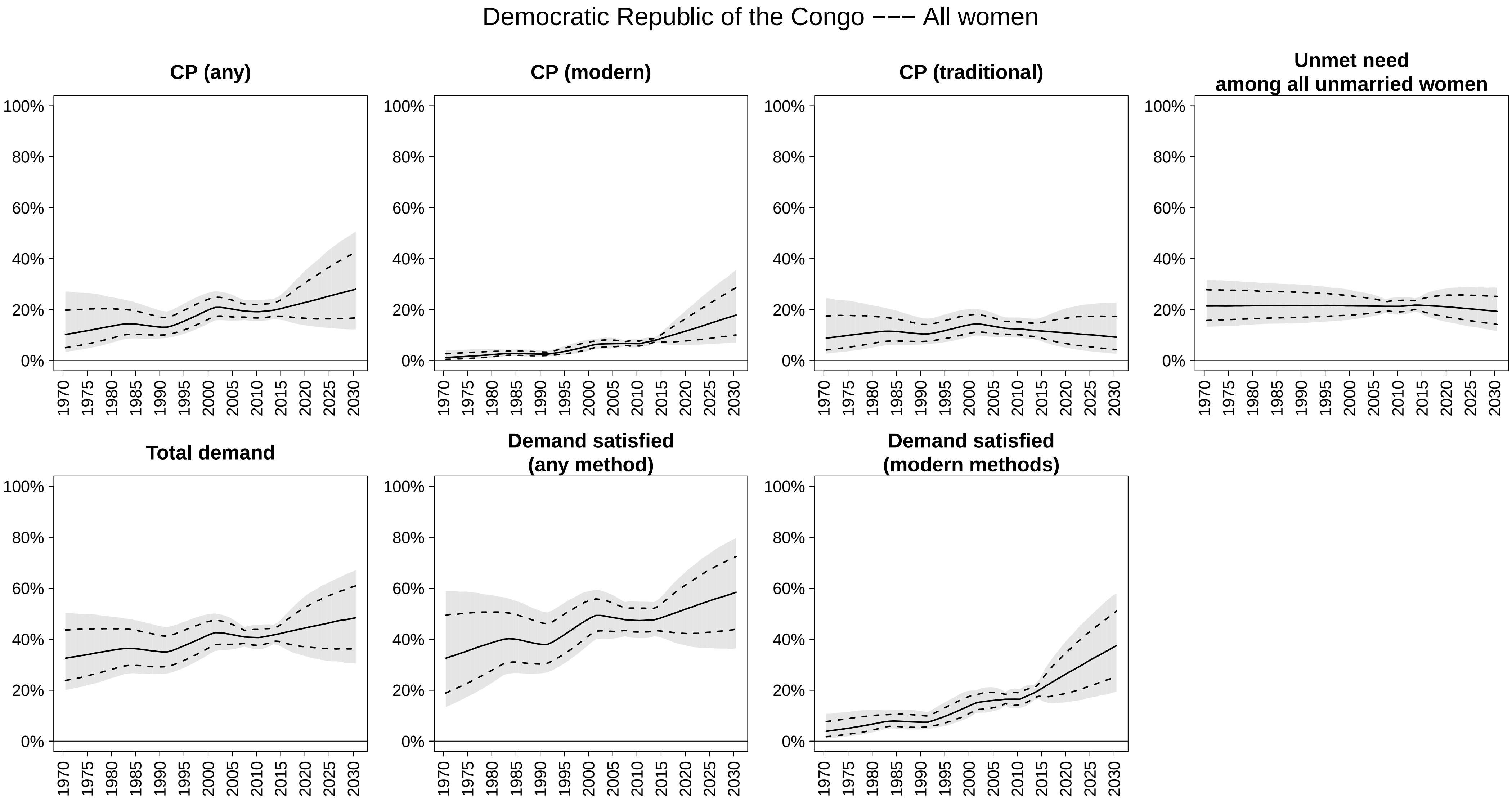
Democratic Republic of the Congo −−− All women
CP (any)
CP (modern)
CP (traditional)
Unmet need
among all unmarried women
Total demand
Demand satisfied
(any method)
Demand satisfied
(modern methods)
0%
20%
40%
60%
80%
100%
1970
1975
1980
1985
1990
1995
2000
2005
2010
2015
2020
2025
2030

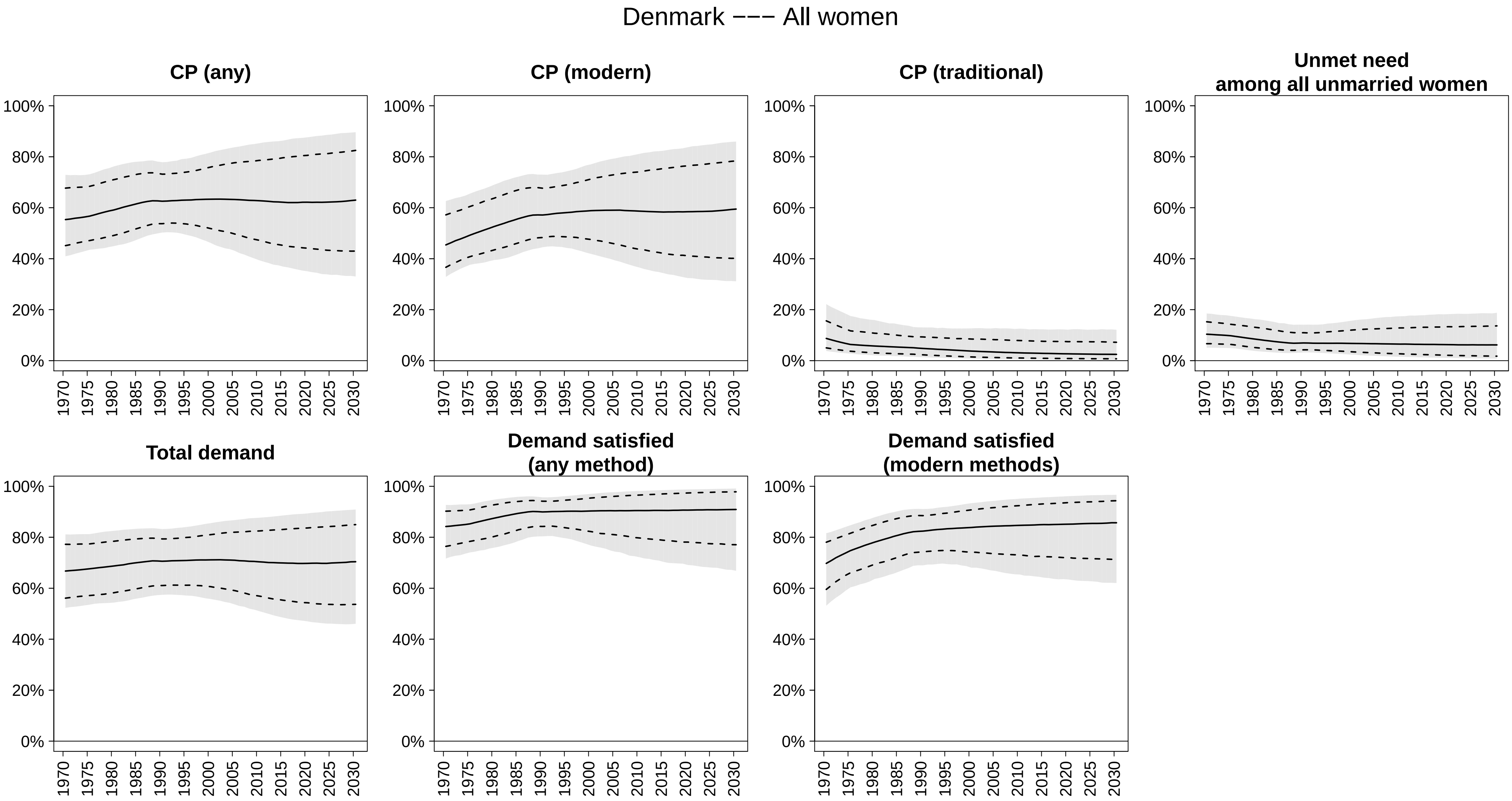
Denmark −−− All women
CP (any)
CP (modern)
CP (traditional)
Unmet need
among all unmarried women
Total demand
Demand satisfied
(any method)
Demand satisfied
(modern methods)
0%
20%
40%
60%
80%
100%
1970
1975
1980
1985
1990
1995
2000
2005
2010
2015
2020
2025
2030

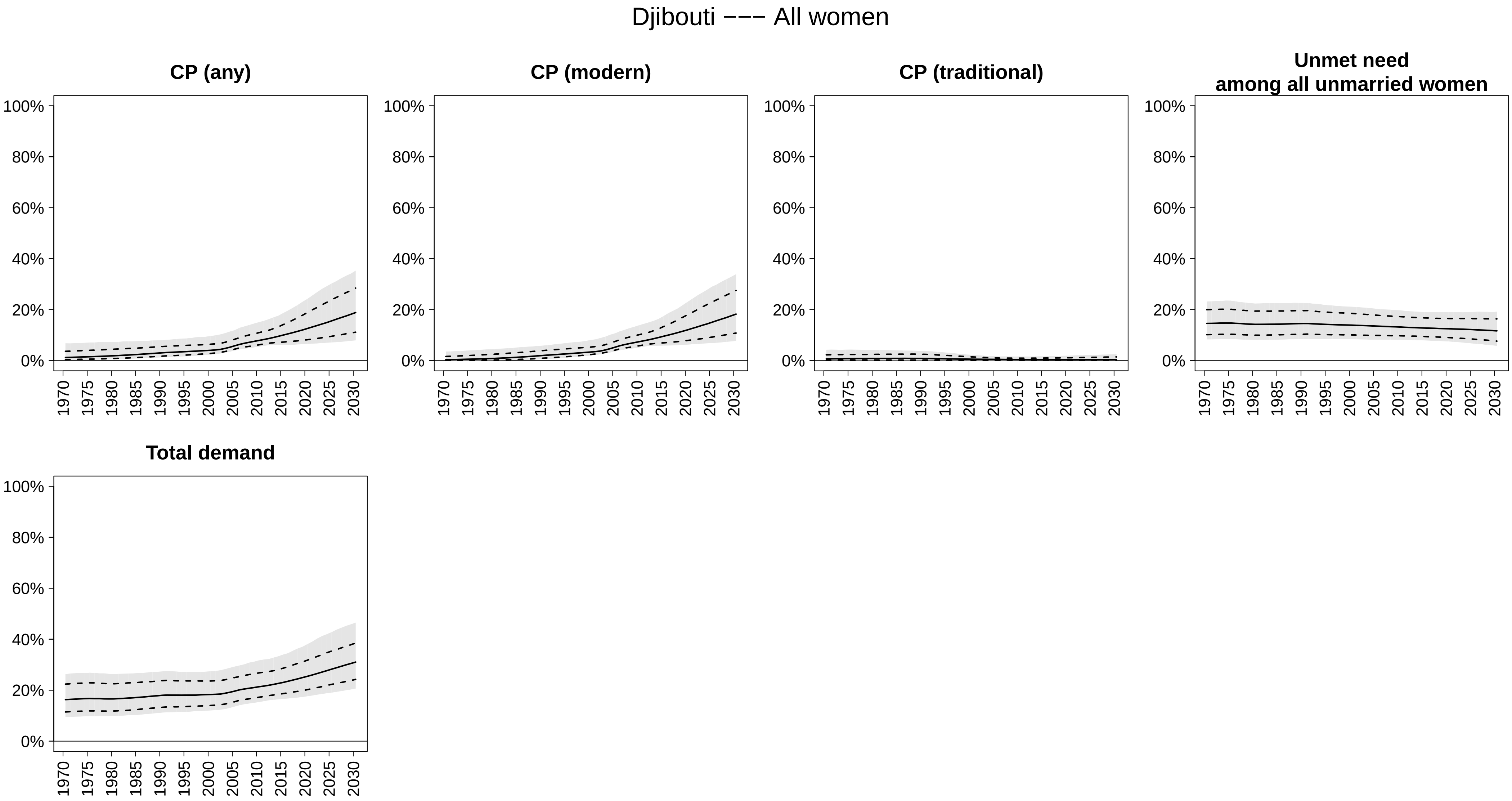
Djibouti −−− All women
CP (any)
CP (modern)
CP (traditional)
Unmet need
among all unmarried women
Total demand
100%
80%
60%
40%
20%
0%
1970
1975
1980
1985
1990
1995
2000
2005
2010
2015
2020
2025
2030

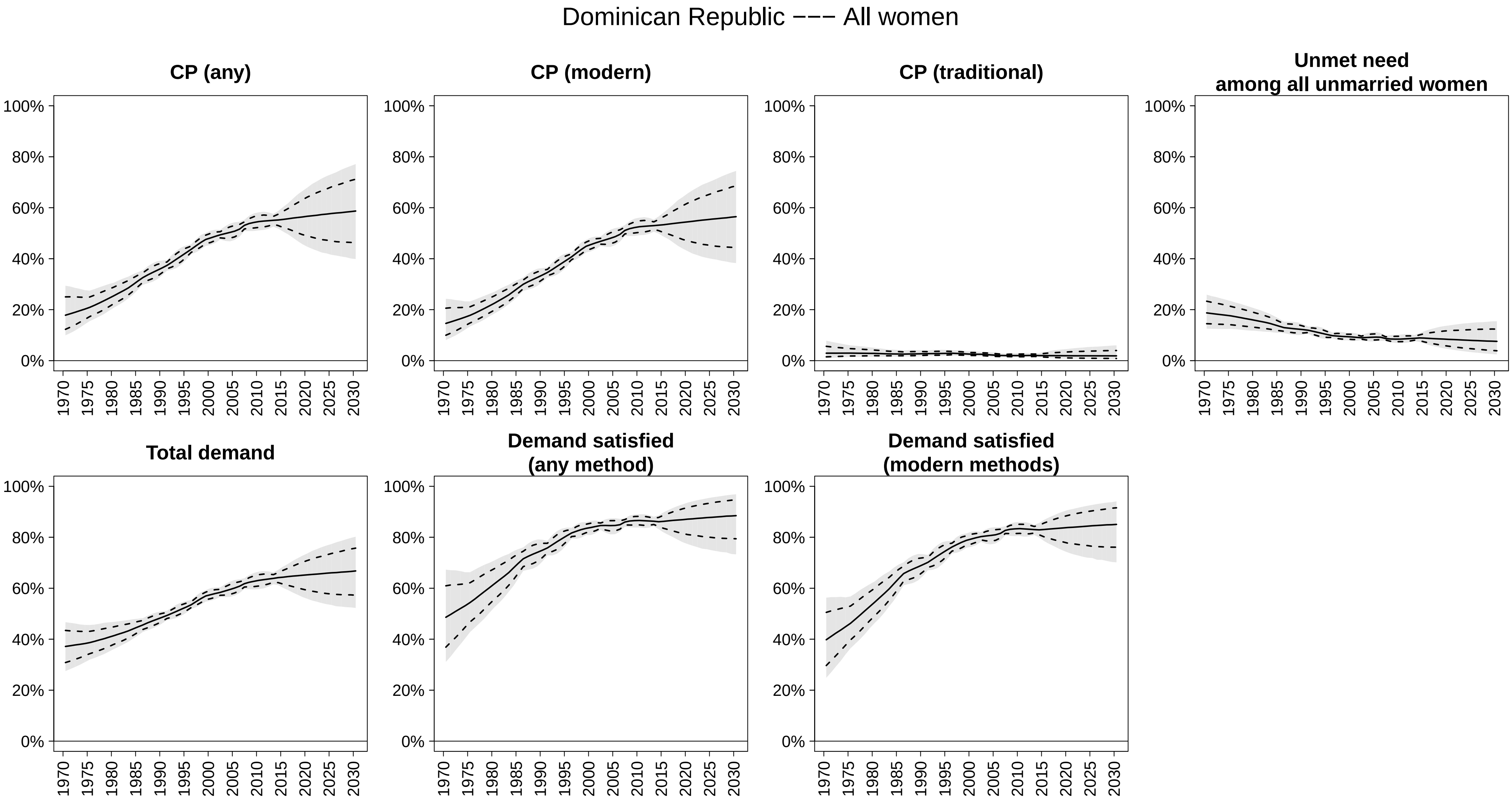
Dominican Republic −−− All women
CP (any)
CP (modern)
CP (traditional)
Unmet need
among all unmarried women
Total demand
Demand satisfied
(any method)
Demand satisfied
(modern methods)
0%
20%
40%
60%
80%
100%
1970
1975
1980
1985
1990
1995
2000
2005
2010
2015
2020
2025
2030

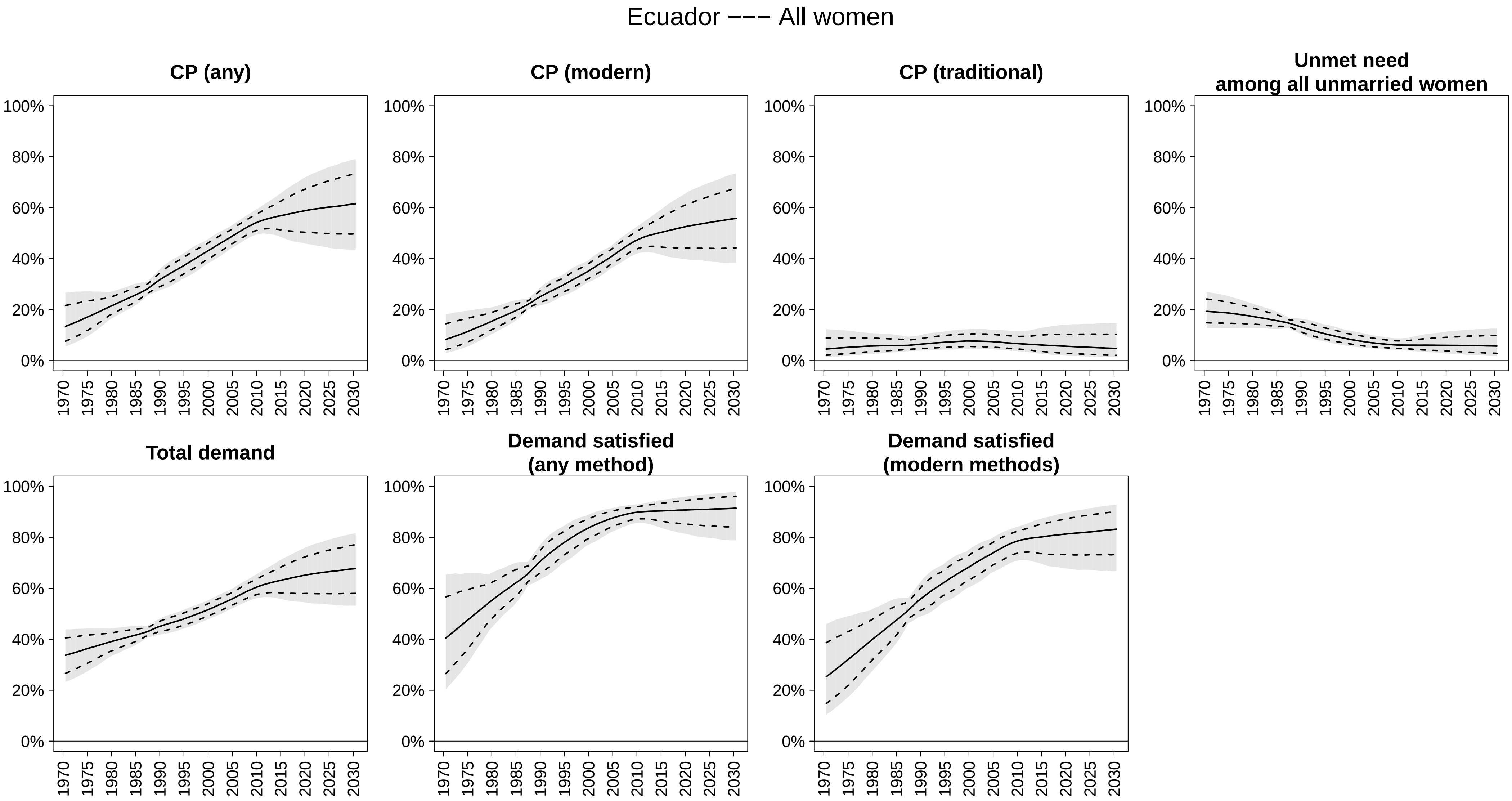

Ecuador −−− All women
CP (any)
CP (modern)
CP (traditional)
Unmet need among all unmarried women
Total demand
Demand satisfied (any method)
Demand satisfied (modern methods)
0%
20%
40%
60%
80%
100%
1970
1975
1980
1985
1990
1995
2000
2005
2010
2015
2020
2025
2030

Egypt −−− All women

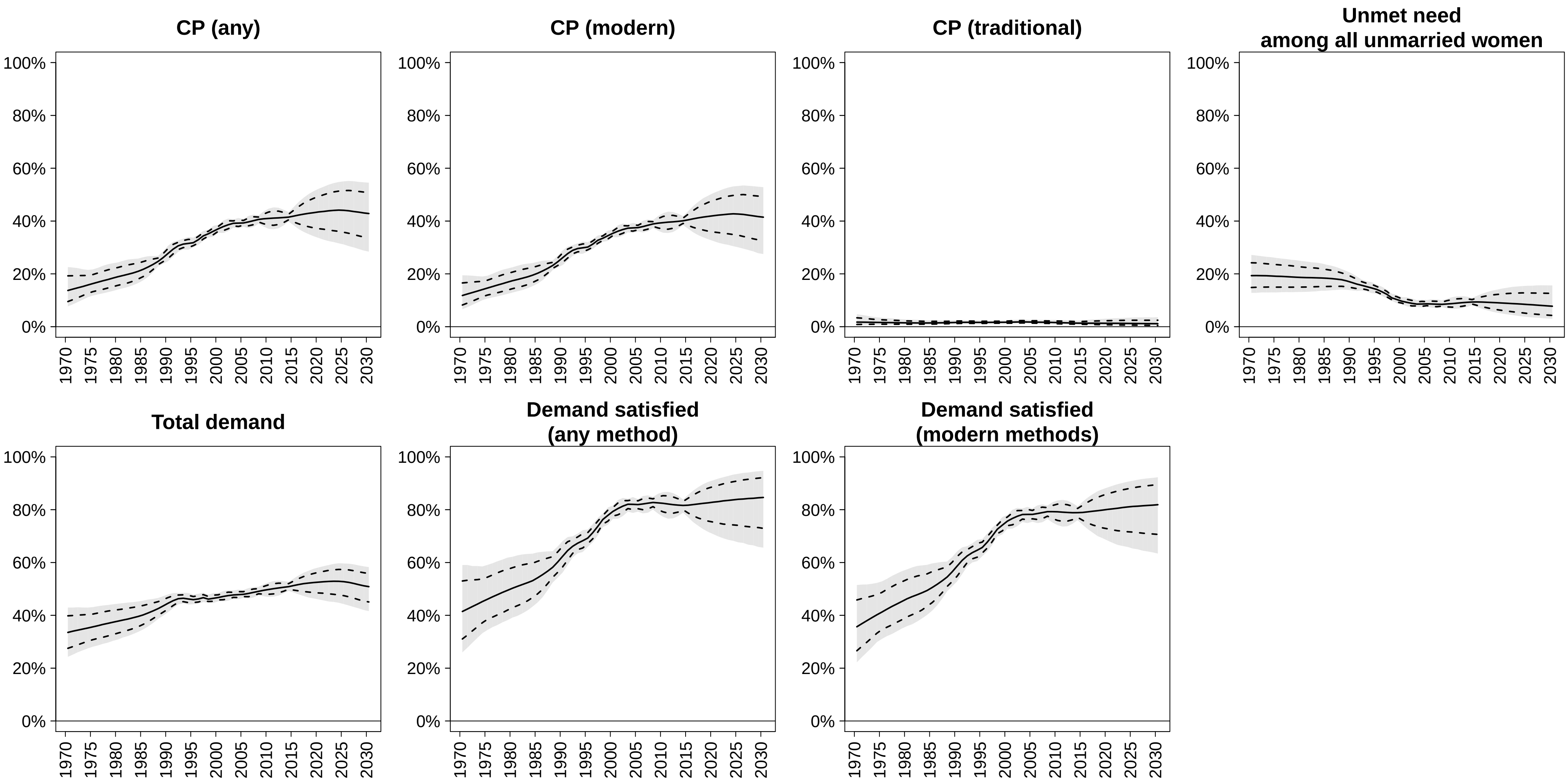

El Salvador −−− All women

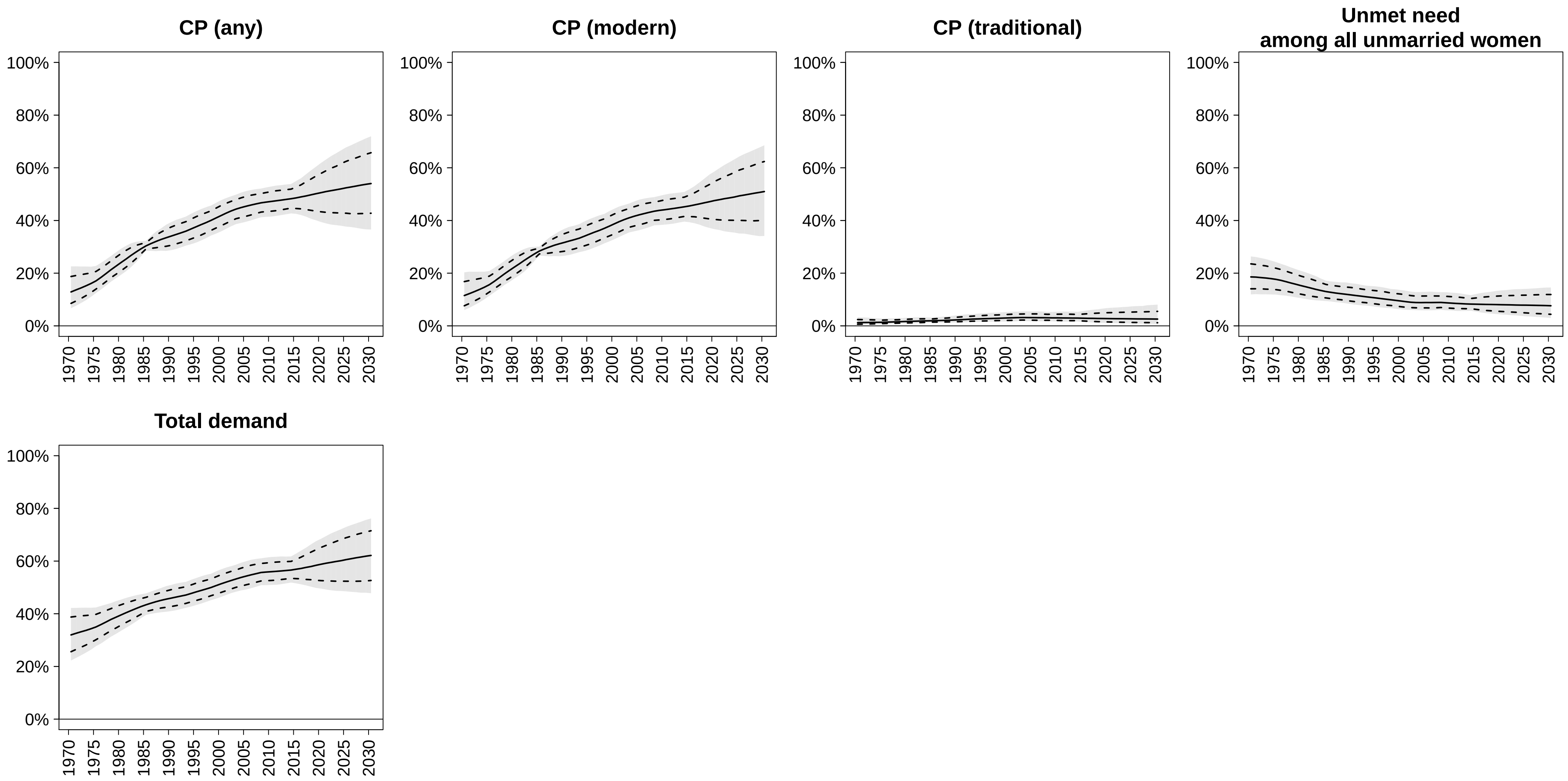

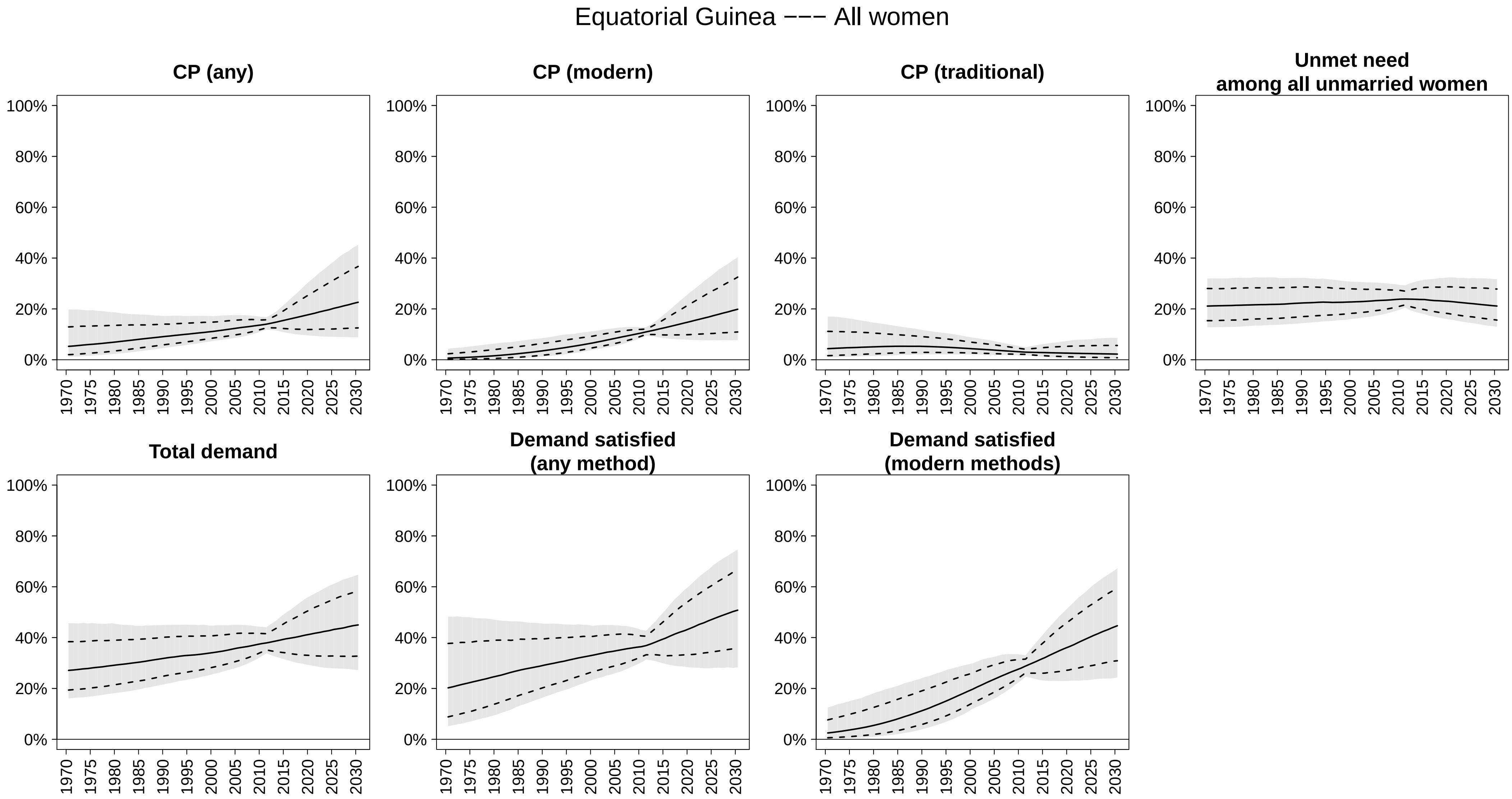
Equatorial Guinea --- All women
CP (any)
CP (modern)
CP (traditional)
Unmet need among all unmarried women
Total demand
Demand satisfied (any method)
Demand satisfied (modern methods)
0%
20%
40%
60%
80%
100%
1970
1975
1980
1985
1990
1995
2000
2005
2010
2015
2020
2025
2030

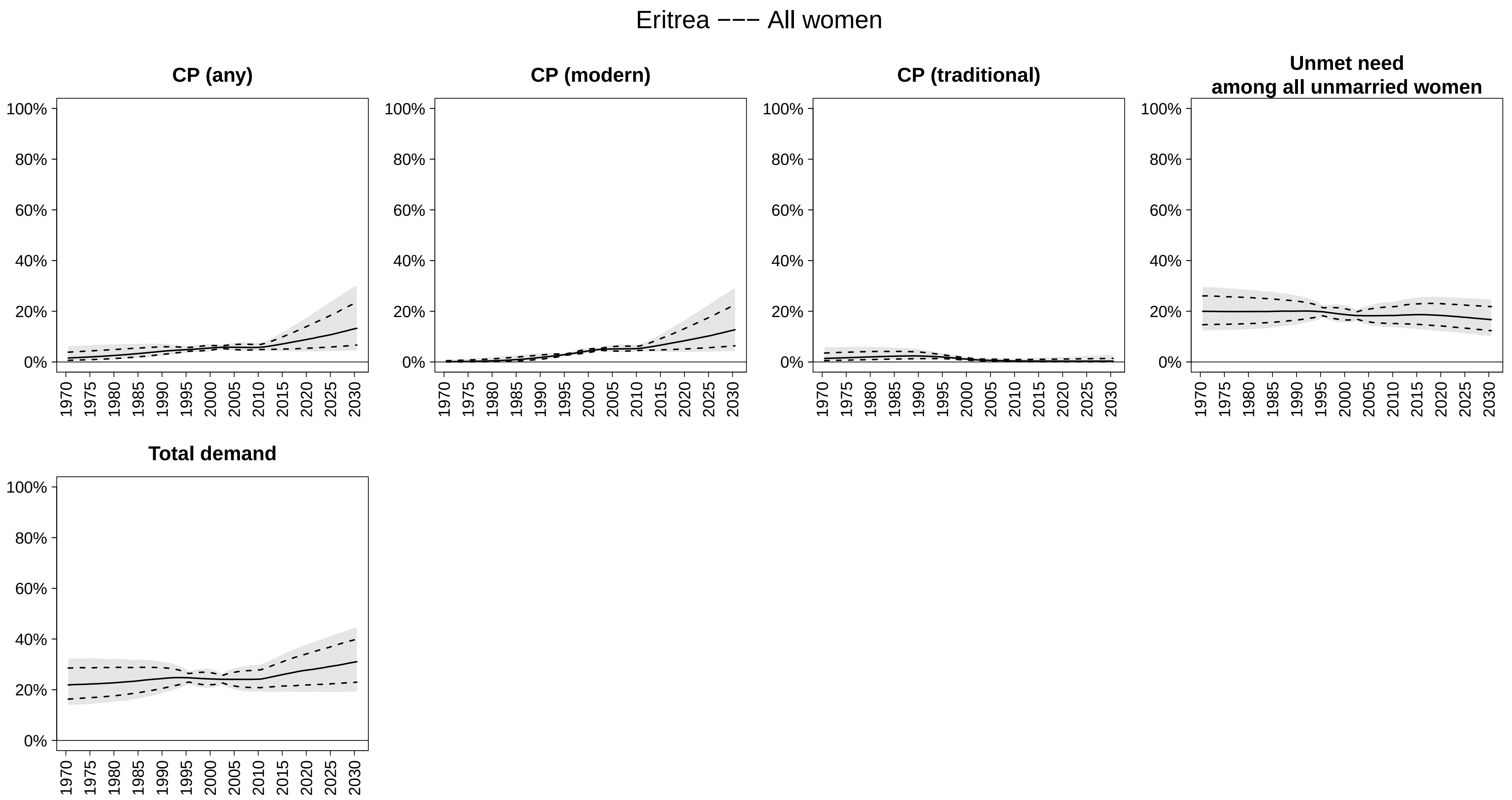
Eritrea −−− All women
CP (any)
CP (modern)
CP (traditional)
Unmet need among all unmarried women
Total demand
0%
20%
40%
60%
80%
100%
1970
1975
1980
1985
1990
1995
2000
2005
2010
2015
2020
2025
2030

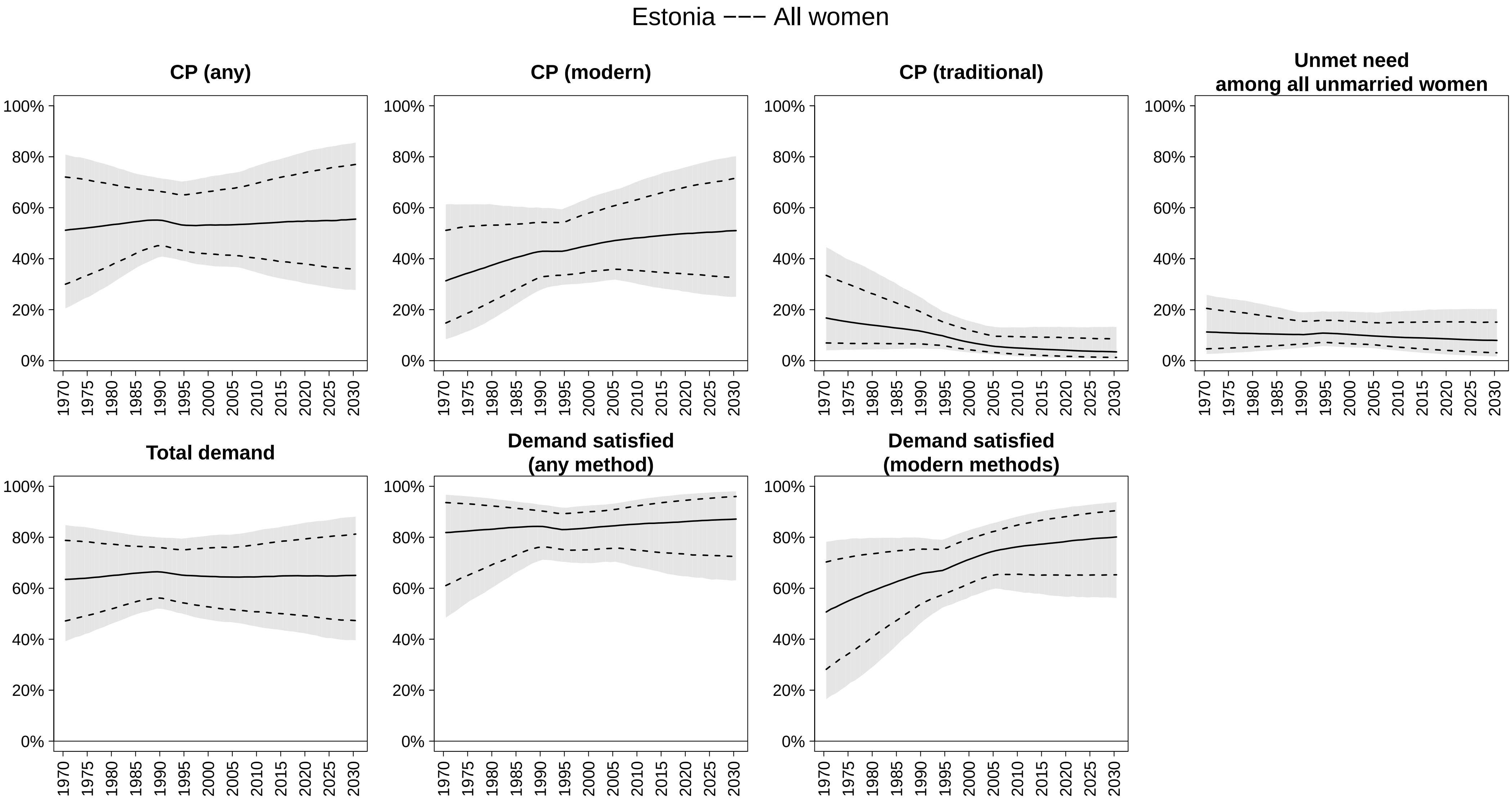
Estonia −−− All women
CP (any)
CP (modern)
CP (traditional)
Unmet need
among all unmarried women
Total demand
Demand satisfied
(any method)
Demand satisfied
(modern methods)
100%
80%
60%
40%
20%
0%
1970
1975
1980
1985
1990
1995
2000
2005
2010
2015
2020
2025
2030

Eswatini −−− All women

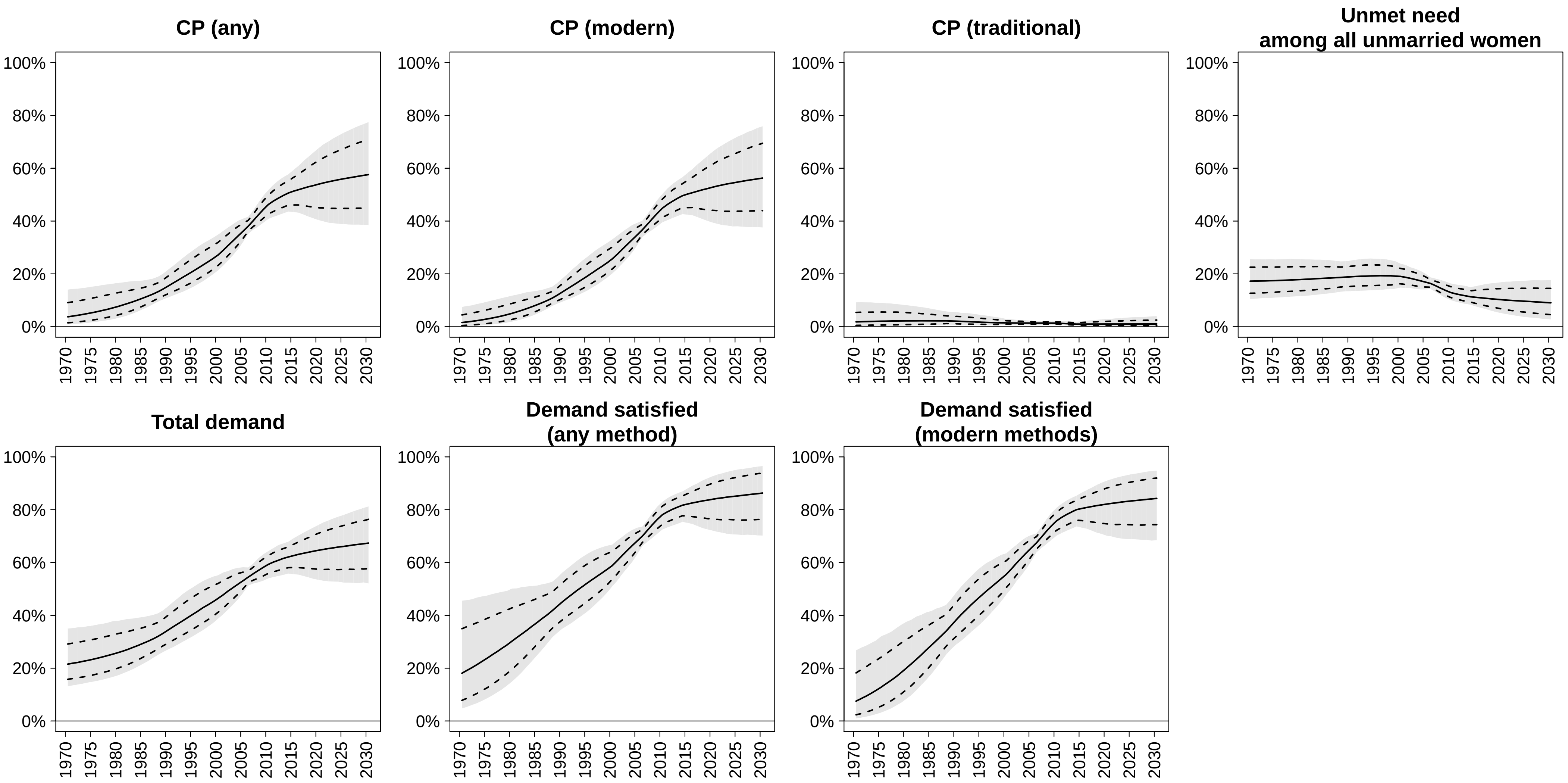

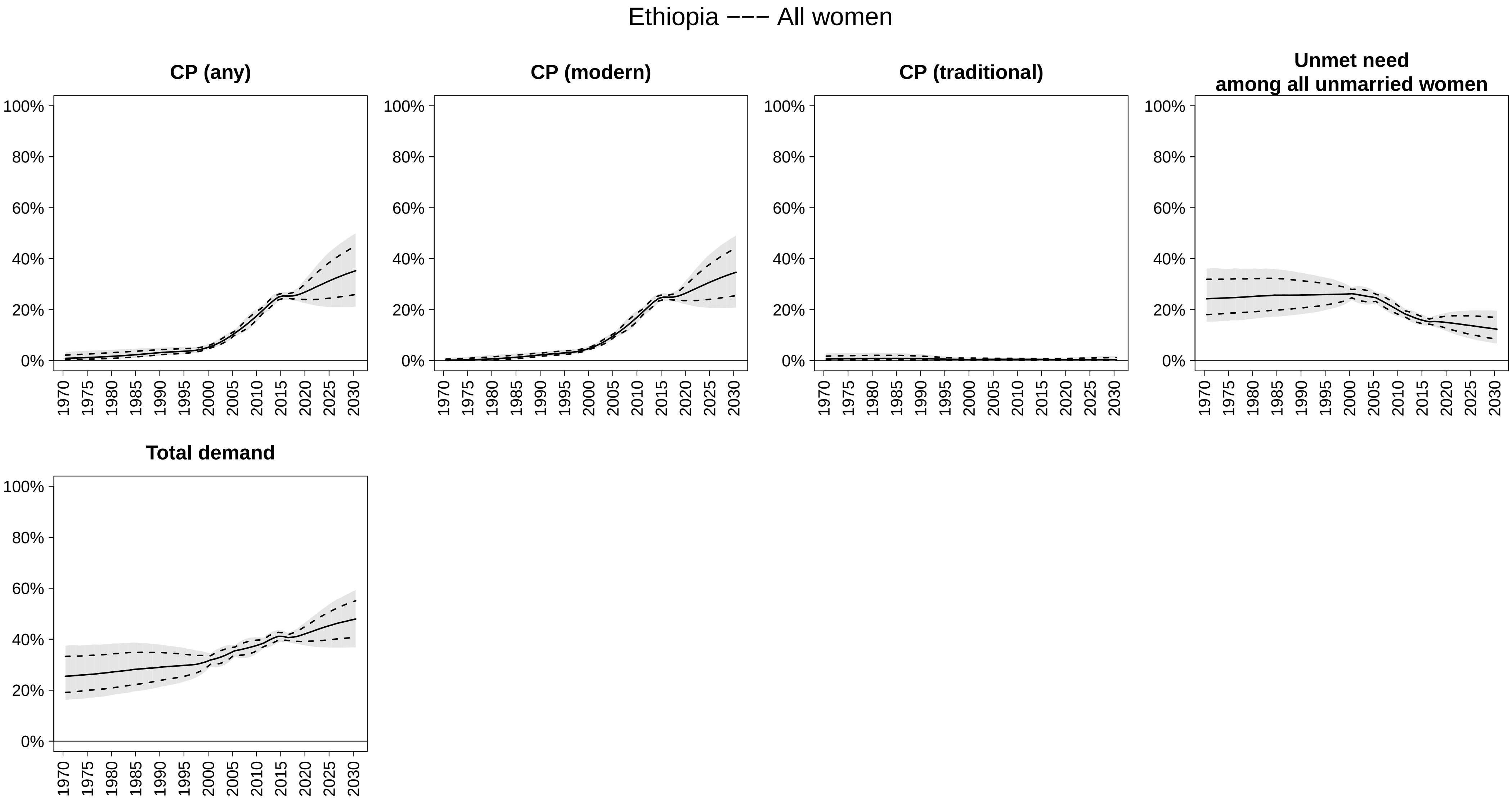
Ethiopia −−− All women
CP (any)
CP (modern)
CP (traditional)
Unmet need
among all unmarried women
Total demand
100%
80%
60%
40%
20%
0%
1970
1975
1980
1985
1990
1995
2000
2005
2010
2015
2020
2025
2030

Fiji −−− All women

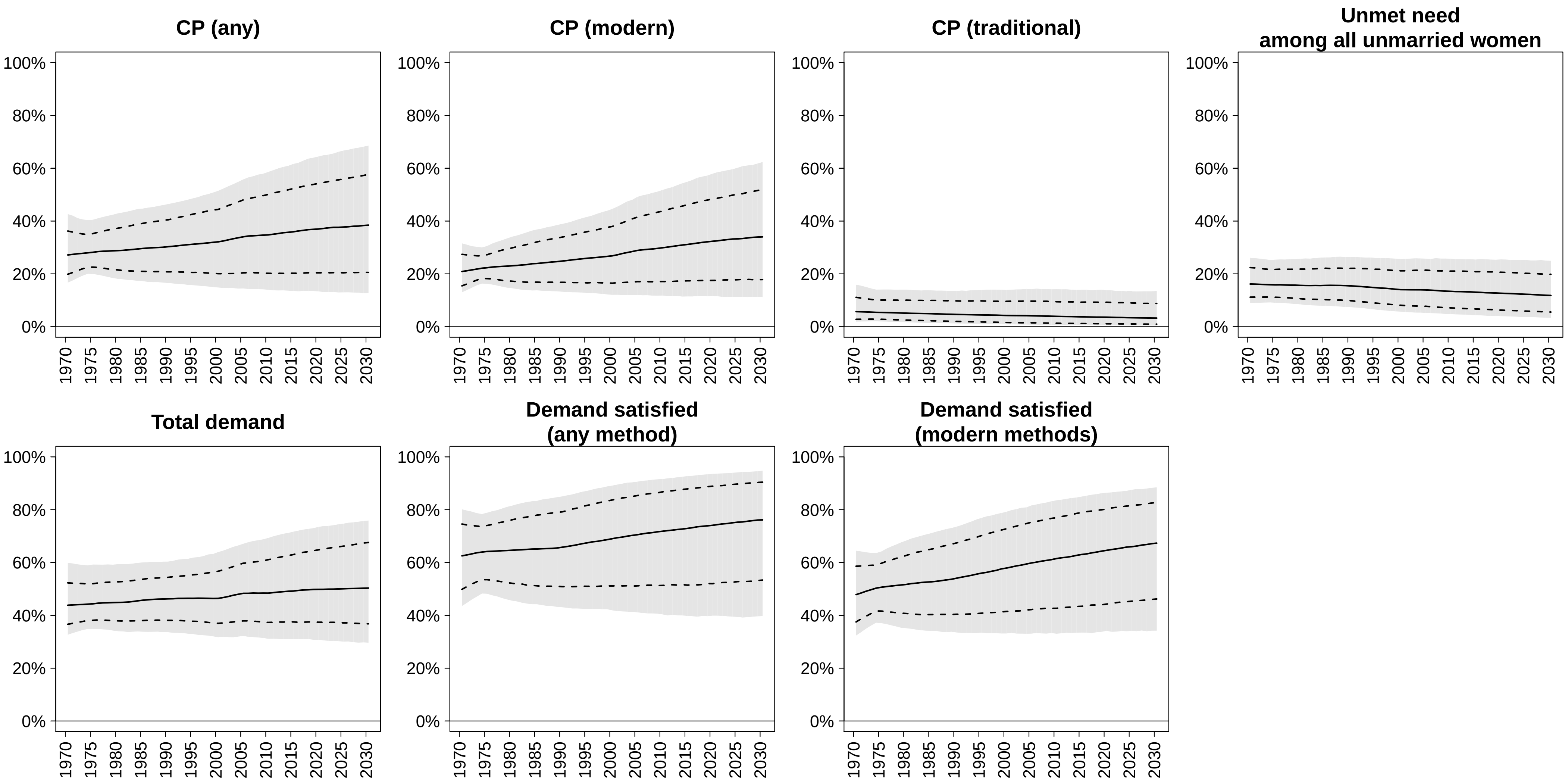

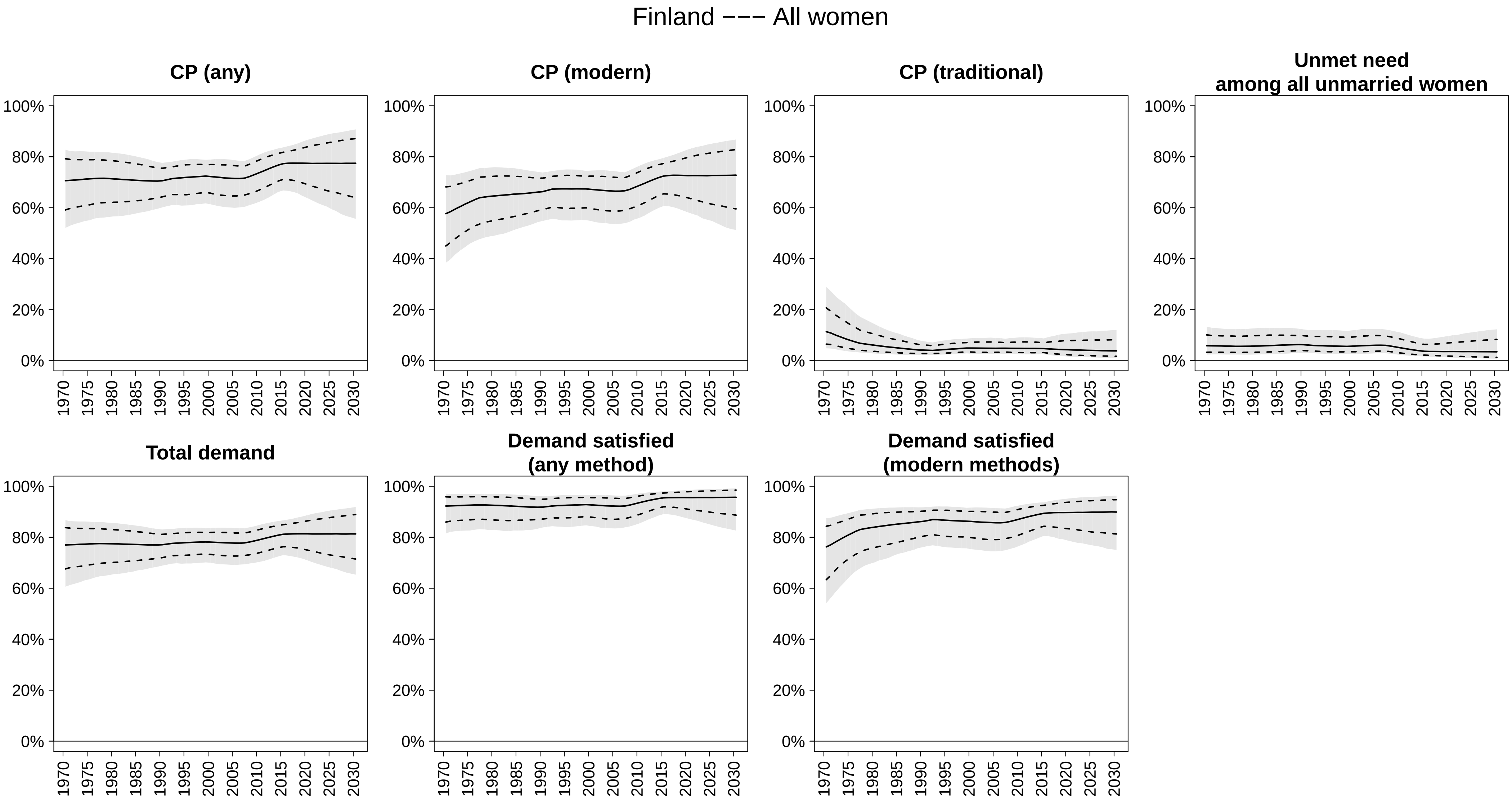
Finland --- All women
CP (any)
CP (modern)
CP (traditional)
Unmet need among all unmarried women
Total demand
Demand satisfied (any method)
Demand satisfied (modern methods)
0%
20%
40%
60%
80%
100%
1970
1975
1980
1985
1990
1995
2000
2005
2010
2015
2020
2025
2030

France −−− All women

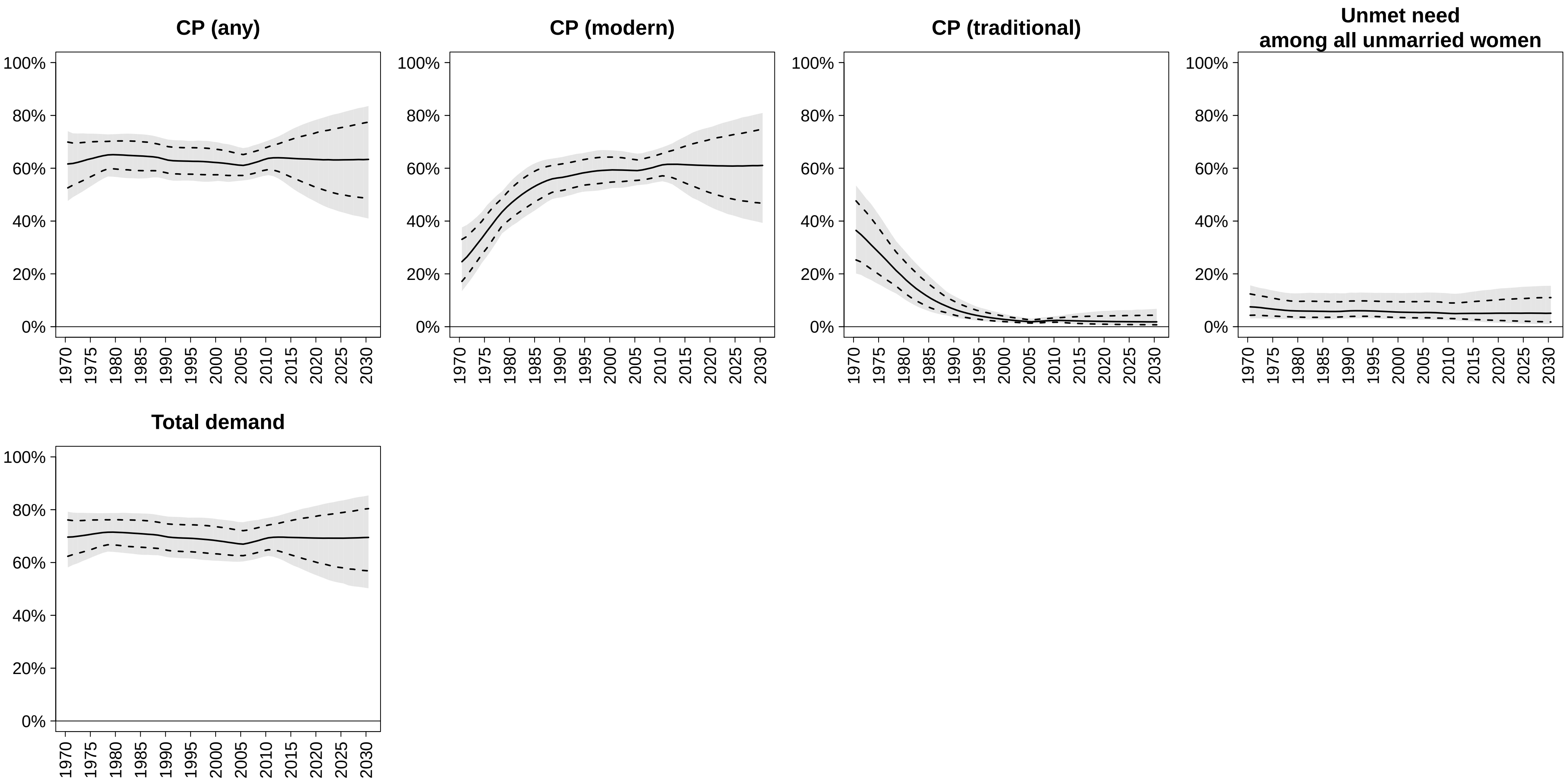

Gabon −−− All women

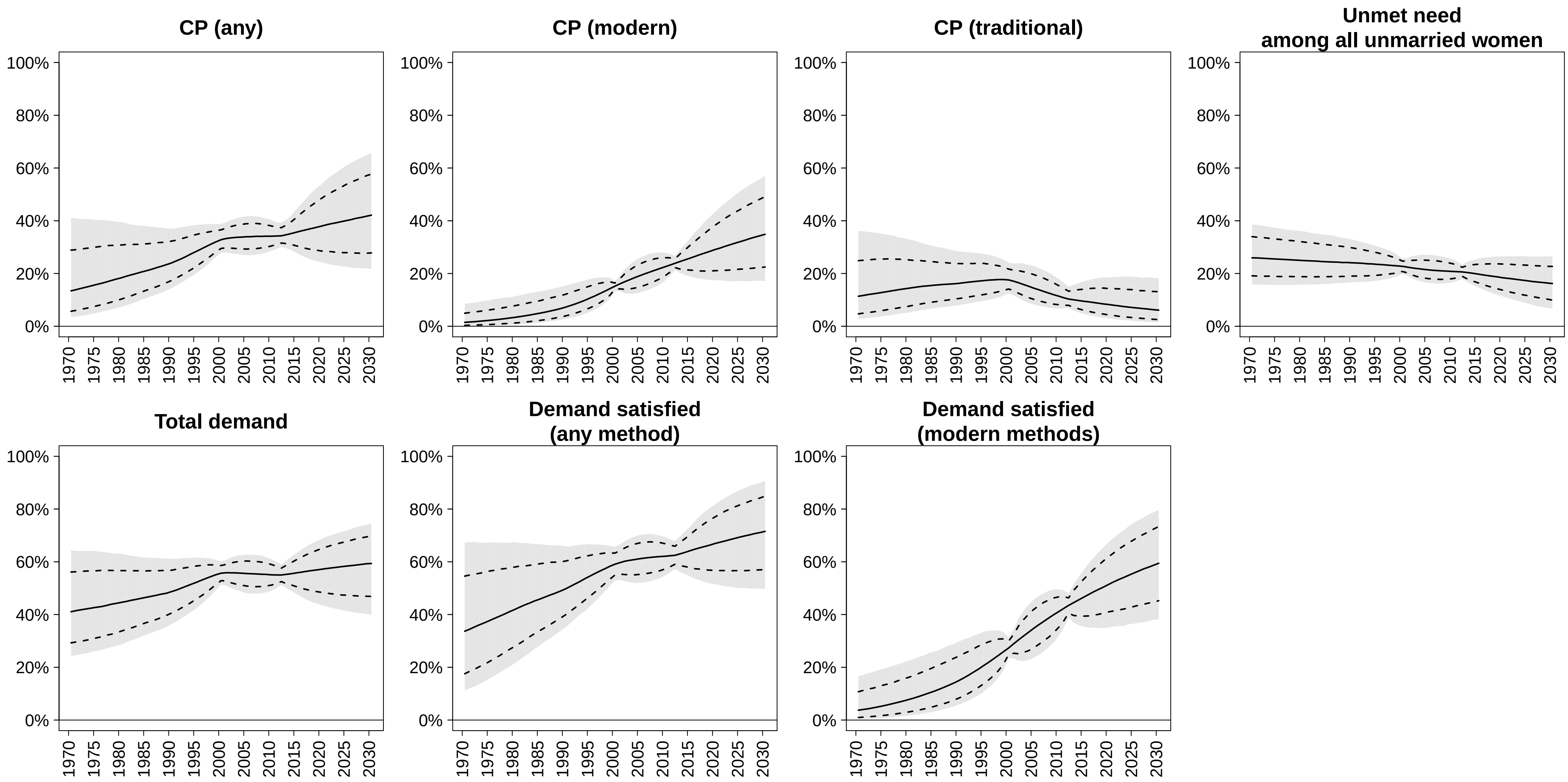

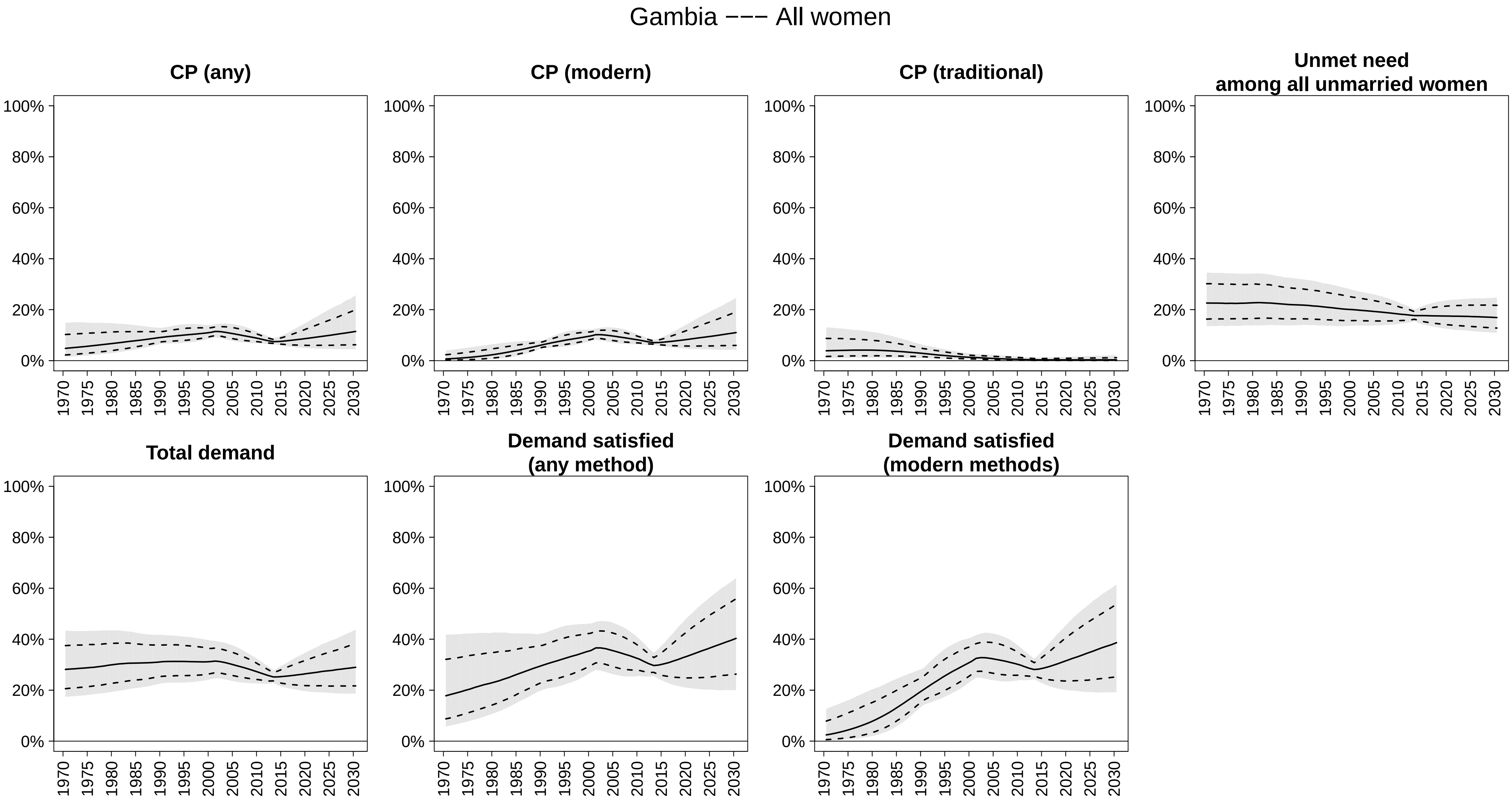
Gambia −−− All women
CP (any)
CP (modern)
CP (traditional)
Unmet need
among all unmarried women
Total demand
Demand satisfied
(any method)
Demand satisfied
(modern methods)
0%
20%
40%
60%
80%
100%
1970
1975
1980
1985
1990
1995
2000
2005
2010
2015
2020
2025
2030

Georgia −−− All women

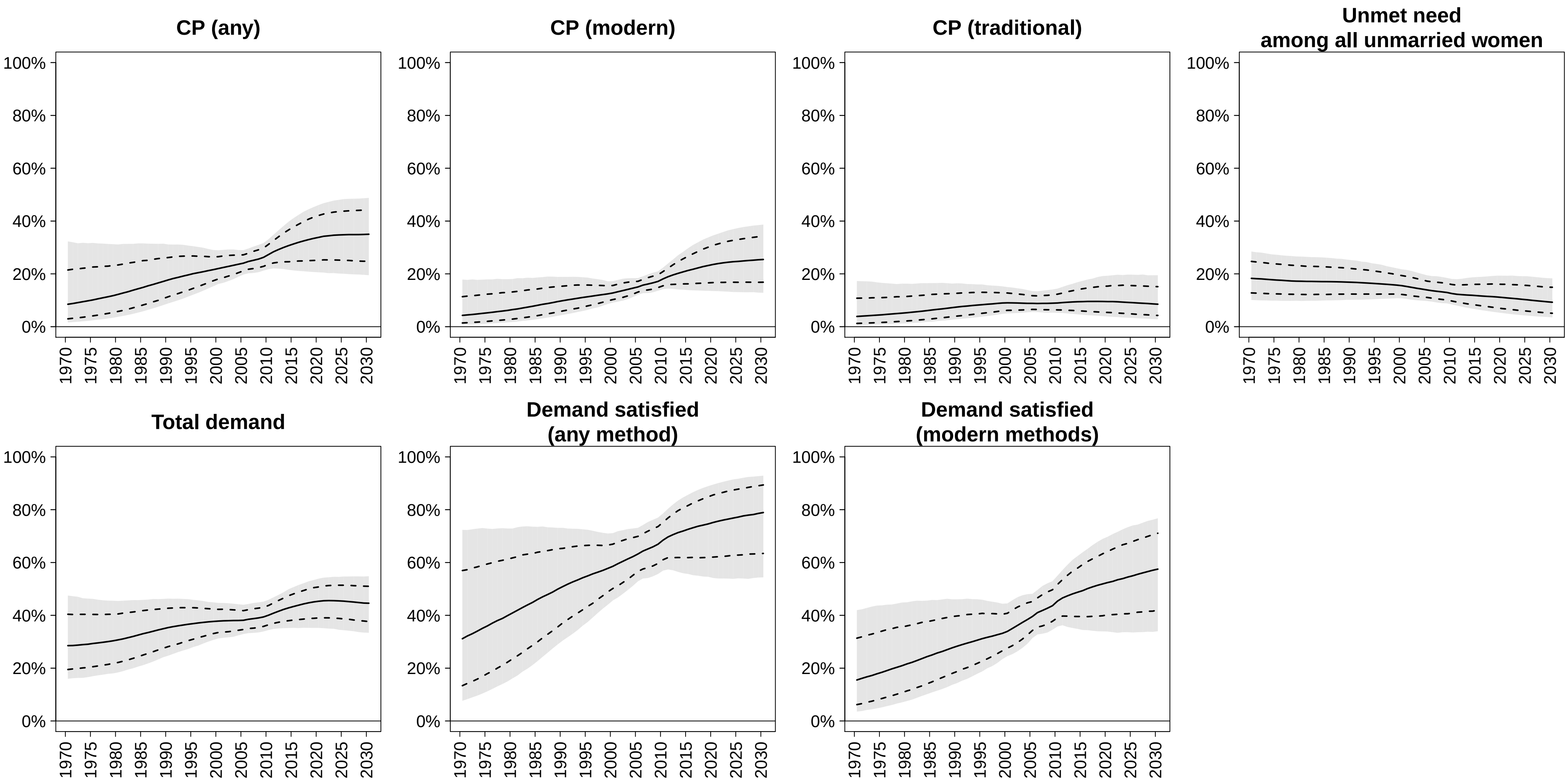

Germany −−− All women

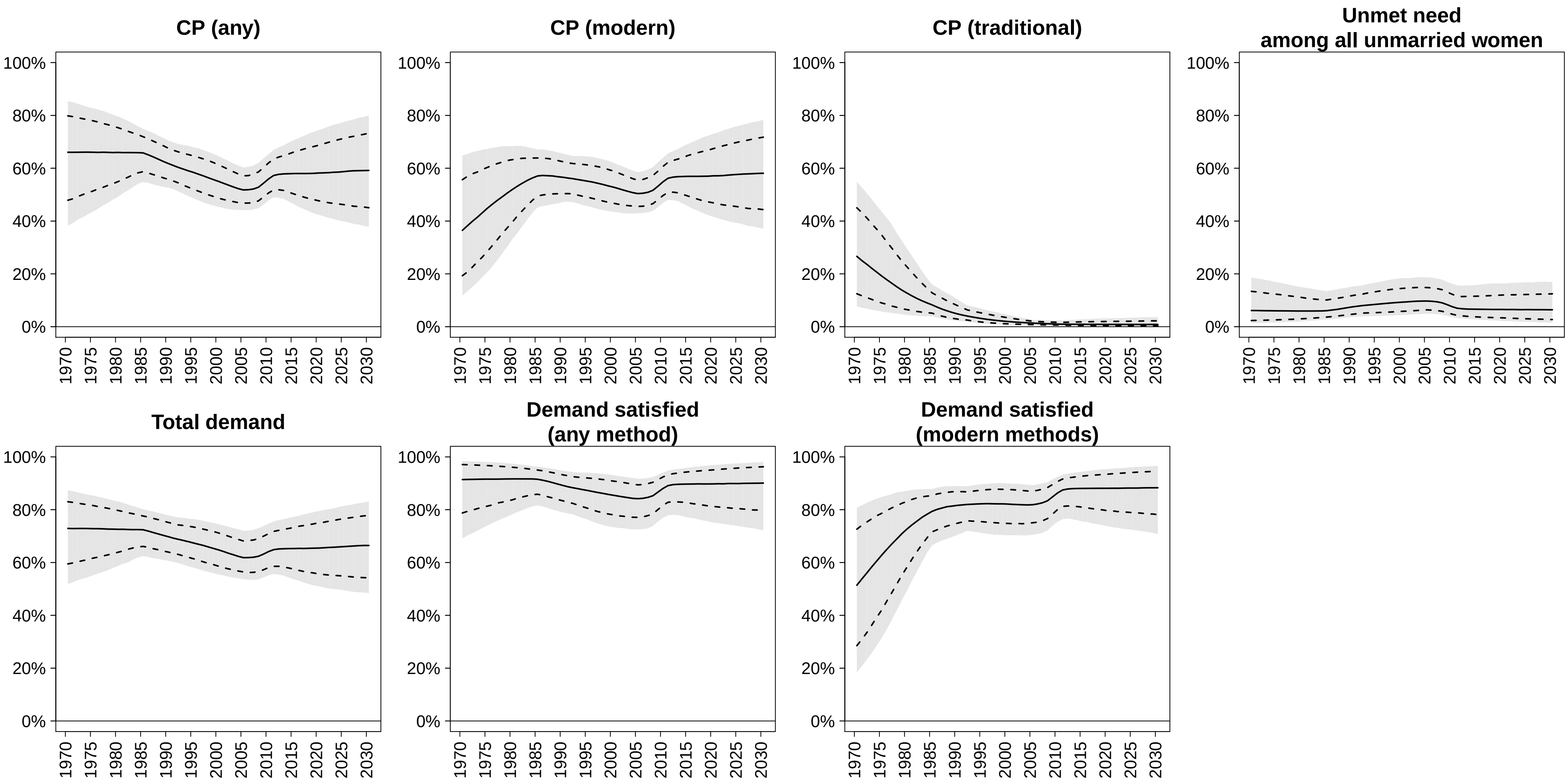

Ghana −−− All women

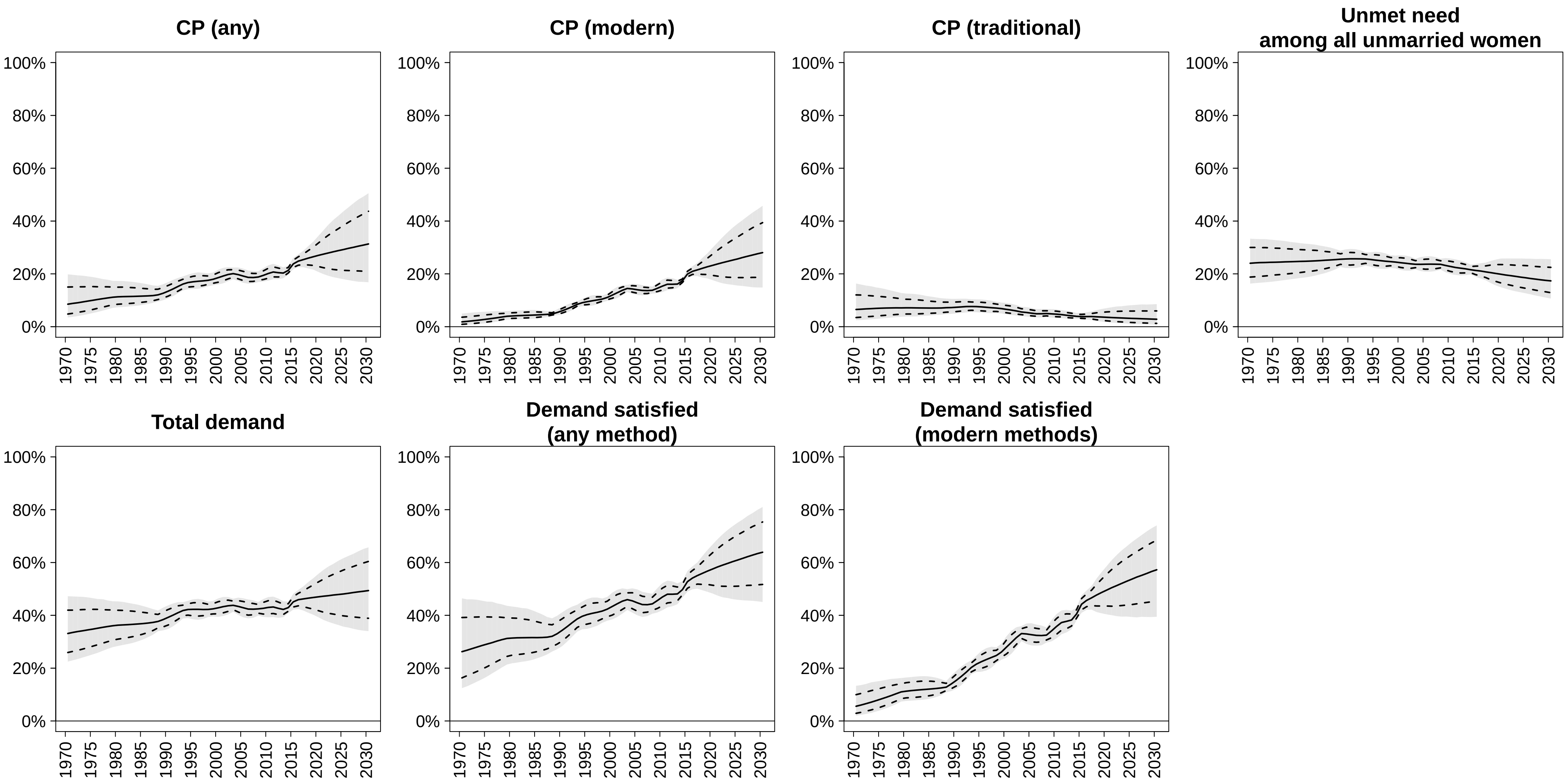

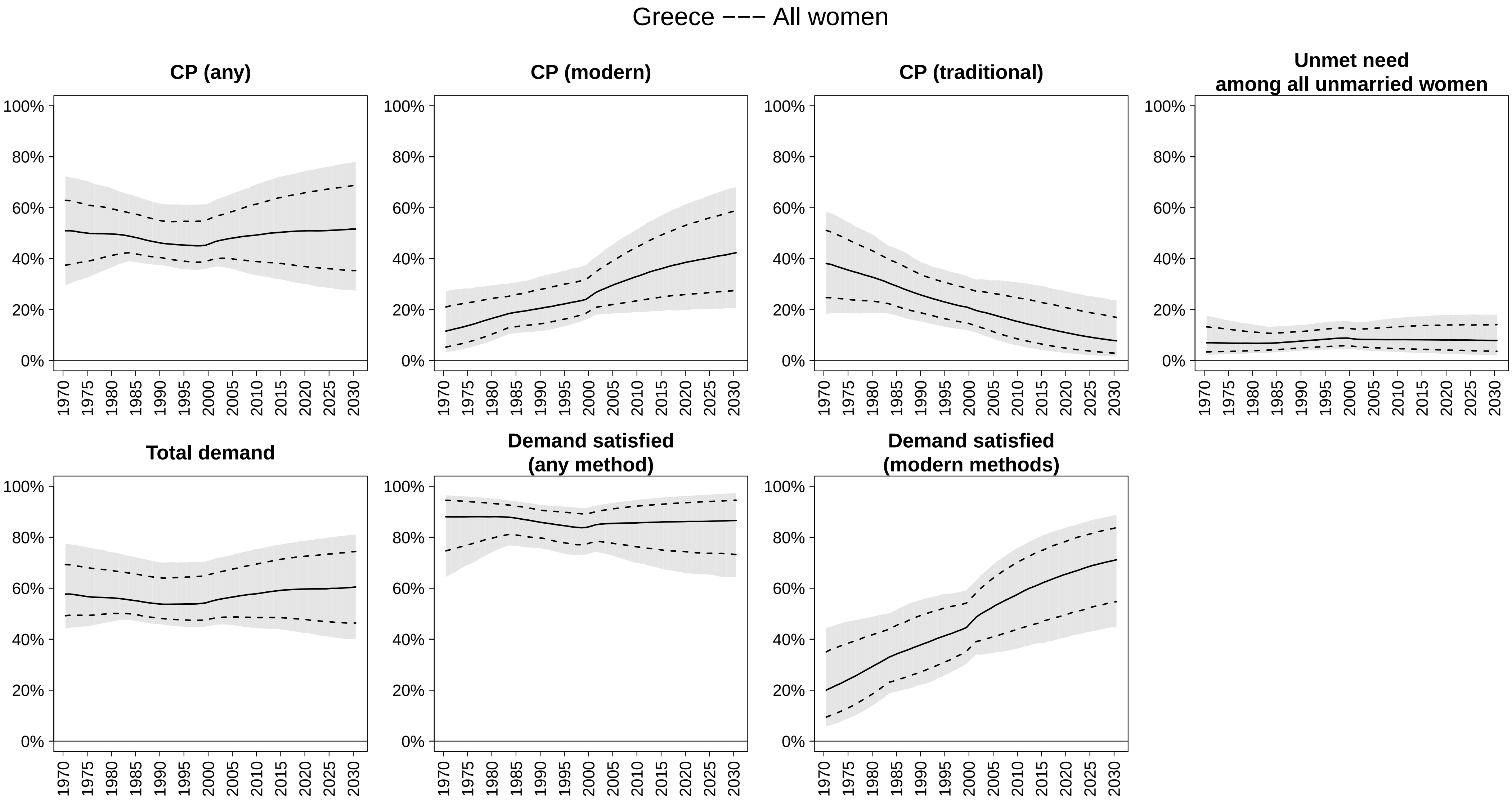
Greece −−− All women
CP (any)
CP (modern)
CP (traditional)
Unmet need among all unmarried women
Total demand
Demand satisfied (any method)
Demand satisfied (modern methods)
0%
20%
40%
60%
80%
100%
1970
1975
1980
1985
1990
1995
2000
2005
2010
2015
2020
2025
2030

Grenada −−− All women

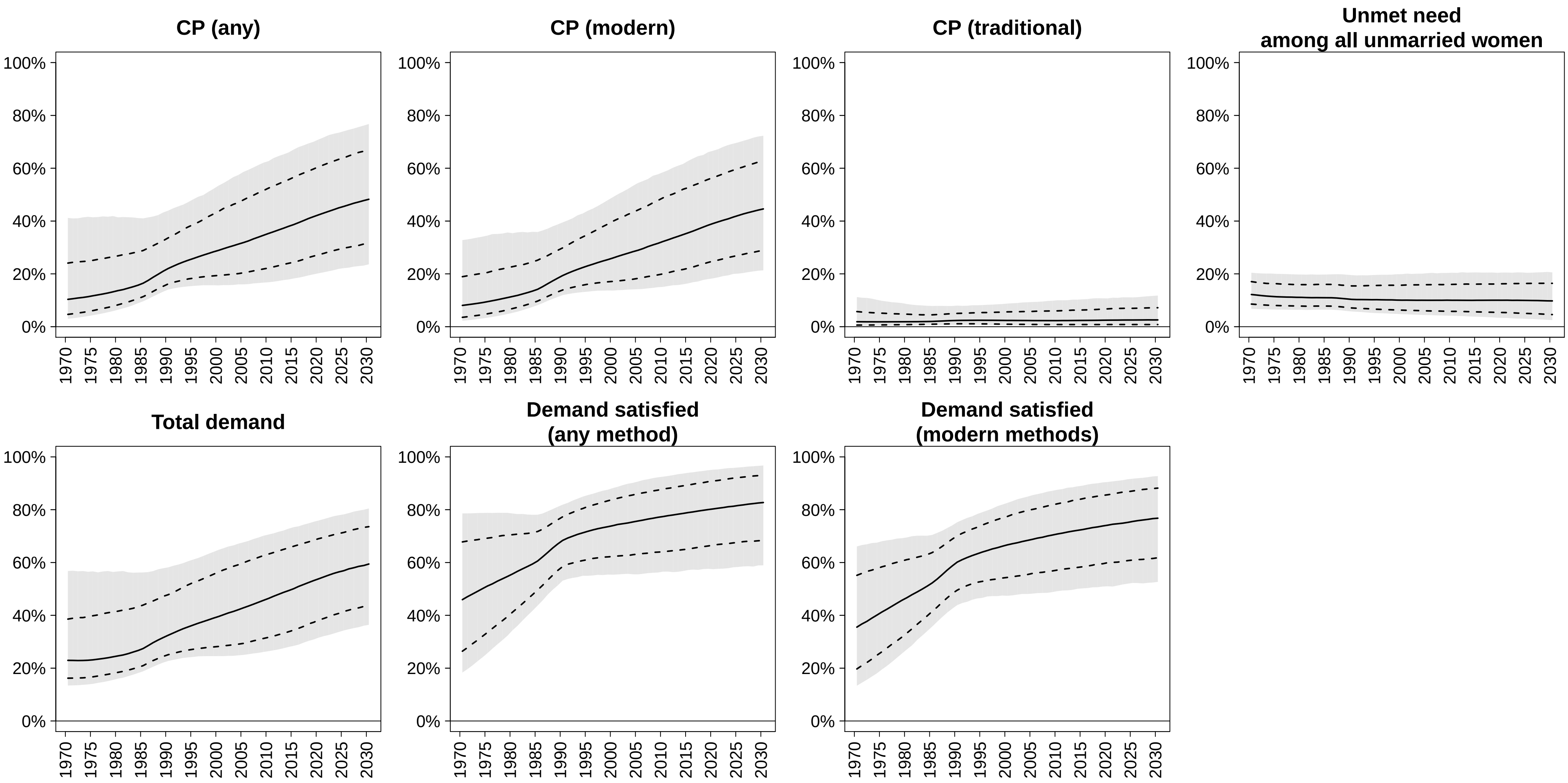

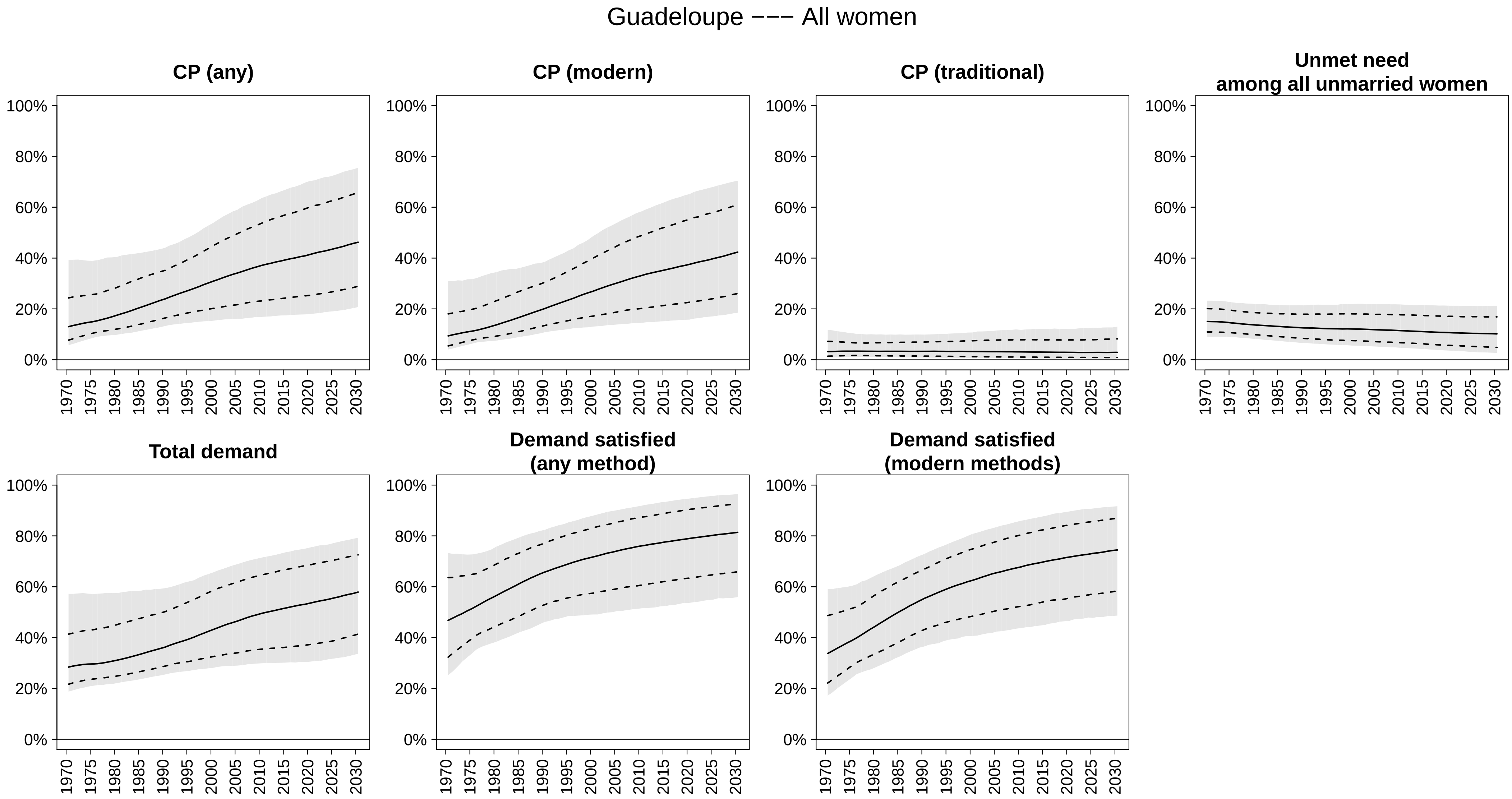
Guadeloupe −−− All women
CP (any)
CP (modern)
CP (traditional)
Unmet need
among all unmarried women
Total demand
Demand satisfied
(any method)
Demand satisfied
(modern methods)
0%
20%
40%
60%
80%
100%
1970
1975
1980
1985
1990
1995
2000
2005
2010
2015
2020
2025
2030

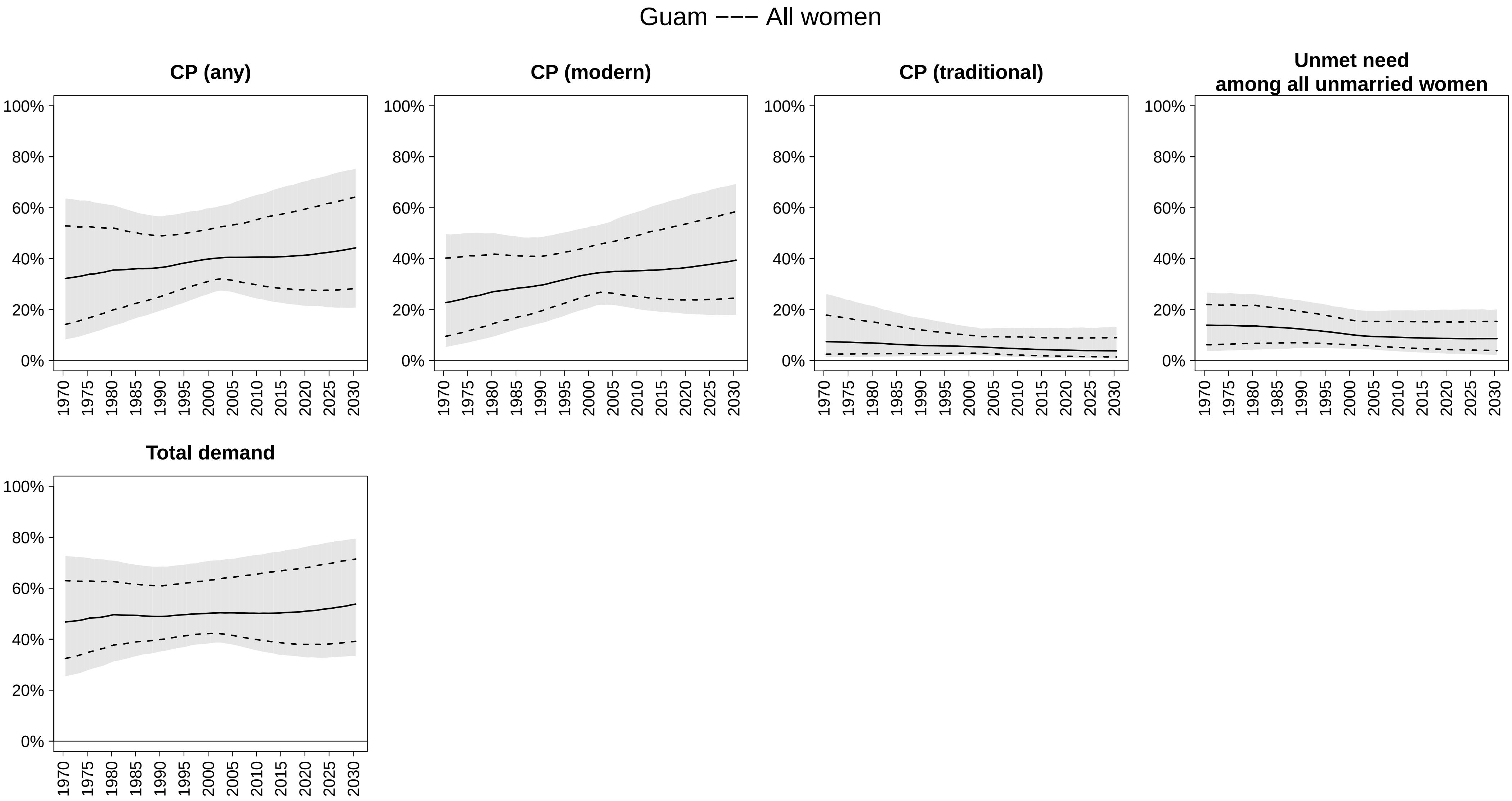
Guam −−− All women
CP (any)
CP (modern)
CP (traditional)
Unmet need
among all unmarried women
Total demand
100%
80%
60%
40%
20%
0%
1970
1975
1980
1985
1990
1995
2000
2005
2010
2015
2020
2025
2030

Guatemala −−− All women

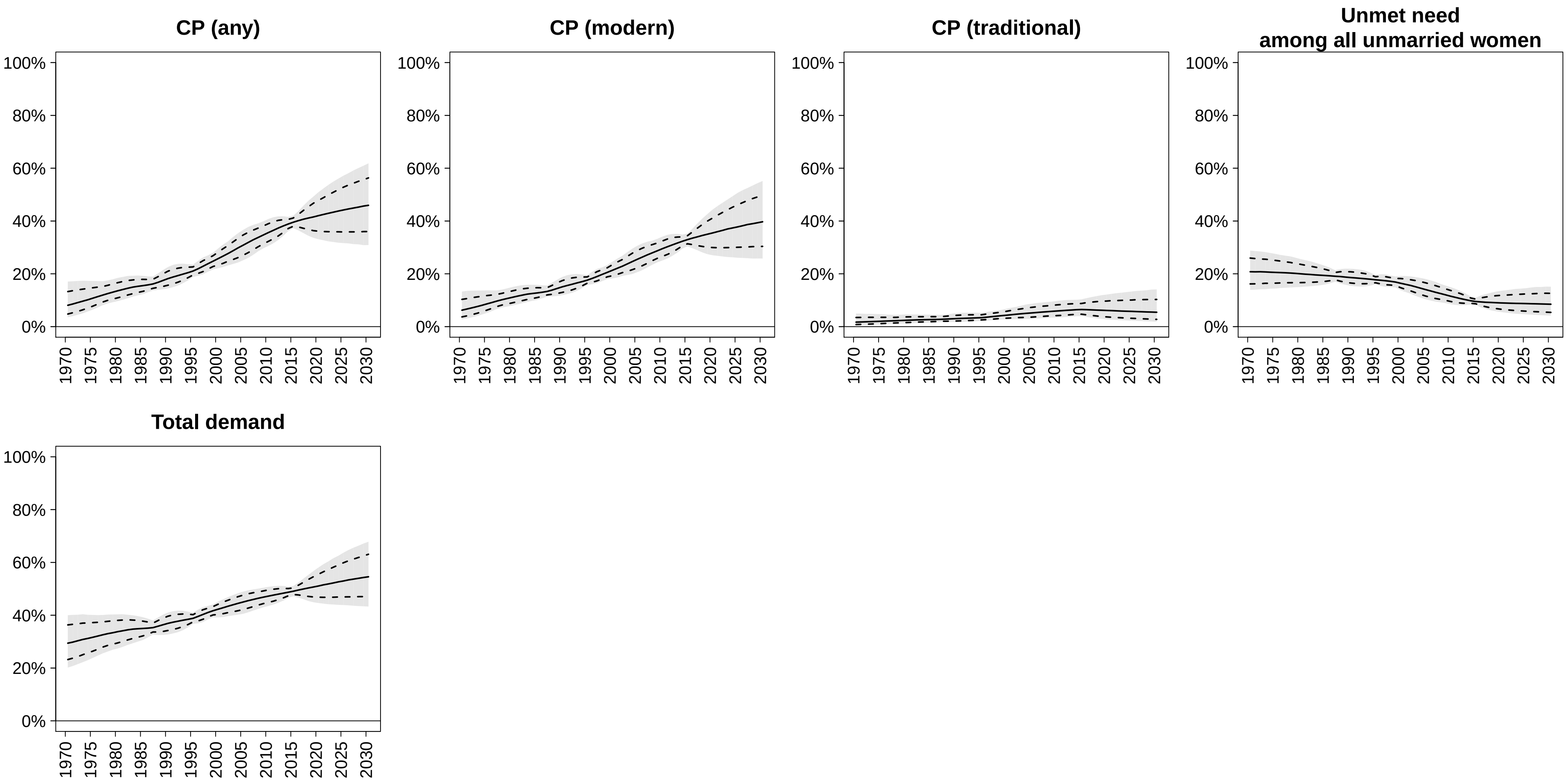

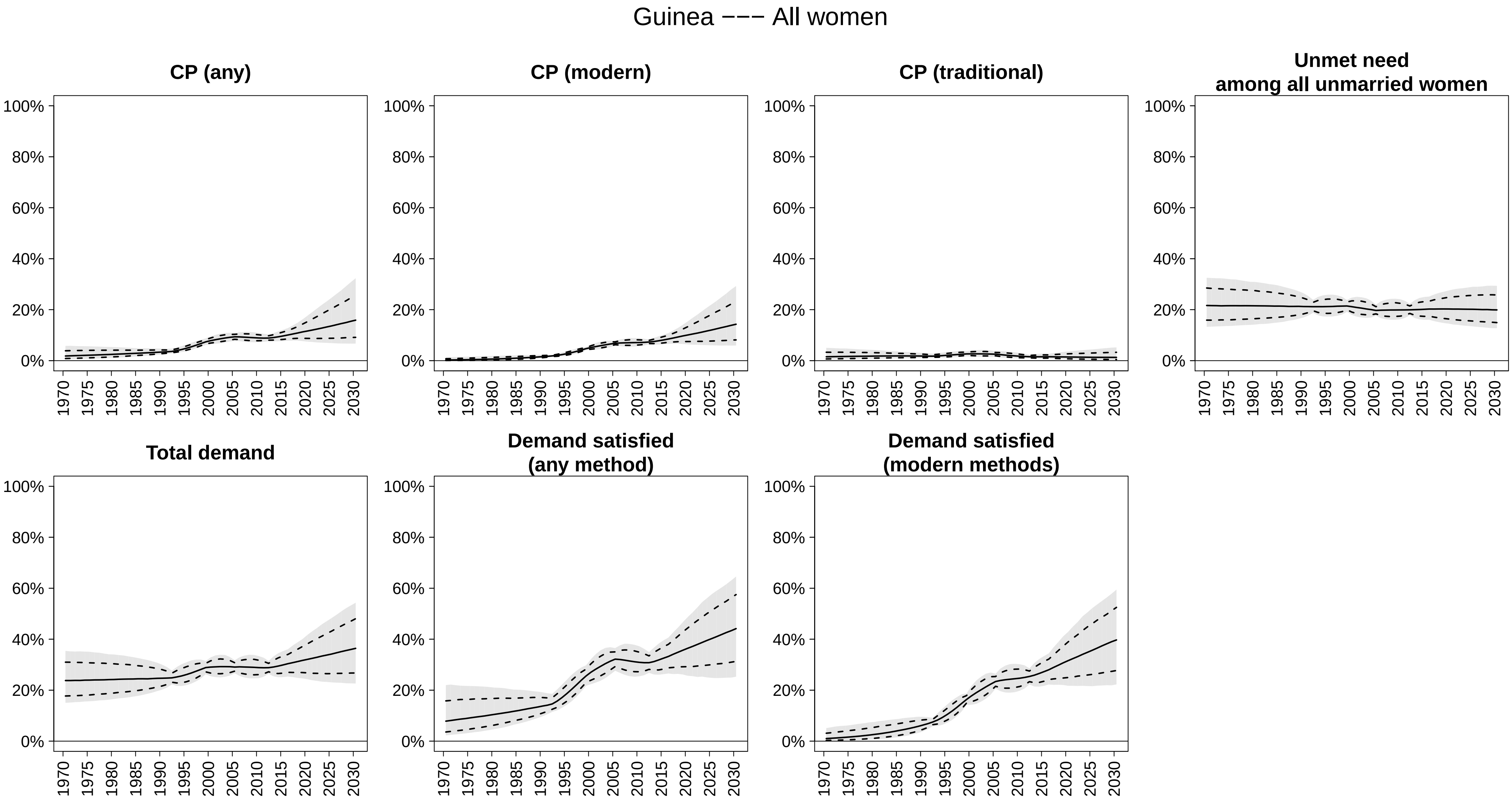
Guinea −−− All women
CP (any)
CP (modern)
CP (traditional)
Unmet need
among all unmarried women
Total demand
Demand satisfied
(any method)
Demand satisfied
(modern methods)
0%
20%
40%
60%
80%
100%
1970
1975
1980
1985
1990
1995
2000
2005
2010
2015
2020
2025
2030

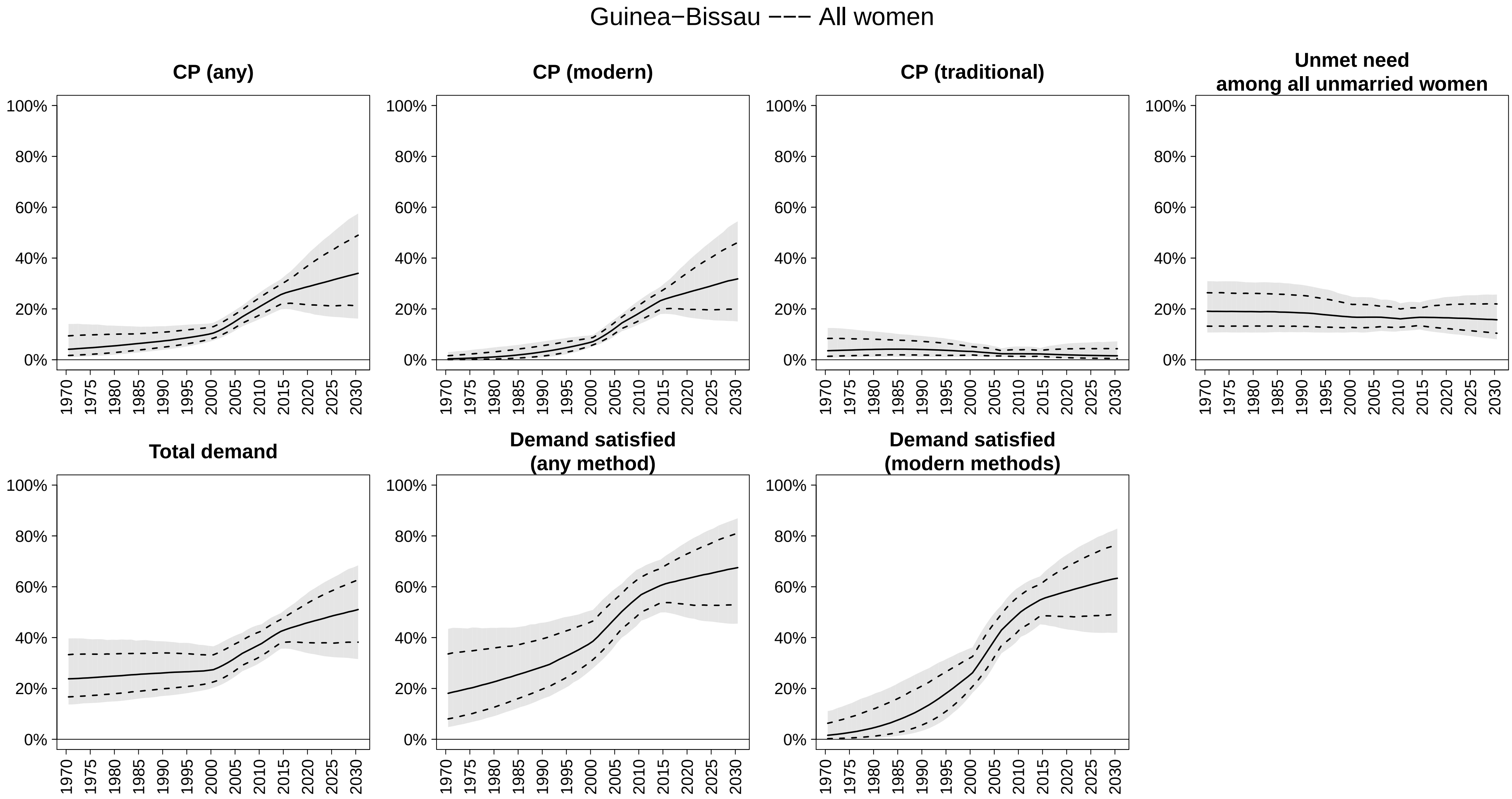
Guinea−Bissau −−− All women
CP (any)
CP (modern)
CP (traditional)
Unmet need
among all unmarried women
Total demand
Demand satisfied
(any method)
Demand satisfied
(modern methods)
0%
20%
40%
60%
80%
100%
1970
1975
1980
1985
1990
1995
2000
2005
2010
2015
2020
2025
2030

Guyana −−− All women

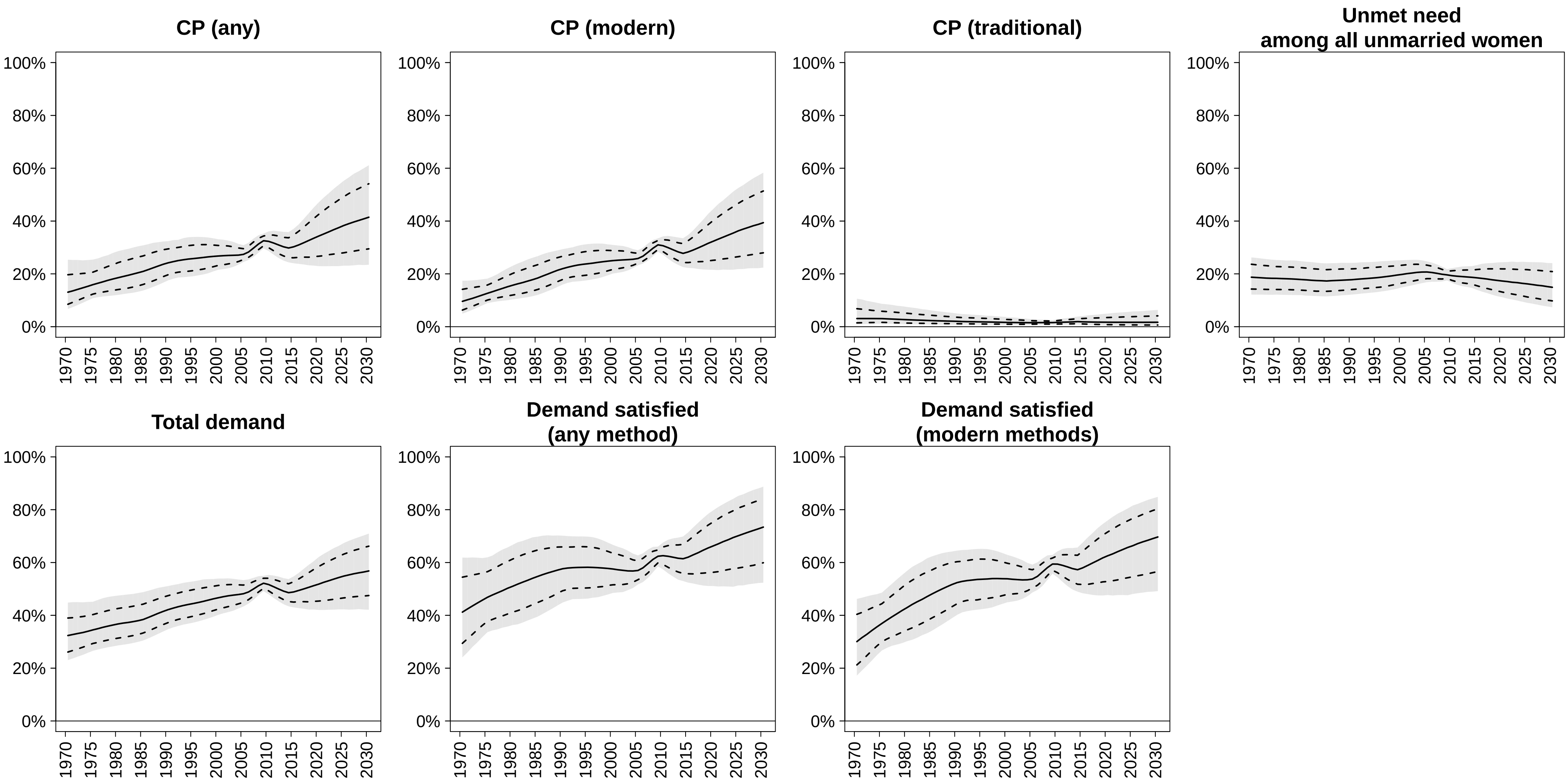

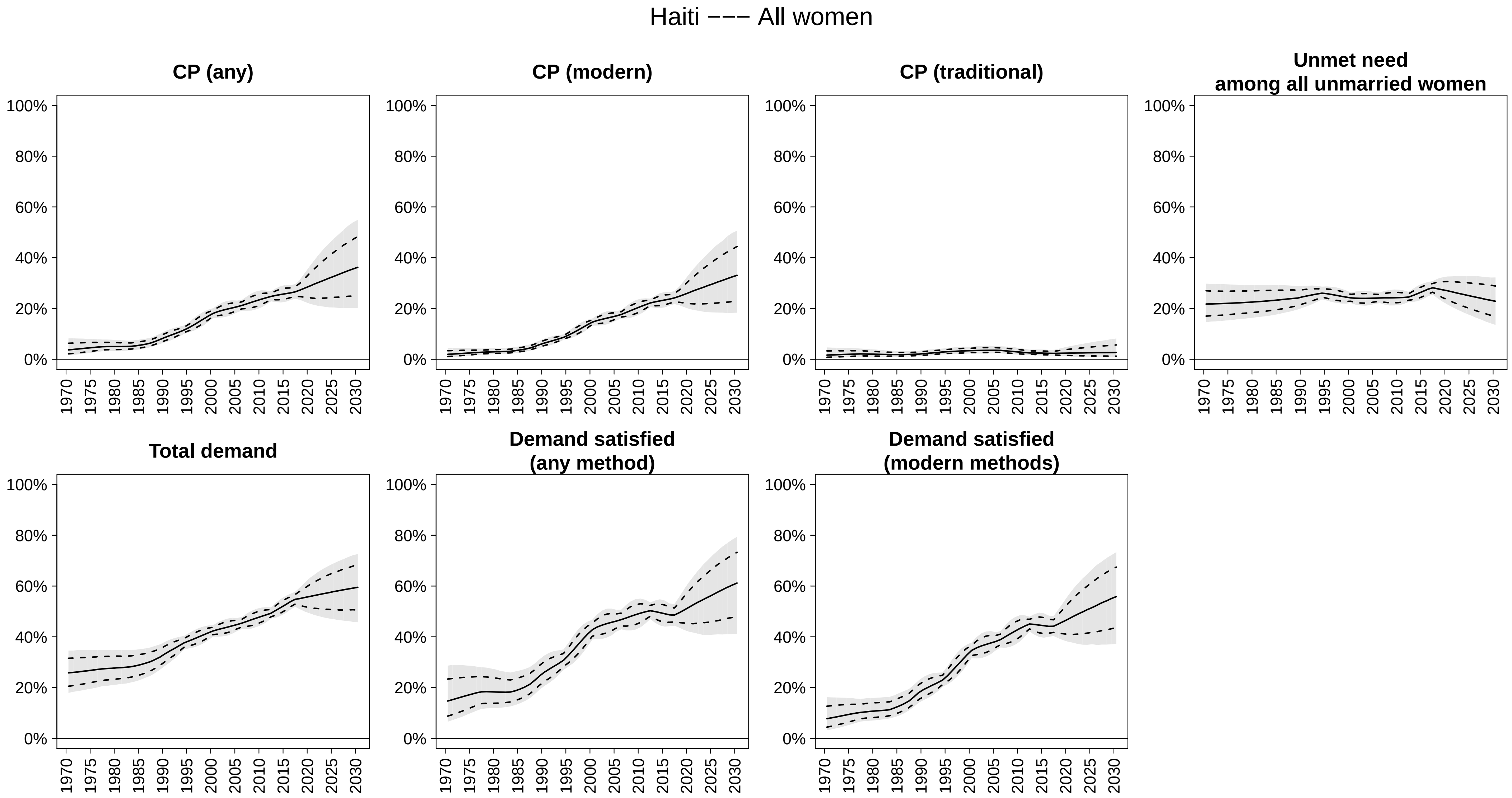
Haiti −−− All women
CP (any)
CP (modern)
CP (traditional)
Unmet need among all unmarried women
Total demand
Demand satisfied (any method)
Demand satisfied (modern methods)
0%
20%
40%
60%
80%
100%
1970
1975
1980
1985
1990
1995
2000
2005
2010
2015
2020
2025
2030

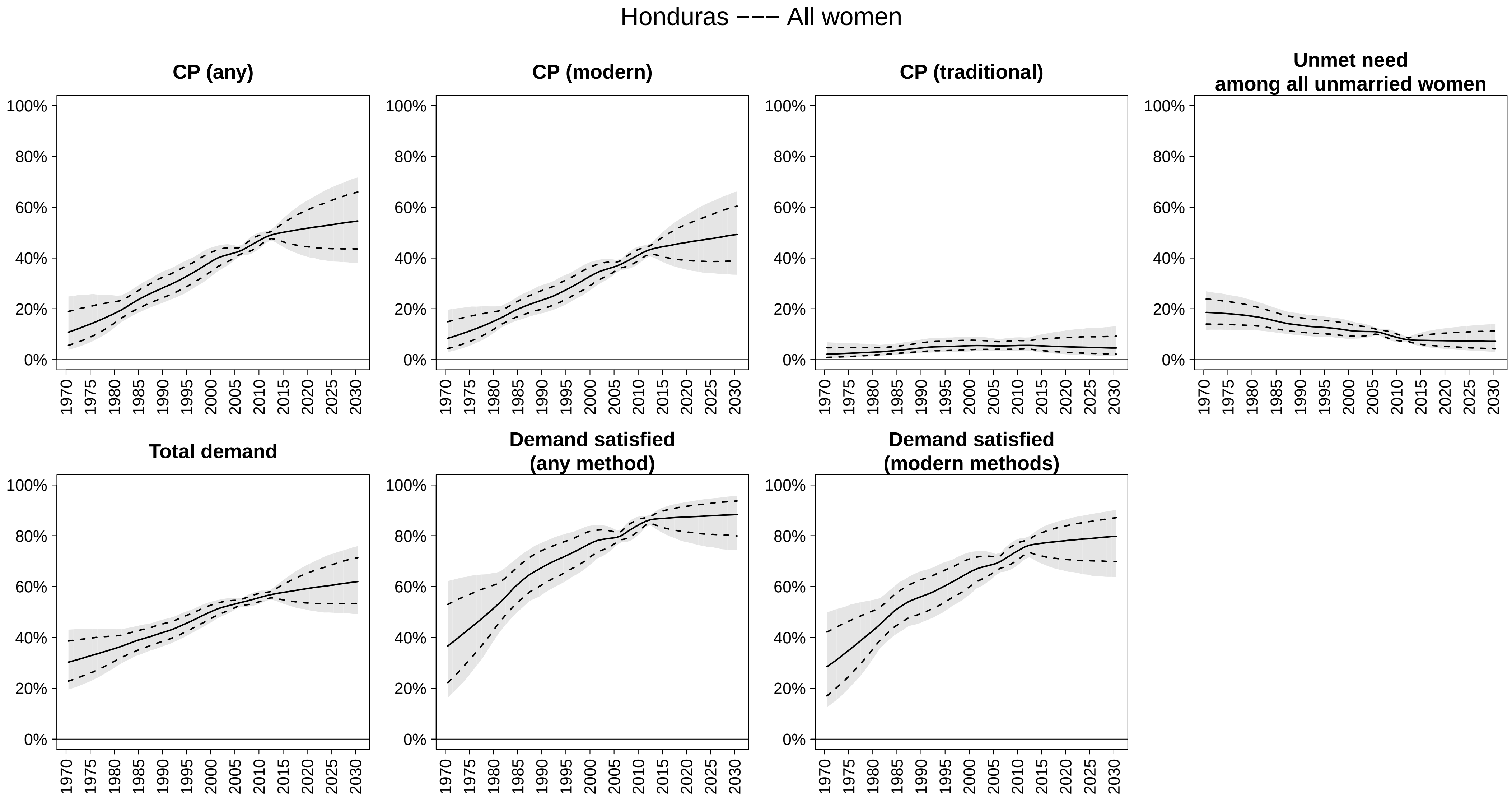
Honduras −−− All women
CP (any)
CP (modern)
CP (traditional)
Unmet need
among all unmarried women
Total demand
Demand satisfied
(any method)
Demand satisfied
(modern methods)
100%
80%
60%
40%
20%
0%
1970
1975
1980
1985
1990
1995
2000
2005
2010
2015
2020
2025
2030

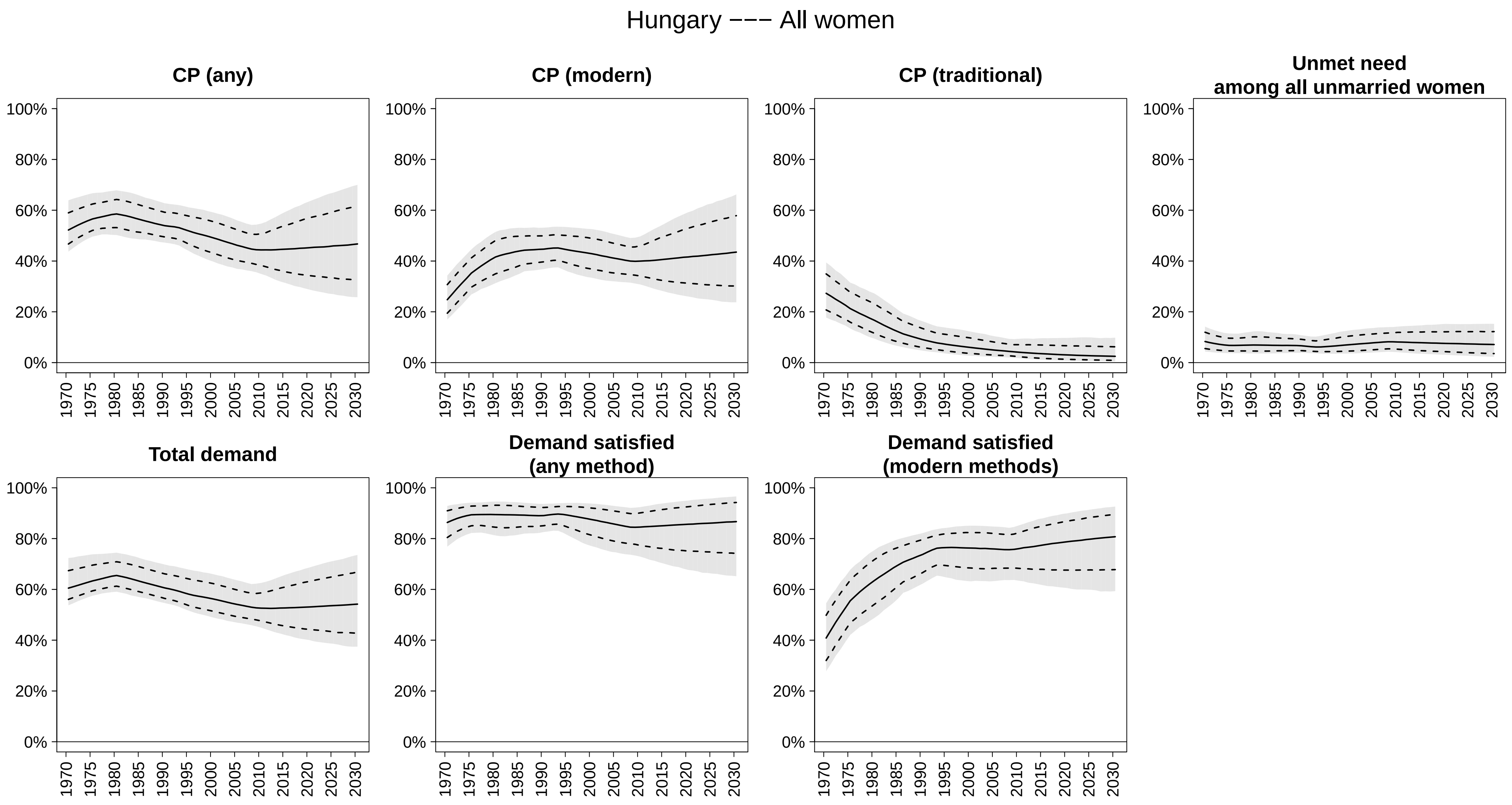
Hungary −−− All women
CP (any)
CP (modern)
CP (traditional)
Unmet need
among all unmarried women
Total demand
Demand satisfied
(any method)
Demand satisfied
(modern methods)
0%
20%
40%
60%
80%
100%
1970
1975
1980
1985
1990
1995
2000
2005
2010
2015
2020
2025
2030

India −−− All women

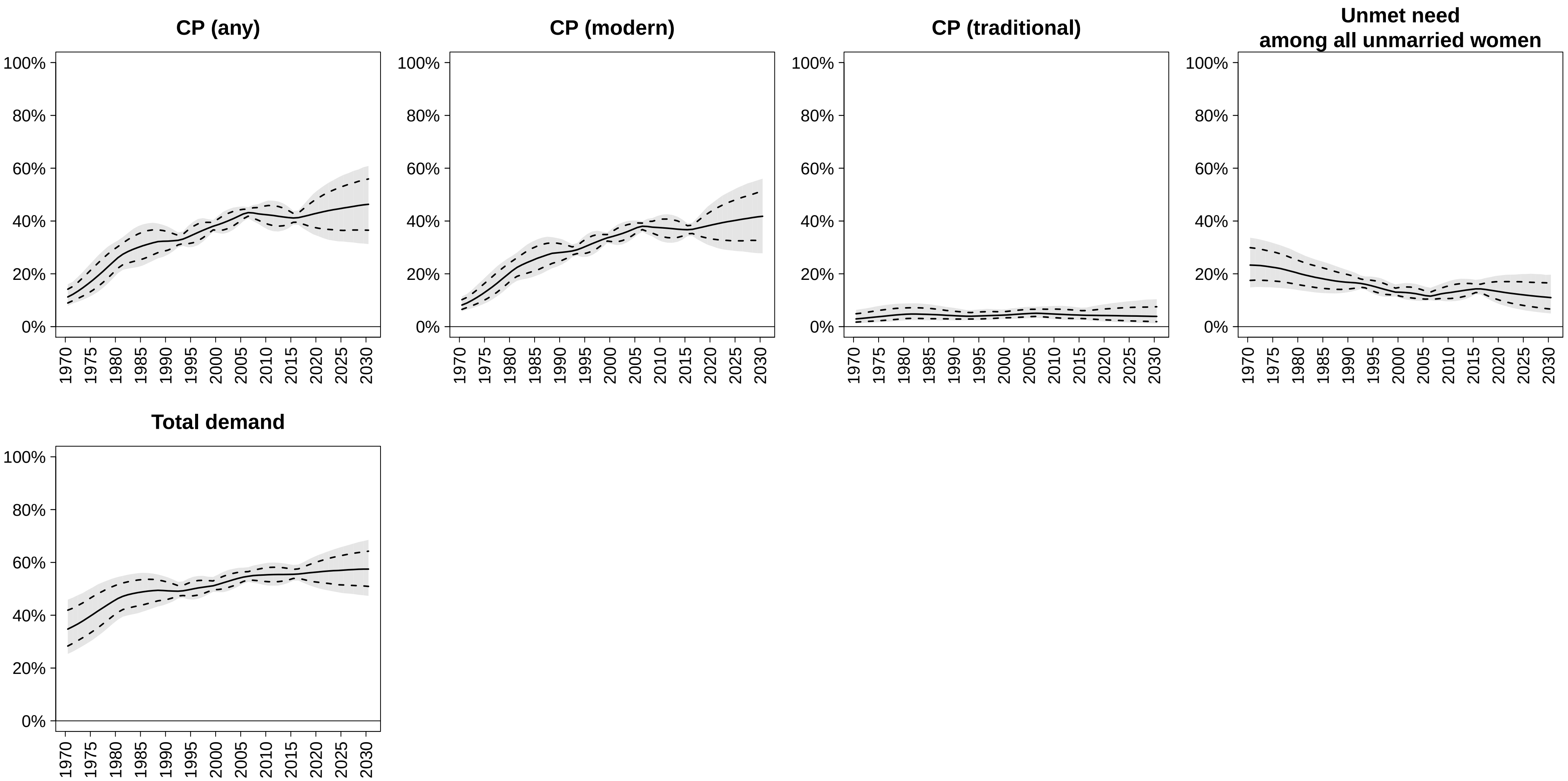

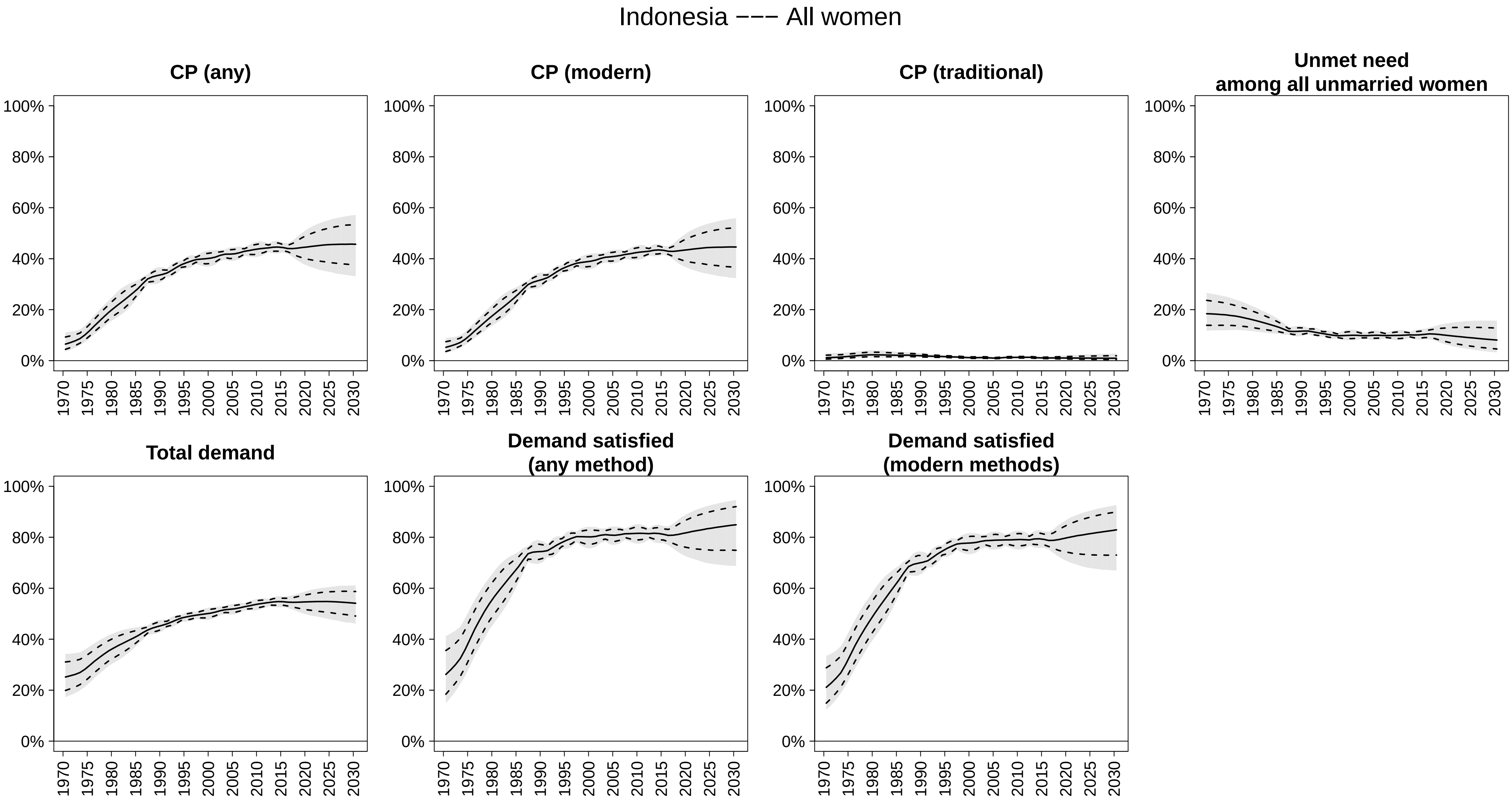
Indonesia −−− All women
CP (any)
CP (modern)
CP (traditional)
Unmet need
among all unmarried women
Total demand
Demand satisfied
(any method)
Demand satisfied
(modern methods)
100%
80%
60%
40%
20%
0%
1970
1975
1980
1985
1990
1995
2000
2005
2010
2015
2020
2025
2030

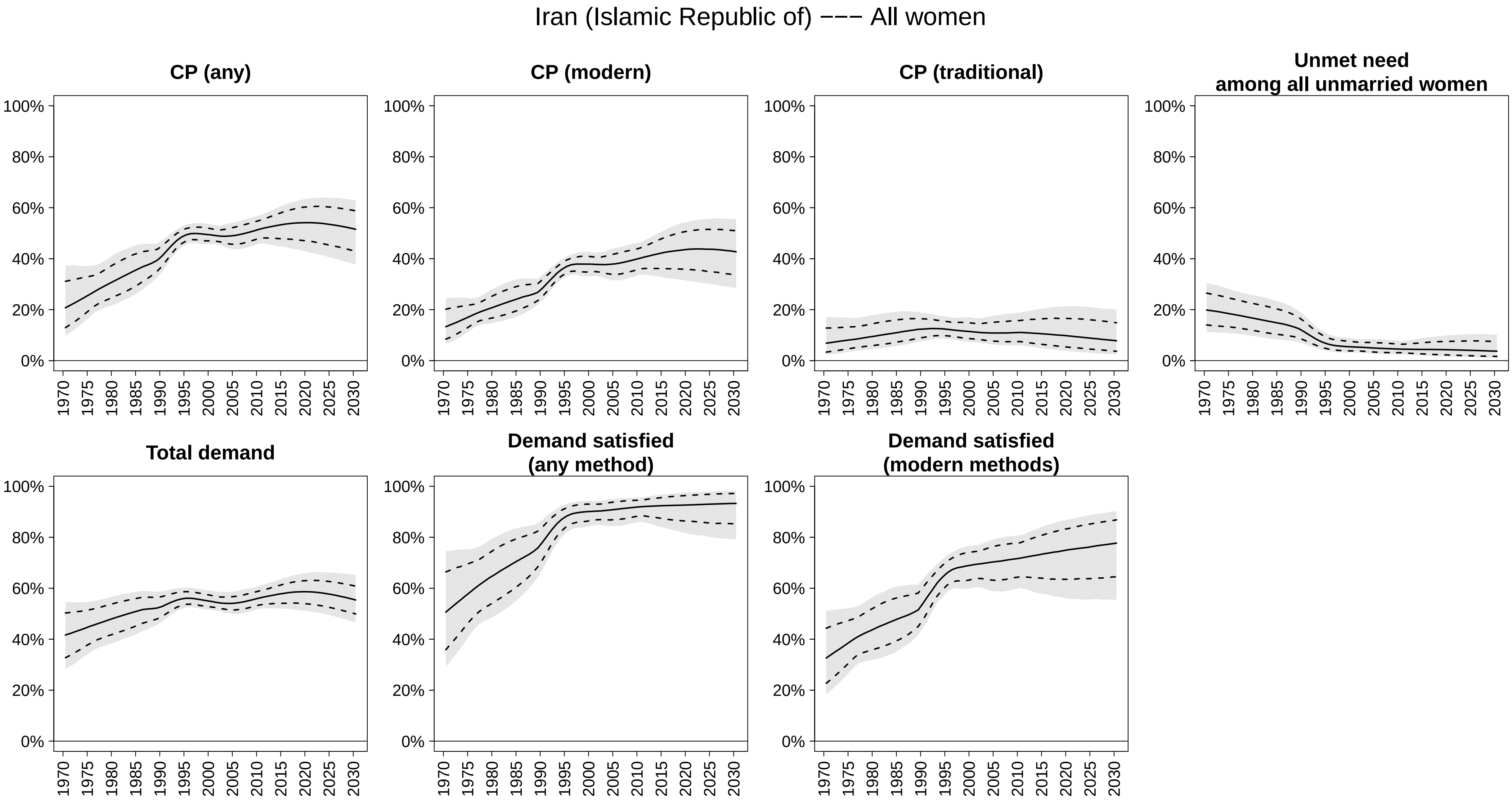
Iran (Islamic Republic of) −−− All women
CP (any)
CP (modern)
CP (traditional)
Unmet need among all unmarried women
Total demand
Demand satisfied (any method)
Demand satisfied (modern methods)
0%
20%
40%
60%
80%
100%
1970
1975
1980
1985
1990
1995
2000
2005
2010
2015
2020
2025
2030

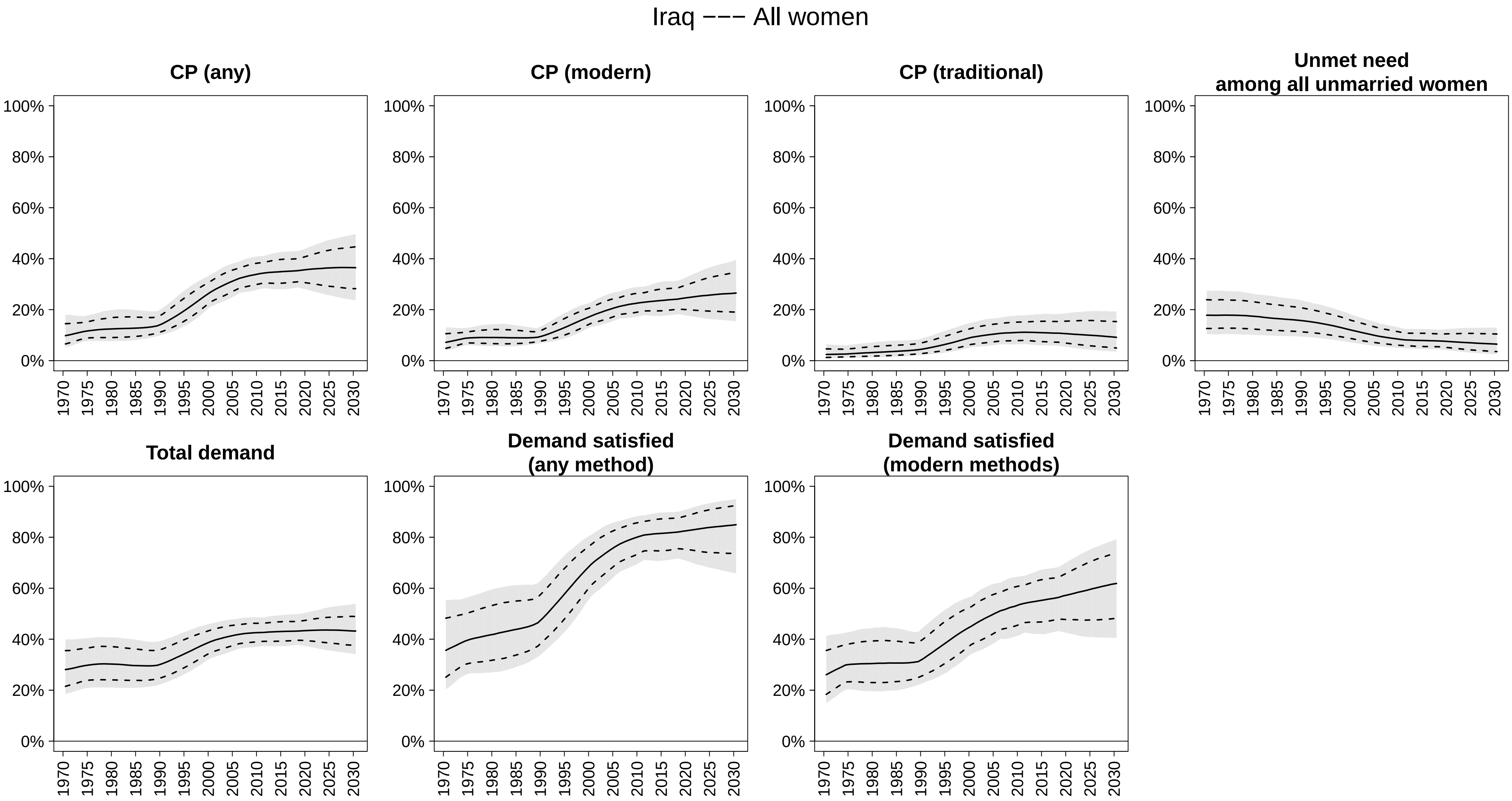
Iraq −−− All women
CP (any)
CP (modern)
CP (traditional)
Unmet need among all unmarried women
Total demand
Demand satisfied (any method)
Demand satisfied (modern methods)
0%
20%
40%
60%
80%
100%
1970
1975
1980
1985
1990
1995
2000
2005
2010
2015
2020
2025
2030

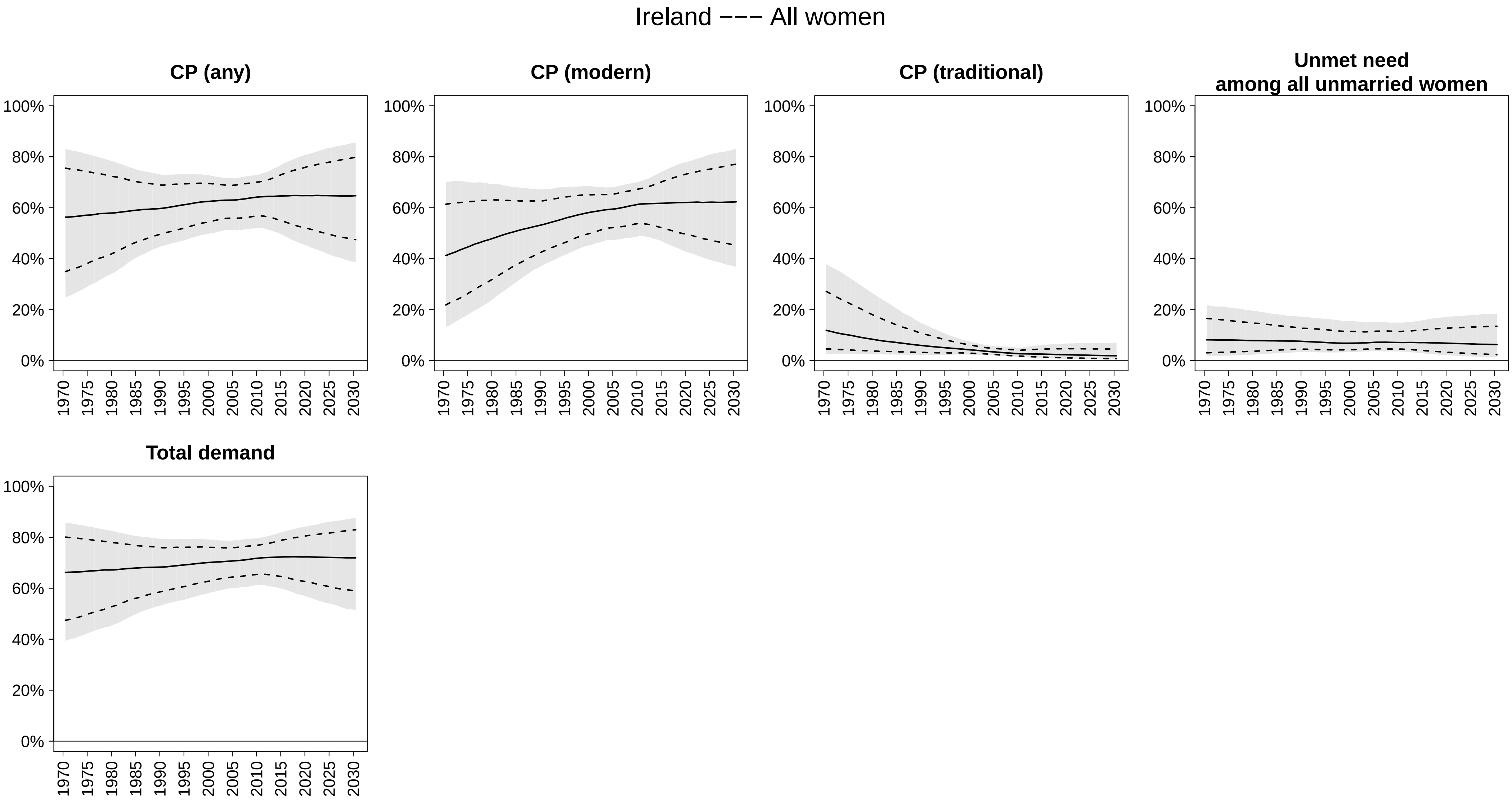
Ireland −−− All women
CP (any)
CP (modern)
CP (traditional)
Unmet need
among all unmarried women
Total demand
100%
80%
60%
40%
20%
0%
1970
1975
1980
1985
1990
1995
2000
2005
2010
2015
2020
2025
2030

Israel −−− All women

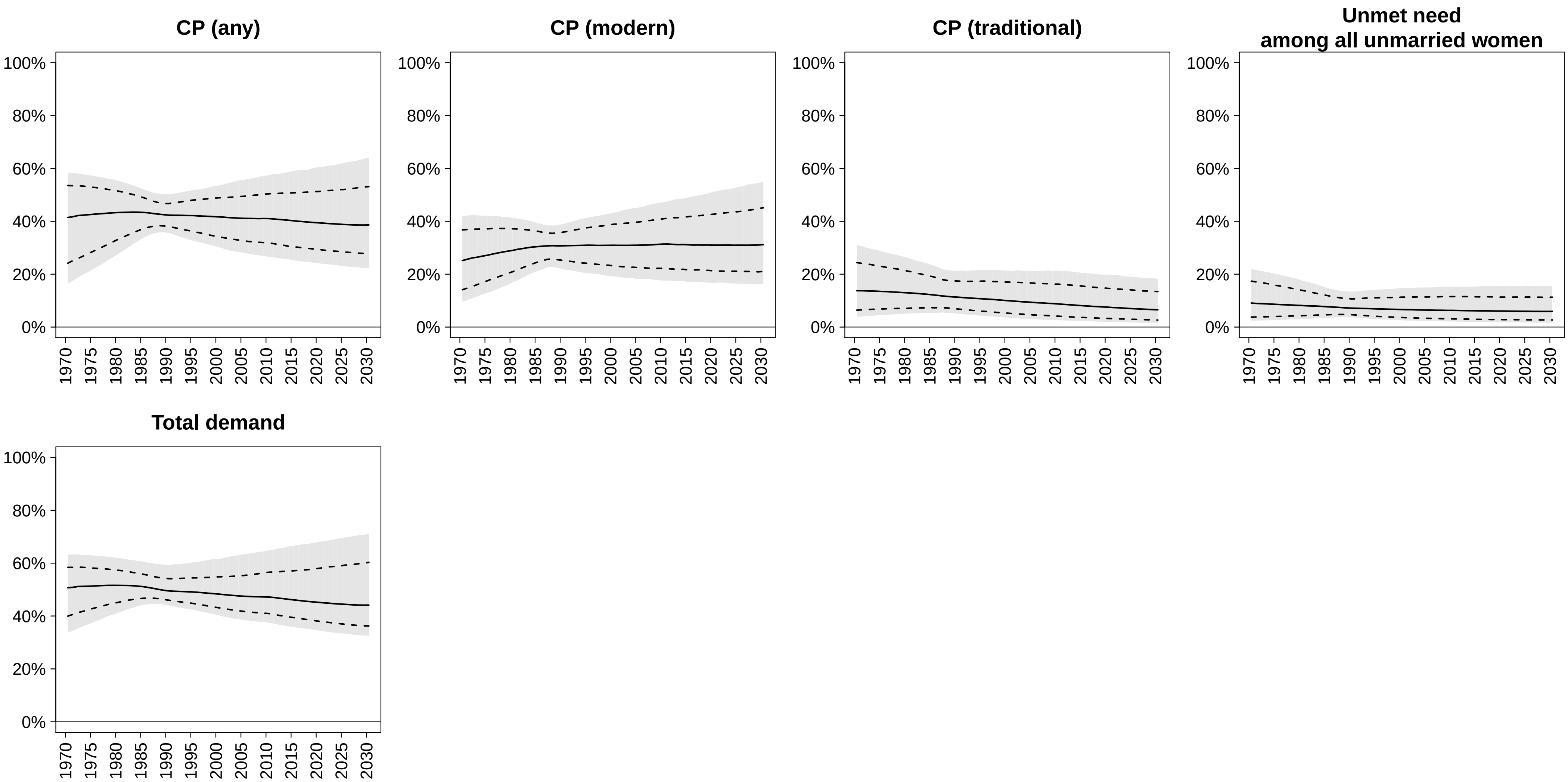

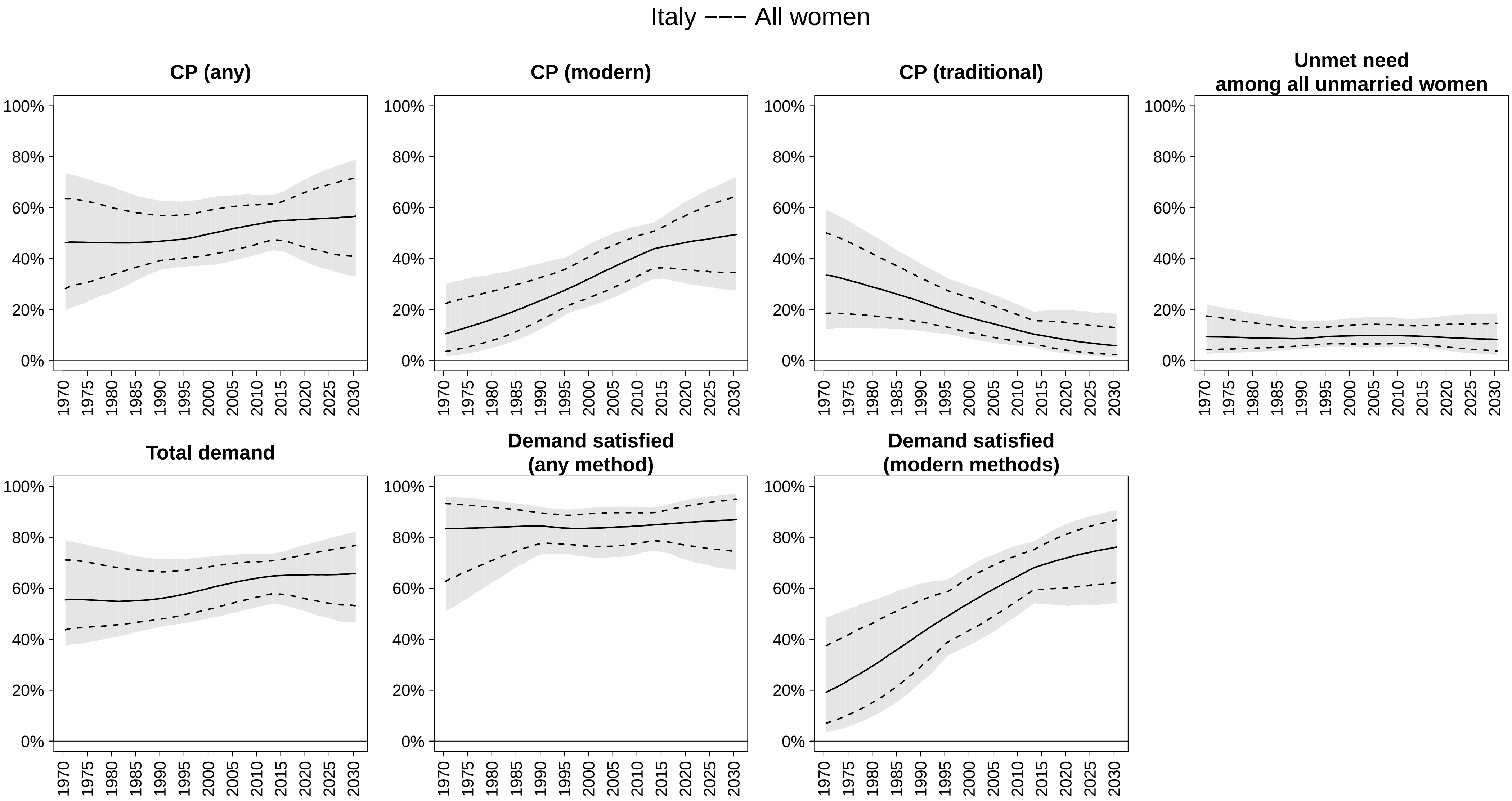
Italy −−− All women
CP (any)
CP (modern)
CP (traditional)
Unmet need among all unmarried women
Total demand
Demand satisfied (any method)
Demand satisfied (modern methods)
0%
20%
40%
60%
80%
100%
1970
1975
1980
1985
1990
1995
2000
2005
2010
2015
2020
2025
2030

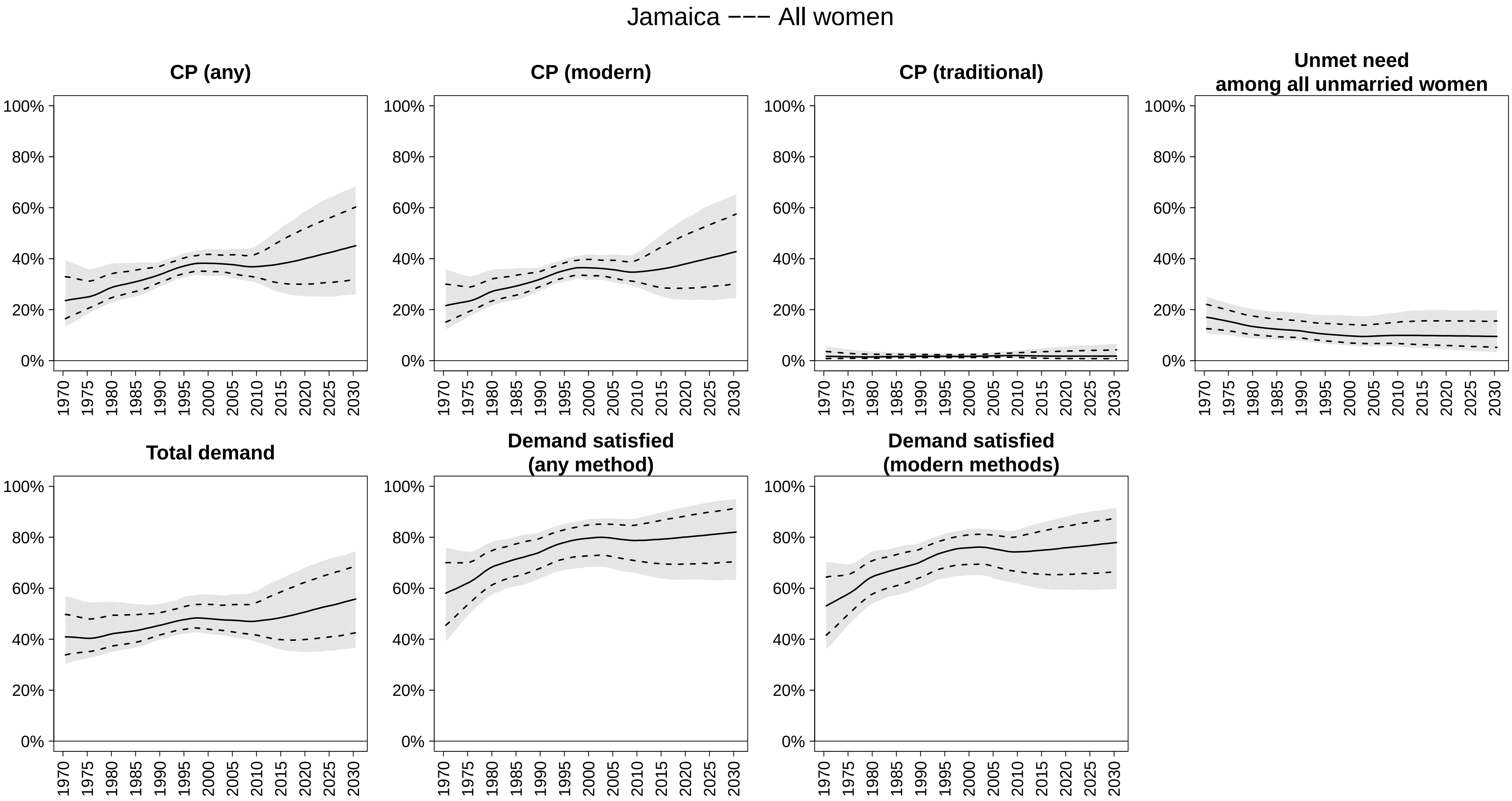
Jamaica −−− All women
CP (any)
CP (modern)
CP (traditional)
Unmet need
among all unmarried women
Total demand
Demand satisfied
(any method)
Demand satisfied
(modern methods)
0%
20%
40%
60%
80%
100%
1970
1975
1980
1985
1990
1995
2000
2005
2010
2015
2020
2025
2030

Japan −−− All women

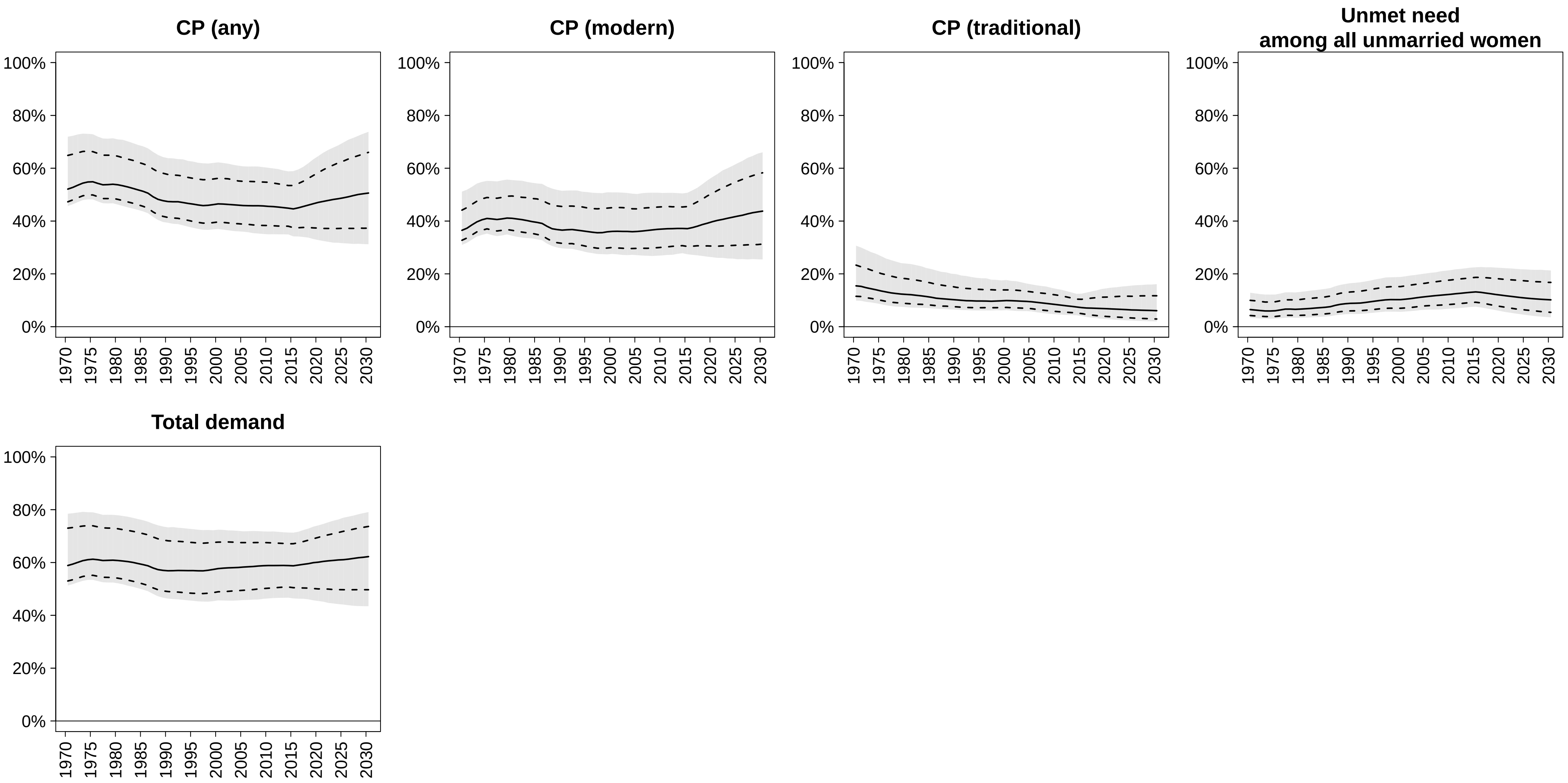

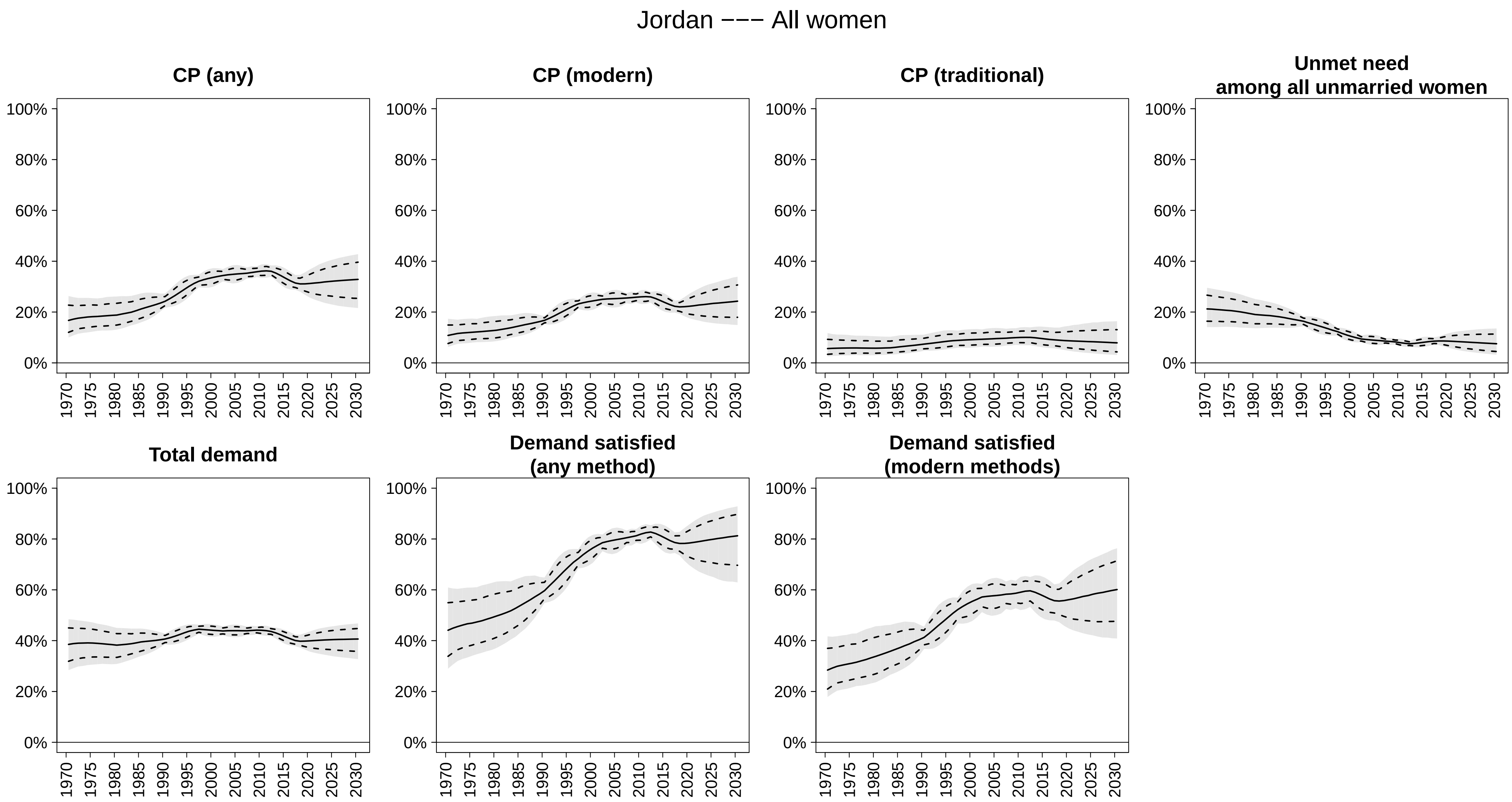
Jordan −−− All women
CP (any)
CP (modern)
CP (traditional)
Unmet need among all unmarried women
Total demand
Demand satisfied (any method)
Demand satisfied (modern methods)
0%
20%
40%
60%
80%
100%
1970
1975
1980
1985
1990
1995
2000
2005
2010
2015
2020
2025
2030

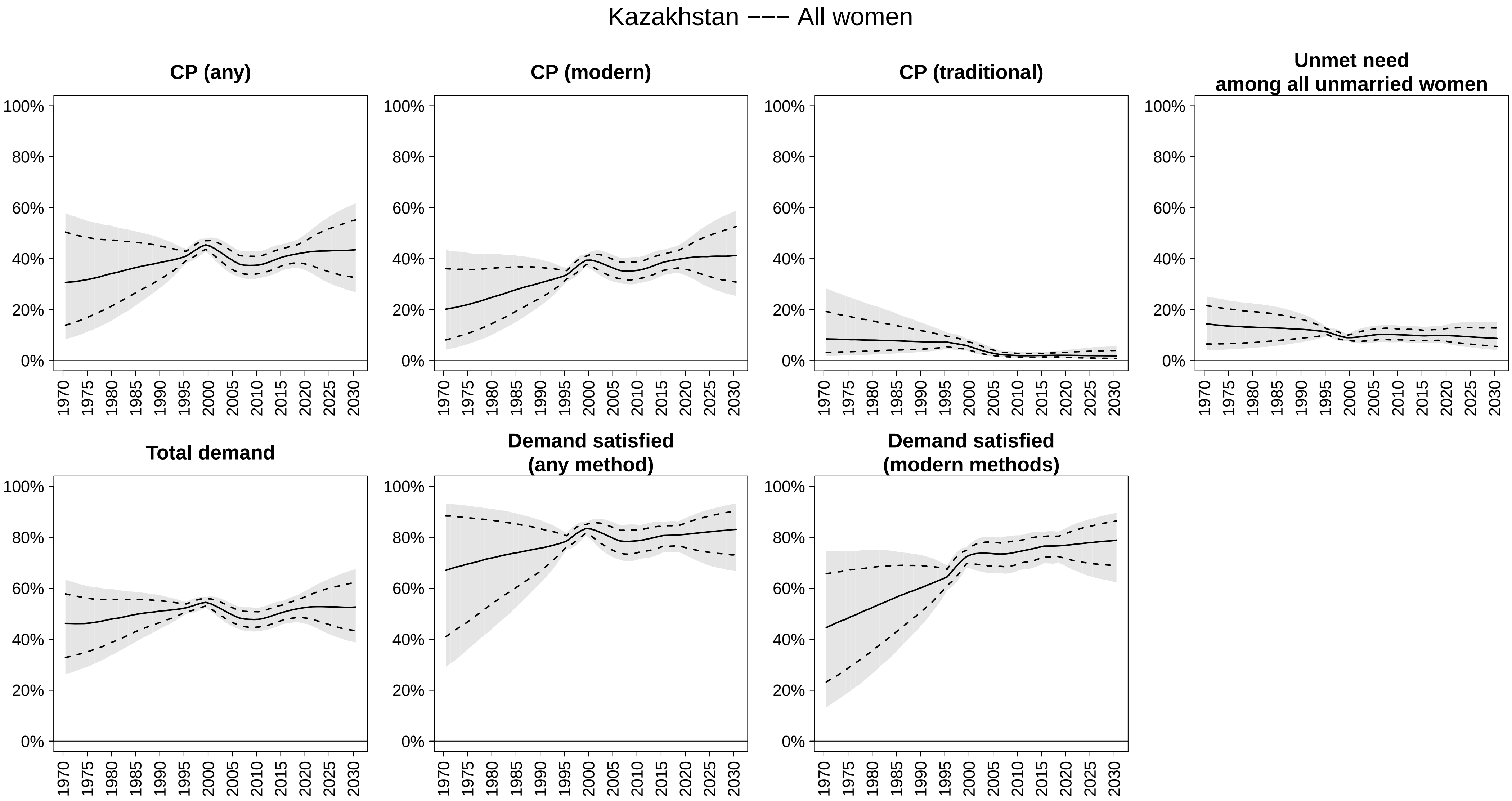
Kazakhstan −−− All women
CP (any)
CP (modern)
CP (traditional)
Unmet need
among all unmarried women
Total demand
Demand satisfied
(any method)
Demand satisfied
(modern methods)
0%
20%
40%
60%
80%
100%
1970
1975
1980
1985
1990
1995
2000
2005
2010
2015
2020
2025
2030

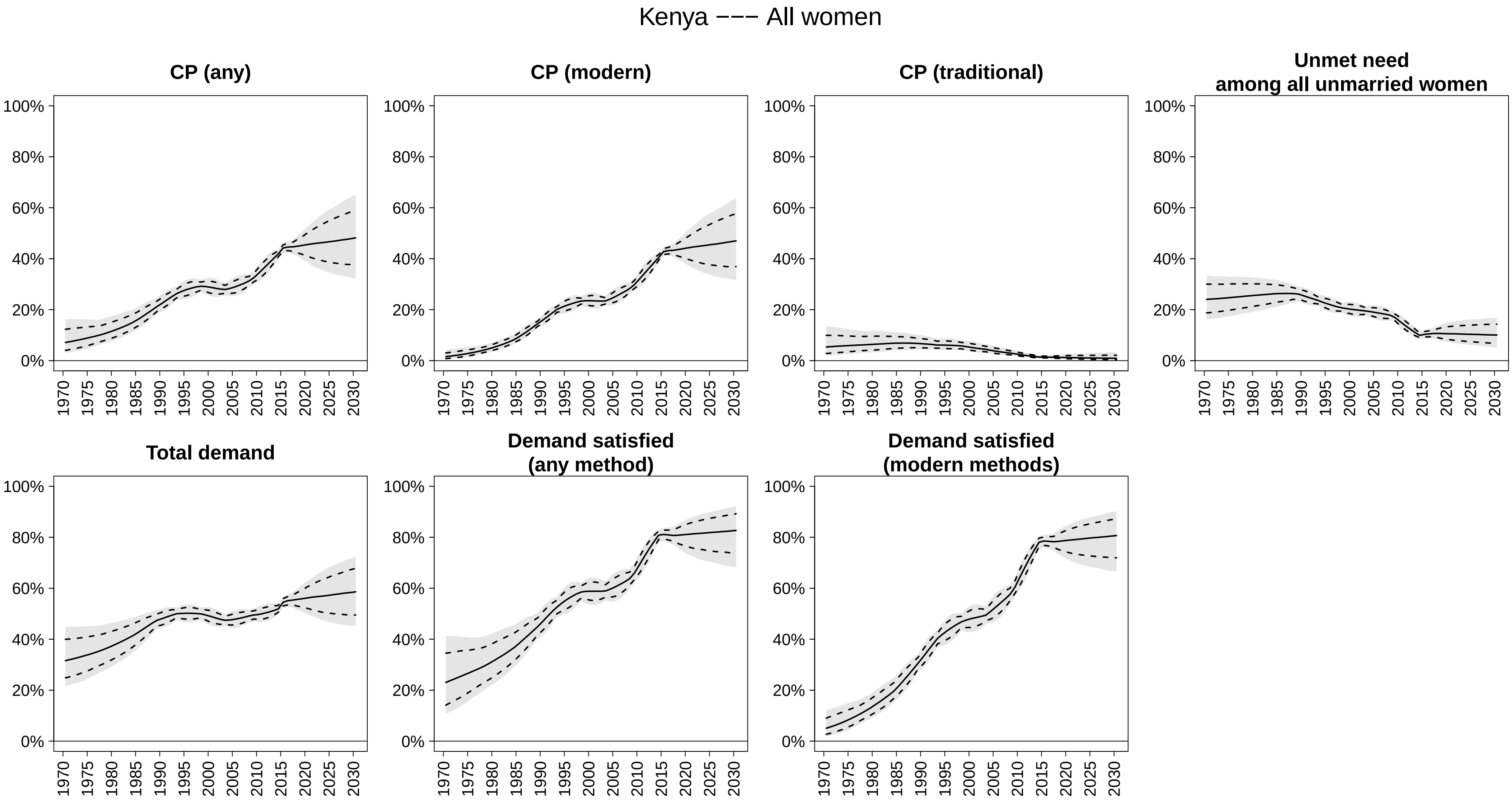
Kenya −−− All women
CP (any)
CP (modern)
CP (traditional)
Unmet need
among all unmarried women
Total demand
Demand satisfied
(any method)
Demand satisfied
(modern methods)
0%
20%
40%
60%
80%
100%
1970
1975
1980
1985
1990
1995
2000
2005
2010
2015
2020
2025
2030

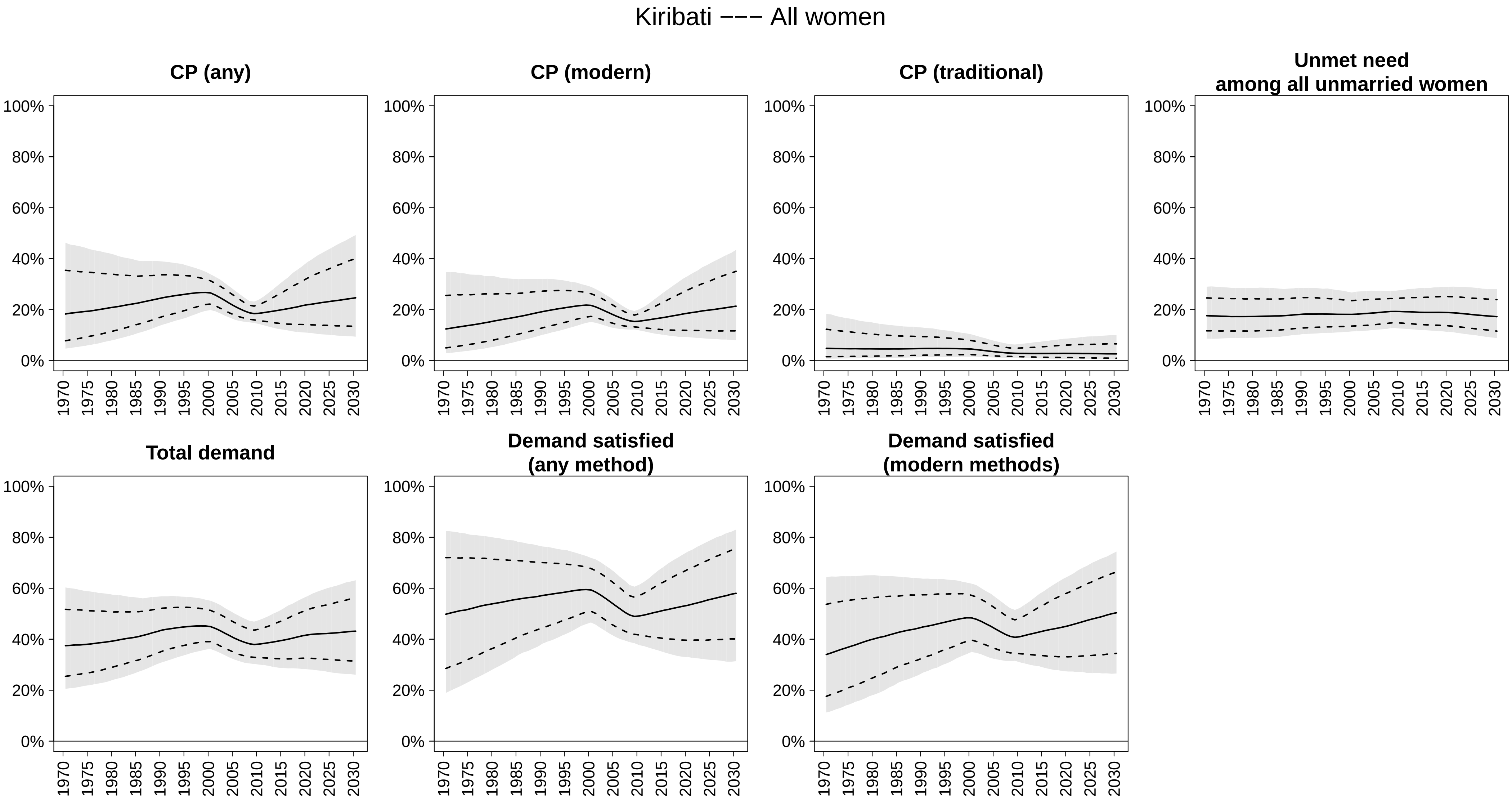
Kiribati −−− All women
CP (any)
CP (modern)
CP (traditional)
Unmet need
among all unmarried women
Total demand
Demand satisfied
(any method)
Demand satisfied
(modern methods)
0%
20%
40%
60%
80%
100%
1970
1975
1980
1985
1990
1995
2000
2005
2010
2015
2020
2025
2030

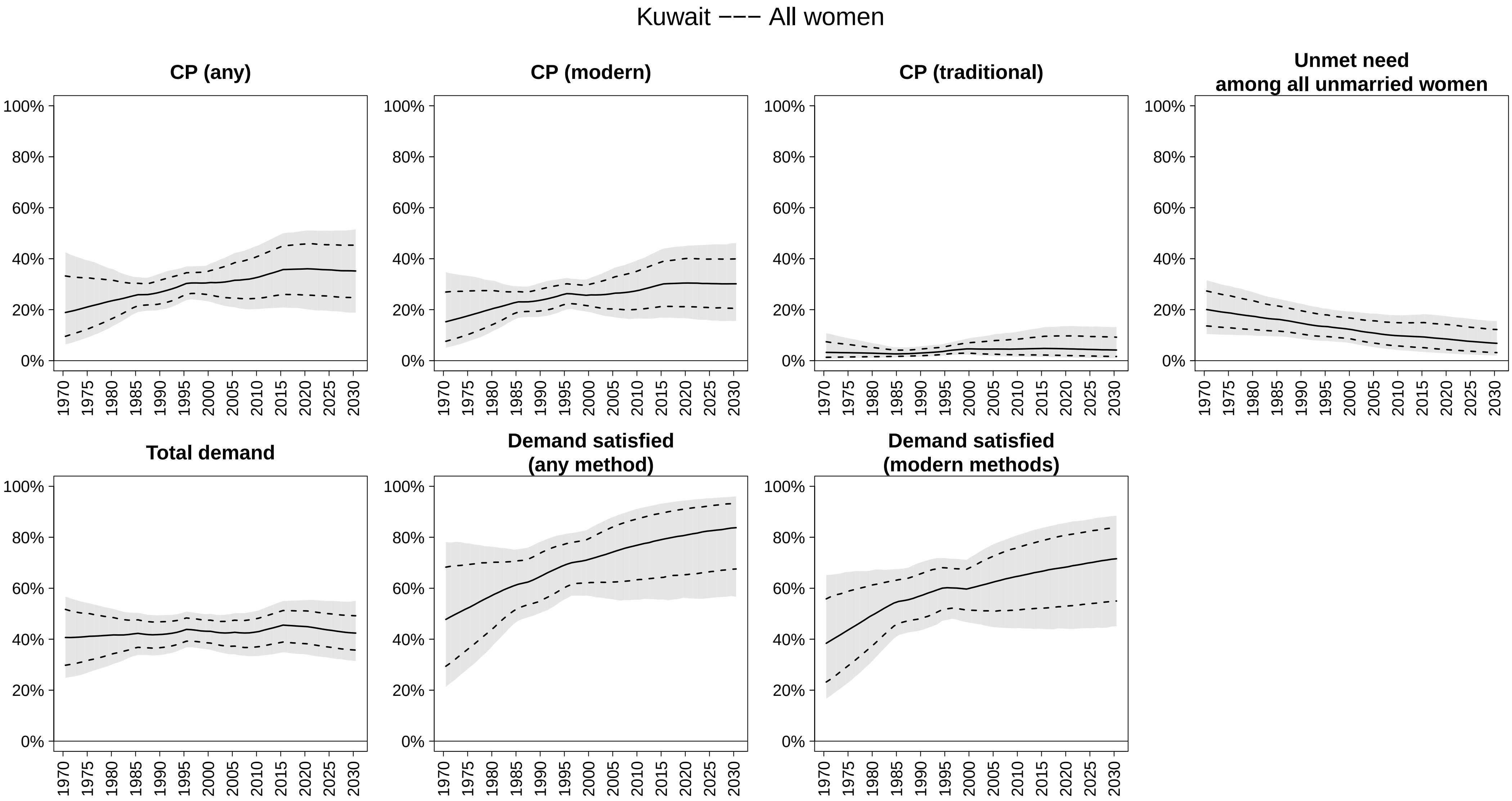
Kuwait −−− All women
CP (any)
CP (modern)
CP (traditional)
Unmet need among all unmarried women
Total demand
Demand satisfied (any method)
Demand satisfied (modern methods)
0%
20%
40%
60%
80%
100%
1970
1975
1980
1985
1990
1995
2000
2005
2010
2015
2020
2025
2030

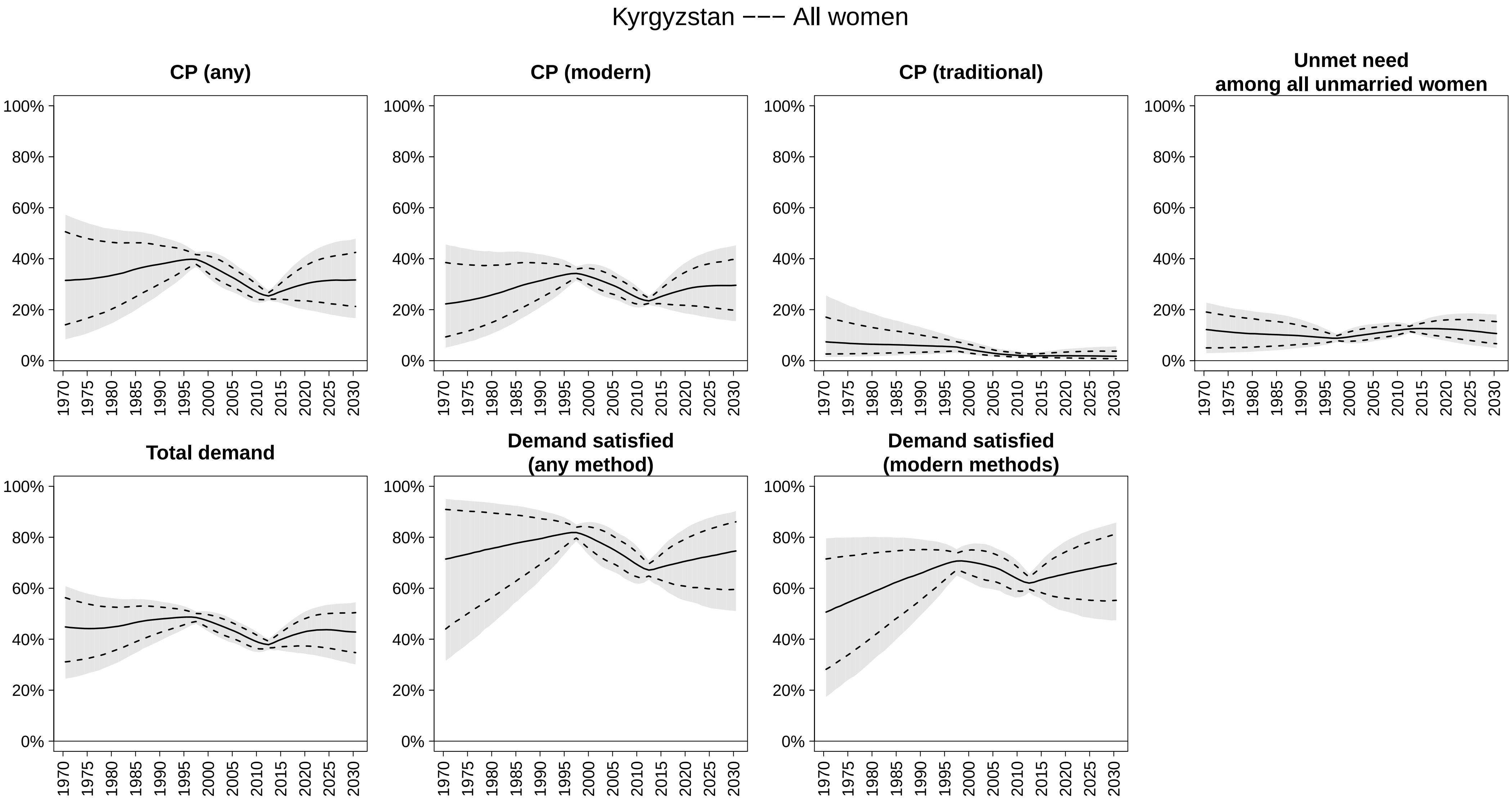
Kyrgyzstan −−− All women
CP (any)
CP (modern)
CP (traditional)
Unmet need
among all unmarried women
Total demand
Demand satisfied
(any method)
Demand satisfied
(modern methods)
0%
20%
40%
60%
80%
100%
1970
1975
1980
1985
1990
1995
2000
2005
2010
2015
2020
2025
2030

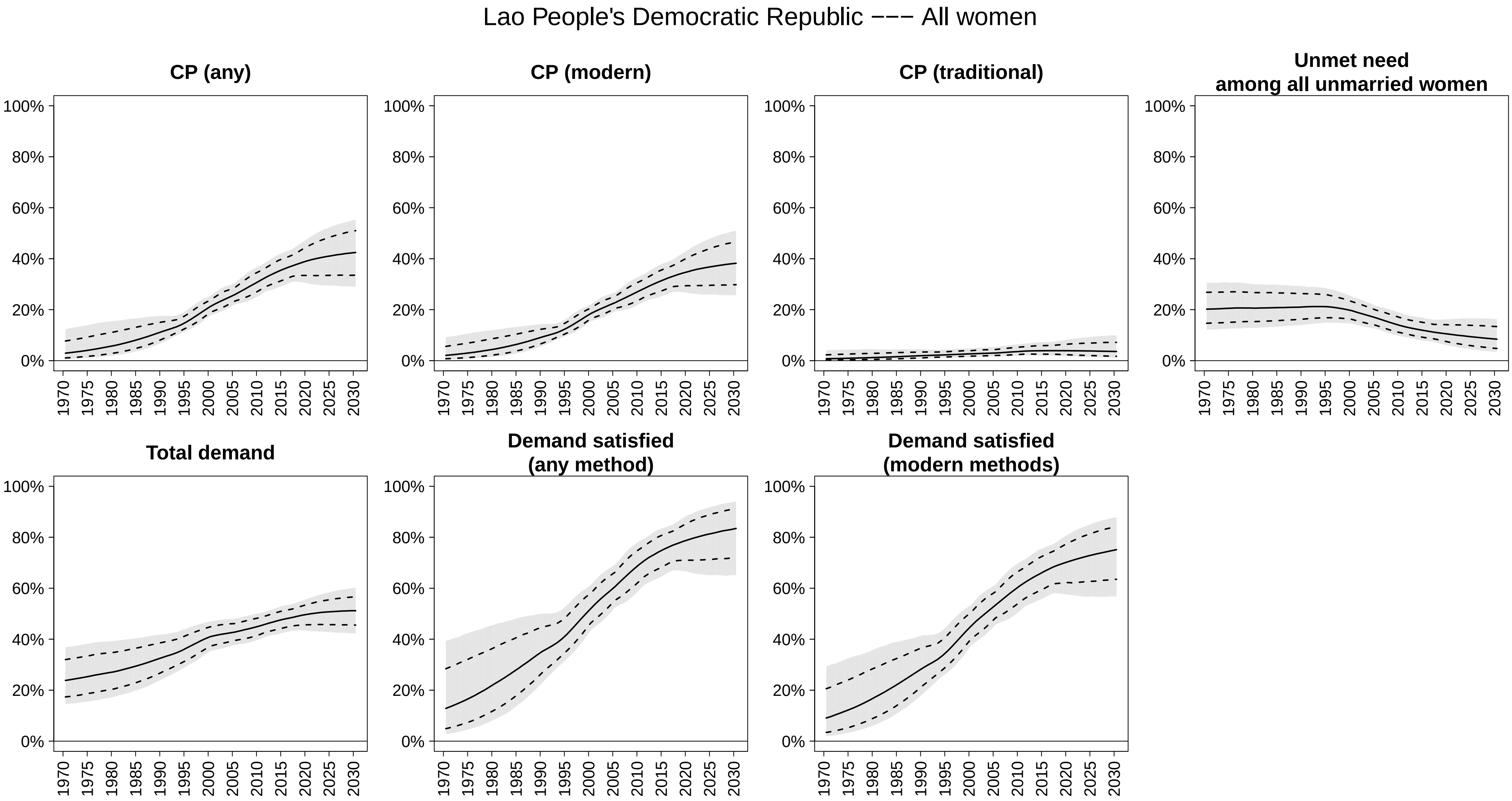
Lao People's Democratic Republic −−− All women
CP (any)
CP (modern)
CP (traditional)
Unmet need among all unmarried women
Total demand
Demand satisfied (any method)
Demand satisfied (modern methods)
0%
20%
40%
60%
80%
100%
1970
1975
1980
1985
1990
1995
2000
2005
2010
2015
2020
2025
2030

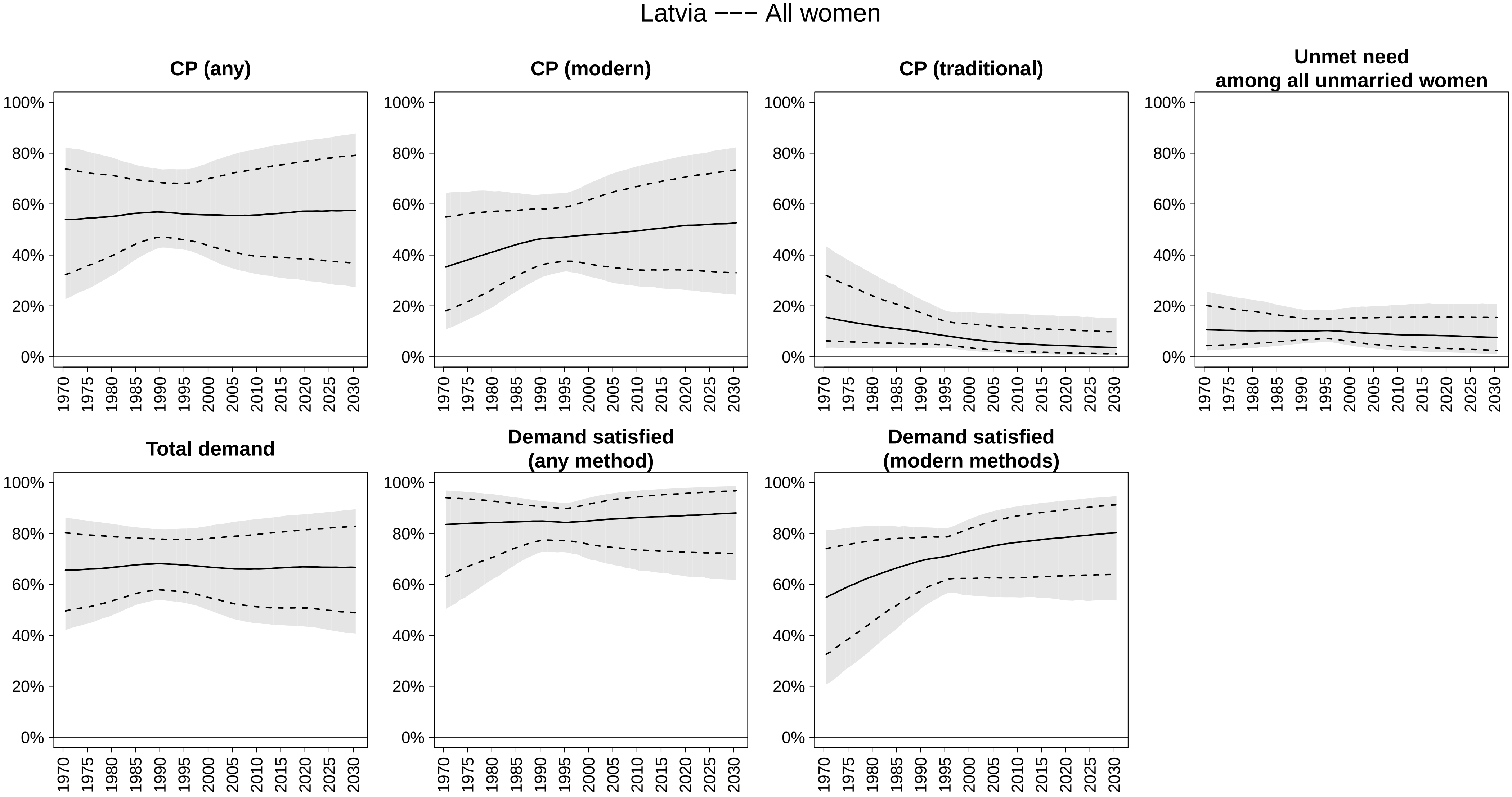
Latvia −−− All women
CP (any)
CP (modern)
CP (traditional)
Unmet need
among all unmarried women
Total demand
Demand satisfied
(any method)
Demand satisfied
(modern methods)
0%
20%
40%
60%
80%
100%
1970
1975
1980
1985
1990
1995
2000
2005
2010
2015
2020
2025
2030

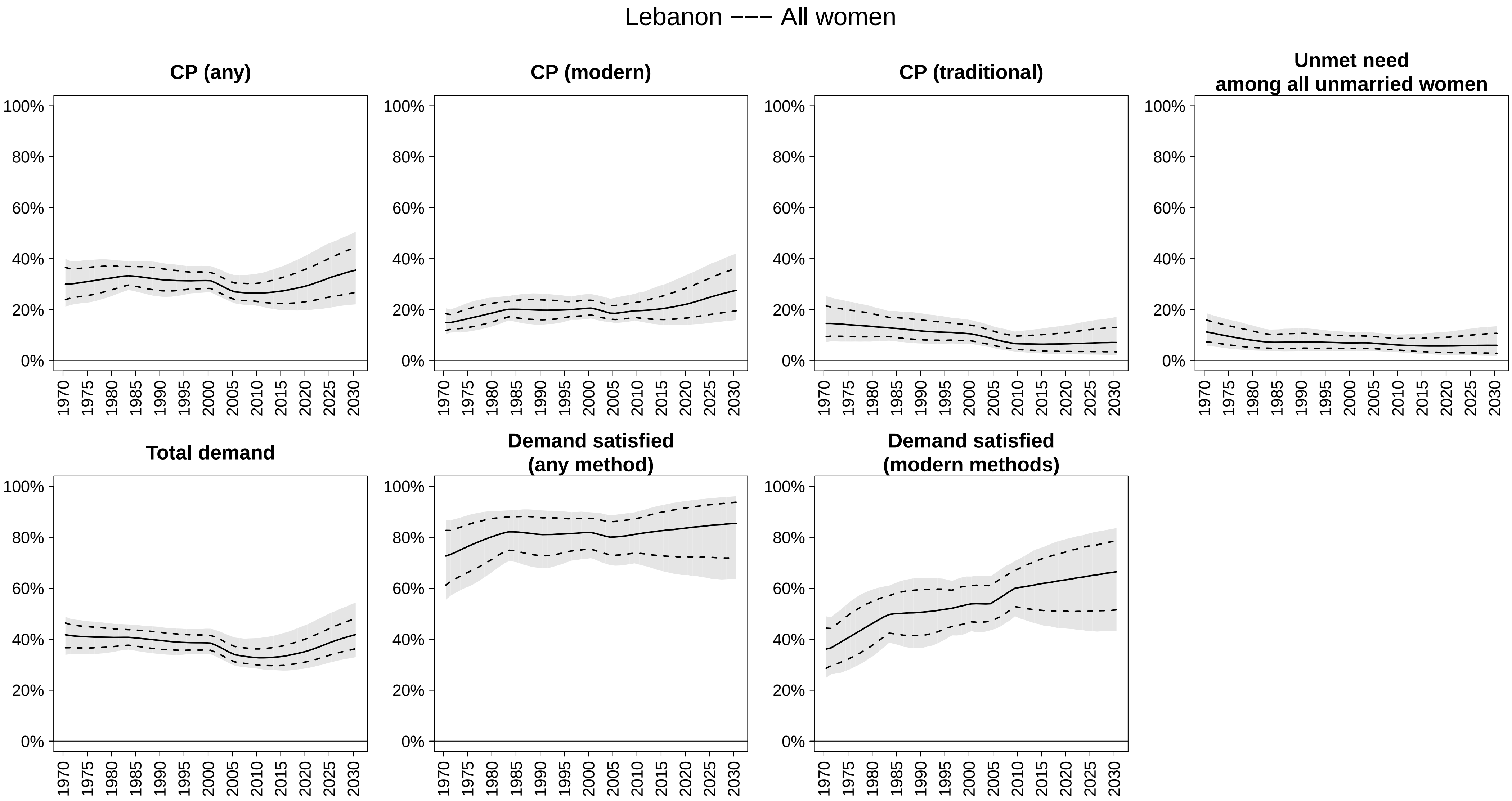
Lebanon −−− All women
CP (any)
CP (modern)
CP (traditional)
Unmet need among all unmarried women
Total demand
Demand satisfied (any method)
Demand satisfied (modern methods)
0%
20%
40%
60%
80%
100%
1970
1975
1980
1985
1990
1995
2000
2005
2010
2015
2020
2025
2030

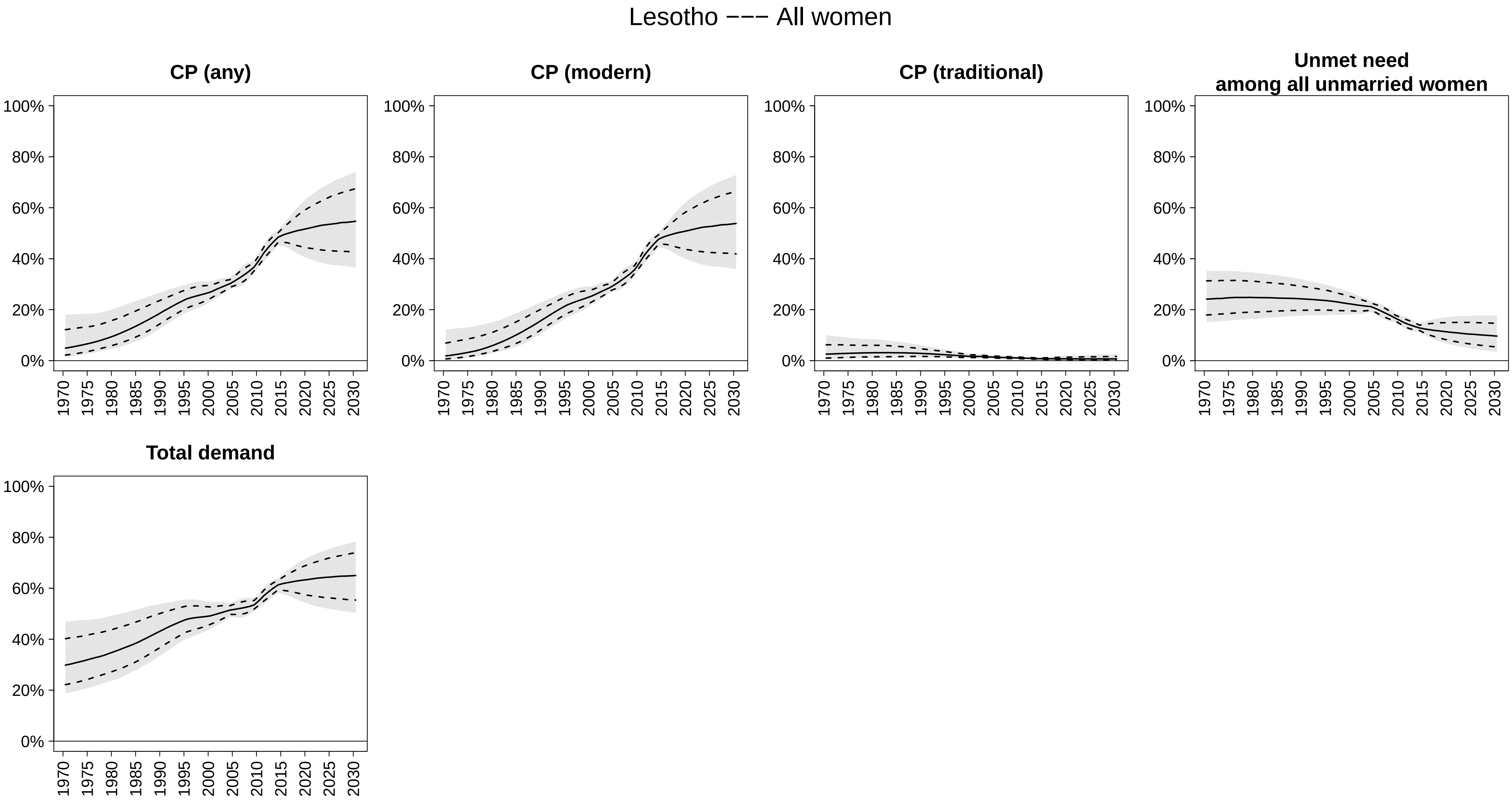
Lesotho −−− All women
CP (any)
CP (modern)
CP (traditional)
Unmet need among all unmarried women
Total demand
0%
20%
40%
60%
80%
100%
1970
1975
1980
1985
1990
1995
2000
2005
2010
2015
2020
2025
2030

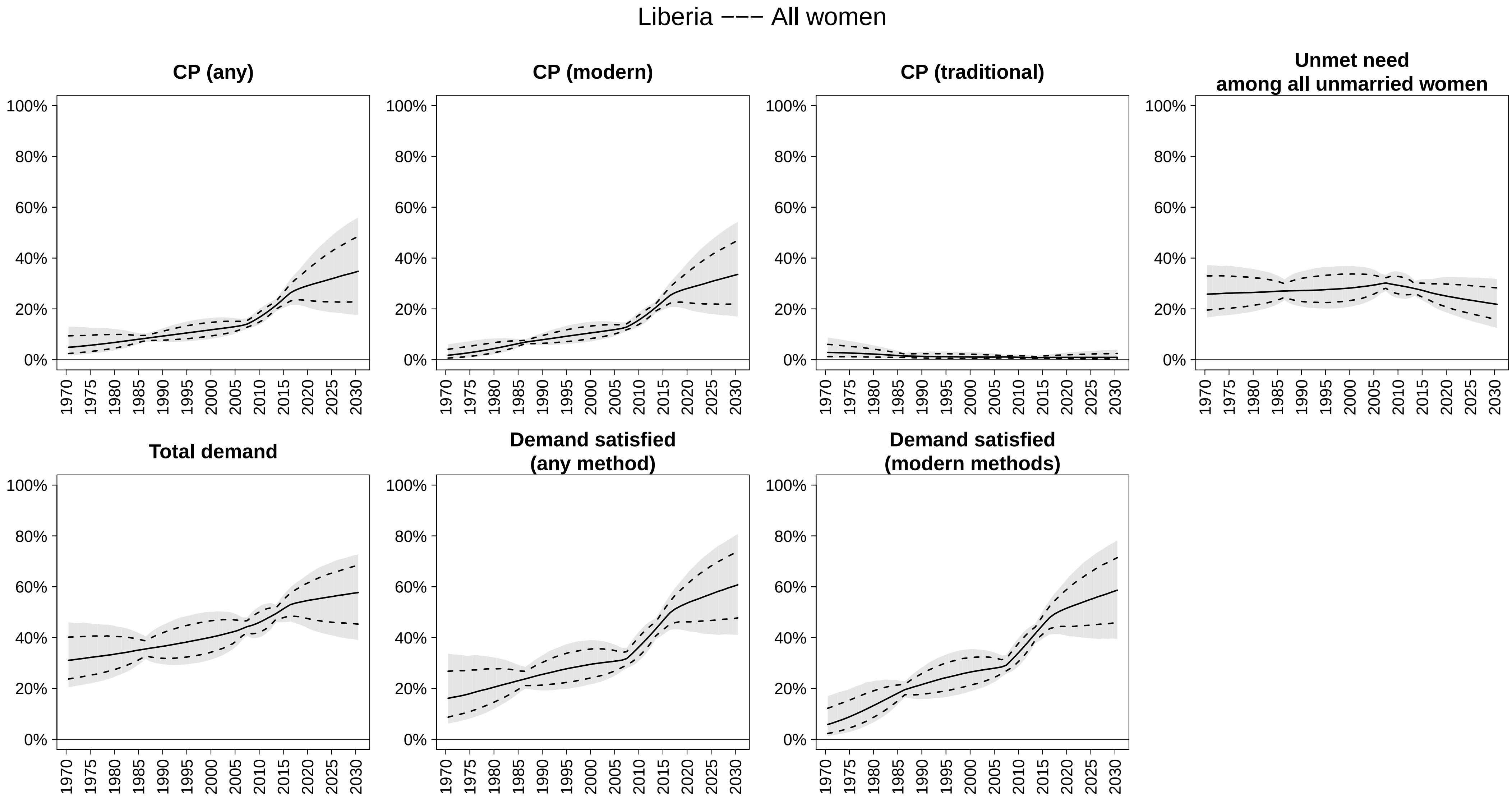
Liberia −−− All women
CP (any)
CP (modern)
CP (traditional)
Unmet need among all unmarried women
Total demand
Demand satisfied (any method)
Demand satisfied (modern methods)
0%
20%
40%
60%
80%
100%
1970
1975
1980
1985
1990
1995
2000
2005
2010
2015
2020
2025
2030

Libya −−− All women

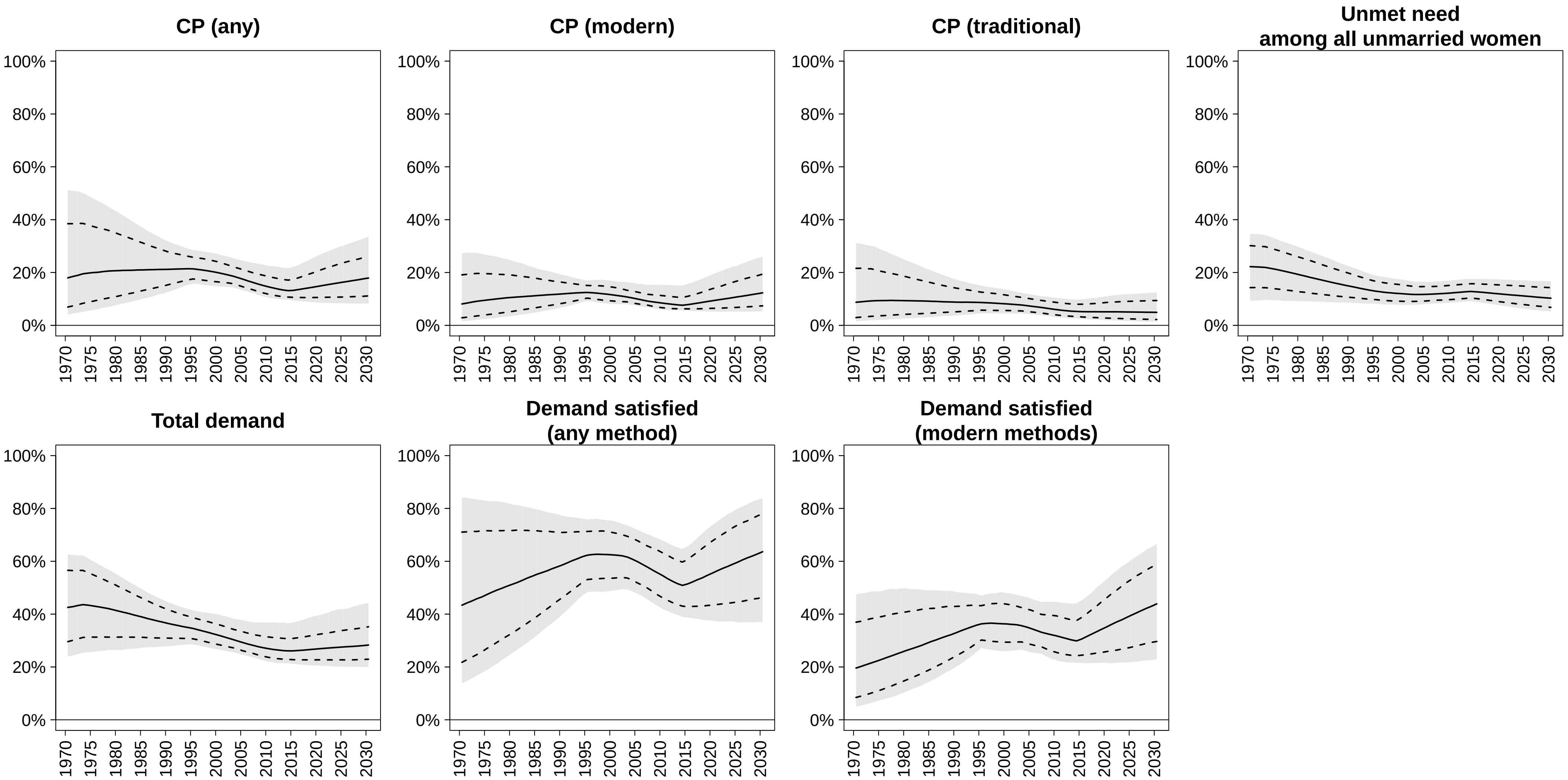

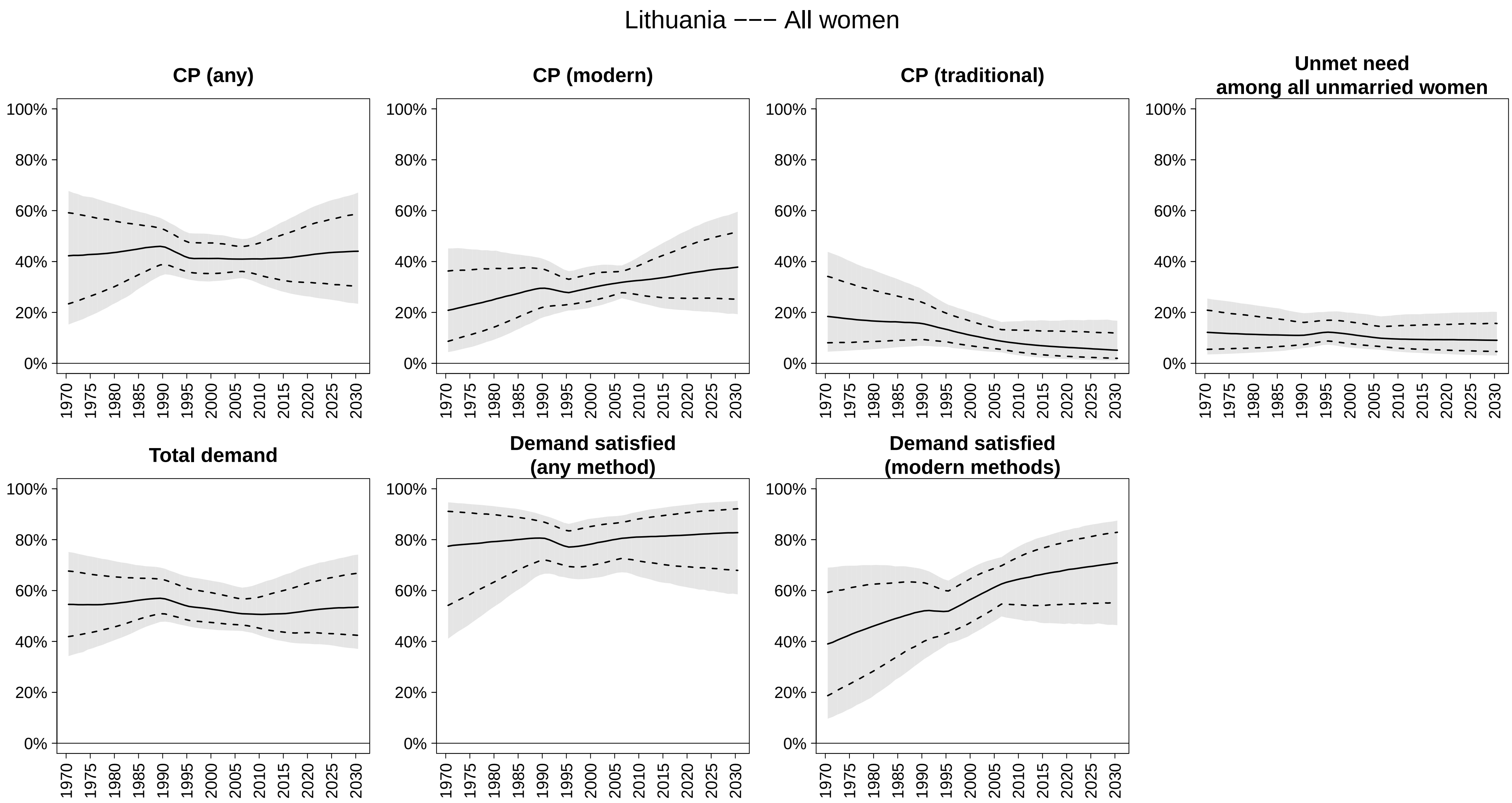
Lithuania −−− All women
CP (any)
CP (modern)
CP (traditional)
Unmet need
among all unmarried women
Total demand
Demand satisfied
(any method)
Demand satisfied
(modern methods)
0%
20%
40%
60%
80%
100%
1970
1975
1980
1985
1990
1995
2000
2005
2010
2015
2020
2025
2030

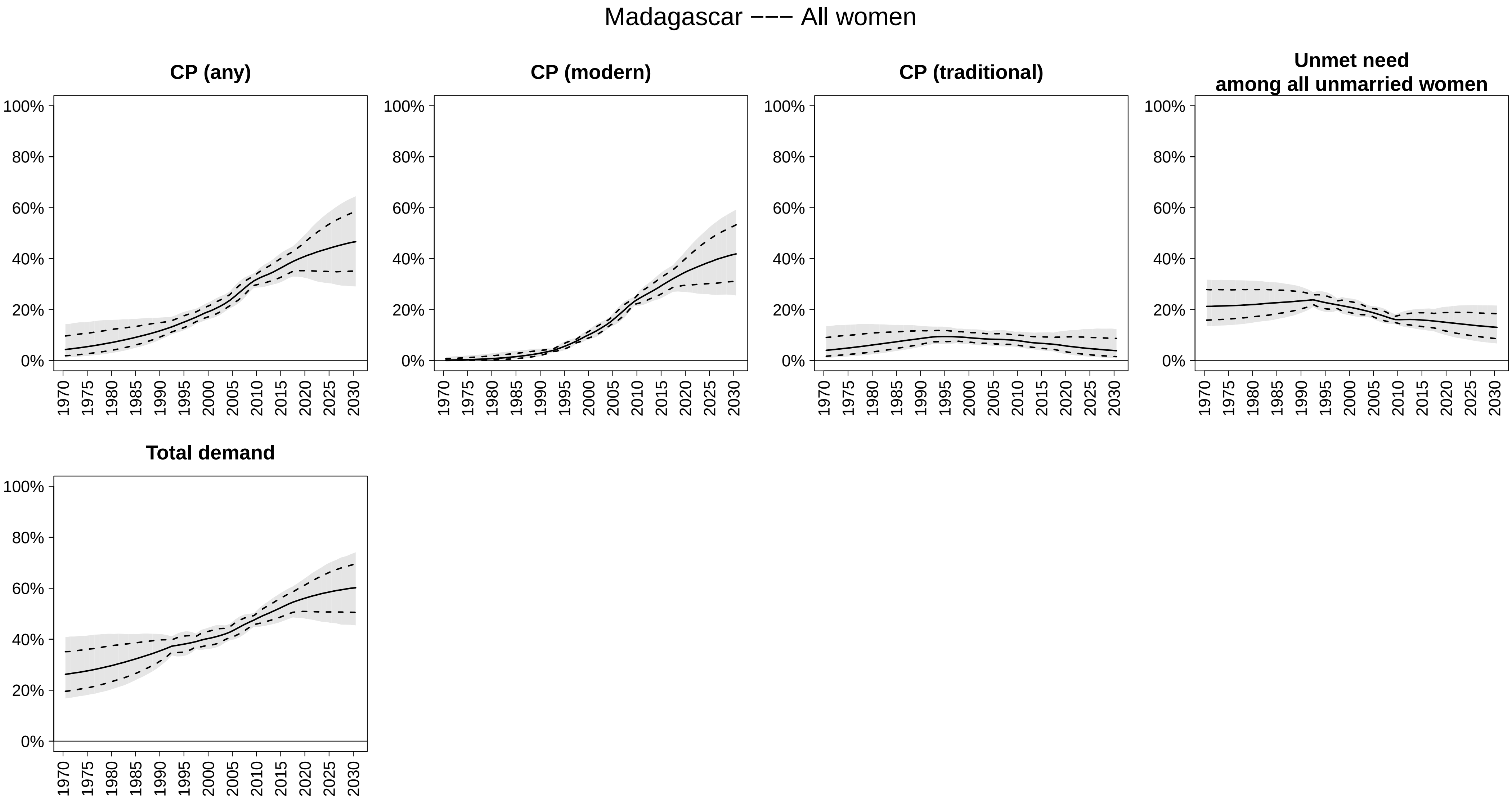
Madagascar −−− All women
CP (any)
CP (modern)
CP (traditional)
Unmet need
among all unmarried women
Total demand
100%
80%
60%
40%
20%
0%
1970
1975
1980
1985
1990
1995
2000
2005
2010
2015
2020
2025
2030

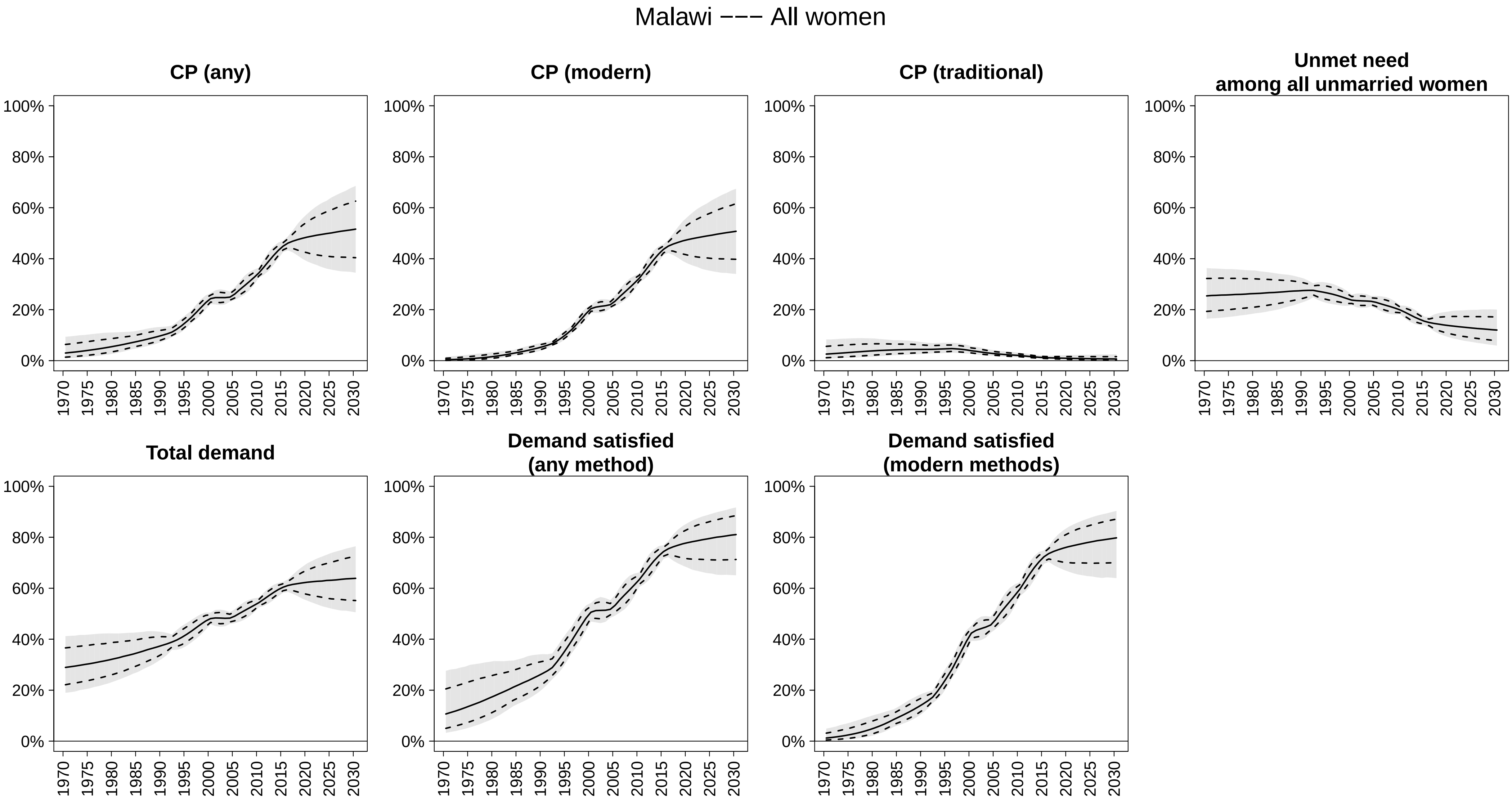
Malawi −−− All women
CP (any)
CP (modern)
CP (traditional)
Unmet need
among all unmarried women
Total demand
Demand satisfied
(any method)
Demand satisfied
(modern methods)
0%
20%
40%
60%
80%
100%
1970
1975
1980
1985
1990
1995
2000
2005
2010
2015
2020
2025
2030

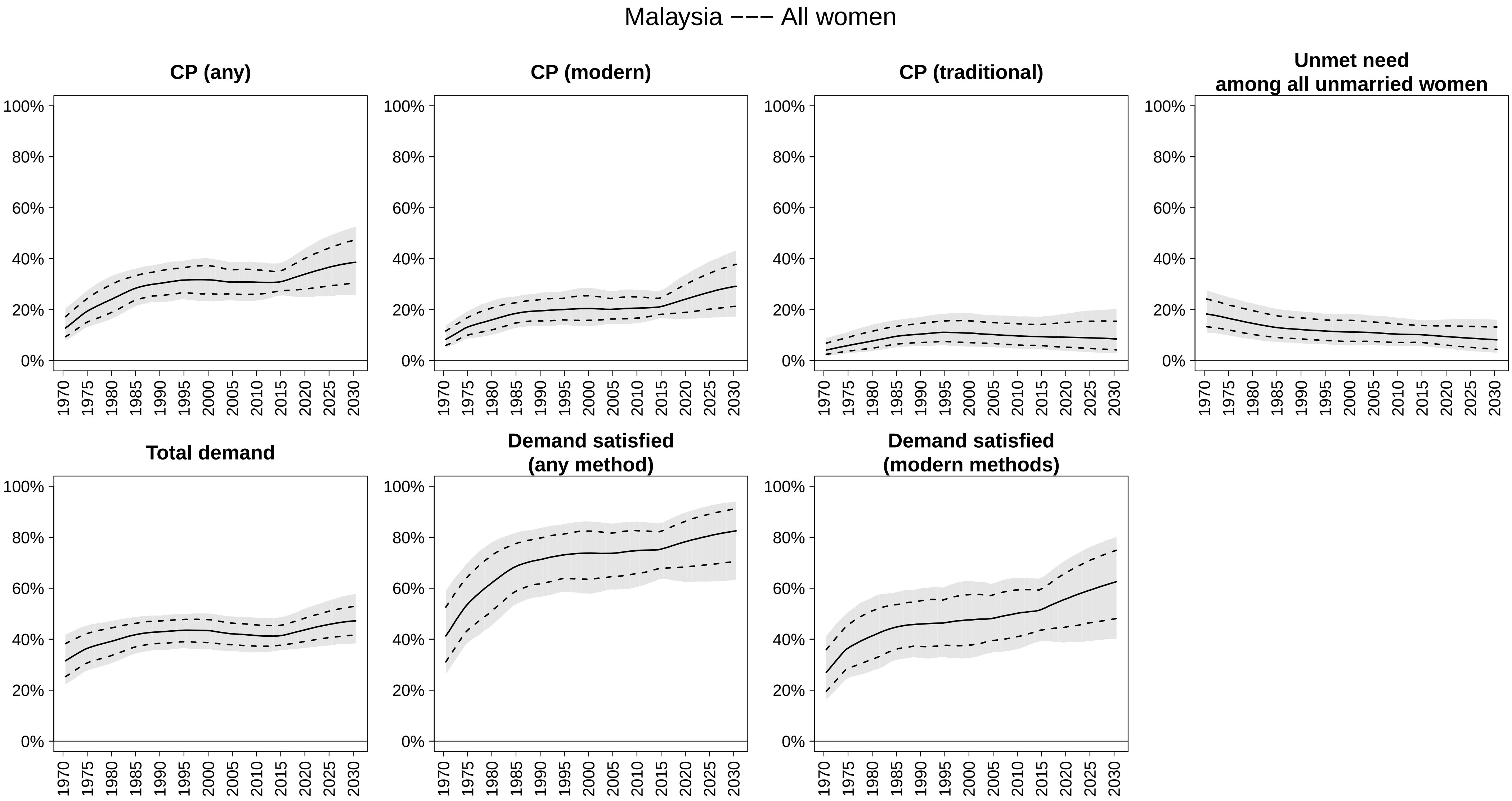
Malaysia −−− All women
CP (any)
CP (modern)
CP (traditional)
Unmet need among all unmarried women
Total demand
Demand satisfied (any method)
Demand satisfied (modern methods)
0%
20%
40%
60%
80%
100%
1970
1975
1980
1985
1990
1995
2000
2005
2010
2015
2020
2025
2030

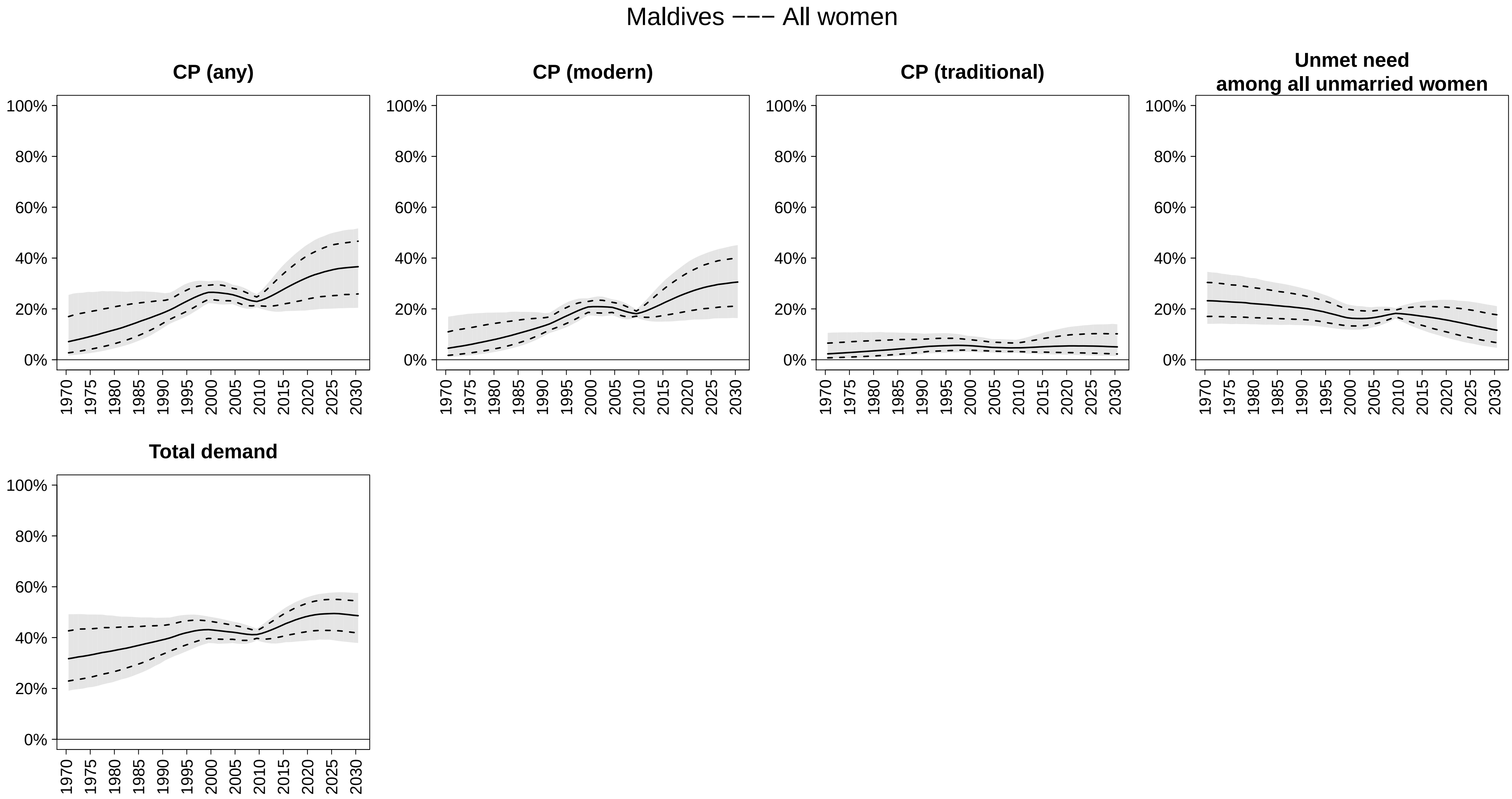
Maldives −−− All women
CP (any)
CP (modern)
CP (traditional)
Unmet need
among all unmarried women
Total demand
100%
80%
60%
40%
20%
0%
1970
1975
1980
1985
1990
1995
2000
2005
2010
2015
2020
2025
2030

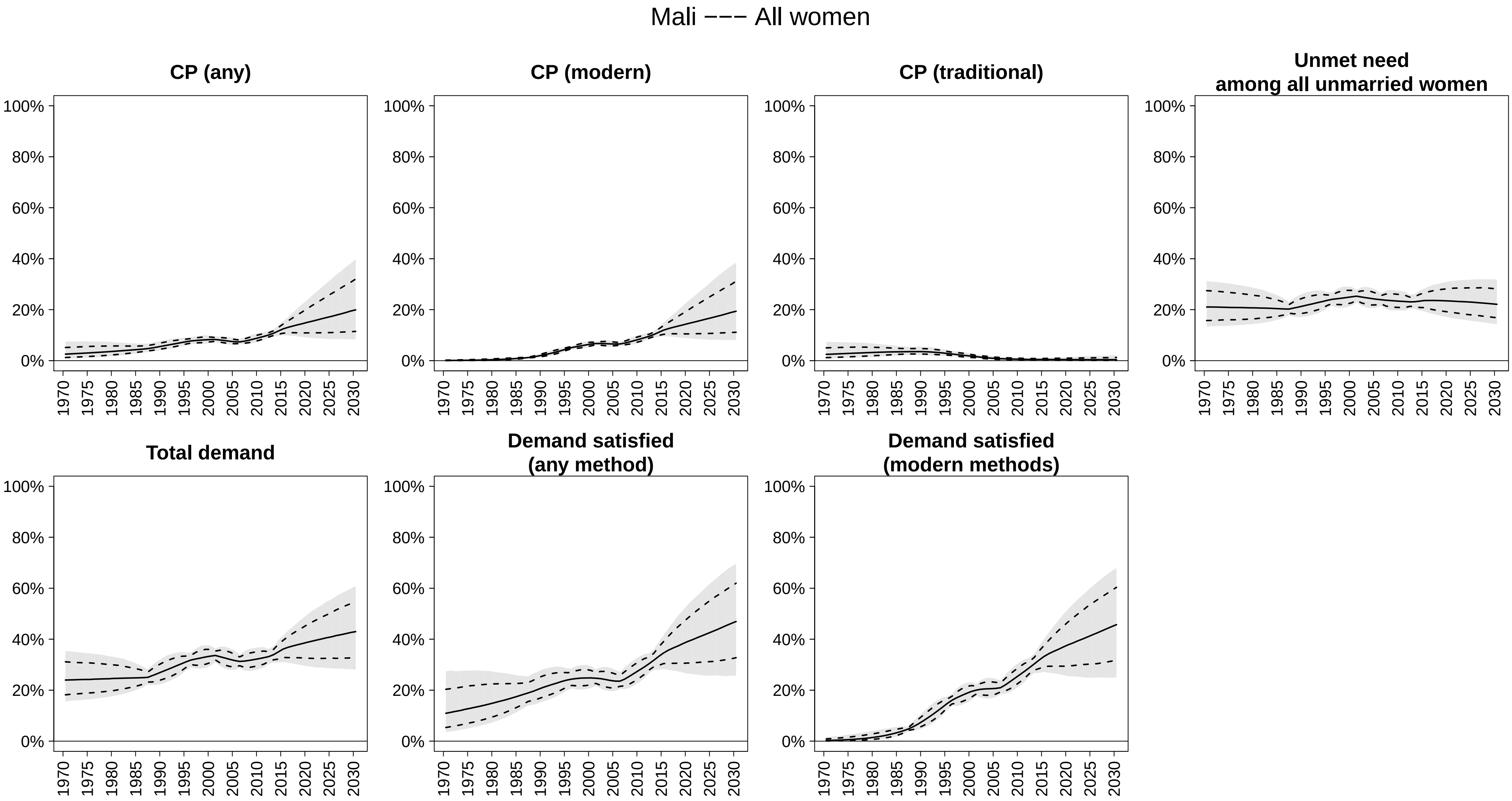
Mali −−− All women
CP (any)
CP (modern)
CP (traditional)
Unmet need
among all unmarried women
Total demand
Demand satisfied
(any method)
Demand satisfied
(modern methods)
0%
20%
40%
60%
80%
100%
1970
1975
1980
1985
1990
1995
2000
2005
2010
2015
2020
2025
2030

Malta −−− All women

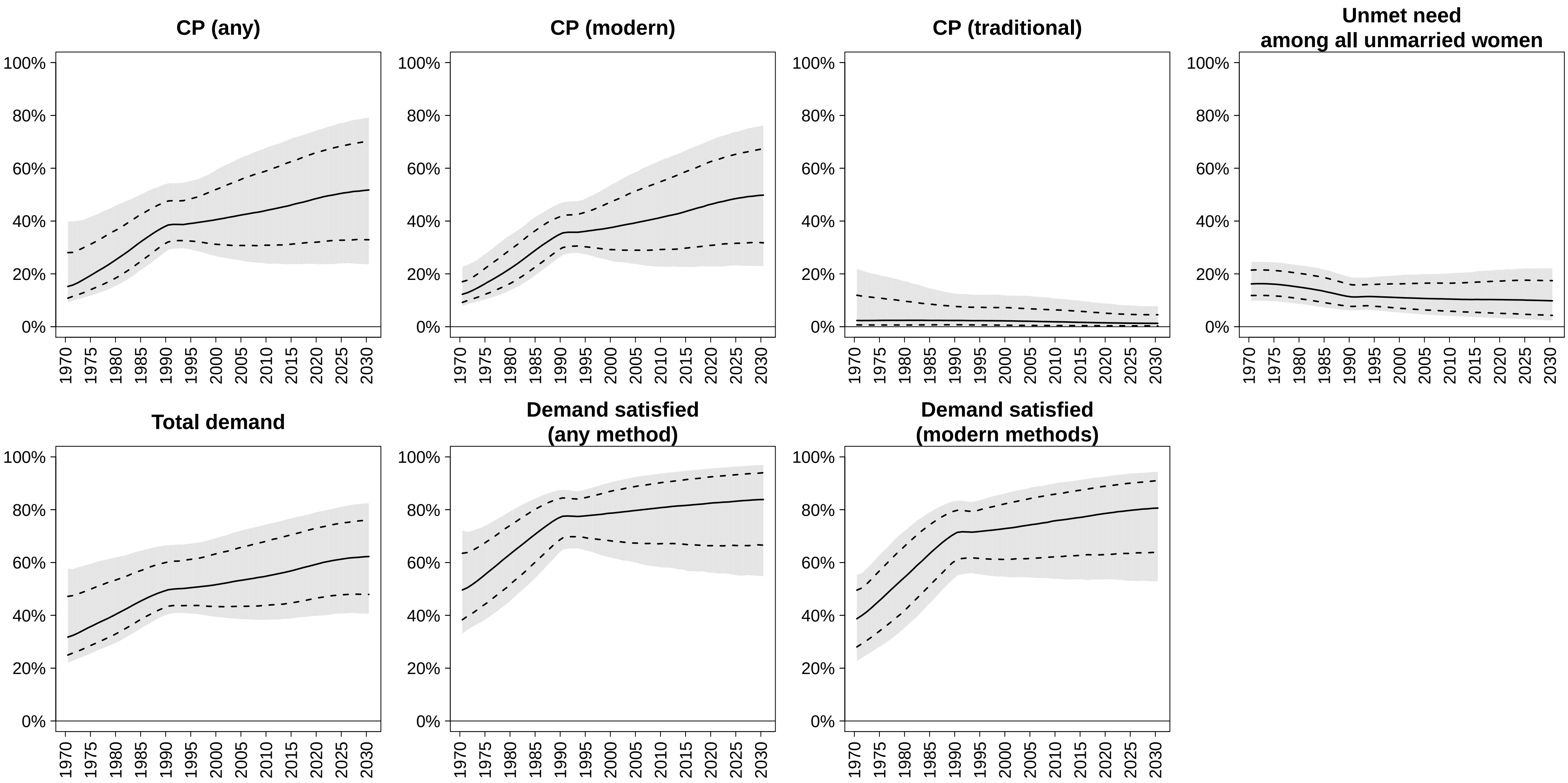

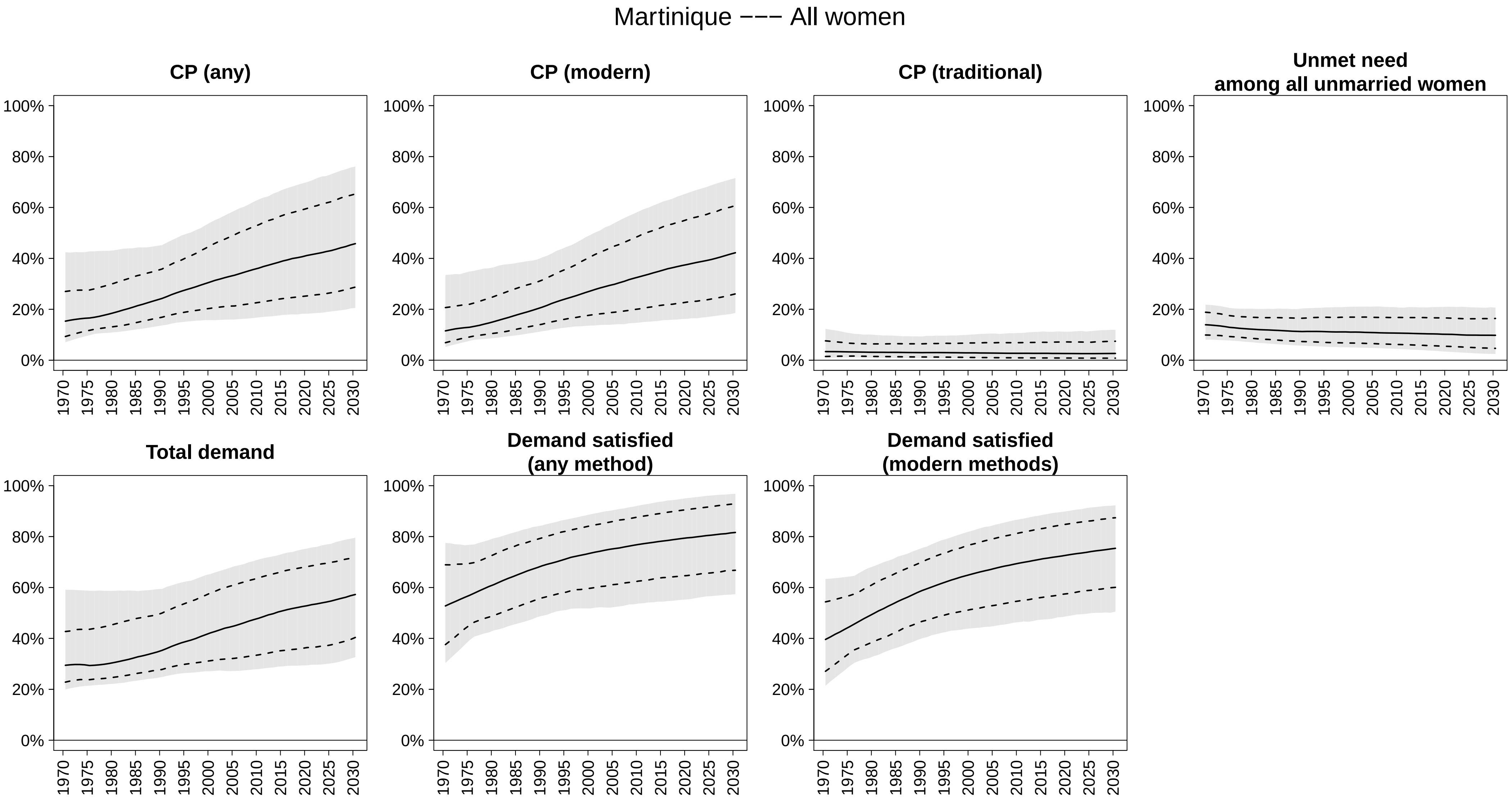
Martinique −−− All women
CP (any)
CP (modern)
CP (traditional)
Unmet need
among all unmarried women
Total demand
Demand satisfied
(any method)
Demand satisfied
(modern methods)
0%
20%
40%
60%
80%
100%
1970
1975
1980
1985
1990
1995
2000
2005
2010
2015
2020
2025
2030

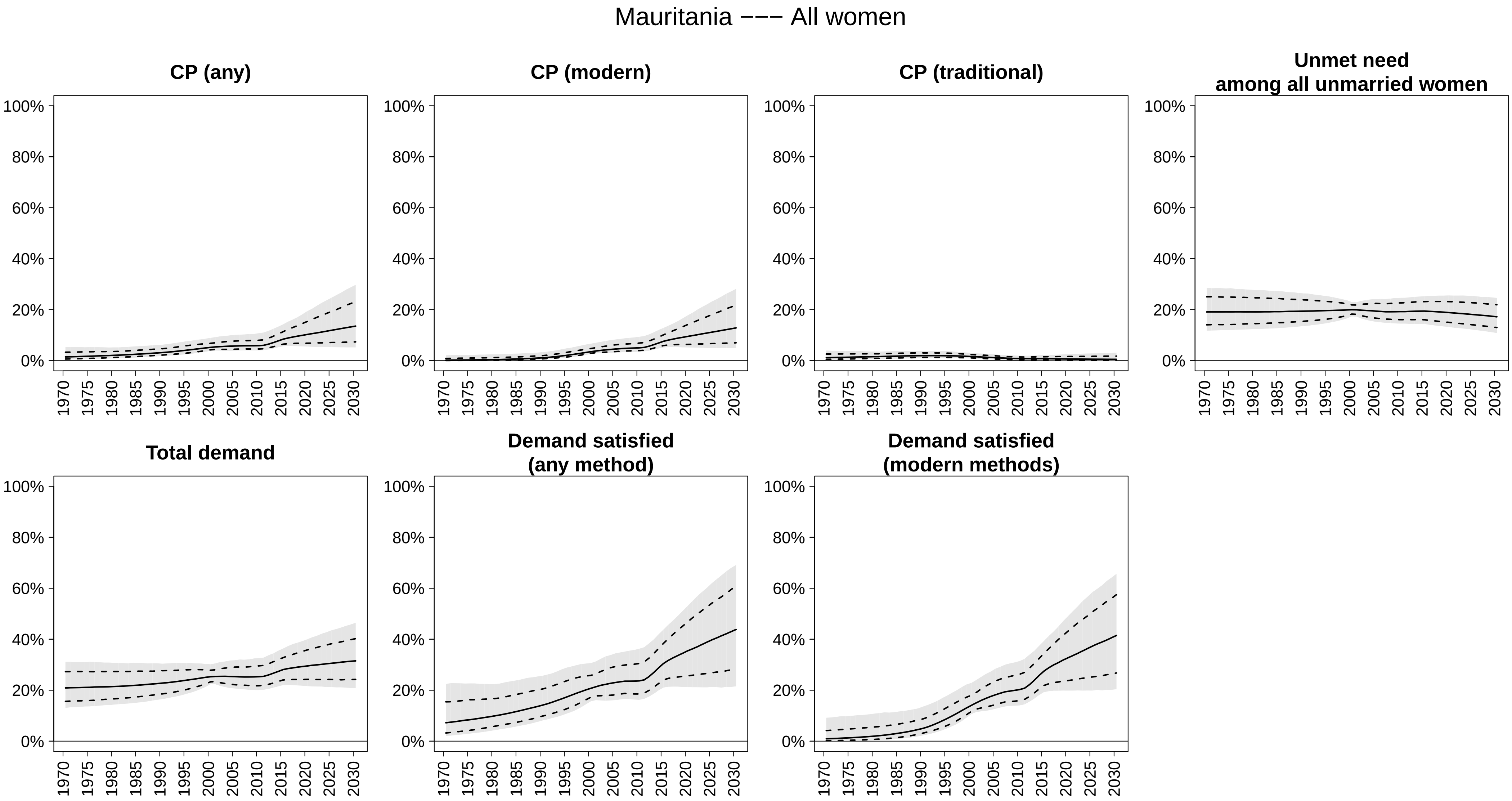
Mauritania −−− All women
CP (any)
CP (modern)
CP (traditional)
Unmet need
among all unmarried women
Total demand
Demand satisfied
(any method)
Demand satisfied
(modern methods)
0%
20%
40%
60%
80%
100%
1970
1975
1980
1985
1990
1995
2000
2005
2010
2015
2020
2025
2030

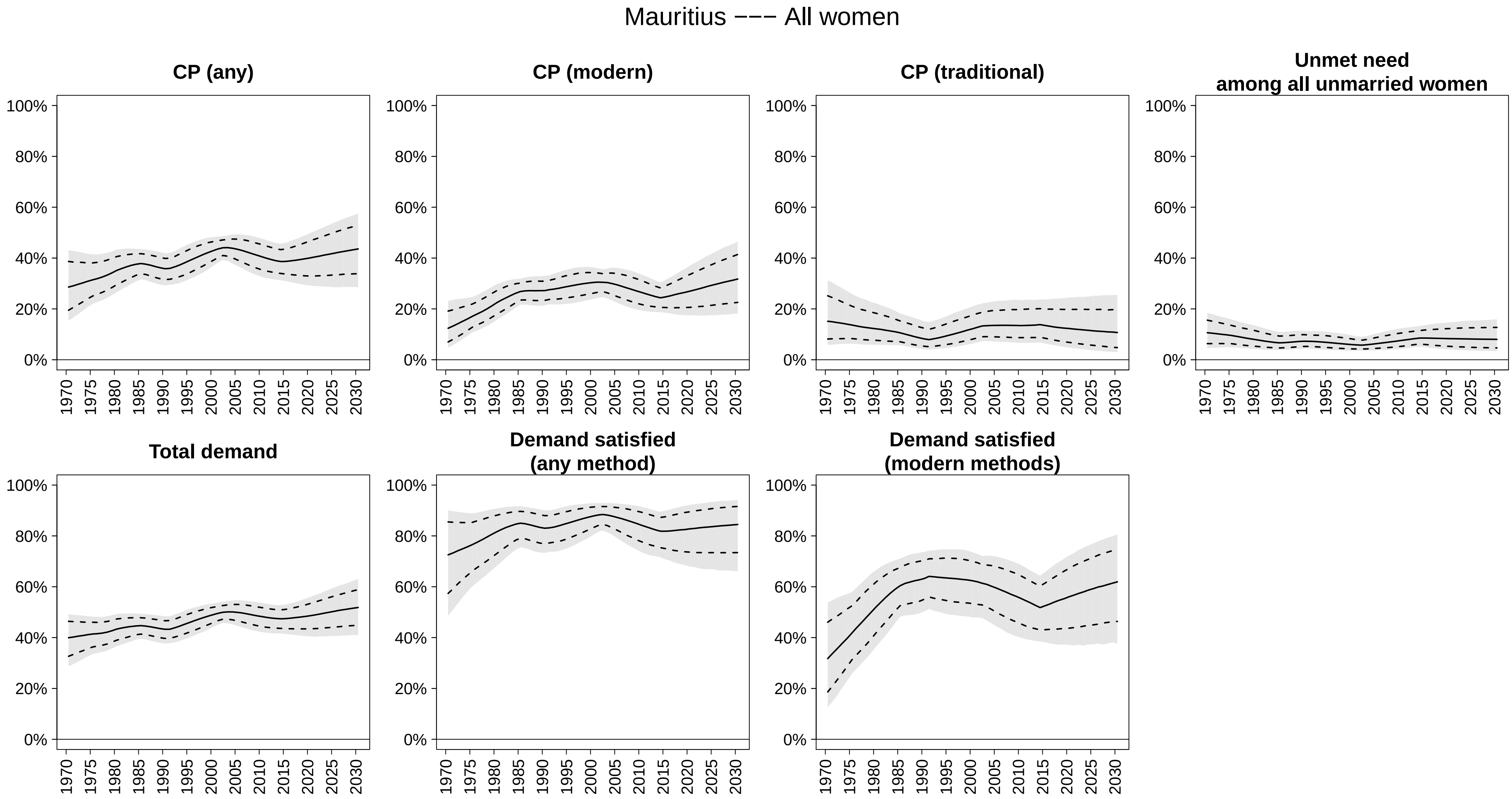
Mauritius −−− All women
CP (any)
CP (modern)
CP (traditional)
Unmet need among all unmarried women
Total demand
Demand satisfied (any method)
Demand satisfied (modern methods)
0%
20%
40%
60%
80%
100%
1970
1975
1980
1985
1990
1995
2000
2005
2010
2015
2020
2025
2030

Mexico −−− All women

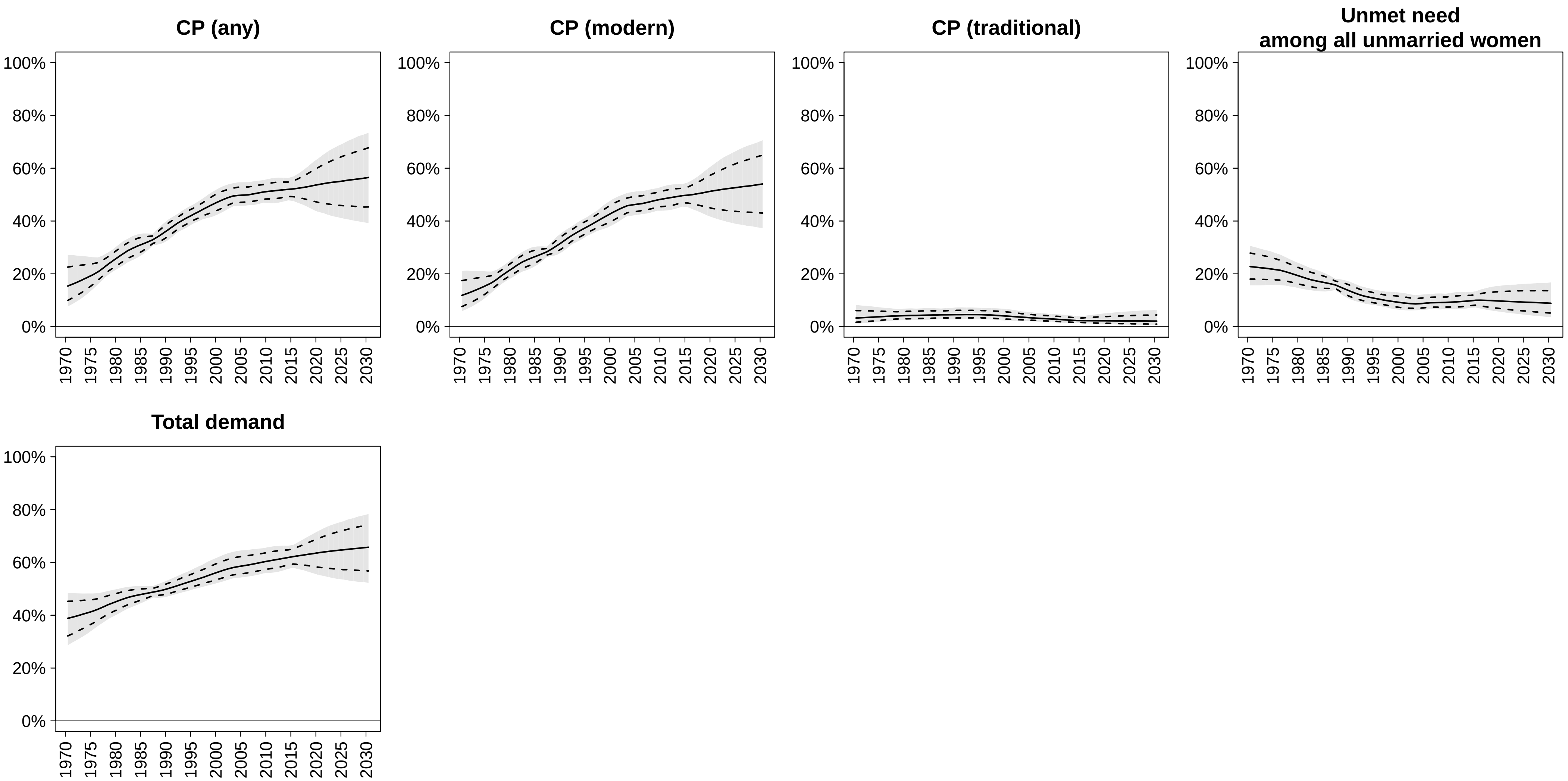

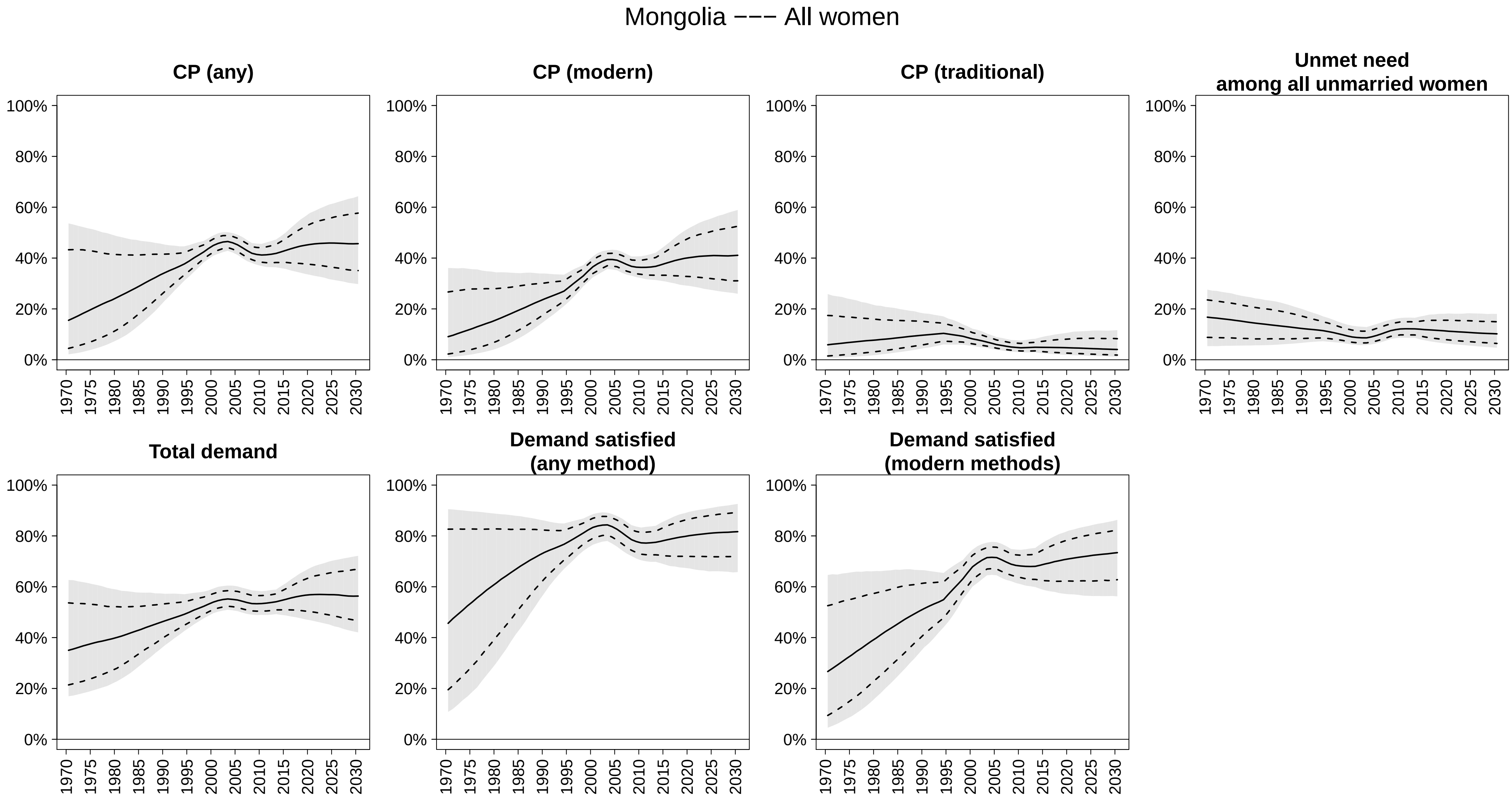
Mongolia −−− All women
CP (any)
CP (modern)
CP (traditional)
Unmet need among all unmarried women
Total demand
Demand satisfied (any method)
Demand satisfied (modern methods)
0%
20%
40%
60%
80%
100%
1970
1975
1980
1985
1990
1995
2000
2005
2010
2015
2020
2025
2030

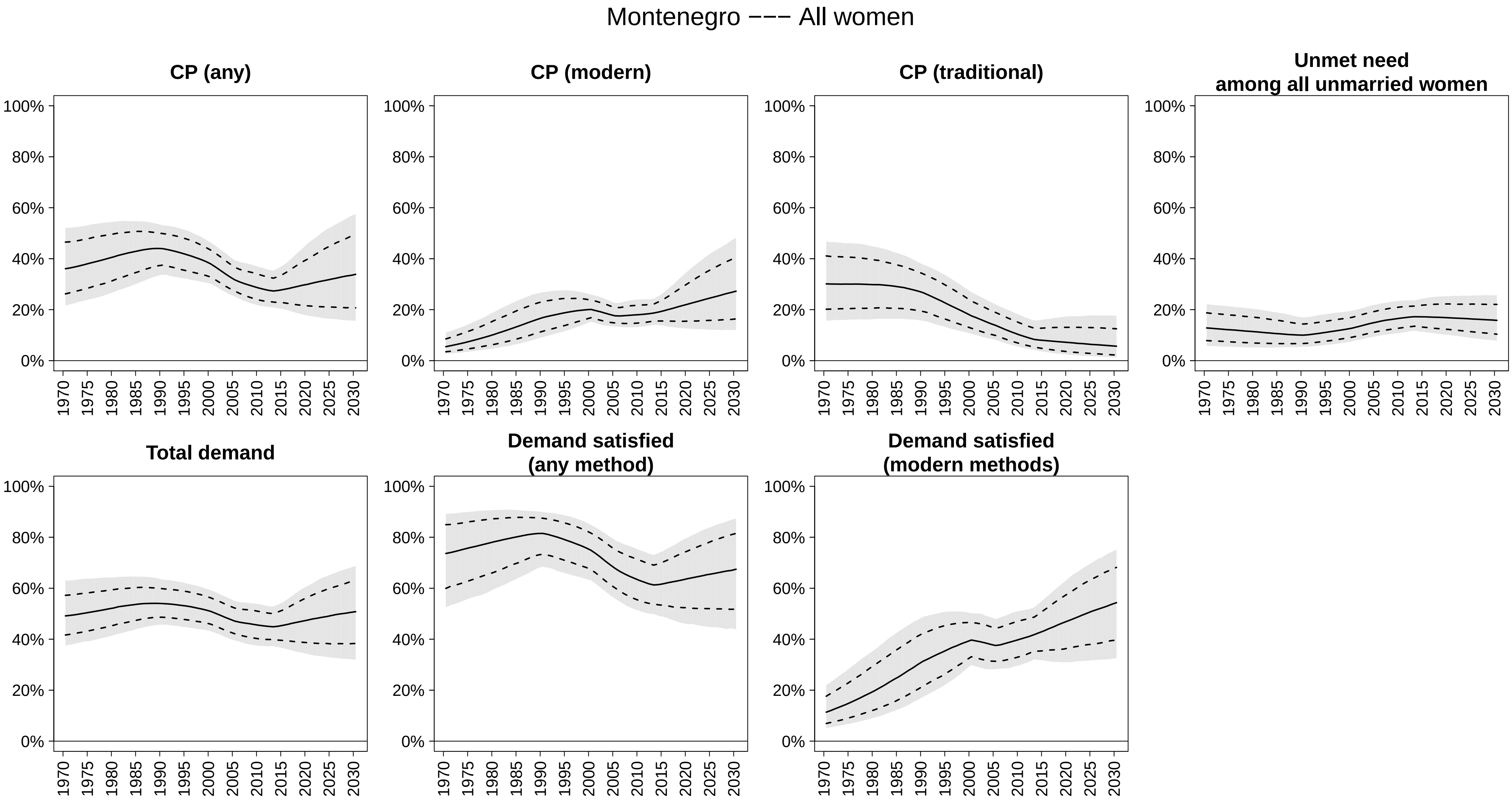
Montenegro −−− All women
CP (any)
CP (modern)
CP (traditional)
Unmet need
among all unmarried women
Total demand
Demand satisfied
(any method)
Demand satisfied
(modern methods)
0%
20%
40%
60%
80%
100%
1970
1975
1980
1985
1990
1995
2000
2005
2010
2015
2020
2025
2030

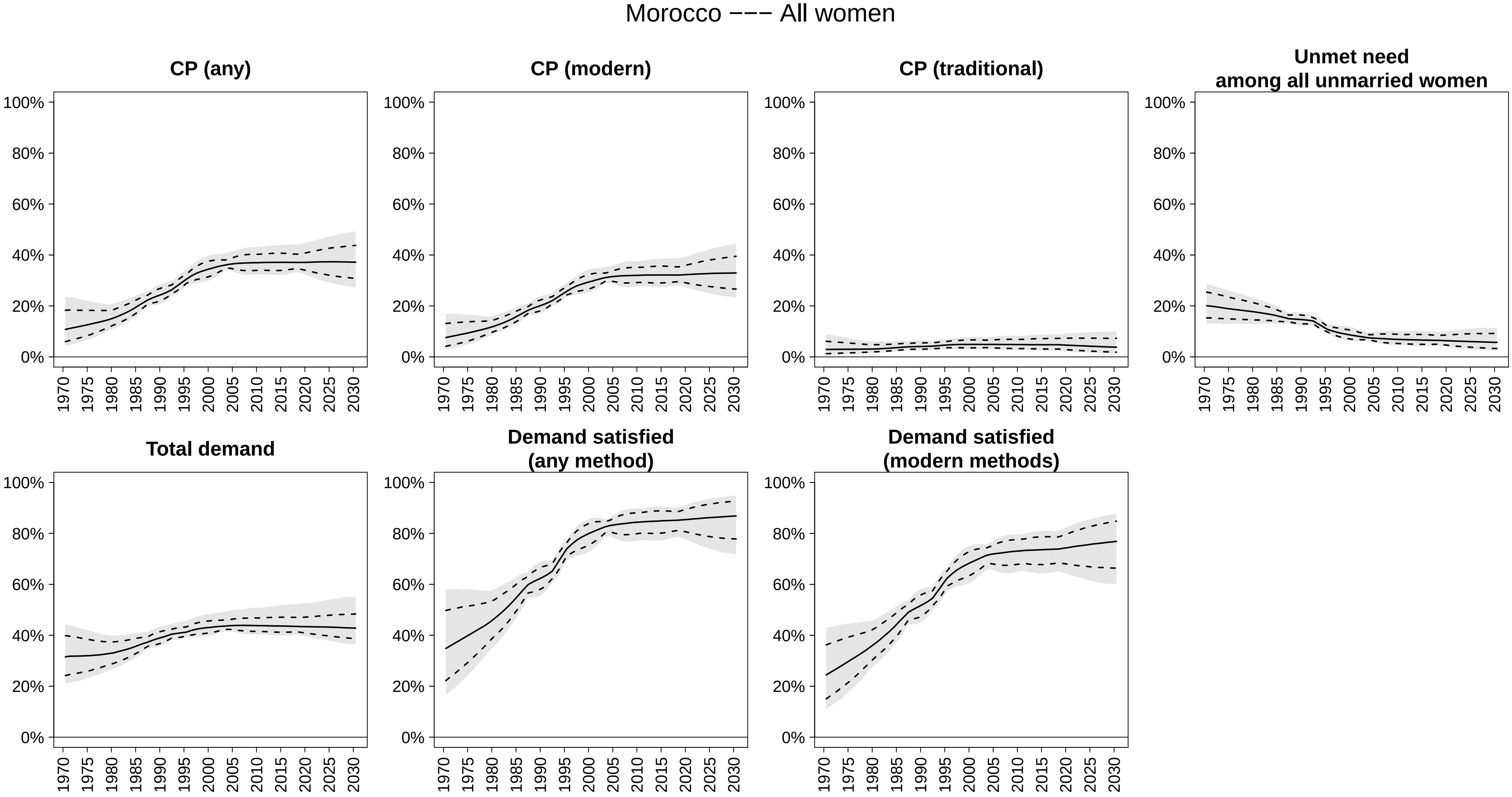
Morocco −−− All women
CP (any)
CP (modern)
CP (traditional)
Unmet need among all unmarried women
Total demand
Demand satisfied (any method)
Demand satisfied (modern methods)
0%
20%
40%
60%
80%
100%
1970
1975
1980
1985
1990
1995
2000
2005
2010
2015
2020
2025
2030

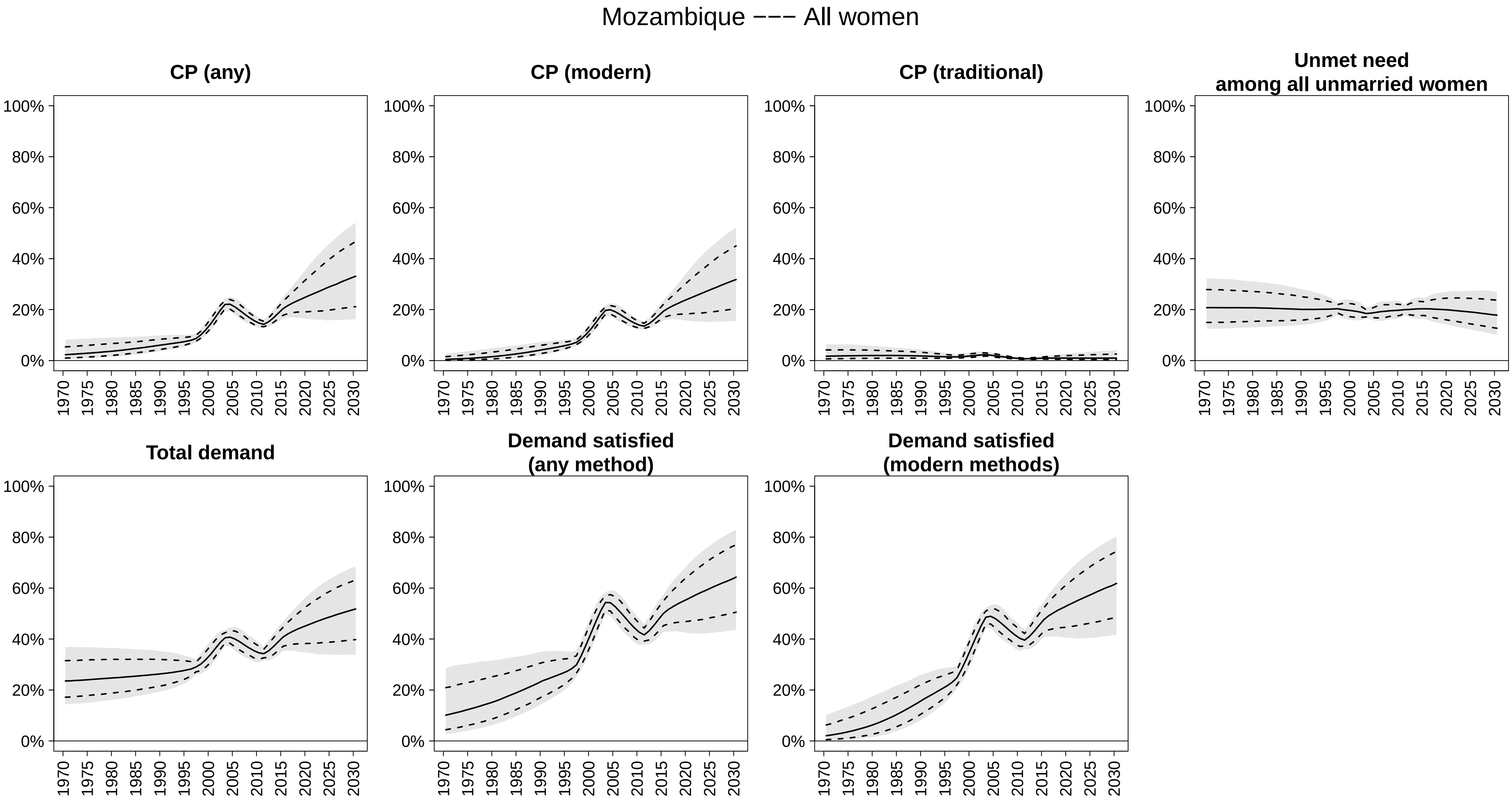
Mozambique −−− All women
CP (any)
CP (modern)
CP (traditional)
Unmet need
among all unmarried women
Total demand
Demand satisfied
(any method)
Demand satisfied
(modern methods)
100%
80%
60%
40%
20%
0%
1970
1975
1980
1985
1990
1995
2000
2005
2010
2015
2020
2025
2030

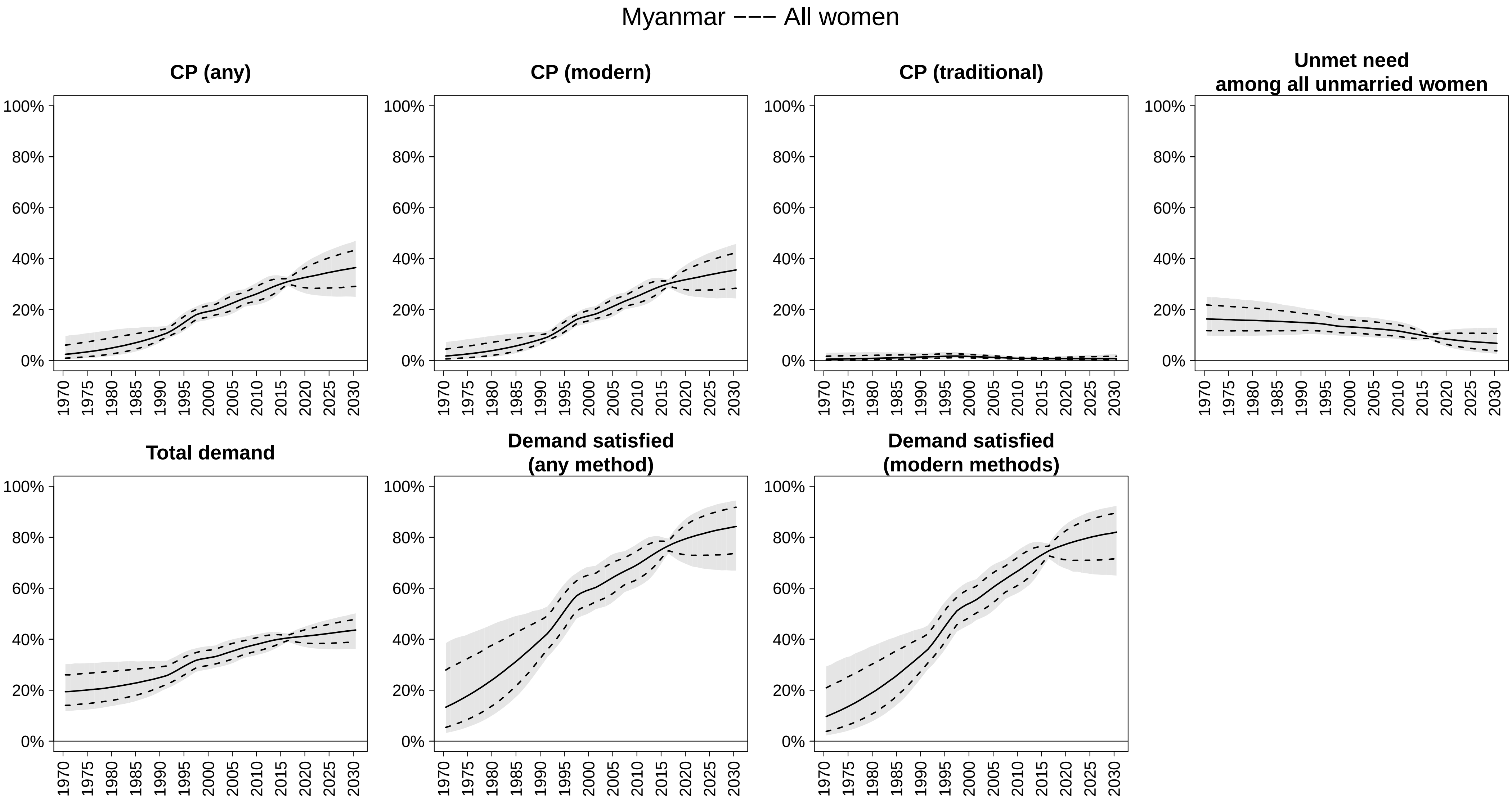
Myanmar −−− All women
CP (any)
CP (modern)
CP (traditional)
Unmet need among all unmarried women
Total demand
Demand satisfied (any method)
Demand satisfied (modern methods)
0%
20%
40%
60%
80%
100%
1970
1975
1980
1985
1990
1995
2000
2005
2010
2015
2020
2025
2030

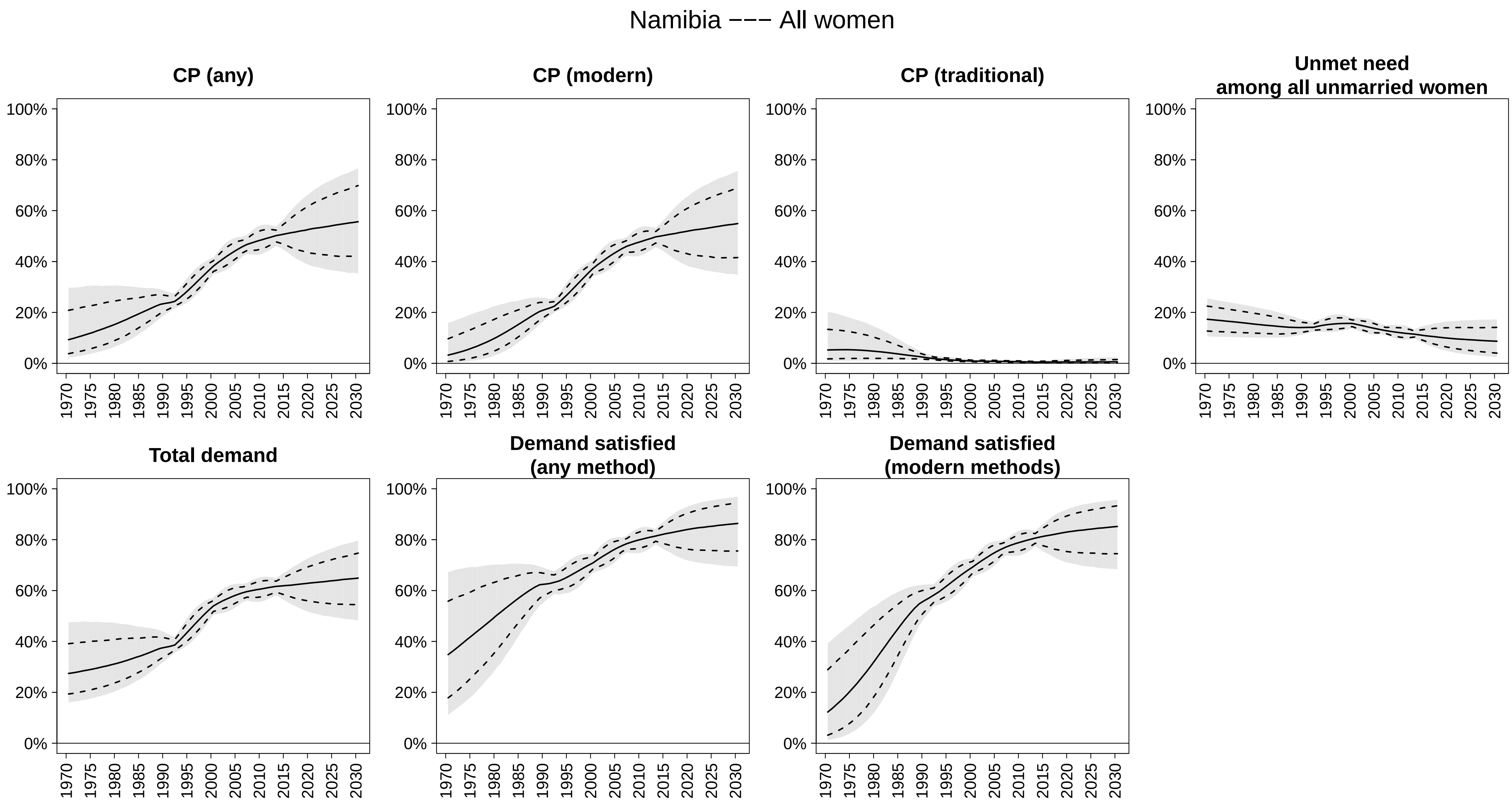
Namibia −−− All women
CP (any)
CP (modern)
CP (traditional)
Unmet need
among all unmarried women
Total demand
Demand satisfied
(any method)
Demand satisfied
(modern methods)
100%
80%
60%
40%
20%
0%
1970
1975
1980
1985
1990
1995
2000
2005
2010
2015
2020
2025
2030

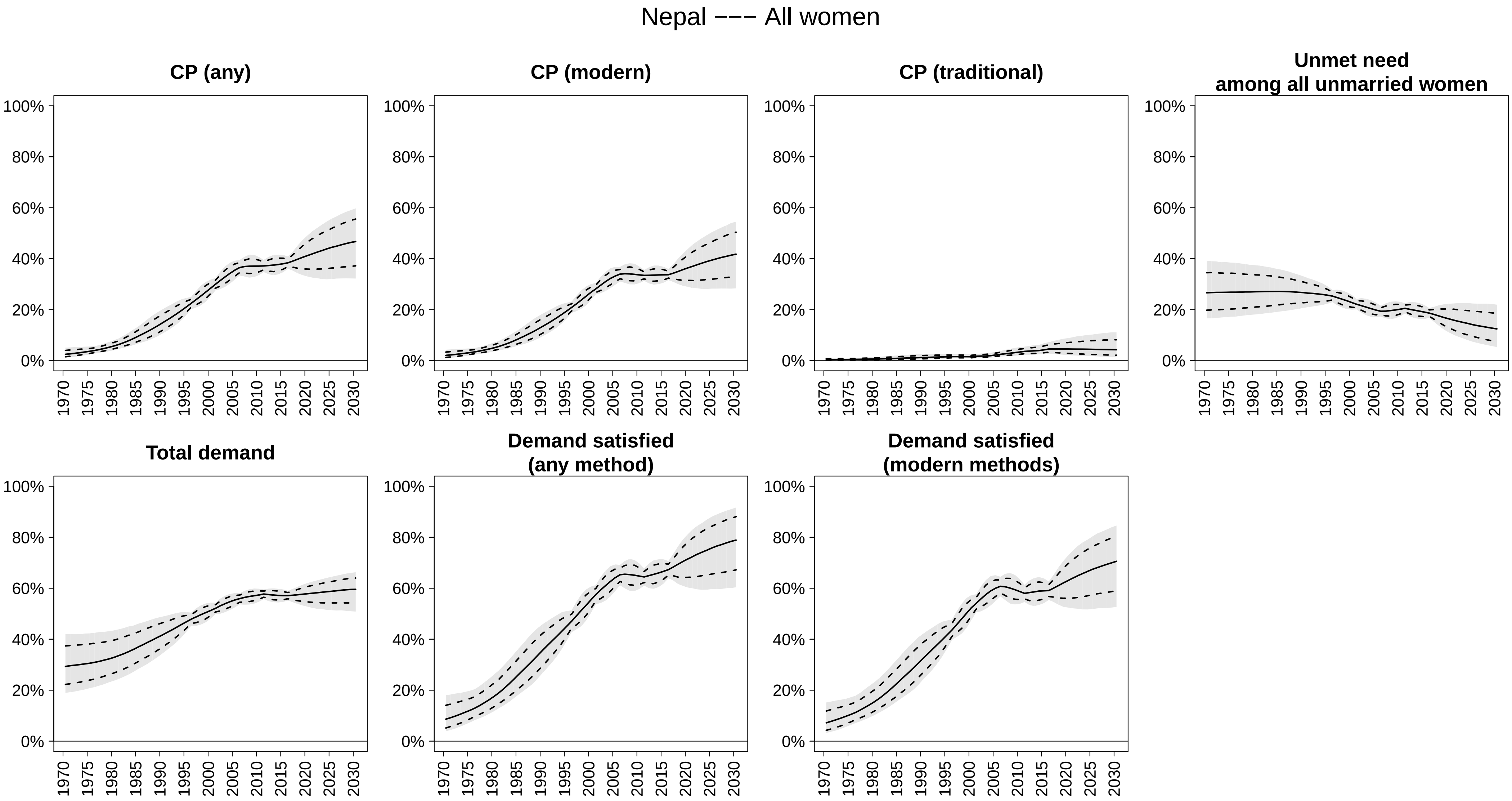
Nepal −−− All women
CP (any)
CP (modern)
CP (traditional)
Unmet need among all unmarried women
Total demand
Demand satisfied (any method)
Demand satisfied (modern methods)
0%
20%
40%
60%
80%
100%
1970
1975
1980
1985
1990
1995
2000
2005
2010
2015
2020
2025
2030

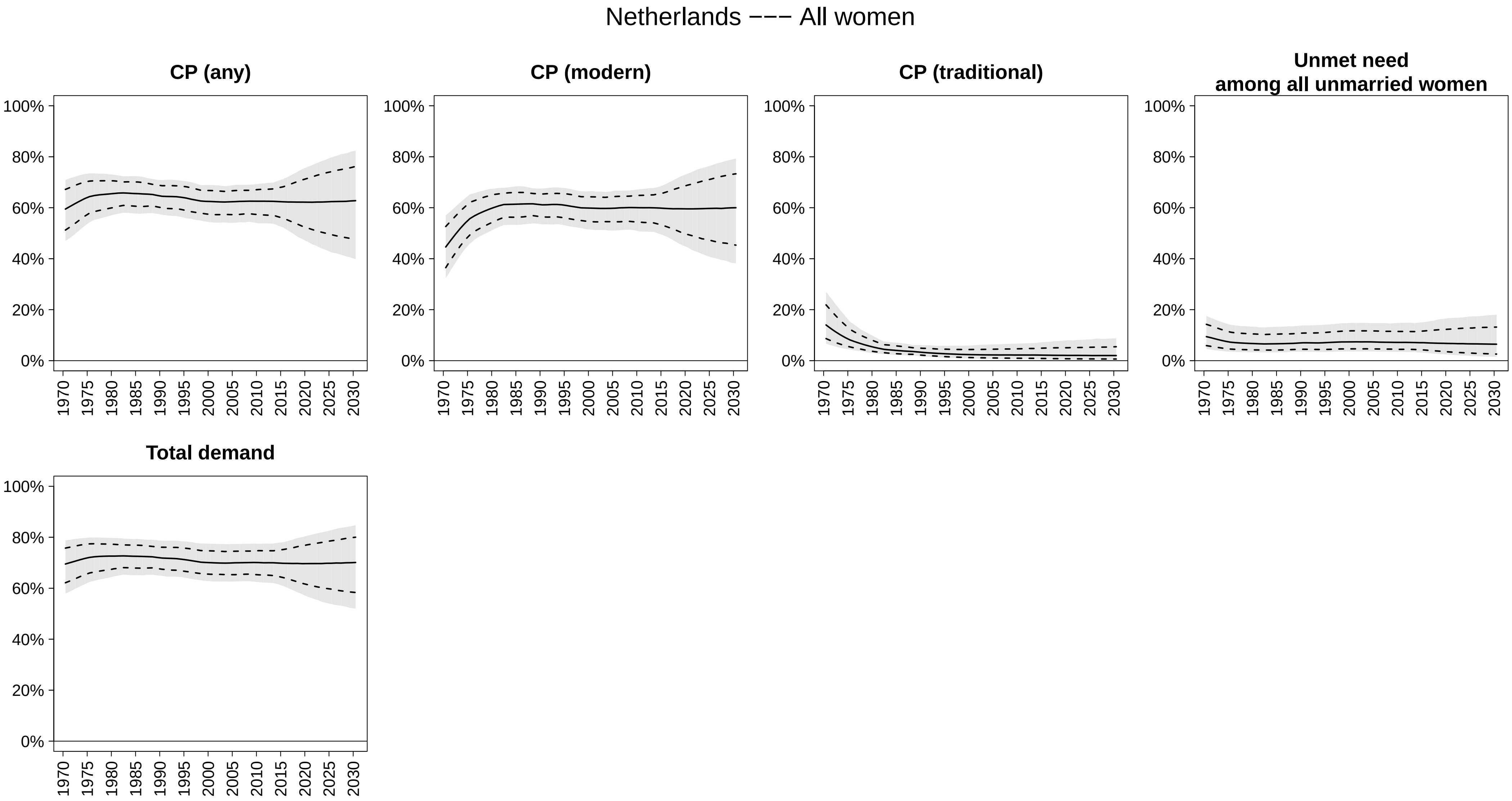
Netherlands −−− All women
CP (any)
CP (modern)
CP (traditional)
Unmet need
among all unmarried women
Total demand
100%
80%
60%
40%
20%
0%
1970
1975
1980
1985
1990
1995
2000
2005
2010
2015
2020
2025
2030

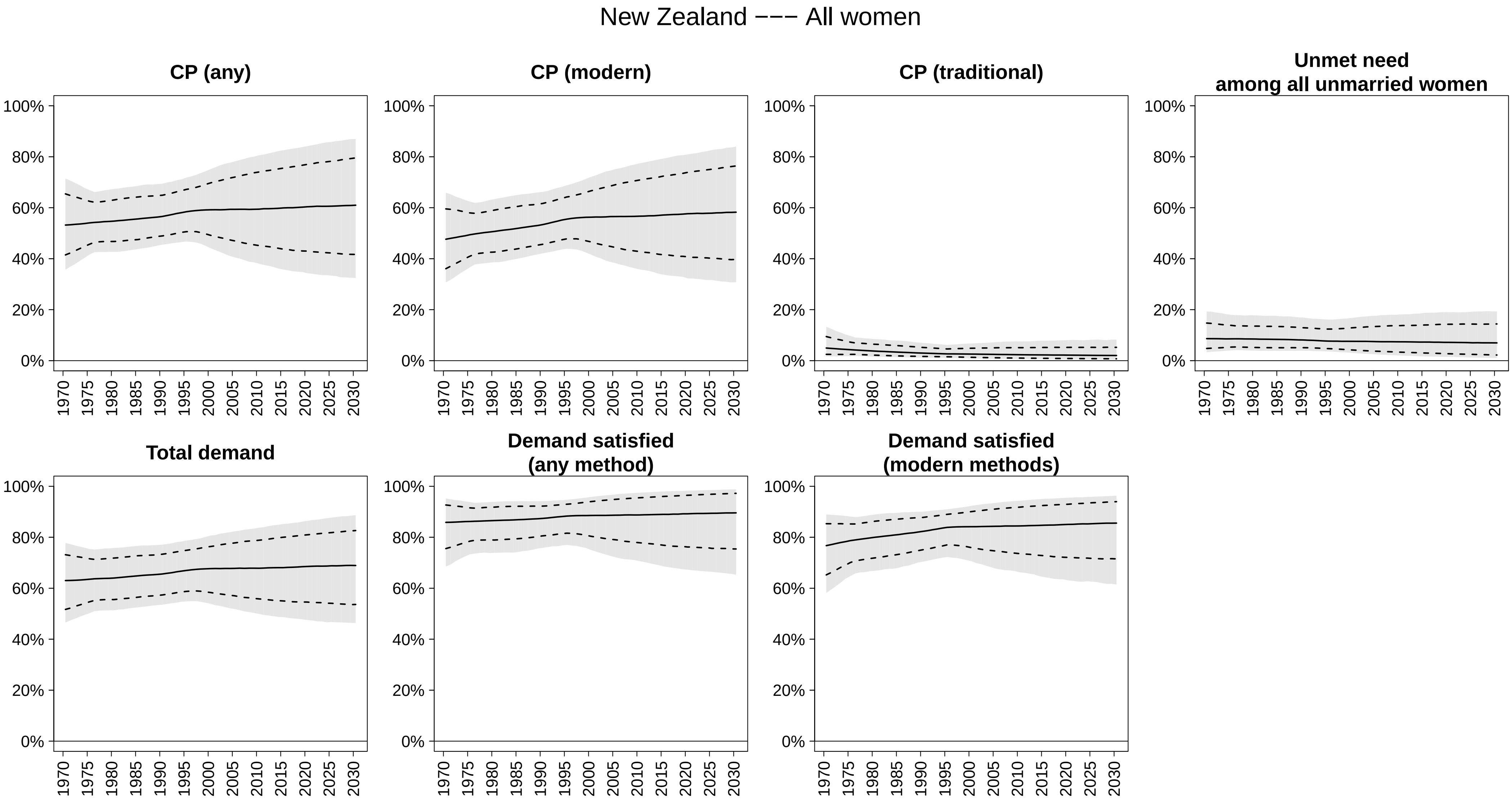
New Zealand −−− All women
CP (any)
CP (modern)
CP (traditional)
Unmet need
among all unmarried women
Total demand
Demand satisfied
(any method)
Demand satisfied
(modern methods)
100%
80%
60%
40%
20%
0%
1970
1975
1980
1985
1990
1995
2000
2005
2010
2015
2020
2025
2030

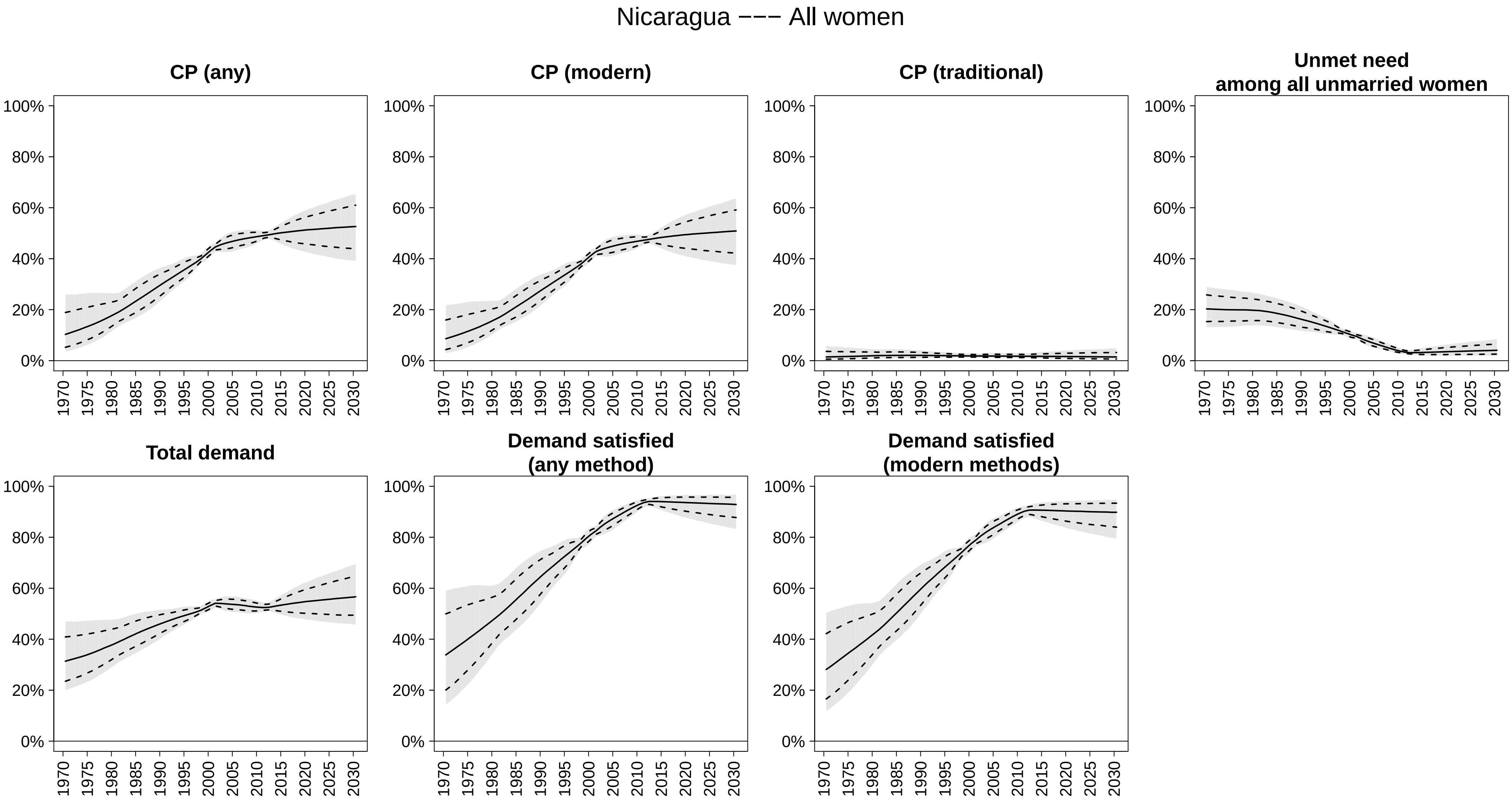
Nicaragua −−− All women
CP (any)
CP (modern)
CP (traditional)
Unmet need among all unmarried women
Total demand
Demand satisfied (any method)
Demand satisfied (modern methods)
0%
20%
40%
60%
80%
100%
1970
1975
1980
1985
1990
1995
2000
2005
2010
2015
2020
2025
2030

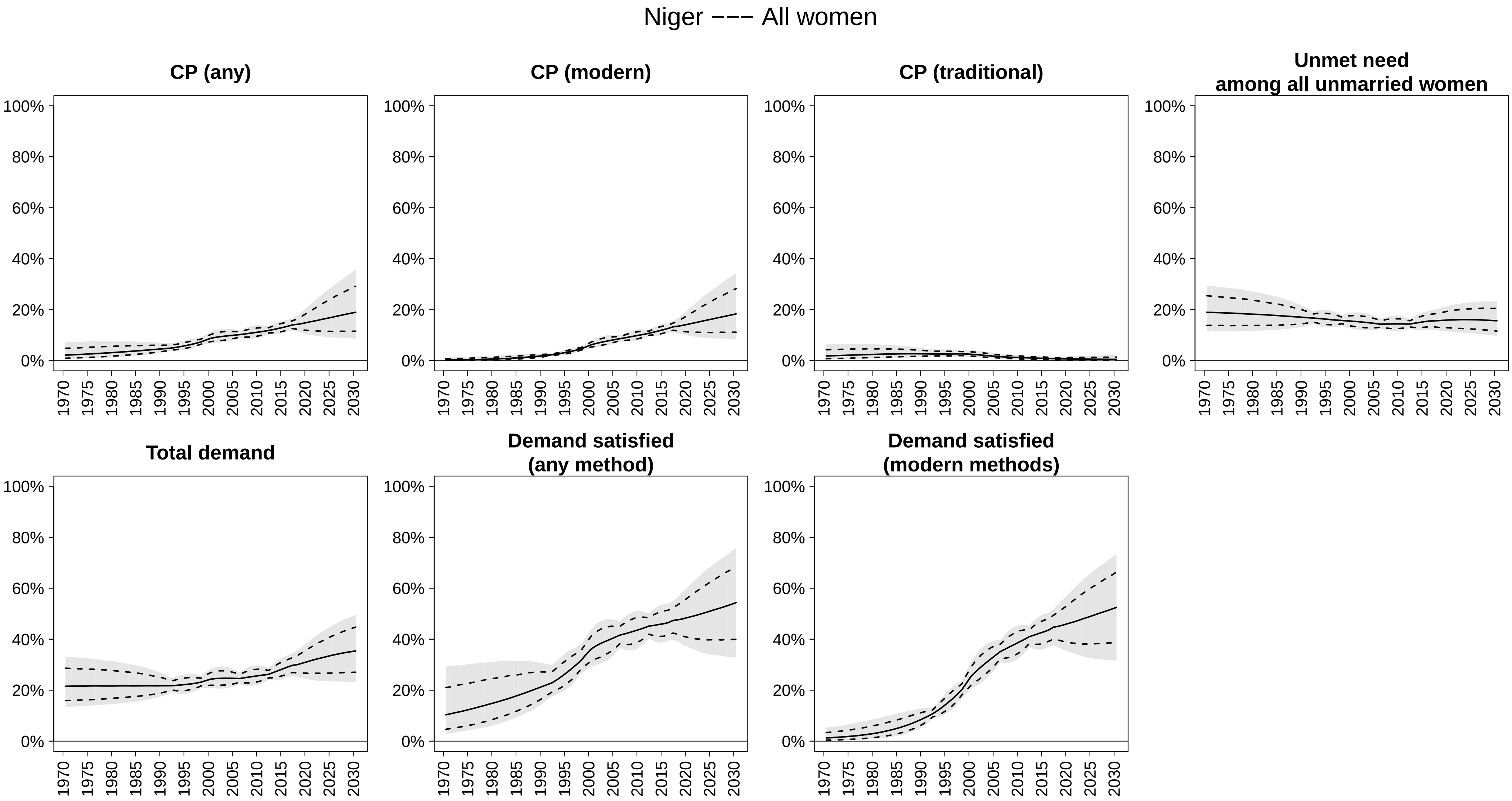
Niger −−− All women
CP (any)
CP (modern)
CP (traditional)
Unmet need
among all unmarried women
Total demand
Demand satisfied
(any method)
Demand satisfied
(modern methods)
100%
80%
60%
40%
20%
0%
1970
1975
1980
1985
1990
1995
2000
2005
2010
2015
2020
2025
2030

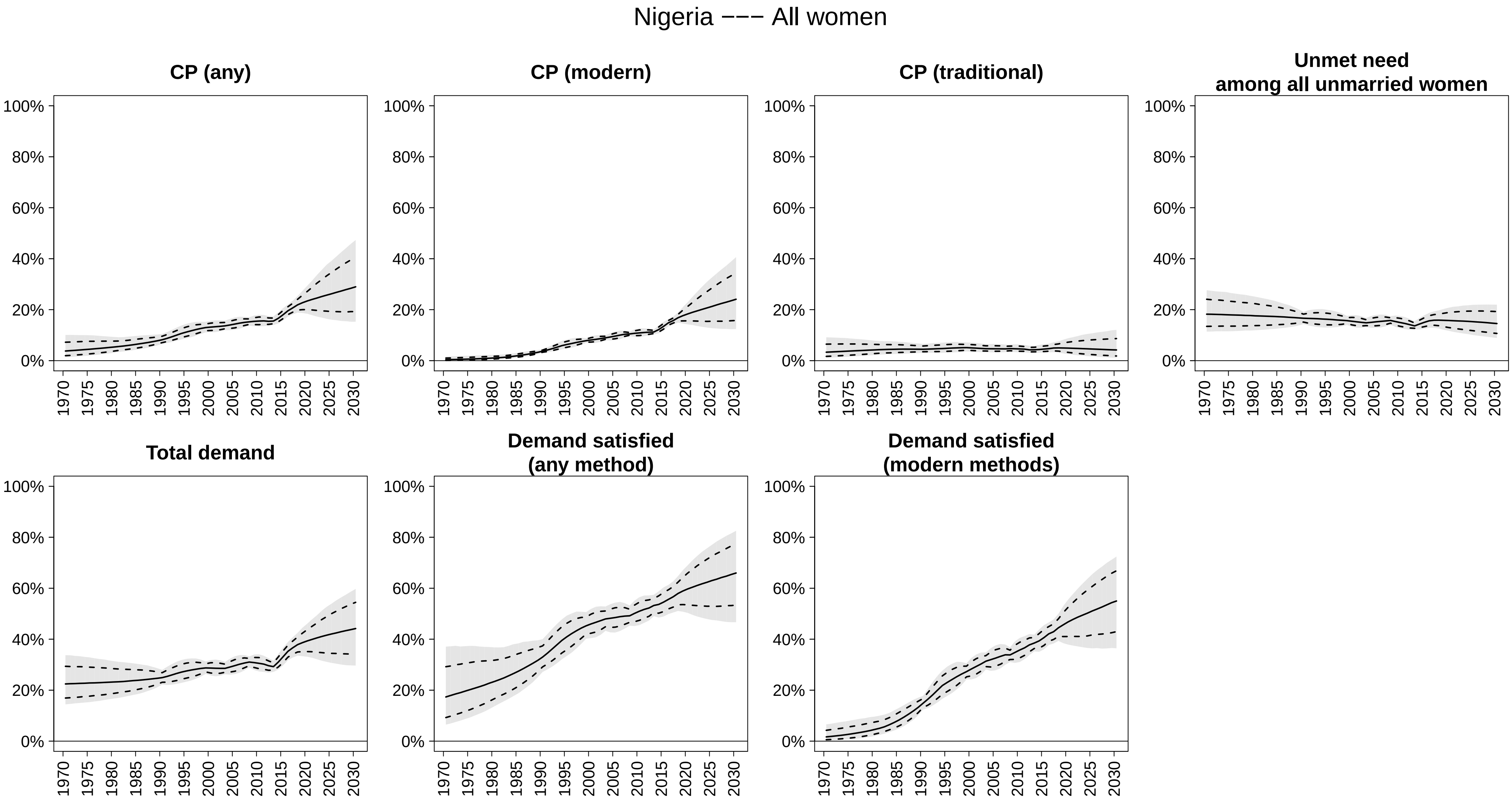
Nigeria −−− All women
CP (any)
CP (modern)
CP (traditional)
Unmet need
among all unmarried women
Total demand
Demand satisfied
(any method)
Demand satisfied
(modern methods)
0%
20%
40%
60%
80%
100%
1970
1975
1980
1985
1990
1995
2000
2005
2010
2015
2020
2025
2030

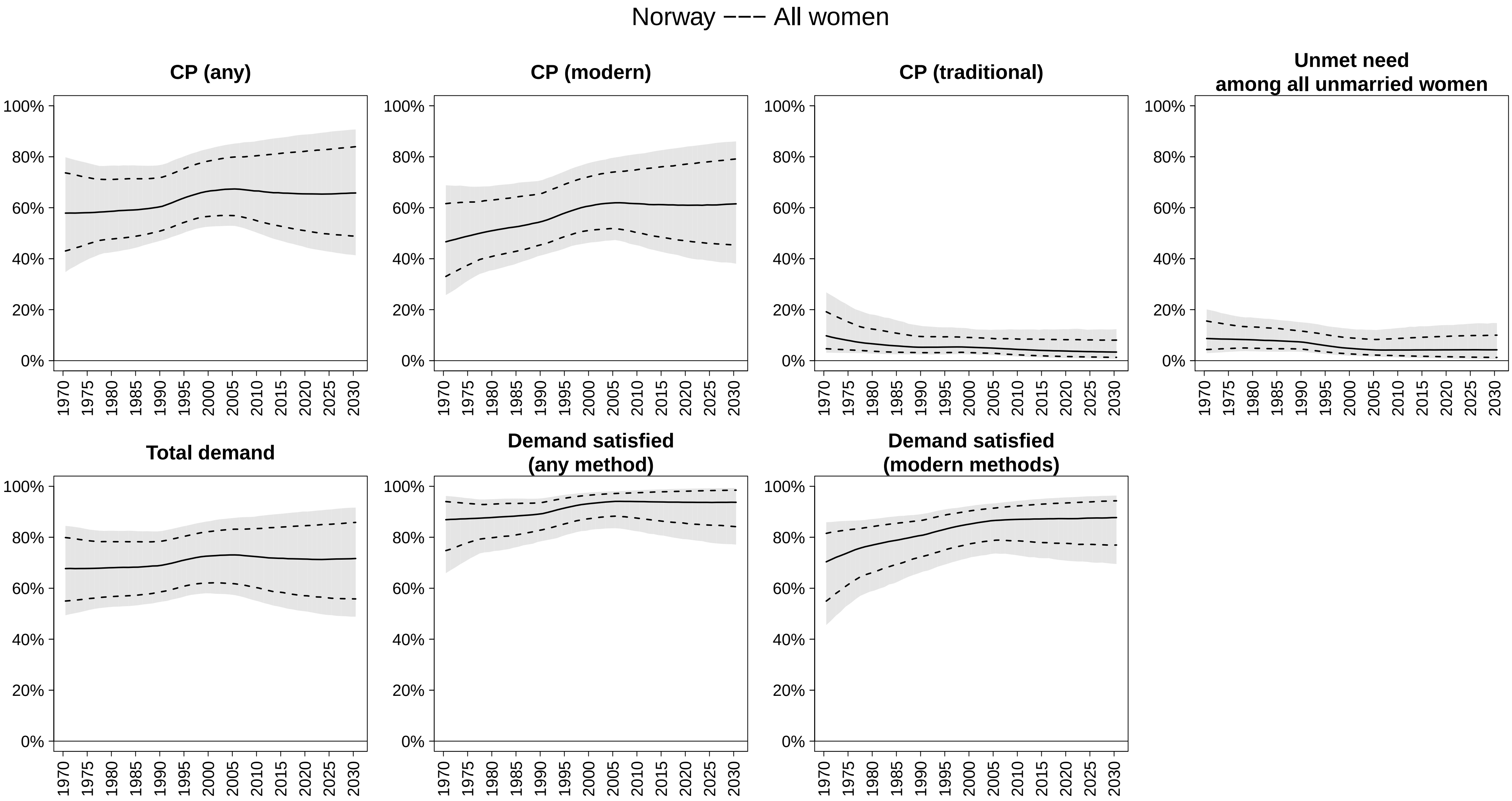
Norway --- All women
CP (any)
CP (modern)
CP (traditional)
Unmet need
among all unmarried women
Total demand
Demand satisfied
(any method)
Demand satisfied
(modern methods)
100%
80%
60%
40%
20%
0%
1970
1975
1980
1985
1990
1995
2000
2005
2010
2015
2020
2025
2030

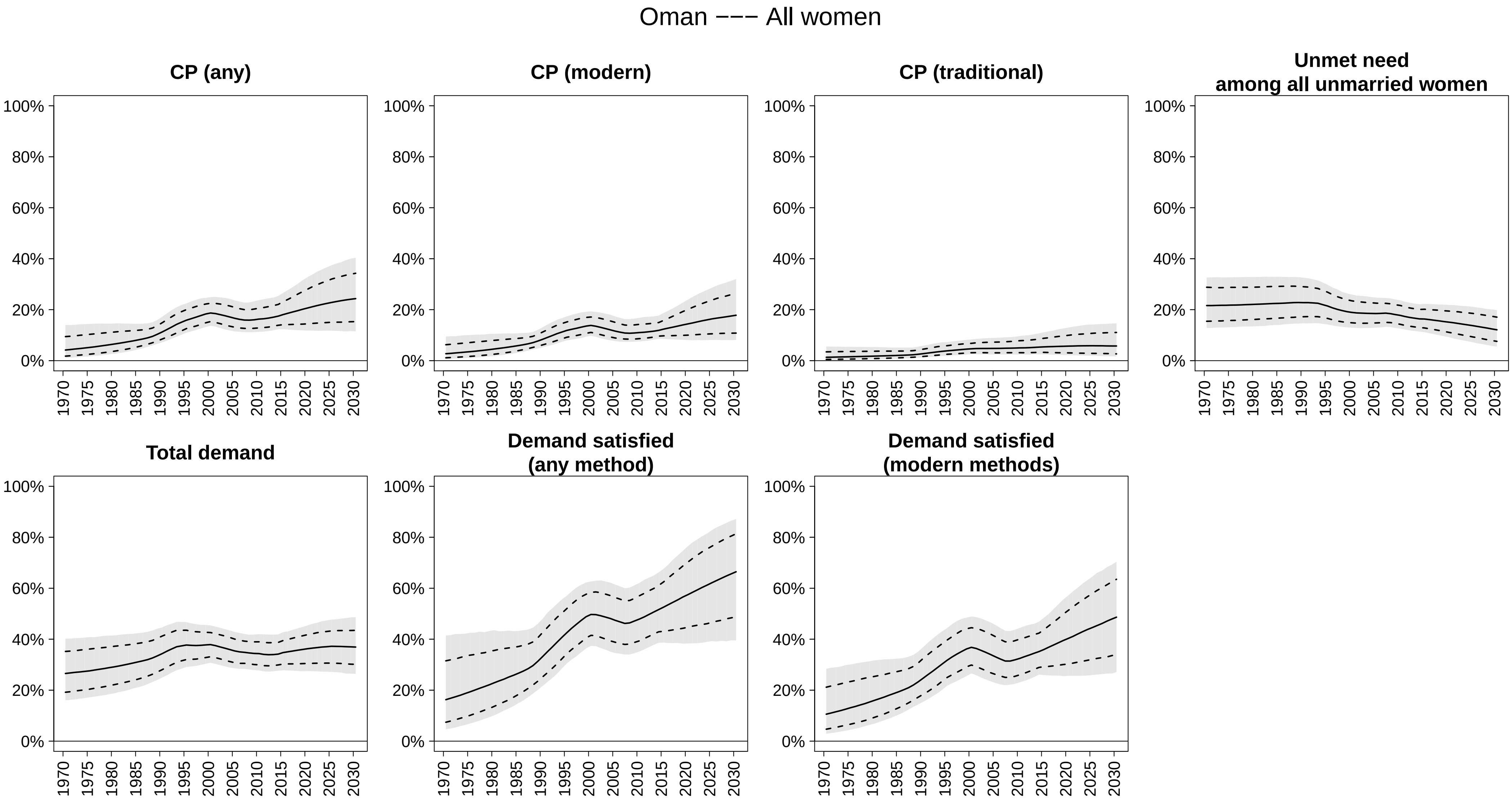
Oman --- All women
CP (any)
CP (modern)
CP (traditional)
Unmet need among all unmarried women
Total demand
Demand satisfied (any method)
Demand satisfied (modern methods)
0%
20%
40%
60%
80%
100%
1970
1975
1980
1985
1990
1995
2000
2005
2010
2015
2020
2025
2030

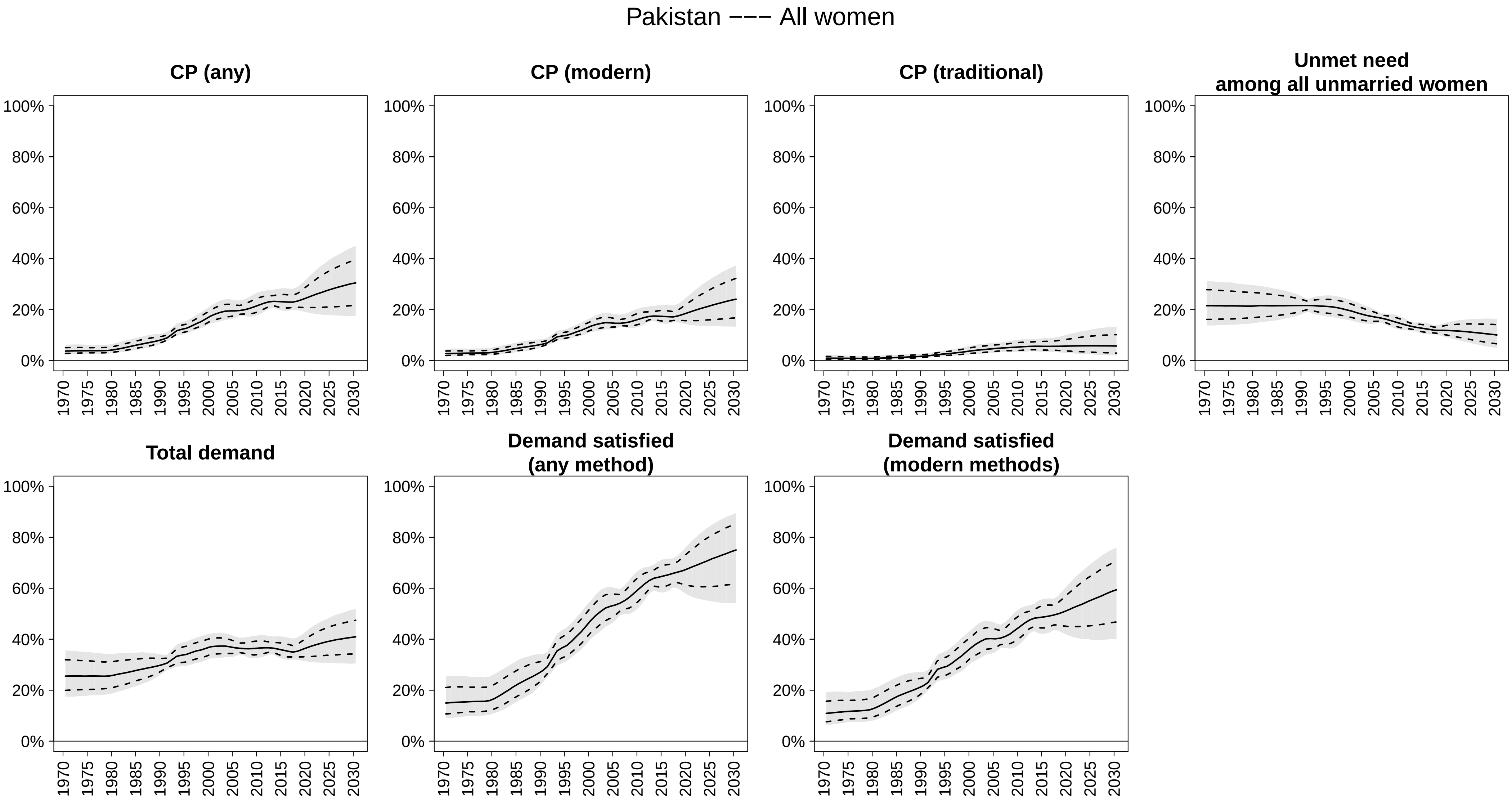
Pakistan −−− All women
CP (any)
CP (modern)
CP (traditional)
Unmet need among all unmarried women
Total demand
Demand satisfied (any method)
Demand satisfied (modern methods)
0%
20%
40%
60%
80%
100%
1970
1975
1980
1985
1990
1995
2000
2005
2010
2015
2020
2025
2030

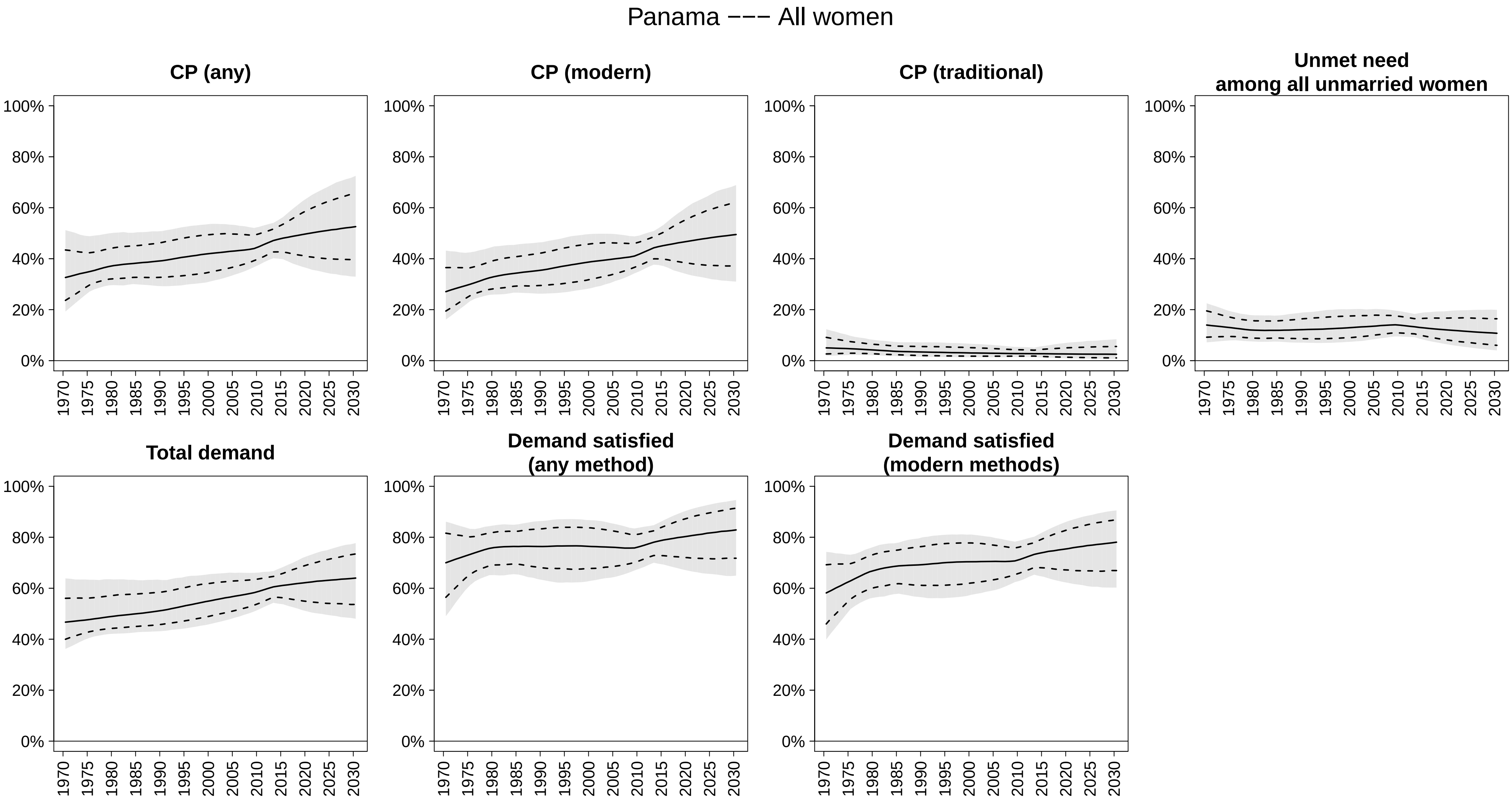
Panama −−− All women
CP (any)
CP (modern)
CP (traditional)
Unmet need
among all unmarried women
Total demand
Demand satisfied
(any method)
Demand satisfied
(modern methods)
100%
80%
60%
40%
20%
0%
1970
1975
1980
1985
1990
1995
2000
2005
2010
2015
2020
2025
2030

Papua New Guinea −−− All women

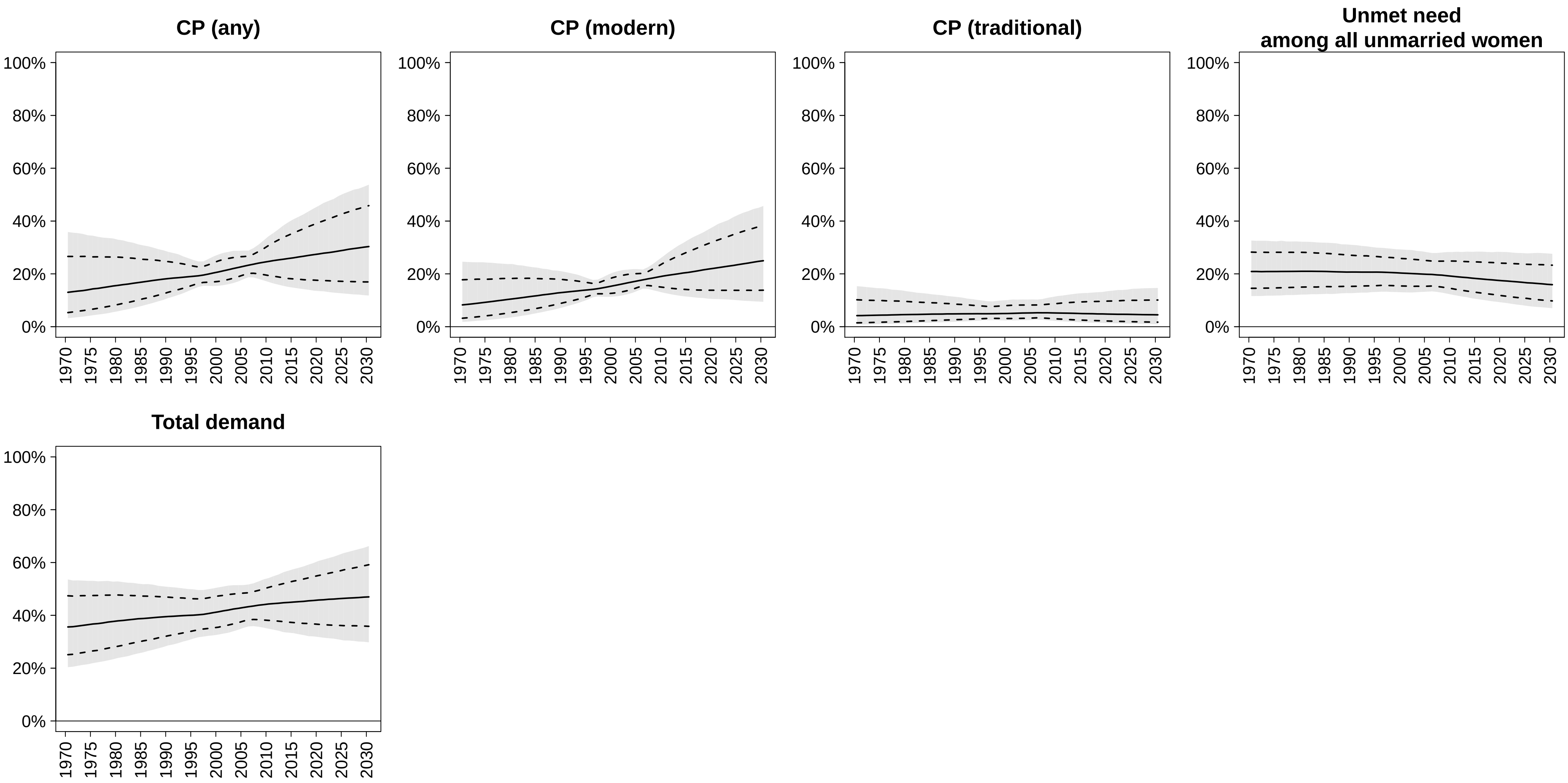

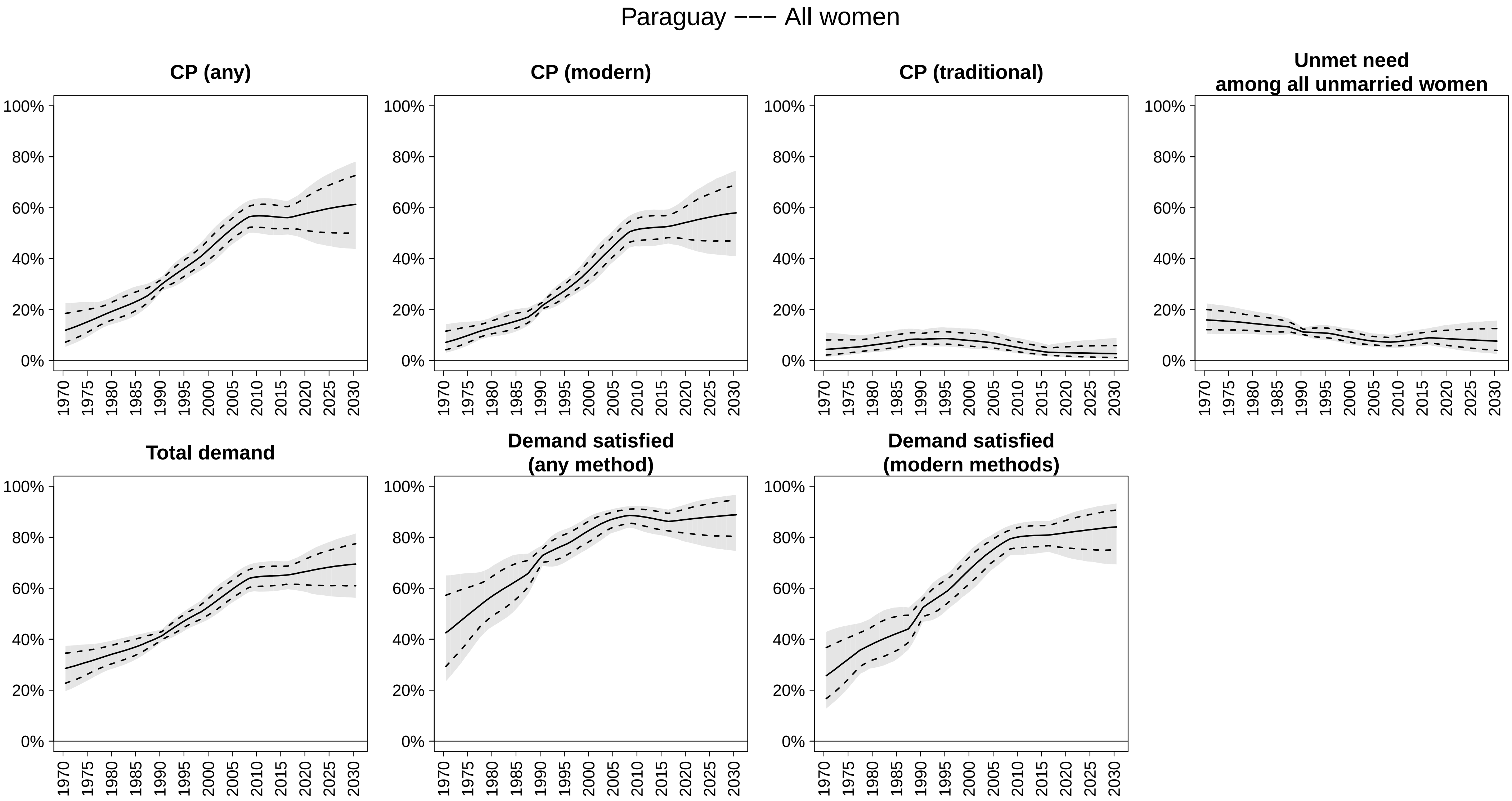
Paraguay −−− All women
CP (any)
CP (modern)
CP (traditional)
Unmet need among all unmarried women
Total demand
Demand satisfied (any method)
Demand satisfied (modern methods)
0%
20%
40%
60%
80%
100%
1970
1975
1980
1985
1990
1995
2000
2005
2010
2015
2020
2025
2030

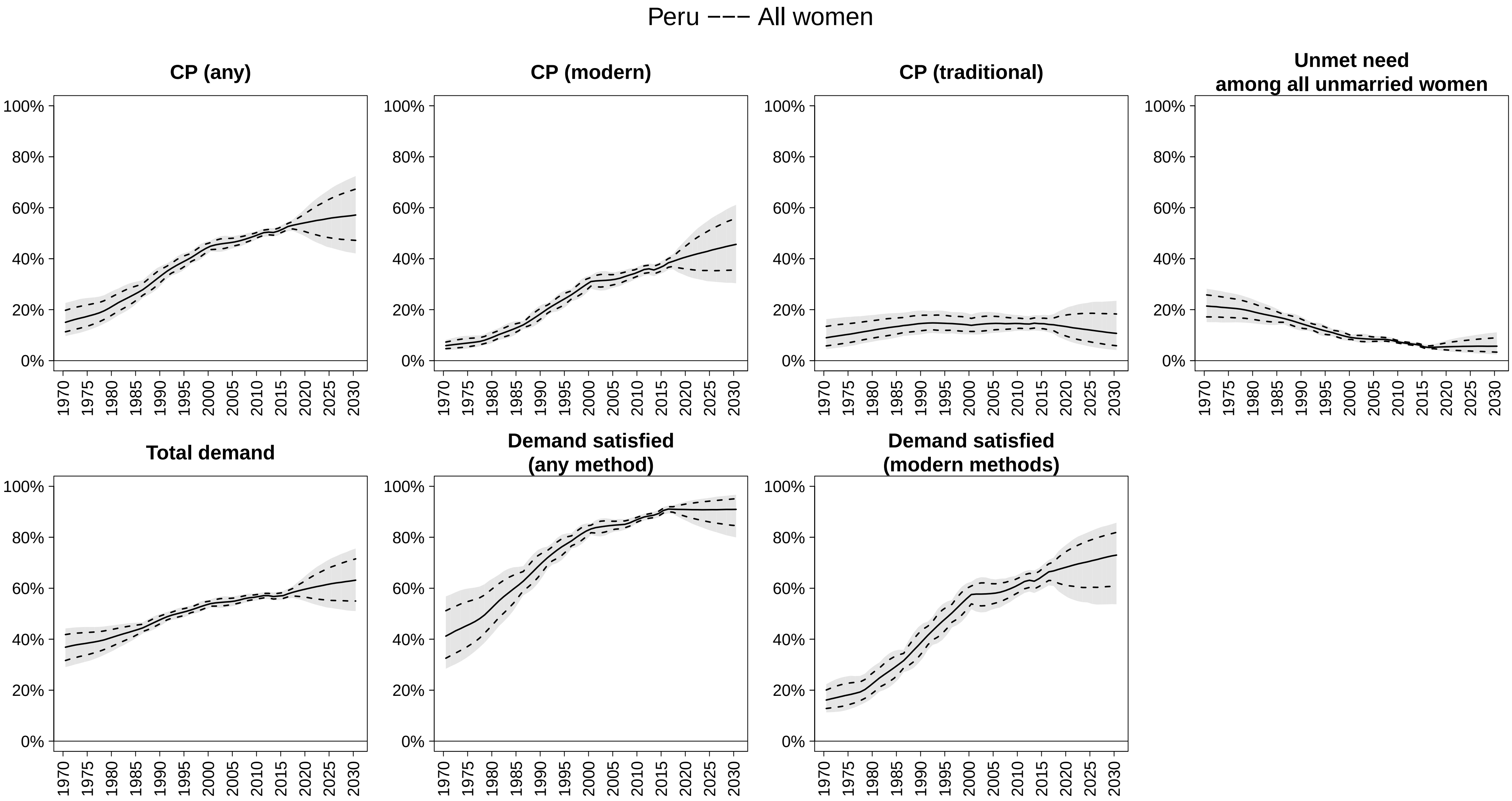
Peru −−− All women
CP (any)
CP (modern)
CP (traditional)
Unmet need
among all unmarried women
Total demand
Demand satisfied
(any method)
Demand satisfied
(modern methods)
0%
20%
40%
60%
80%
100%
1970
1975
1980
1985
1990
1995
2000
2005
2010
2015
2020
2025
2030

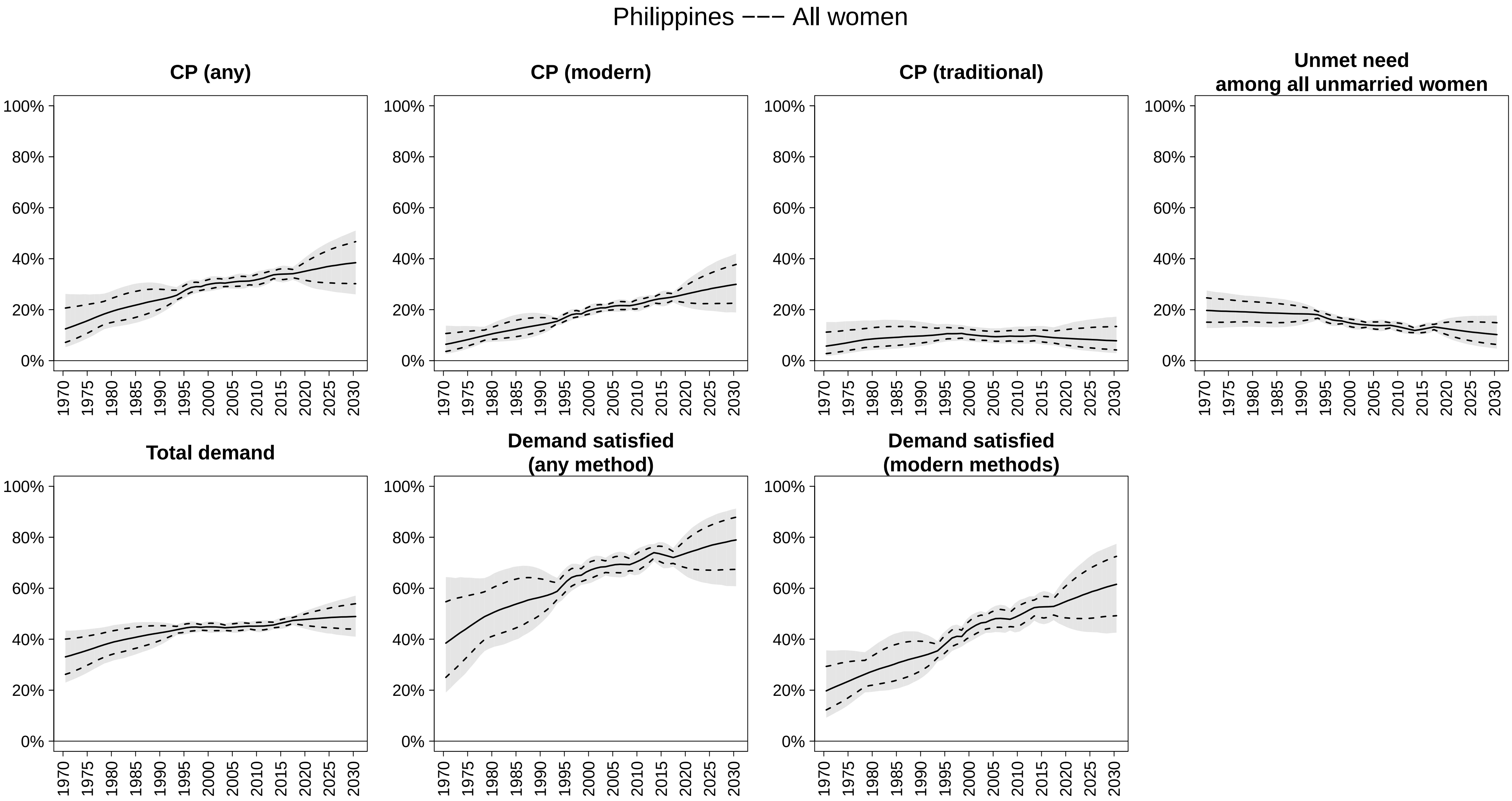
Philippines --- All women
CP (any)
CP (modern)
CP (traditional)
Unmet need among all unmarried women
Total demand
Demand satisfied (any method)
Demand satisfied (modern methods)
0%
20%
40%
60%
80%
100%
1970
1975
1980
1985
1990
1995
2000
2005
2010
2015
2020
2025
2030

Poland −−− All women

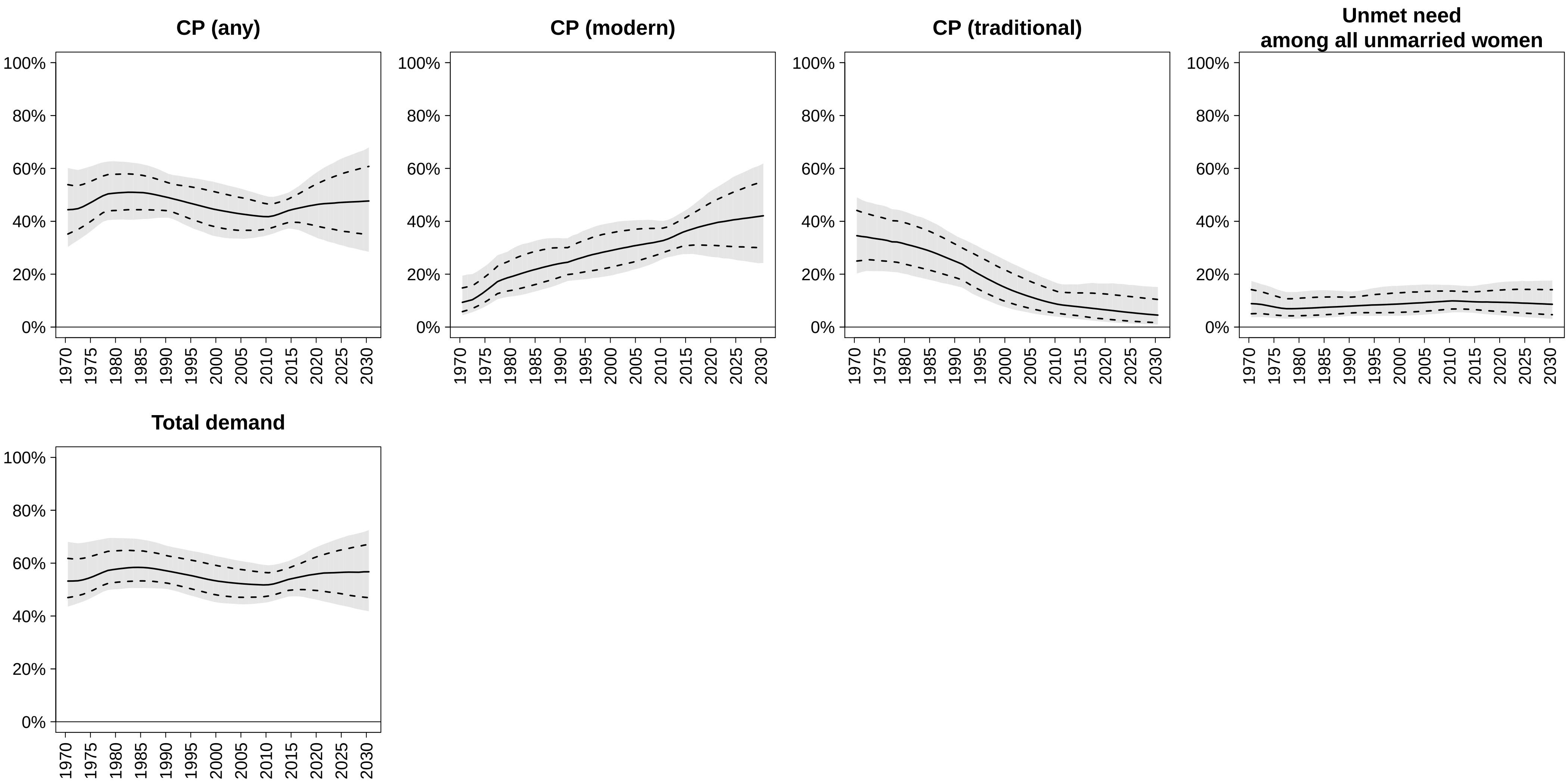

Portugal −−− All women

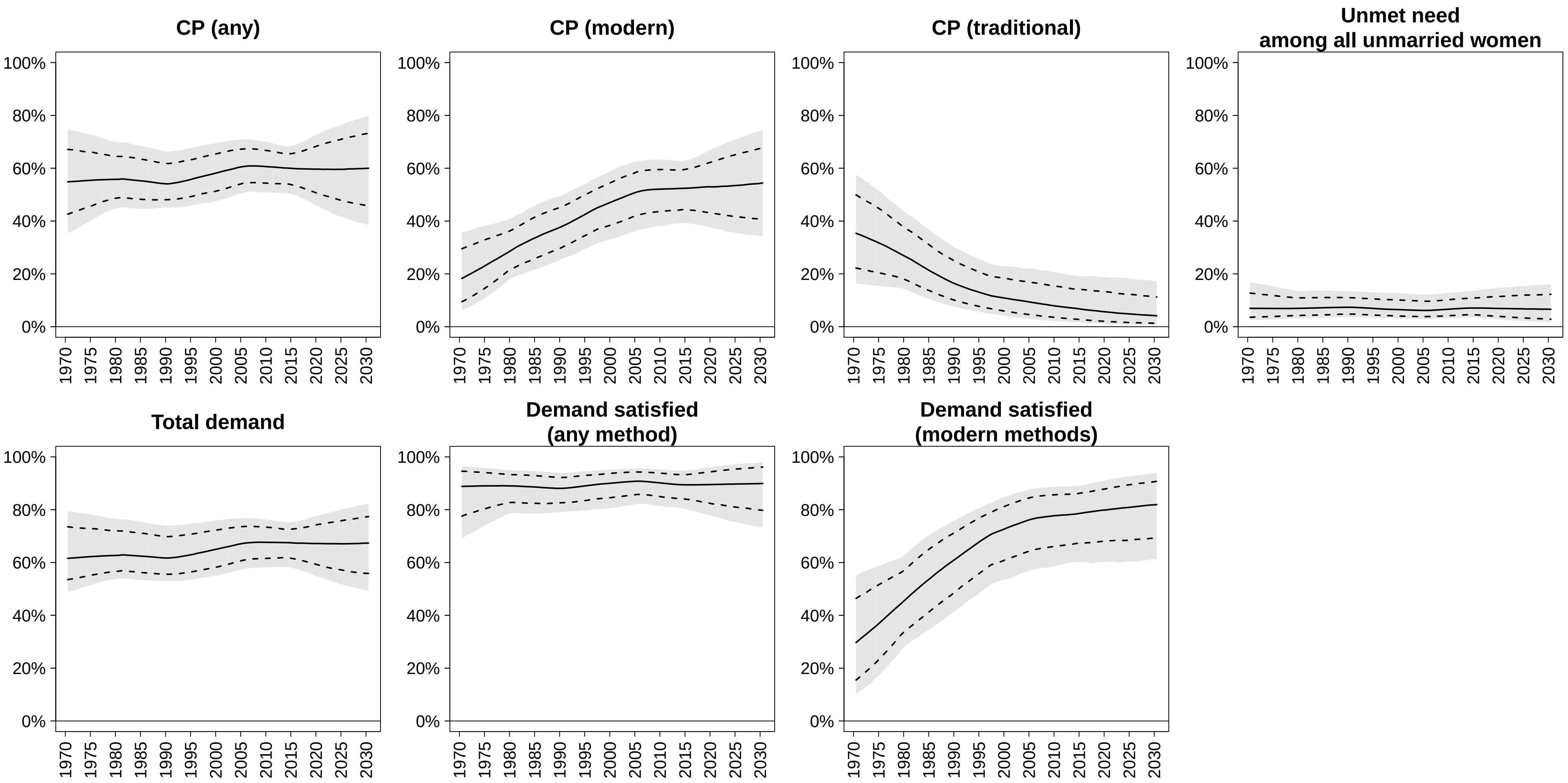

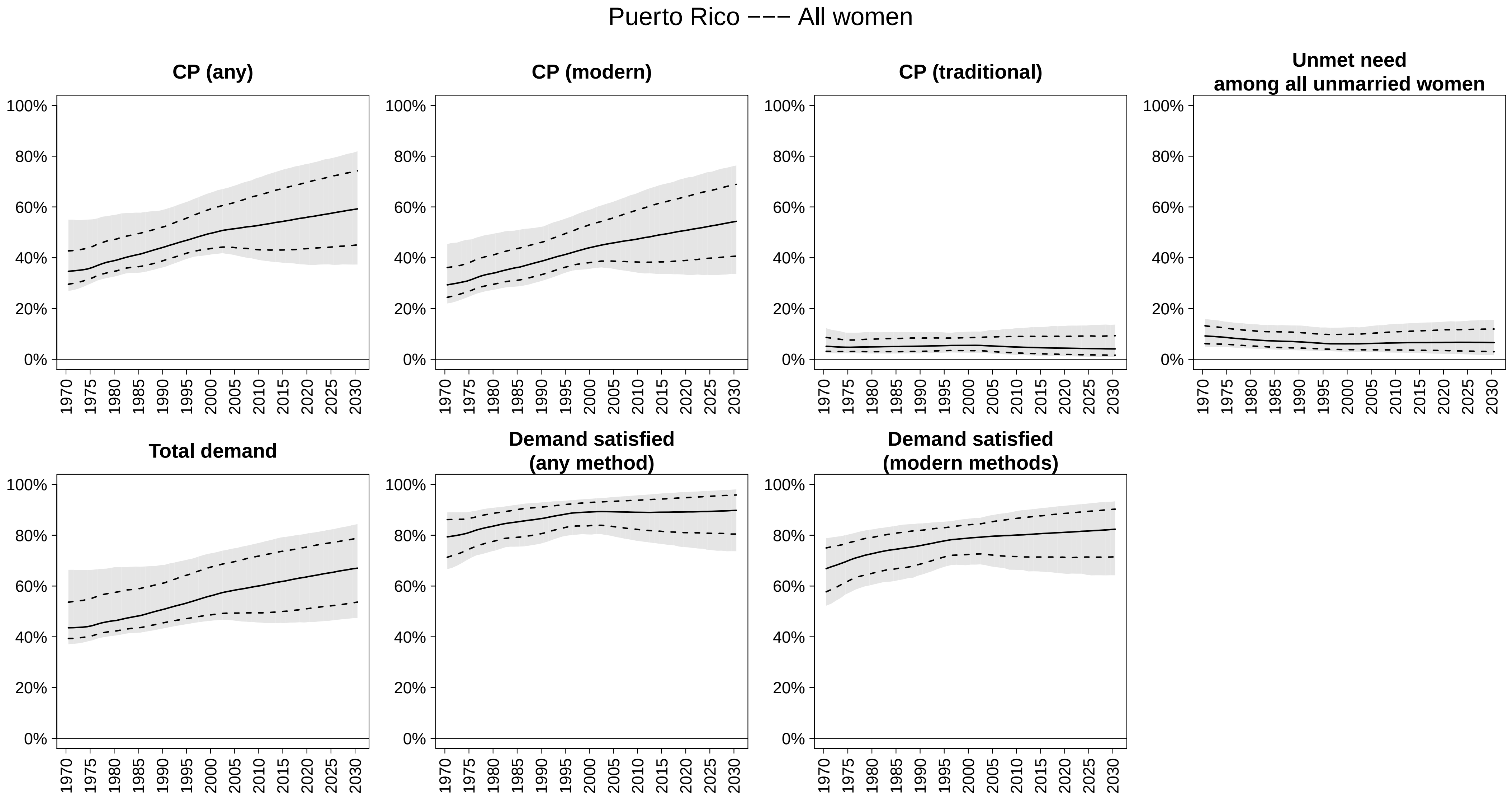
Puerto Rico −−− All women
CP (any)
CP (modern)
CP (traditional)
Unmet need among all unmarried women
Total demand
Demand satisfied (any method)
Demand satisfied (modern methods)
0%
20%
40%
60%
80%
100%
1970
1975
1980
1985
1990
1995
2000
2005
2010
2015
2020
2025
2030

Qatar −−− All women

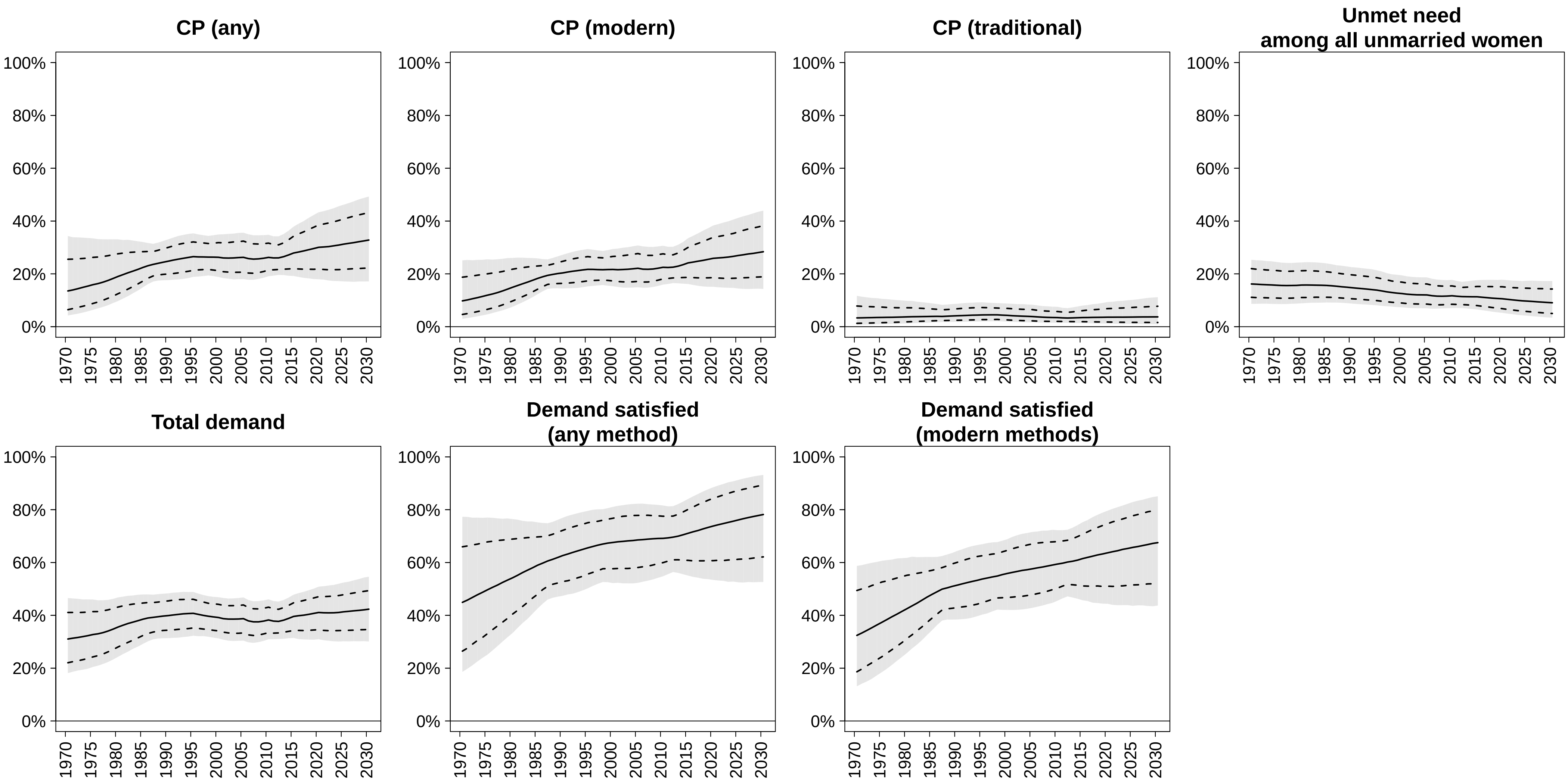

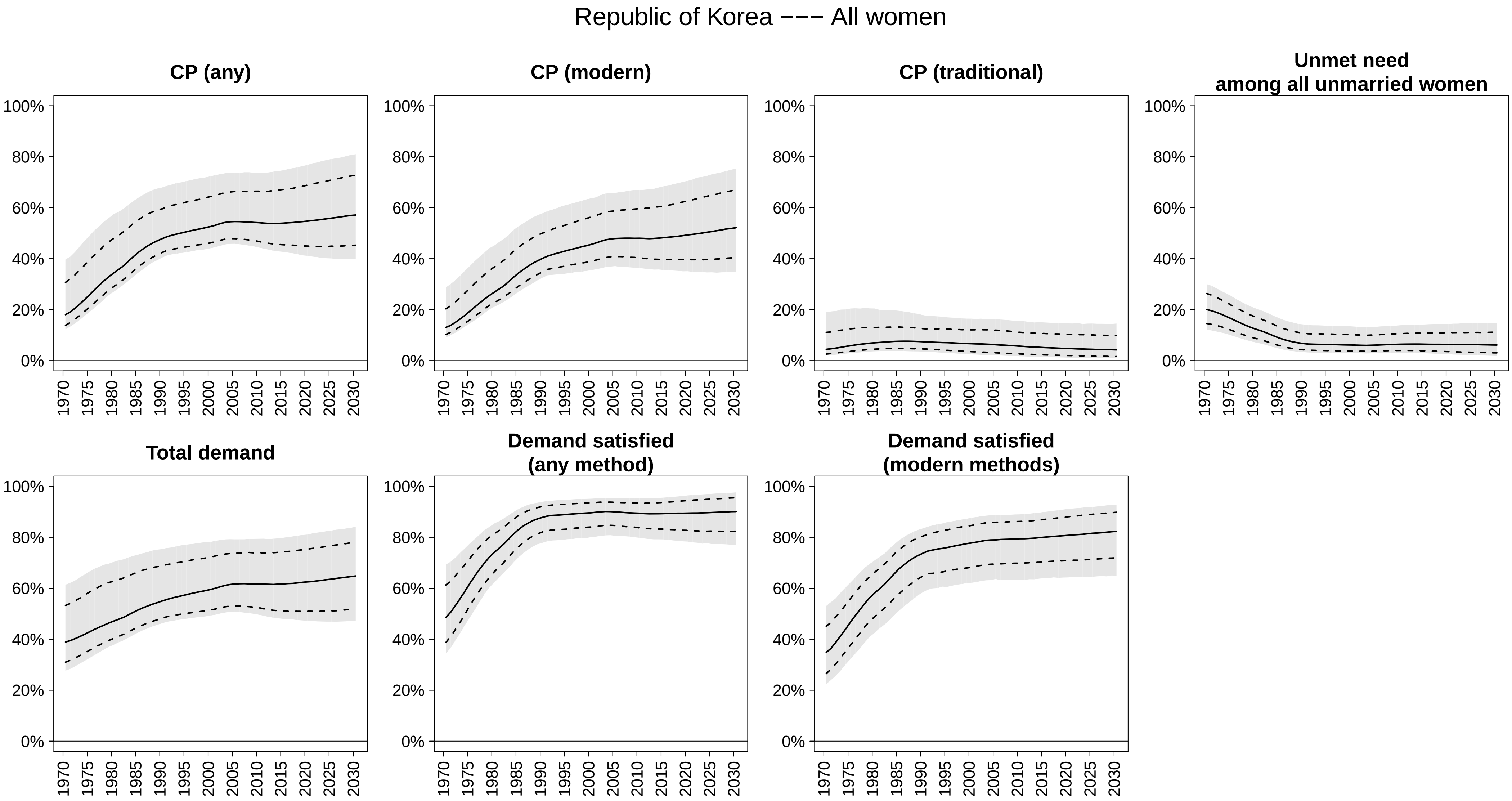
Republic of Korea −−− All women
CP (any)
CP (modern)
CP (traditional)
Unmet need among all unmarried women
Total demand
Demand satisfied (any method)
Demand satisfied (modern methods)
0%
20%
40%
60%
80%
100%
1970
1975
1980
1985
1990
1995
2000
2005
2010
2015
2020
2025
2030

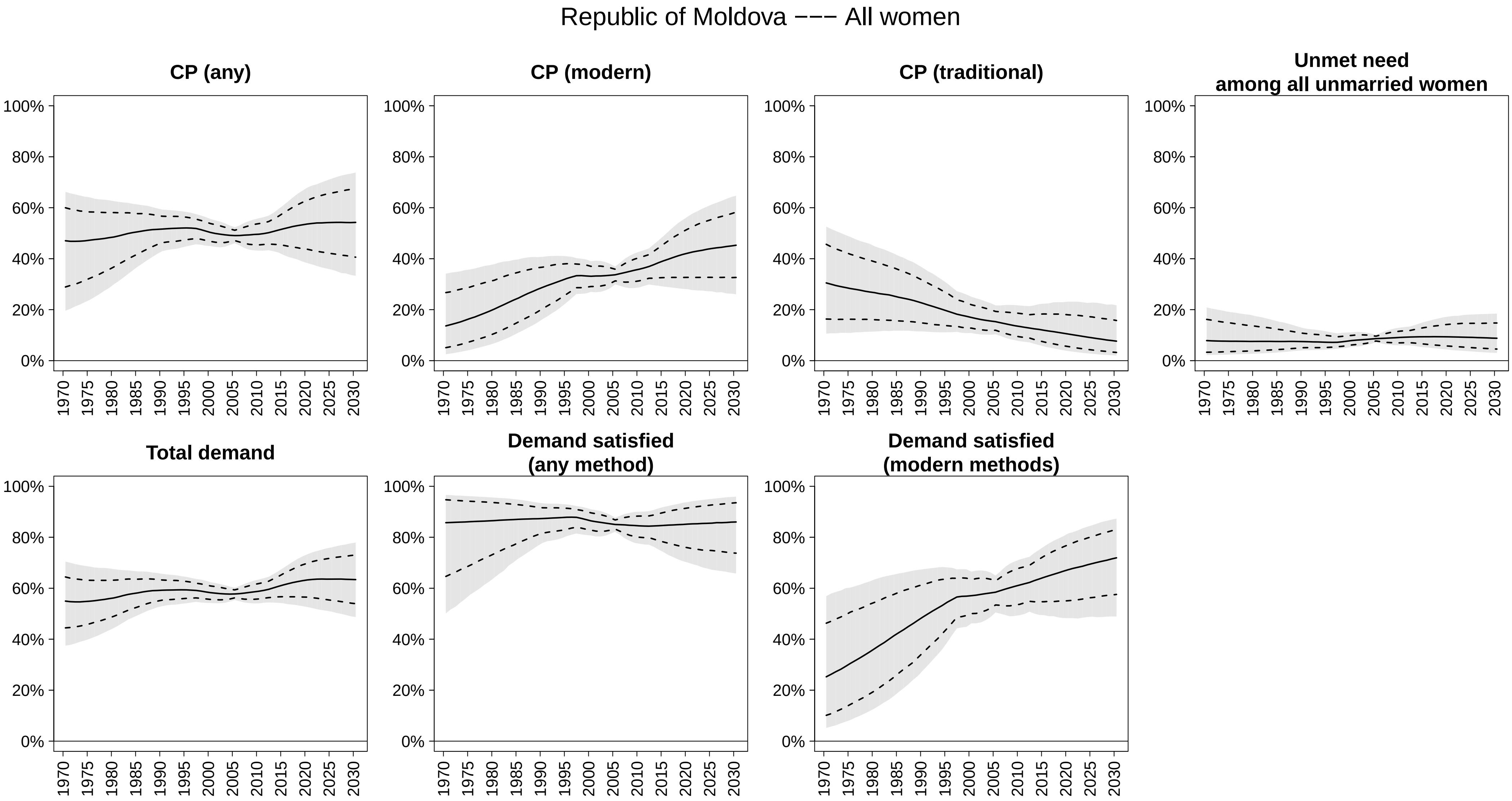
Republic of Moldova −−− All women
CP (any)
CP (modern)
CP (traditional)
Unmet need
among all unmarried women
Total demand
Demand satisfied
(any method)
Demand satisfied
(modern methods)
0%
20%
40%
60%
80%
100%
1970
1975
1980
1985
1990
1995
2000
2005
2010
2015
2020
2025
2030

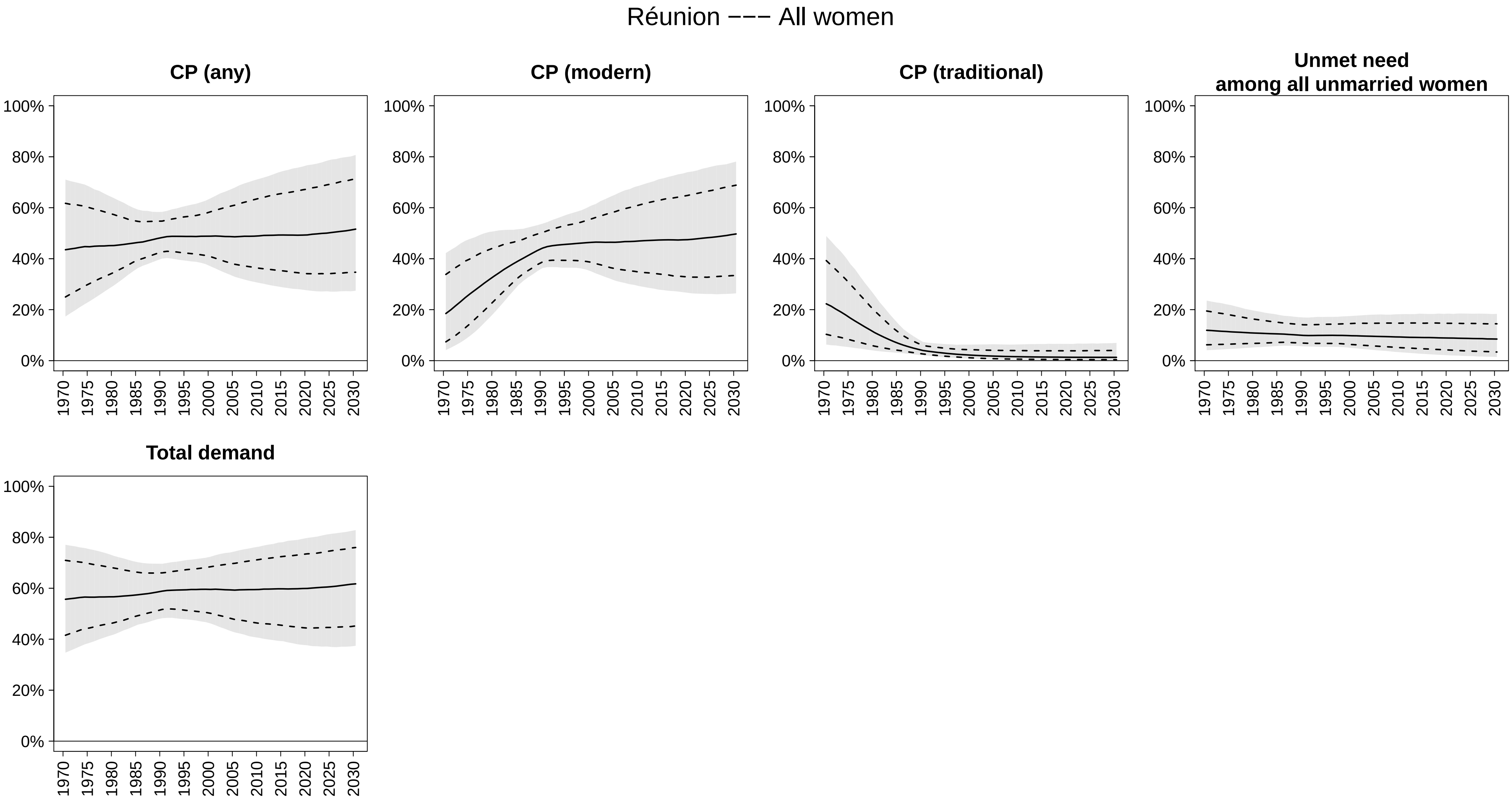
Réunion −−− All women
CP (any)
CP (modern)
CP (traditional)
Unmet need
among all unmarried women
Total demand
0%
20%
40%
60%
80%
100%
1970
1975
1980
1985
1990
1995
2000
2005
2010
2015
2020
2025
2030

Romania −−− All women

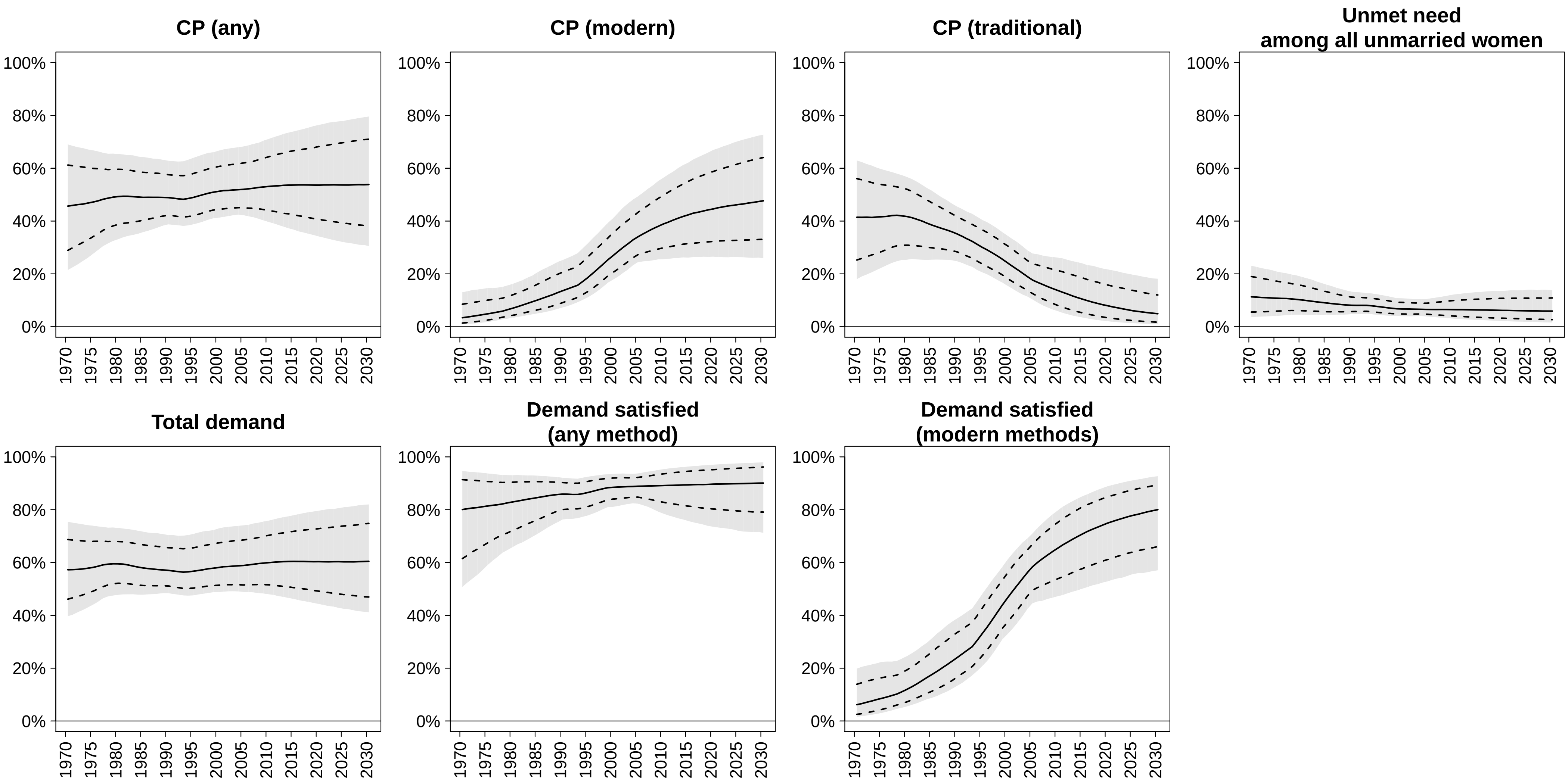

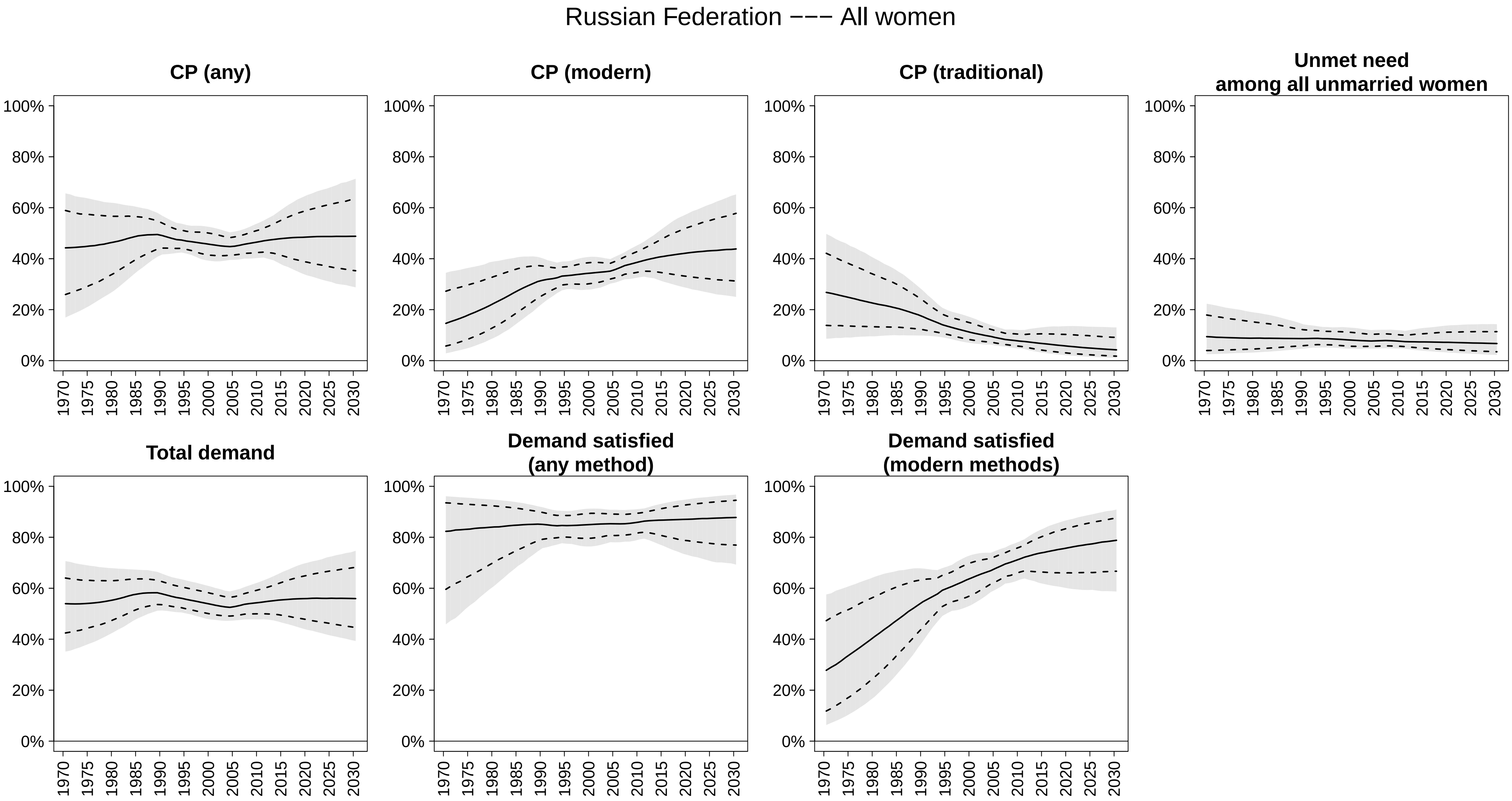
Russian Federation --- All women
CP (any)
CP (modern)
CP (traditional)
Unmet need among all unmarried women
Total demand
Demand satisfied (any method)
Demand satisfied (modern methods)
0%
20%
40%
60%
80%
100%
1970
1975
1980
1985
1990
1995
2000
2005
2010
2015
2020
2025
2030

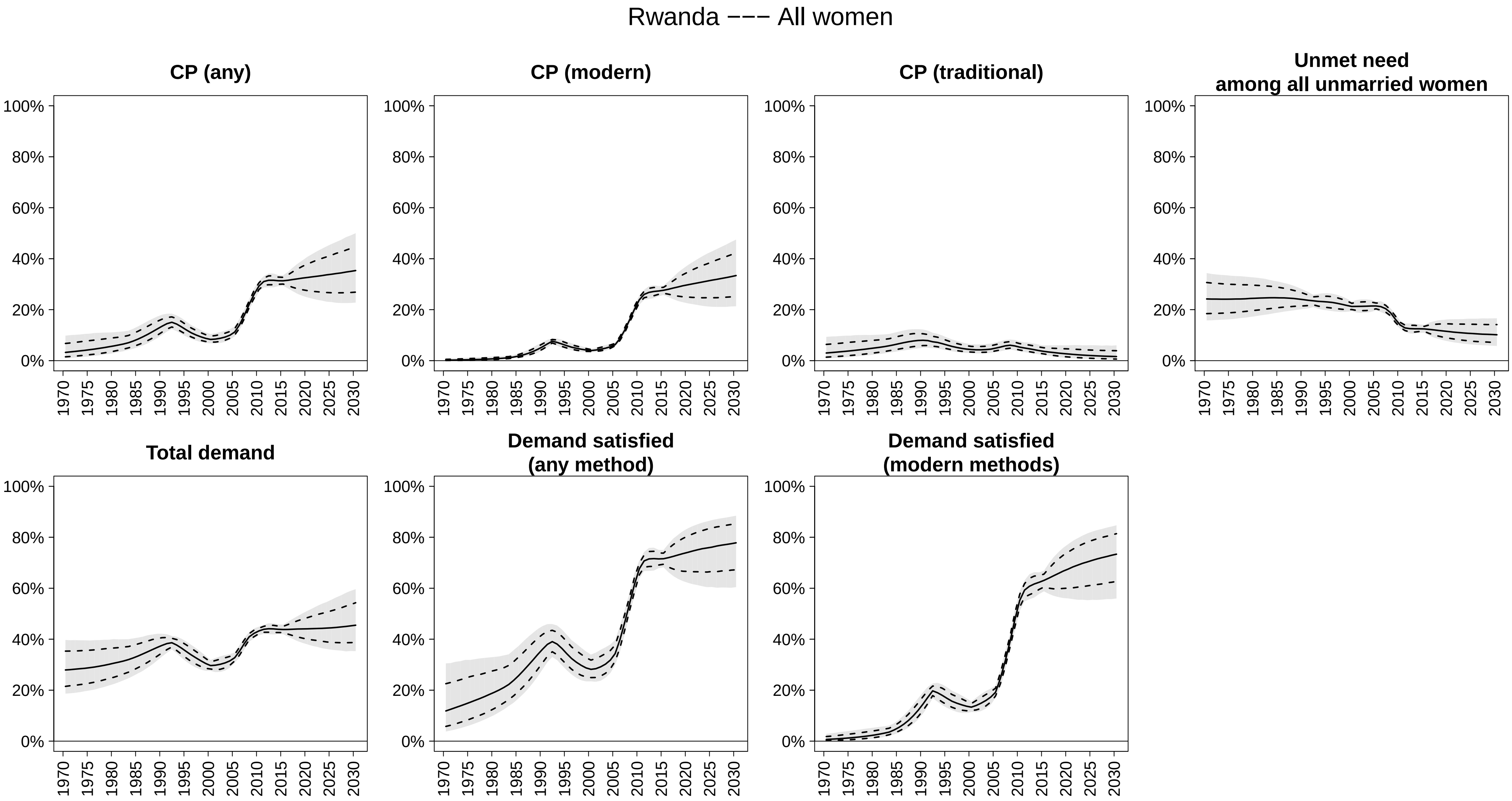
Rwanda −−− All women
CP (any)
CP (modern)
CP (traditional)
Unmet need among all unmarried women
Total demand
Demand satisfied (any method)
Demand satisfied (modern methods)
0%
20%
40%
60%
80%
100%
1970
1975
1980
1985
1990
1995
2000
2005
2010
2015
2020
2025
2030

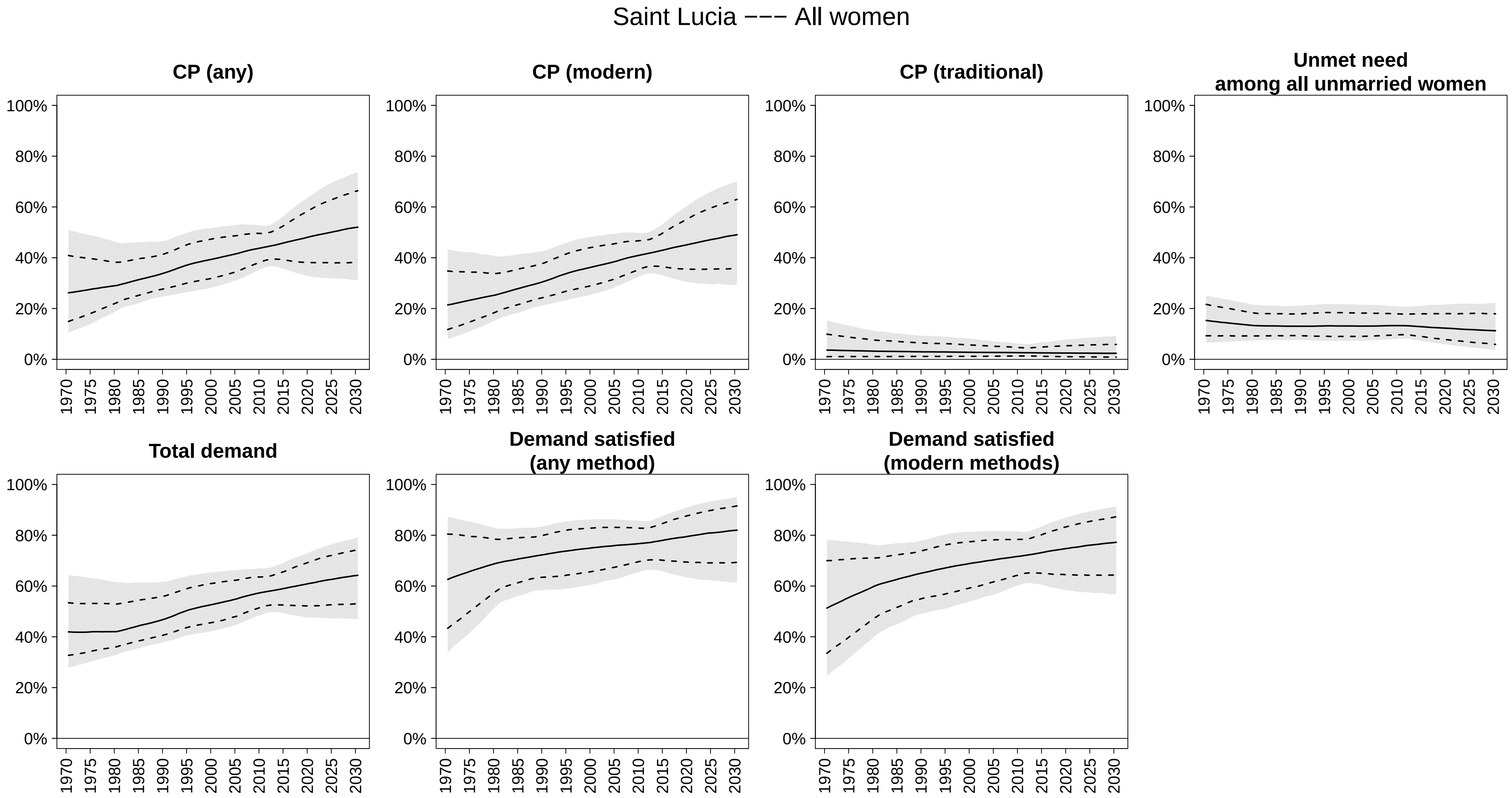
Saint Lucia −−− All women
CP (any)
CP (modern)
CP (traditional)
Unmet need
among all unmarried women
Total demand
Demand satisfied
(any method)
Demand satisfied
(modern methods)
100%
80%
60%
40%
20%
0%
1970
1975
1980
1985
1990
1995
2000
2005
2010
2015
2020
2025
2030

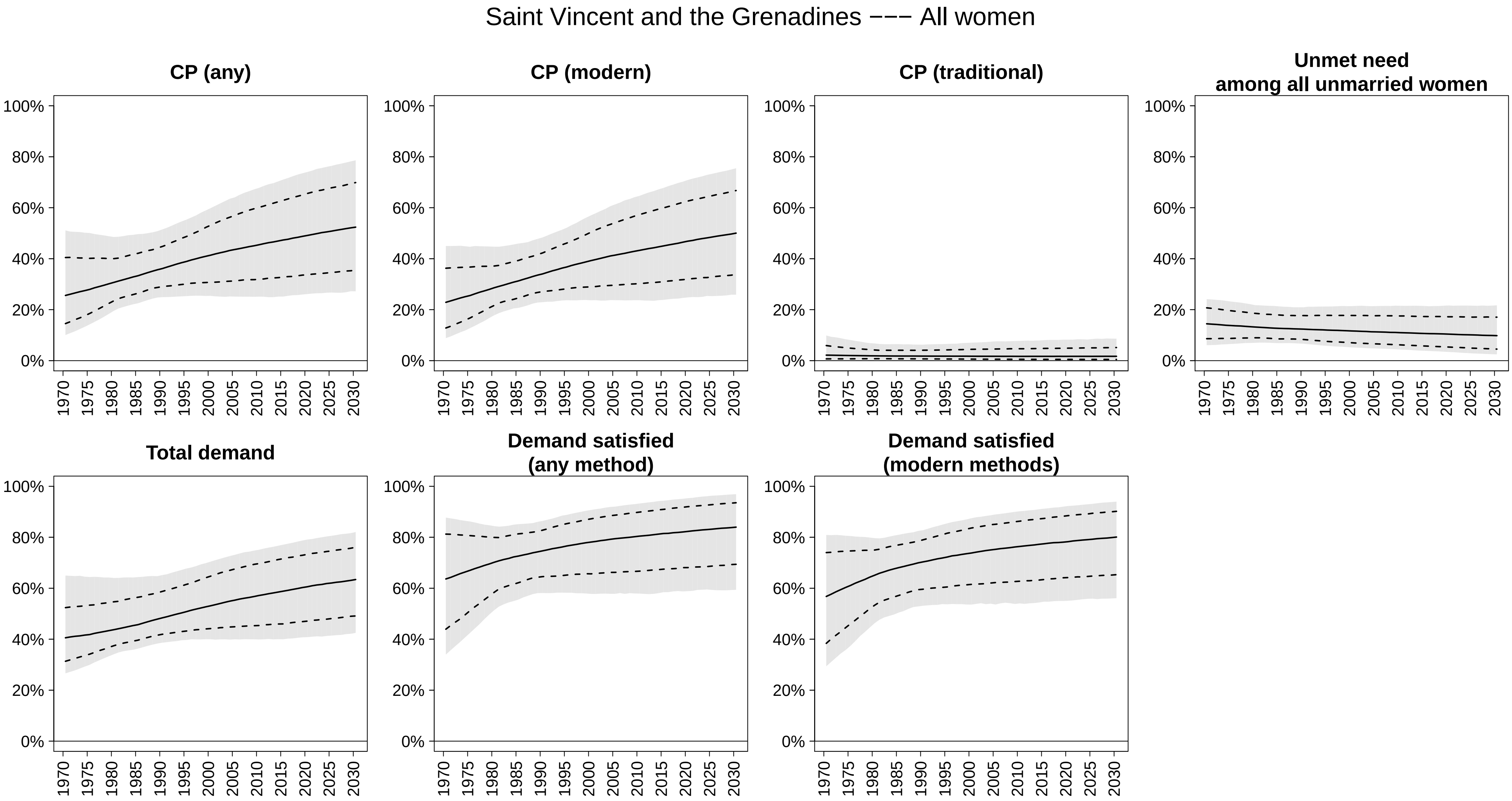
Saint Vincent and the Grenadines −−− All women
CP (any)
CP (modern)
CP (traditional)
Unmet need among all unmarried women
Total demand
Demand satisfied (any method)
Demand satisfied (modern methods)
0%
20%
40%
60%
80%
100%
1970
1975
1980
1985
1990
1995
2000
2005
2010
2015
2020
2025
2030

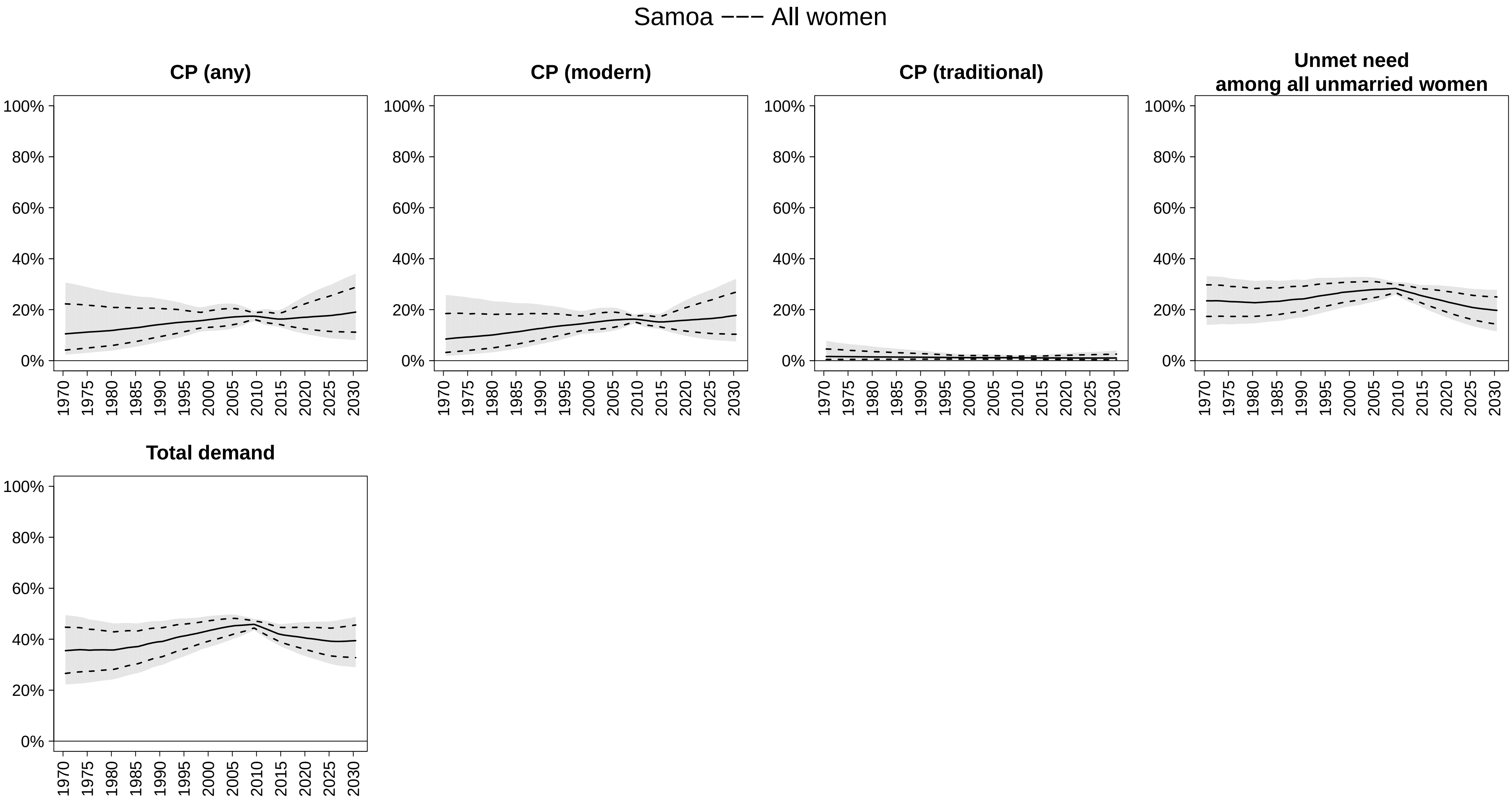
Samoa −−− All women
CP (any)
CP (modern)
CP (traditional)
Unmet need among all unmarried women
Total demand
100%
80%
60%
40%
20%
0%
1970 1975 1980 1985 1990 1995 2000 2005 2010 2015 2020 2025 2030

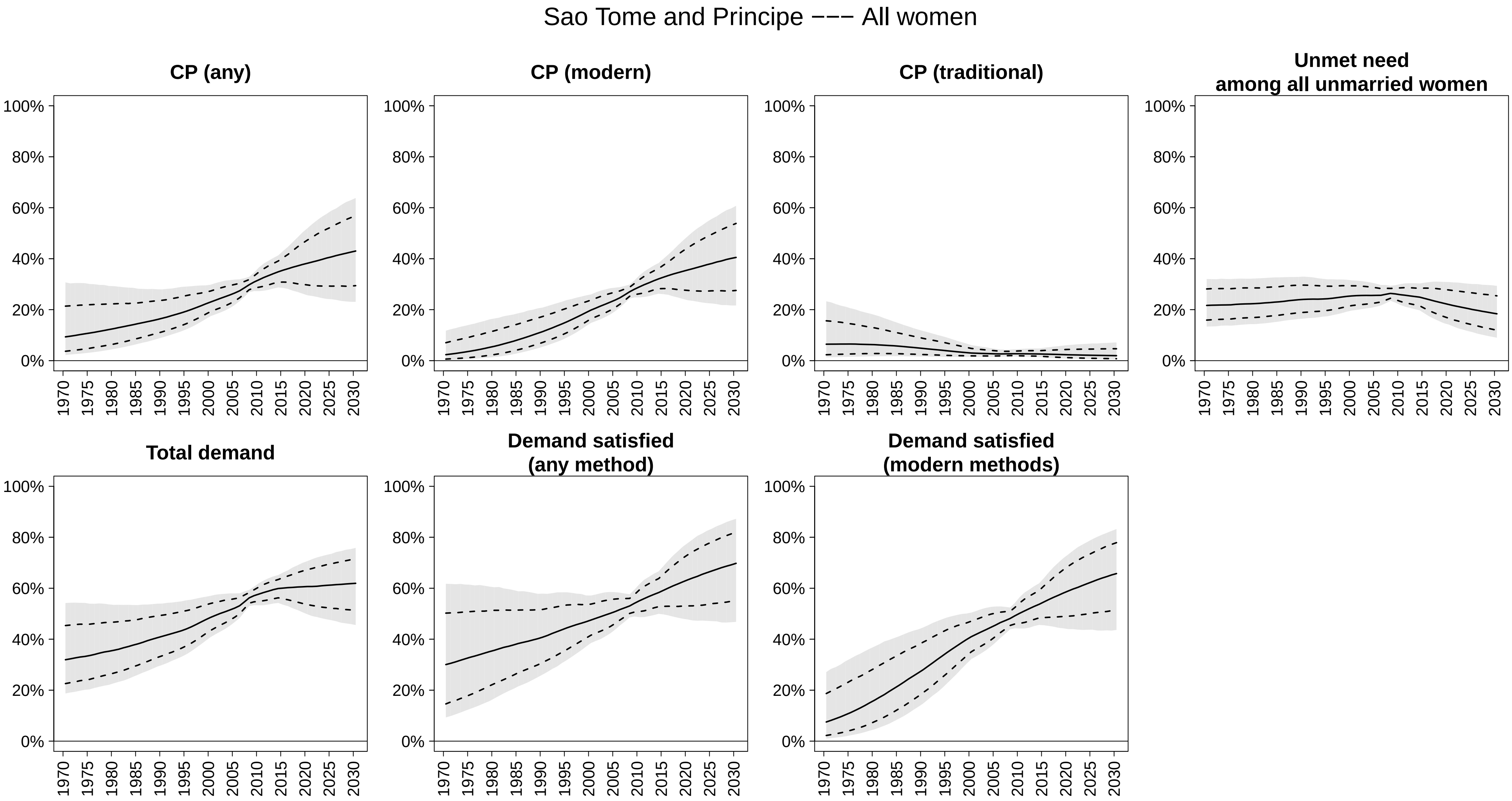
Sao Tome and Principe --- All women
CP (any)
CP (modern)
CP (traditional)
Unmet need among all unmarried women
Total demand
Demand satisfied (any method)
Demand satisfied (modern methods)
0%
20%
40%
60%
80%
100%
1970
1975
1980
1985
1990
1995
2000
2005
2010
2015
2020
2025
2030

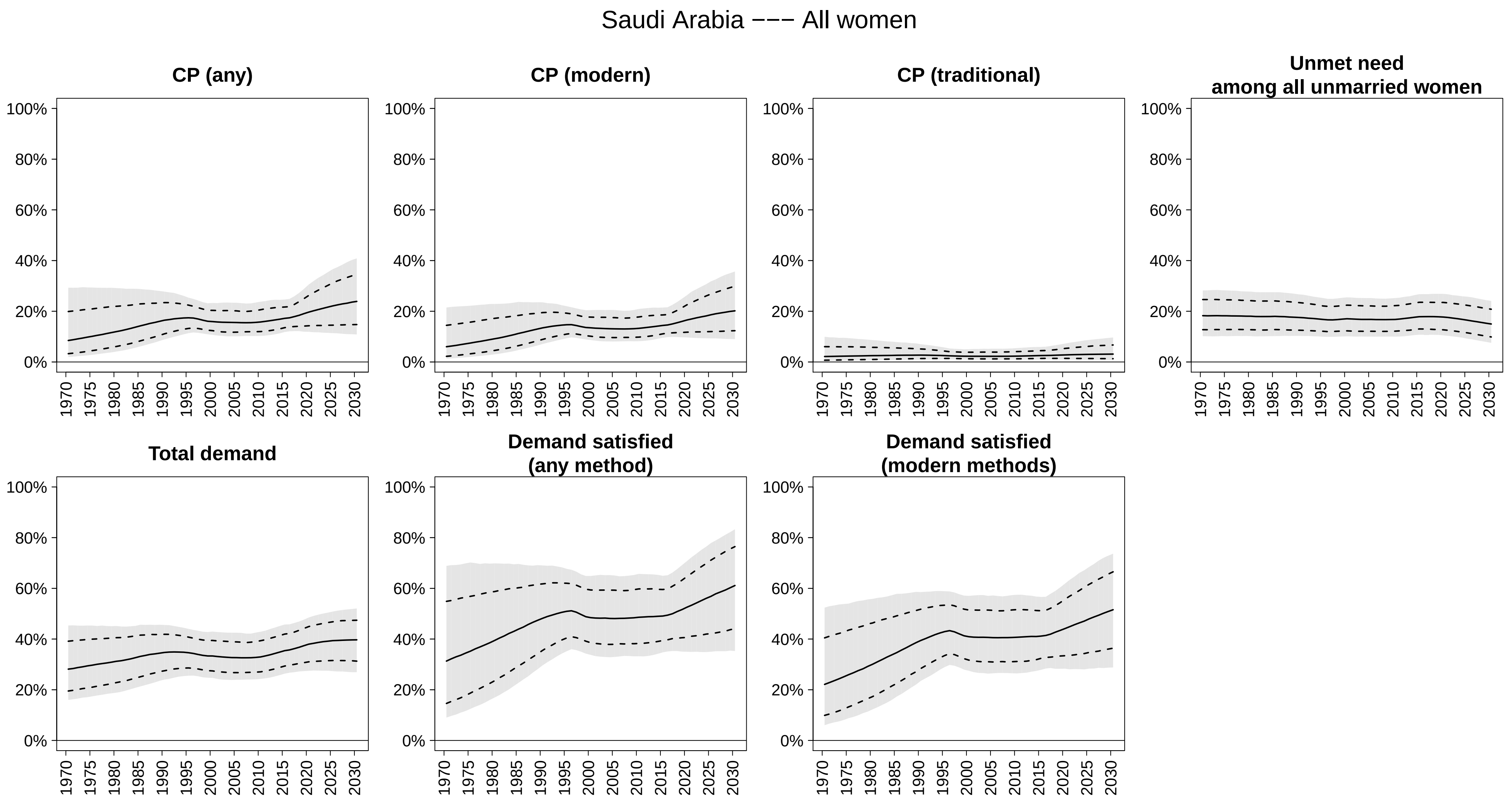
Saudi Arabia −−− All women
CP (any)
CP (modern)
CP (traditional)
Unmet need among all unmarried women
Total demand
Demand satisfied (any method)
Demand satisfied (modern methods)
0%
20%
40%
60%
80%
100%
1970
1975
1980
1985
1990
1995
2000
2005
2010
2015
2020
2025
2030

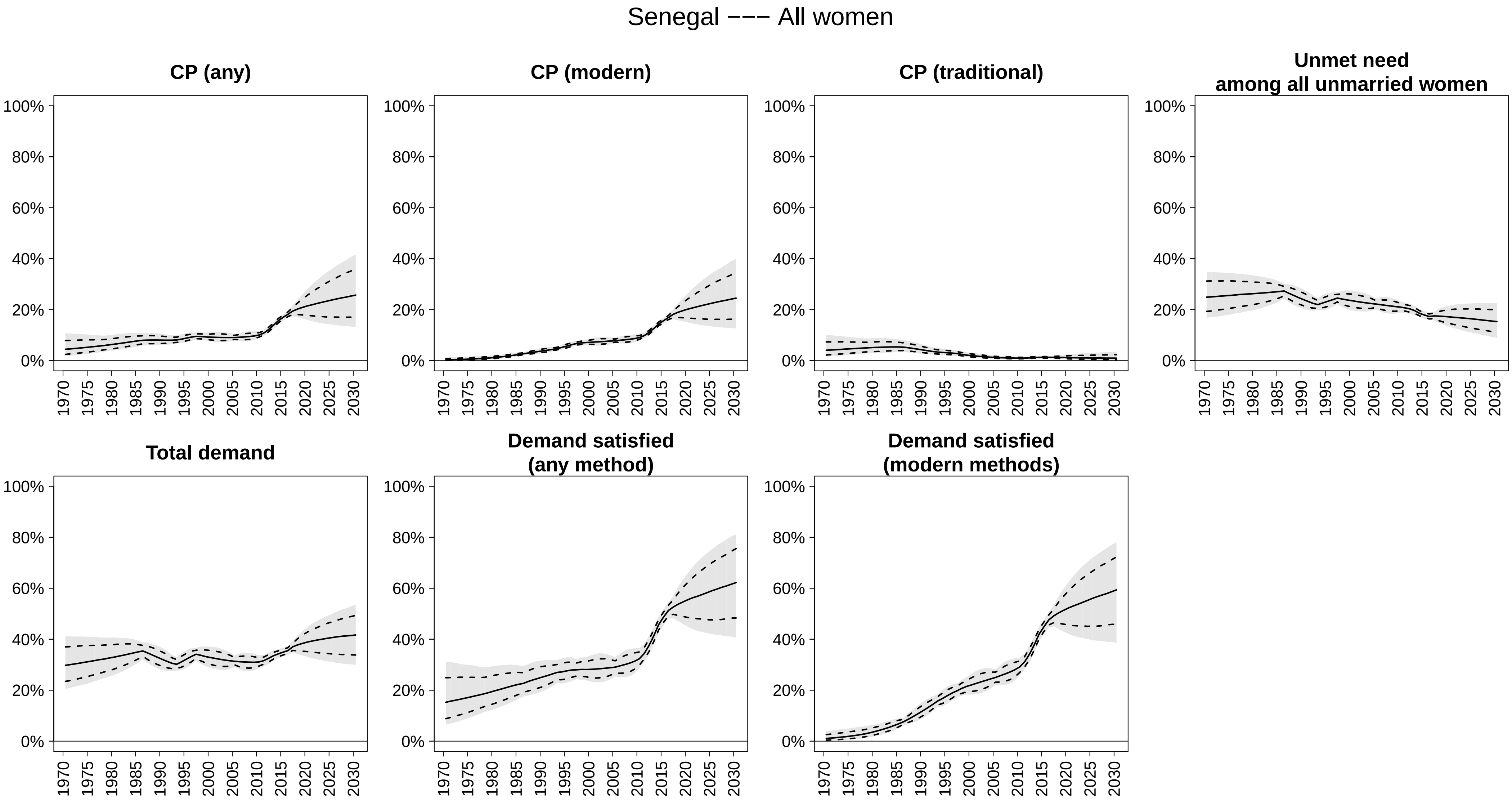
Senegal −−− All women
CP (any)
CP (modern)
CP (traditional)
Unmet need among all unmarried women
Total demand
Demand satisfied (any method)
Demand satisfied (modern methods)
100%
80%
60%
40%
20%
0%
1970
1975
1980
1985
1990
1995
2000
2005
2010
2015
2020
2025
2030

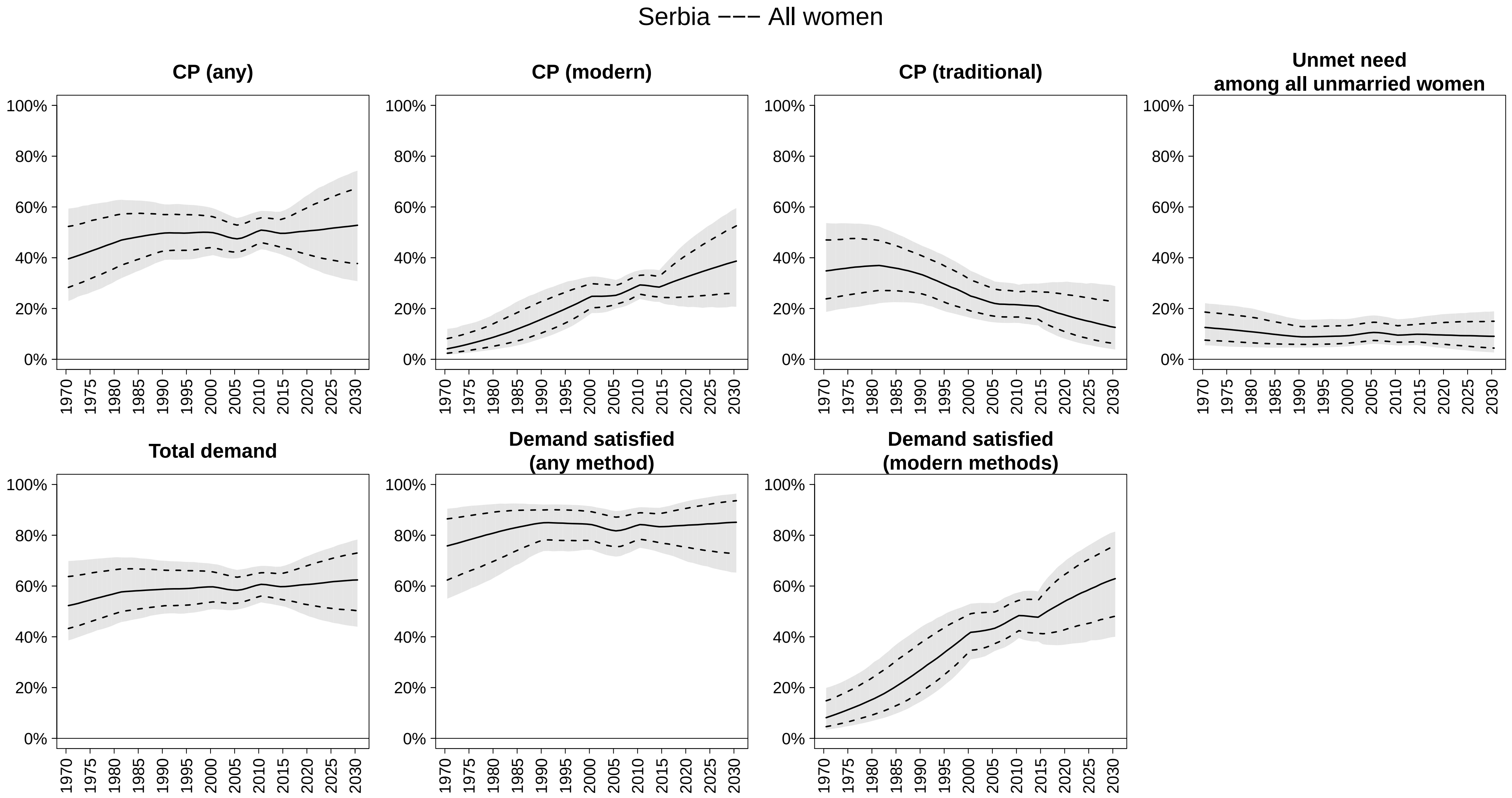
Serbia −−− All women
CP (any)
CP (modern)
CP (traditional)
Unmet need among all unmarried women
Total demand
Demand satisfied (any method)
Demand satisfied (modern methods)
0%
20%
40%
60%
80%
100%
1970
1975
1980
1985
1990
1995
2000
2005
2010
2015
2020
2025
2030

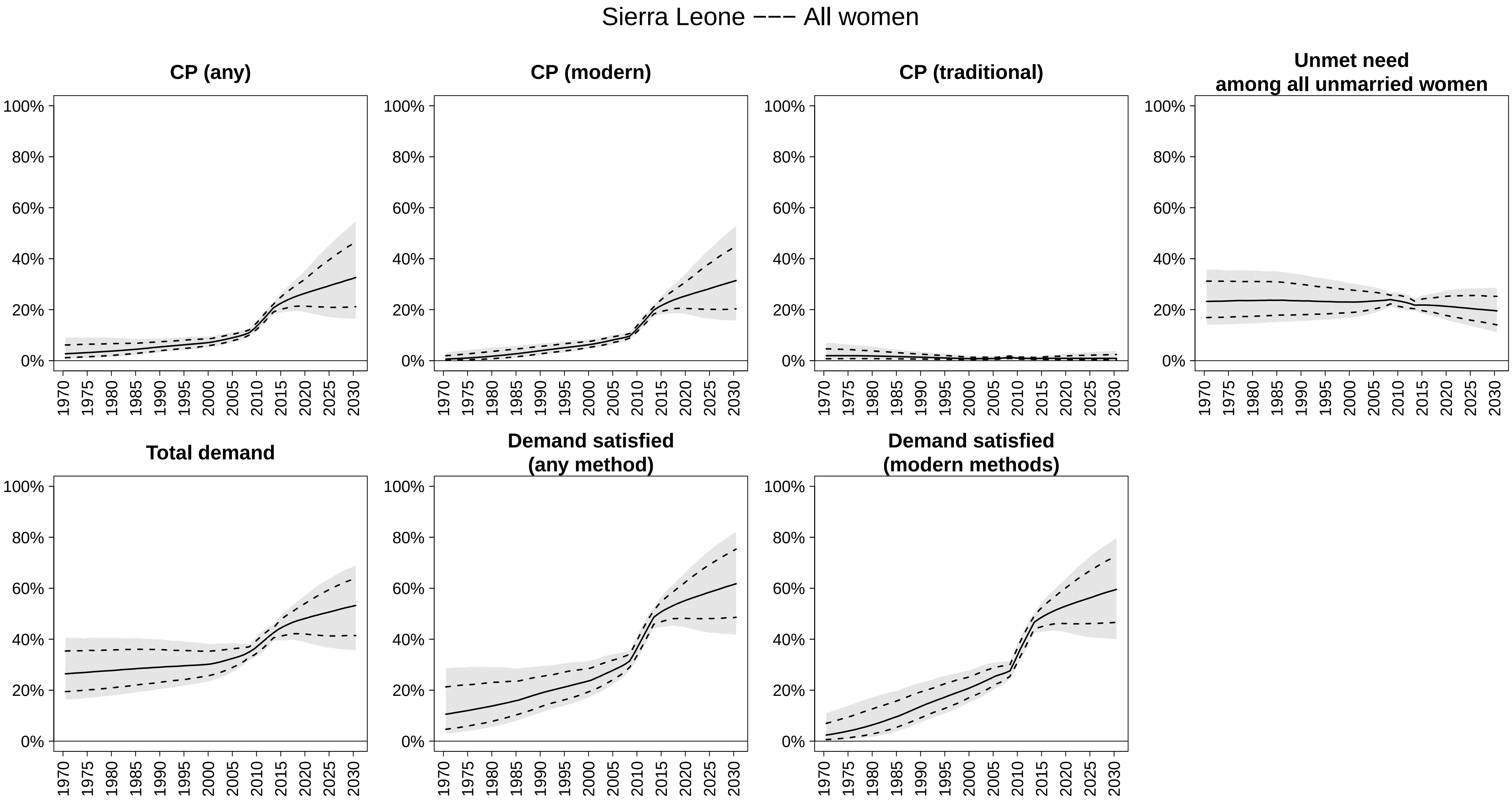
Sierra Leone −−− All women
CP (any)
CP (modern)
CP (traditional)
Unmet need
among all unmarried women
Total demand
Demand satisfied
(any method)
Demand satisfied
(modern methods)
0%
20%
40%
60%
80%
100%
1970
1975
1980
1985
1990
1995
2000
2005
2010
2015
2020
2025
2030

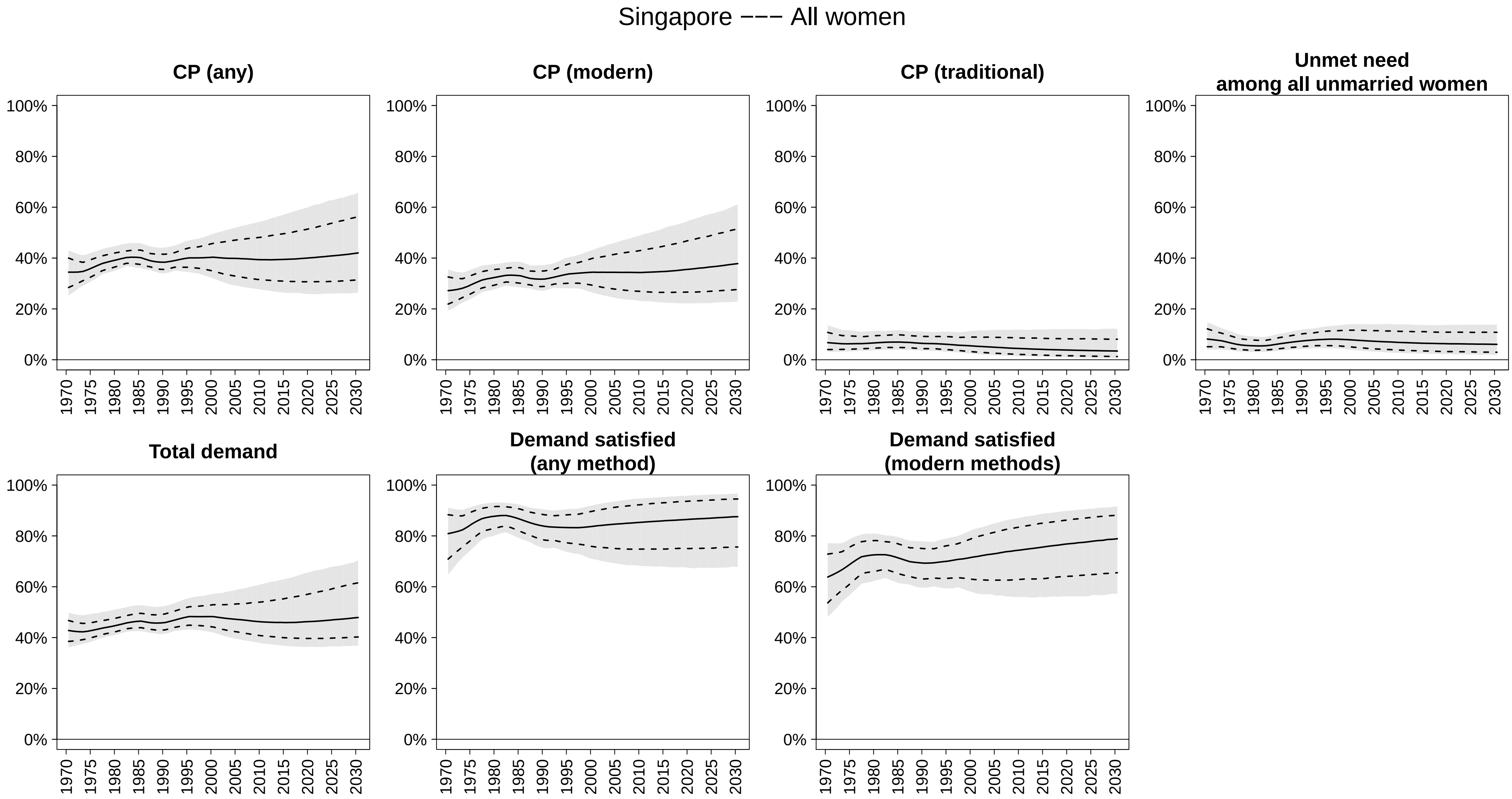
Singapore --- All women
CP (any)
CP (modern)
CP (traditional)
Unmet need
among all unmarried women
Total demand
Demand satisfied
(any method)
Demand satisfied
(modern methods)
0%
20%
40%
60%
80%
100%
1970
1975
1980
1985
1990
1995
2000
2005
2010
2015
2020
2025
2030

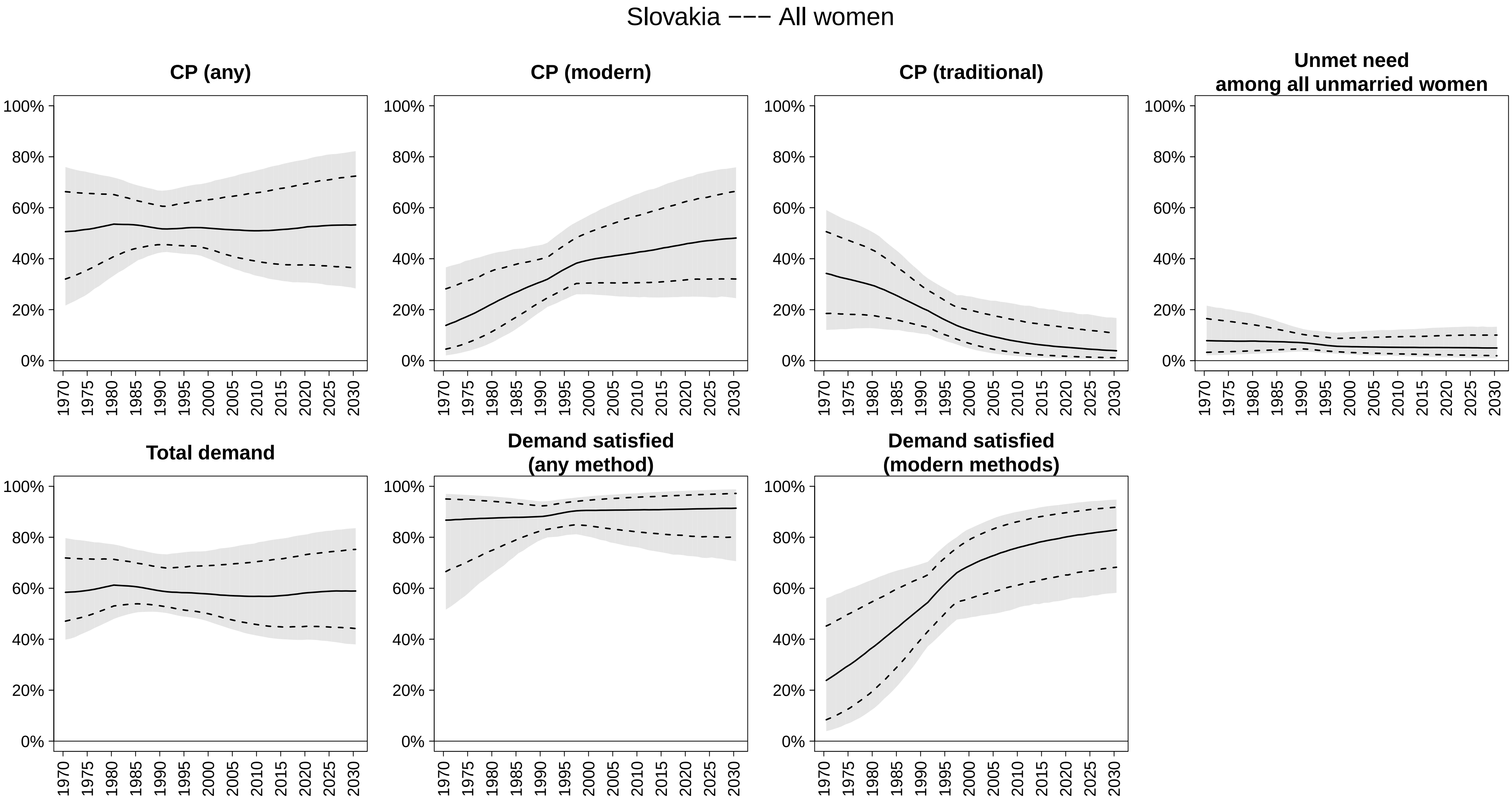
Slovakia −−− All women
CP (any)
CP (modern)
CP (traditional)
Unmet need among all unmarried women
Total demand
Demand satisfied (any method)
Demand satisfied (modern methods)
0%
20%
40%
60%
80%
100%
1970
1975
1980
1985
1990
1995
2000
2005
2010
2015
2020
2025
2030

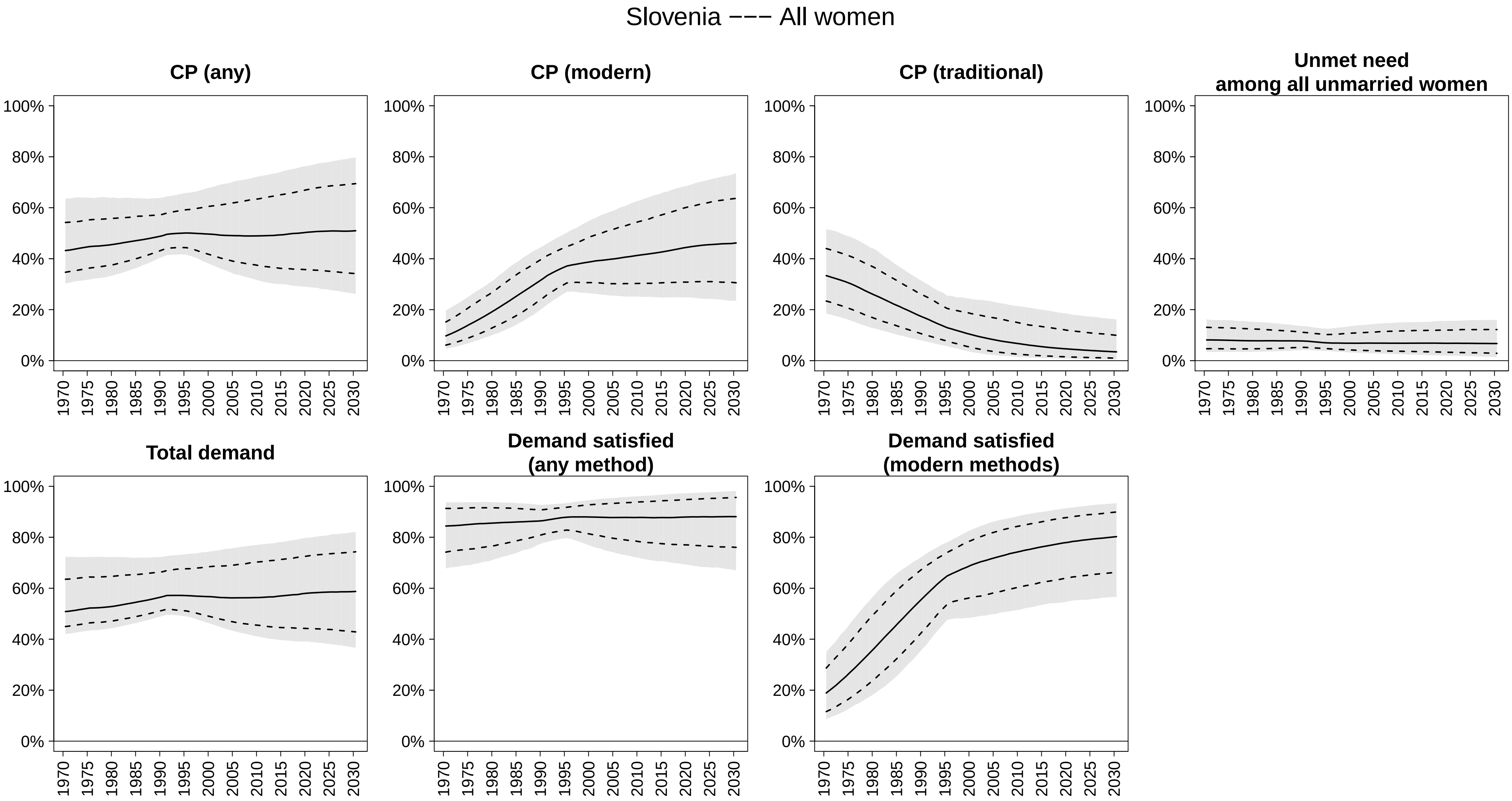
Slovenia −−− All women
CP (any)
CP (modern)
CP (traditional)
Unmet need
among all unmarried women
Total demand
Demand satisfied
(any method)
Demand satisfied
(modern methods)
0%
20%
40%
60%
80%
100%
1970
1975
1980
1985
1990
1995
2000
2005
2010
2015
2020
2025
2030

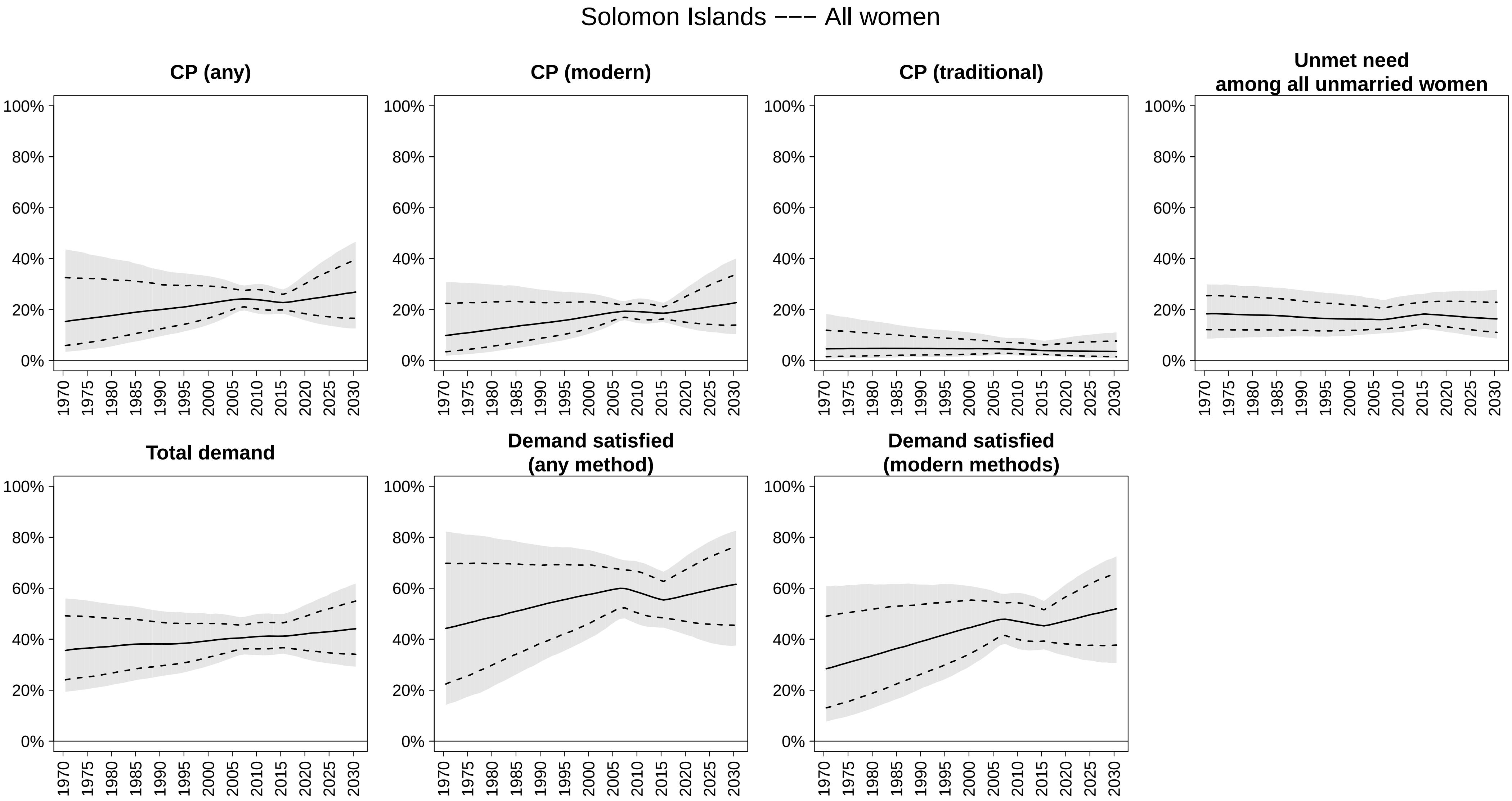
Solomon Islands −−− All women
CP (any)
CP (modern)
CP (traditional)
Unmet need among all unmarried women
Total demand
Demand satisfied (any method)
Demand satisfied (modern methods)
0%
20%
40%
60%
80%
100%
1970
1975
1980
1985
1990
1995
2000
2005
2010
2015
2020
2025
2030

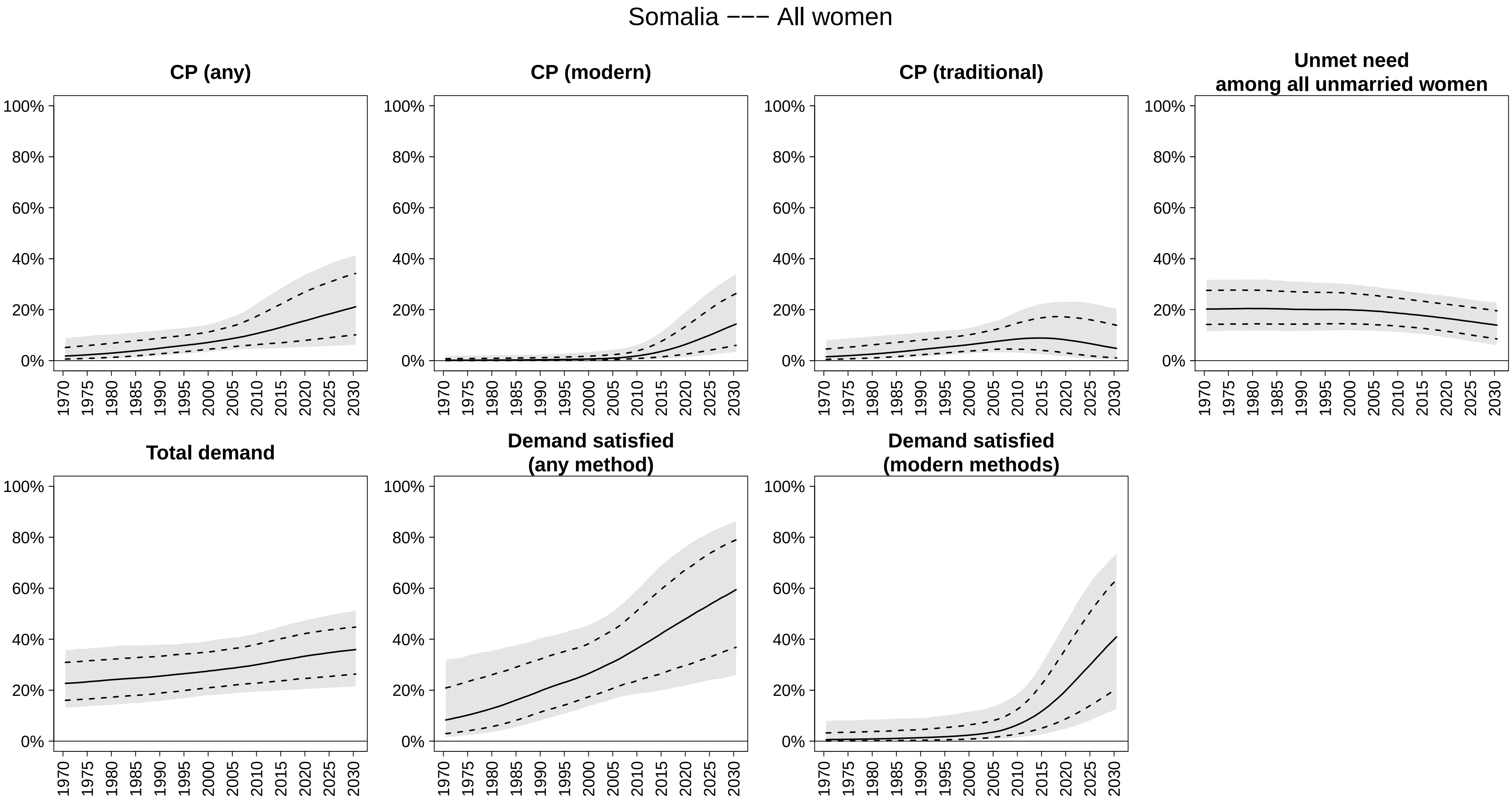
Somalia −−− All women
CP (any)
CP (modern)
CP (traditional)
Unmet need
among all unmarried women
Total demand
Demand satisfied
(any method)
Demand satisfied
(modern methods)
0%
20%
40%
60%
80%
100%
1970
1975
1980
1985
1990
1995
2000
2005
2010
2015
2020
2025
2030

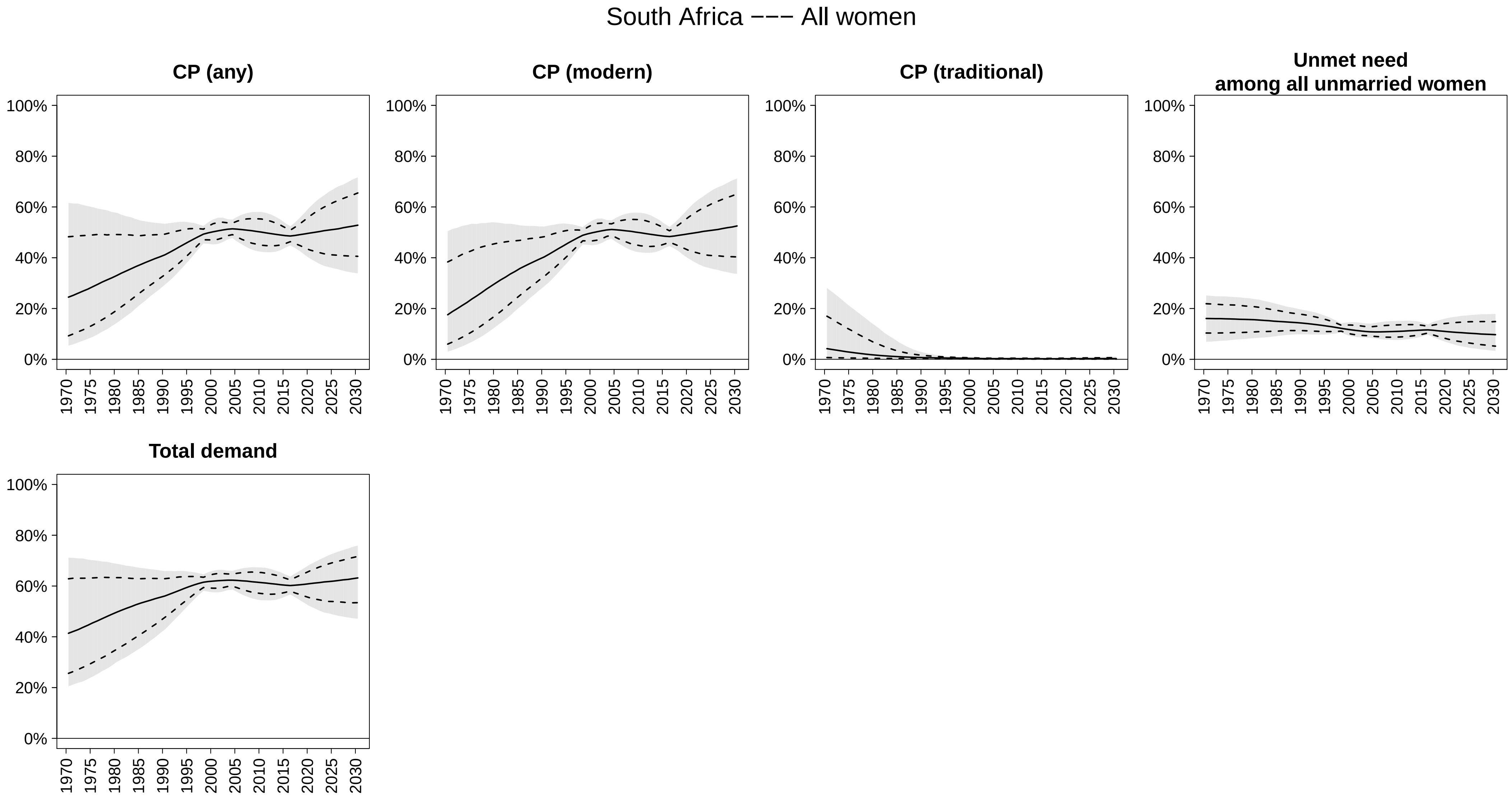
South Africa −−− All women
CP (any)
CP (modern)
CP (traditional)
Unmet need
among all unmarried women
Total demand
0%
20%
40%
60%
80%
100%
1970
1975
1980
1985
1990
1995
2000
2005
2010
2015
2020
2025
2030

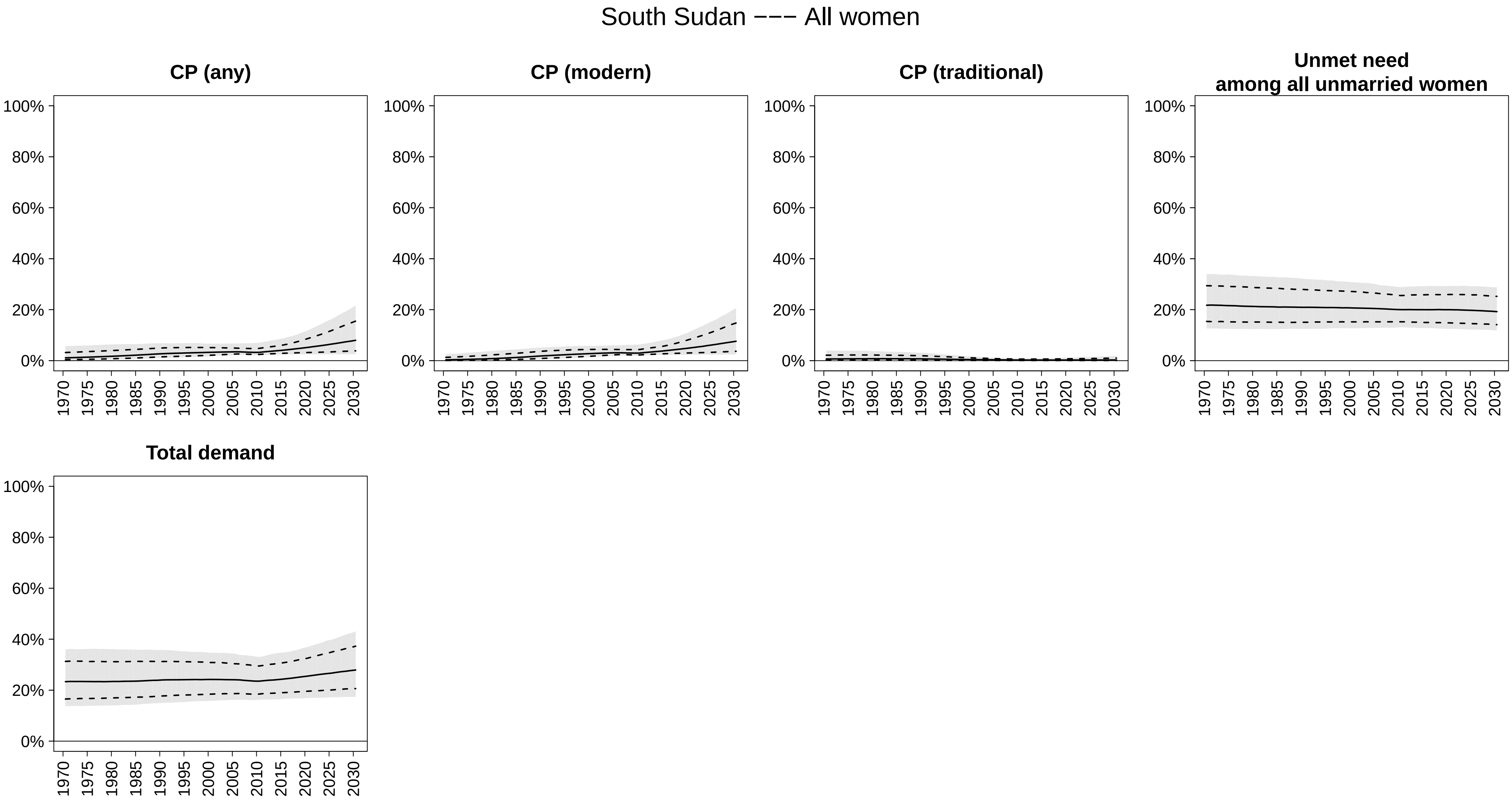
South Sudan −−− All women
CP (any)
CP (modern)
CP (traditional)
Unmet need
among all unmarried women
Total demand
100%
80%
60%
40%
20%
0%
1970
1975
1980
1985
1990
1995
2000
2005
2010
2015
2020
2025
2030

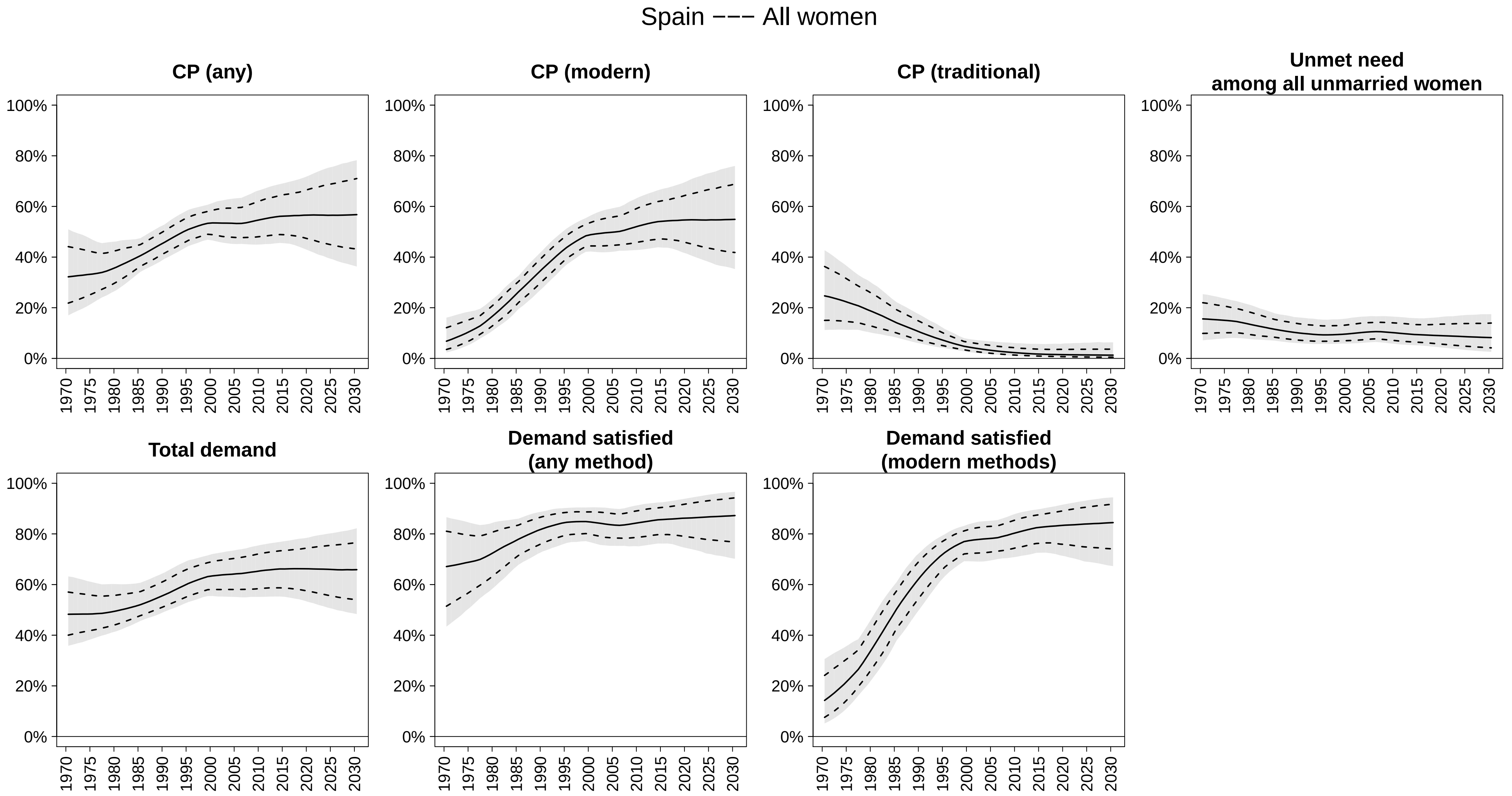
Spain --- All women
CP (any)
CP (modern)
CP (traditional)
Unmet need
among all unmarried women
Total demand
Demand satisfied
(any method)
Demand satisfied
(modern methods)
0%
20%
40%
60%
80%
100%
1970
1975
1980
1985
1990
1995
2000
2005
2010
2015
2020
2025
2030

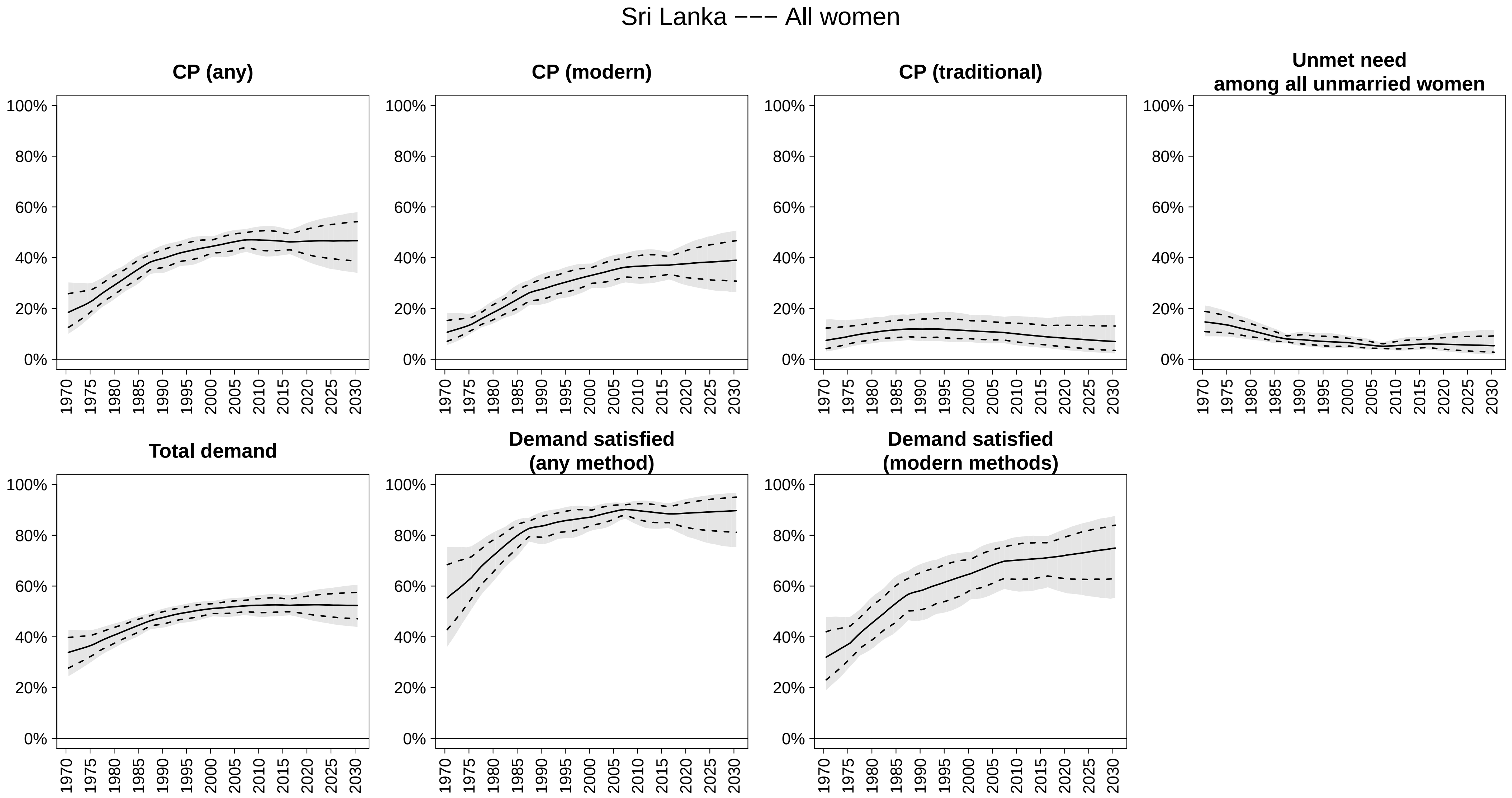
Sri Lanka −−− All women
CP (any)
CP (modern)
CP (traditional)
Unmet need among all unmarried women
Total demand
Demand satisfied (any method)
Demand satisfied (modern methods)
0%
20%
40%
60%
80%
100%
1970
1975
1980
1985
1990
1995
2000
2005
2010
2015
2020
2025
2030

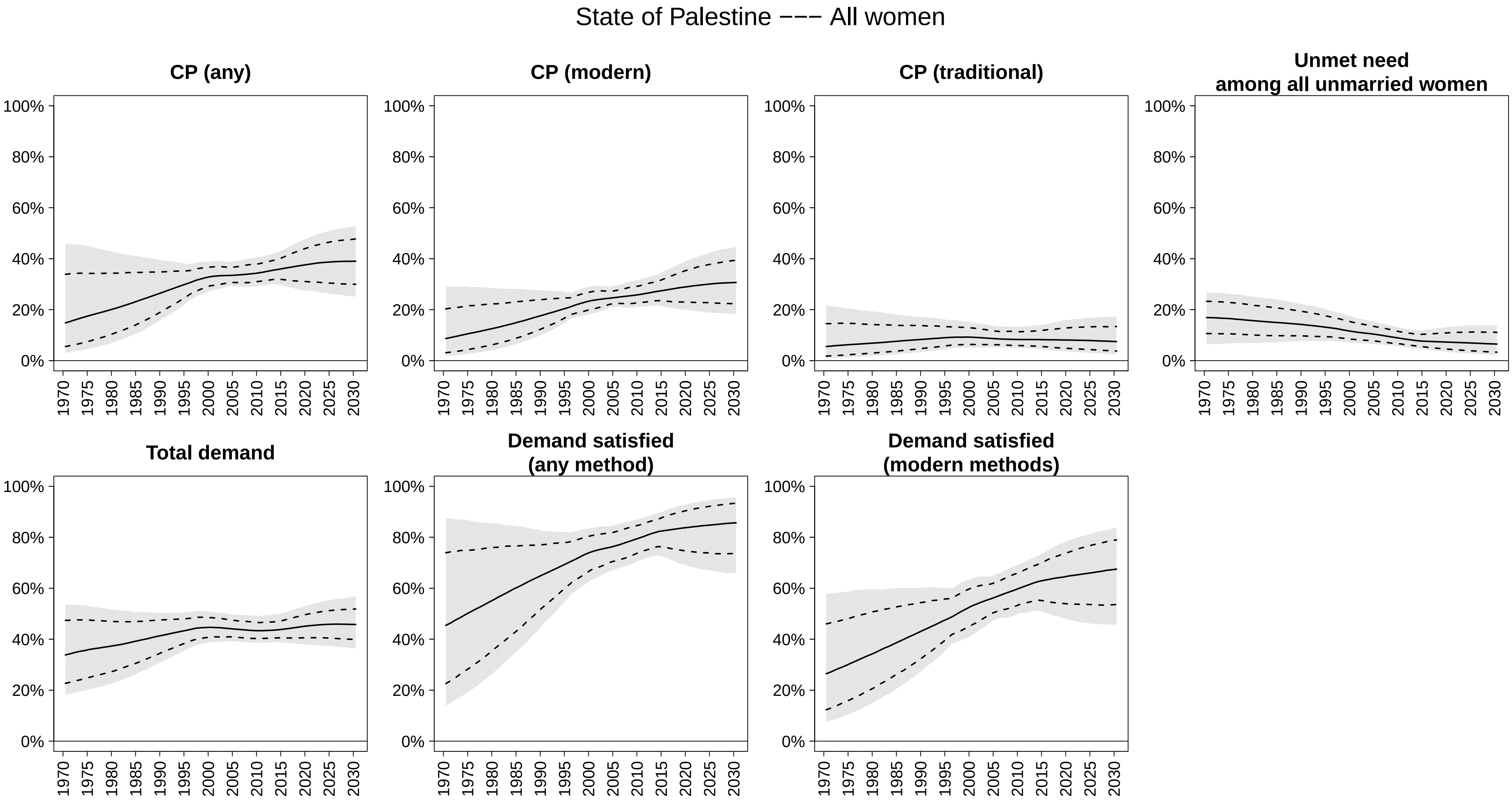
State of Palestine --- All women
CP (any)
CP (modern)
CP (traditional)
Unmet need among all unmarried women
Total demand
Demand satisfied (any method)
Demand satisfied (modern methods)
0%
20%
40%
60%
80%
100%
1970
1975
1980
1985
1990
1995
2000
2005
2010
2015
2020
2025
2030

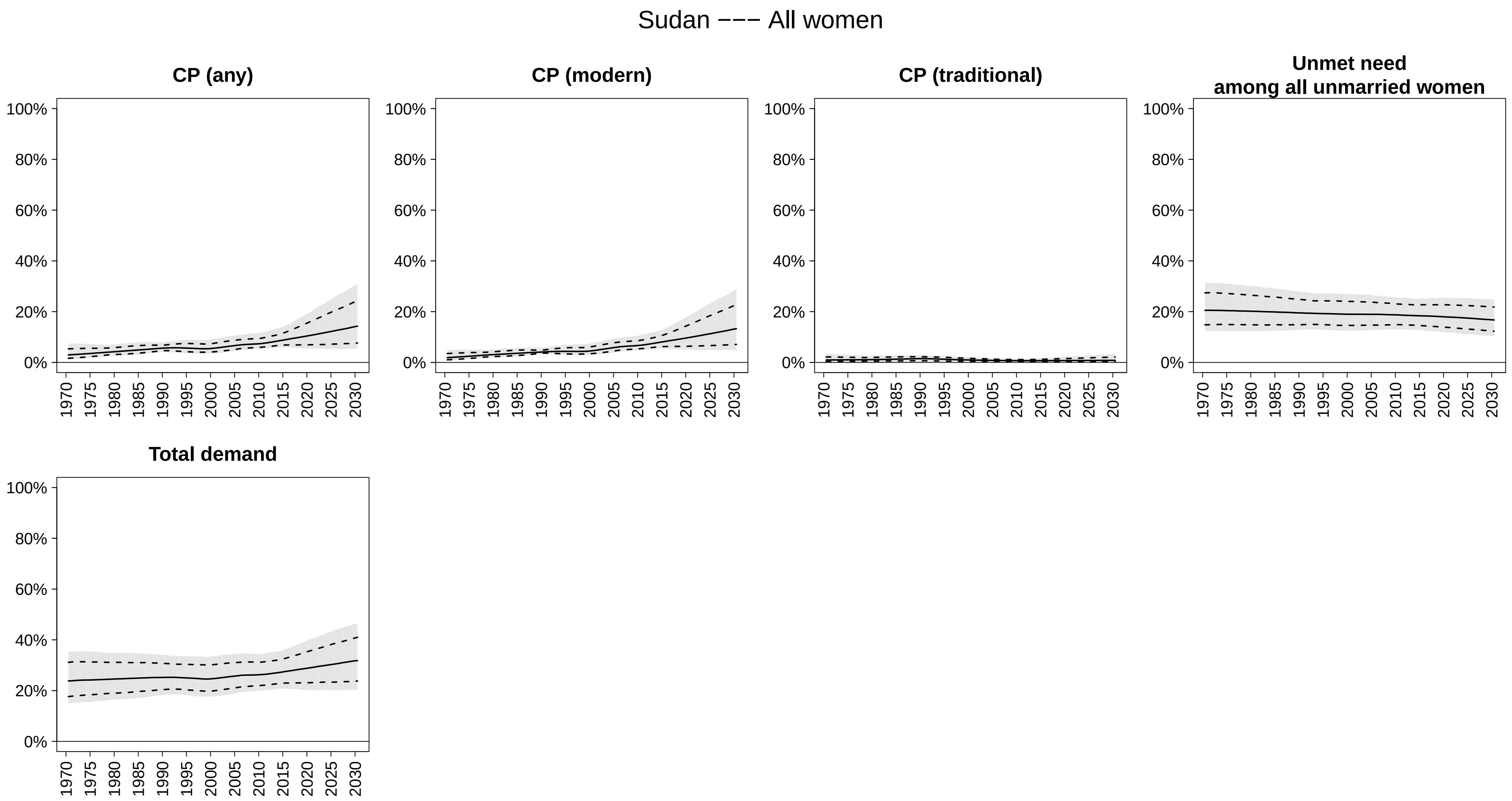
Sudan −−− All women
CP (any)
CP (modern)
CP (traditional)
Unmet need
among all unmarried women
Total demand
0%
20%
40%
60%
80%
100%
1970
1975
1980
1985
1990
1995
2000
2005
2010
2015
2020
2025
2030

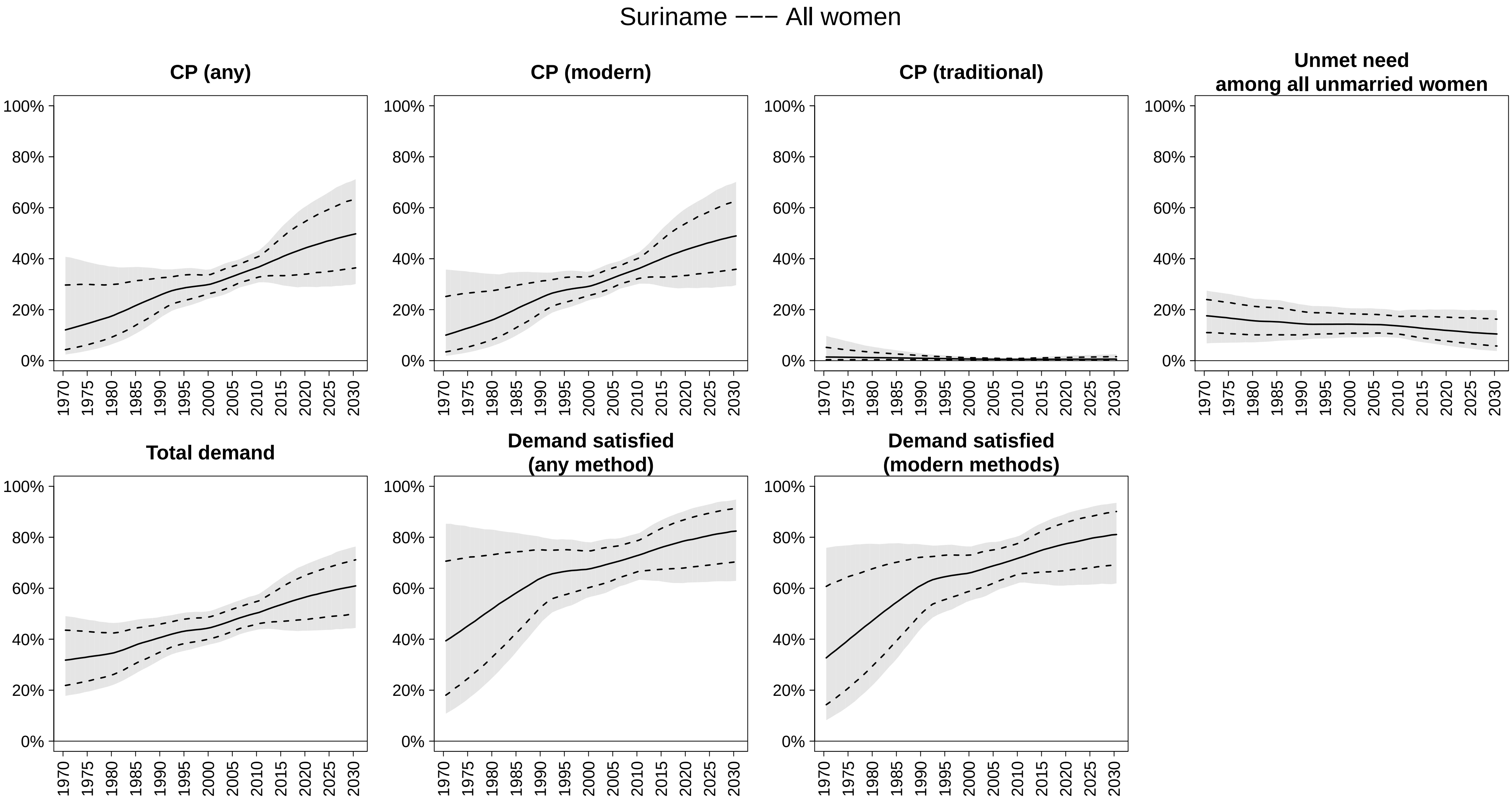
Suriname −−− All women
CP (any)
CP (modern)
CP (traditional)
Unmet need among all unmarried women
Total demand
Demand satisfied (any method)
Demand satisfied (modern methods)
0%
20%
40%
60%
80%
100%
1970
1975
1980
1985
1990
1995
2000
2005
2010
2015
2020
2025
2030

Sweden −−− All women

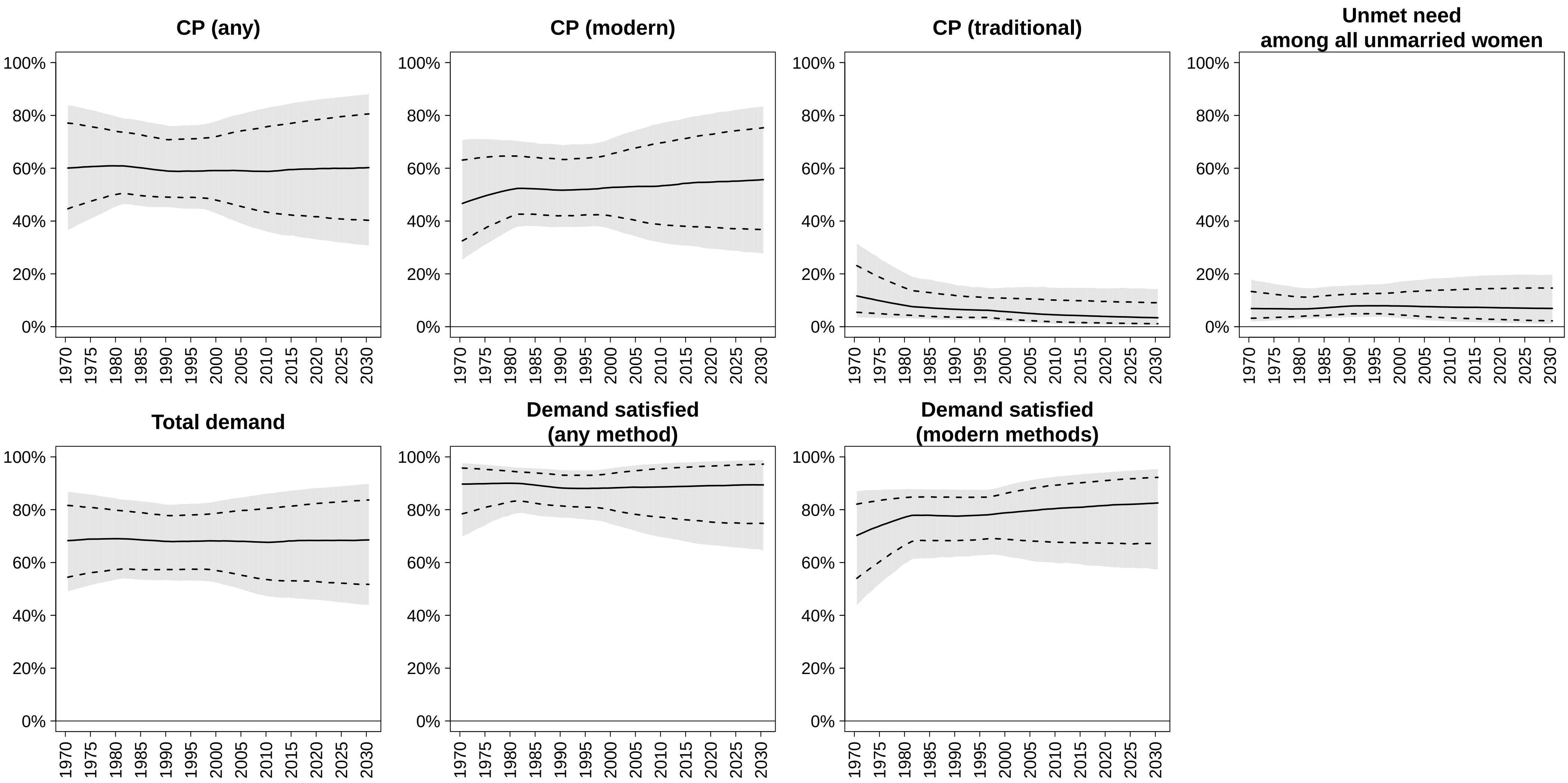

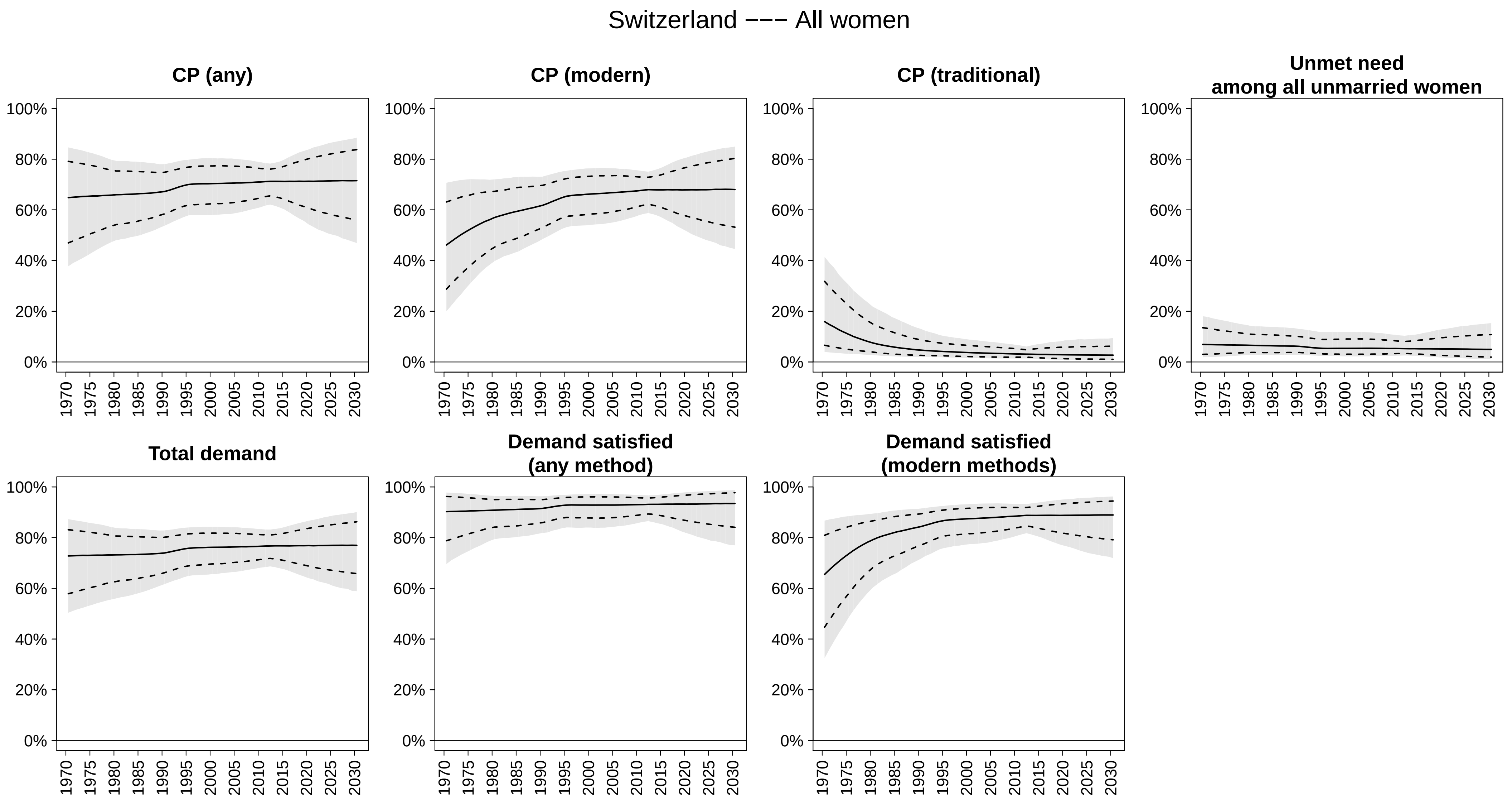
Switzerland −−− All women
CP (any)
CP (modern)
CP (traditional)
Unmet need
among all unmarried women
Total demand
Demand satisfied
(any method)
Demand satisfied
(modern methods)
0%
20%
40%
60%
80%
100%
1970
1975
1980
1985
1990
1995
2000
2005
2010
2015
2020
2025
2030

Syrian Arab Republic −−− All women

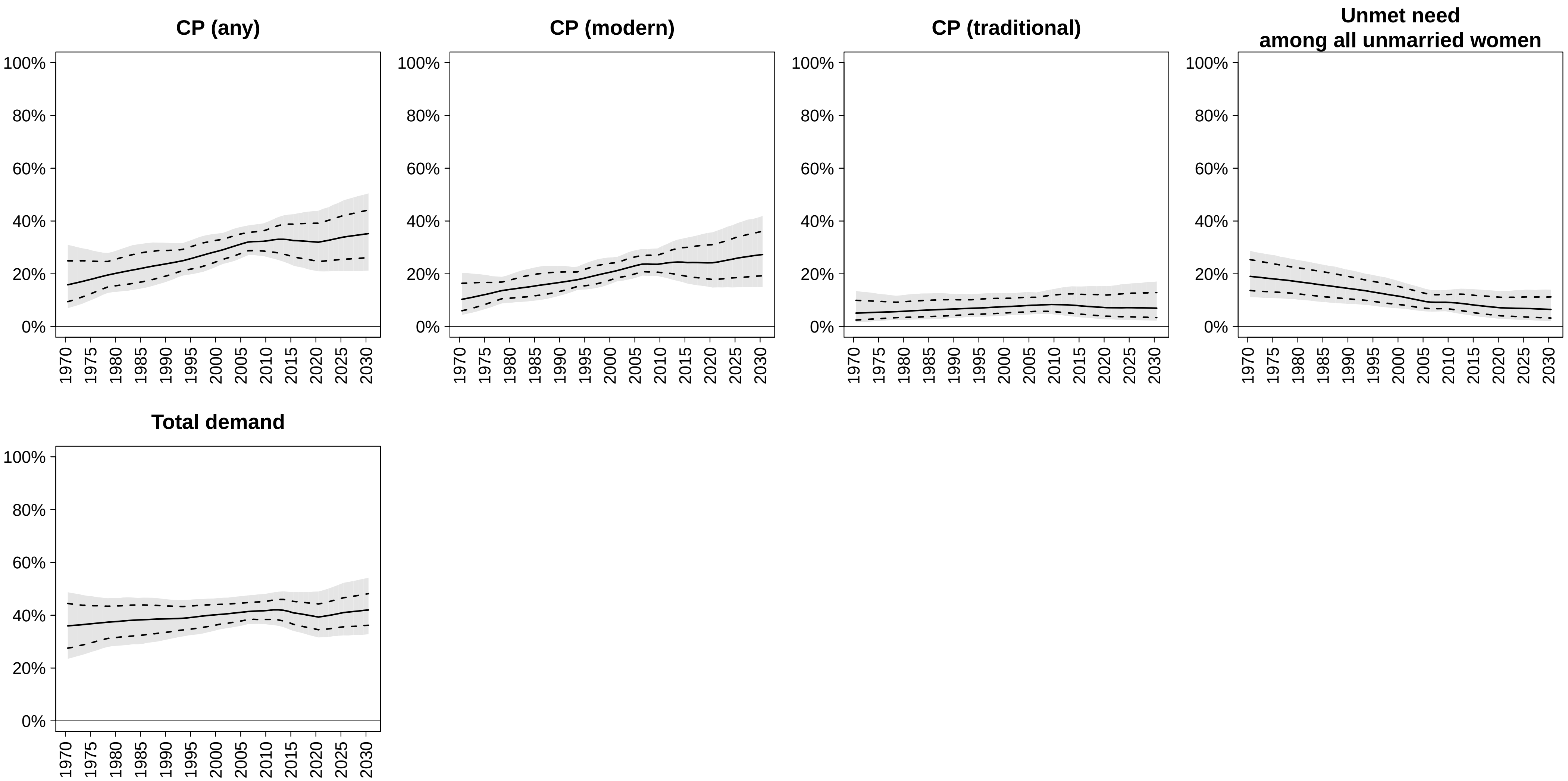

Tajikistan --- All women

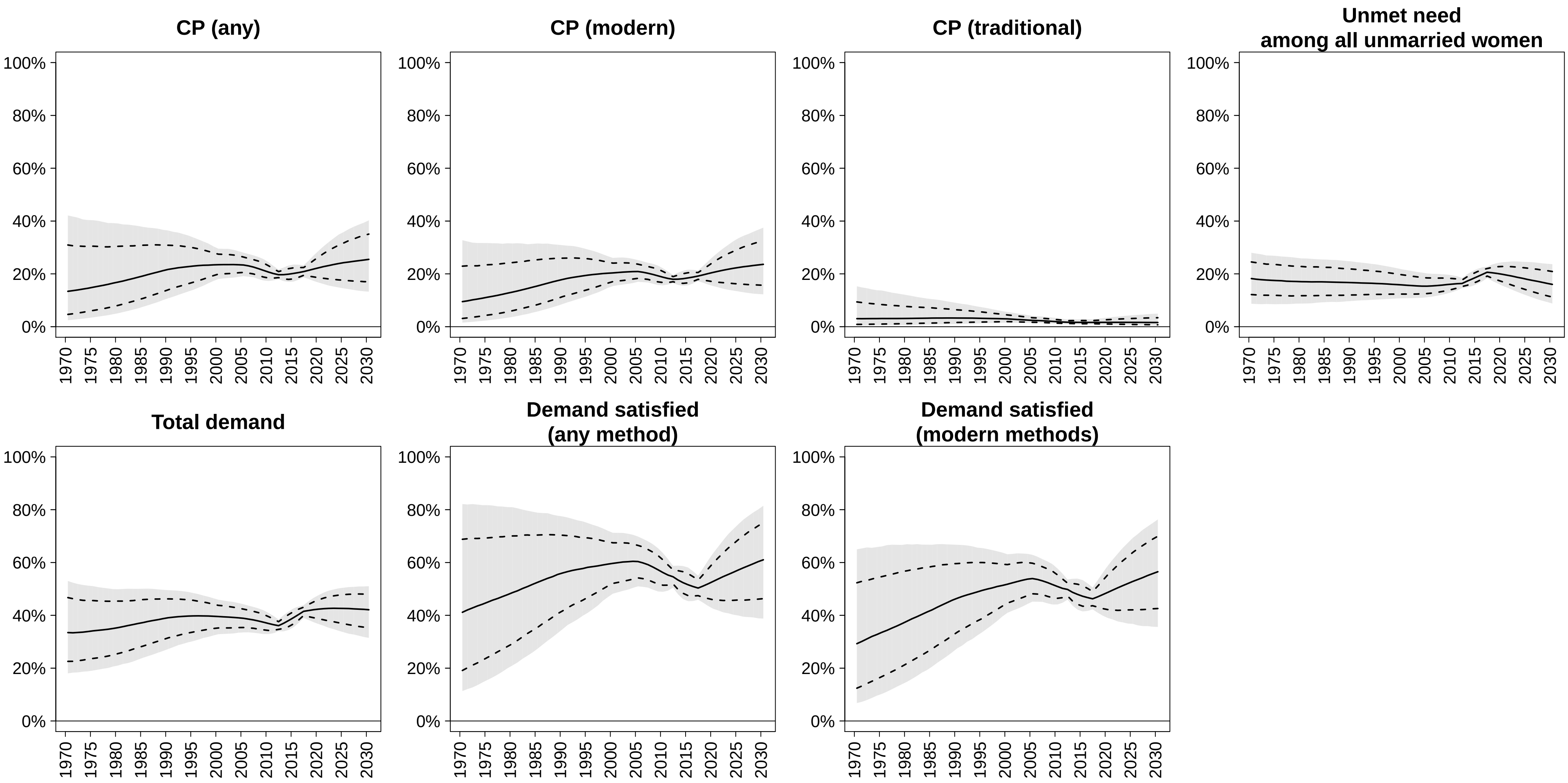

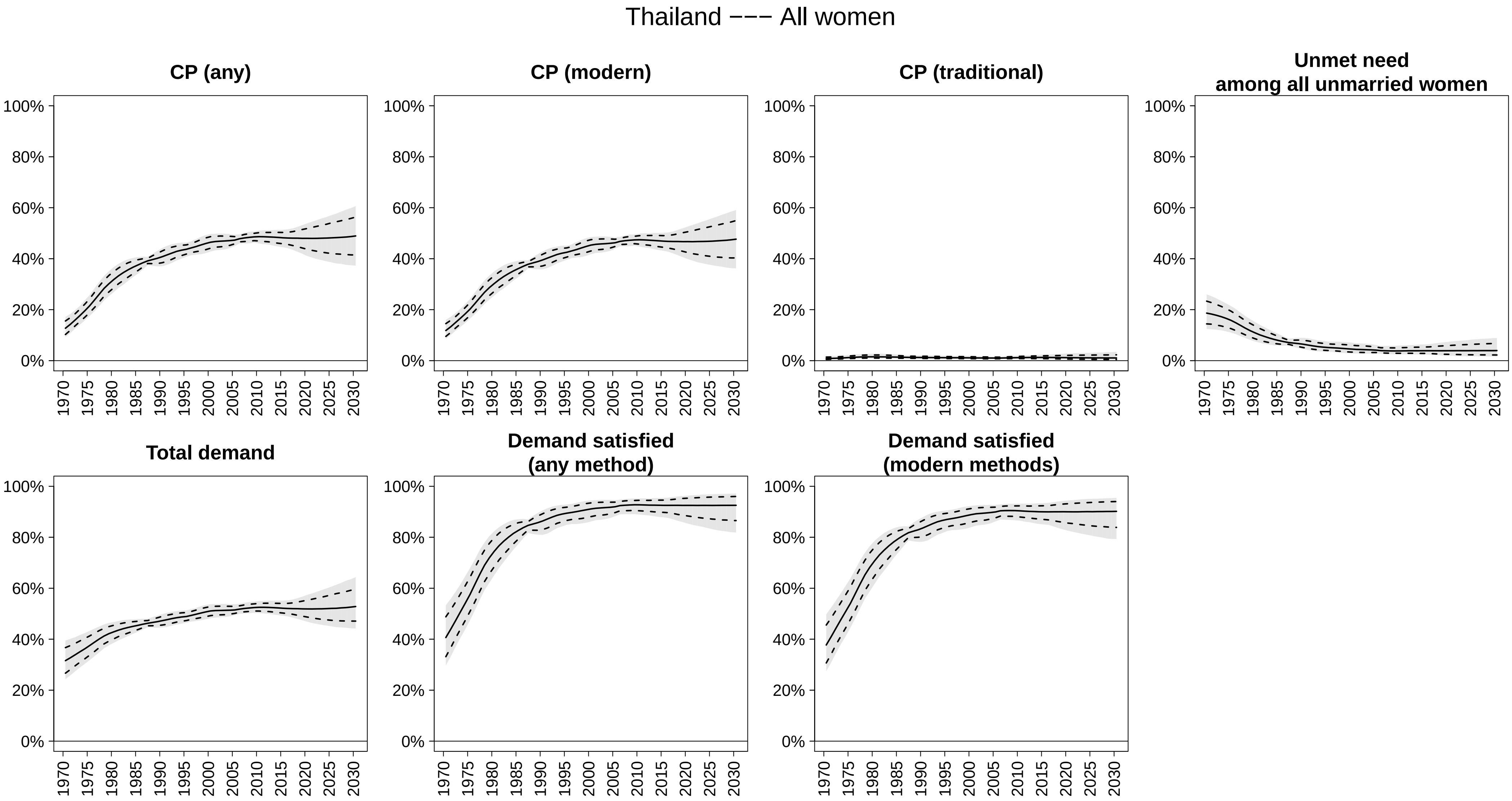
Thailand −−− All women
CP (any)
CP (modern)
CP (traditional)
Unmet need among all unmarried women
Total demand
Demand satisfied (any method)
Demand satisfied (modern methods)
0%
20%
40%
60%
80%
100%
1970
1975
1980
1985
1990
1995
2000
2005
2010
2015
2020
2025
2030

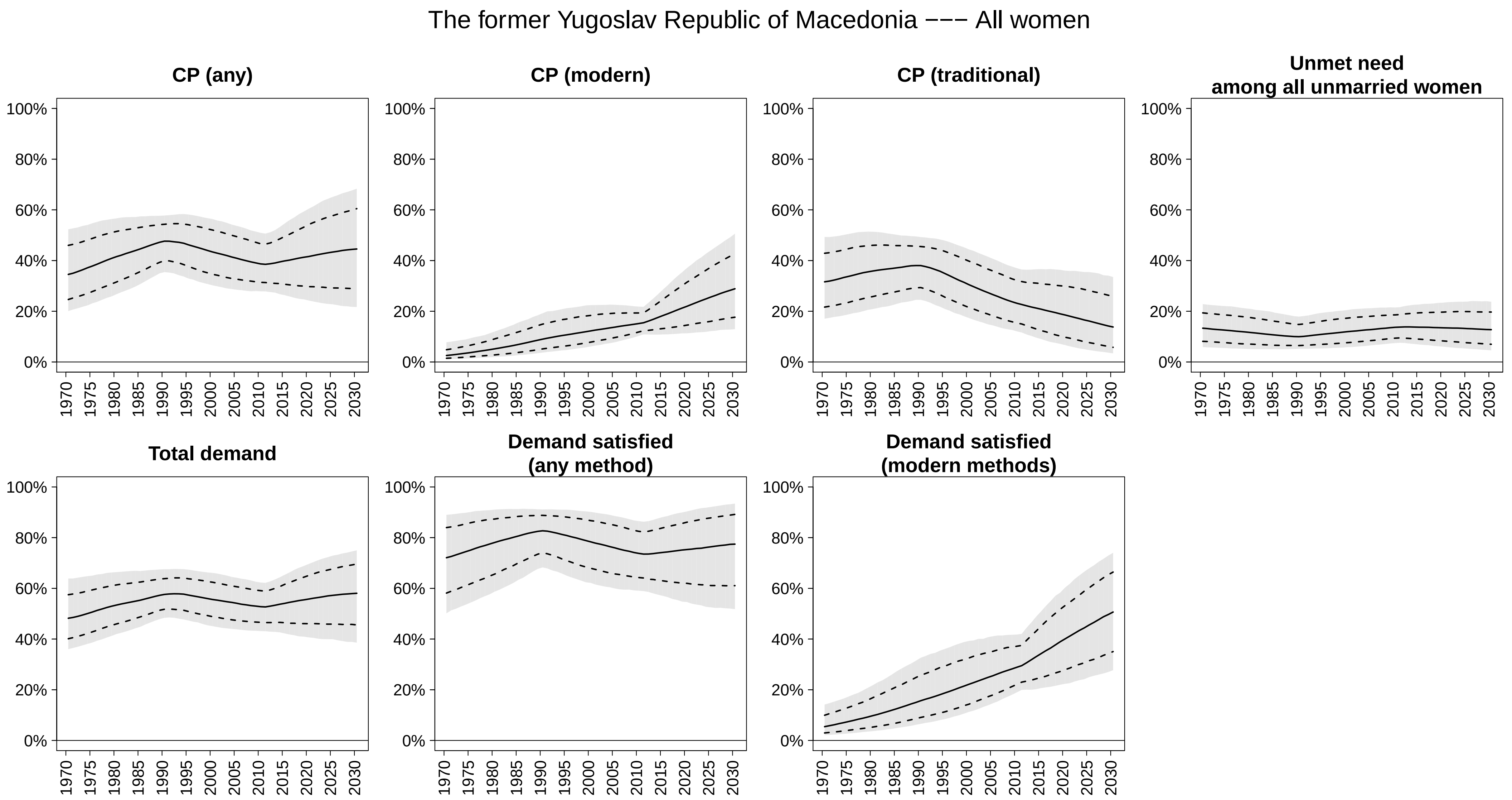
The former Yugoslav Republic of Macedonia −−− All women
CP (any)
CP (modern)
CP (traditional)
Unmet need among all unmarried women
Total demand
Demand satisfied (any method)
Demand satisfied (modern methods)
0%
20%
40%
60%
80%
100%
1970
1975
1980
1985
1990
1995
2000
2005
2010
2015
2020
2025
2030

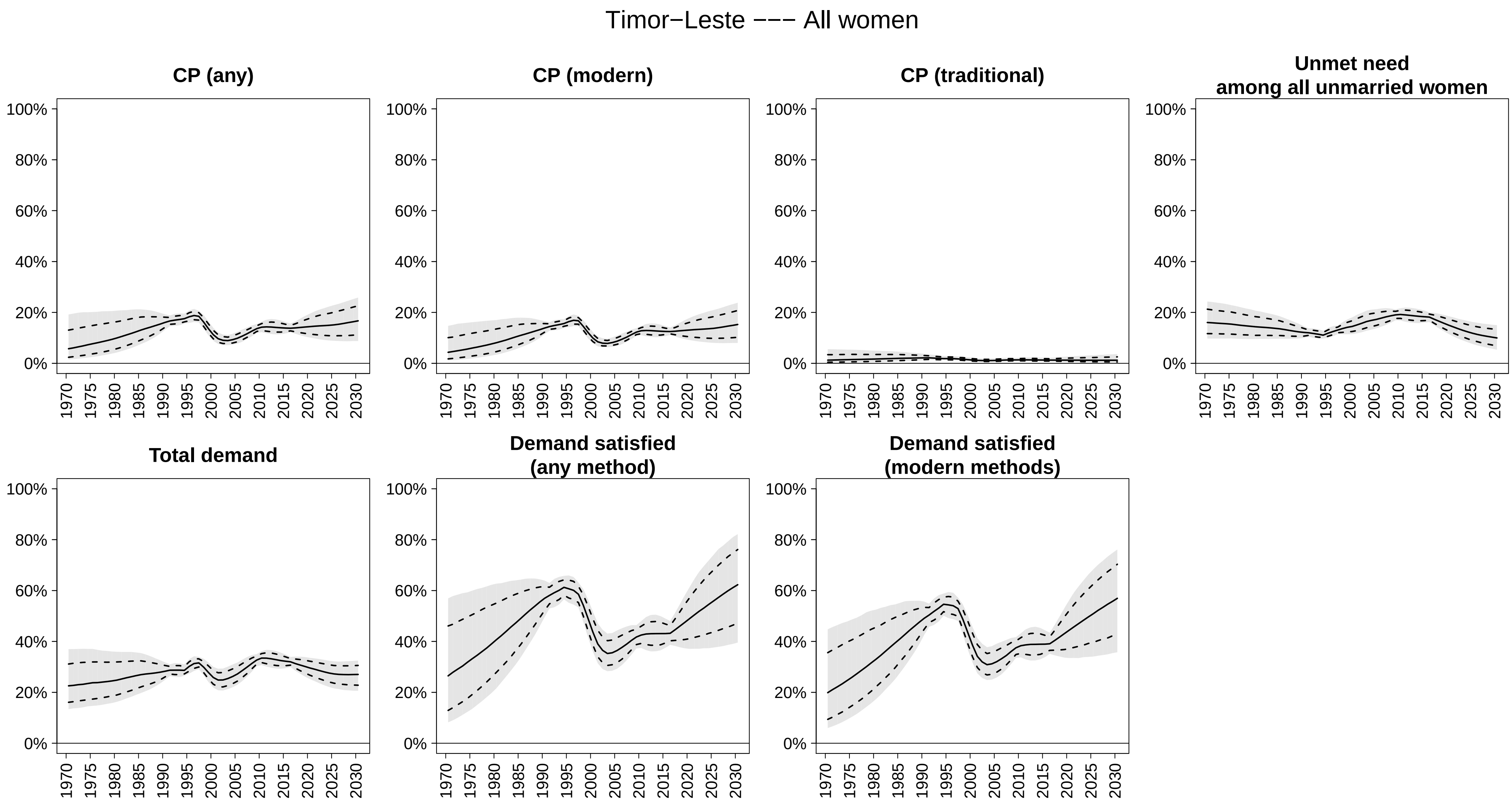
Timor−Leste −−− All women
CP (any)
CP (modern)
CP (traditional)
Unmet need among all unmarried women
Total demand
Demand satisfied (any method)
Demand satisfied (modern methods)
0%
20%
40%
60%
80%
100%
1970
1975
1980
1985
1990
1995
2000
2005
2010
2015
2020
2025
2030

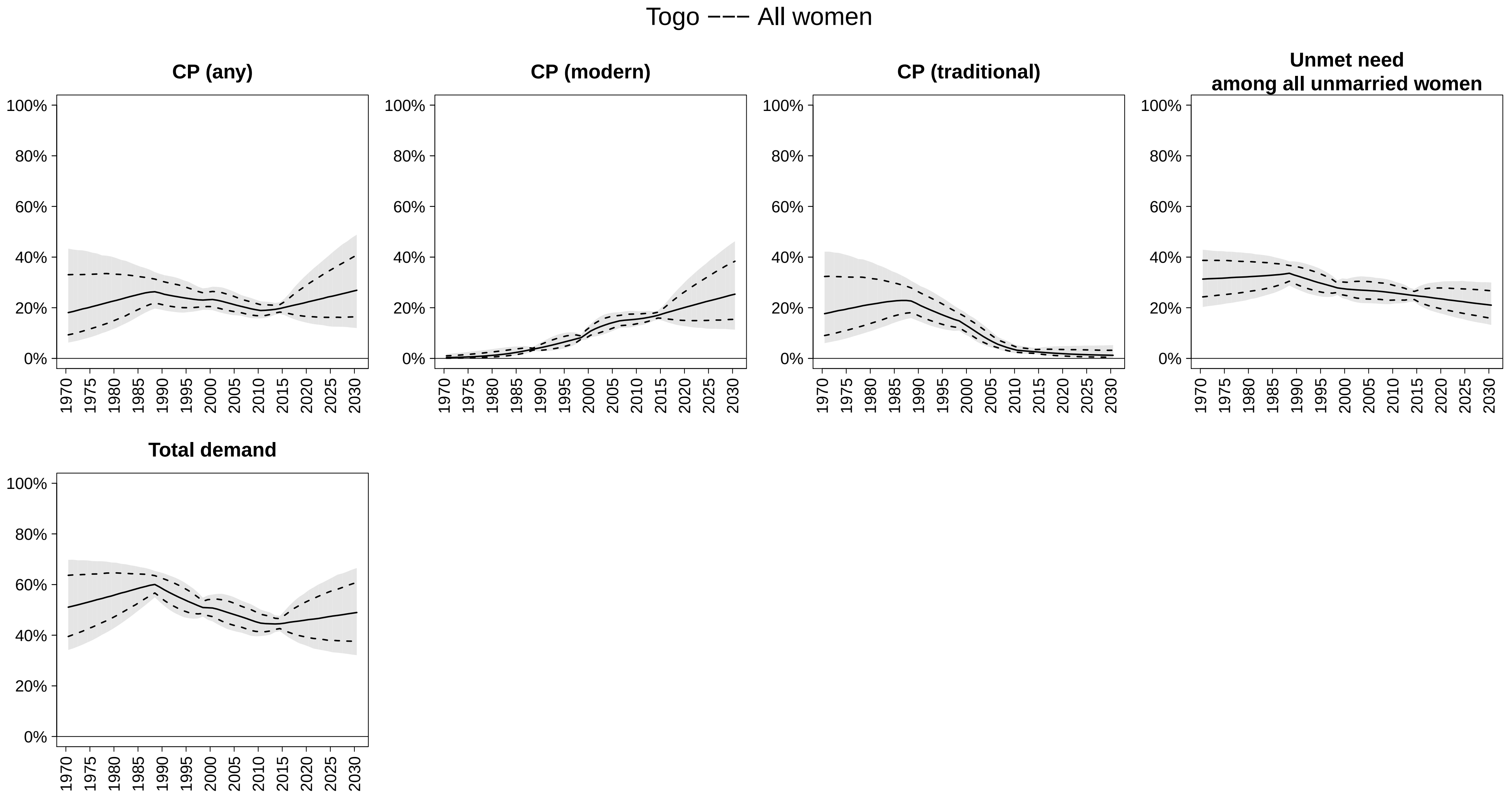
Togo −−− All women
CP (any)
CP (modern)
CP (traditional)
Unmet need among all unmarried women
Total demand
0%
20%
40%
60%
80%
100%
1970
1975
1980
1985
1990
1995
2000
2005
2010
2015
2020
2025
2030

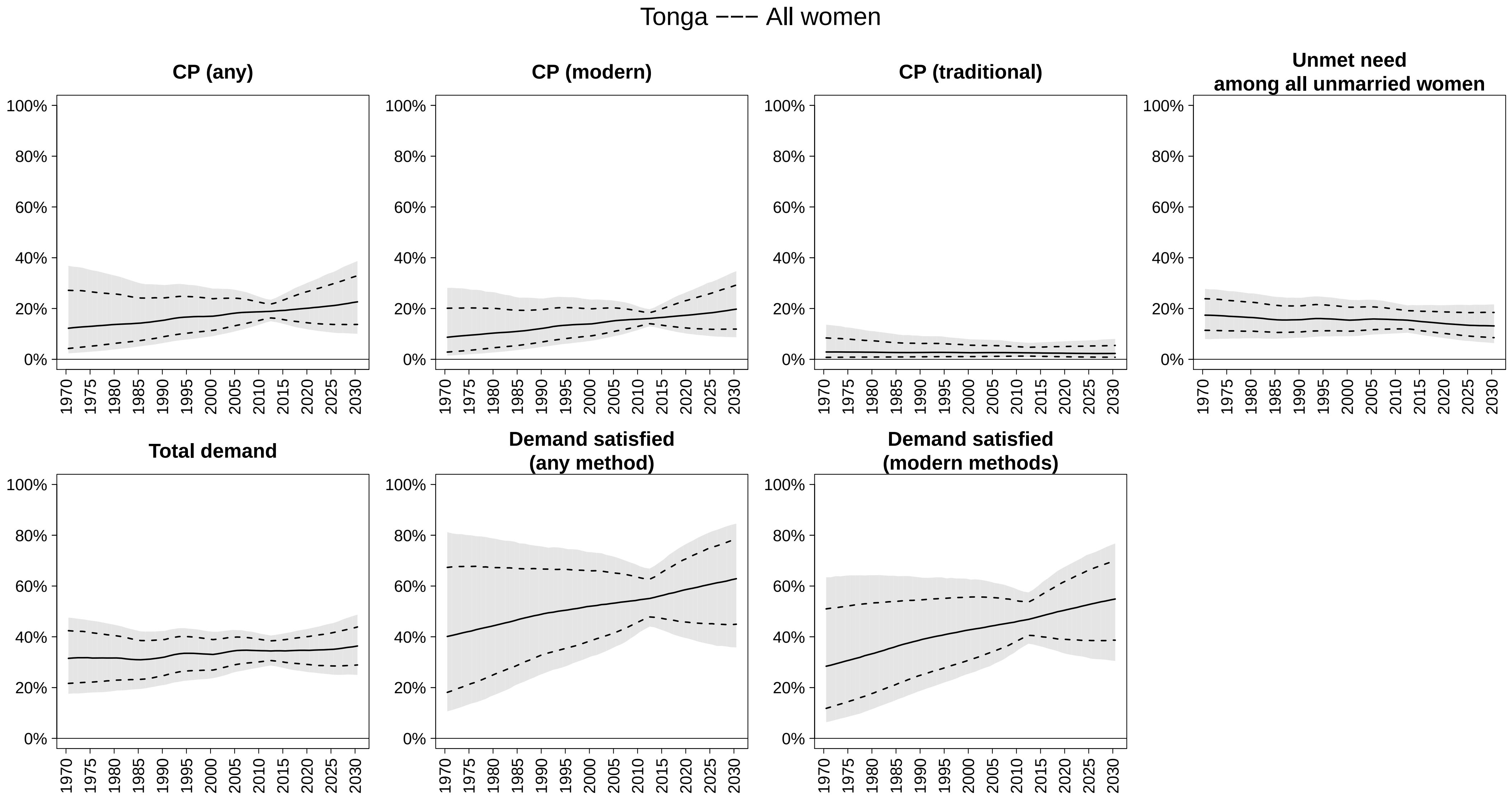
Tonga −−− All women
CP (any)
CP (modern)
CP (traditional)
Unmet need
among all unmarried women
Total demand
Demand satisfied
(any method)
Demand satisfied
(modern methods)
0%
20%
40%
60%
80%
100%
1970
1975
1980
1985
1990
1995
2000
2005
2010
2015
2020
2025
2030

Trinidad and Tobago −−− All women

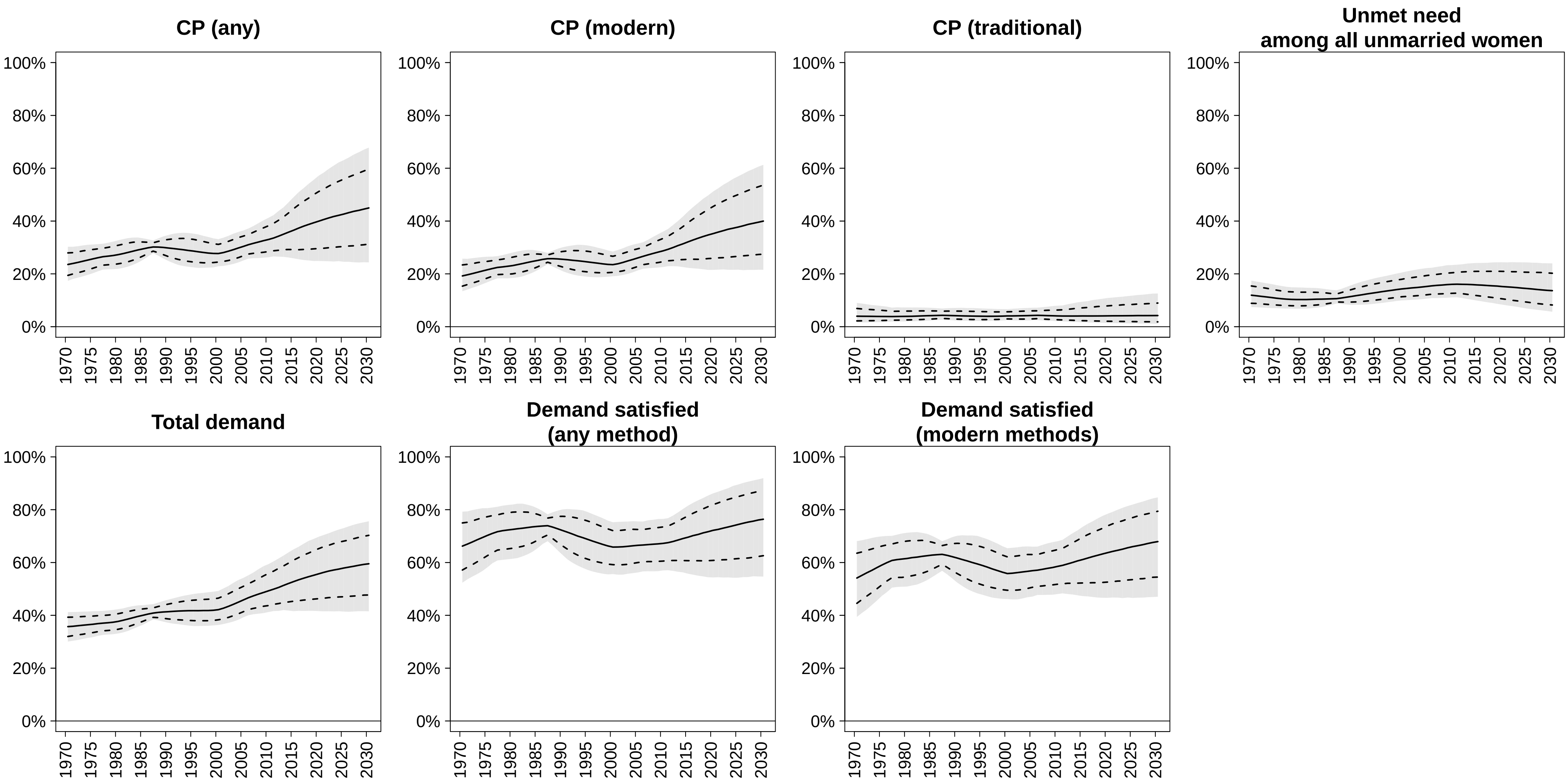

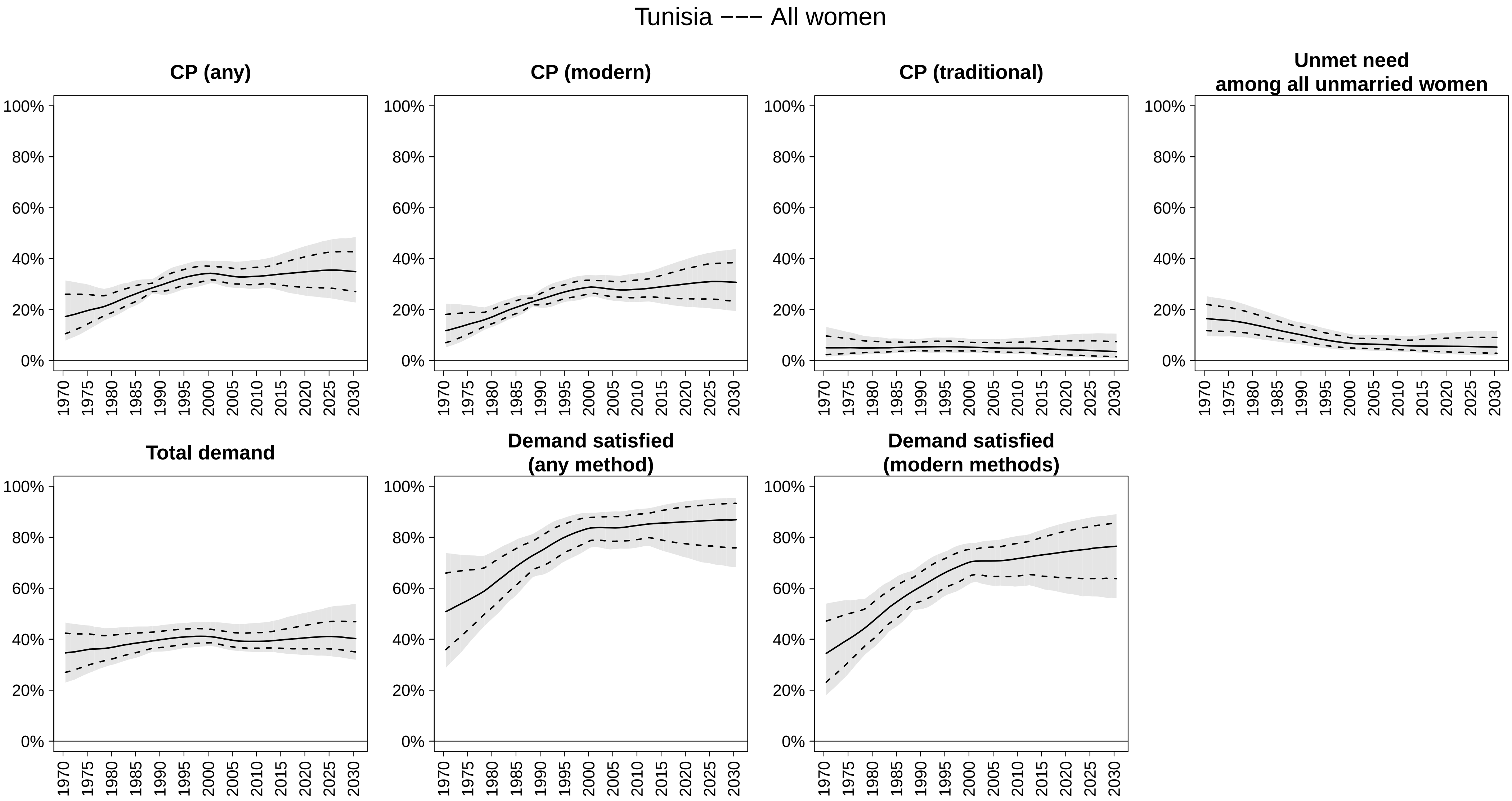
Tunisia −−− All women
CP (any)
CP (modern)
CP (traditional)
Unmet need
among all unmarried women
Total demand
Demand satisfied
(any method)
Demand satisfied
(modern methods)
0%
20%
40%
60%
80%
100%
1970
1975
1980
1985
1990
1995
2000
2005
2010
2015
2020
2025
2030

Turkey −−− All women

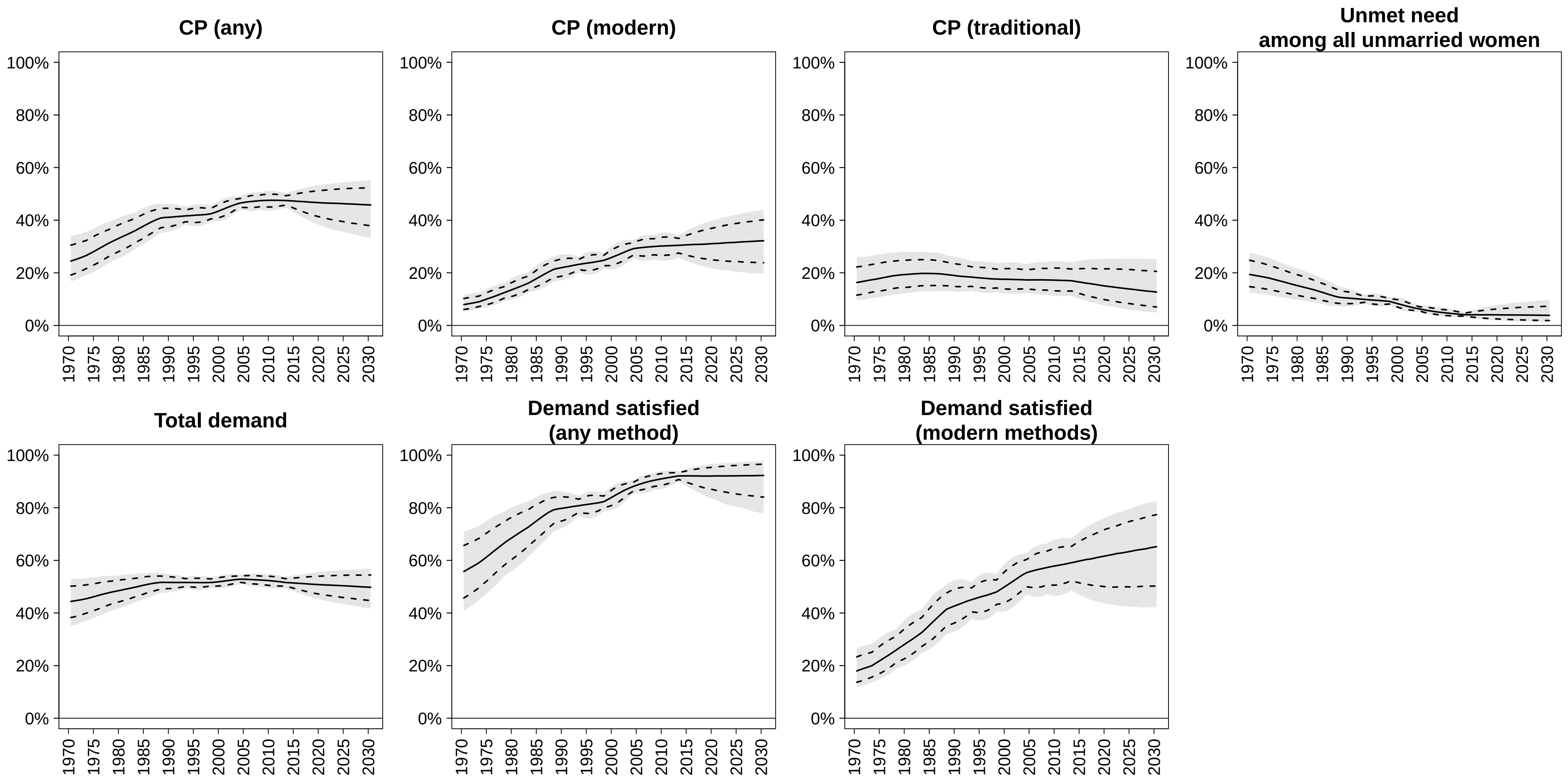

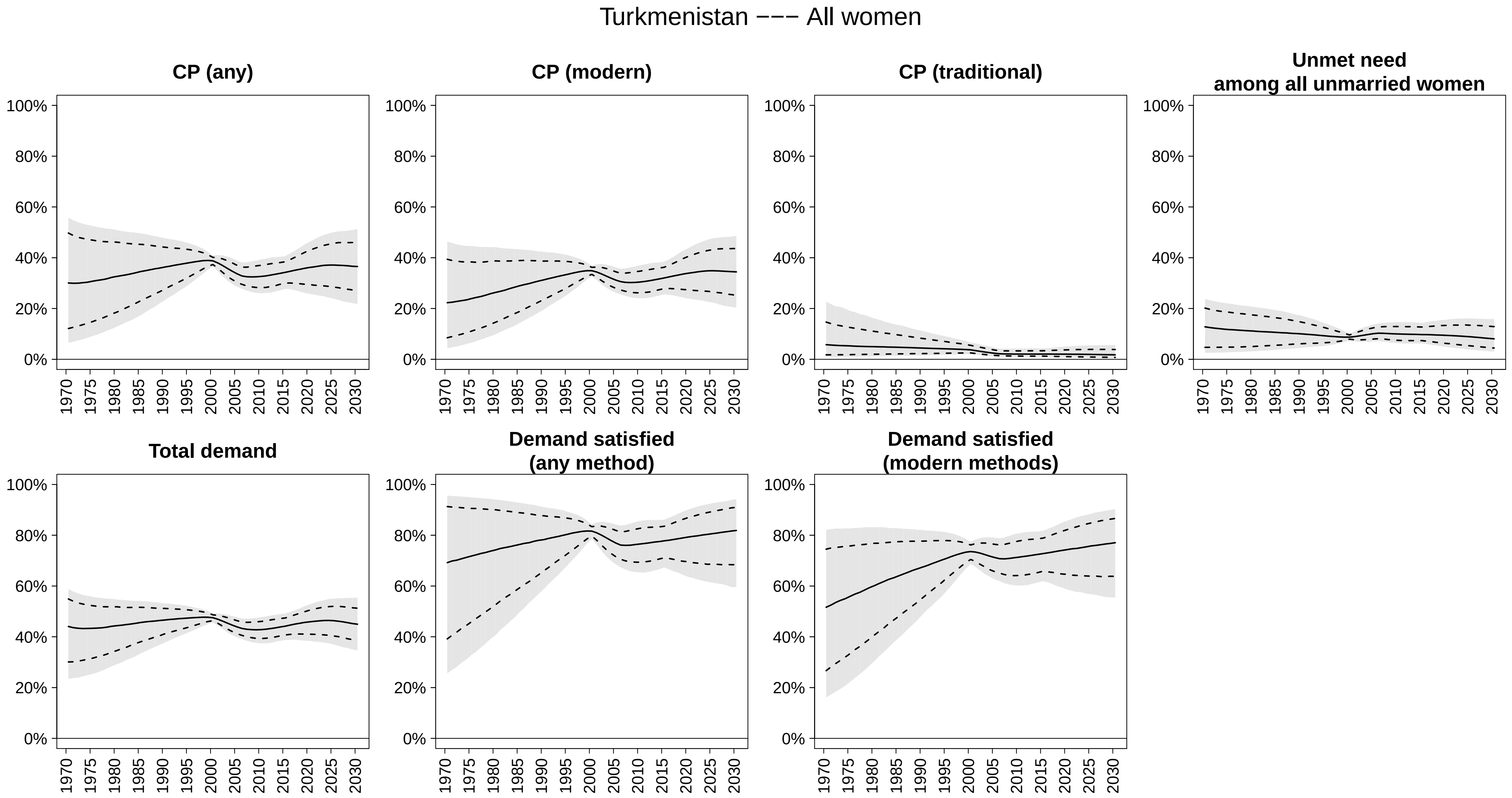
Turkmenistan −−− All women
CP (any)
CP (modern)
CP (traditional)
Unmet need
among all unmarried women
Total demand
Demand satisfied
(any method)
Demand satisfied
(modern methods)
0%
20%
40%
60%
80%
100%
1970
1975
1980
1985
1990
1995
2000
2005
2010
2015
2020
2025
2030

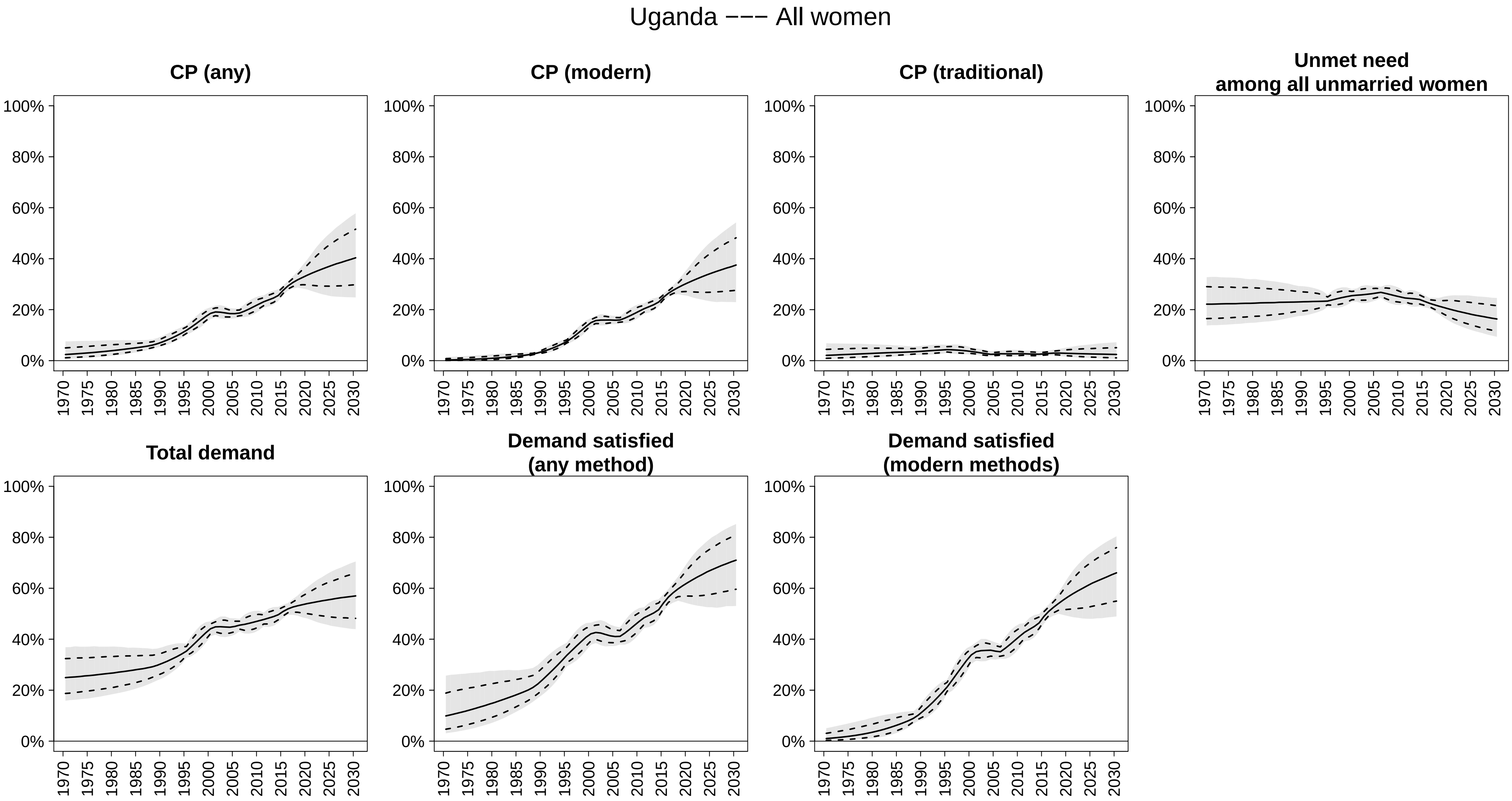
Uganda −−− All women
CP (any)
CP (modern)
CP (traditional)
Unmet need
among all unmarried women
Total demand
Demand satisfied
(any method)
Demand satisfied
(modern methods)
0%
20%
40%
60%
80%
100%
1970
1975
1980
1985
1990
1995
2000
2005
2010
2015
2020
2025
2030

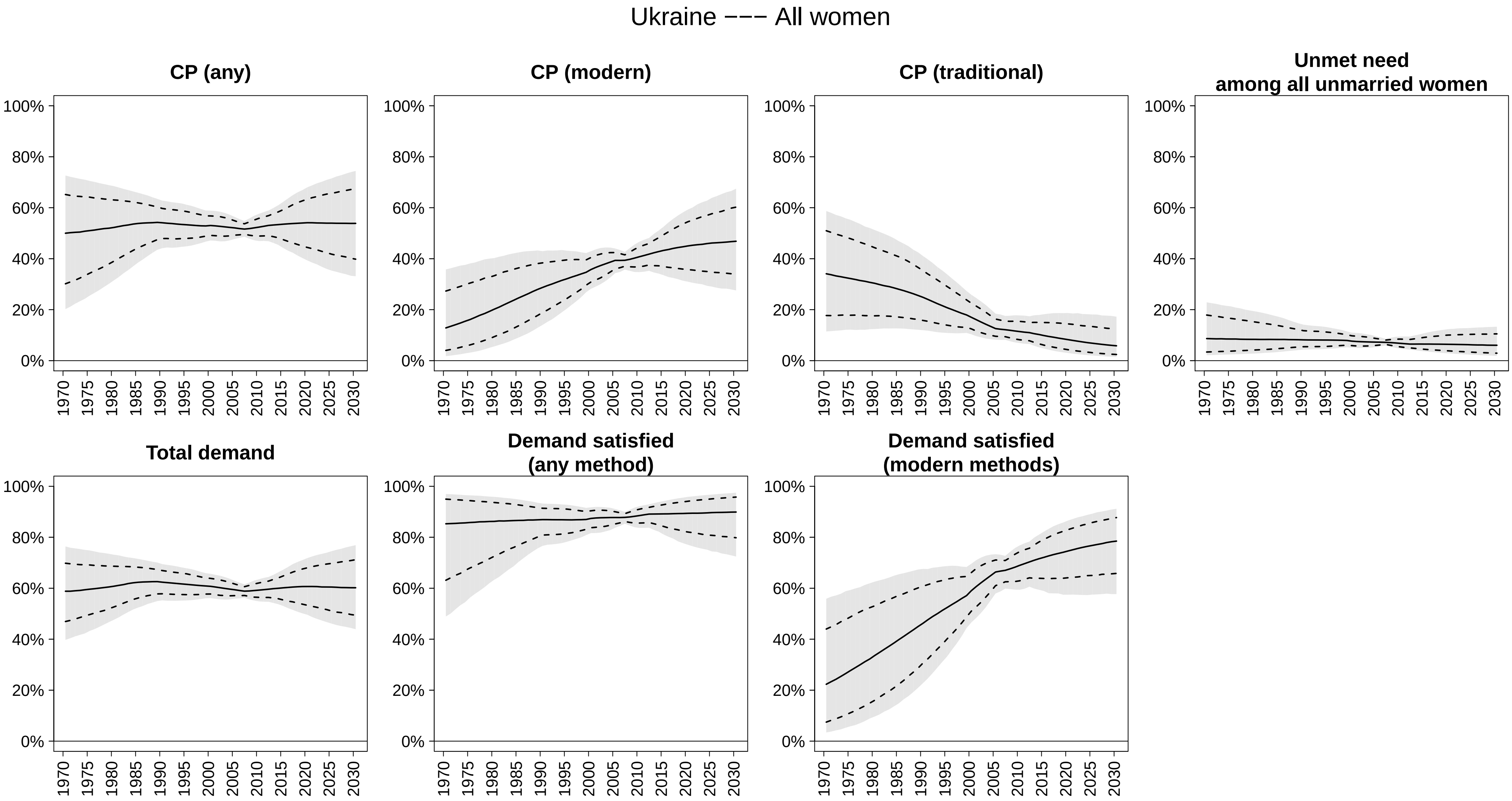
Ukraine −−− All women
CP (any)
CP (modern)
CP (traditional)
Unmet need among all unmarried women
Total demand
Demand satisfied (any method)
Demand satisfied (modern methods)
0%
20%
40%
60%
80%
100%
1970
1975
1980
1985
1990
1995
2000
2005
2010
2015
2020
2025
2030

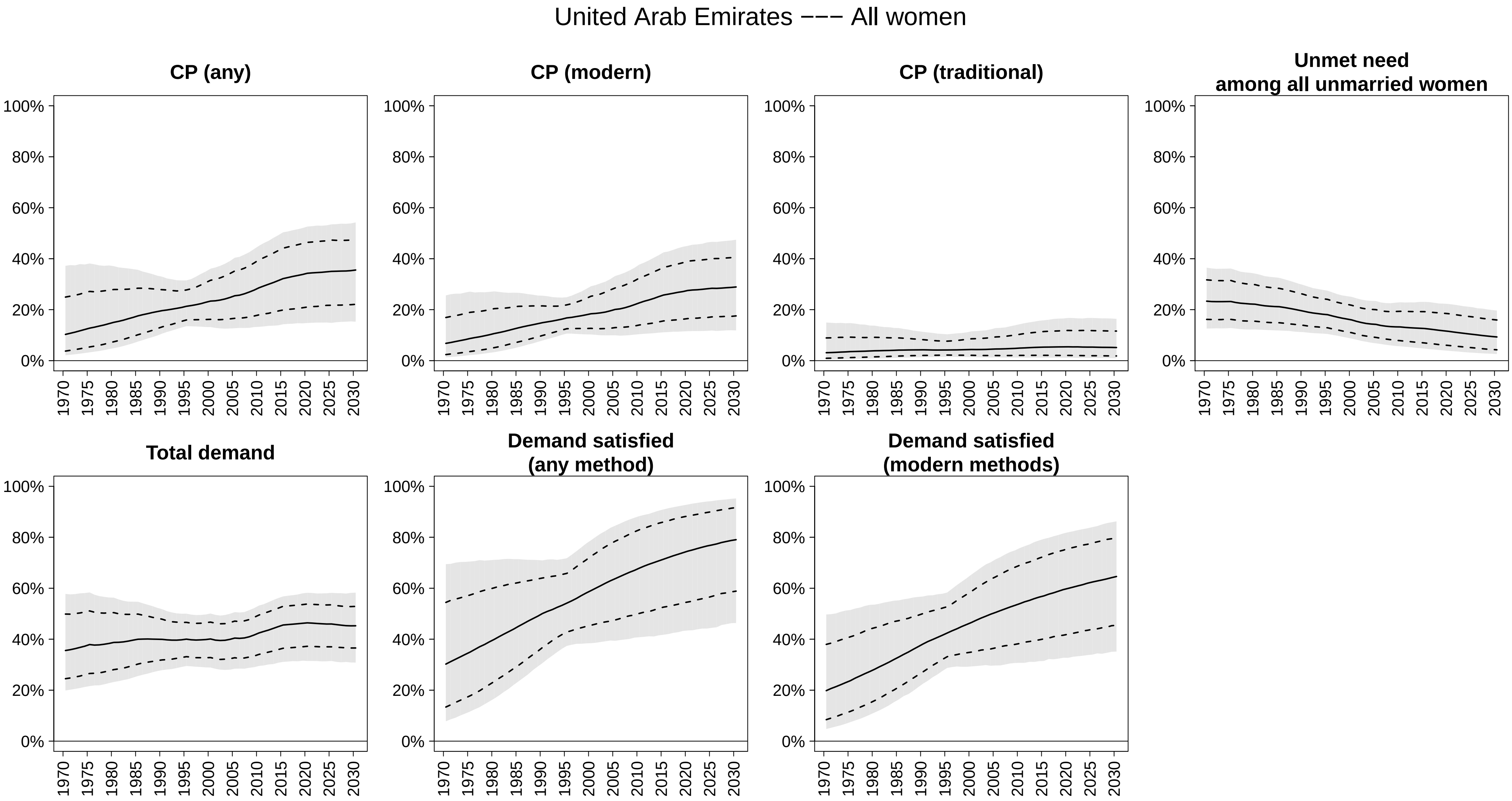
United Arab Emirates −−− All women
CP (any)
CP (modern)
CP (traditional)
Unmet need
among all unmarried women
Total demand
Demand satisfied
(any method)
Demand satisfied
(modern methods)
0%
20%
40%
60%
80%
100%
1970
1975
1980
1985
1990
1995
2000
2005
2010
2015
2020
2025
2030

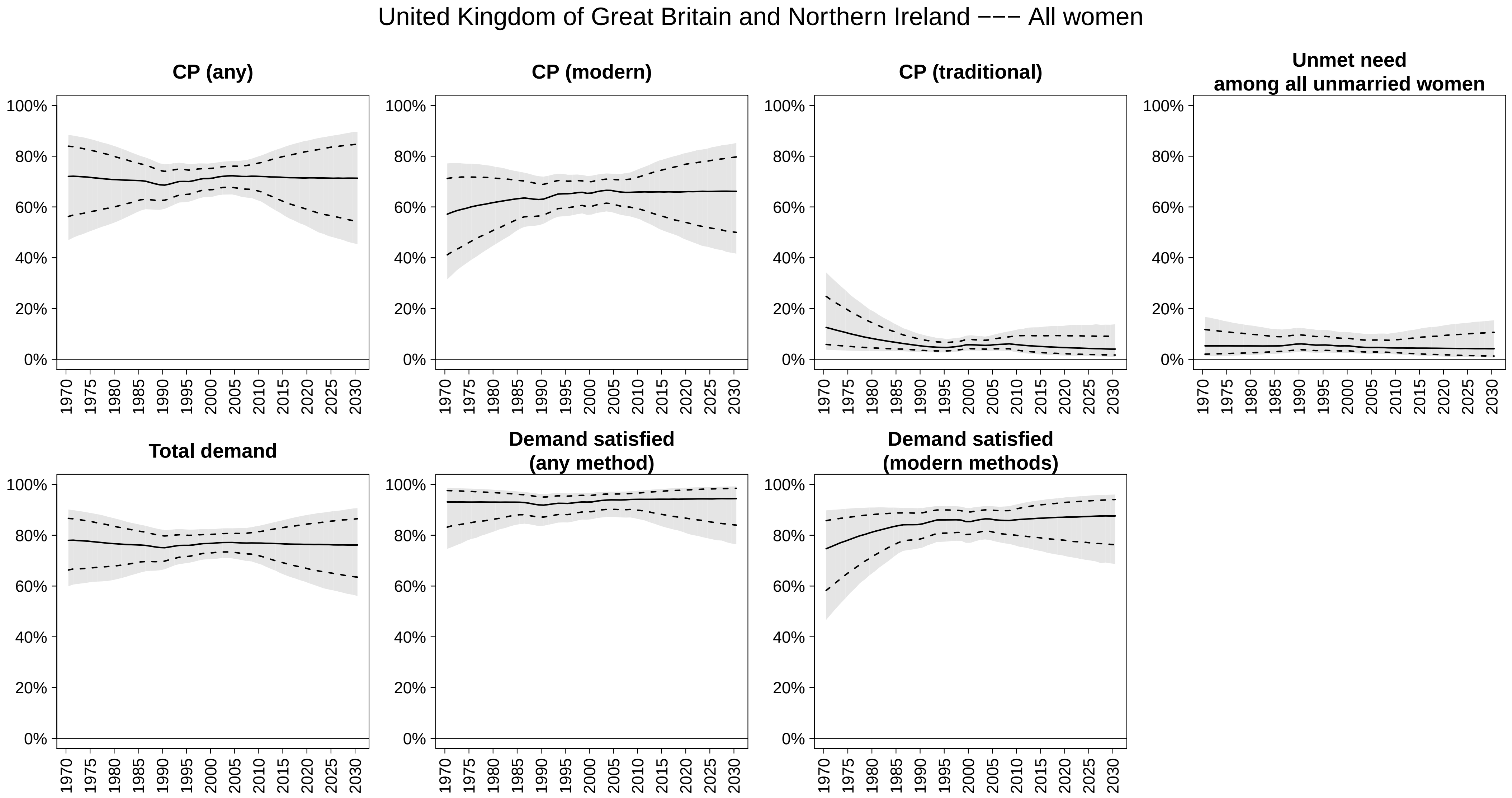
United Kingdom of Great Britain and Northern Ireland −−− All women
CP (any)
CP (modern)
CP (traditional)
Unmet need among all unmarried women
Total demand
Demand satisfied (any method)
Demand satisfied (modern methods)
0%
20%
40%
60%
80%
100%
1970
1975
1980
1985
1990
1995
2000
2005
2010
2015
2020
2025
2030

United Republic of Tanzania −−− All women

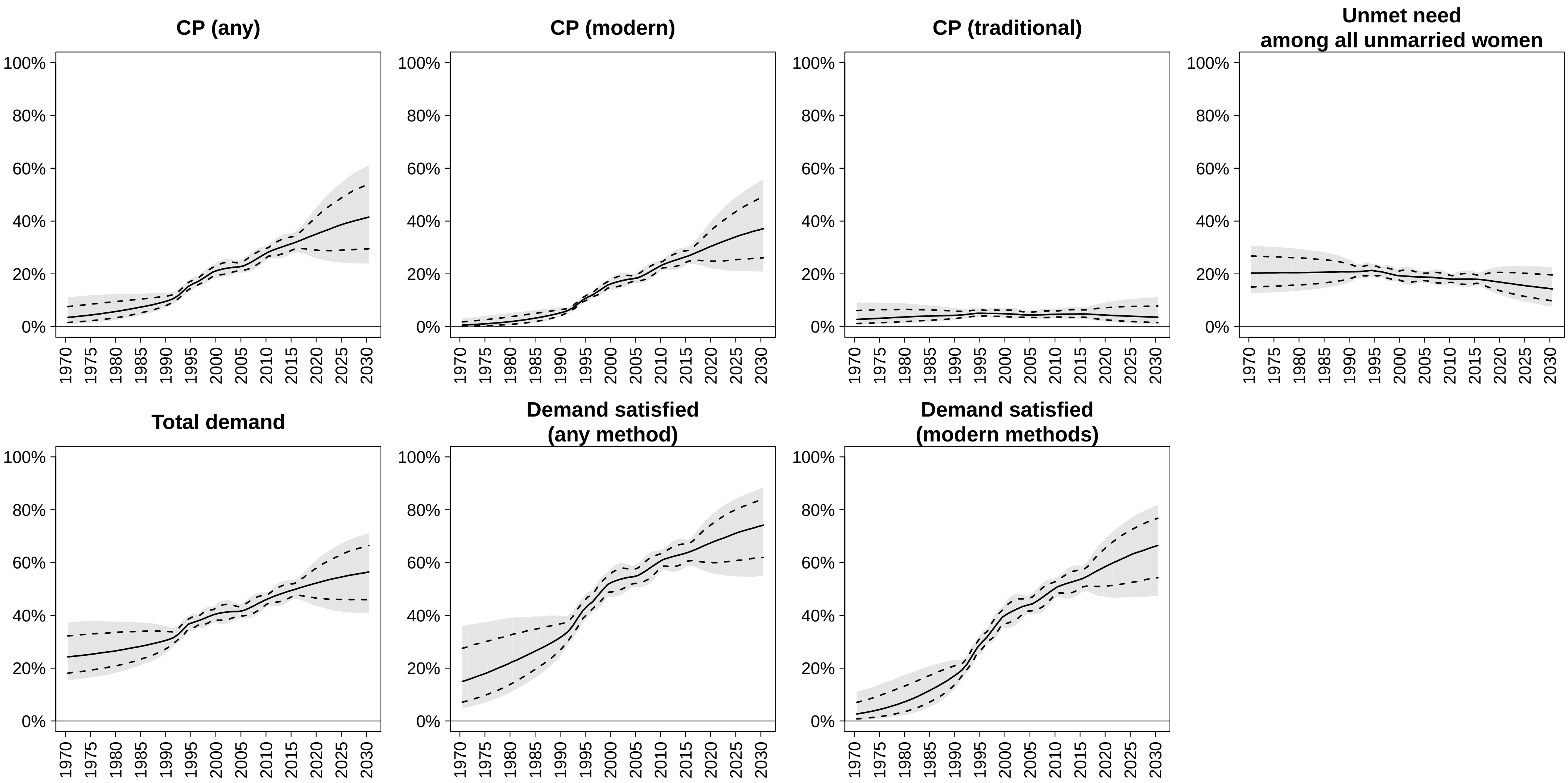

United States of America −−− All women

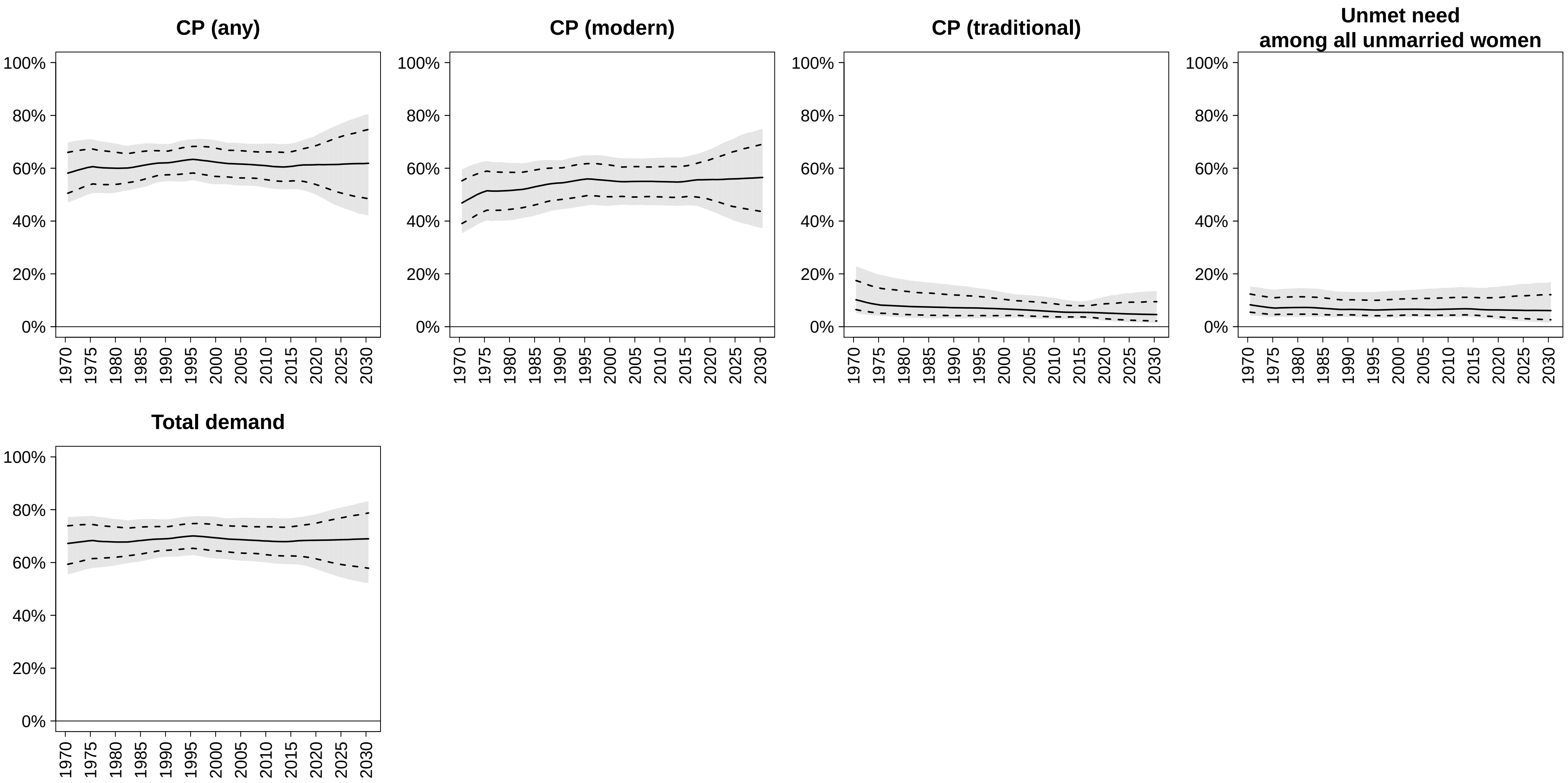

United States Virgin Islands −−− All women

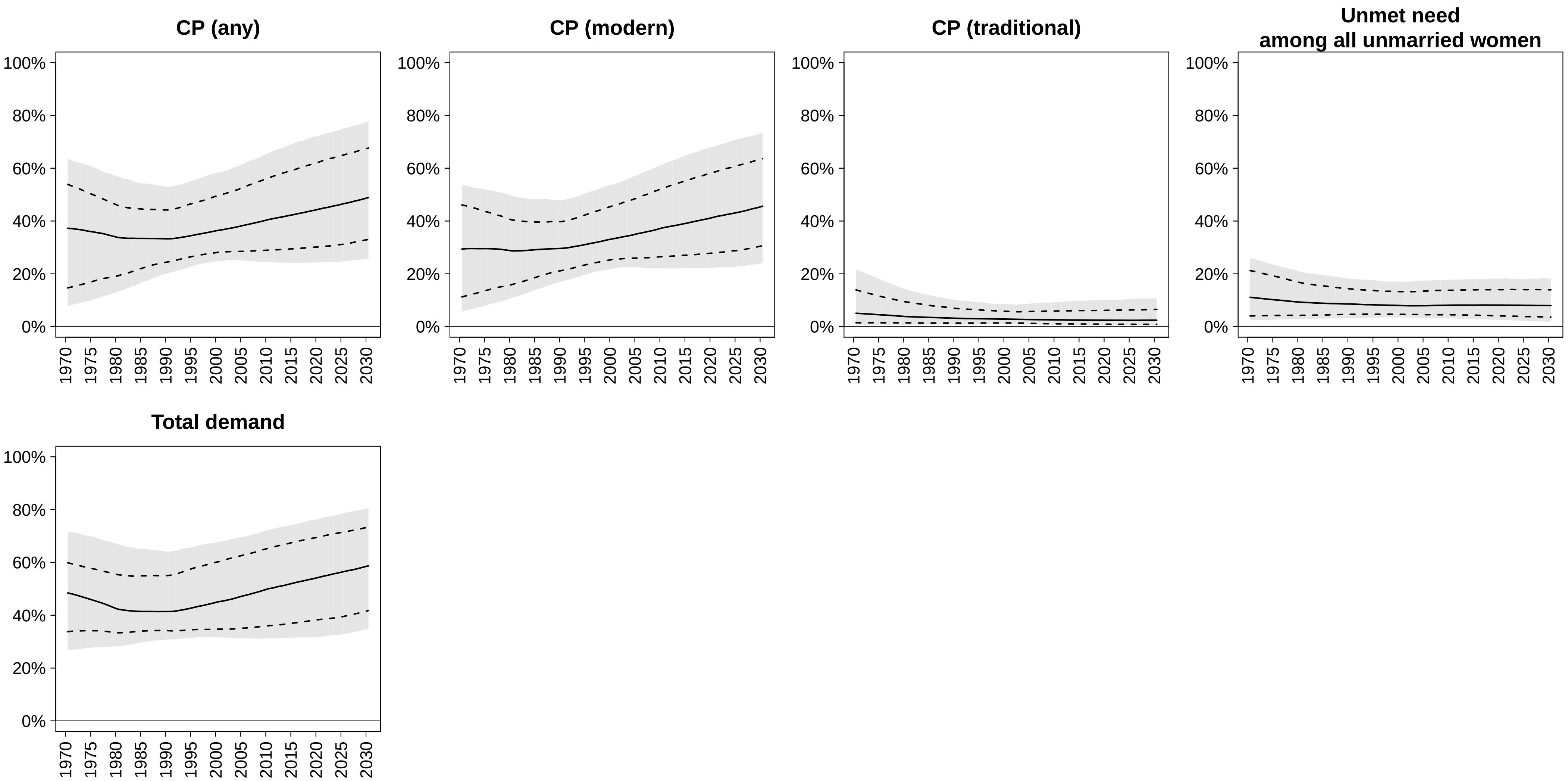

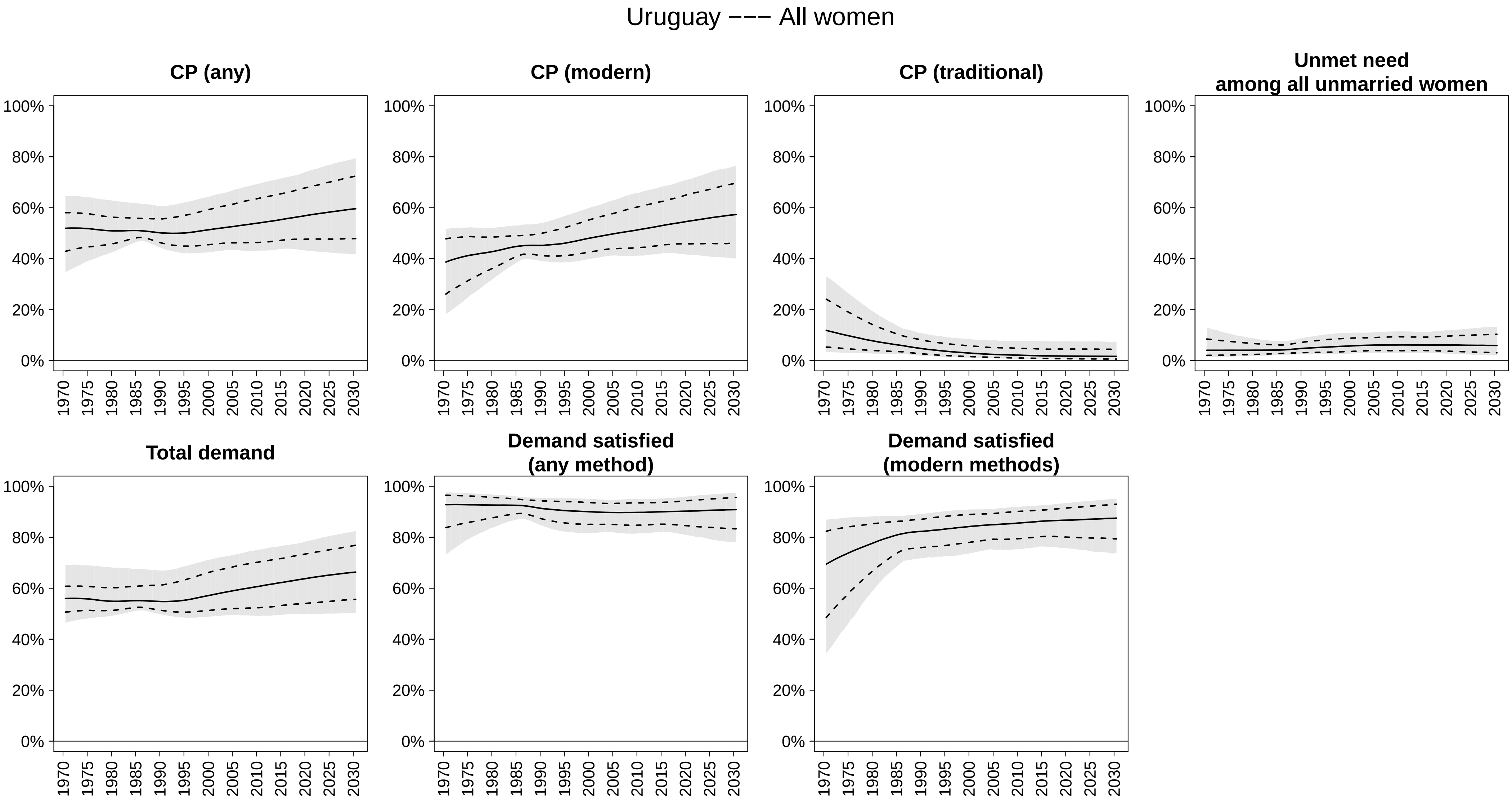
Uruguay −−− All women
CP (any)
CP (modern)
CP (traditional)
Unmet need among all unmarried women
Total demand
Demand satisfied (any method)
Demand satisfied (modern methods)
0%
20%
40%
60%
80%
100%
1970
1975
1980
1985
1990
1995
2000
2005
2010
2015
2020
2025
2030

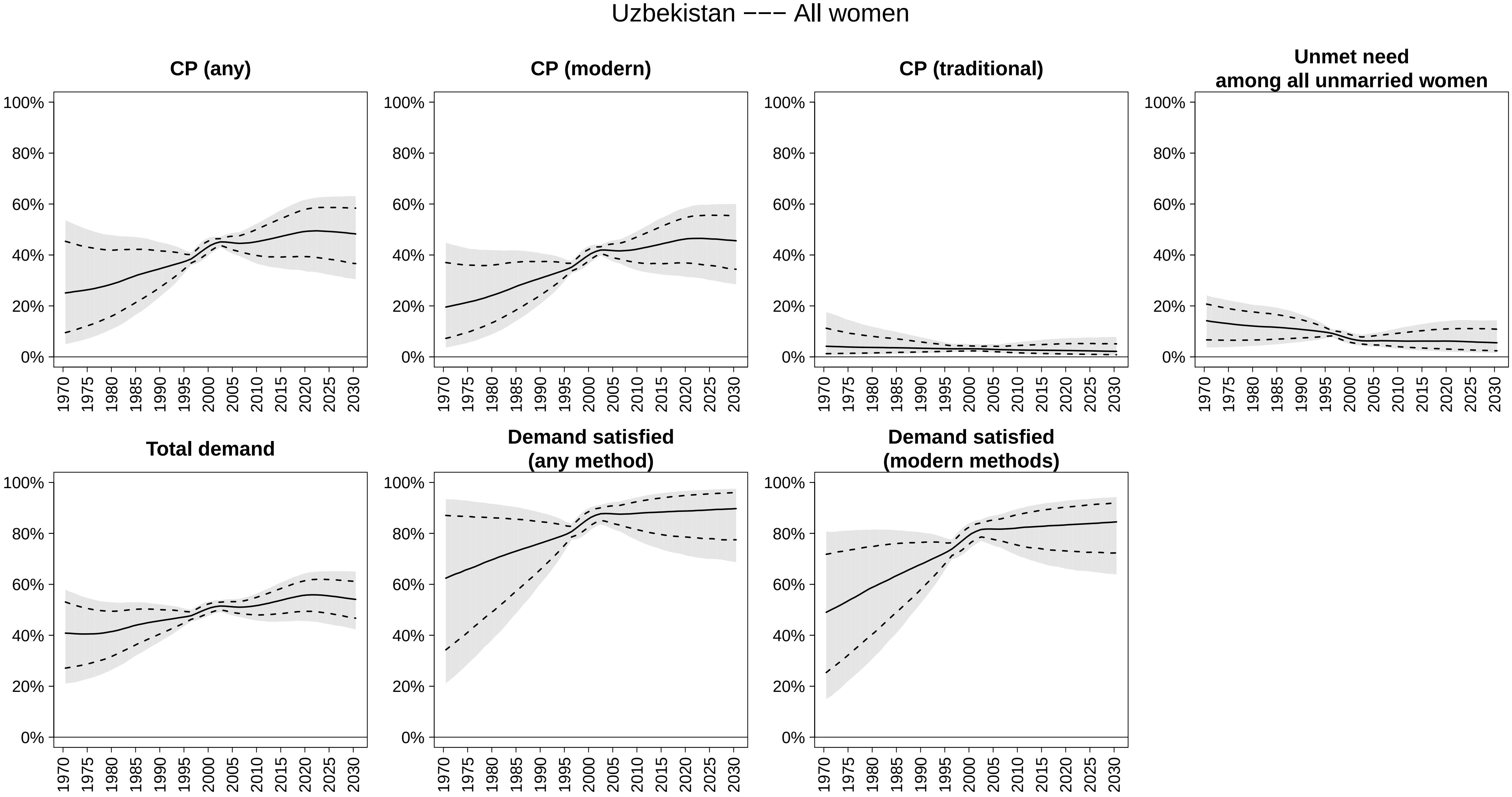
Uzbekistan −−− All women
CP (any)
CP (modern)
CP (traditional)
Unmet need
among all unmarried women
Total demand
Demand satisfied
(any method)
Demand satisfied
(modern methods)
0%
20%
40%
60%
80%
100%
1970
1975
1980
1985
1990
1995
2000
2005
2010
2015
2020
2025
2030

Vanuatu −−− All women

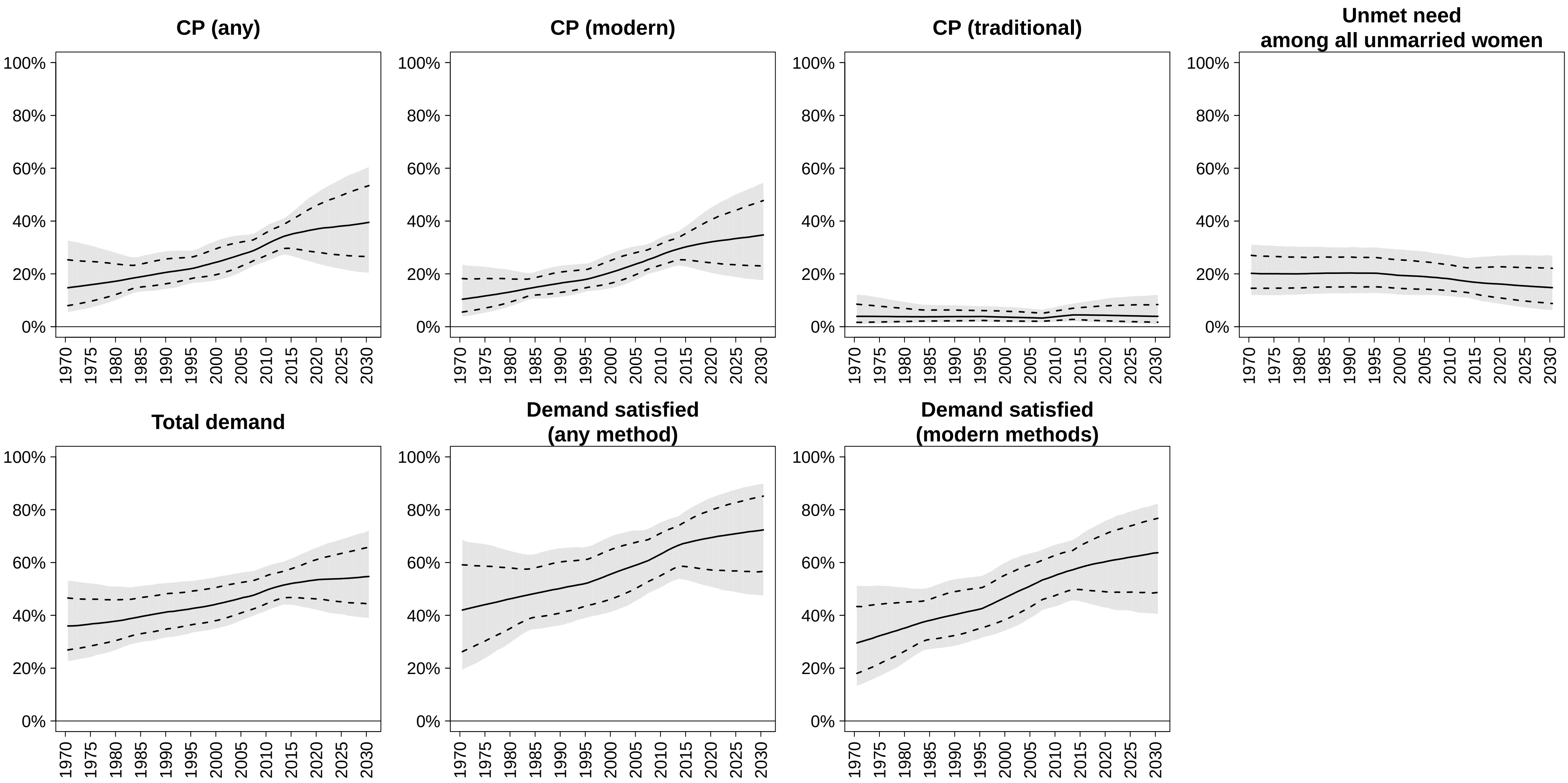

Venezuela (Bolivarian Republic of) −−− All women

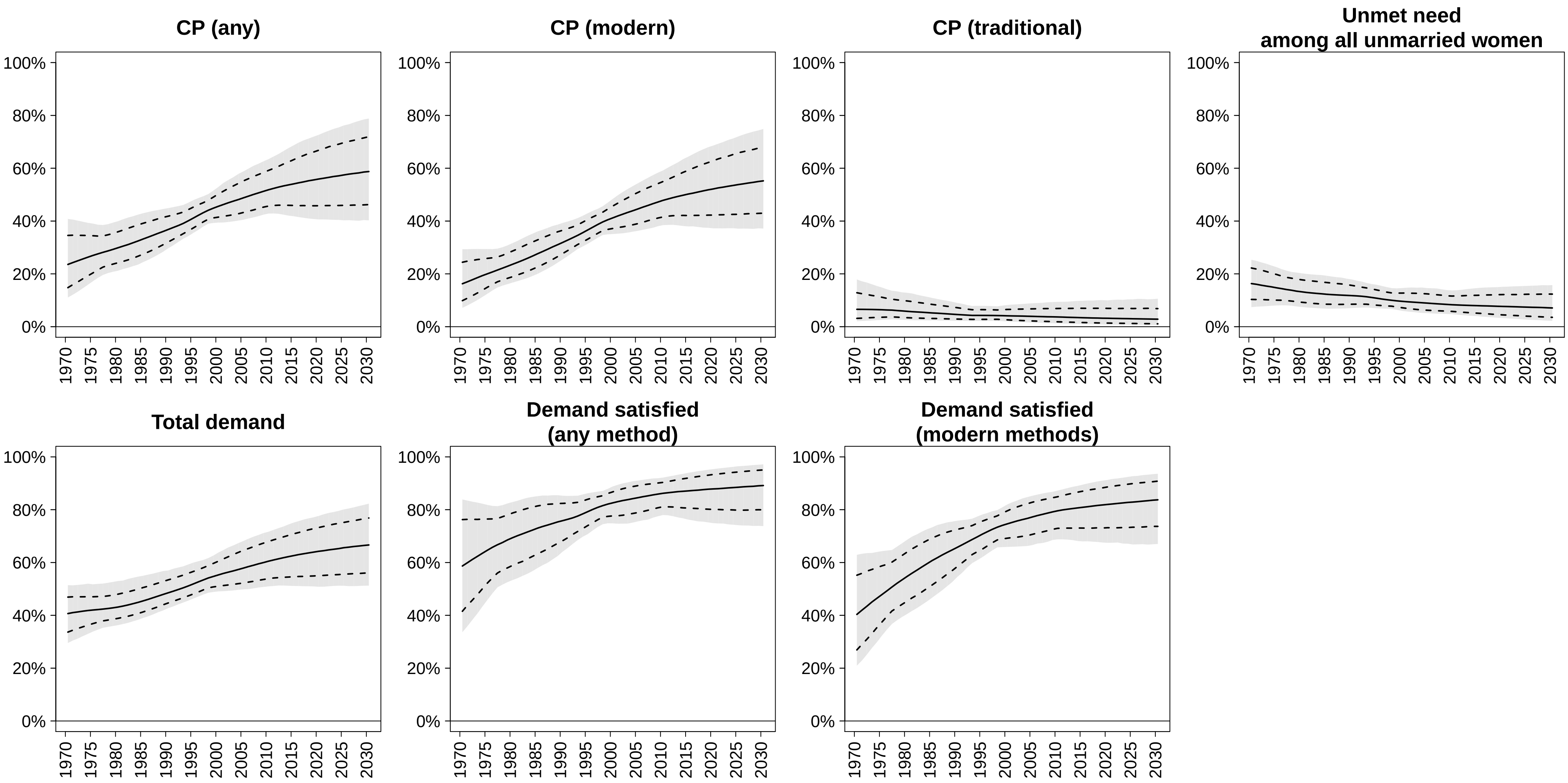

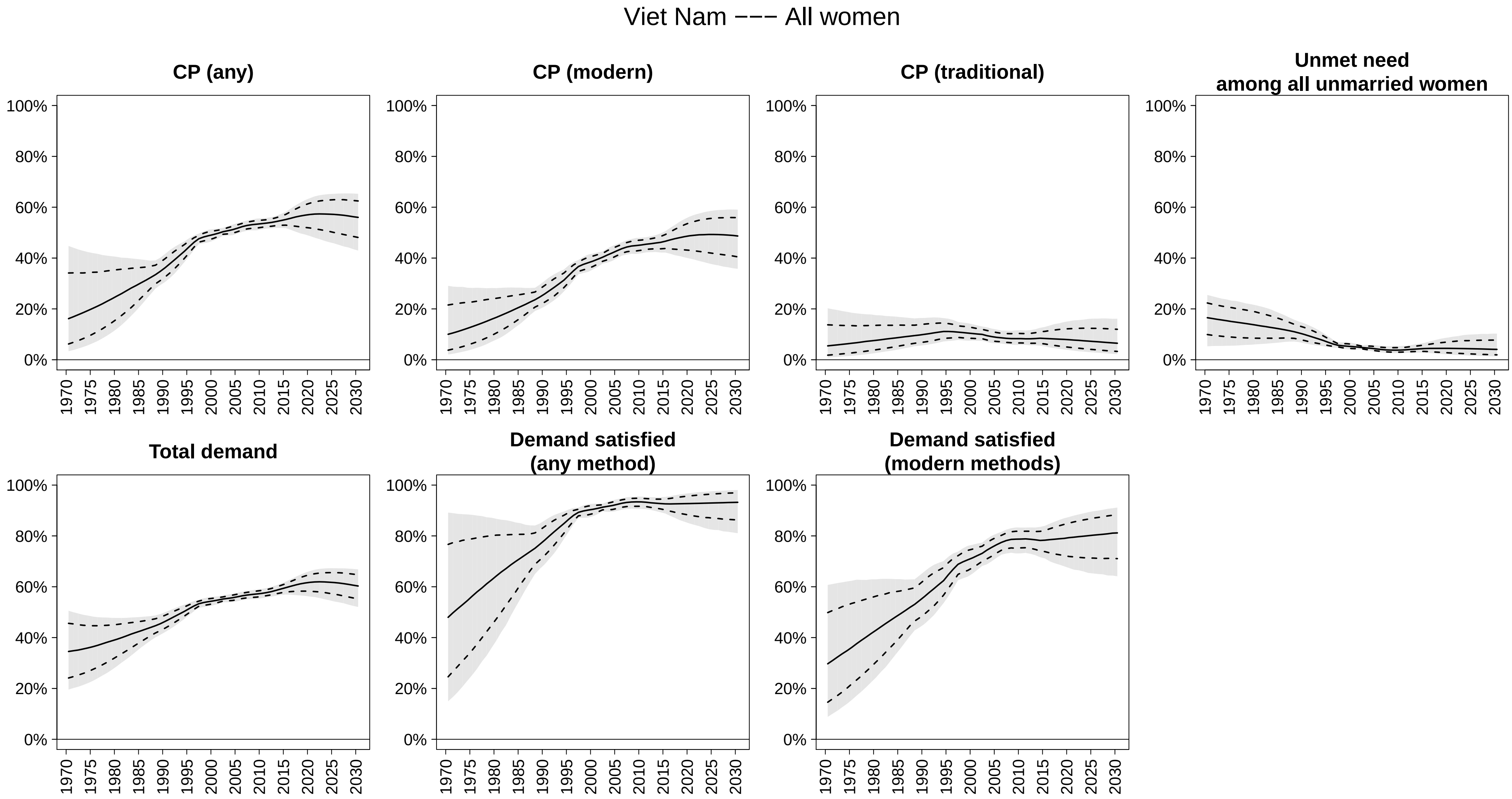
Viet Nam −−− All women
CP (any)
CP (modern)
CP (traditional)
Unmet need
among all unmarried women
Total demand
Demand satisfied
(any method)
Demand satisfied
(modern methods)
0%
20%
40%
60%
80%
100%
1970
1975
1980
1985
1990
1995
2000
2005
2010
2015
2020
2025
2030

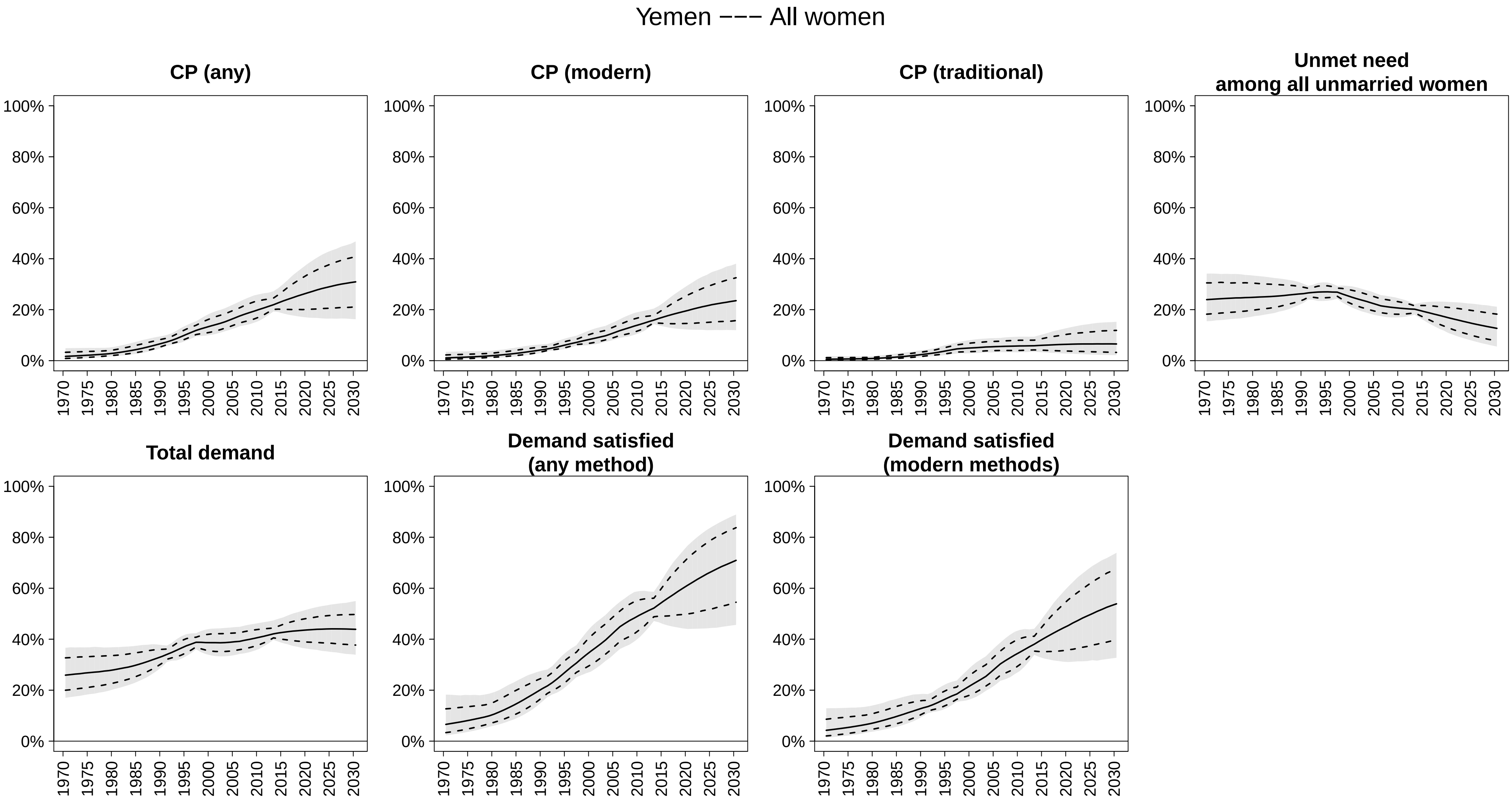
Yemen −−− All women
CP (any)
CP (modern)
CP (traditional)
Unmet need among all unmarried women
Total demand
Demand satisfied (any method)
Demand satisfied (modern methods)
0%
20%
40%
60%
80%
100%
1970
1975
1980
1985
1990
1995
2000
2005
2010
2015
2020
2025
2030

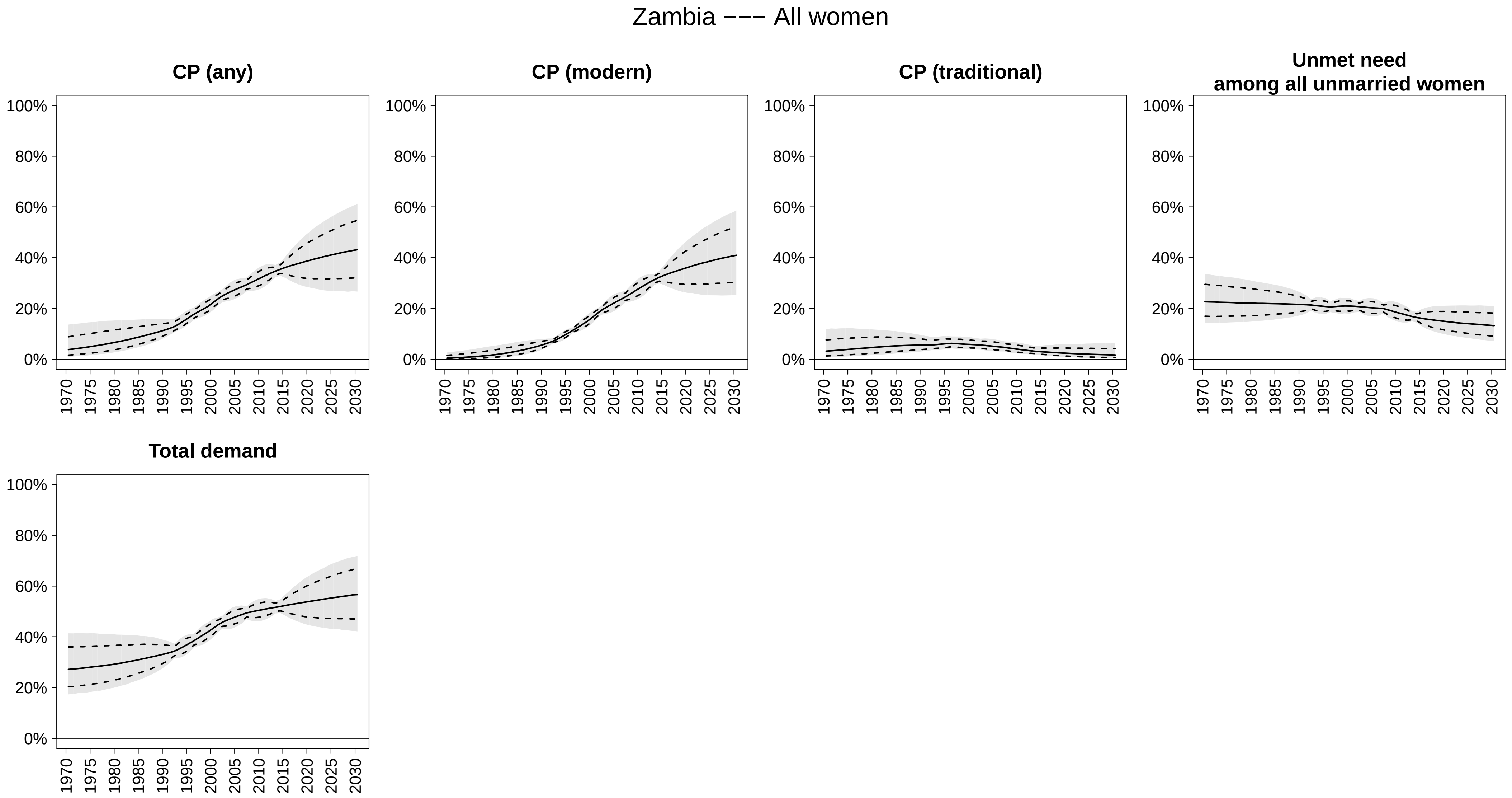
Zambia −−− All women
CP (any)
CP (modern)
CP (traditional)
Unmet need
among all unmarried women
Total demand
0%
20%
40%
60%
80%
100%
1970
1975
1980
1985
1990
1995
2000
2005
2010
2015
2020
2025
2030

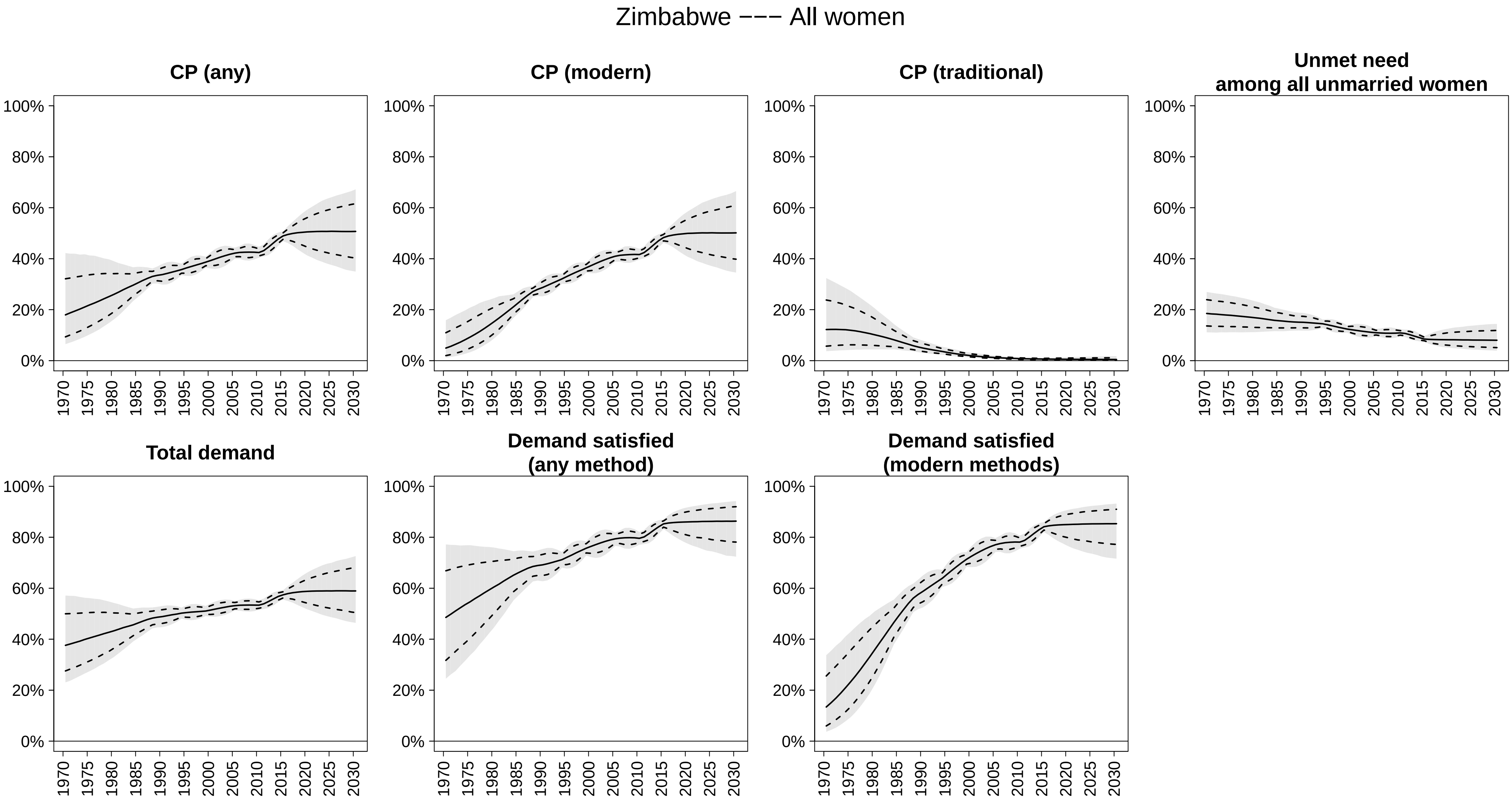
Zimbabwe −−− All women
CP (any)
CP (modern)
CP (traditional)
Unmet need among all unmarried women
Total demand
Demand satisfied (any method)
Demand satisfied (modern methods)
0% 20% 40% 60% 80% 100%
1970 1975 1980 1985 1990 1995 2000 2005 2010 2015 2020 2025 2030

3.2 Country aggregates

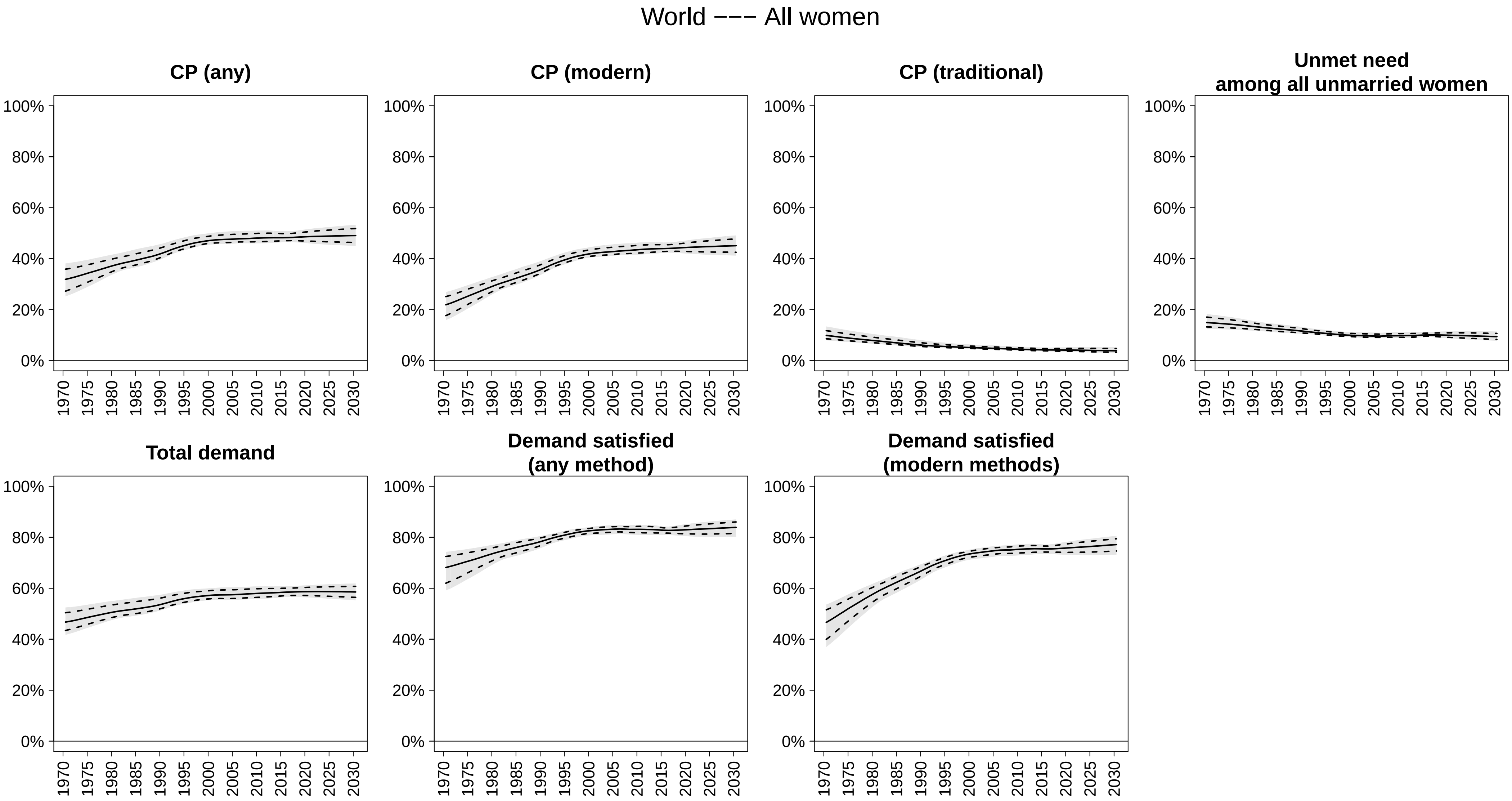
World −−− All women
CP (any)
CP (modern)
CP (traditional)
Unmet need
among all unmarried women
Total demand
Demand satisfied
(any method)
Demand satisfied
(modern methods)
0%
20%
40%
60%
80%
100%
1970
1975
1980
1985
1990
1995
2000
2005
2010
2015
2020
2025
2030

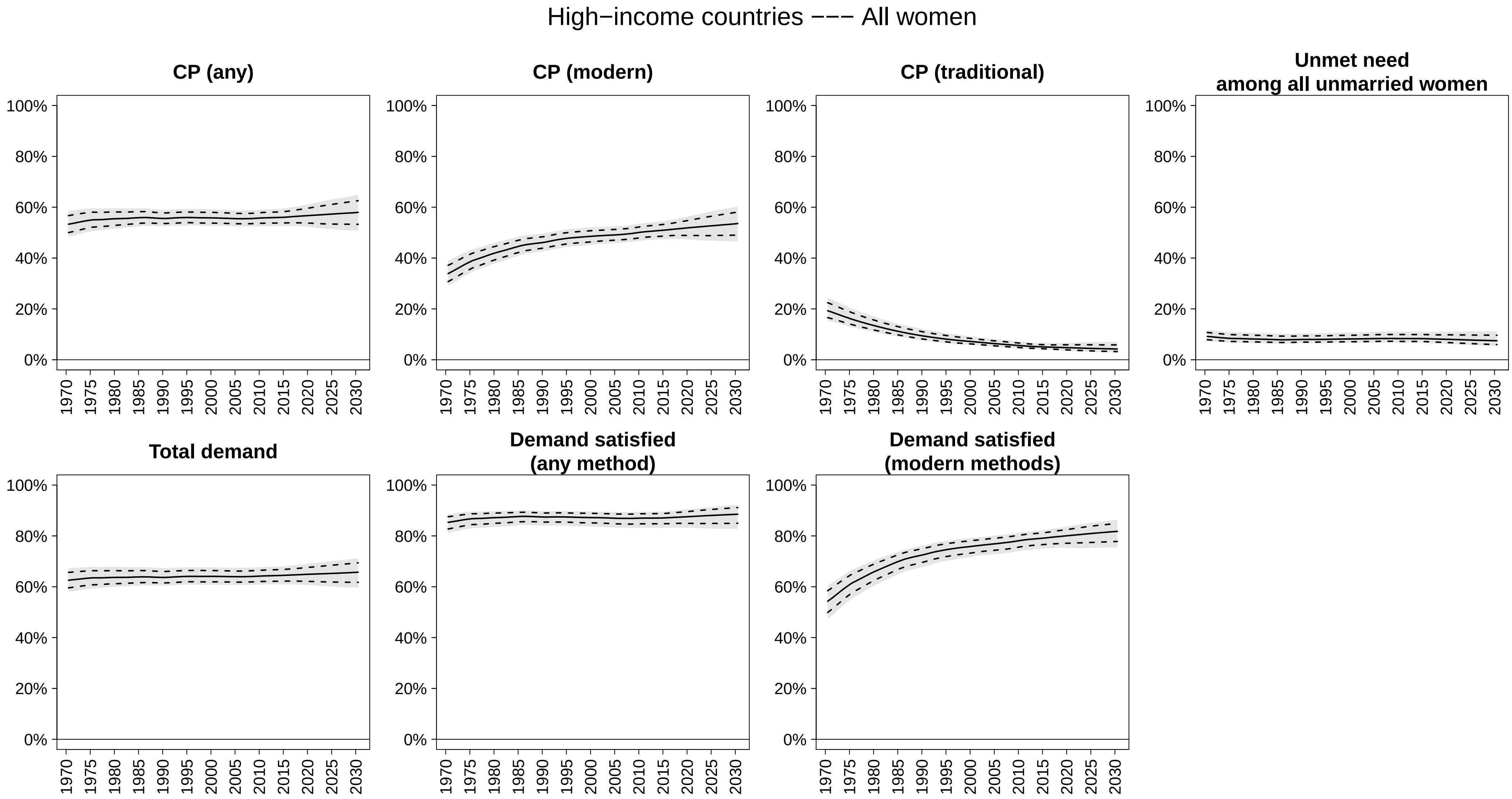
High−income countries −−− All women
CP (any)
CP (modern)
CP (traditional)
Unmet need
among all unmarried women
Total demand
Demand satisfied
(any method)
Demand satisfied
(modern methods)
0%
20%
40%
60%
80%
100%
1970
1975
1980
1985
1990
1995
2000
2005
2010
2015
2020
2025
2030

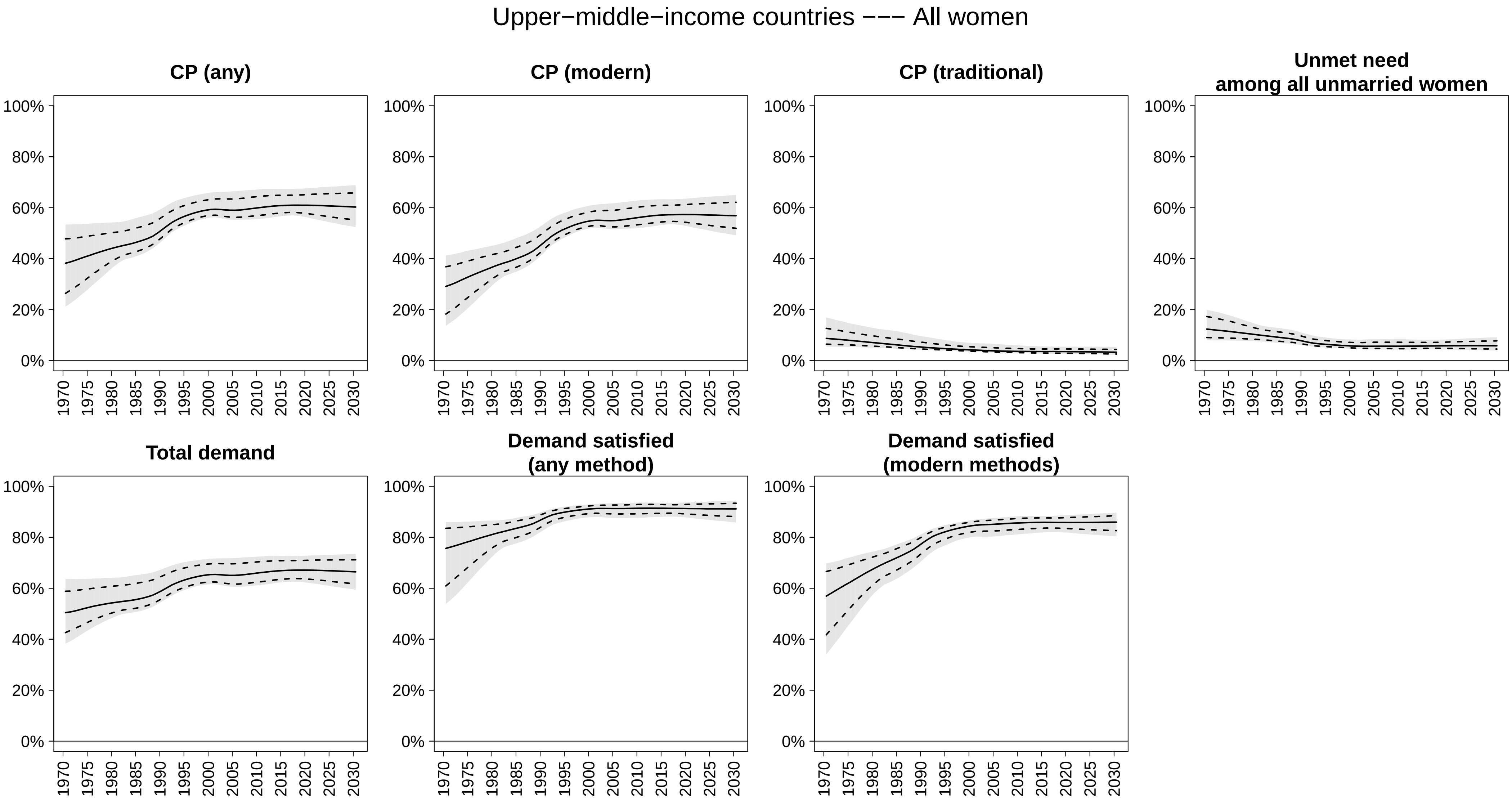

Upper−middle−income countries −−− All women
CP (any)
CP (modern)
CP (traditional)
Unmet need among all unmarried women
Total demand
Demand satisfied (any method)
Demand satisfied (modern methods)
0%
20%
40%
60%
80%
100%
1970
1975
1980
1985
1990
1995
2000
2005
2010
2015
2020
2025
2030

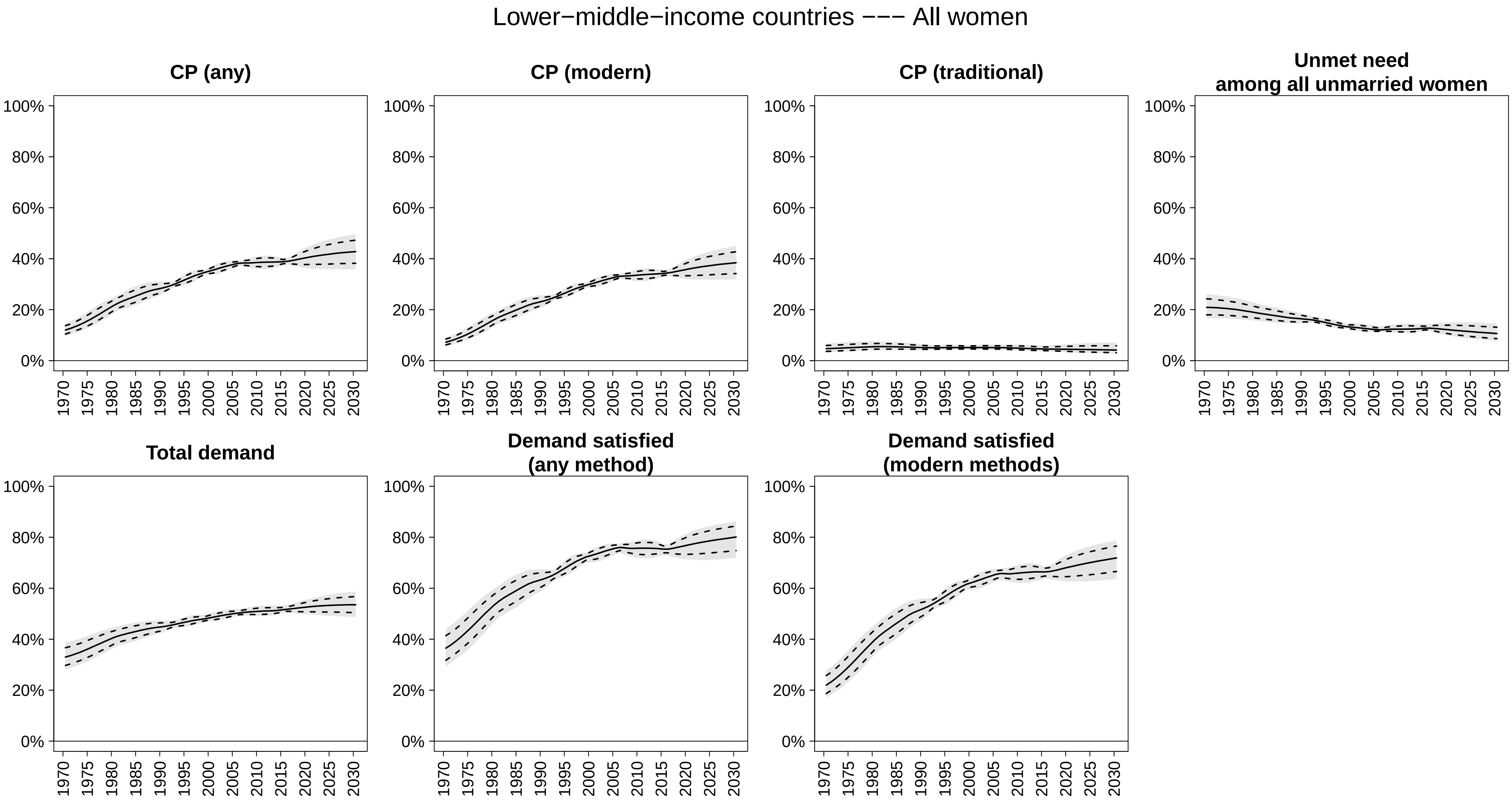
Lower−middle−income countries −−− All women
CP (any)
CP (modern)
CP (traditional)
Unmet need
among all unmarried women
Total demand
Demand satisfied
(any method)
Demand satisfied
(modern methods)
0%
20%
40%
60%
80%
100%
1970
1975
1980
1985
1990
1995
2000
2005
2010
2015
2020
2025
2030

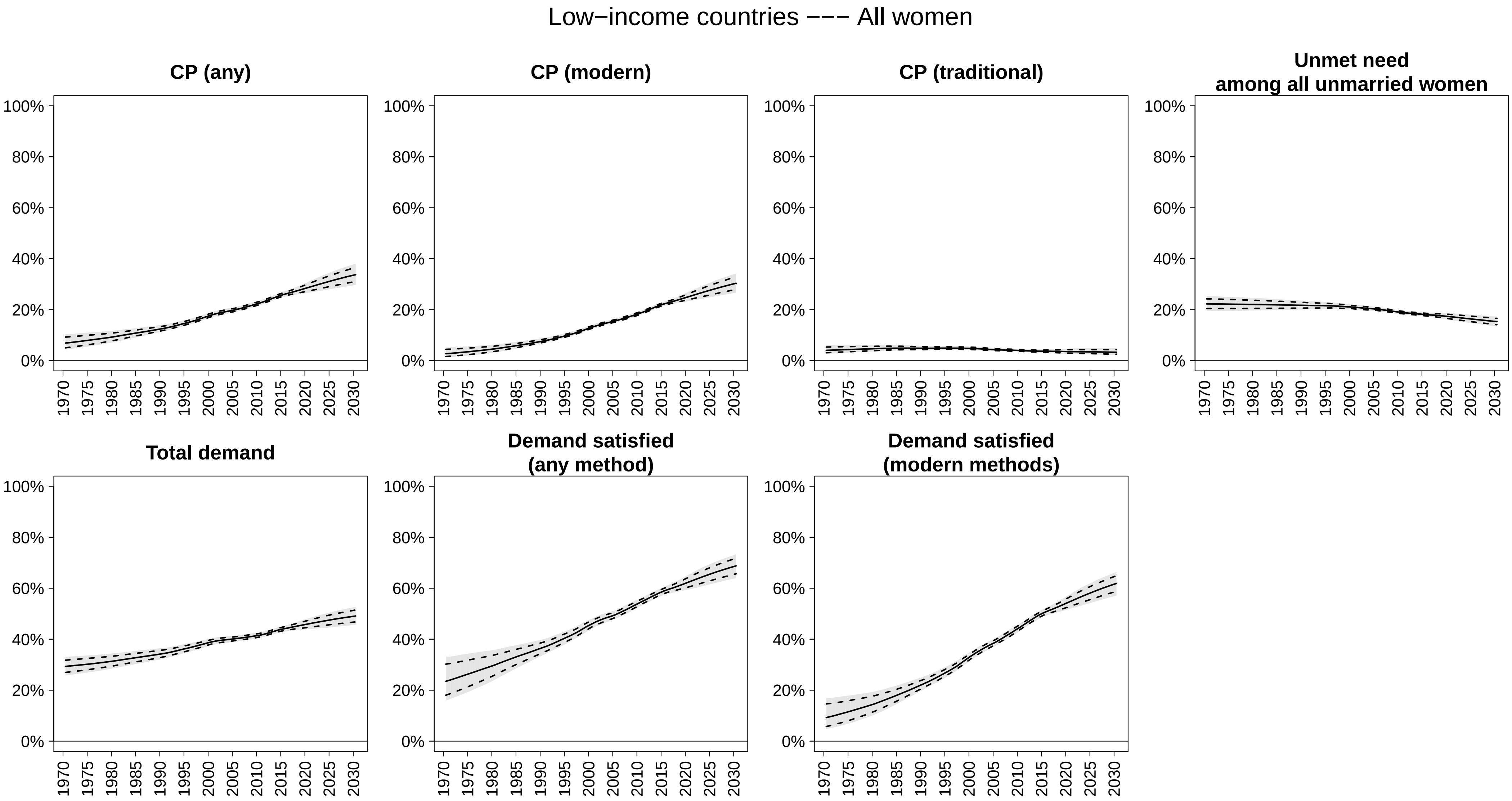
Low−income countries −−− All women
CP (any)
CP (modern)
CP (traditional)
Unmet need
among all unmarried women
Total demand
Demand satisfied
(any method)
Demand satisfied
(modern methods)
0%
20%
40%
60%
80%
100%
1970
1975
1980
1985
1990
1995
2000
2005
2010
2015
2020
2025
2030

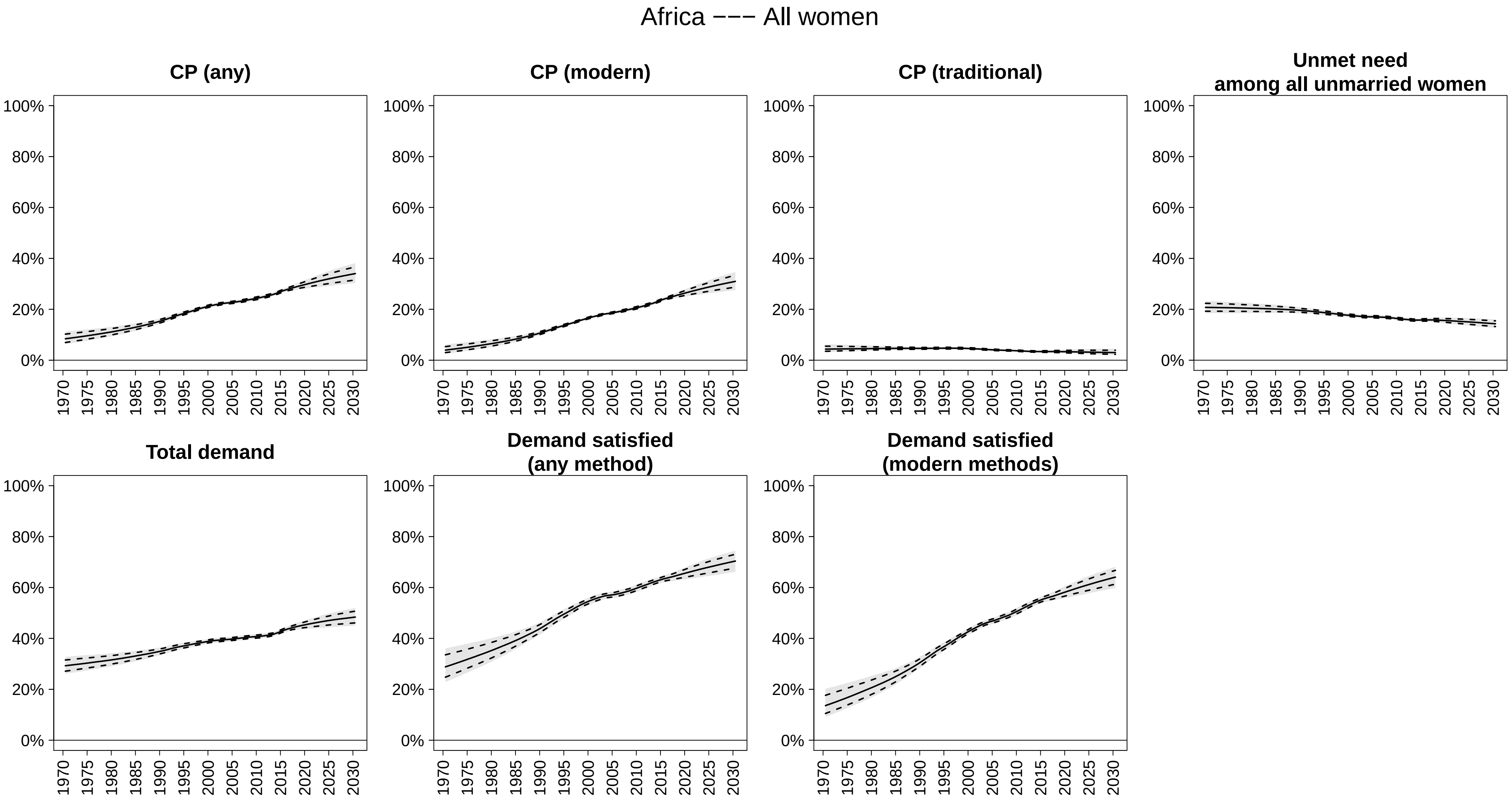
Africa −−− All women
CP (any)
CP (modern)
CP (traditional)
Unmet need
among all unmarried women
Total demand
Demand satisfied
(any method)
Demand satisfied
(modern methods)
0%
20%
40%
60%
80%
100%
1970
1975
1980
1985
1990
1995
2000
2005
2010
2015
2020
2025
2030

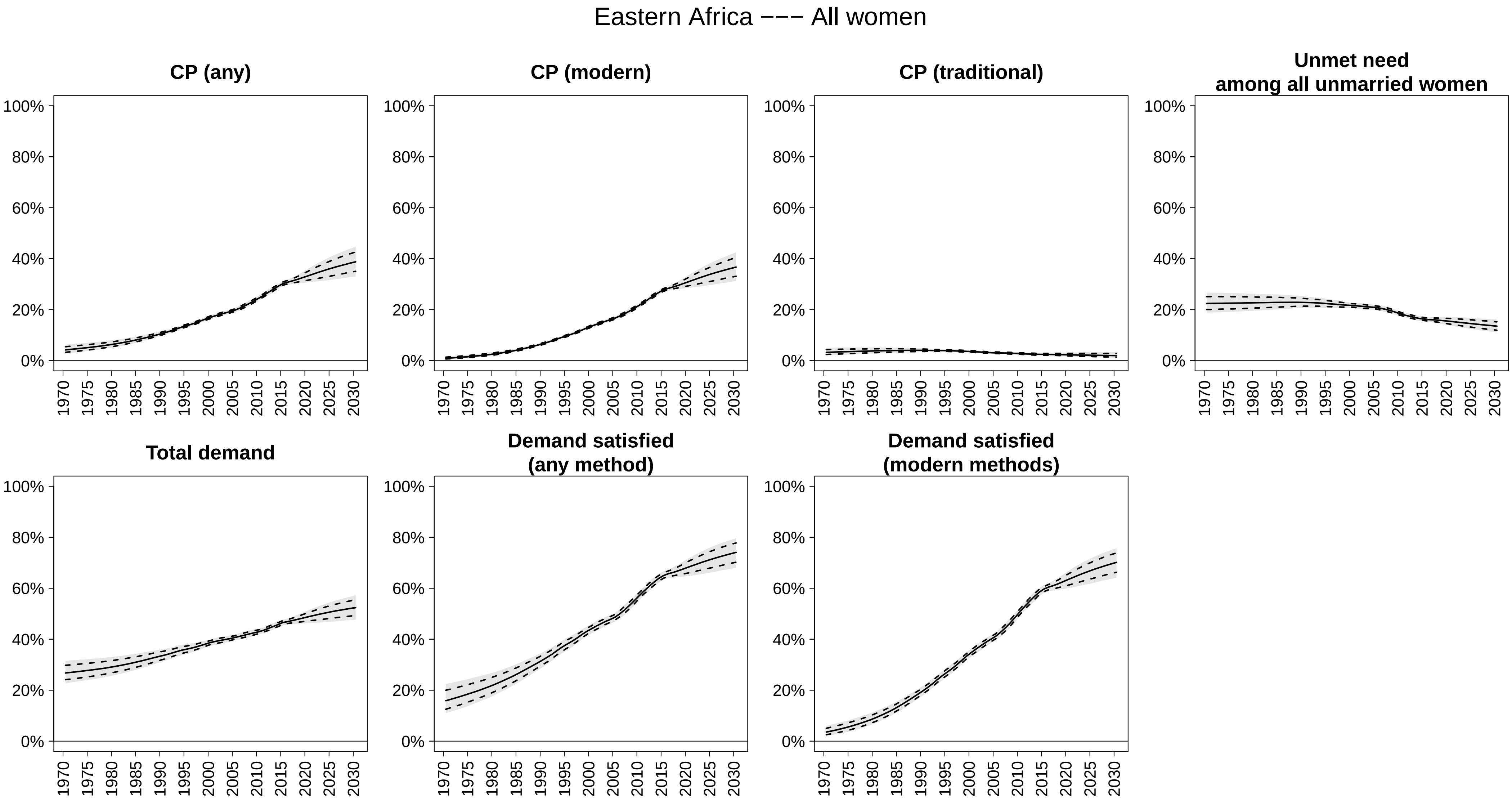
Eastern Africa --- All women
CP (any)
CP (modern)
CP (traditional)
Unmet need
among all unmarried women
Total demand
Demand satisfied
(any method)
Demand satisfied
(modern methods)
100%
80%
60%
40%
20%
0%
1970
1975
1980
1985
1990
1995
2000
2005
2010
2015
2020
2025
2030

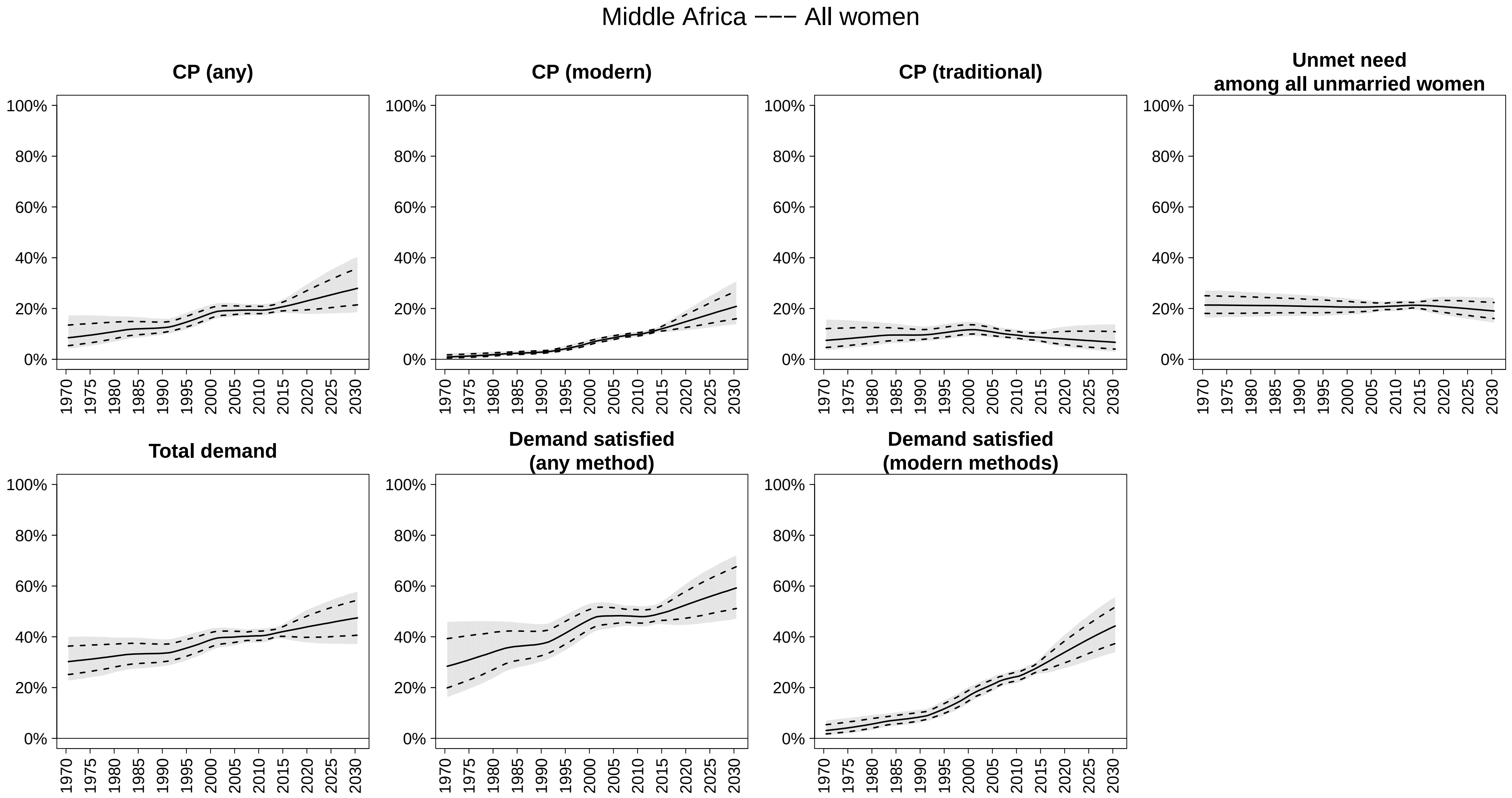
Middle Africa −−− All women
CP (any)
CP (modern)
CP (traditional)
Unmet need among all unmarried women
Total demand
Demand satisfied (any method)
Demand satisfied (modern methods)
0%
20%
40%
60%
80%
100%
1970
1975
1980
1985
1990
1995
2000
2005
2010
2015
2020
2025
2030

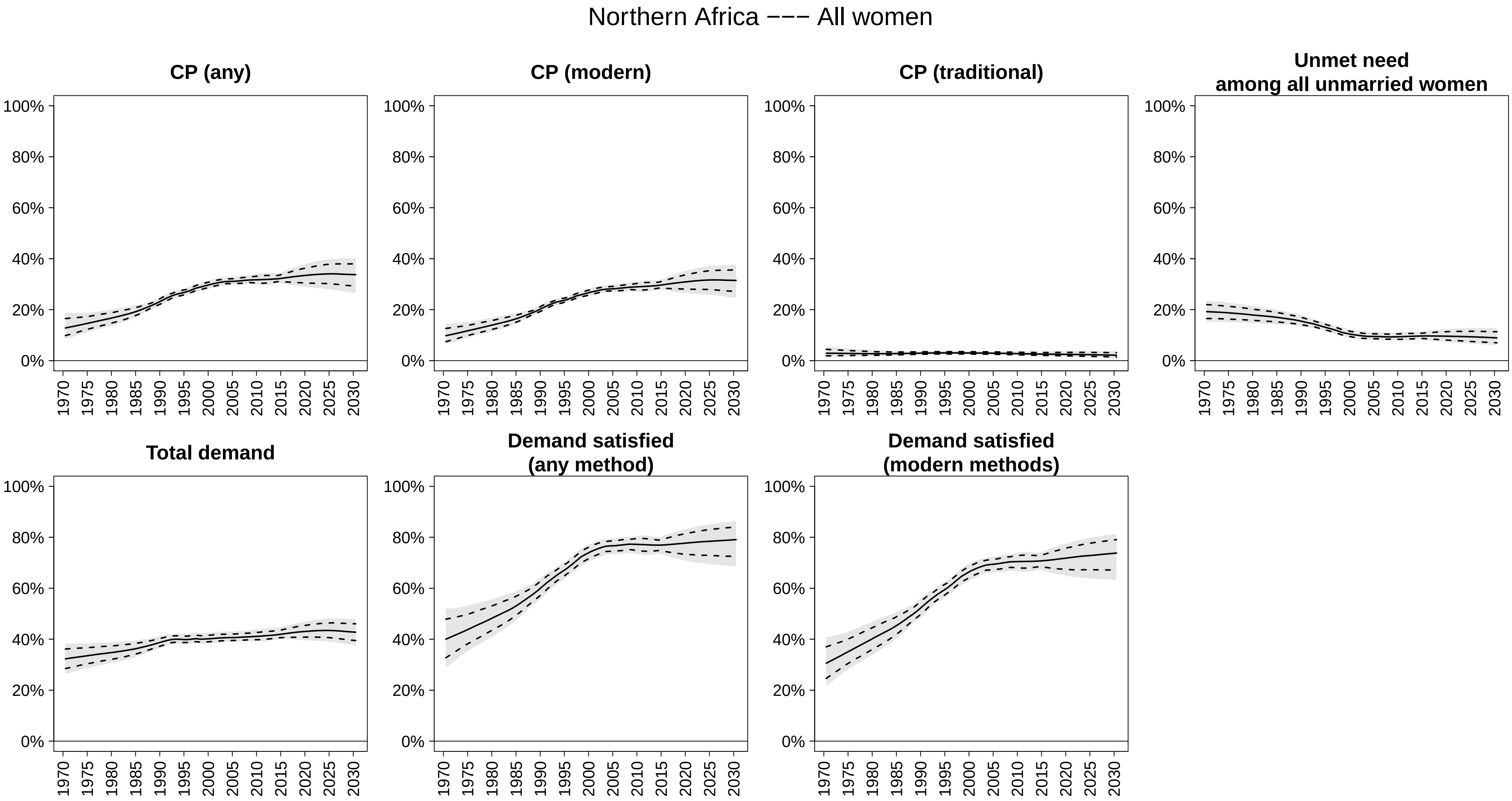
Northern Africa −−− All women
CP (any)
CP (modern)
CP (traditional)
Unmet need among all unmarried women
Total demand
Demand satisfied (any method)
Demand satisfied (modern methods)
0%
20%
40%
60%
80%
100%
1970
1975
1980
1985
1990
1995
2000
2005
2010
2015
2020
2025
2030

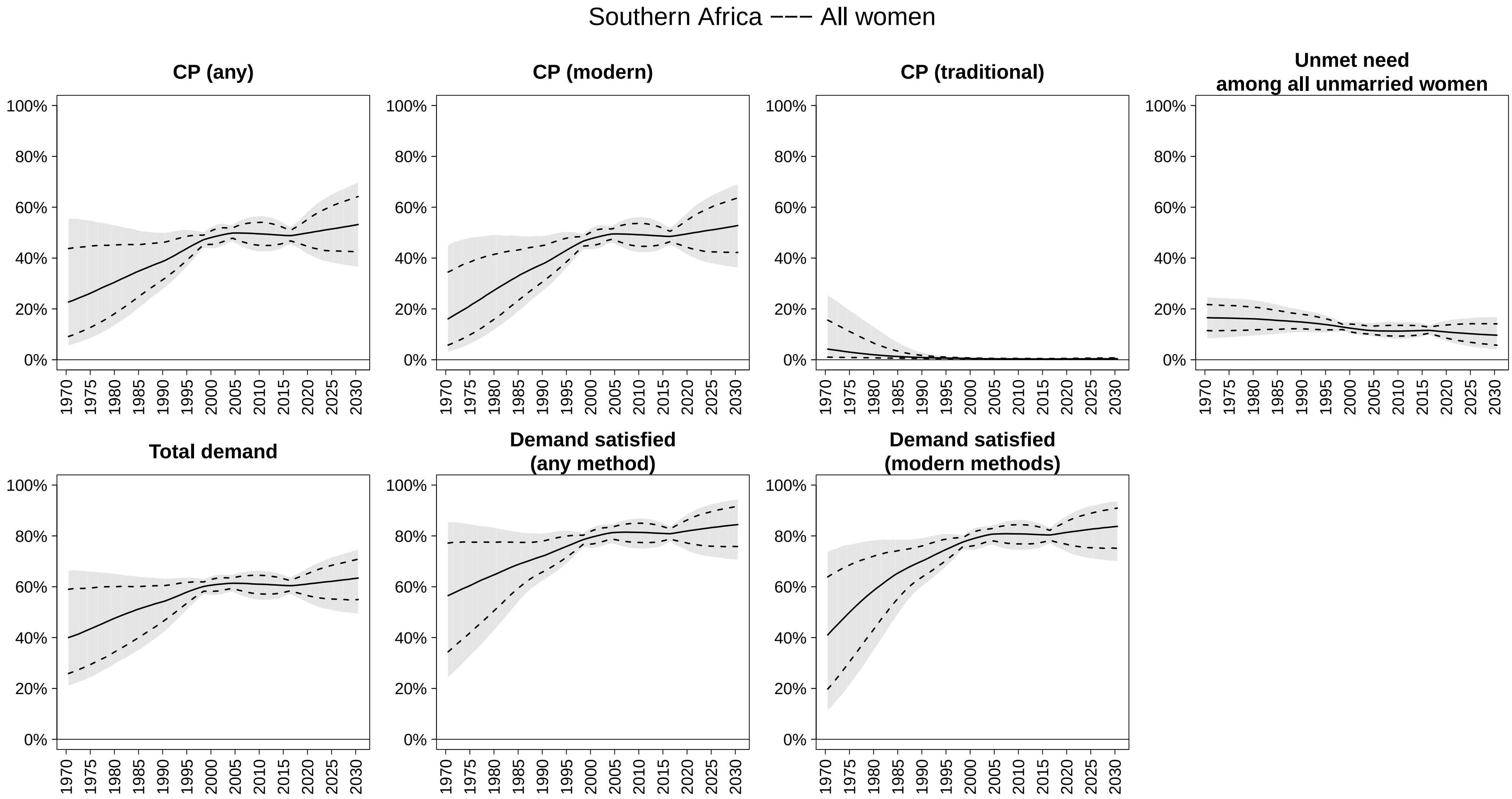
Southern Africa −−− All women
CP (any)
CP (modern)
CP (traditional)
Unmet need among all unmarried women
Total demand
Demand satisfied (any method)
Demand satisfied (modern methods)
0%
20%
40%
60%
80%
100%
1970
1975
1980
1985
1990
1995
2000
2005
2010
2015
2020
2025
2030

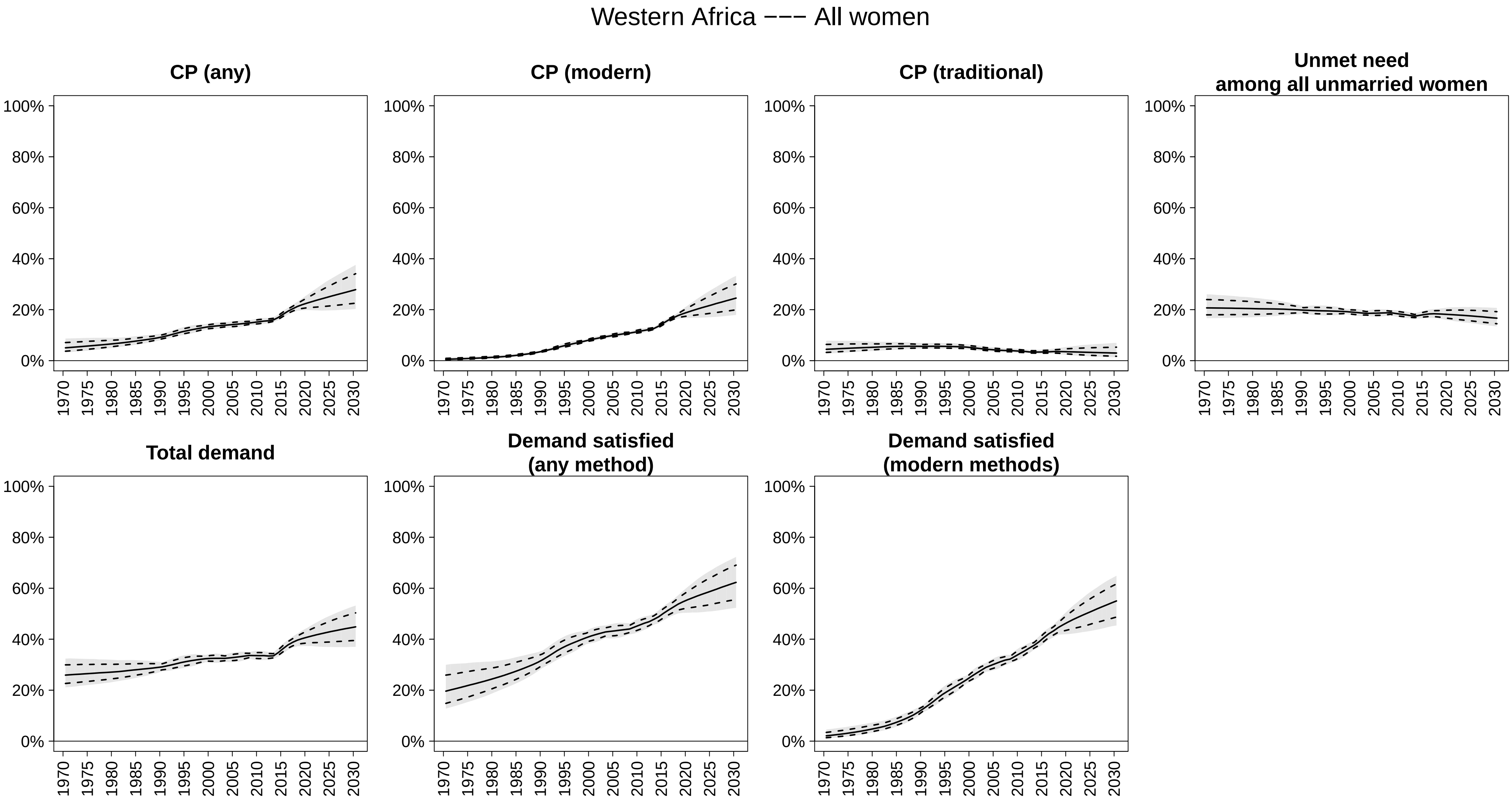
Western Africa --- All women
CP (any)
CP (modern)
CP (traditional)
Unmet need
among all unmarried women
Total demand
Demand satisfied
(any method)
Demand satisfied
(modern methods)
100%
80%
60%
40%
20%
0%
1970
1975
1980
1985
1990
1995
2000
2005
2010
2015
2020
2025
2030

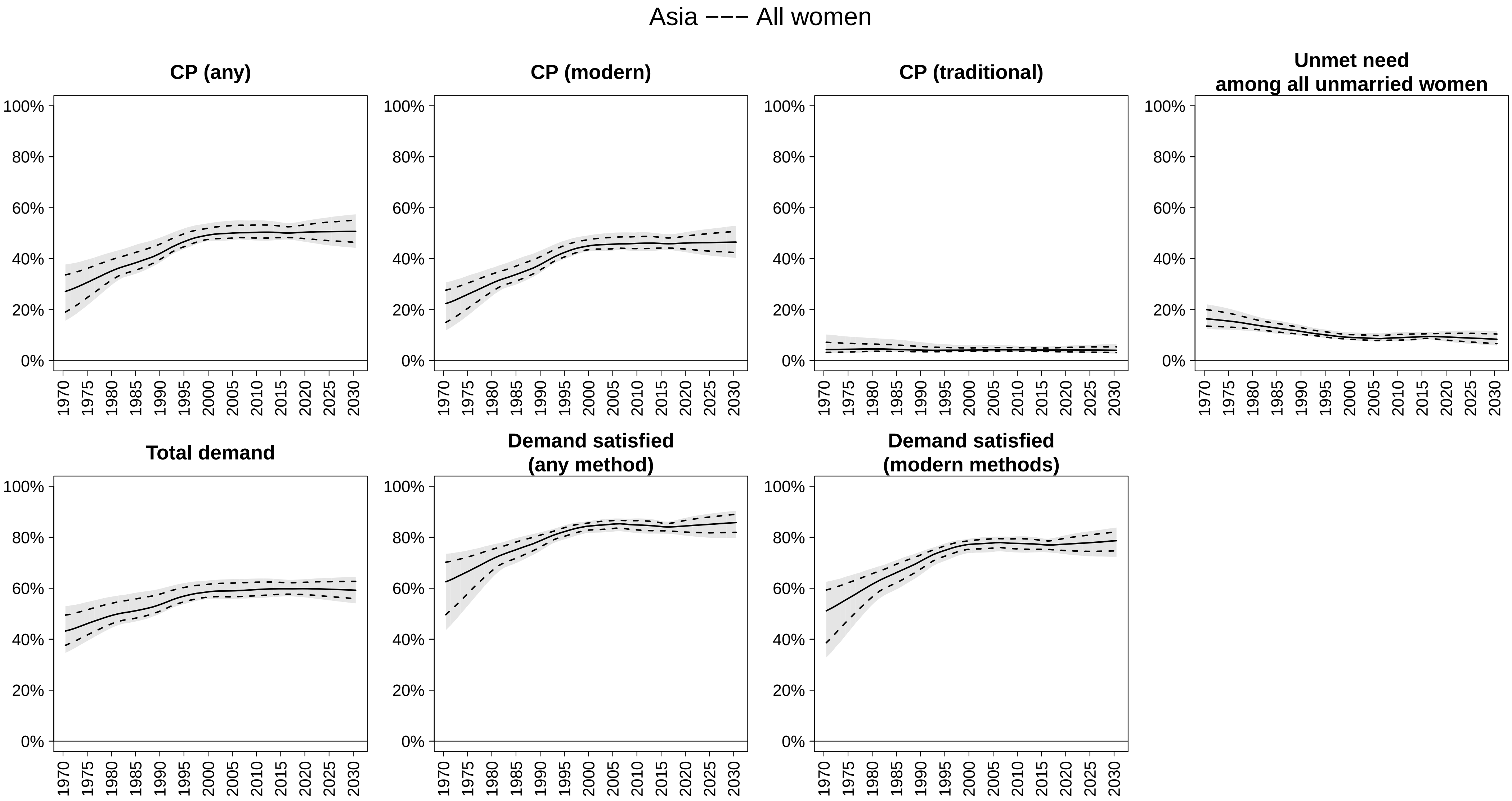

Asia −−− All women
CP (any)
CP (modern)
CP (traditional)
Unmet need among all unmarried women
Total demand
Demand satisfied (any method)
Demand satisfied (modern methods)
0%
20%
40%
60%
80%
100%
1970
1975
1980
1985
1990
1995
2000
2005
2010
2015
2020
2025
2030

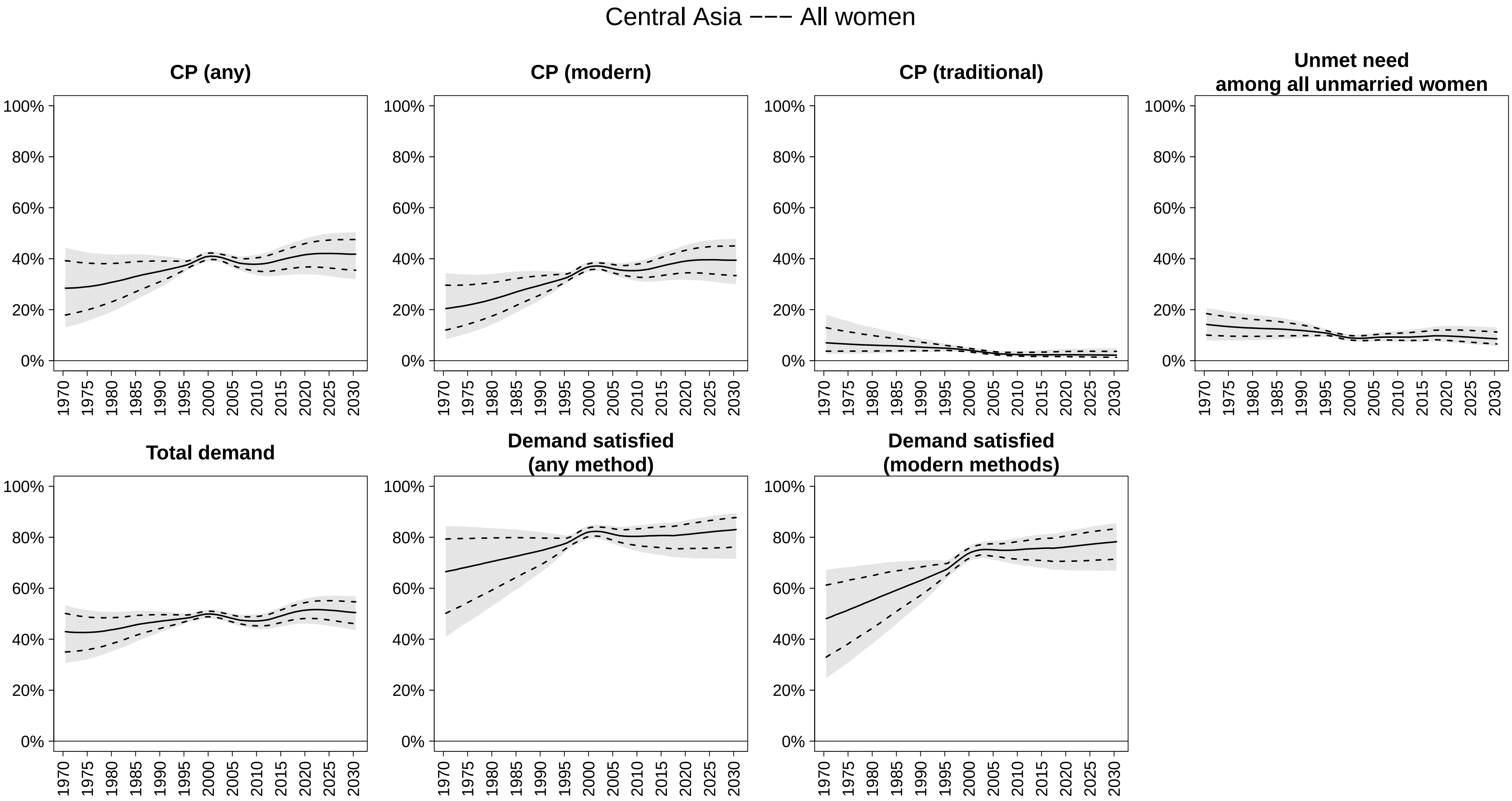
Central Asia −−− All women
CP (any)
CP (modern)
CP (traditional)
Unmet need
among all unmarried women
Total demand
Demand satisfied
(any method)
Demand satisfied
(modern methods)
0%
20%
40%
60%
80%
100%
1970
1975
1980
1985
1990
1995
2000
2005
2010
2015
2020
2025
2030

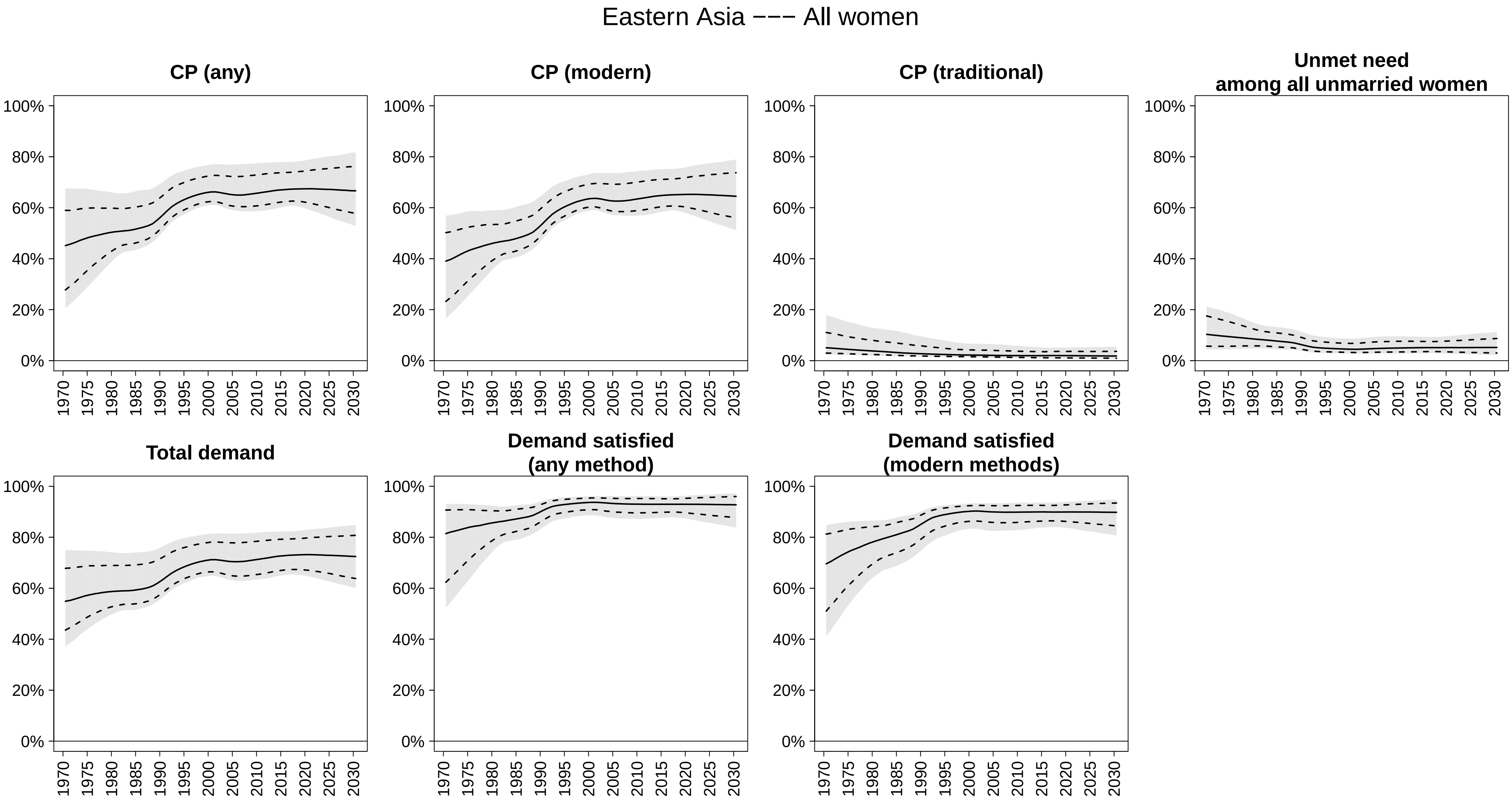
Eastern Asia −−− All women
CP (any)
CP (modern)
CP (traditional)
Unmet need
among all unmarried women
Total demand
Demand satisfied
(any method)
Demand satisfied
(modern methods)
0%
20%
40%
60%
80%
100%
1970
1975
1980
1985
1990
1995
2000
2005
2010
2015
2020
2025
2030

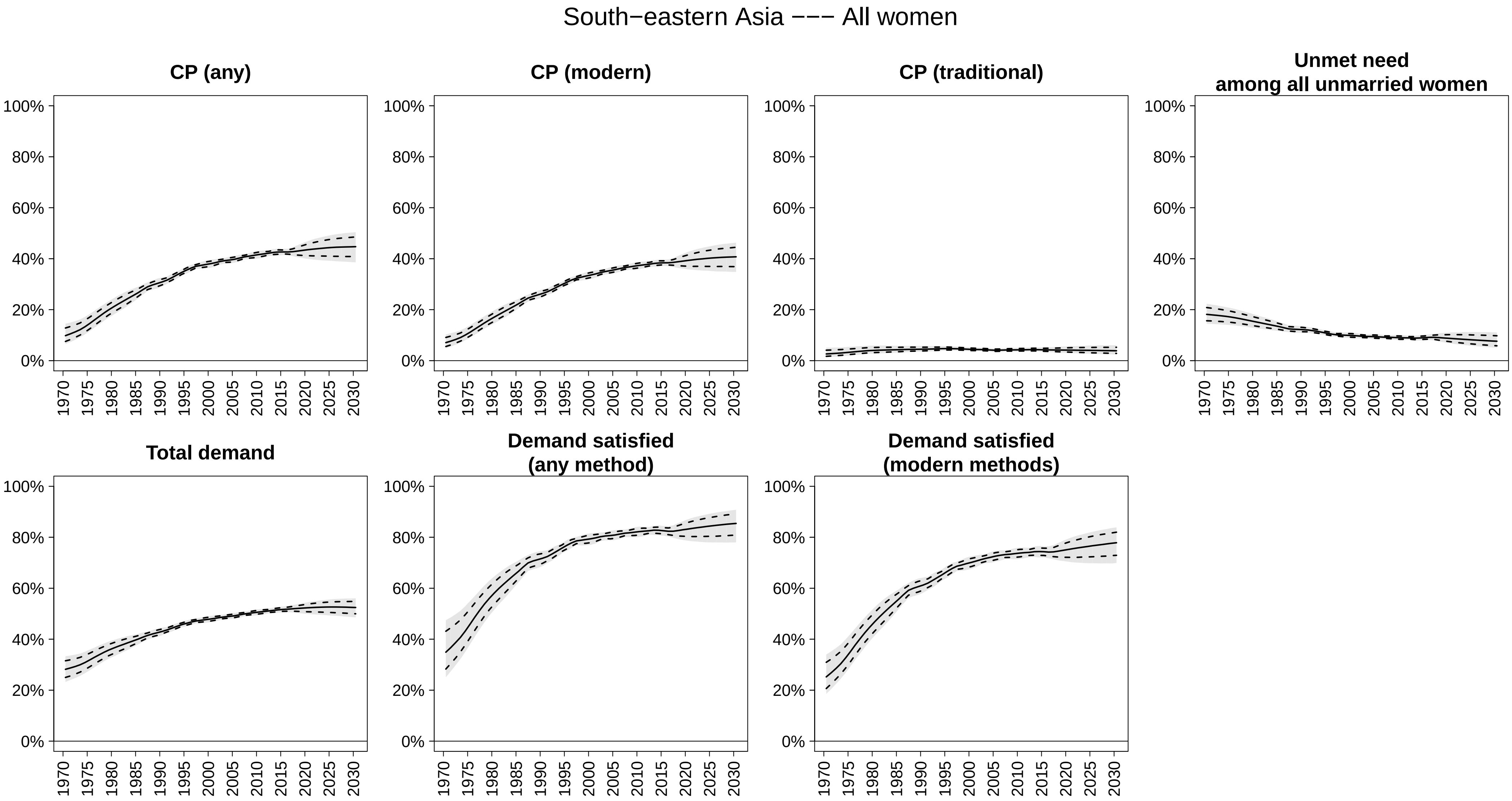
South−eastern Asia −−− All women
CP (any)
CP (modern)
CP (traditional)
Unmet need
among all unmarried women
Total demand
Demand satisfied
(any method)
Demand satisfied
(modern methods)
0%
20%
40%
60%
80%
100%
1970
1975
1980
1985
1990
1995
2000
2005
2010
2015
2020
2025
2030

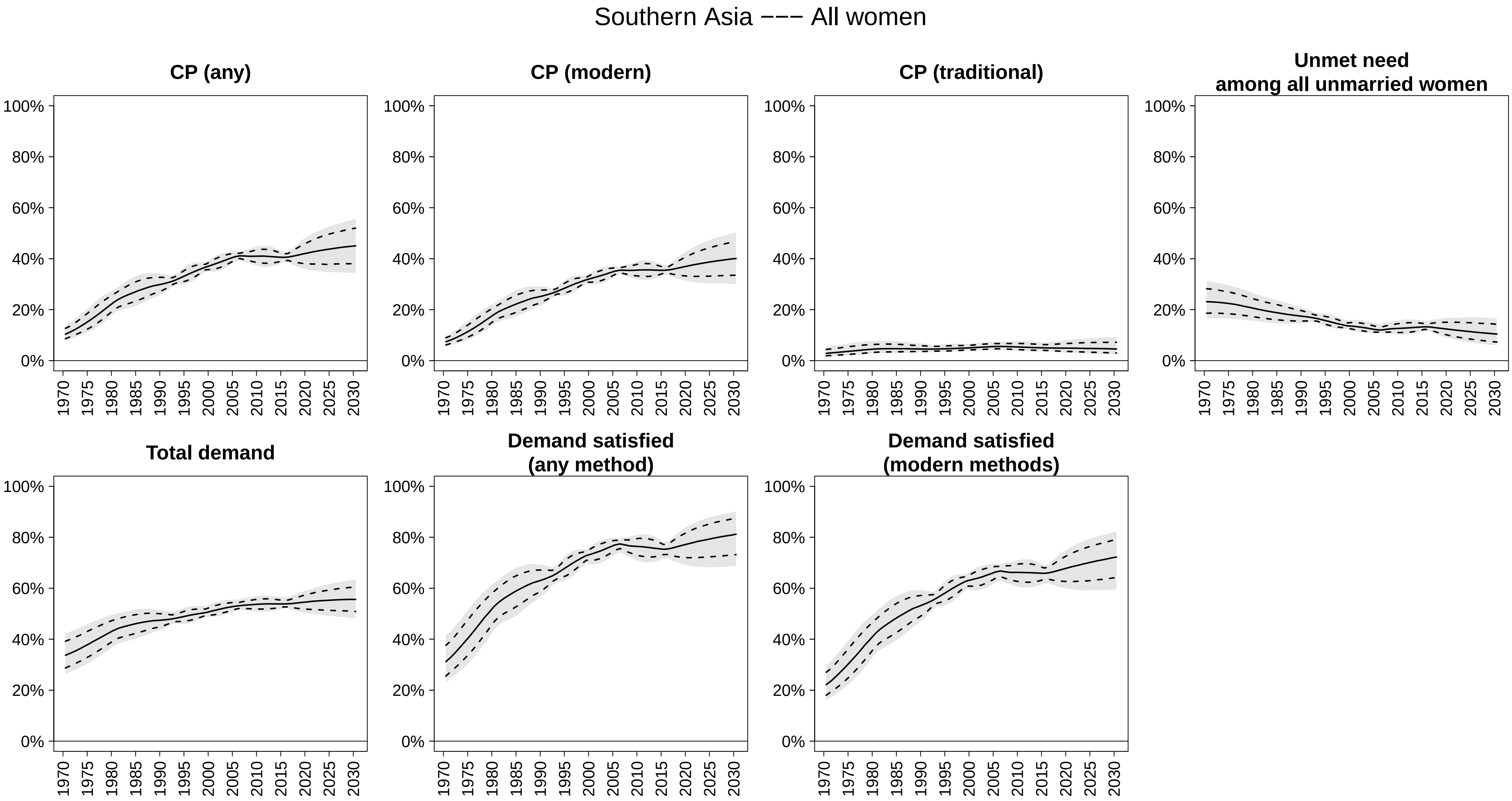
Southern Asia --- All women
CP (any)
CP (modern)
CP (traditional)
Unmet need among all unmarried women
Total demand
Demand satisfied (any method)
Demand satisfied (modern methods)
0%
20%
40%
60%
80%
100%
1970
1975
1980
1985
1990
1995
2000
2005
2010
2015
2020
2025
2030

Western Asia −−− All women

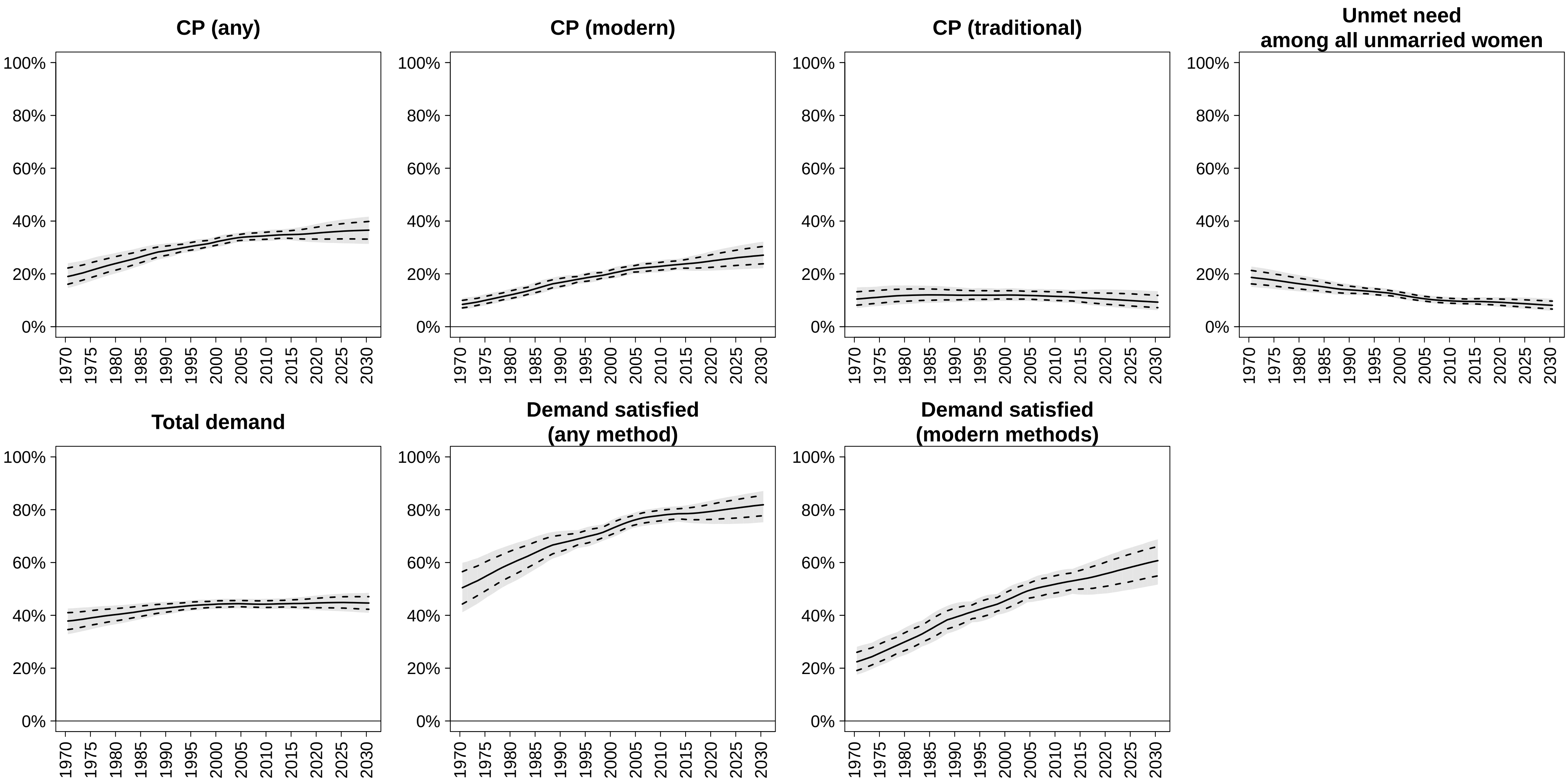

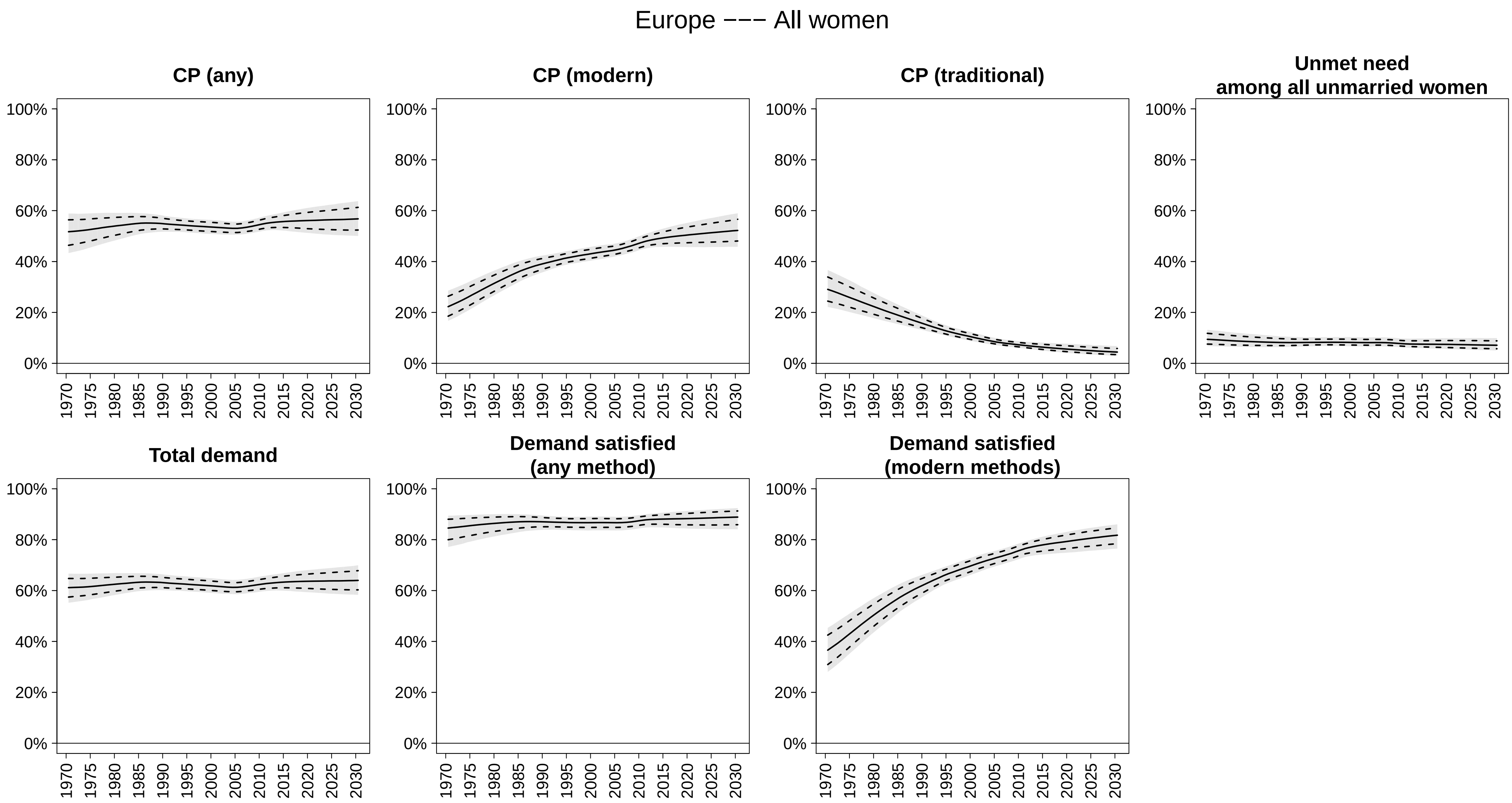
Europe --- All women
CP (any)
CP (modern)
CP (traditional)
Unmet need
among all unmarried women
Total demand
Demand satisfied
(any method)
Demand satisfied
(modern methods)
100%
80%
60%
40%
20%
0%
1970
1975
1980
1985
1990
1995
2000
2005
2010
2015
2020
2025
2030

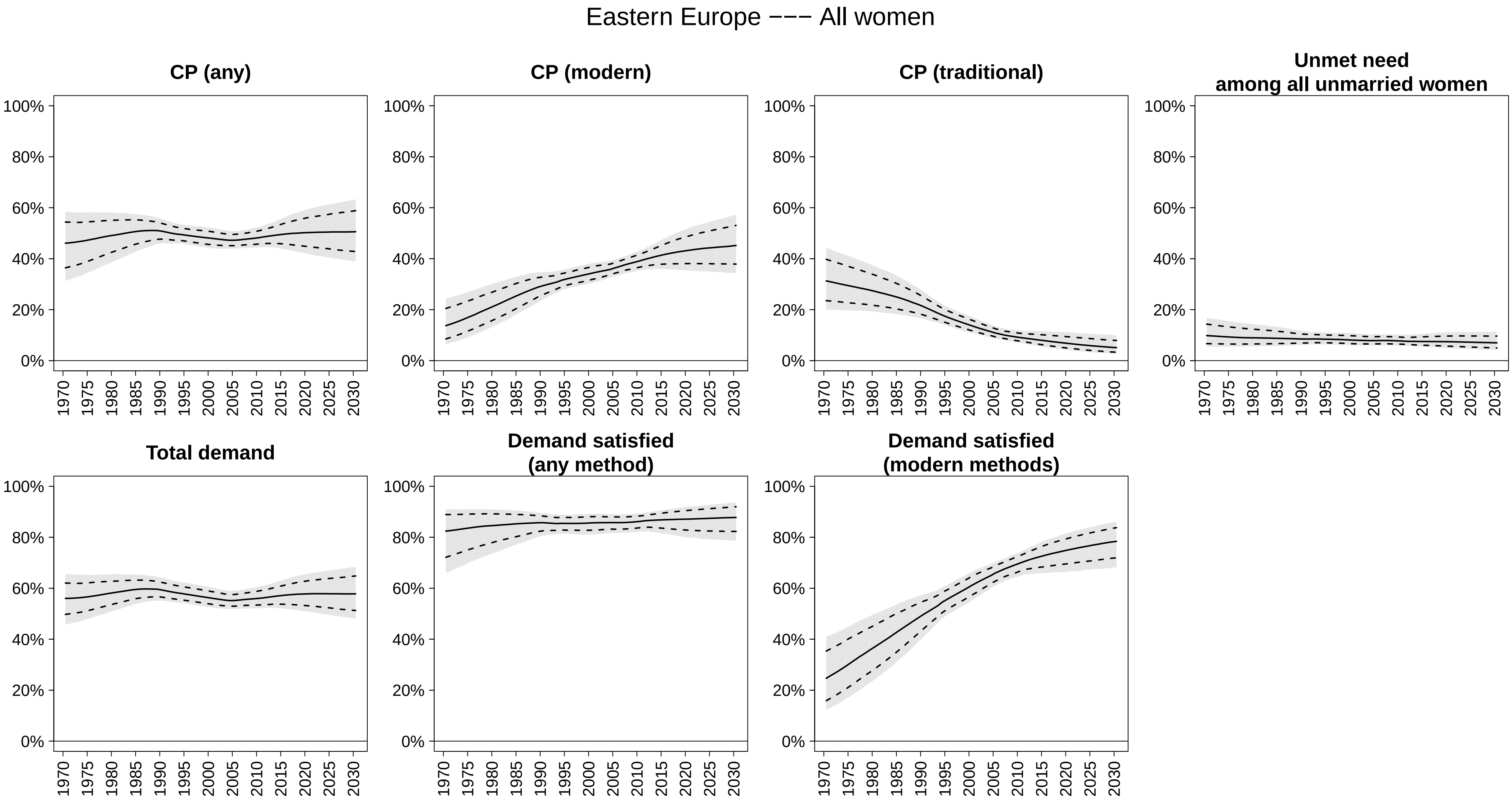
Eastern Europe −−− All women
CP (any)
CP (modern)
CP (traditional)
Unmet need
among all unmarried women
Total demand
Demand satisfied
(any method)
Demand satisfied
(modern methods)
0%
20%
40%
60%
80%
100%
1970
1975
1980
1985
1990
1995
2000
2005
2010
2015
2020
2025
2030

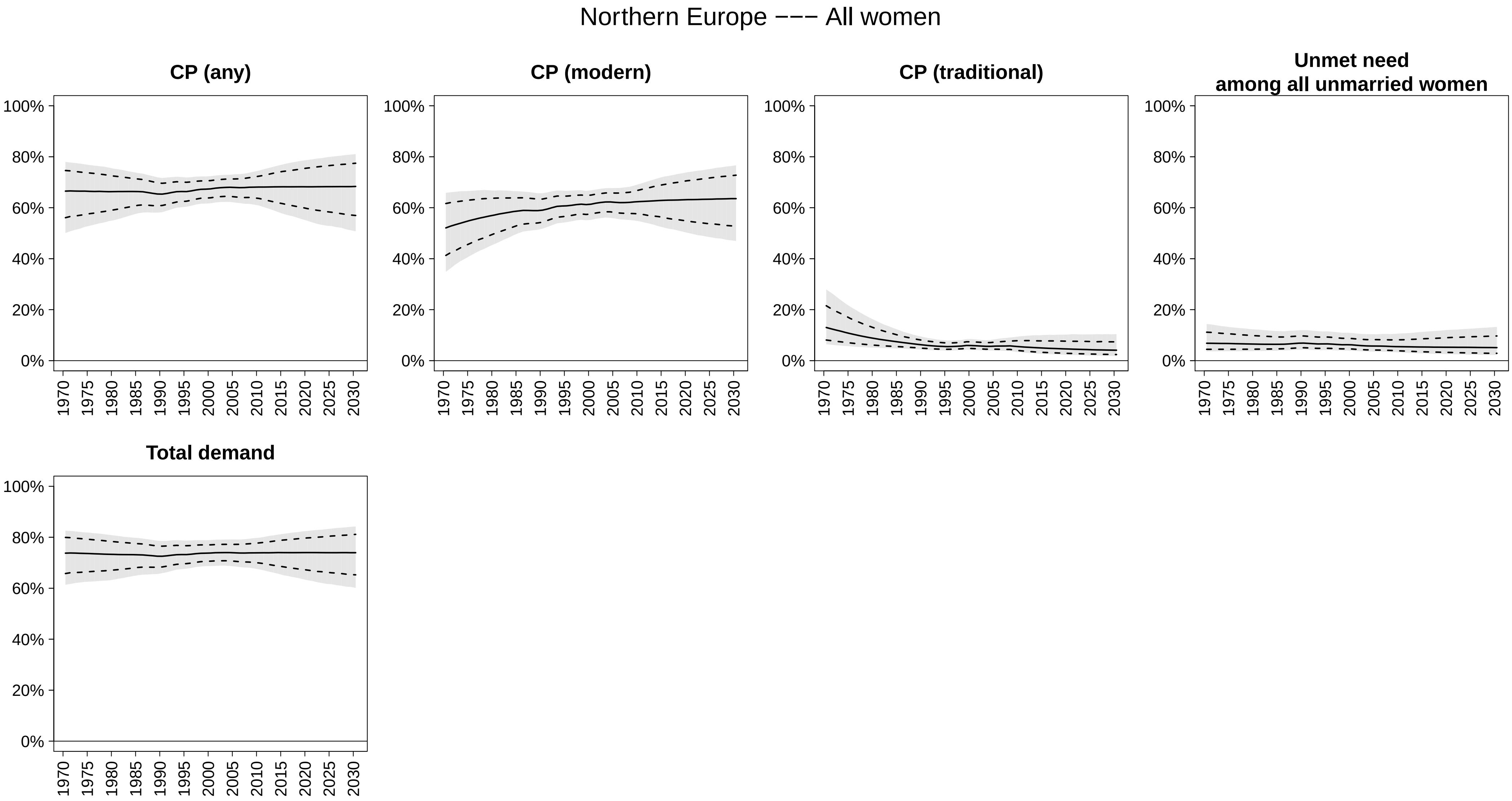
Northern Europe −−− All women
CP (any)
CP (modern)
CP (traditional)
Unmet need
among all unmarried women
Total demand
100%
80%
60%
40%
20%
0%
1970
1975
1980
1985
1990
1995
2000
2005
2010
2015
2020
2025
2030

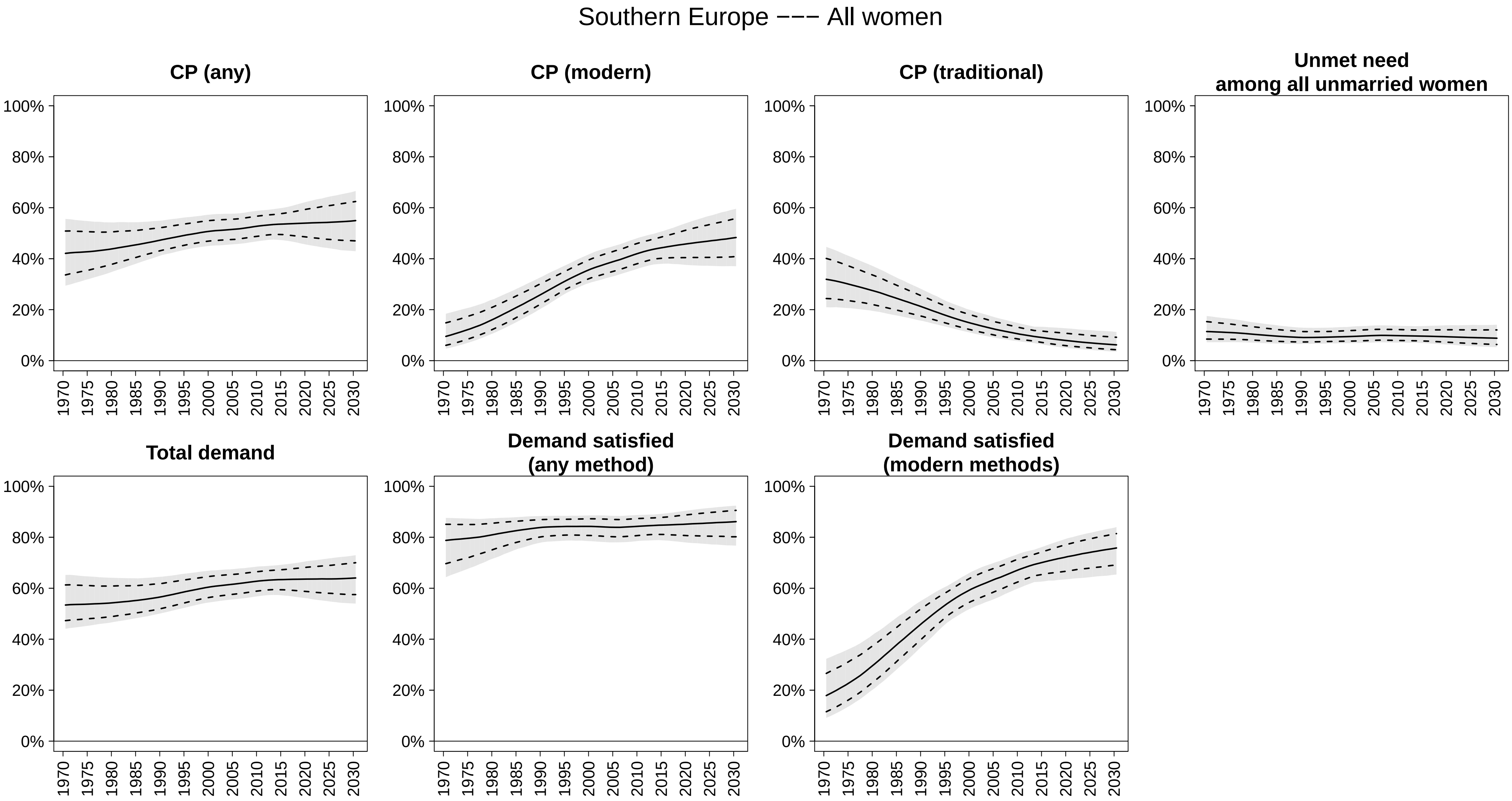
Southern Europe −−− All women
CP (any)
CP (modern)
CP (traditional)
Unmet need
among all unmarried women
Total demand
Demand satisfied
(any method)
Demand satisfied
(modern methods)
0%
20%
40%
60%
80%
100%
1970
1975
1980
1985
1990
1995
2000
2005
2010
2015
2020
2025
2030

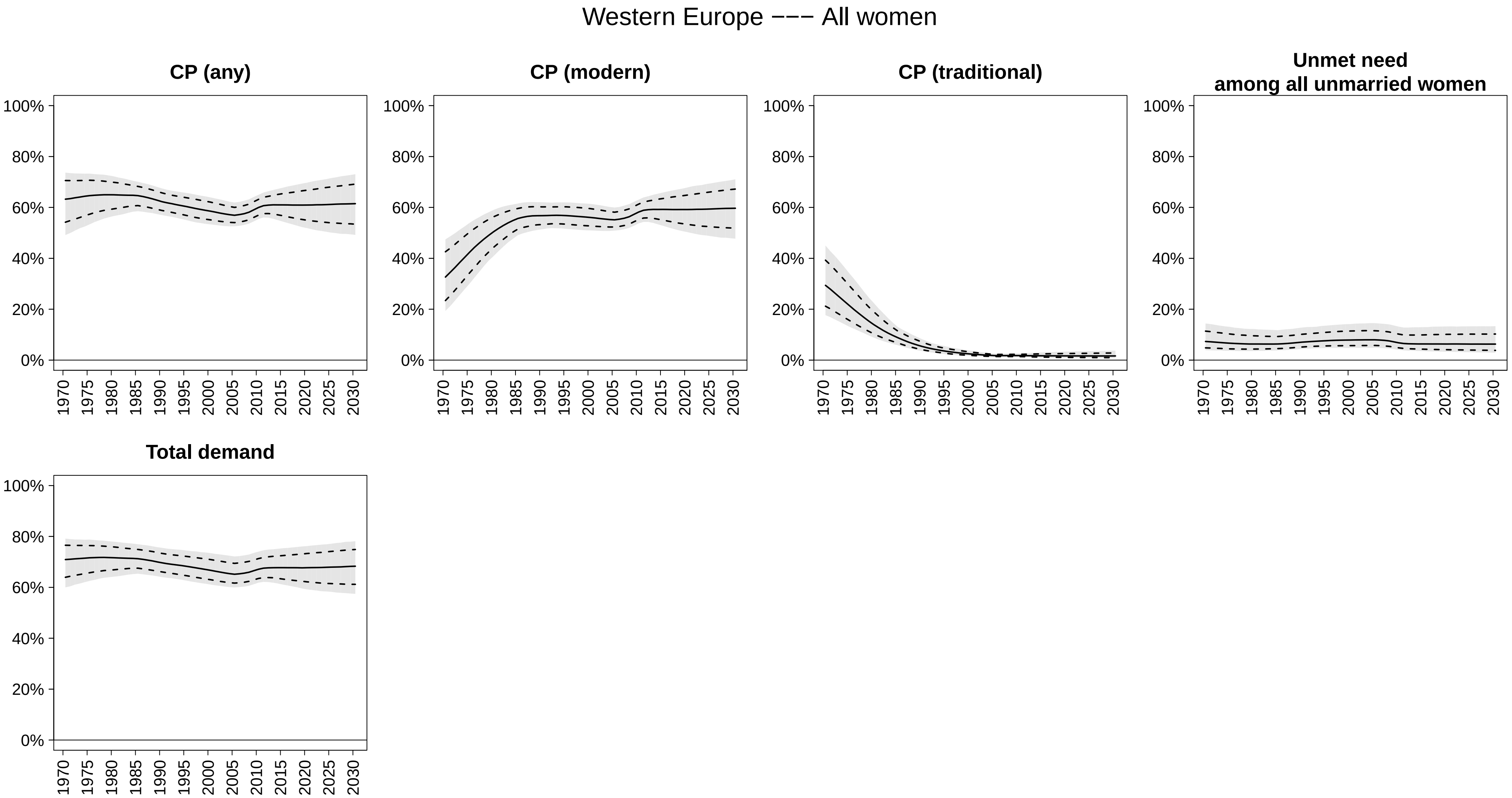
Western Europe −−− All women
CP (any)
CP (modern)
CP (traditional)
Unmet need
among all unmarried women
Total demand
0%
20%
40%
60%
80%
100%
1970
1975
1980
1985
1990
1995
2000
2005
2010
2015
2020
2025
2030

Latin America and the Caribbean −−− All women

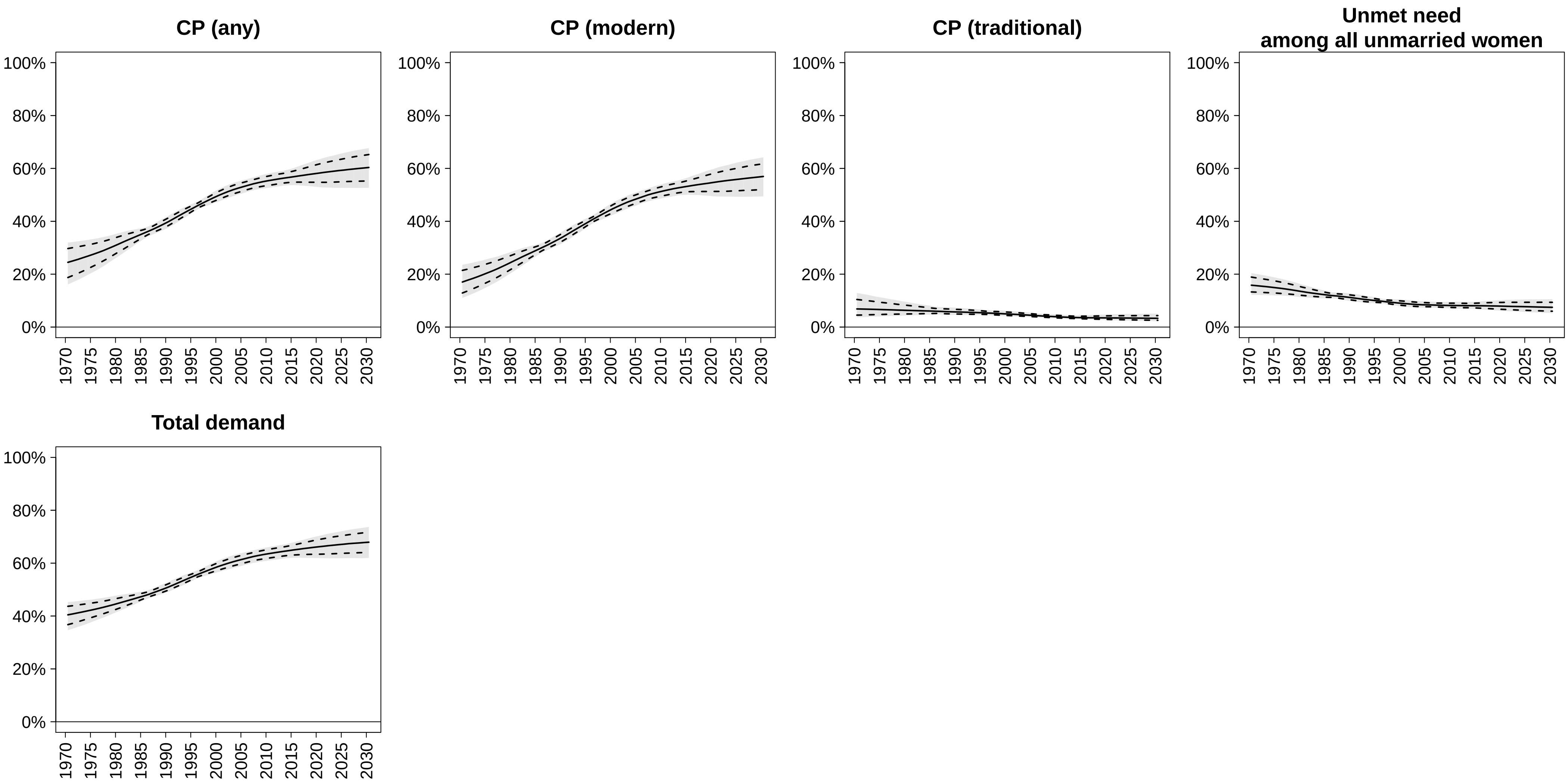

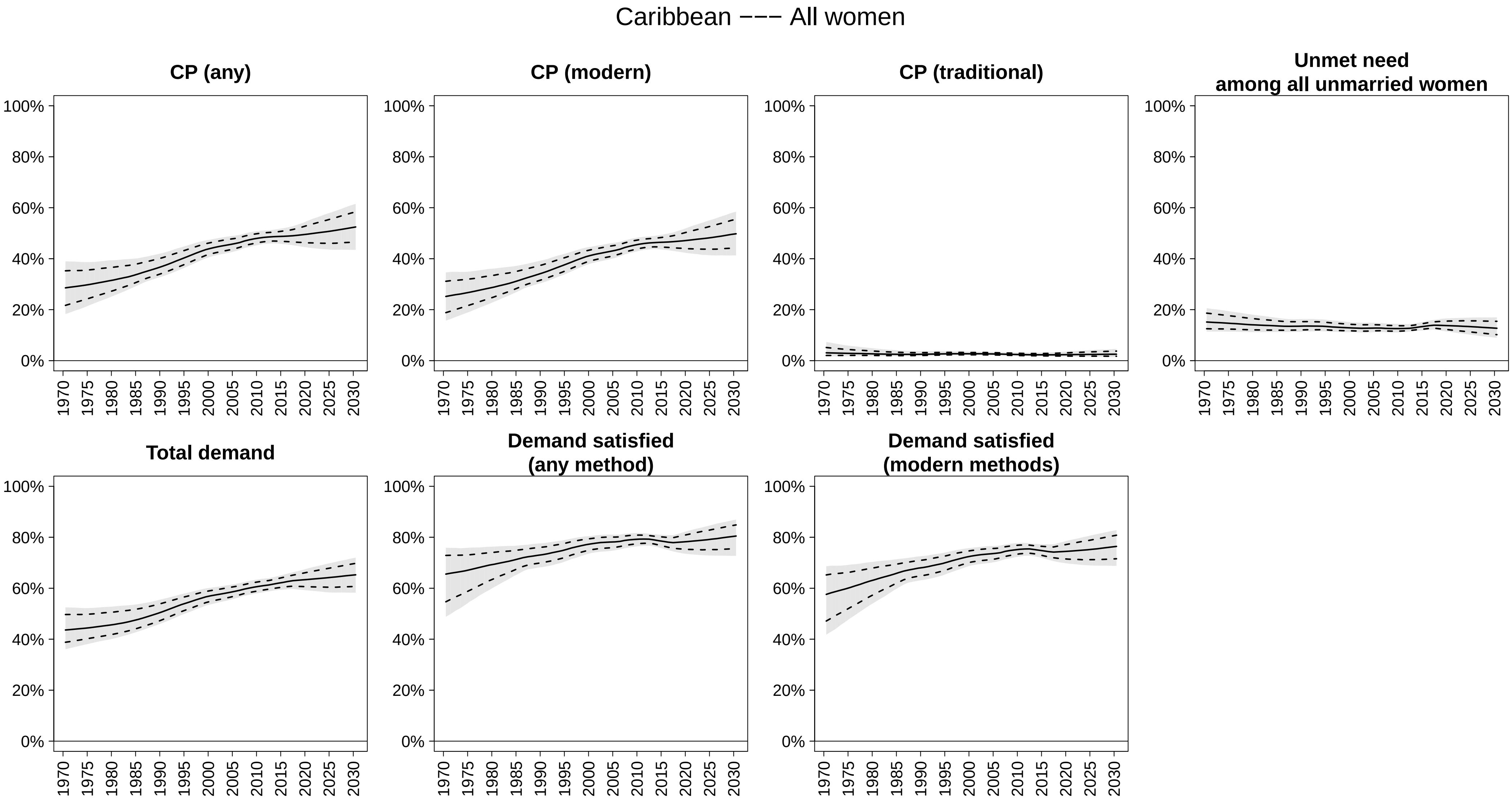
Caribbean −−− All women
CP (any)
CP (modern)
CP (traditional)
Unmet need among all unmarried women
Total demand
Demand satisfied (any method)
Demand satisfied (modern methods)
0%
20%
40%
60%
80%
100%
1970
1975
1980
1985
1990
1995
2000
2005
2010
2015
2020
2025
2030

Central America −−− All women

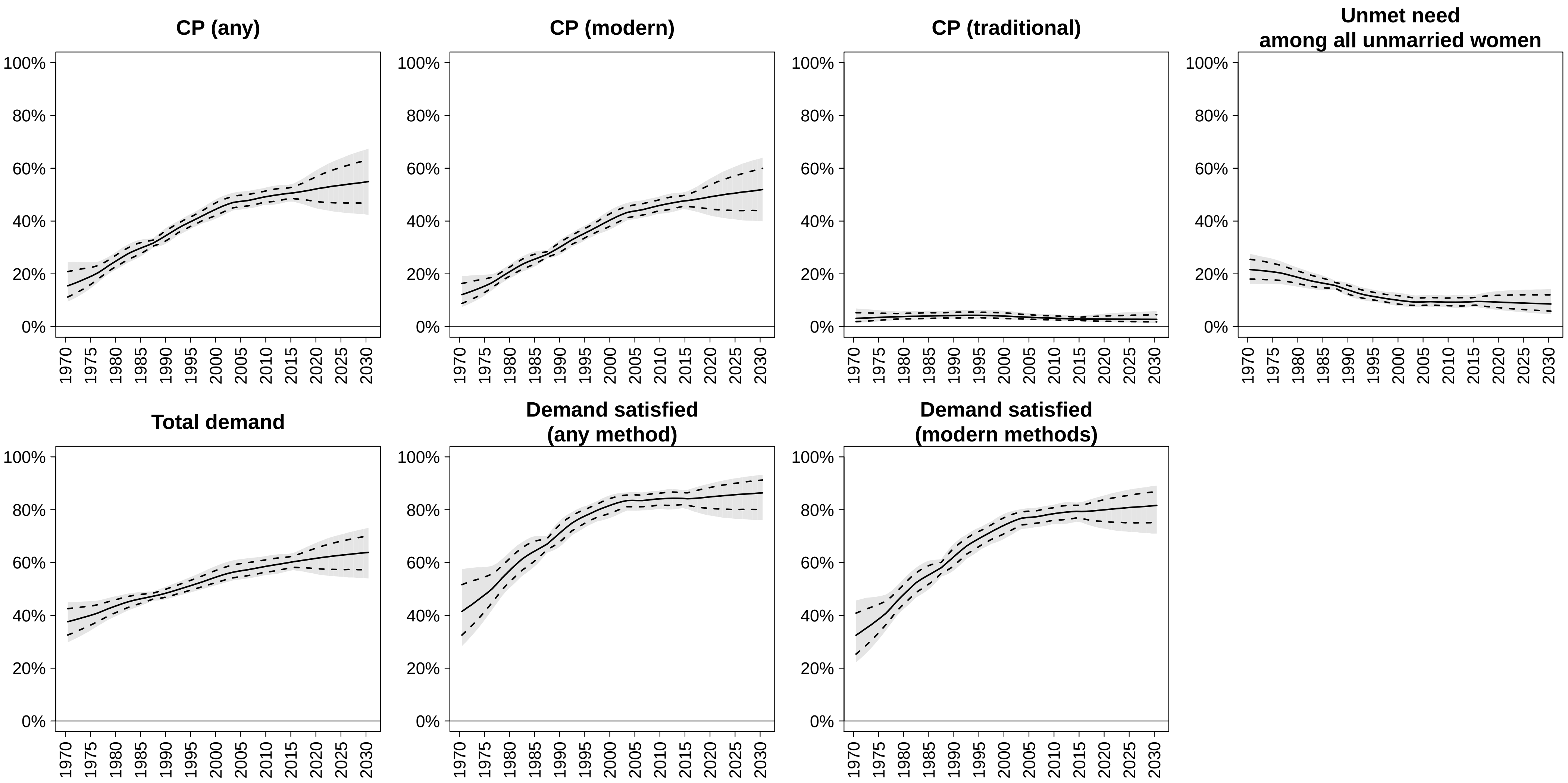

South America −−− All women

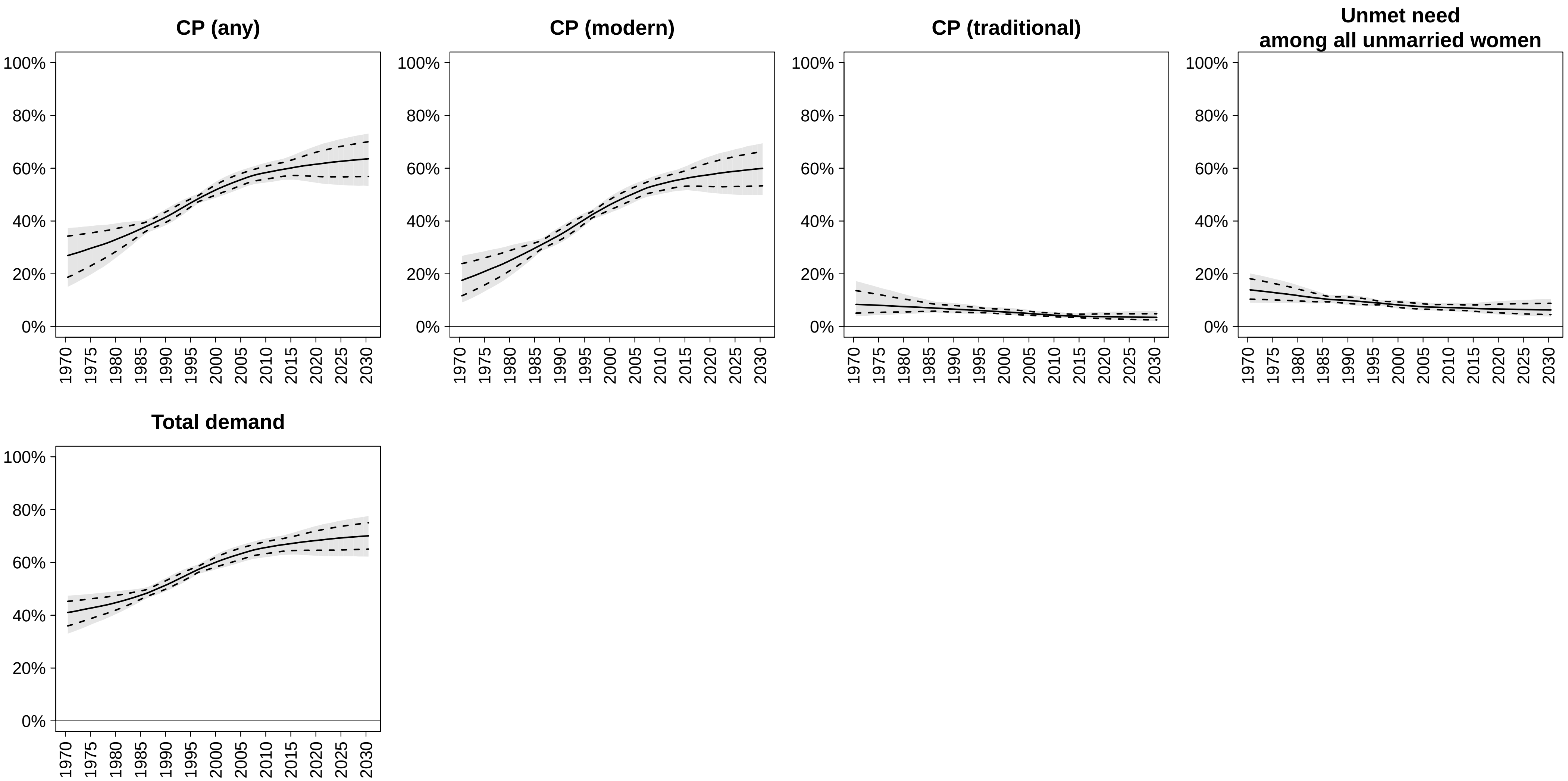

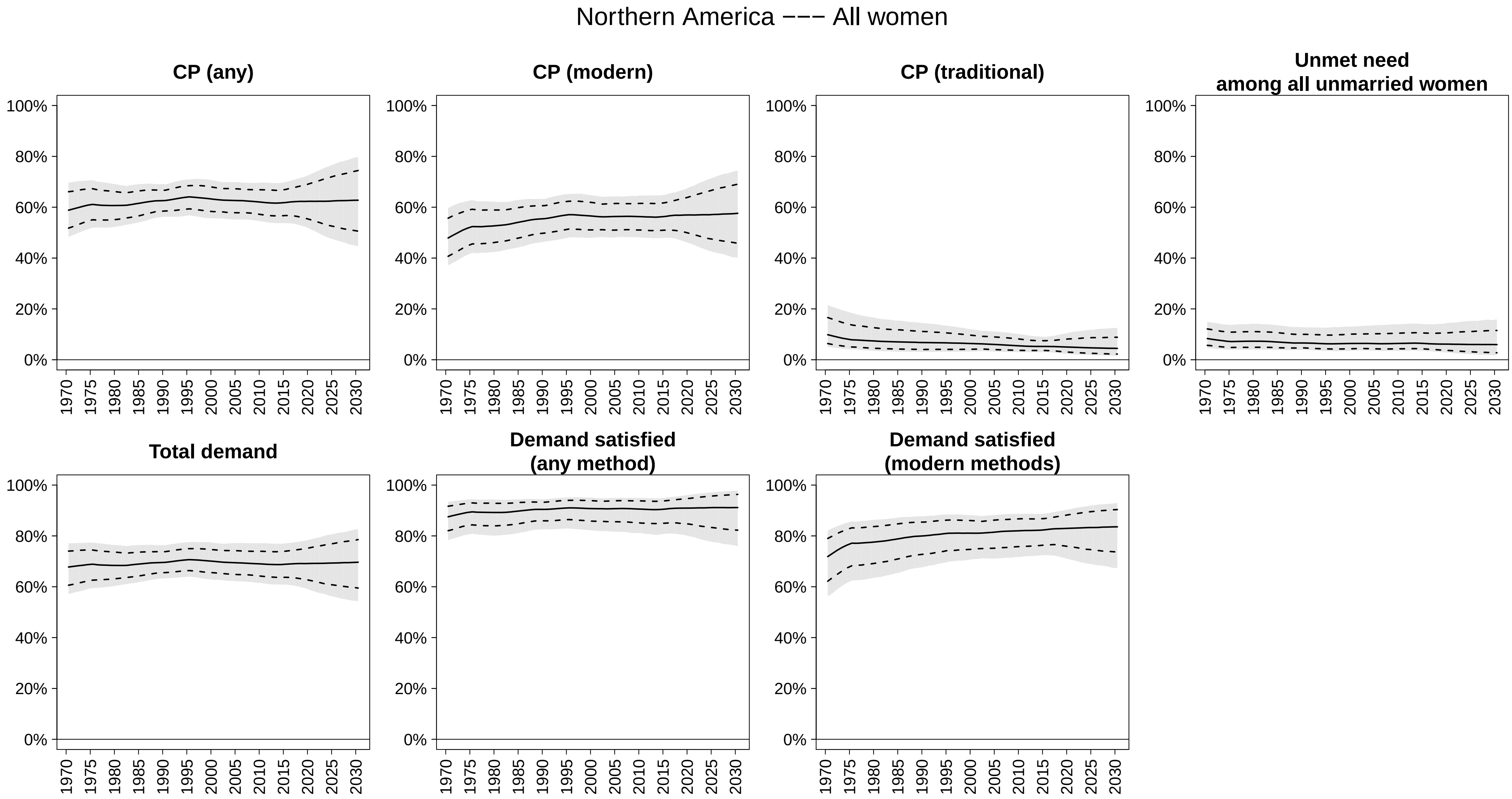
Northern America −−− All women
CP (any)
CP (modern)
CP (traditional)
Unmet need among all unmarried women
Total demand
Demand satisfied (any method)
Demand satisfied (modern methods)
0%
20%
40%
60%
80%
100%
1970
1975
1980
1985
1990
1995
2000
2005
2010
2015
2020
2025
2030

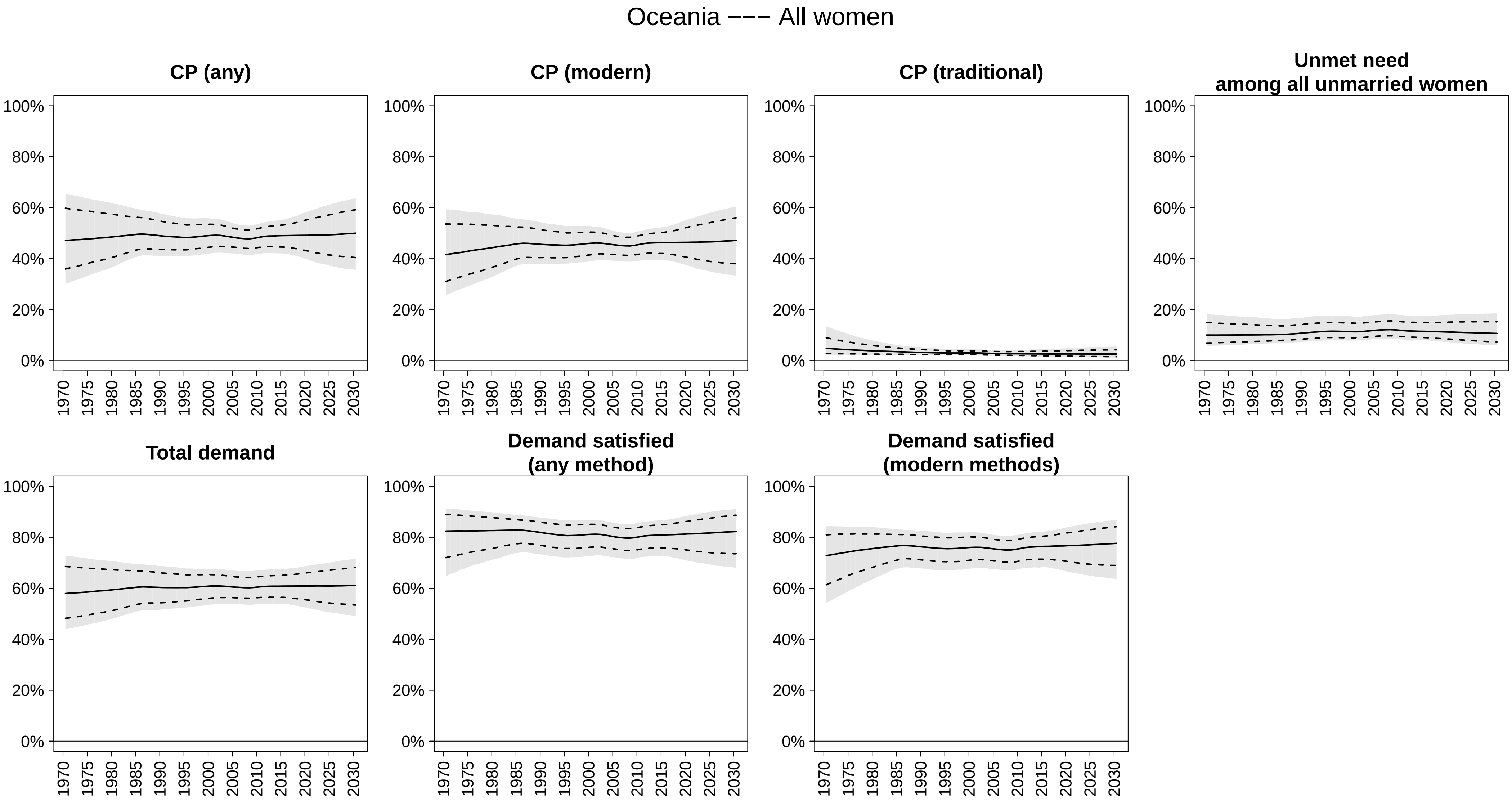
Oceania −−− All women
CP (any)
CP (modern)
CP (traditional)
Unmet need
among all unmarried women
Total demand
Demand satisfied
(any method)
Demand satisfied
(modern methods)
0%
20%
40%
60%
80%
100%
1970
1975
1980
1985
1990
1995
2000
2005
2010
2015
2020
2025
2030

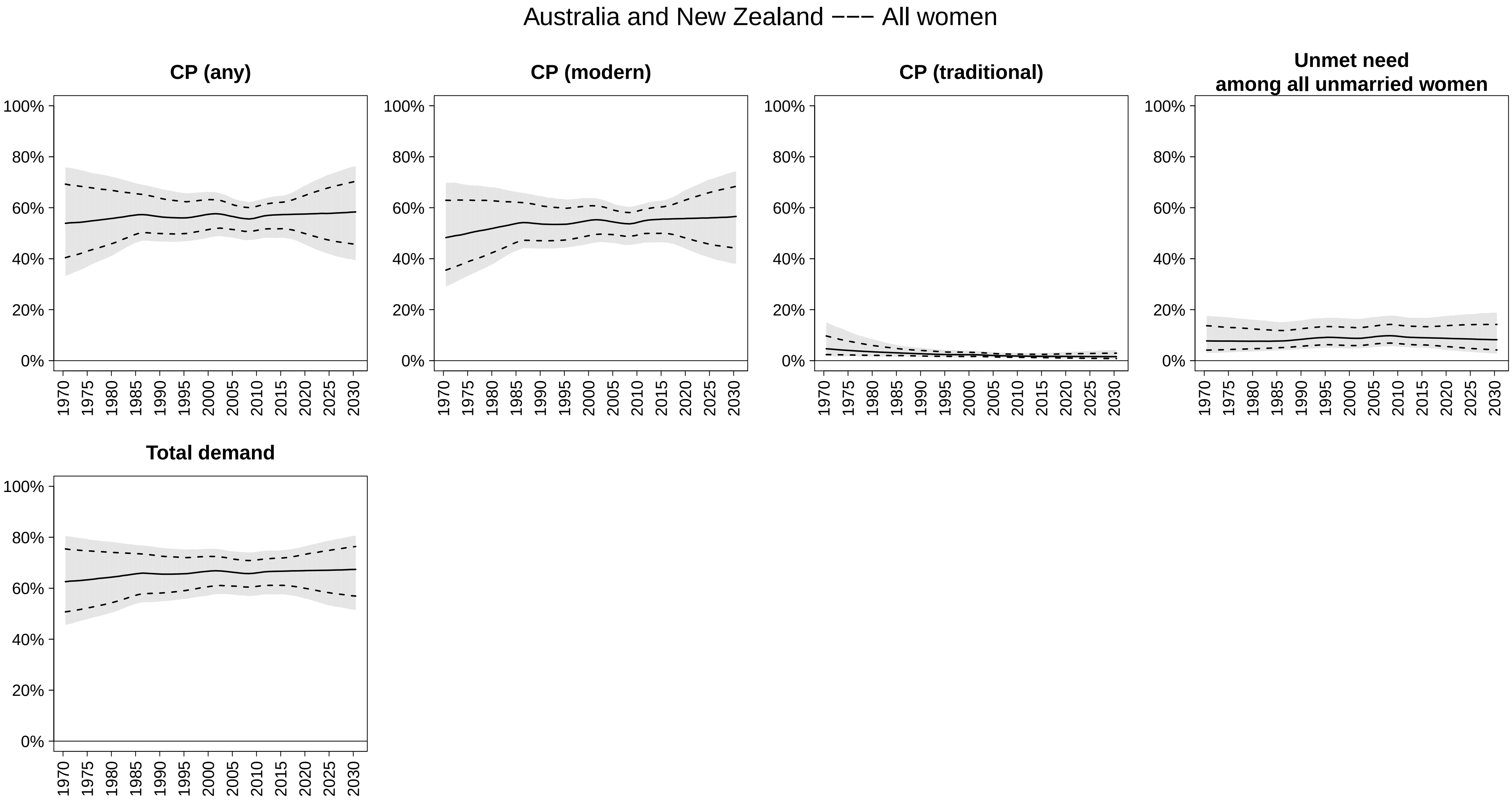
Australia and New Zealand −−− All women
CP (any)
CP (modern)
CP (traditional)
Unmet need among all unmarried women
Total demand
100%
80%
60%
40%
20%
0%
1970 1975 1980 1985 1990 1995 2000 2005 2010 2015 2020 2025 2030

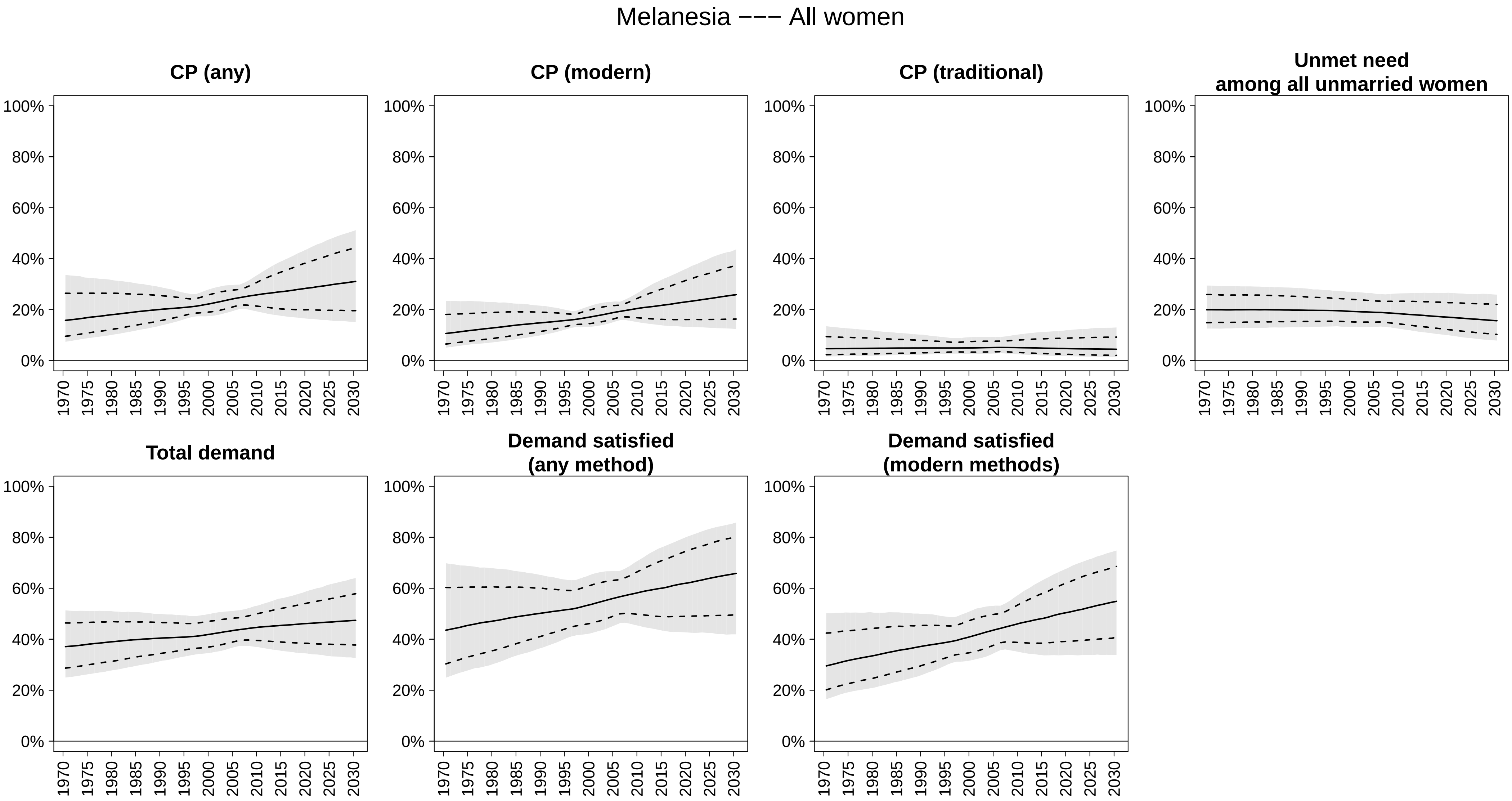
Melanesia −−− All women
CP (any)
CP (modern)
CP (traditional)
Unmet need among all unmarried women
Total demand
Demand satisfied (any method)
Demand satisfied (modern methods)
0%
20%
40%
60%
80%
100%
1970
1975
1980
1985
1990
1995
2000
2005
2010
2015
2020
2025
2030

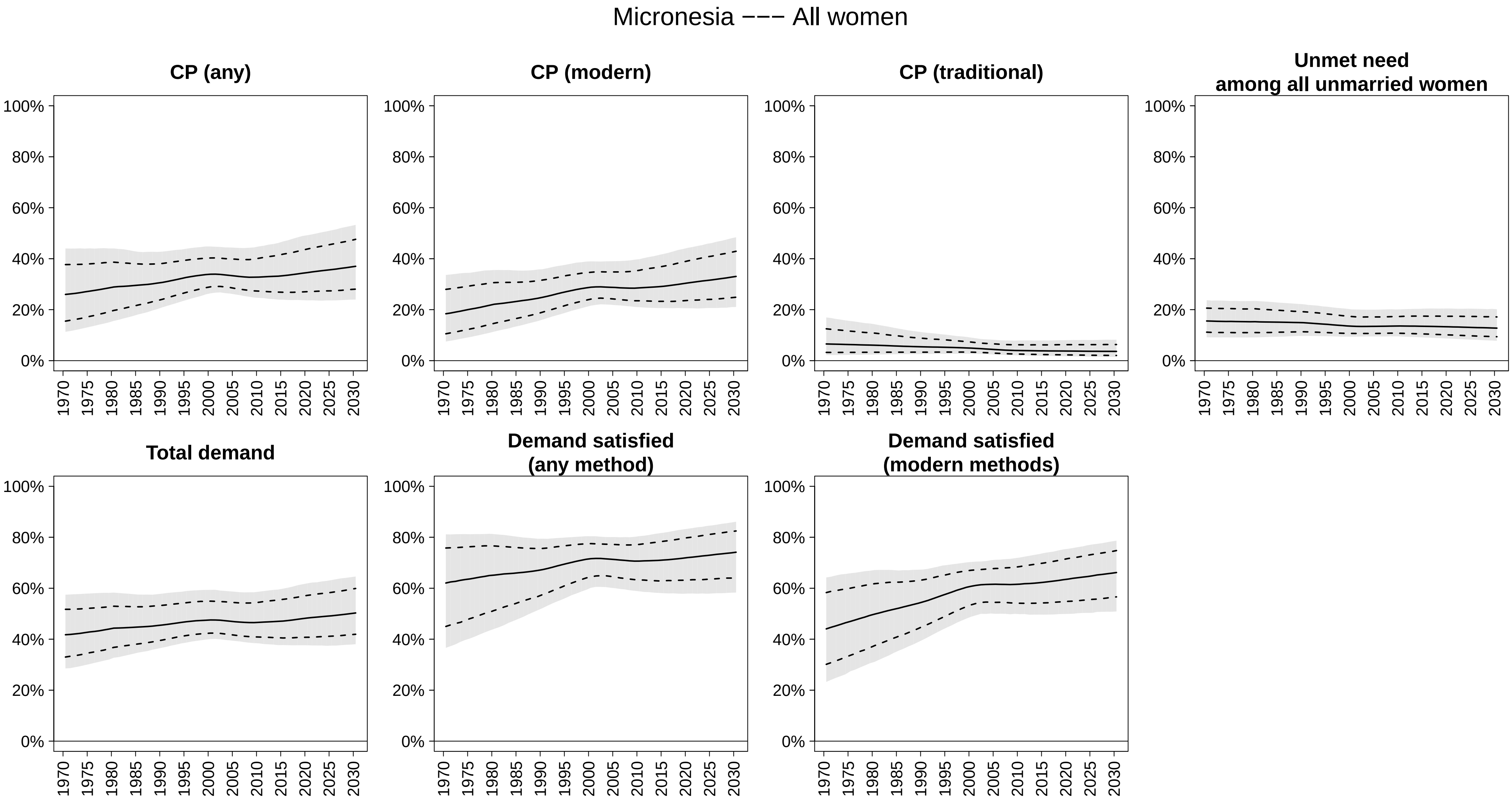
Micronesia −−− All women
CP (any)
CP (modern)
CP (traditional)
Unmet need
among all unmarried women
Total demand
Demand satisfied
(any method)
Demand satisfied
(modern methods)
100%
80%
60%
40%
20%
0%
1970
1975
1980
1985
1990
1995
2000
2005
2010
2015
2020
2025
2030

Polynesia −−− All women

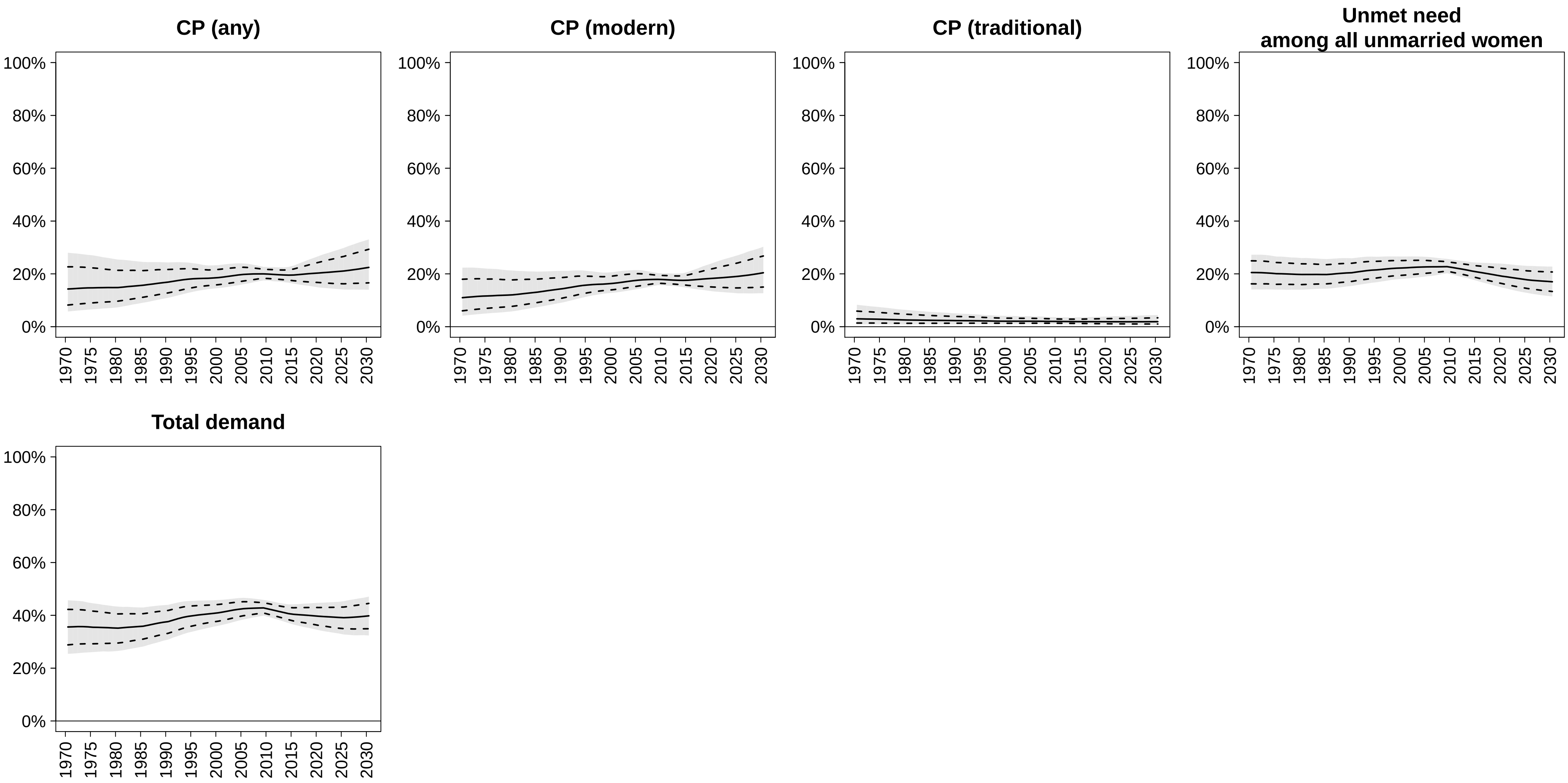

Supplement: S2 Appendix — (PDF) [file pmed.1003026.s002.pdf]
