# Supplementary material for: Estimating progress towards meeting women’s contraceptive needs in 185 countries: A Bayesian hierarchical modelling study
Source: PLoS Med. 2020 Feb 18;17(2):e1003026. doi: 10.1371/journal.pmed.1003026 (PMC7028249; doi:10.1371/journal.pmed.1003026)
Supplement: Attachment — Submitted filename: Replies to reviewers&editors_20191109.docx [file pmed.1003026.s014.docx]

Response to Reviewers’ Comments

PMEDICINE-D-19-03179

We have copied the editors’ and reviewers’ comments in **bold** and replied underneath in normal font. All modifications can be clearly seen in the track changes version of the manuscript also supplied with the resubmission.

We thank the three reviewers and the editors for their constructive comments and hope that we have been able to satisfy their concerns.

# Requests from the editors:

[Please note that we have numbered these comments for easier reference]

1. Title – it’s quite long and we also need a study descriptor, can we instead change to: Progress in 2030 sustainable development goals for contraceptive access and use: predictive model estimates from 185 countries

Thank you for the suggestions of including the study descriptor and mentioning of SDGs. We would like to suggest the following title:

Progress towards meeting contraceptive needs in the SDG era: Bayesian hierarchical model estimates and projections for 185 countries

2. Abstract – Please provide summary demographic details of women included in the survey; please use 95% Cis and p values here (and throughout including the main text and tables, etc) for any quantifiable data.

We present the median and the 95% uncertainty intervals, and posterior probabilities of increase/decrease (PPI/D) for the changes for all results throughout the paper and appendixes. We use Bayesian methods so we do not compute p-values. We updated the formatting to reflect the PLOS Medicine style.

In the Abstract, we added text that the population of the estimates are women of reproductive age (15-49 years). We provide more information on the demographic details of women included in the surveys in input files (age groups) and describe the data categorization based on characteristics of the population sampled and perturbation multipliers in S. Appendix, section 3.5.7. Age groups characteristics are included in the input file.

3. At this stage, we ask that you include a short, non-technical Author Summary of your research to make findings accessible to a wide audience that includes both scientists and non-scientists. The Author Summary should immediately follow the Abstract in your revised manuscript. This text is subject to editorial change and should be distinct from the scientific abstract. Please see our author guidelines for more information: <https://journals.plos.org/plosmedicine/s/revising-your-manuscript#loc-author-summary>

This has been done and uploaded with the resubmission.

4. Please avoid using itals font for emphasis

We found one instance of this in the second paragraph of the Introduction and have removed it.

5. Refs in the main text, please use square brackets instead of superscript and the brackets and refs should occur before the full stop.

We corrected all references in the text to square brackets.

6. Did your study have a prospective protocol or analysis plan? Please state this (either way) early in the Methods section.

a) If a prospective analysis plan (from your funding proposal, IRB or other ethics committee submission, study protocol, or other planning document written before analyzing the data) was used in designing the study, please include the relevant prospectively written document with your revised manuscript as a Supporting Information file to be published alongside your study, and cite it in the Methods section. A legend for this file should be included at the end of your manuscript.

b) If no such document exists, please make sure that the Methods section transparently describes when analyses were planned, and when/why any data-driven changes to analyses took place.

c) In either case, changes in the analysis-- including those made in response to peer review comments-- should be identified as such in the Methods section of the paper, with rationale.

No study protocol or analysis plan was published prior to submission. The statistical model for unmarried women estimates and projections is one of the main contributions of our submission (the statistical model for married women is the same as that used by Cahill et al. (2017), but applied to a larger and more recent data set covering all countries globally).

We have added the following paragraph to the end of the ‘Statistical model’ subjection of ‘Methods’:

*A key contribution of this article is the statistical model for estimating contraceptive prevalence and unmet need for family planning among UWRA itself, hence a protocol or analysis plan was not pre-published. The statistical model for MWRA [8,18] is applied to a larger and more recent data set. As in [18], model development began with substantive reasoning about the prevalence of contraceptive use and needs and how they might differ between MWRA and UWRA. We identified sexual activity among UWRA as a major factor to be included in the hierarchical structure of the model for UWRA.*

The model was checked for predictive accuracy using cross-validation exercises. We have modified the last paragraph of the ‘Statistical model’ subjection of ‘Methods’. It now reads:

The validity of the model was assessed with three cross-validation exercises [22]. These entailed leaving out certain sets of observations (depending on the exercise), re-fitting the model, and checking model results against the left-out observations for calibration and predictive accuracy. The results indicated that the model was robust and adequately calibrated to generate the estimates for family planning indicators among UWRA (S1 Appendix, Sec. 4.3). We also tested the sexual activity classification for UWRA. Since there were two levels (‘low’ and ‘other’), we identified countries with data that were close to the threshold of 2% sexually active among UWRA. The country closest to a threshold was Indonesia, which was assigned to the ‘low’ sexual activity group. A sensitivity analysis was conducted, assigning Indonesia to the ‘other’ group, but its relatively abundant data resulted in no meaningful change in the results.

Other modifications to the ‘Methods’ section that provide additional detail and clarification on the model-building process have been made in response to reviewers’ comments, particularly Reviewer 2. These are detailed under the respective comments below.

7. Please ensure the doc is clean and clear of track changes – page 8.

Done.

8. Please clarify how many countries are included – abstract you say 185, under the ‘Data compilation’ section you say 195.

We have added the following to the end of the subsection ‘Indicators used’ in ‘Methods’:

*We present results for the 185 countries with at least 90,000 inhabitants in 2017 [25]. Estimates from the additional 10 countries and areas with total population below 90,000 inhabitants are included in the aggregated results.*

9. Please clarify in the main text what traditional and modern contraceptives are, with examples for relevance. Please place this ahead of the first mention in table 1.

We added in the main paper the text from S1 Appendix, Sec 2.2.:

*For analytical purposes, contraceptive methods are classified as either modern or traditional. Modern methods of contraception include female and male sterilization, the intra-uterine device (IUD), the implant, injectables, oral contraceptive pills, male and female condoms, vaginal barrier methods (including the diaphragm, cervical cap and spermicidal foam, jelly, cream and sponge), the lactational amenorrhoea method (LAM), emergency contraception and other modern methods not reported separately (e.g., the contraceptive patch or vaginal ring). Traditional methods of contraception include rhythm (e.g., fertility awareness-based methods, periodic abstinence), withdrawal, and other traditional methods not reported separately.*

10. Fig 4 – more developed / less developed countries. This is rather vague. Is this common terminology? If not please use something more specific.

It is a classification commonly used by the United Nations. We have added the following explanation to the figure caption including the clarification of categories by income level by the World Bank:

The more developed countries comprise all countries of Europe and Northern America, and Australia, New Zealand and Japan. The less developed countries comprise all other countries. The country classification by income level is based on June 2019 GNI per capita from the World Bank.

11. Please provide a completed GATHER checklist as a Supp file.

We completed GATHER checklist and provide it as a Supplementary file.

# Comments from the reviewers:

## Reviewer #1:

The paper is well written and organized and addresses and important global health issue. It will likely be widely cited. Past studies of FP indicators (eg. by the UN) have often focused on married/in union women, thus neglecting a growing population of unmarried women. The present study provides for the first time estimates and projections of FP indicators by country and region for both married and unmarried women.

There are no issues that are problematic or require major revision. However I have several suggestions for clarifying the presentation.

Thank you for your comments; our responses follow.

1) The abstract and introduction should state clearly that you will present estimates from 1990 to 2019 and projections from 2019 to 2030

We added in the abstract:

*We produced estimates from 1990 to 2019 and projections from 2019 to 2030 of contraceptive prevalence and unmet need for family planning…*

2) Introduction: between the first and second sentence add something like "past estimates of FP indicators have been largely limited to married/in union women but recent changes in ….."

We added the sentence in the first paragraph of the introduction.

3) To provide the reader with a better understanding of the importance of unmet need and unsatisfied demand, note that there x million abortions and Y million unplanned pregnancies annually with wide ranging adverse consequences.

We elaborated the existing paragraph in the Discussion section and noted the high numbers of unintended pregnancies and abortions citing previous studies:

*Furthermore, the results of our study are needed to underpin models of the impact of increased use of family planning on other reproductive health outcomes [13,32] and models estimating the wider impact on health, schooling, and economic outcomes. Worldwide, an estimated 44% of pregnancies were unintended in 2010-14 and some 56% of all unintended pregnancies ended in abortion [33]; these findings underscore the continuing need for investments to meet women's and couples' contraceptive needs. Globally, unmarried women account for a significant share of unintended pregnancies and abortions and these events could potentially lead to more serious consequences for unmarried women than for married women. The annual number of abortions worldwide is estimated at 56.3 million in 2010–14, of which 27% were obtained by unmarried women [34]. Both studies [33,34] pointed out that the lack of estimates of contraceptive use and unmet need for family planning among unmarried women limited their ability to determine the extent to which these trends were associated with need for contraception. Our study will close this gap.*

4)The term "posterior" (used from page 8 onwards) should be defined for readers who are not familiar with Baysian methods

What was paragraph 3 of the ‘Statistical model’ subsection of ‘Methods’ has been split in two and the first part expanded. It now reads:

Country-specific estimates of model parameters were obtained by fitting a Bayesian hierarchical model [22], which is fully described in S1 Appendix Sec. 3.6. The output of a Bayesian model is a ‘posterior’ distribution, a high-dimensional probability distribution for the parameters of interest. It is the result of combining information about the parameters contained in the data (encapsulated in the ‘likelihood’ component of the model) with existing prior knowledge (encapsulated by the ‘prior distribution’ component). Standard practice is to summarize the posterior distribution with quantiles of key parameters that provide point-estimates and ranges representing the magnitude of uncertainty. We report posterior medians for point-estimates and uncertainty intervals (UI) constructed from the 2.5 and 97.5 percentiles. Under the model, and given the available data, there is a 95% probability these uncertainty intervals contain the true value of the parameter.

5) The PPI measure should be defined and the reader should be given some guidance as to how to interpret a value of say 0.9.

We have added the following paragraph to the ‘Indicators used’ subsection of ‘Methods’

For example, PPI>0.9 means that there is more than 90% probability that the actual change was greater than 0.

6) p13 line 5 and 6 says: " ...are largely a reflection of differences in sexual activity among UWRA" This statement is probably correct but how do you determine this?

We changed the text to be descriptive of the results and less of an interpretation. It now reads:

Contraceptive prevalence and the scale of its increase varied widely across geographic subregions and estimates of the proportion of contraceptive users (expressed here as among all UWRA irrespective of sexual activity status) are low in countries where sexual activity among UWRA is also low.

The influence of sexual activity on measuring contraceptive prevalence is part of the ‘Discussion’ section and we provide also reference to [29]

Dasgupta A, Ueffing P and Kantorová V (2017). Sexual Activity by Marital Status and Age: A Comparative Perspective. United Nations, Department of Economic and Social Affairs, Population Division, Technical Paper No 11. New York: United Nations.

7) Page 23 first para: Isn't a rise in sexual activity among UWRA part of the explanation?

We mention sexual activity as part of the explanation; however, the findings of previous study [29] observed no generalised pattern with both declines and increases in sexual activity among UWRA based on available data. We therefore changed the language in the discussion section to be more cautious about the trends in the future across wide range of countries.

The contribution of UWRA to the total demand for family planning will likely continue to increase due to further postponement of marriage and union formation; however, it will also depend on the changes in sexual activity among UWRA. A previous study of trends in sexual activity among UWRA [29] observed no generalised pattern of increasing sexual activity among UWRA over time and across countries.

## Reviewer #2:

Monitoring progress towards universal access to family planning, and associated indicators has been identified as a global priority. Unmarried women are often excluded from studies and demographic health surveys, which biases reporting of key family planning indicators. The authors present a model to help quantify the contribution of unmarried adolescents to these FP indicators, which is helpful for programs and policymakers. However, the authors miss a few opportunities to be more transparent in the main text on how the indicators were derived for unmarried women, particularly sexual activity which is used to create the indicators, and the sources of information that were used to make this estimate. In addition, the authors could make more comparisons to put their results in context. For example, some countries have included unmarried adolescents in their DHS surveys. How do the results compare for these specific countries. Also, it is unclear what the intention of including the married women indicators are, and how these indicators are different than prior estimates. It would be plausible to compare the unmarried/married, and also the total (All women) to show the impact of excluding the unmarried women is. Overall the analysis is interesting, and transparency in key methods in the primary text (not appendices) would improve comprehension about the methods used to create the model. Finally, the article is quite long and it would be helpful for the authors to more briefly describe the methods (omitting some of the modeling jargon but adding details critical to understanding how estimates were derived), cut the text to highlight key results and make more comparisons between unmarried/married/all women, and synthesize results more concisely in the discussion. These edits would greatly improve the manuscript.

Thank you for your detailed comments. We would like to point out that our unmarried estimates are for women aged 15–49, not just adolescents (though the adolescents are a majority of unmarried women of reproductive age in most countries). How our results for UWRA compare to the estimates derived from DHS and MICS microdata are presented for all countries with available data in S2 Appendix. Our response to your comment no. 4 may also be relevant here.

With regards your comments on the ‘Methods’ section, Reviewers 1 and 3 also suggested modifications there. Many have been made in response to all these comments and we also refer you to our responses to Reviewer 1 (comments 4 and 5) and Reviewer 3 (first three comments) in addition to our specific responses to your comments below. We have tried to balance the need to be clear and technically precise with the need to remain accessible to the non-technical reader.

Specific comments are included below:

*Abstract*

1. The conclusions state the CPR and demand for FP differ by marital status yet only unmarried women are described in findings. Add in findings from married women.

We understand the reason for your comment but we prefer not to lengthen the abstract, particularly since our main focus are the results for unmarried and all women. Results for married women are discussed in a separate section in the ‘Results’ and presented in all figures.

2.	The last sentence of the conclusion is essentially same as the background. The data do not clearly indicate why "accurate monitoring" is essential.

Yes, we agree that the language was not accurate. We changed it to:

*Monitoring of family planning indicators for all women, not just those who are married or in a union, is essential for accurately monitoring progress towards ensuring universal access to sexual and reproductive health care services, including family planning, by 2030 in Sustainable Development Goals with its emphasis on ‘leaving no-one behind’.*

*Introduction*

3.	The last sentence could be shortened to more concisely describe what the objective was and the other details can only be mentioned in methods.

We reduced the text in the last para of the introduction.

4.	More details on unmet need for UWRA who have not initiated sexual activity should be briefly discussed as you include this specific group in your methods.

We added text to point out that UWRA comprises not just of young unmarried women, but of unmarried women aged 15-49. We follow the definition and computation used by the Demographic and Health Surveys and UWRA women who have not initiated sexual activity cannot be assigned to “unmet need for family planning”. We give detailed indicator definitions in the appendix and have added a pointer to the specific appendix section (underlined here to identify the additional text):

Unmet need calculation is based on the definition and computation used by the Demographic and Health Surveys [19] (S1 Appendix, Sec. 2.2).

*Methods*

5.	Please specify the duration of delaying future childbearing in your definition. Also, be clear that when you say delay you mean future children, not necessarily delay first pregnancy.

We have changed the text in the first paragraph of ‘Definitions’ under ‘Methods’ to include the duration in the definition. It now reads (underline added here to identify the additional text):

Unmet need for family planning is the percentage of women who want to stop or delay childbearing for at least two years but are not using any contraceptive method.

6.	Omit the rational "caution" in the methods. "While FP indicators…"

We deleted the word caution here.

7.	It would be helpful if definitions were applied universally when indicated, ie at risk of pregnancy, rather than described for one group and referred to as being calculated in the same way.

We have changed the text in the last paragraph of the ‘Definitions’ subsection of ‘Methods’ to be more precise and to avoid referring to the same way of calculation:

*Unmet need for UWRA who are pregnant or postpartum amenorrheic is determined regardless of their most recent sexual activity either as pregnant UWRA whose pregnancies were unwanted or mistimed at the time of conception, or postpartum amenorrheic UWRA who are not using family planning and whose last birth was unwanted or mistimed.*

8.	Suggest re-defining calculation of unmet need rather than just citation.

We follow the DHS revised definition of unmet need for family planning; we hope it is enough to cite this. We changed the text to make this more clear (underline added here to identify the additional text).

Unmet need calculation is based on the definition and computation used by the Demographic and Health Surveys [19] (S1 Appendix, Sec. 2.2).

9.	It would be helpful to briefly describe how you determined if "social values shows most of the population regarded sexual activity among unmarried as not acceptable/justifiable". This is a critical component of parameterizing your model and should be described in methods not just in appendix.

This point was also raised by Reviewer 3 (fourth comment). We have added extra explanation to the paragraph now beginning “The model for MWRA used a purely geographic clustering …” in ‘Statistical model’ subsection of ‘Methods’. The paragraph now reads:

The model for MWRA used a purely geographic clustering [18]; in other words, countries geographically near each other were clustered together. To improve estimation for UWRA in countries with very few, or no, data points, and to account for the cross-country variations in reproductive behaviour and sexual activity among UWRA that has an impact on family planning indicators, we developed a two-category sexual activity classification and combined it with geographical clusters. The two categories were: i) countries with very low levels of sexual activity (Group 0), and ii) all other countries (Group 1). Eighty-one countries were assigned using information about proportion sexually active from the most recent DHS or MICS; countries with less than 2% sexually active (defined as having a sexual intercourse in past 28 days) among UWRA were assigned to Group 0, the rest to Group 1. A further 43 countries were classified using information about the acceptance of sex between unmarried adults reported in the Pew 2013 Global Attitudes Survey (29 countries) or the World Values Survey Wave 6 (14 countries) and remaining 71 countries, all in Asia and Northern Africa, were assigned on the basis of the proportion religious in the population (S1 Sec 2.4 and Fig 2.3).

We have also added a new table to the Appendix (S1 Sec 2.4) showing how countries were classified:

*Frequency table of countries by sexual activity classification and survey or method used to classify them.*

| *Survey/Method* | *Sexual Activity Group 0* | *Sexual Activity Group 1* | *Total* |
| --- | --- | --- | --- |
| *DHS and MICS* | *16* | *65* | *81* |
| *Pew 2013 Global Attitudes Survey* | *8* | *21* | *29* |
| *World Values Survey Wave 6* | *2* | *12* | *14* |
| *Main religion* | *18* | *53* | *71* |
| *Total* | *44* | *151* | *195* |

10.	The methods would benefit from more specificity rather than general approaches. Ie "estimates for countries and indicators with no data….", which countries or how many where there? Which indicators? More transparency in the methods would improve readability in the methods, yet also ensure description of methods is appropriate for the journal audience (most of whom aren't modelers/statisticians).

We re-arranged some sentences in the first paragraph of subsection ‘Statistical model’ of ‘Methods’ to improve clarity and better link it to the subsequent modifications. These are shown in the track changes version of the manuscript.

We added additional explanations of the hierarchy that reads:

Many countries had only a few data points and some countries had none. For example, 59 countries with data on contraceptive prevalence for MWRA had no such data for UWRA. Of the 136 countries that did have data, only 26% of them had any data before 1990 (compared with 63% for MWRA). To improve the precision and accuracy of estimates and projections, a hierarchical structure was added to the Bayesian model. Hierarchical structures cluster countries together based on common characteristics and allow ‘borrowing of strength’ among observations within the same cluster. Estimates for countries with little, biased or no data are based on data for other countries in the cluster; partly if they have some data, entirely if they have none. The model implicitly weights data from other countries based on the hierarchical structure positions of the countries.

We made additions to what was paragraph 4 in subsection ‘Statistical model’ of ‘Methods’ to provide examples. It now reads

We included additional parameters in the model to account for surveys that used different target populations (e.g. non-standard age groups, omission of subnational geographies) or a different categorization of contraceptive method use (the bias and misclassification parameters are described in S1 Appendix Sec. 3.5.) For instance, some surveys include women of different age groups compared to the baseline population of women aged 15–49 years (e.g., the Gender and Generations Surveys (GGS) that surveyed only women aged 18 and over).

In the paragraph beginning “Estimates for years outside the periods of data availability … has been modified. It now reads:

Estimates for years outside the periods of data availability were obtained from the fitted Bayesian model by interpolation and extrapolation of the systematic logistic trends and the auto-correlated error processes.

On re-reading the paragraph previously the sixth in ‘Statistical model’ (it began “Estimates for countries and indicators with no data were obtained from weighted averages…”), we realized it also contained a lot of technical language. It has been deleted and the explanation of how estimates for countries with no data were obtained has been transferred to an earlier paragraph, namely the one mentioned just above under this same comment (it begins “Many countries had only a few data points and some countries had none. For example, 59 countries with data…”).

11.	How did the authors determine if the model was "robust and adequately calibrated"? Be more specific.

The model was checked for predictive accuracy using cross-validation exercises. Please see also our response to the Editor’s requests, sixth comment, for modifications to this paragraph.

12.	Define traditional method use.

We added the definition of traditional and modern methods from the Appendix to the main text.

13.	I suggest adding prior published estimates of indicators on graphs where available, as data points or lines. This would improve interpretation of how the model for married women compares with prior estimates, as it is unclear what added value the model has for calculated indicators for married women rather than as a comparison using similar methodology for unmarried women.

There was no change to the method for married women relative to the earlier published results in Cahill et al. (2017). However, they applied the married women model only to the 69 Family Planning 2020 focus countries (<http://www.familyplanning2020.org/countries>) and reported estimates for 2012 and 2017 only. We report estimates for 1990 to 2019 and projections to 2030 for 185 countries, and also we report regional and global aggregates. We do not believe that adding estimates from Cahill et al. (2017) to our plots would be a useful comparison as they are so similar in the countries and years that overlap. We do, however, present country plots with the survey source data in S2 Appendix both for MWRA and UWRA.

We also modified the first part of method section for clarification:

For MWRA, we used existing statistical model [8,18] on the updated data compilation. We expanded the model to UWRA (see S1 Appendix, Sec. 3). Both models were fitted to the data described above and we report the model-based estimates and projections.

*Results*

14.	The authors begin by summarizing the changes in FP indicates among married women over time and geographically. However the primary objective is to show unmarried women, this should be the focus of the results and comparisons made with married women.

We needed to make a decision in terms of how to organize the results. We started with MWRA. While the methods were not new for MWRA they represent majority of women of reproductive age (and contraceptive users and women with unmet need). Since we presented updted and expanded results for MWRA compared to previous papers, we considered it important to present them to “set the scene” on what is more familiar already to the reader.

15.	Figure 1 shows data through 2030 but the text refers to 2019. Suggest harmonizing presentation to include 2030 for consistency, even though it is projected.

For Fig 1a and 1b, estimates are indicated for the years 1990, 2000, 2010, and 2019 by solid circles and interpolated by solid lines. Projections for the year 2030 are indicated by open circles and interpolated by dashed lines.

16.	Results could be presented more succinctly to highlight key points rather than more comprehensive text on results (ie perhaps not all geographic regions need to be mentioned in text for all indicators).

We acknowledge your comment but we do believe that the results across geographic regions are important so as to draw attention to differences in contraceptive use and needs both for MWRA and UWRA.

17.	Presentation of married/unmarried women separately in the text makes it more difficult to compare findings. I suggest reorganizing to pair the indicators and present married/unmarried together.

We needed to make a decision on the presentation of the results and opted for the patterns and trends for regional aggregates. However, in tables and figures the results for MWRA and UWRA are presented side by side.

18.	Figure 4, need legend to understand income group. Also, suggest moving to appendix.

We added the following in respective places below figures, as needed:

*The more developed countries comprise all countries of Europe and Northern America, and Australia, New Zealand and Japan. The less developed countries comprise all other countries. The country classification by income level is based on June 2019 GNI per capita from the World Bank.*

We would prefer to keep this in the main text.

19.	Avoid interpreting results in results section, ie interpreting the explanation why total need for FP increases slightly. It is also redundant with the discussion.

We deleted these occasions from the results – either completely, or moved them to the discussion.

20.	The comparison of indicates with Guttmacher is helpful; there appear to be a few nuances of the data prior to or including 2017. Could you do a sensitivity analysis and compare your 2016 estimates to their 2017 to help account for this difference?

We compare and discuss the findings for at the aggregate level (developing countries aggregates and sub-Saharan Africa). We cannot compare the results at the country level or do any sensistiveity analysis because the countries results for Adding It Up 2017 were not published.

We added this sentence:

*Adding It Up 2017 did not publish results for individual countries and therefore we cannot directly compare the results.*

## Reviewer #3:

[Please note that we have numbered these comments for easier reference]

This is an interesting paper concerned with unmet need for family planning. It is well written and represents the result of many laborious tasks. The methodological aspects of the work are well selected and suitable for the applied problem.

Specific comments are given below.

Thank you for your comments; our responses follow.

1. Linking the evidence on MWRA and UWRA is a vital aspect of the work. The way this is performed is described in the appendix where the details of the hierarchical model which allows for borrowing of strength are outlined. However, it would be illuminating if the principles of this aspect are also given in the main manuscript, especially the intuition of how is this crucial task accomplished, including the flow of information.

We added language on the hierarchical model:

*To improve the precision and accuracy of estimates and projections, a hierarchical structure was added to the Bayesian model. Hierarchical structures cluster countries together based on common characteristics and allow ‘borrowing of strength’ among observations within the same cluster. Estimates for countries with little, biased or no data are based on data for other countries in the cluster; partly if they have some data, entirely if they have none. The model implicitly weights data from other countries based on the hierarchical structure positions of the countries.*

The creation of all-women estimates from MWRA and UWRA results was described at the penultimate paragraph of the ‘Statistical model’ subsection of ‘Methods’ (p.8). We have re-worded this to make it clearer what we did. It now reads:

The samples from the posterior were transformed to produce probabilistic results for other indicators, such as need for family planning satisfied by modern methods. Results on the count scale, such as the number of contraceptive users or women with unmet need, were produced by multiplying posterior sample element by country-specific estimates and projections of the number of UWRA or MWRA, as appropriate [25,26]. For instance, the posterior distribution of the number of UWRA using any method of contraception was obtained by multiplying each element of the posterior sample for contraceptive prevalence by the number of UWRA in the appropriate year, within country. Estimates for country aggregates (e.g., sub-Saharan Africa) were obtained by summing the posterior distributions of the counts across the countries in the aggregate. Estimates for UWRA and MWRA were combined to provide WRA estimates in the same way. That is, for each country at each year, UWRA and MWRA estimates were summed on the count scale to produce WRA estimates.

2. It would be useful to add the priors for the (co)variance matrices (like in 3.47), especially since their density and parameters may affect the results, particularly in hierarchical models with borrowing of information and sparse data, as in the present paper.

The elements of the covariance matrix in (3.47) are made up of the sampling errors, $\nu_{i,k}$, the nonsampling errors, $\zeta_{i,k}$, and the correlations, $r_{S\left[ i \right]}$. The sampling errors are estimated from the survey designs and are fixed throughout. The sentence “The $\nu_{i,k}$ were fixed at these estimated values” has been added after the equation display in Subsection 3.5.4 in S1 Appendix to clarify this.

The priors for the $\zeta_{i,k}$ and $r_{S\left[ i \right]}$ were given in Eqns (3.120) and (3.121) but have been moved to their own paragraph with additional commentary. It reads:

The priors on the correlations and non-sampling error variance parameters in the data model (see Sections 3.5.1–3.5.4 and Eqns (3.93)–(3.51)) were defined marginally in JAGS as:

σ T ∼ IGamma(0.5,0.5 · 0.15 2 ) (3.118)

ζ S,k ∼ U(0.01,2) (3.119)

r S ∼ U(−1,1) (3.120)

The covariance matrices for observations with modern/traditional breakdown, Σ S[i] were constrained to be positive definite. The prior on σ T was somewhat informative due to the small number of observations with no modern/traditional breakdown. It was set using estimates from observations that did provide a modern/traditional breakdown. The priors for the ζ S,k s and r S were intended to be proper but non-informative. Examination of traceplots and prior/posterior plots for these parameters did not suggest the posteriors were restricted by the priors.

3. Also, please discuss the choice of priors when restricted ranges are employed and informative selections are made, like in 3.120, say.

Please see our response above.

4. Please discuss the selection of two (high/low) sexual activity categories. Would a third (say intermediate) category be feasible in terms of data availability? Would this be expected to improve the accuracy of the results?

We have discussed this in new paragraph in the methods sections. It reads:

We also tested the sexual activity classification for UWRA. Since there were two levels (‘low’ and ‘other’), we identified countries with data that were close to the threshold of 2% sexually active among UWRA. The country closest to a threshold was Indonesia, which was assigned to the ‘low’ sexual activity group. A sensitivity analysis was conducted, assigning Indonesia to the ‘other’ group, but its relatively abundant data resulted in no meaningful change in the results.

5. Please add the process of reviewing the evidence which resulted in the data collection. Was that done via a systematic review? Add the details where necessary, including the databases searched and the keywords used

The review started with the surveys that provided information on family planning indicators among married women (MWRA) and which were listed in the previous revisions of *World Contraceptive Use* publication of the United Nations Population Division (which were used in previous work [18]). These are mainly from well-known international survey programs (such as DHS, MICS, etc) that routinely collect data on family planning indicators, or national surveys done by national statistical offices. As many of these are not disseminated through the scientific literature, there was no systematic review process involved keyword searches of data bases. We have split the first paragraph under ‘Data compilation’ to make this clearer:

The data compilation uses data from multiple sources and employs unified definition and computation of survey-based estimates of family planning indicators for unmarried women of reproductive age (15-49 years). We reviewed all nationally-representative household surveys that provided information on contraceptive prevalence. The starting point was the data sources used for estimating contraceptive use for MWRA [18], complemented by queries to national statistical offices, research institutes and international survey programs.

# Additional Changes

We have made a small number of additional corrections and minor changes to improve readability and syntax. These are all fully visible in the track-changes document. Notable among these are:

- Minor corrections to transcription errors in some of the numbers quoted in the ‘Results’ section have been made. All changes are small and have no bearing on any of the substantive findings and conclusions.
- We added in the introduction a reference to the Programme of Action of the International Conference on Population and Development, a key document for reproductive rights, which celebrates 25^th^ anniversary this year.
- Additionally, in para 1 of the Discussion we also added 2019 estimates of contraceptive users and women with unmet need for developing countries. These aggregated values will be useful for advocacy purposes by UNFPA, UN Foundation, FP2020, UNWomen, WHO, UNICEF and others.
- Numbers cited from the Adding It Up 2017 report by the Guttmacher Institute in the ‘Discussion’ section have been corrected. Previously the cited numbers were for any method of contraception (modern + traditional) but the surrounding text specifies modern methods, so we have made the numbers consistent with the text. The corrections have no bearing on the conclusions and assertions made.
- We added additional rows to the supplementary tables in Section 5 of S1 Appendix.
- Ref #21 was corrected to the 3^rd^ edition (previously was 2^nd^ ed.).
