# Supplementary material for: Estimating progress towards meeting women’s contraceptive needs in 185 countries: A Bayesian hierarchical modelling study
Source: PLoS Med. 2020 Feb 18;17(2):e1003026. doi: 10.1371/journal.pmed.1003026 (PMC7028249; doi:10.1371/journal.pmed.1003026)
Supplement: Attachment — Submitted filename: Replies to reviewers&editors_20191212.docx [file pmed.1003026.s015.docx]

Response to Reviewers’ Comments

PMEDICINE-D-19-03179R1

We have copied the editors’ and reviewers’ comments in **bold** and replied underneath in normal font. All modifications can be clearly seen in the track changes version of the manuscript also supplied with the resubmission.

We thank the three reviewers and the editors for their constructive comments on the revised manuscript and hope that we have been able to satisfy their concerns.

# Requests from the editors:

Title: The title is still rather long. We suggest “"Estimating progress towards meeting women's contraceptive needs in 185 countries: a Bayesian hierarchical modelling study" or similar.

We have changed the title to: “Estimating progress towards meeting women's contraceptive needs in 185 countries: a Bayesian hierarchical modelling study". We prefer not to add “from 1990 to 2019” since our paper also includes projections to 2030.

We have updated the titles in S1 Appendix and S2 Appendix to match.

Abstract: Background - The final sentence should clearly state the study question. Please avoid assertions of primacy ("This study is the first..."); we suggest adding ‘To our knowledge...’

We have changed to “To our knowledge…”. We have added the sentence “We seek to quantify the gaps that remain in meeting family planning needs among all women of reproductive age” to the end of this section.

Please begin your Abstract: Conclusions with "In this study, we observed ..." or similar

We have revised the first few sentences of the Abstract Conclusion in response to this comment and Reviewer 2’s comment (below).

Please add the title ‘Author Summary’ ahead of your author summary

We have added this.

At the start of your methods section, please adapt the text to "In this study, contraceptive prevalence was defined ..." or similar.

This is done.

At the top of p.11 of the PDF, you state that "a protocol or analysis plan was not pre-published". Please adapt this wording to state whether or not a prespecified plan was prepared, and if so attach the relevant document as a supplementary file. Please highlight non-prespecified analyses.

The first sentence of this paragraph has been revised to read:

A key contribution of this article is the data compilation and statistical model for estimating contraceptive prevalence and unmet need for family planning among UWRA itself, hence a prespecified analysis plan was not prepared, and all analysis are non-prespecified.

Please add a brief statement to your methods section to note (for example) that ethics approval was not required for this study.

We have added the sentence “Ethics approval was not required for this study” to the end of the subsection ‘Data compilation’ of the Methods section (p. 8).

To begin the first paragraph of your discussion section, please add "Our findings indicate that ..." or similar.

We have made the change.

Please revise any references in superscript and present in square brackets e.g. line 284

Done.

Line 322 - Please add a space between ‘changewith’

Done.

Line 334 - please revise to ‘Estimates from an additional’

Done.

Fig 1a, 1b, 4 - please define GNI in the figure legend

Done in these locations and elsewhere in the text.

Line 463 - please revise to ‘Table 1’

Done in this location and elsewhere in the text.

Line 513 - please revise to ‘The majority...’

Done.

References - please ensure that references are appropriately formatted e.g. removal of ‘The ‘ before ‘Lancet’ in ref 8 and others

We have applied the journal name abbreviations found in the National Center for Biotechnology Information (NCBI) databases to all references.

Please remove quotation marks from reference titles, e.g., reference 31.

Done.

Where necessary (e.g., reference 33), please ensure that 6 author names are listed, followed by "et al.".

Done.

Please adapt the format of reference 24 to match the other references.

Done.

# Comments from the reviewers:

## Reviewer #2:

The revised manuscript has improved the flow and clarity. Only one comment on the revision: Abstract - while I understand the rationale for not including married women in results of abstract, the conclusion of the abstract is based on this comparison of unmarried/married. I suggest revising your conclusion, "Trends in contraceptive prevalence and demand for family planning differ substantially by marital status" or showing the results on married women in the abstract. Otherwise your primary conclusion is not substantiated with evidence in abstract.

We decided to remove the reference to married women. The Abstract’s Conclusion now reads:

In this study, we observed that large gaps remain in meeting family planning needs. The projected increase in the number of women with need for family planning will create challenges to expand family planning services … [as before]

# Requests from Publishing

1. Please note that your figures do not currently comply with our publication requirements. For further instructions to assist with your Figure revisions, please refer to our Figure Guidelines at: <http://journals.plos.org/plosmedicine/s/figures>.

If you need assistance, please contact us directly at [graphics@jjeditorial.com](mailto:graphics@jjeditorial.com) so we can further help with this matter. Please send any relevant source files and tell us what application(s) and what operating system you are using.

- Figures 1 and 3 are multipanel figures. Multipanel figures (those with parts A, B, C, and D) should be submitted as a single file that contains all parts of the figure. Label the figure itself with capital letters, Arial bold font, 12 points.

- Please crop Figures 3a, 3b, and 3c to remove the whitespace around the figure itself. PLOS recommends a 2-point whitespace border, but more will cause the figure to be incorrectly placed in the published PDF.

We have split Figures 1a and 1b into Figure 1 and Figure 2. Figure 4 (previously Figure 3) remains as a multi-panel figure. We have recreated this figure to remove the white margins.

*Please note*: we have supplied Figure 4 (previously Figure 3) as an .eps file. Please let us know if this is of insufficient quality so we can provide alternative formats.

2. Please remove embedded figures from your manuscript file and ensure the most up to date versions of your figures are uploaded to our submission system as separate TIF or EPS files. Please do not remove the figure legends and captions.

This is done.

3. Please ensure that all main figure files are cited in ascending numerical order in the main text of the article.

Done

4. Please ensure that all main figures are referenced in the manuscript as Fig 1, Fig 2, etc. (including capitalization), rather than Figure 1, Figure 2, etc. Please note, however, that the file names themselves must not include the space, i.e. Fig1.tif, Fig2.tif, etc.

Done

5. Please ensure that all main figure legends are placed after the paragraph containing their first in-text citation.

Done

6. Please include the location from which you obtained the base map used in [specific figure] in the legends for this figure. Please note that, because all PLOS articles are published under a CC BY license (creativecommons.org/licenses/by/4.0/), we cannot publish proprietary maps such as Google Maps, Mapquest or other copyrighted maps. If your map was obtained from a copyrighted source please amend the figure so that the base map used is from an openly available source.

We have added the following to the caption of what is now Figure 4:

The base map was obtained from United Nations Cartographic Section (https://www.un.org/Depts/Cartographic/english). The boundaries and names shown and the designations used on this map do not imply official endorsement or acceptance by the United Nations.

TABLES and BOXES:

1. Please ensure that all main tables in their entirety (including titles and footnotes), are placed after the paragraph containing their first in-text citation.

Done.

2. Tables cannot contain images. Please either remove this or change the image to a main figure (in TIF or EPS format) and cite this figure in the table where you would like the image to appear. Please update the order of figure citations as necessary, as well as include a legend for the figure and ensure it follows all figure requirements.

We have replaced the image of Table 1 with a table pasted in from Microsoft Excel. We have *also* included this table in .xlsx format as S1_Table.xlsx.

METADATA:

1. We still require confirmation of authorship and a declaration of any potential competing interests in relation to this manuscript from co-authors Vladimira Kantorova. We have re-sent our authorship email, but also request that you contact these co-authors directly to ensure they respond.

2. Please provide a detailed Financial Disclosure statement on the submission form in full sentences, and include URLs, the initials of the receiving author, and specific grant numbers wherever possible.

The Financial Disclosure in the submission reads:

This work was supported by Grants Nos. OPP1110679 and OPP1183453, Making Family Planning Count, from the Bill & Melinda Gates Foundation (https://www.gatesfoundation.org/) to the Population Division in the Department of Economic and Social Affairs, United Nations (https://www.un.org/en/development/desa/population/theme/making-family-planning-count/index.asp). The funders had no role in study design, data collection and analysis, decision to publish, or preparation of the manuscript.

3. Please ensure that the funders and grant numbers match between the Financial Disclosure field and the Funding Information tab in your submission form. Note that the funders must be provided in the same order in both places as well.

4. In accordance with PLOS' data policy, please ensure that your Data Availability Statement in the submission form clearly identifies how readers can access your data. Data cannot be available on request, or only accessible by contacting one of the authors. Note that your Data Availability Statement will be typeset as it is written, so please ensure it is in complete sentences and appears as you would like it to in the published version.

For details on our data policy, please see <http://journals.plos.org/plosmedicine/s/data-availability>

Data have been uploaded as S1 Data and S2 Data. They are also available for download from the URL entered in the Data Availability box in during submission.

ARTICLE FILE:

1. Please indicate the corresponding author in the byline by placing an asterisk (*) after their affiliation number.

Done.

2. Please include the corresponding author's email address on the title page of your manuscript, indicated by an asterisk (*). Only an asterisk and the email address itself are required.

Done

3. Please remove author degree titles from the byline of your article file.

Done.

4. Please ensure that all authors have at least one affiliation listed on the title page, and that all listed affiliations are attributed to at least one author. Affiliations should be listed in ascending numerical order in both the affiliation list and in the author byline.

Done.

5. The name for your co-authors Vladimíra Kantorová and Mark Wheldon do not match across the article file and the submission form. Please ensure that all author names match verbatim, including the use of a middle name/initials and any special characters (accents, umlauts, etc.), as correct spelling and formatting of author names is vital for accurate indexing in PubMed.

We have updated as follows:

- “Vladimíra Kantorová” is now entered with the diacritics in the submission system (second ‘i’ in first name is ‘í’, last ‘a’ in surname is ‘á’).
- “Mark C. Wheldon” now has the middle initial in the manuscript.
- “Aisha N. Z. Dasgupta” now has middle initials in the manuscript and the submission system.

6. Please remove the data availability information from your manuscript file as this is automatically pulled from the "Data Availability" field in the submission form and must not be duplicated. Please therefore take this opportunity to check that the information in this field is correct and completed in full.

We have removed this form the manuscript and checked that the statement is correct in the submission system.

REFERENCES:

1. Please ensure your references are in the style of PLOS Medicine (ICMJE/Vancouver style). The reference style guide can be found here: <http://journals.plos.org/plosmedicine/s/submission-guidelines#loc-references>. You can also find a downloadable EndNote file in these guidelines to assist you. Please do a manual check as well, as the EndNote file does not catch everything. Please note that the references will be typeset as they appear so please ensure the format is consistent.

Done.

2. Please put all reference citations in square brackets separated by either commas or dashes with no spaces, e.g. [1,2,3] or [1-3].

Done.

3. Please make sure all references are cited in ascending numerical order in the text.

Done.

SUPPORTING INFORMATION:

1. Please upload your supporting information as individual files, and use the following naming format: S1 Text; S1 Fig; S1 Table, S1 Data, etc. Each file type should start as S1 and increase numerically: S1 Text, S2 Text, etc. Note that all relevant in-text citations and captions will need to be updated accordingly.

Done.

2. If you'd prefer to keep your supporting information in one file, please edit any citation of embedded figures and/or tables within your Supporting Information files and the main manuscript to use letters rather than numbers, e.g. “Table A in S1 Text” or "Fig B in S1 Appendix.".

We have re-labelled the main Appendices to S1 Appendix and S2 Appendix. All figures and tables in these appendices are now enumerated using capital letters A, B, C,….

3. Please ensure that all Supporting Information files use the following format verbatim throughout the manuscript (including legends and all in-text citations): S1 Fig, S1 Table, S1 Text, S1 Data, etc. Each file type should start at 1 and increase numerically. Please note, however, that the file names themselves must use an underscore rather than a space, i.e. S1_Fig.tif, S1_Table.xlsx, S1_Text.doc, S1_Data.xlsx, S1_PRISMA_Checklist.docx, etc.

Done.

4. Please ensure that each supporting information file has a legend at the end of the manuscript file, after the Reference list.

Done.

5. Please note that your submission cannot contain any references to generic "supporting information" files and instead must refer to specific files using our naming convention (S1 Fig, S1 Table, S1 Text, S1 Data, SI PRISMA Checklist, etc.)

We now reference only the renamed ‘S*x*’ supplementaries.

# Additional Changes

1. We added a reference to the input data contained in supplementary files S1 Data, S2 Data, and S3 Data in the ‘Data compilation’ subsection of ‘Methods’.
2. We added the URL from which the source code used to produce the estimates can be downloaded in the ‘Statistical analysis’ section of ‘Methods’.
3. We have changed the corresponding author to Vladimíra Kantorová.
